# Supplementary figures and images for: Biomechanical properties of a buzz-pollinated flower
Source: R Soc Open Sci. 2020 Sep 16;7(9):201010. doi: 10.1098/rsos.201010 (PMC7540744; doi:10.1098/rsos.201010)

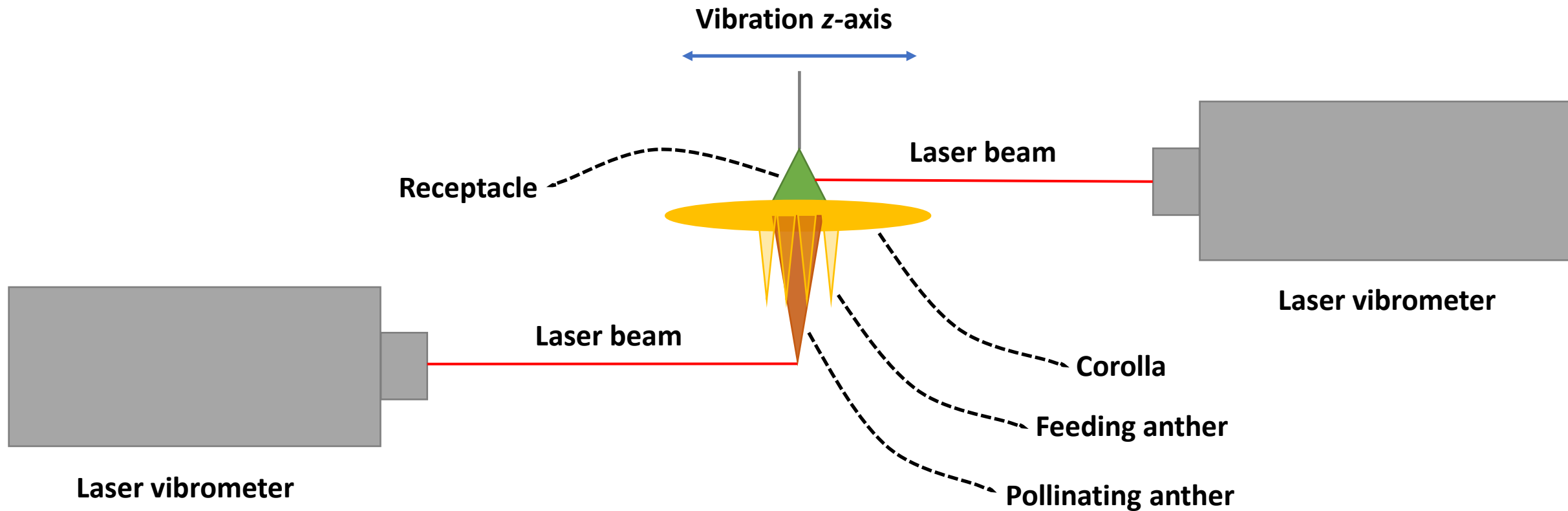

Supplement: Supplementary Figure S1 [file rsos201010supp1.pdf]
