# Supplementary material for: Biomechanical properties of a buzz-pollinated flower
Source: R Soc Open Sci. 2020 Sep 16;7(9):201010. doi: 10.1098/rsos.201010 (PMC7540744; doi:10.1098/rsos.201010)

Vel. = 0.014 ; Str. = Corolla ; Axis = x ; Fl. accession = 10−s−81−1AA

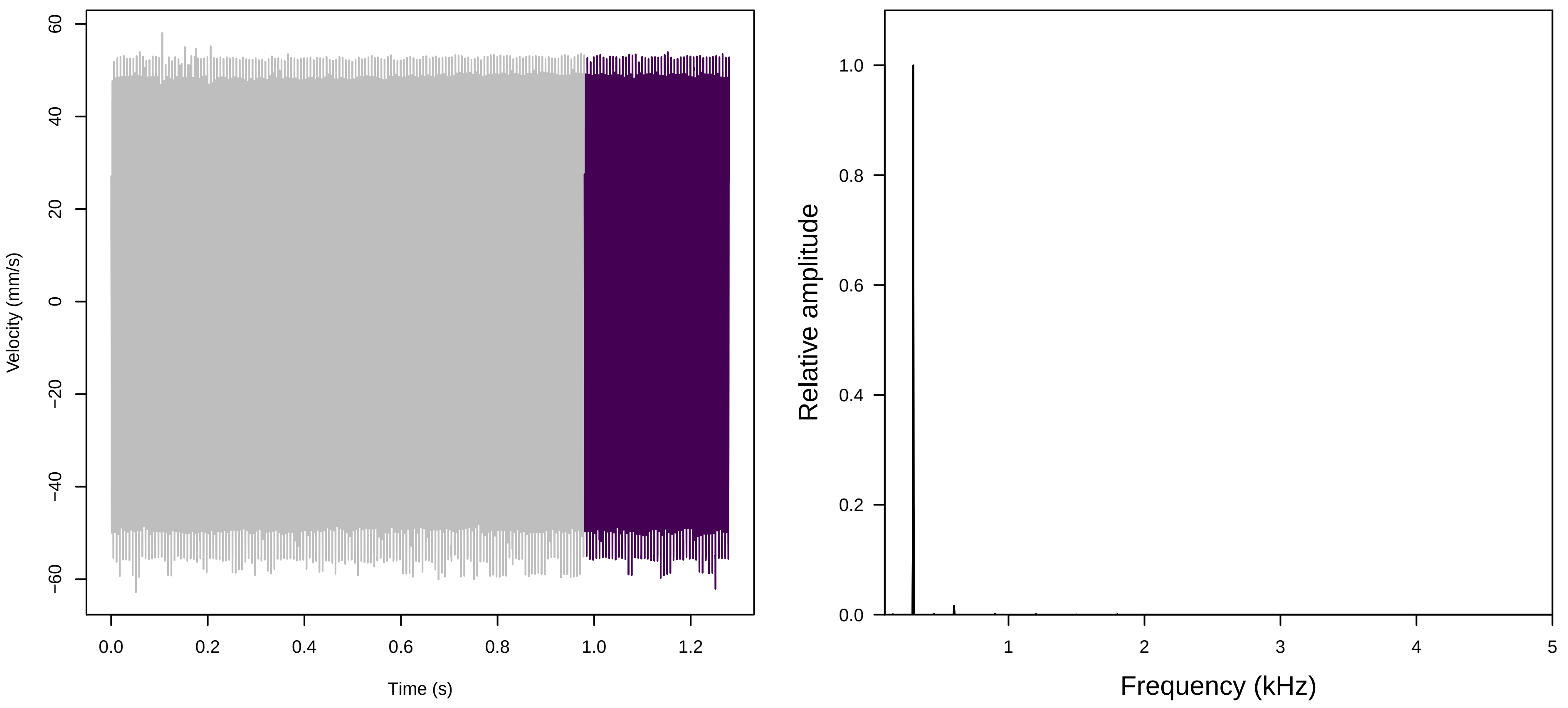

Vel. = 0.014 ; Str. = Receptacle ; Axis = x ; Fl. accession = 10−s−81−1AA

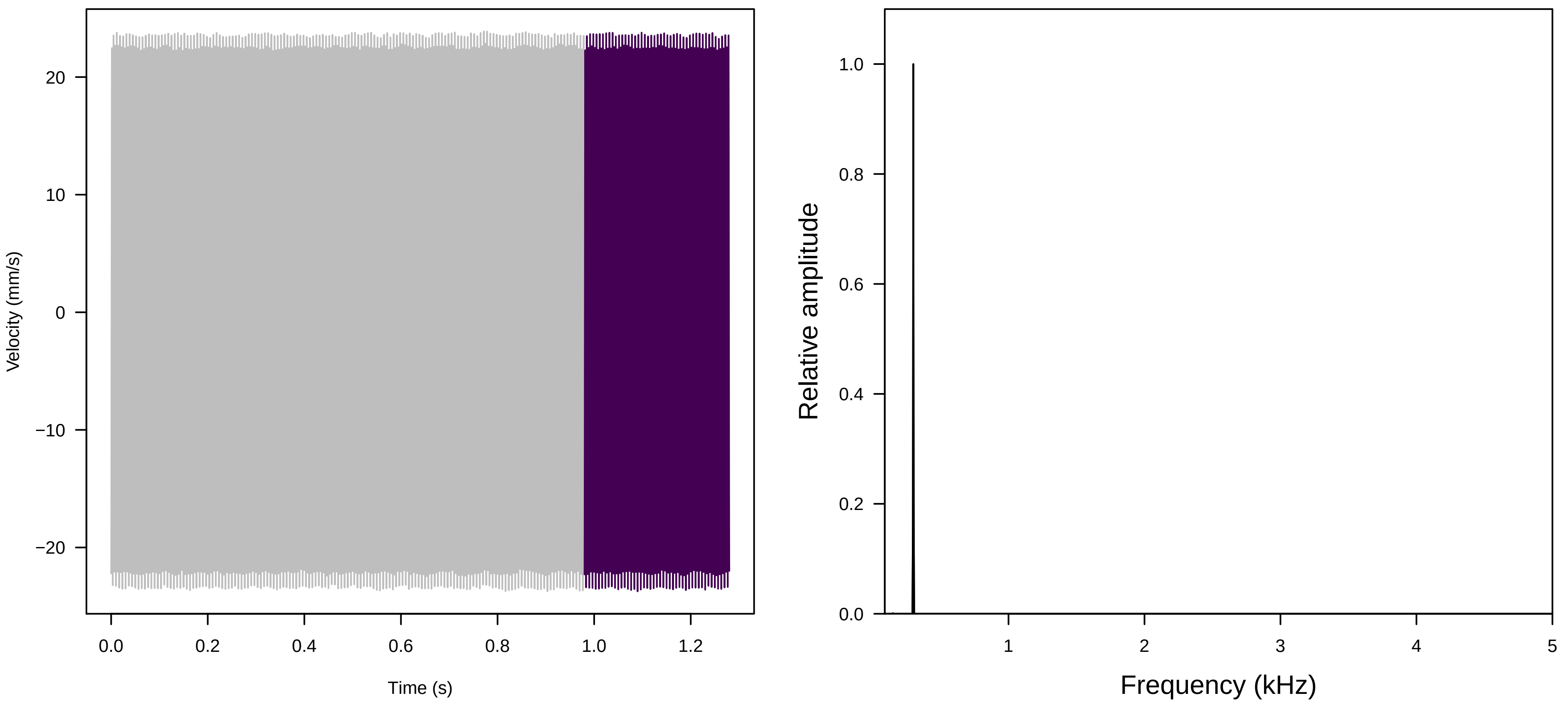

Vel. = 0.014 ; Str. = FA ; Axis = x ; Fl. accession = 10−s−81−1AA

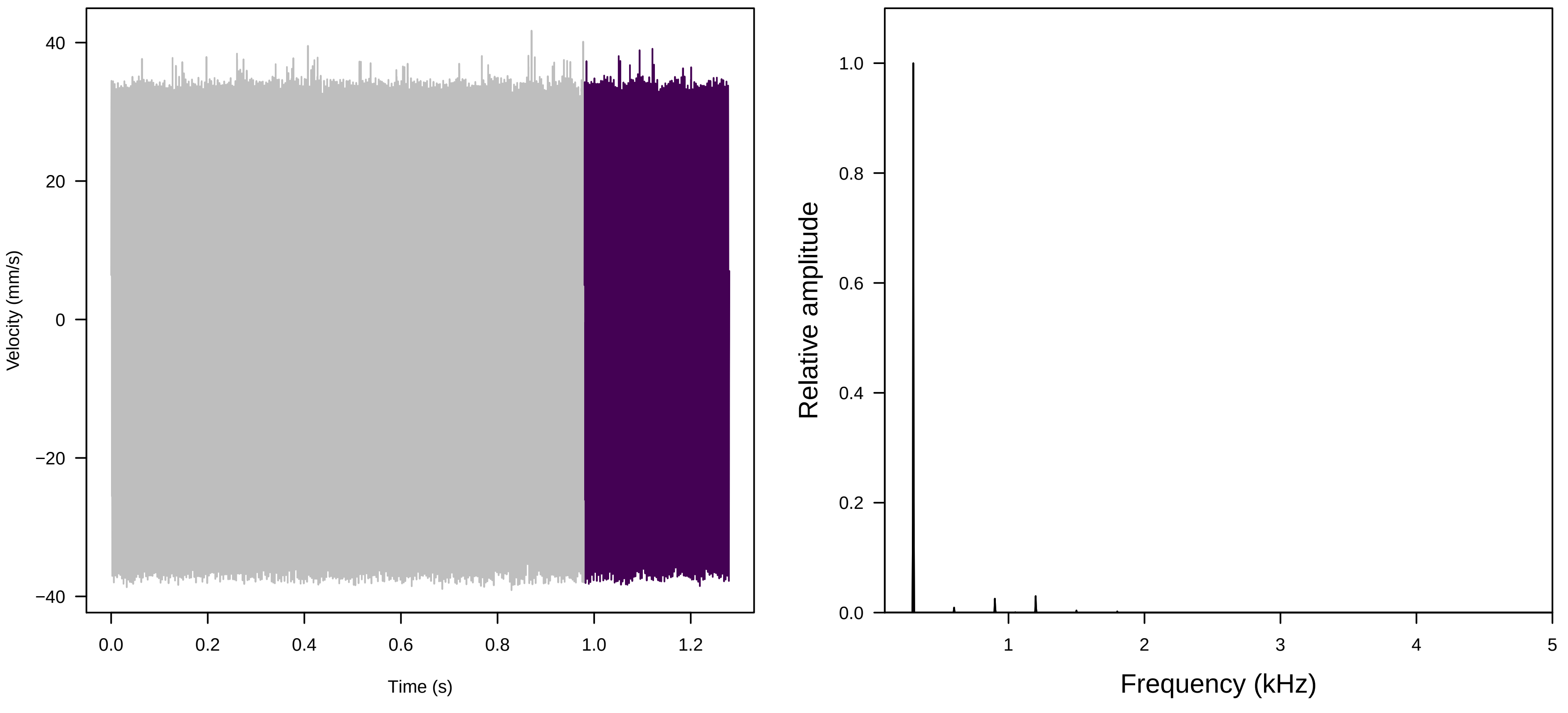

Vel. = 0.014 ; Str. = Receptacle ; Axis = x ; Fl. accession = 10−s−81−1AA

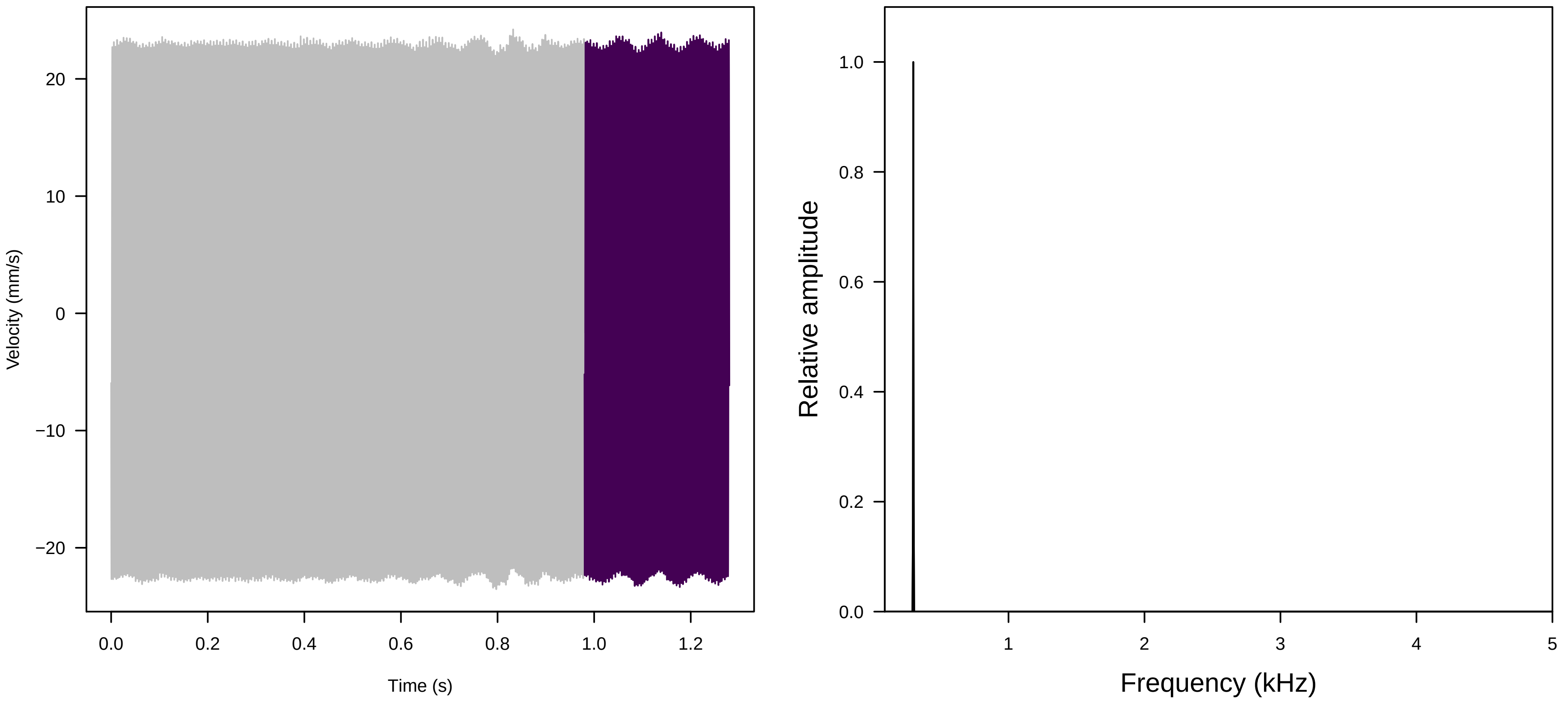

Vel. = 0.014 ; Str. = PA ; Axis = x ; Fl. accession = 10−s−81−1AA

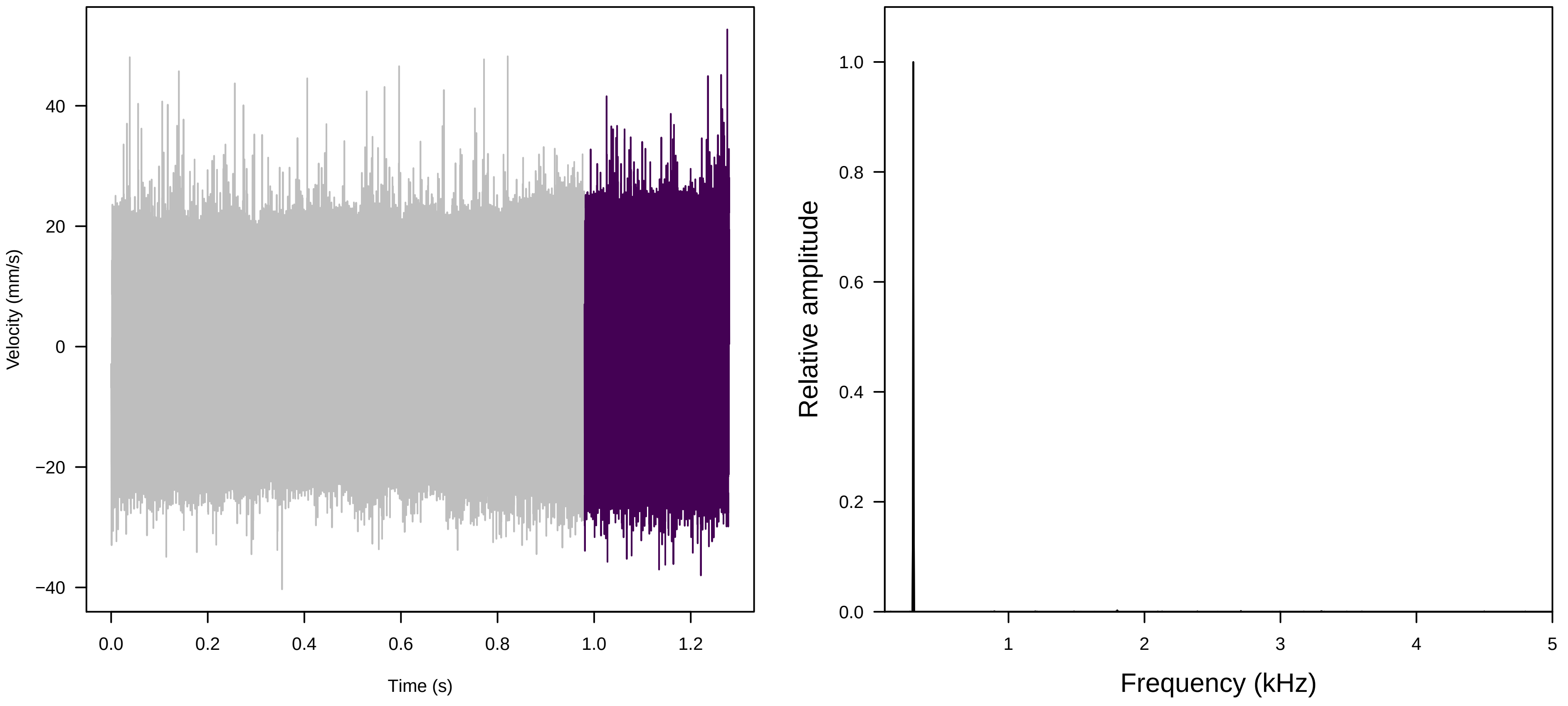

Vel. = 0.014 ; Str. = Receptacle ; Axis = x ; Fl. accession = 10−s−81−1AA

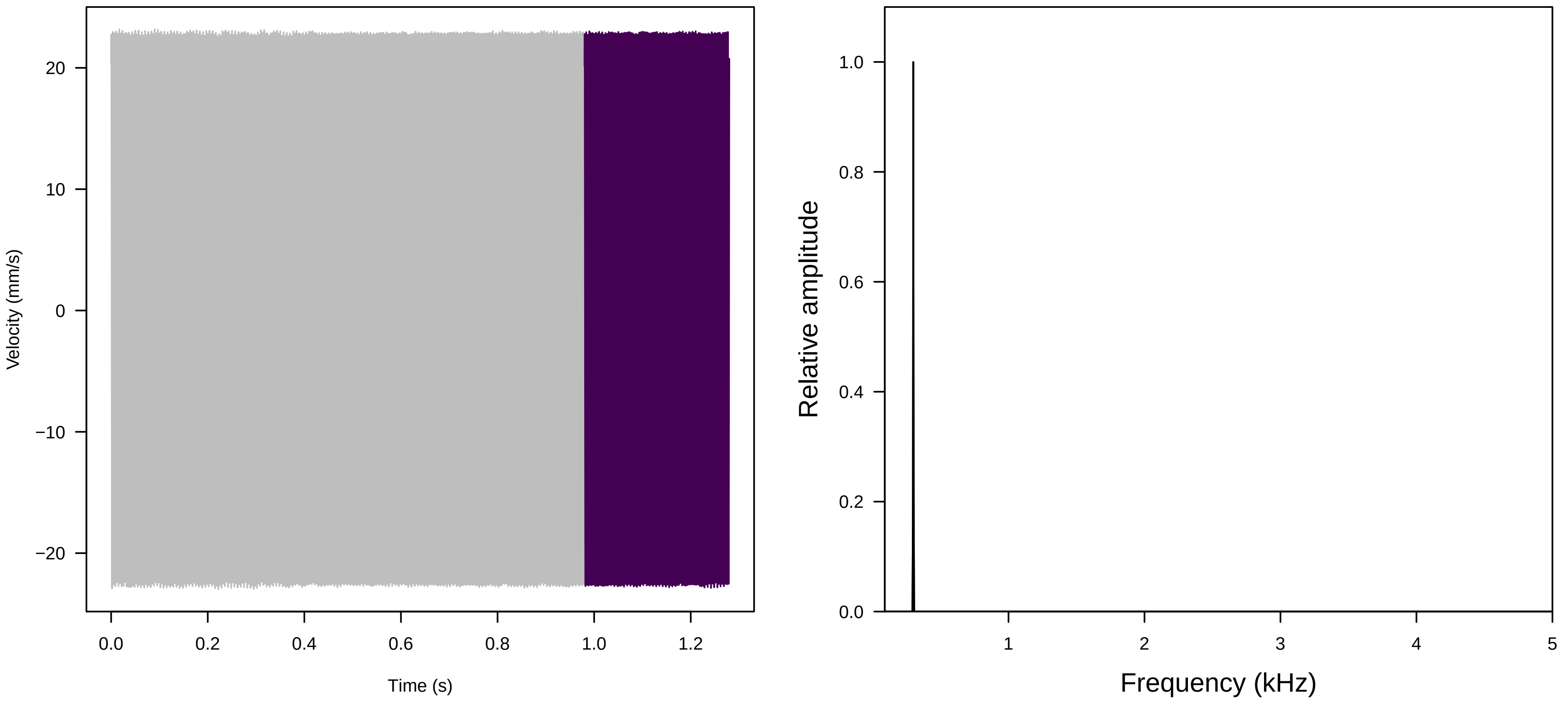

Vel. = 0.028 ; Str. = PA ; Axis = x ; Fl. accession = 10−s−81−1AA

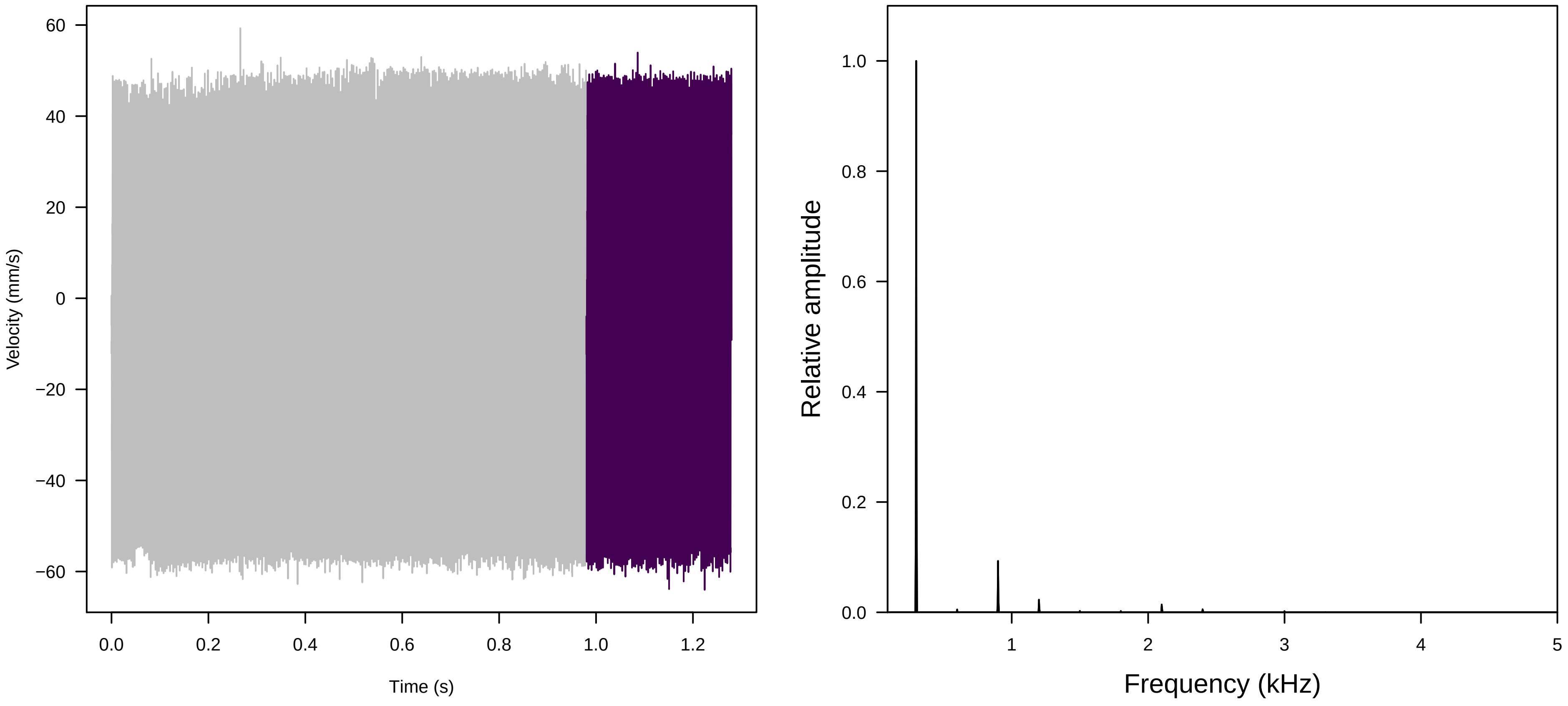

Vel. = 0.028 ; Str. = Receptacle ; Axis = x ; Fl. accession = 10−s−81−1AA

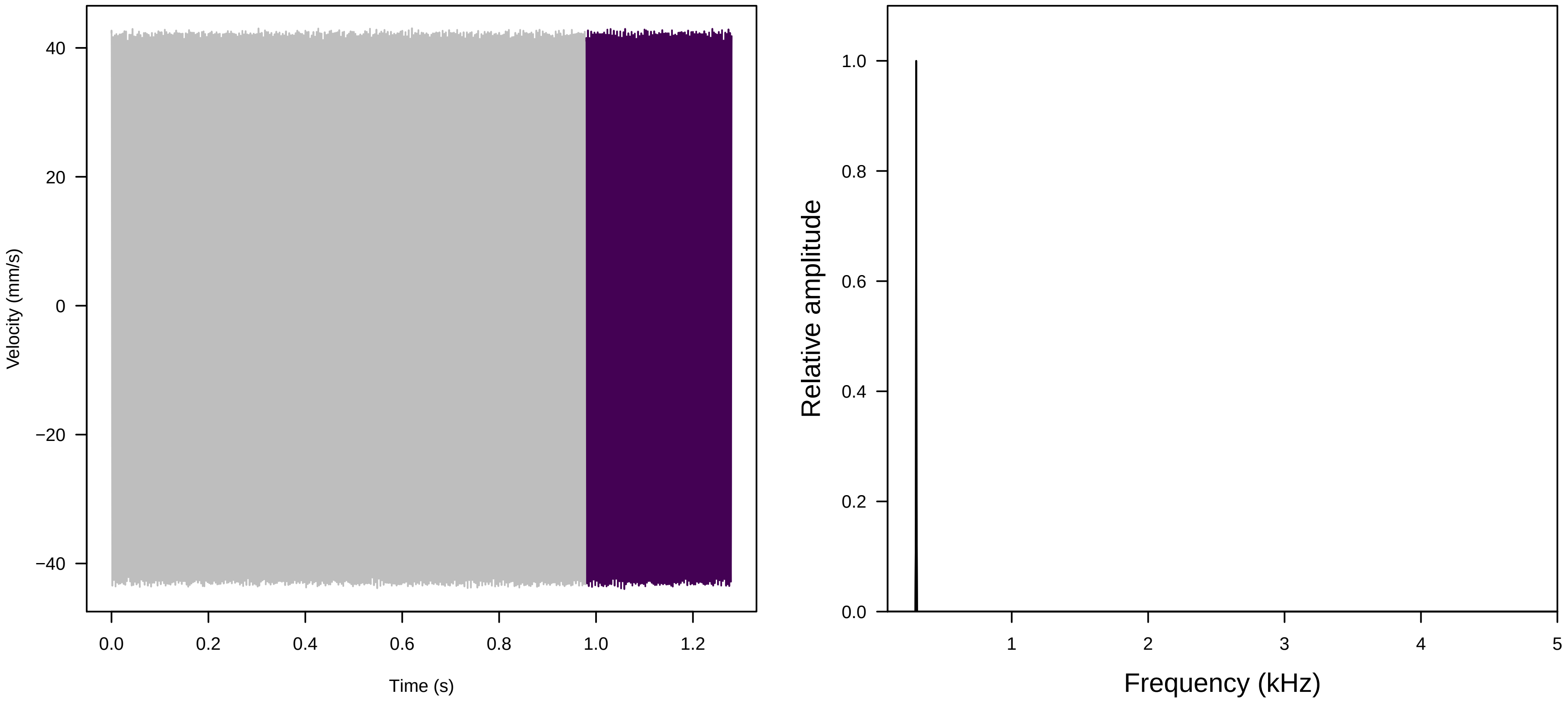

Vel. = 0.028 ; Str. = FA ; Axis = x ; Fl. accession = 10−s−81−1AA

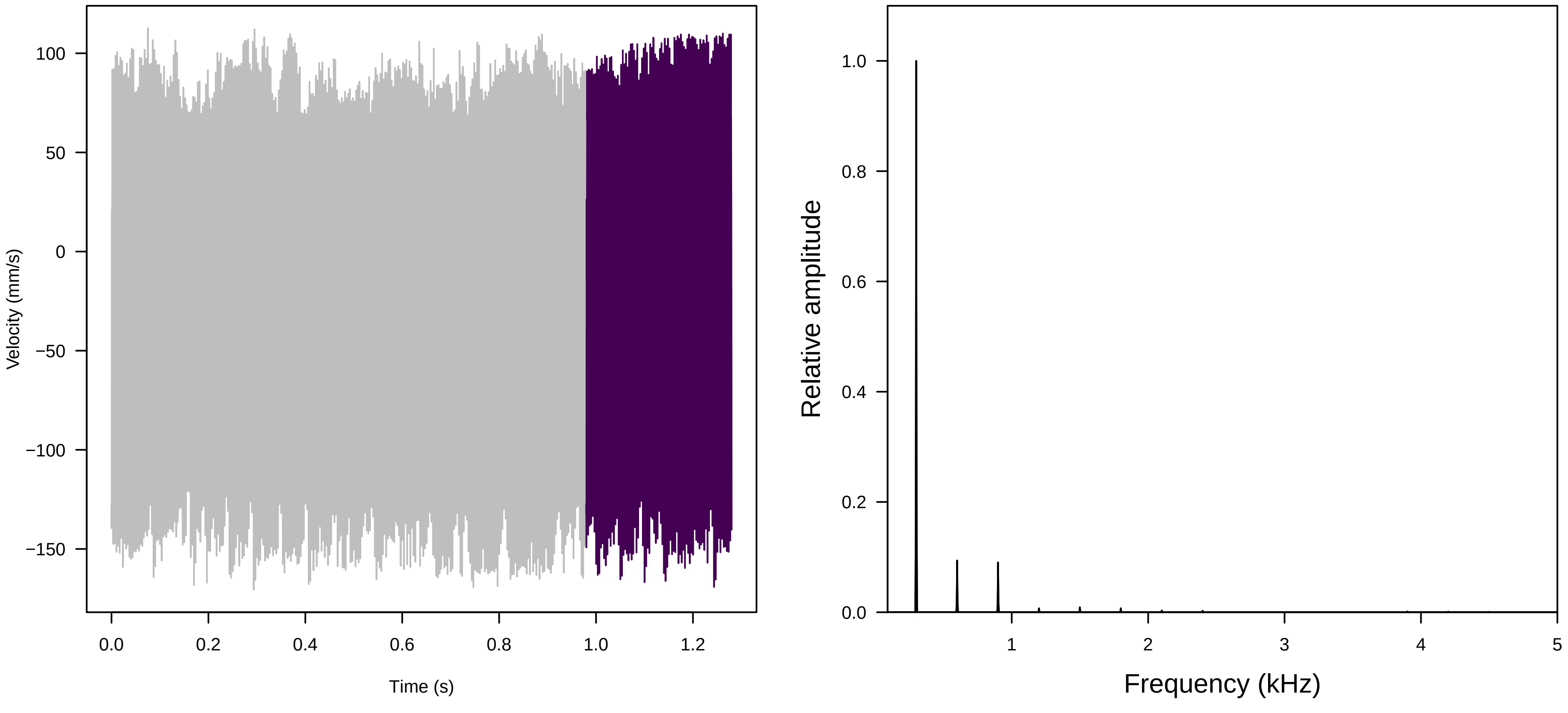

Vel. = 0.028 ; Str. = Receptacle ; Axis = x ; Fl. accession = 10−s−81−1AA

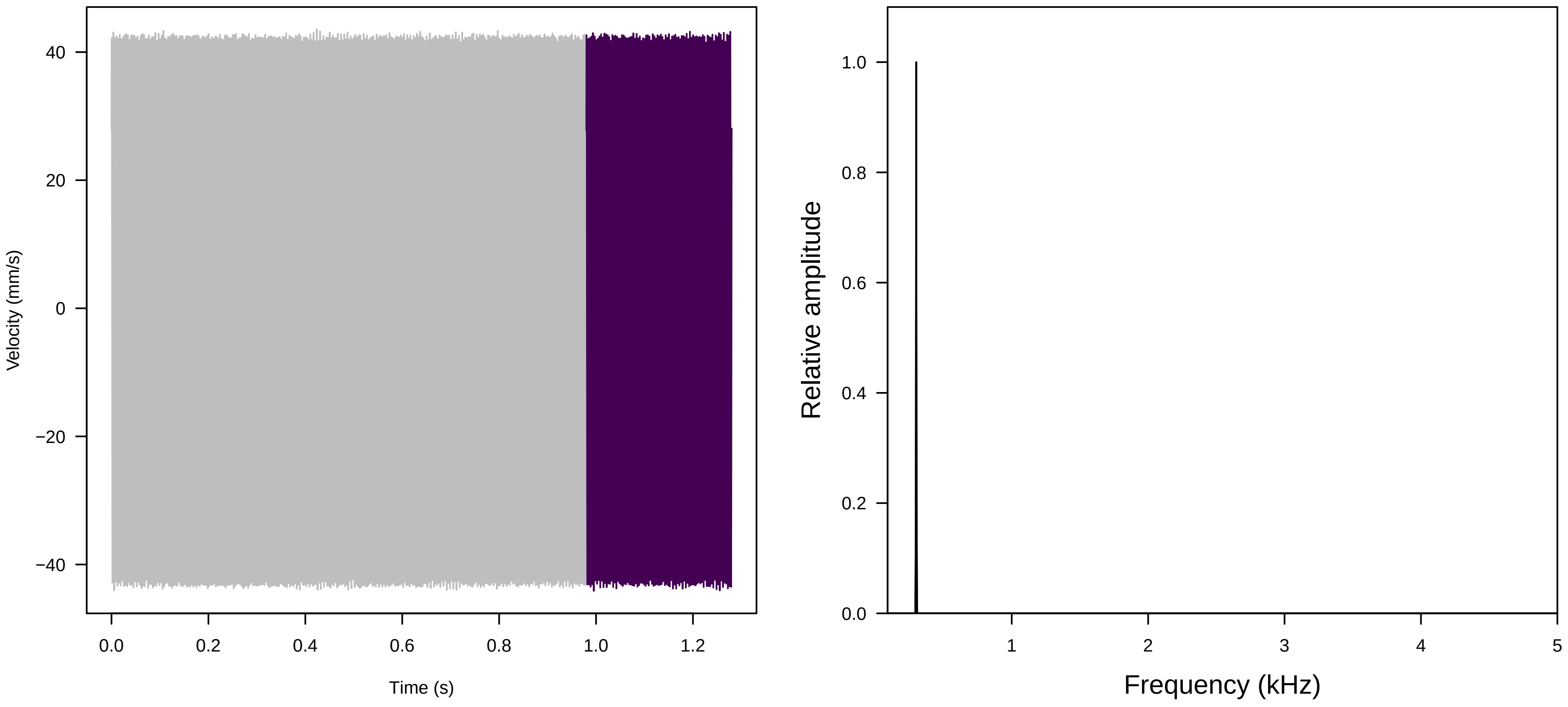

Vel. = 0.028 ; Str. = Corolla ; Axis = x ; Fl. accession = 10−s−81−1AA

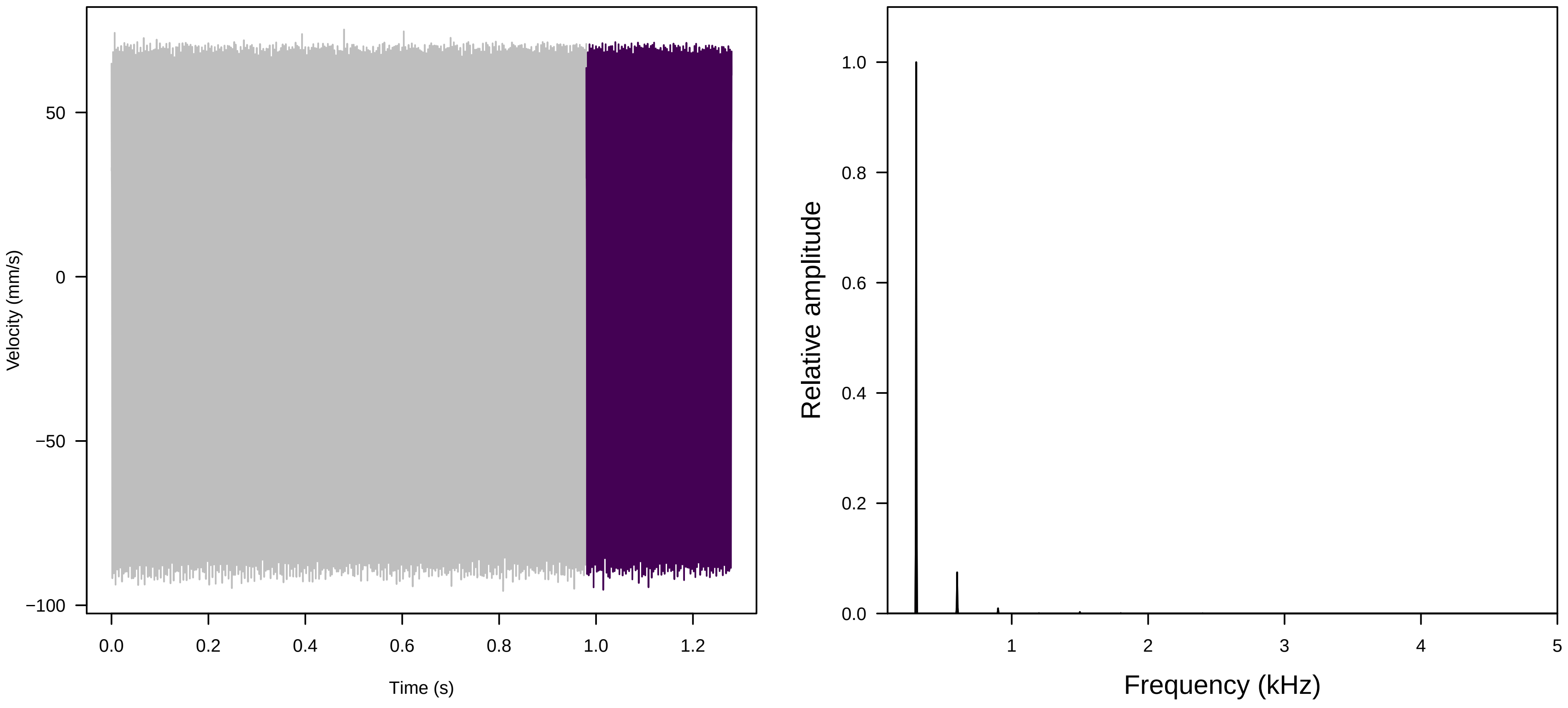

Vel. = 0.028 ; Str. = Receptacle ; Axis = x ; Fl. accession = 10−s−81−1AA

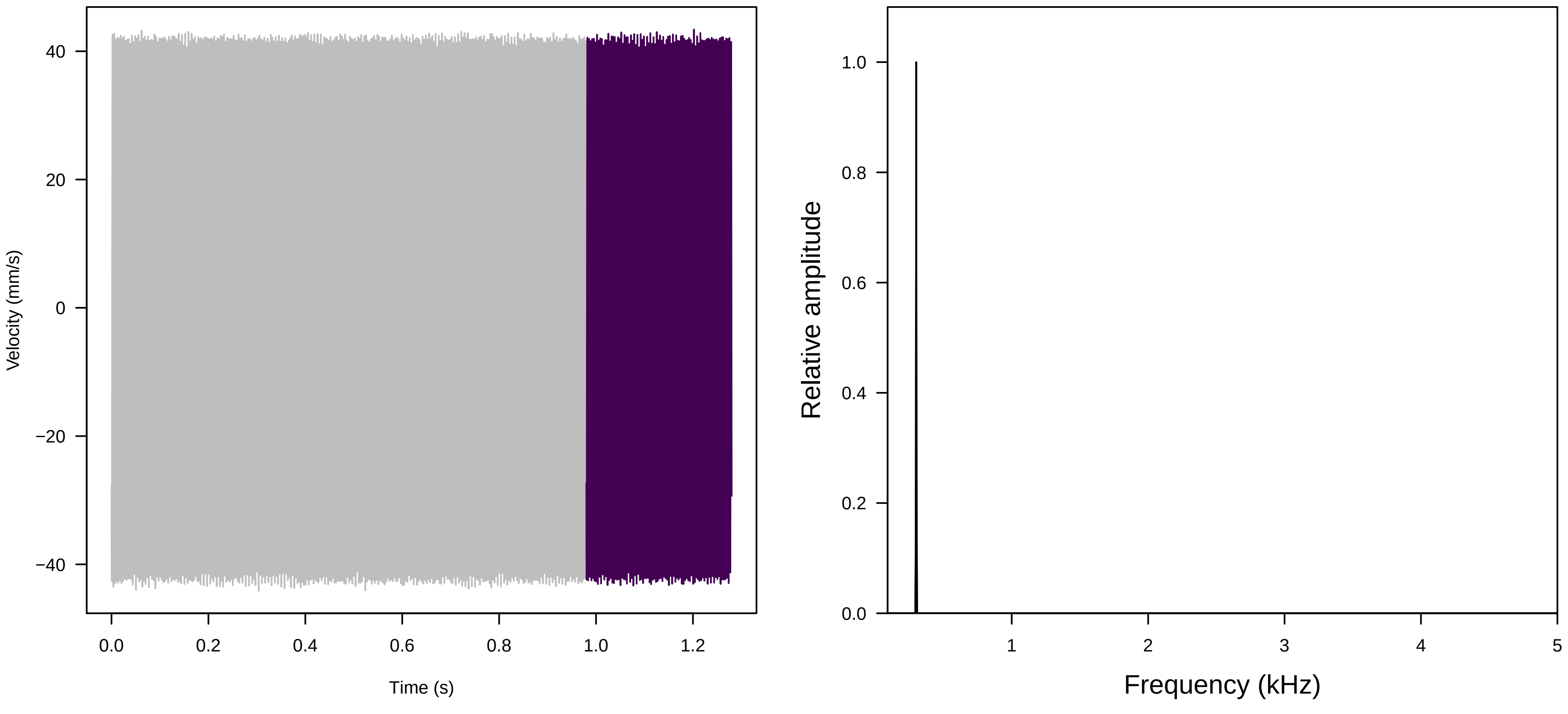

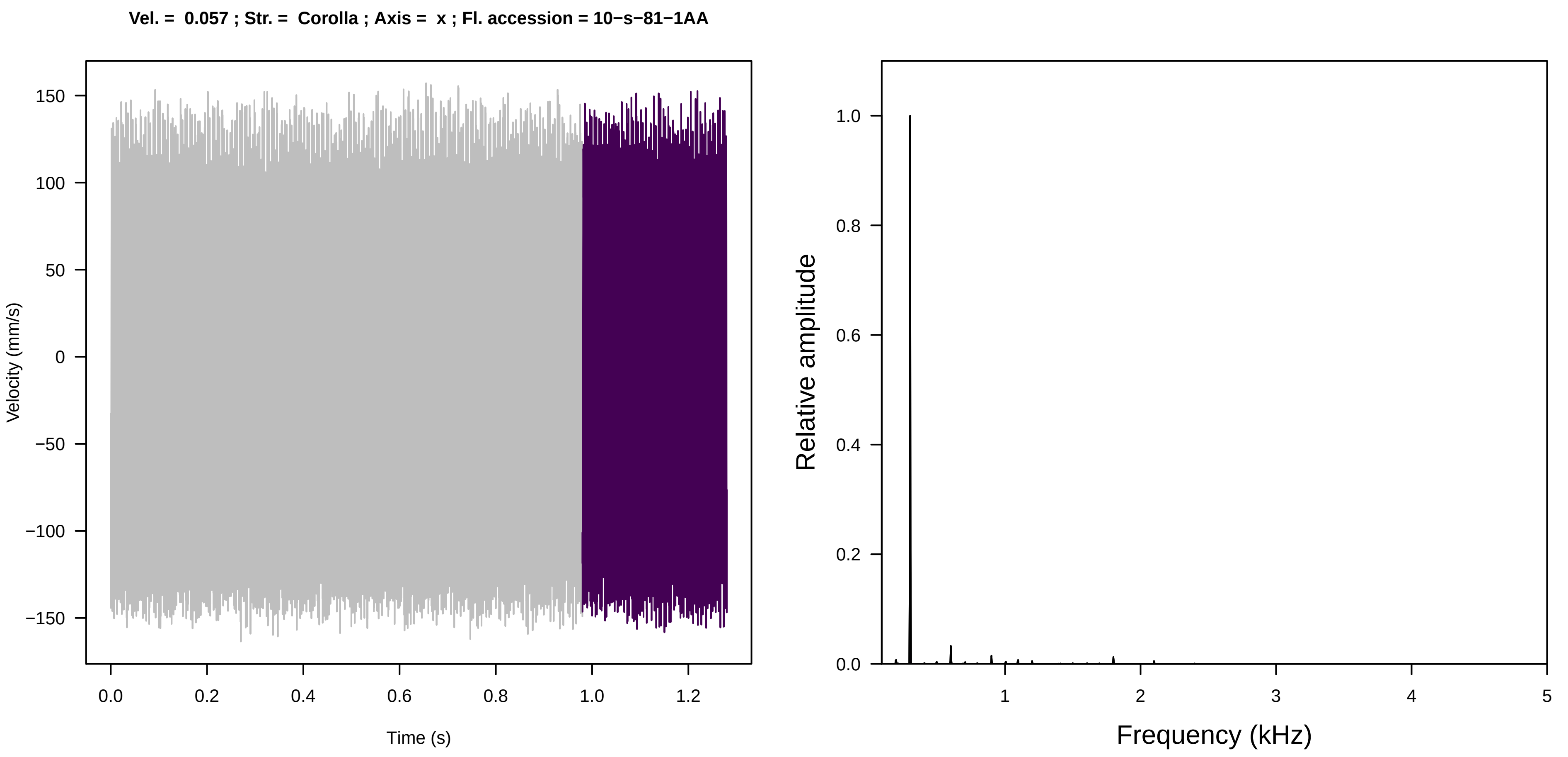
Vel. = 0.057 ; Str. = Corolla ; Axis = x ; Fl. accession = 10−s−81−1AA
Velocity (mm/s)
Time (s)
Relative amplitude
Frequency (kHz)

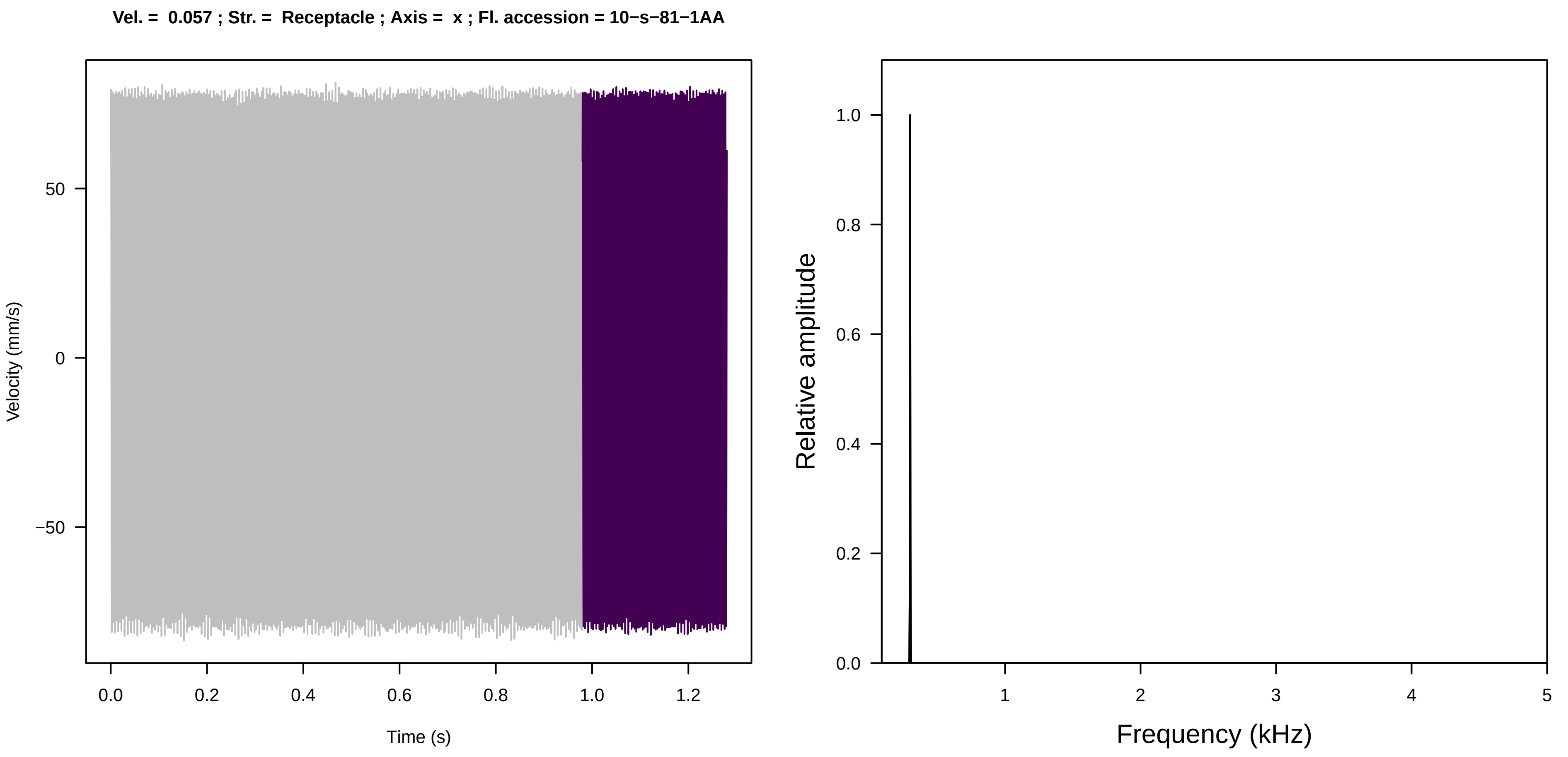
Vel. = 0.057 ; Str. = Receptacle ; Axis = x ; Fl. accession = 10−s−81−1AA
Velocity (mm/s)
Time (s)
Relative amplitude
Frequency (kHz)

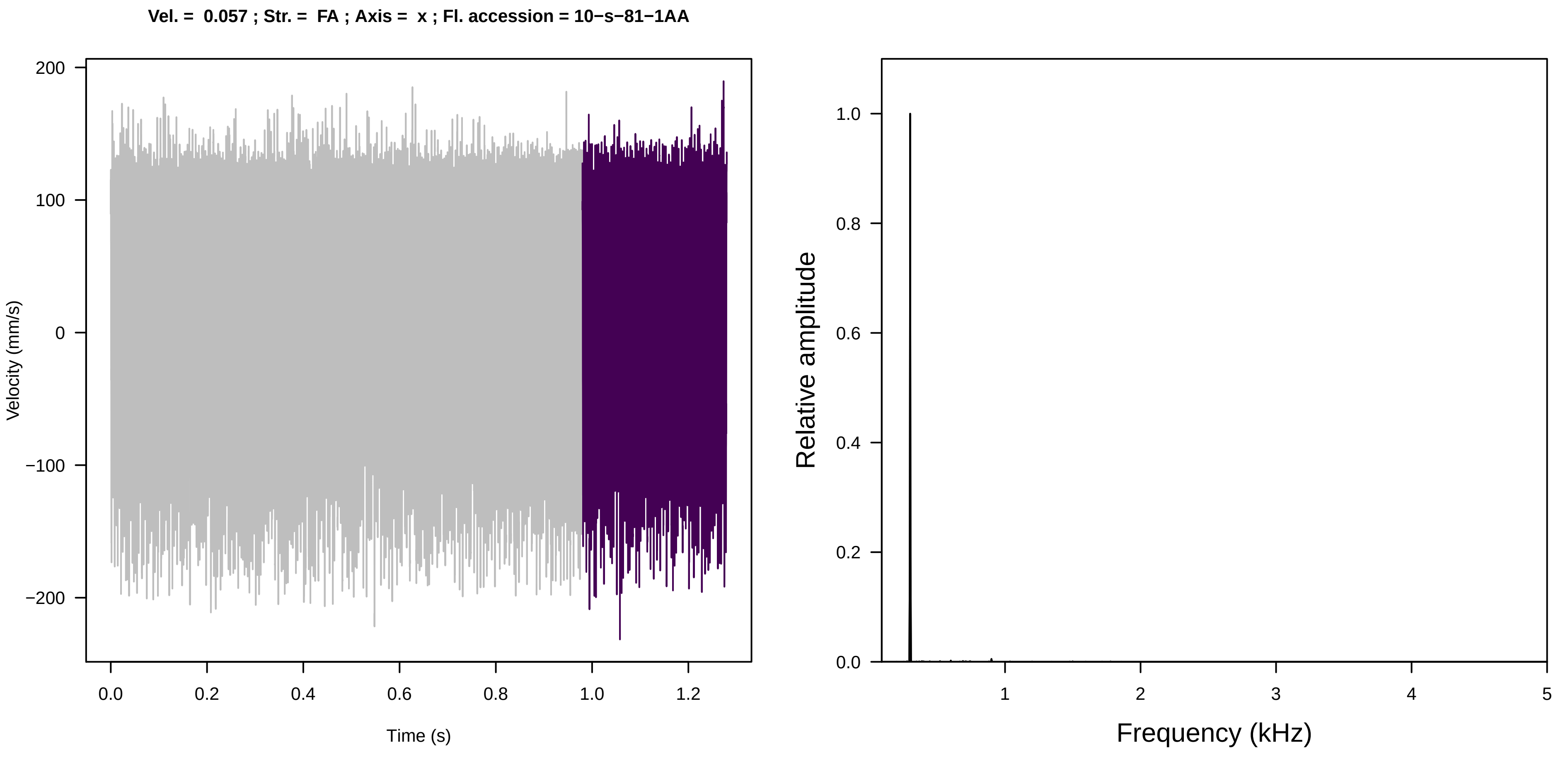
Vel. = 0.057 ; Str. = FA ; Axis = x ; Fl. accession = 10−s−81−1AA
Velocity (mm/s)
Time (s)
Relative amplitude
Frequency (kHz)

Vel. = 0.057 ; Str. = Receptacle ; Axis = x ; Fl. accession = 10−s−81−1AA

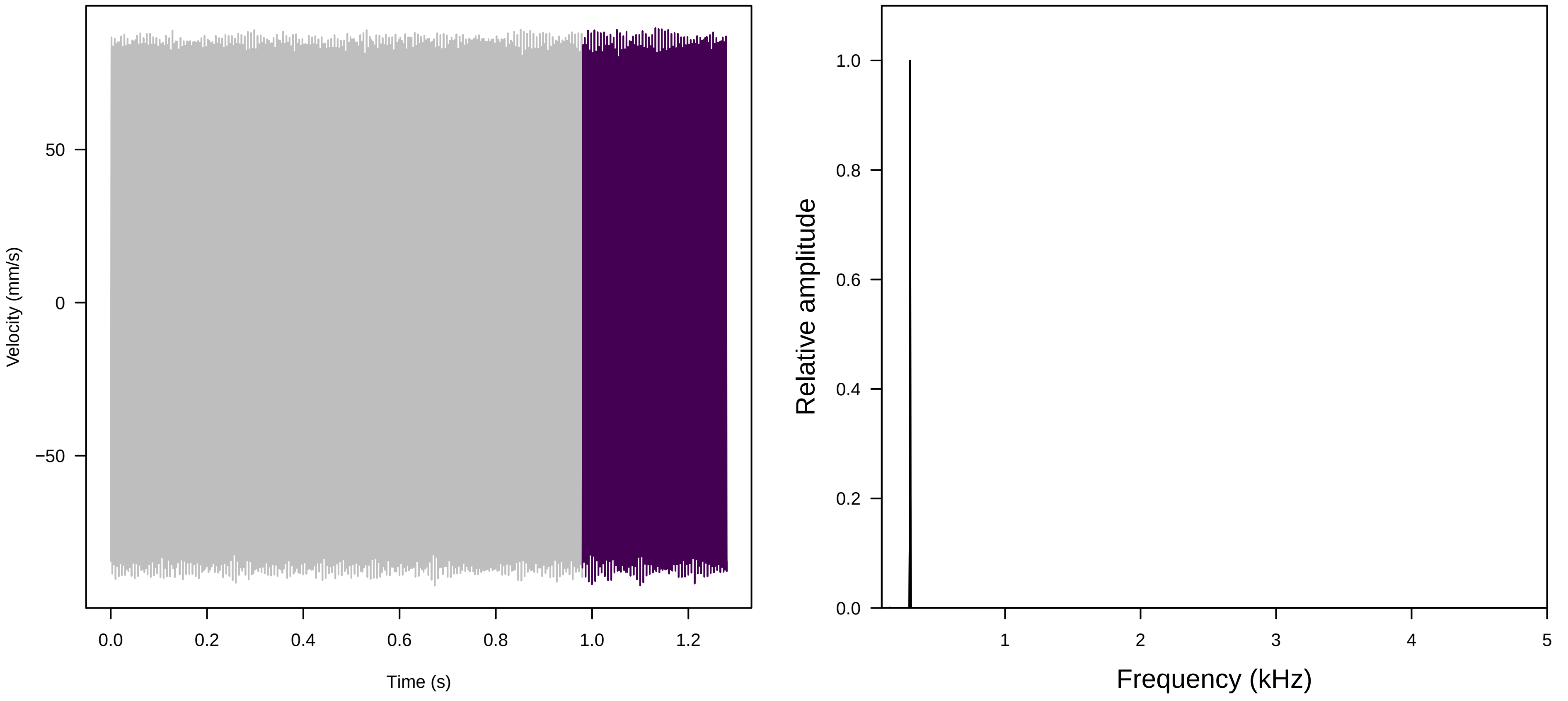

Vel. = 0.057 ; Str. = PA ; Axis = x ; Fl. accession = 10−s−81−1AA

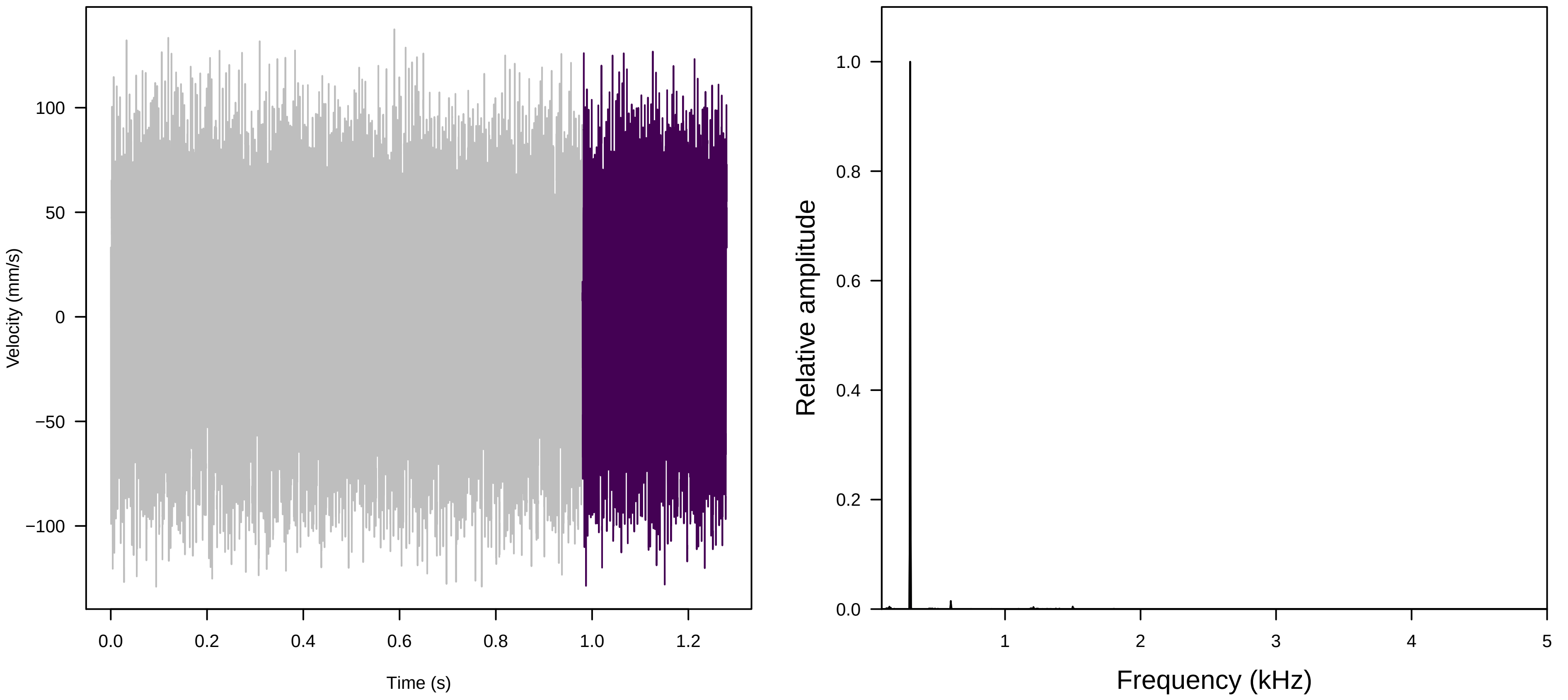

Vel. = 0.057 ; Str. = Receptacle ; Axis = x ; Fl. accession = 10−s−81−1AA

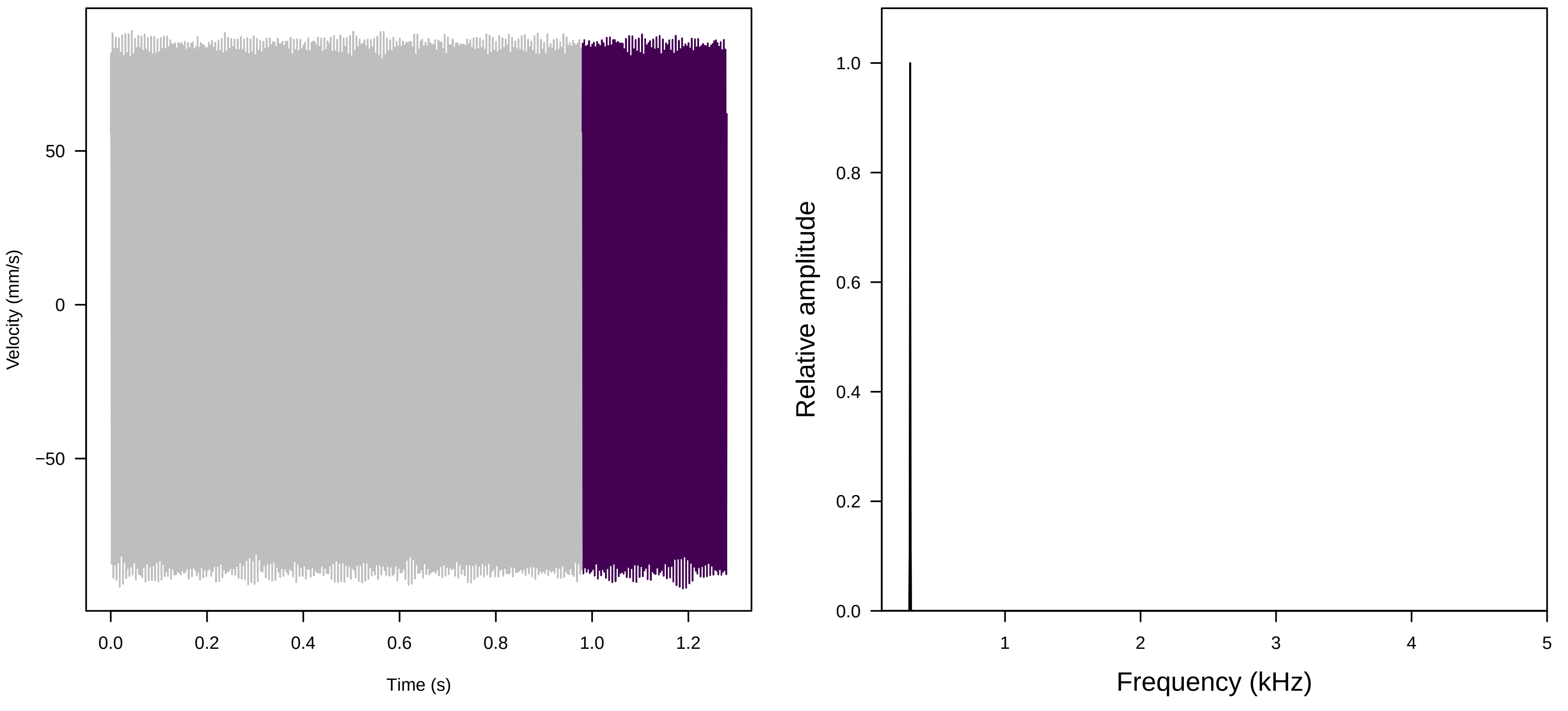

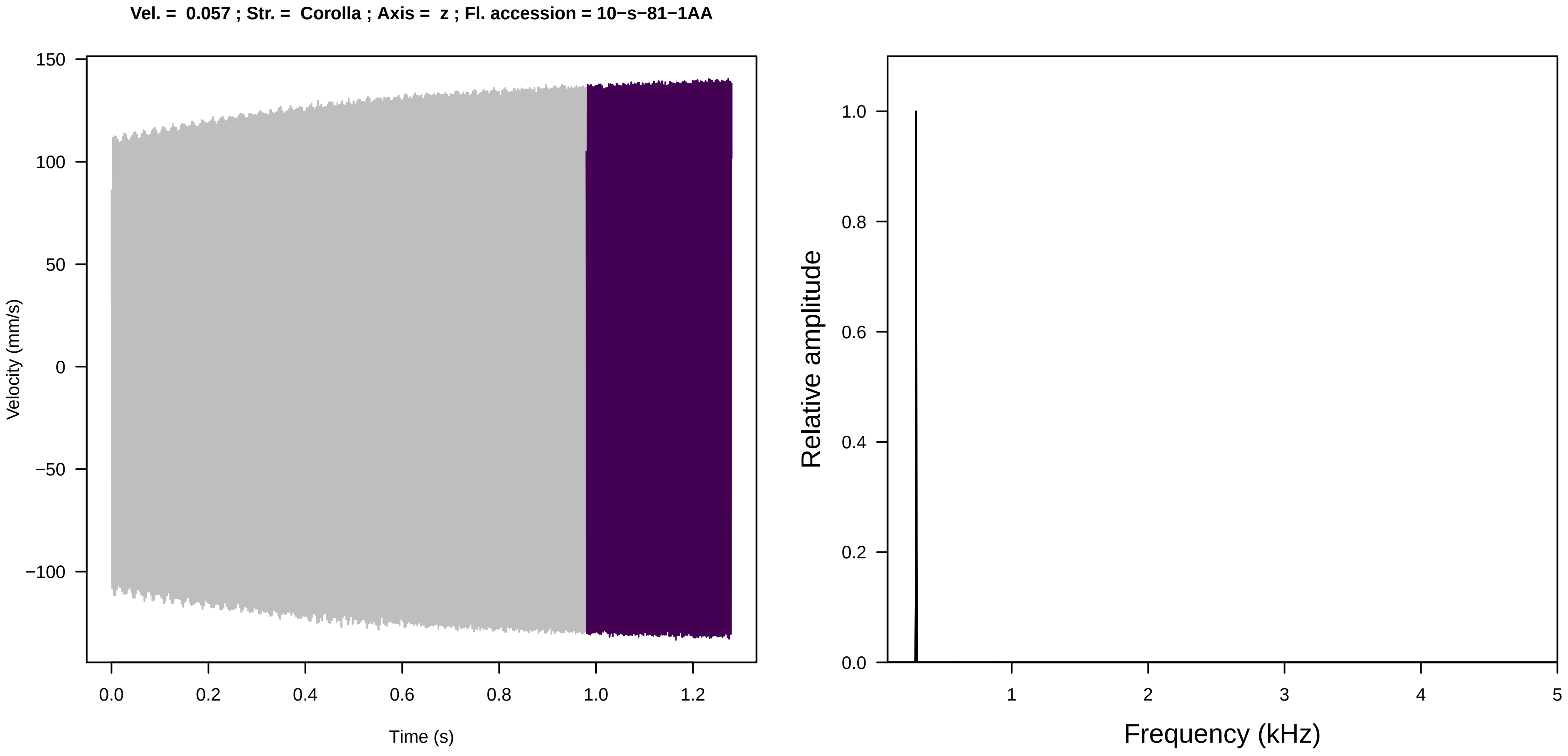
Vel. = 0.057 ; Str. = Corolla ; Axis = z ; Fl. accession = 10−s−81−1AA
Velocity (mm/s)
Time (s)
Relative amplitude
Frequency (kHz)

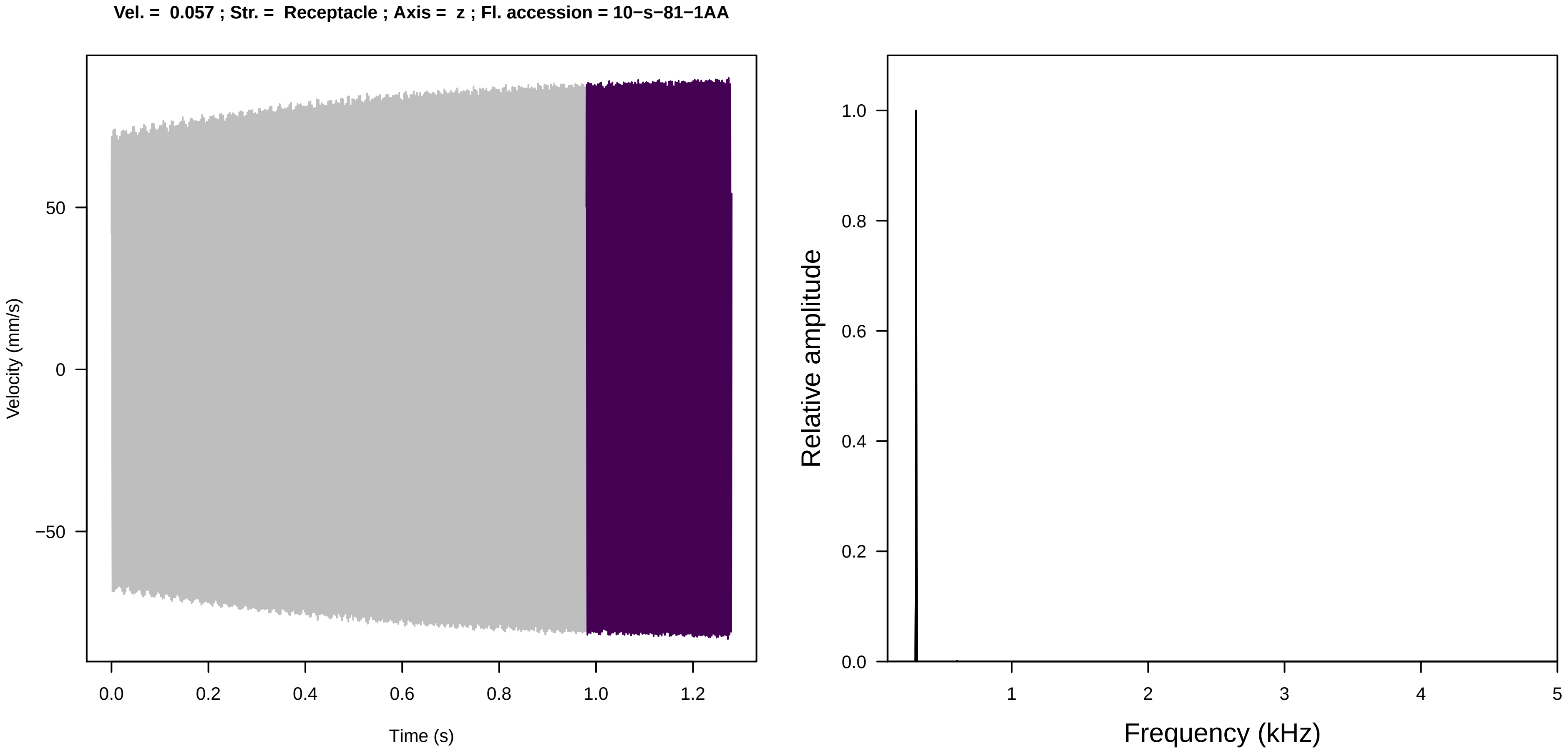
Vel. = 0.057 ; Str. = Receptacle ; Axis = z ; Fl. accession = 10−s−81−1AA
Velocity (mm/s)
Time (s)
Relative amplitude
Frequency (kHz)

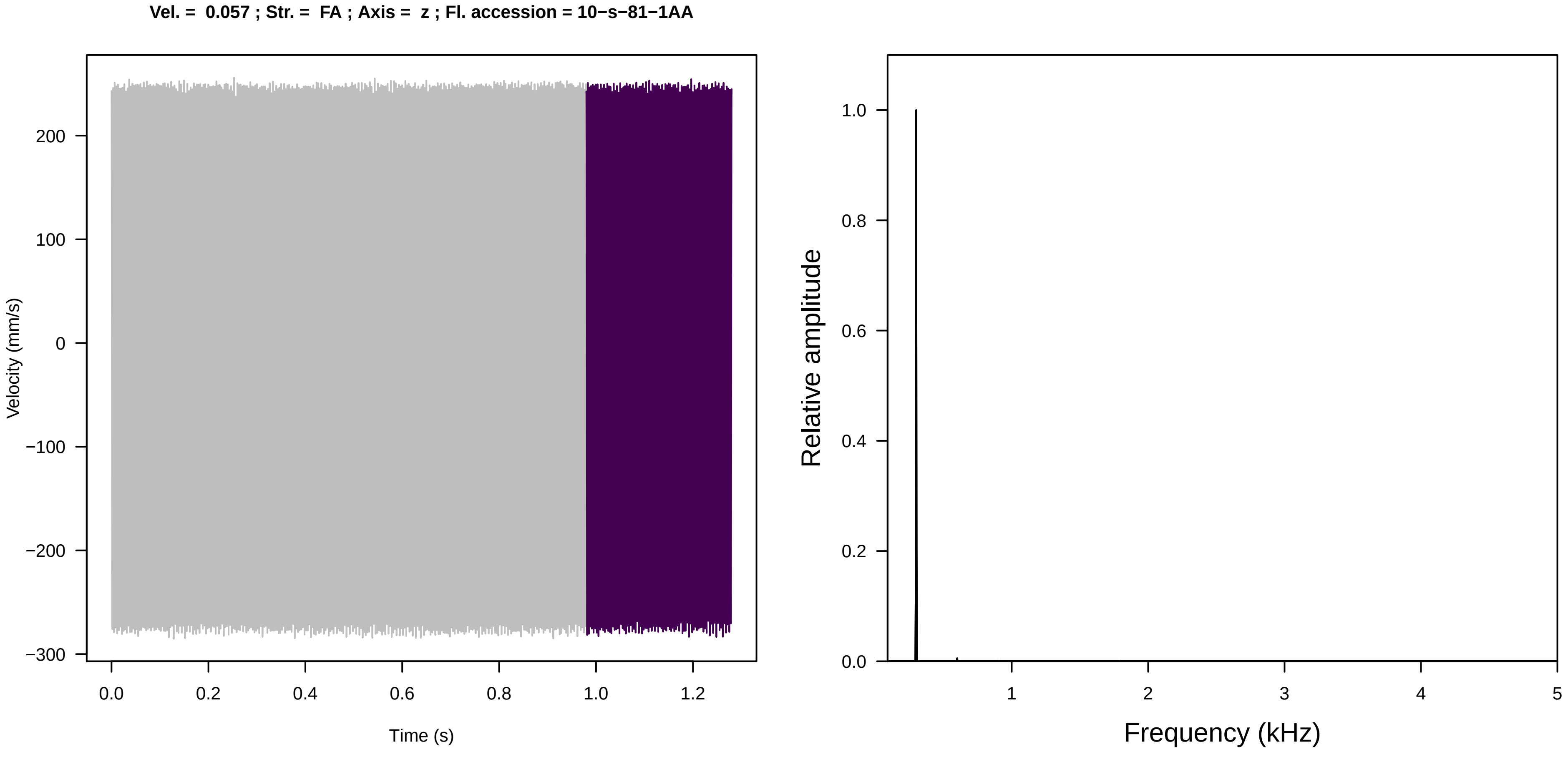
Vel. = 0.057 ; Str. = FA ; Axis = z ; Fl. accession = 10−s−81−1AA
Velocity (mm/s)
Time (s)
Relative amplitude
Frequency (kHz)

Vel. = 0.057 ; Str. = Receptacle ; Axis = z ; Fl. accession = 10−s−81−1AA

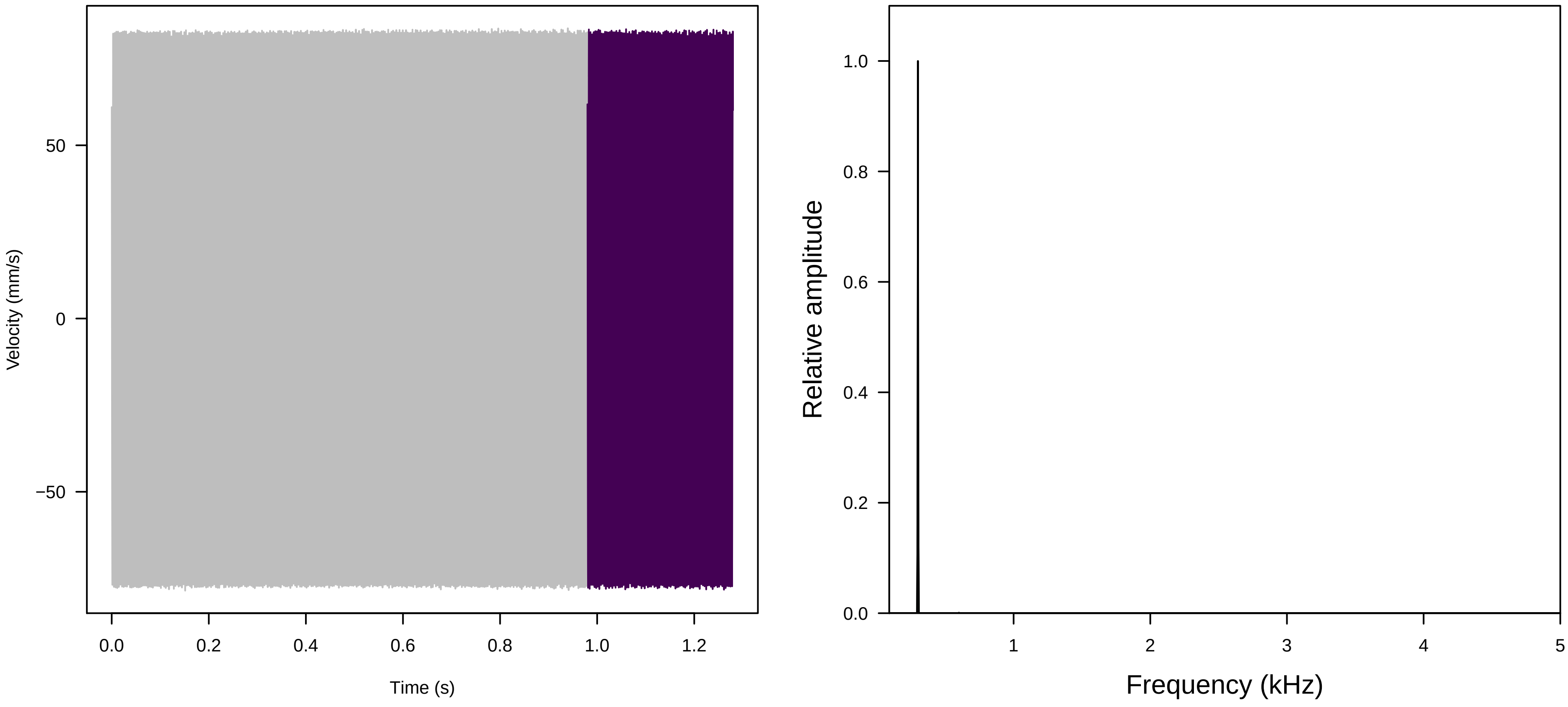

Vel. = 0.057 ; Str. = PA ; Axis = z ; Fl. accession = 10−s−81−1AA

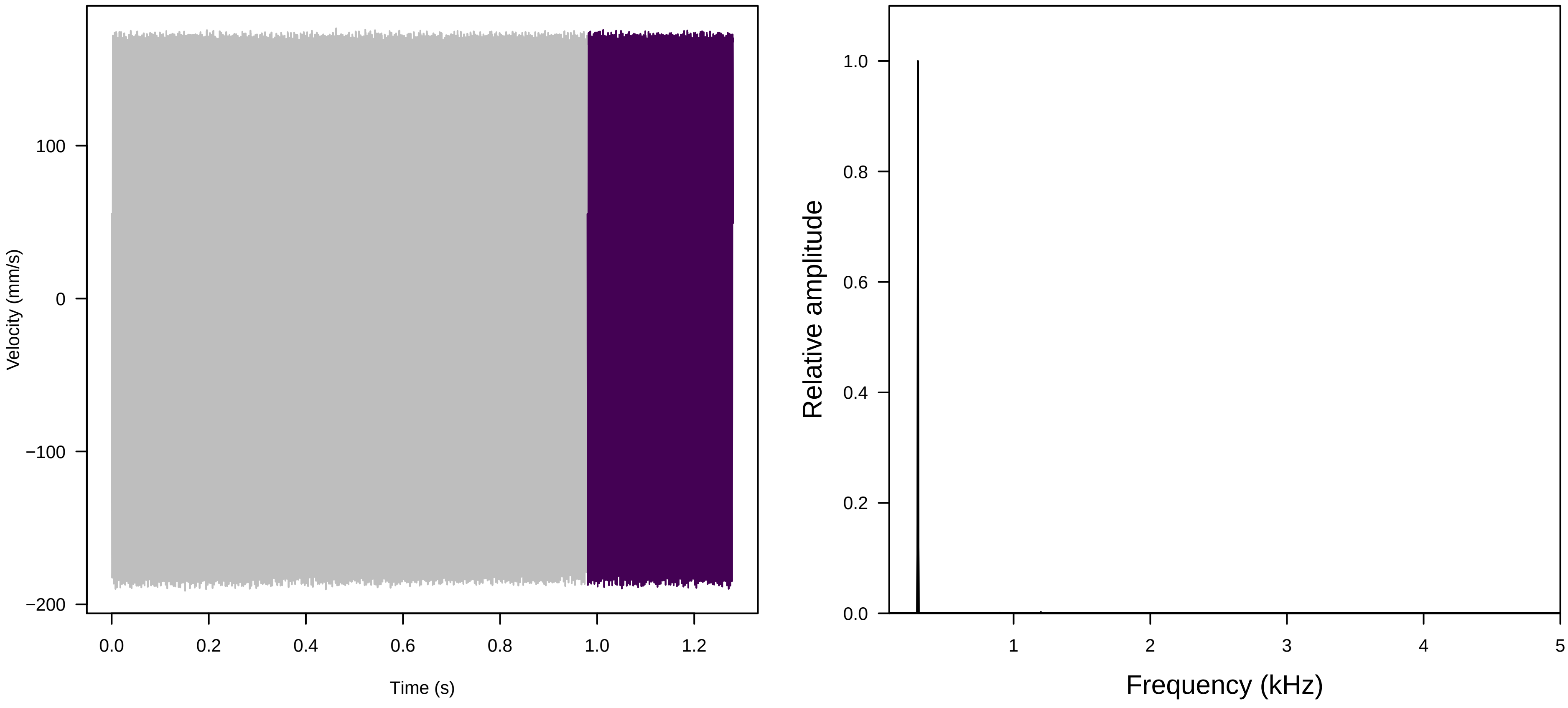

Vel. = 0.057 ; Str. = Receptacle ; Axis = z ; Fl. accession = 10−s−81−1AA

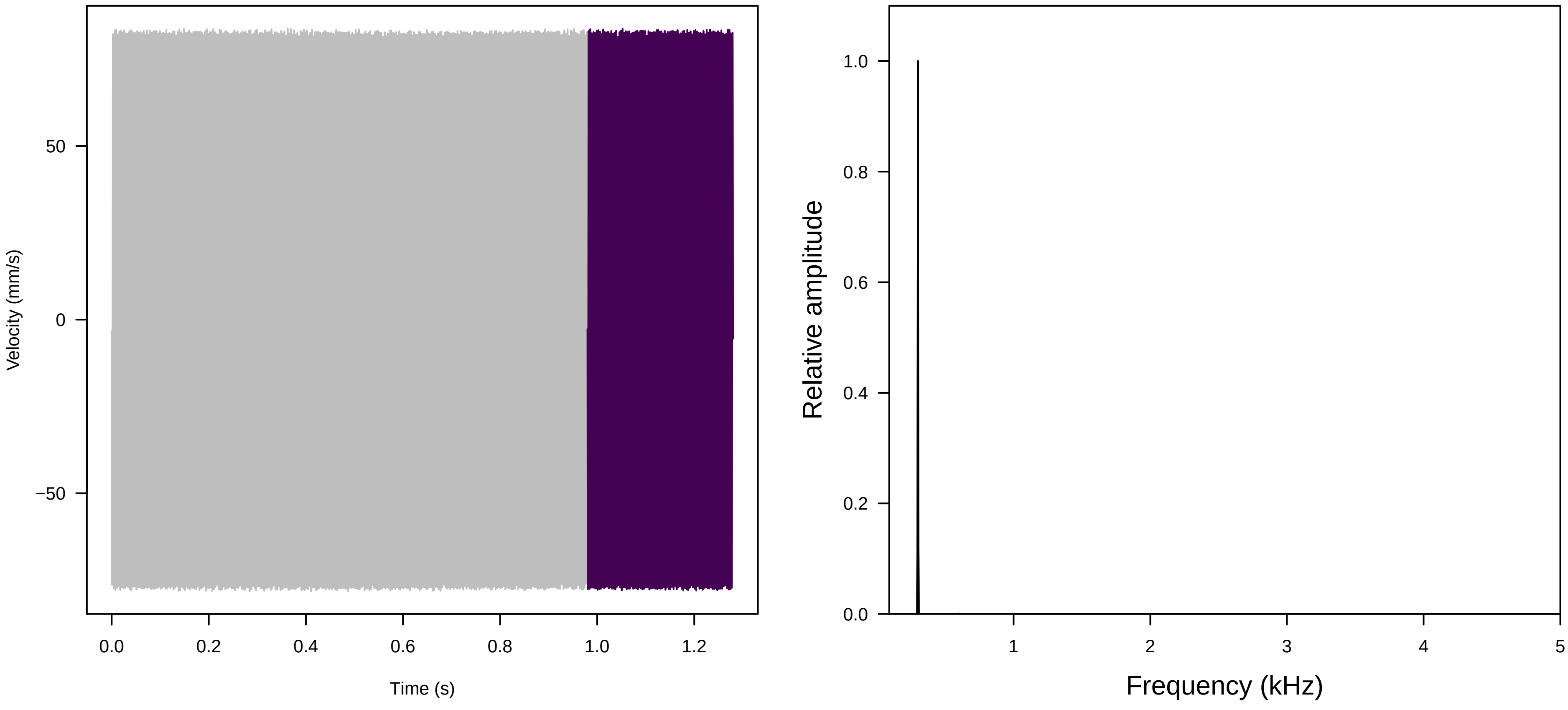

Vel. = 0.028 ; Str. = PA ; Axis = z ; Fl. accession = 10−s−81−1AA

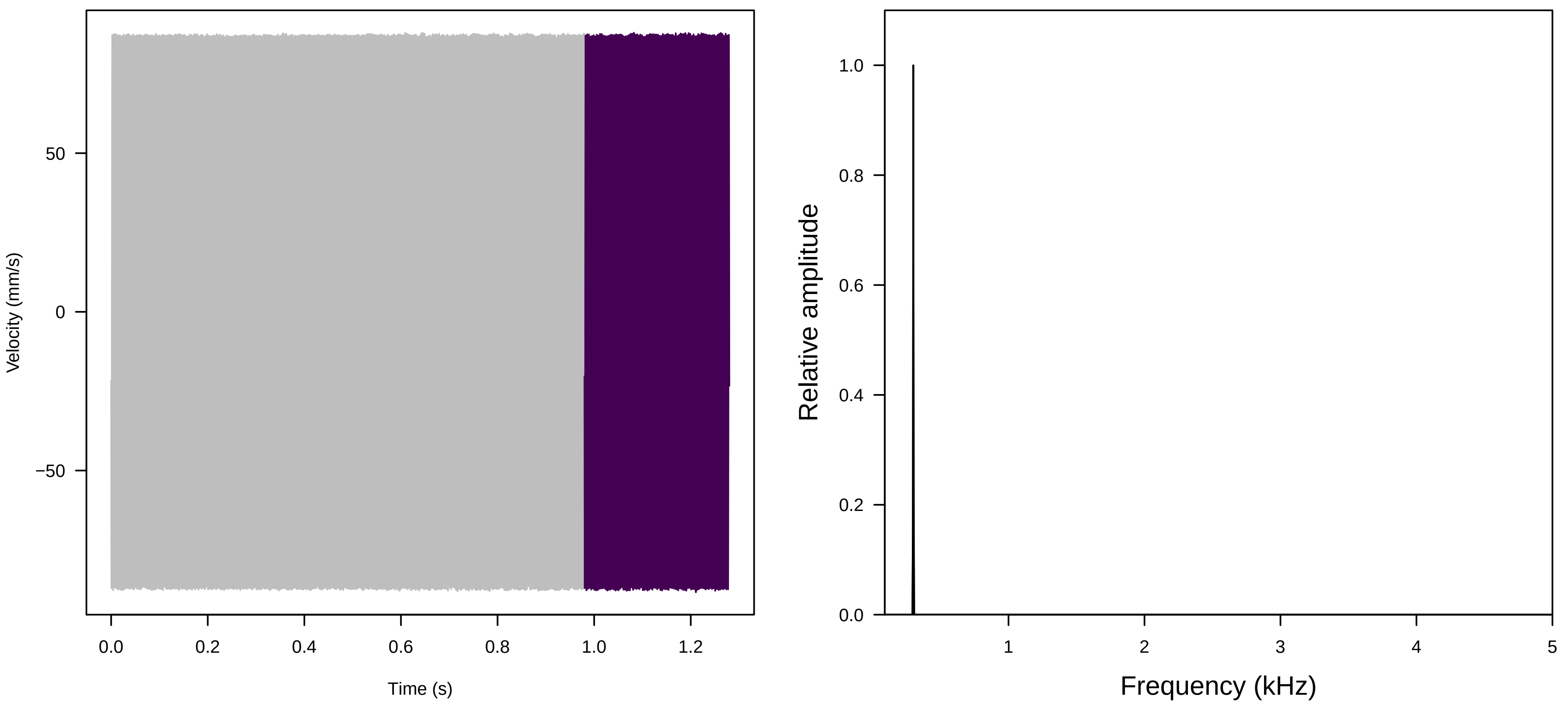

Vel. = 0.028 ; Str. = Receptacle ; Axis = z ; Fl. accession = 10−s−81−1AA

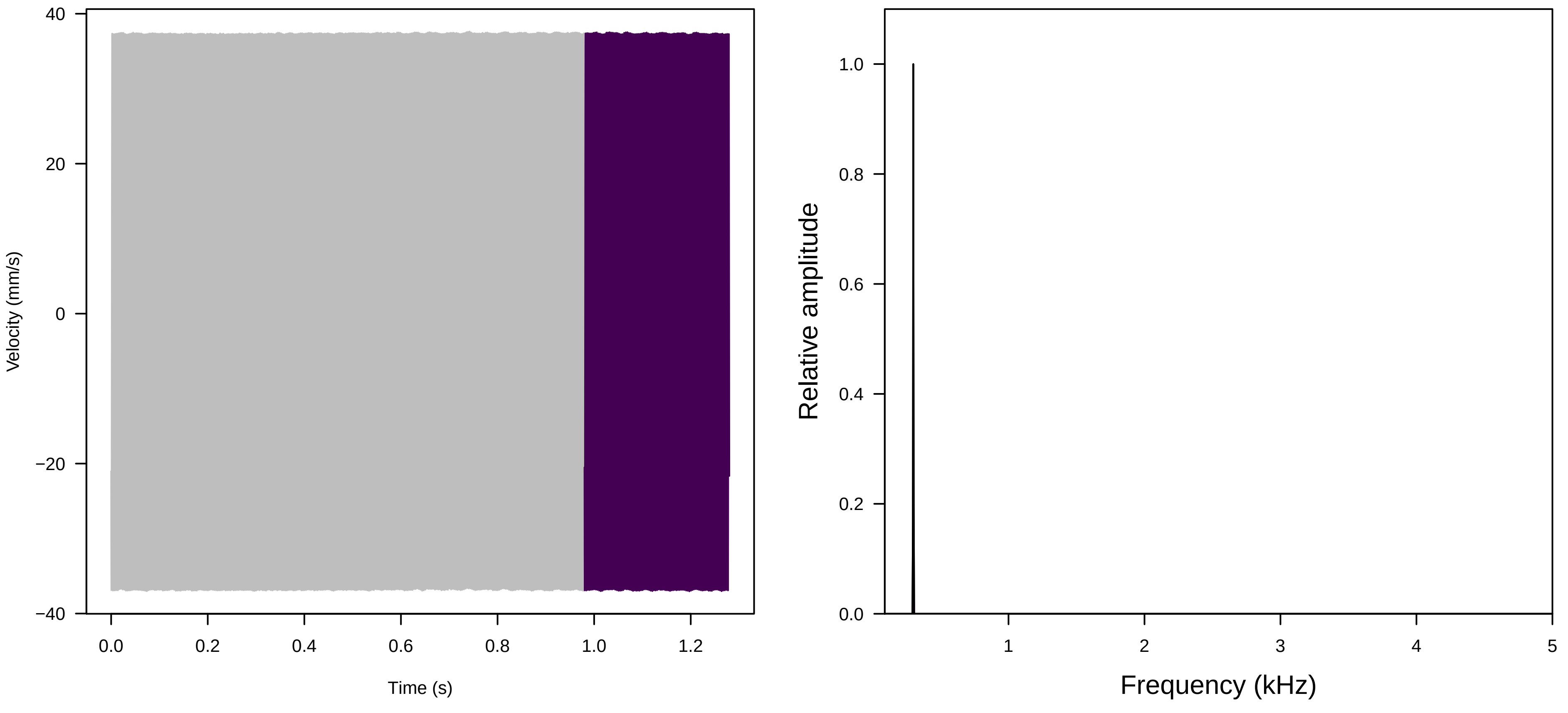

Vel. = 0.028 ; Str. = FA ; Axis = z ; Fl. accession = 10−s−81−1AA

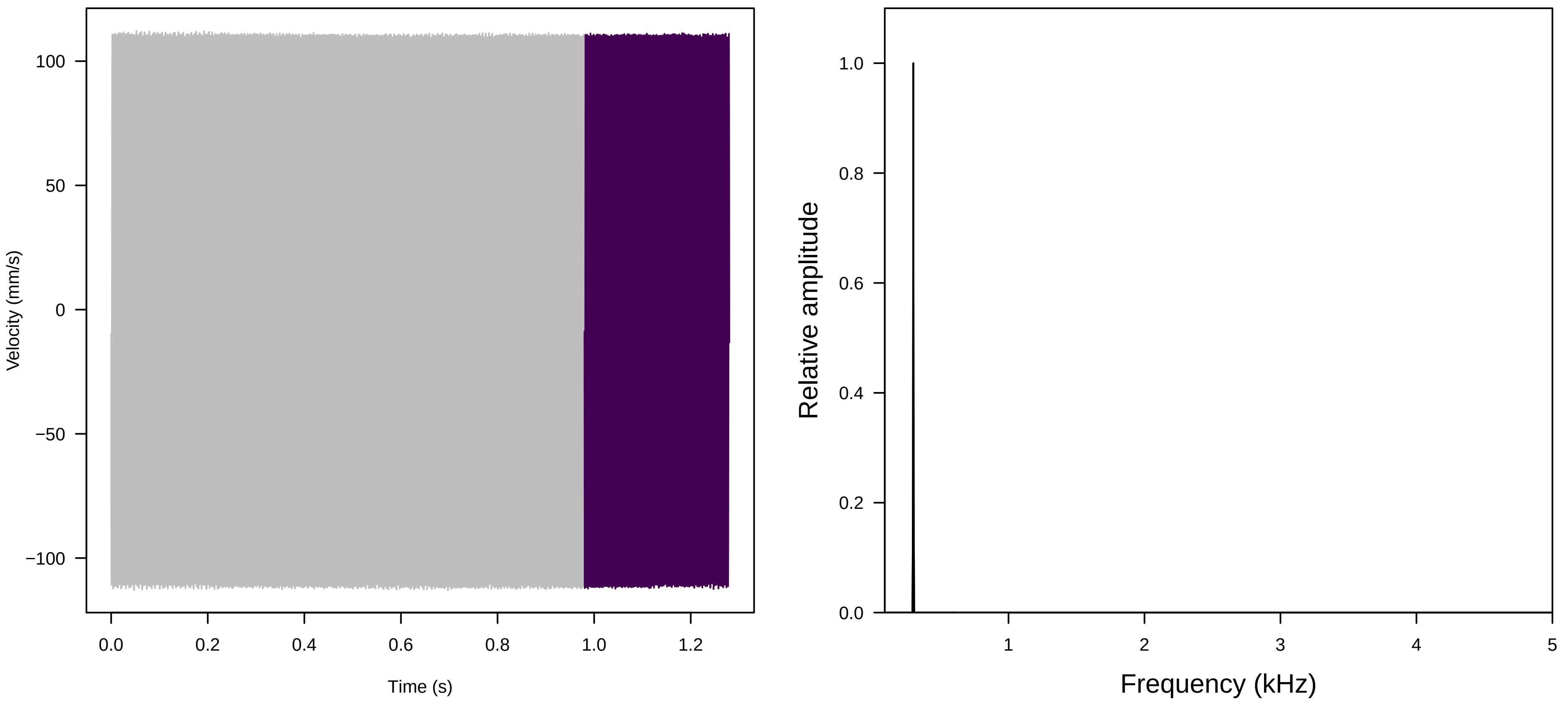

Vel. = 0.028 ; Str. = Receptacle ; Axis = z ; Fl. accession = 10−s−81−1AA

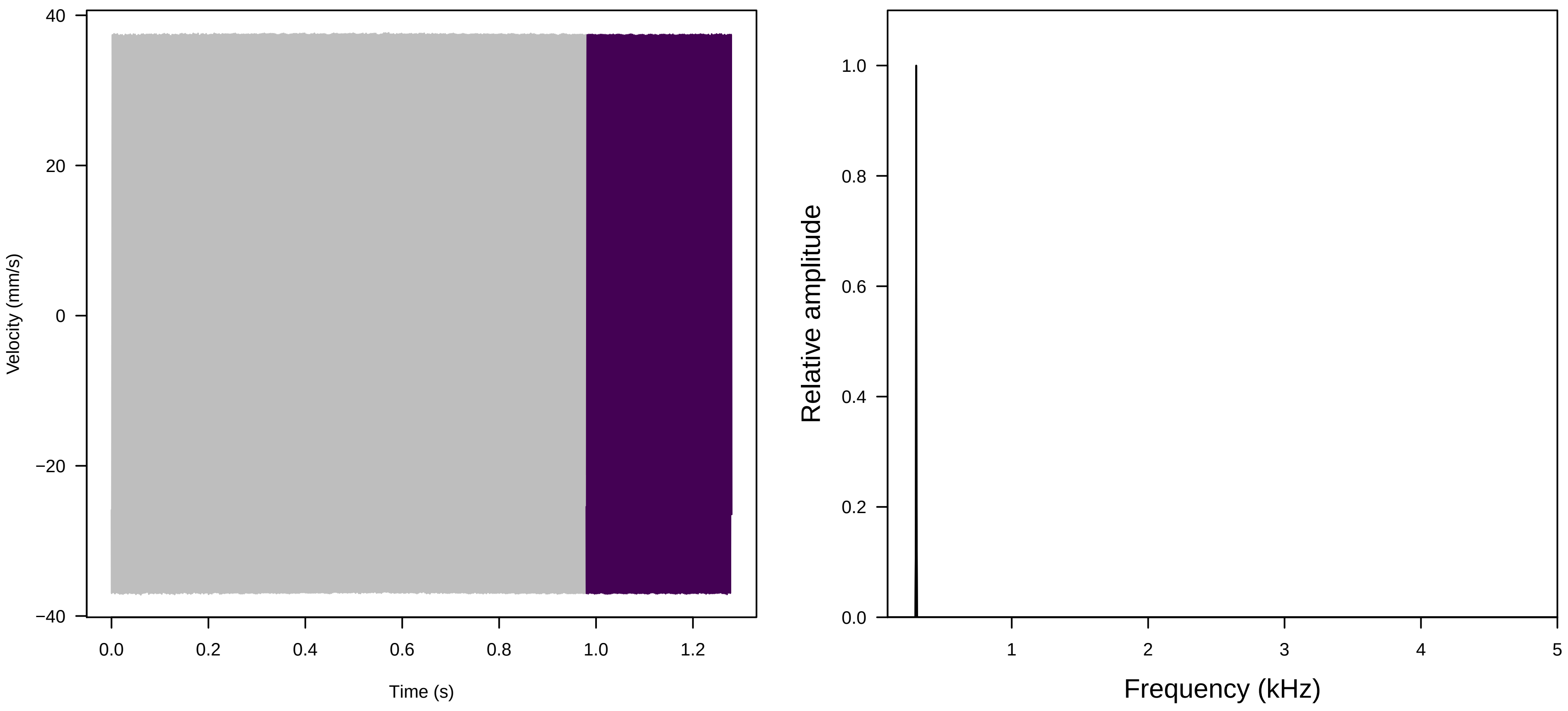

Vel. = 0.028 ; Str. = Corolla ; Axis = z ; Fl. accession = 10−s−81−1AA

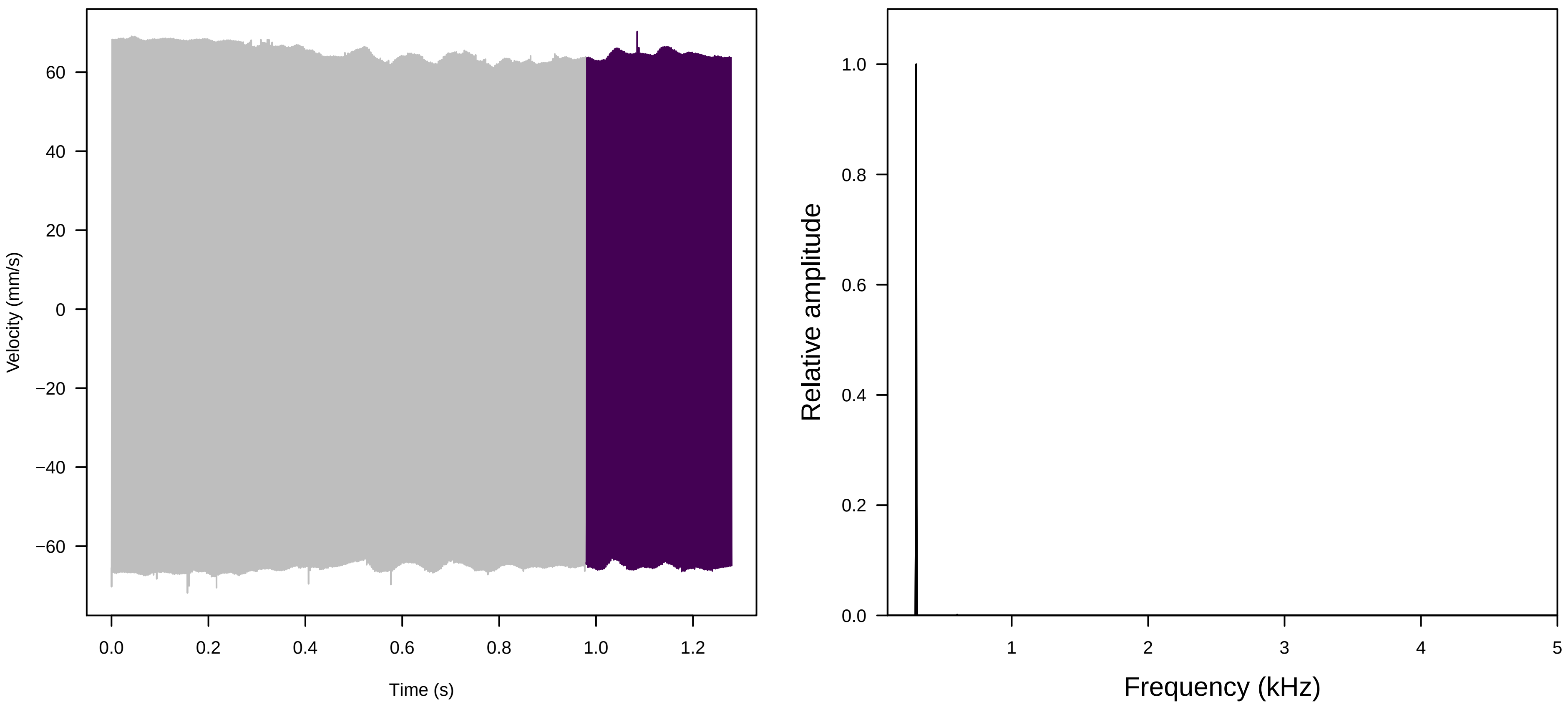

Vel. = 0.028 ; Str. = Receptacle ; Axis = z ; Fl. accession = 10−s−81−1AA

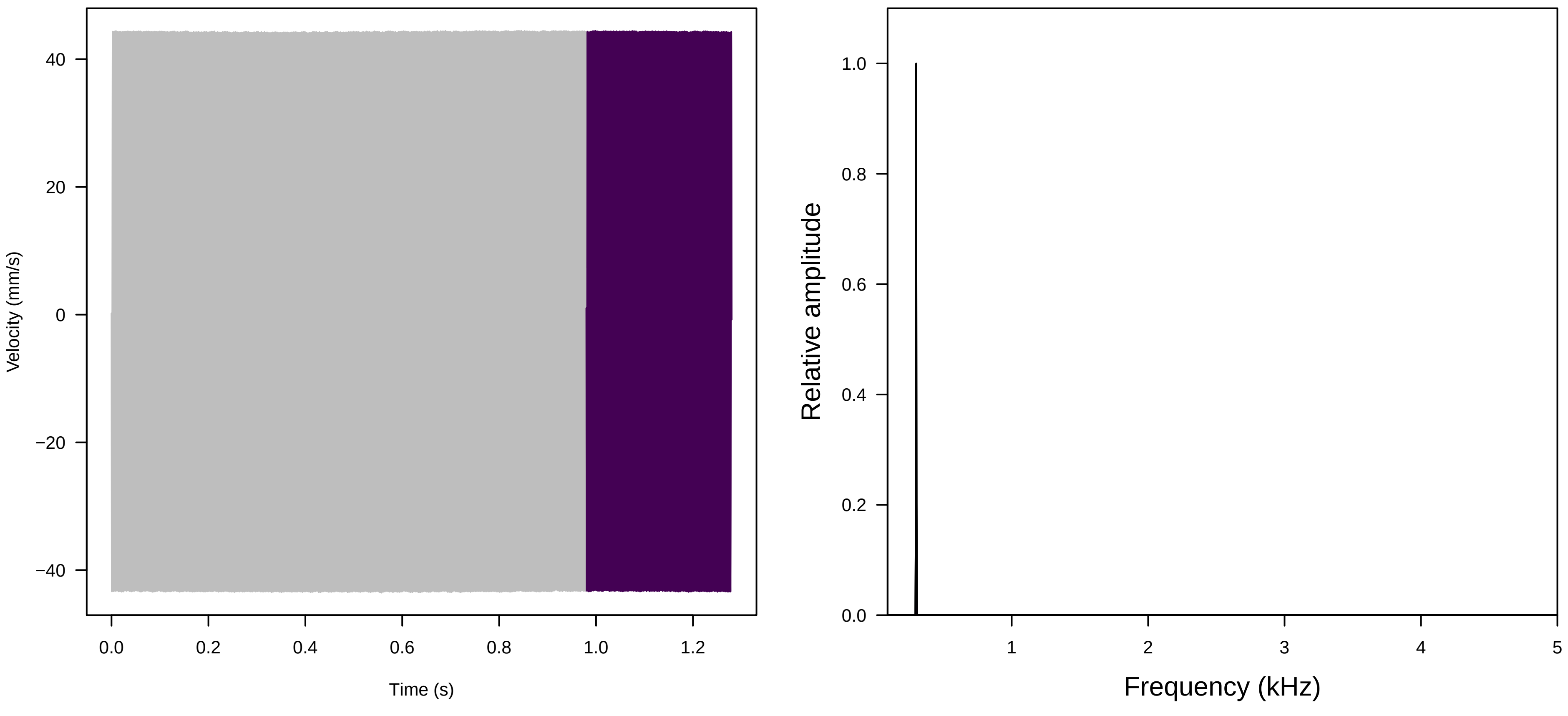

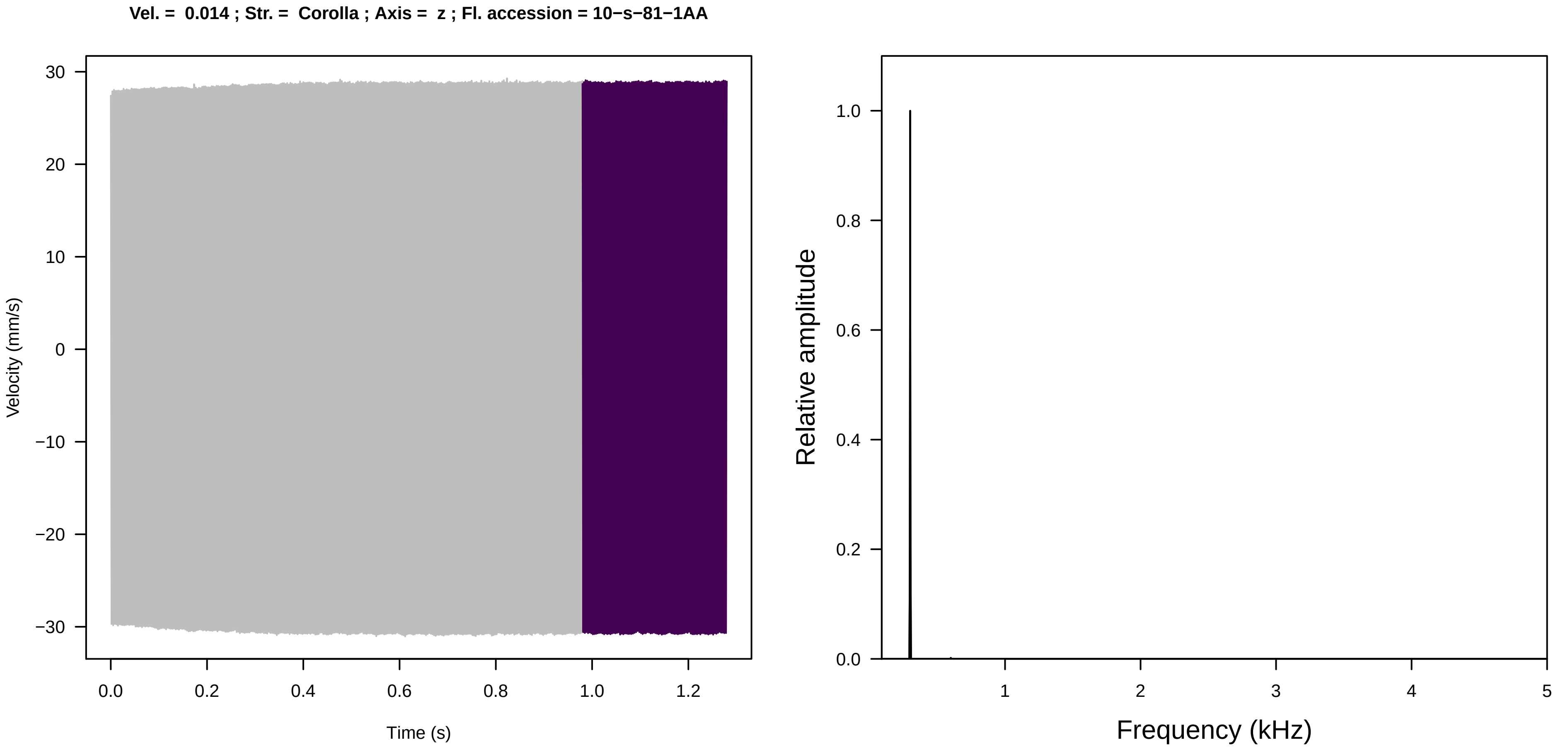
Vel. = 0.014 ; Str. = Corolla ; Axis = z ; Fl. accession = 10−s−81−1AA
Velocity (mm/s)
Time (s)
Relative amplitude
Frequency (kHz)

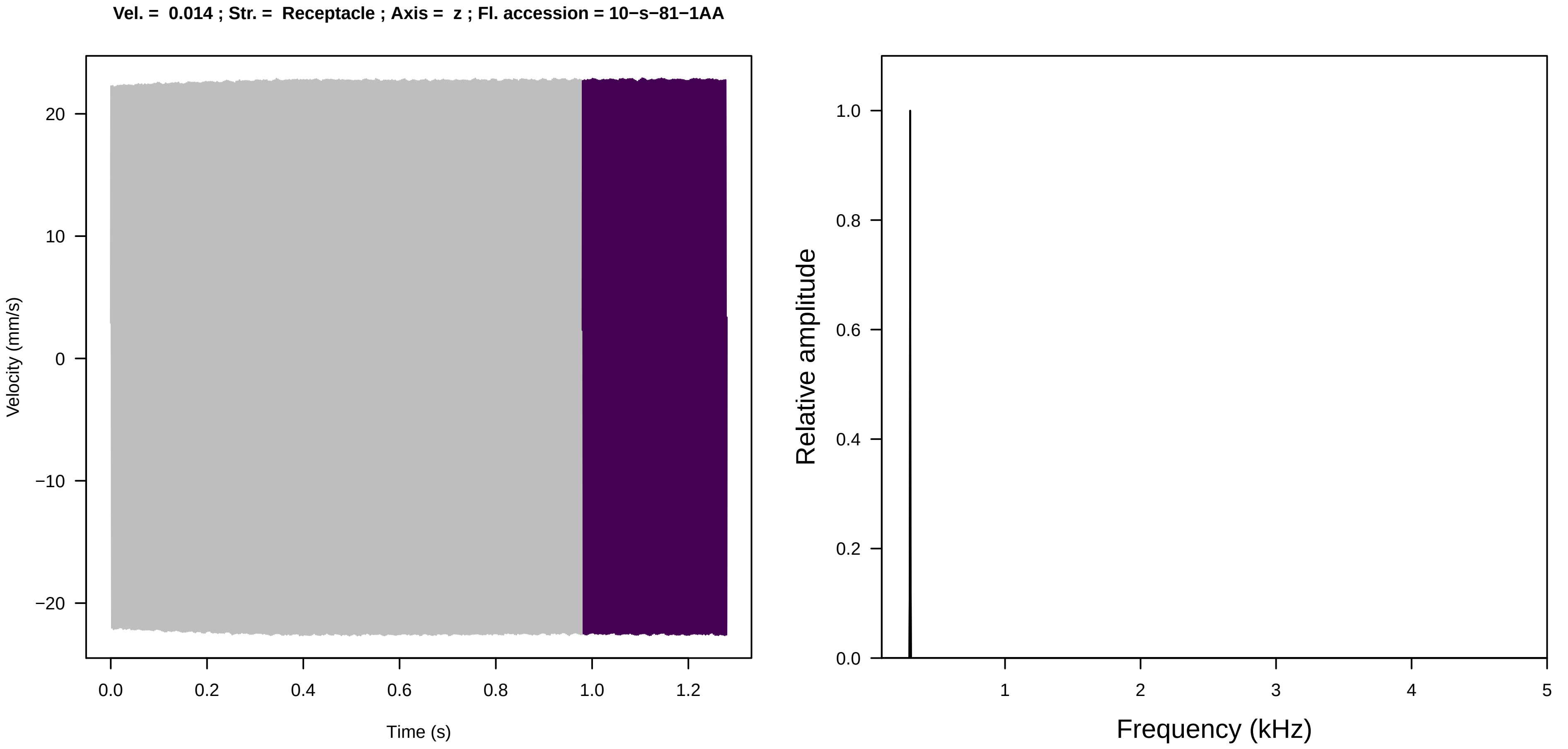
Vel. = 0.014 ; Str. = Receptacle ; Axis = z ; Fl. accession = 10−s−81−1AA
Velocity (mm/s)
Time (s)
Relative amplitude
Frequency (kHz)

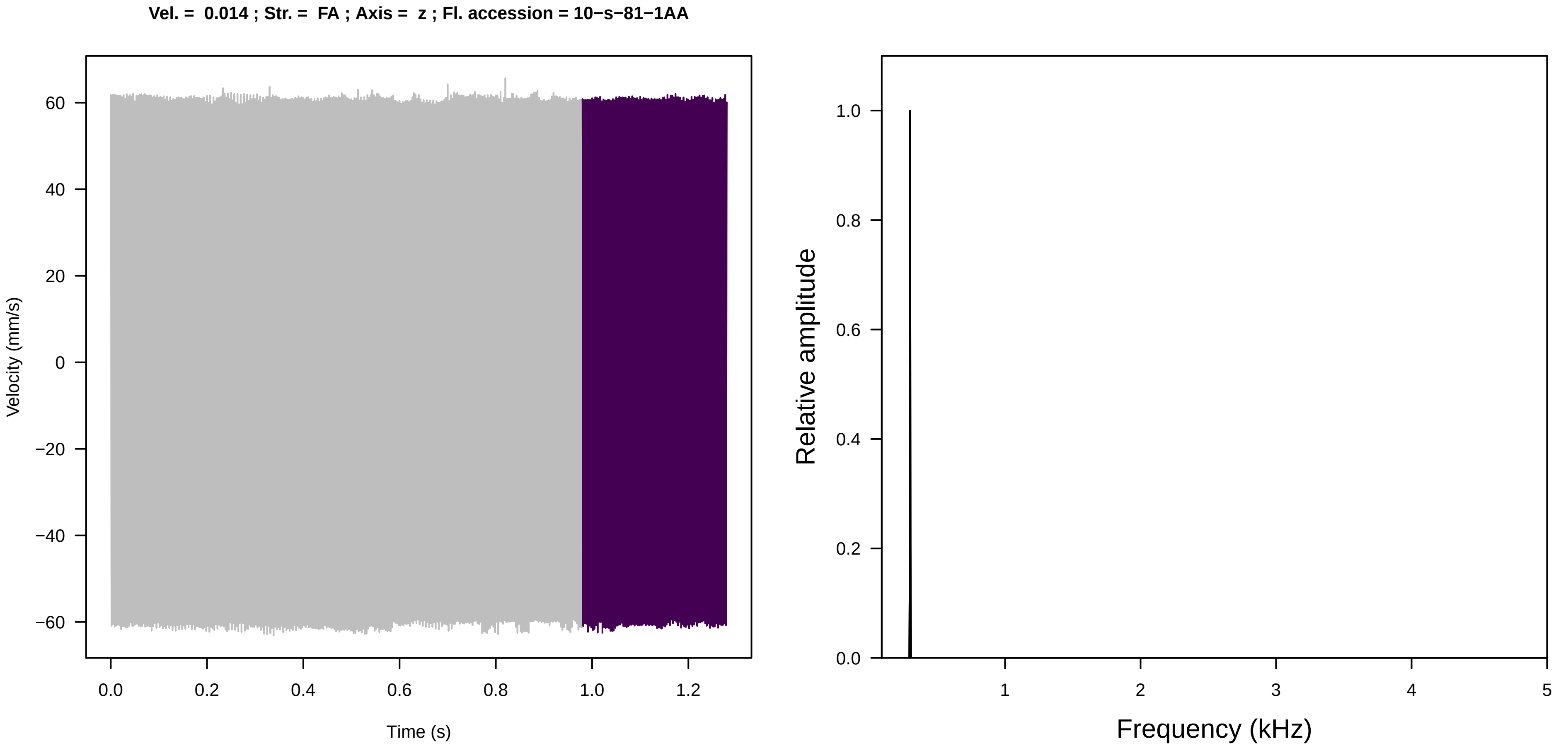
Vel. = 0.014 ; Str. = FA ; Axis = z ; Fl. accession = 10−s−81−1AA
Velocity (mm/s)
Time (s)
Relative amplitude
Frequency (kHz)

Vel. = 0.014 ; Str. = Receptacle ; Axis = z ; Fl. accession = 10−s−81−1AA

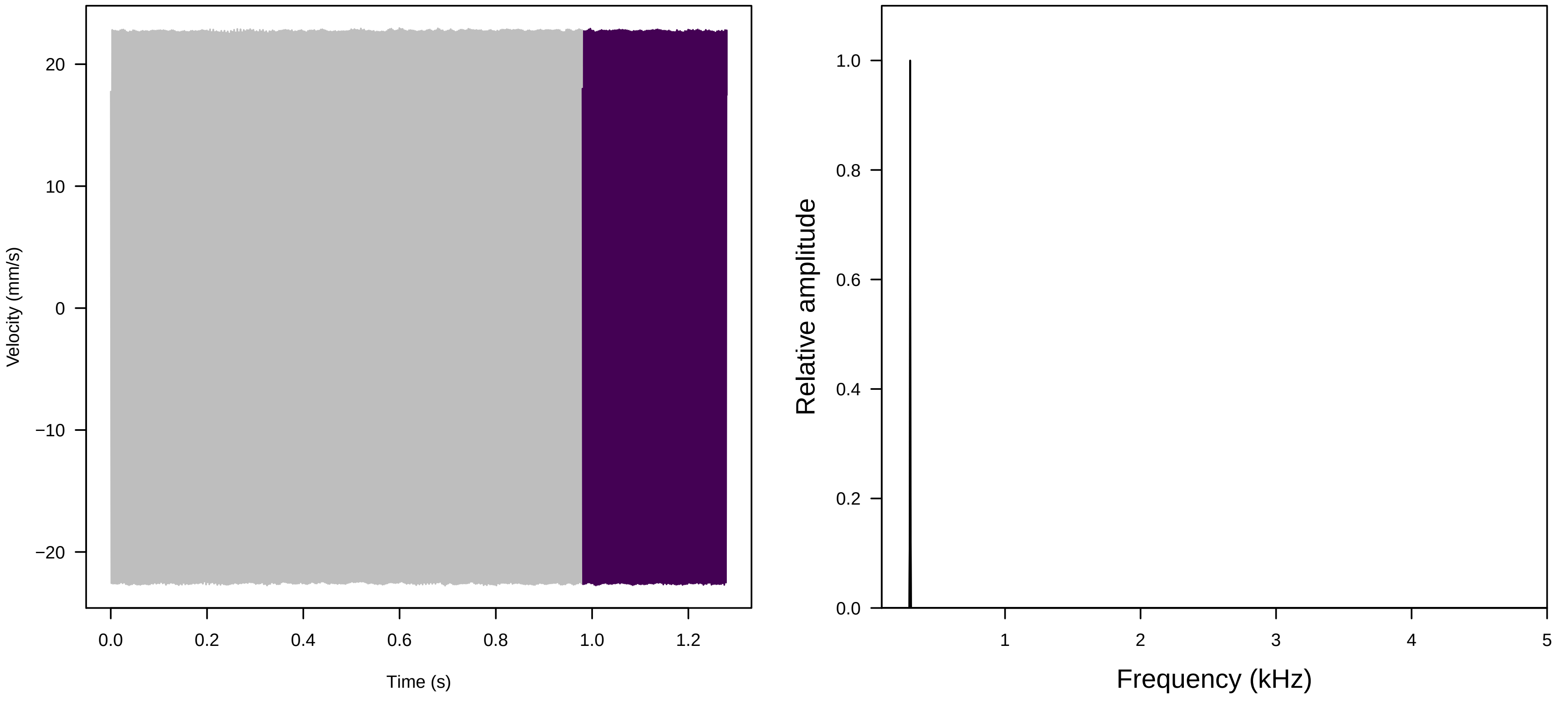

Vel. = 0.014 ; Str. = PA ; Axis = z ; Fl. accession = 10−s−81−1AA

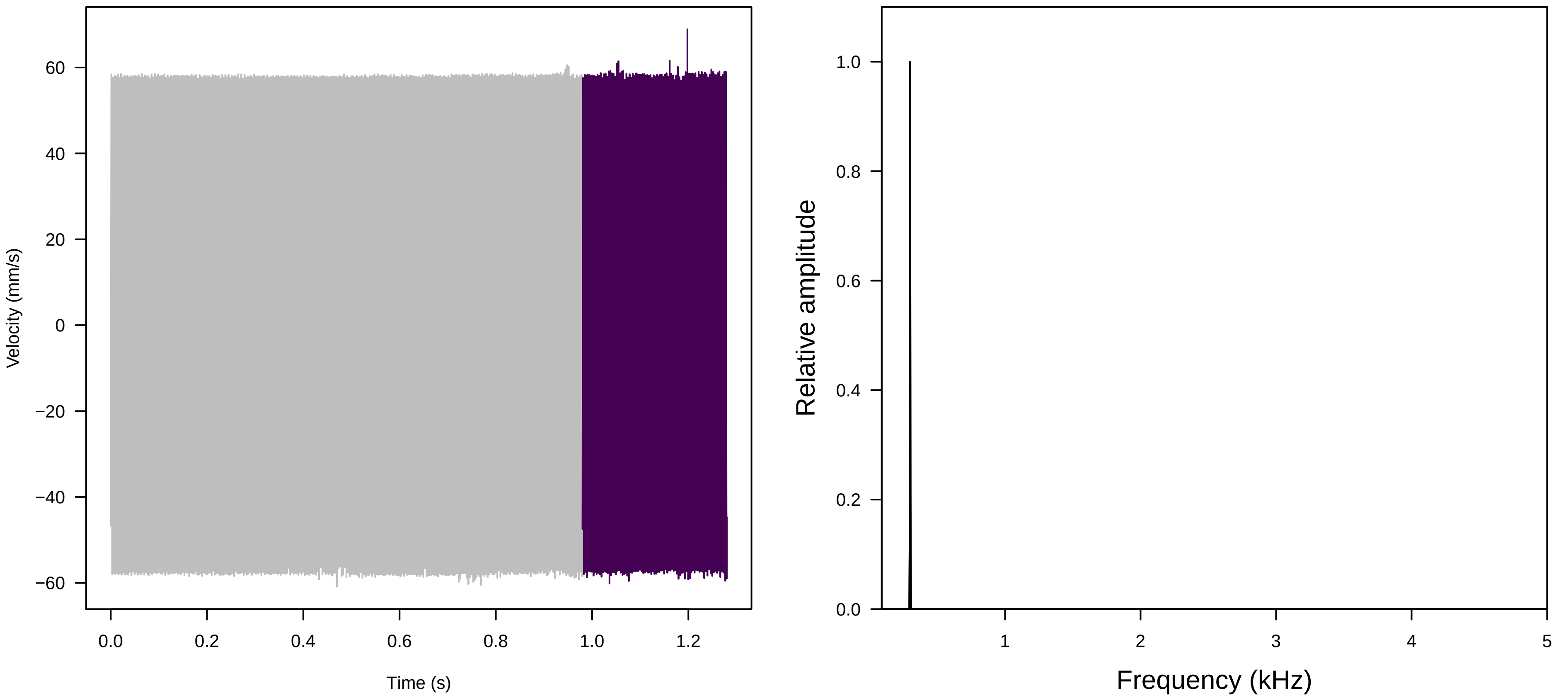

Vel. = 0.014 ; Str. = Receptacle ; Axis = z ; Fl. accession = 10−s−81−1AA

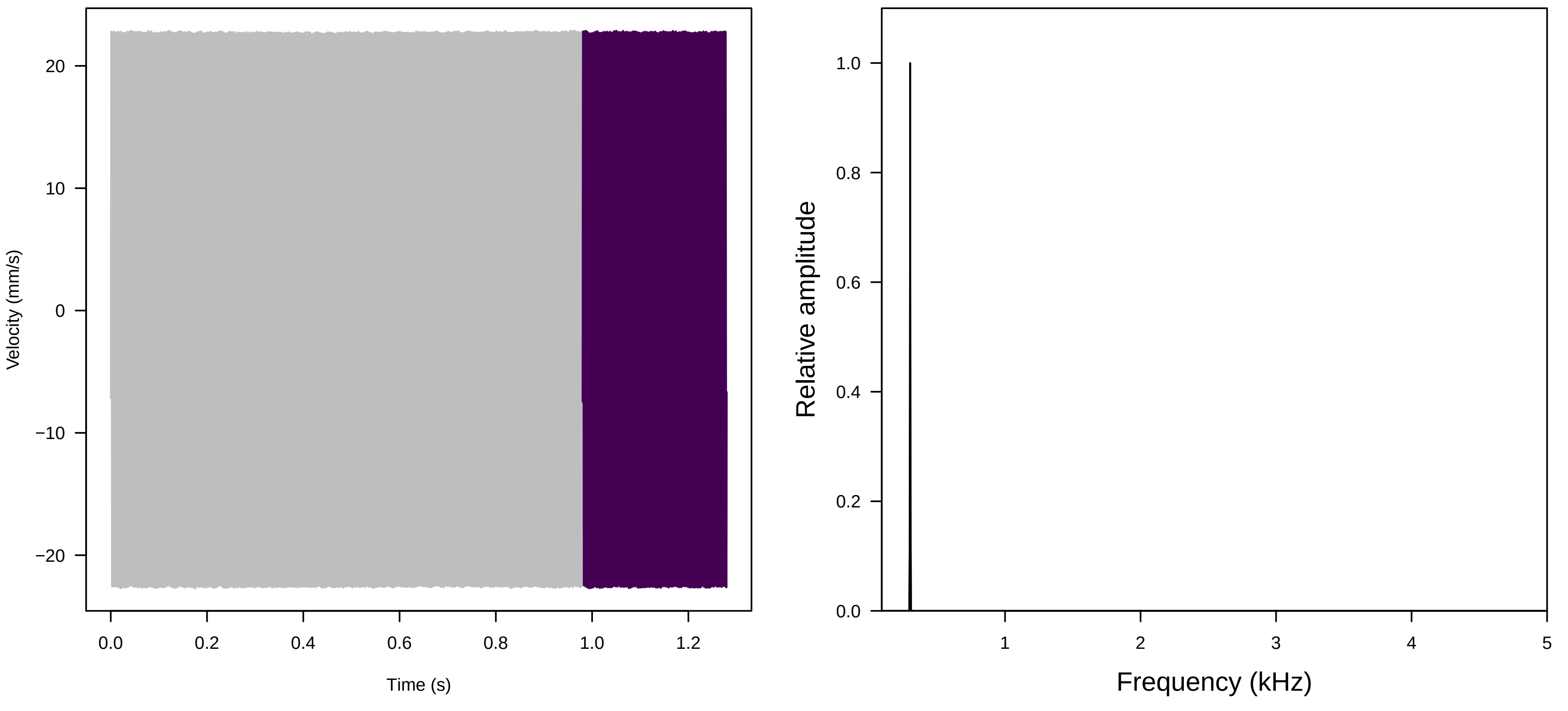

Vel. = 0.014 ; Str. = Corolla ; Axis = y ; Fl. accession = 10−s−81−1AA

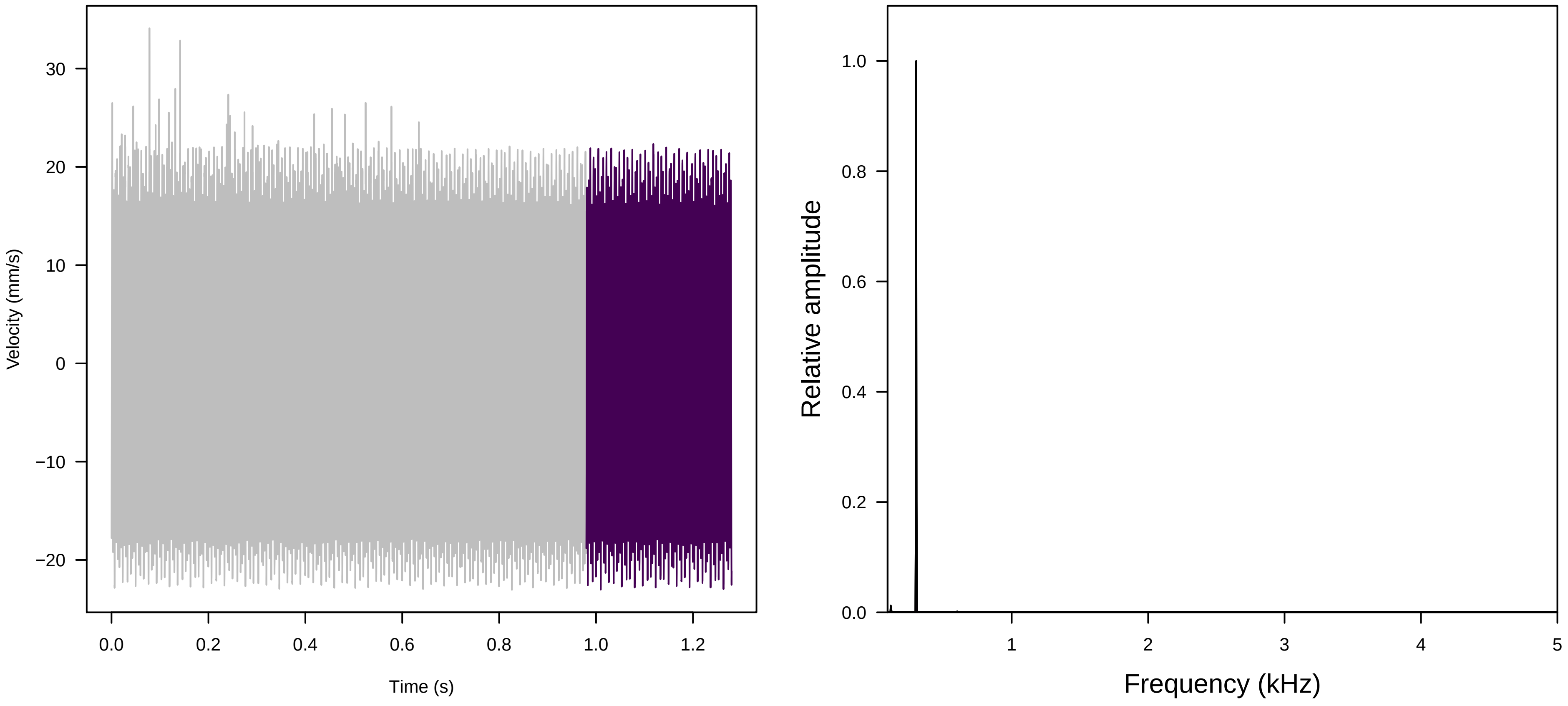

Vel. = 0.014 ; Str. = Receptacle ; Axis = y ; Fl. accession = 10−s−81−1AA

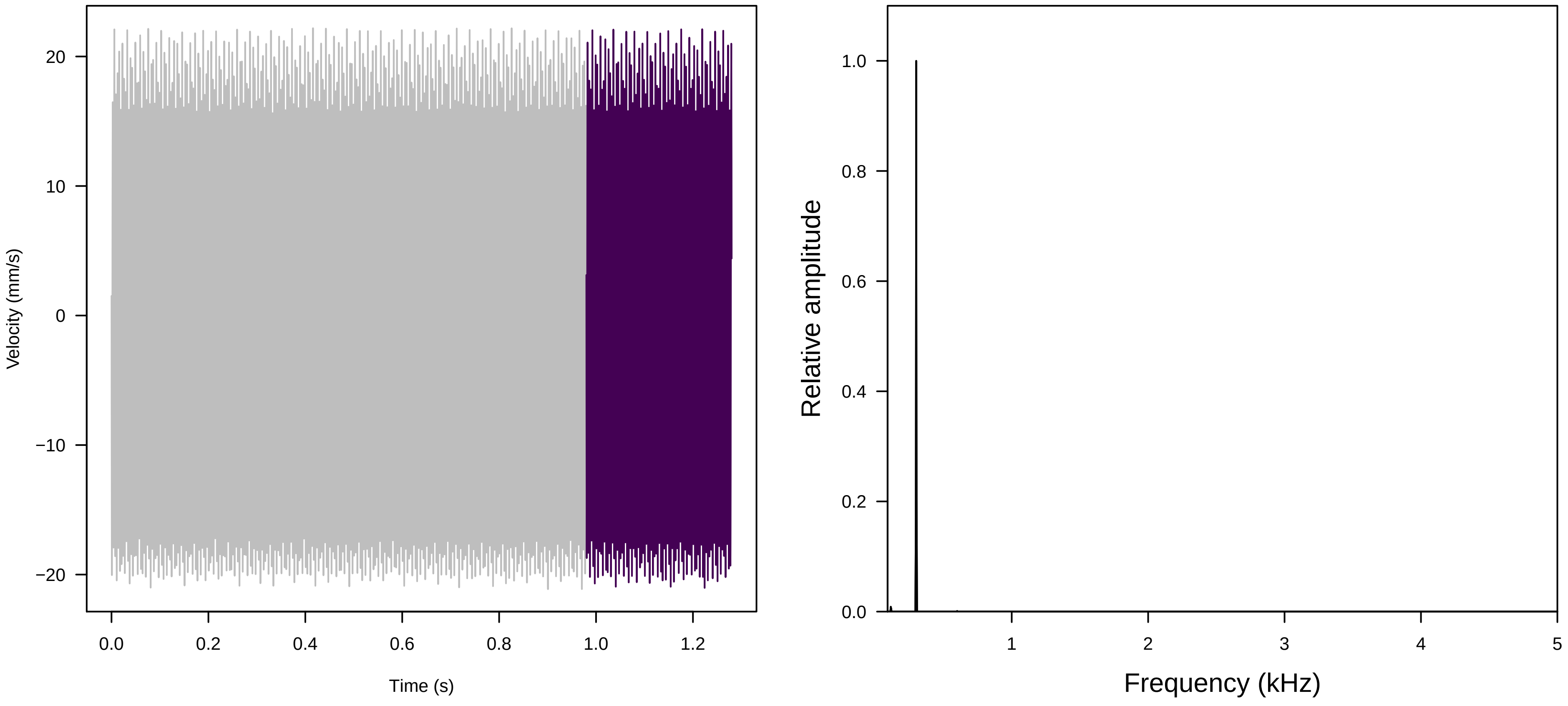

Vel. = 0.014 ; Str. = FA ; Axis = y ; Fl. accession = 10−s−81−1AA

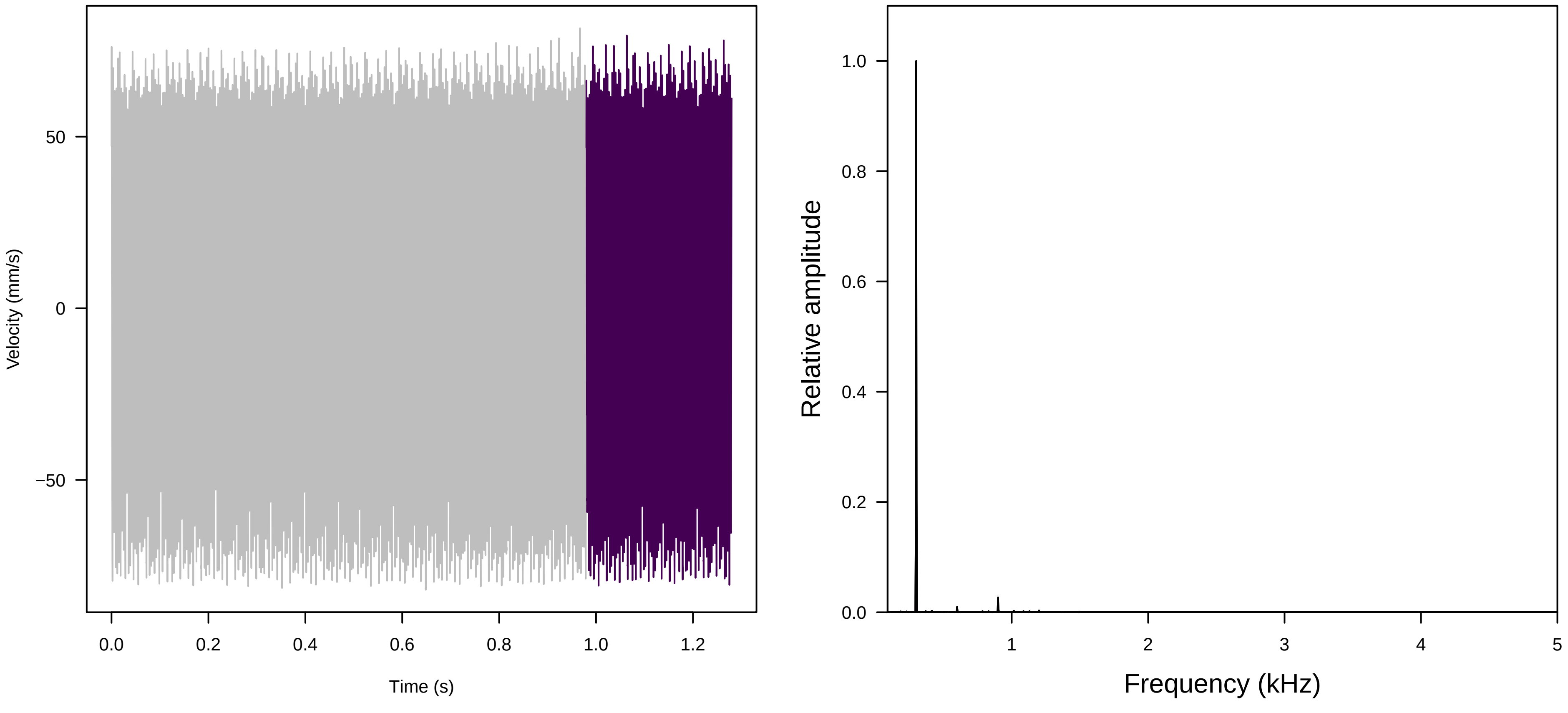

Vel. = 0.014 ; Str. = Receptacle ; Axis = y ; Fl. accession = 10−s−81−1AA

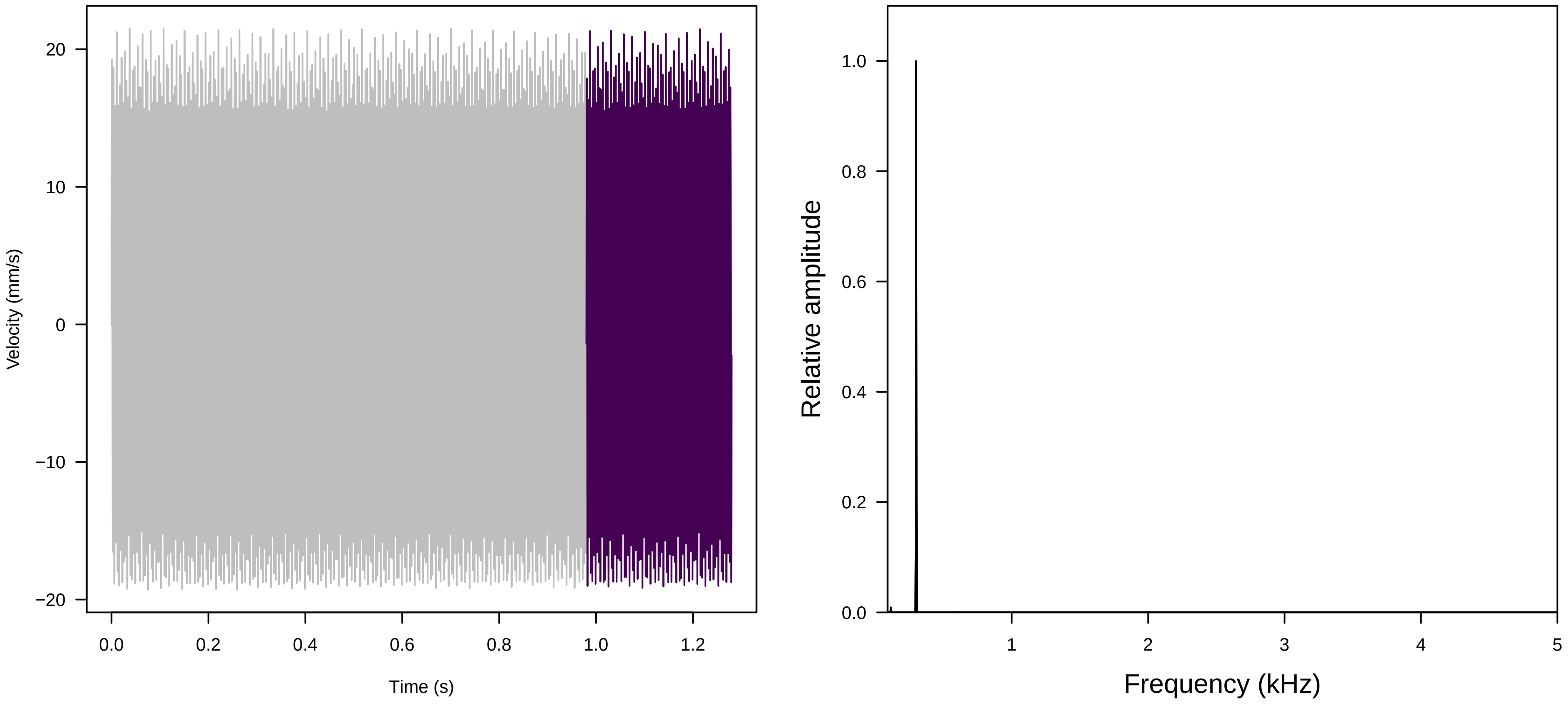

Vel. = 0.014 ; Str. = PA ; Axis = y ; Fl. accession = 10−s−81−1AA

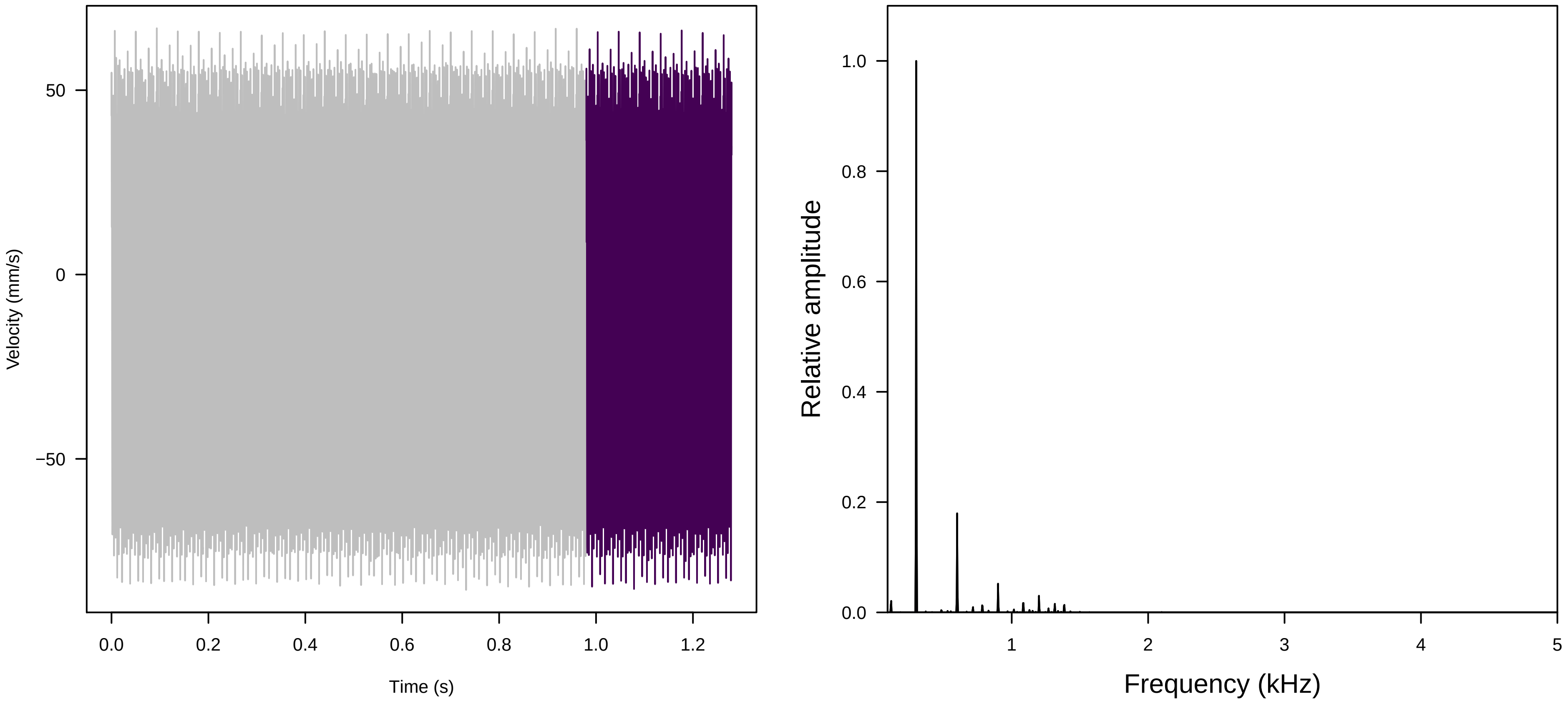

Vel. = 0.014 ; Str. = Receptacle ; Axis = y ; Fl. accession = 10−s−81−1AA

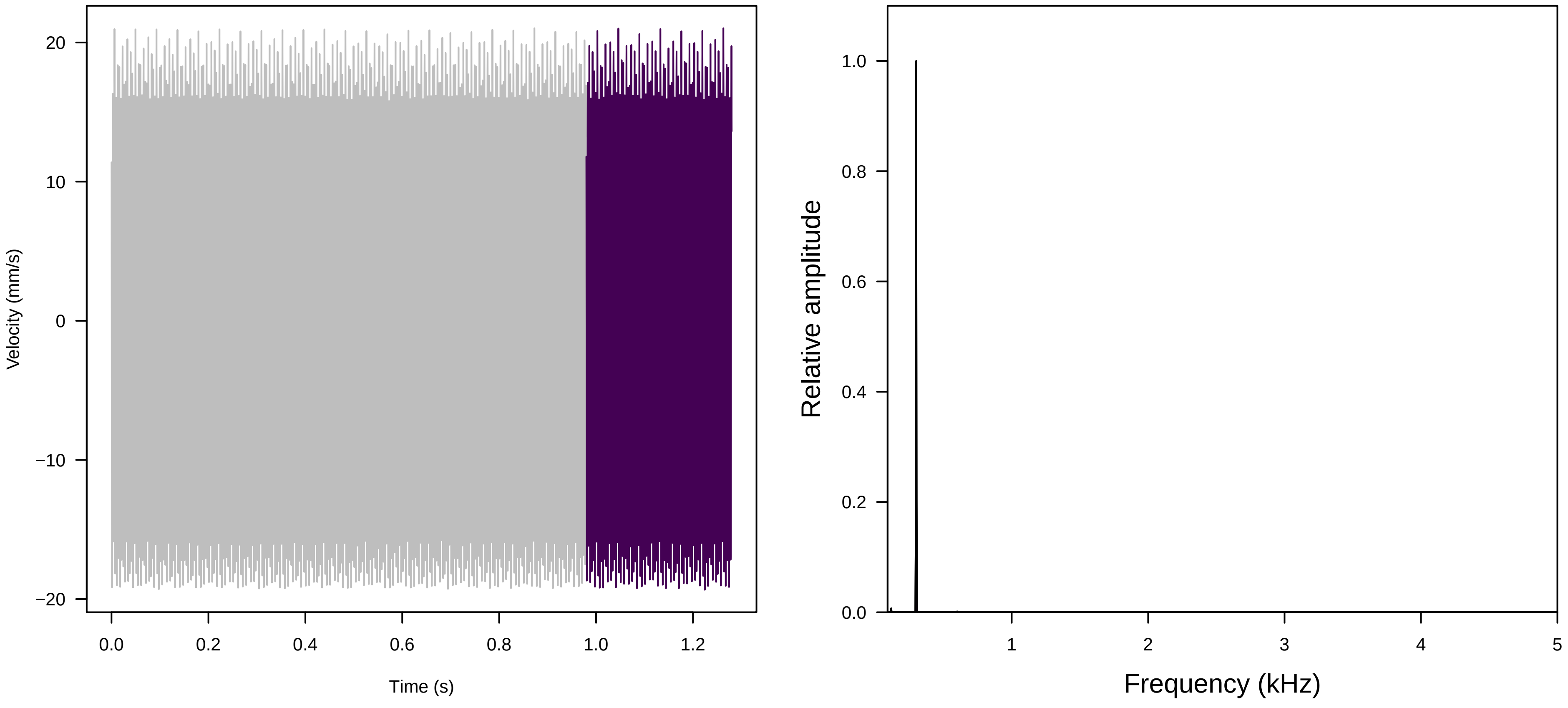

Vel. = 0.028 ; Str. = PA ; Axis = y ; Fl. accession = 10−s−81−1AA

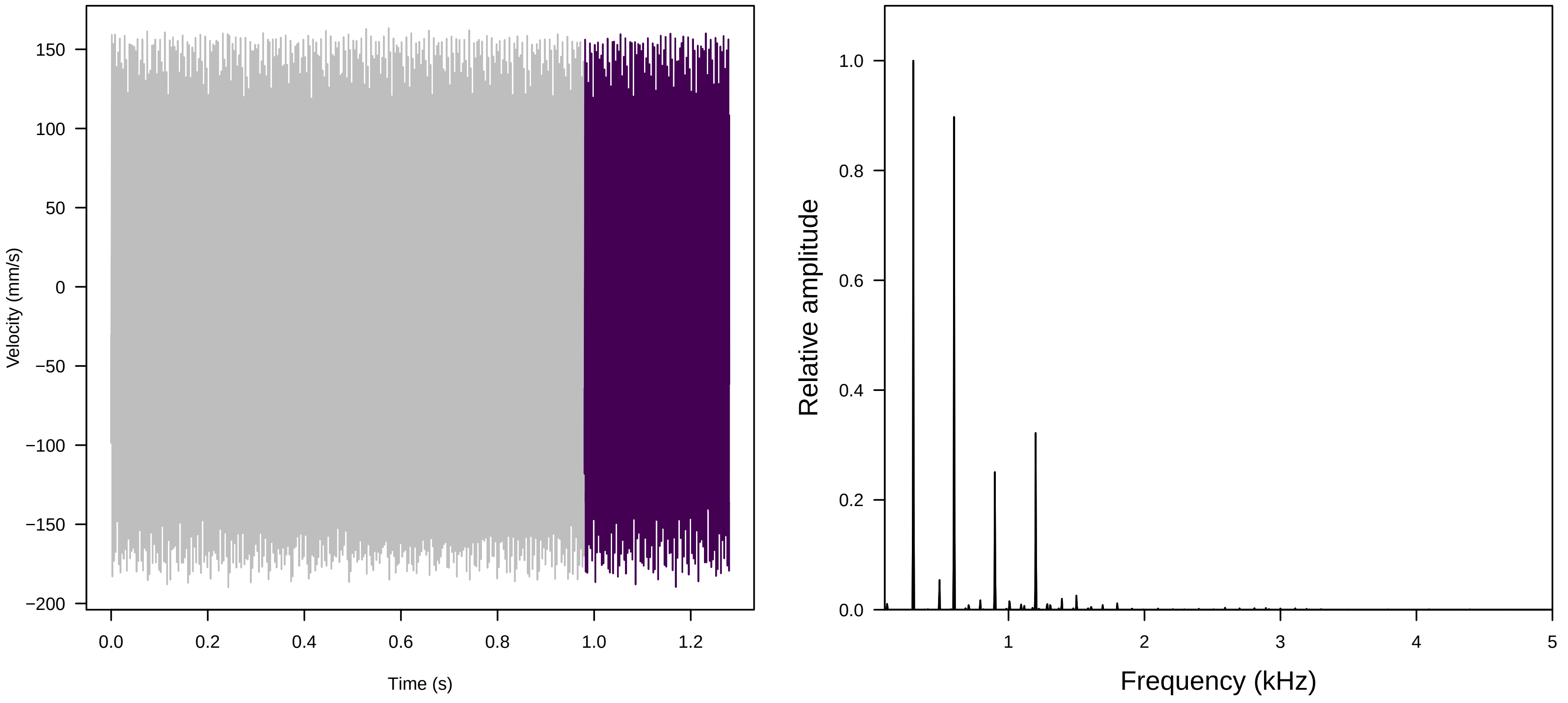

Vel. = 0.028 ; Str. = Receptacle ; Axis = y ; Fl. accession = 10−s−81−1AA

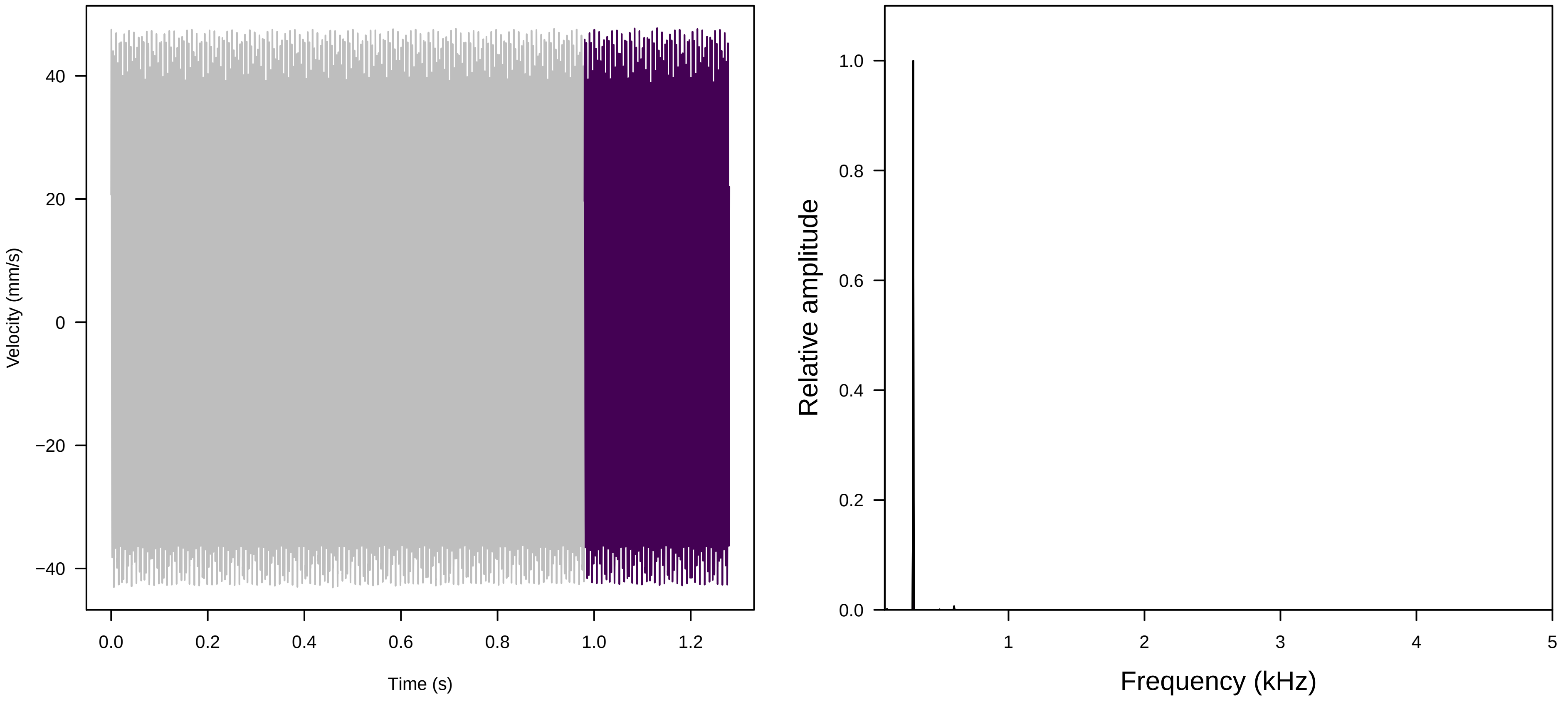

Vel. = 0.028 ; Str. = FA ; Axis = y ; Fl. accession = 10−s−81−1AA

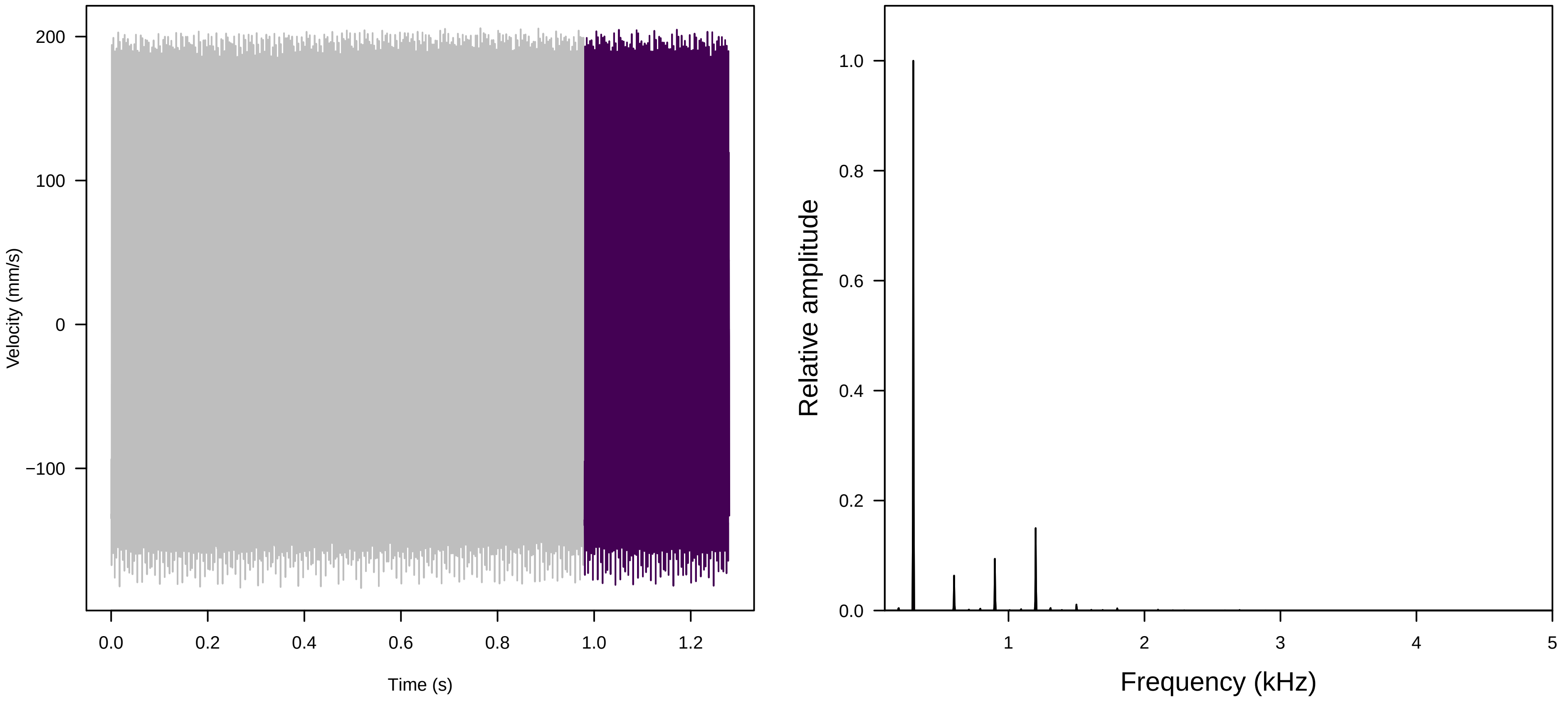

Vel. = 0.028 ; Str. = Receptacle ; Axis = y ; Fl. accession = 10−s−81−1AA

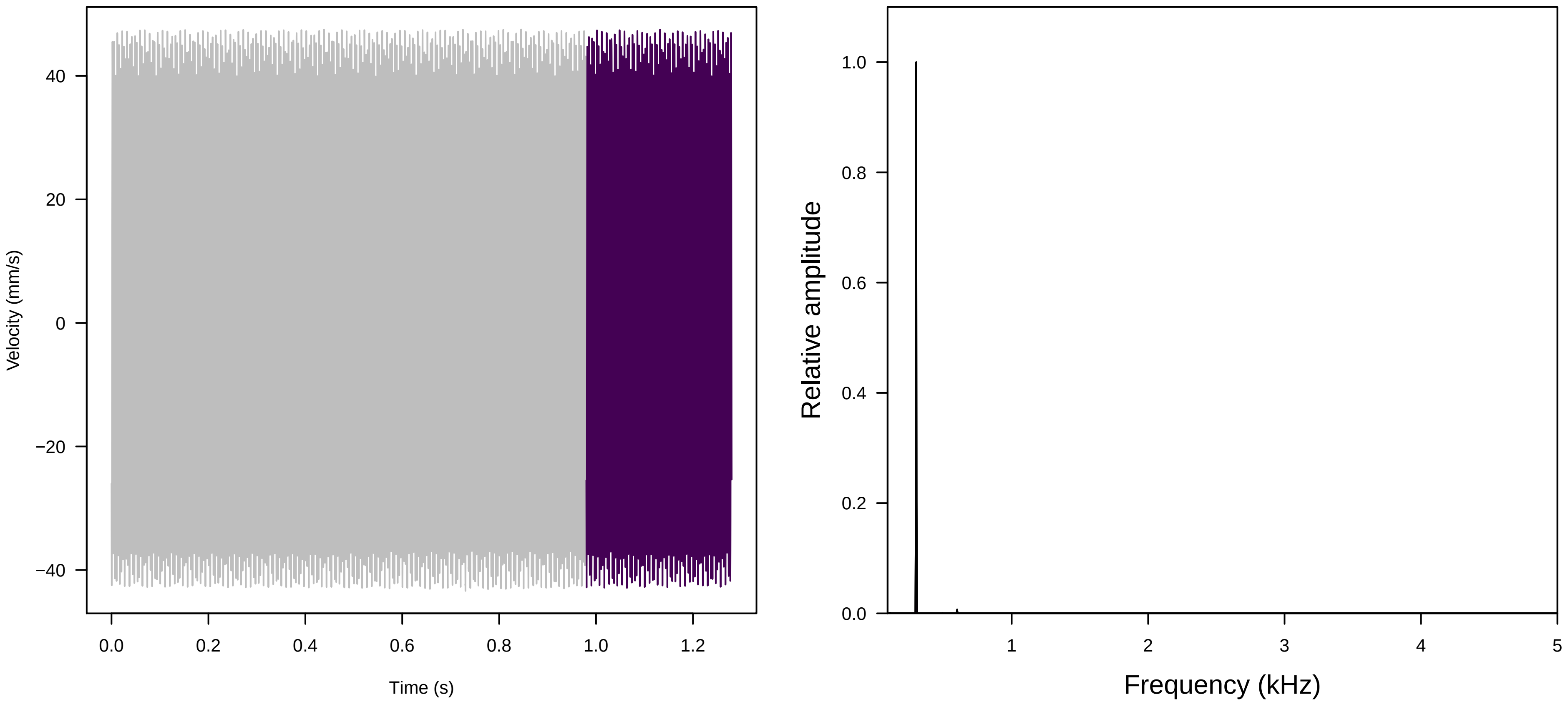

Vel. = 0.028 ; Str. = Corolla ; Axis = y ; Fl. accession = 10−s−81−1AA

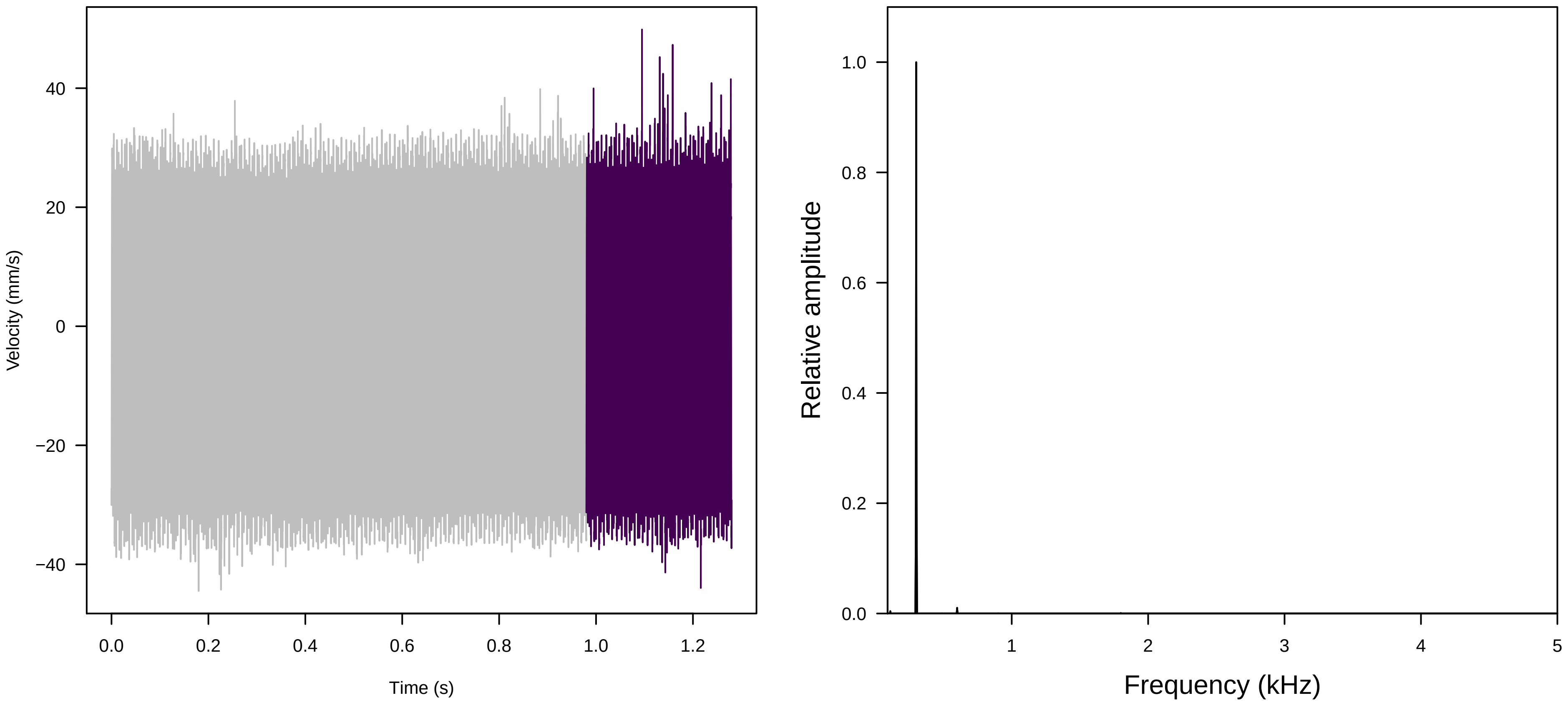

Vel. = 0.028 ; Str. = Receptacle ; Axis = y ; Fl. accession = 10−s−81−1AA

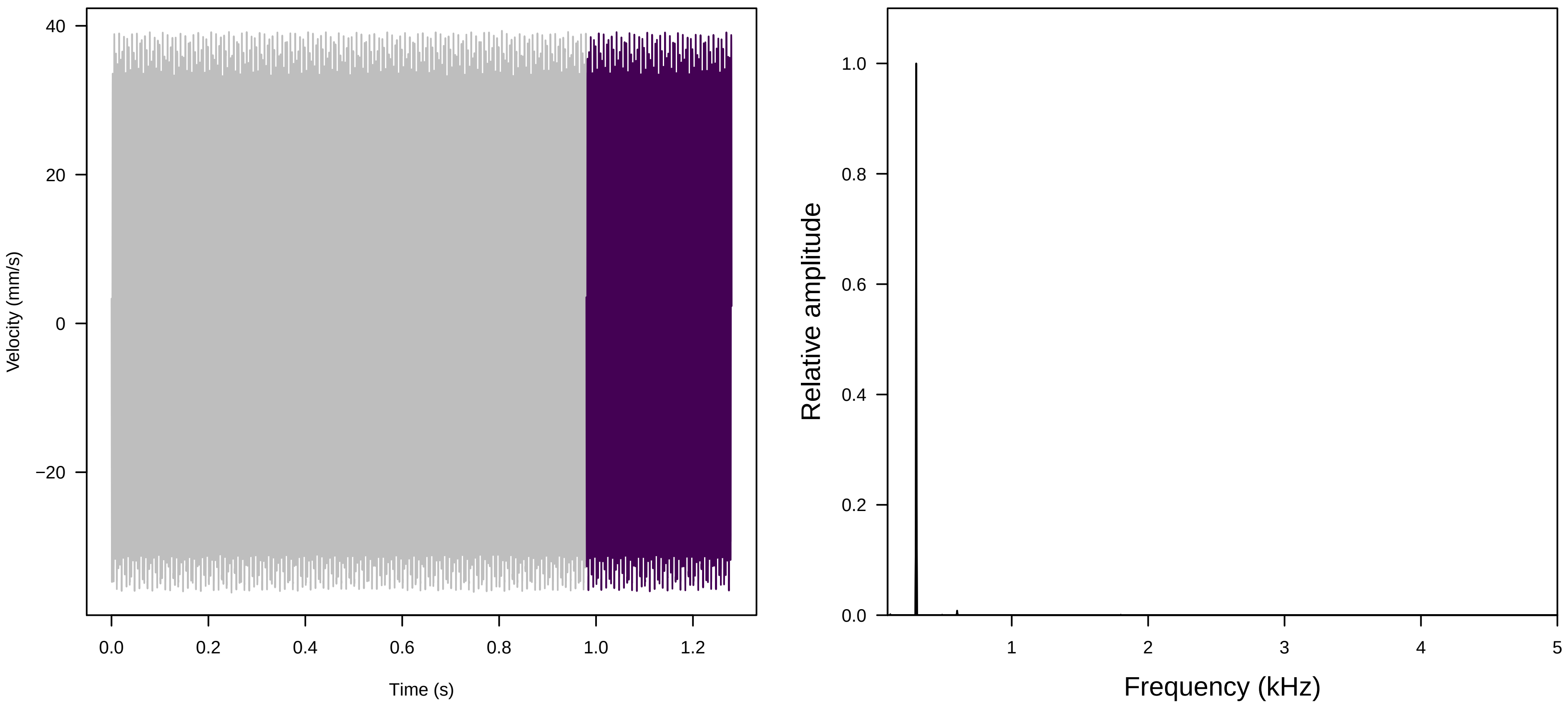

Vel. = 0.057 ; Str. = Corolla ; Axis = y ; Fl. accession = 10−s−81−1AA

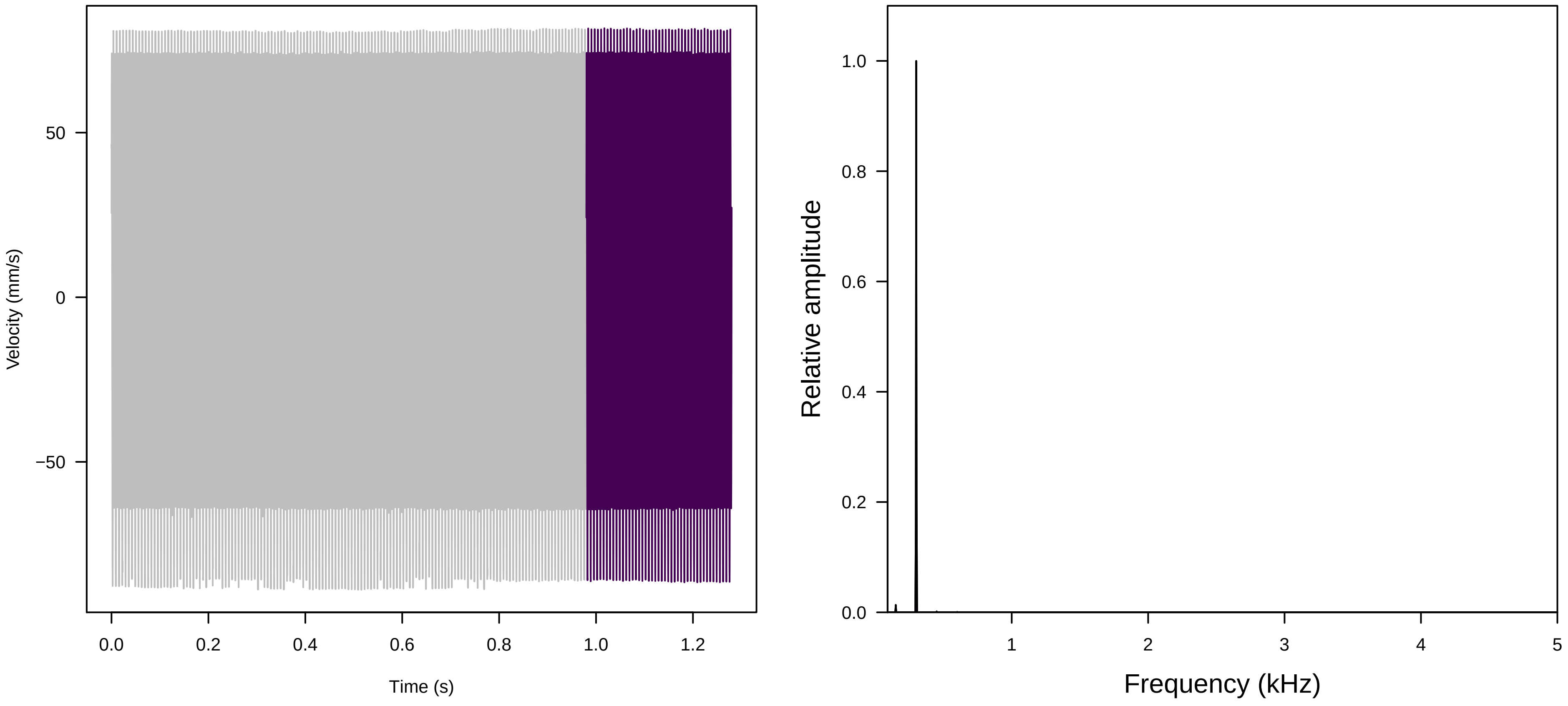

Vel. = 0.057 ; Str. = Receptacle ; Axis = y ; Fl. accession = 10−s−81−1AA

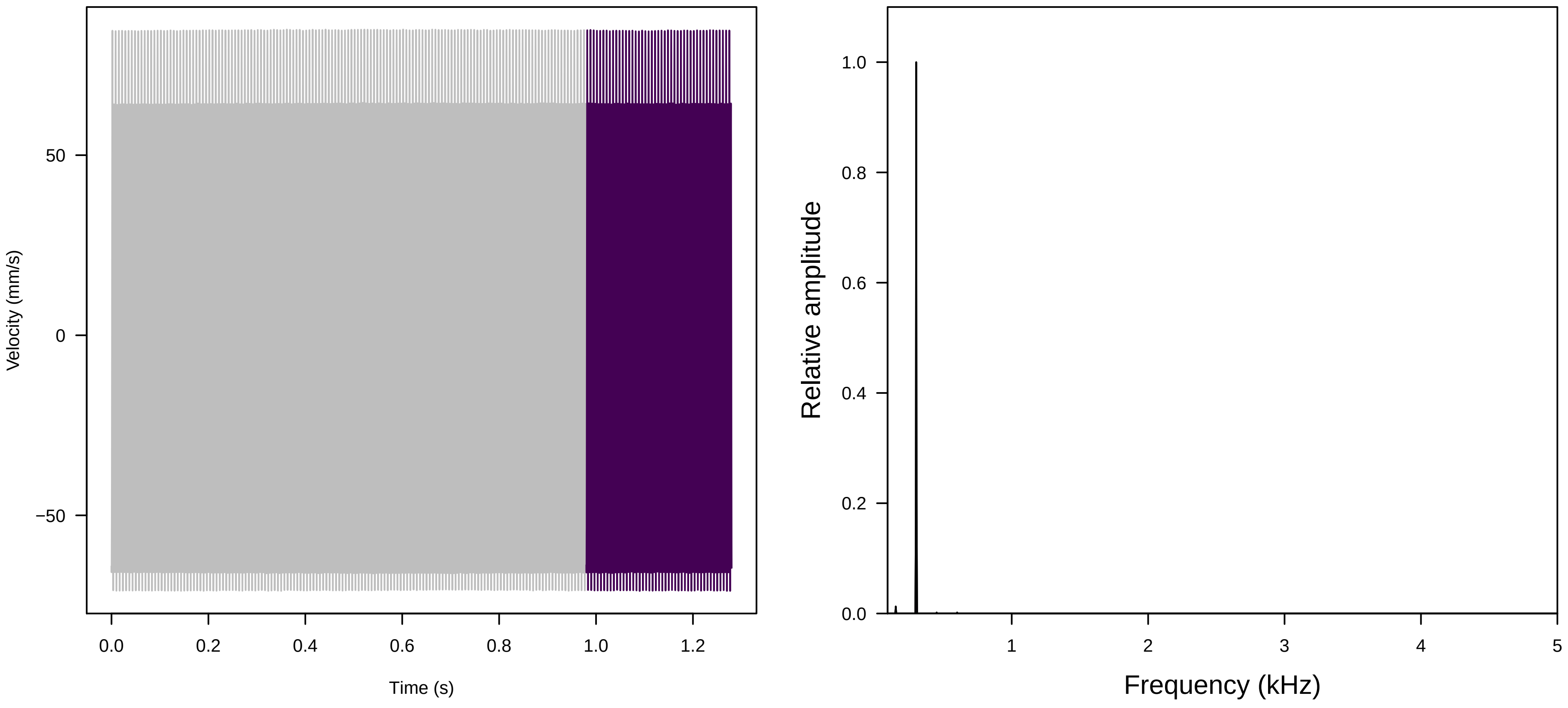

Vel. = 0.057 ; Str. = FA ; Axis = y ; Fl. accession = 10−s−81−1AA

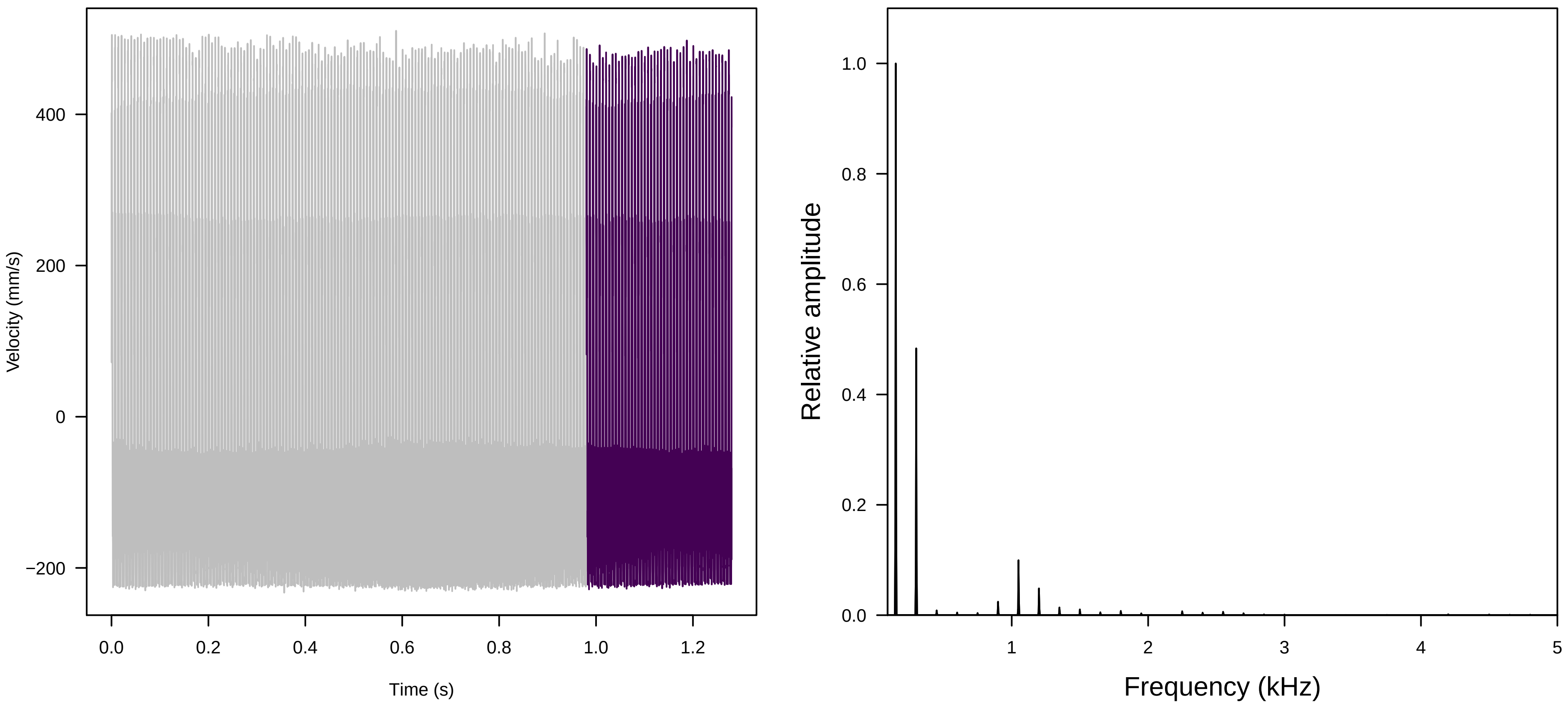

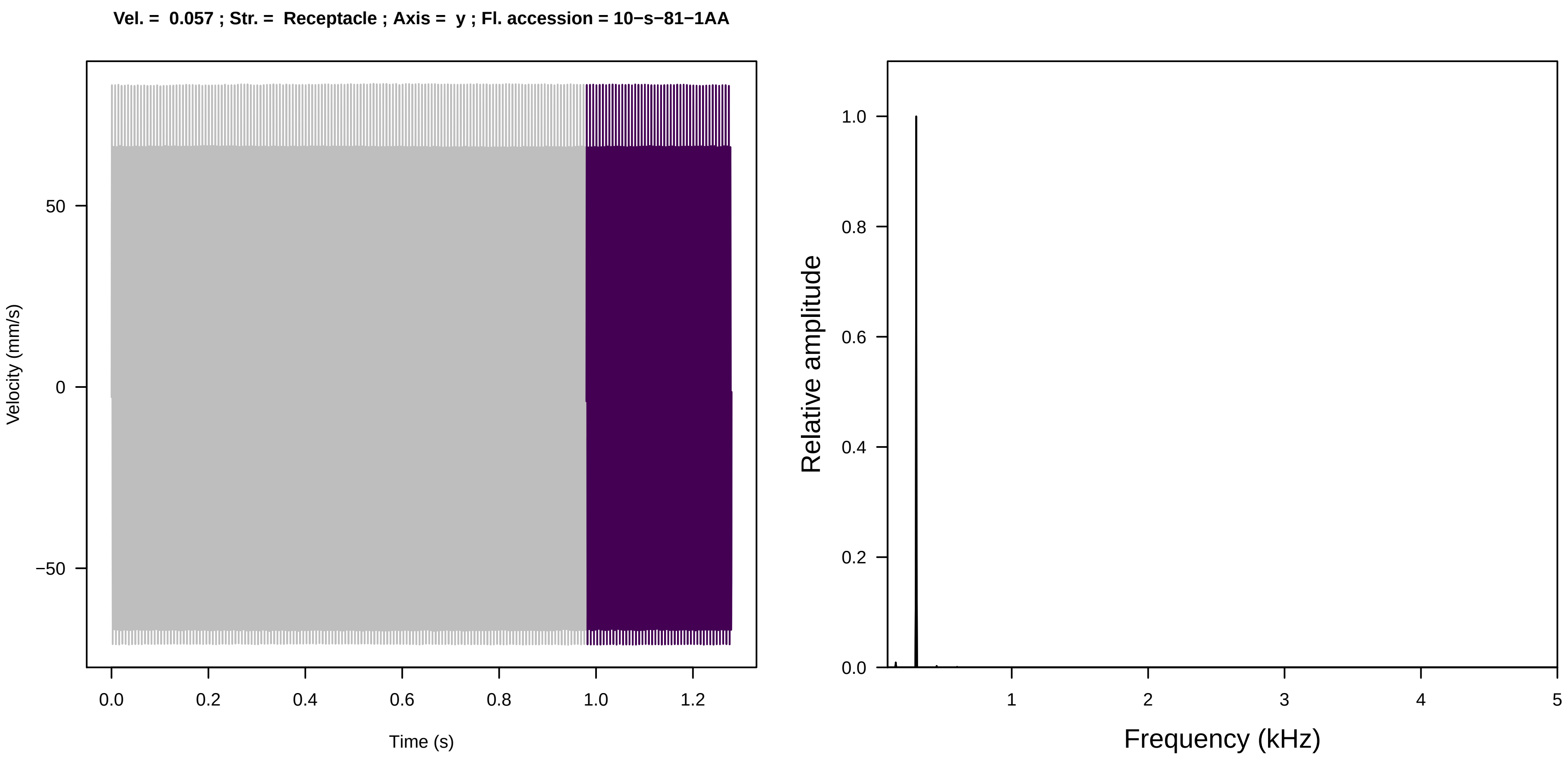
Vel. = 0.057 ; Str. = Receptacle ; Axis = y ; Fl. accession = 10−s−81−1AA
Velocity (mm/s)
Time (s)
Relative amplitude
Frequency (kHz)

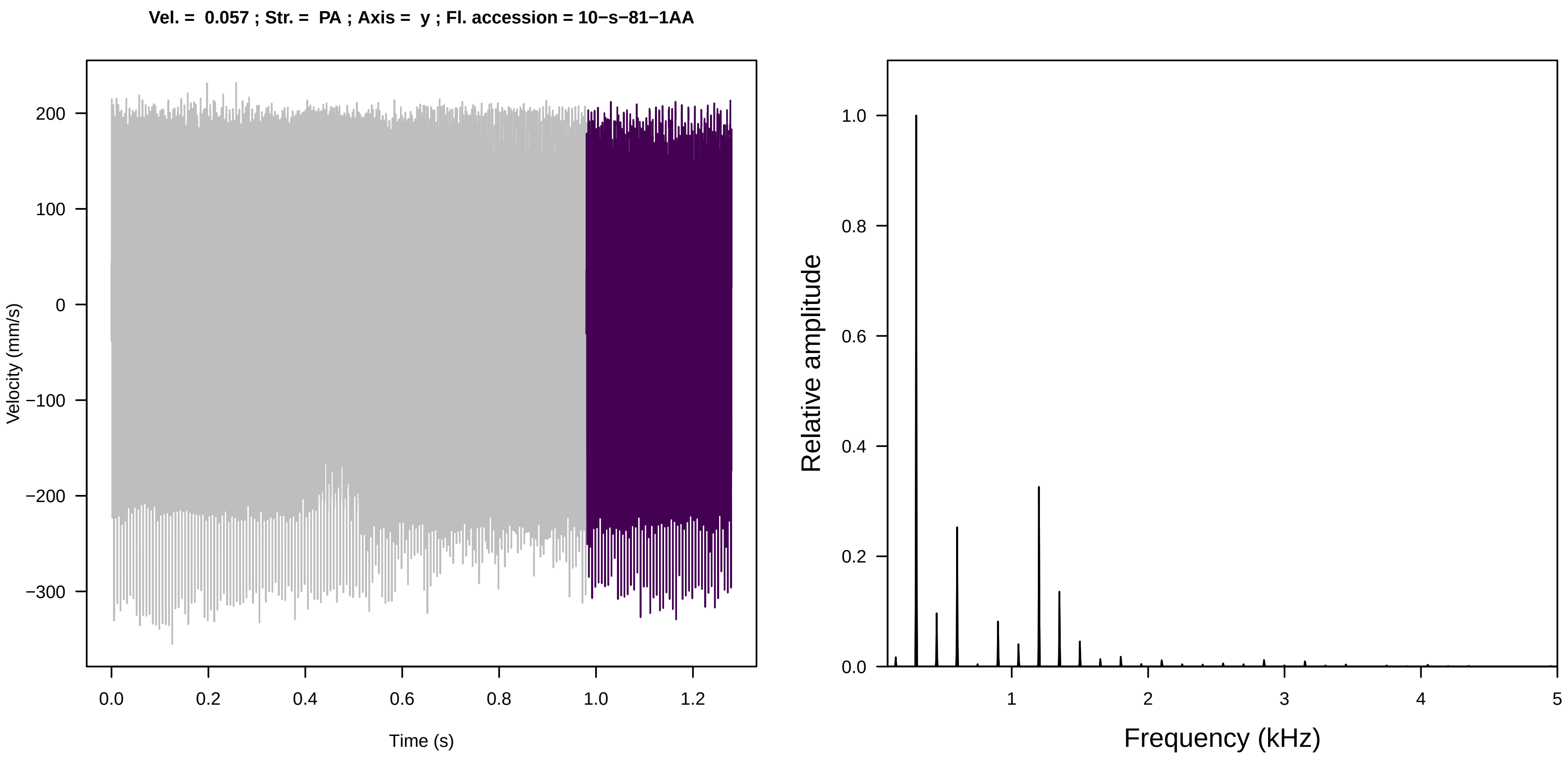
Vel. = 0.057 ; Str. = PA ; Axis = y ; Fl. accession = 10−s−81−1AA
Velocity (mm/s)
Time (s)
Relative amplitude
Frequency (kHz)

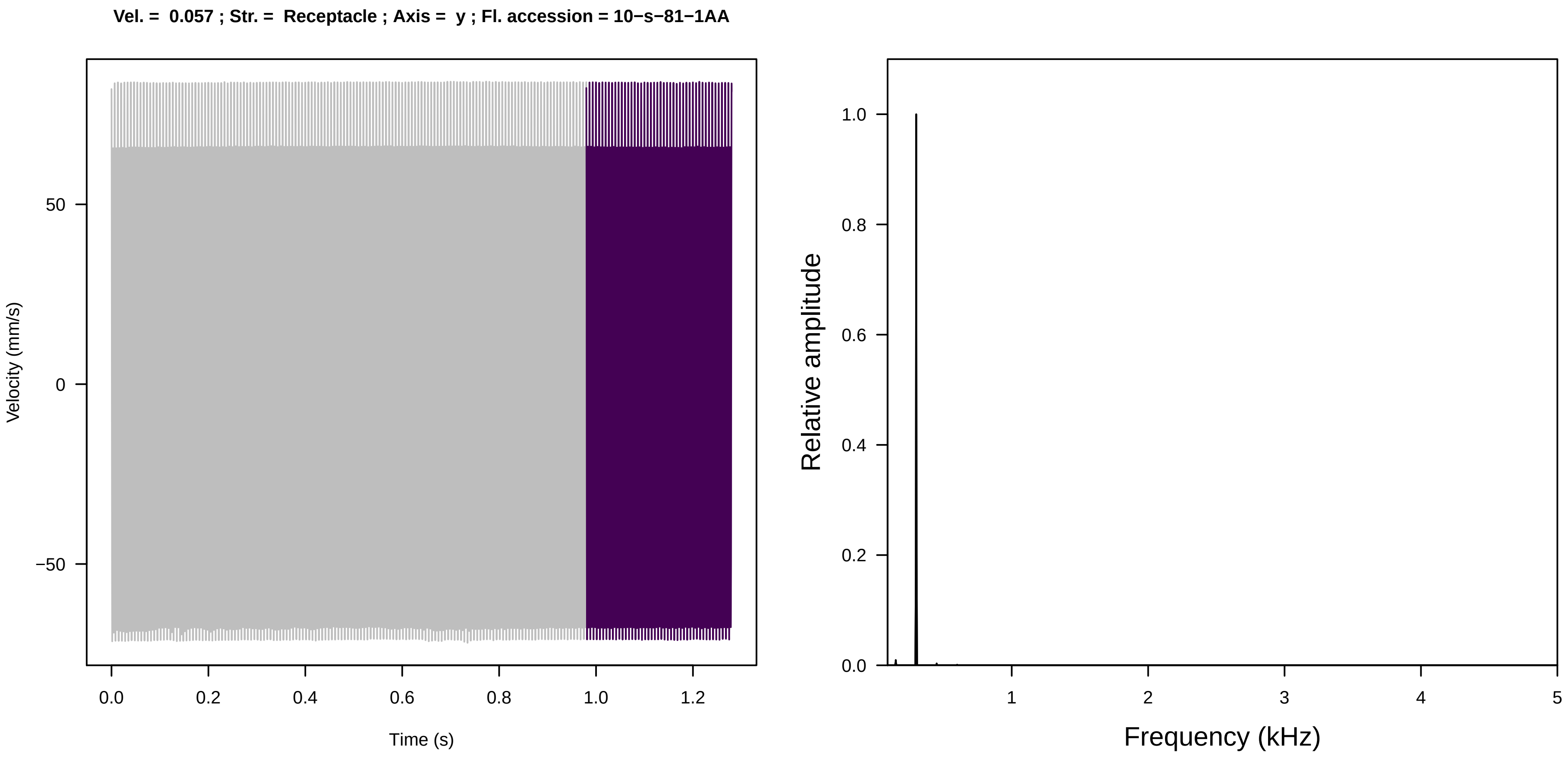
Vel. = 0.057 ; Str. = Receptacle ; Axis = y ; Fl. accession = 10−s−81−1AA
Velocity (mm/s)
Time (s)
Relative amplitude
Frequency (kHz)

Vel. = 0.057 ; Str. = Corolla ; Axis = y ; Fl. accession = 10−s−81−1AB

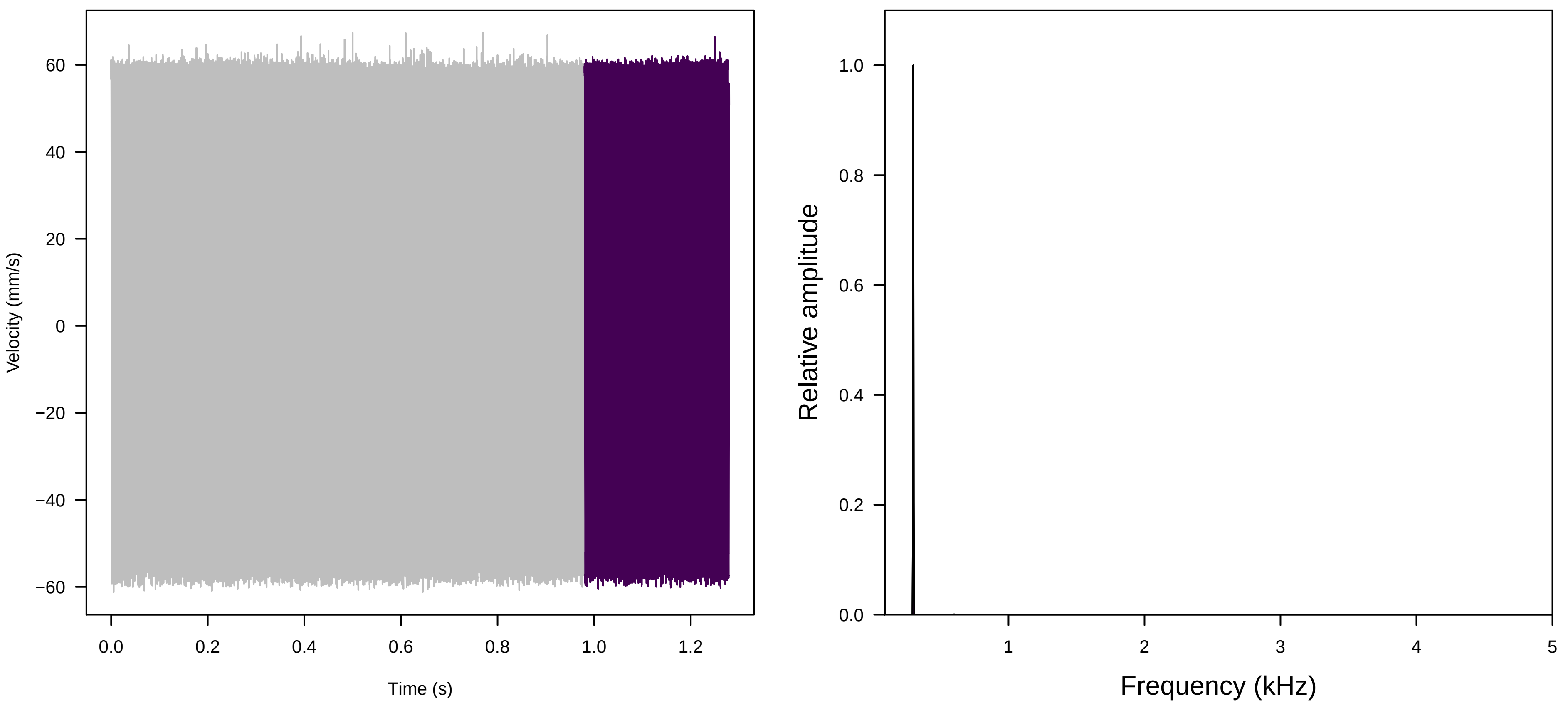

Vel. = 0.057 ; Str. = Receptacle ; Axis = y ; Fl. accession = 10−s−81−1AB

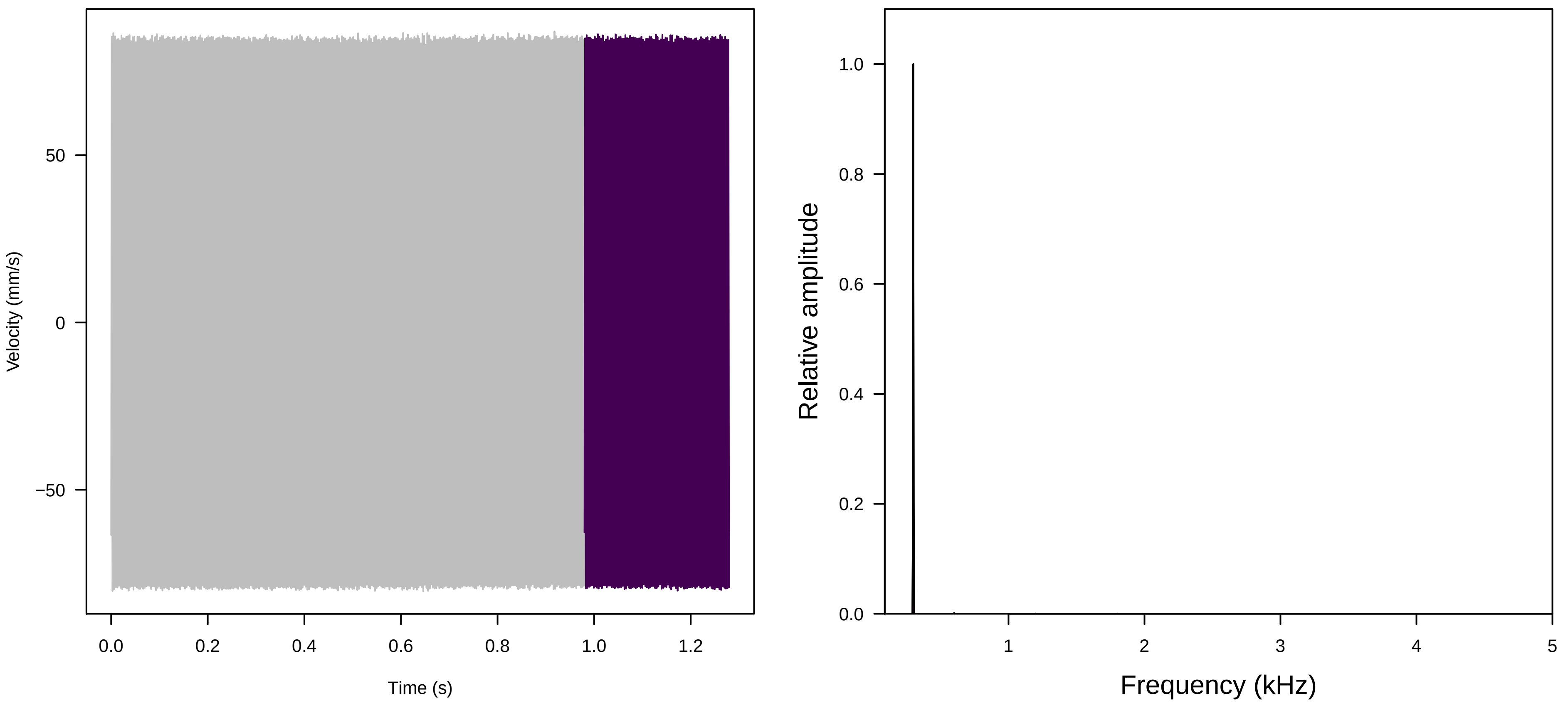

Vel. = 0.057 ; Str. = FA ; Axis = y ; Fl. accession = 10−s−81−1AB

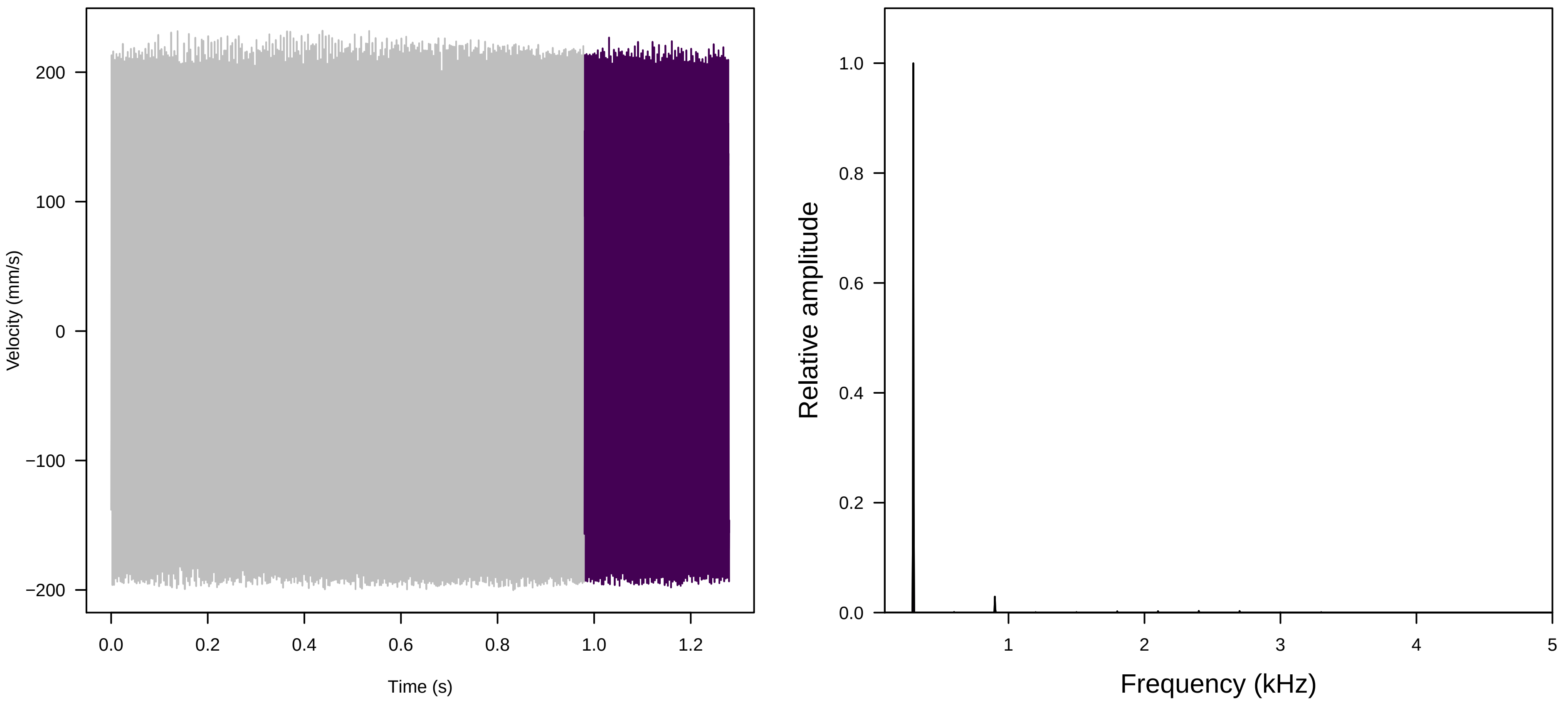

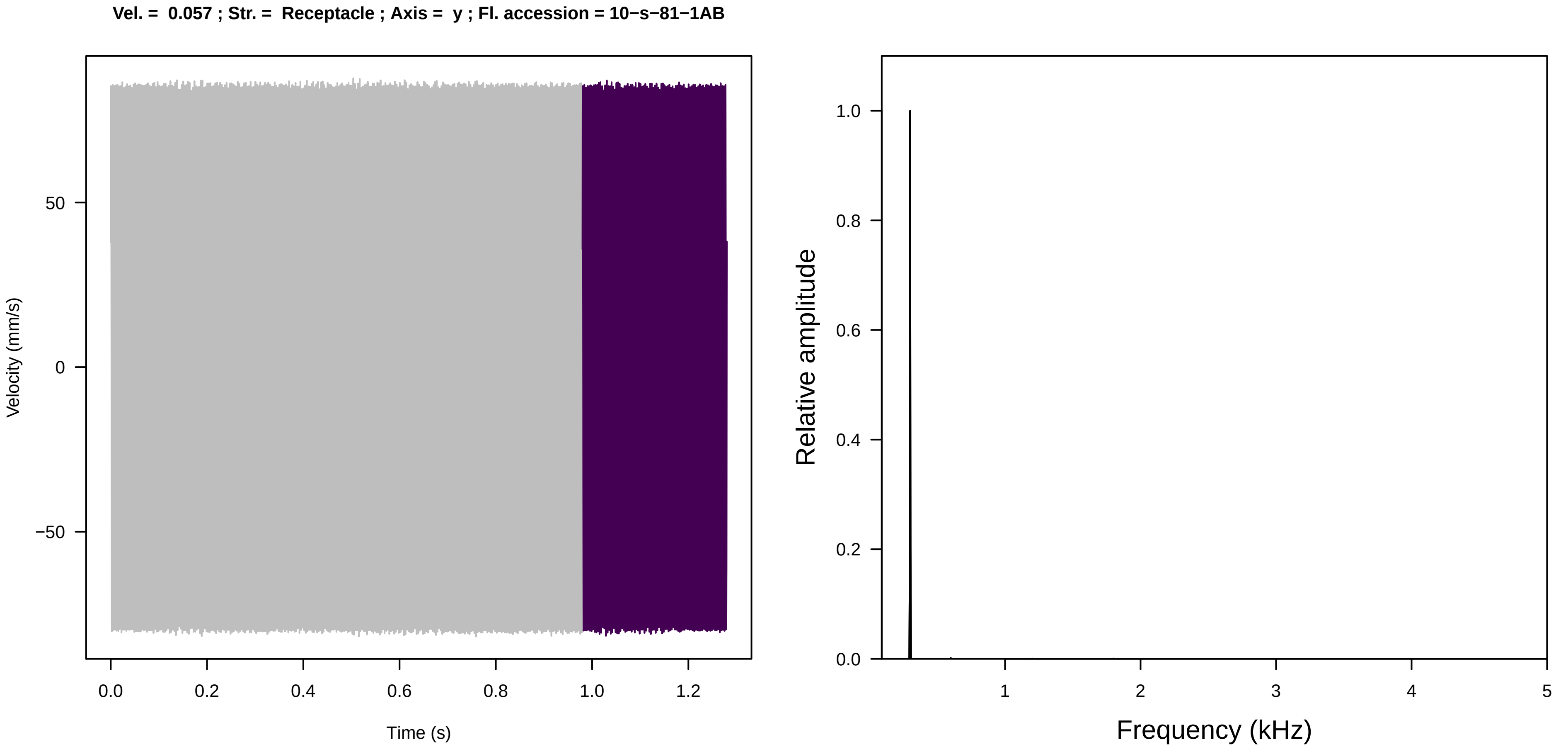
Vel. = 0.057 ; Str. = Receptacle ; Axis = y ; Fl. accession = 10−s−81−1AB
Velocity (mm/s)
Time (s)
Relative amplitude
Frequency (kHz)

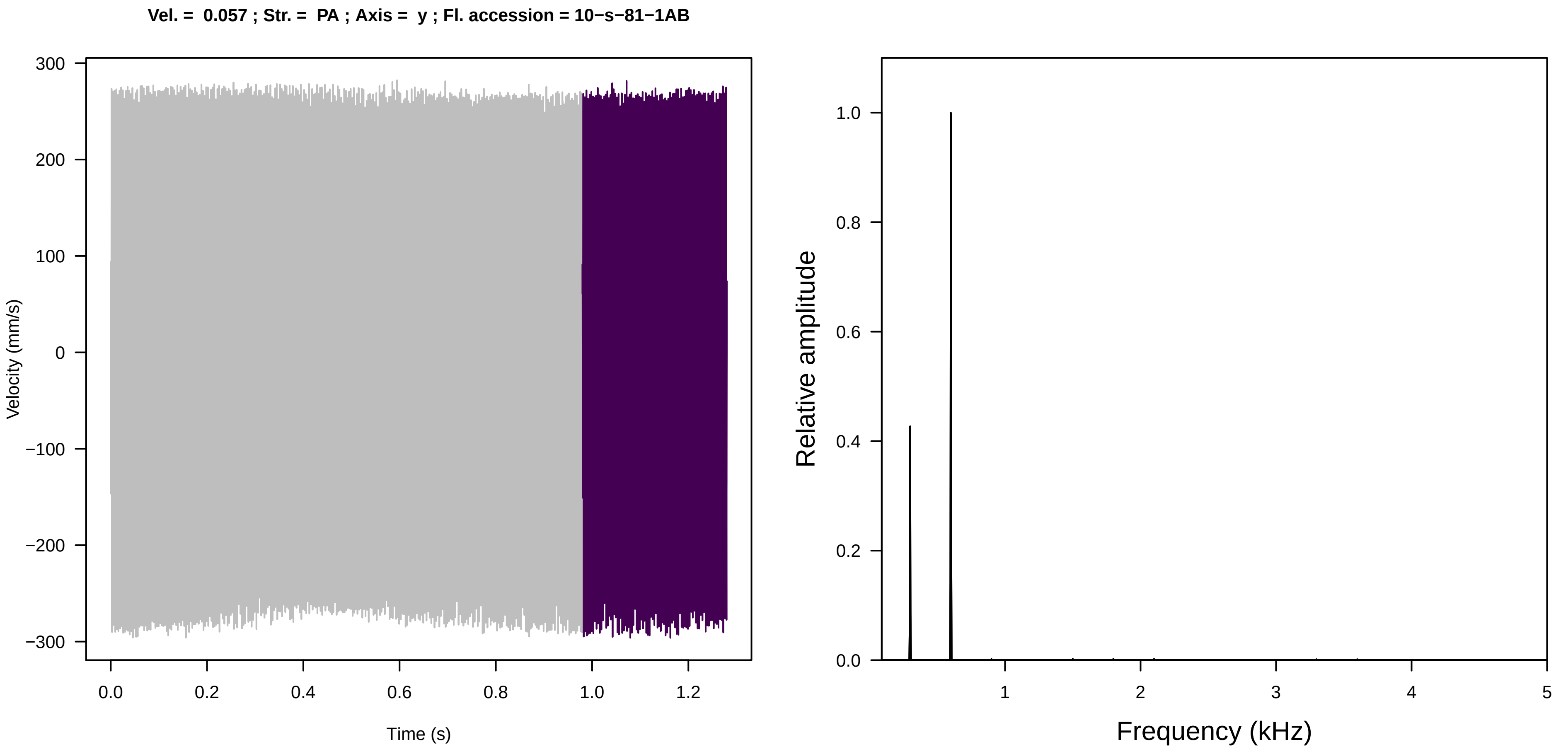
Vel. = 0.057 ; Str. = PA ; Axis = y ; Fl. accession = 10−s−81−1AB
Velocity (mm/s)
Time (s)
Relative amplitude
Frequency (kHz)

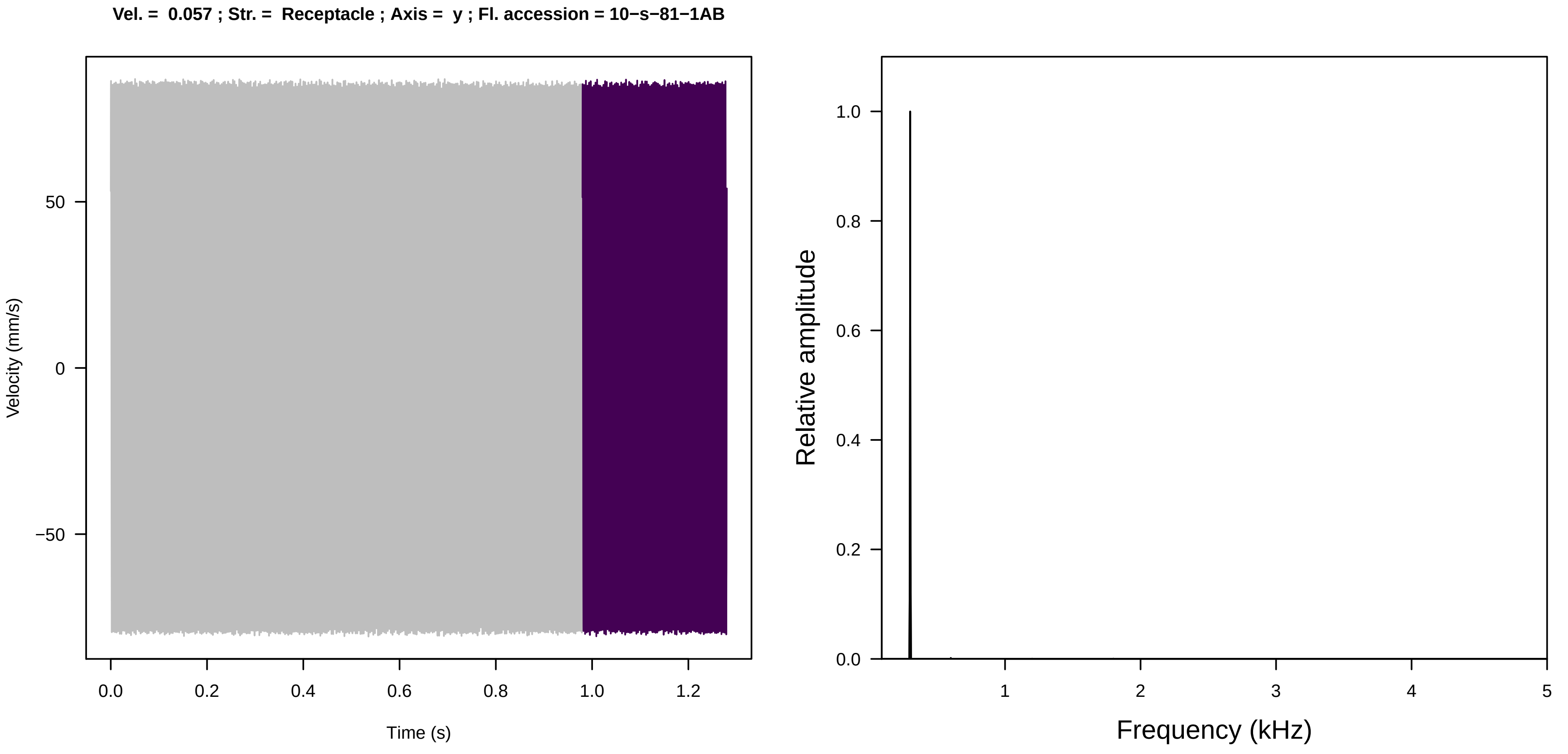
Vel. = 0.057 ; Str. = Receptacle ; Axis = y ; Fl. accession = 10−s−81−1AB
Velocity (mm/s)
Time (s)
Relative amplitude
Frequency (kHz)

Vel. = 0.028 ; Str. = PA ; Axis = y ; Fl. accession = 10−s−81−1AB

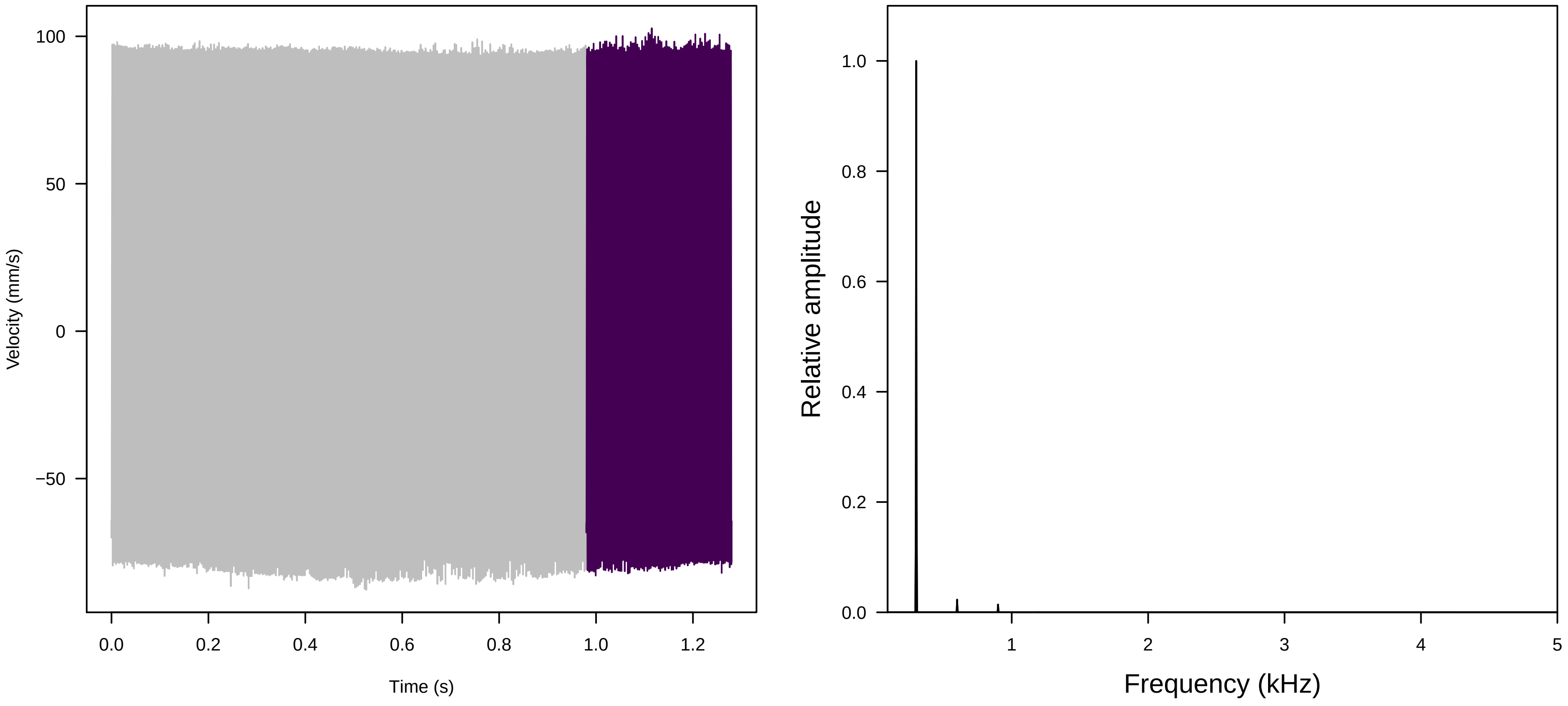

Vel. = 0.028 ; Str. = Receptacle ; Axis = y ; Fl. accession = 10−s−81−1AB

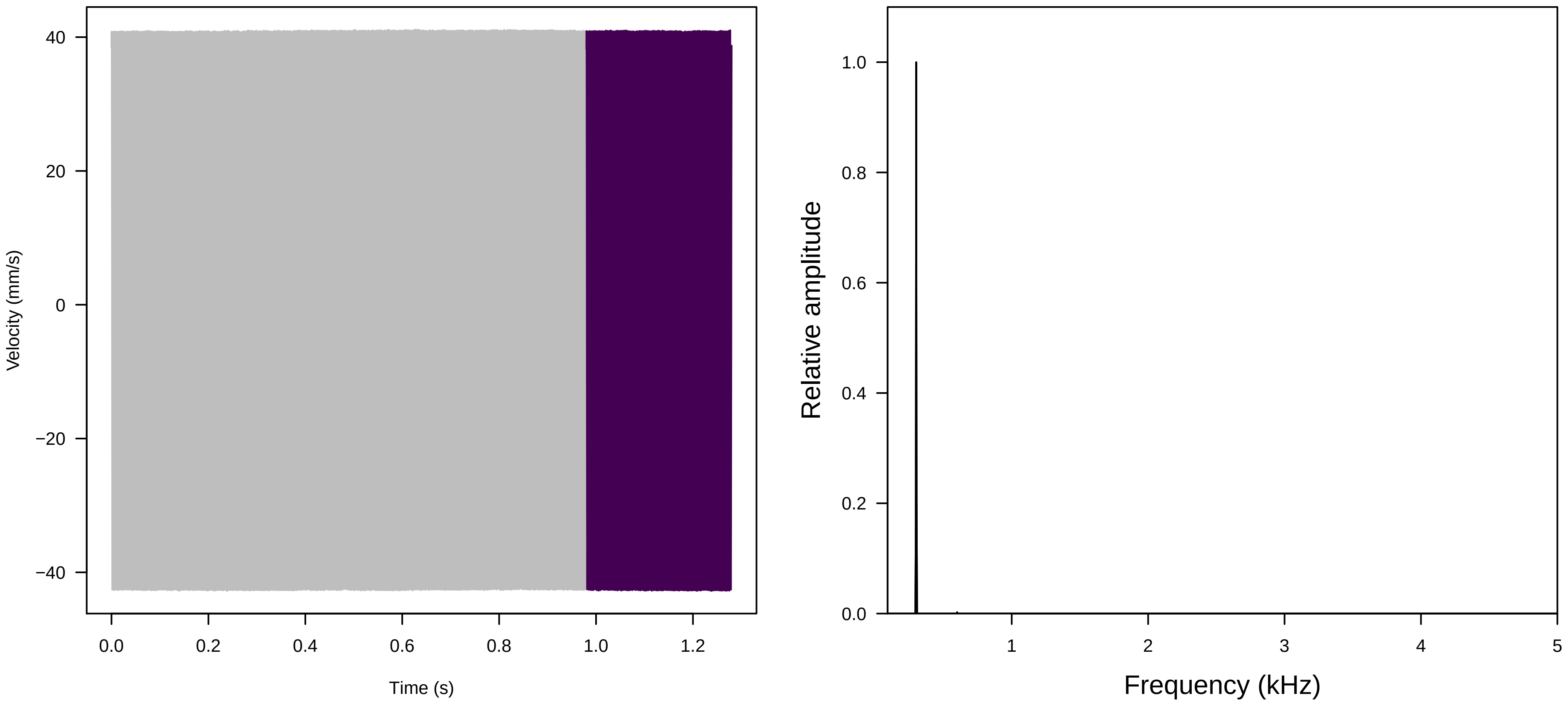

Vel. = 0.028 ; Str. = FA ; Axis = y ; Fl. accession = 10−s−81−1AB

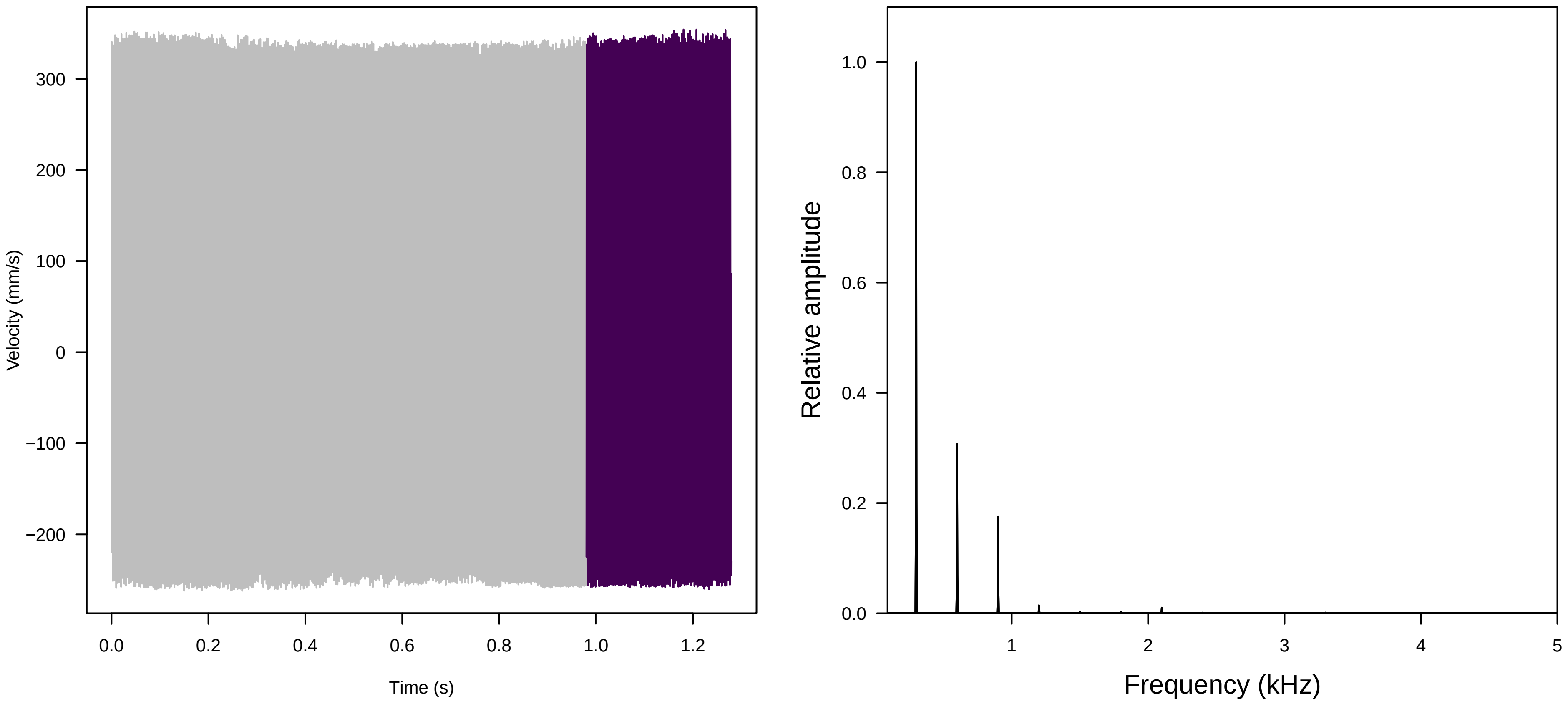

Vel. = 0.028 ; Str. = Receptacle ; Axis = y ; Fl. accession = 10−s−81−1AB

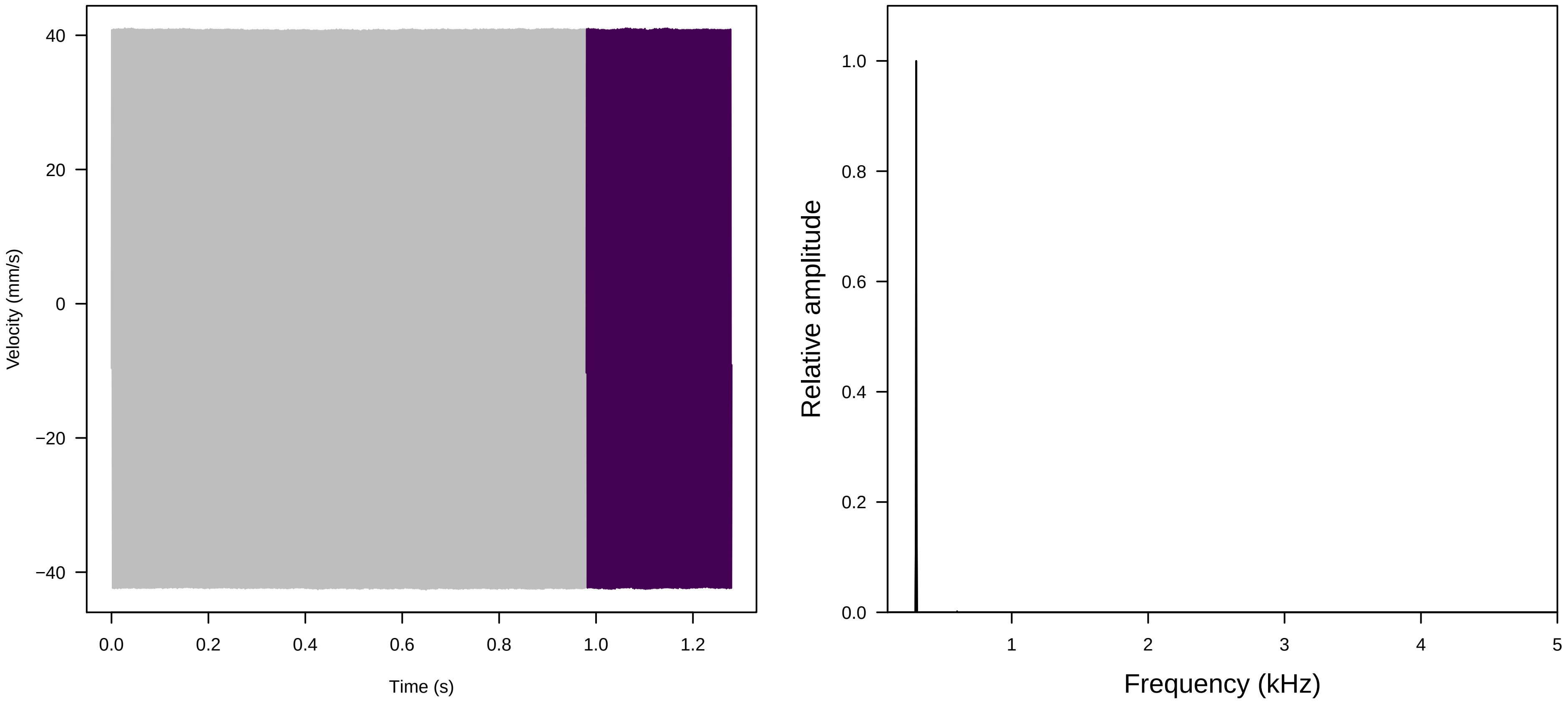

Vel. = 0.028 ; Str. = Corolla ; Axis = y ; Fl. accession = 10−s−81−1AB

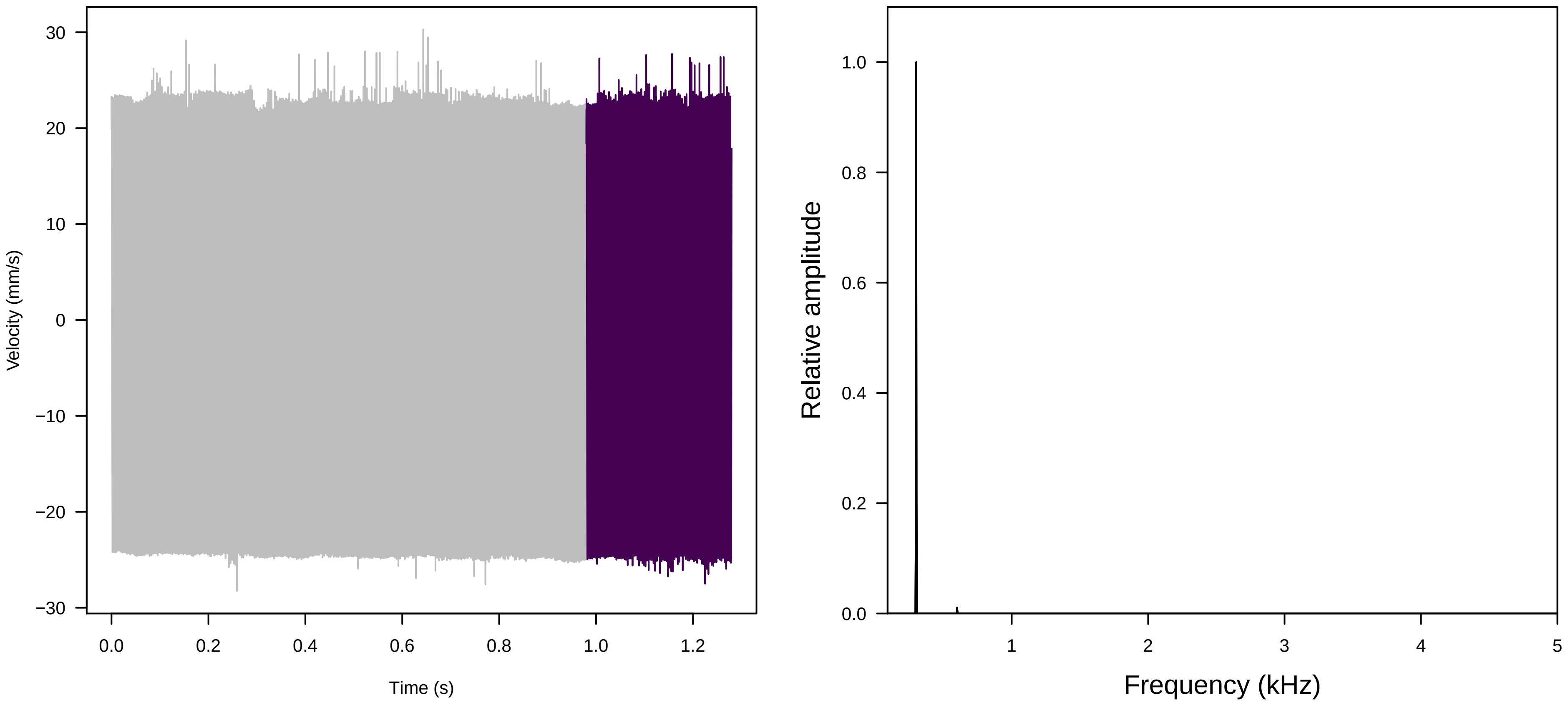

Vel. = 0.028 ; Str. = Receptacle ; Axis = y ; Fl. accession = 10−s−81−1AB

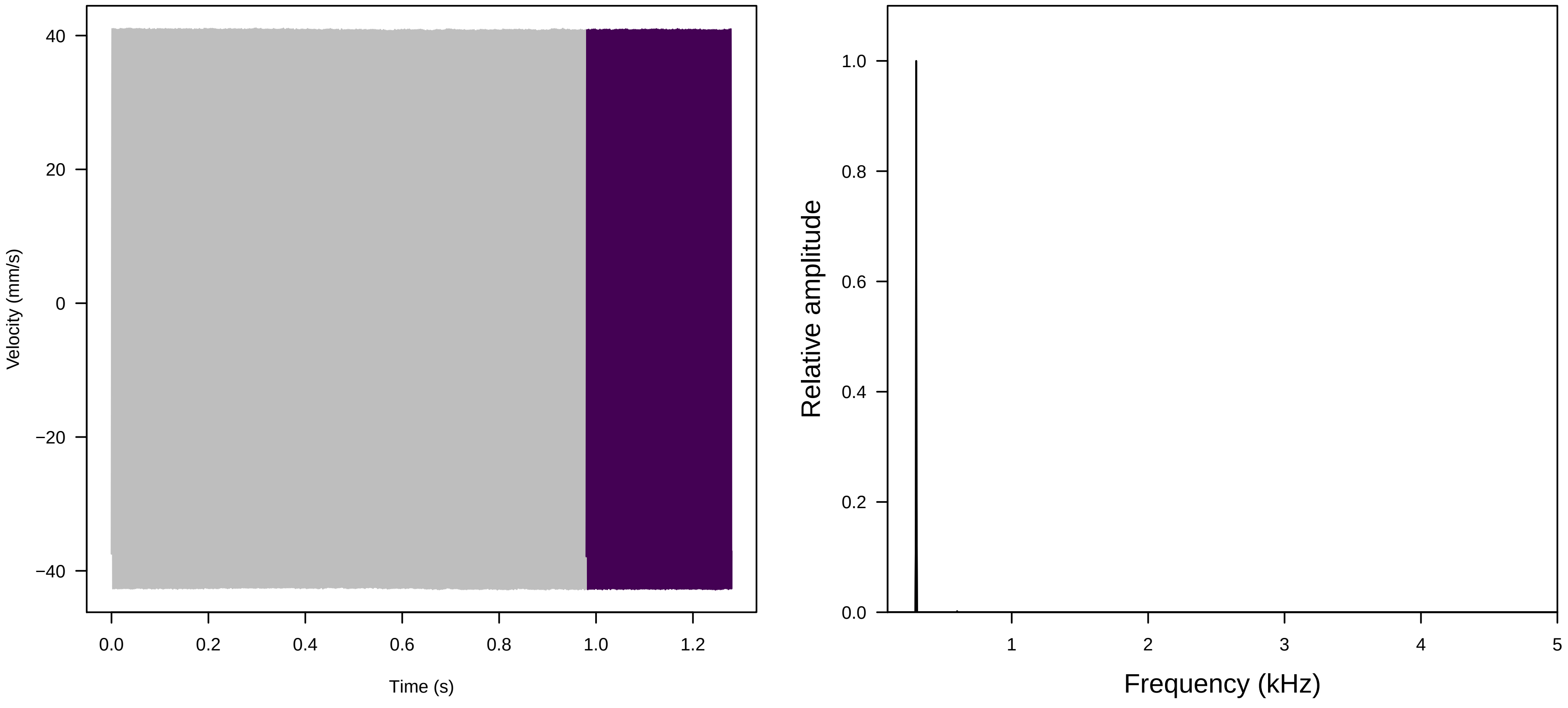

Vel. = 0.014 ; Str. = Corolla ; Axis = y ; Fl. accession = 10−s−81−1AB

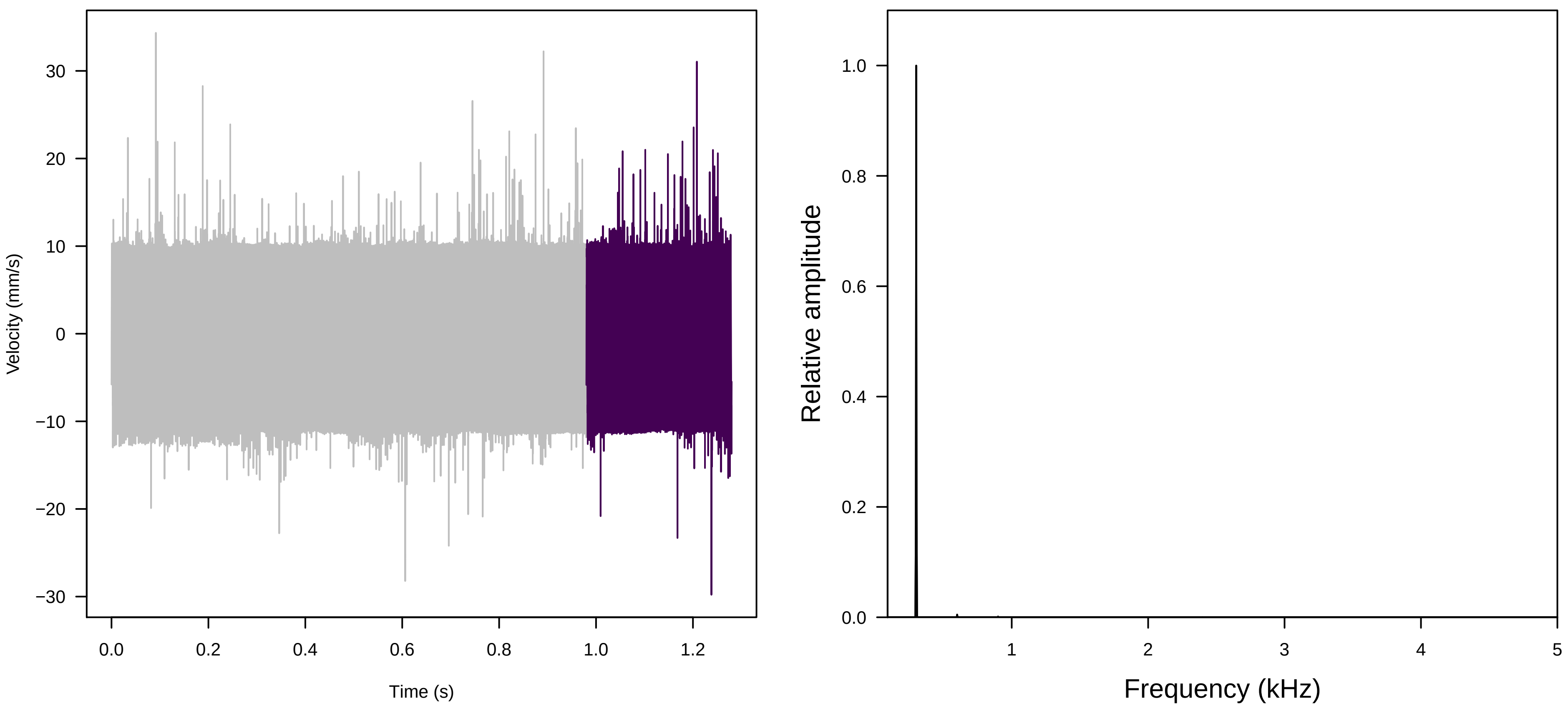

Vel. = 0.014 ; Str. = Receptacle ; Axis = y ; Fl. accession = 10−s−81−1AB

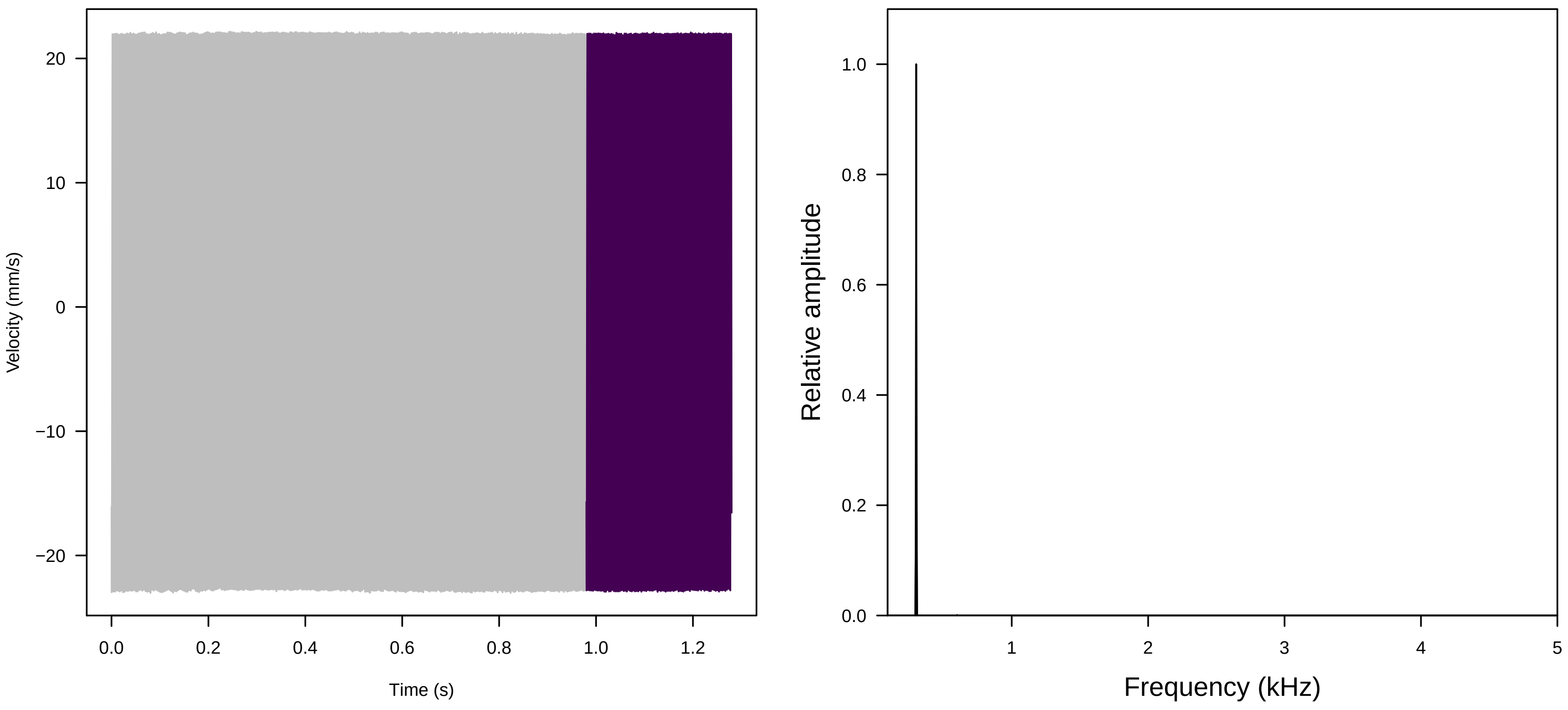

Vel. = 0.014 ; Str. = FA ; Axis = y ; Fl. accession = 10−s−81−1AB

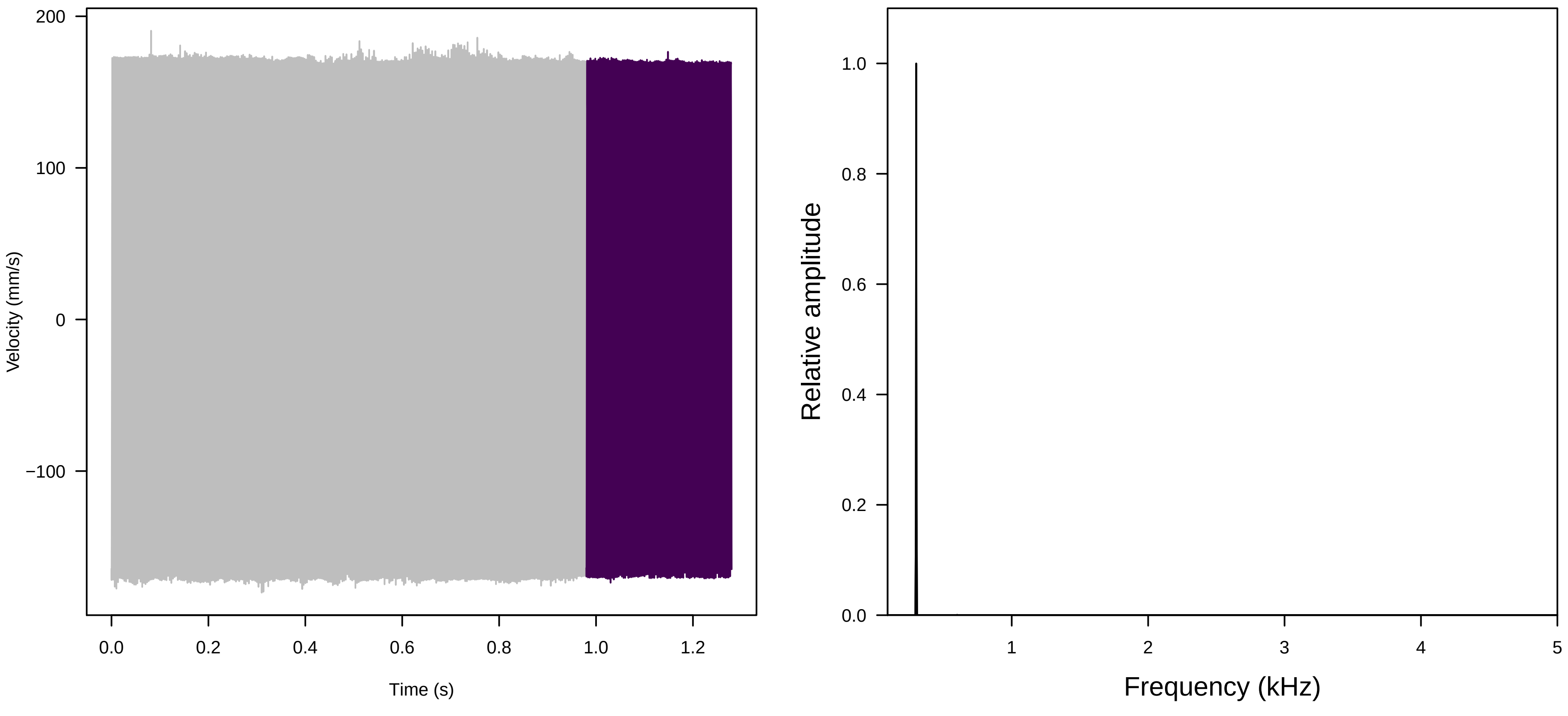

Vel. = 0.014 ; Str. = Receptacle ; Axis = y ; Fl. accession = 10−s−81−1AB

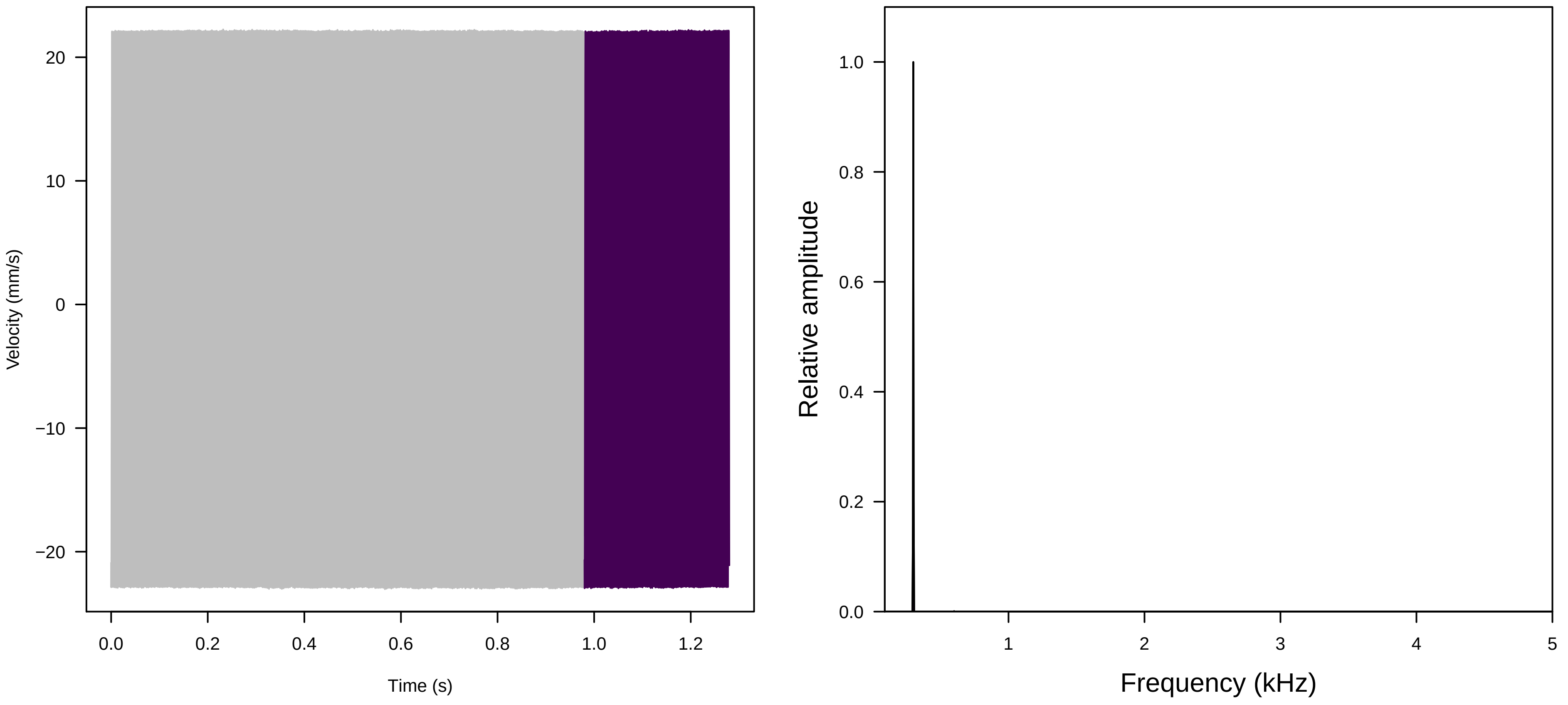

Vel. = 0.014 ; Str. = PA ; Axis = y ; Fl. accession = 10−s−81−1AB

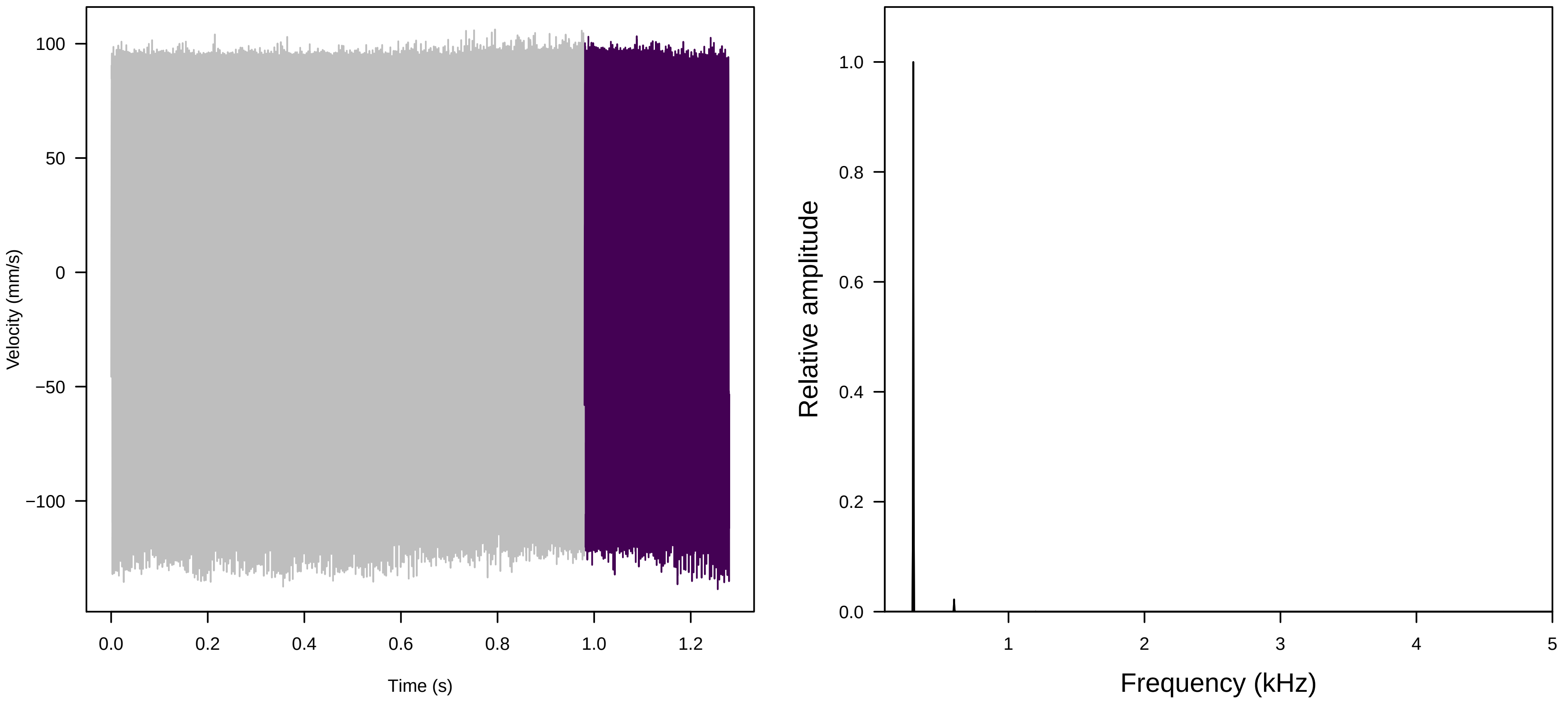

Vel. = 0.014 ; Str. = Receptacle ; Axis = y ; Fl. accession = 10−s−81−1AB

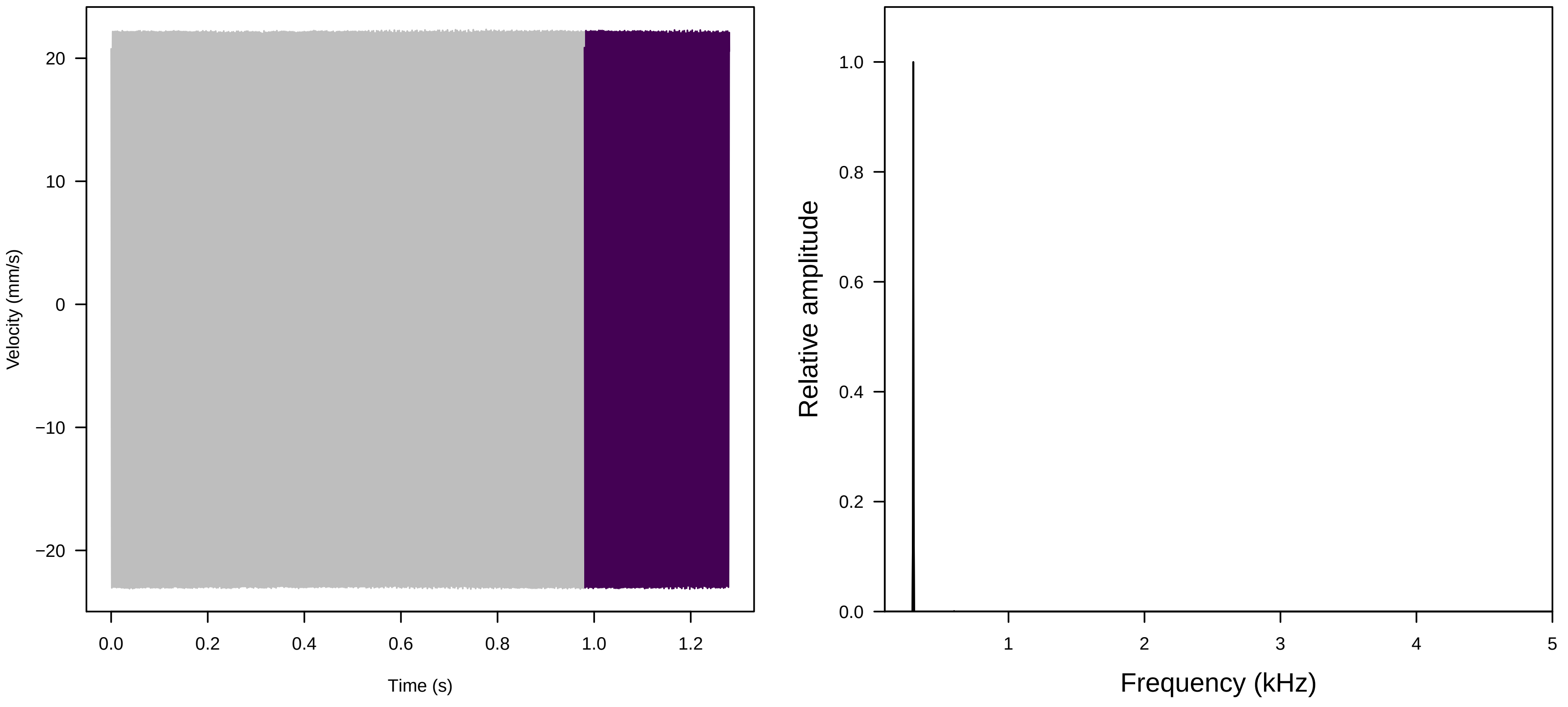

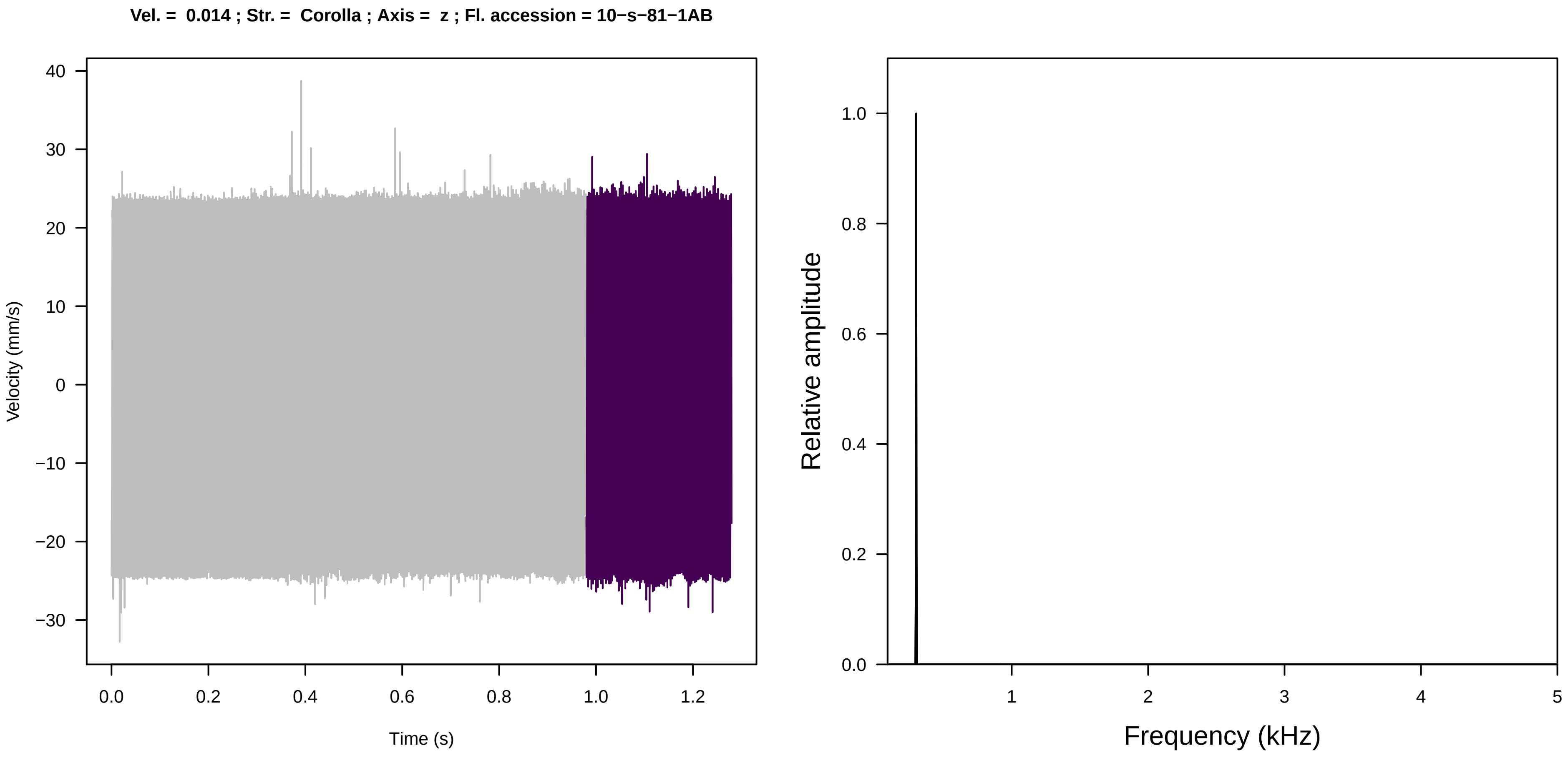
Vel. = 0.014 ; Str. = Corolla ; Axis = z ; Fl. accession = 10−s−81−1AB
Velocity (mm/s)
40
30
20
10
0
−10
−20
−30
0.0
0.2
0.4
0.6
0.8
1.0
1.2
Time (s)
Relative amplitude
1.0
0.8
0.6
0.4
0.2
0.0
1
2
3
4
5
Frequency (kHz)

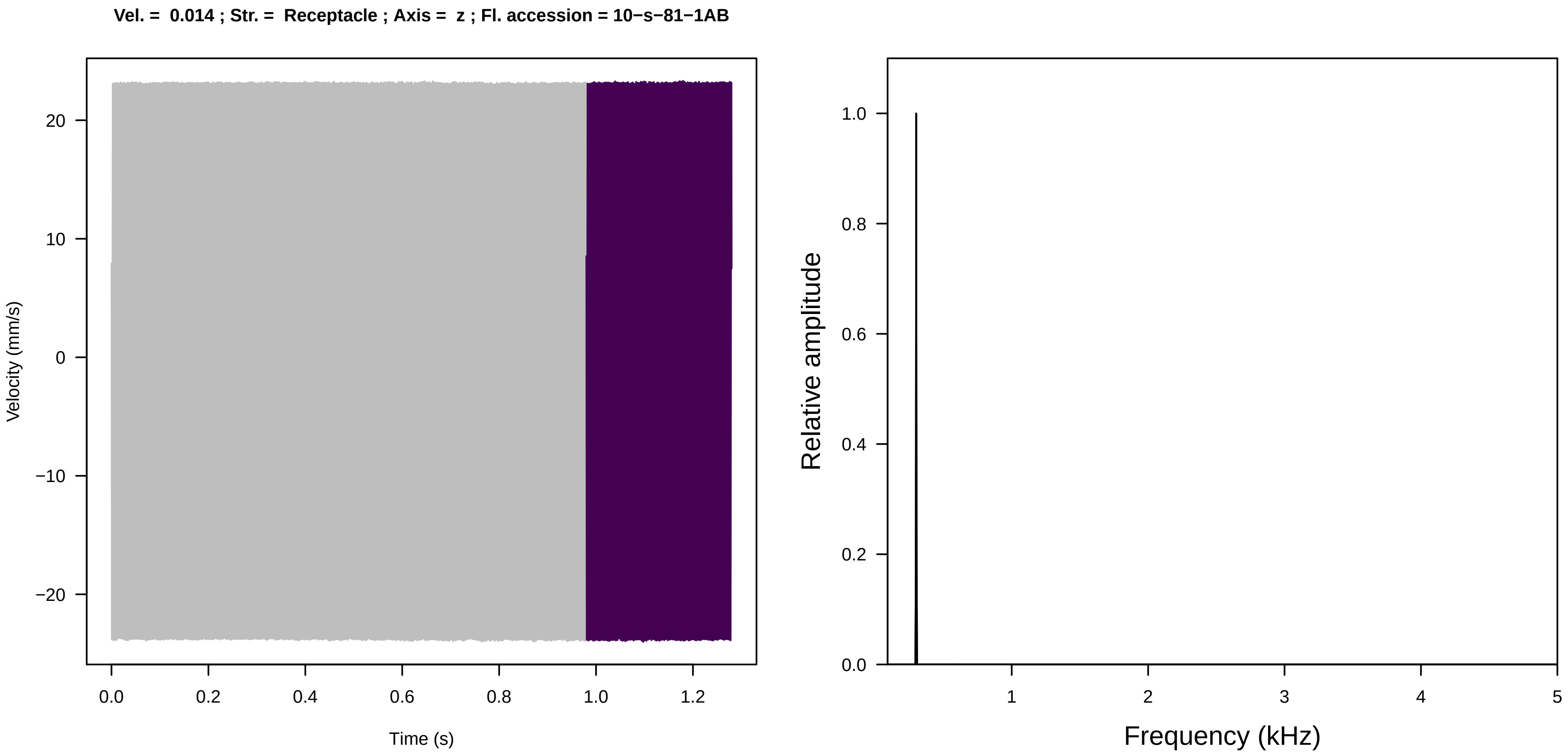
Vel. = 0.014 ; Str. = Receptacle ; Axis = z ; Fl. accession = 10−s−81−1AB
Velocity (mm/s)
20
10
0
−10
−20
0.0
0.2
0.4
0.6
0.8
1.0
1.2
Time (s)
Relative amplitude
1.0
0.8
0.6
0.4
0.2
0.0
1
2
3
4
5
Frequency (kHz)

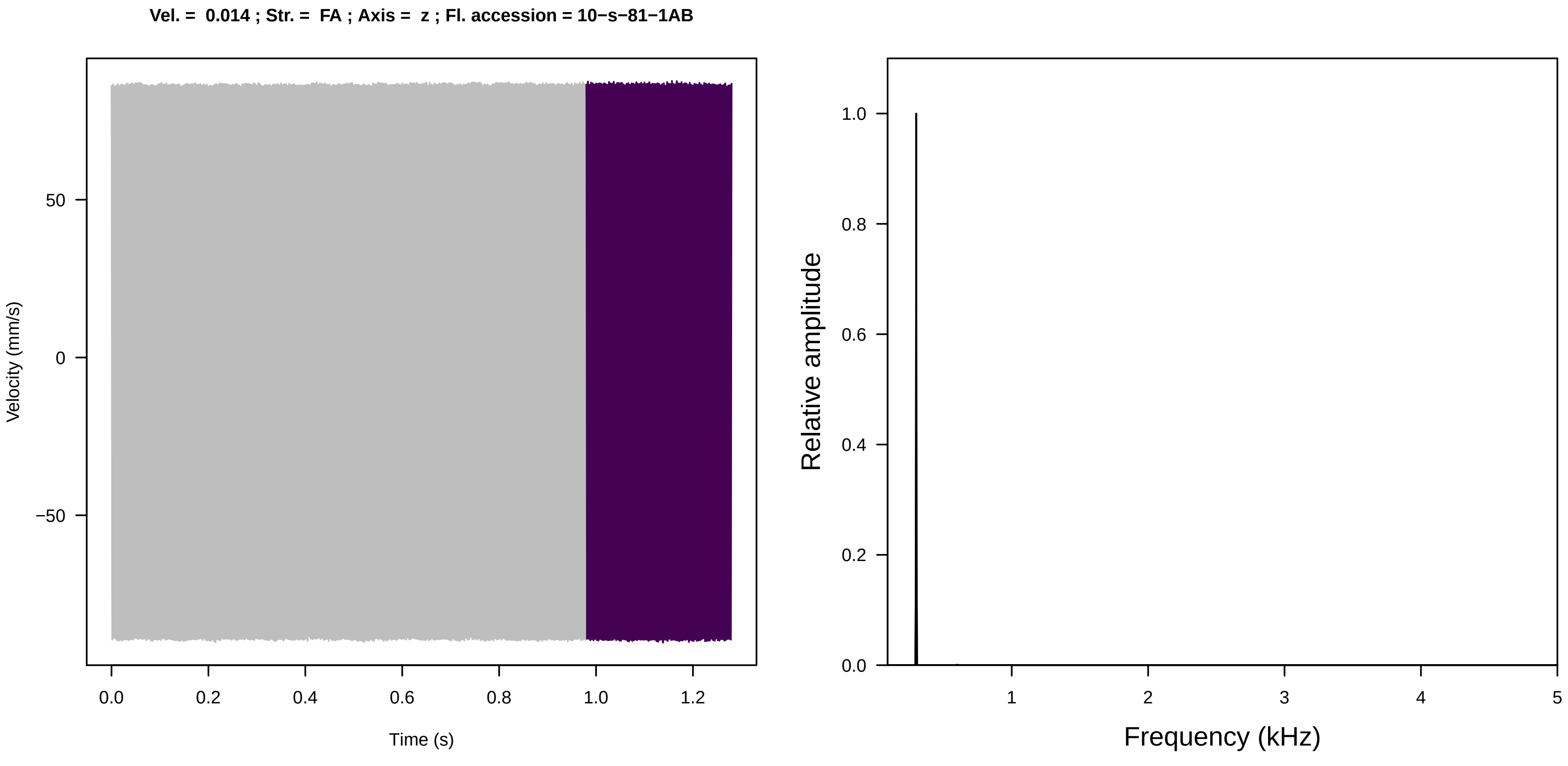
Vel. = 0.014 ; Str. = FA ; Axis = z ; Fl. accession = 10−s−81−1AB
Velocity (mm/s)
50
0
−50
0.0
0.2
0.4
0.6
0.8
1.0
1.2
Time (s)
Relative amplitude
1.0
0.8
0.6
0.4
0.2
0.0
1
2
3
4
5
Frequency (kHz)

Vel. = 0.014 ; Str. = Receptacle ; Axis = z ; Fl. accession = 10−s−81−1AB

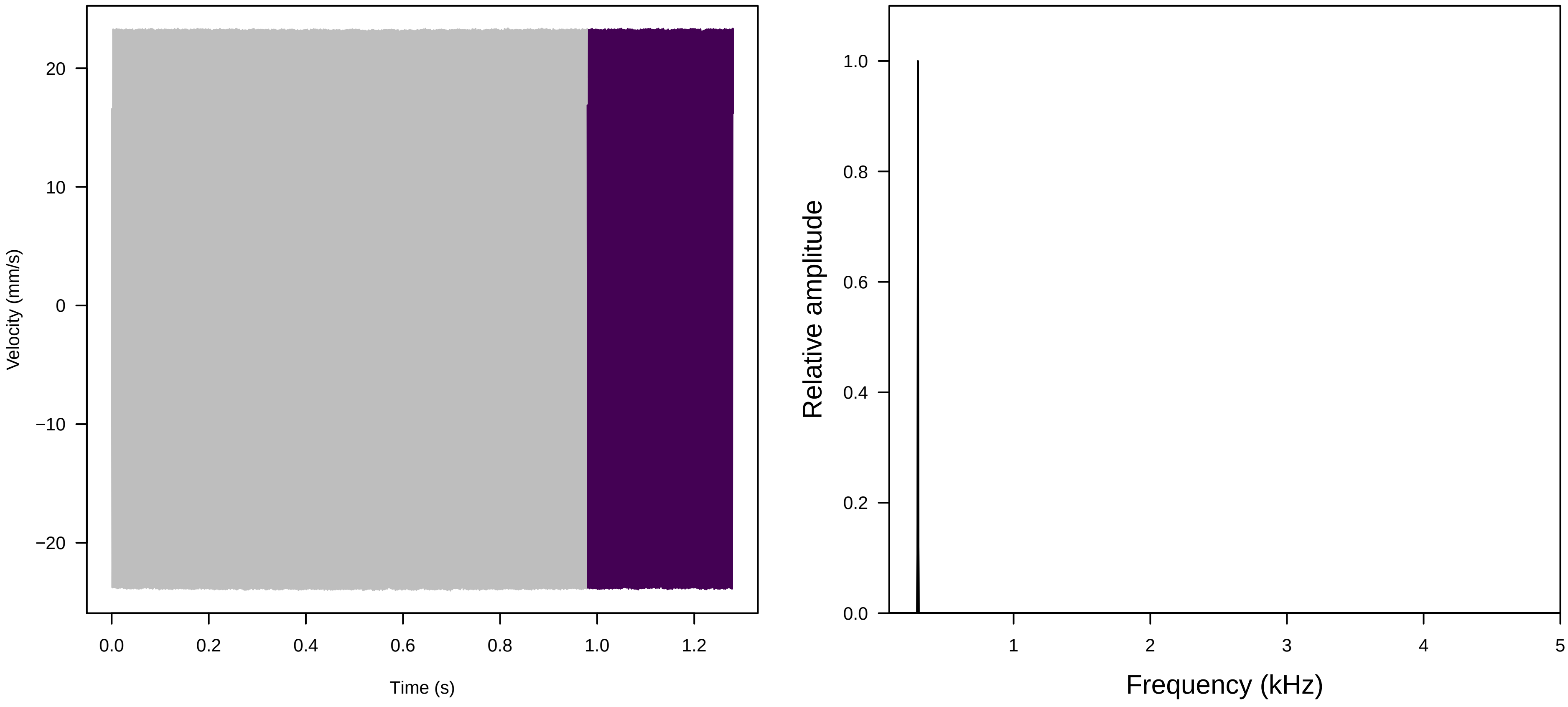

Vel. = 0.014 ; Str. = PA ; Axis = z ; Fl. accession = 10−s−81−1AB

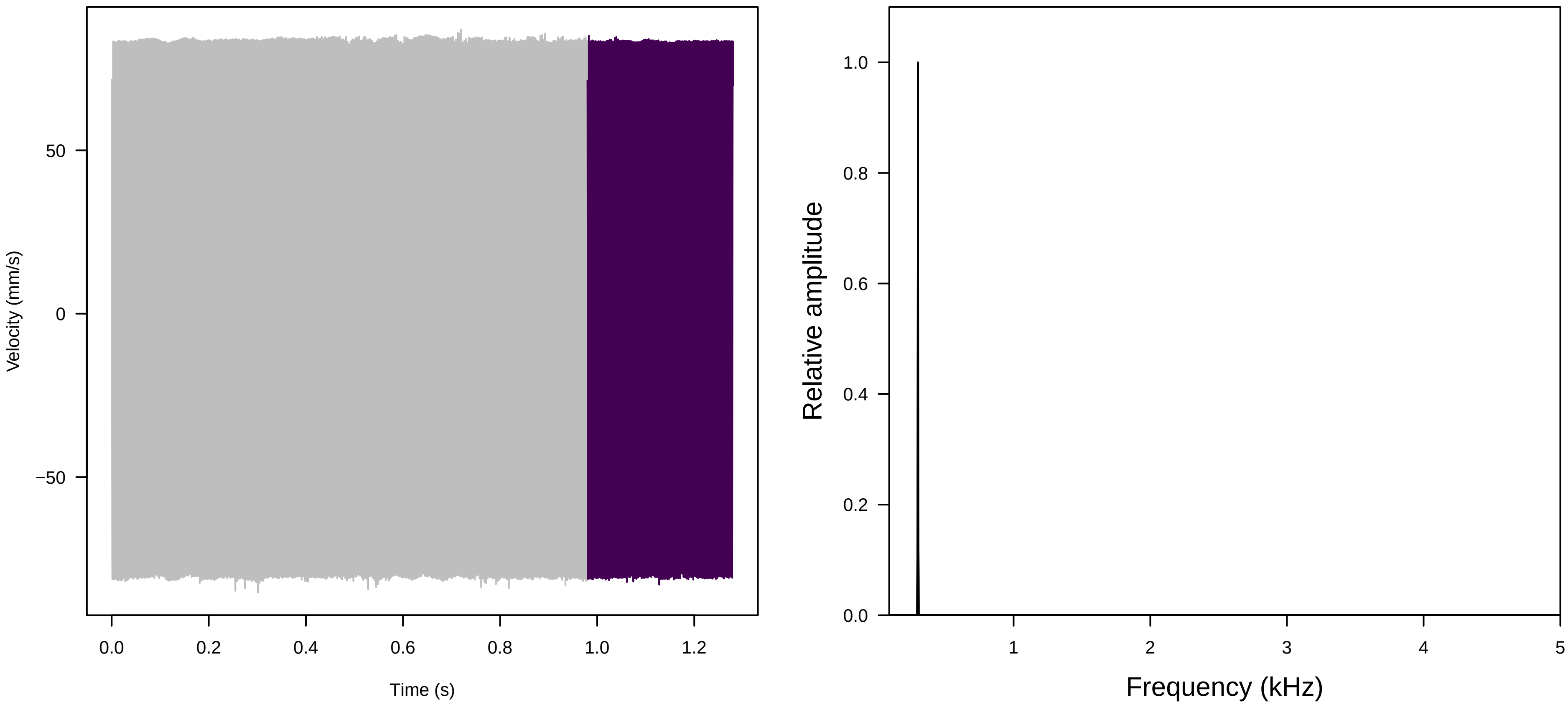

Vel. = 0.014 ; Str. = Receptacle ; Axis = z ; Fl. accession = 10−s−81−1AB

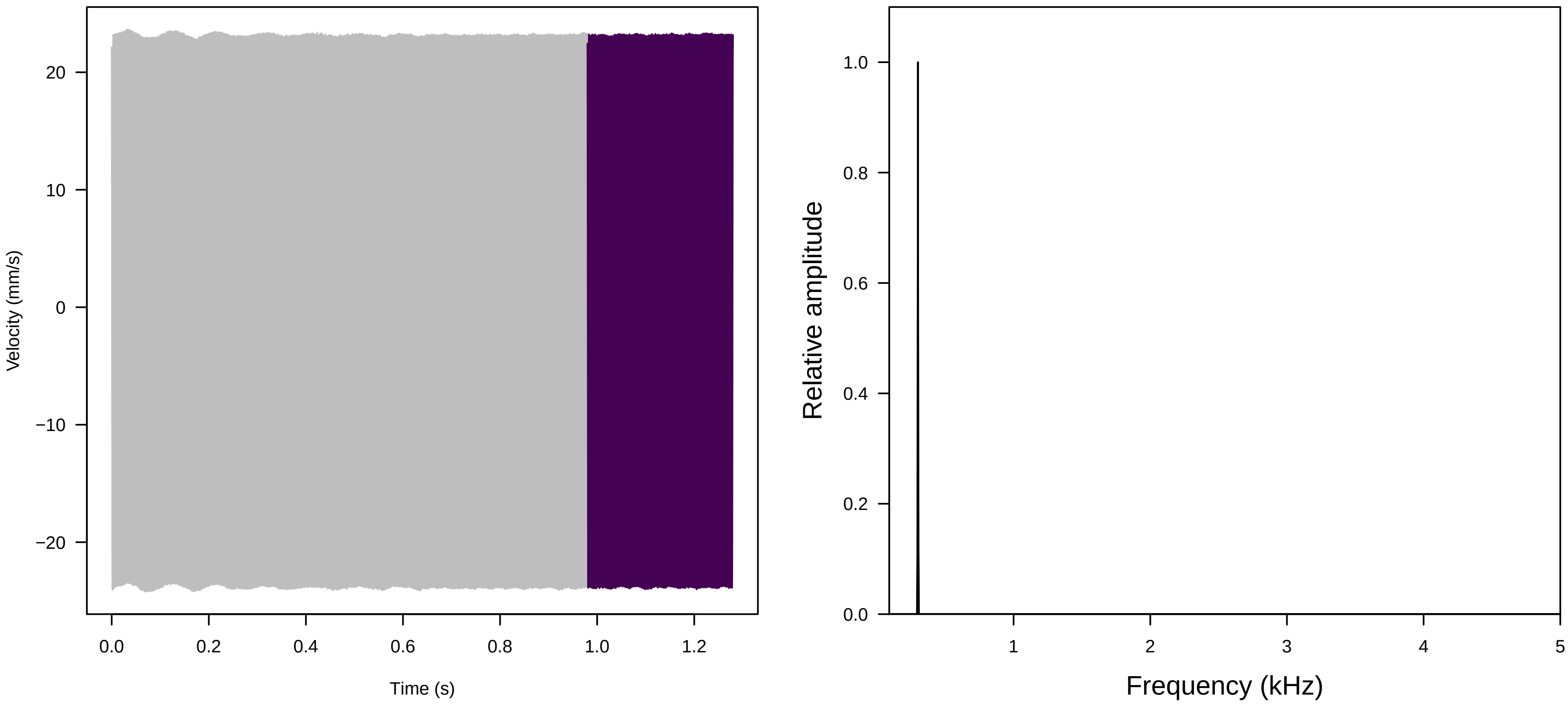

Vel. = 0.028 ; Str. = PA ; Axis = z ; Fl. accession = 10−s−81−1AB

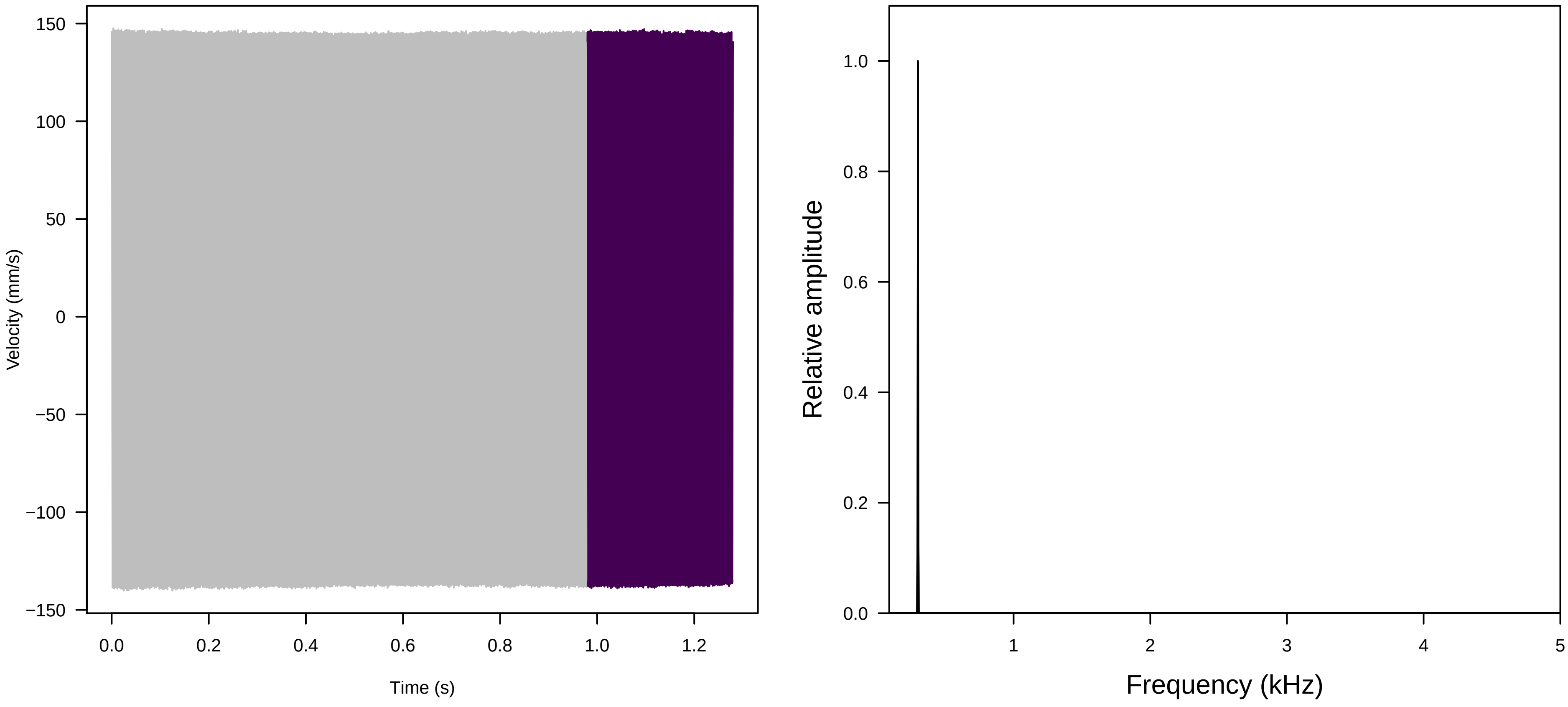

Vel. = 0.028 ; Str. = Receptacle ; Axis = z ; Fl. accession = 10−s−81−1AB

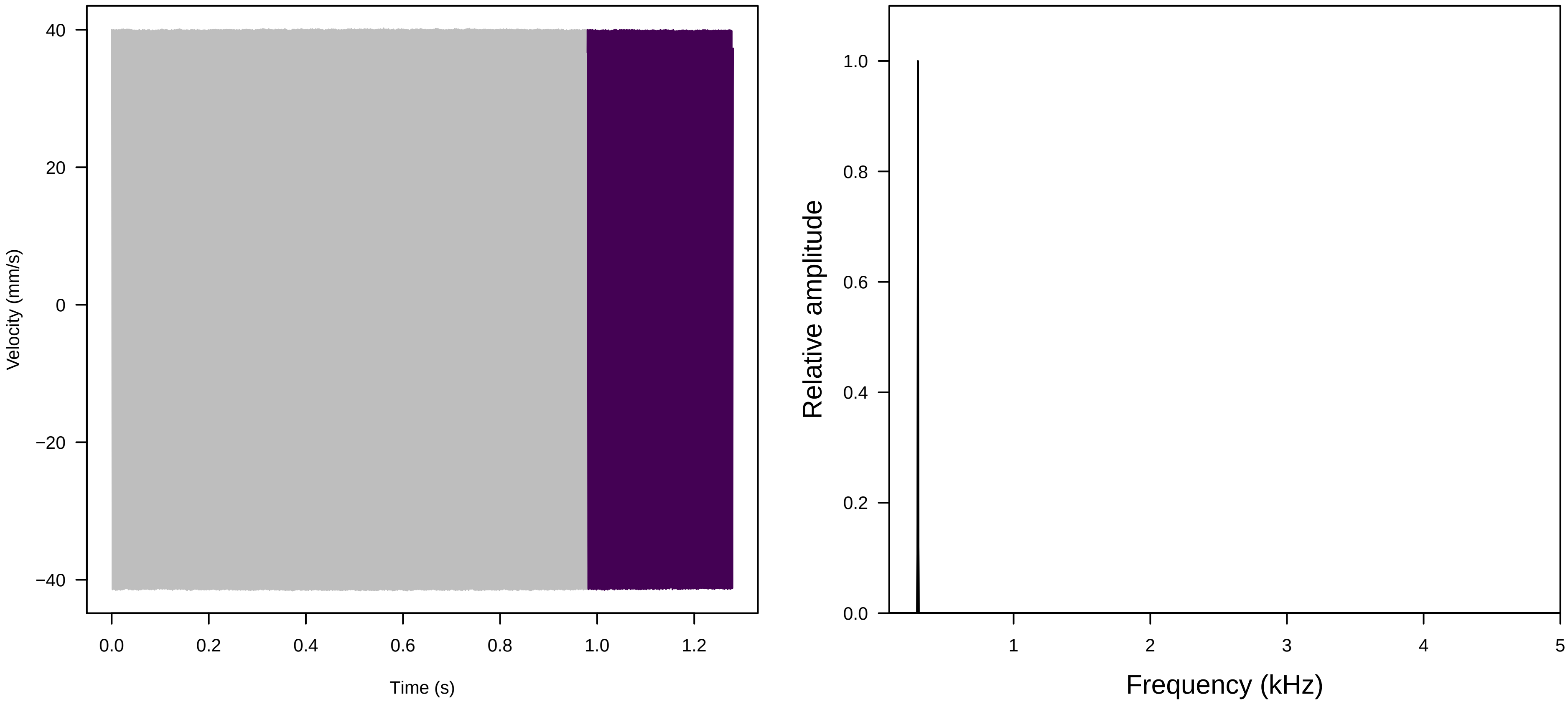

Vel. = 0.028 ; Str. = FA ; Axis = z ; Fl. accession = 10−s−81−1AB

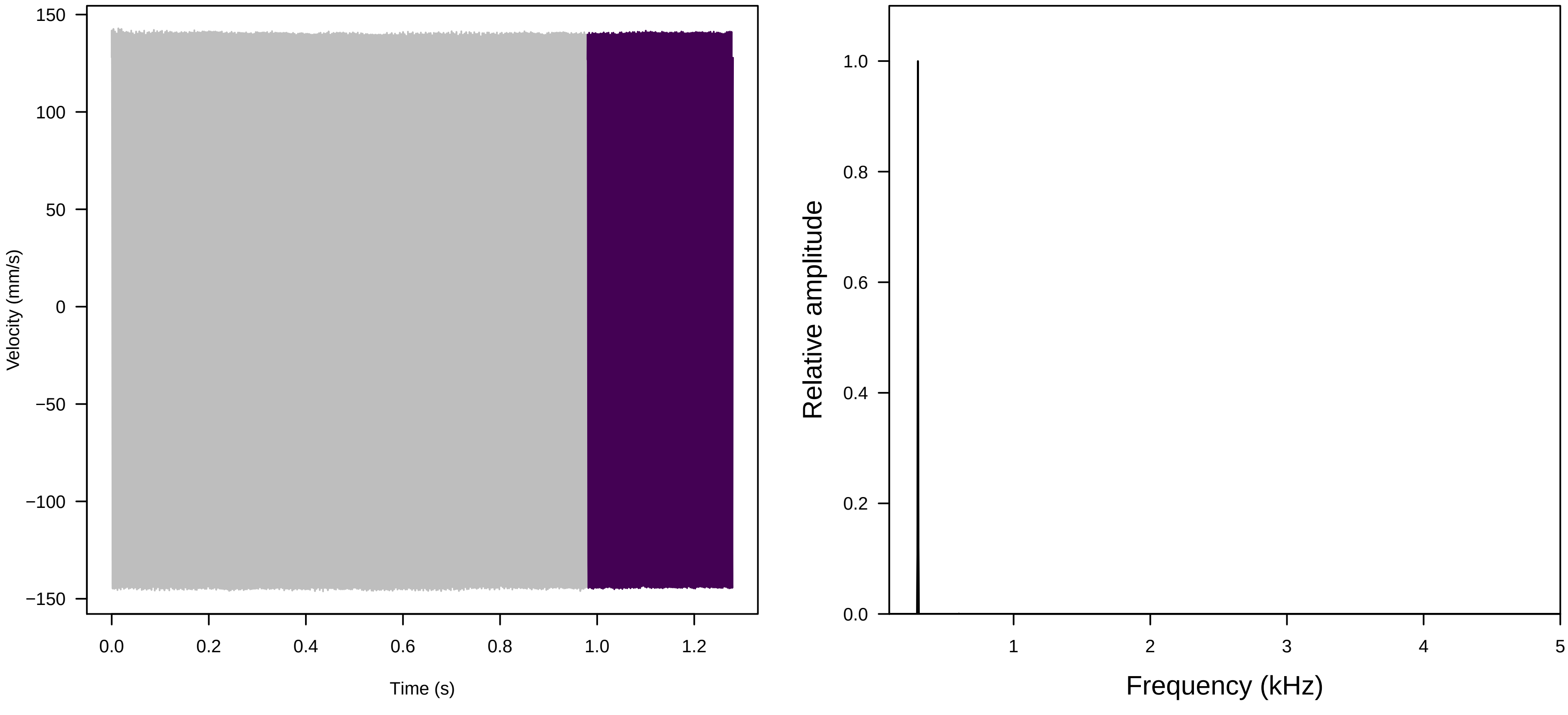

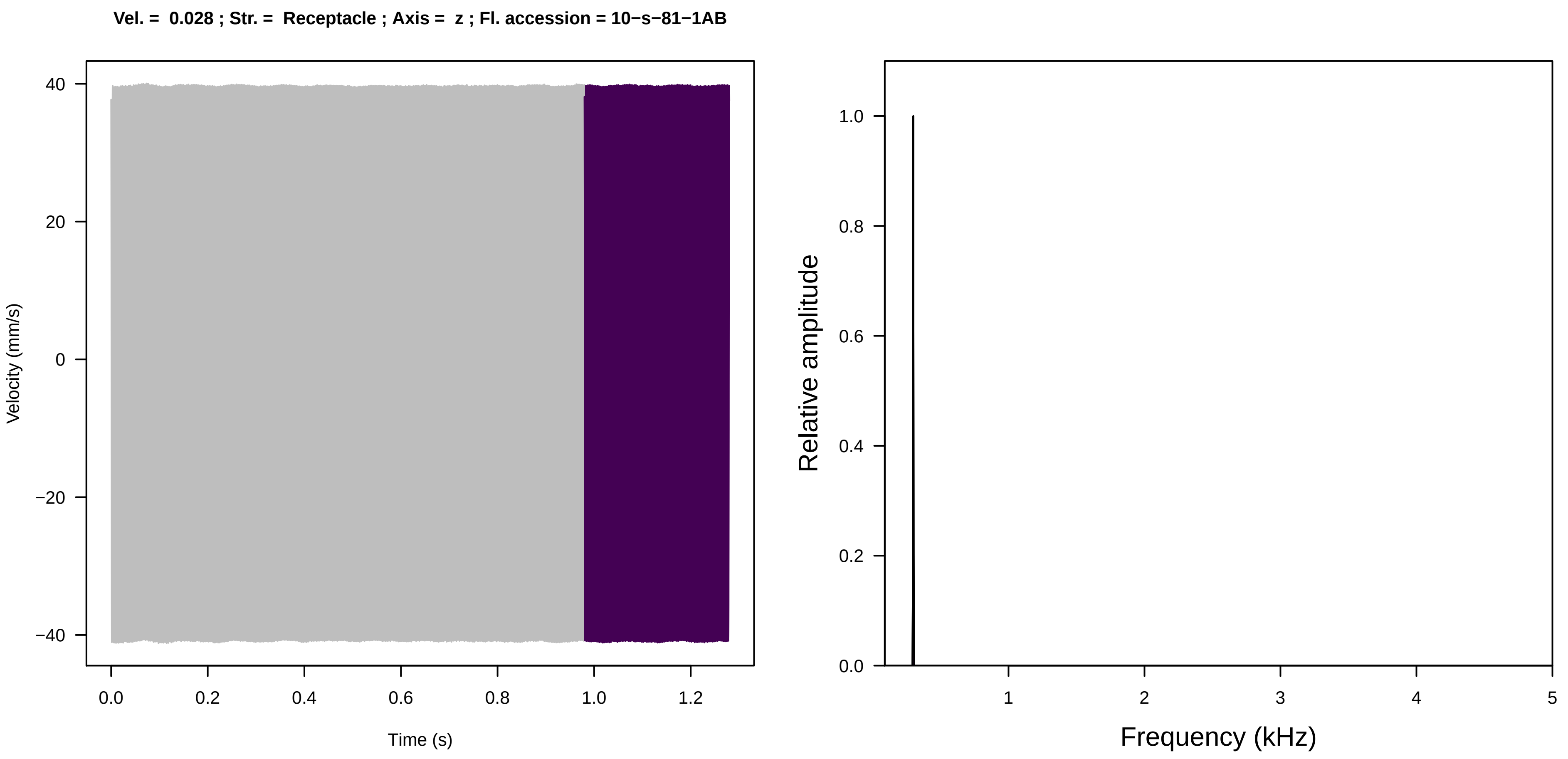
Vel. = 0.028 ; Str. = Receptacle ; Axis = z ; Fl. accession = 10−s−81−1AB
Velocity (mm/s)
Time (s)
Relative amplitude
Frequency (kHz)

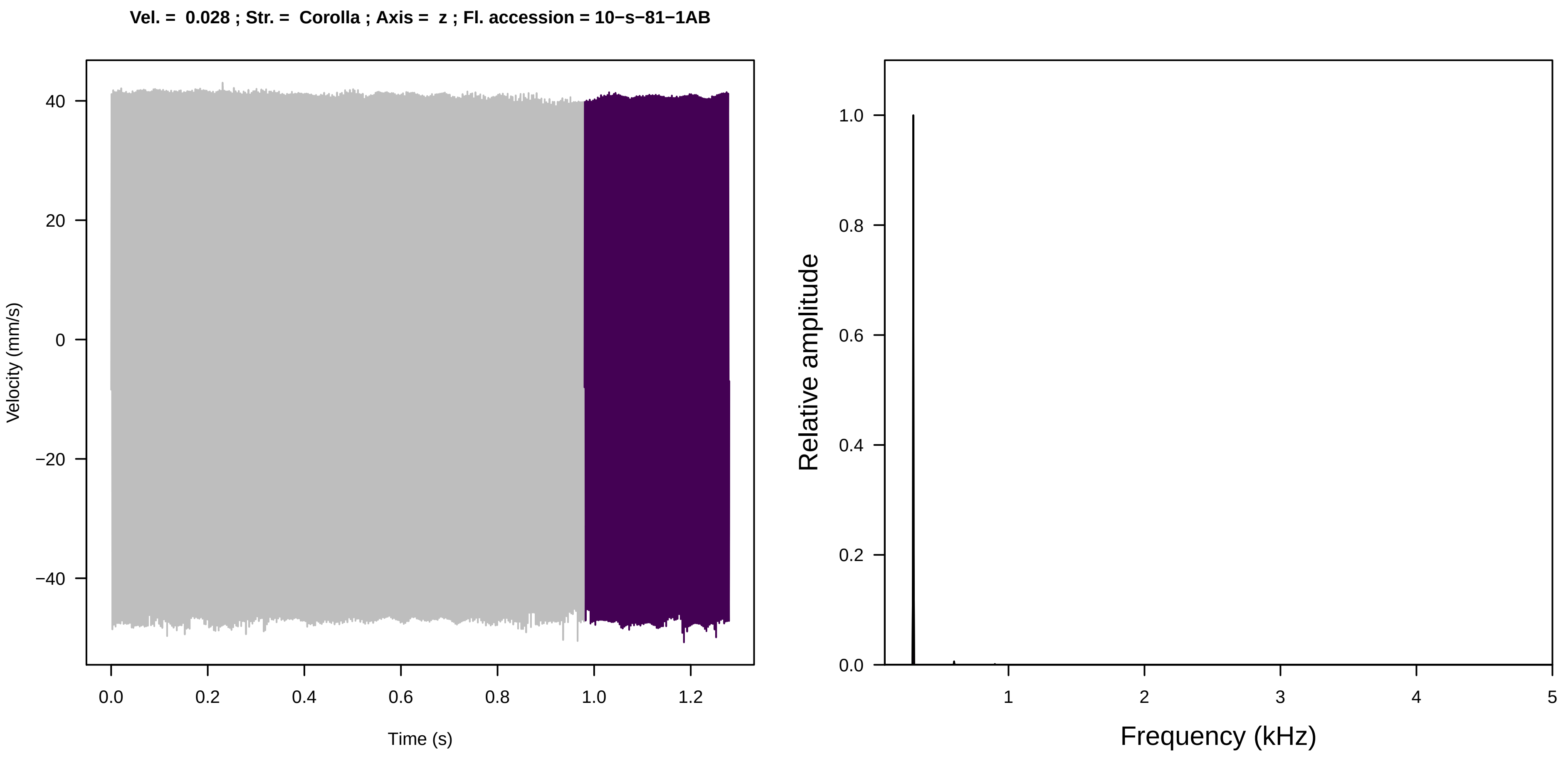
Vel. = 0.028 ; Str. = Corolla ; Axis = z ; Fl. accession = 10−s−81−1AB
Velocity (mm/s)
Time (s)
Relative amplitude
Frequency (kHz)

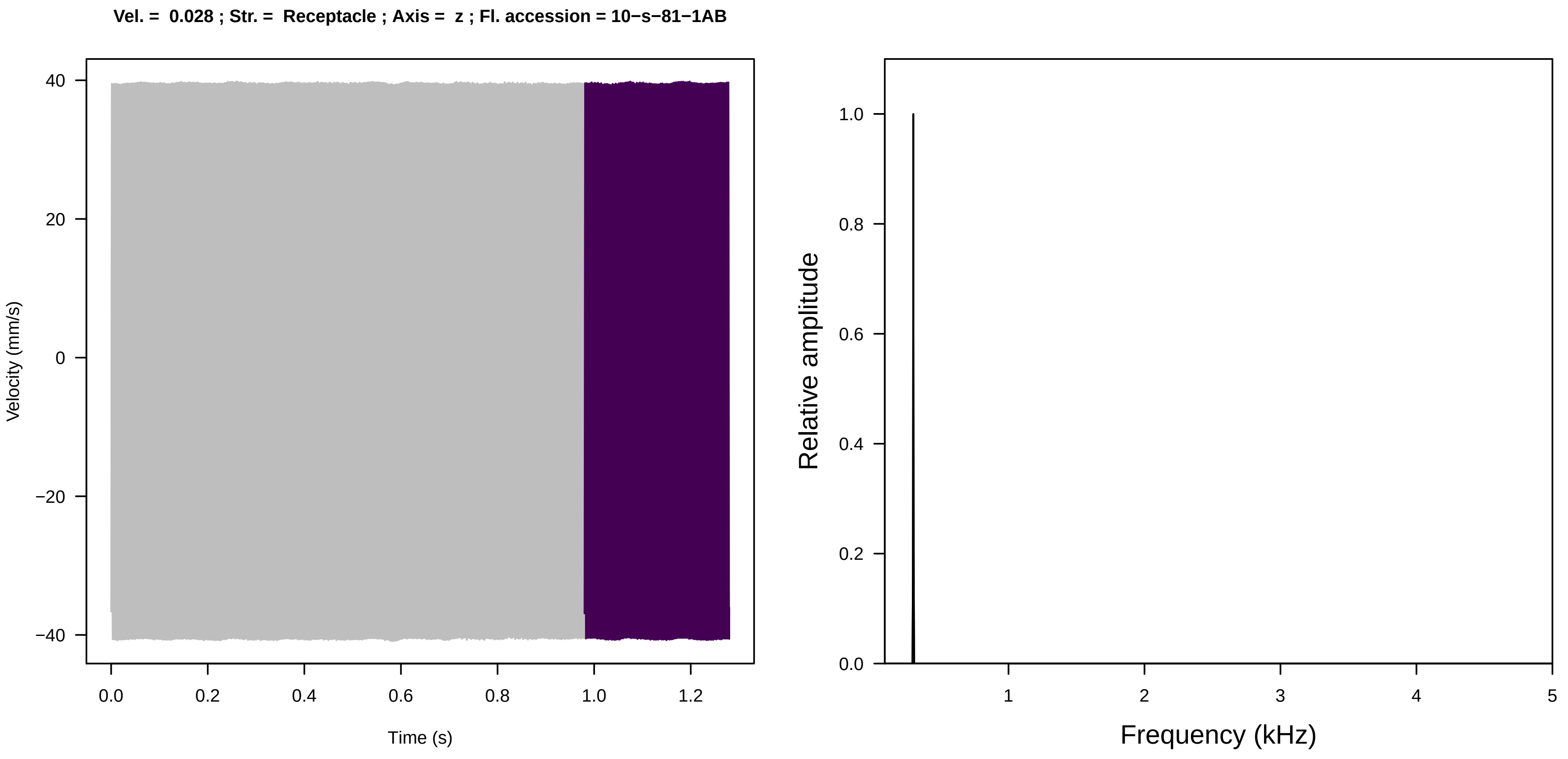
Vel. = 0.028 ; Str. = Receptacle ; Axis = z ; Fl. accession = 10−s−81−1AB
Velocity (mm/s)
Time (s)
Relative amplitude
Frequency (kHz)

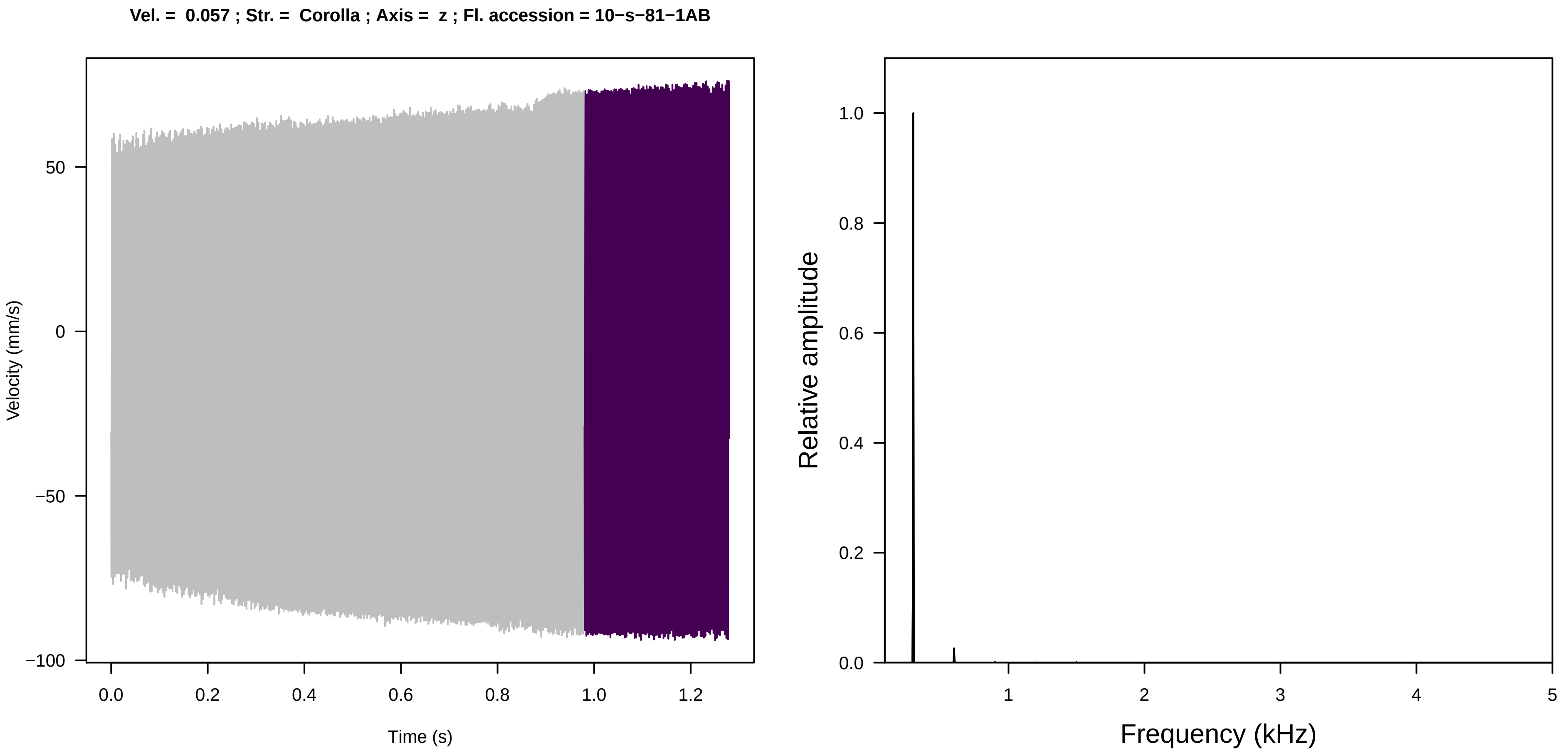
Vel. = 0.057 ; Str. = Corolla ; Axis = z ; Fl. accession = 10−s−81−1AB
Velocity (mm/s)
Time (s)
Relative amplitude
Frequency (kHz)

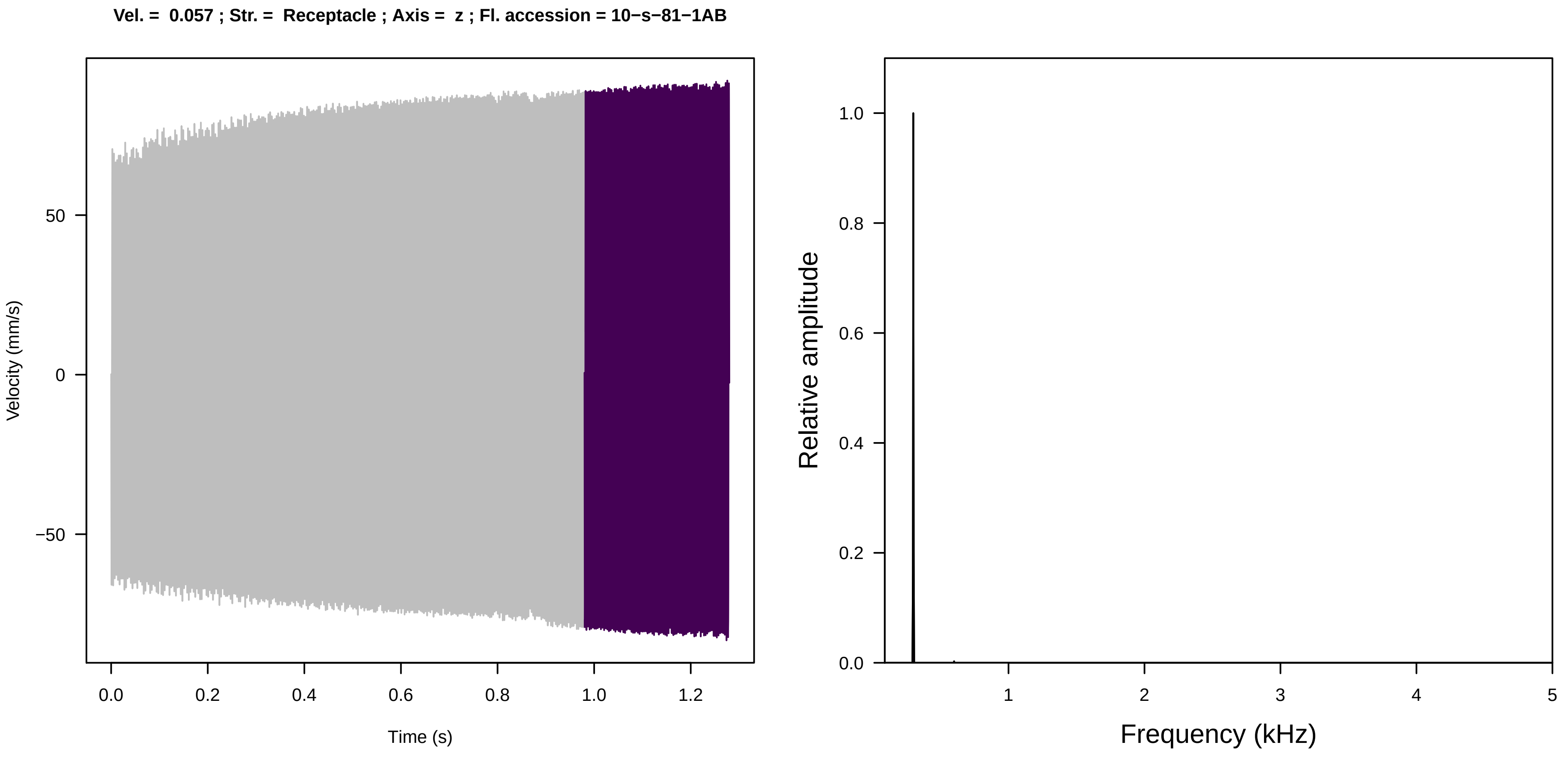
Vel. = 0.057 ; Str. = Receptacle ; Axis = z ; Fl. accession = 10−s−81−1AB
Velocity (mm/s)
Time (s)
Relative amplitude
Frequency (kHz)

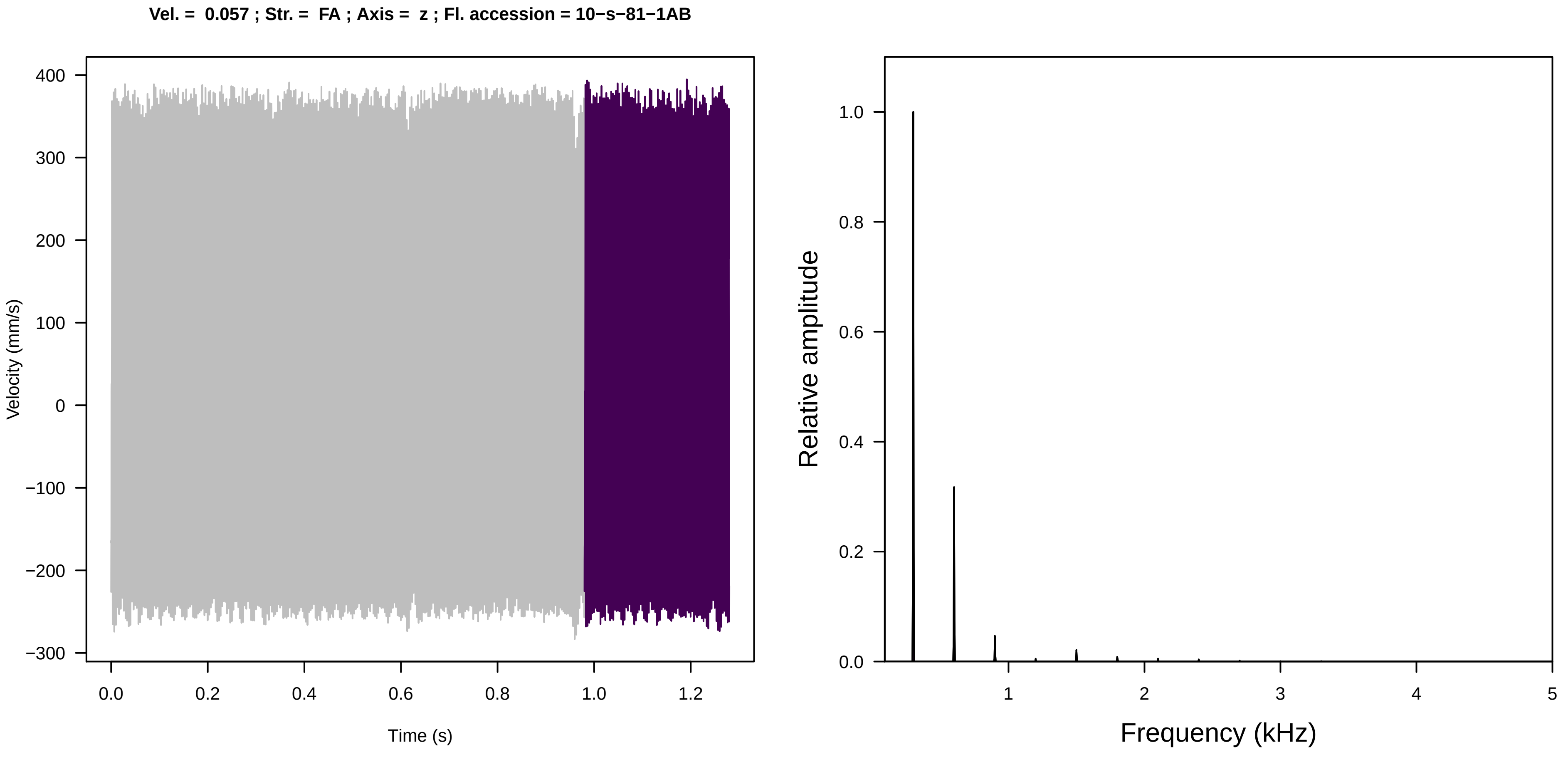
Vel. = 0.057 ; Str. = FA ; Axis = z ; Fl. accession = 10−s−81−1AB
Velocity (mm/s)
Time (s)
Relative amplitude
Frequency (kHz)

Vel. = 0.057 ; Str. = Receptacle ; Axis = z ; Fl. accession = 10−s−81−1AB

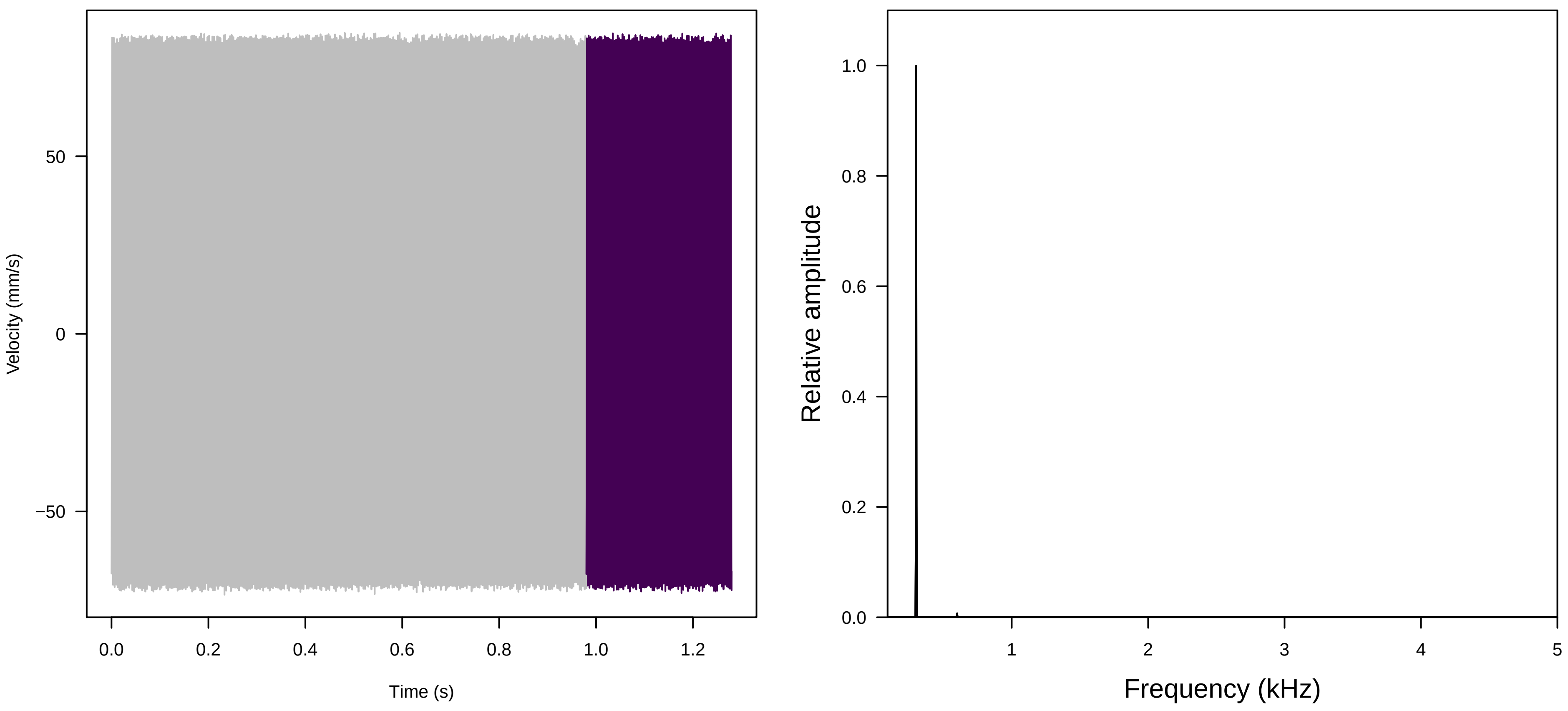

Vel. = 0.057 ; Str. = PA ; Axis = z ; Fl. accession = 10−s−81−1AB

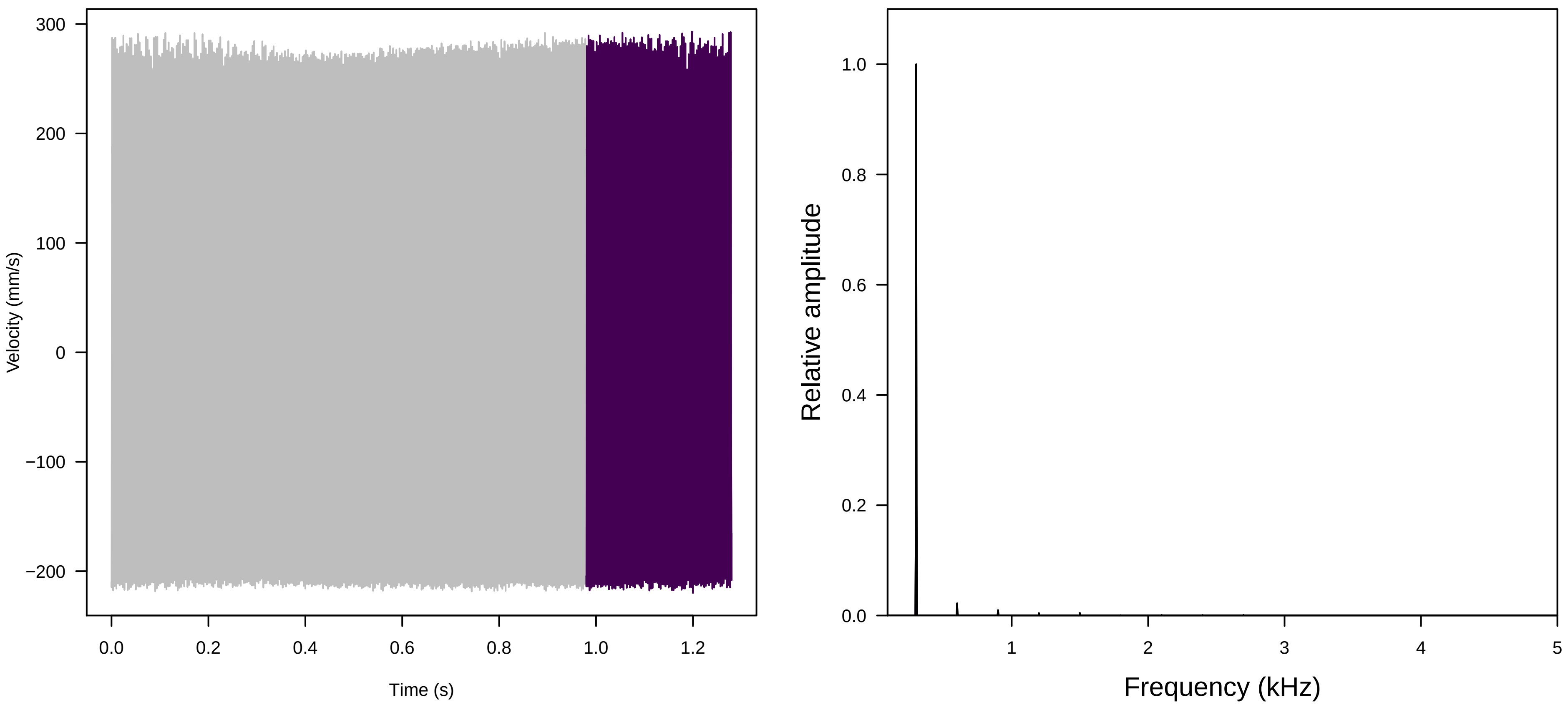

Vel. = 0.057 ; Str. = Receptacle ; Axis = z ; Fl. accession = 10−s−81−1AB

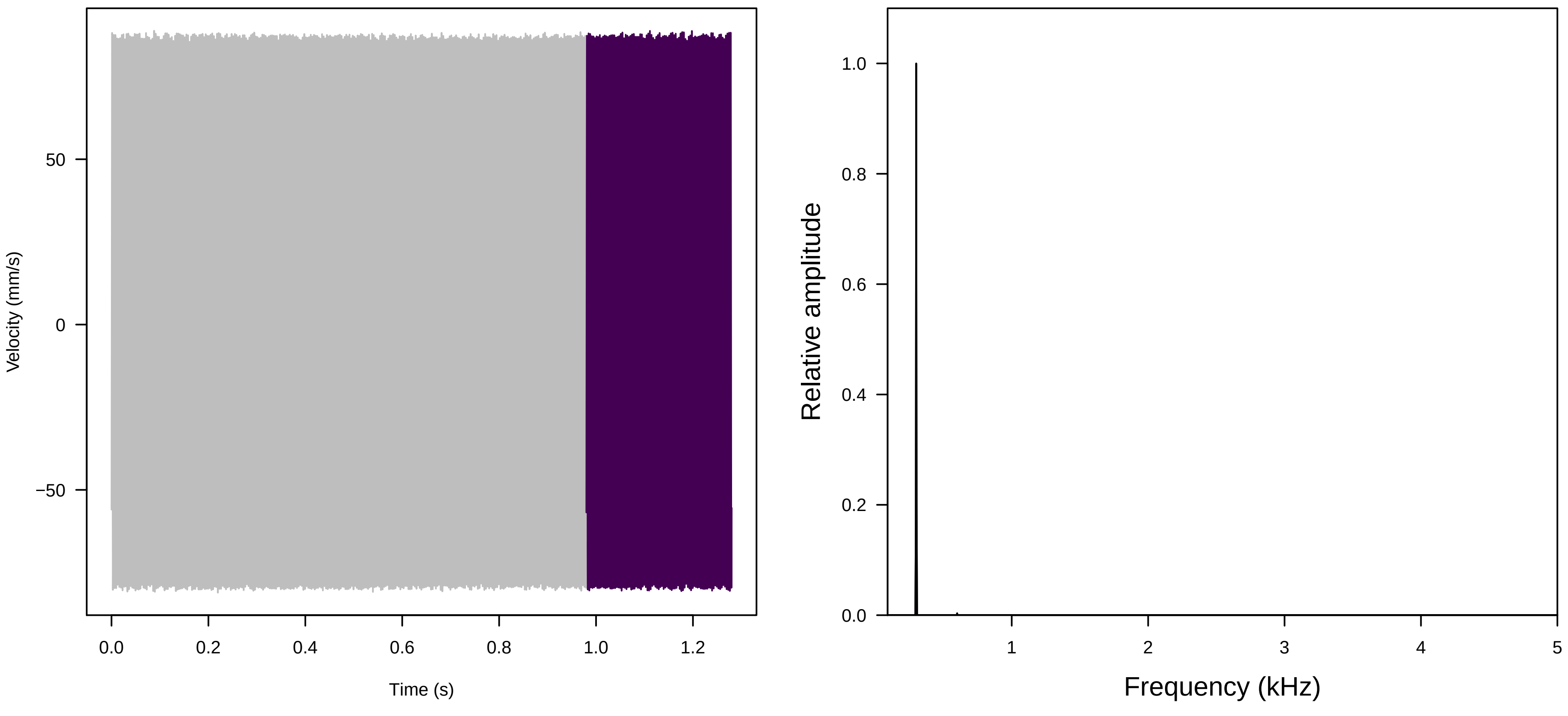

Vel. = 0.057 ; Str. = Corolla ; Axis = x ; Fl. accession = 10−s−81−1AB

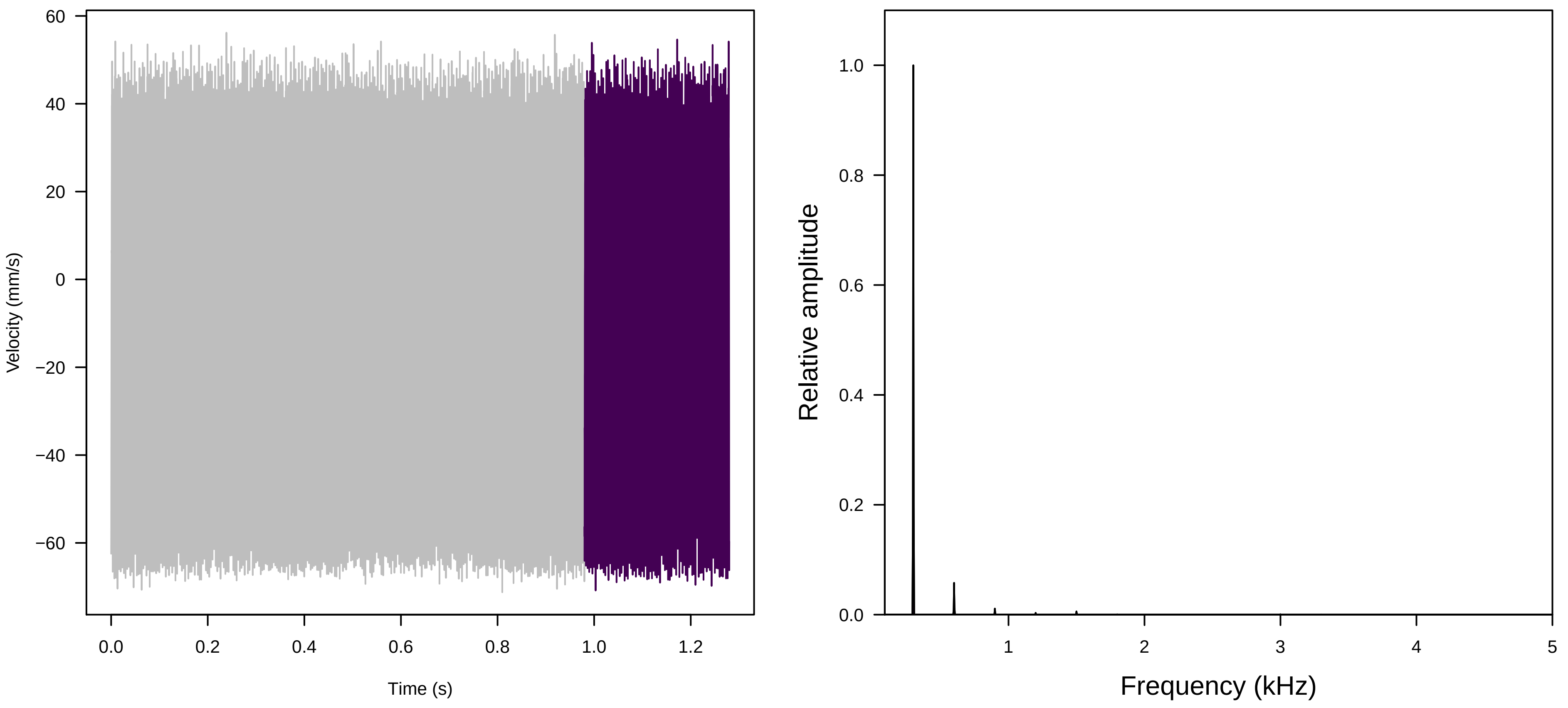

Vel. = 0.057 ; Str. = Receptacle ; Axis = x ; Fl. accession = 10−s−81−1AB

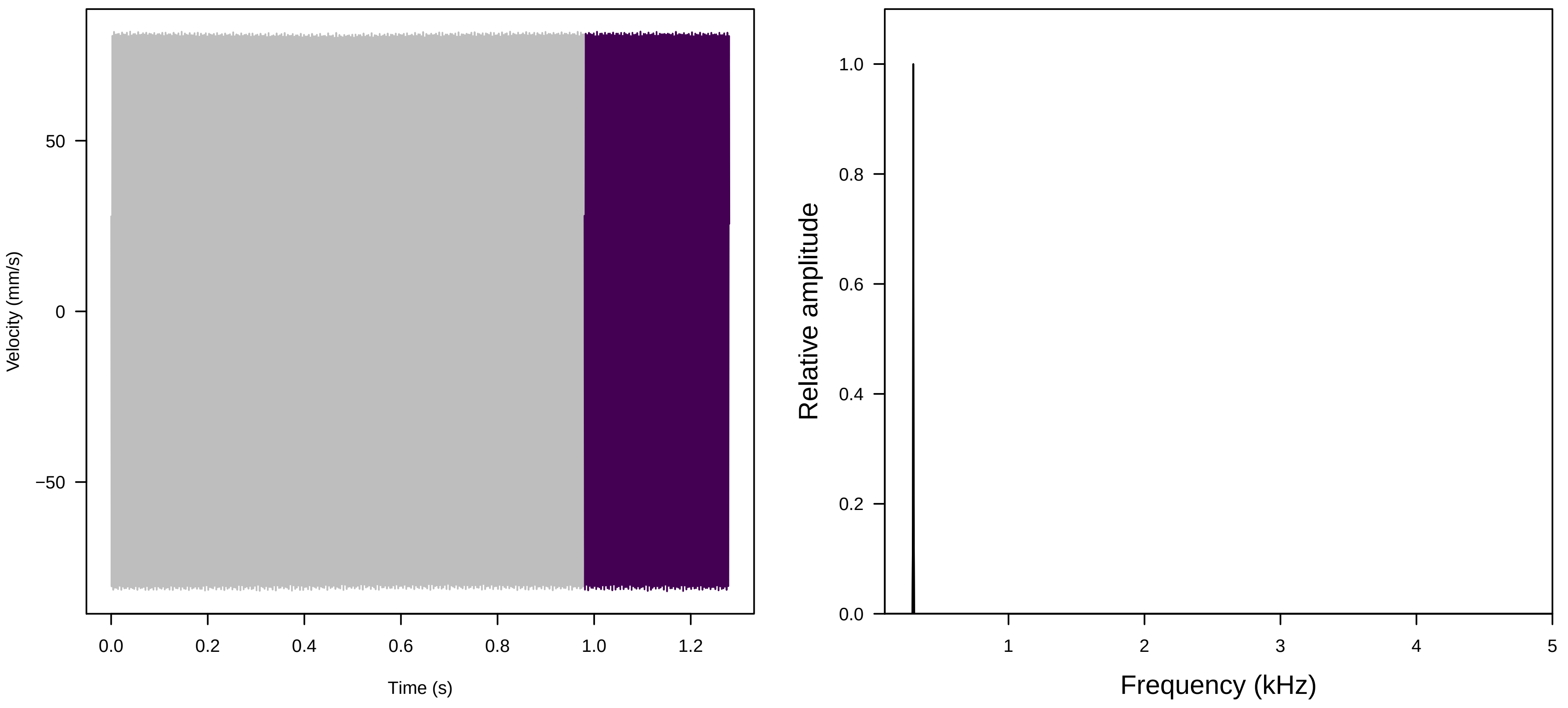

Vel. = 0.057 ; Str. = FA ; Axis = x ; Fl. accession = 10−s−81−1AB

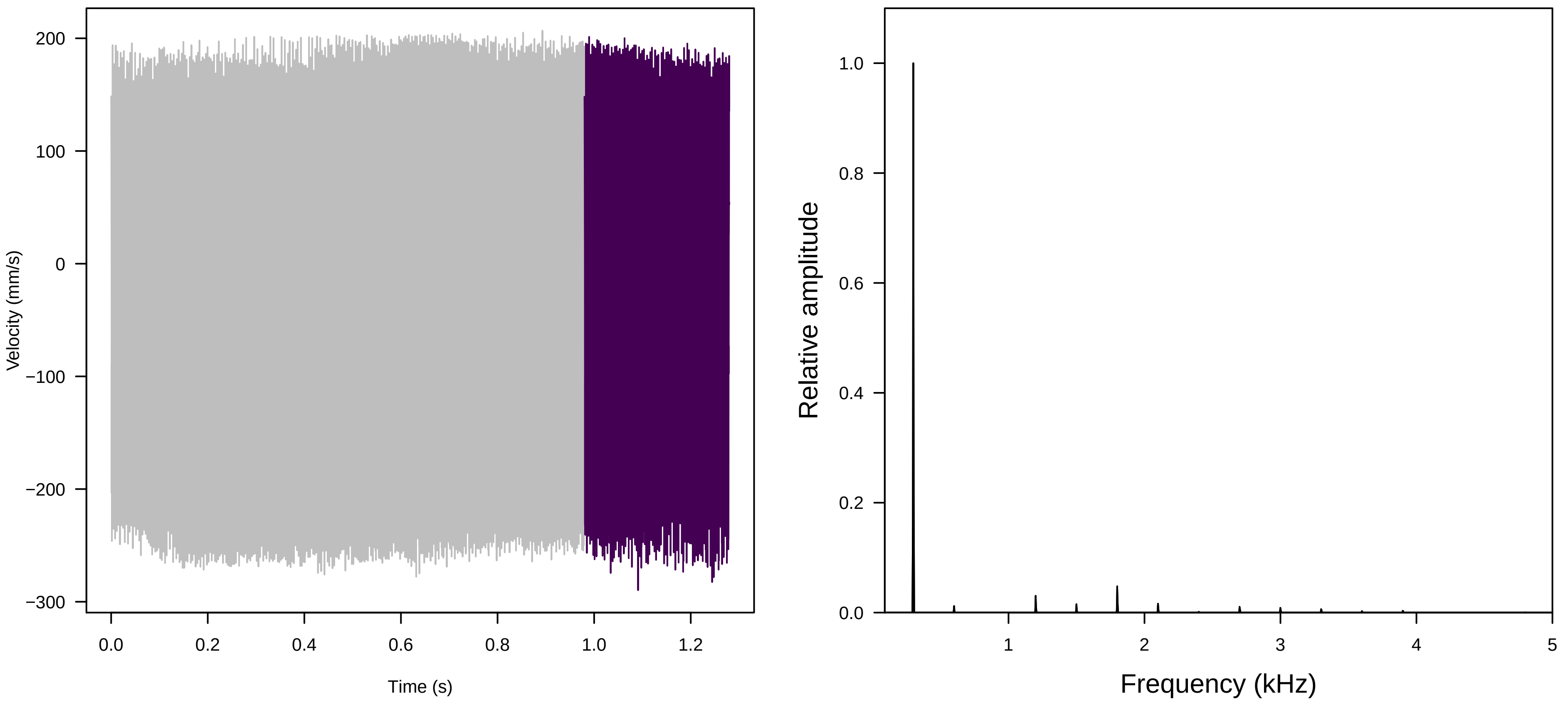

Vel. = 0.057 ; Str. = Receptacle ; Axis = x ; Fl. accession = 10−s−81−1AB

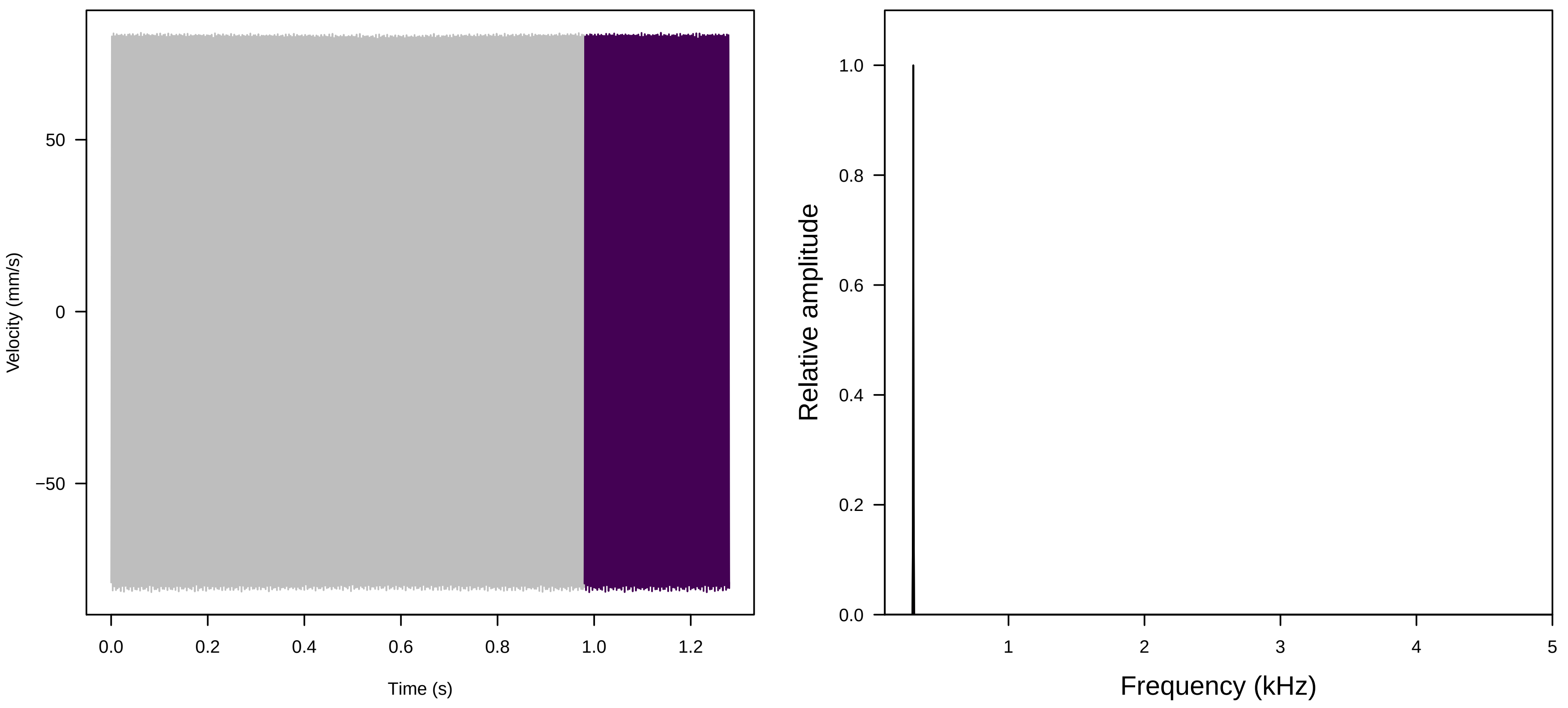

Vel. = 0.057 ; Str. = PA ; Axis = x ; Fl. accession = 10−s−81−1AB

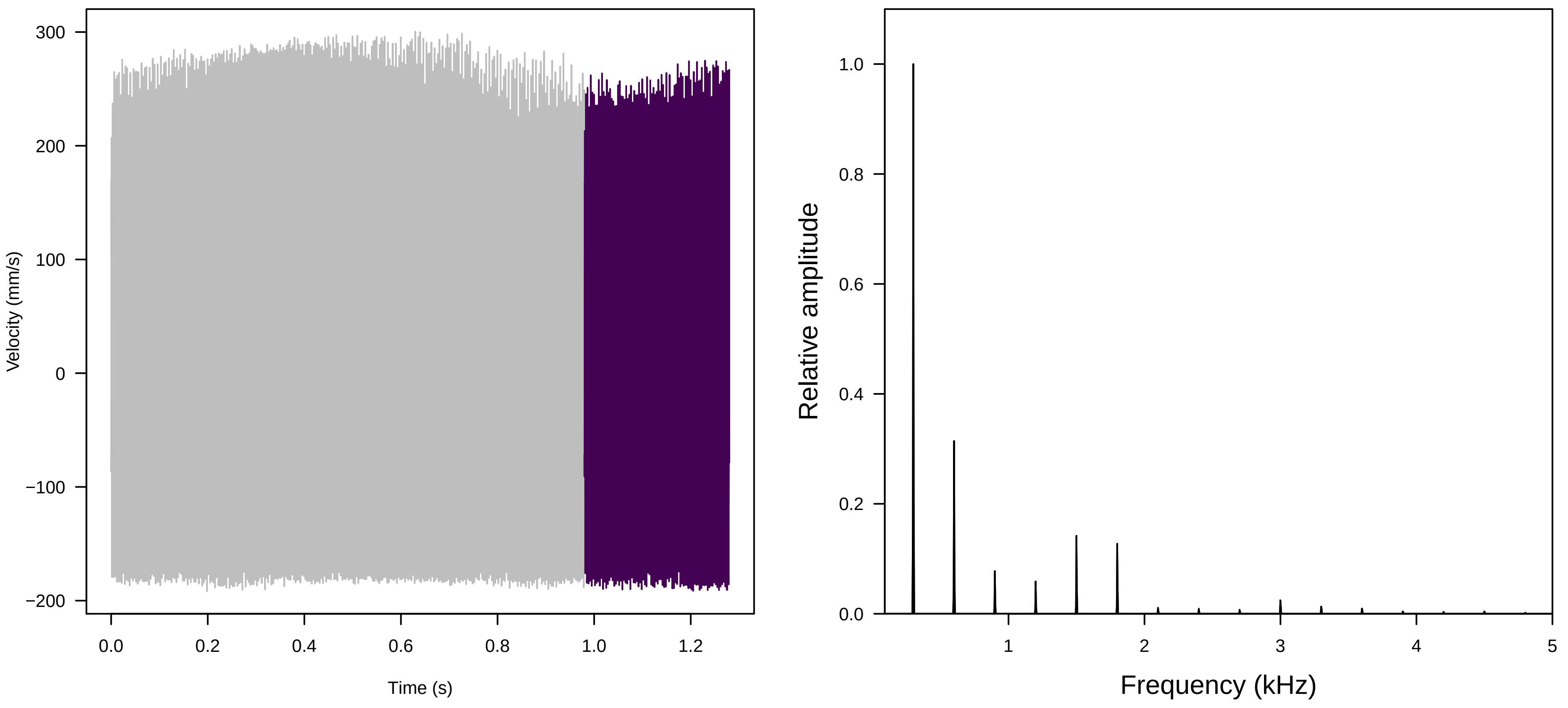

Vel. = 0.057 ; Str. = Receptacle ; Axis = x ; Fl. accession = 10−s−81−1AB

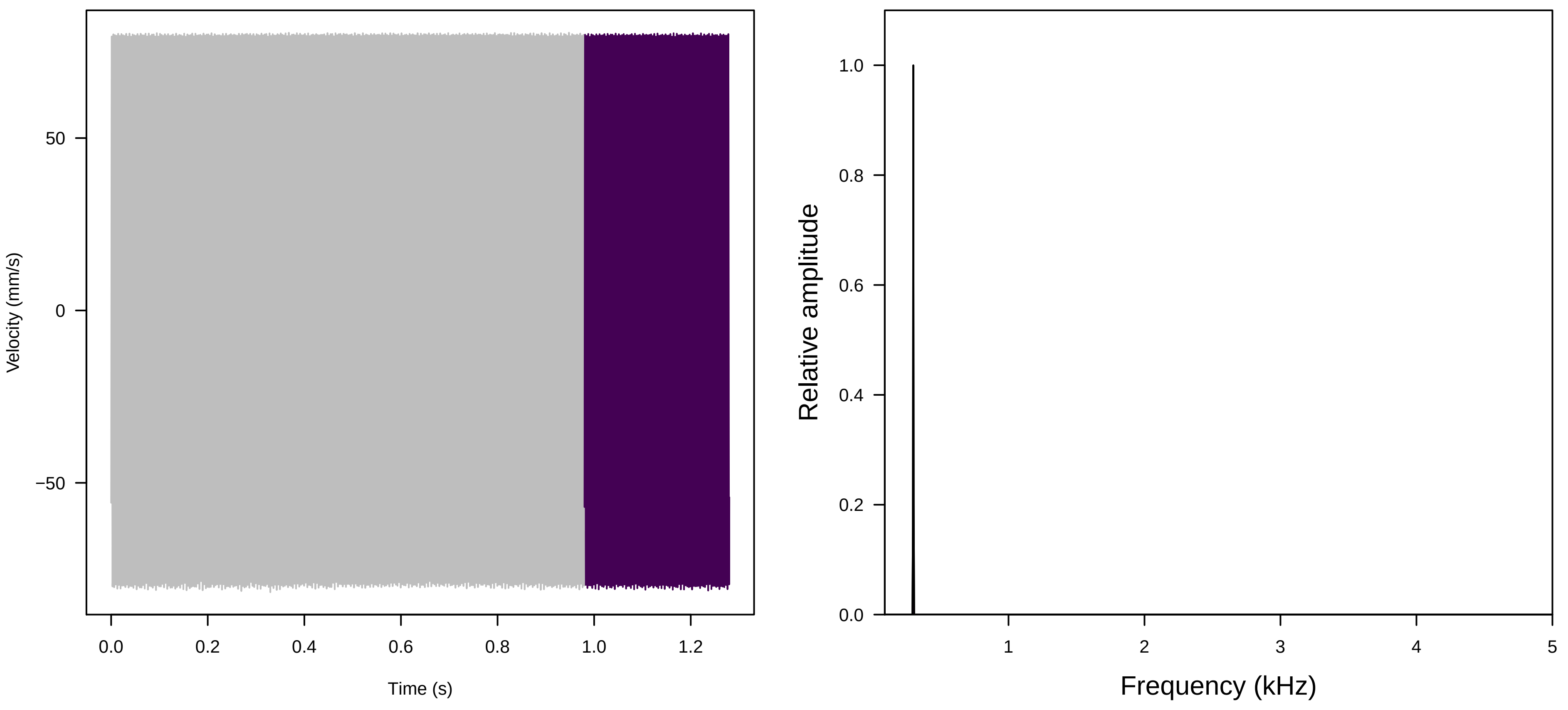

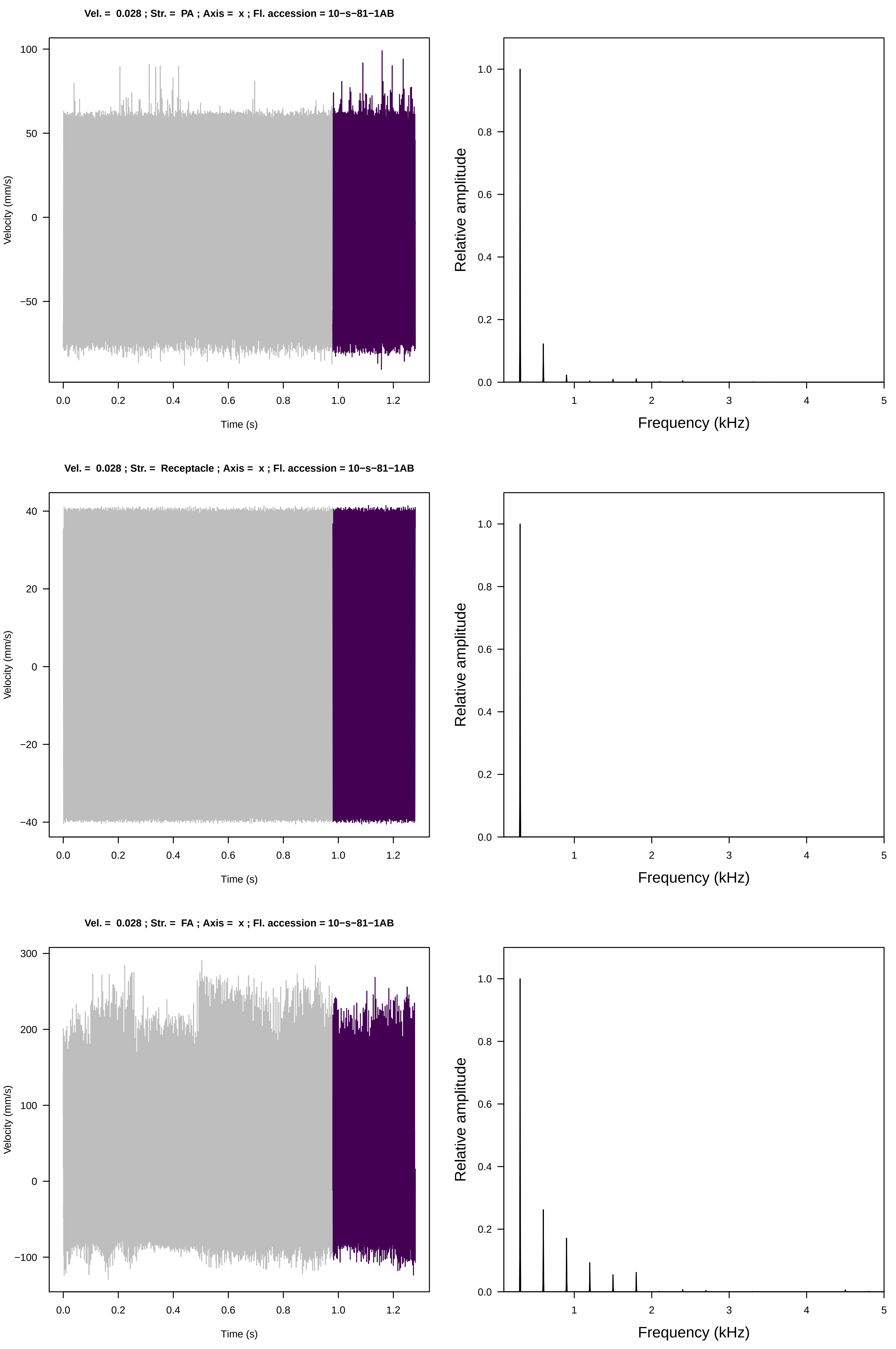

Vel. = 0.028 ; Str. = PA ; Axis = x ; Fl. accession = 10−s−81−1AB
Velocity (mm/s)
Time (s)
Relative amplitude
Frequency (kHz)
Vel. = 0.028 ; Str. = Receptacle ; Axis = x ; Fl. accession = 10−s−81−1AB
Velocity (mm/s)
Time (s)
Relative amplitude
Frequency (kHz)
Vel. = 0.028 ; Str. = FA ; Axis = x ; Fl. accession = 10−s−81−1AB
Velocity (mm/s)
Time (s)
Relative amplitude
Frequency (kHz)

Vel. = 0.028 ; Str. = Receptacle ; Axis = x ; Fl. accession = 10−s−81−1AB

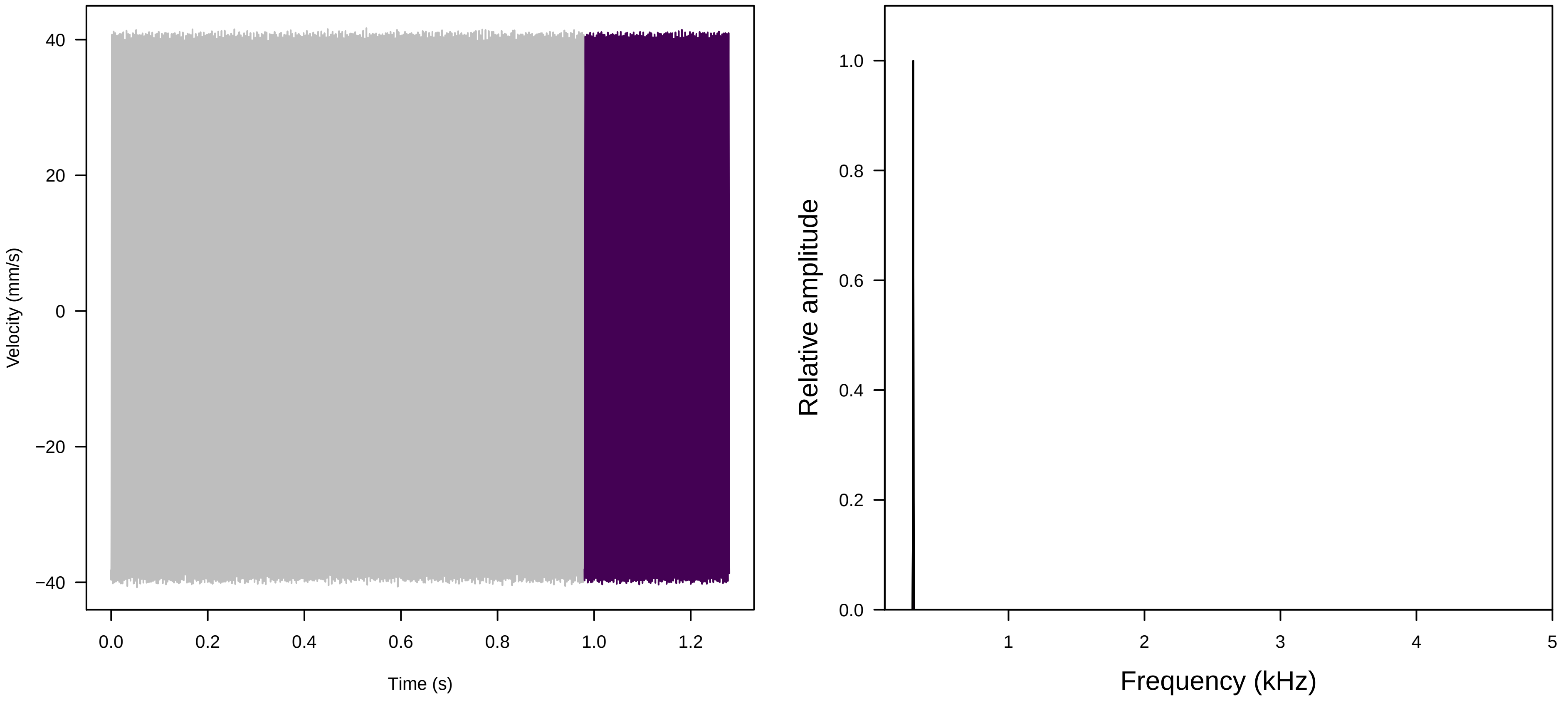

Vel. = 0.028 ; Str. = Corolla ; Axis = x ; Fl. accession = 10−s−81−1AB

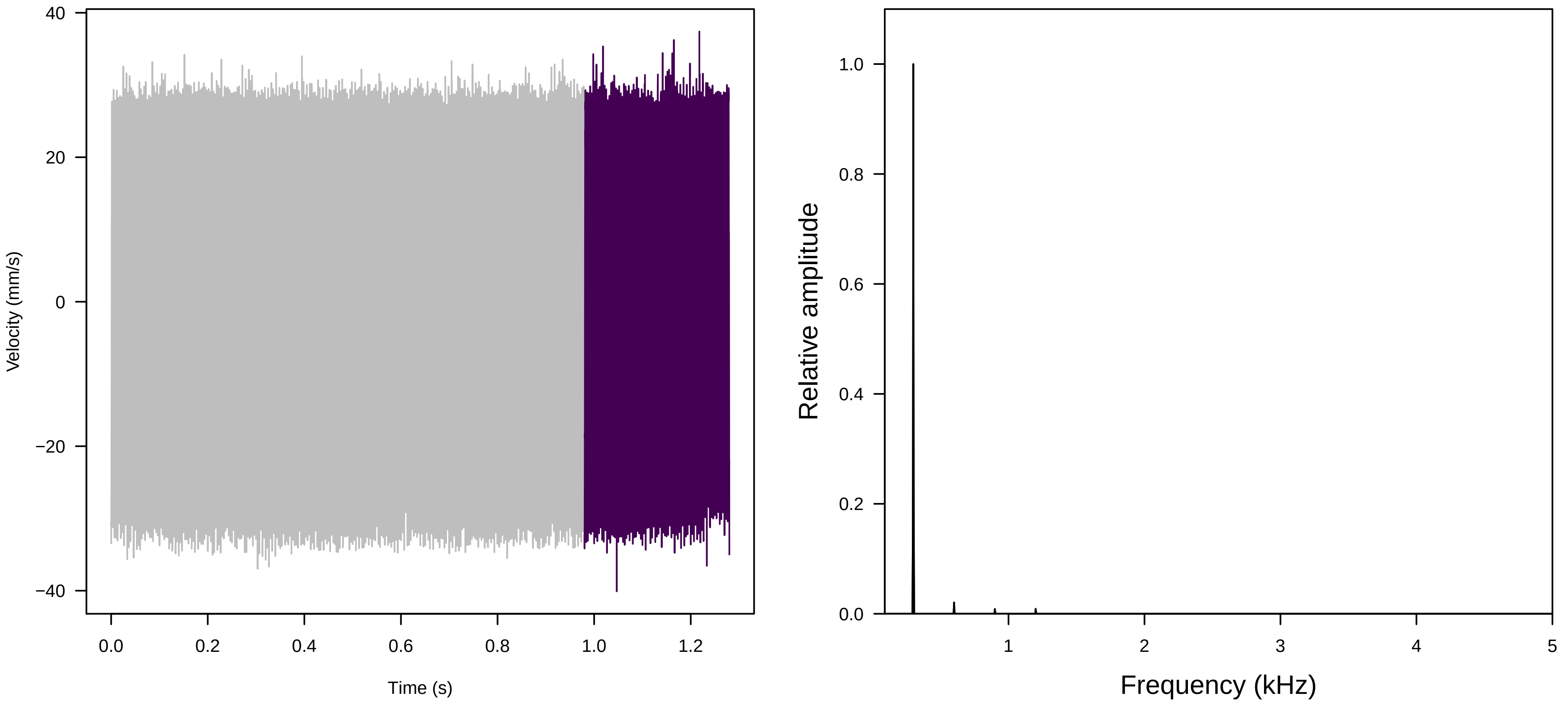

Vel. = 0.028 ; Str. = Receptacle ; Axis = x ; Fl. accession = 10−s−81−1AB

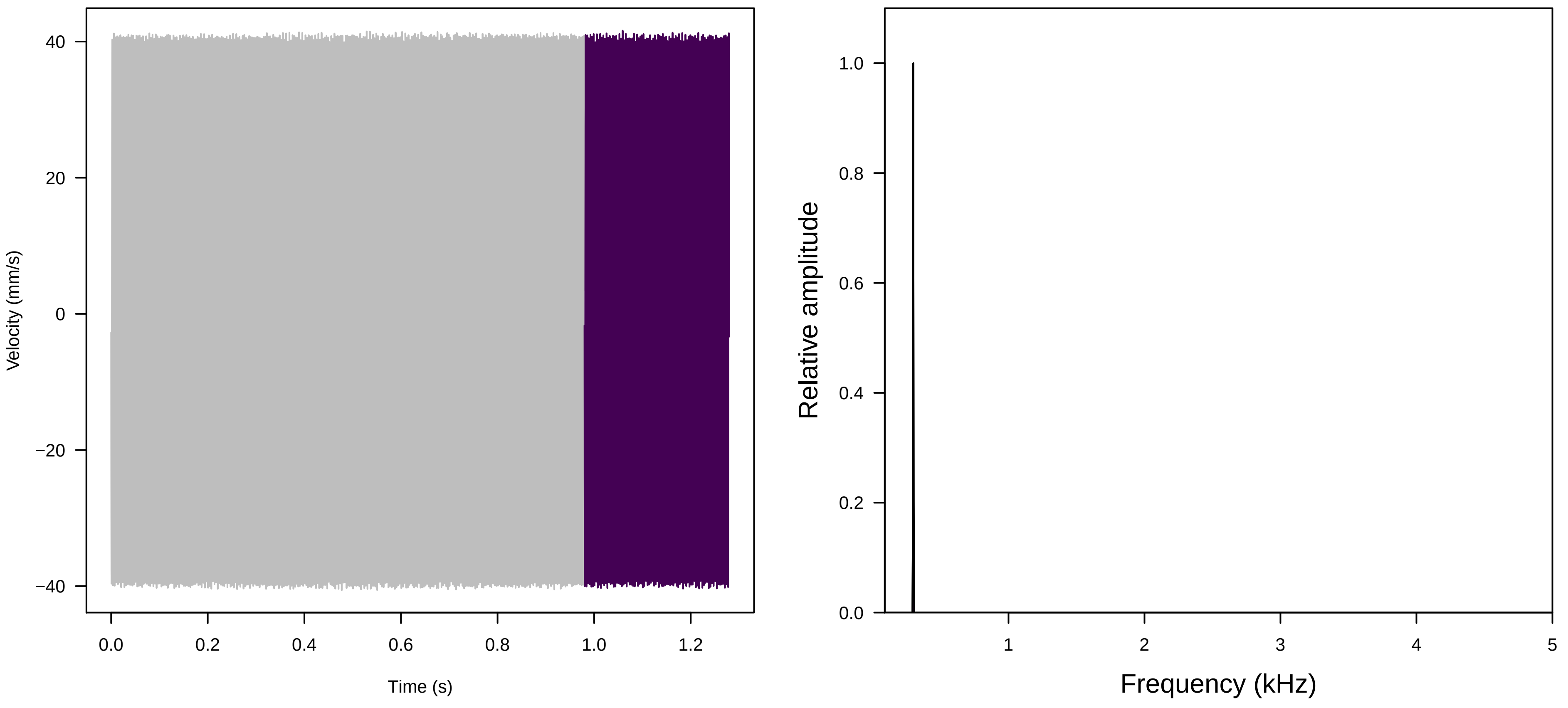

Vel. = 0.014 ; Str. = Corolla ; Axis = x ; Fl. accession = 10−s−81−1AB

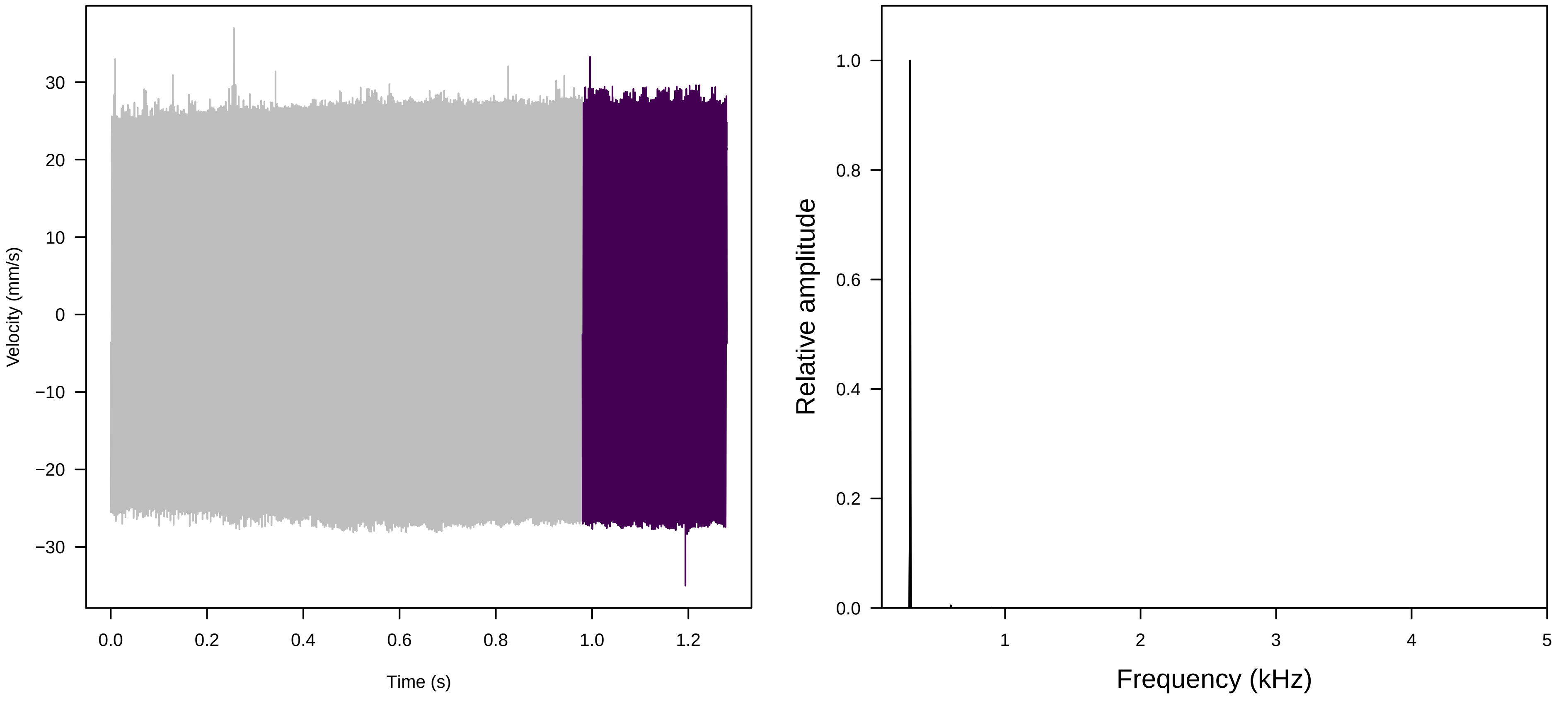

Vel. = 0.014 ; Str. = Receptacle ; Axis = x ; Fl. accession = 10−s−81−1AB

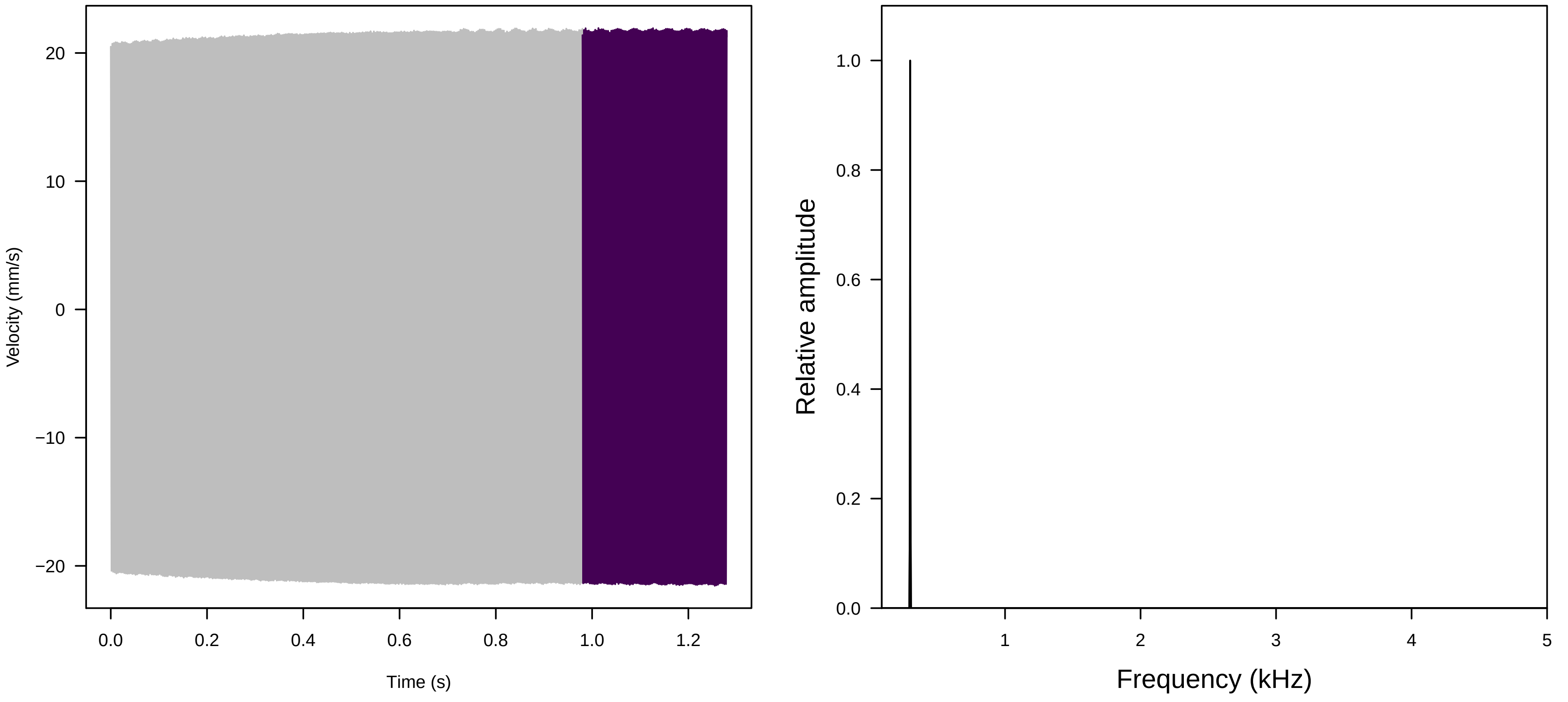

Vel. = 0.014 ; Str. = FA ; Axis = x ; Fl. accession = 10−s−81−1AB

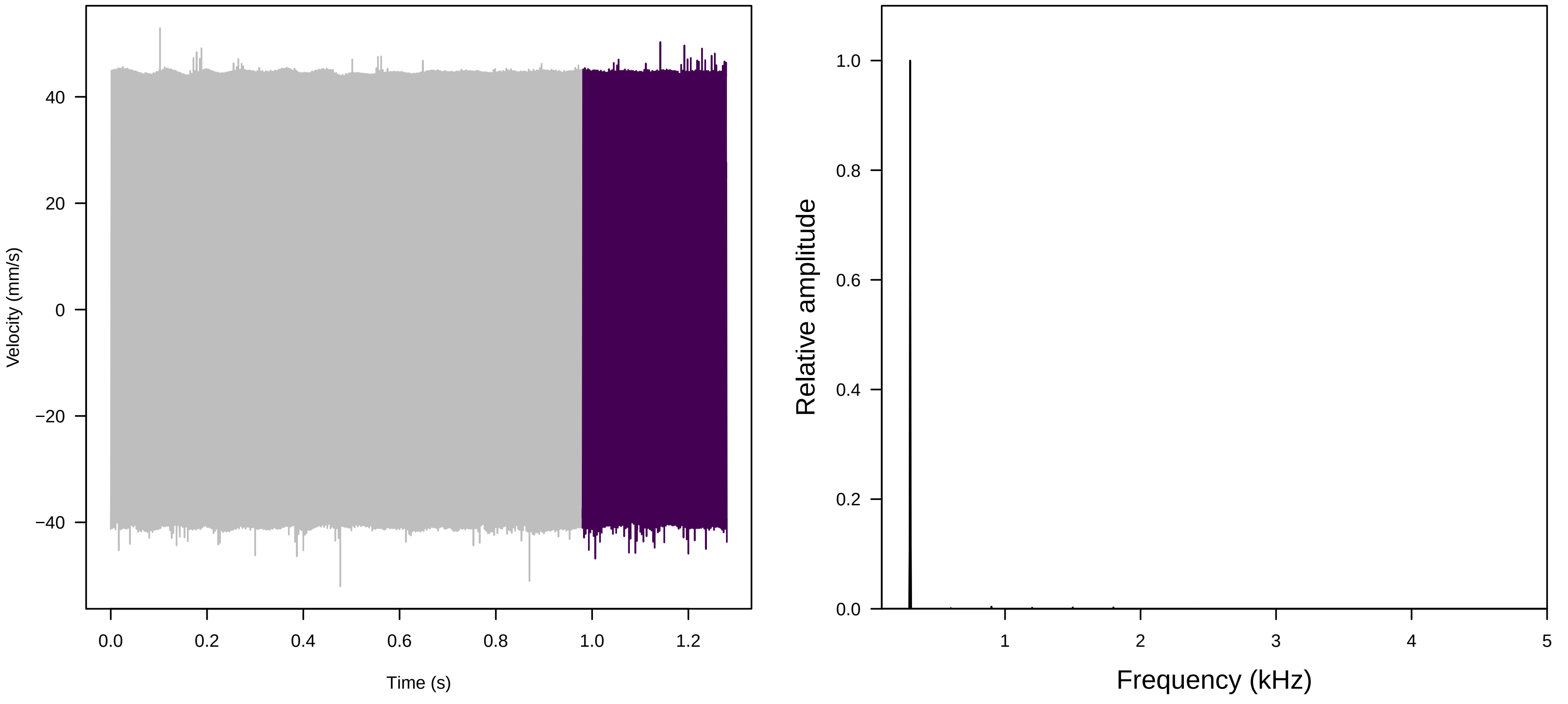

Vel. = 0.014 ; Str. = Receptacle ; Axis = x ; Fl. accession = 10−s−81−1AB

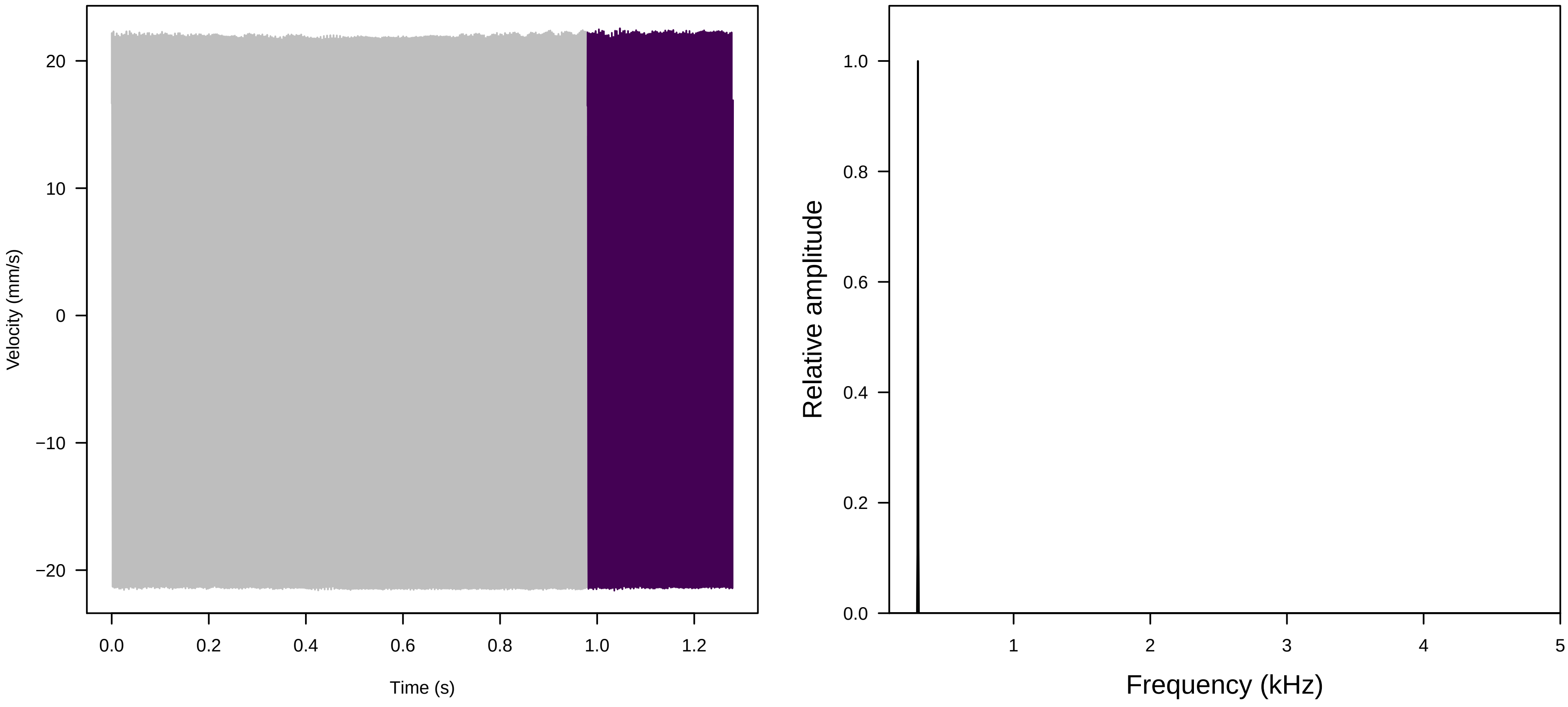

Vel. = 0.014 ; Str. = PA ; Axis = x ; Fl. accession = 10−s−81−1AB

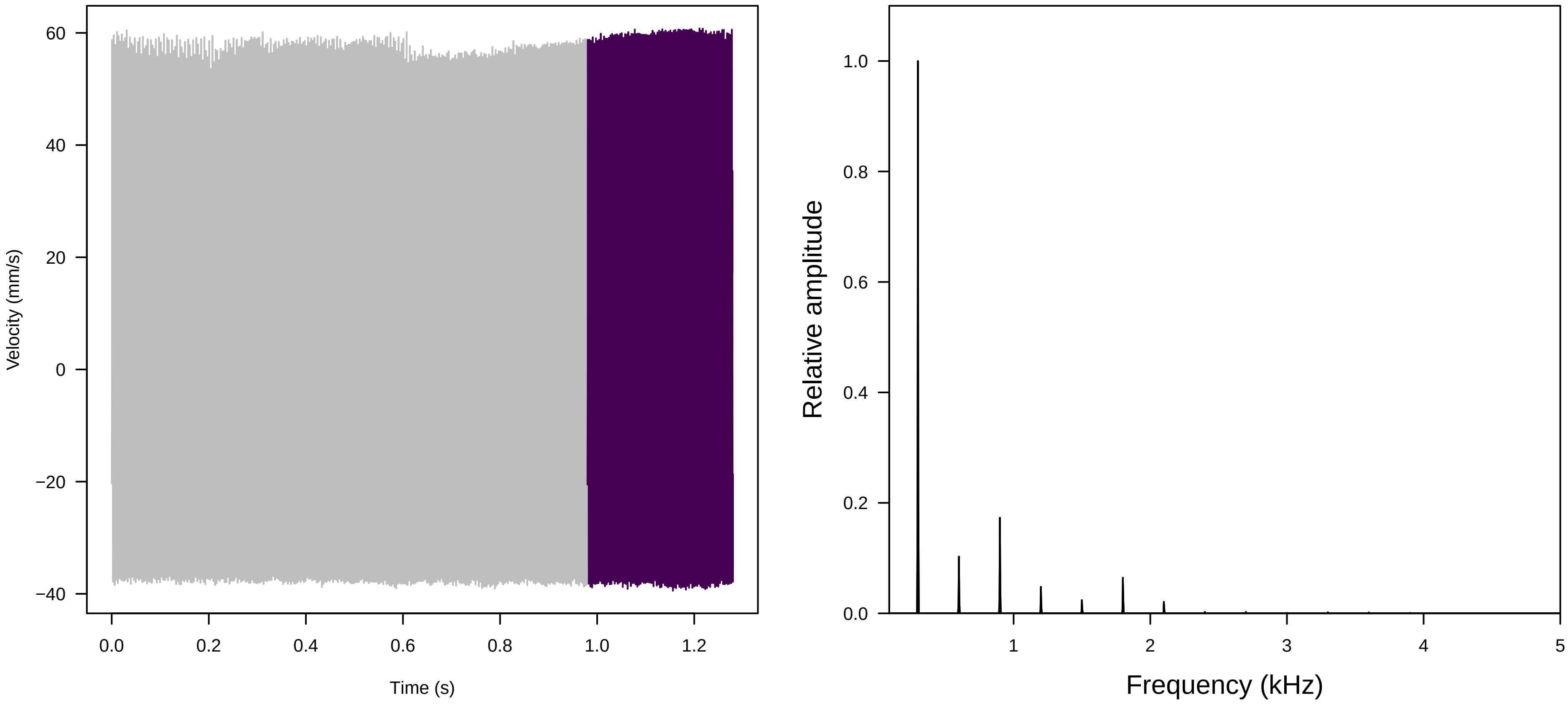

Vel. = 0.014 ; Str. = Receptacle ; Axis = x ; Fl. accession = 10−s−81−1AB

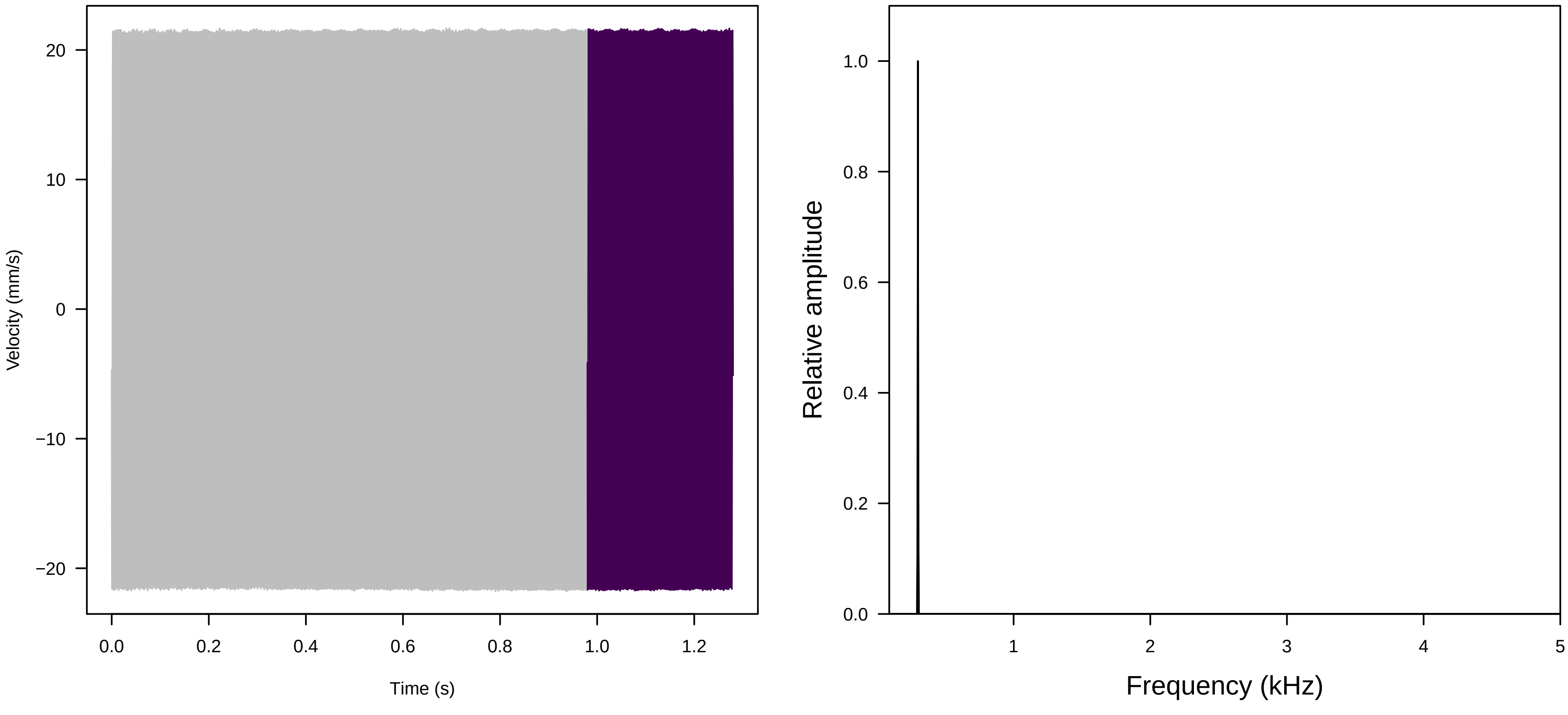

Vel. = 0.014 ; Str. = Corolla ; Axis = x ; Fl. accession = 10−s−81−8

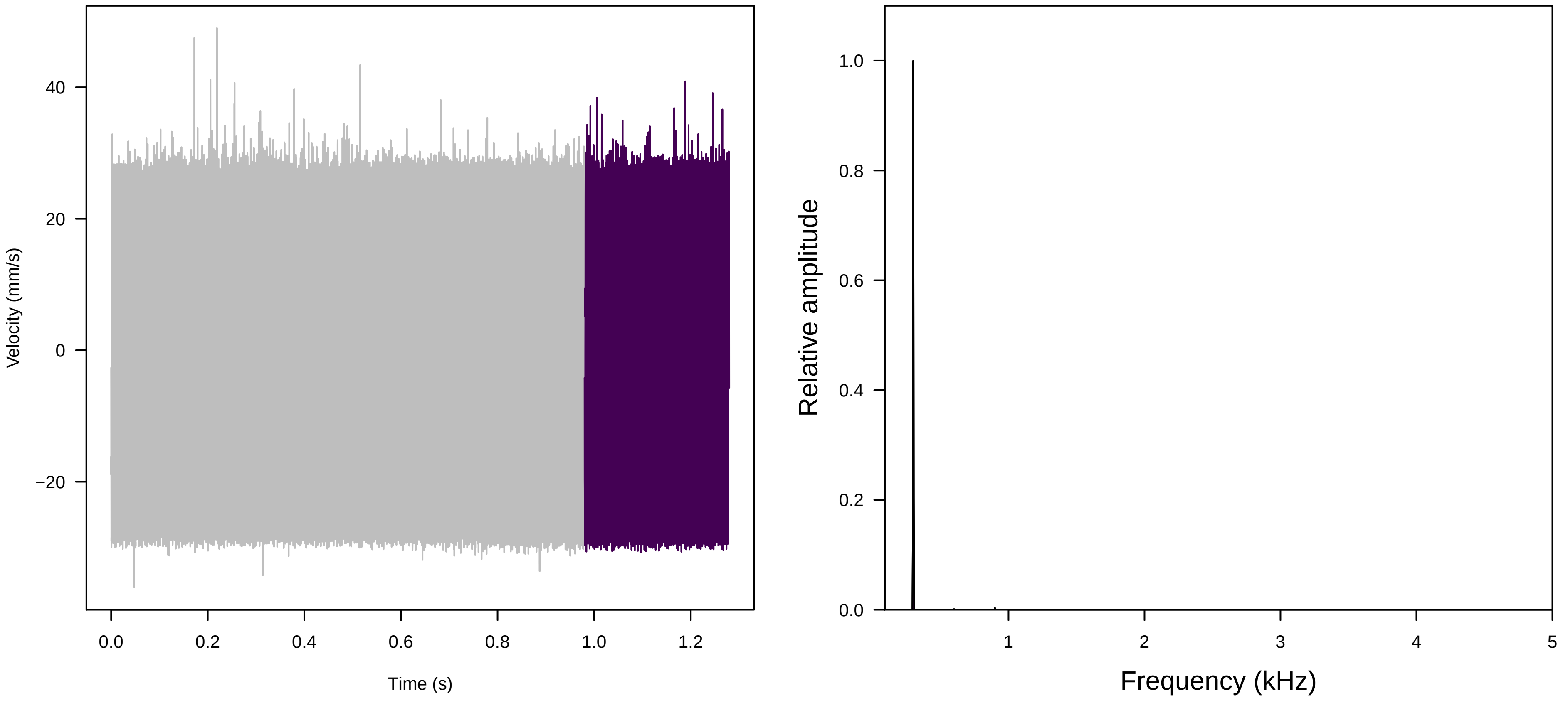

Vel. = 0.014 ; Str. = Receptacle ; Axis = x ; Fl. accession = 10−s−81−8

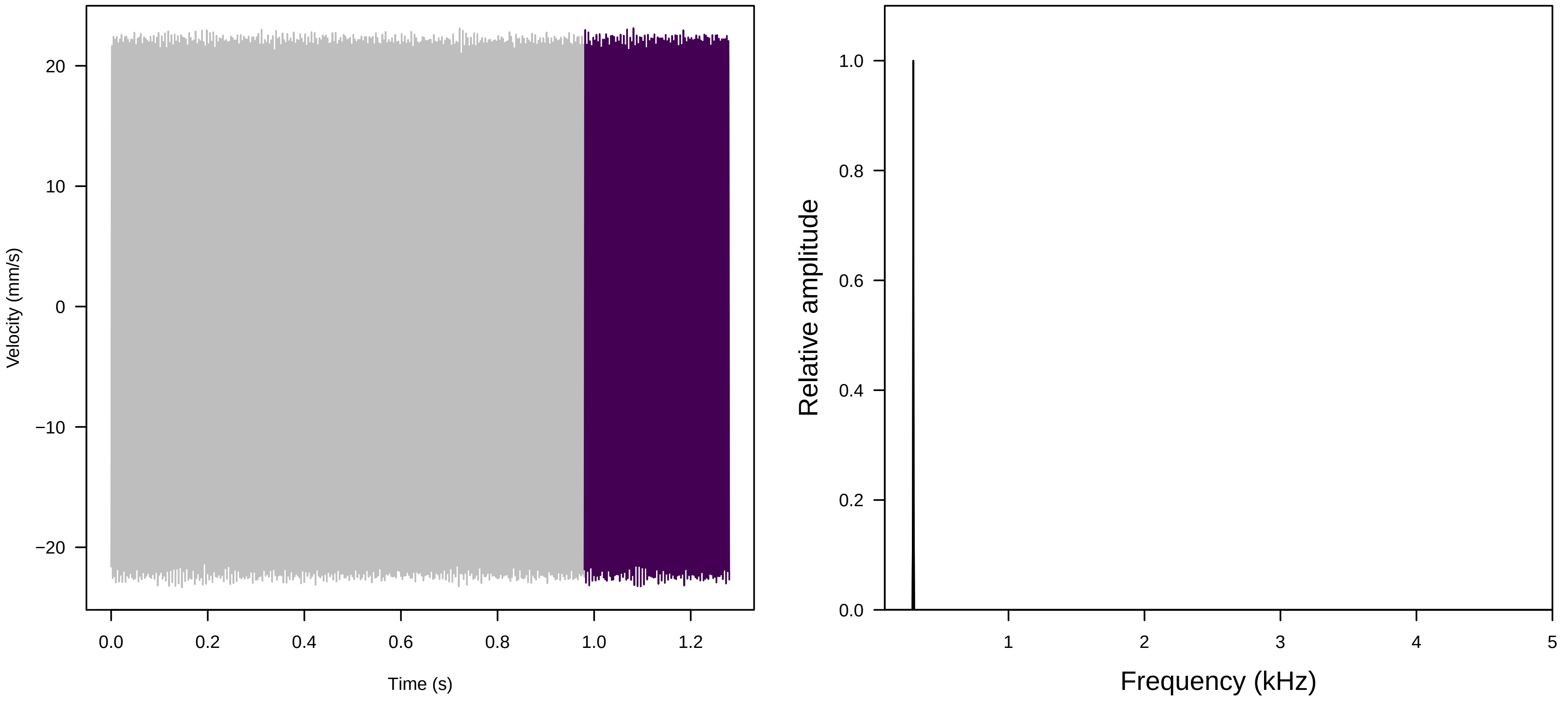

Vel. = 0.014 ; Str. = FA ; Axis = x ; Fl. accession = 10−s−81−8

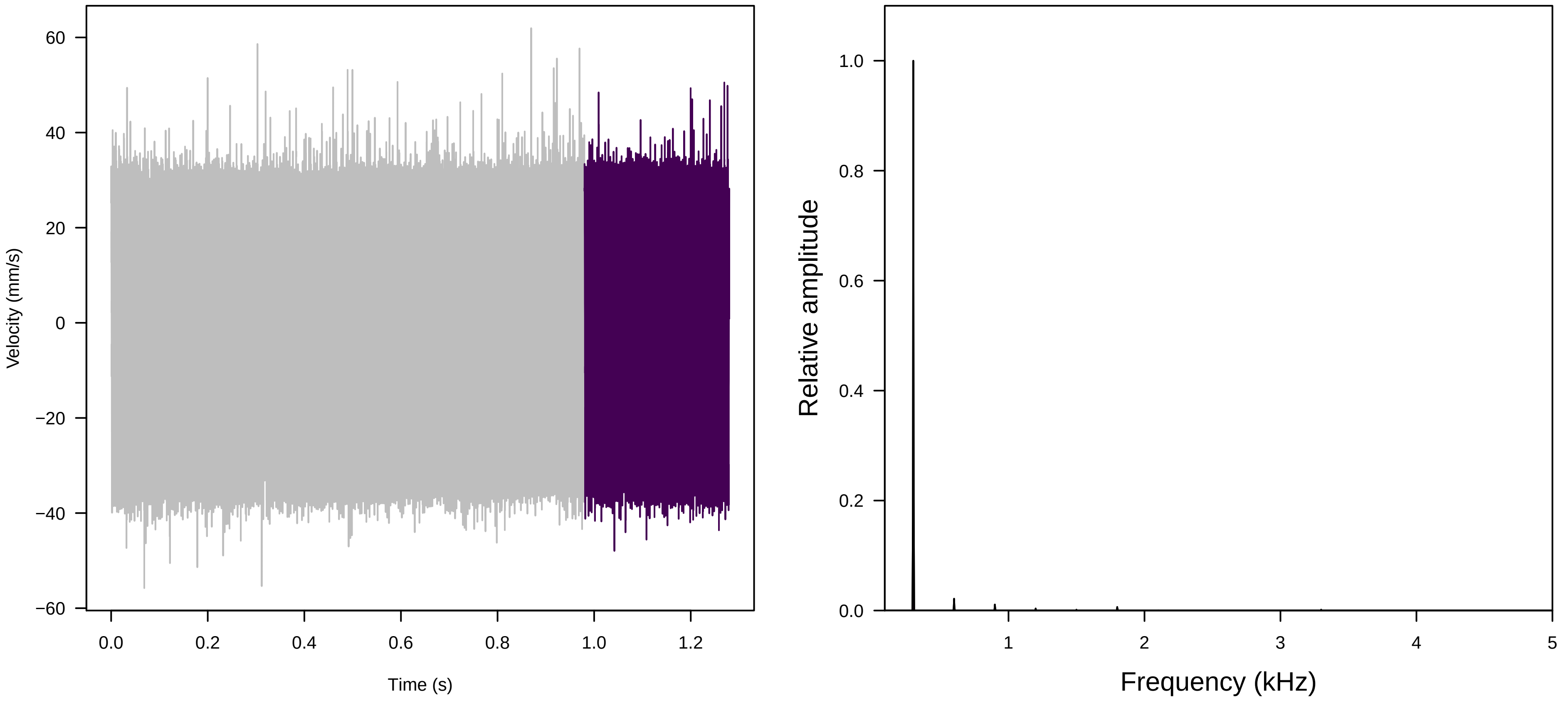

Vel. = 0.014 ; Str. = Receptacle ; Axis = x ; Fl. accession = 10−s−81−8

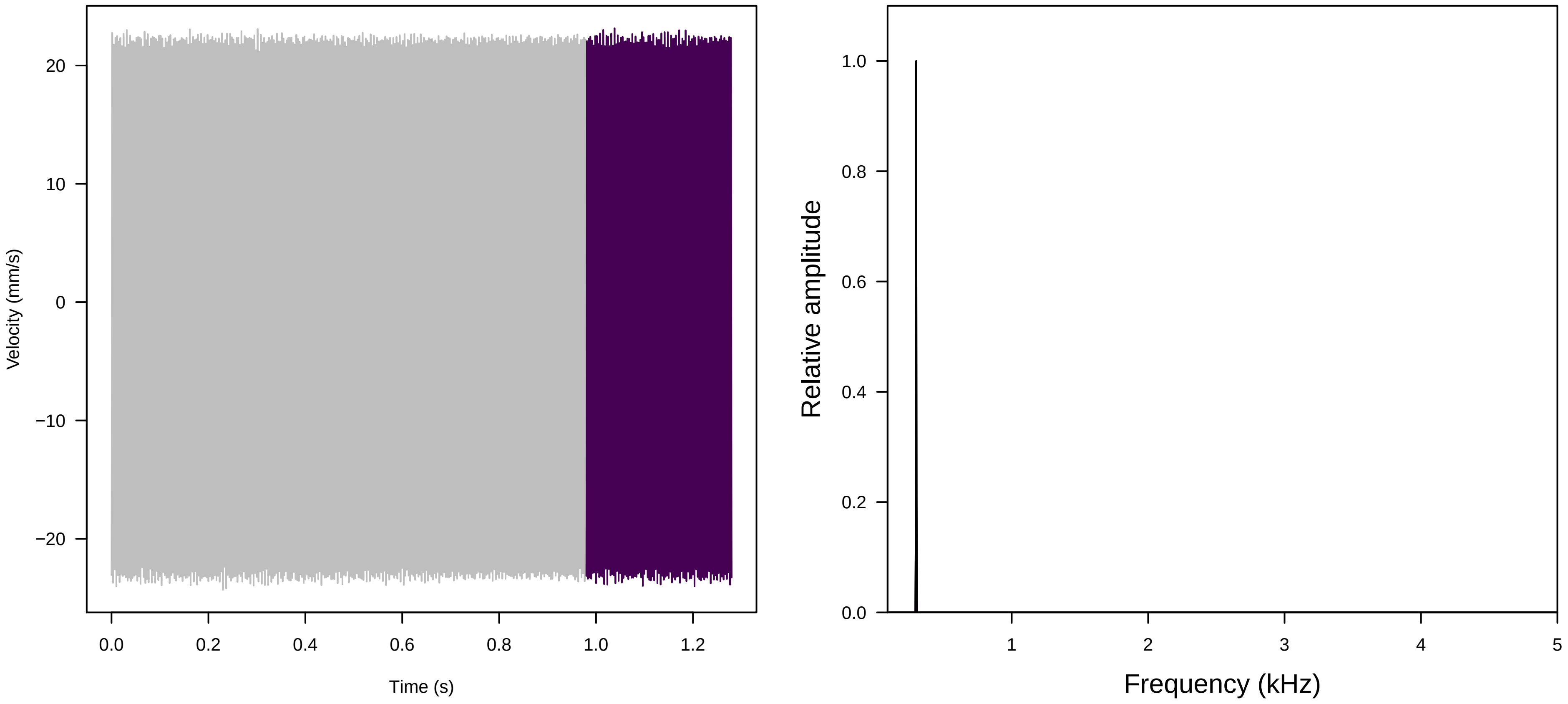

Vel. = 0.014 ; Str. = PA ; Axis = x ; Fl. accession = 10−s−81−8

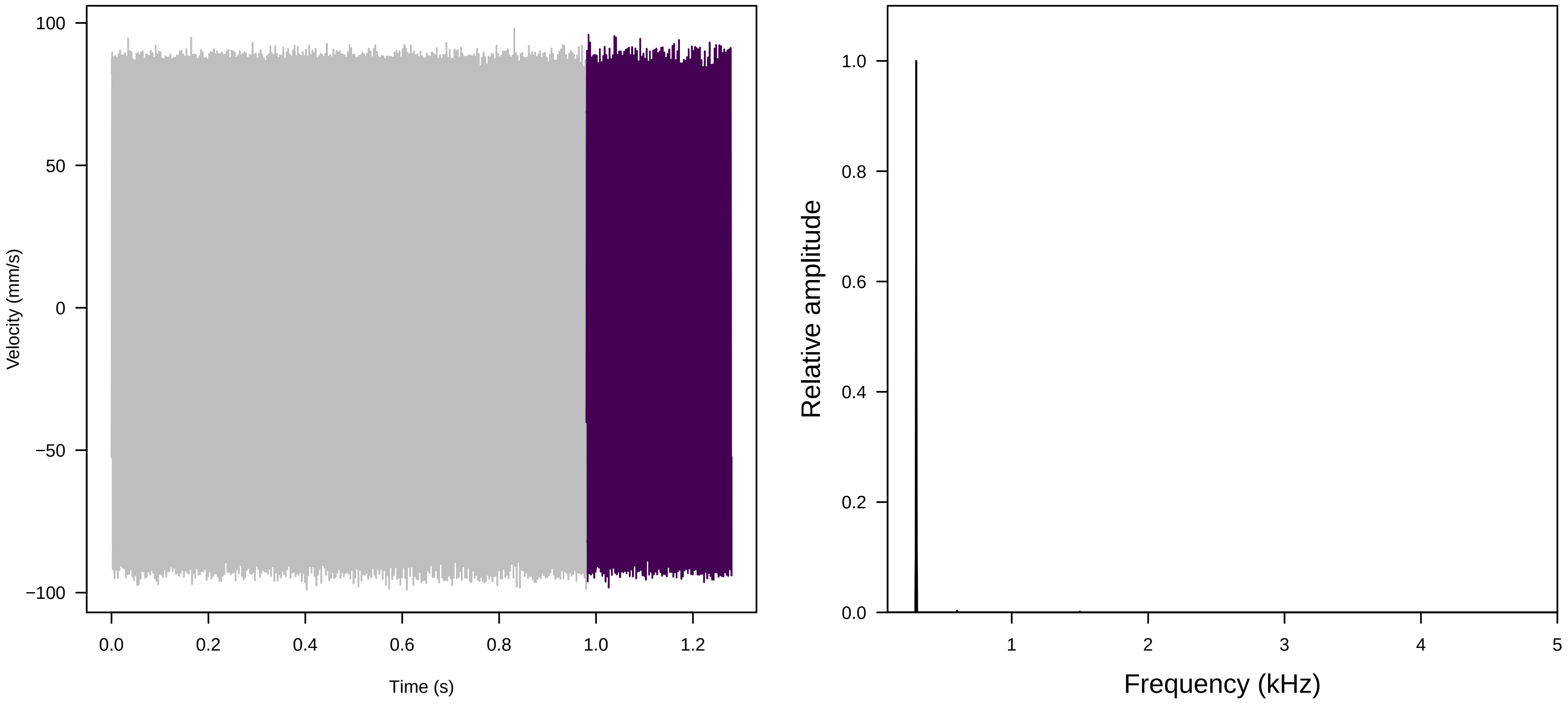

Vel. = 0.014 ; Str. = Receptacle ; Axis = x ; Fl. accession = 10−s−81−8

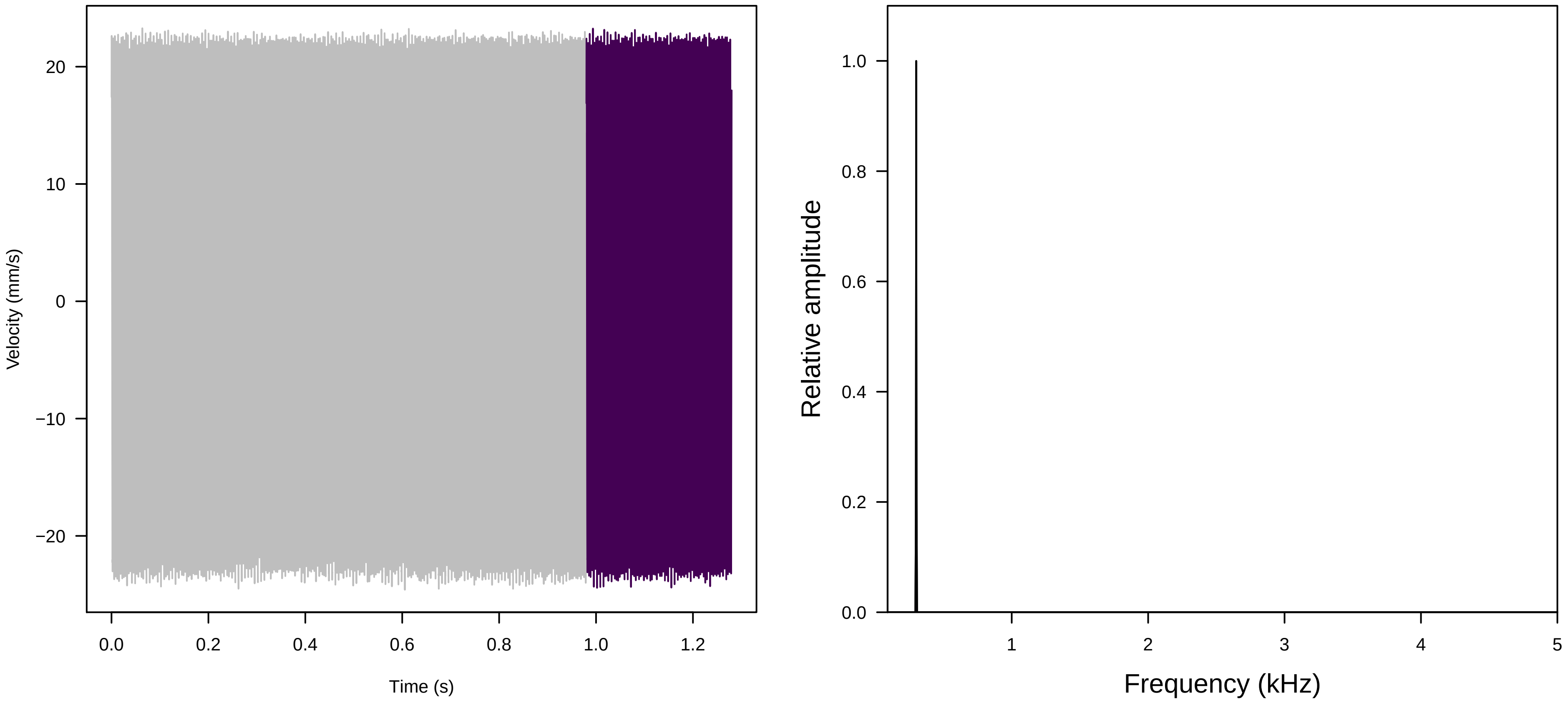

Vel. = 0.028 ; Str. = PA ; Axis = x ; Fl. accession = 10−s−81−8

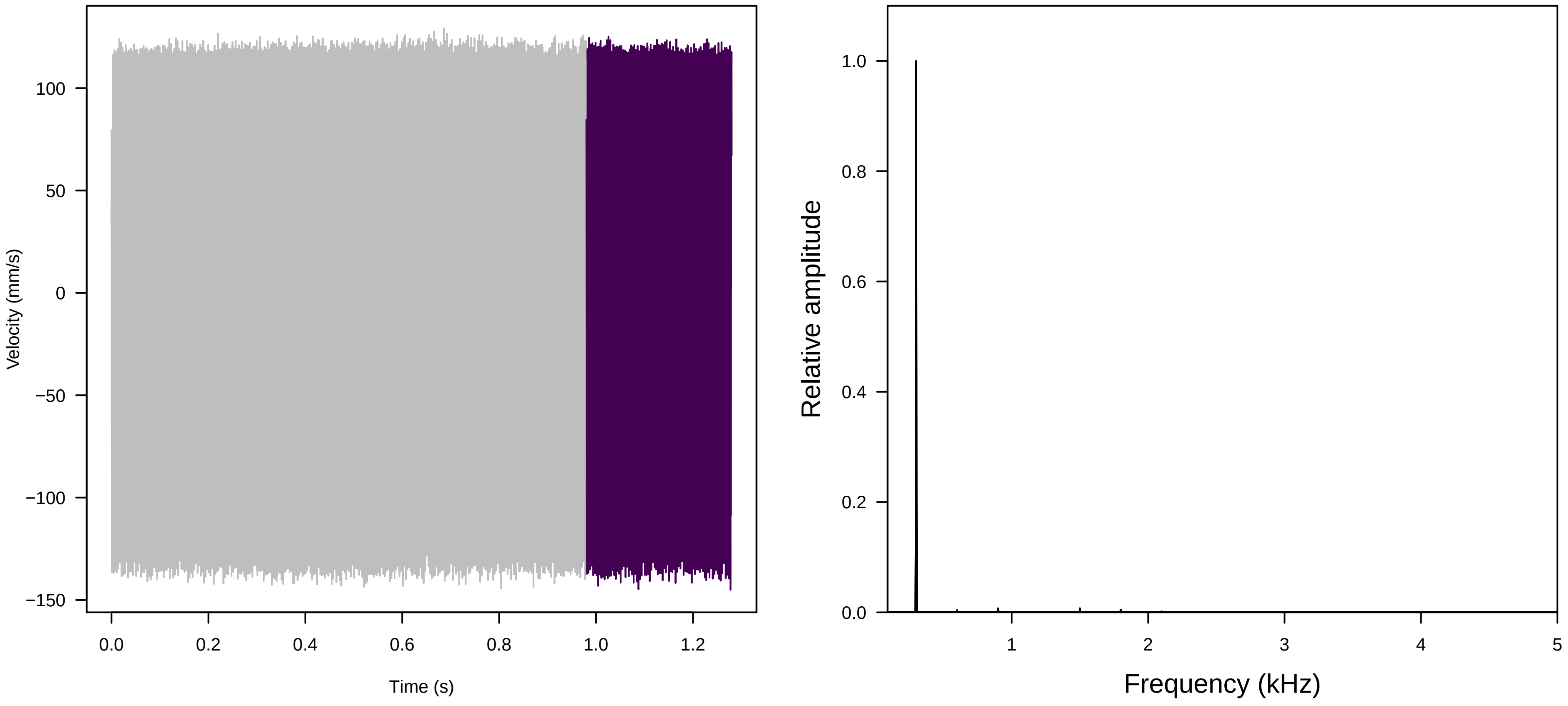

Vel. = 0.028 ; Str. = Receptacle ; Axis = x ; Fl. accession = 10−s−81−8

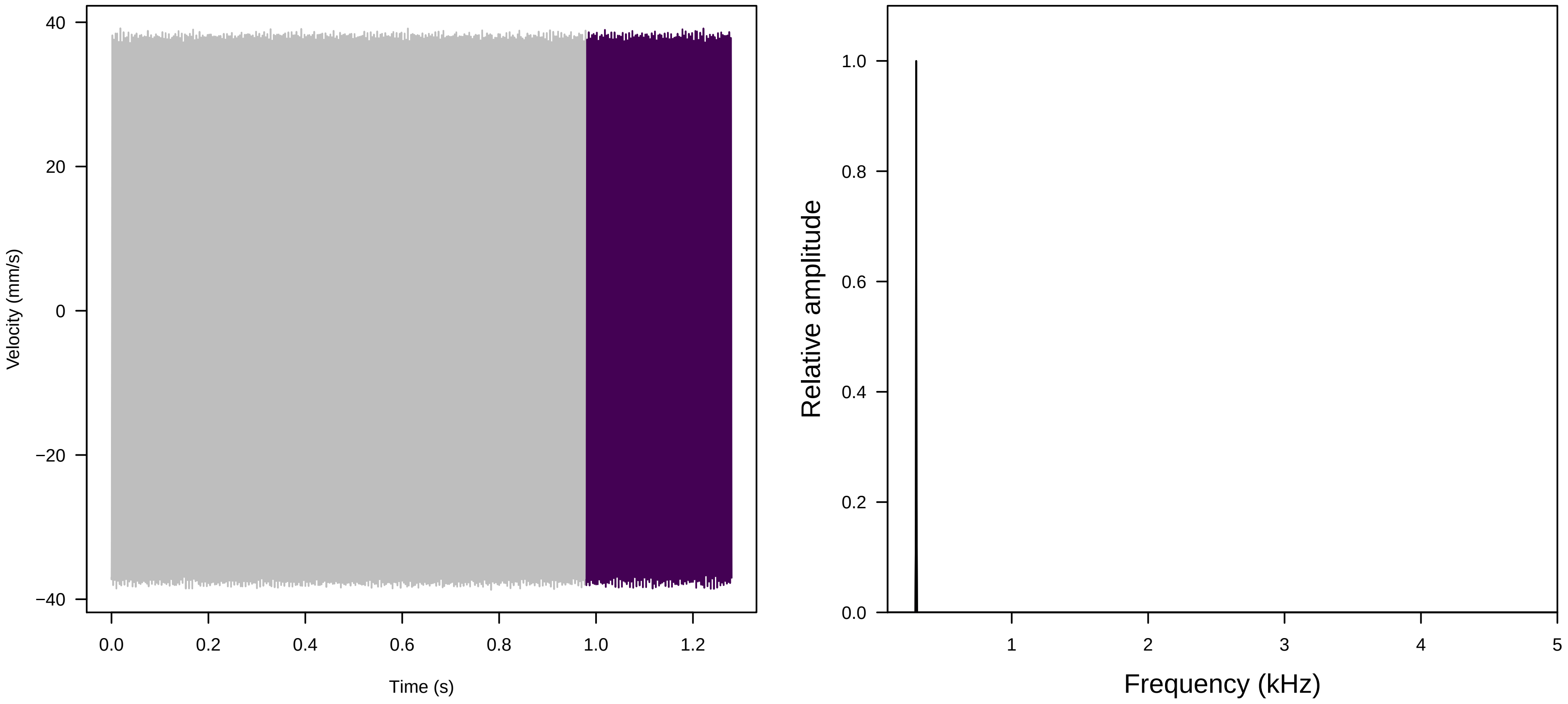

Vel. = 0.028 ; Str. = FA ; Axis = x ; Fl. accession = 10−s−81−8

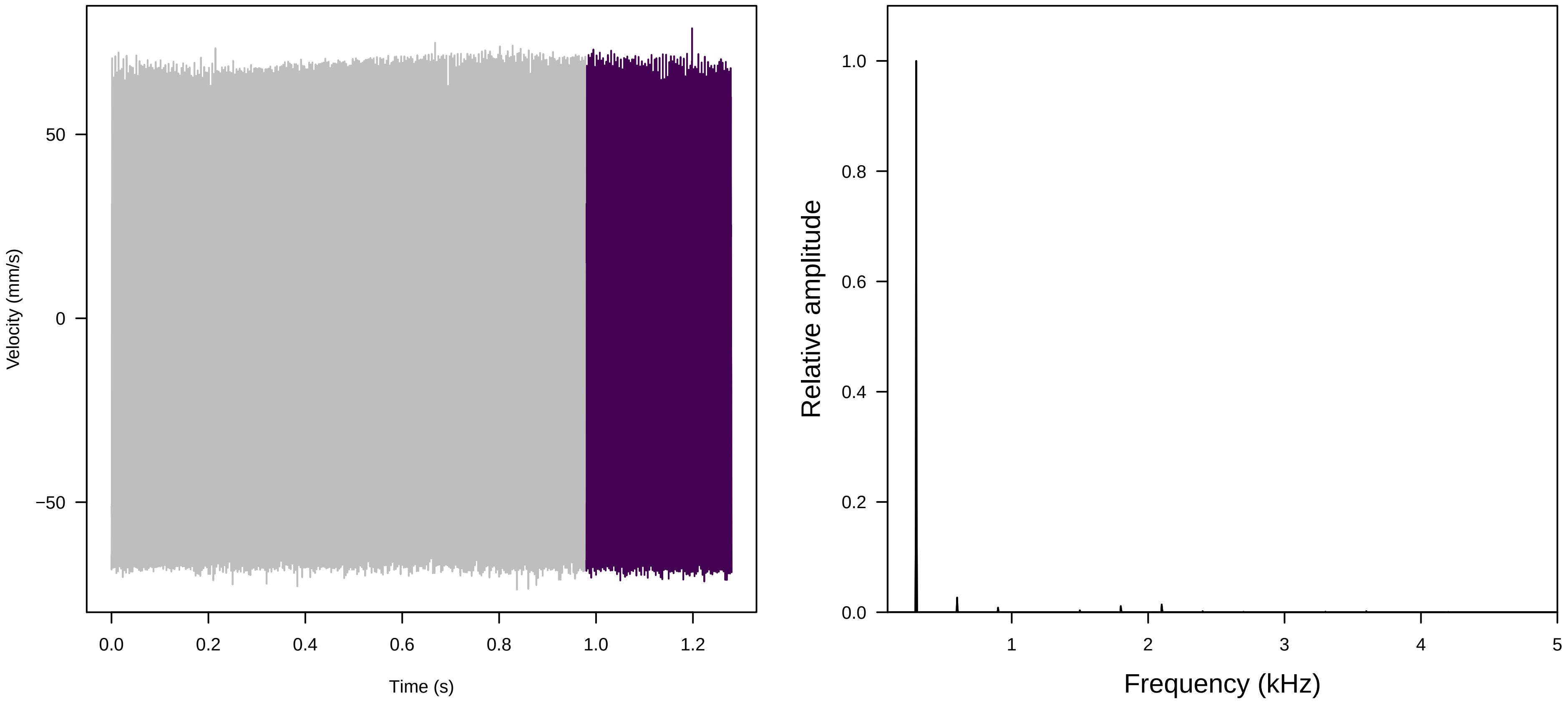

Vel. = 0.028 ; Str. = Receptacle ; Axis = x ; Fl. accession = 10−s−81−8

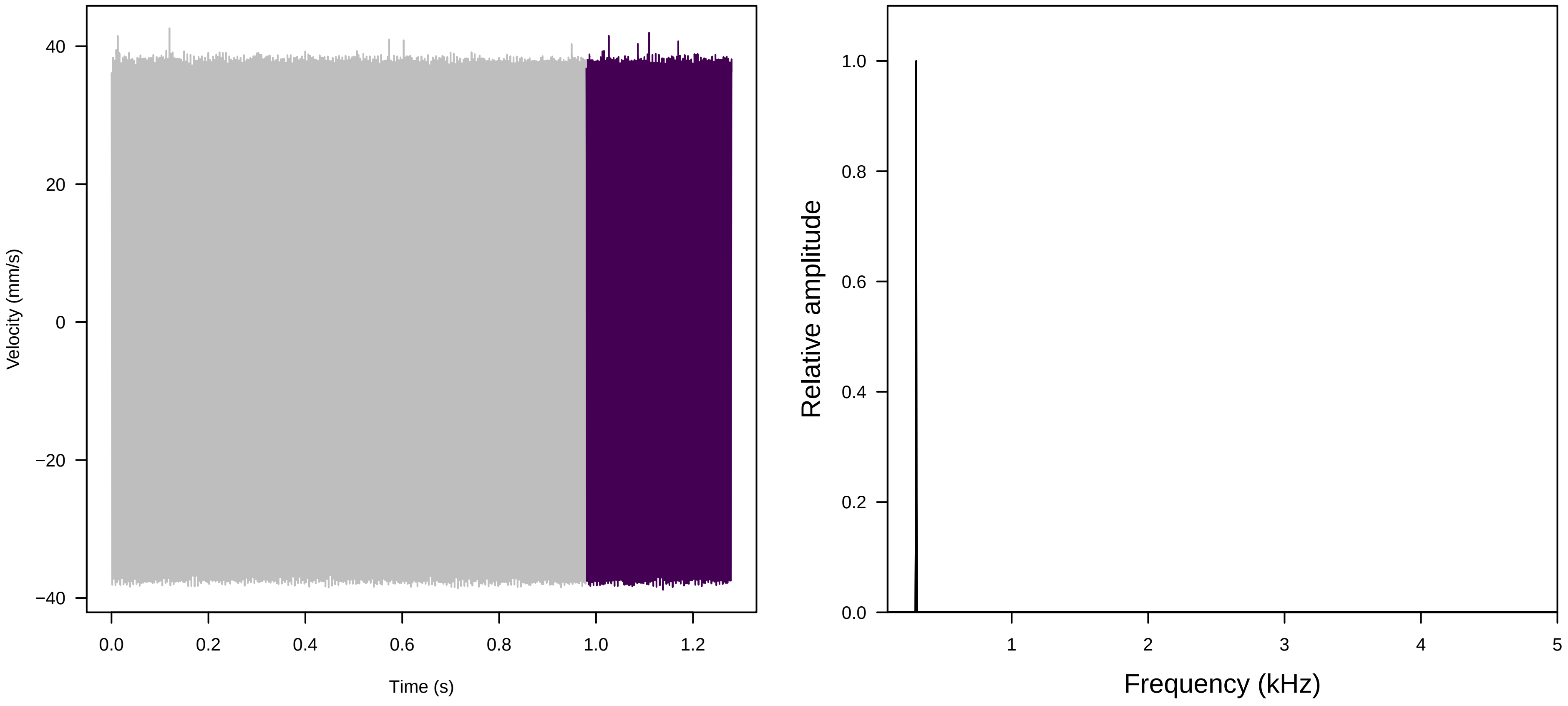

Vel. = 0.028 ; Str. = Corolla ; Axis = x ; Fl. accession = 10−s−81−8

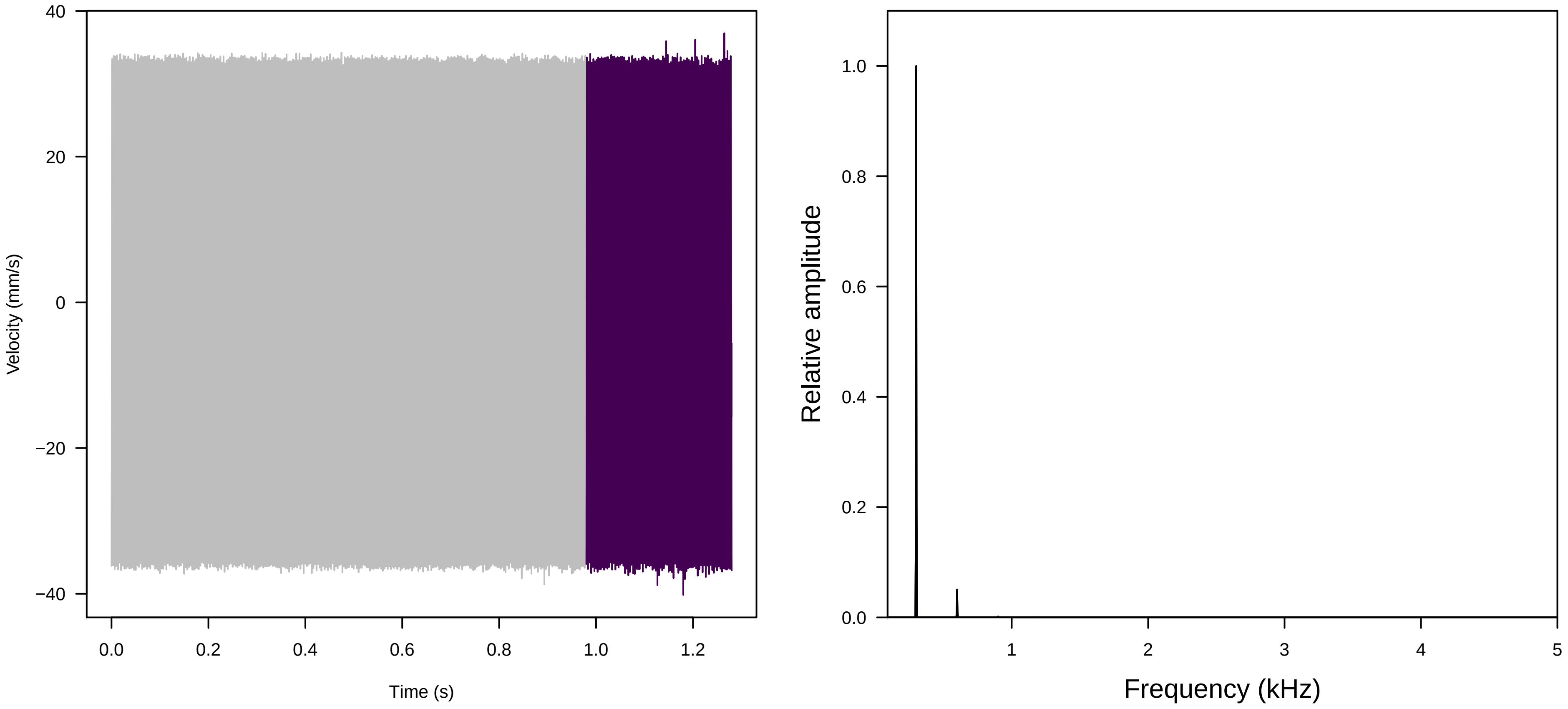

Vel. = 0.028 ; Str. = Receptacle ; Axis = x ; Fl. accession = 10−s−81−8

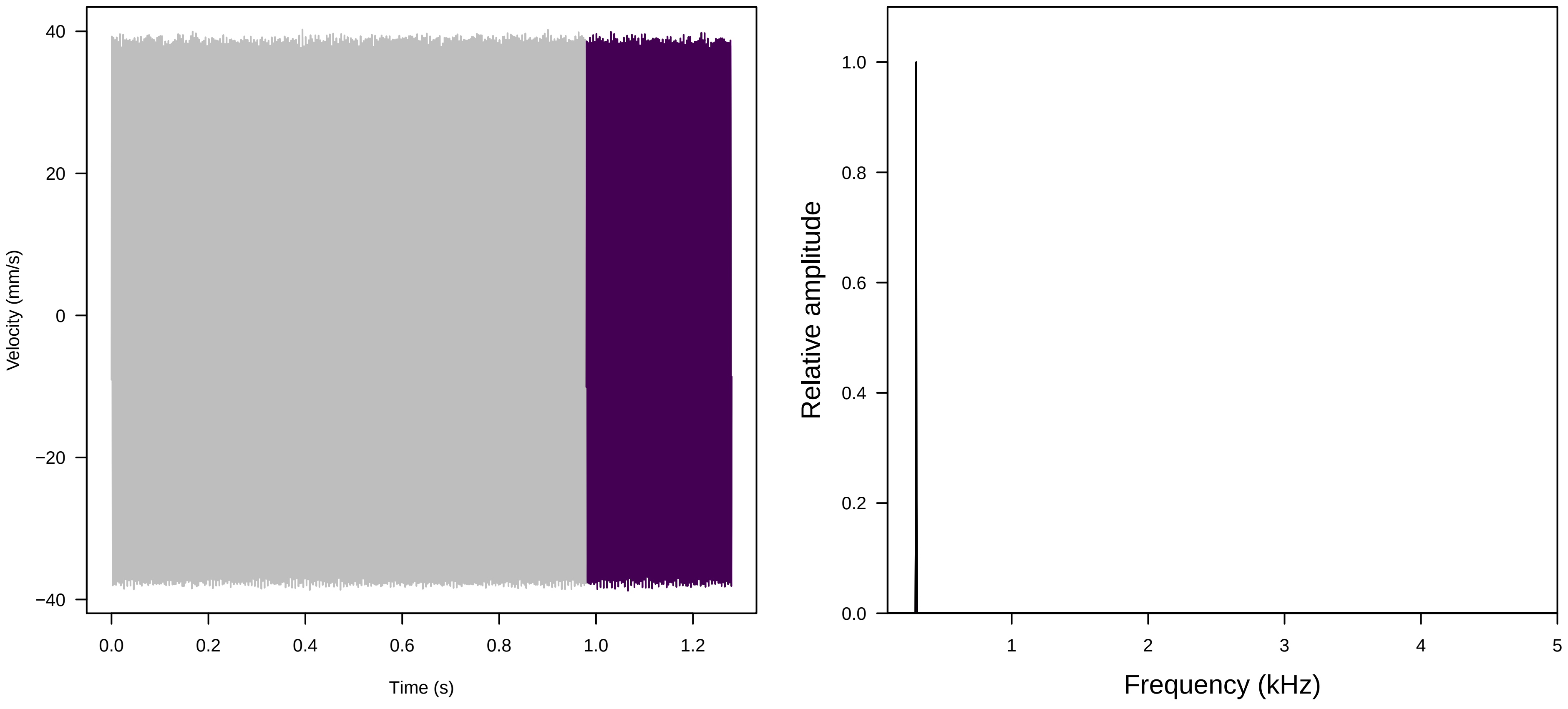

Vel. = 0.057 ; Str. = Corolla ; Axis = x ; Fl. accession = 10−s−81−8

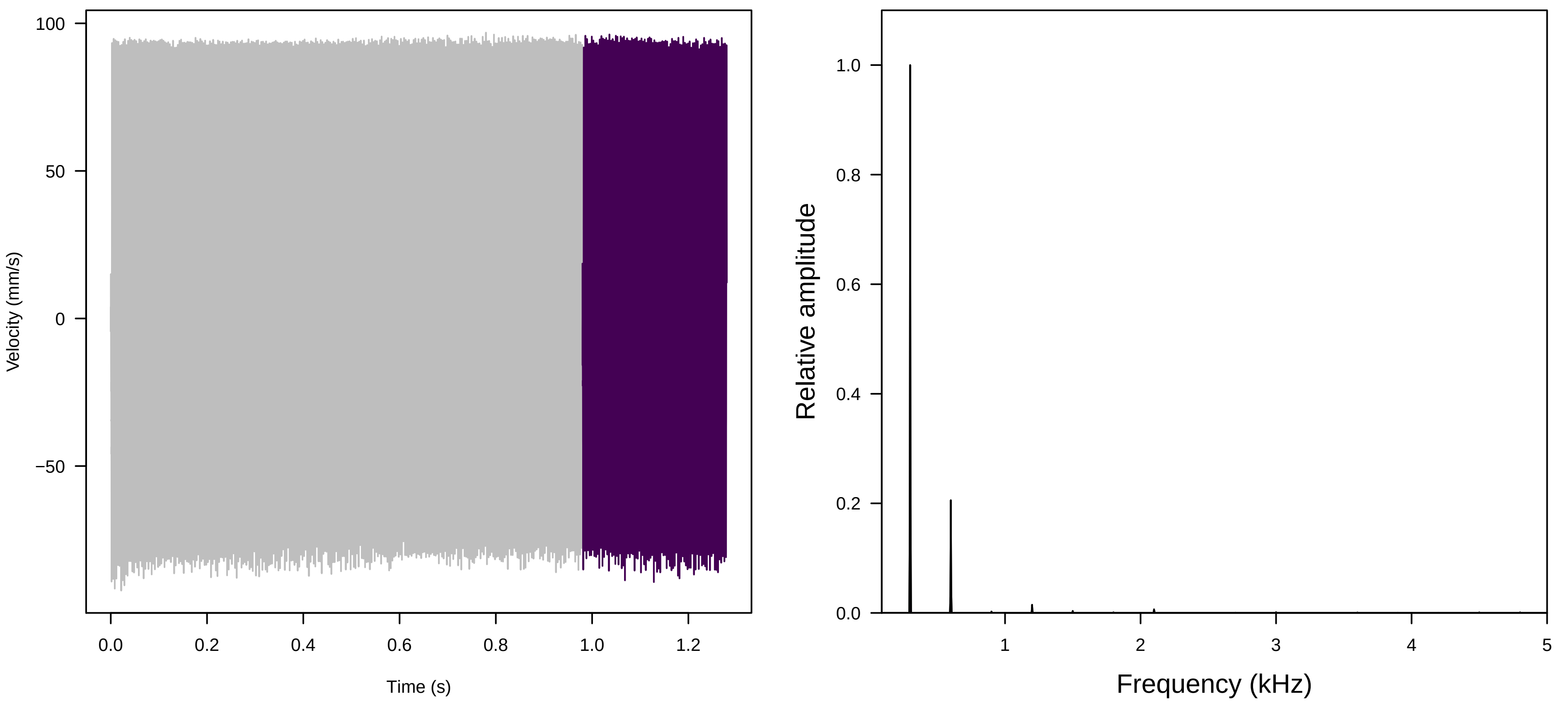

Vel. = 0.057 ; Str. = Receptacle ; Axis = x ; Fl. accession = 10−s−81−8

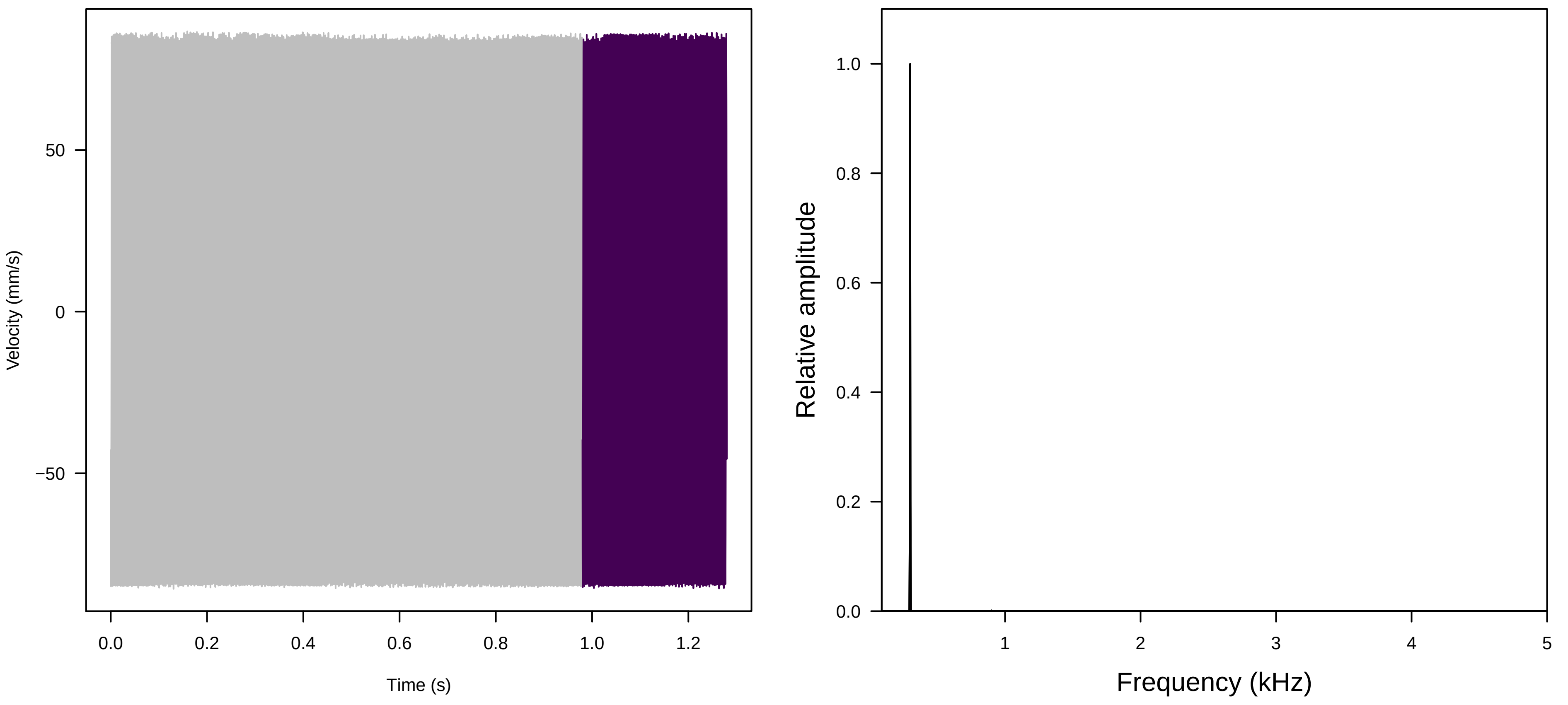

Vel. = 0.057 ; Str. = FA ; Axis = x ; Fl. accession = 10−s−81−8

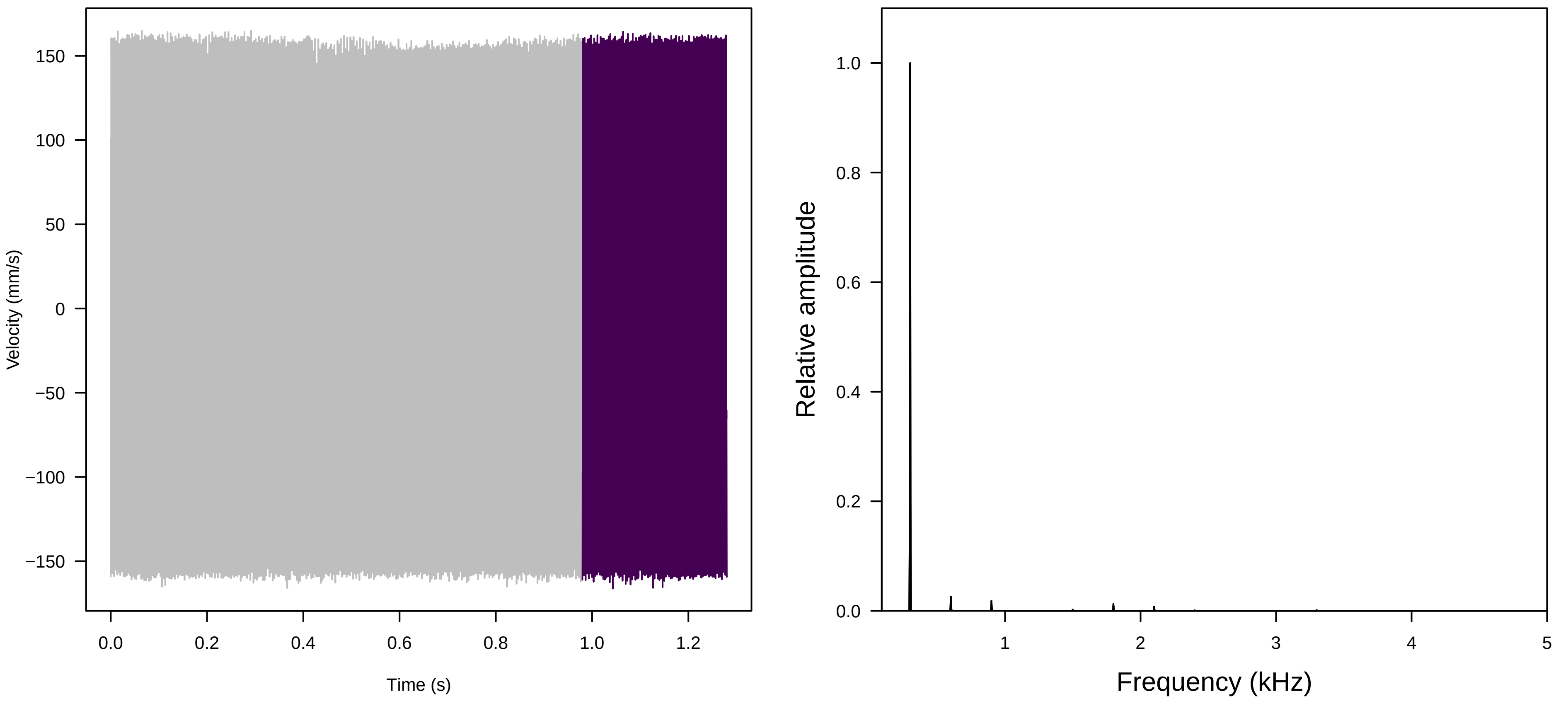

Vel. = 0.057 ; Str. = Receptacle ; Axis = x ; Fl. accession = 10−s−81−8

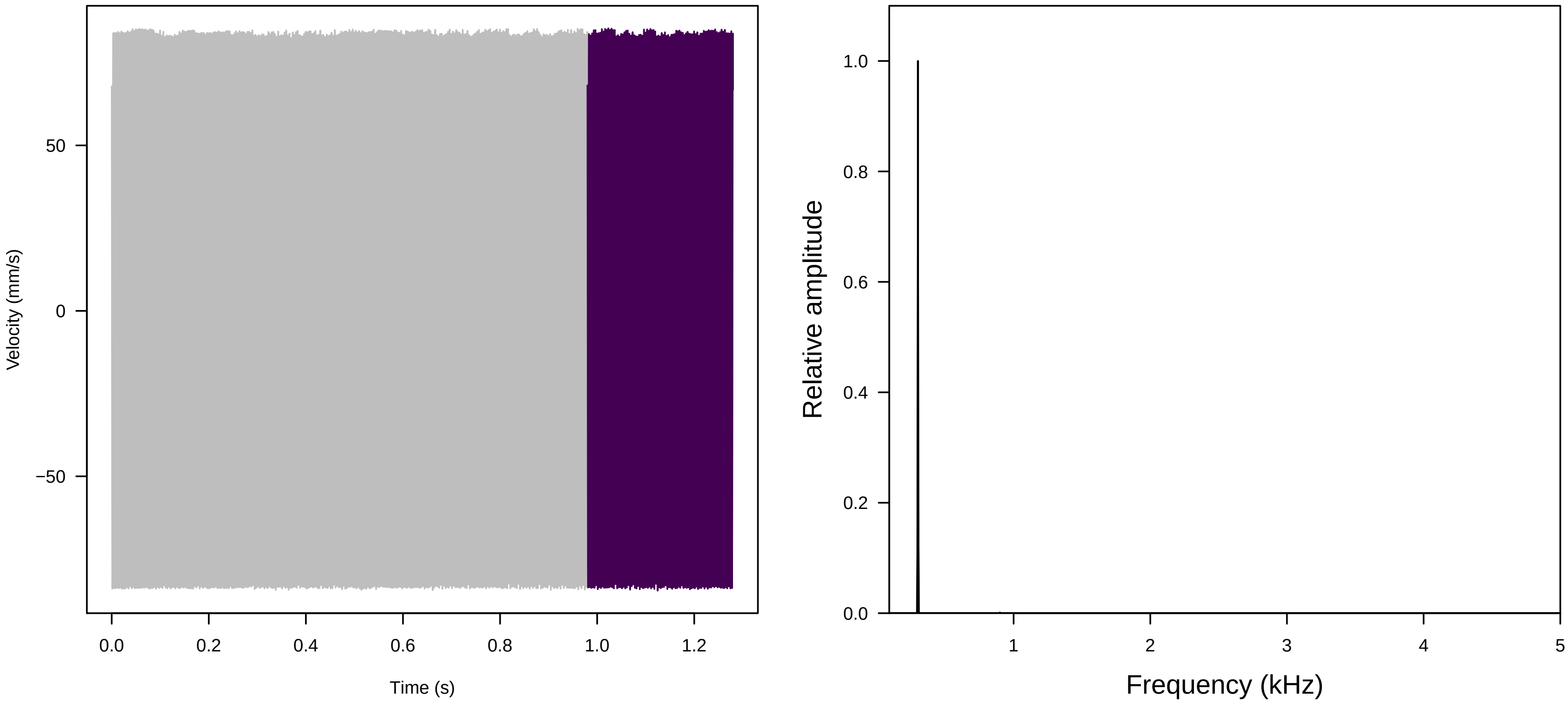

Vel. = 0.057 ; Str. = PA ; Axis = x ; Fl. accession = 10−s−81−8

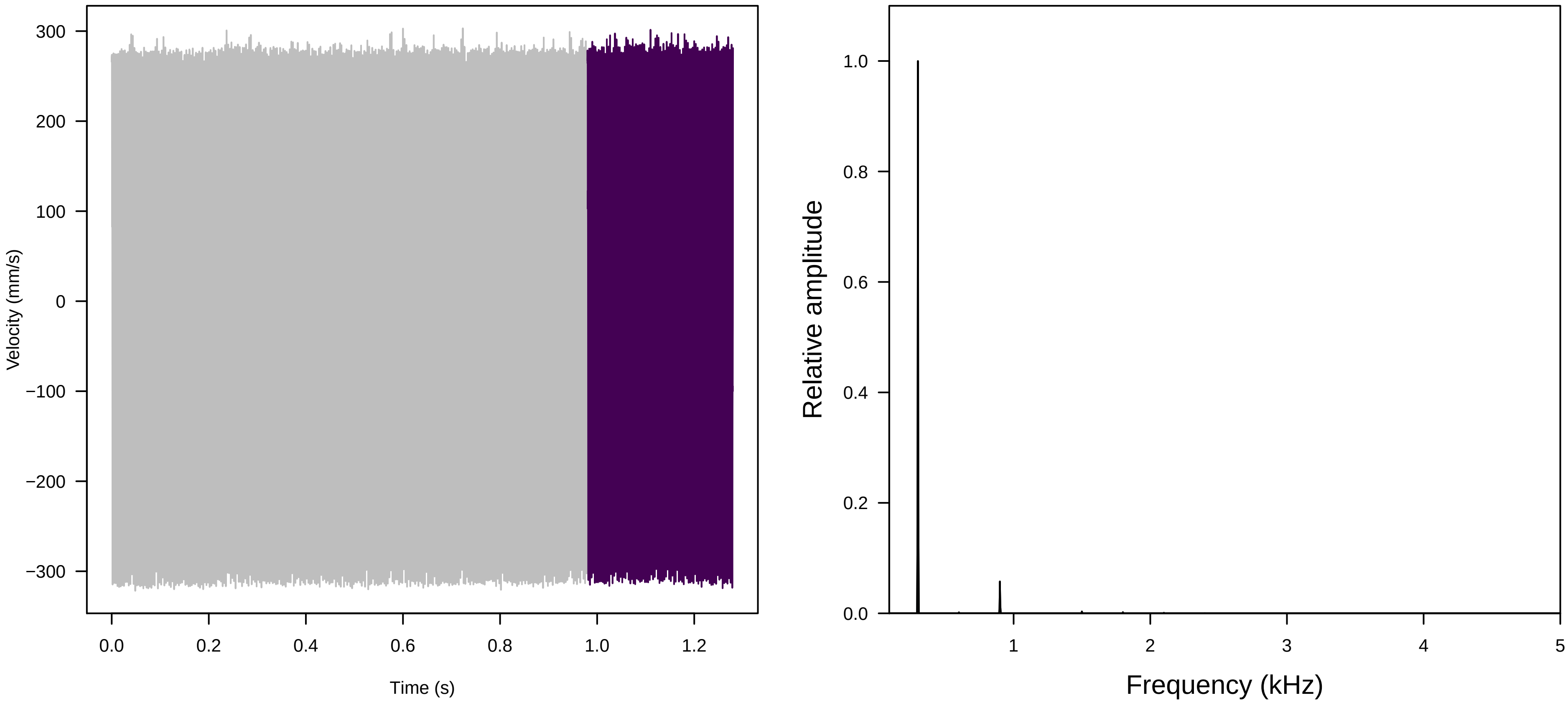

Vel. = 0.057 ; Str. = Receptacle ; Axis = x ; Fl. accession = 10−s−81−8

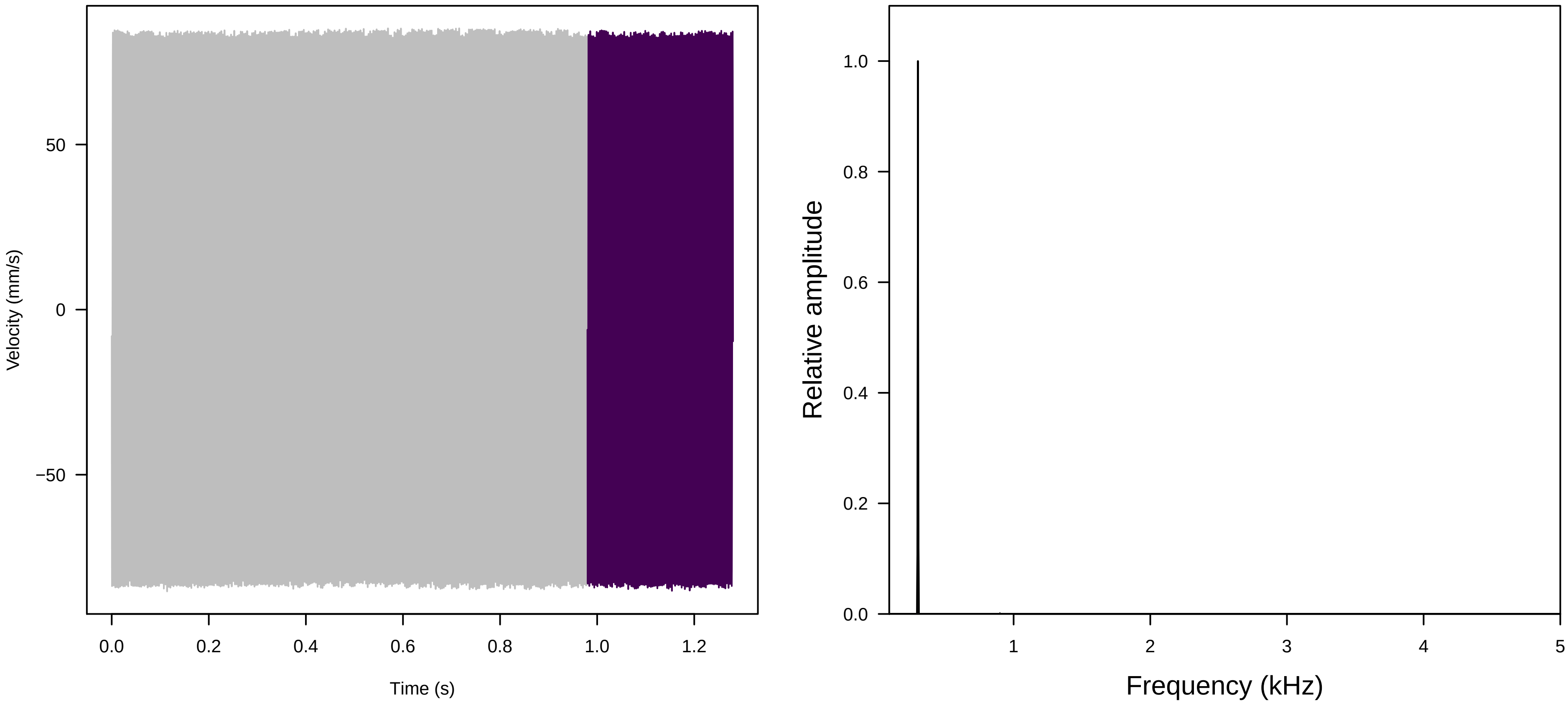

Vel. = 0.057 ; Str. = Corolla ; Axis = z ; Fl. accession = 10−s−81−8

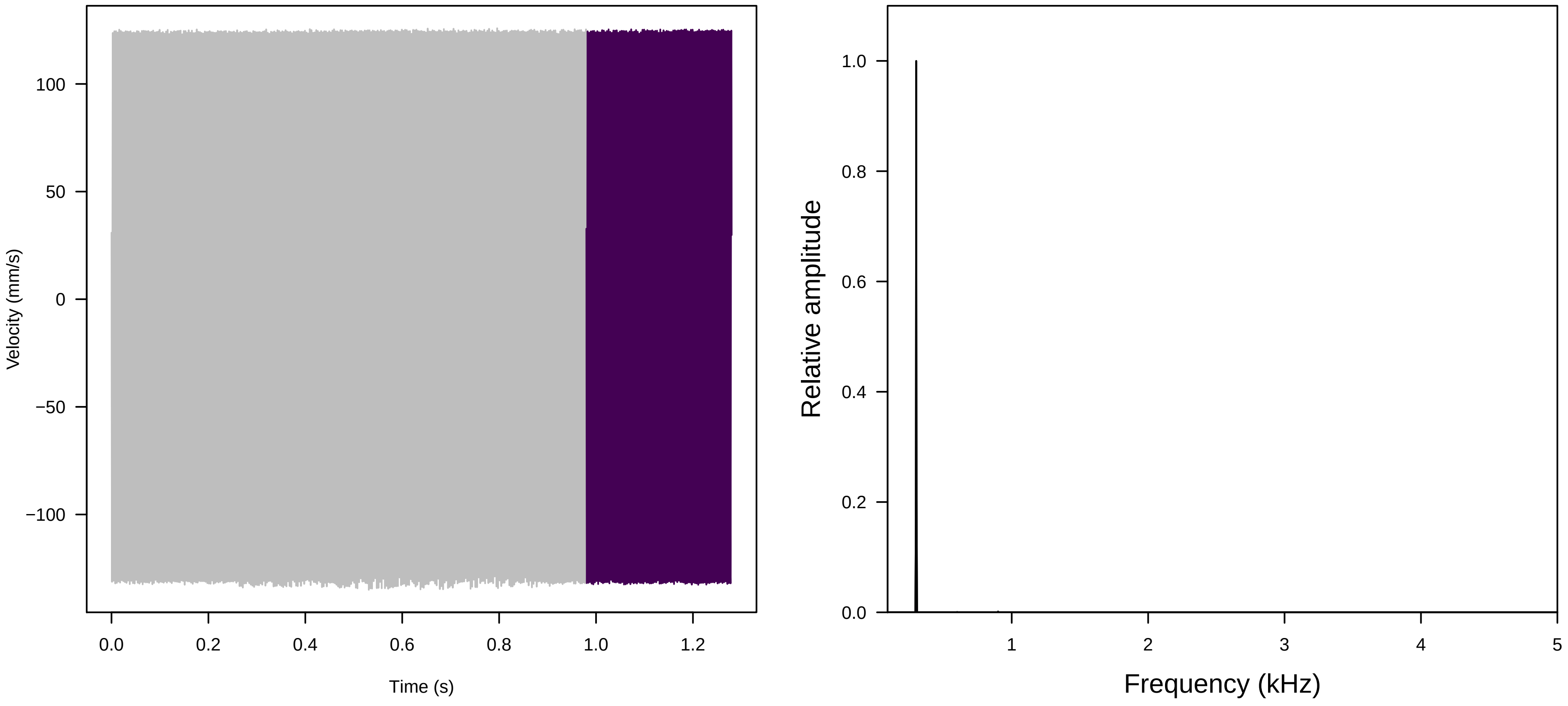

Vel. = 0.057 ; Str. = Receptacle ; Axis = z ; Fl. accession = 10−s−81−8

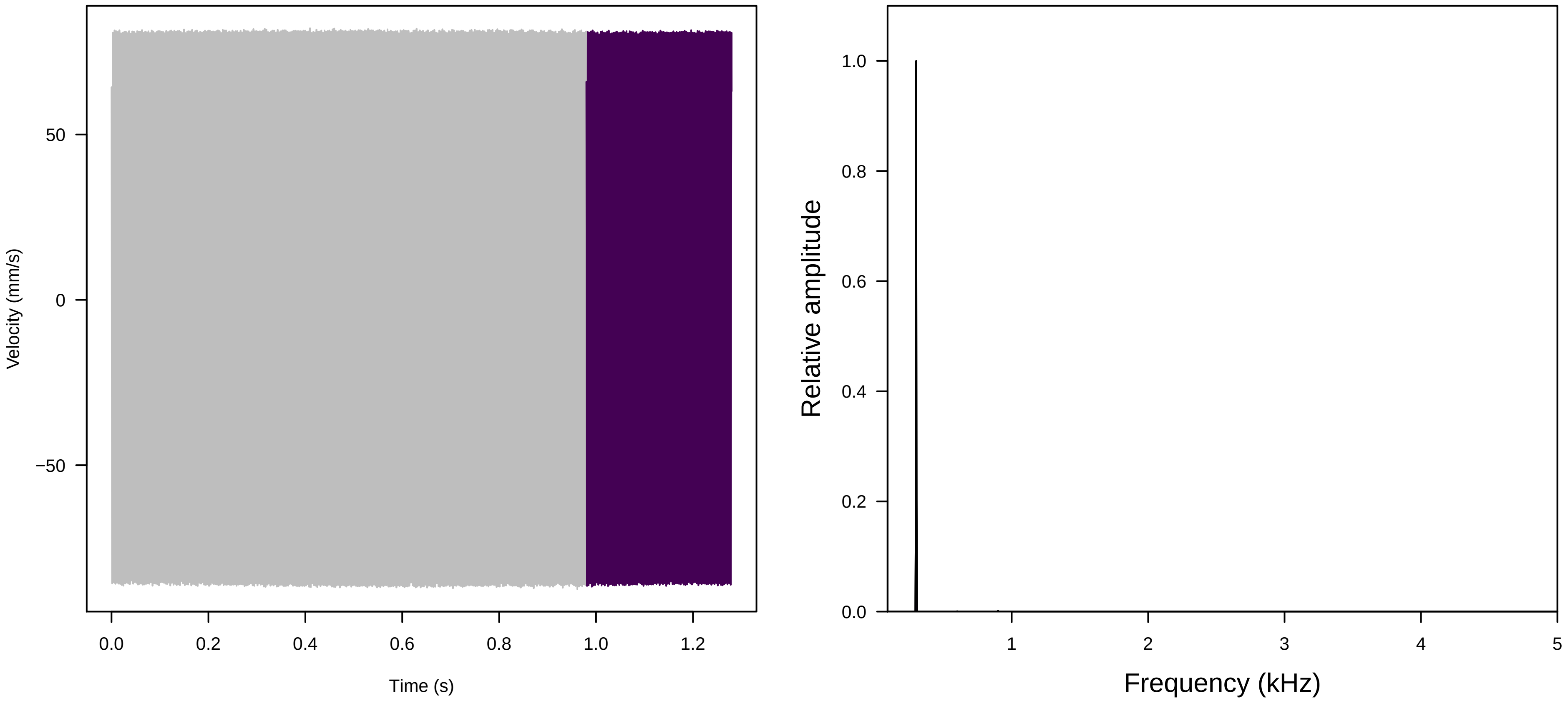

Vel. = 0.057 ; Str. = FA ; Axis = z ; Fl. accession = 10−s−81−8

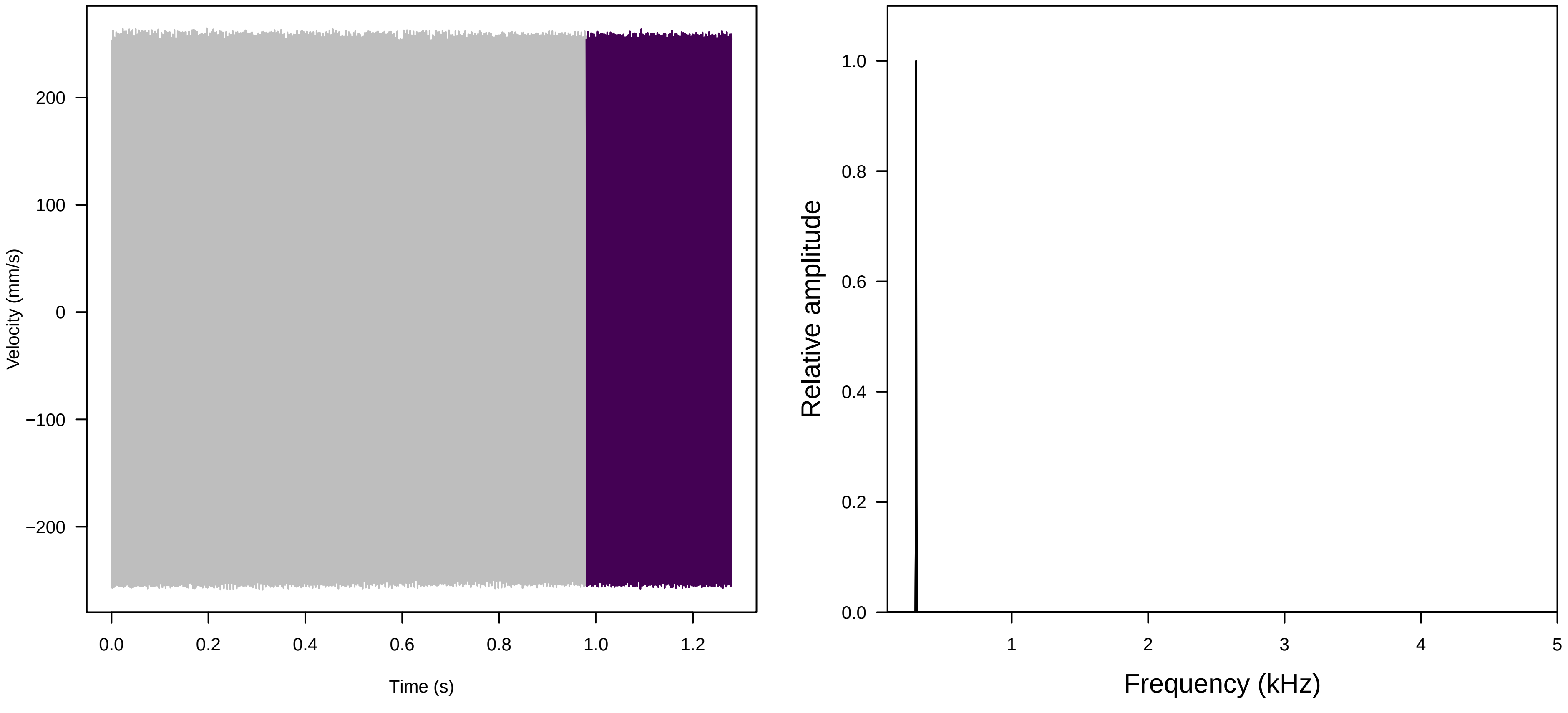

Vel. = 0.057 ; Str. = Receptacle ; Axis = z ; Fl. accession = 10−s−81−8

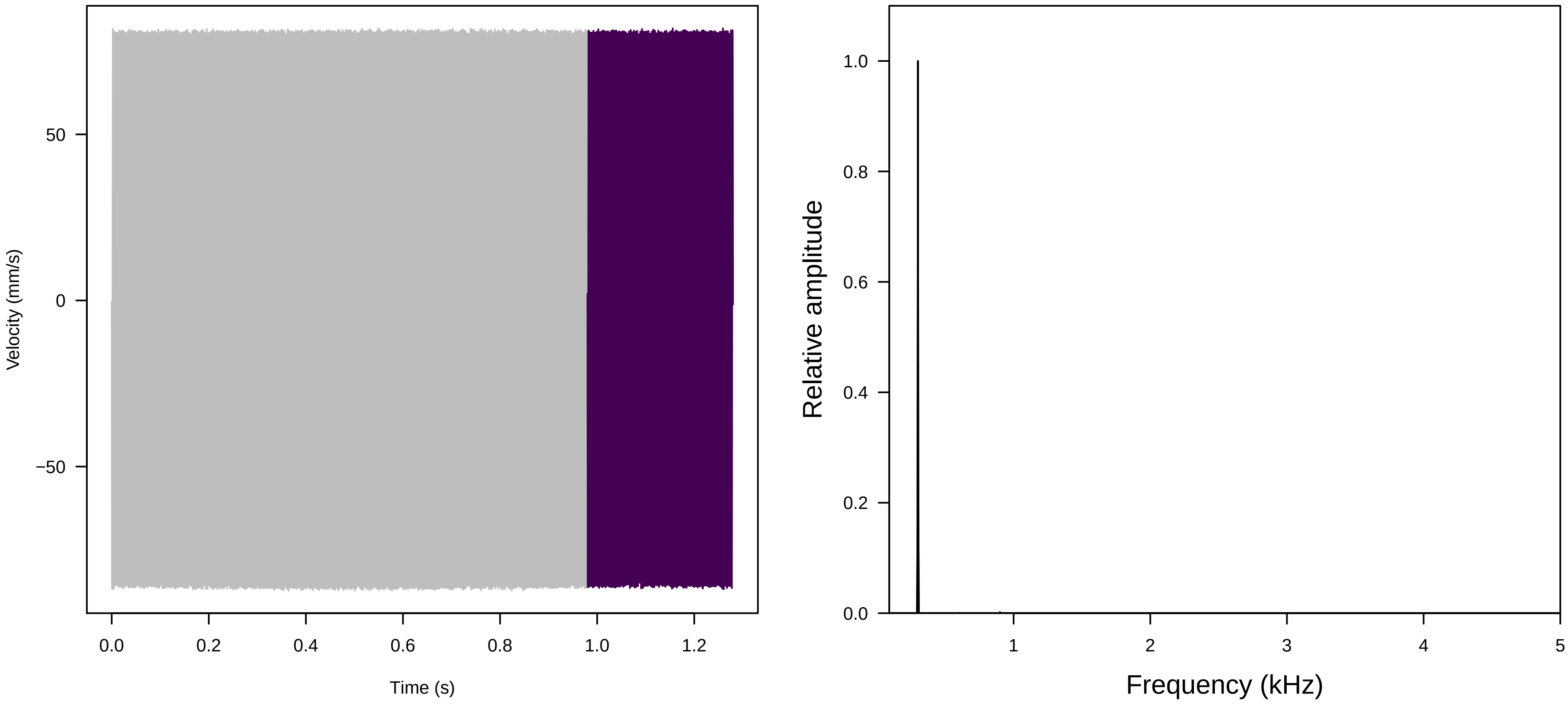

Vel. = 0.057 ; Str. = PA ; Axis = z ; Fl. accession = 10−s−81−8

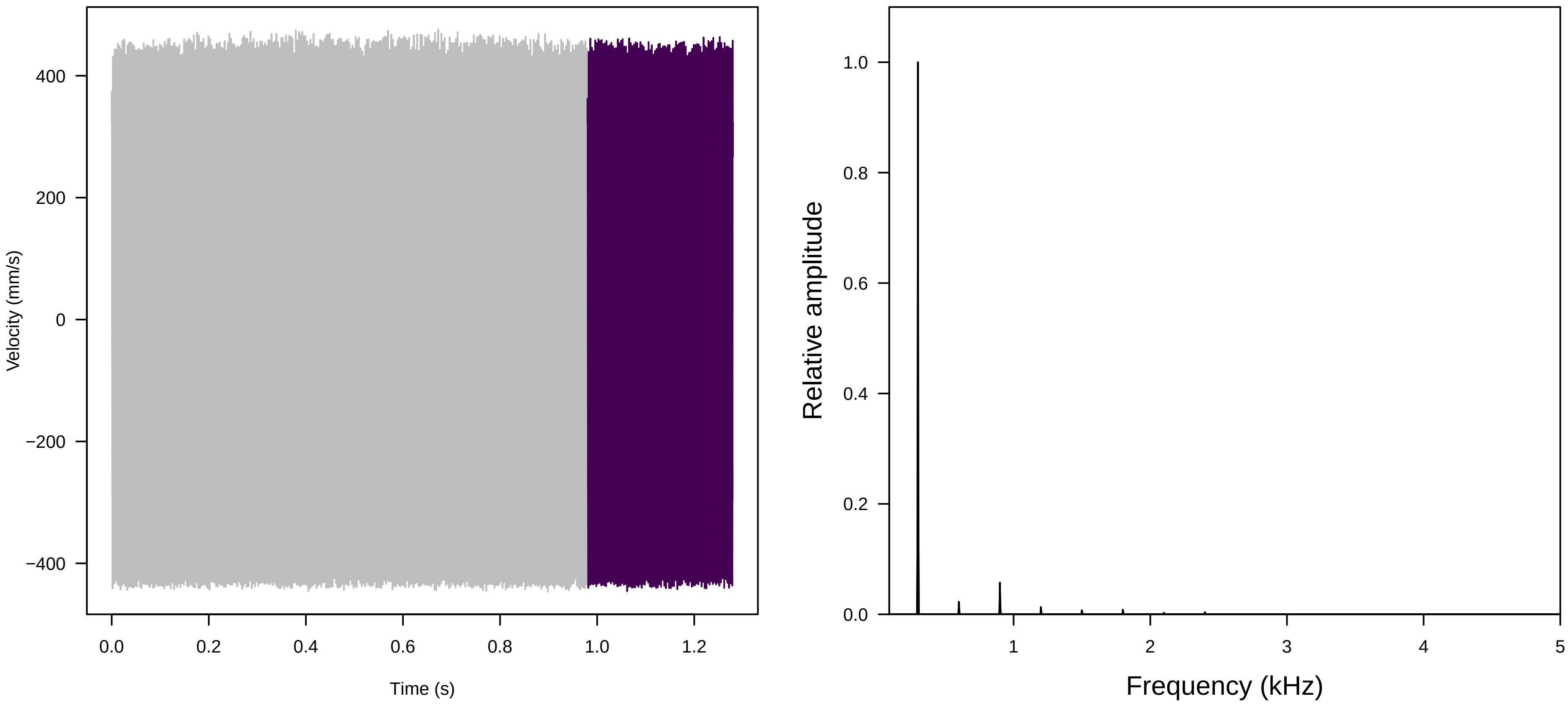

Vel. = 0.057 ; Str. = Receptacle ; Axis = z ; Fl. accession = 10−s−81−8

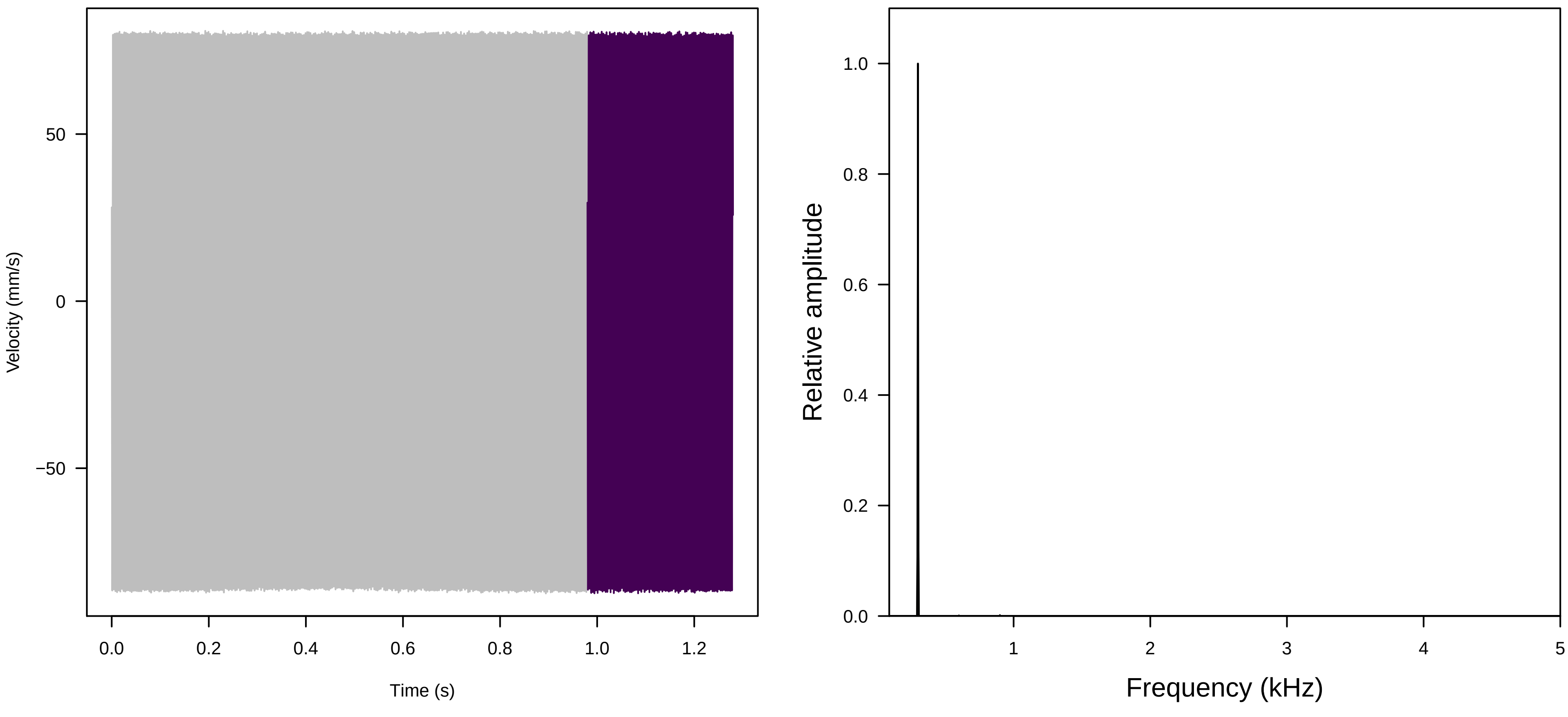

Vel. = 0.028 ; Str. = PA ; Axis = z ; Fl. accession = 10−s−81−8

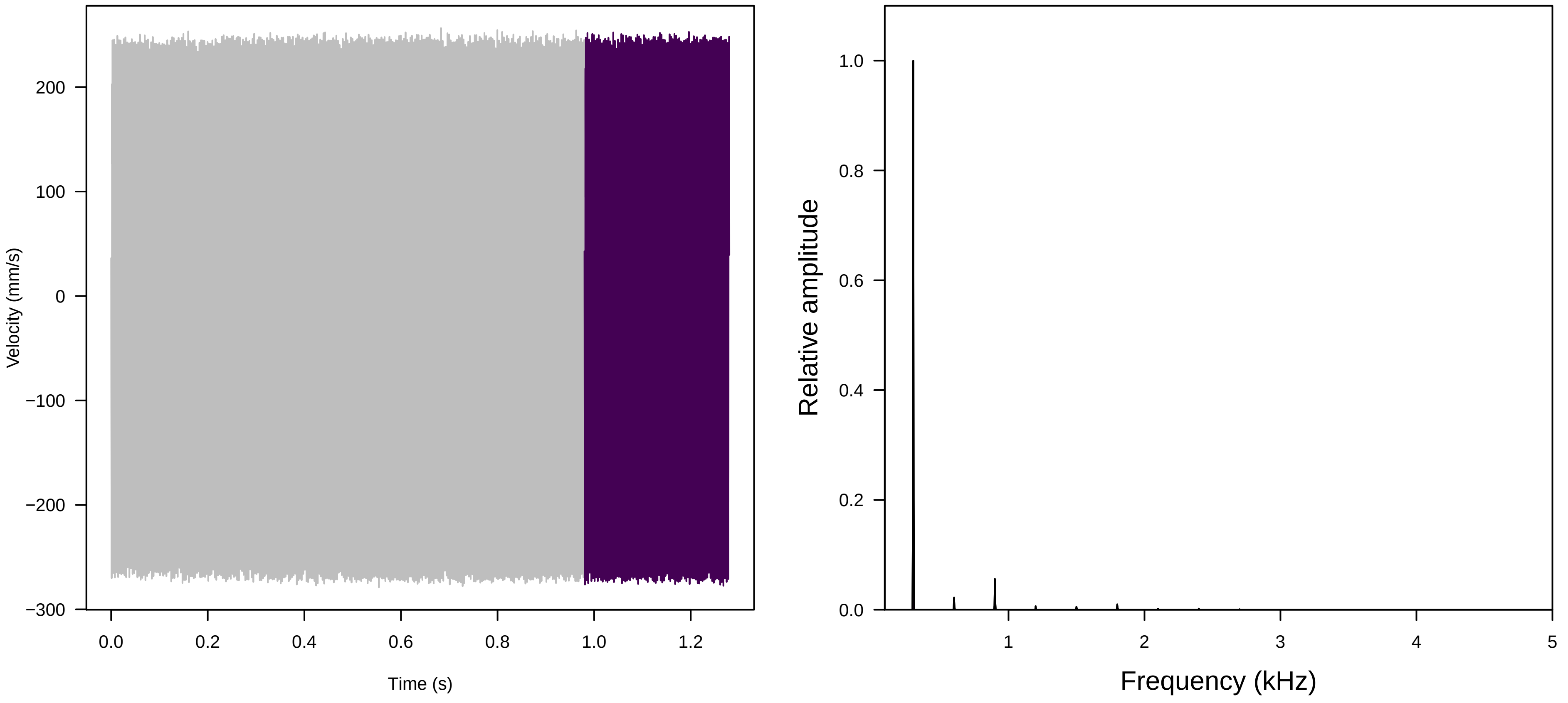

Vel. = 0.028 ; Str. = Receptacle ; Axis = z ; Fl. accession = 10−s−81−8

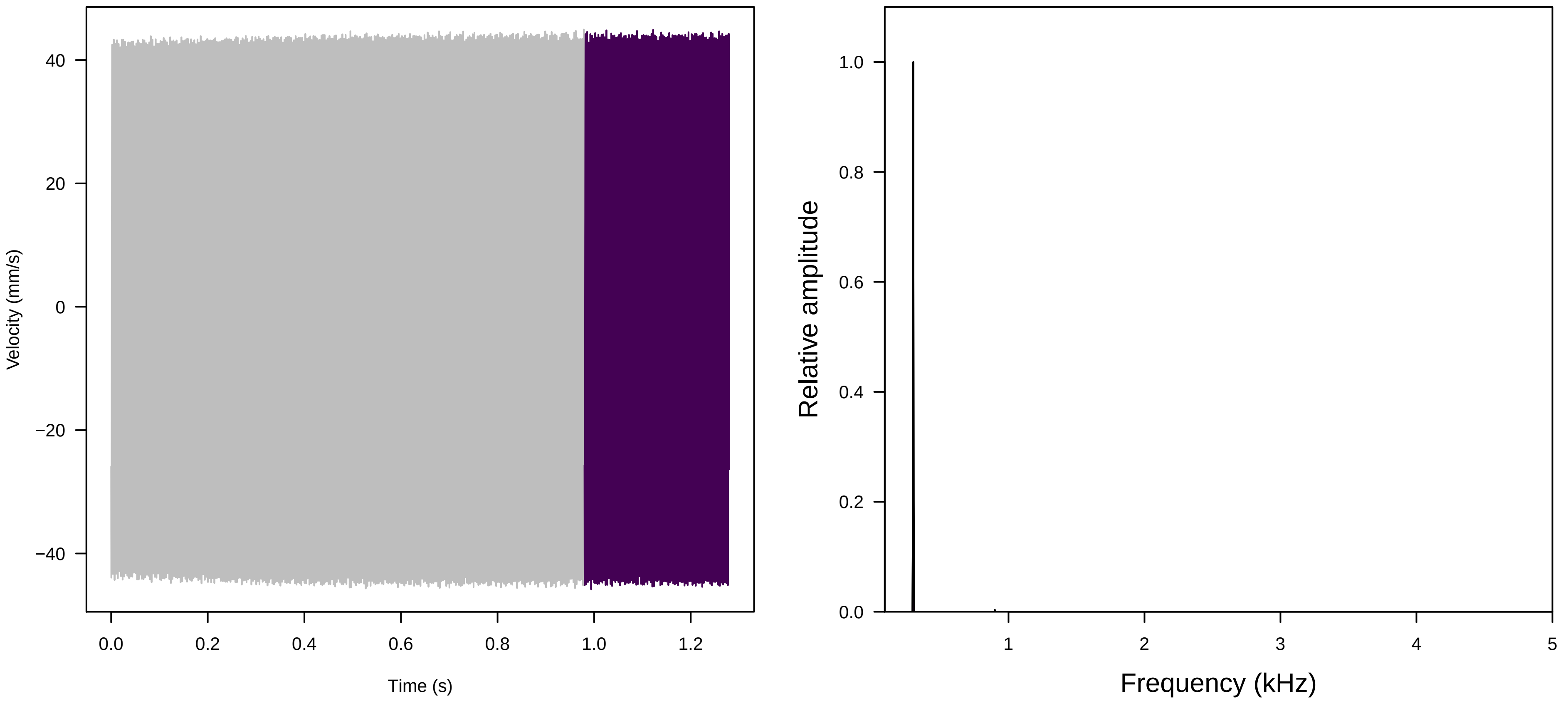

Vel. = 0.028 ; Str. = FA ; Axis = z ; Fl. accession = 10−s−81−8

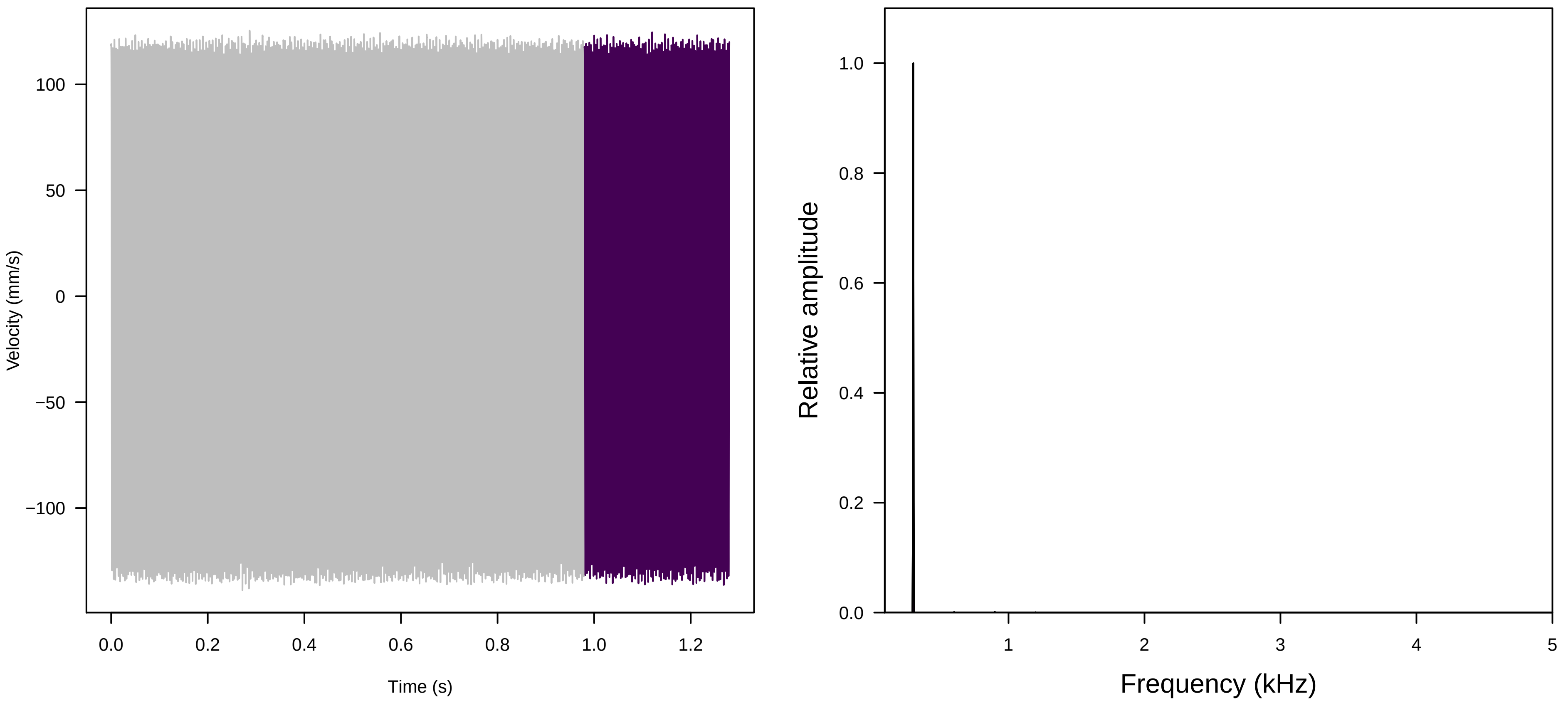

Vel. = 0.028 ; Str. = Receptacle ; Axis = z ; Fl. accession = 10−s−81−8

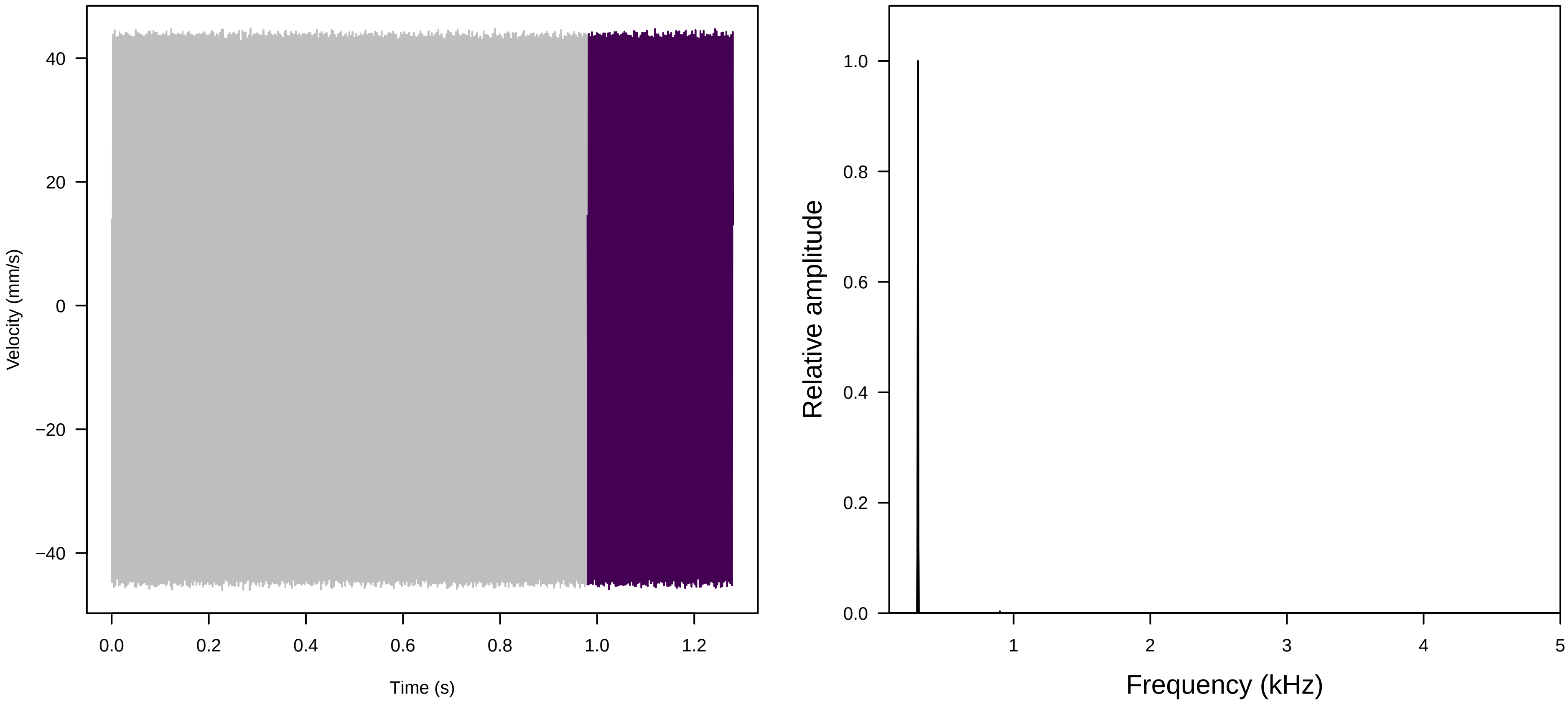

Vel. = 0.028 ; Str. = Corolla ; Axis = z ; Fl. accession = 10−s−81−8

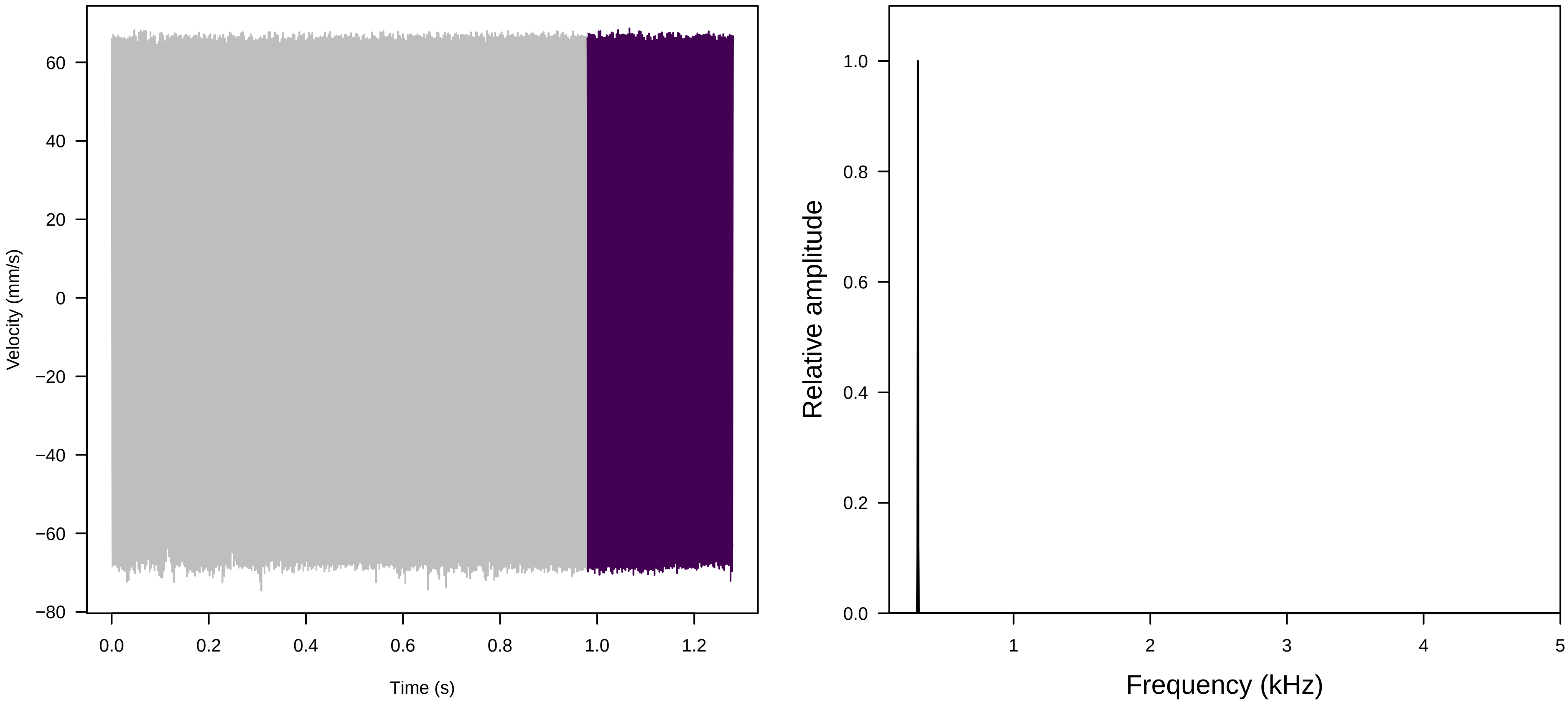

Vel. = 0.028 ; Str. = Receptacle ; Axis = z ; Fl. accession = 10−s−81−8

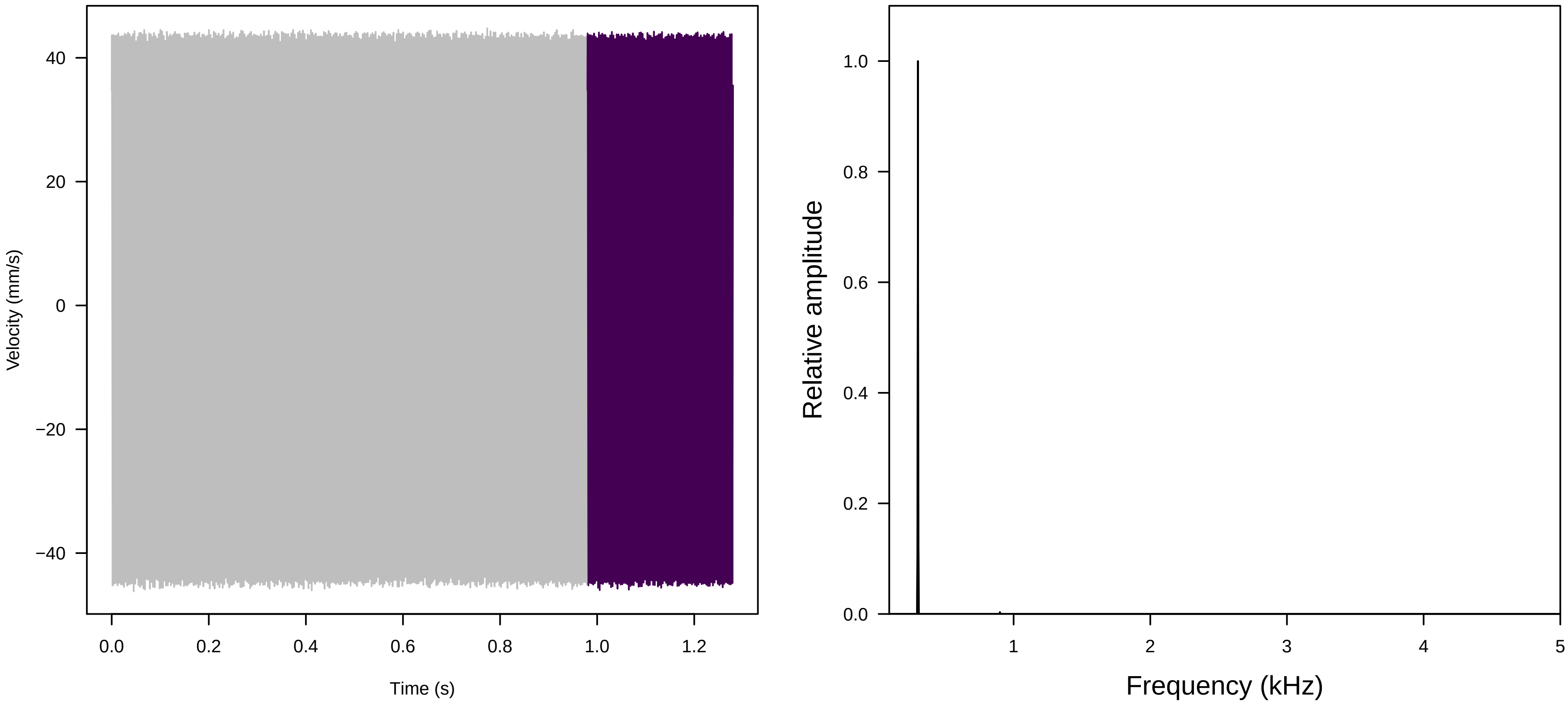

Vel. = 0.014 ; Str. = Corolla ; Axis = z ; Fl. accession = 10−s−81−8

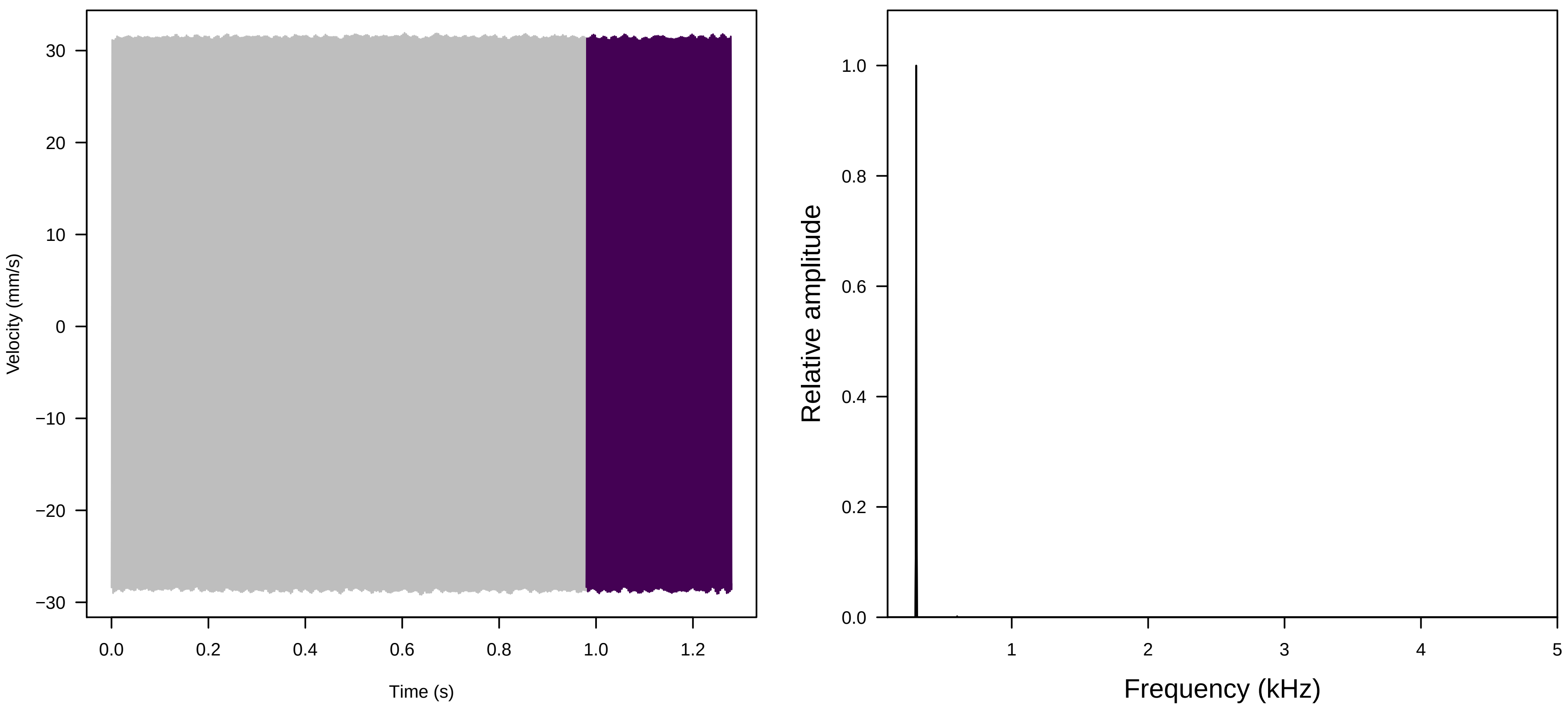

Vel. = 0.014 ; Str. = Receptacle ; Axis = z ; Fl. accession = 10−s−81−8

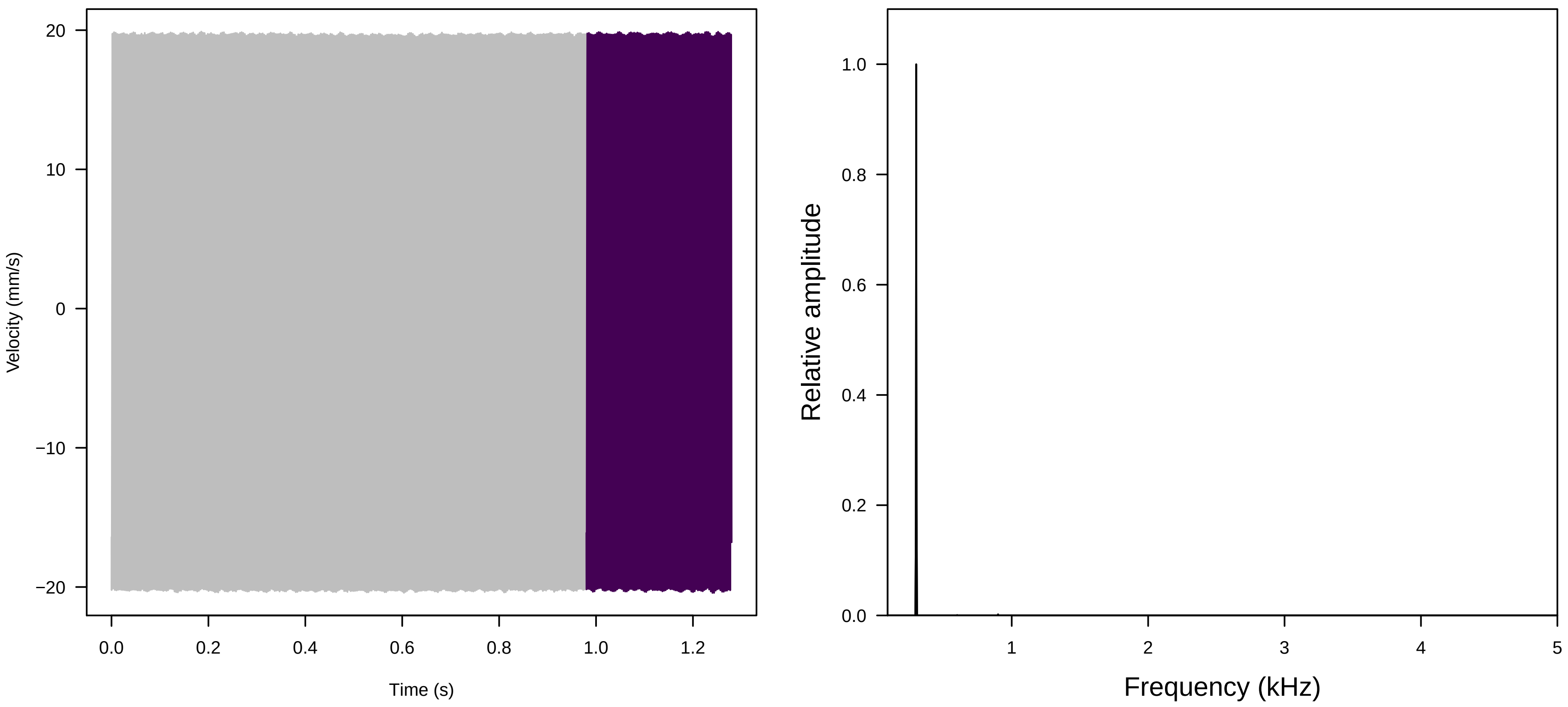

Vel. = 0.014 ; Str. = FA ; Axis = z ; Fl. accession = 10−s−81−8

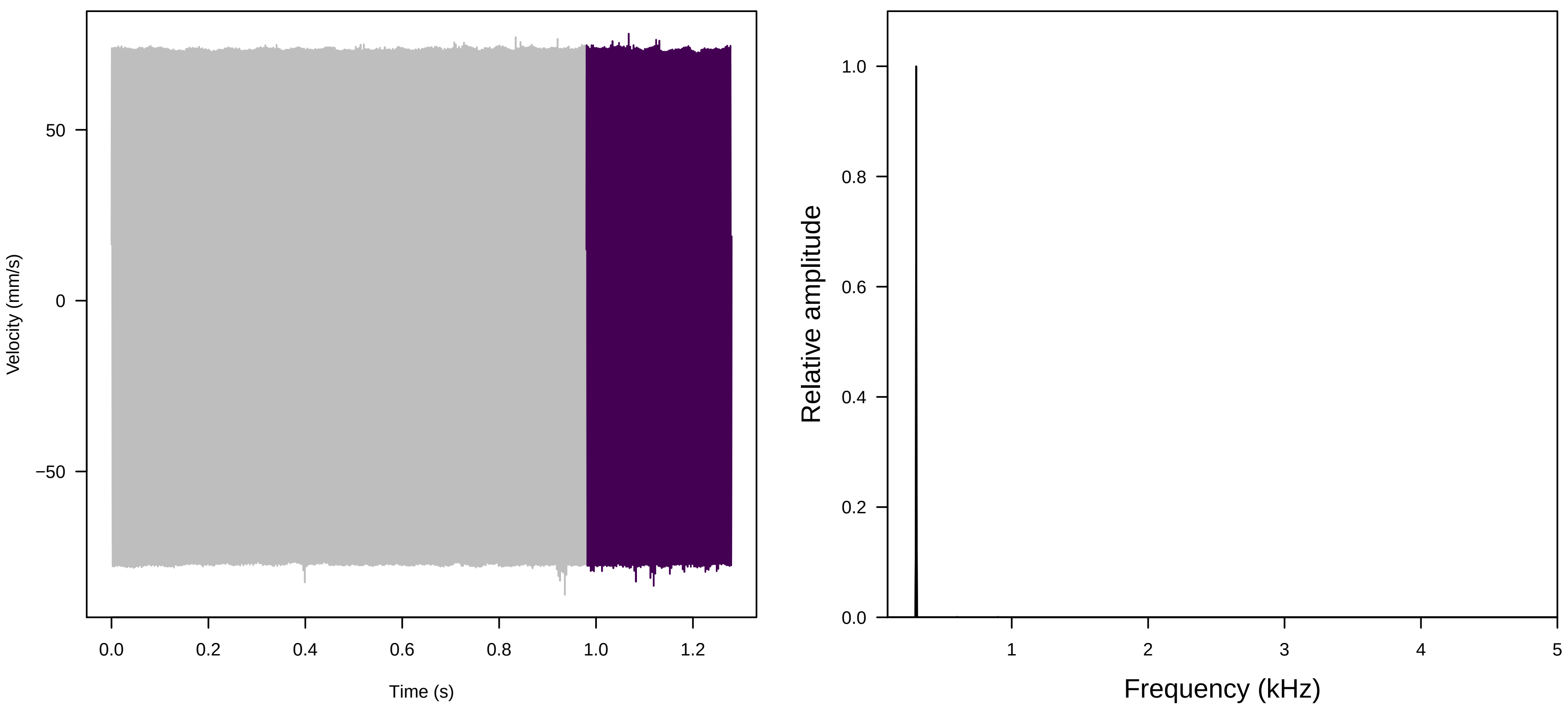

Vel. = 0.014 ; Str. = Receptacle ; Axis = z ; Fl. accession = 10−s−81−8

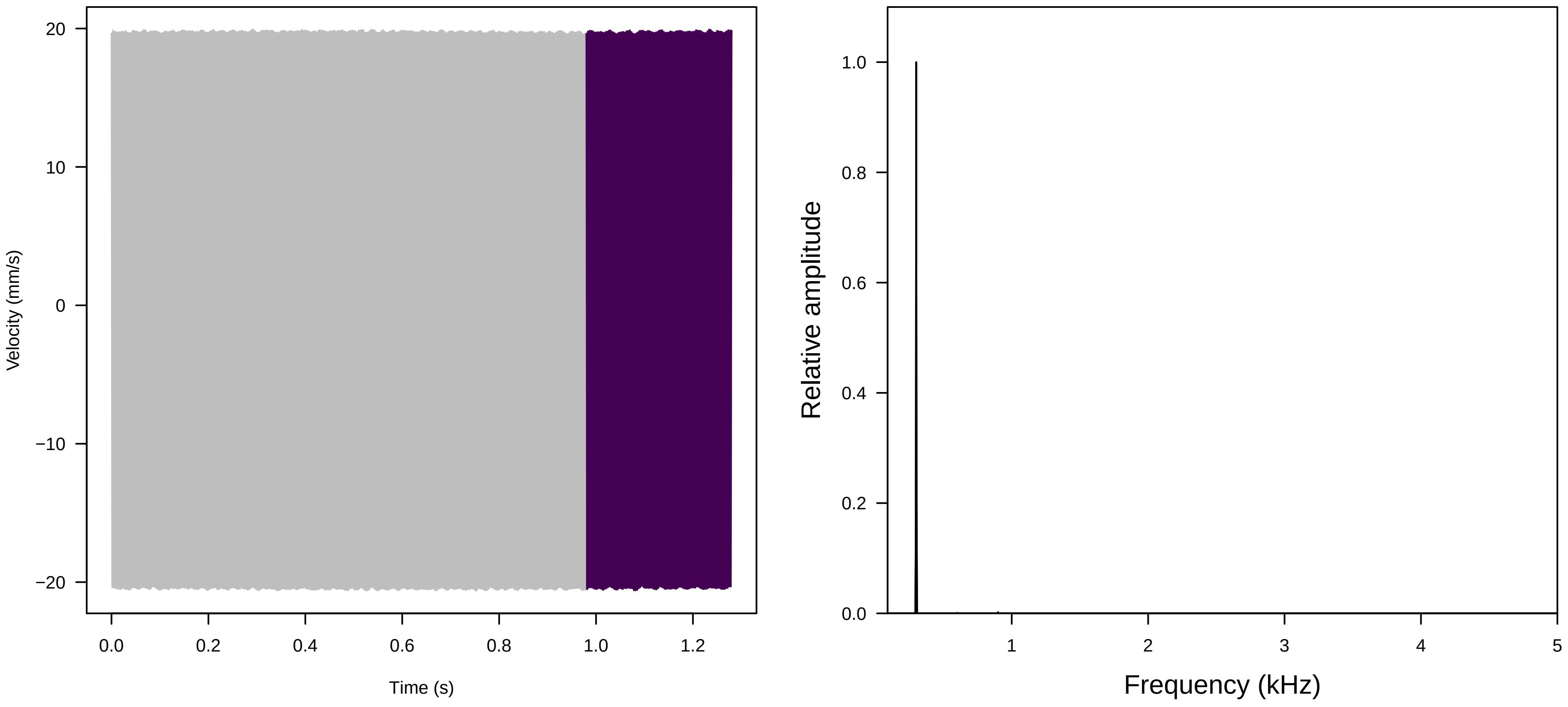

Vel. = 0.014 ; Str. = PA ; Axis = z ; Fl. accession = 10−s−81−8

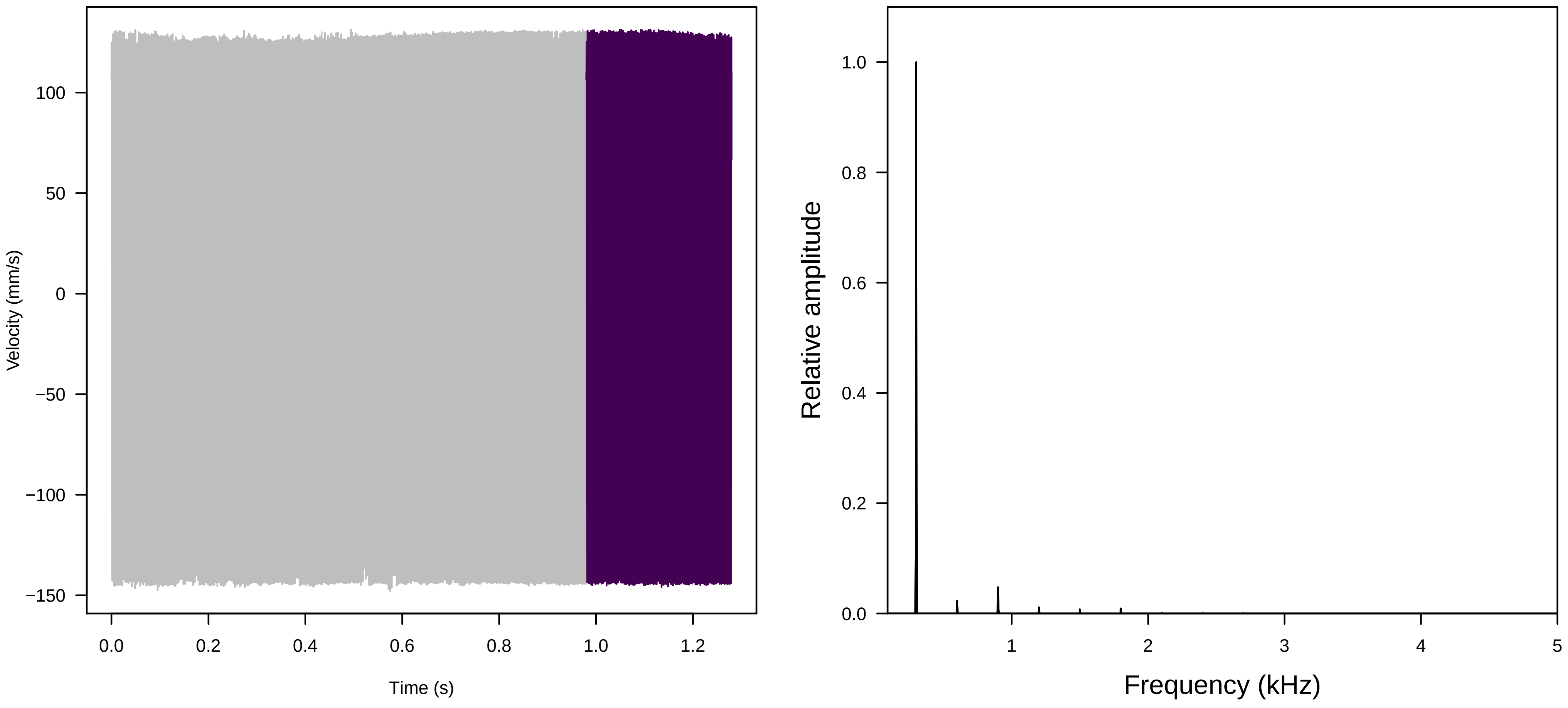

Vel. = 0.014 ; Str. = Receptacle ; Axis = z ; Fl. accession = 10−s−81−8

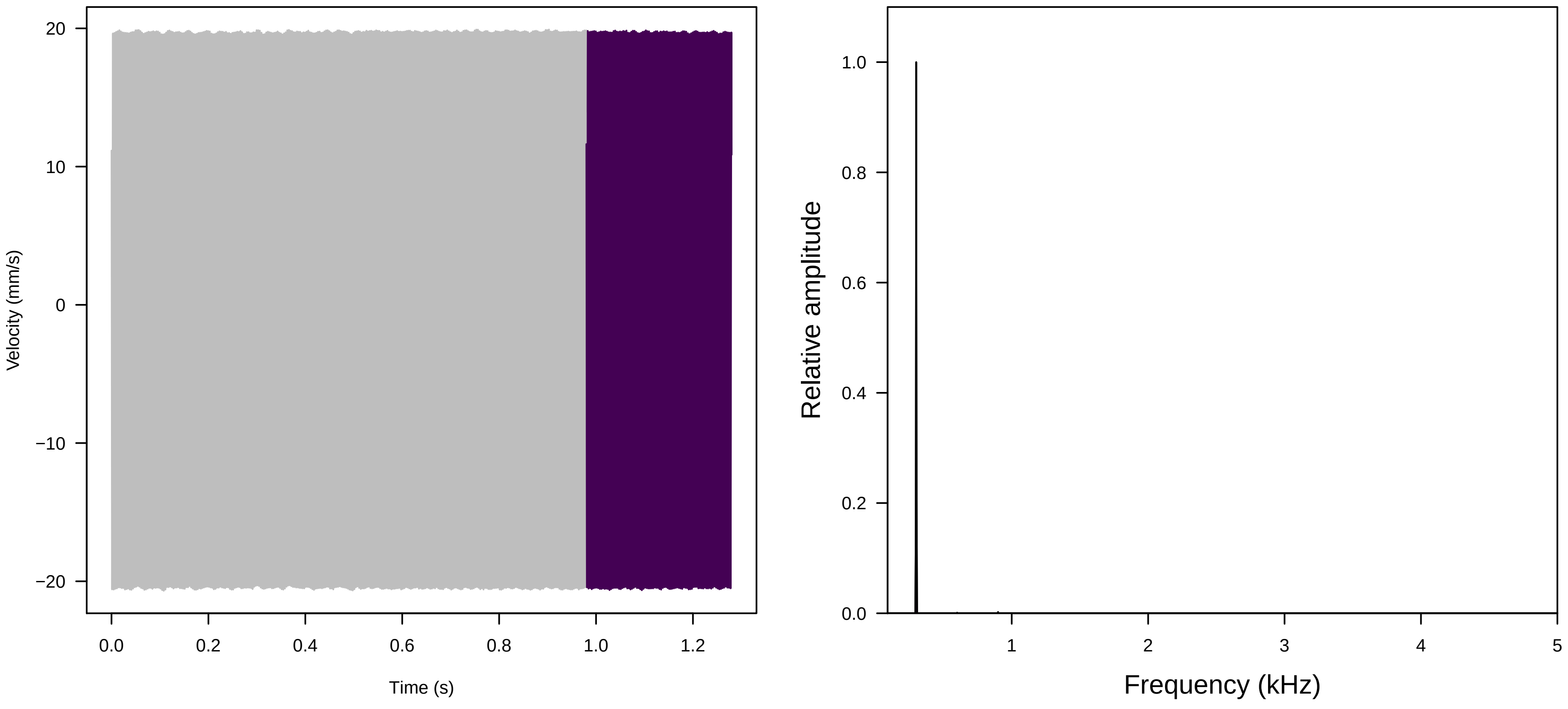

Vel. = 0.014 ; Str. = Corolla ; Axis = y ; Fl. accession = 10−s−81−8

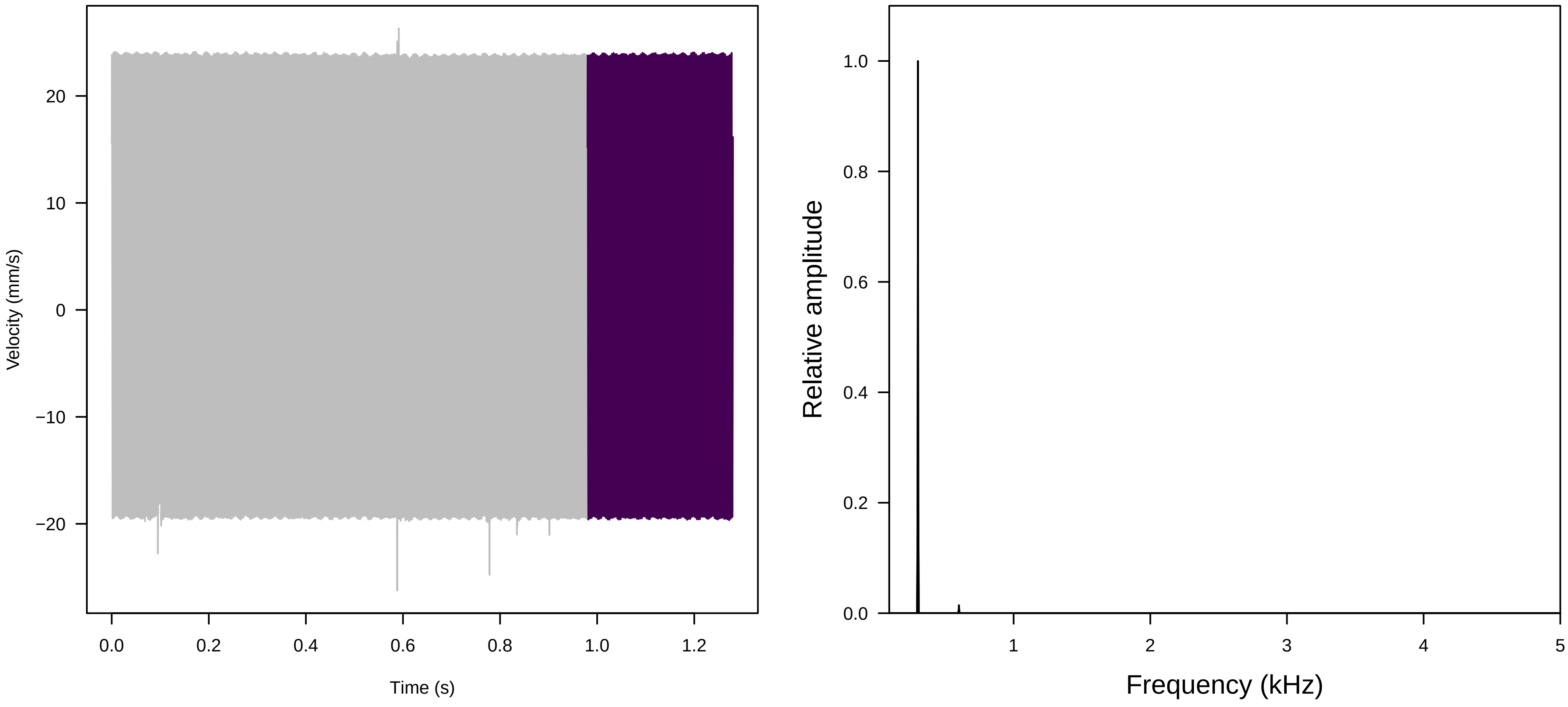

Vel. = 0.014 ; Str. = Receptacle ; Axis = y ; Fl. accession = 10−s−81−8

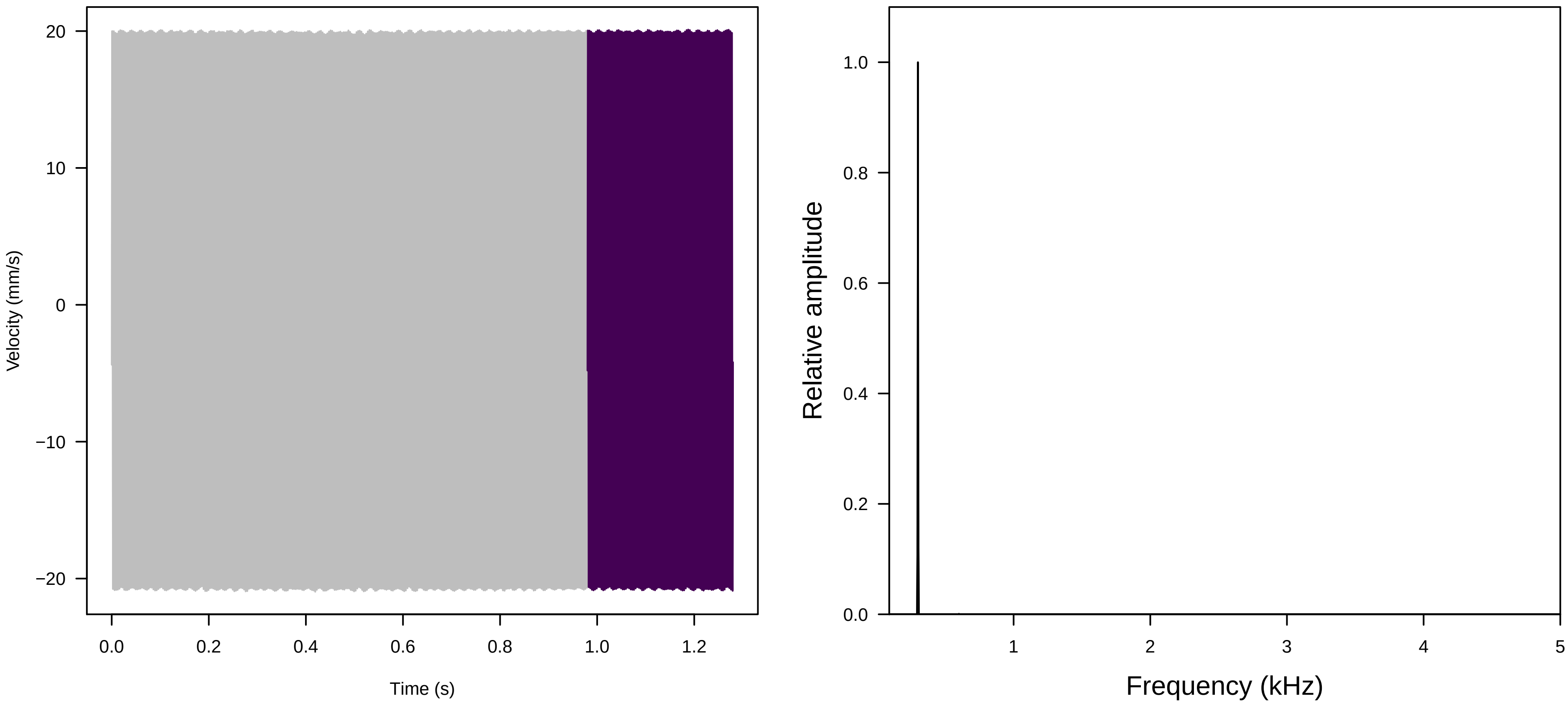

Vel. = 0.014 ; Str. = FA ; Axis = y ; Fl. accession = 10−s−81−8

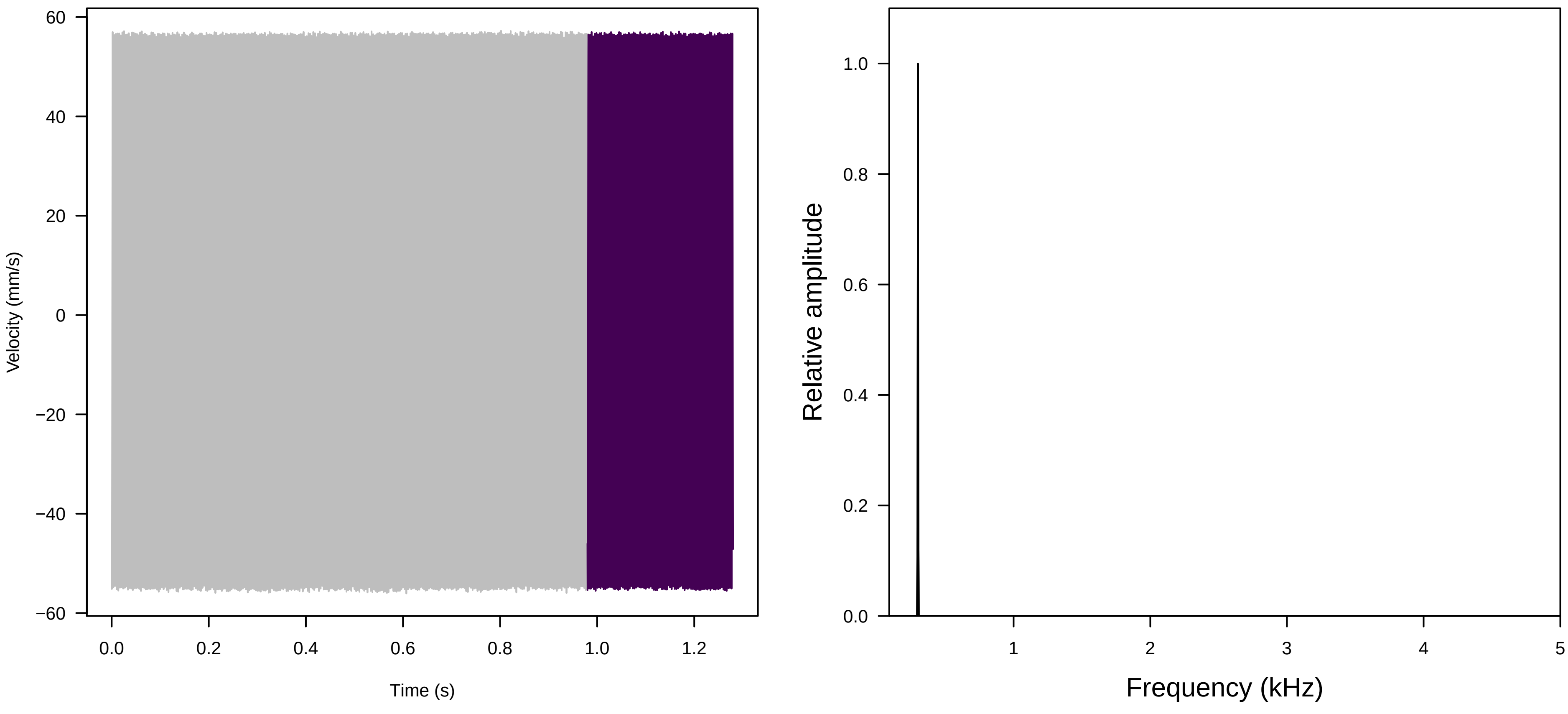

Vel. = 0.014 ; Str. = Receptacle ; Axis = y ; Fl. accession = 10−s−81−8

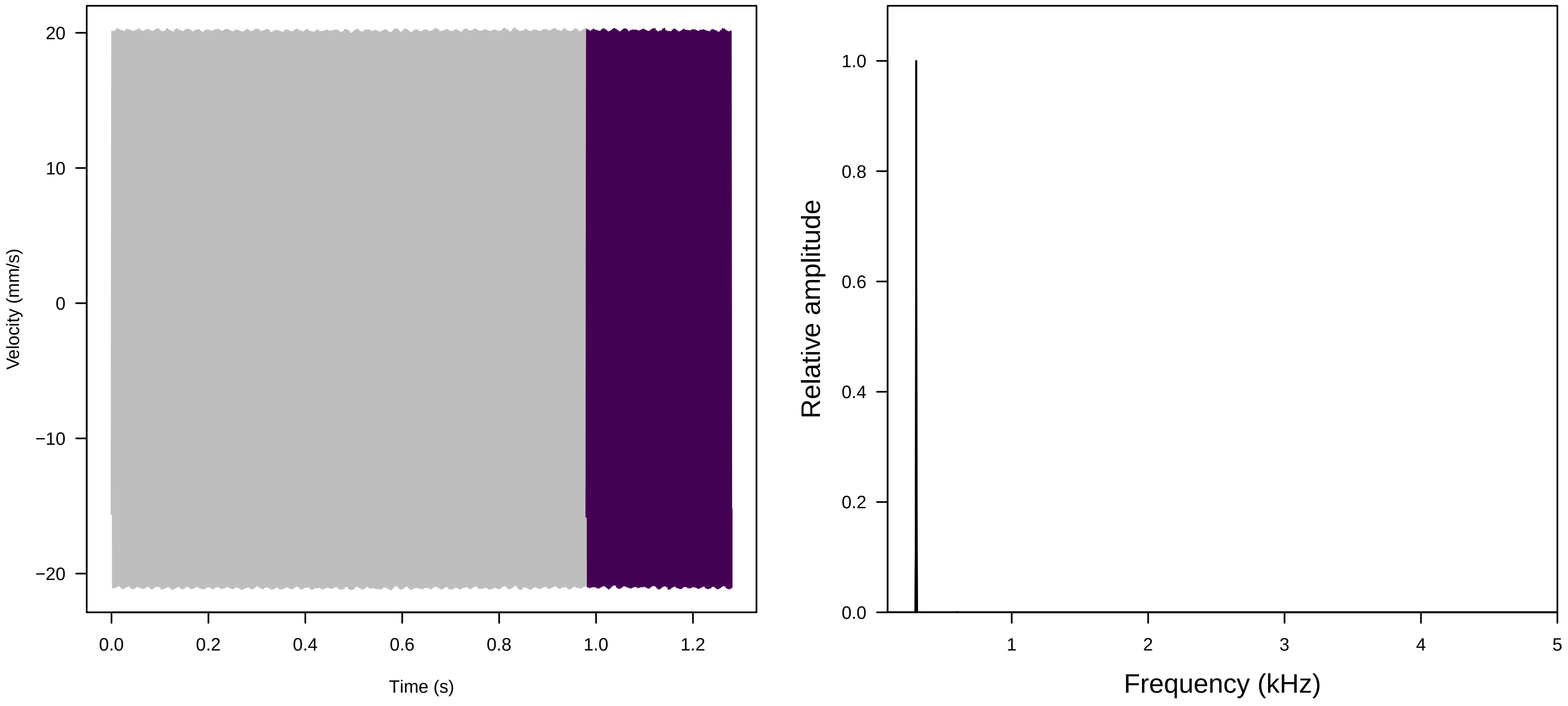

Vel. = 0.014 ; Str. = PA ; Axis = y ; Fl. accession = 10−s−81−8

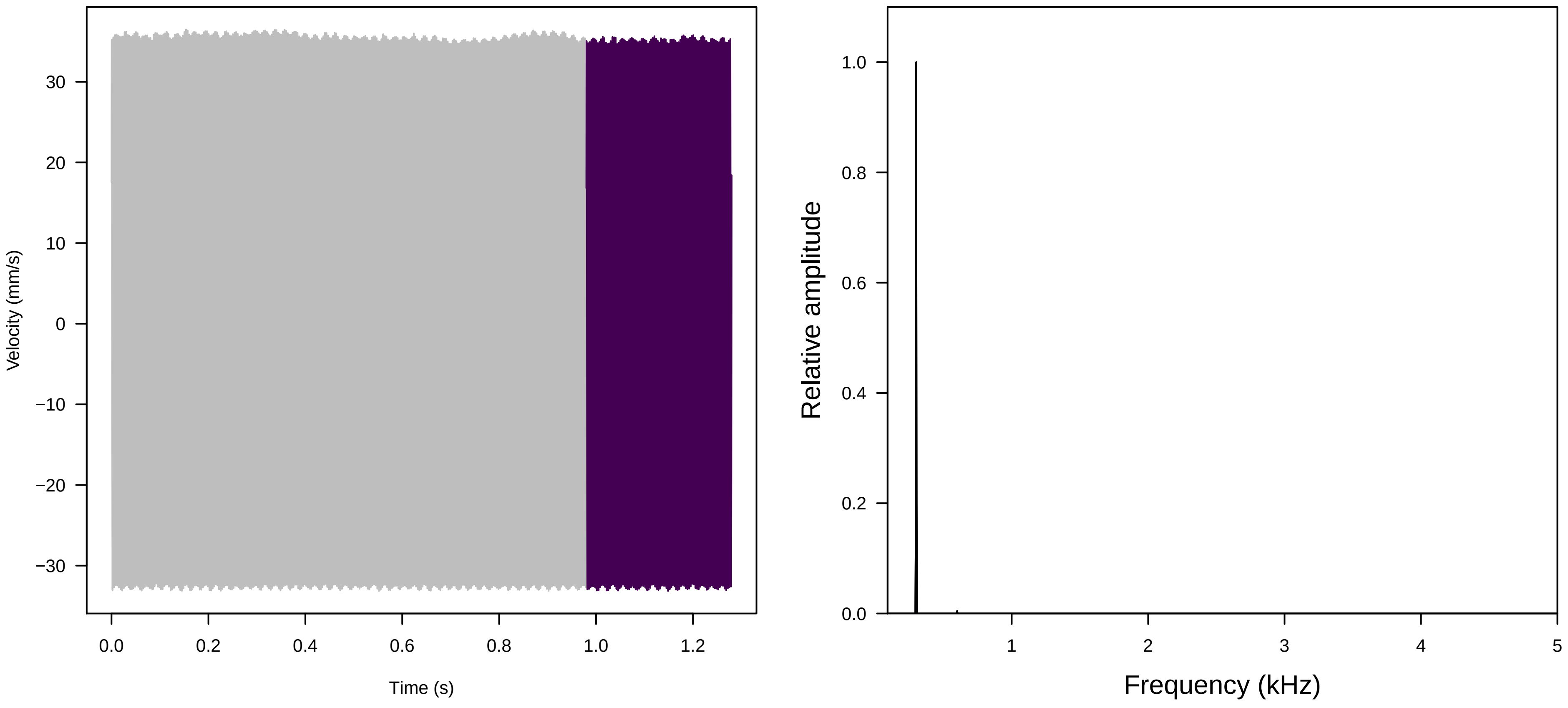

Vel. = 0.014 ; Str. = Receptacle ; Axis = y ; Fl. accession = 10−s−81−8

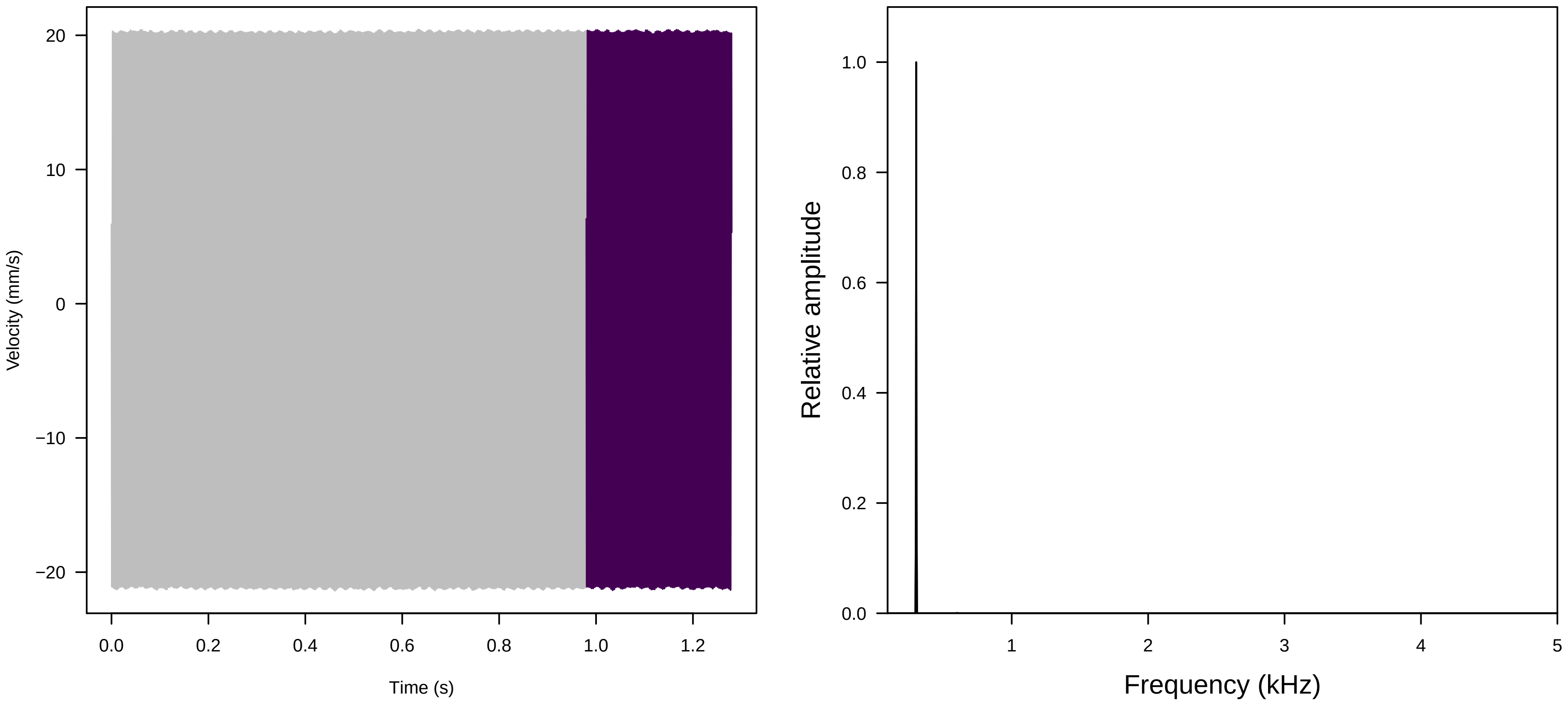

Vel. = 0.028 ; Str. = PA ; Axis = y ; Fl. accession = 10−s−81−8

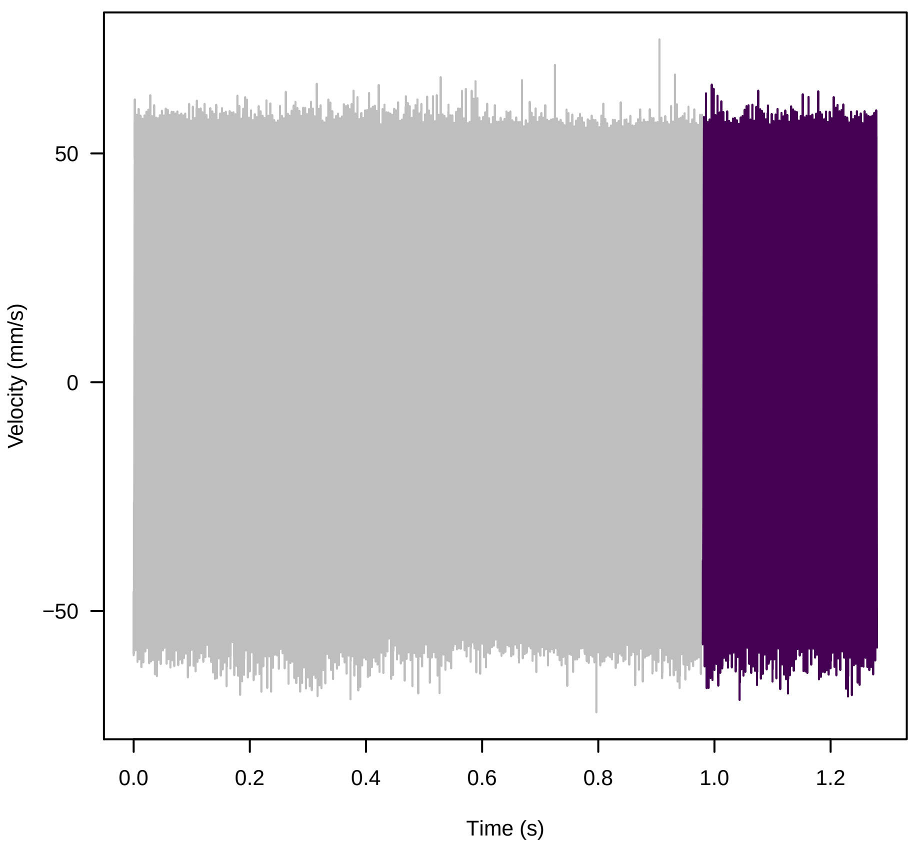

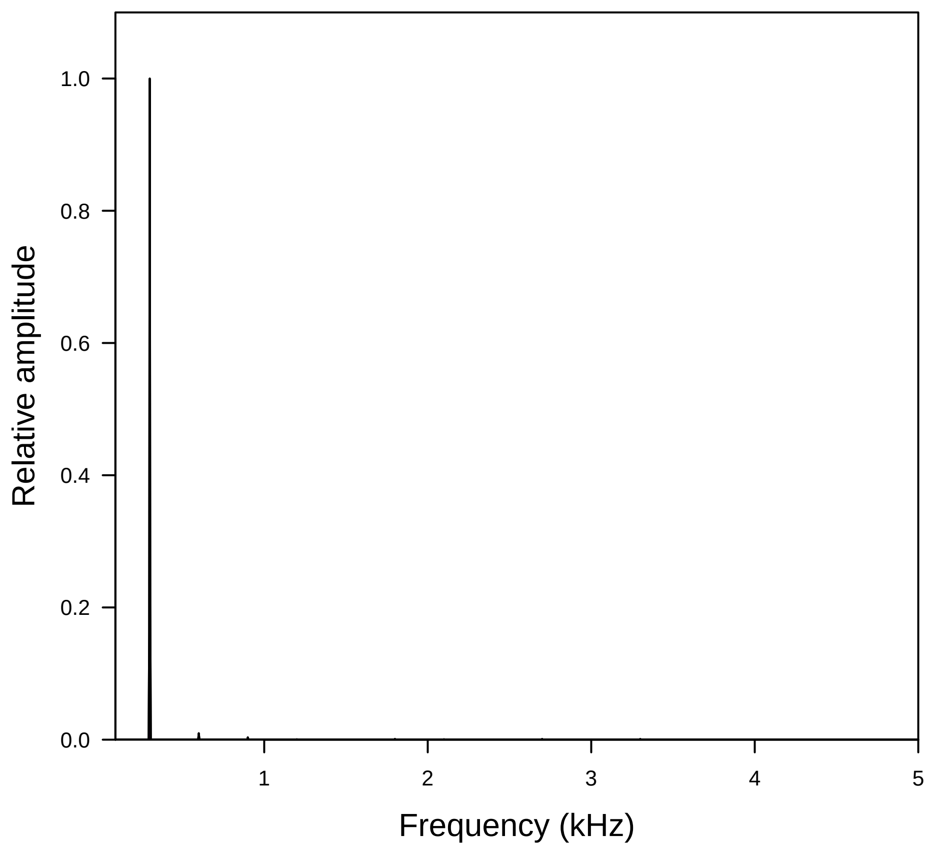

Vel. = 0.028 ; Str. = Receptacle ; Axis = y ; Fl. accession = 10−s−81−8

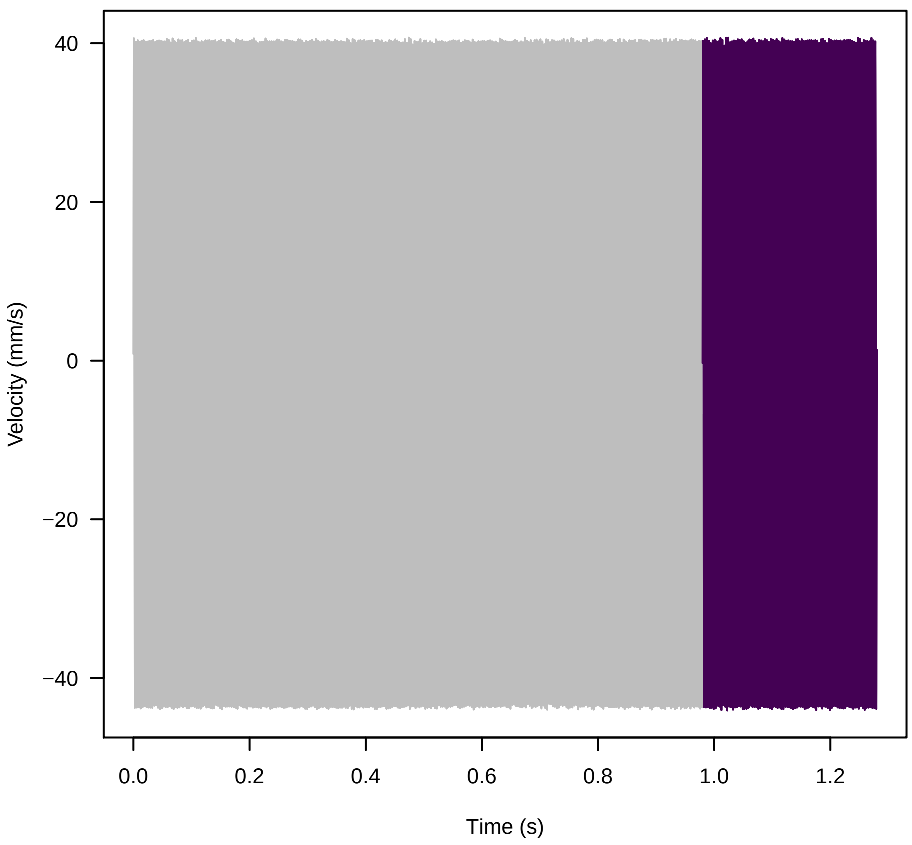

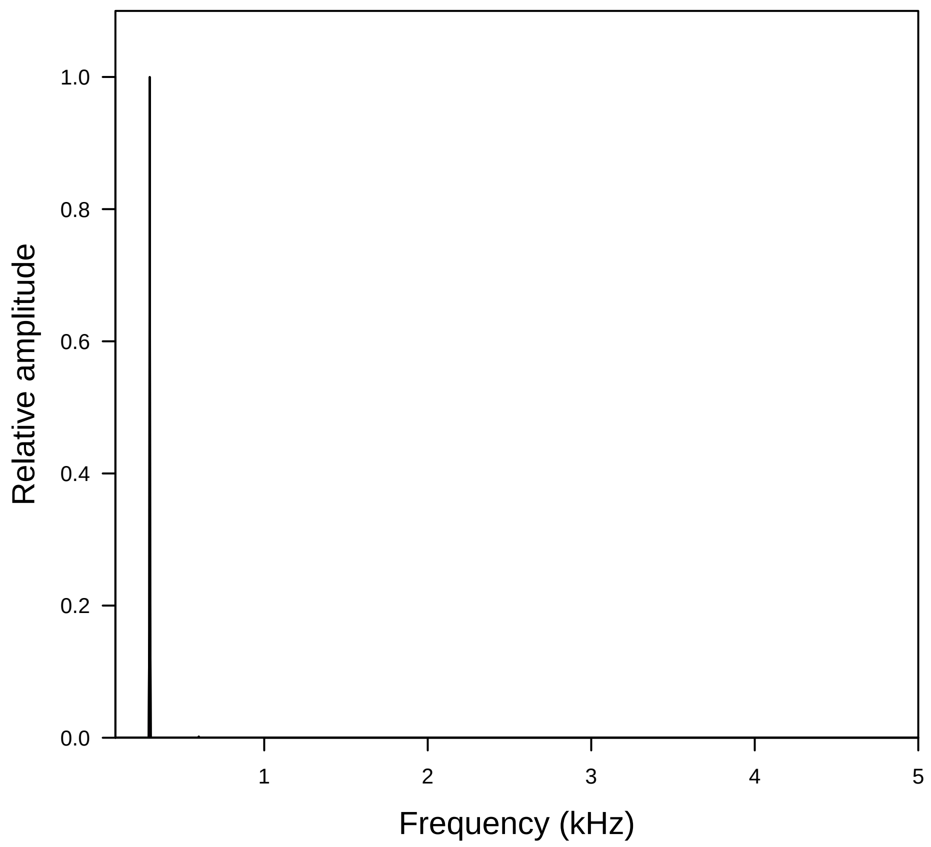

Vel. = 0.028 ; Str. = FA ; Axis = y ; Fl. accession = 10−s−81−8

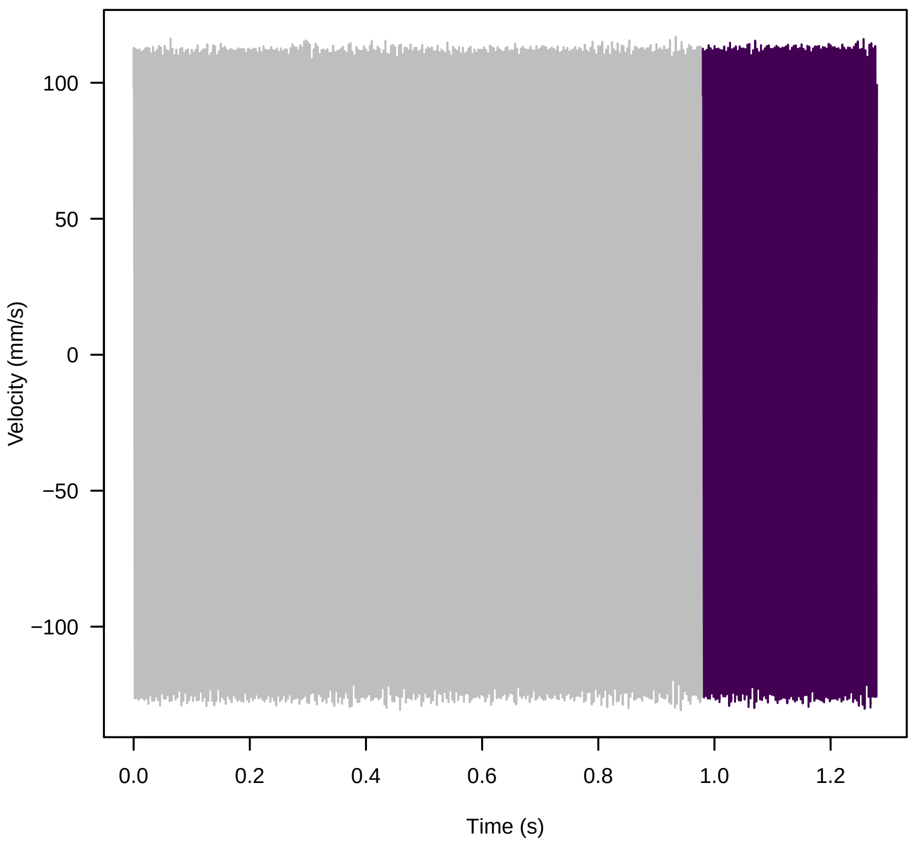

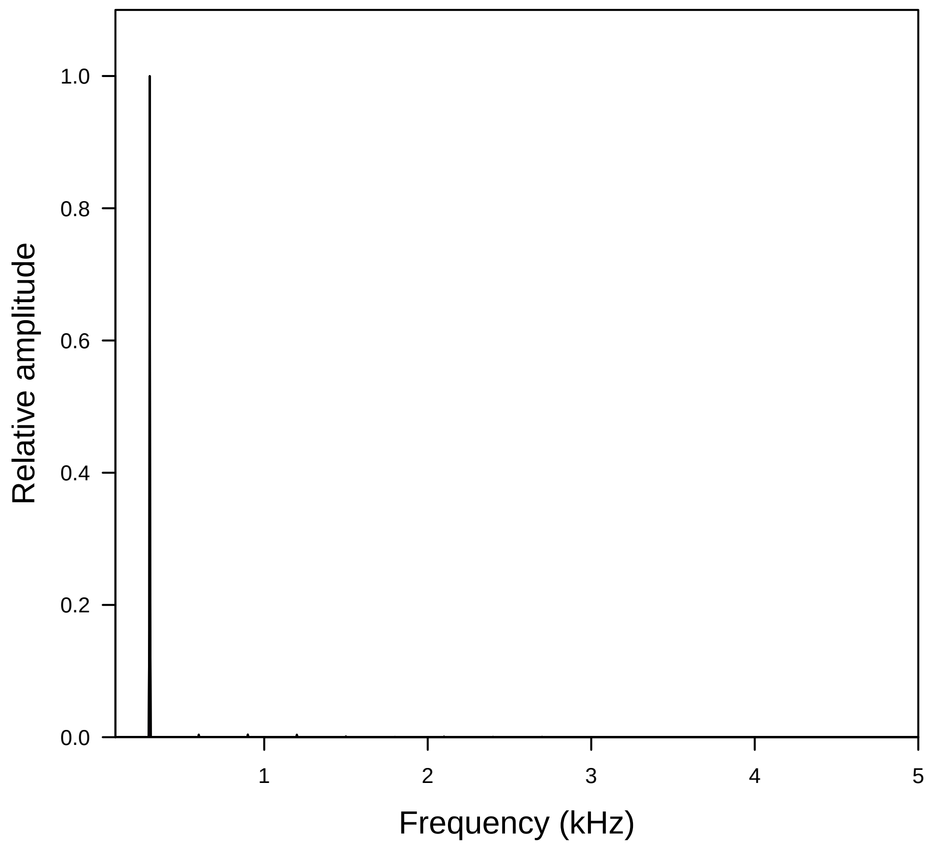

Vel. = 0.028 ; Str. = Receptacle ; Axis = y ; Fl. accession = 10−s−81−8

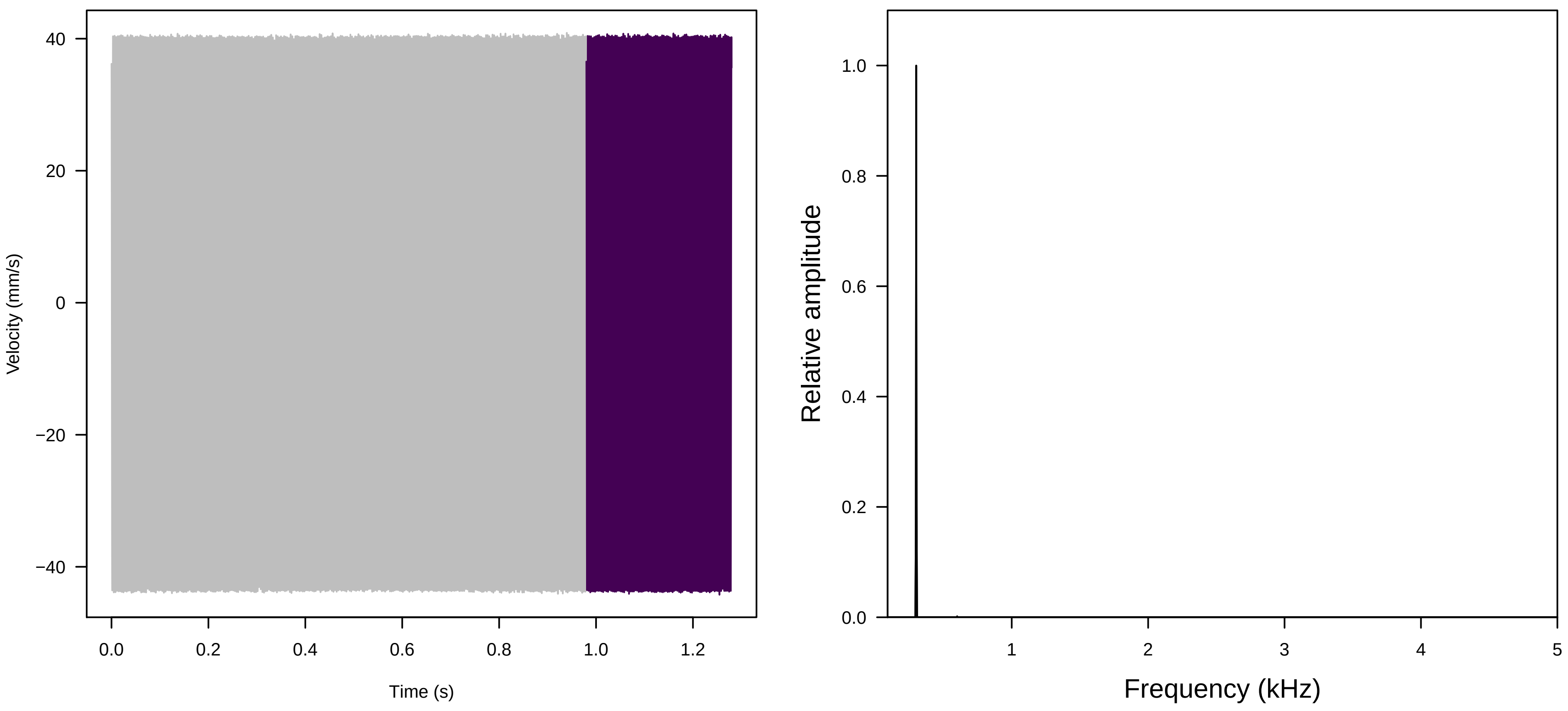

Vel. = 0.028 ; Str. = Corolla ; Axis = y ; Fl. accession = 10−s−81−8

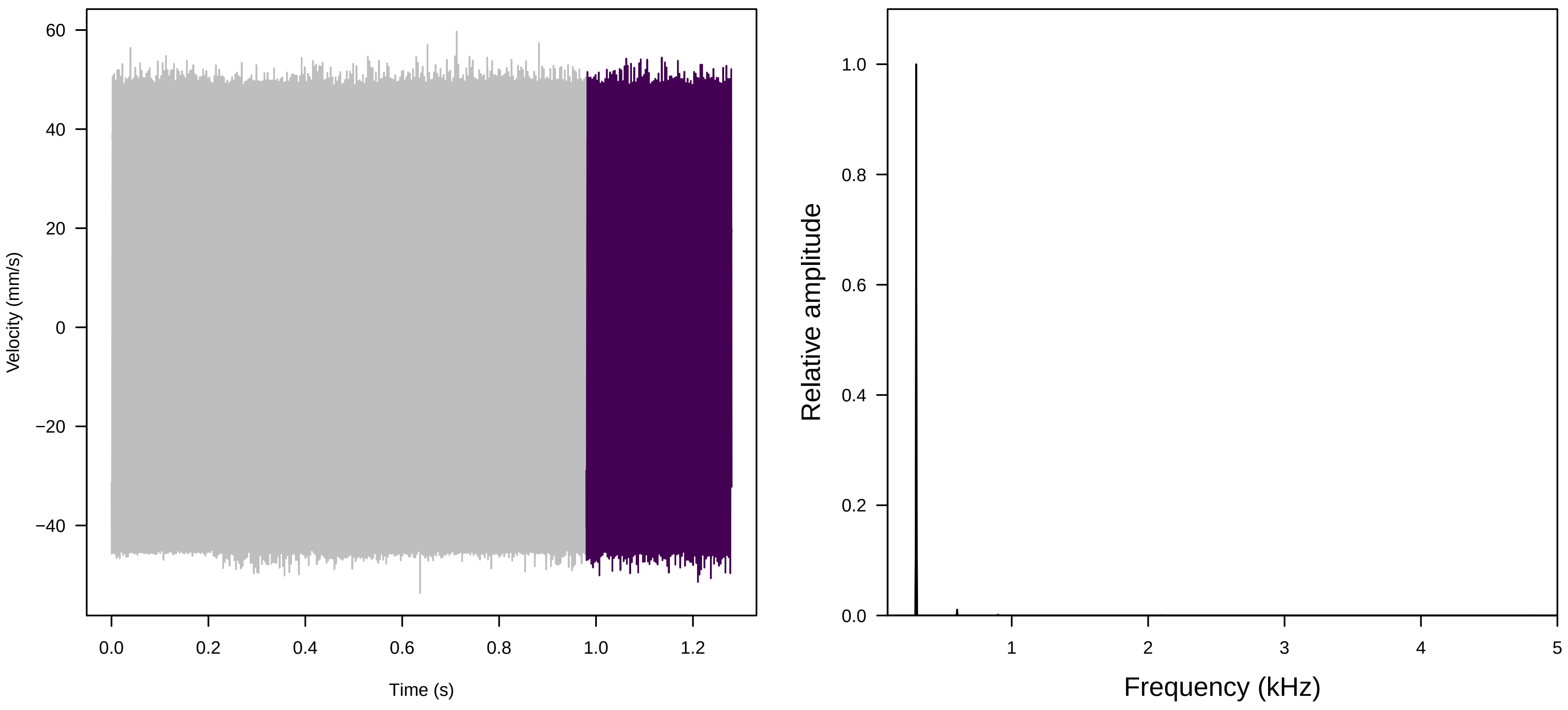

Vel. = 0.028 ; Str. = Receptacle ; Axis = y ; Fl. accession = 10−s−81−8

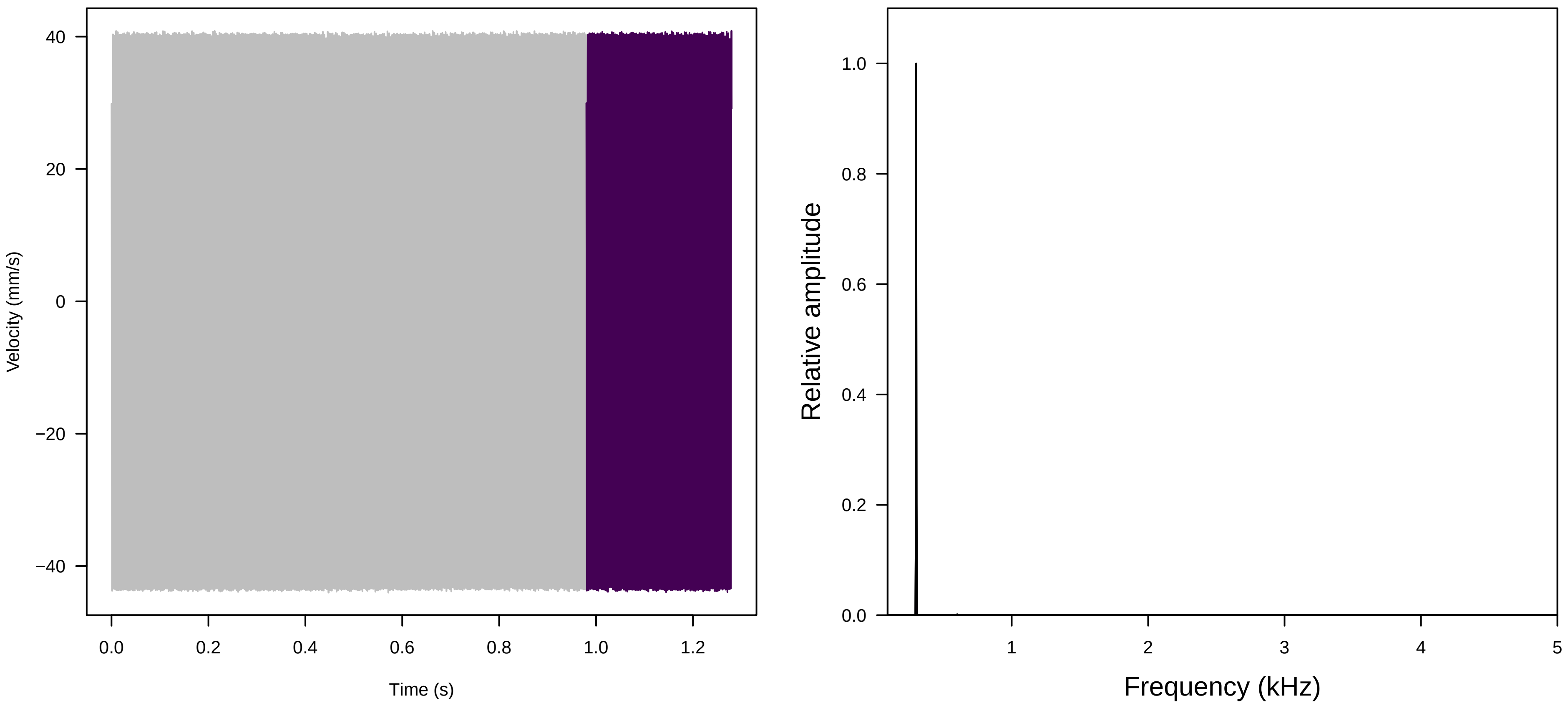

Vel. = 0.057 ; Str. = Corolla ; Axis = y ; Fl. accession = 10−s−81−8

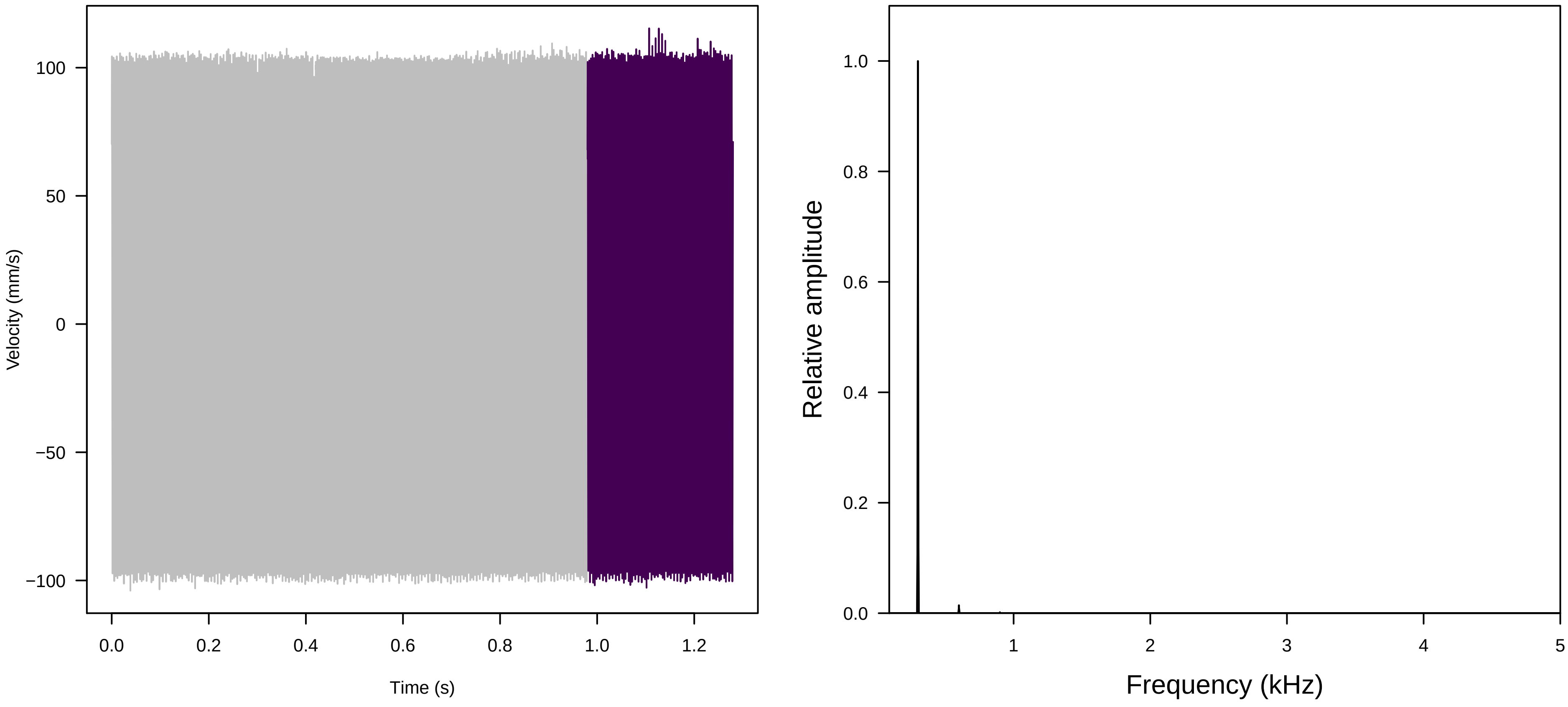

Vel. = 0.057 ; Str. = Receptacle ; Axis = y ; Fl. accession = 10−s−81−8

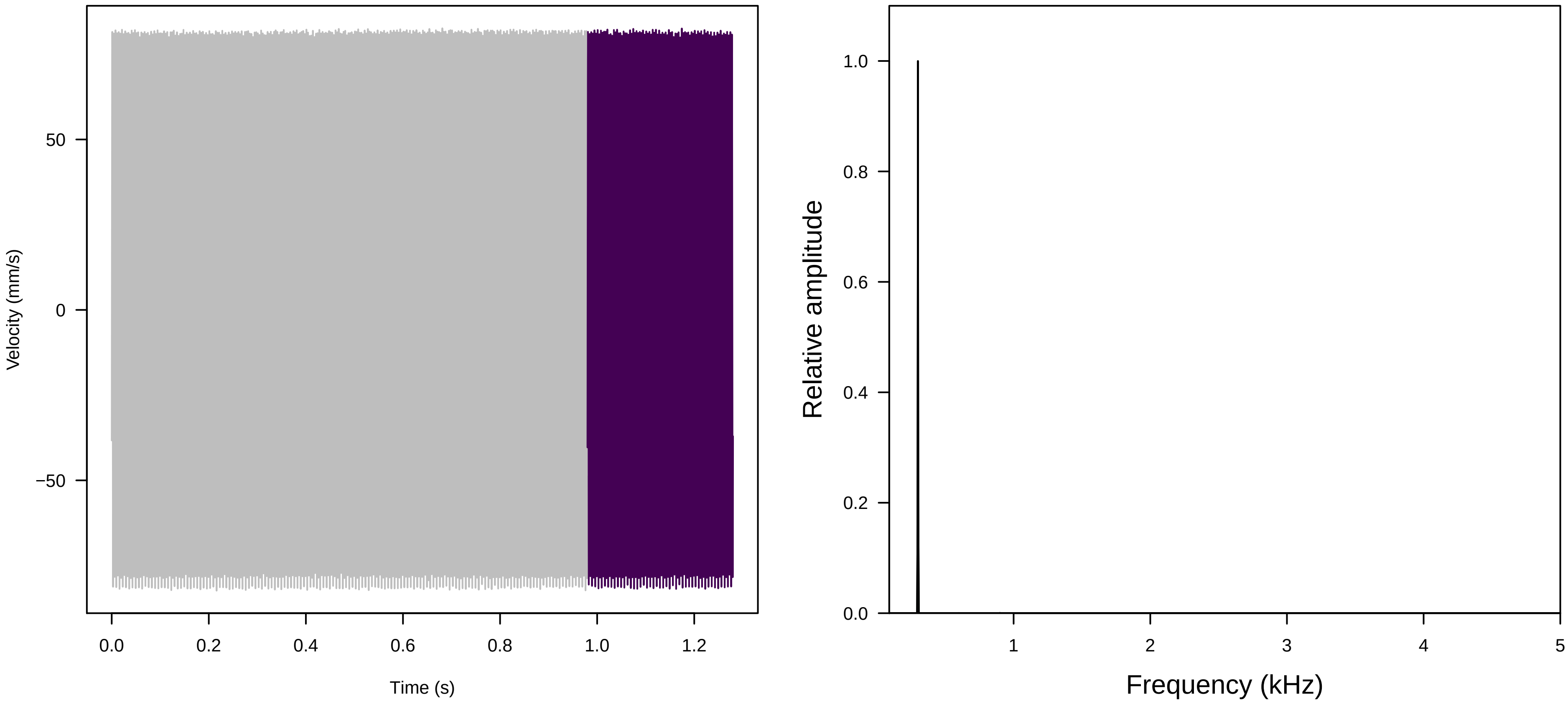

Vel. = 0.057 ; Str. = FA ; Axis = y ; Fl. accession = 10−s−81−8

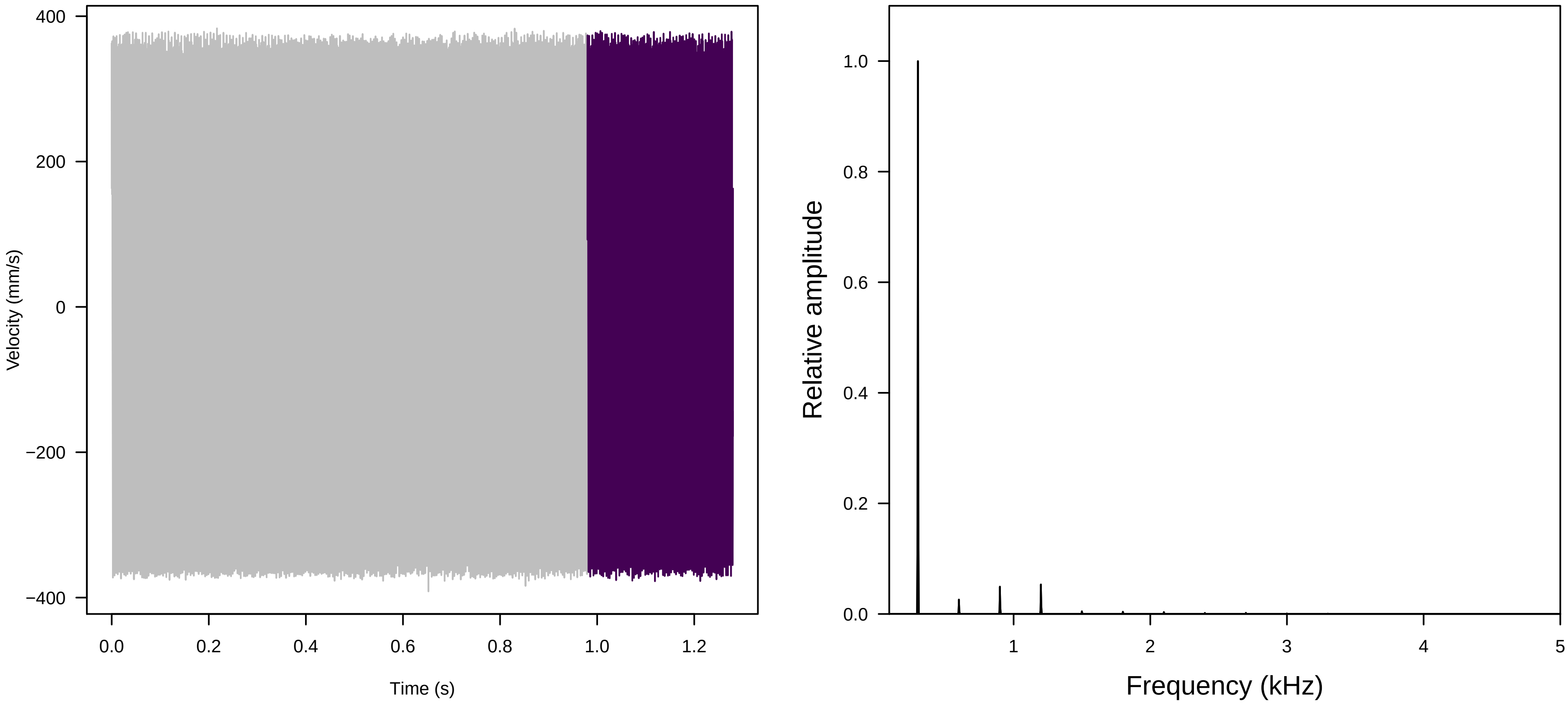

Vel. = 0.057 ; Str. = Receptacle ; Axis = y ; Fl. accession = 10−s−81−8

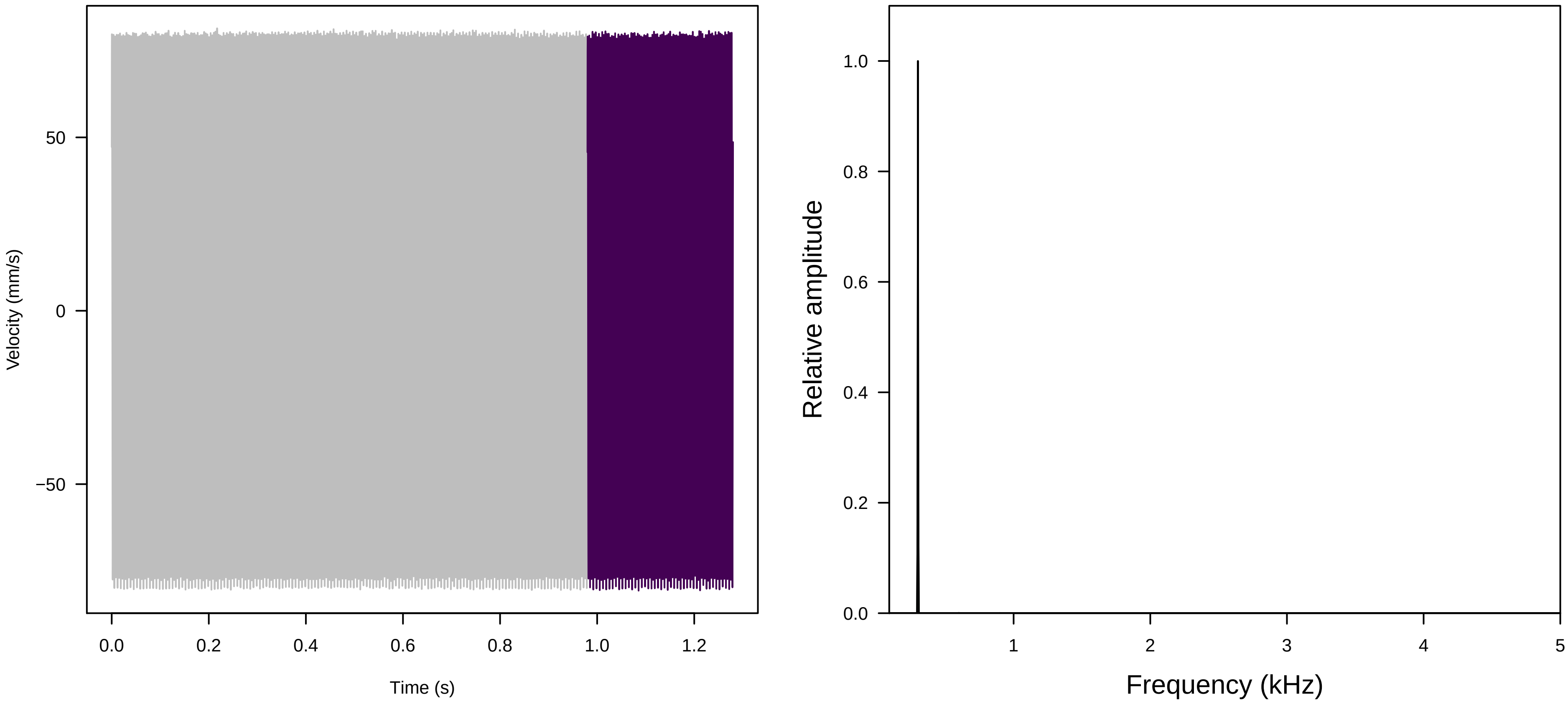

Vel. = 0.057 ; Str. = PA ; Axis = y ; Fl. accession = 10−s−81−8

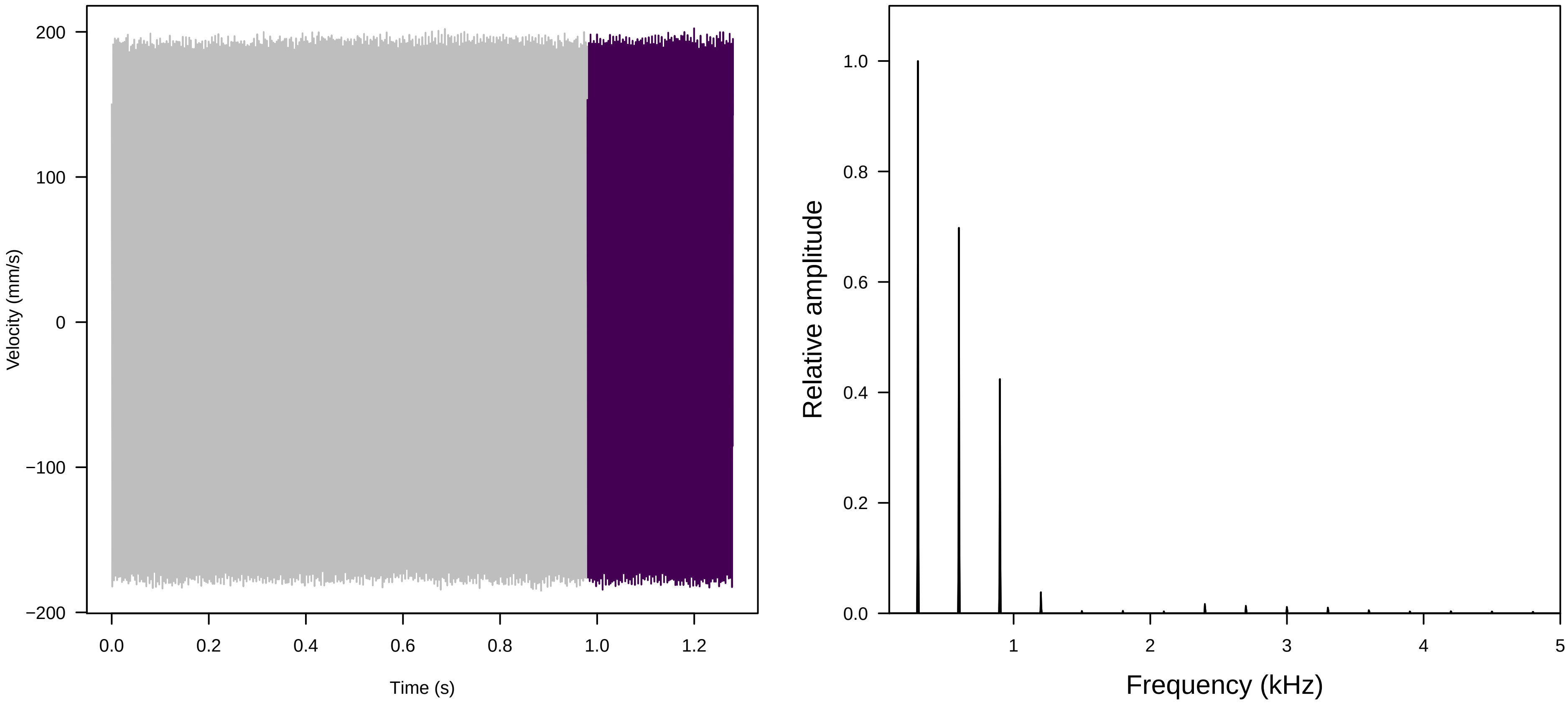

Vel. = 0.057 ; Str. = Receptacle ; Axis = y ; Fl. accession = 10−s−81−8

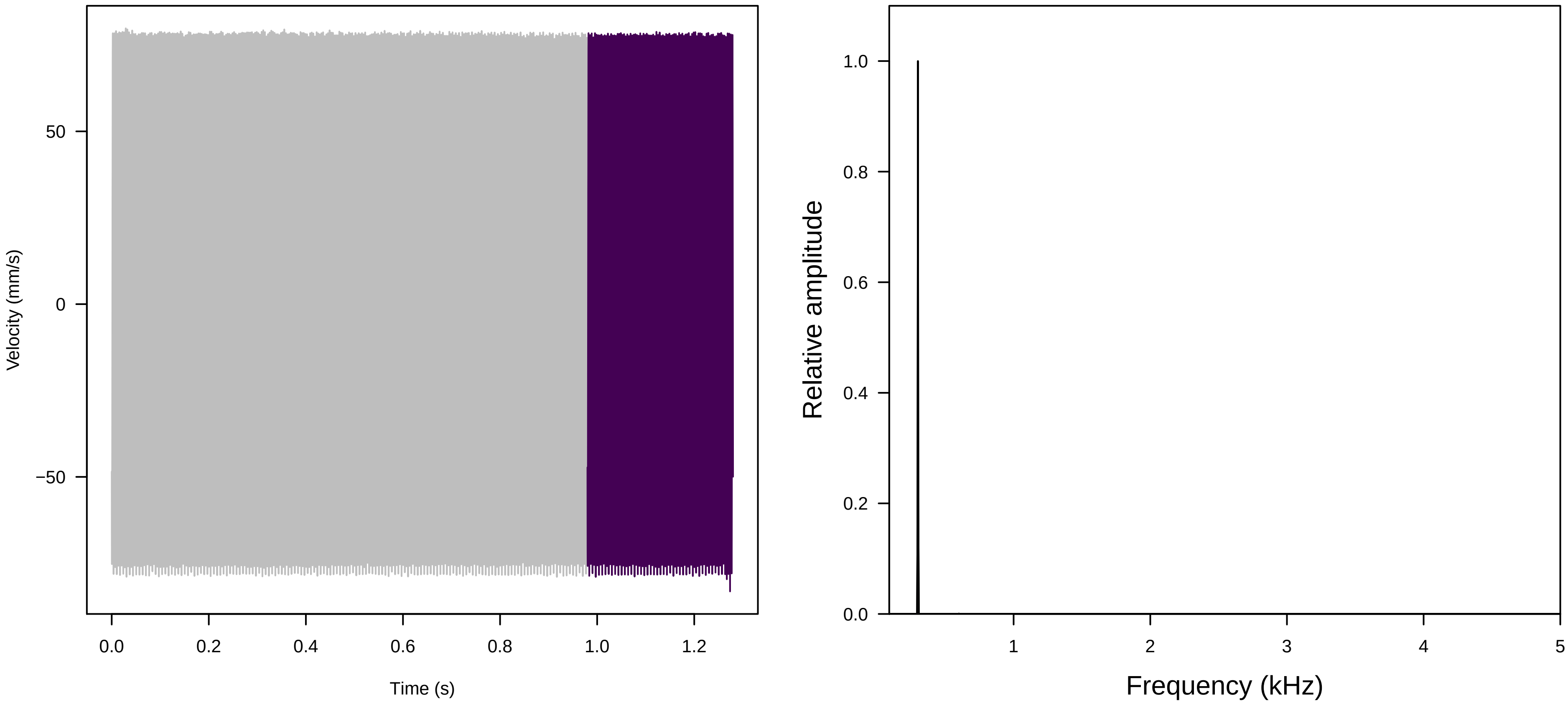

Vel. = 0.057 ; Str. = Corolla ; Axis = y ; Fl. accession = 10−s−79−2

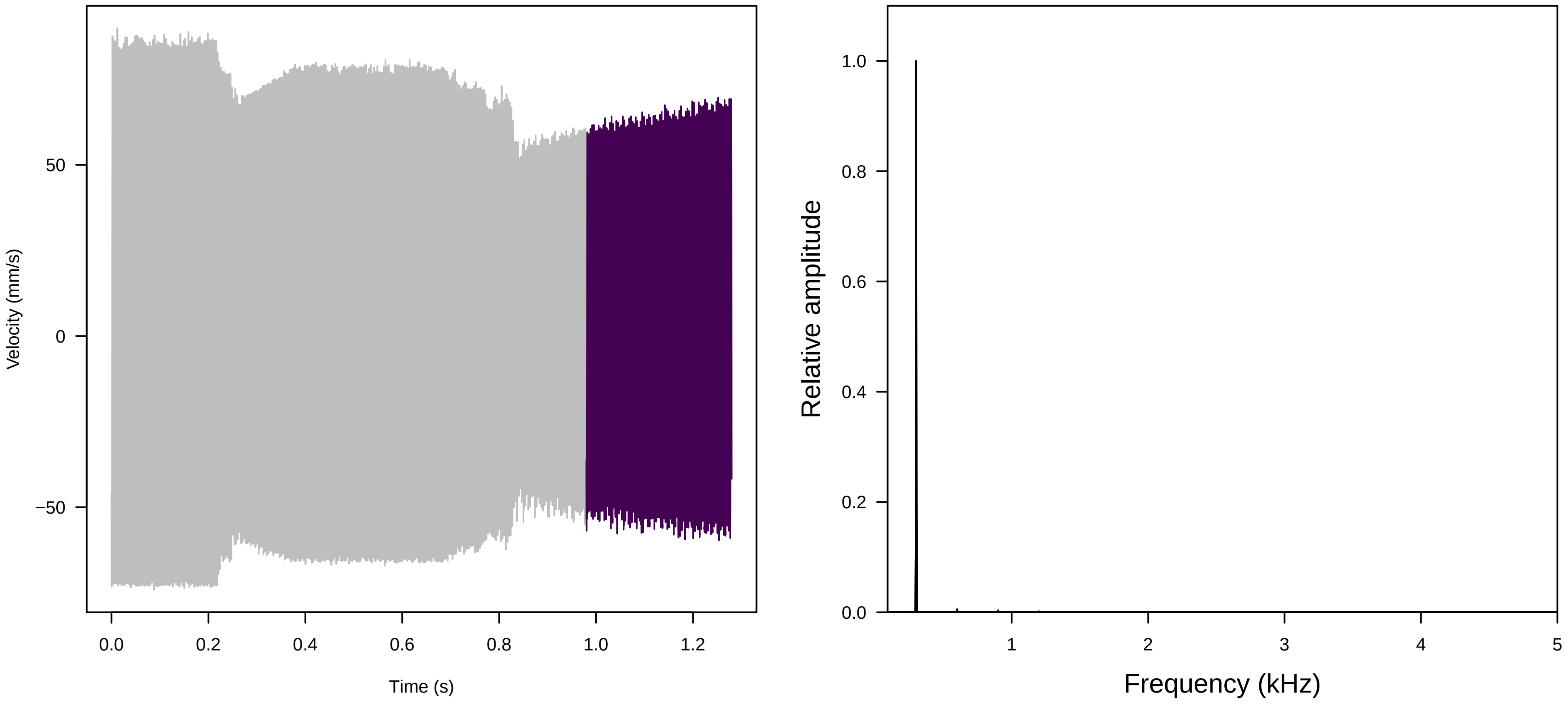

Vel. = 0.057 ; Str. = Receptacle ; Axis = y ; Fl. accession = 10−s−79−2

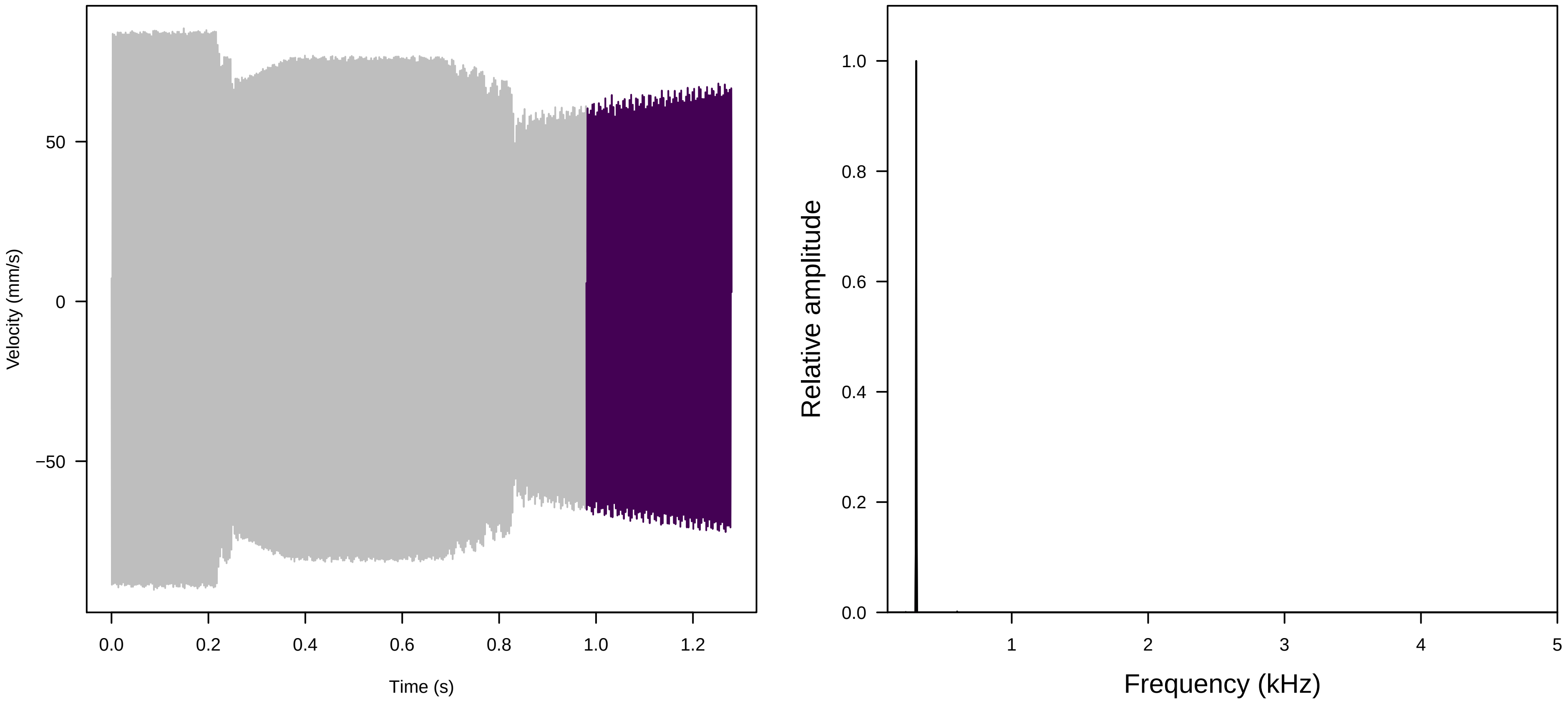

Vel. = 0.057 ; Str. = FA ; Axis = y ; Fl. accession = 10−s−79−2

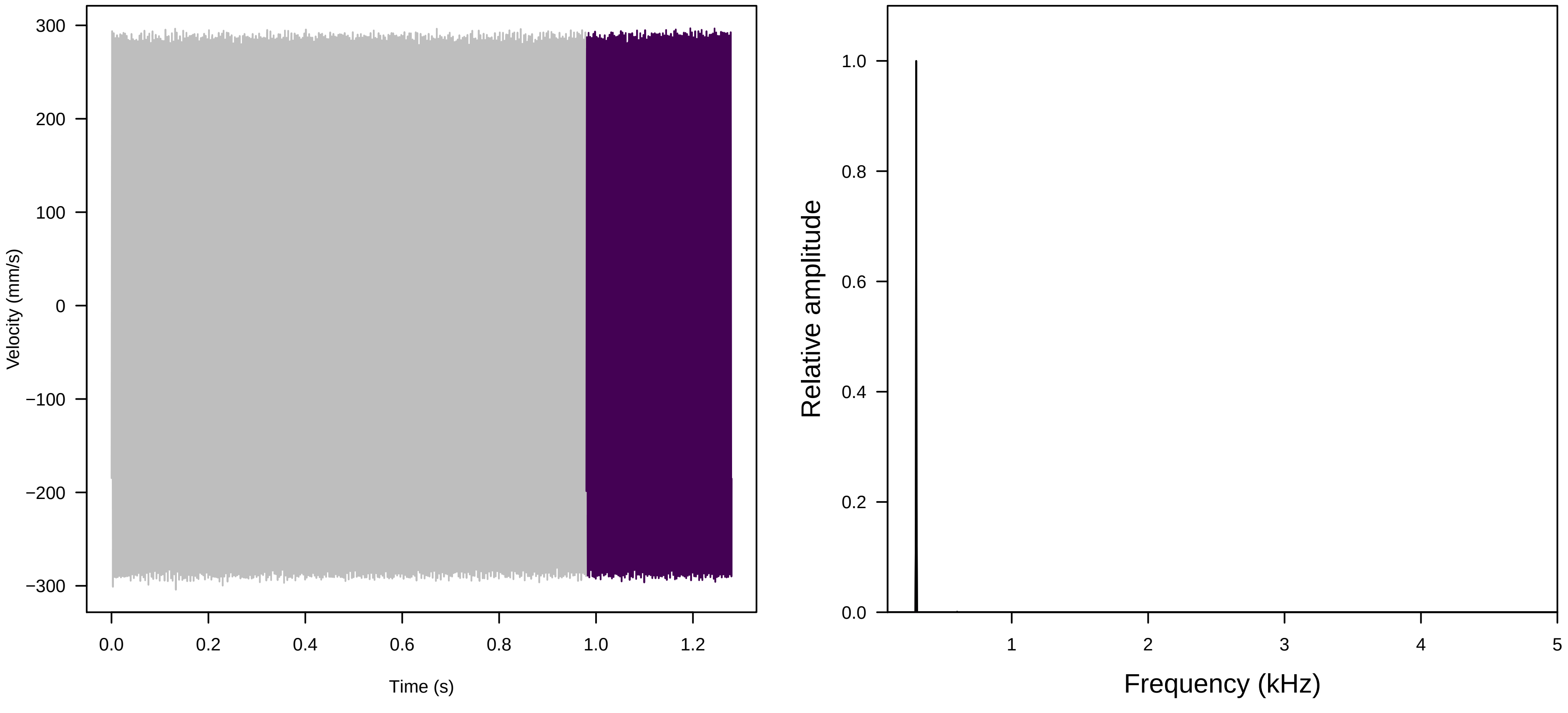

Vel. = 0.057 ; Str. = Receptacle ; Axis = y ; Fl. accession = 10−s−79−2

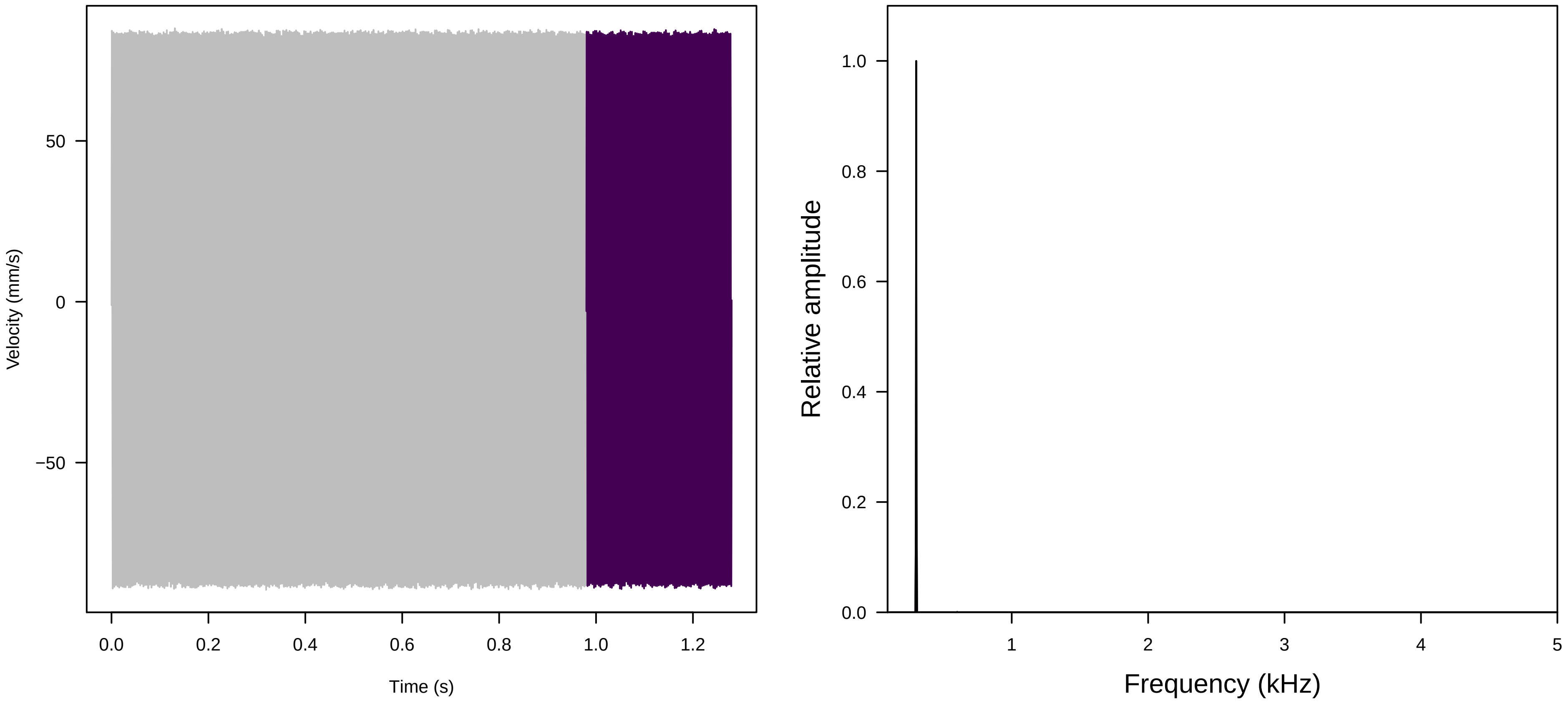

Vel. = 0.057 ; Str. = PA ; Axis = y ; Fl. accession = 10−s−79−2

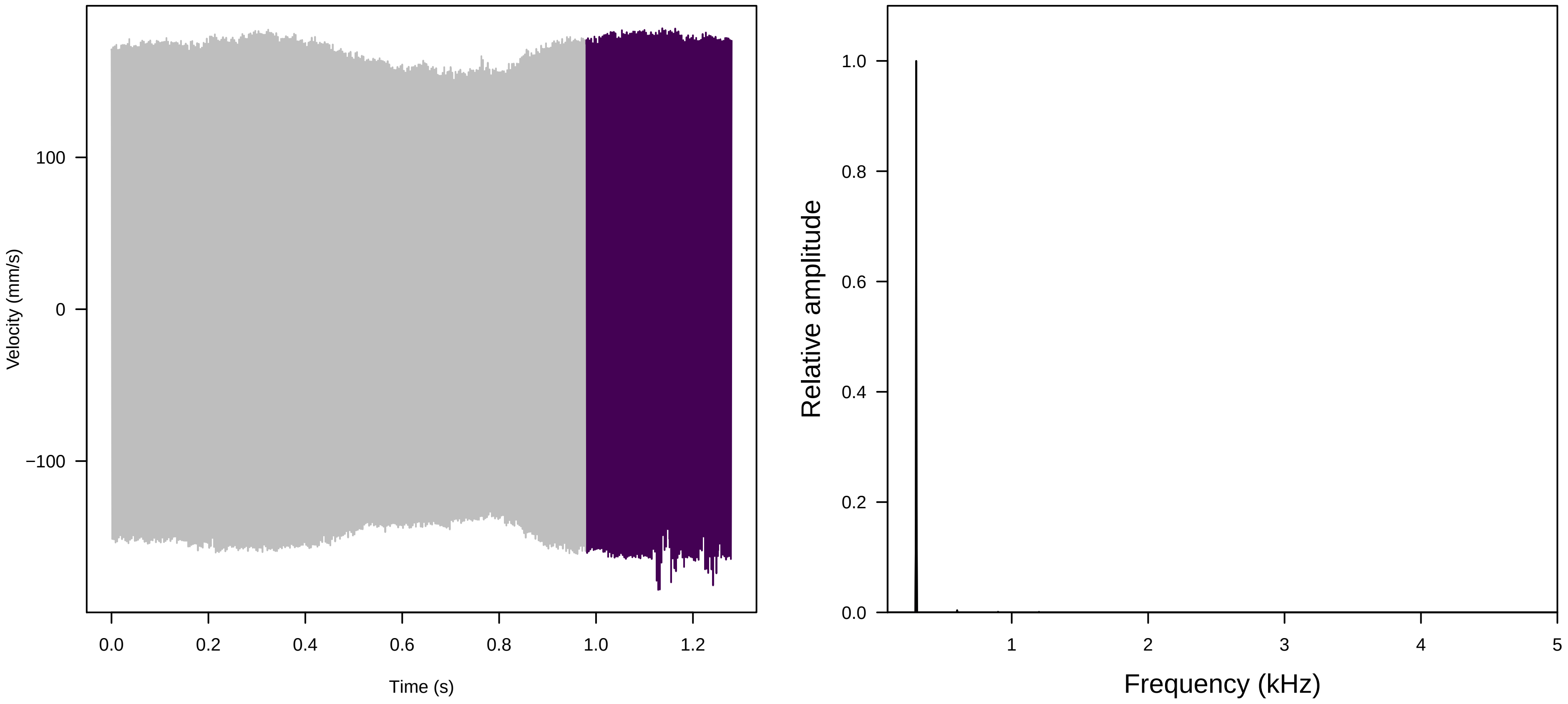

Vel. = 0.057 ; Str. = Receptacle ; Axis = y ; Fl. accession = 10−s−79−2

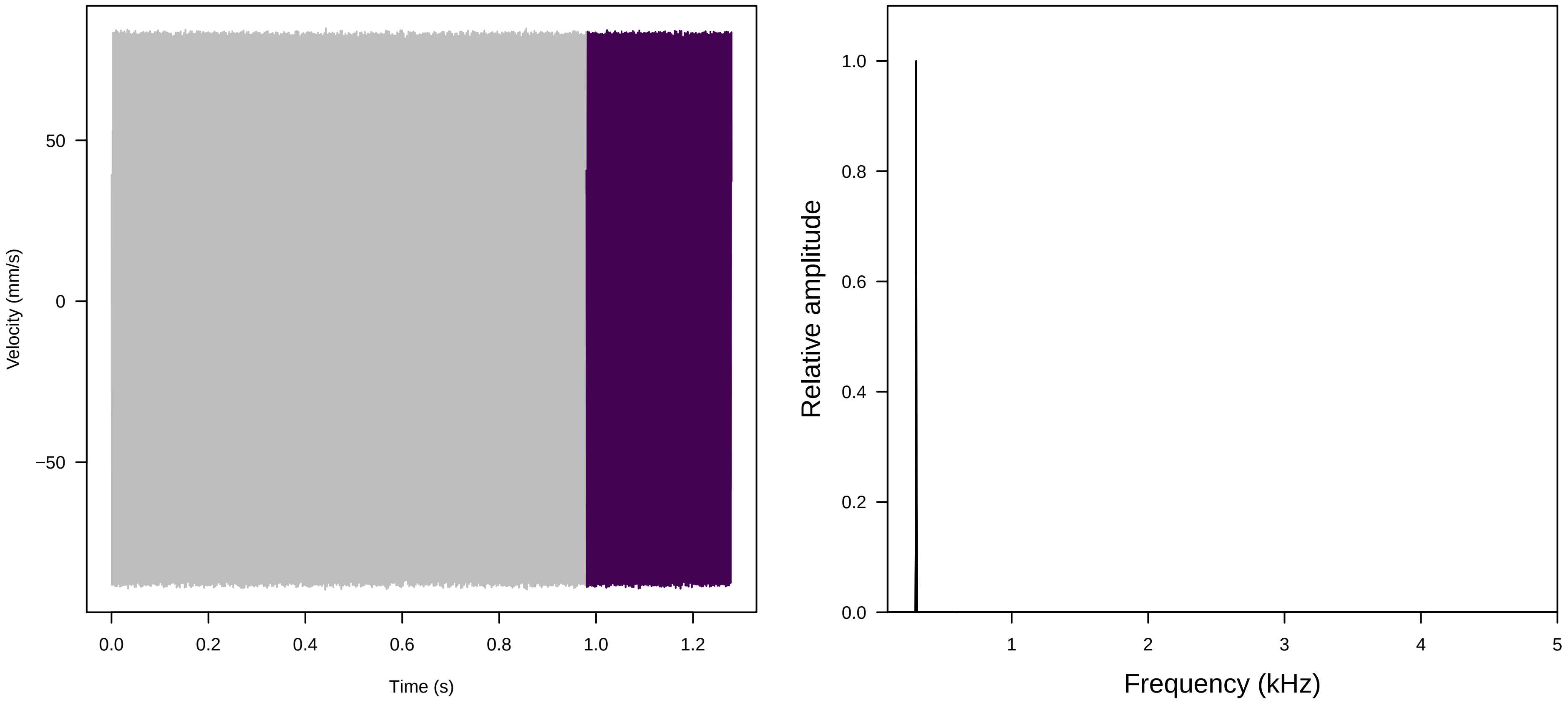

Vel. = 0.028 ; Str. = PA ; Axis = y ; Fl. accession = 10−s−79−2

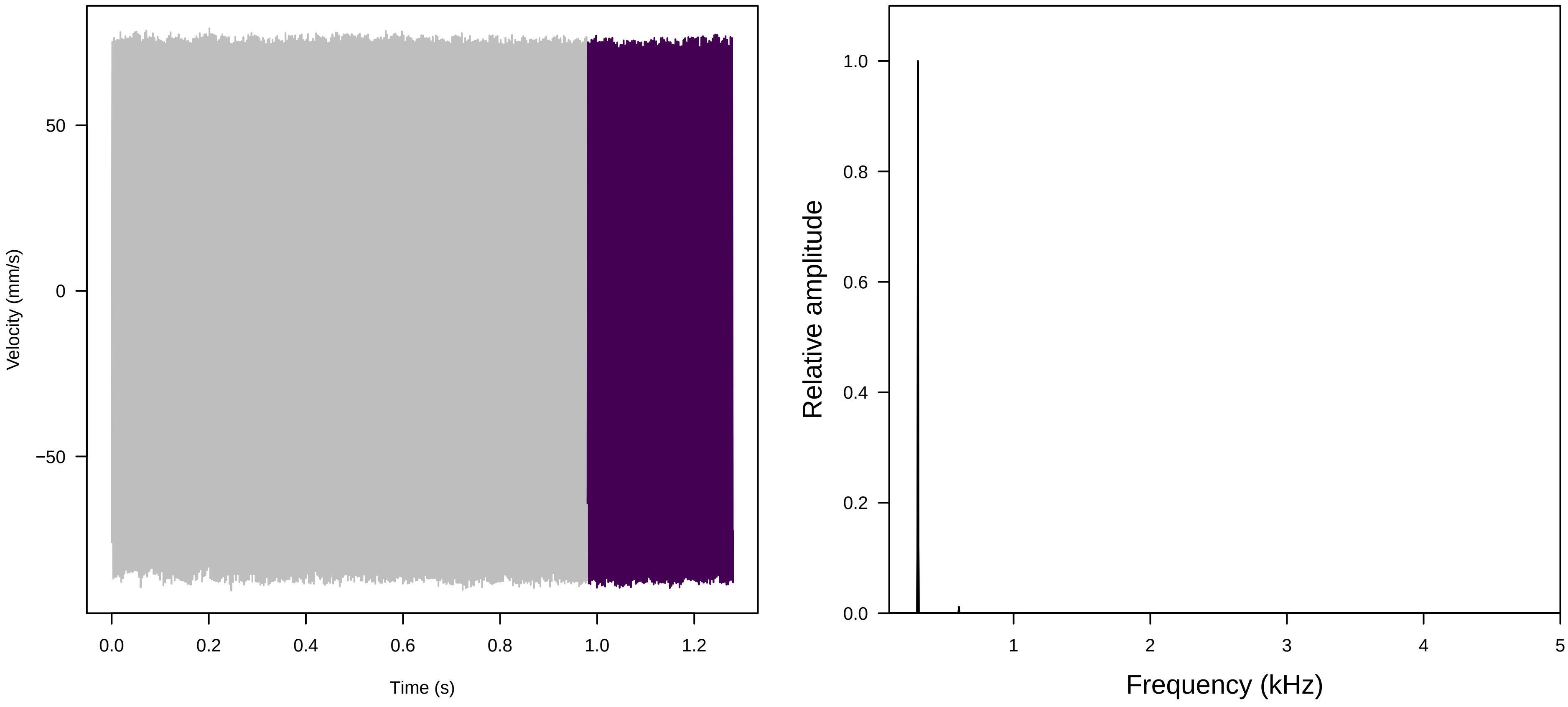

Vel. = 0.028 ; Str. = Receptacle ; Axis = y ; Fl. accession = 10−s−79−2

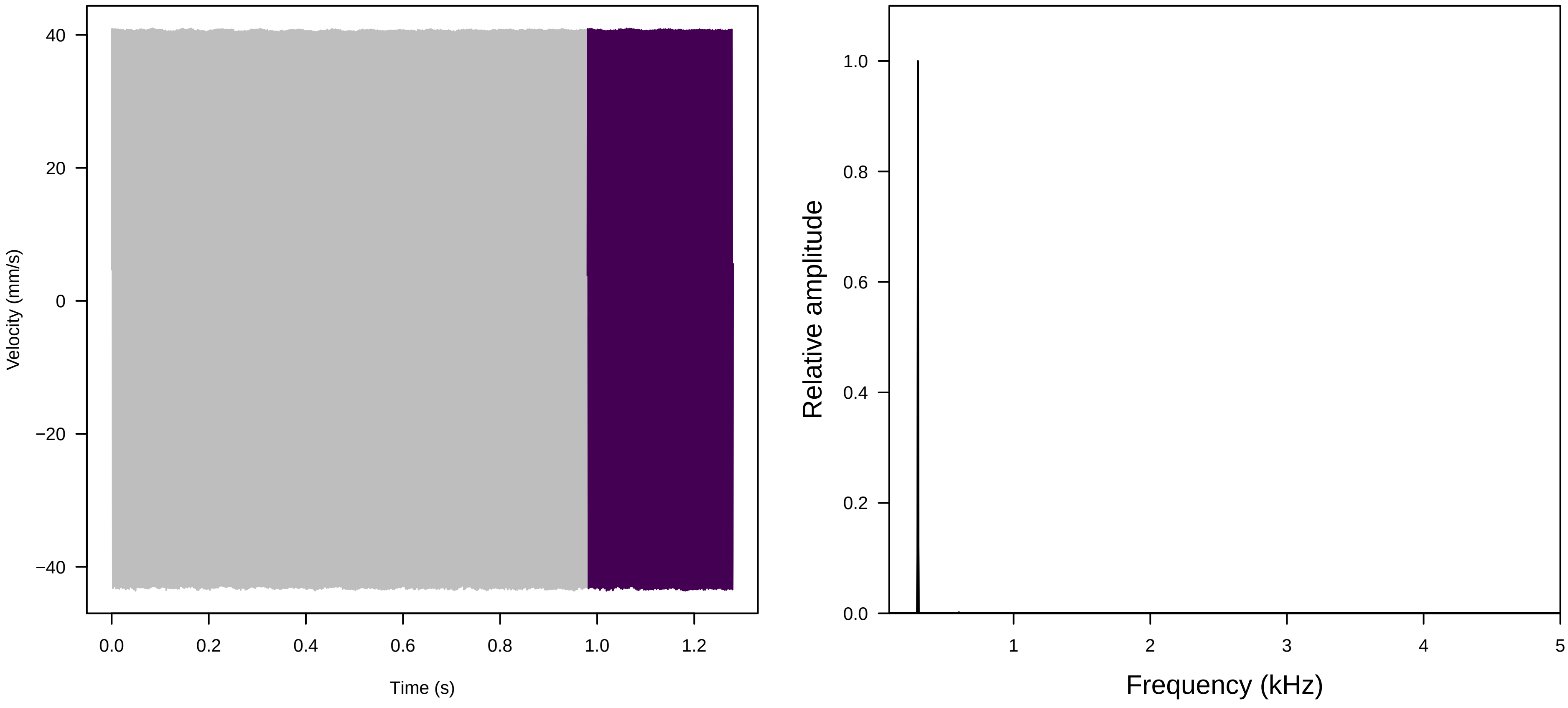

Vel. = 0.028 ; Str. = FA ; Axis = y ; Fl. accession = 10−s−79−2

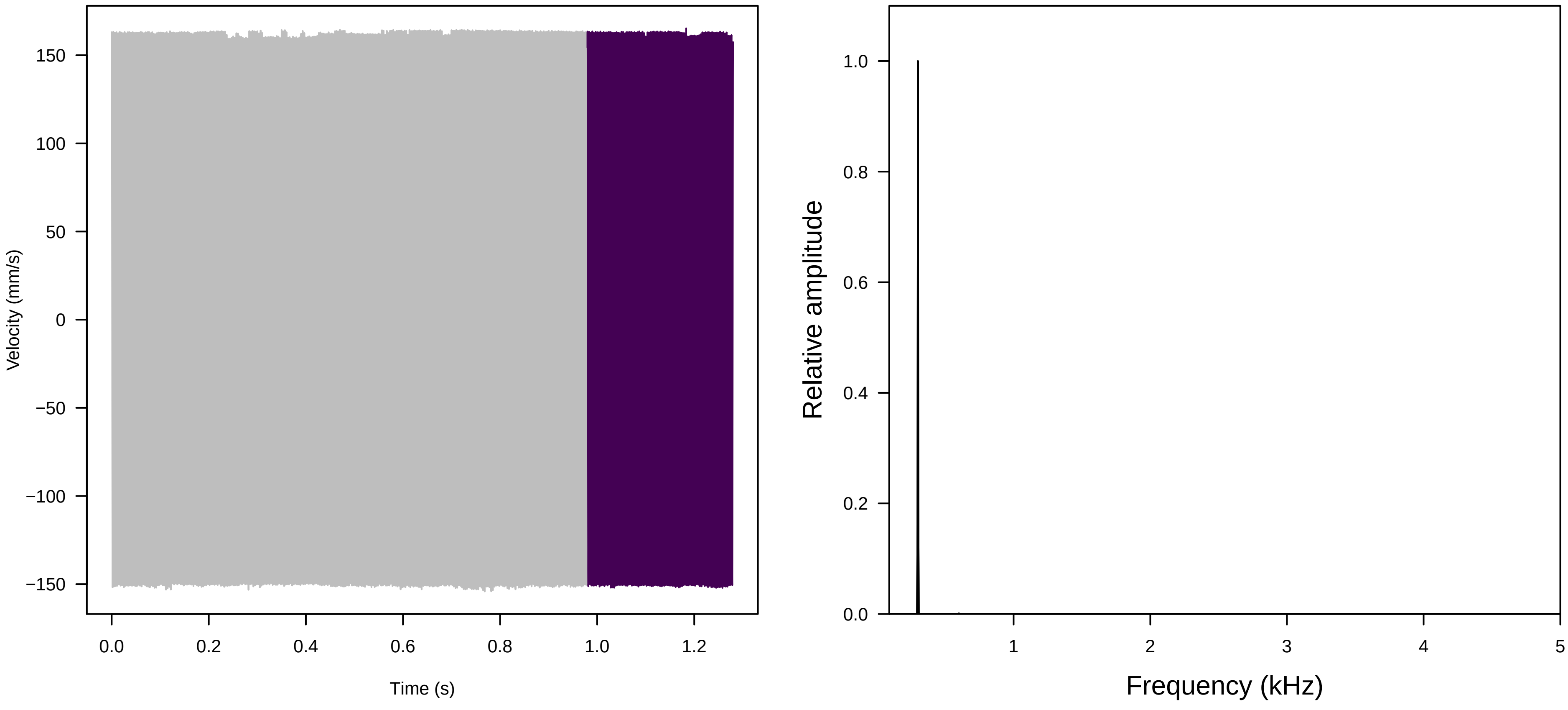

Vel. = 0.028 ; Str. = Receptacle ; Axis = y ; Fl. accession = 10−s−79−2

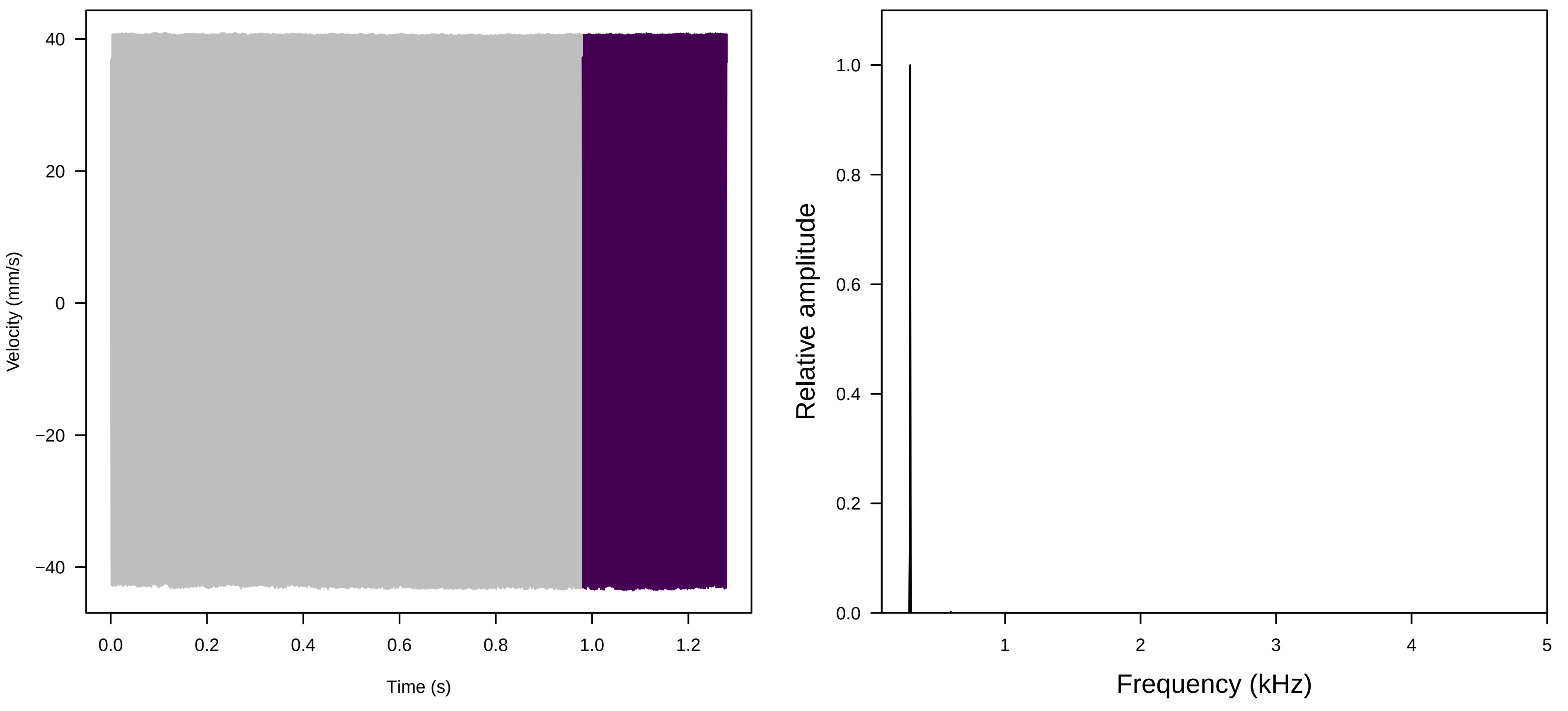

Vel. = 0.028 ; Str. = Corolla ; Axis = y ; Fl. accession = 10−s−79−2

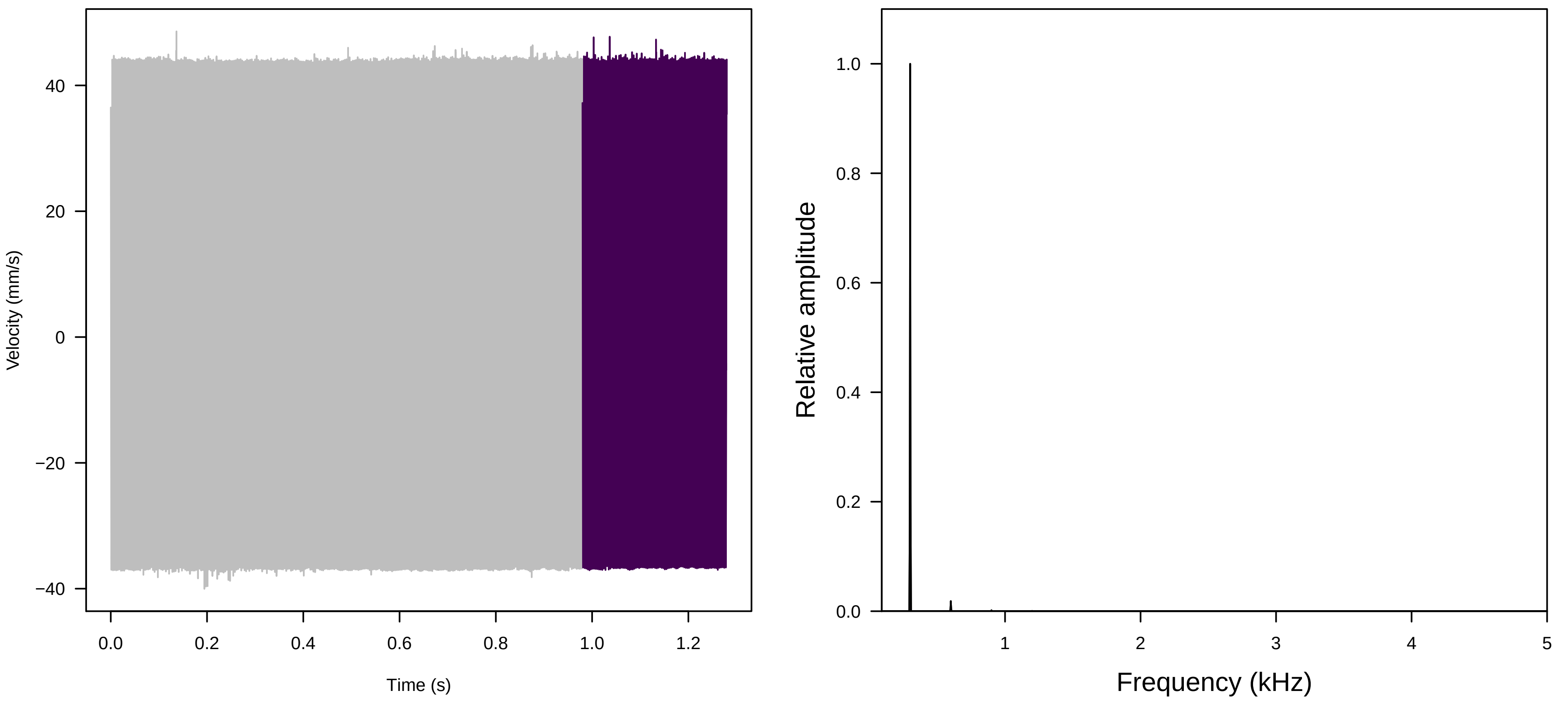

Vel. = 0.028 ; Str. = Receptacle ; Axis = y ; Fl. accession = 10−s−79−2

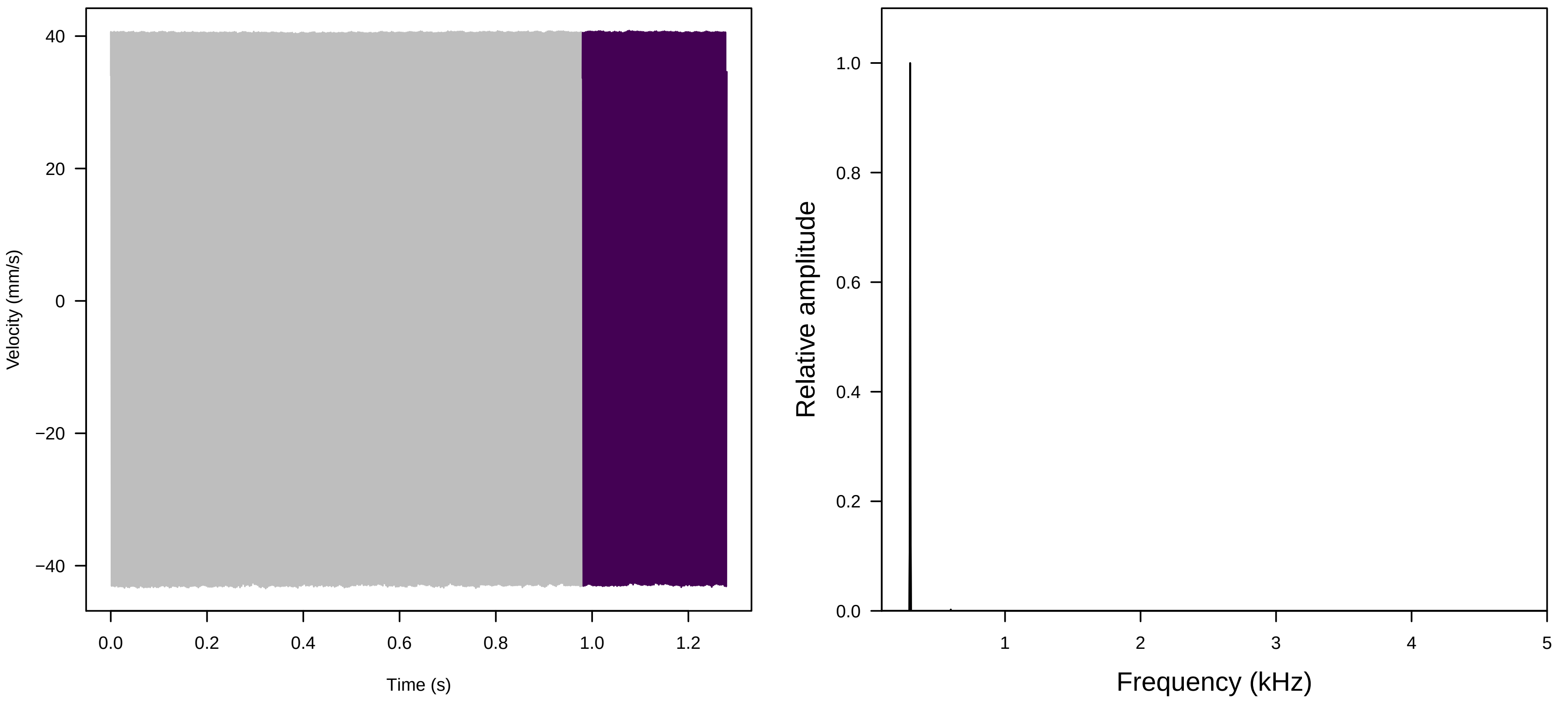

Vel. = 0.014 ; Str. = Corolla ; Axis = y ; Fl. accession = 10−s−79−2

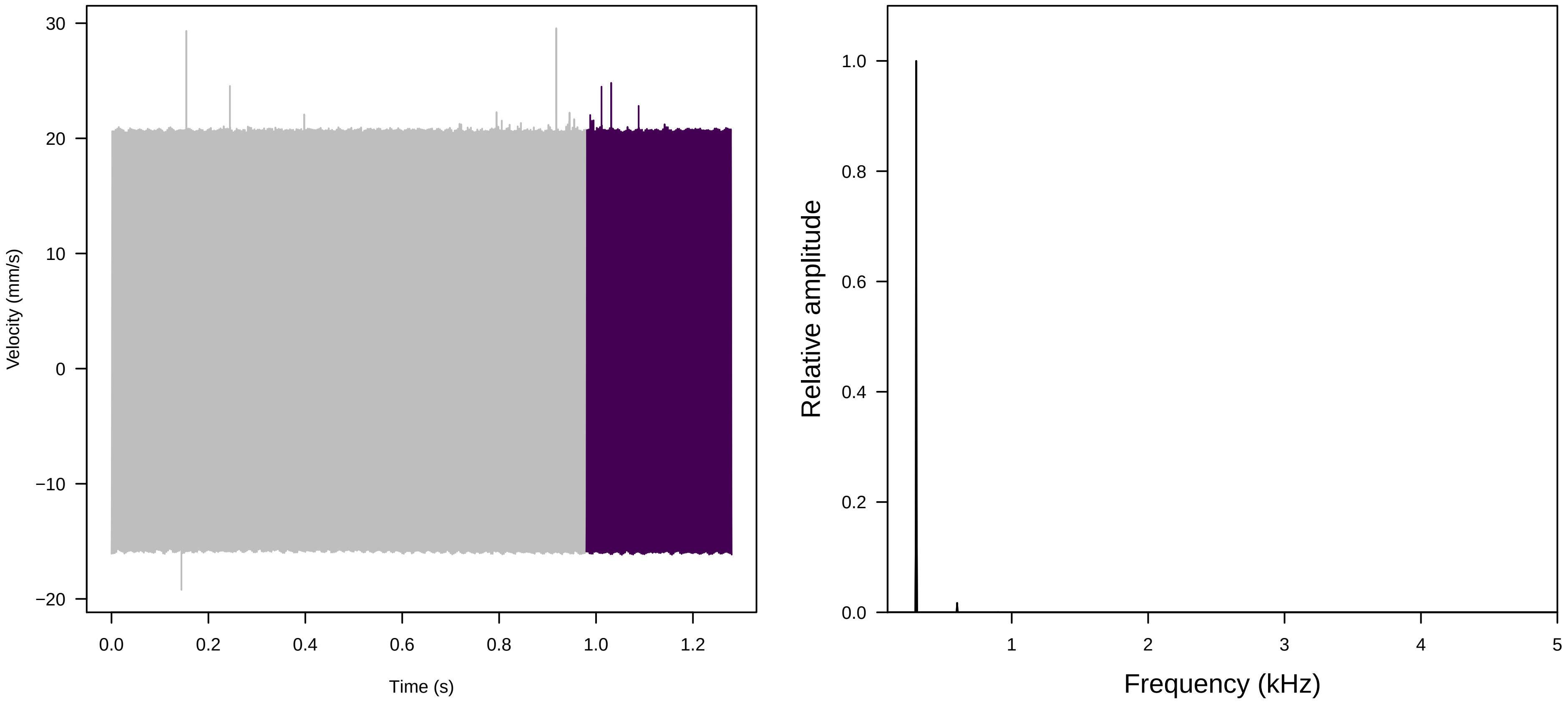

Vel. = 0.014 ; Str. = Receptacle ; Axis = y ; Fl. accession = 10−s−79−2

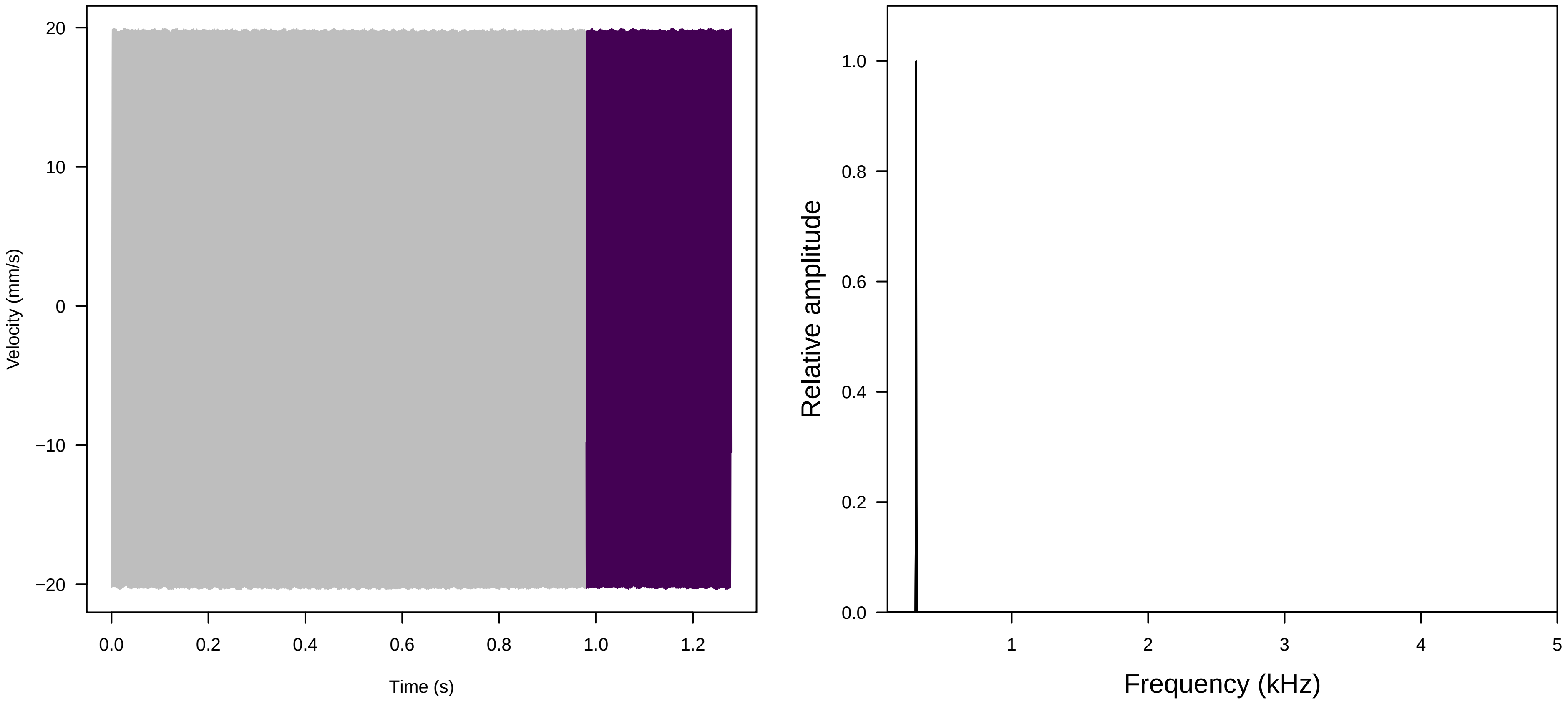

Vel. = 0.014 ; Str. = FA ; Axis = y ; Fl. accession = 10−s−79−2

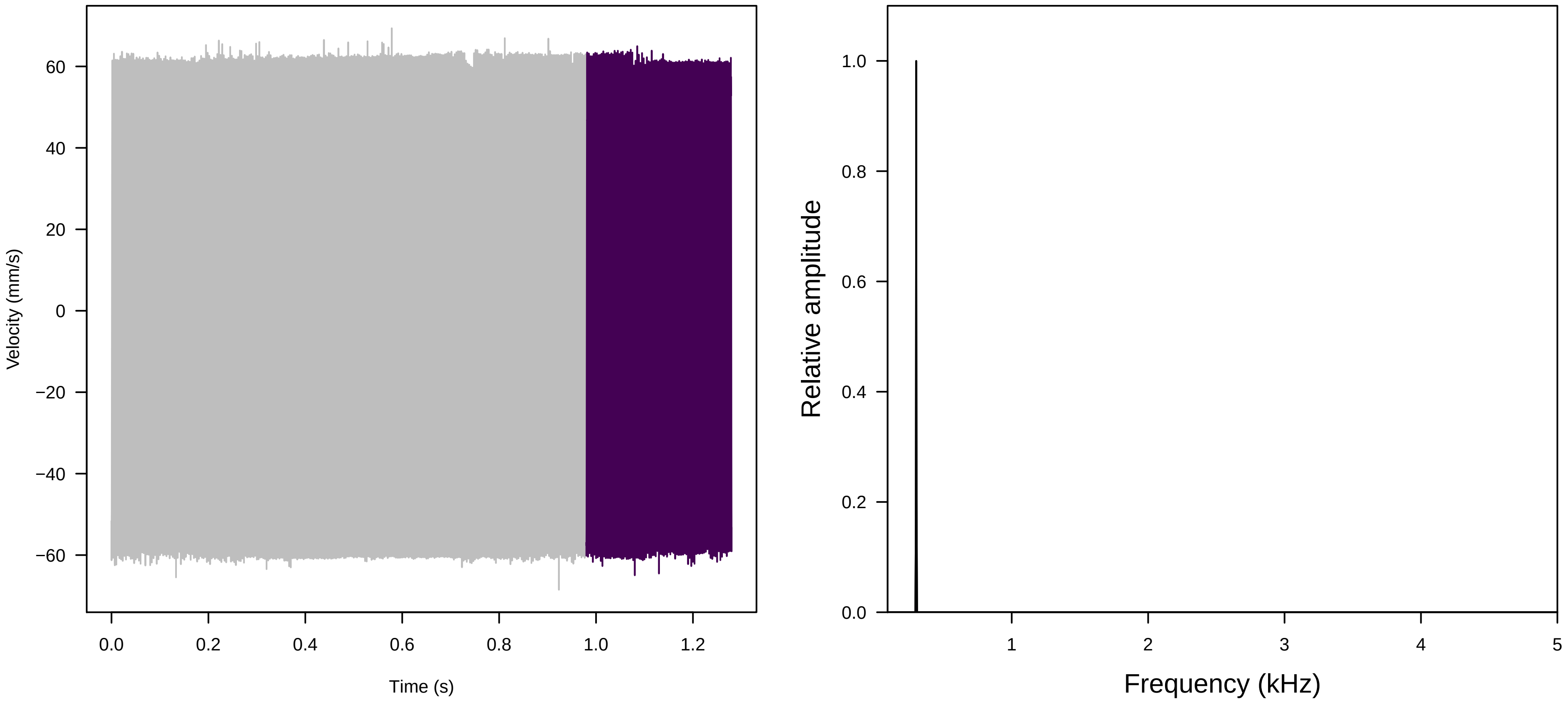

Vel. = 0.014 ; Str. = Receptacle ; Axis = y ; Fl. accession = 10−s−79−2

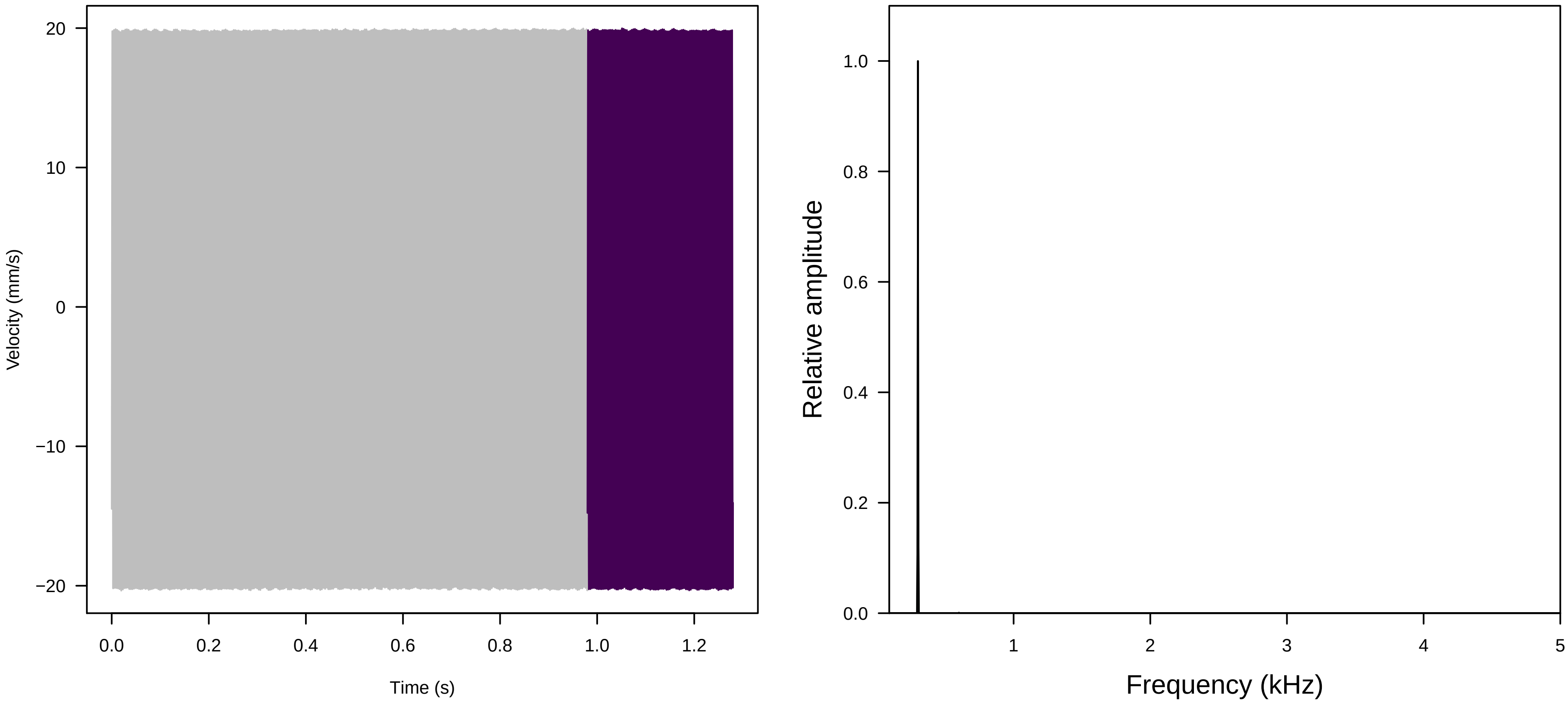

Vel. = 0.014 ; Str. = PA ; Axis = y ; Fl. accession = 10−s−79−2

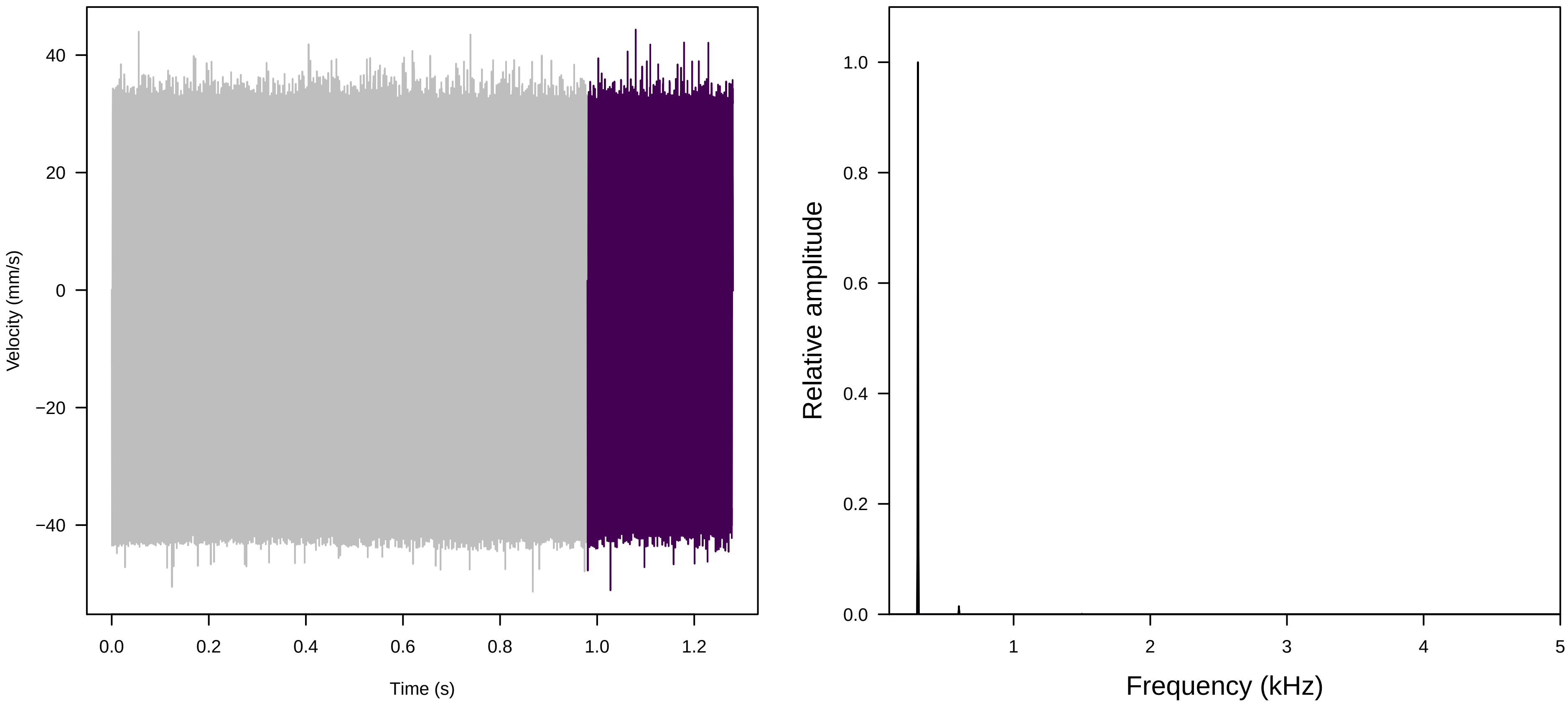

Vel. = 0.014 ; Str. = Receptacle ; Axis = y ; Fl. accession = 10−s−79−2

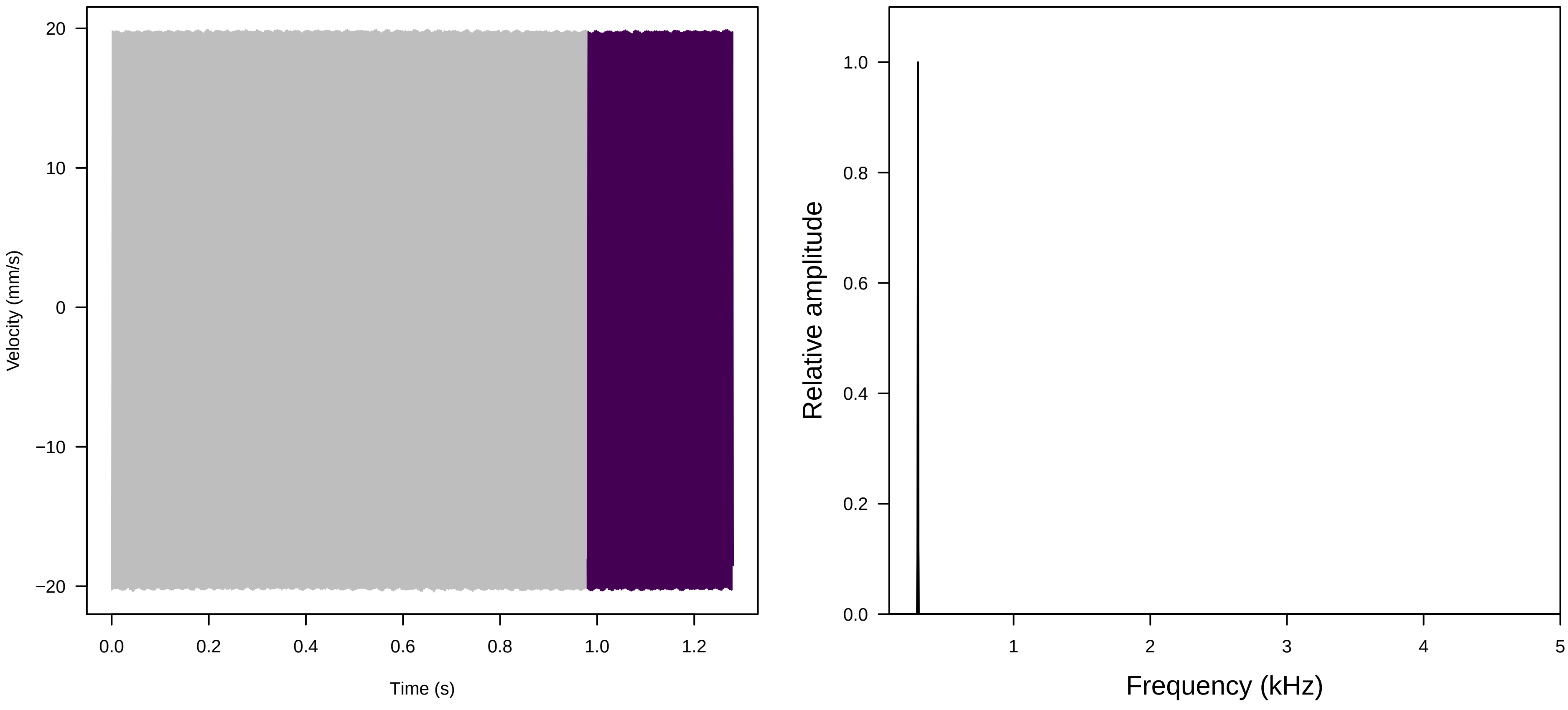

Vel. = 0.014 ; Str. = Corolla ; Axis = z ; Fl. accession = 10−s−79−2

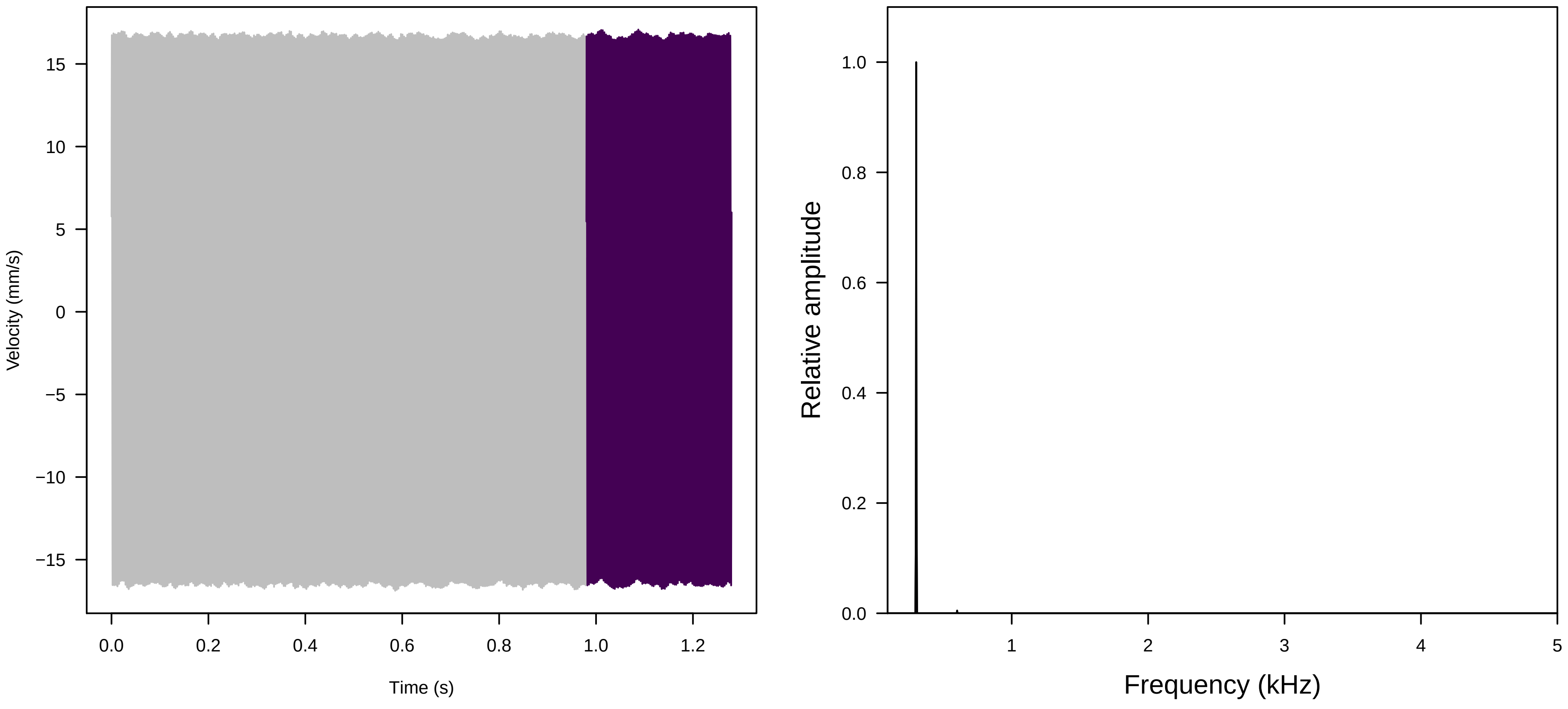

Vel. = 0.014 ; Str. = Receptacle ; Axis = z ; Fl. accession = 10−s−79−2

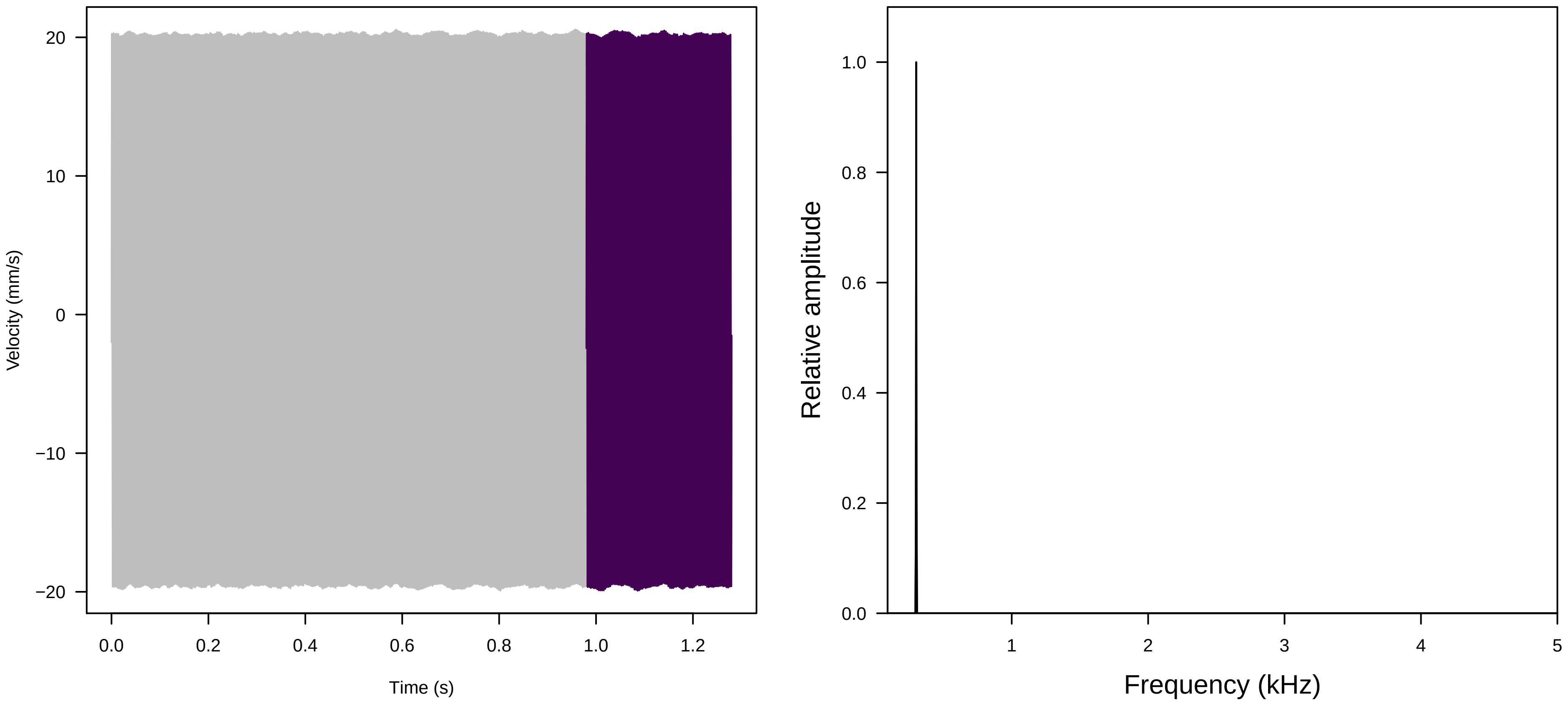

Vel. = 0.014 ; Str. = FA ; Axis = z ; Fl. accession = 10−s−79−2

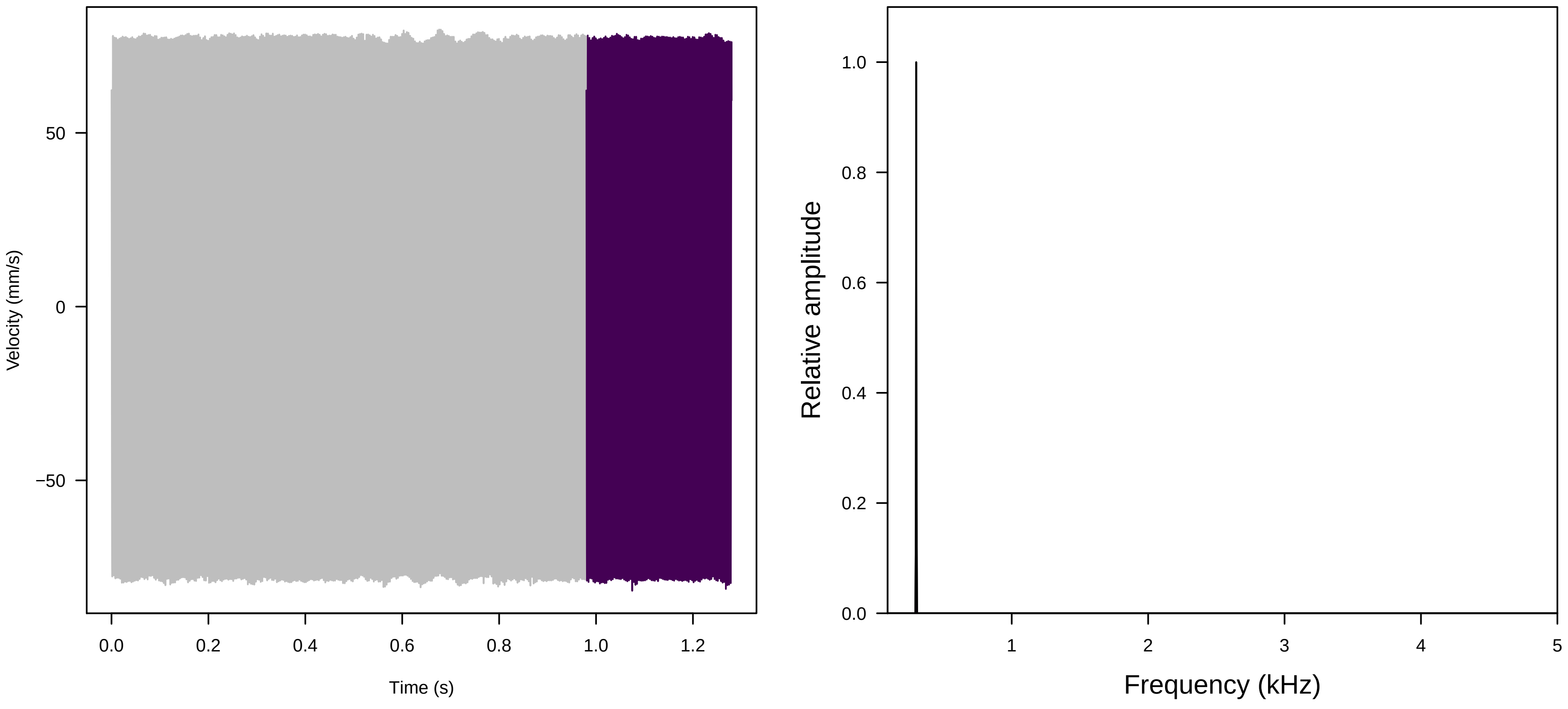

Vel. = 0.014 ; Str. = Receptacle ; Axis = z ; Fl. accession = 10−s−79−2

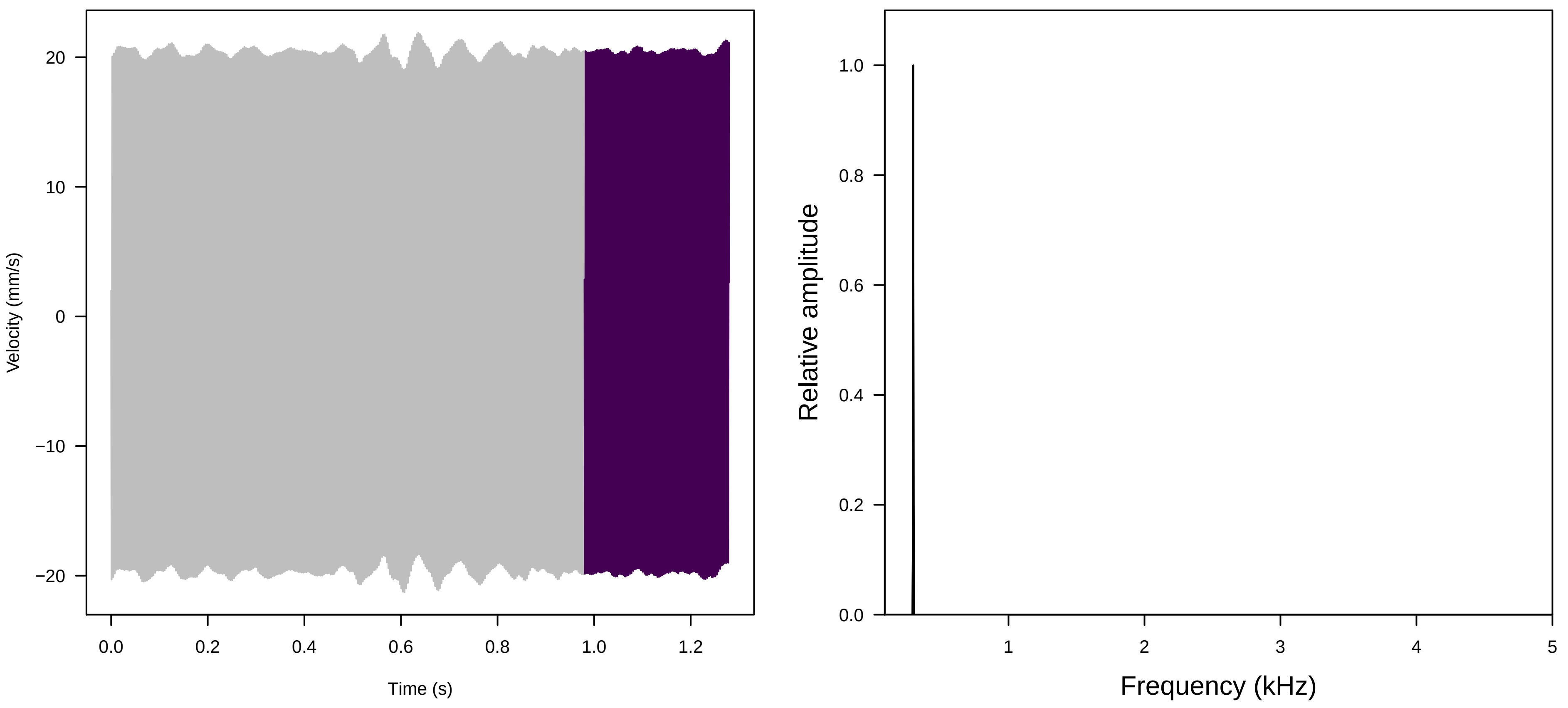

Vel. = 0.014 ; Str. = PA ; Axis = z ; Fl. accession = 10−s−79−2

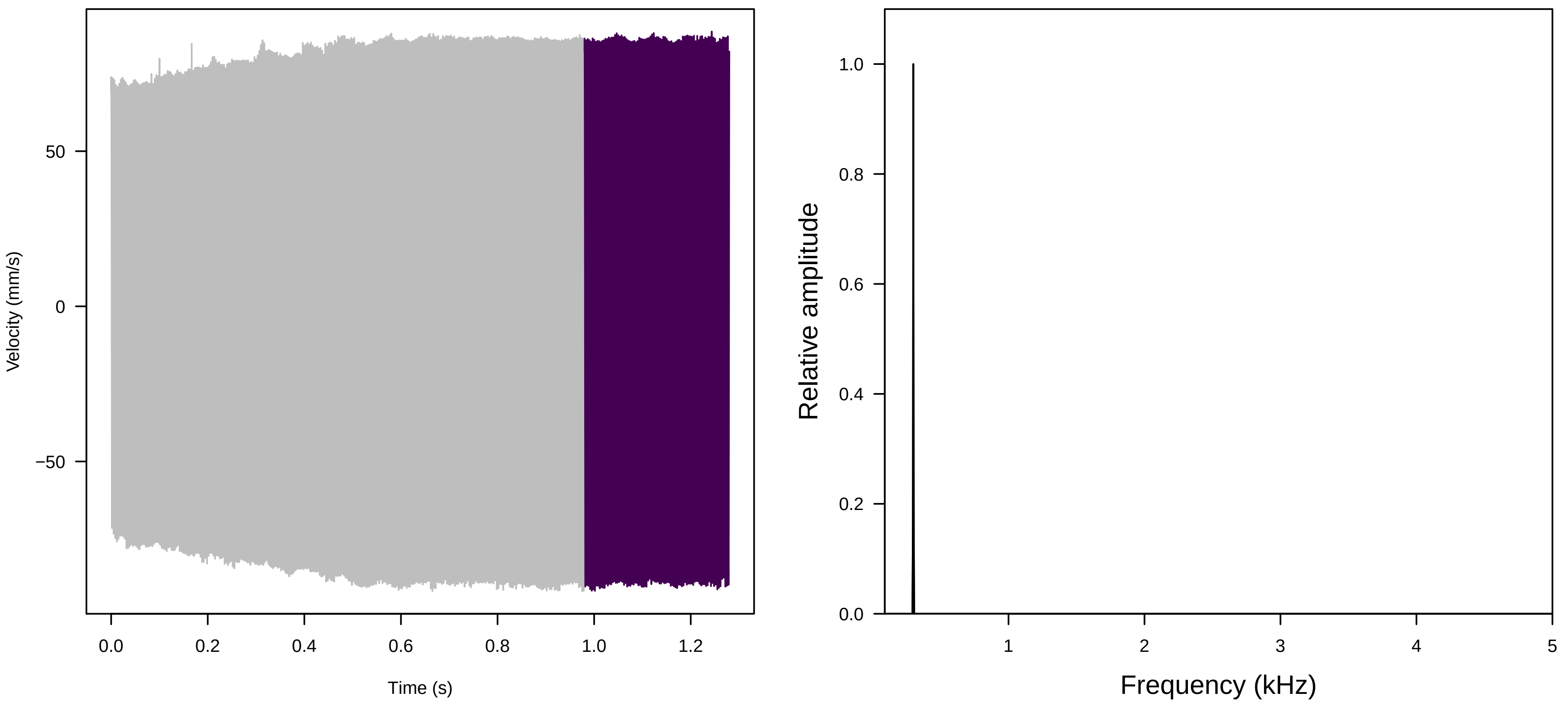

Vel. = 0.014 ; Str. = Receptacle ; Axis = z ; Fl. accession = 10−s−79−2

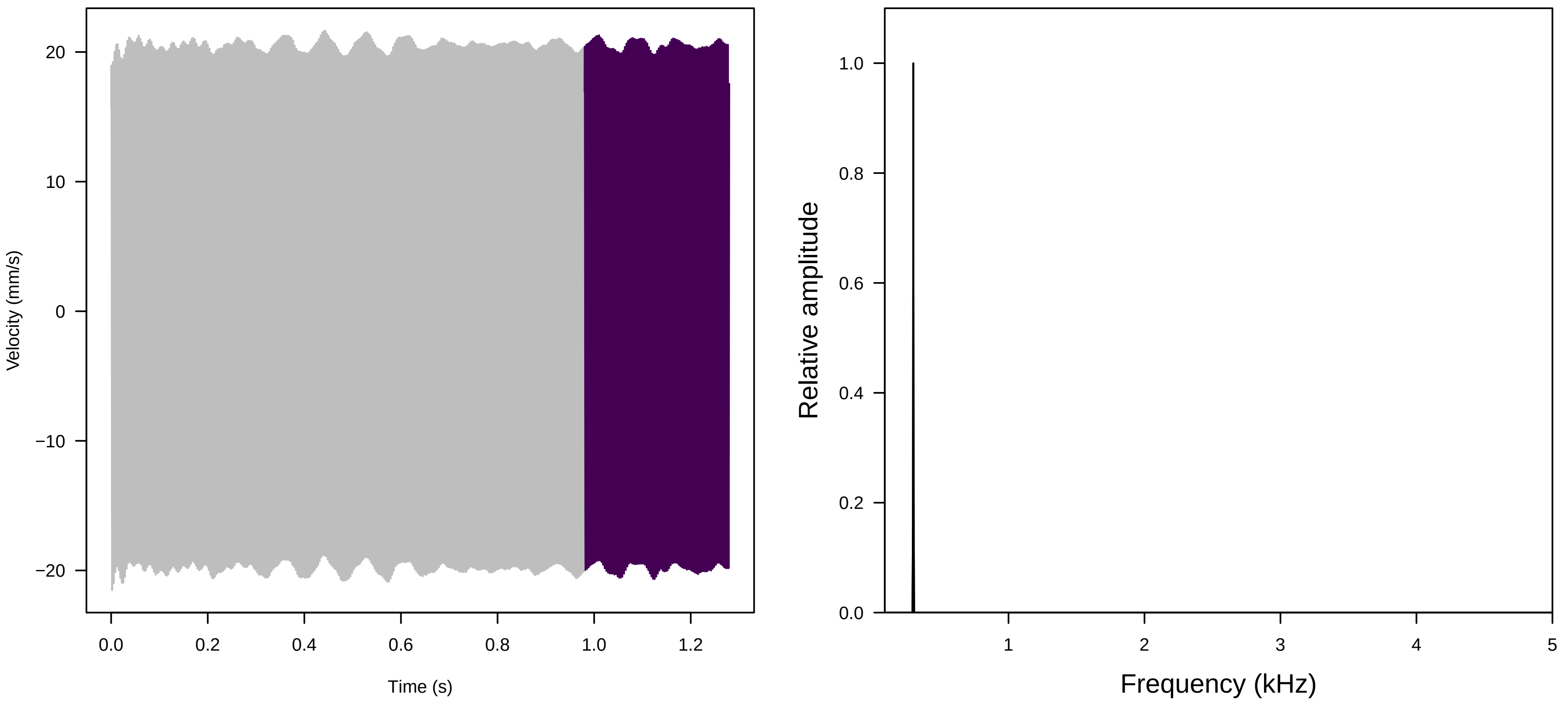

Vel. = 0.028 ; Str. = PA ; Axis = z ; Fl. accession = 10−s−79−2

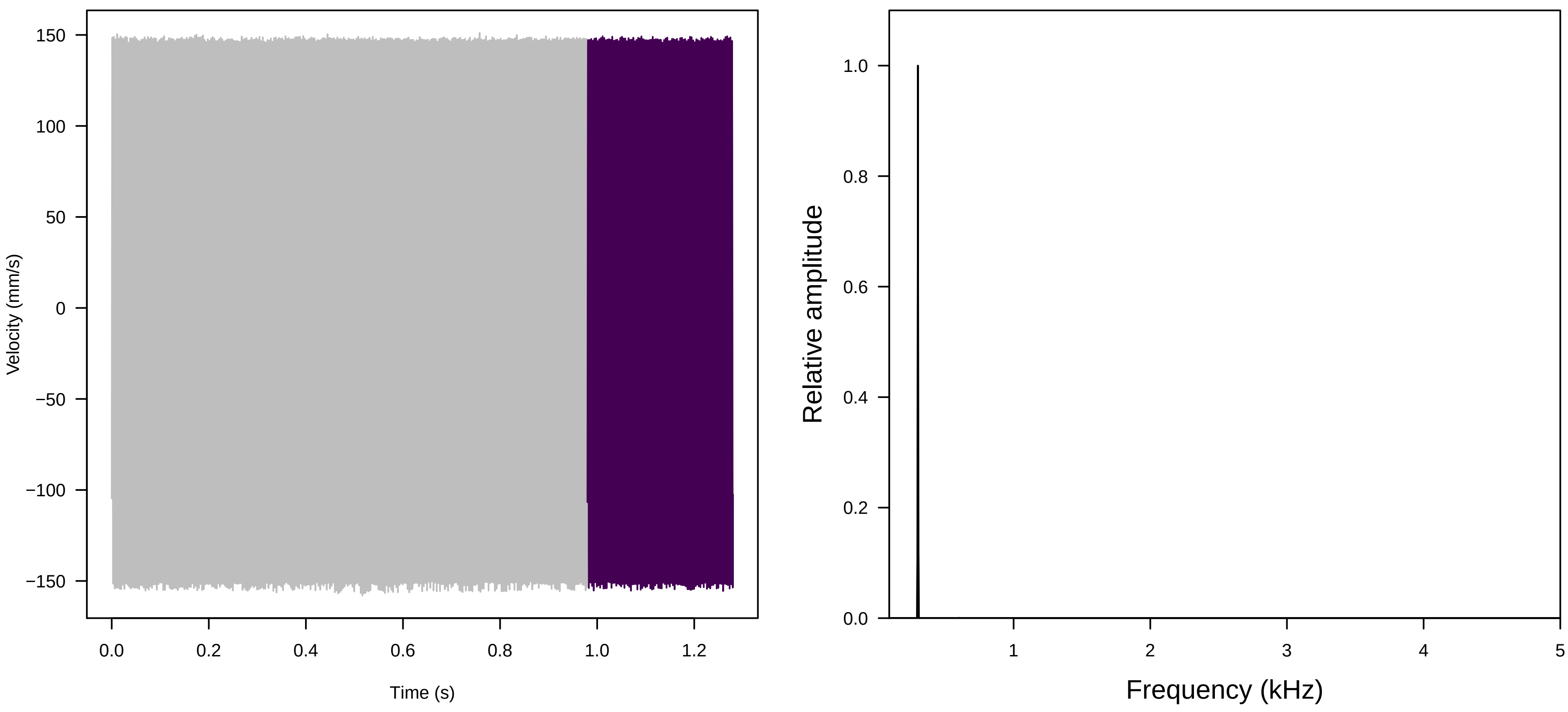

Vel. = 0.028 ; Str. = Receptacle ; Axis = z ; Fl. accession = 10−s−79−2

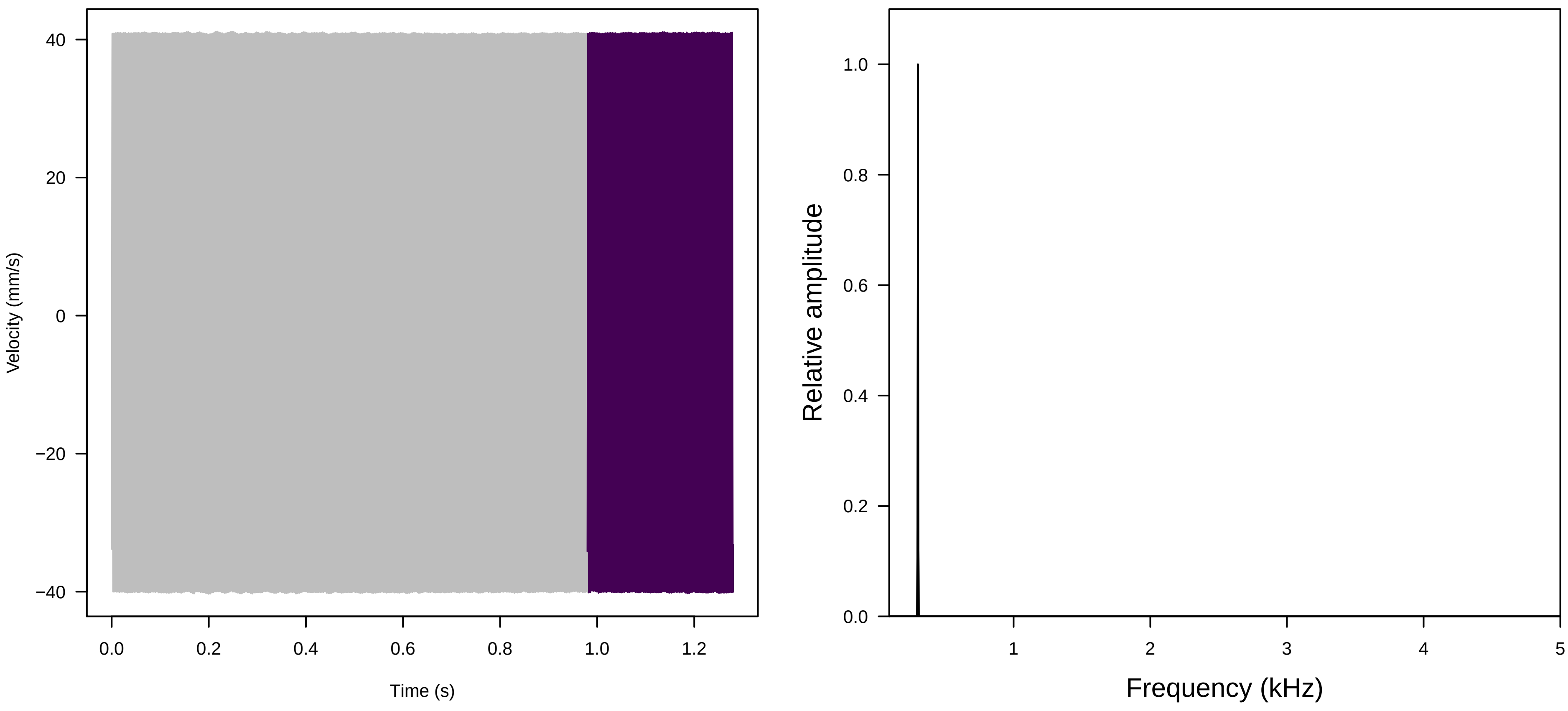

Vel. = 0.028 ; Str. = FA ; Axis = z ; Fl. accession = 10−s−79−2

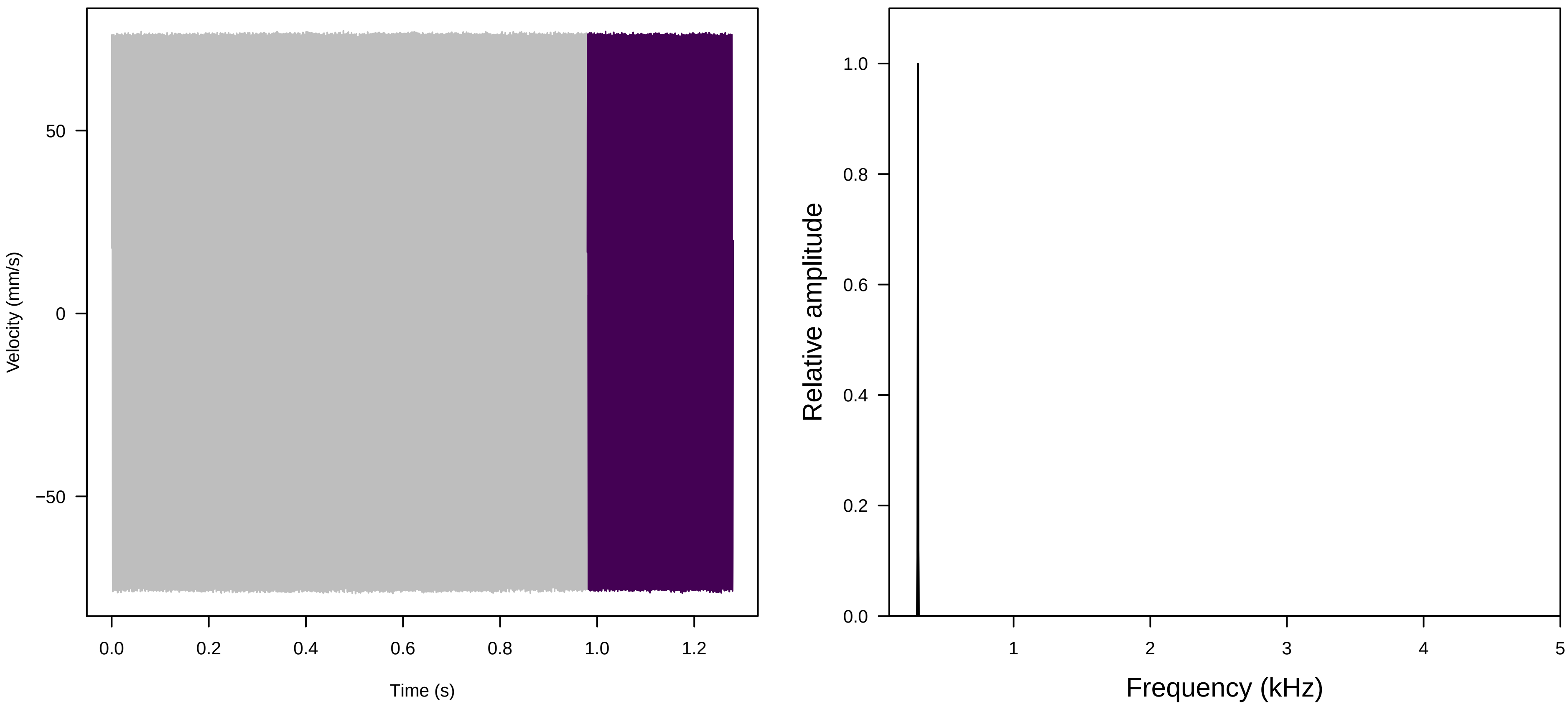

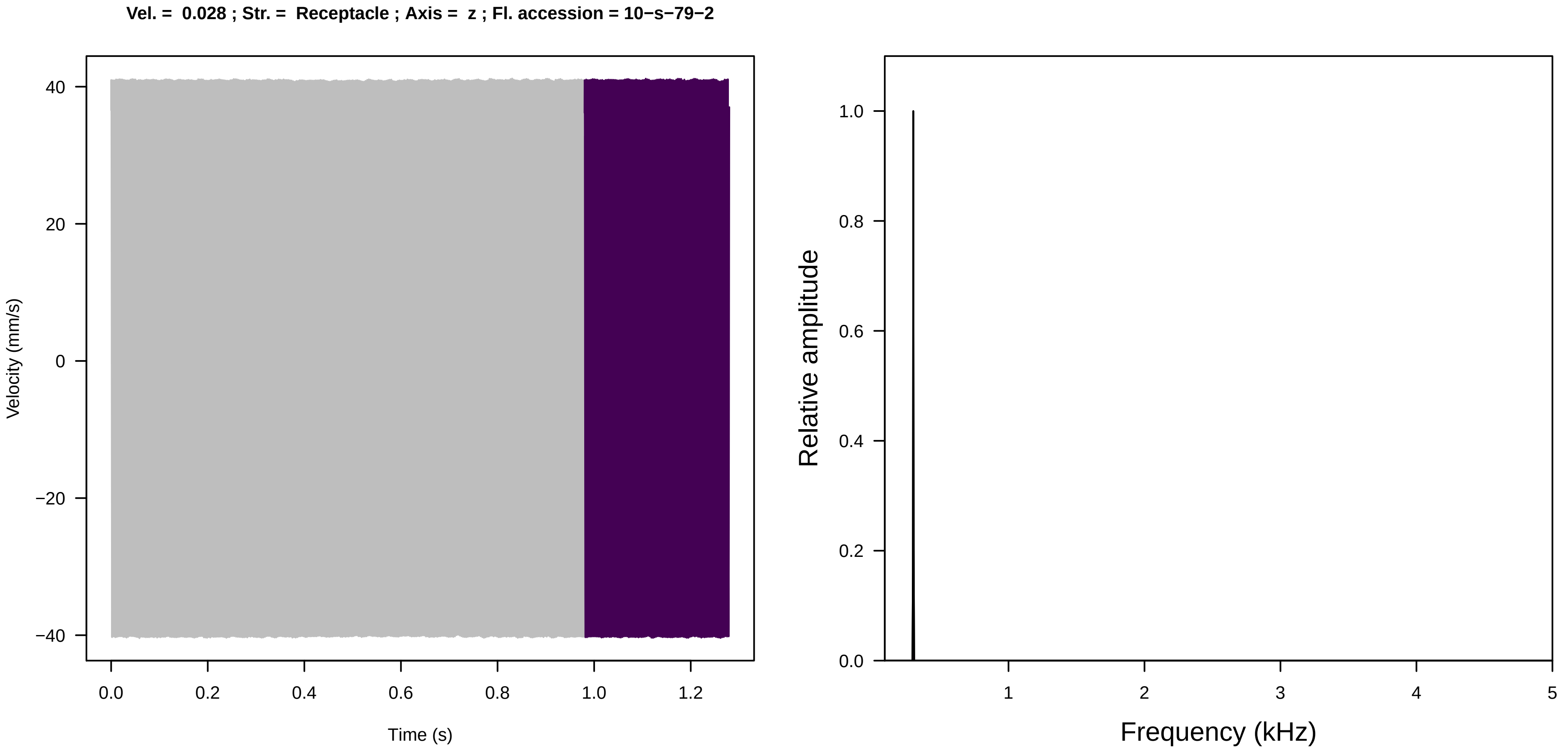
Vel. = 0.028 ; Str. = Receptacle ; Axis = z ; Fl. accession = 10−s−79−2
Velocity (mm/s)
40
20
0
−20
−40
0.0
0.2
0.4
0.6
0.8
1.0
1.2
Time (s)
Relative amplitude
1.0
0.8
0.6
0.4
0.2
0.0
1
2
3
4
5
Frequency (kHz)

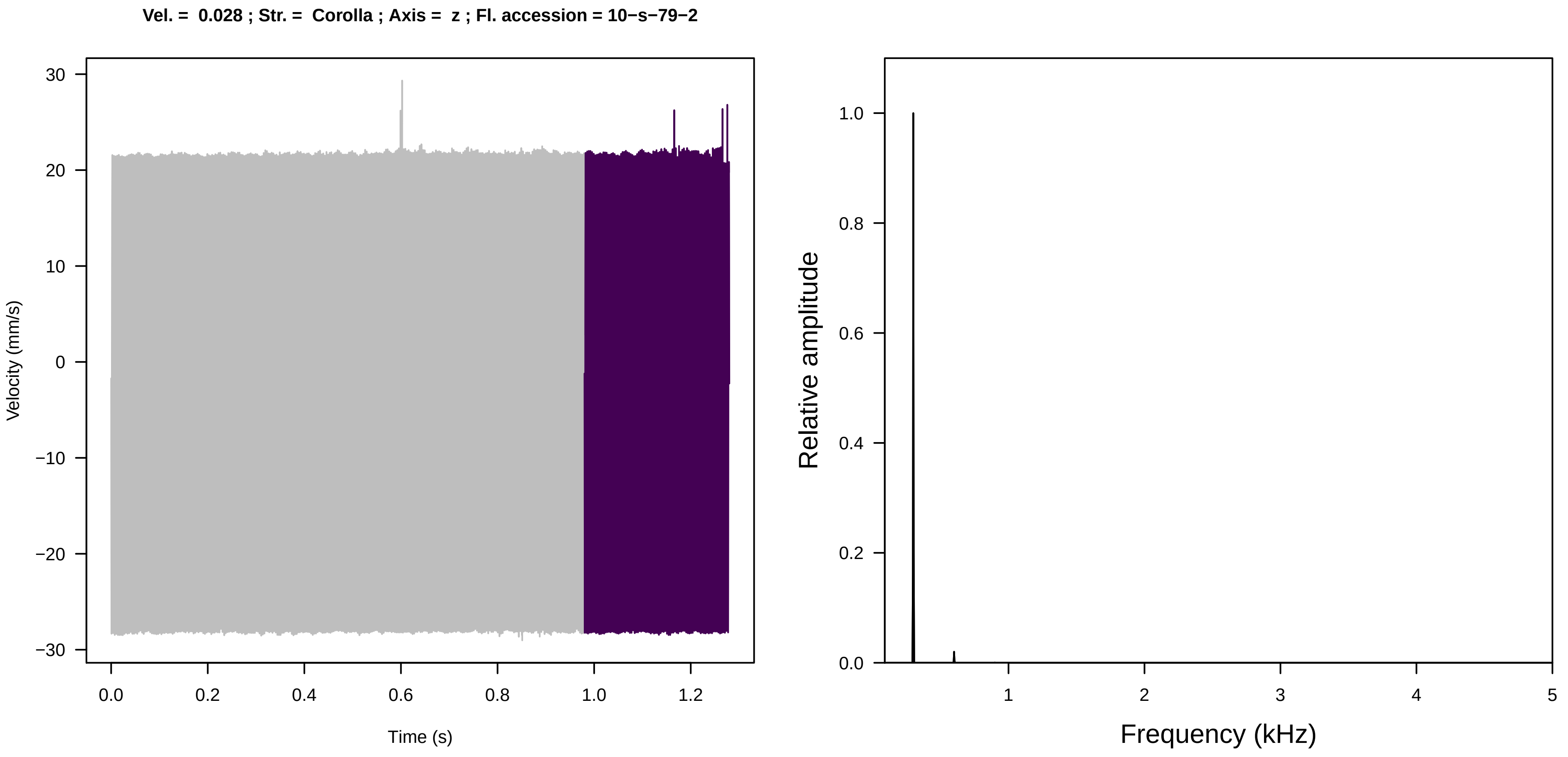
Vel. = 0.028 ; Str. = Corolla ; Axis = z ; Fl. accession = 10−s−79−2
Velocity (mm/s)
30
20
10
0
−10
−20
−30
0.0
0.2
0.4
0.6
0.8
1.0
1.2
Time (s)
Relative amplitude
1.0
0.8
0.6
0.4
0.2
0.0
1
2
3
4
5
Frequency (kHz)

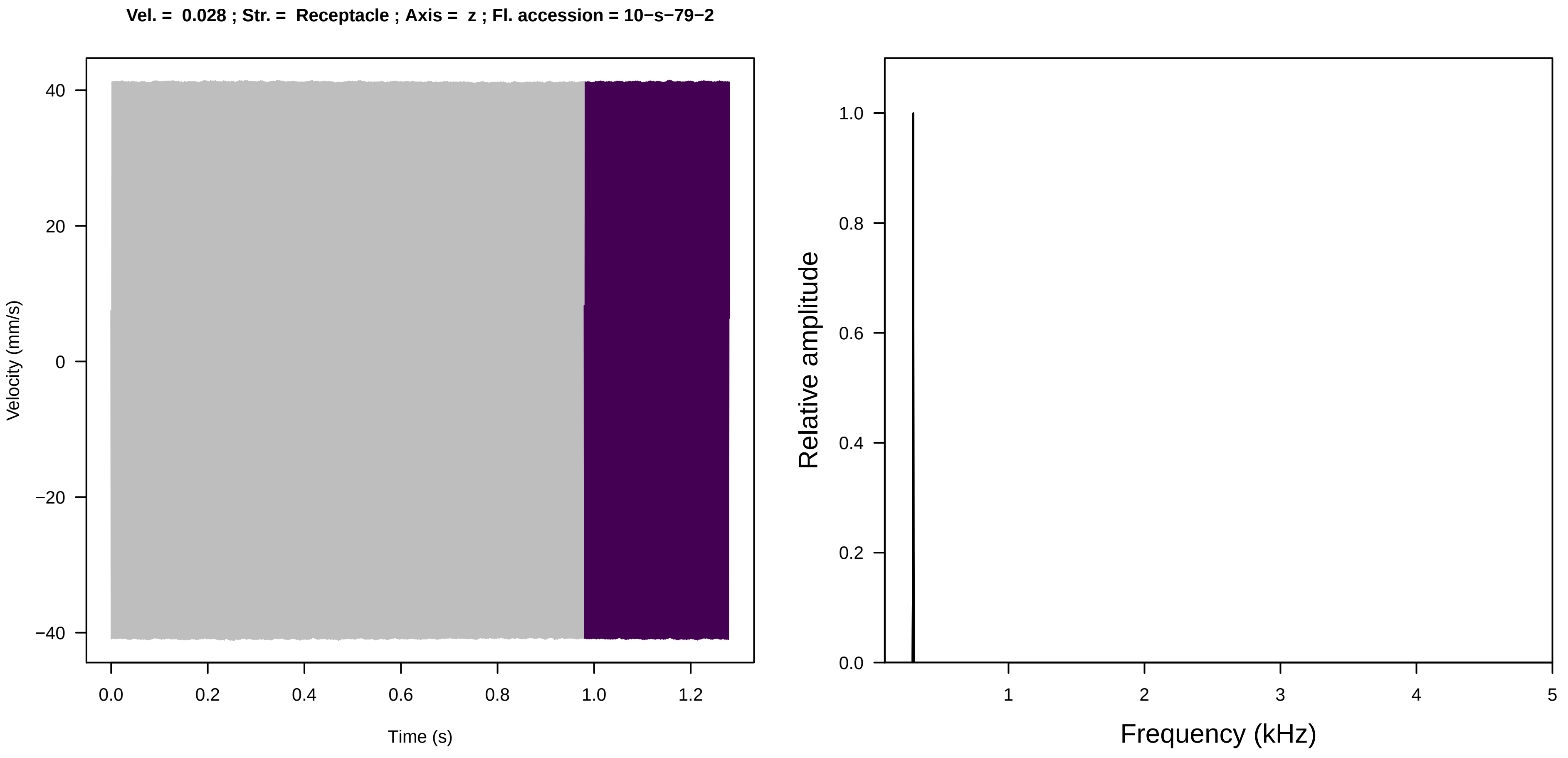
Vel. = 0.028 ; Str. = Receptacle ; Axis = z ; Fl. accession = 10−s−79−2
Velocity (mm/s)
40
20
0
−20
−40
0.0
0.2
0.4
0.6
0.8
1.0
1.2
Time (s)
Relative amplitude
1.0
0.8
0.6
0.4
0.2
0.0
1
2
3
4
5
Frequency (kHz)

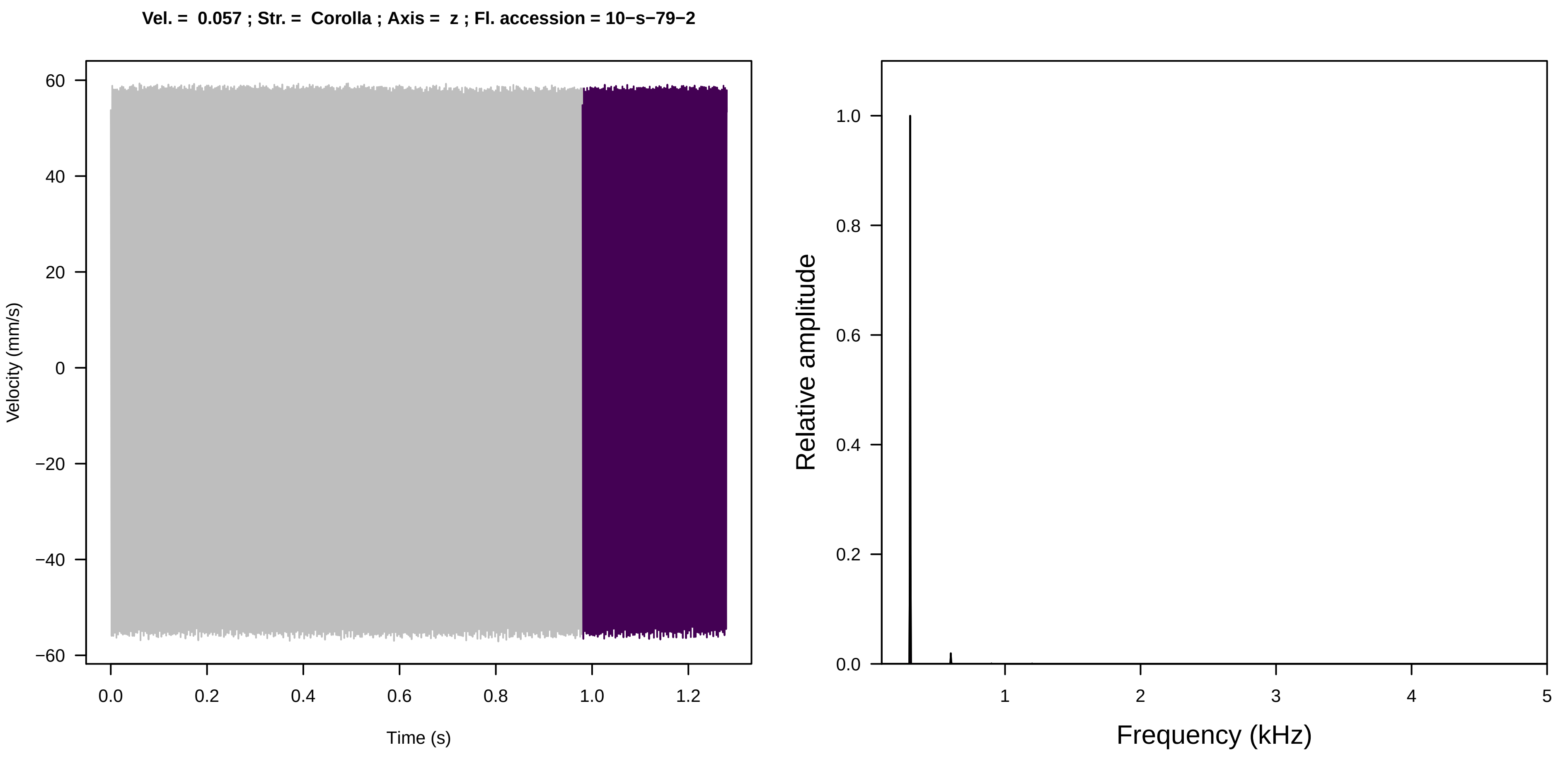
Vel. = 0.057 ; Str. = Corolla ; Axis = z ; Fl. accession = 10−s−79−2
Velocity (mm/s)
Time (s)
Relative amplitude
Frequency (kHz)

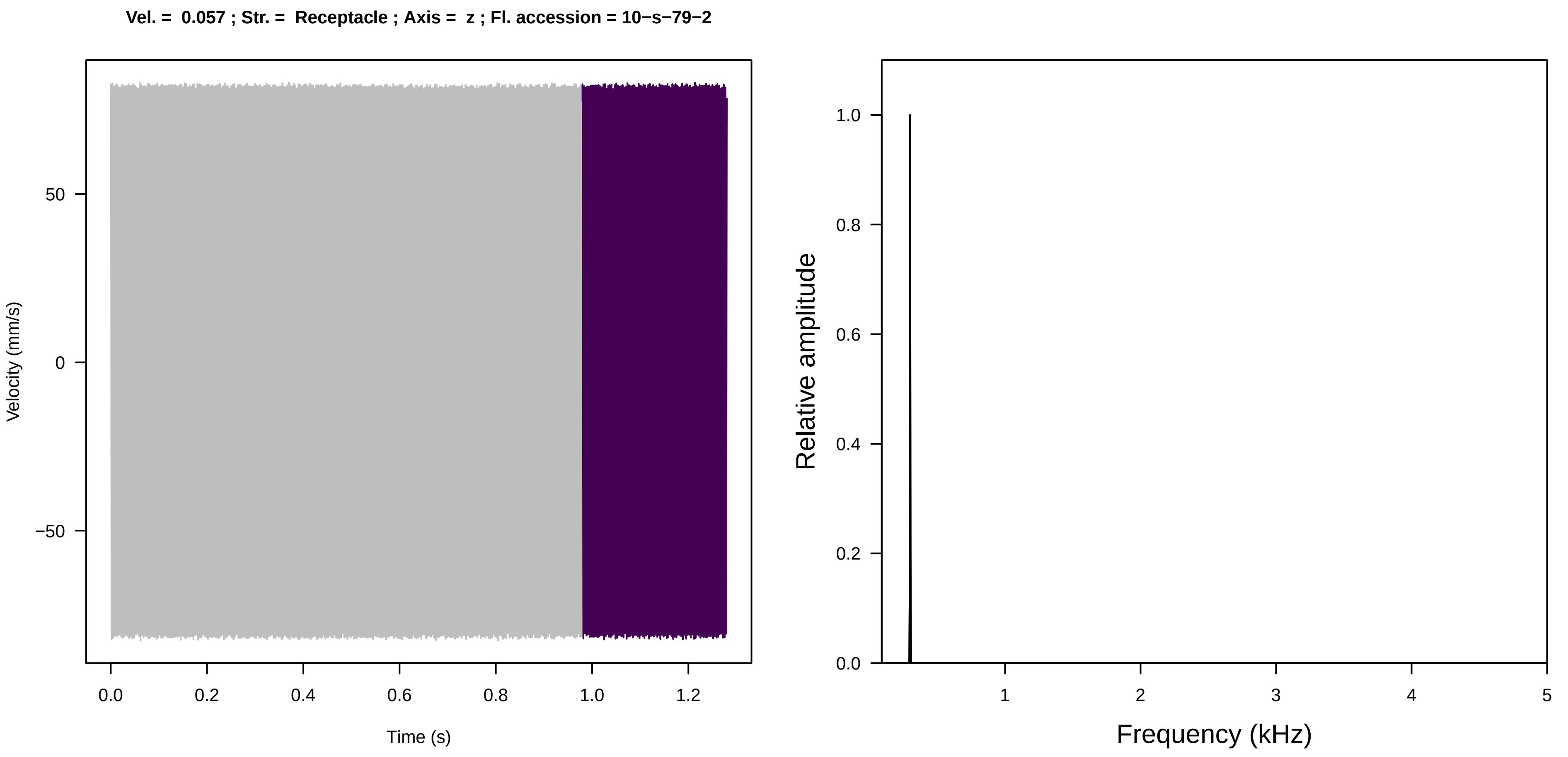
Vel. = 0.057 ; Str. = Receptacle ; Axis = z ; Fl. accession = 10−s−79−2
Velocity (mm/s)
Time (s)
Relative amplitude
Frequency (kHz)

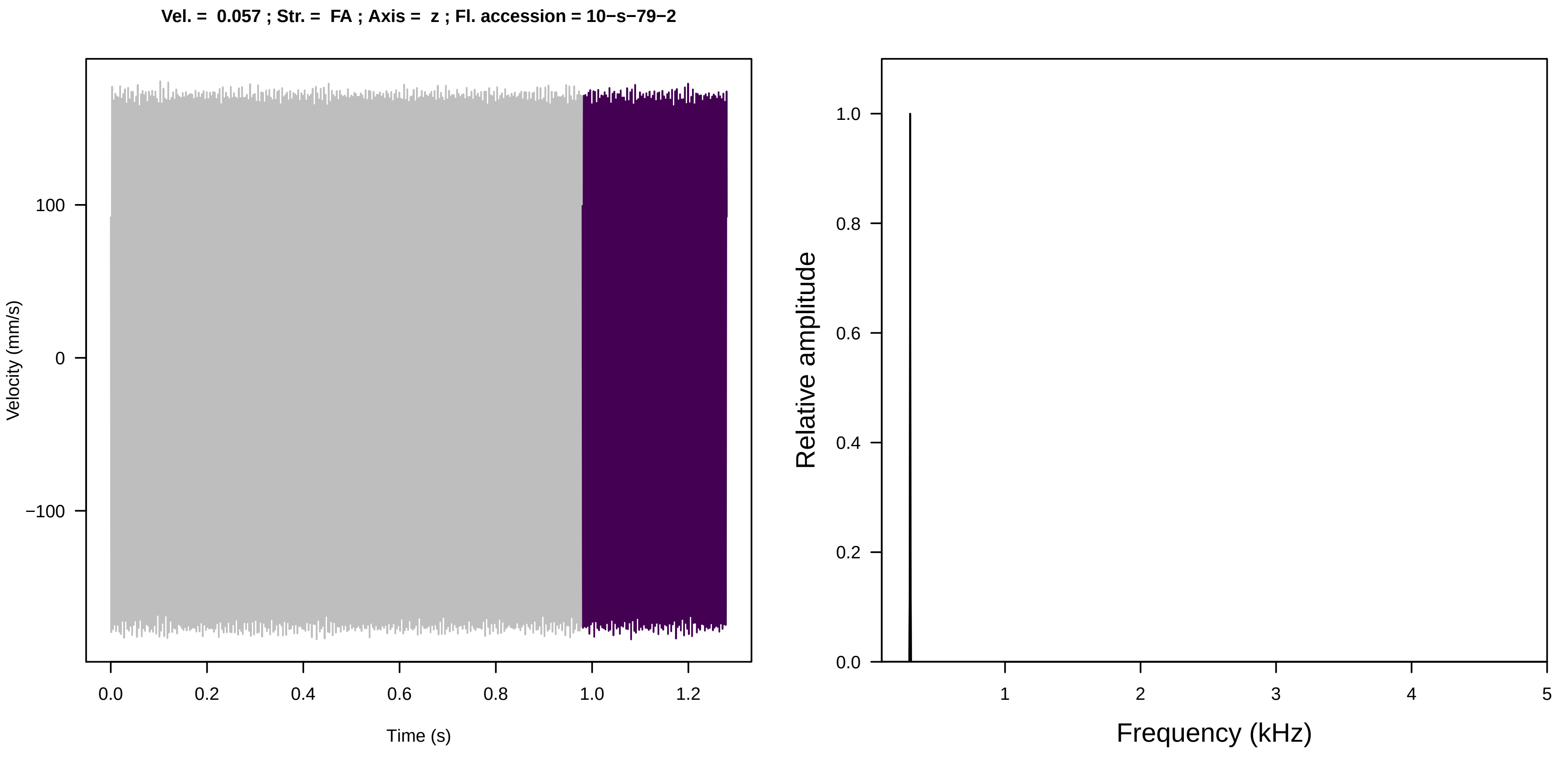
Vel. = 0.057 ; Str. = FA ; Axis = z ; Fl. accession = 10−s−79−2
Velocity (mm/s)
Time (s)
Relative amplitude
Frequency (kHz)

Vel. = 0.057 ; Str. = Receptacle ; Axis = z ; Fl. accession = 10−s−79−2

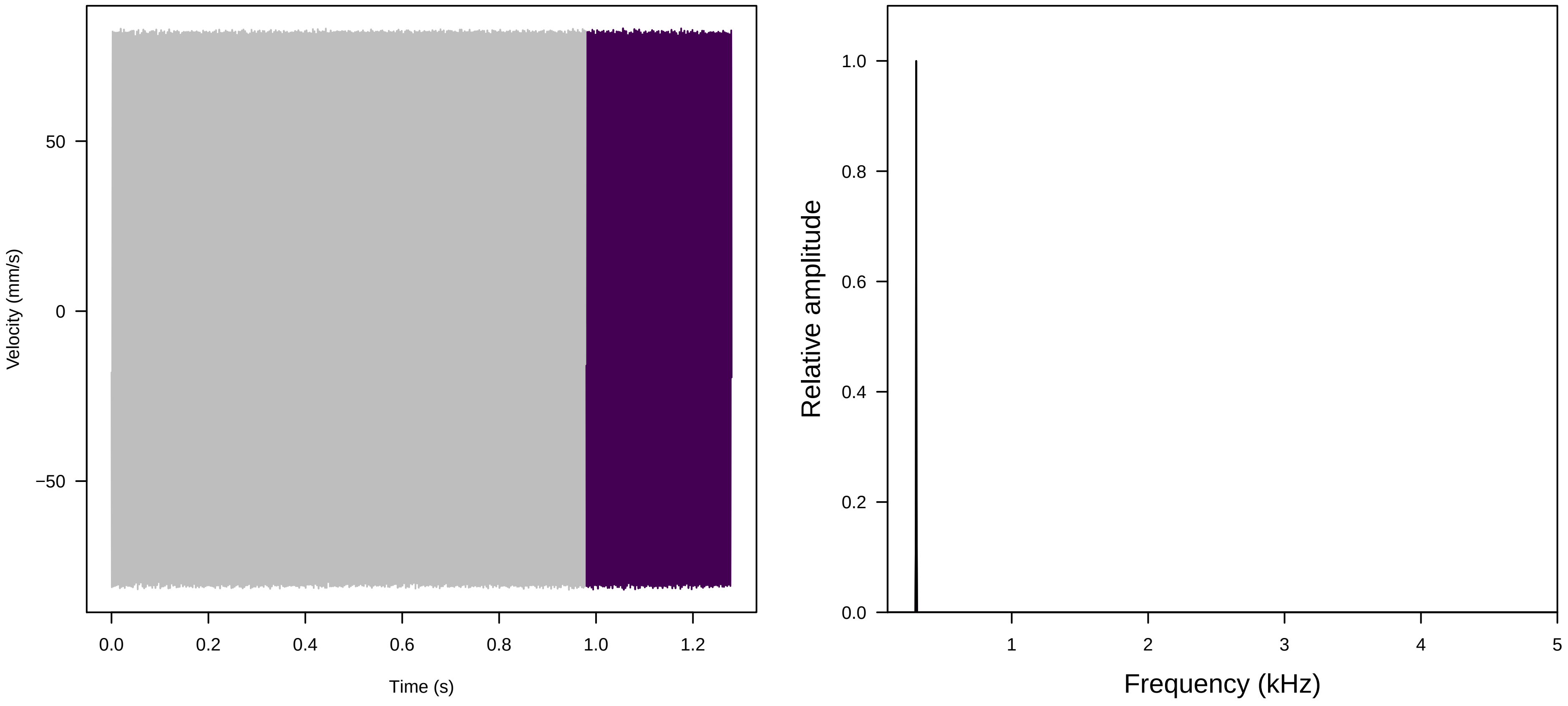

Vel. = 0.057 ; Str. = PA ; Axis = z ; Fl. accession = 10−s−79−2

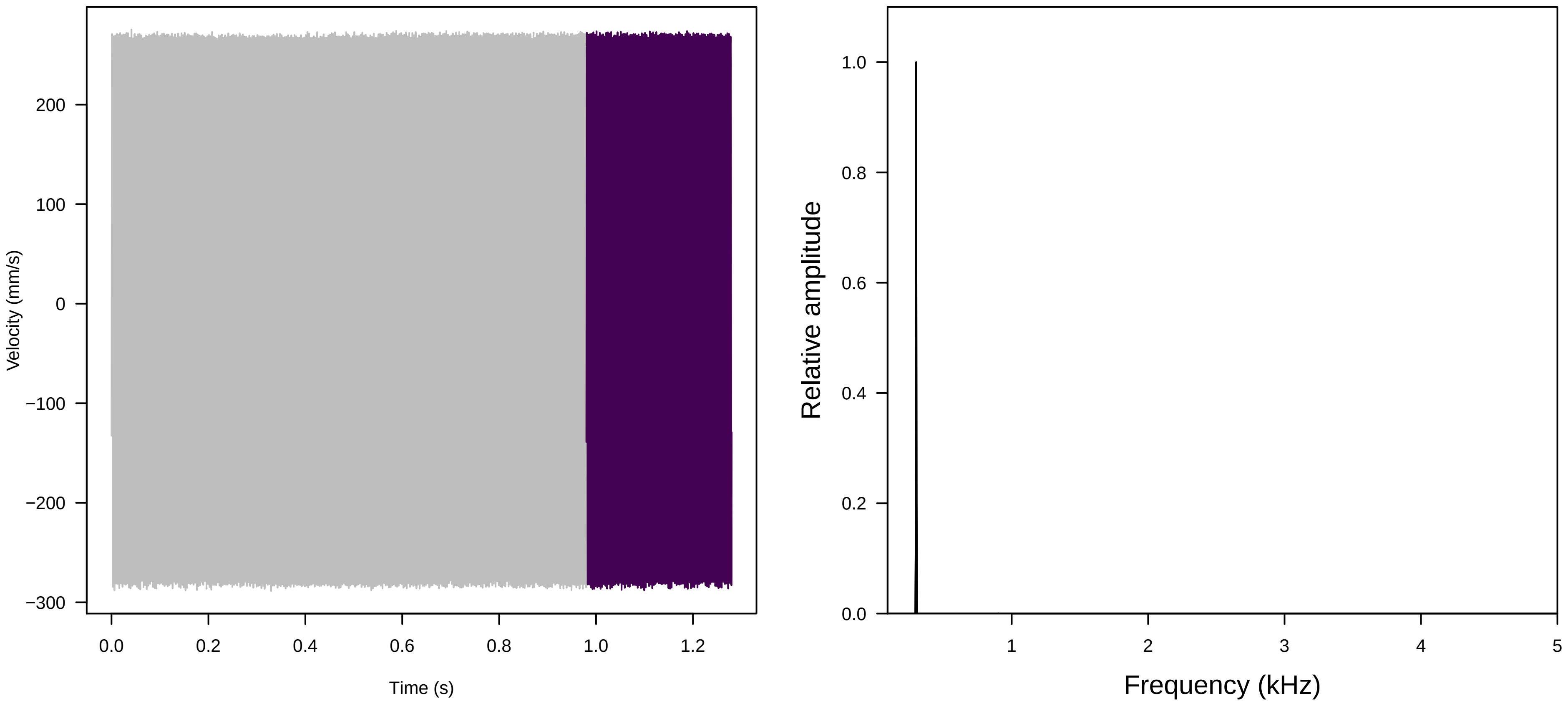

Vel. = 0.057 ; Str. = Receptacle ; Axis = z ; Fl. accession = 10−s−79−2

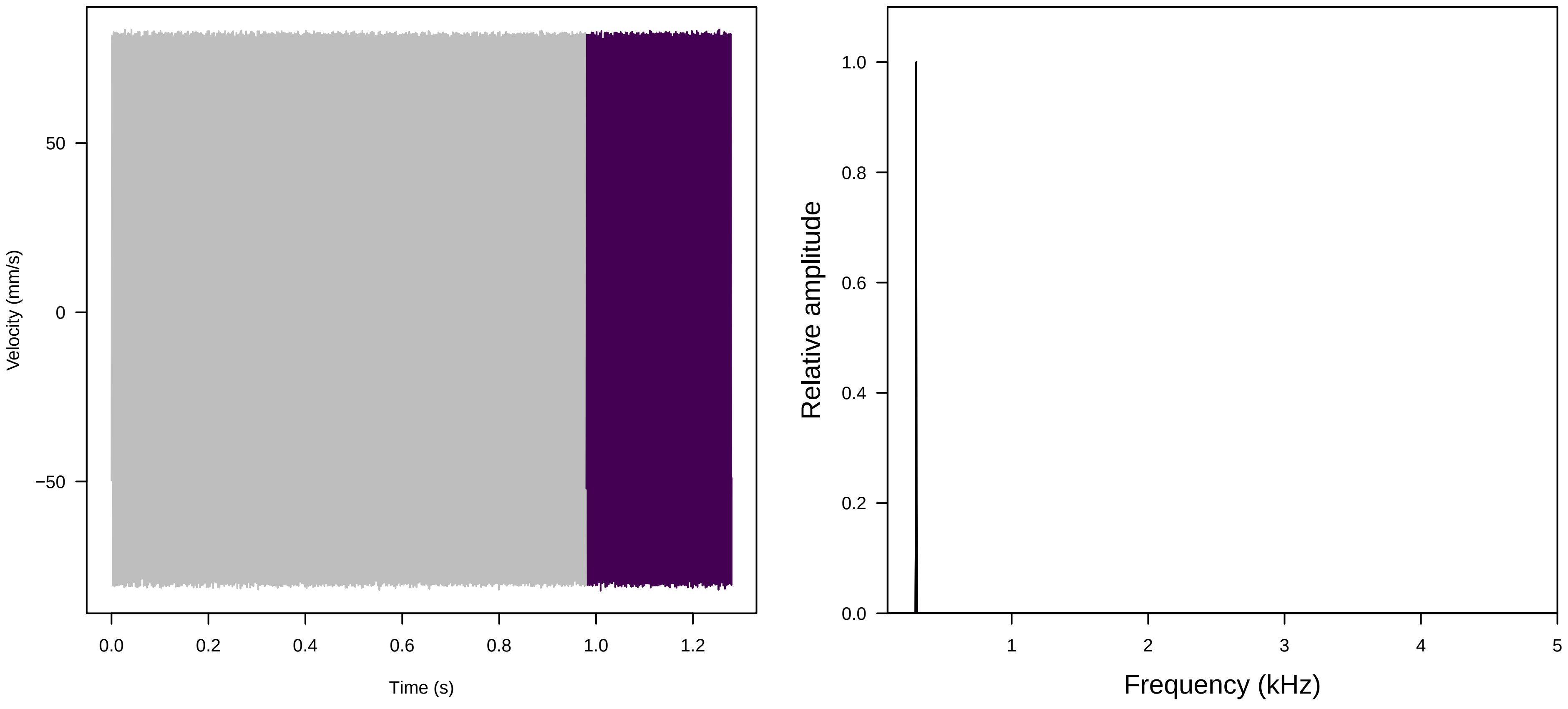

Vel. = 0.057 ; Str. = Corolla ; Axis = x ; Fl. accession = 10−s−79−2

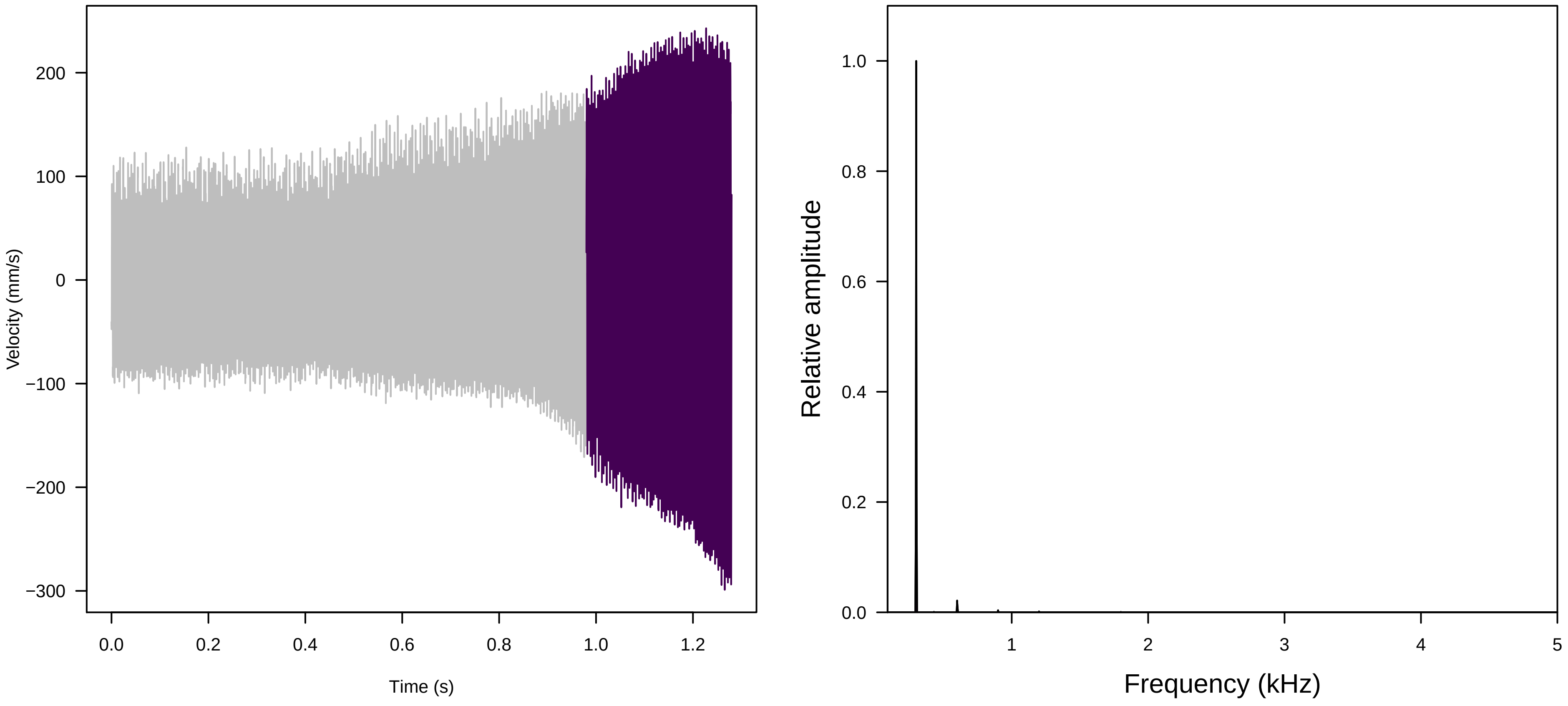

Vel. = 0.057 ; Str. = Receptacle ; Axis = x ; Fl. accession = 10−s−79−2

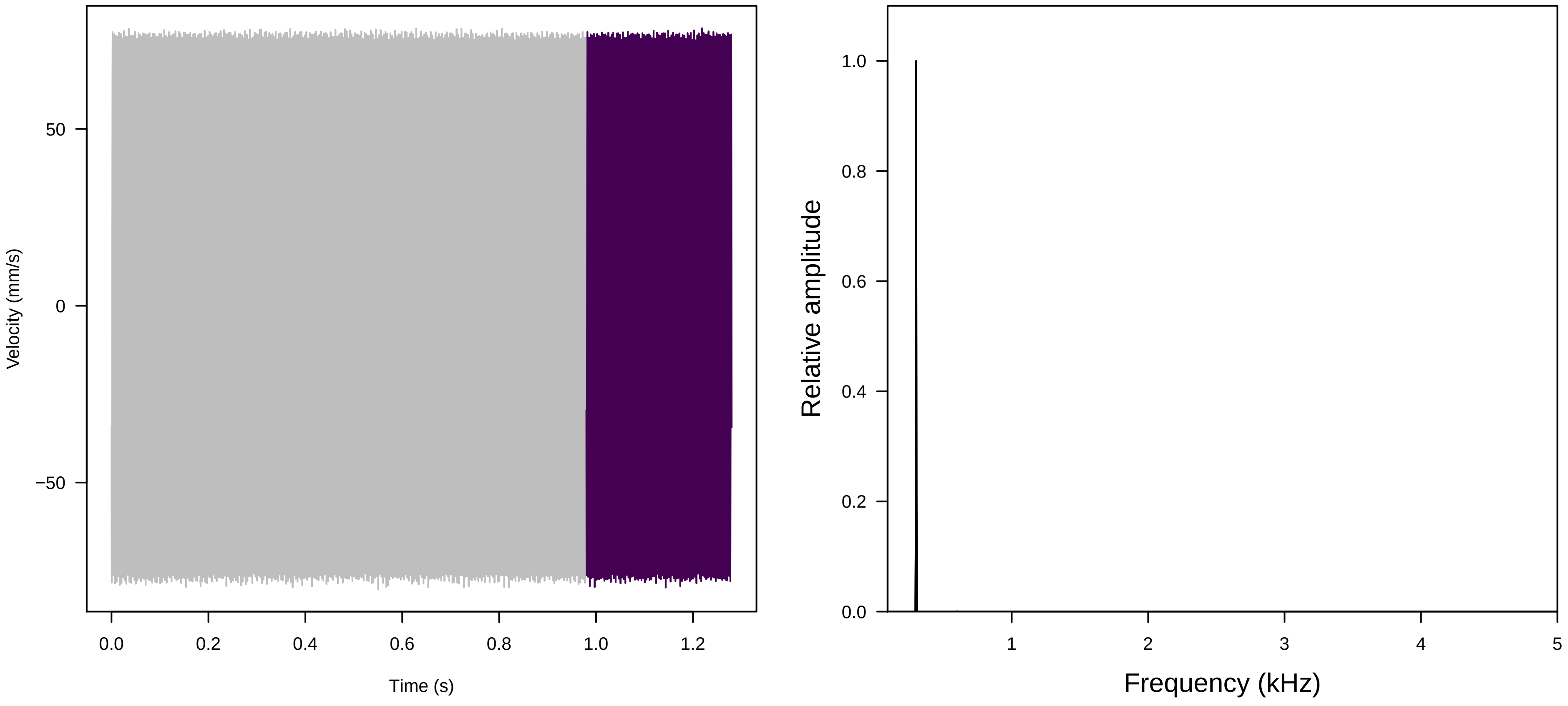

Vel. = 0.057 ; Str. = FA ; Axis = x ; Fl. accession = 10−s−79−2

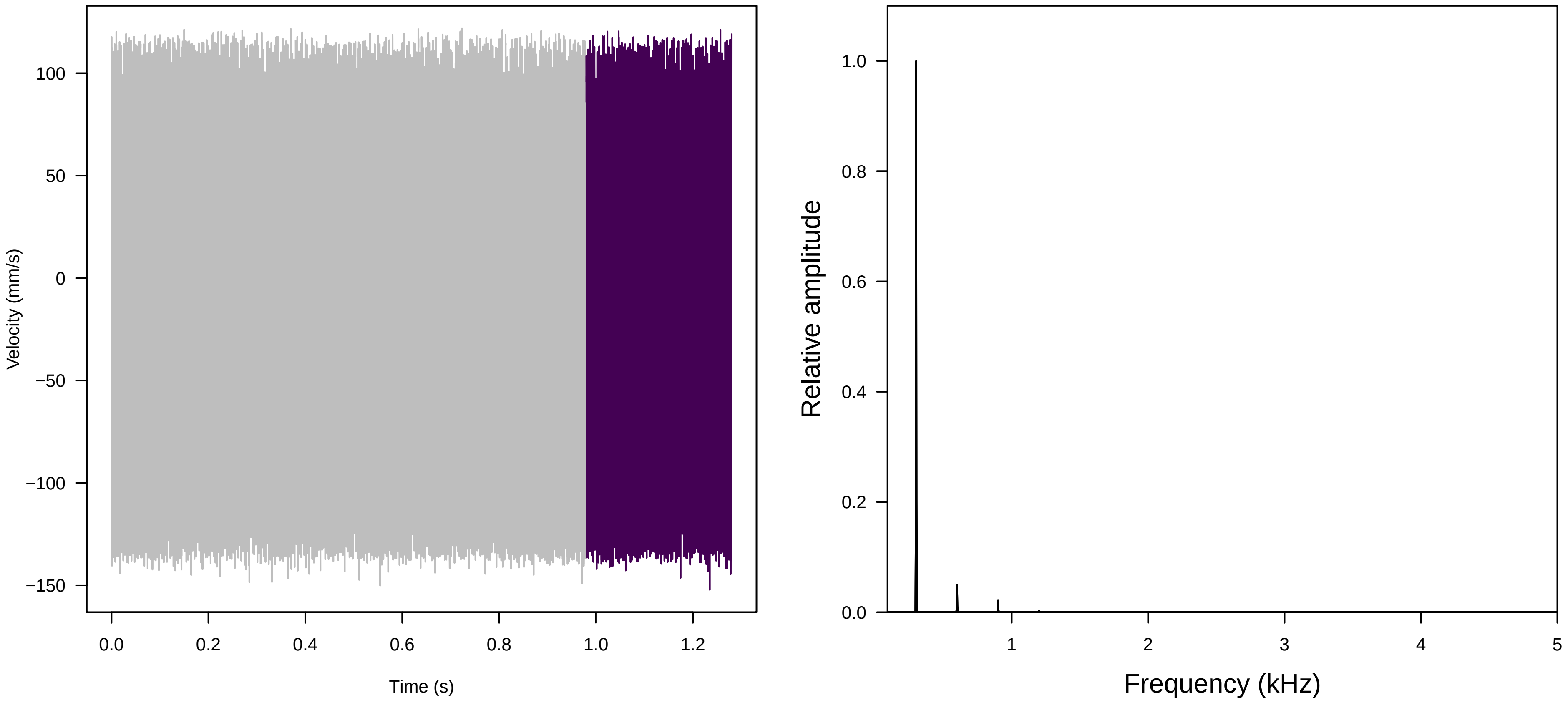

Vel. = 0.057 ; Str. = Receptacle ; Axis = x ; Fl. accession = 10−s−79−2

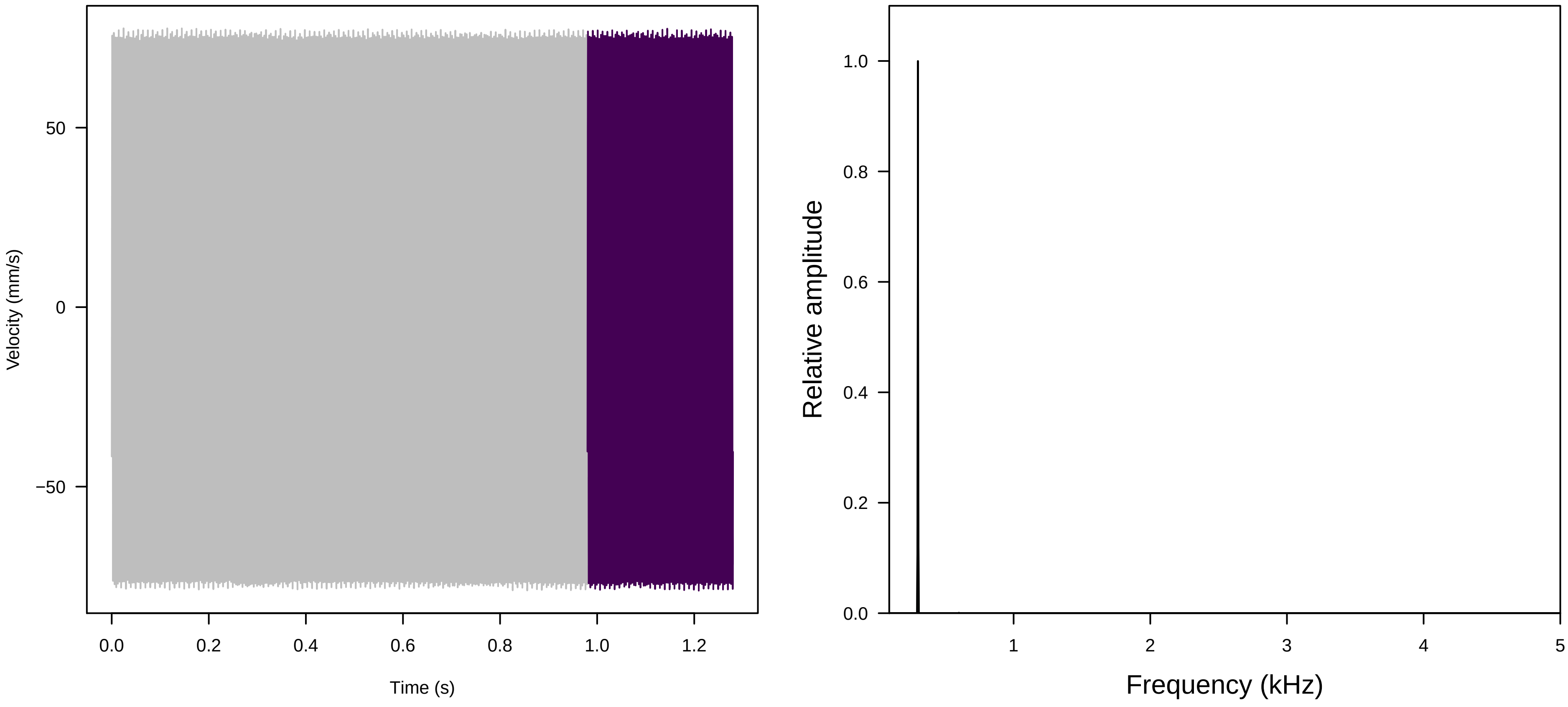

Vel. = 0.057 ; Str. = PA ; Axis = x ; Fl. accession = 10−s−79−2

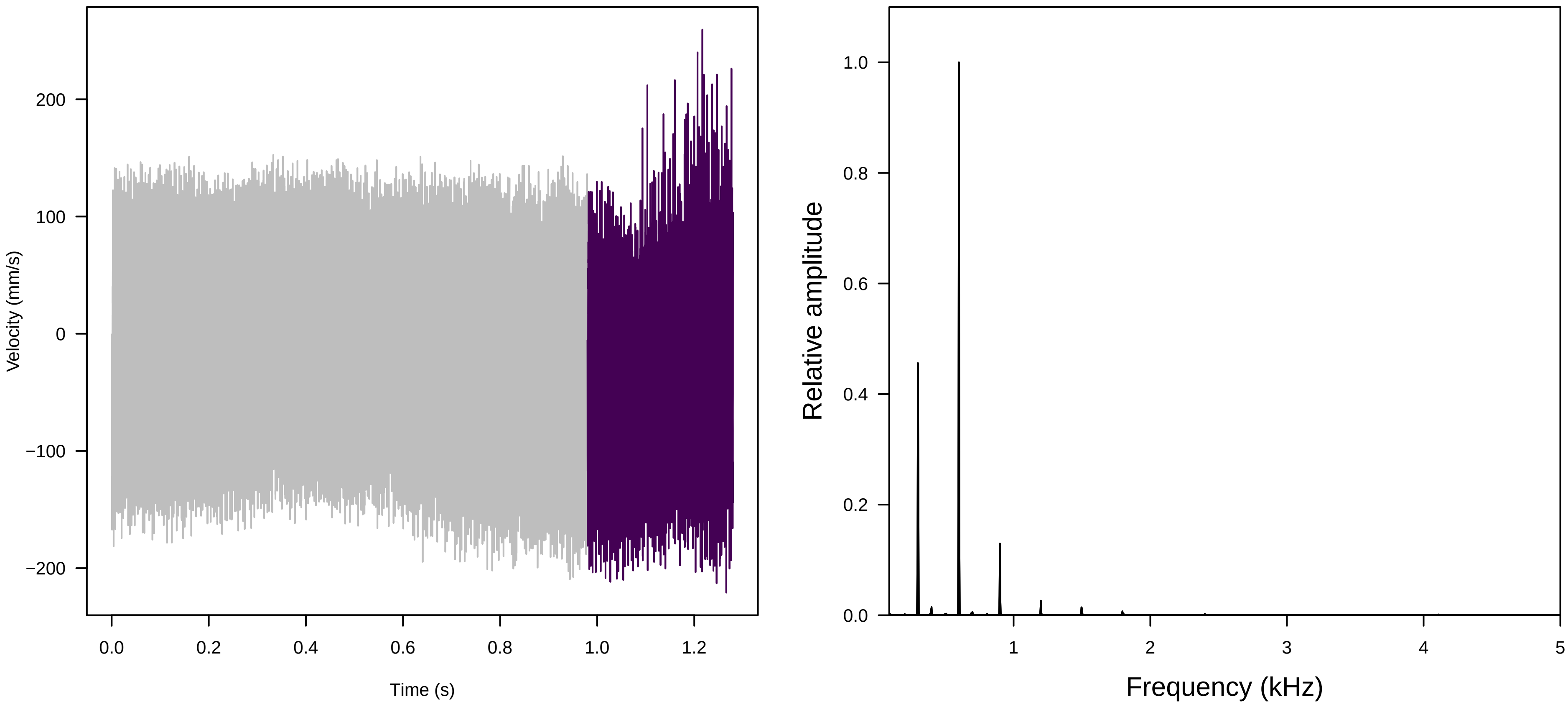

Vel. = 0.057 ; Str. = Receptacle ; Axis = x ; Fl. accession = 10−s−79−2

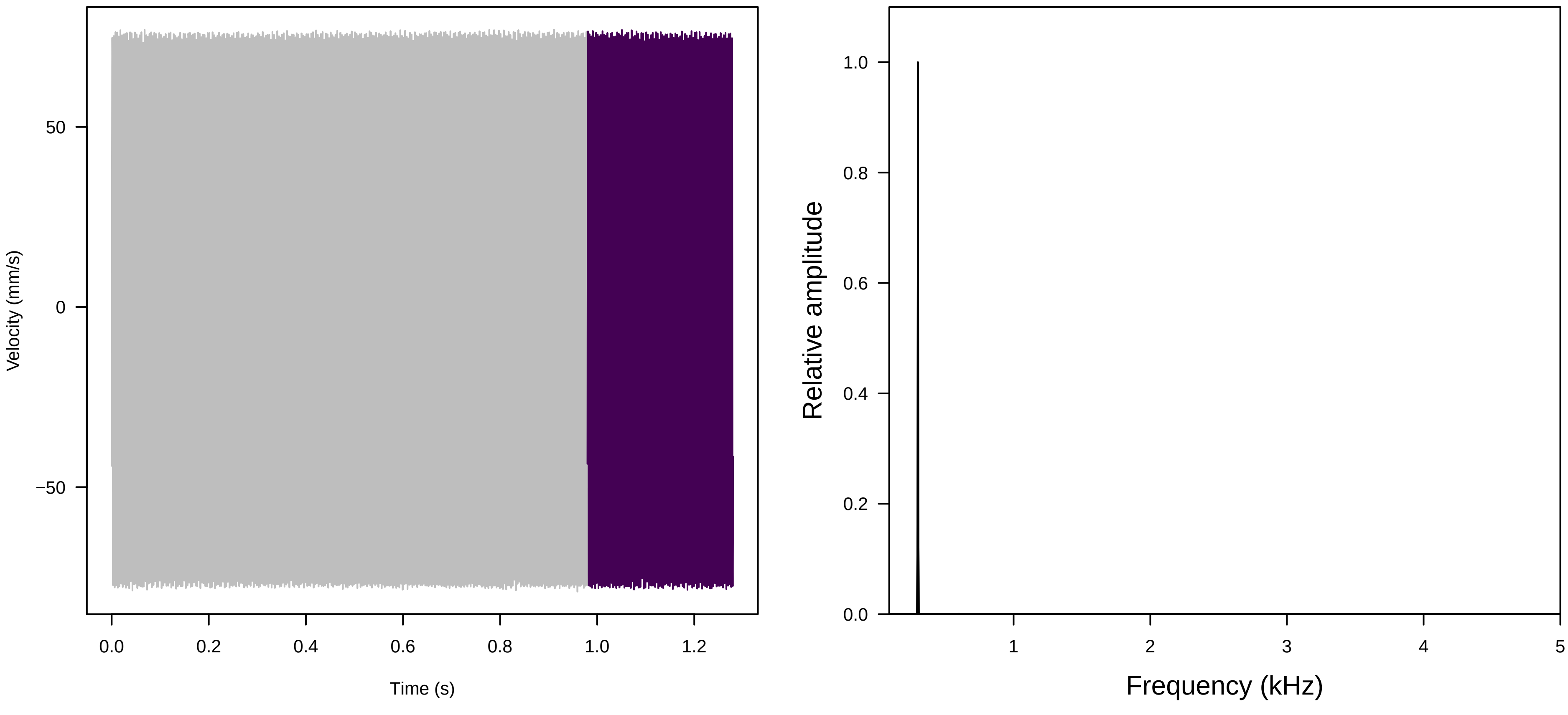

Vel. = 0.028 ; Str. = PA ; Axis = x ; Fl. accession = 10−s−79−2

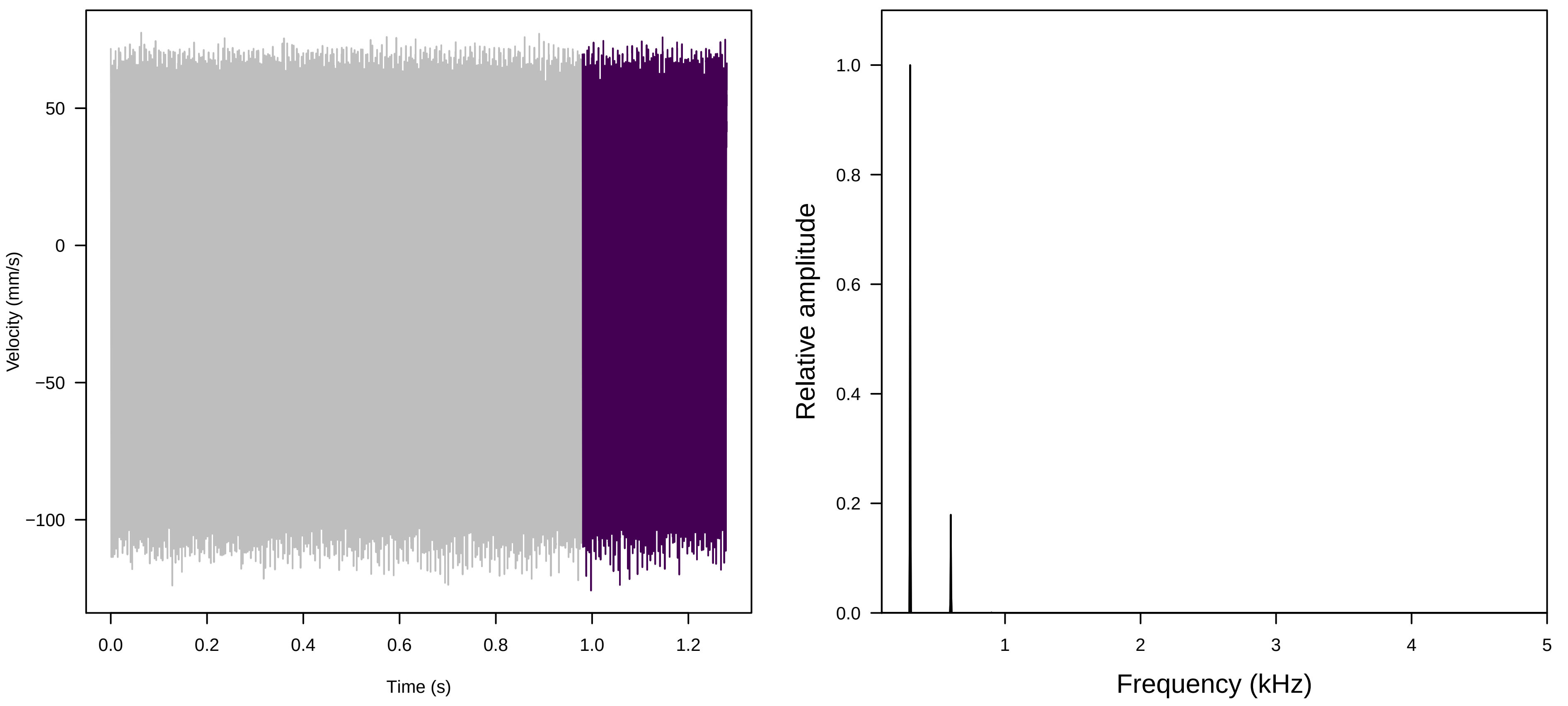

Vel. = 0.028 ; Str. = Receptacle ; Axis = x ; Fl. accession = 10−s−79−2

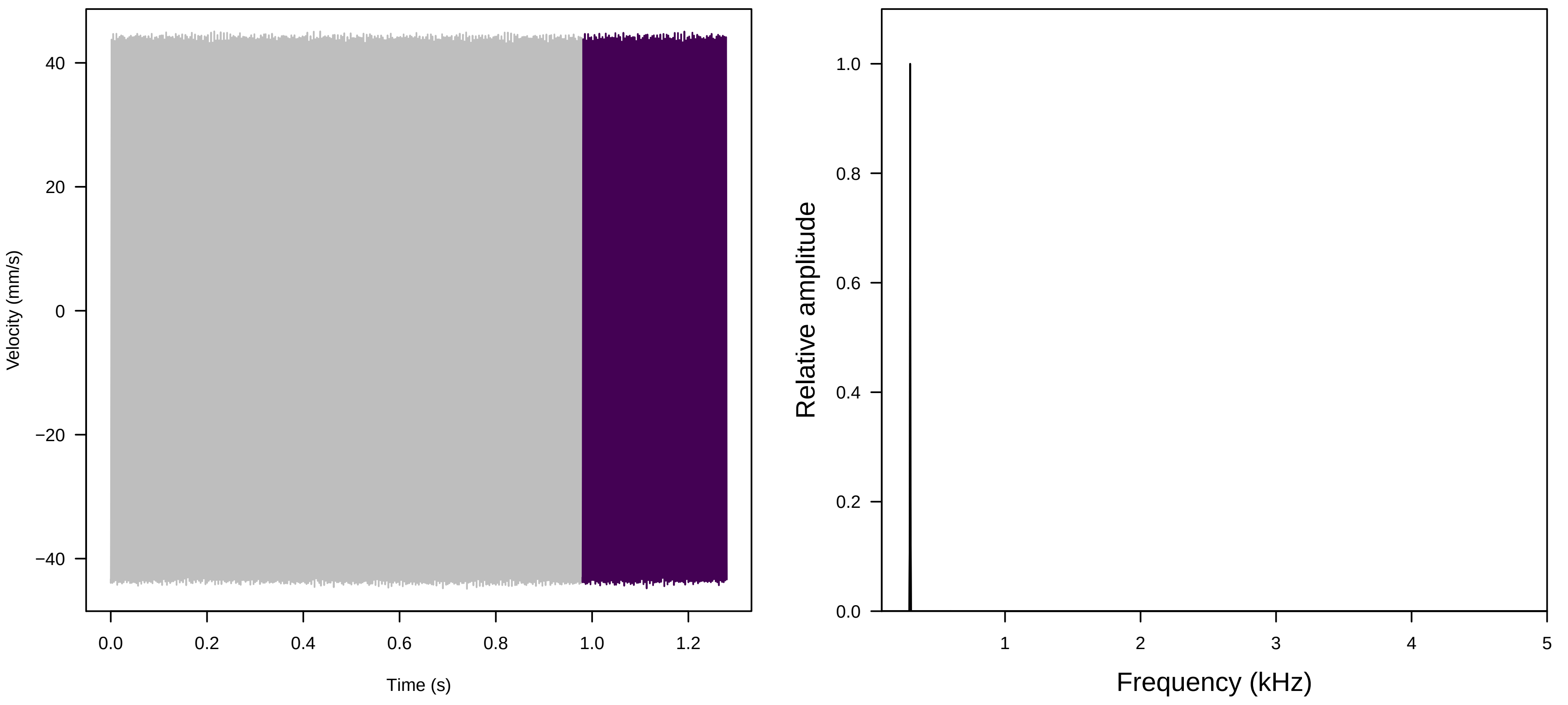

Vel. = 0.028 ; Str. = FA ; Axis = x ; Fl. accession = 10−s−79−2

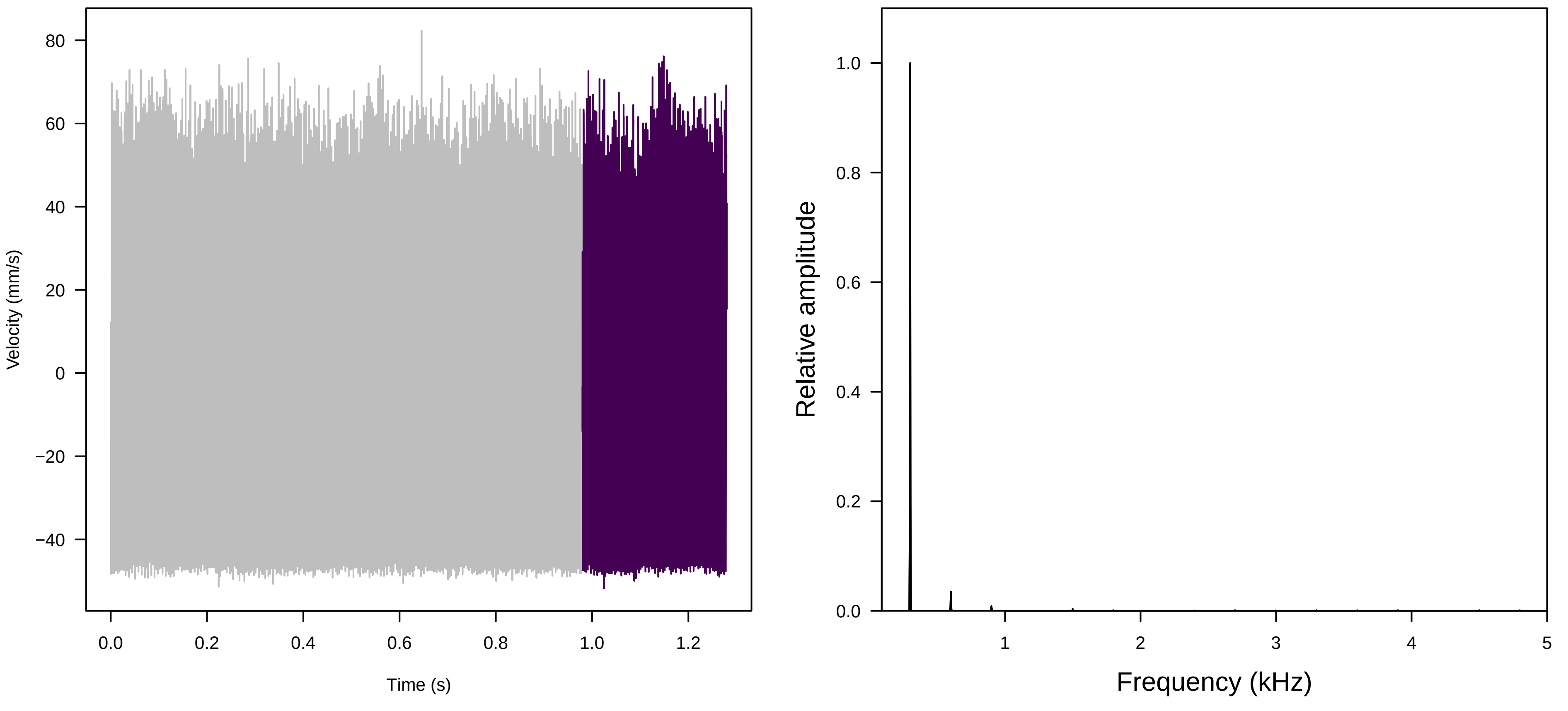

Vel. = 0.028 ; Str. = Receptacle ; Axis = x ; Fl. accession = 10−s−79−2

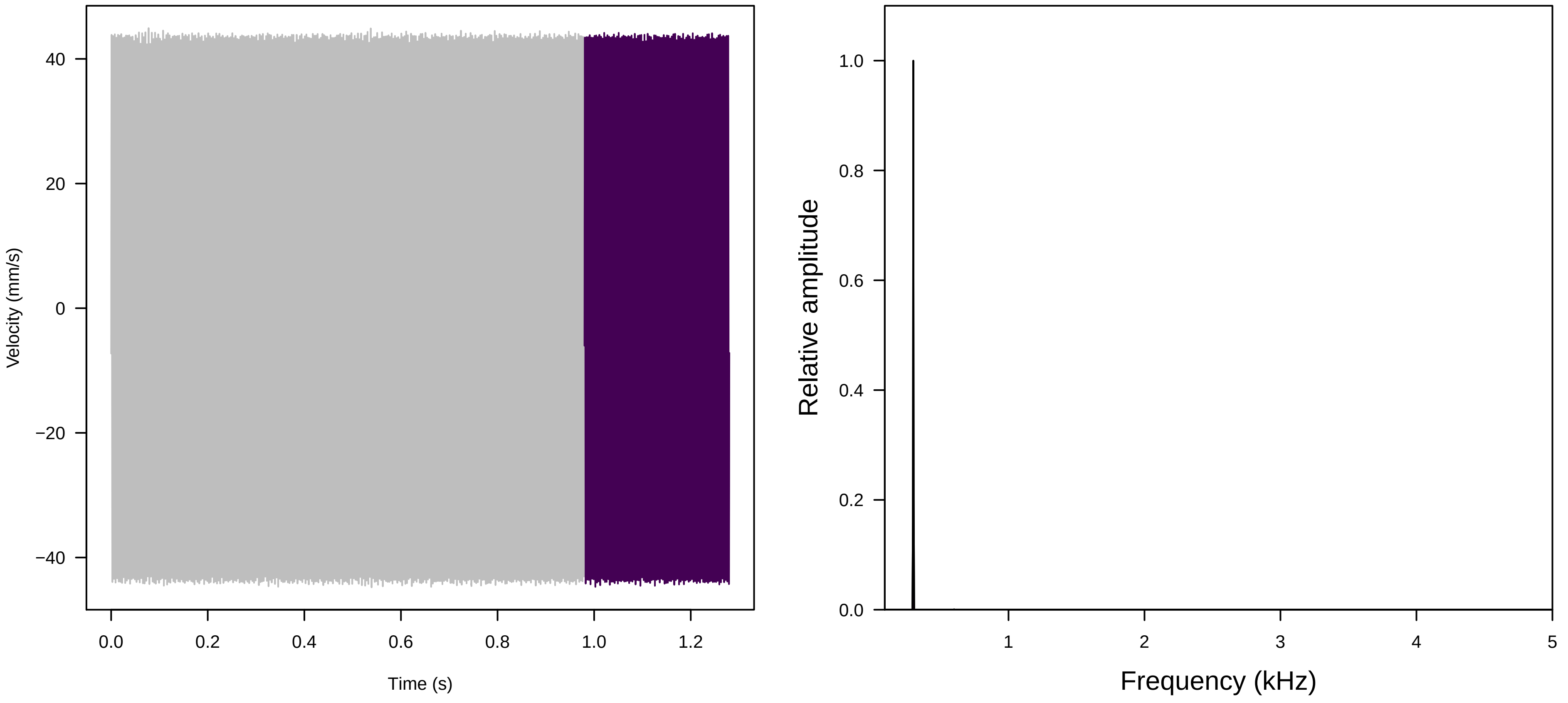

Vel. = 0.028 ; Str. = Corolla ; Axis = x ; Fl. accession = 10−s−79−2

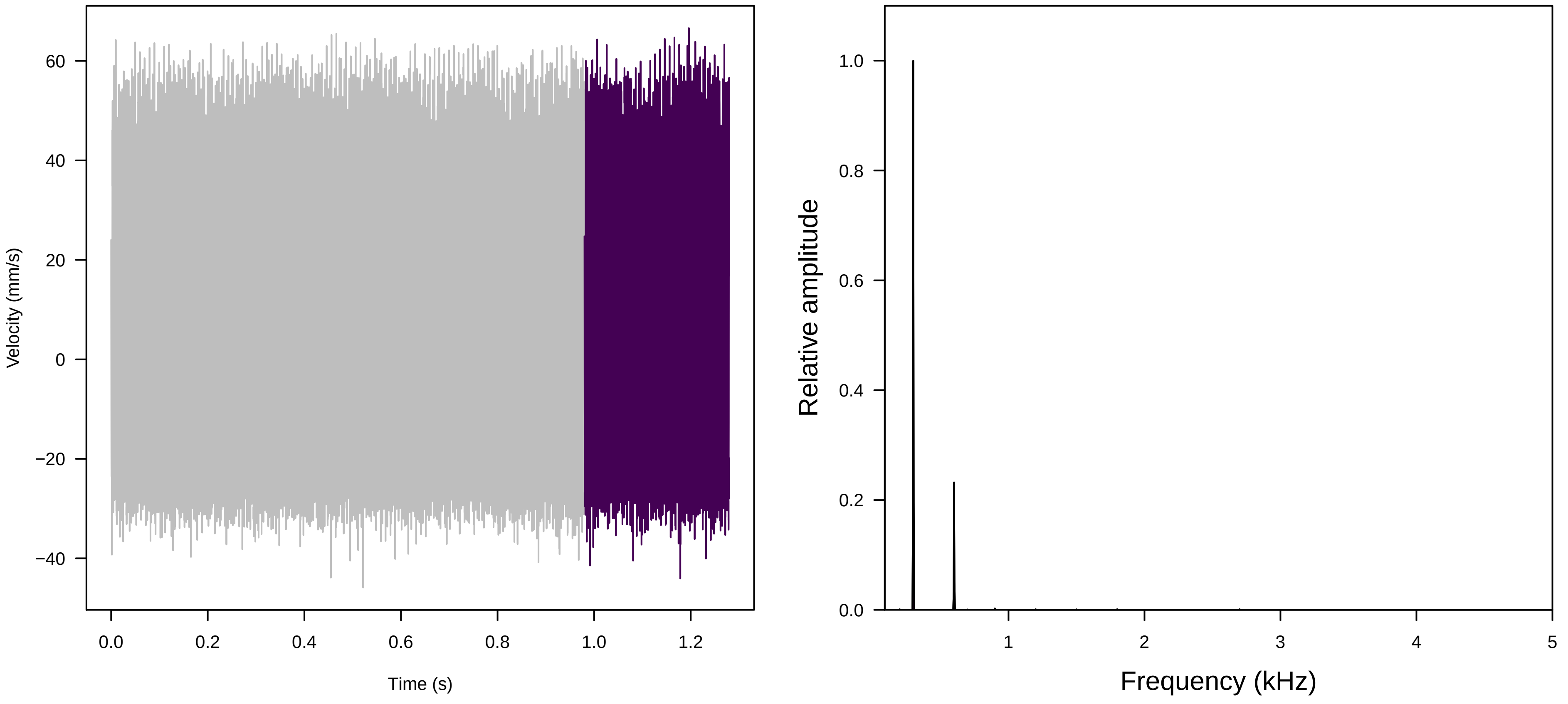

Vel. = 0.028 ; Str. = Receptacle ; Axis = x ; Fl. accession = 10−s−79−2

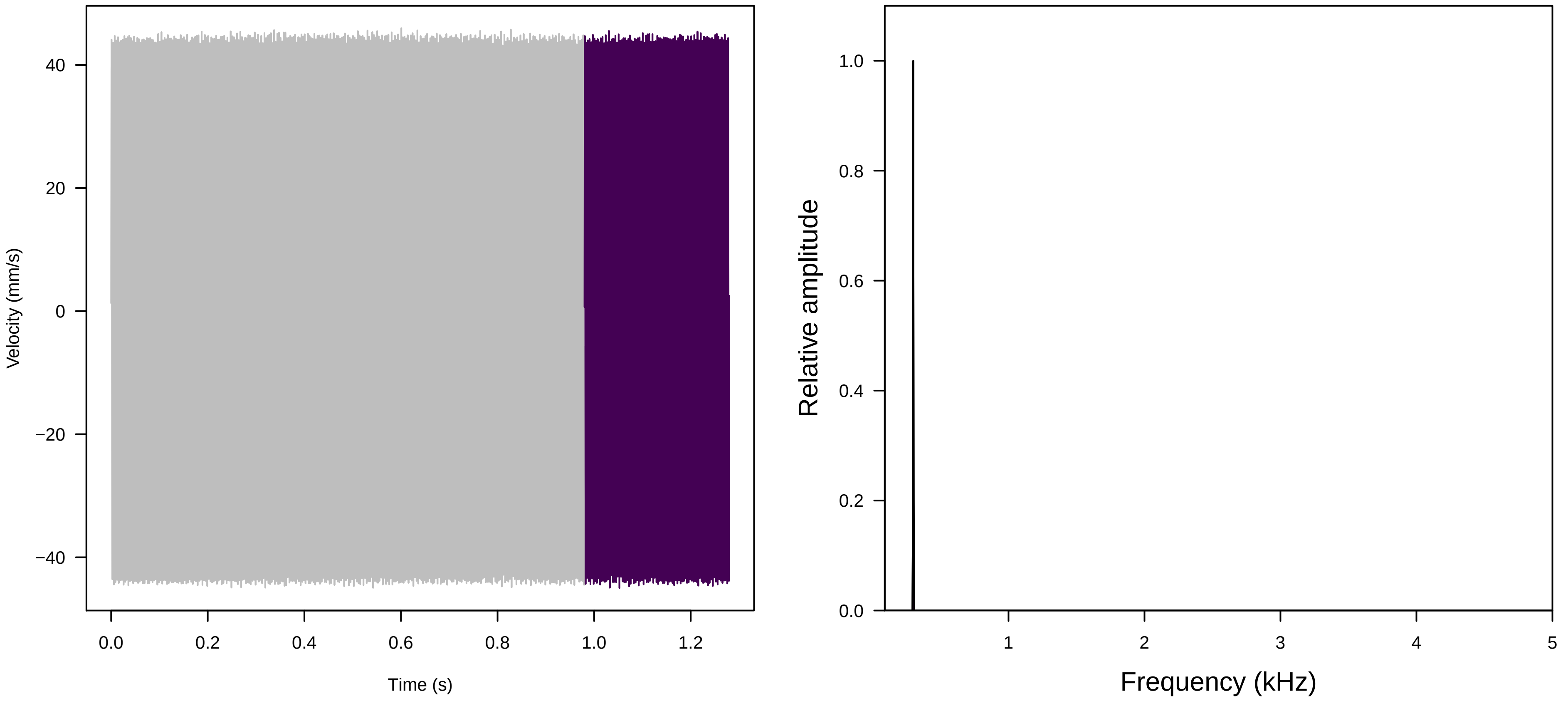

Vel. = 0.014 ; Str. = Corolla ; Axis = x ; Fl. accession = 10−s−79−2

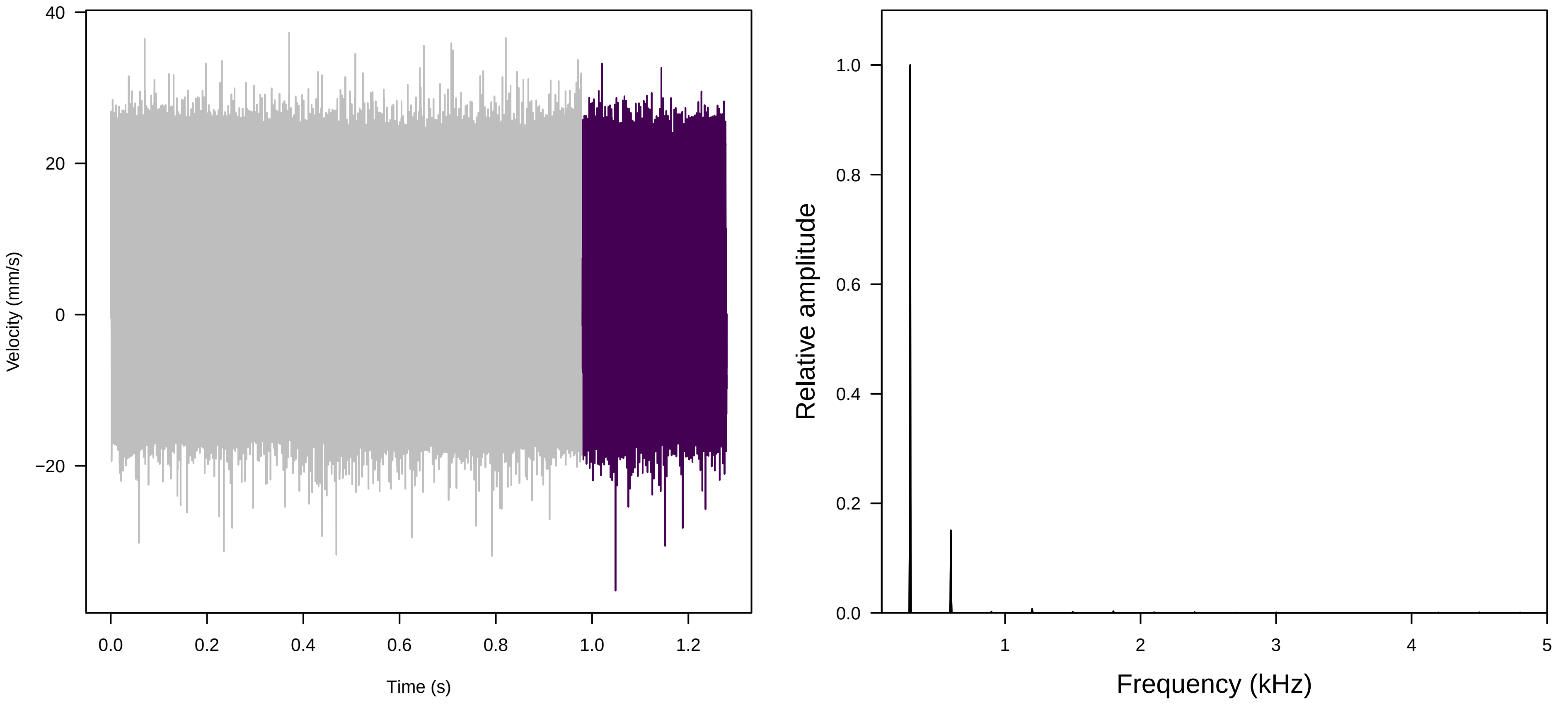

Vel. = 0.014 ; Str. = Receptacle ; Axis = x ; Fl. accession = 10−s−79−2

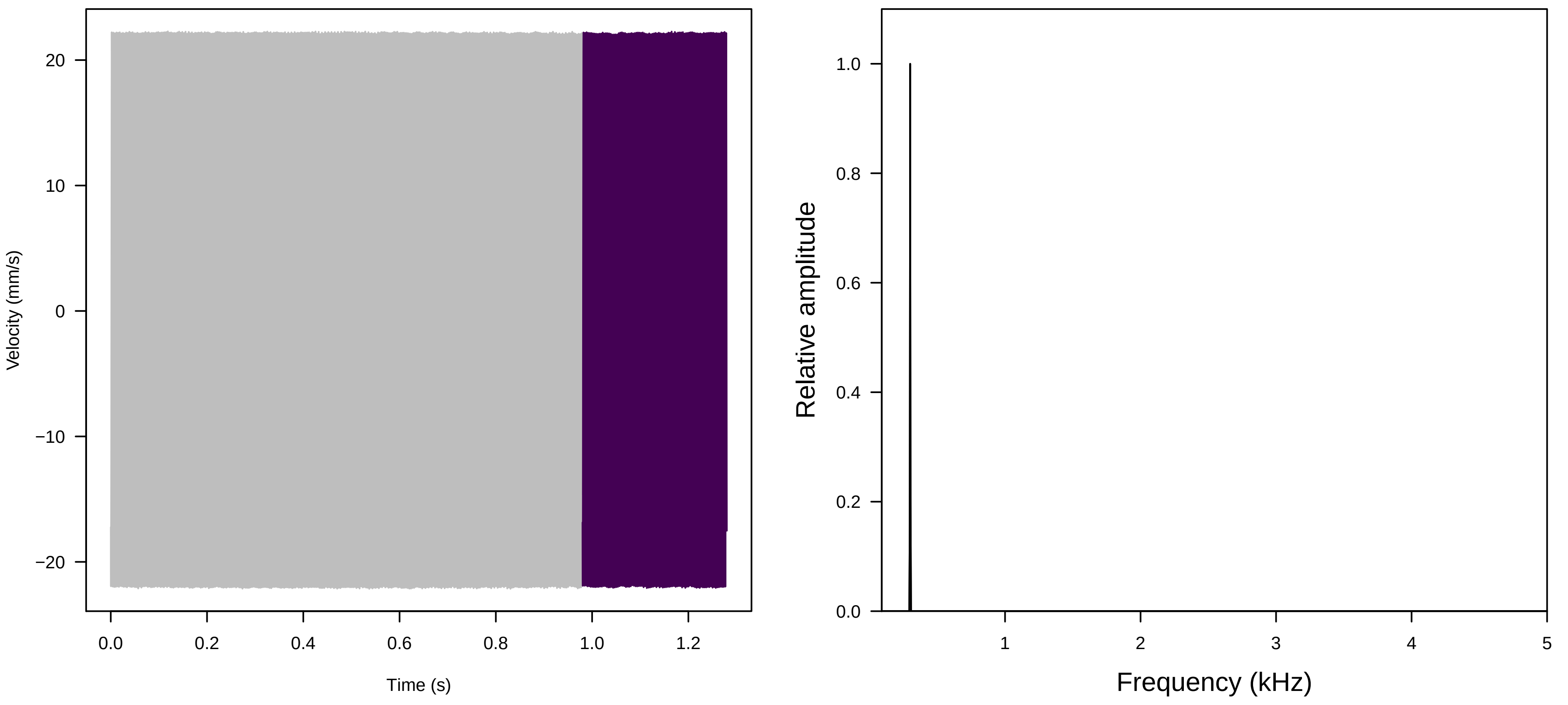

Vel. = 0.014 ; Str. = FA ; Axis = x ; Fl. accession = 10−s−79−2

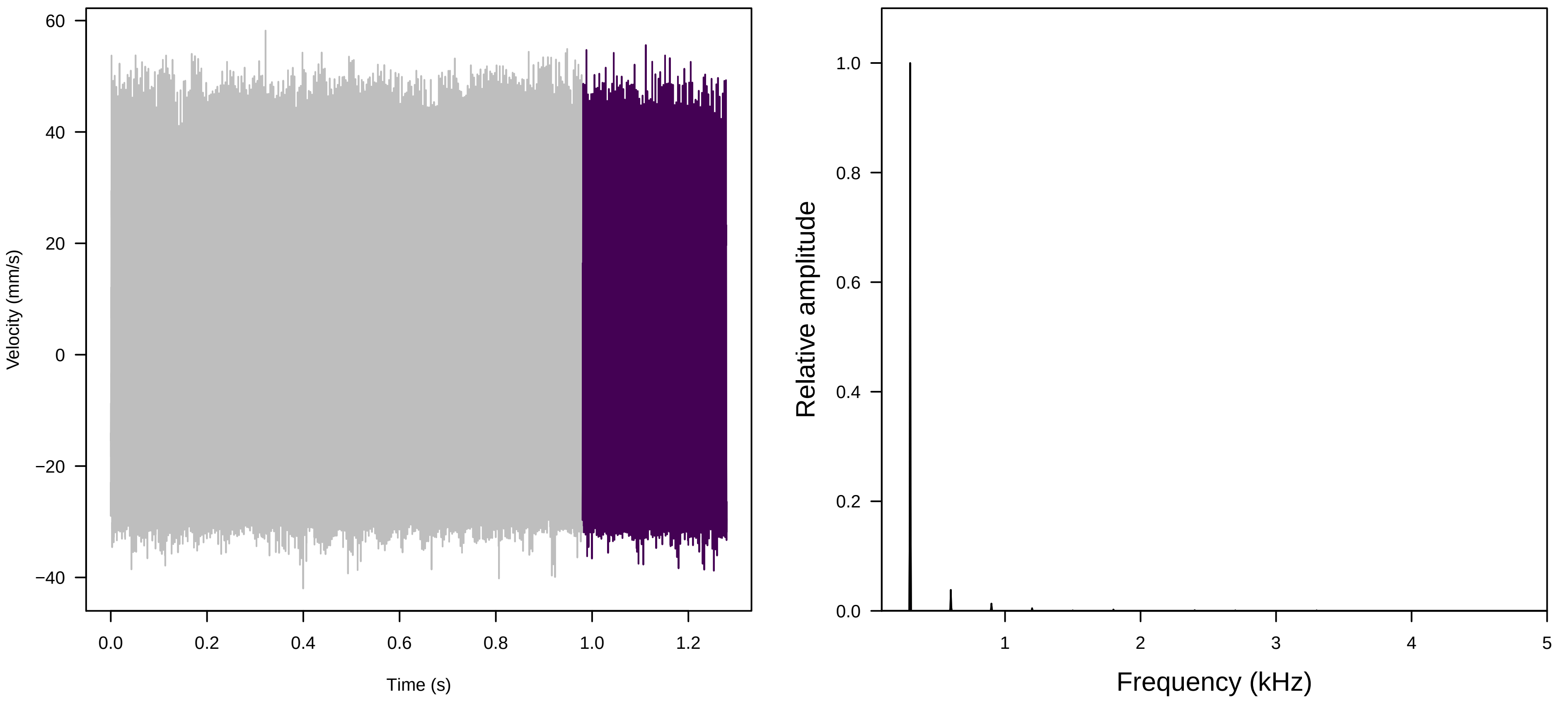

Vel. = 0.014 ; Str. = Receptacle ; Axis = x ; Fl. accession = 10−s−79−2

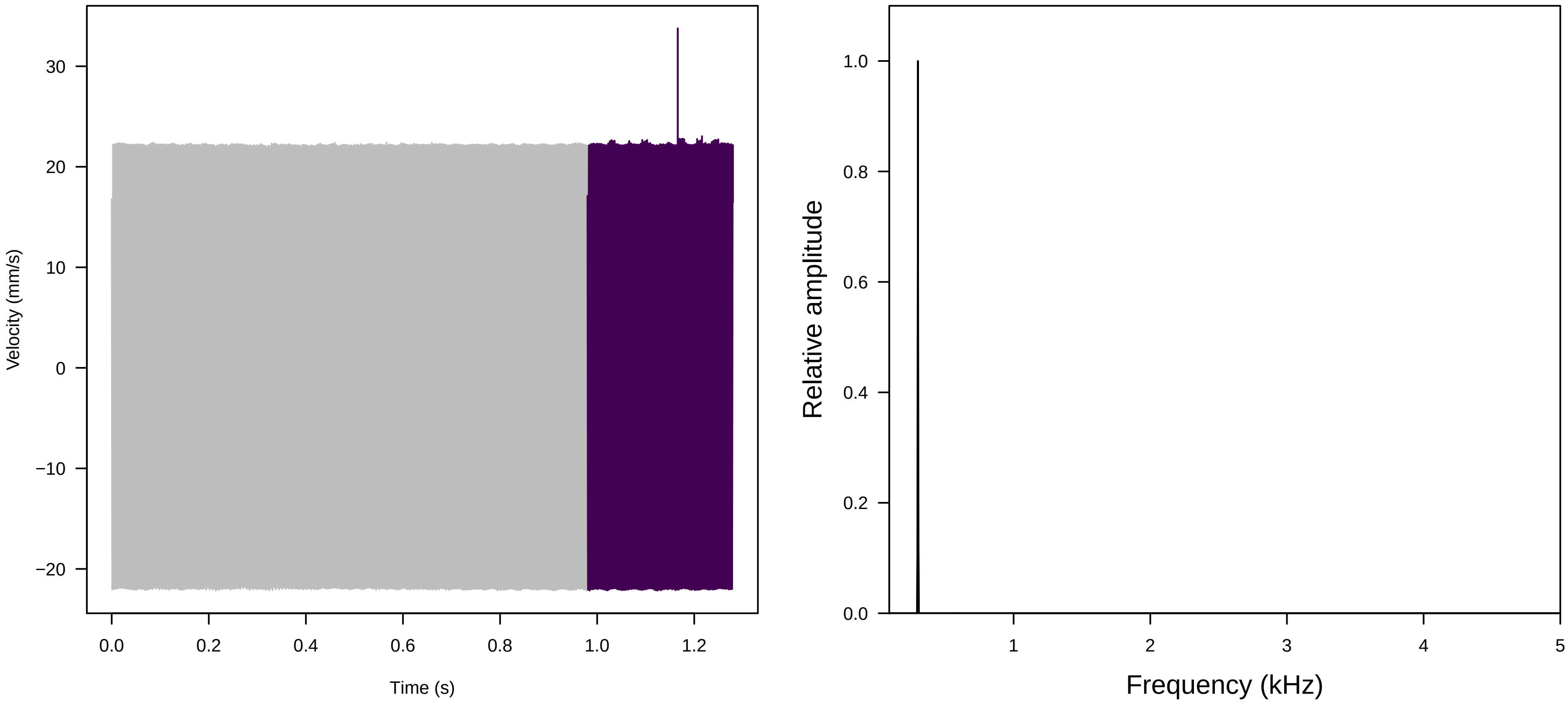

Vel. = 0.014 ; Str. = PA ; Axis = x ; Fl. accession = 10−s−79−2

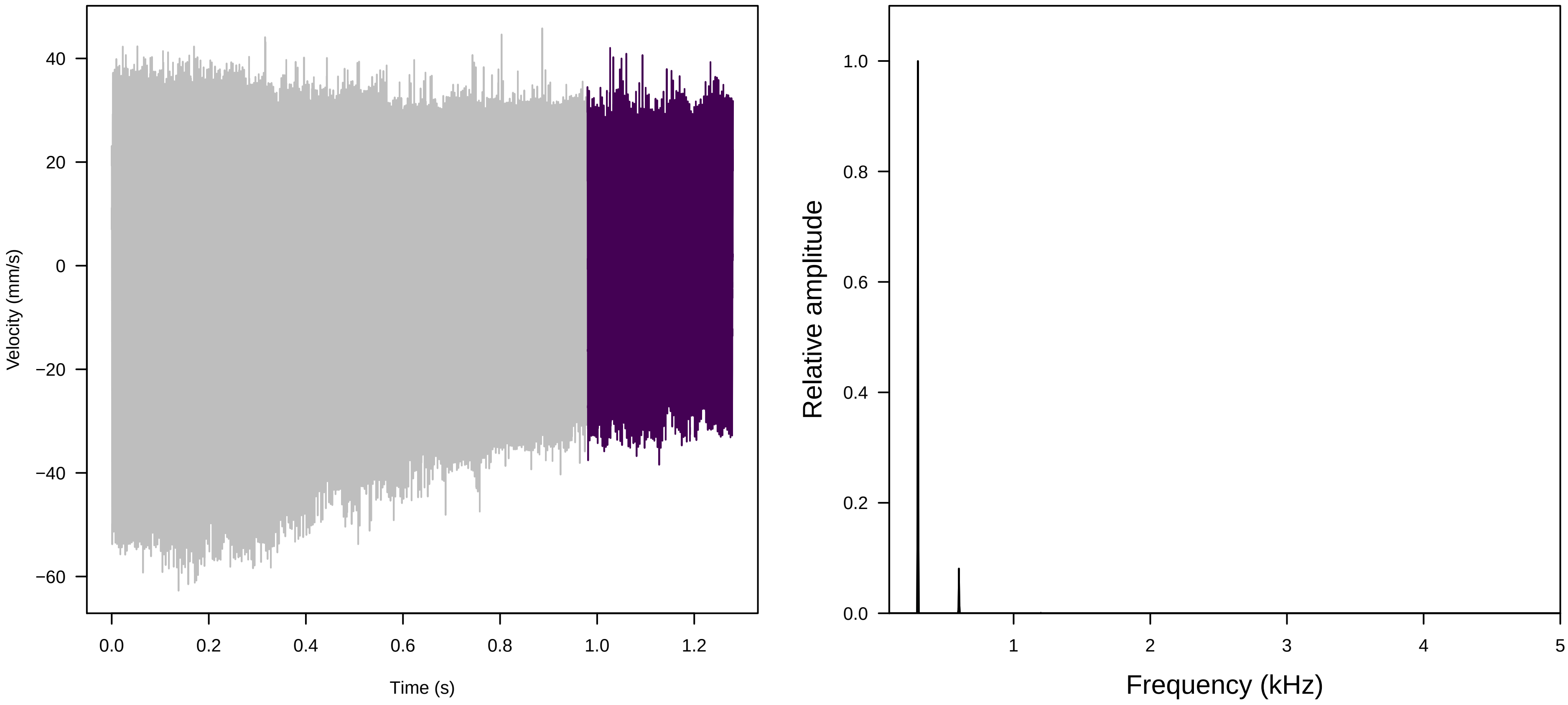

Vel. = 0.014 ; Str. = Receptacle ; Axis = x ; Fl. accession = 10−s−79−2

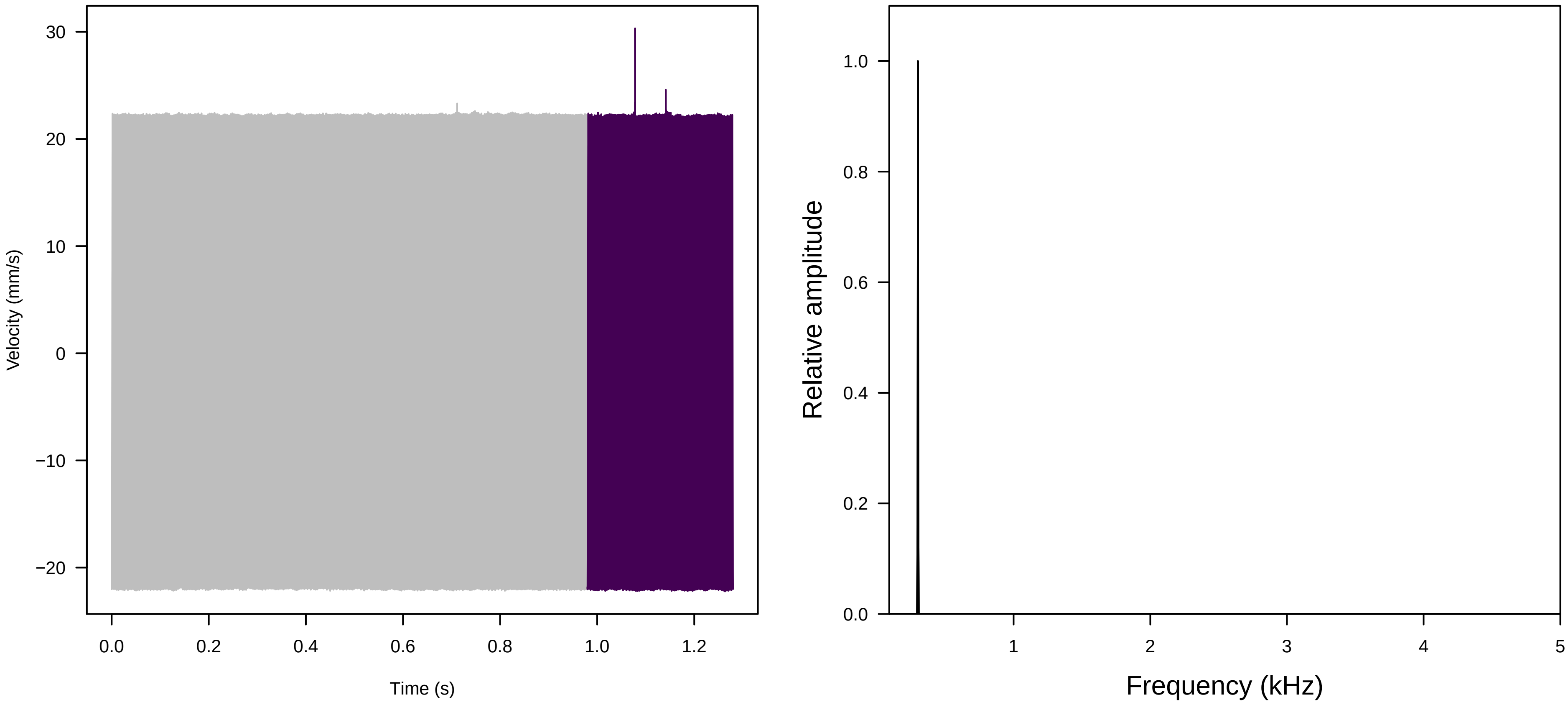

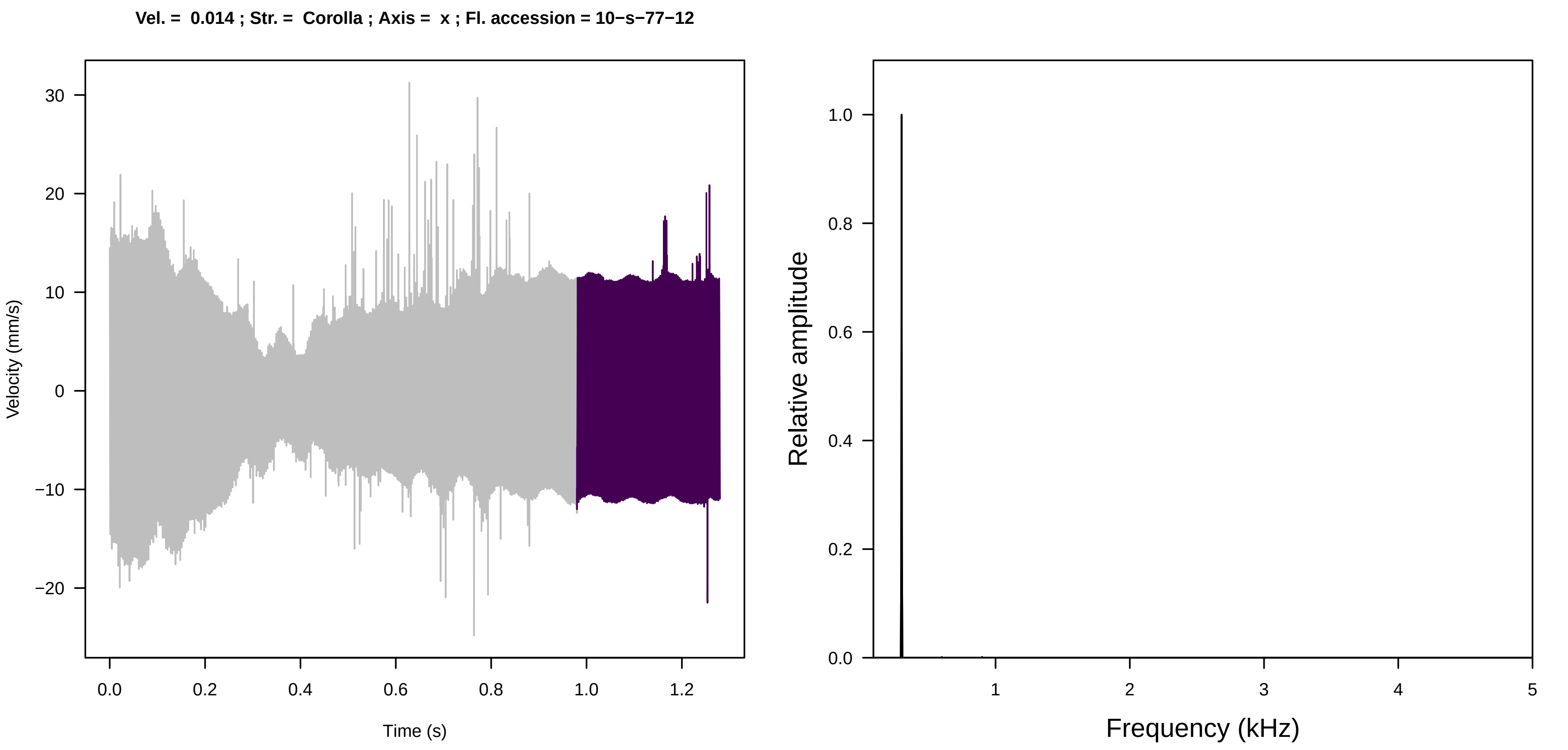
Vel. = 0.014 ; Str. = Corolla ; Axis = x ; Fl. accession = 10−s−77−12
Velocity (mm/s)
Time (s)
Relative amplitude
Frequency (kHz)

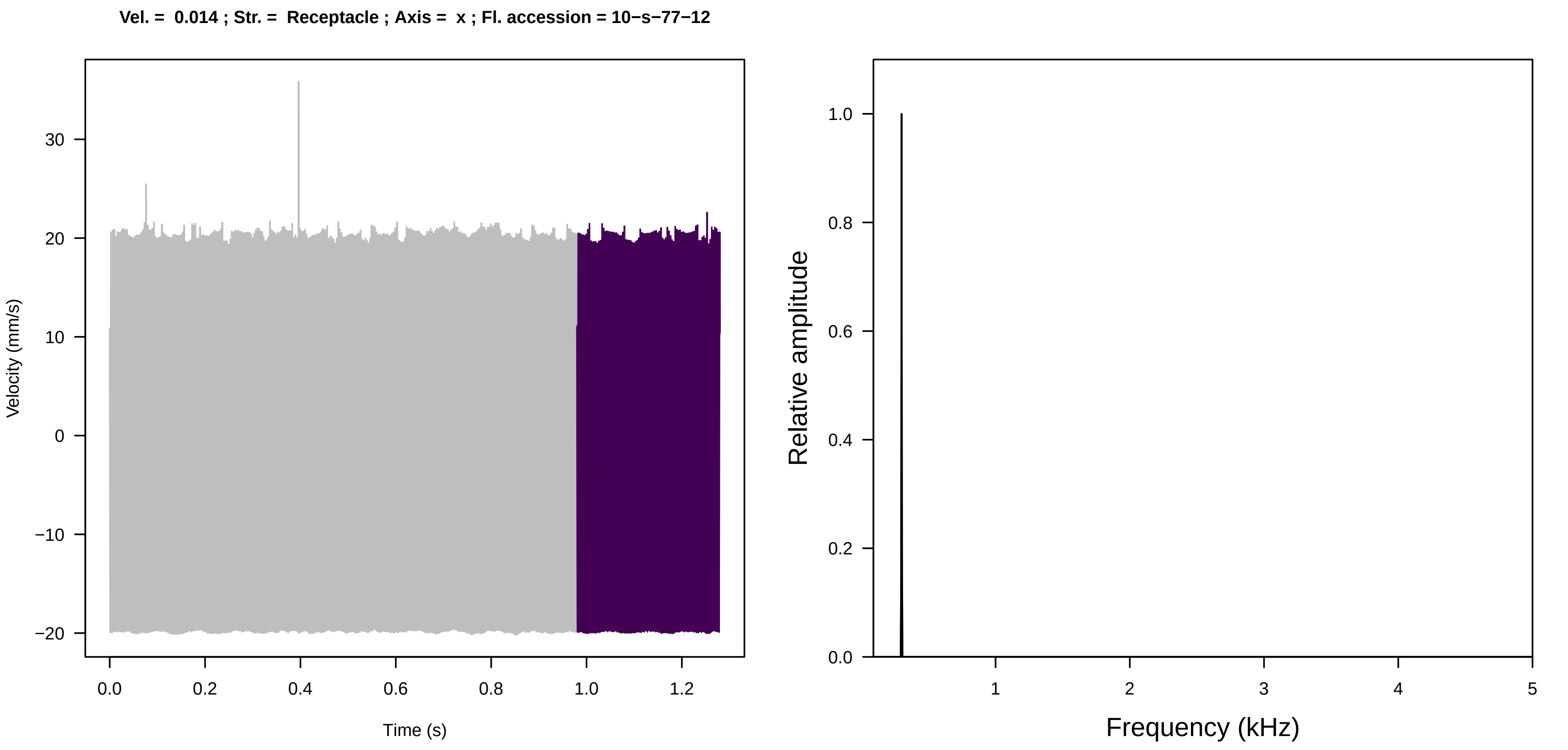
Vel. = 0.014 ; Str. = Receptacle ; Axis = x ; Fl. accession = 10−s−77−12
Velocity (mm/s)
Time (s)
Relative amplitude
Frequency (kHz)

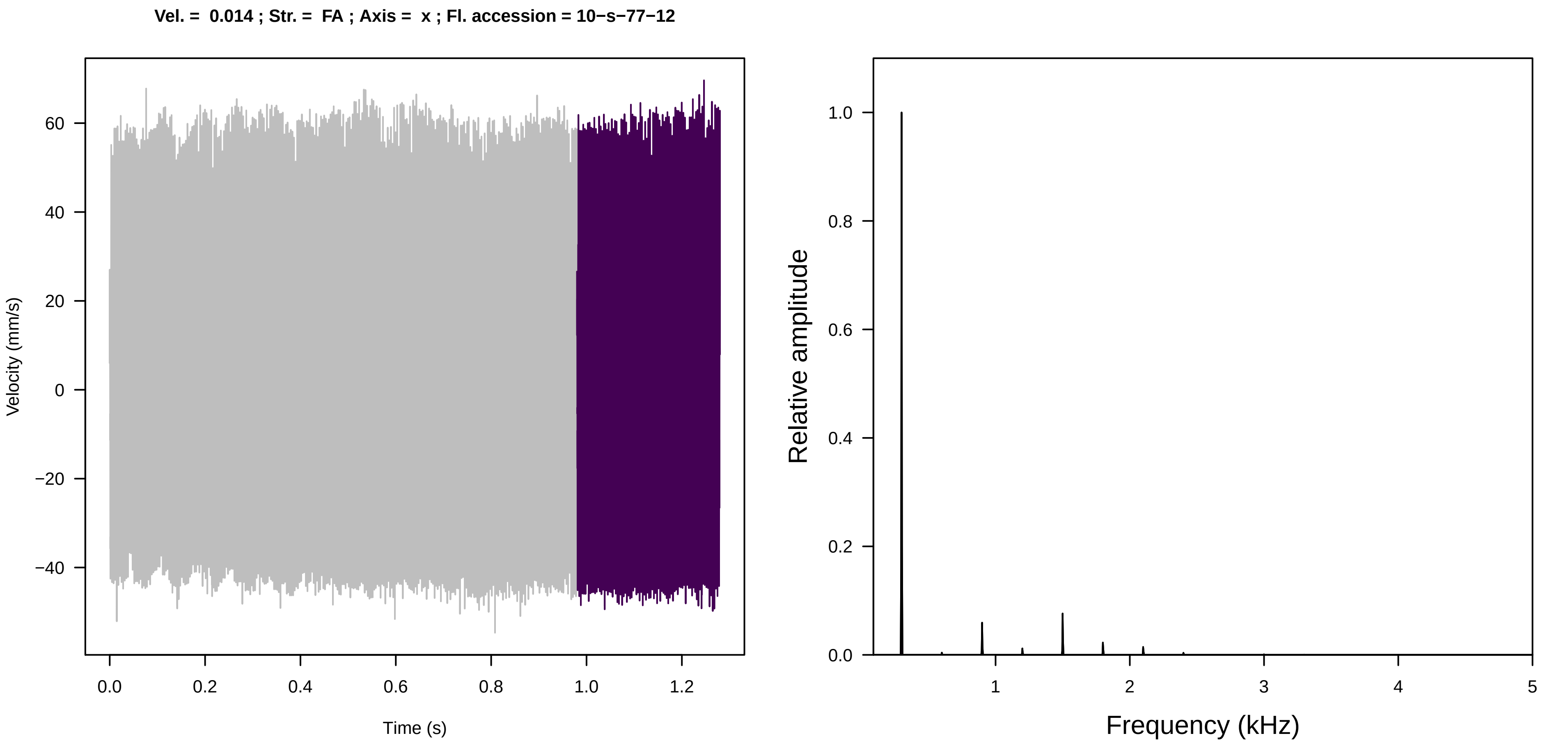
Vel. = 0.014 ; Str. = FA ; Axis = x ; Fl. accession = 10−s−77−12
Velocity (mm/s)
Time (s)
Relative amplitude
Frequency (kHz)

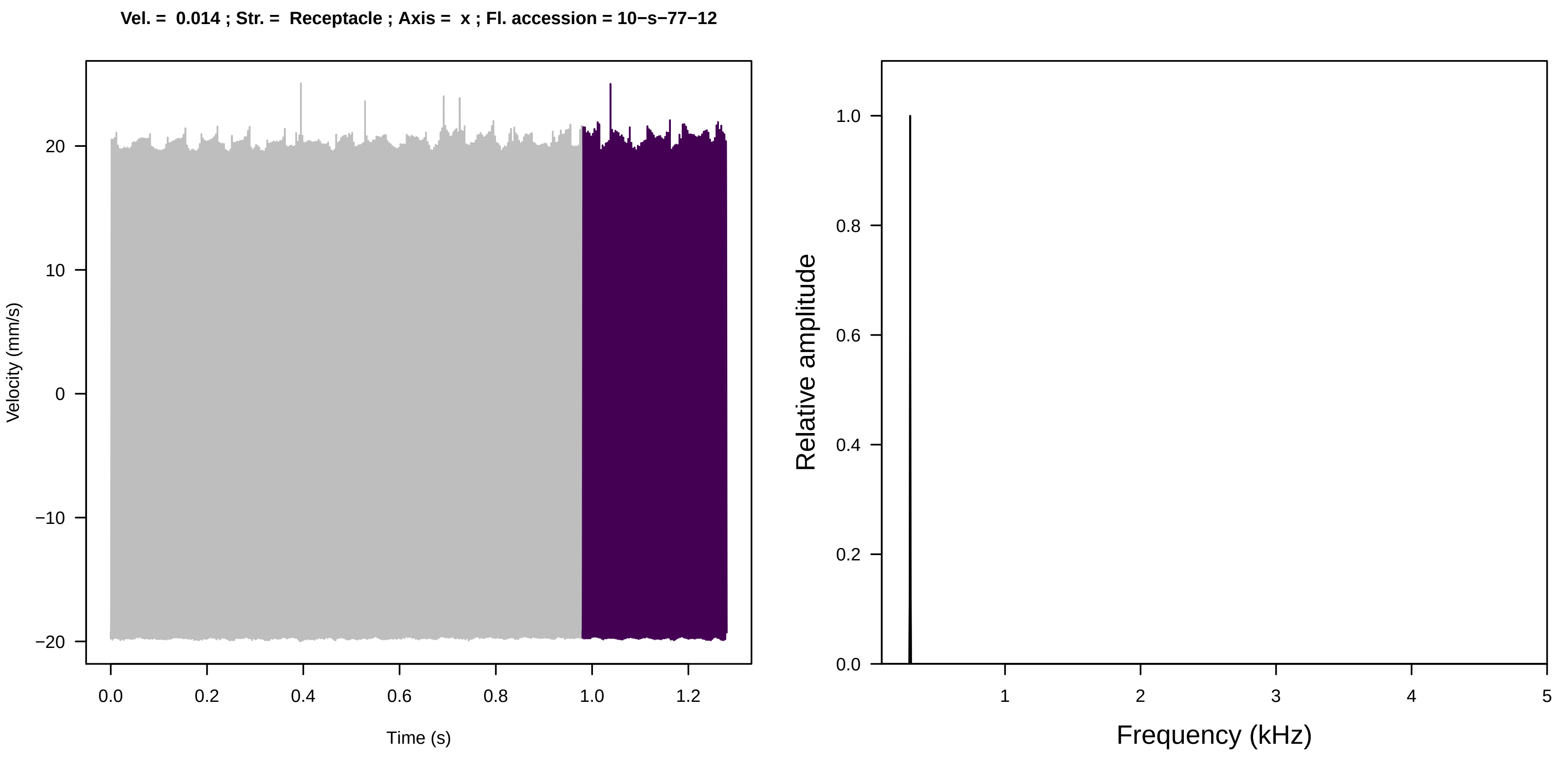
Vel. = 0.014 ; Str. = Receptacle ; Axis = x ; Fl. accession = 10−s−77−12
Velocity (mm/s)
20
10
0
−10
−20
0.0
0.2
0.4
0.6
0.8
1.0
1.2
Time (s)
Relative amplitude
1.0
0.8
0.6
0.4
0.2
0.0
1
2
3
4
5
Frequency (kHz)

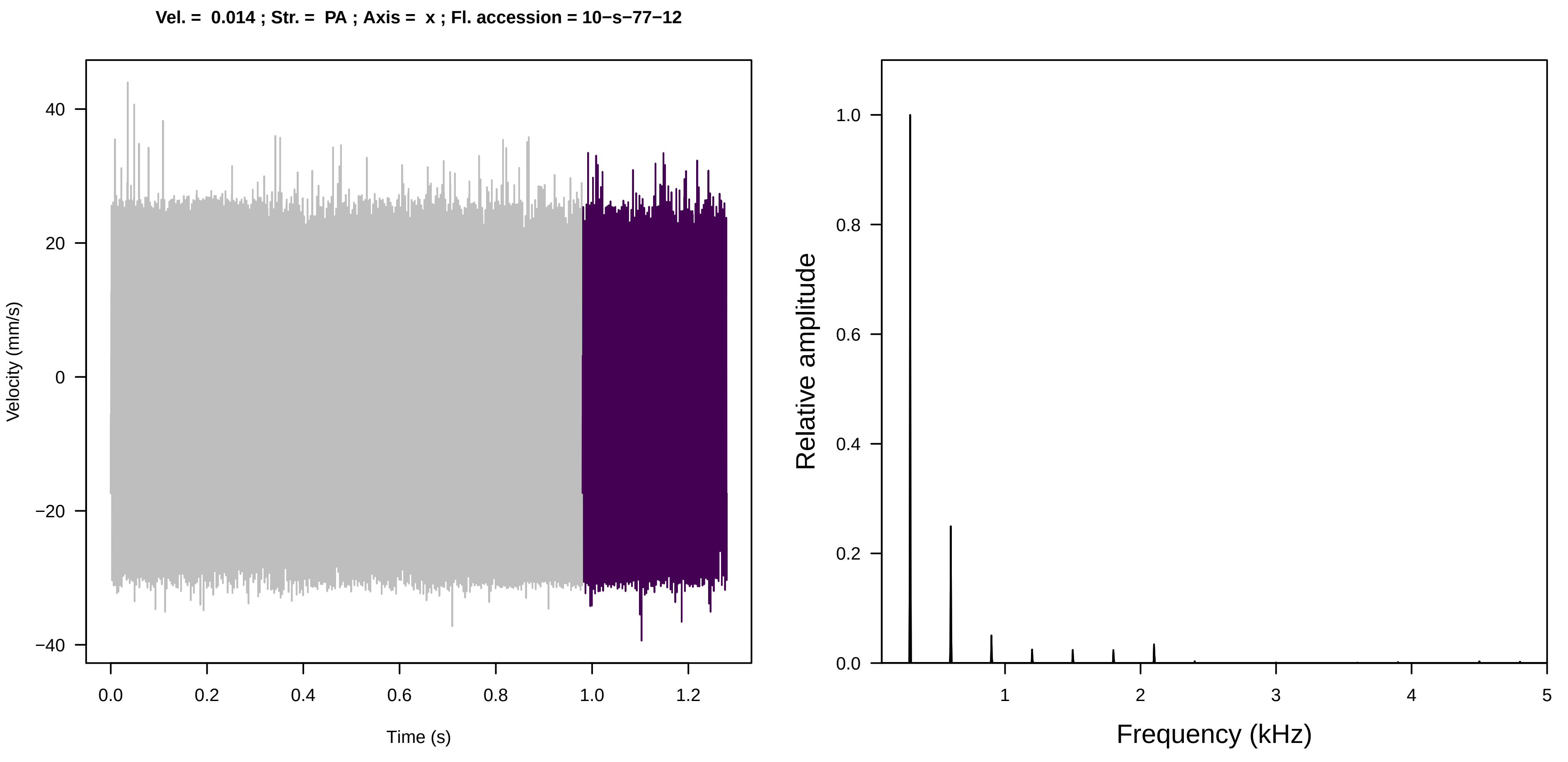
Vel. = 0.014 ; Str. = PA ; Axis = x ; Fl. accession = 10−s−77−12
Velocity (mm/s)
40
20
0
−20
−40
0.0
0.2
0.4
0.6
0.8
1.0
1.2
Time (s)
Relative amplitude
1.0
0.8
0.6
0.4
0.2
0.0
1
2
3
4
5
Frequency (kHz)

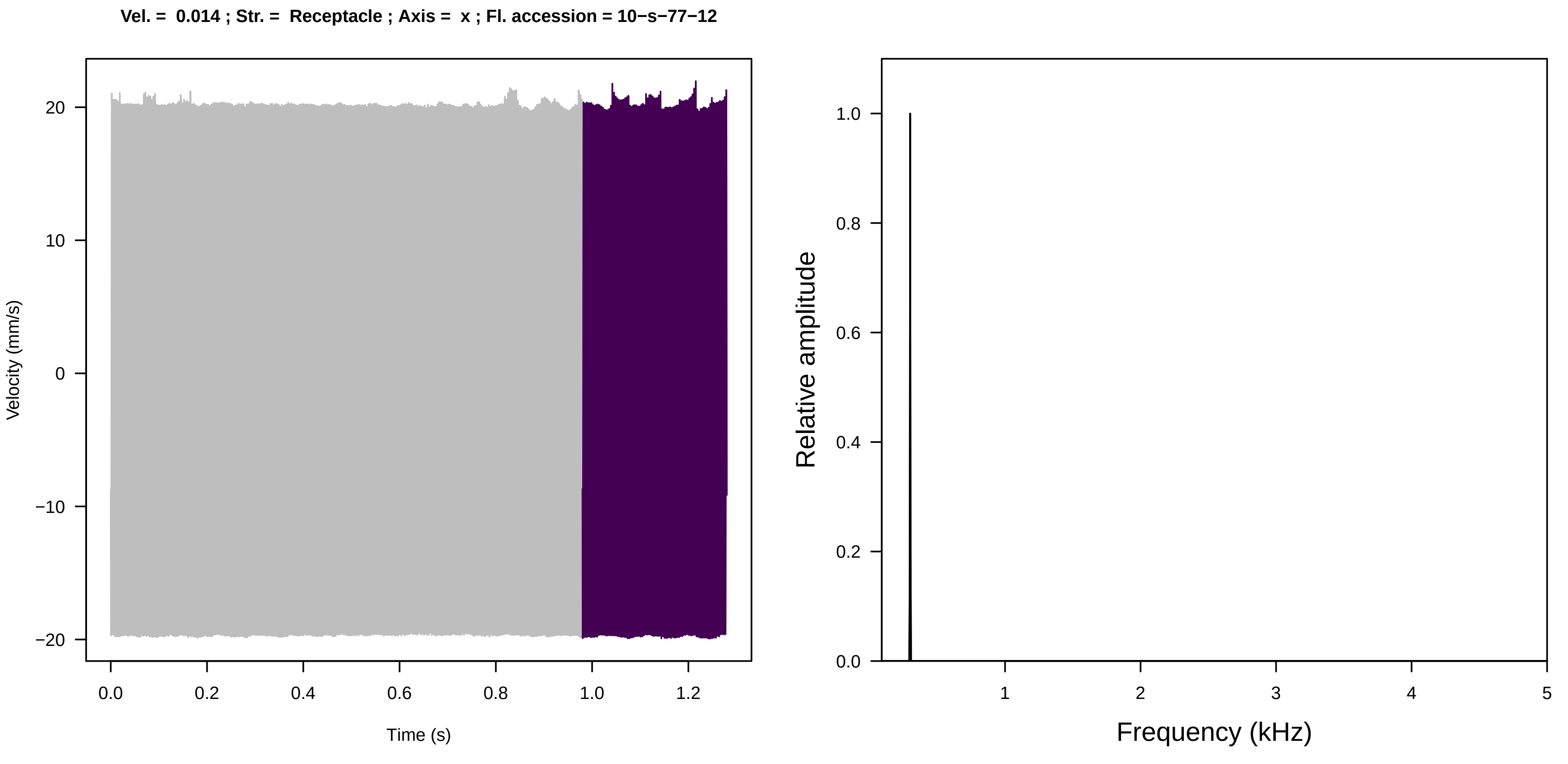
Vel. = 0.014 ; Str. = Receptacle ; Axis = x ; Fl. accession = 10−s−77−12
Velocity (mm/s)
20
10
0
−10
−20
0.0
0.2
0.4
0.6
0.8
1.0
1.2
Time (s)
Relative amplitude
1.0
0.8
0.6
0.4
0.2
0.0
1
2
3
4
5
Frequency (kHz)

Vel. = 0.028 ; Str. = PA ; Axis = x ; Fl. accession = 10−s−77−12

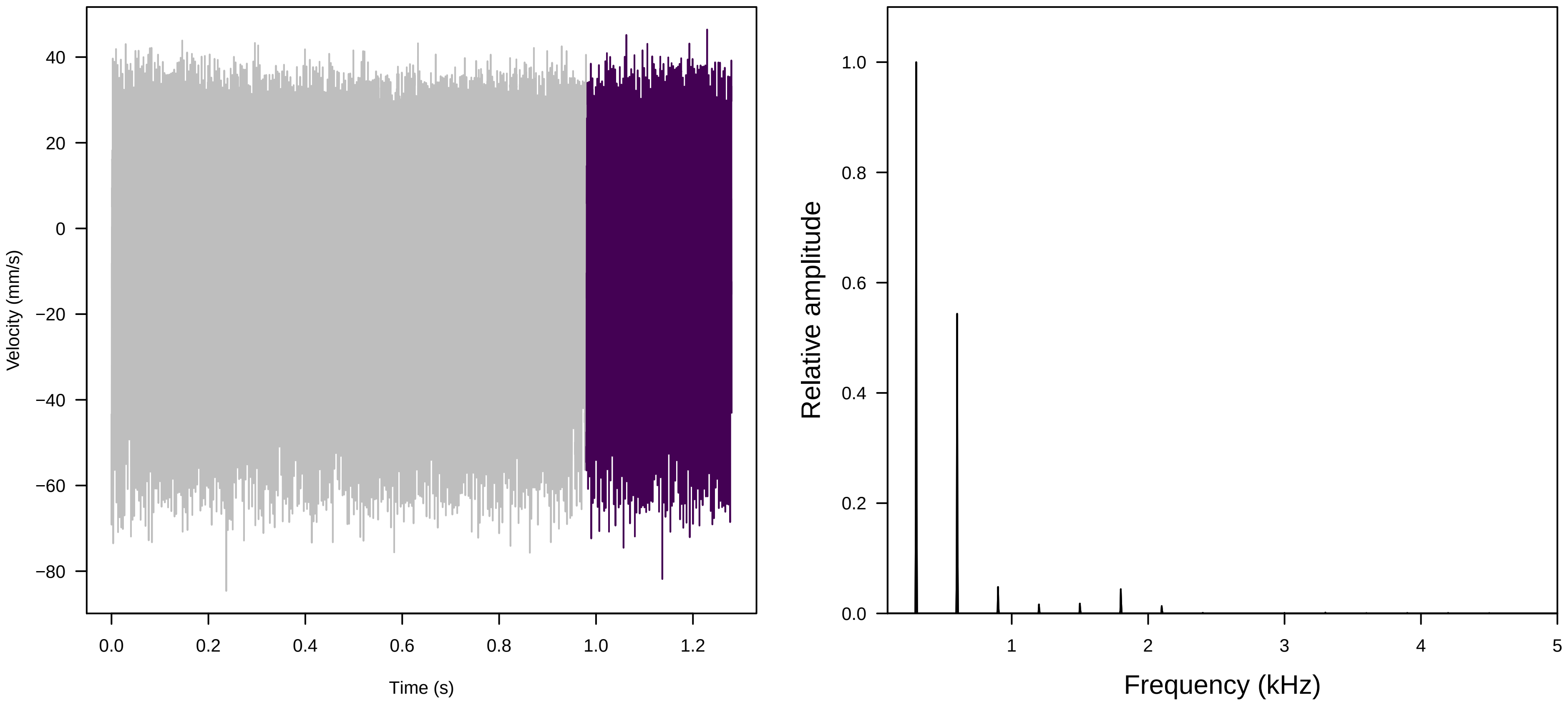

Vel. = 0.028 ; Str. = Receptacle ; Axis = x ; Fl. accession = 10−s−77−12

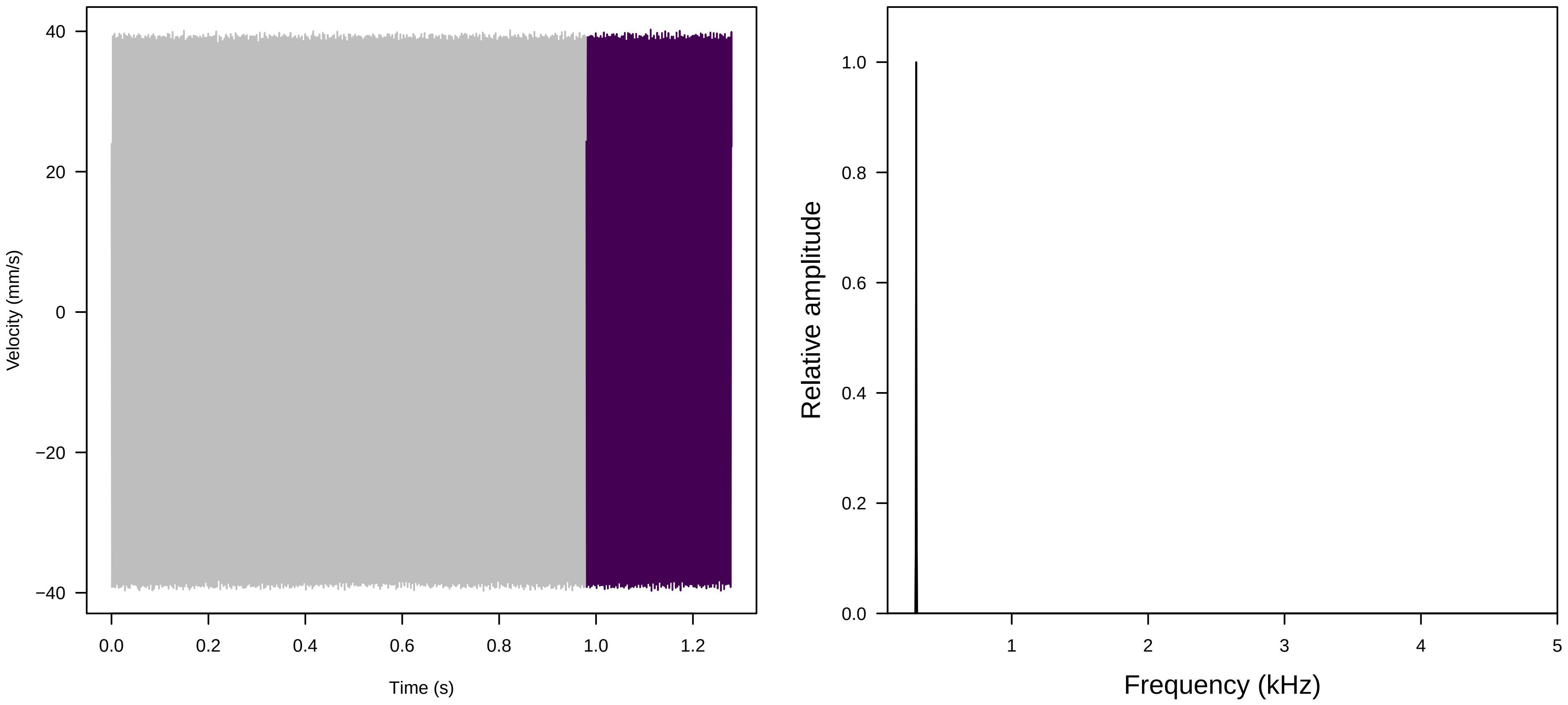

Vel. = 0.028 ; Str. = FA ; Axis = x ; Fl. accession = 10−s−77−12

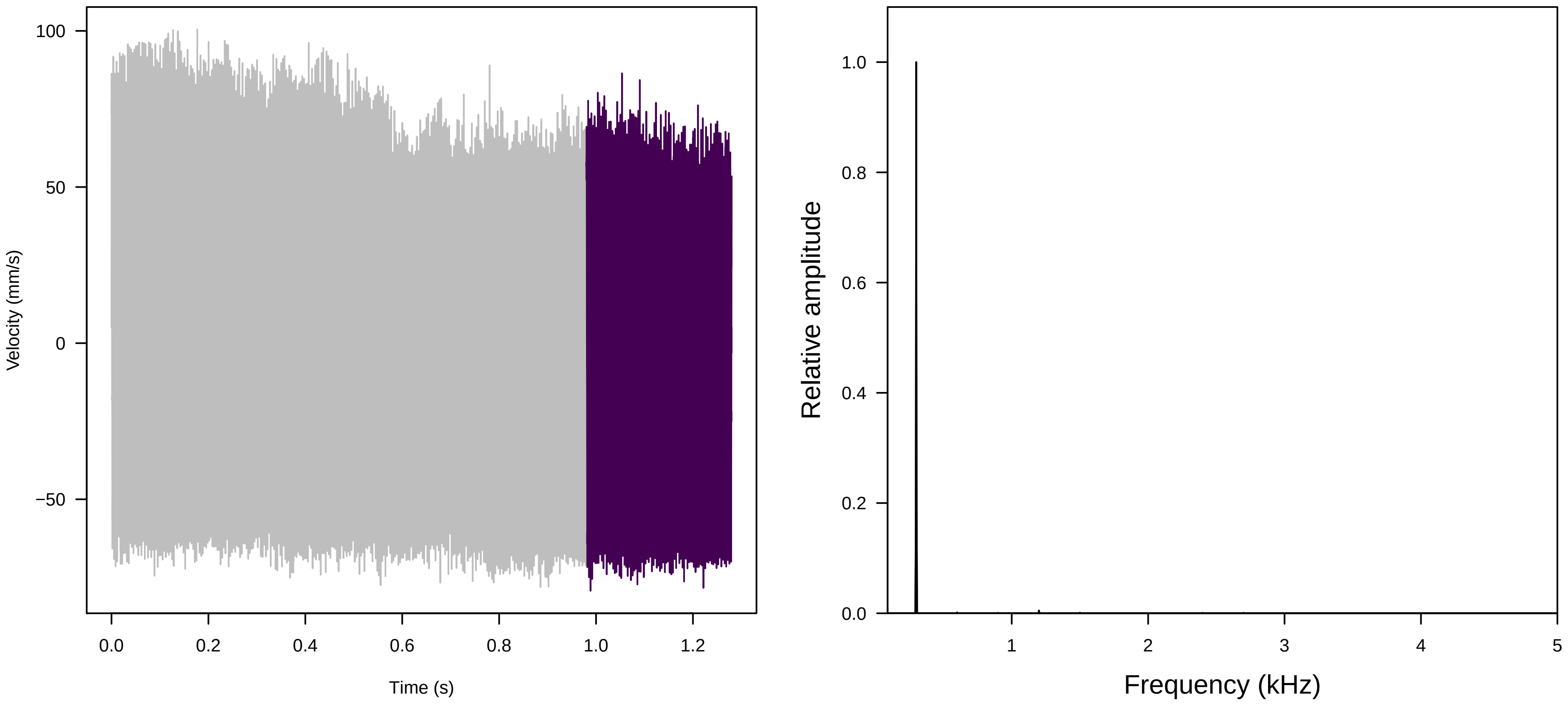

Vel. = 0.028 ; Str. = Receptacle ; Axis = x ; Fl. accession = 10−s−77−12

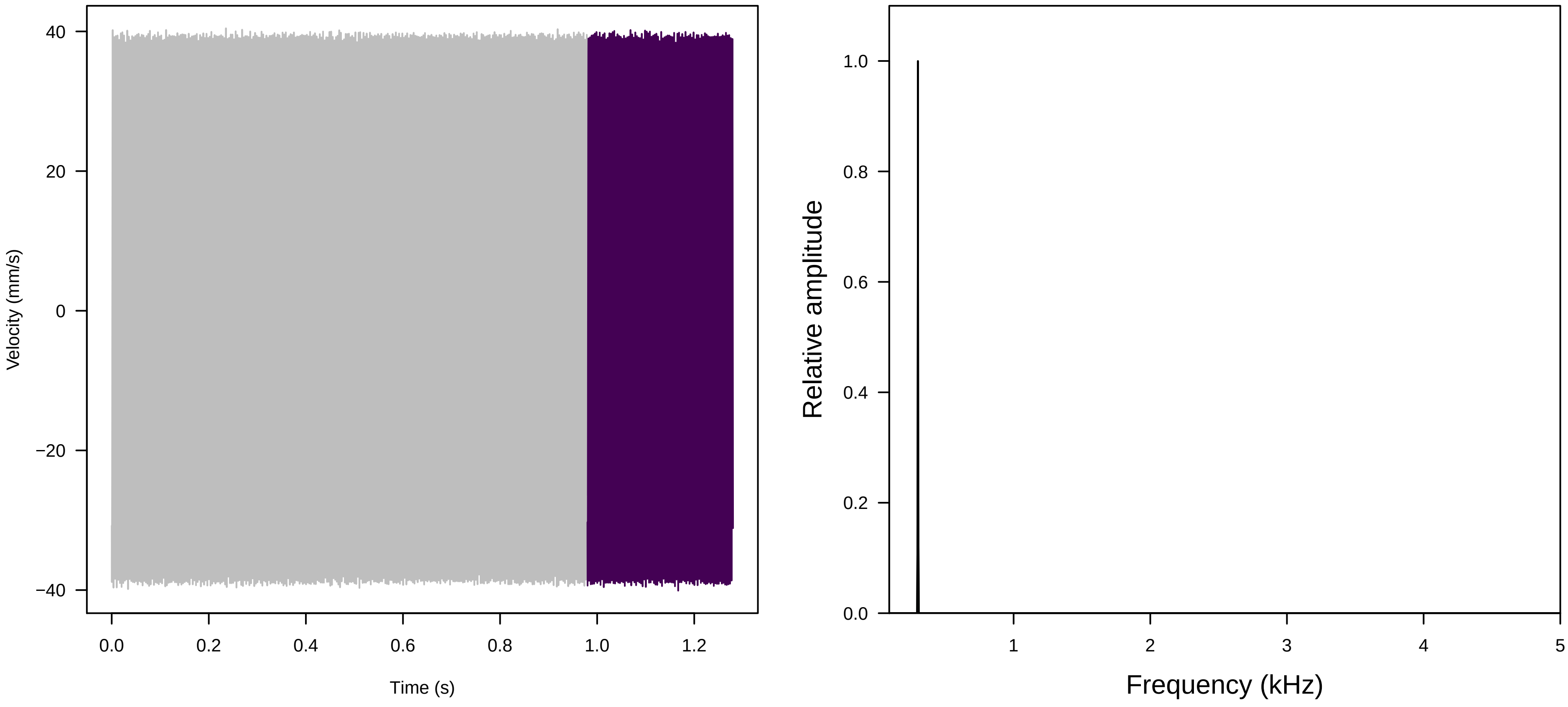

Vel. = 0.028 ; Str. = Corolla ; Axis = x ; Fl. accession = 10−s−77−12

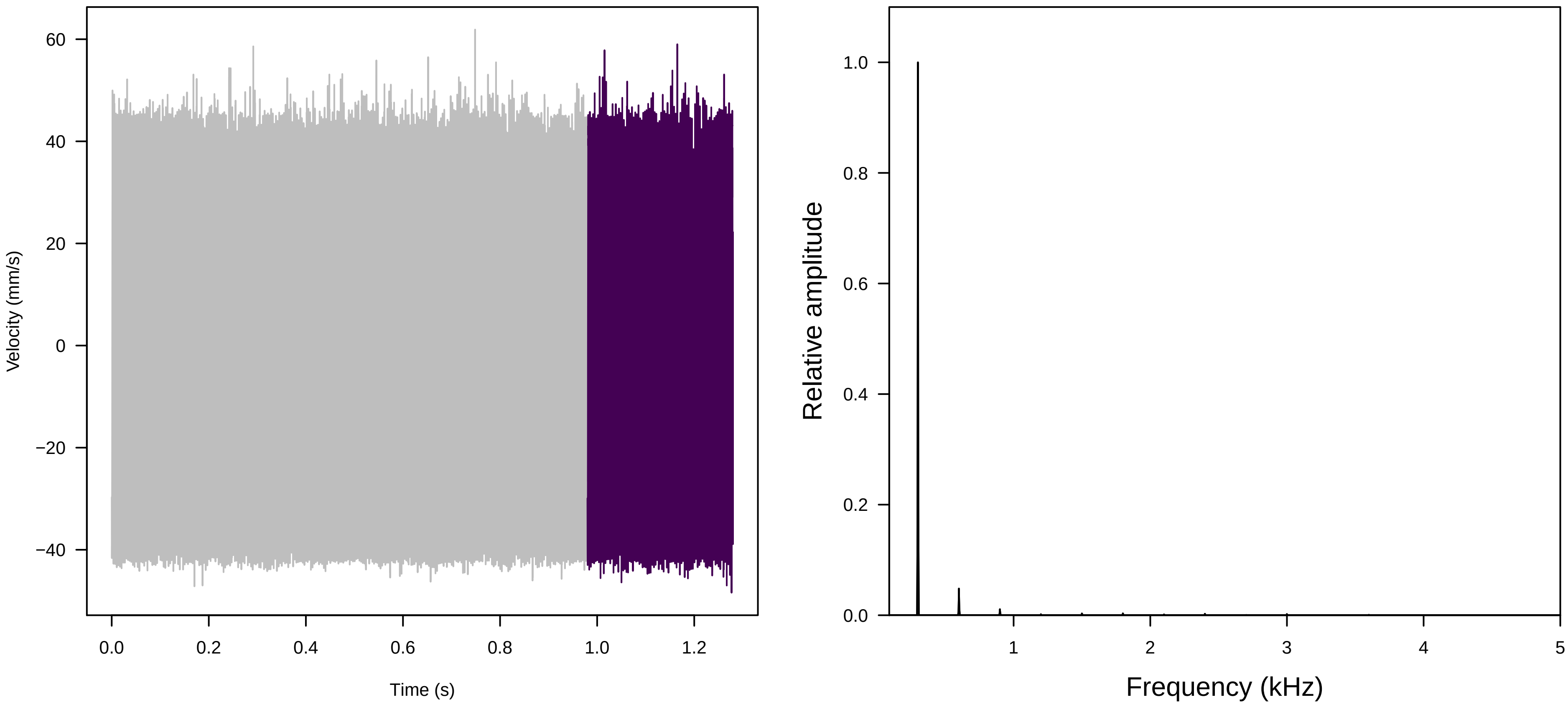

Vel. = 0.028 ; Str. = Receptacle ; Axis = x ; Fl. accession = 10−s−77−12

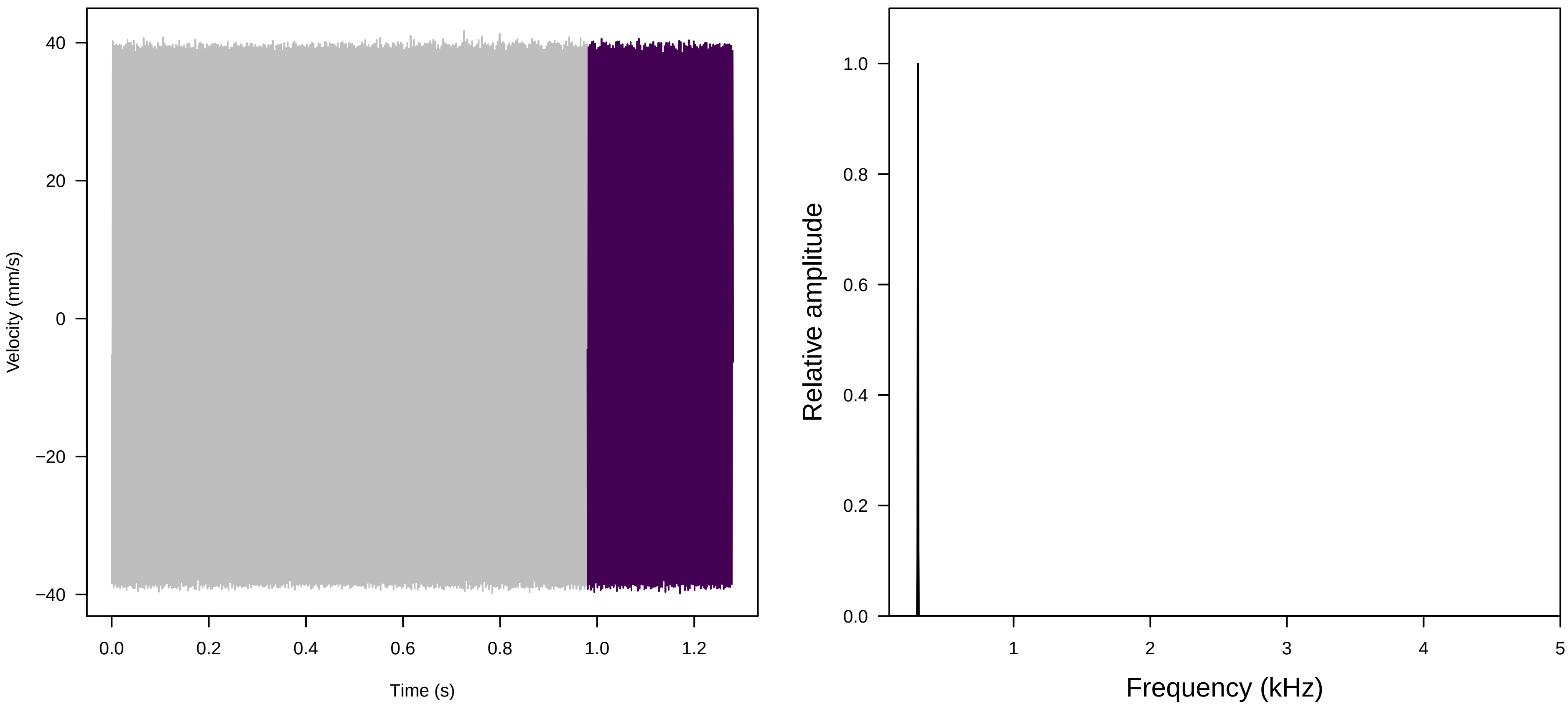

Vel. = 0.057 ; Str. = Corolla ; Axis = x ; Fl. accession = 10−s−77−12

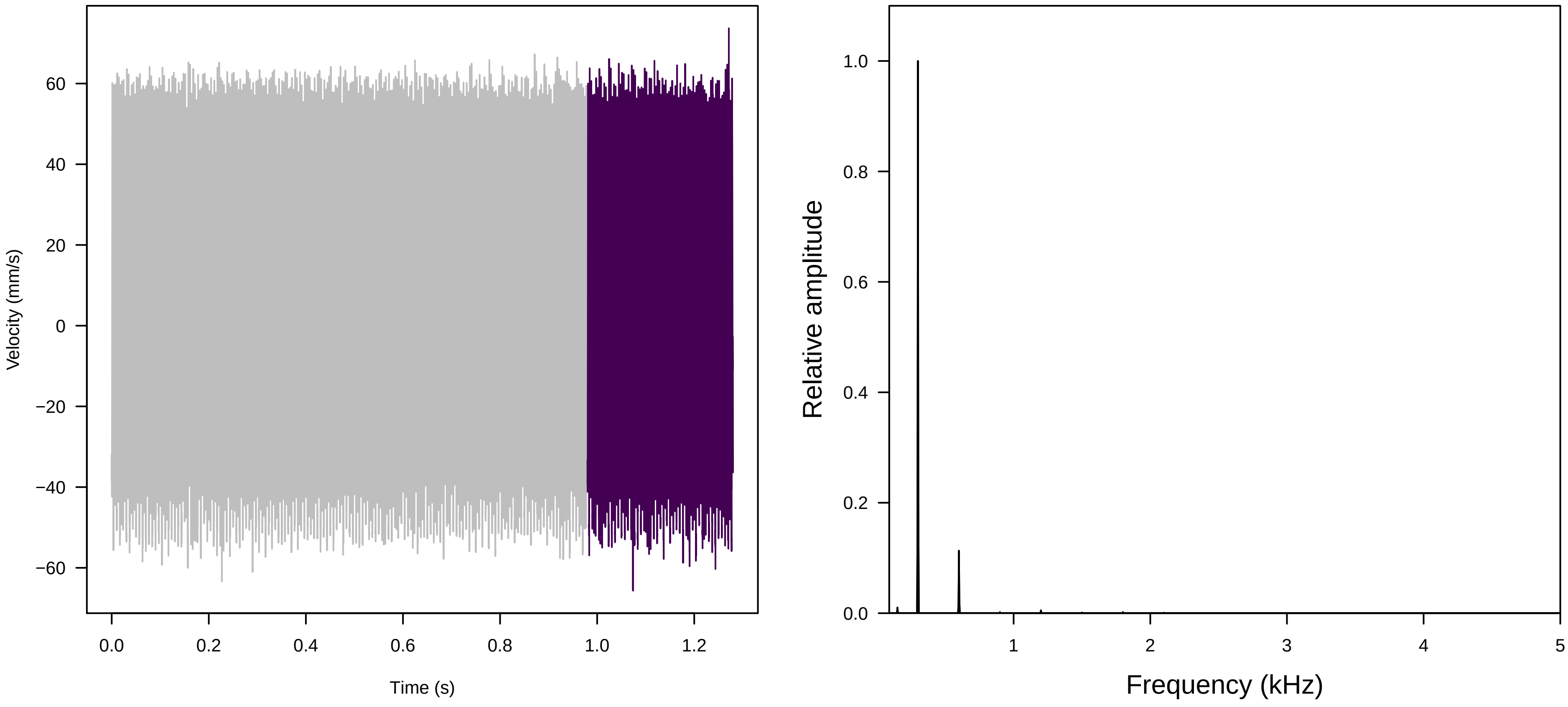

Vel. = 0.057 ; Str. = Receptacle ; Axis = x ; Fl. accession = 10−s−77−12

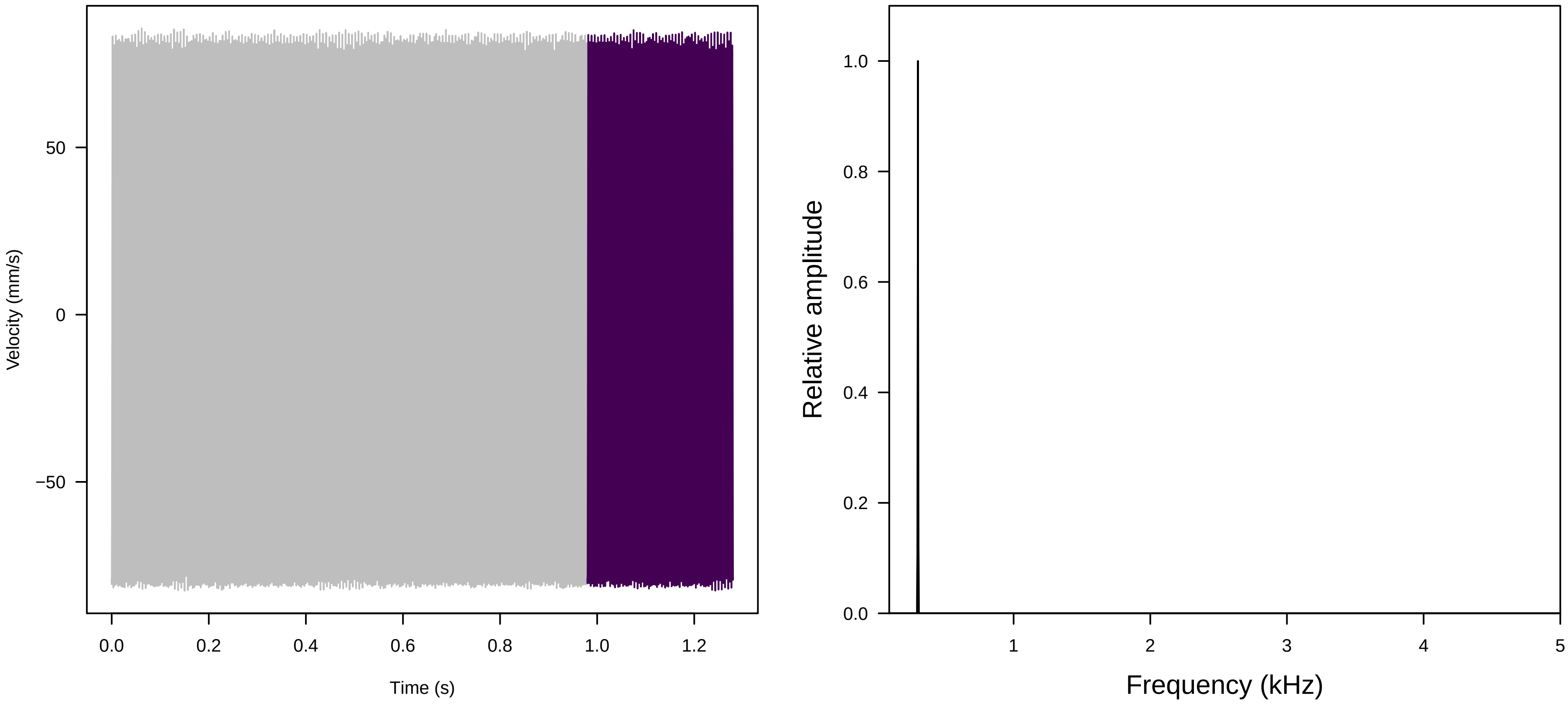

Vel. = 0.057 ; Str. = FA ; Axis = x ; Fl. accession = 10−s−77−12

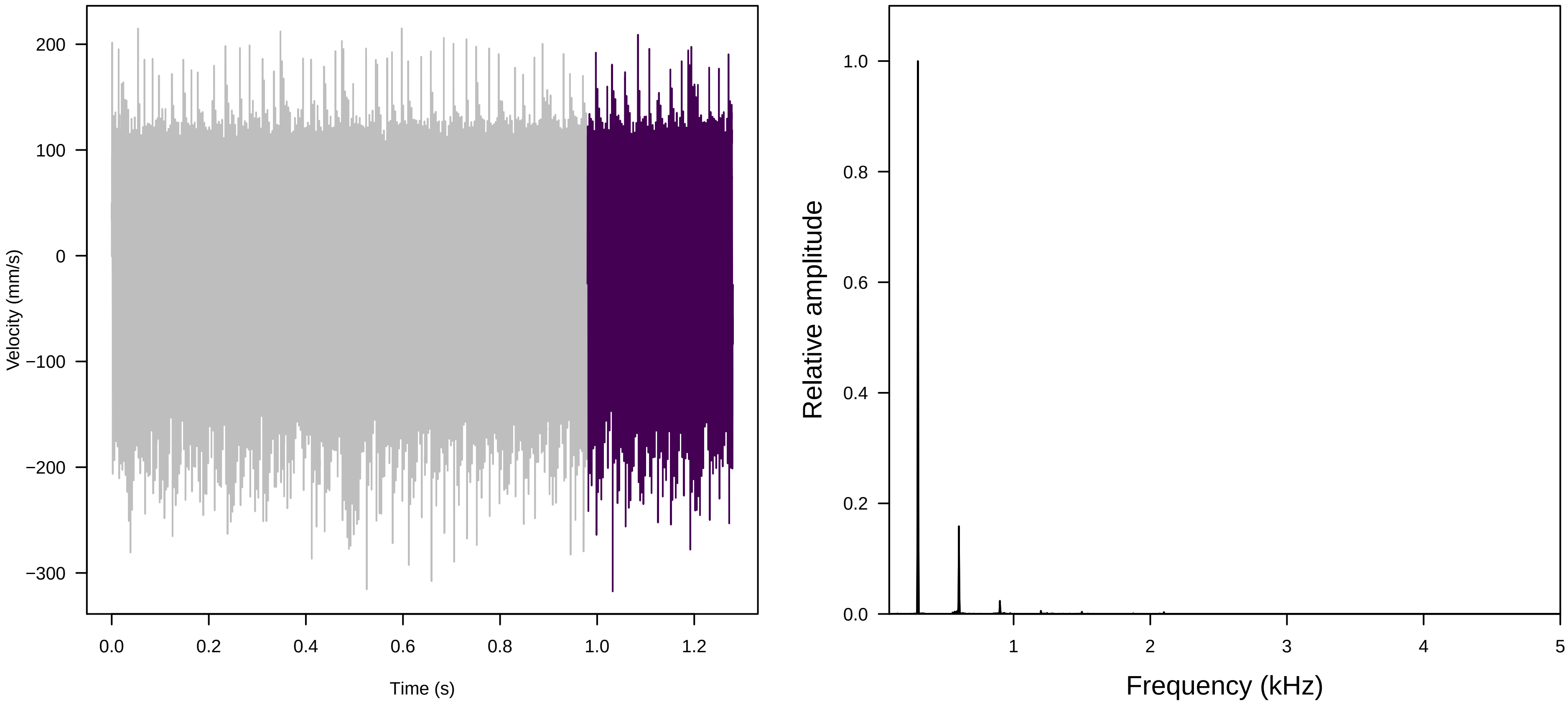

Vel. = 0.057 ; Str. = Receptacle ; Axis = x ; Fl. accession = 10−s−77−12

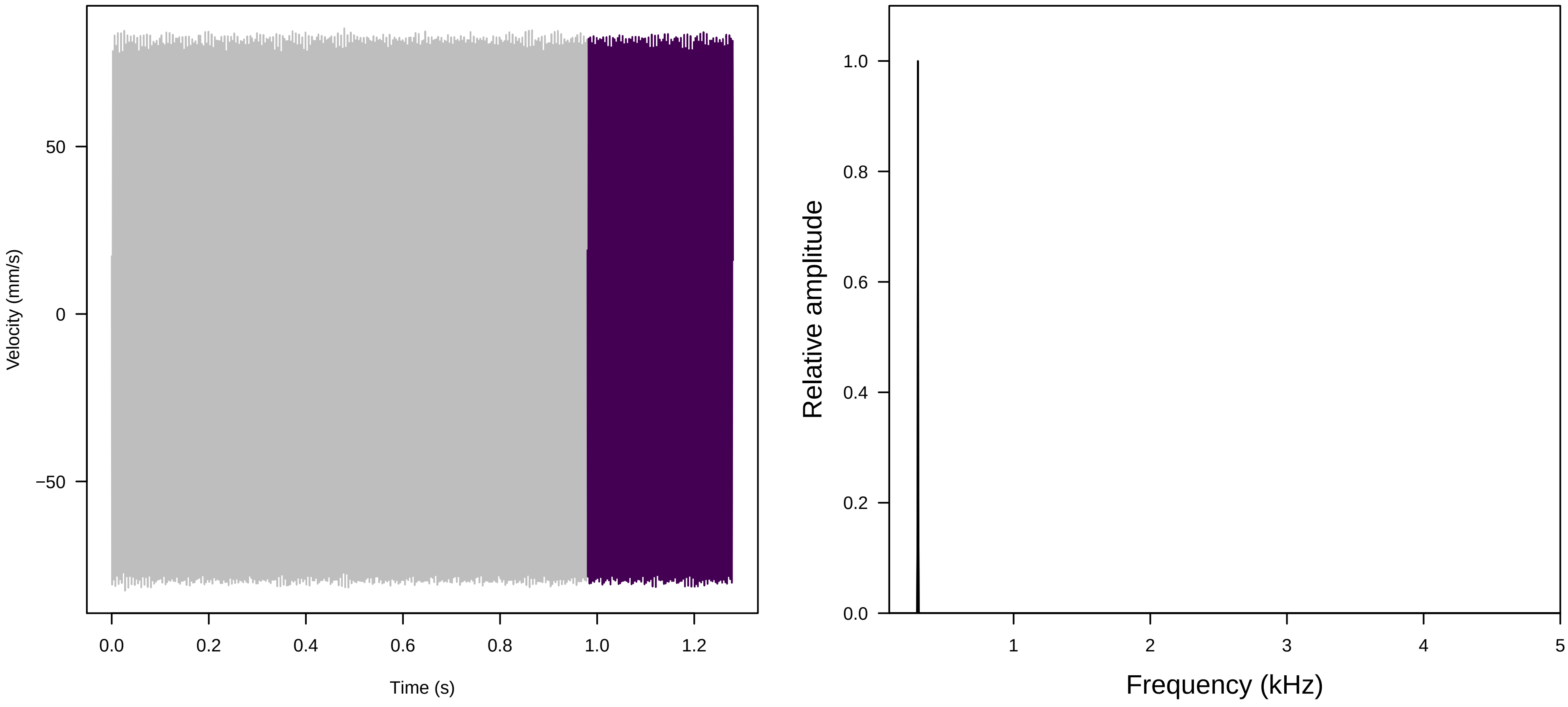

Vel. = 0.057 ; Str. = PA ; Axis = x ; Fl. accession = 10−s−77−12

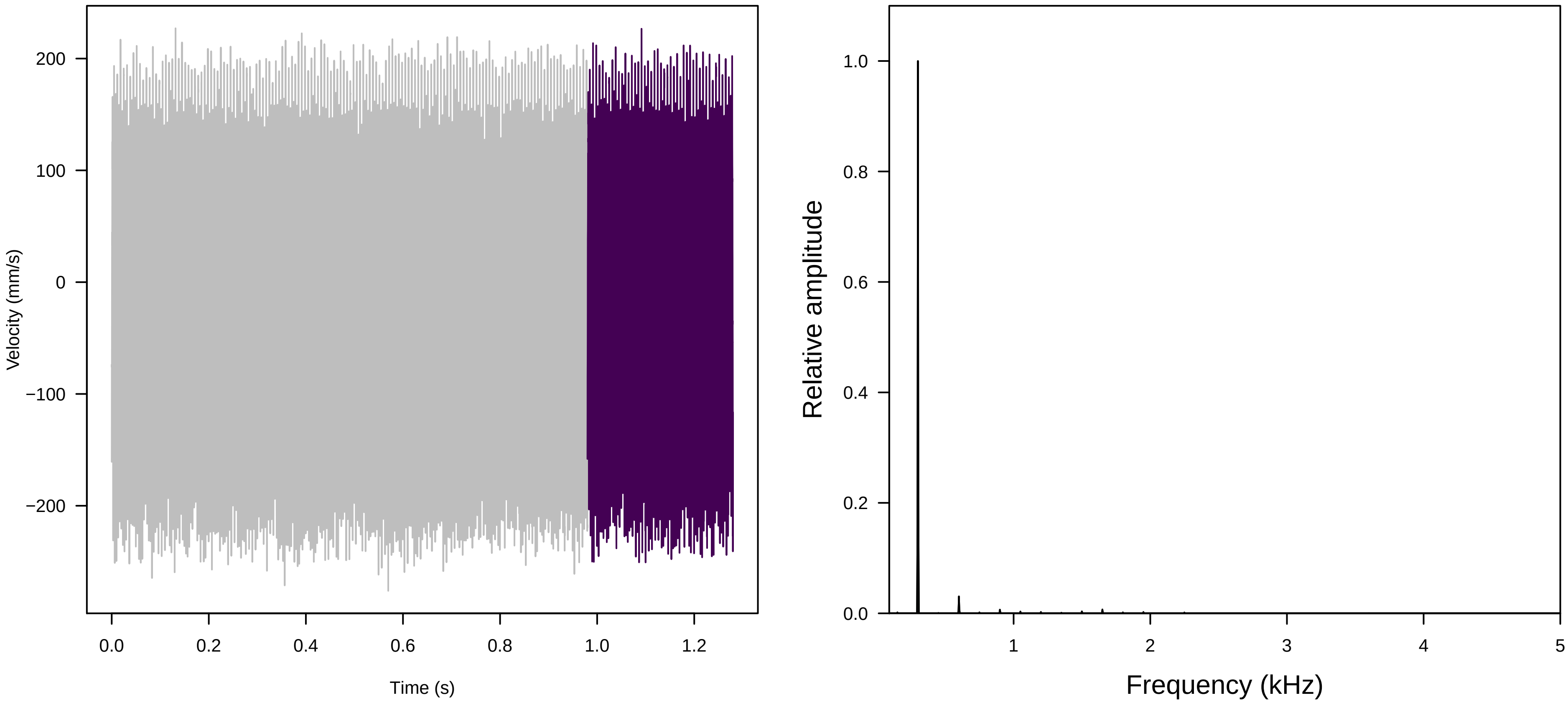

Vel. = 0.057 ; Str. = Receptacle ; Axis = x ; Fl. accession = 10−s−77−12

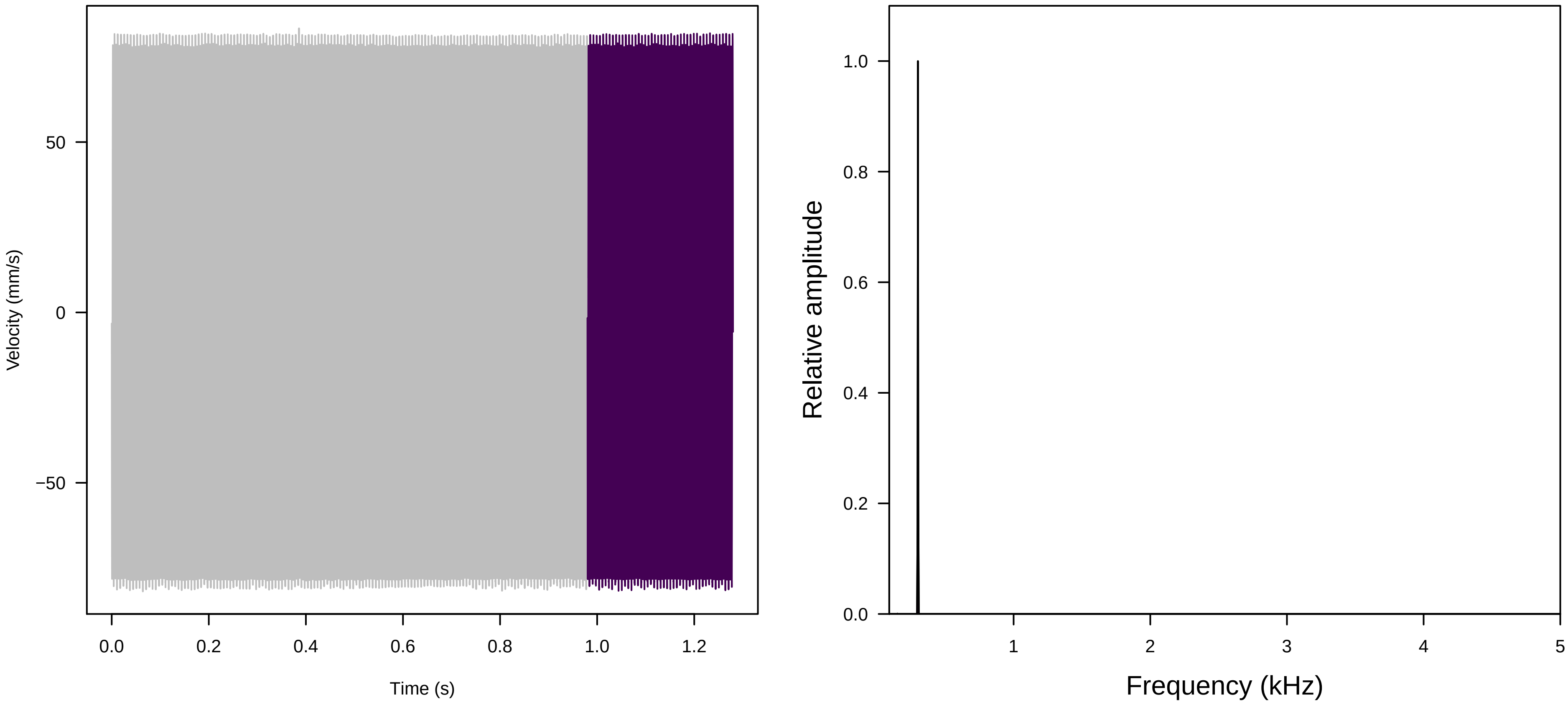

Vel. = 0.057 ; Str. = Corolla ; Axis = z ; Fl. accession = 10−s−77−12

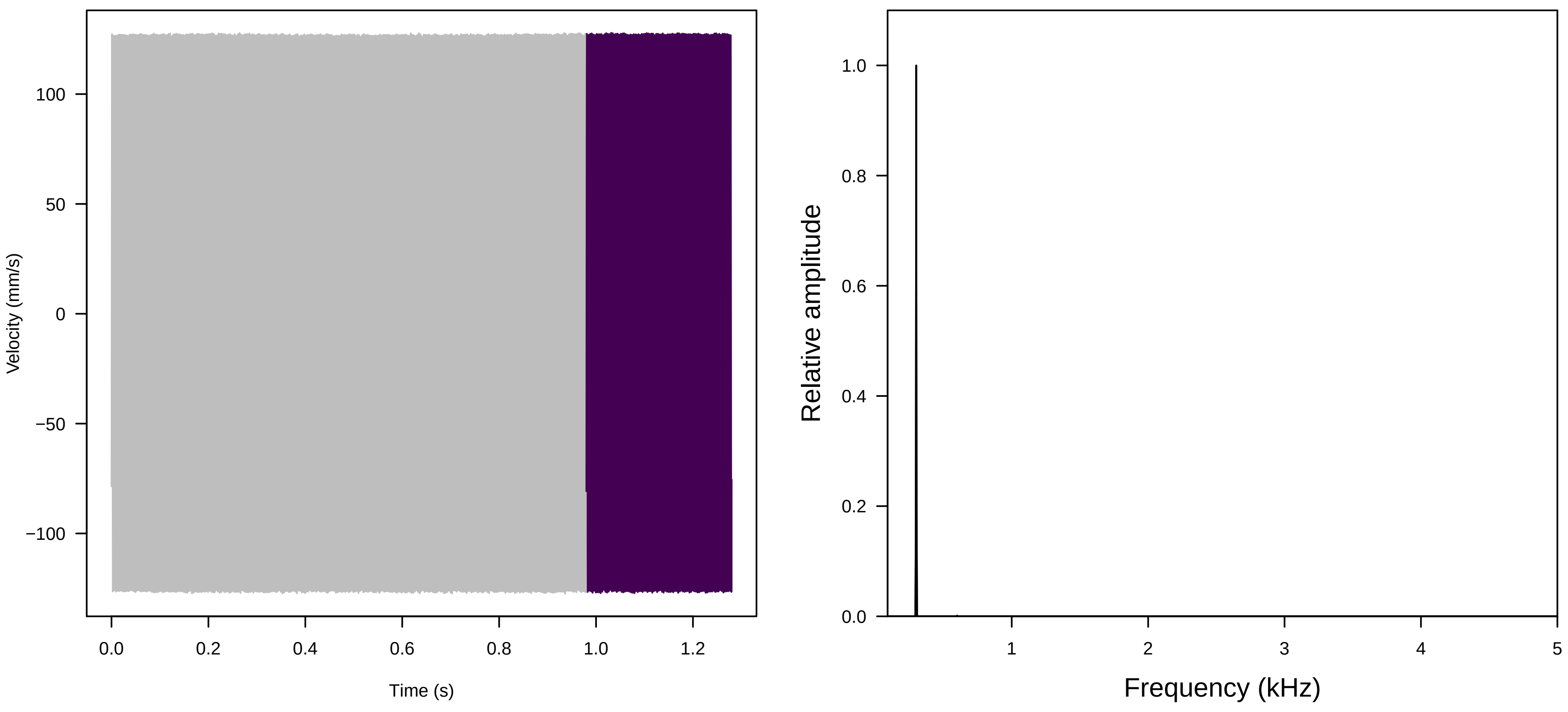

Vel. = 0.057 ; Str. = Receptacle ; Axis = z ; Fl. accession = 10−s−77−12

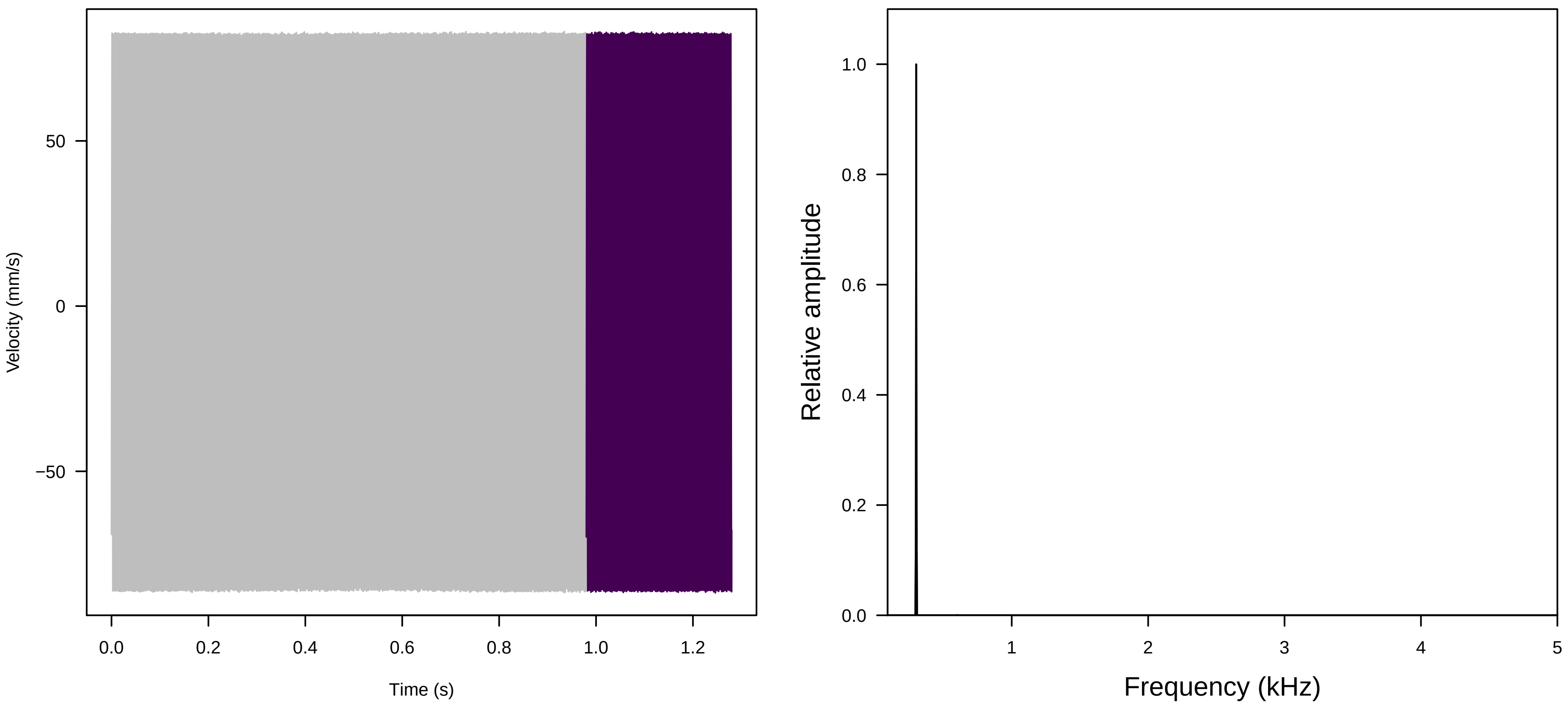

Vel. = 0.057 ; Str. = FA ; Axis = z ; Fl. accession = 10−s−77−12

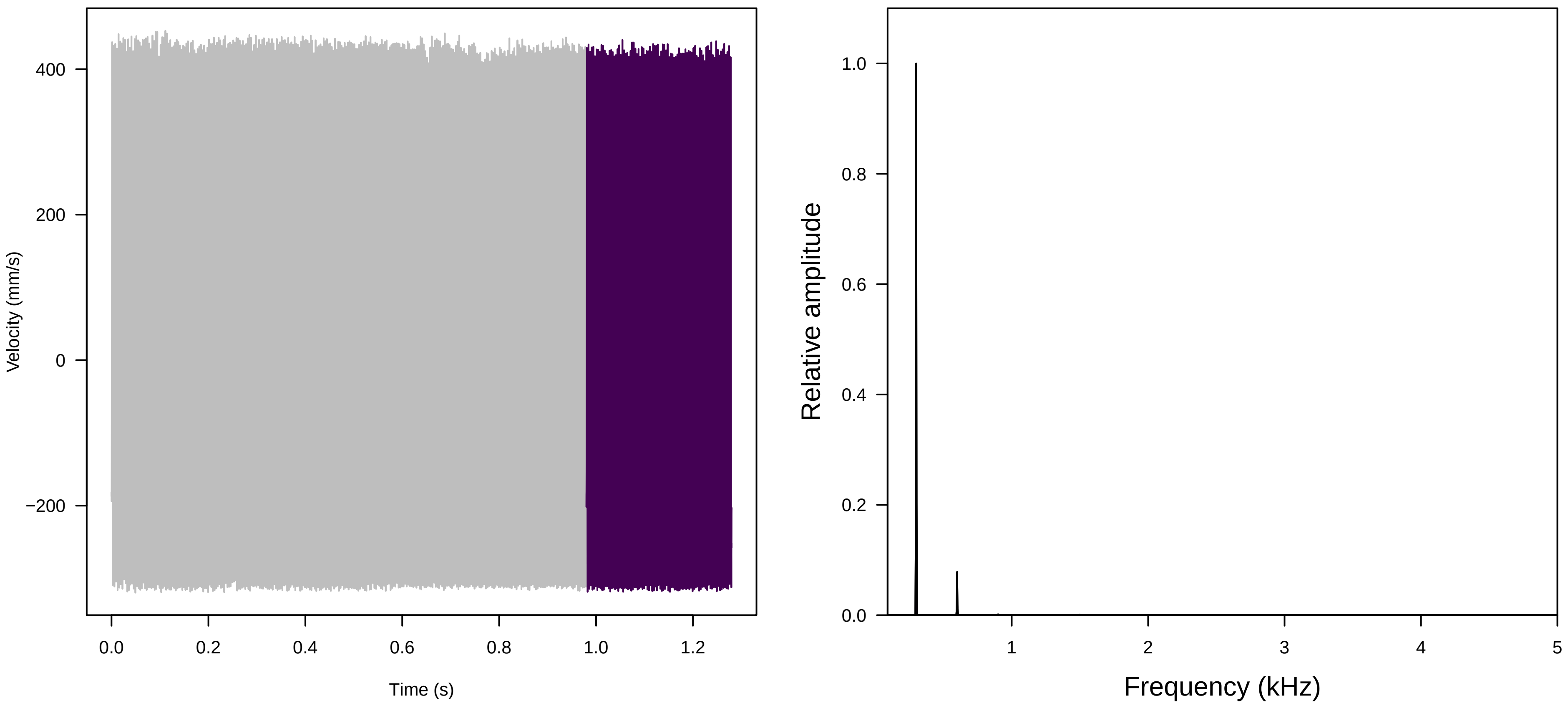

Vel. = 0.057 ; Str. = Receptacle ; Axis = z ; Fl. accession = 10−s−77−12

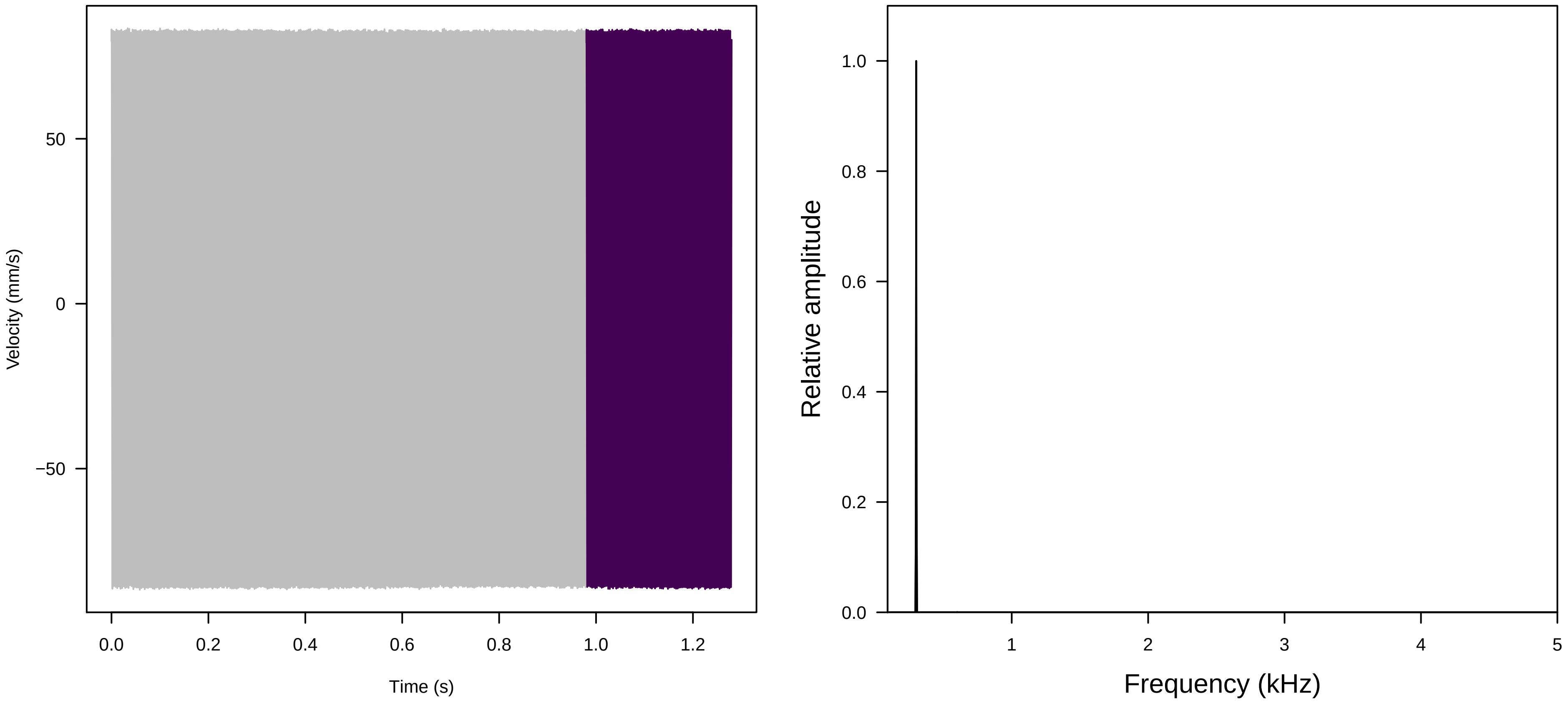

Vel. = 0.057 ; Str. = PA ; Axis = z ; Fl. accession = 10−s−77−12

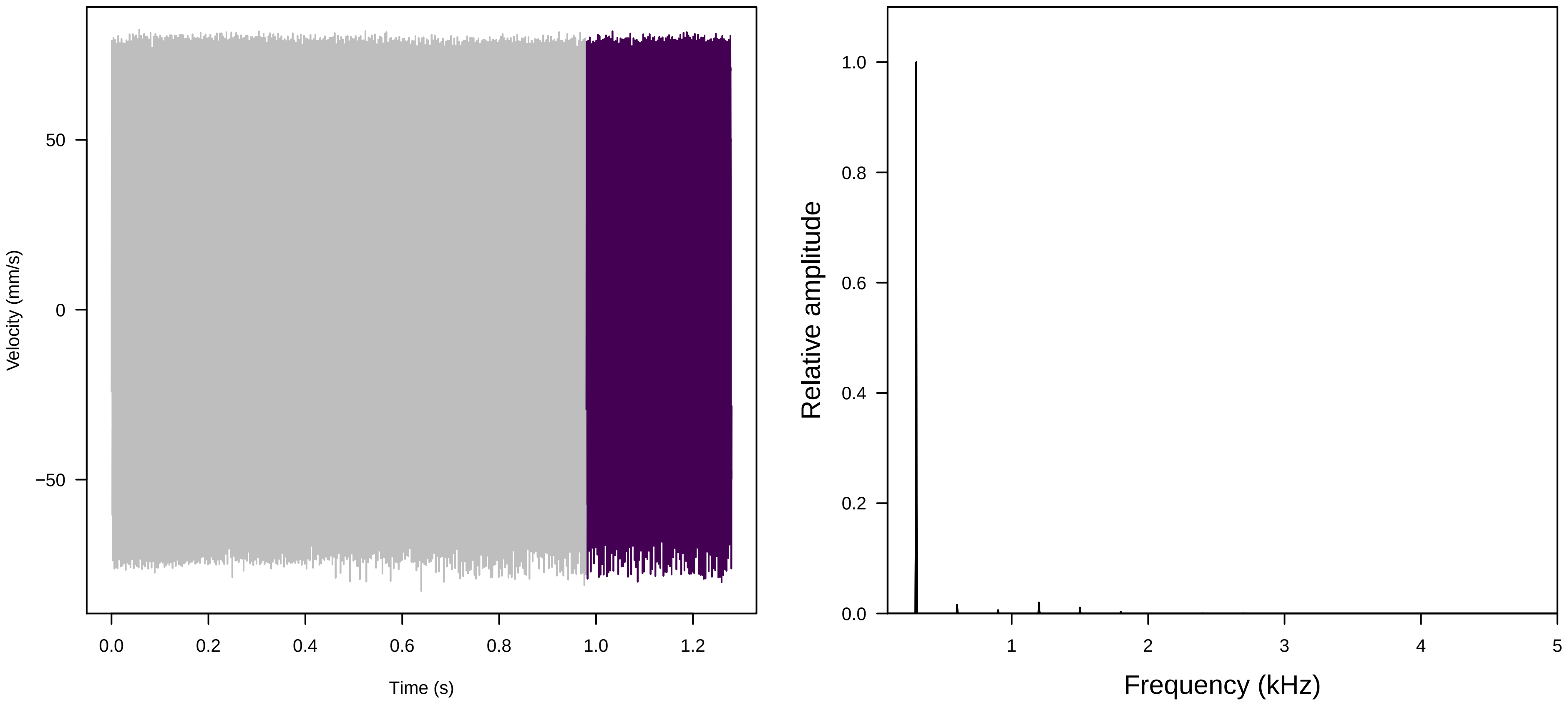

Vel. = 0.057 ; Str. = Receptacle ; Axis = z ; Fl. accession = 10−s−77−12

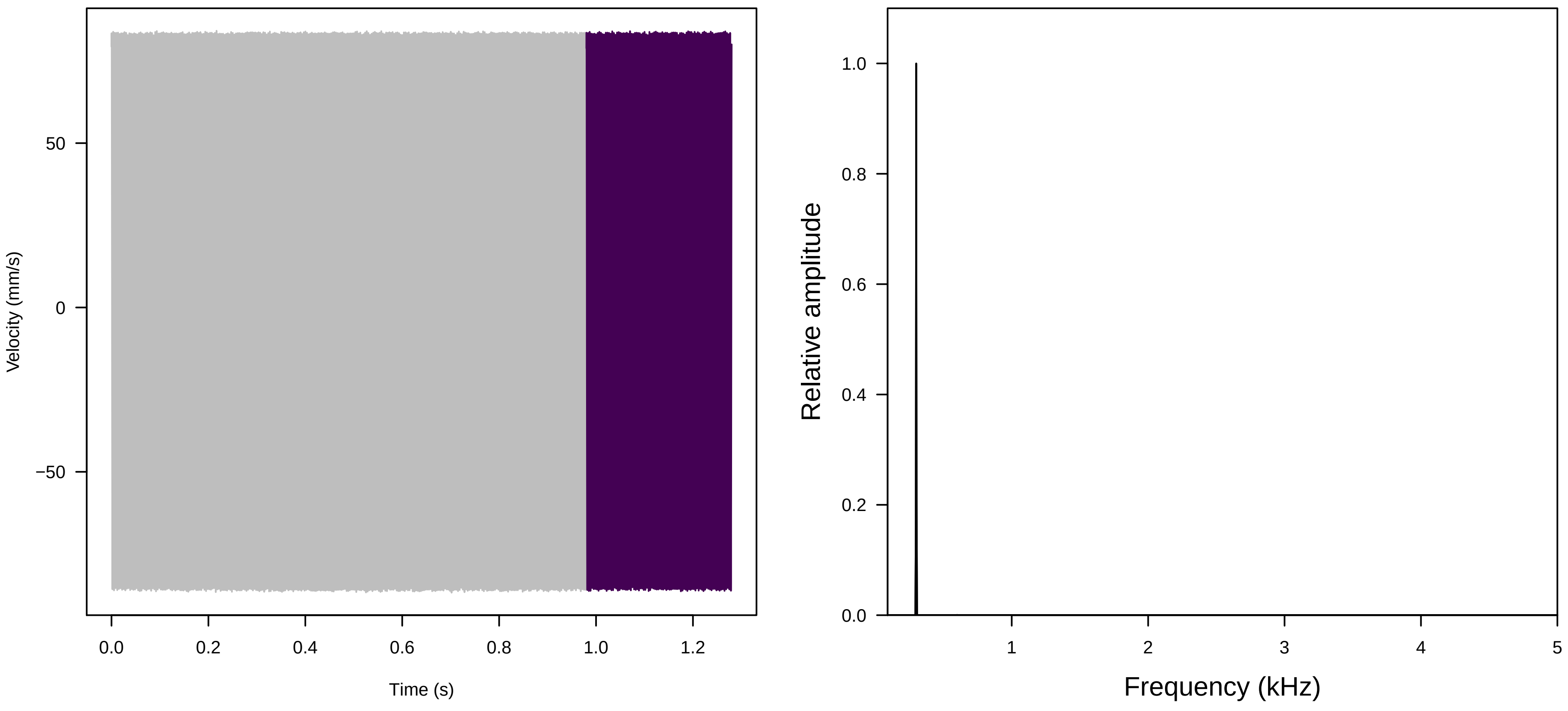

Vel. = 0.028 ; Str. = PA ; Axis = z ; Fl. accession = 10−s−77−12

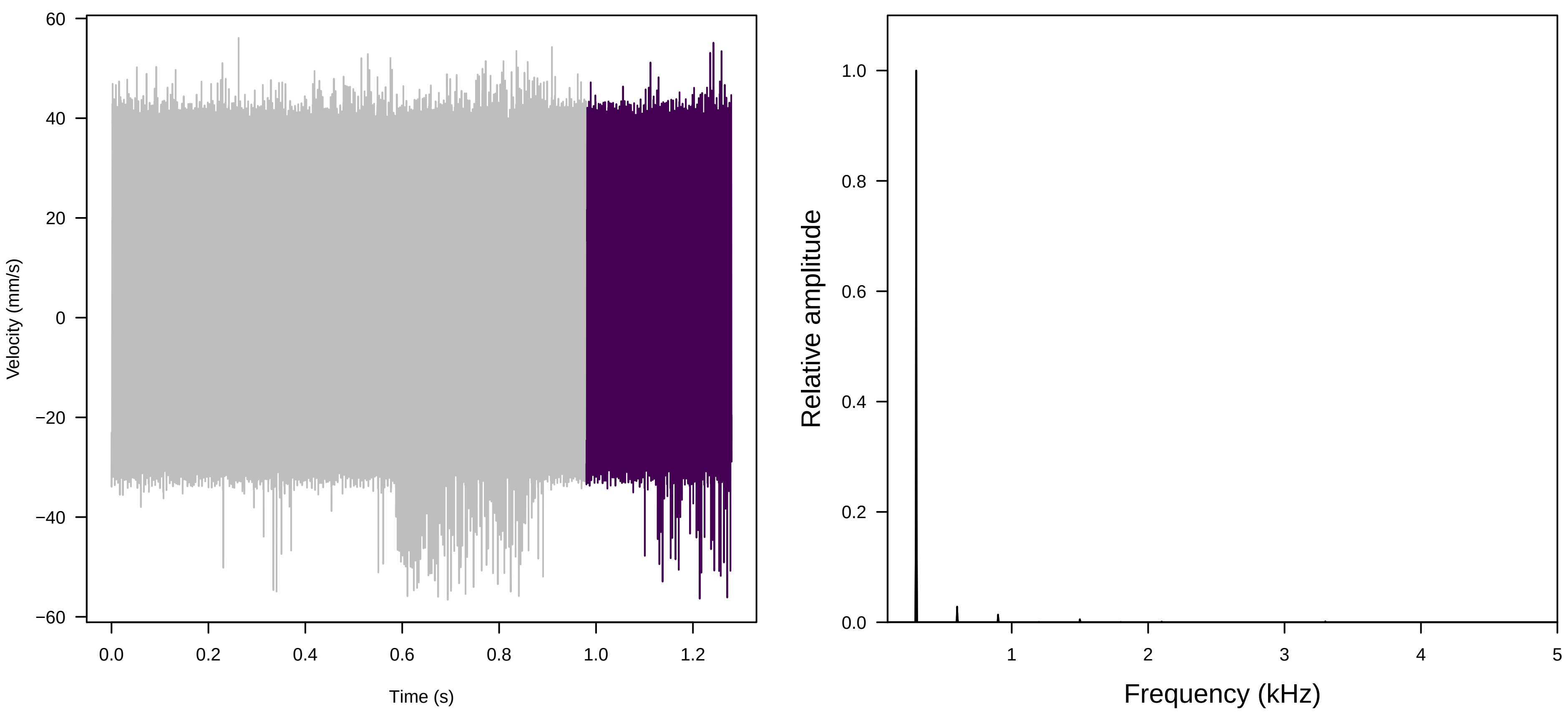

Vel. = 0.028 ; Str. = Receptacle ; Axis = z ; Fl. accession = 10−s−77−12

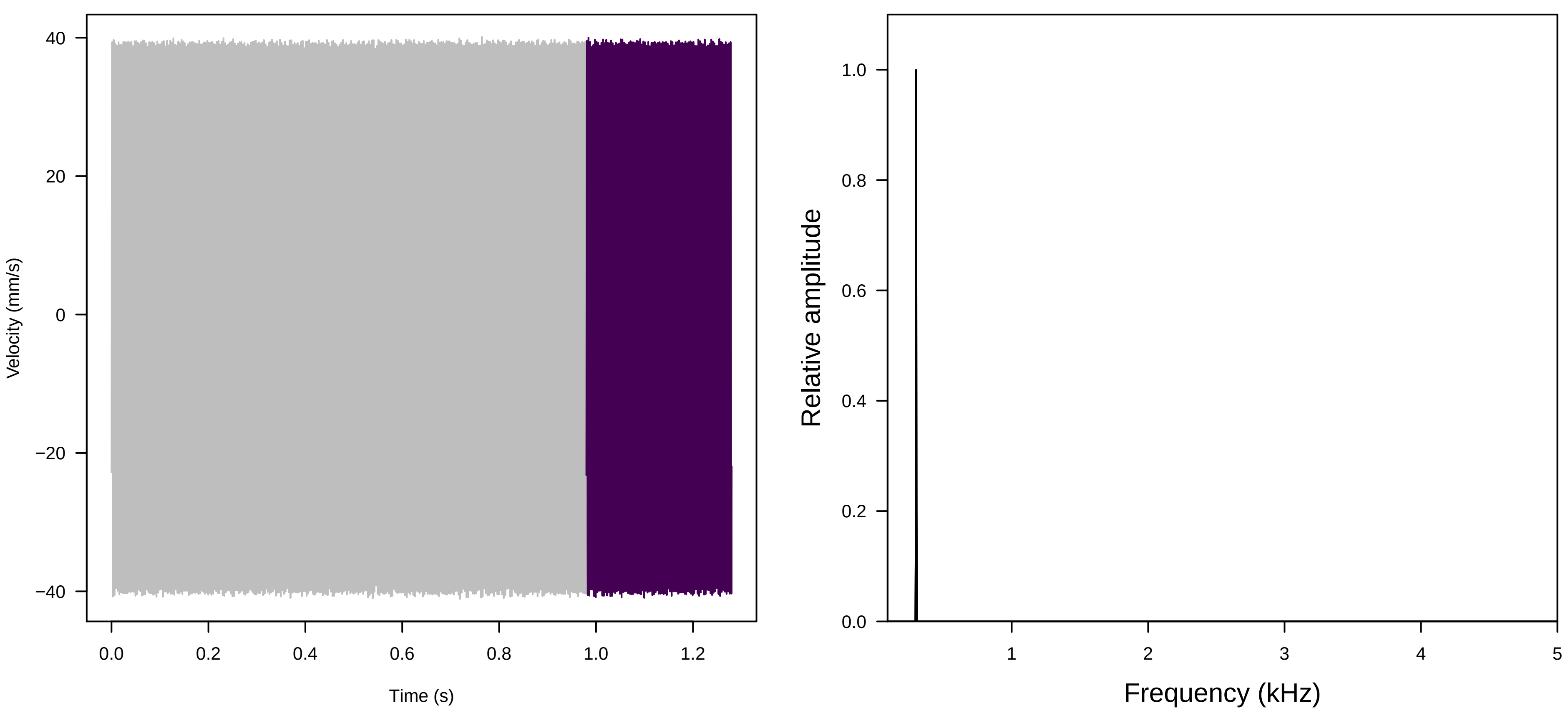

Vel. = 0.028 ; Str. = FA ; Axis = z ; Fl. accession = 10−s−77−12

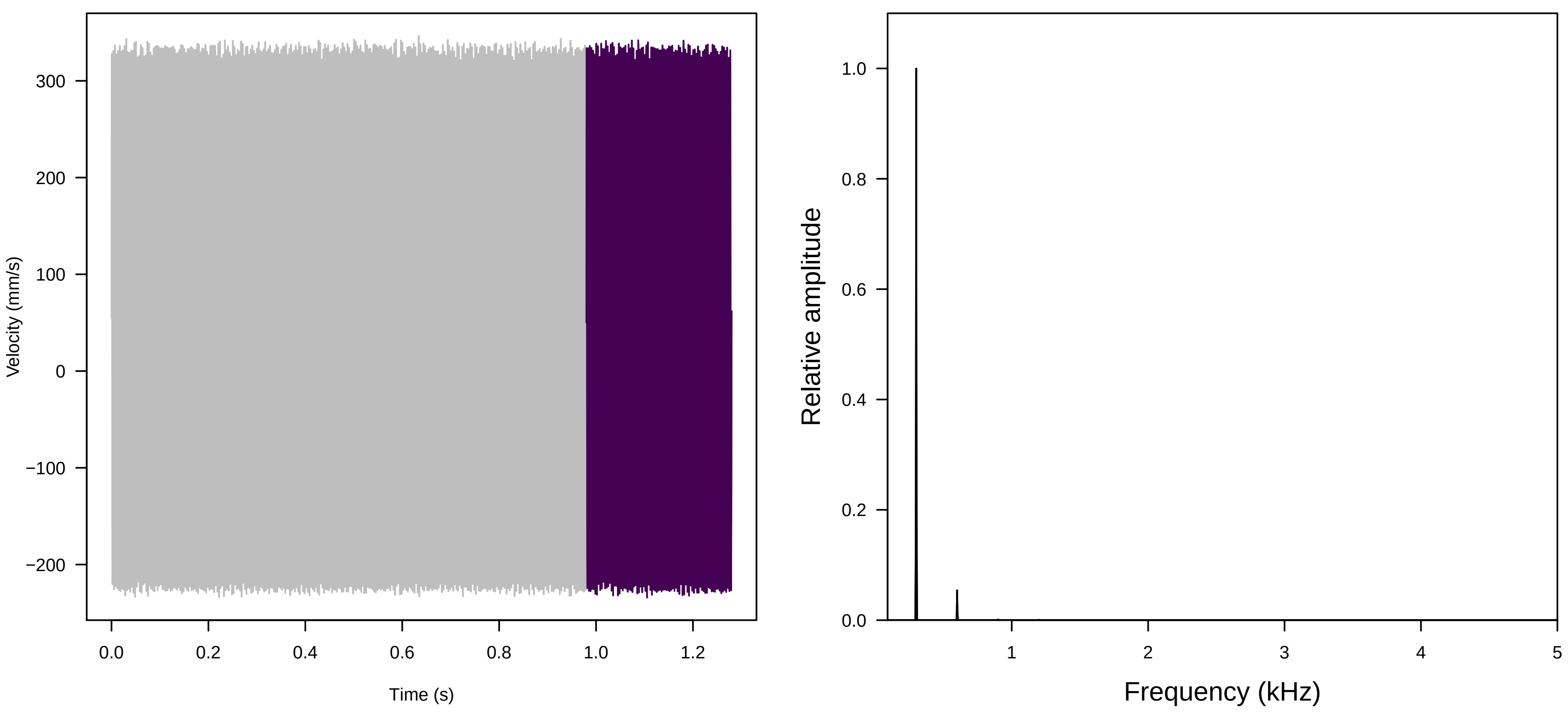

Vel. = 0.028 ; Str. = Receptacle ; Axis = z ; Fl. accession = 10−s−77−12

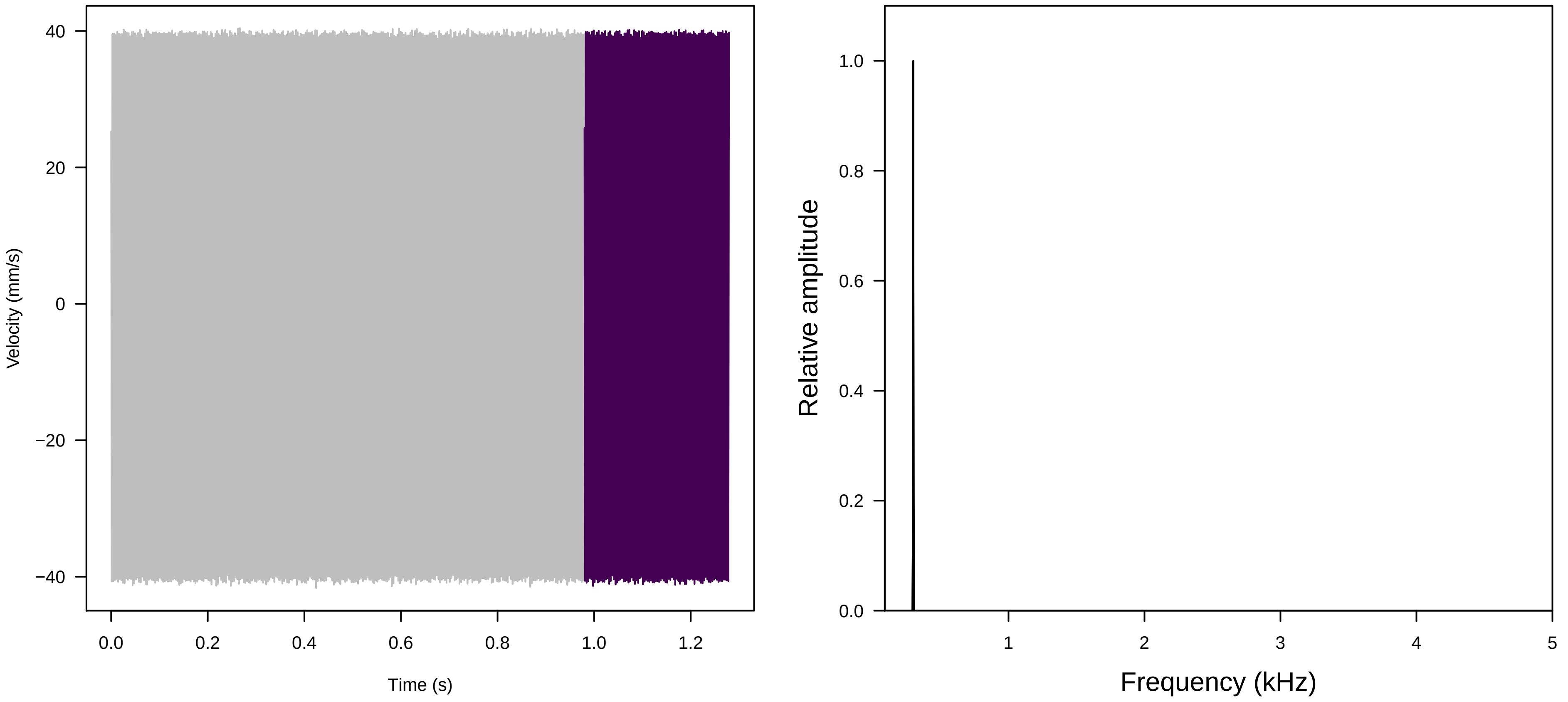

Vel. = 0.028 ; Str. = Corolla ; Axis = z ; Fl. accession = 10−s−77−12

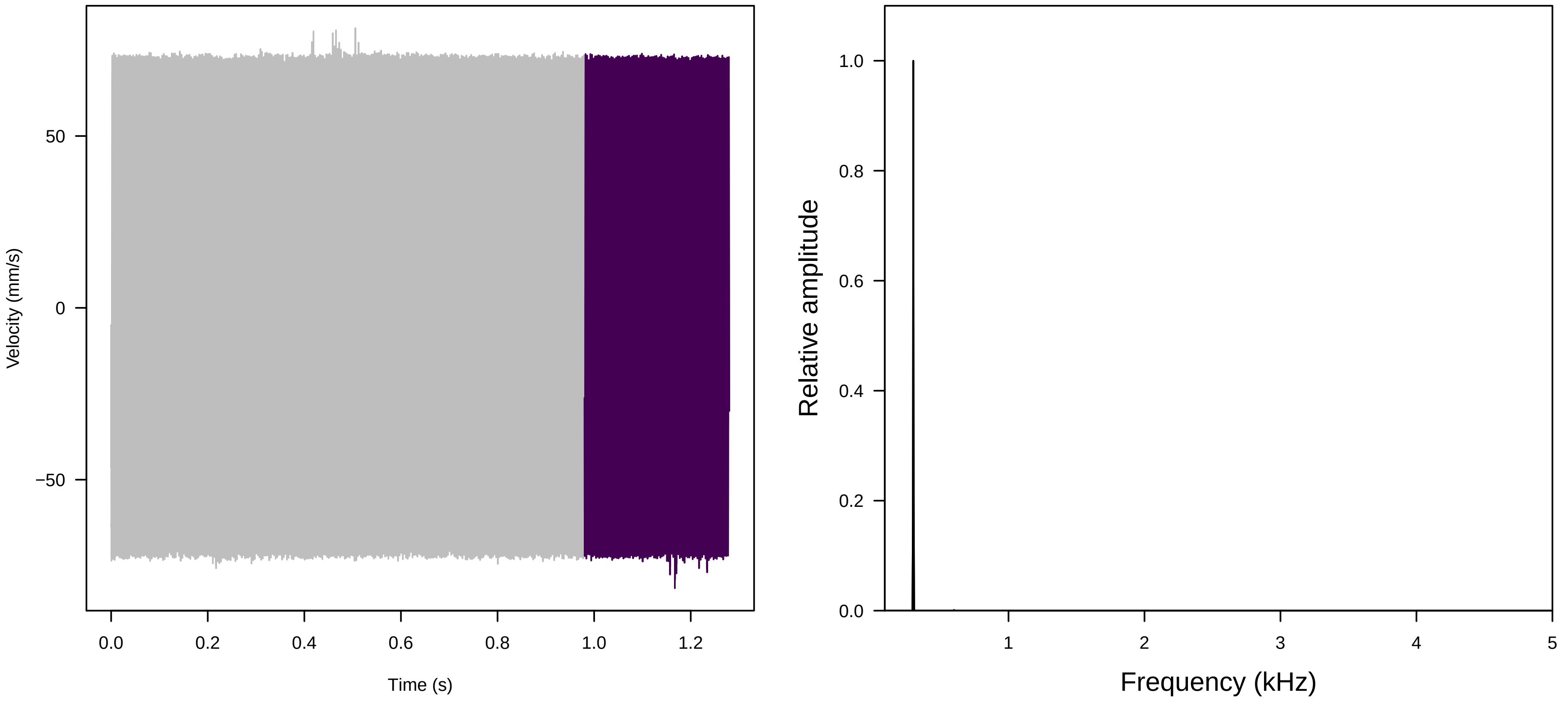

Vel. = 0.028 ; Str. = Receptacle ; Axis = z ; Fl. accession = 10−s−77−12

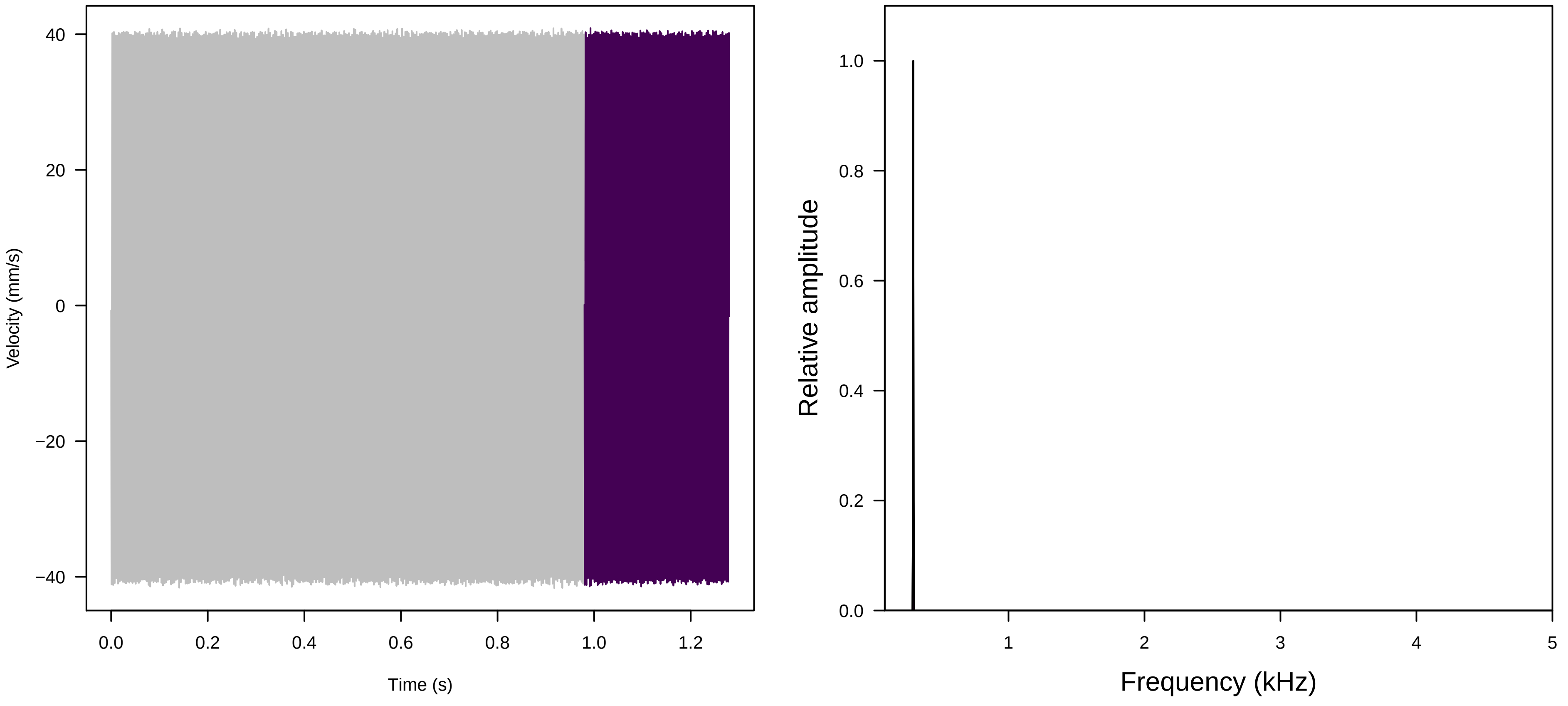

Vel. = 0.014 ; Str. = Corolla ; Axis = z ; Fl. accession = 10−s−77−12

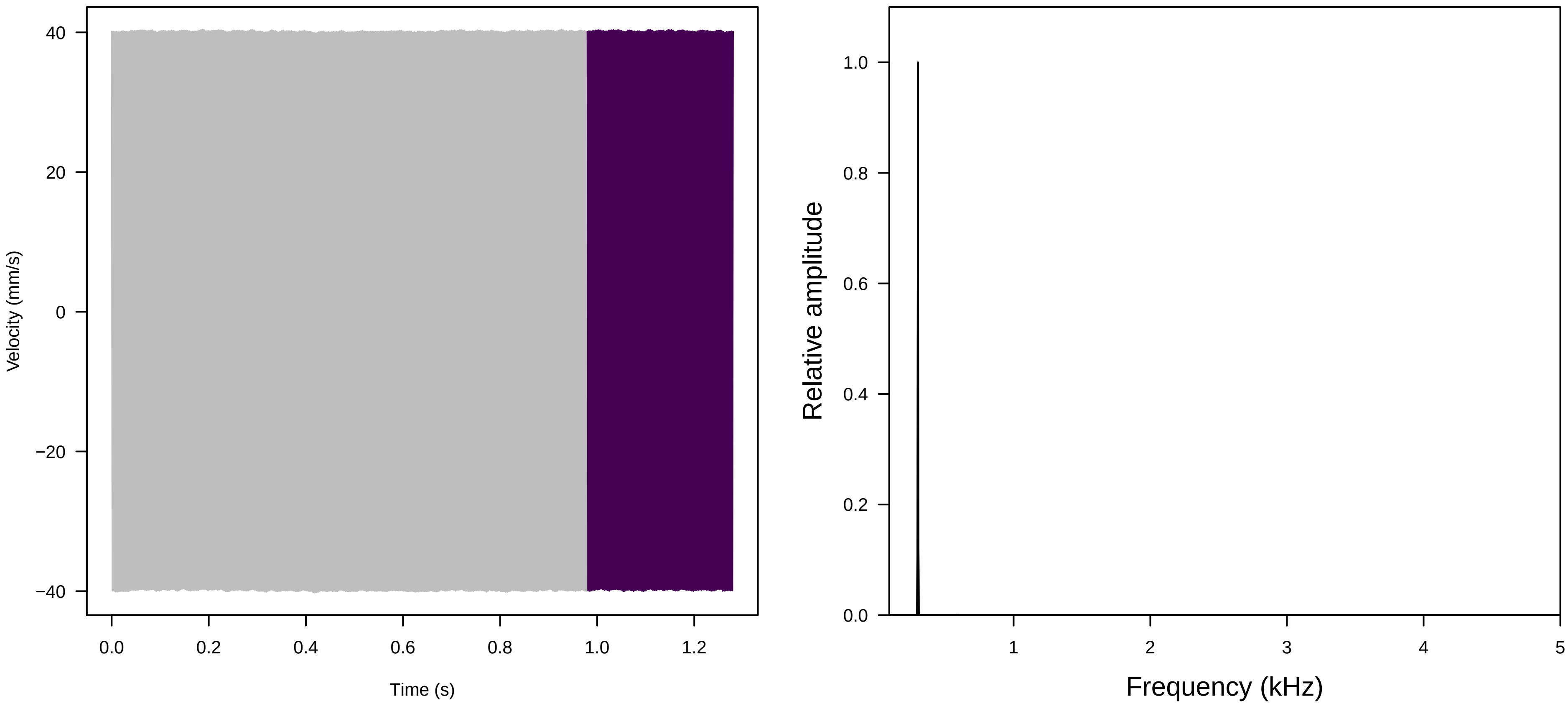

Vel. = 0.014 ; Str. = Receptacle ; Axis = z ; Fl. accession = 10−s−77−12

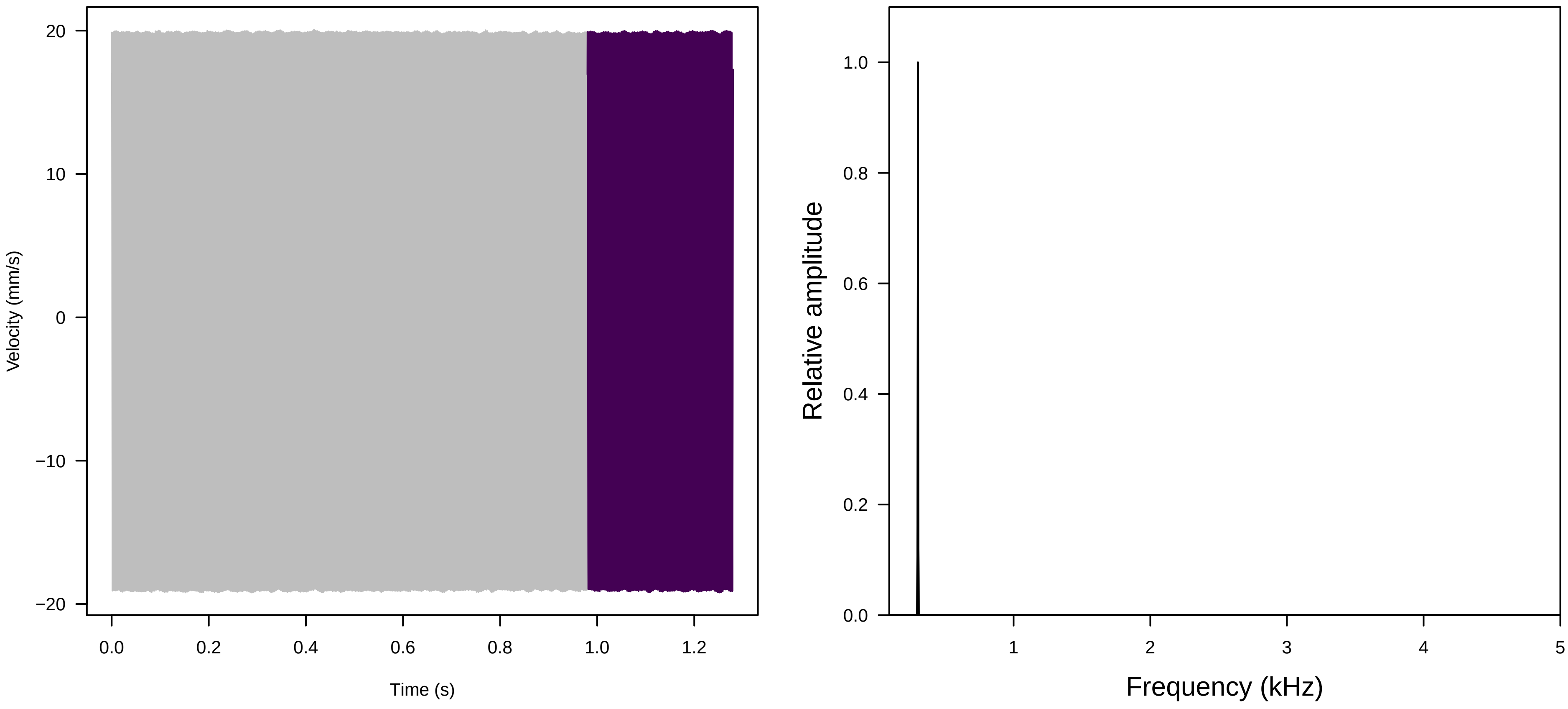

Vel. = 0.014 ; Str. = FA ; Axis = z ; Fl. accession = 10−s−77−12

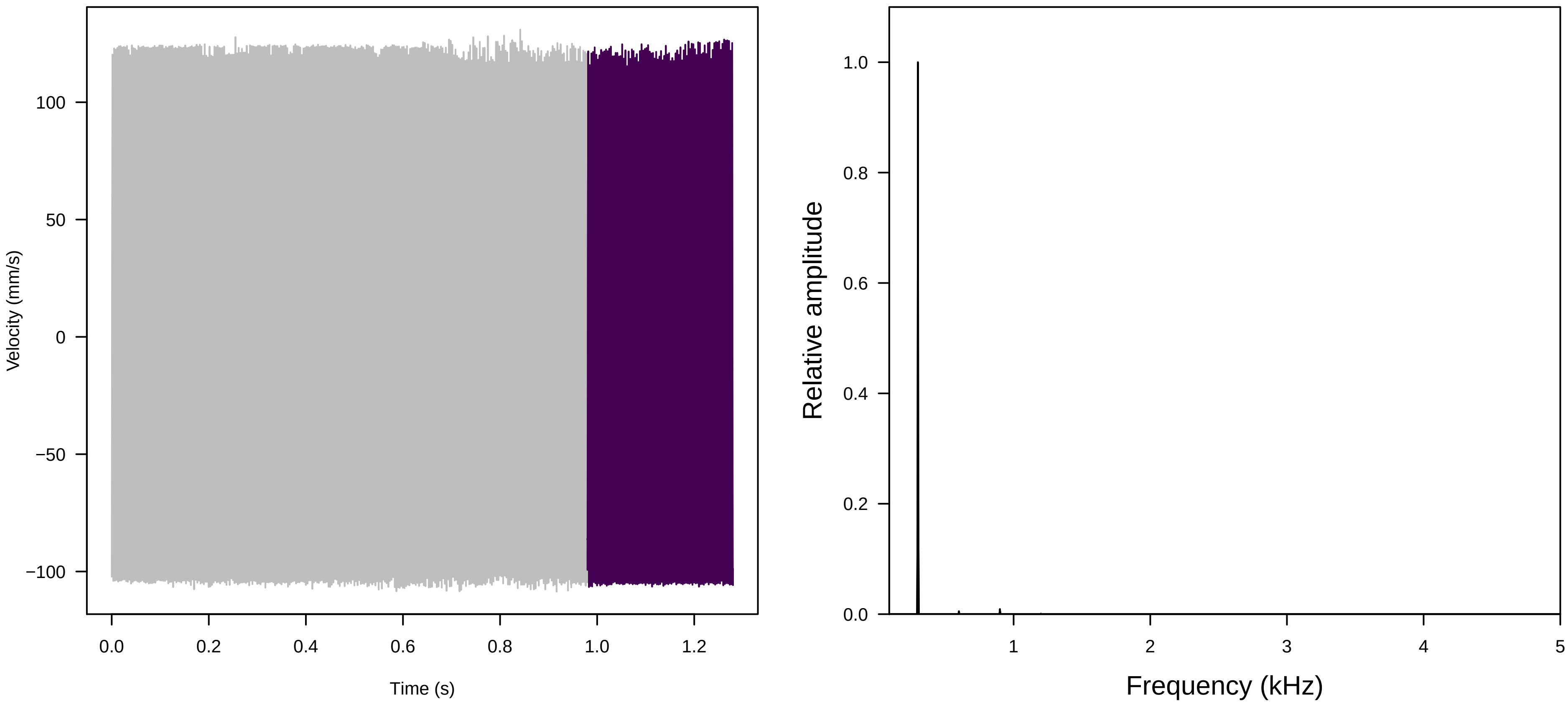

Vel. = 0.014 ; Str. = Receptacle ; Axis = z ; Fl. accession = 10−s−77−12

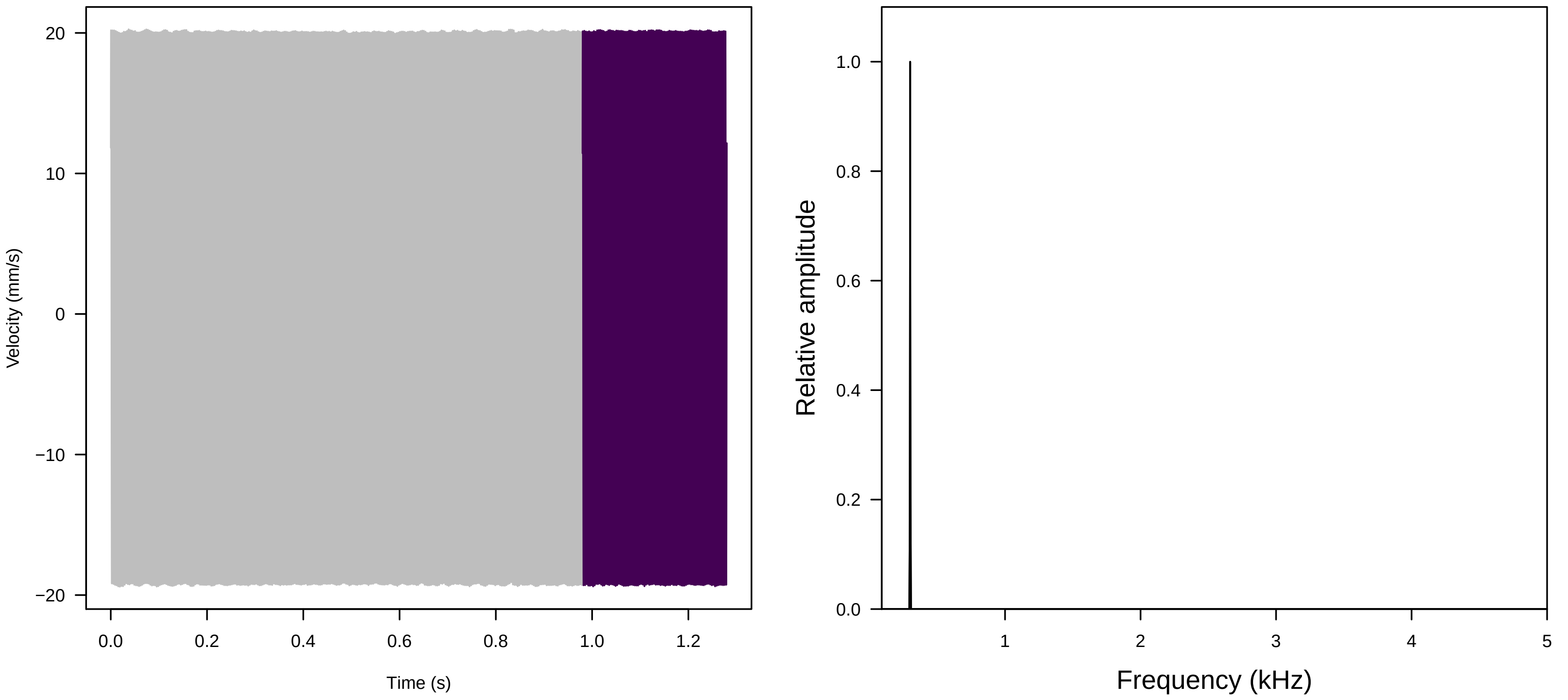

Vel. = 0.014 ; Str. = PA ; Axis = z ; Fl. accession = 10−s−77−12

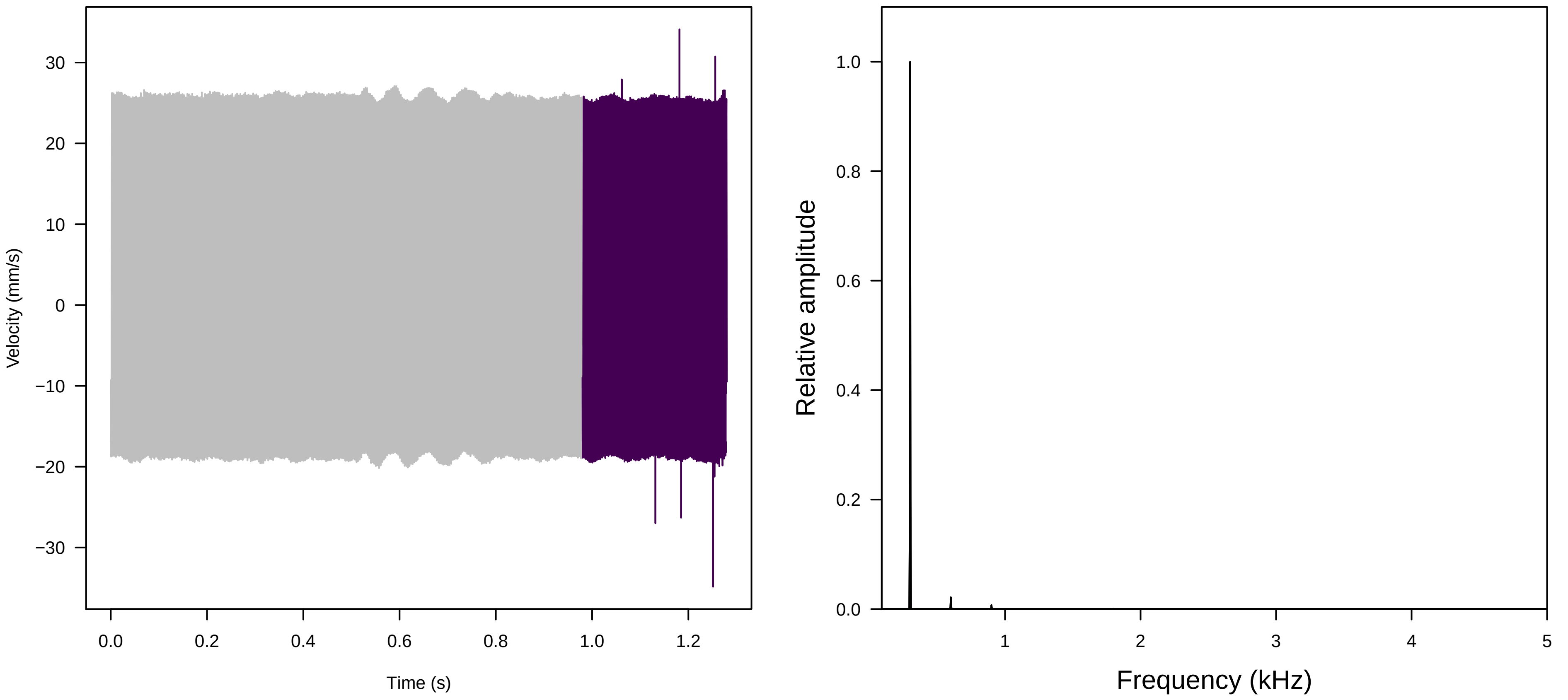

Vel. = 0.014 ; Str. = Receptacle ; Axis = z ; Fl. accession = 10−s−77−12

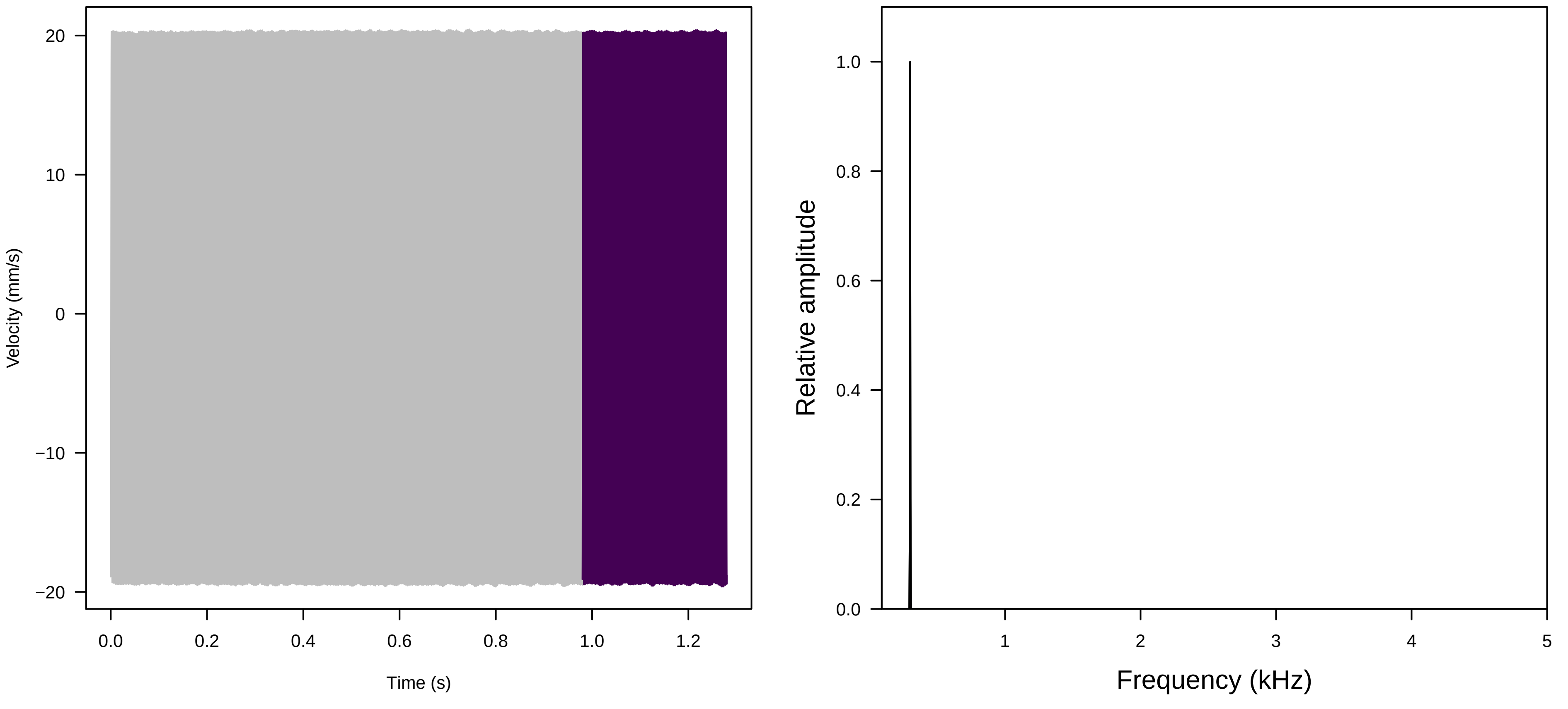

Vel. = 0.014 ; Str. = Corolla ; Axis = y ; Fl. accession = 10−s−77−12

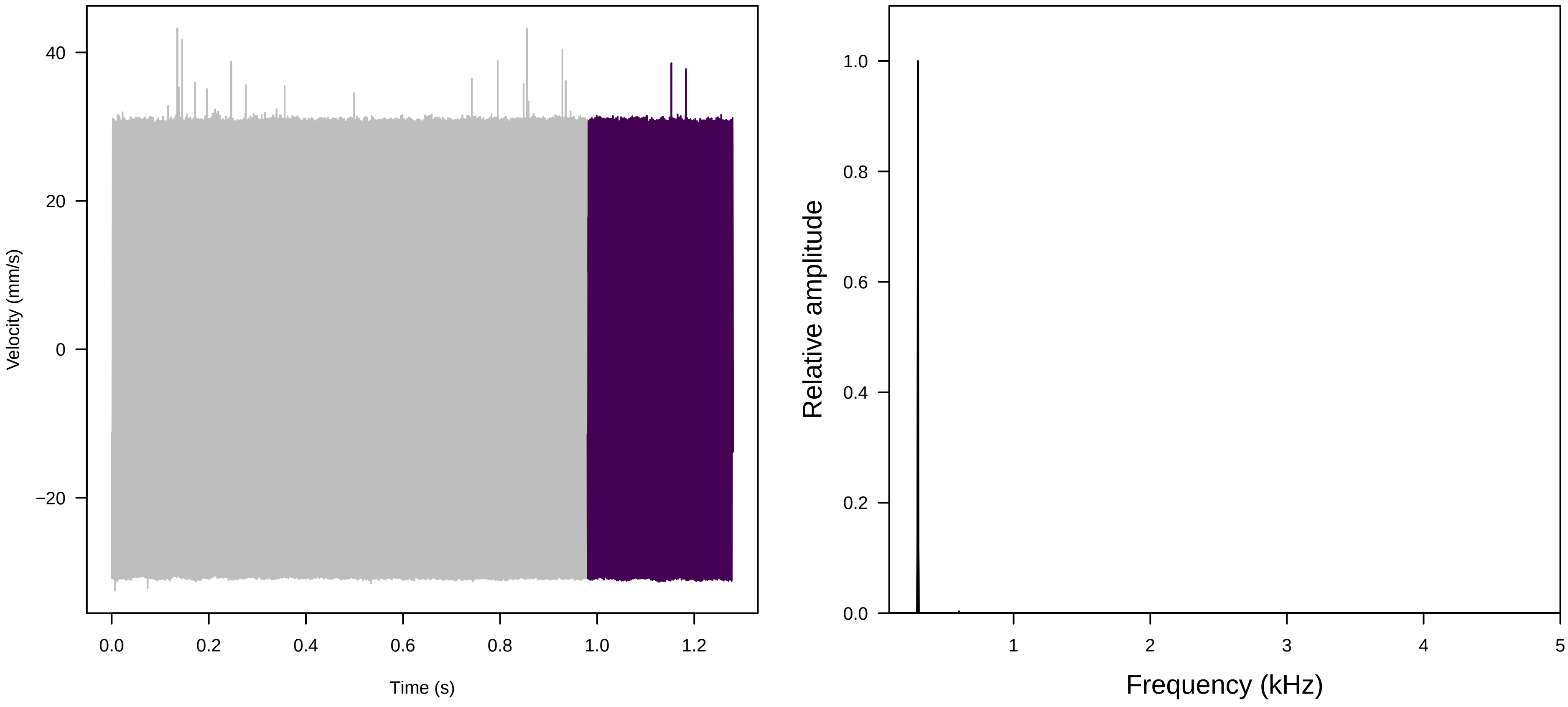

Vel. = 0.014 ; Str. = Receptacle ; Axis = y ; Fl. accession = 10−s−77−12

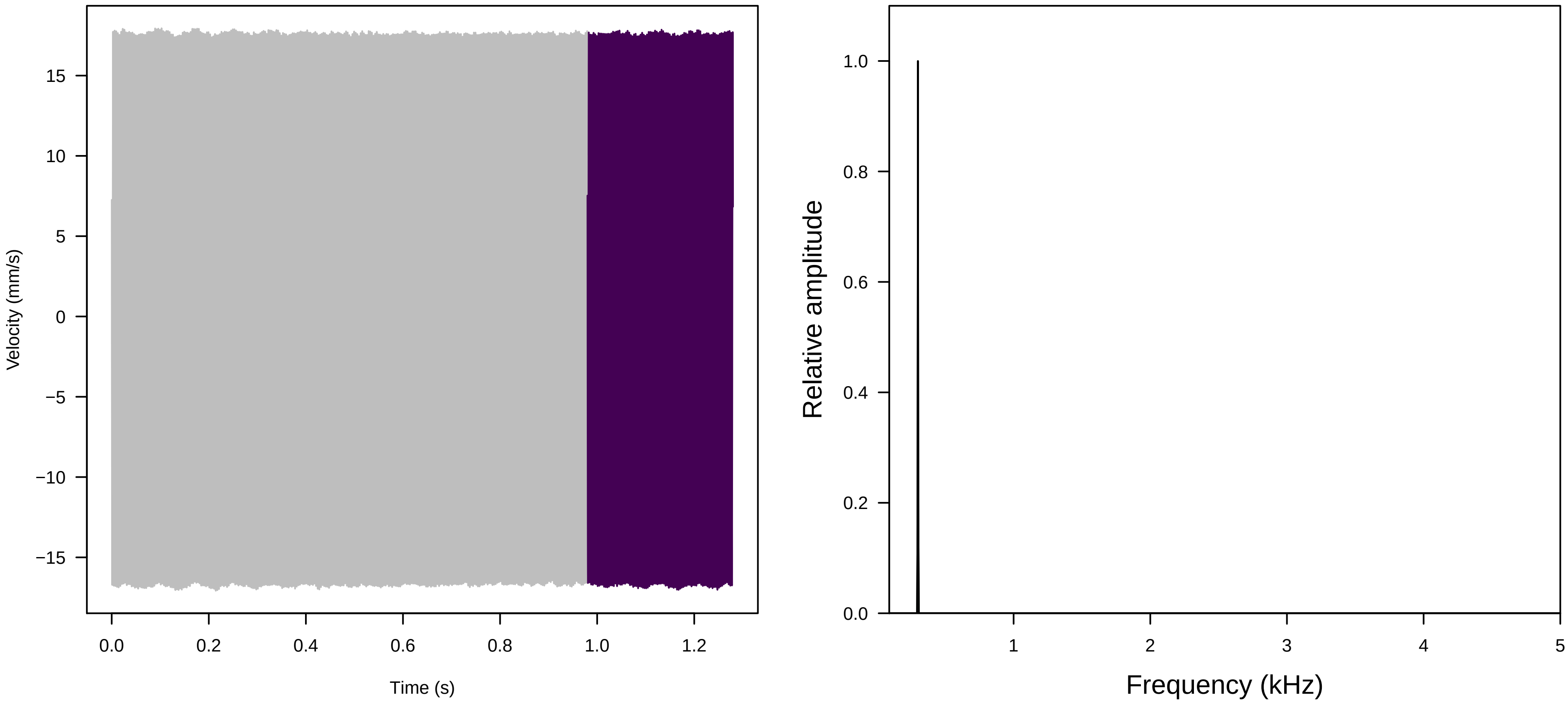

Vel. = 0.014 ; Str. = FA ; Axis = y ; Fl. accession = 10−s−77−12

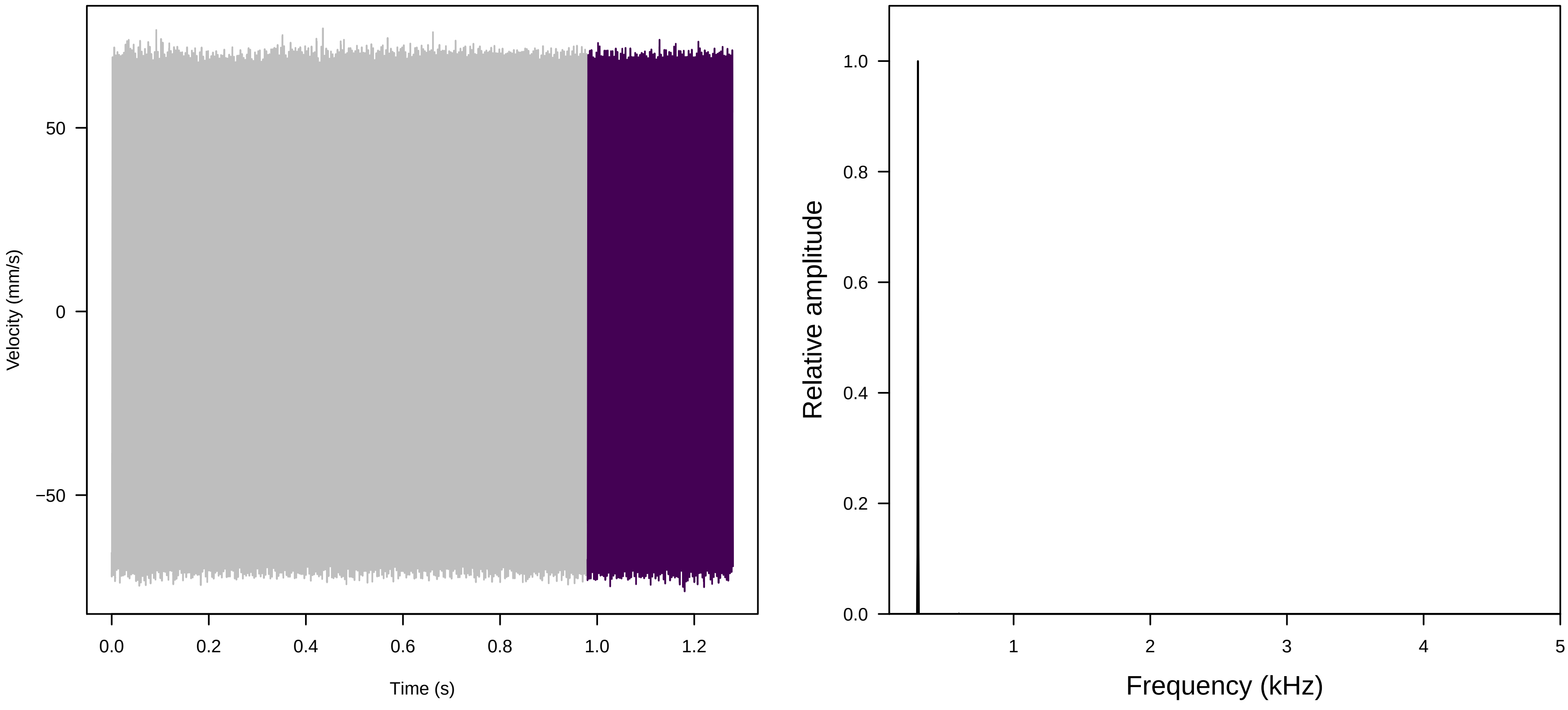

Vel. = 0.014 ; Str. = Receptacle ; Axis = y ; Fl. accession = 10−s−77−12

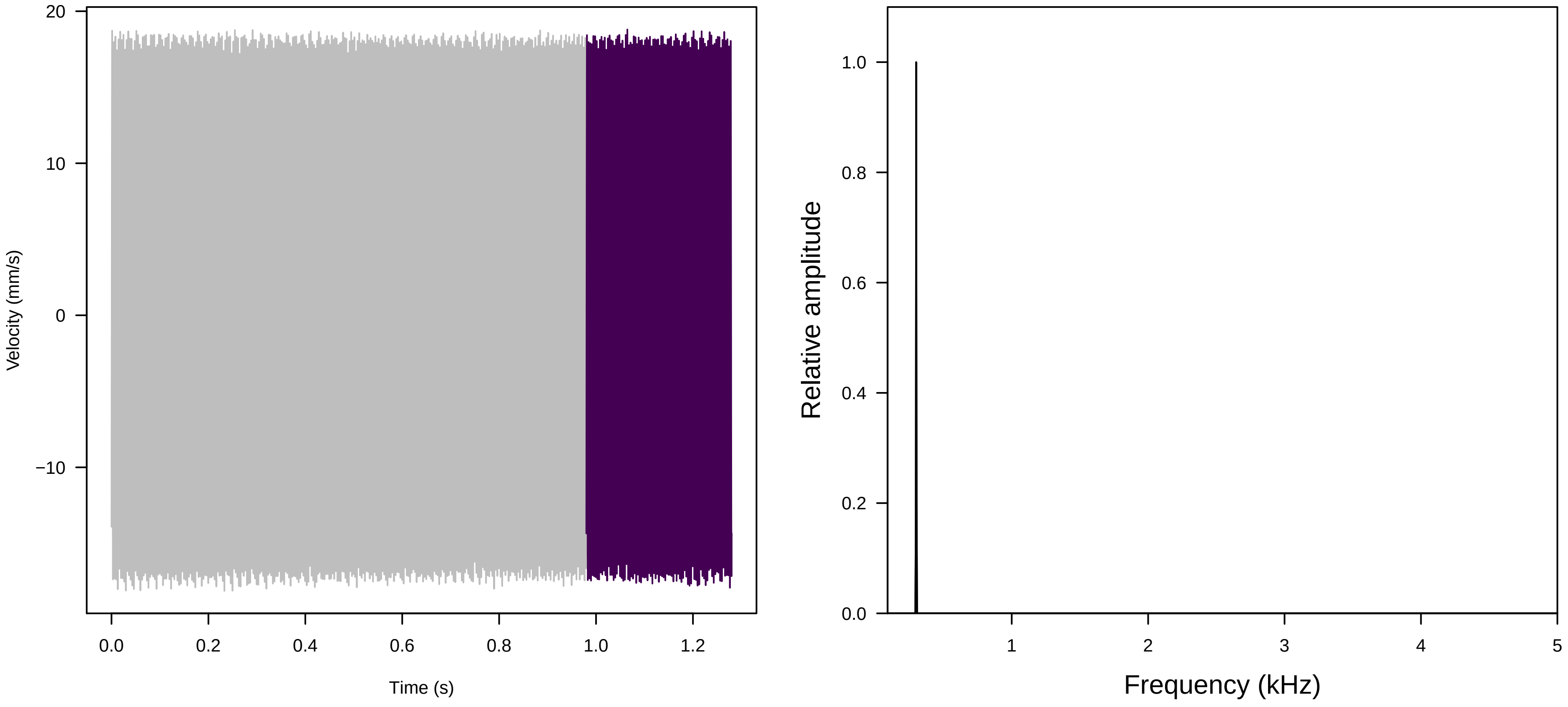

Vel. = 0.014 ; Str. = PA ; Axis = y ; Fl. accession = 10−s−77−12

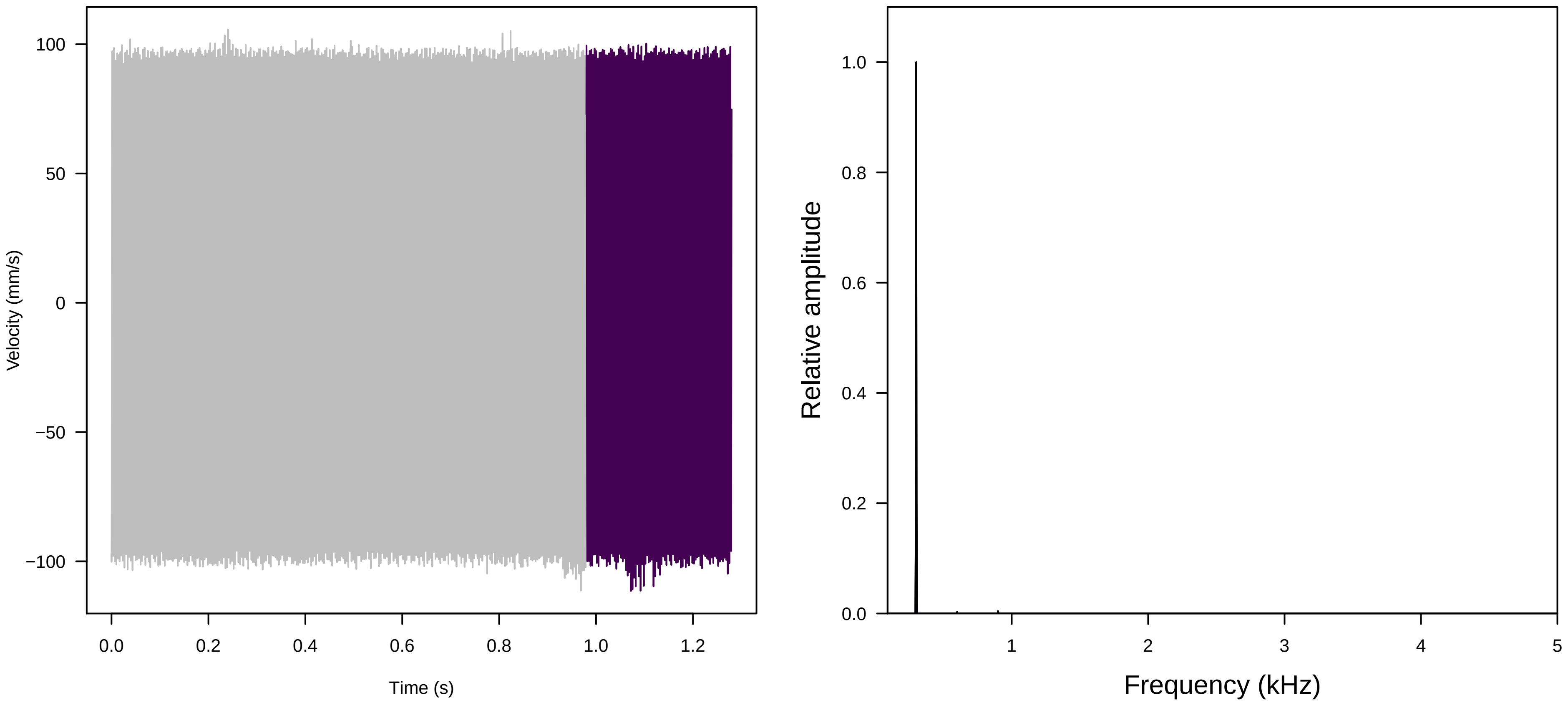

Vel. = 0.014 ; Str. = Receptacle ; Axis = y ; Fl. accession = 10−s−77−12

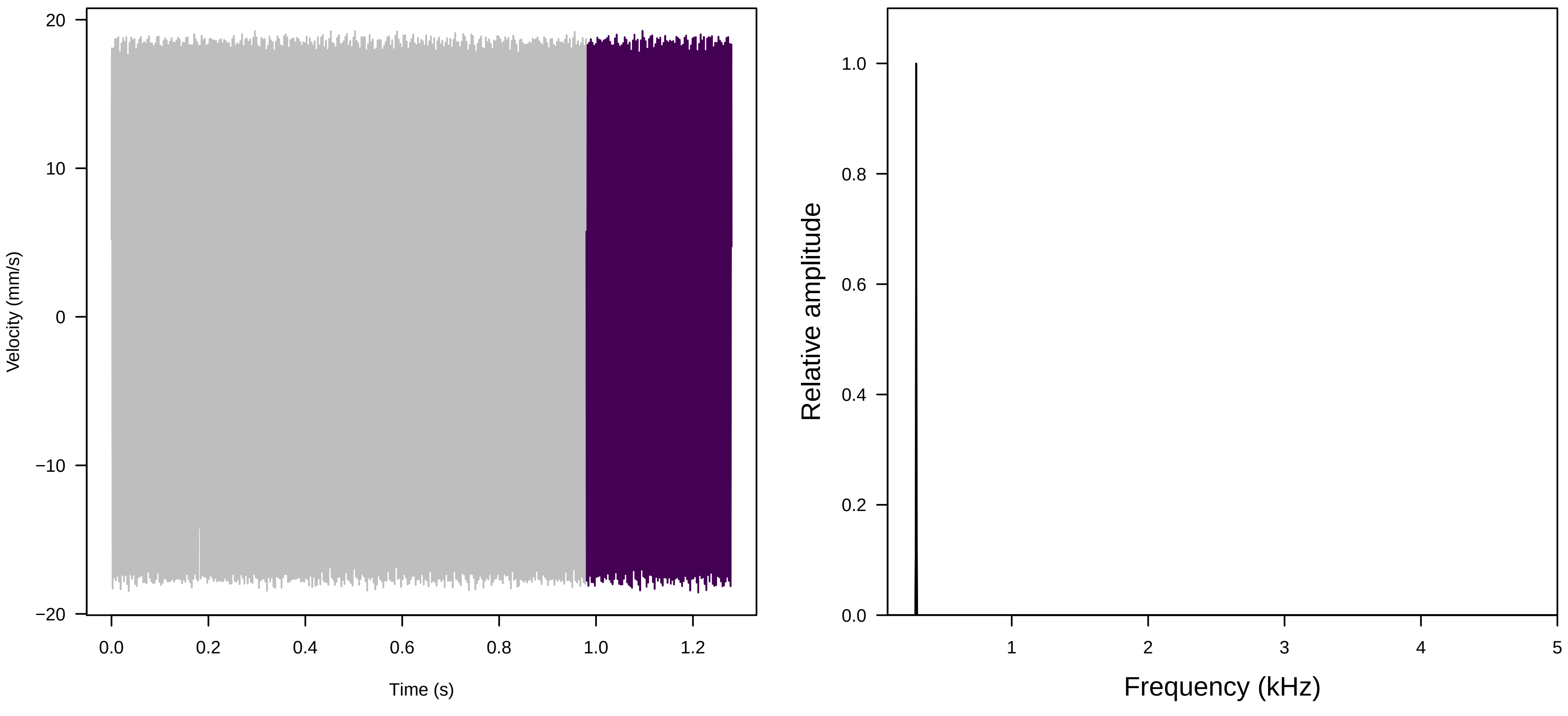

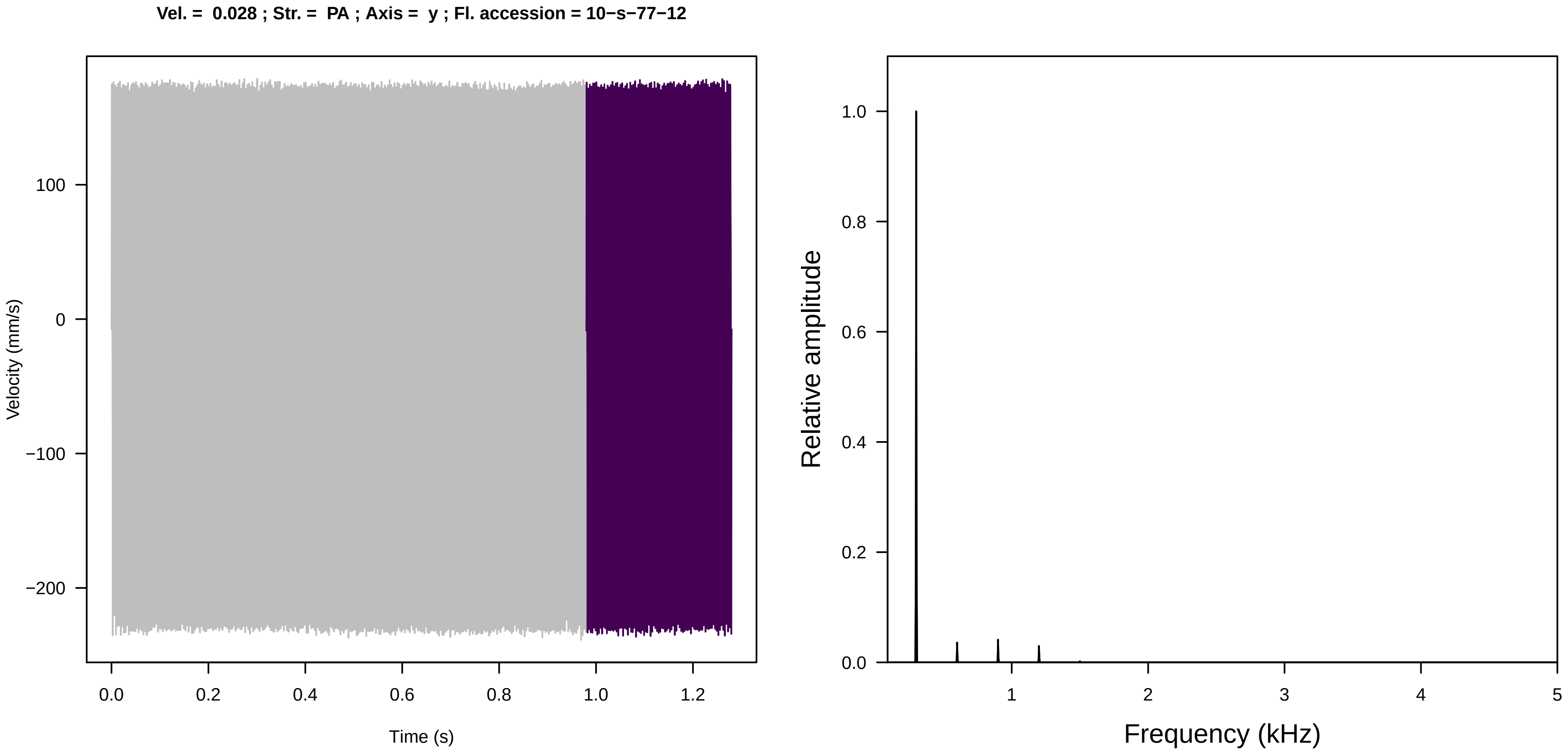
Vel. = 0.028 ; Str. = PA ; Axis = y ; Fl. accession = 10−s−77−12
Velocity (mm/s)
Time (s)
Relative amplitude
Frequency (kHz)

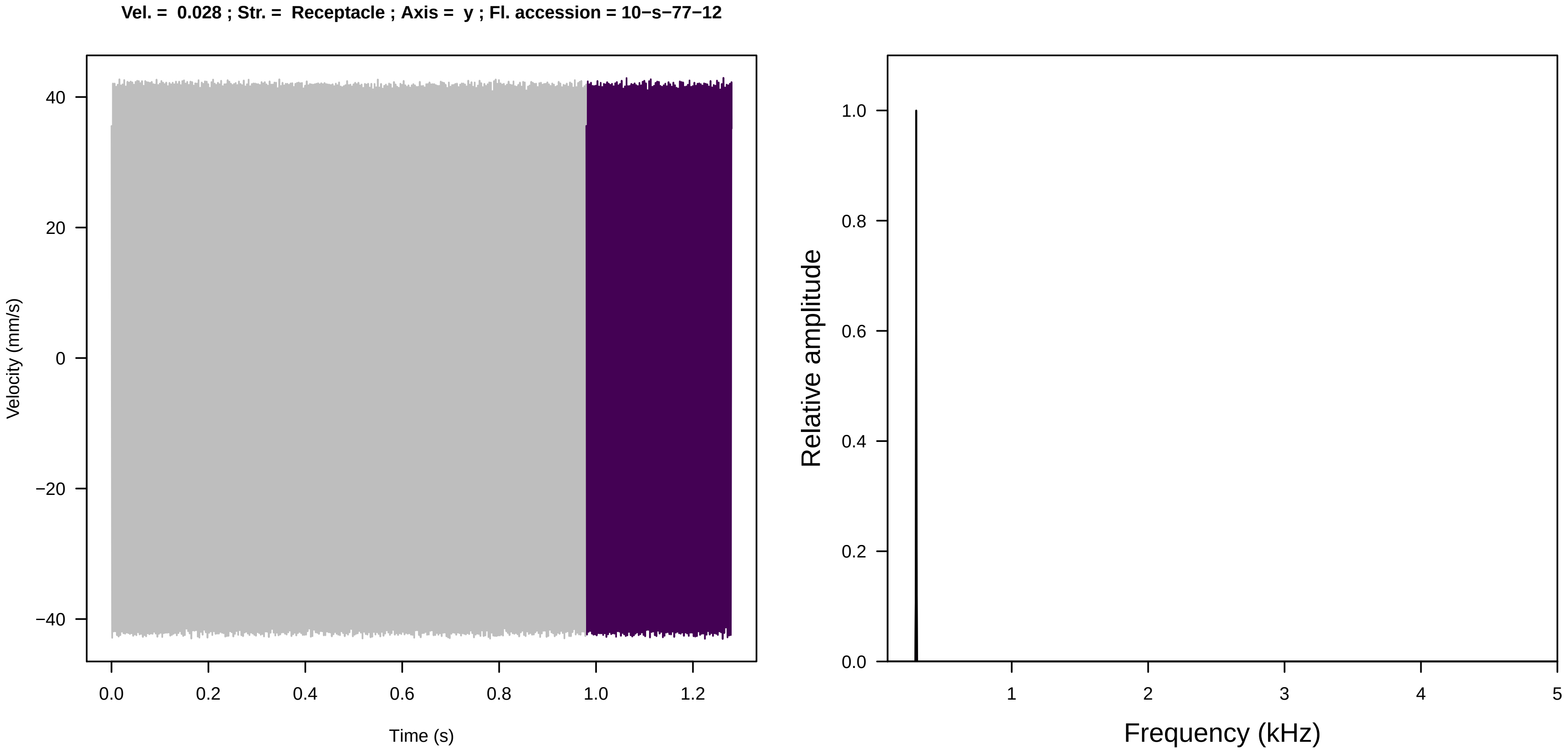
Vel. = 0.028 ; Str. = Receptacle ; Axis = y ; Fl. accession = 10−s−77−12
Velocity (mm/s)
Time (s)
Relative amplitude
Frequency (kHz)

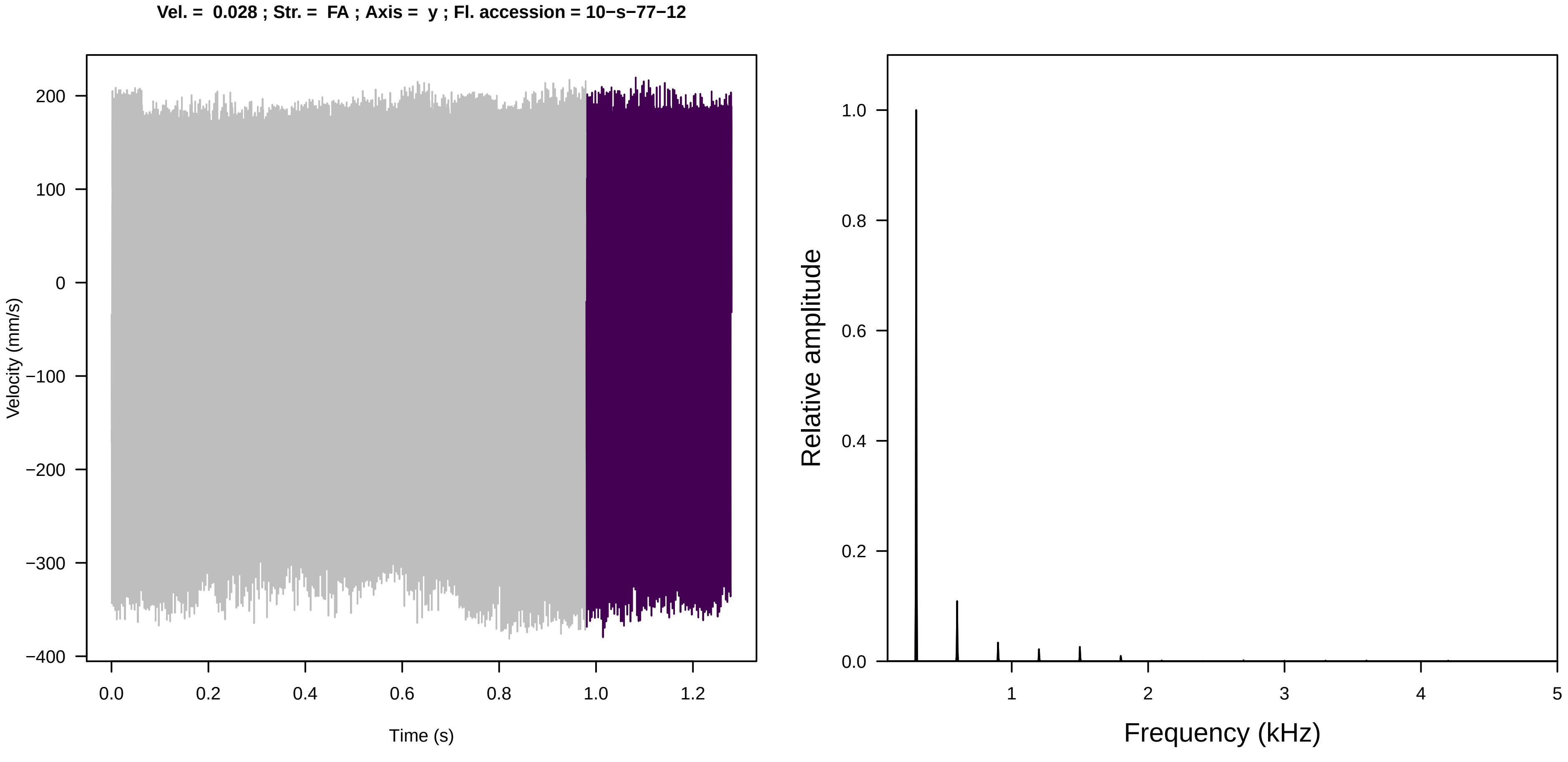
Vel. = 0.028 ; Str. = FA ; Axis = y ; Fl. accession = 10−s−77−12
Velocity (mm/s)
Time (s)
Relative amplitude
Frequency (kHz)

Vel. = 0.028 ; Str. = Receptacle ; Axis = y ; Fl. accession = 10−s−77−12

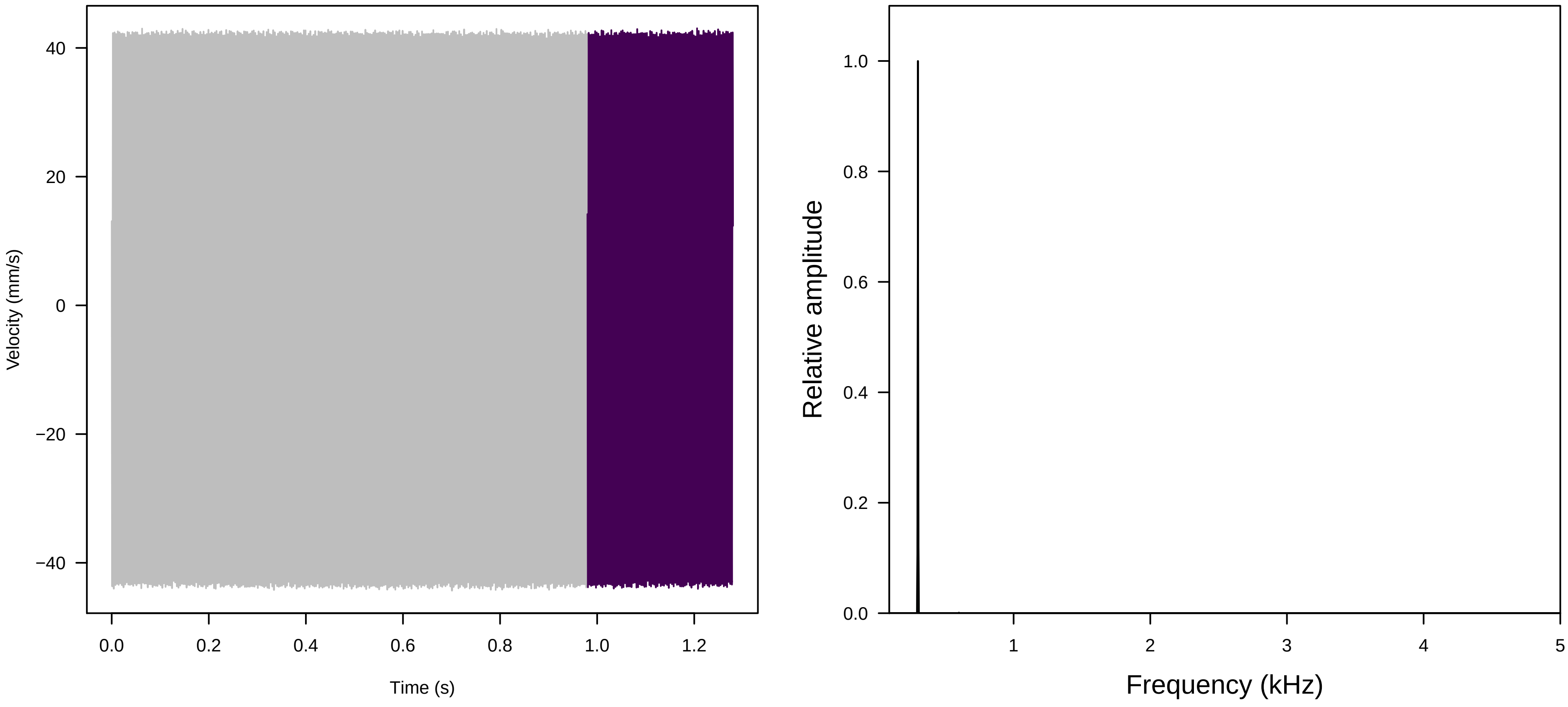

Vel. = 0.028 ; Str. = Corolla ; Axis = y ; Fl. accession = 10−s−77−12

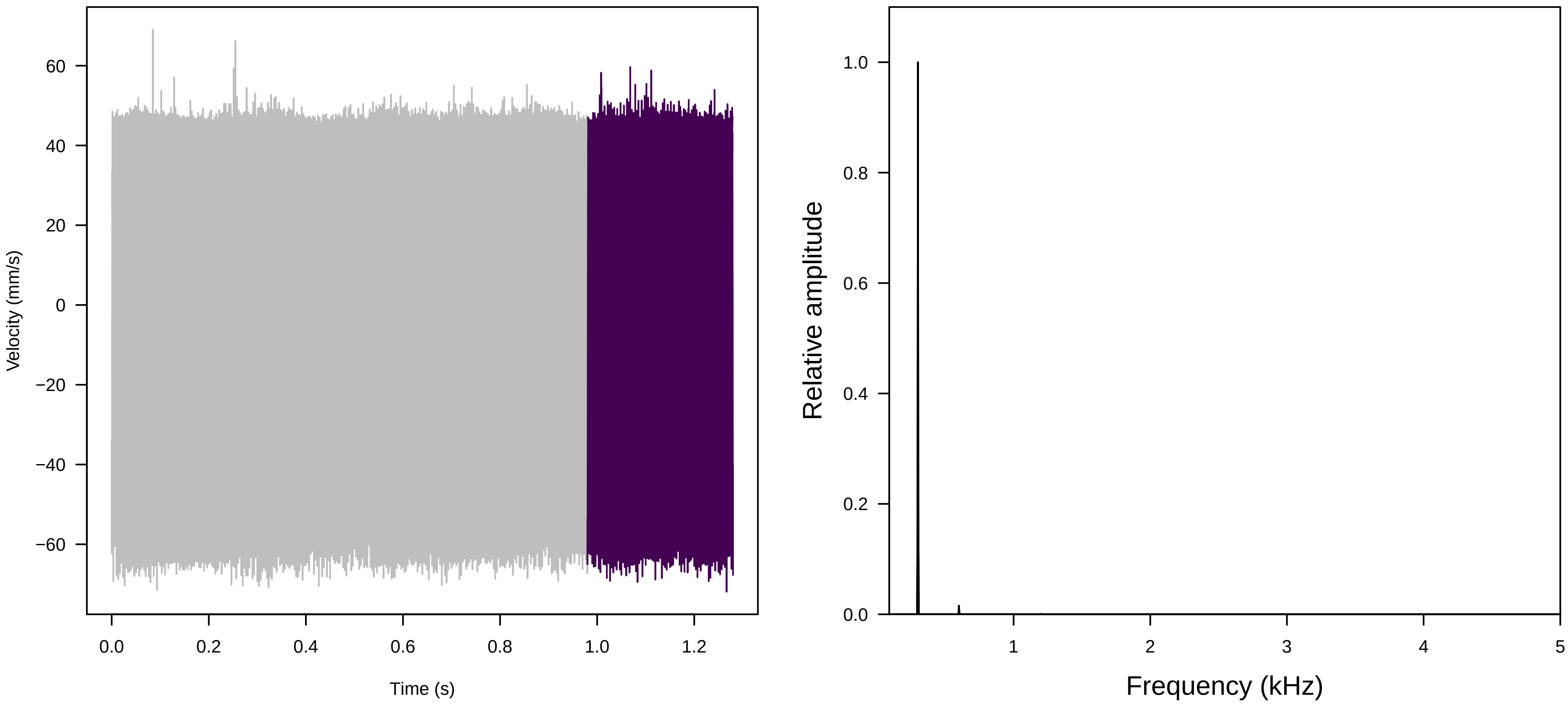

Vel. = 0.028 ; Str. = Receptacle ; Axis = y ; Fl. accession = 10−s−77−12

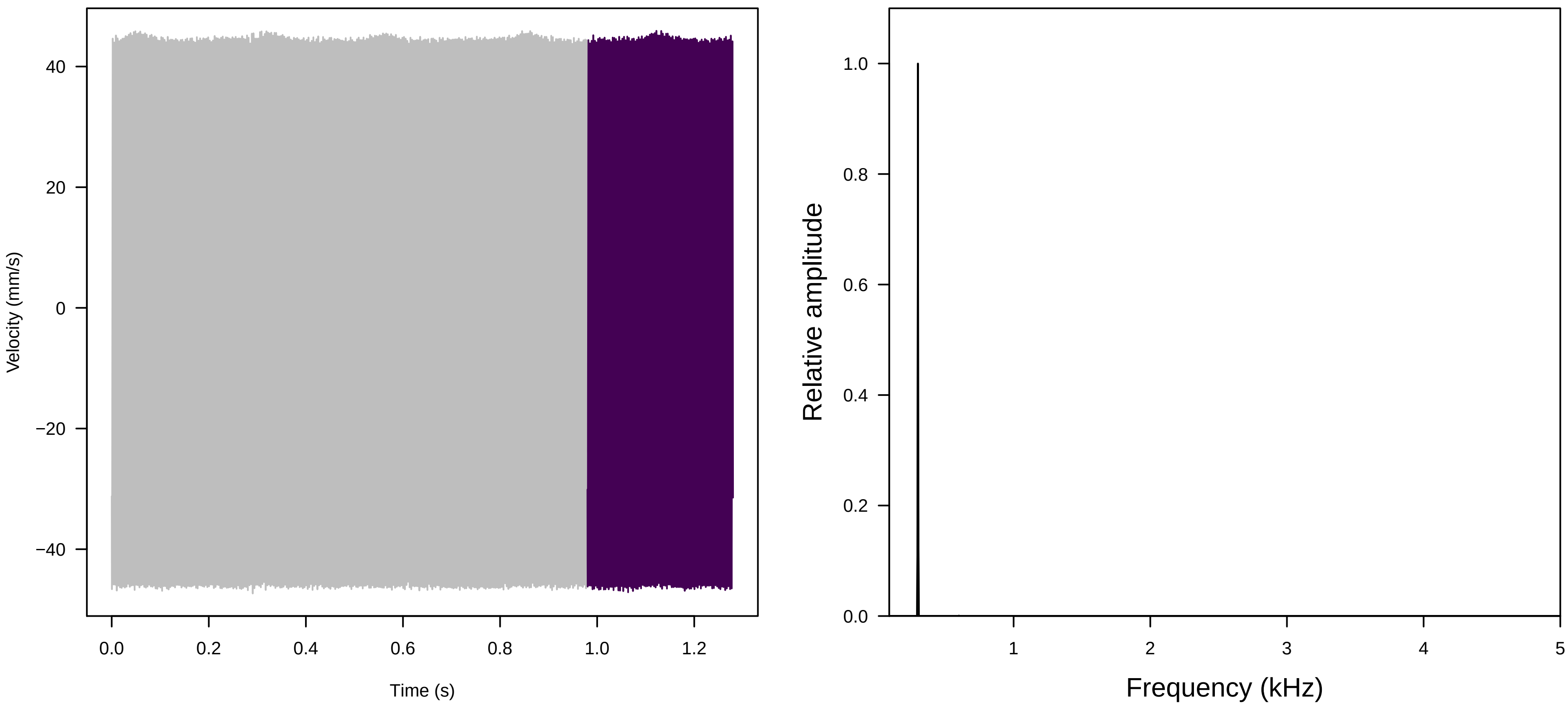

Vel. = 0.057 ; Str. = Corolla ; Axis = y ; Fl. accession = 10−s−77−12

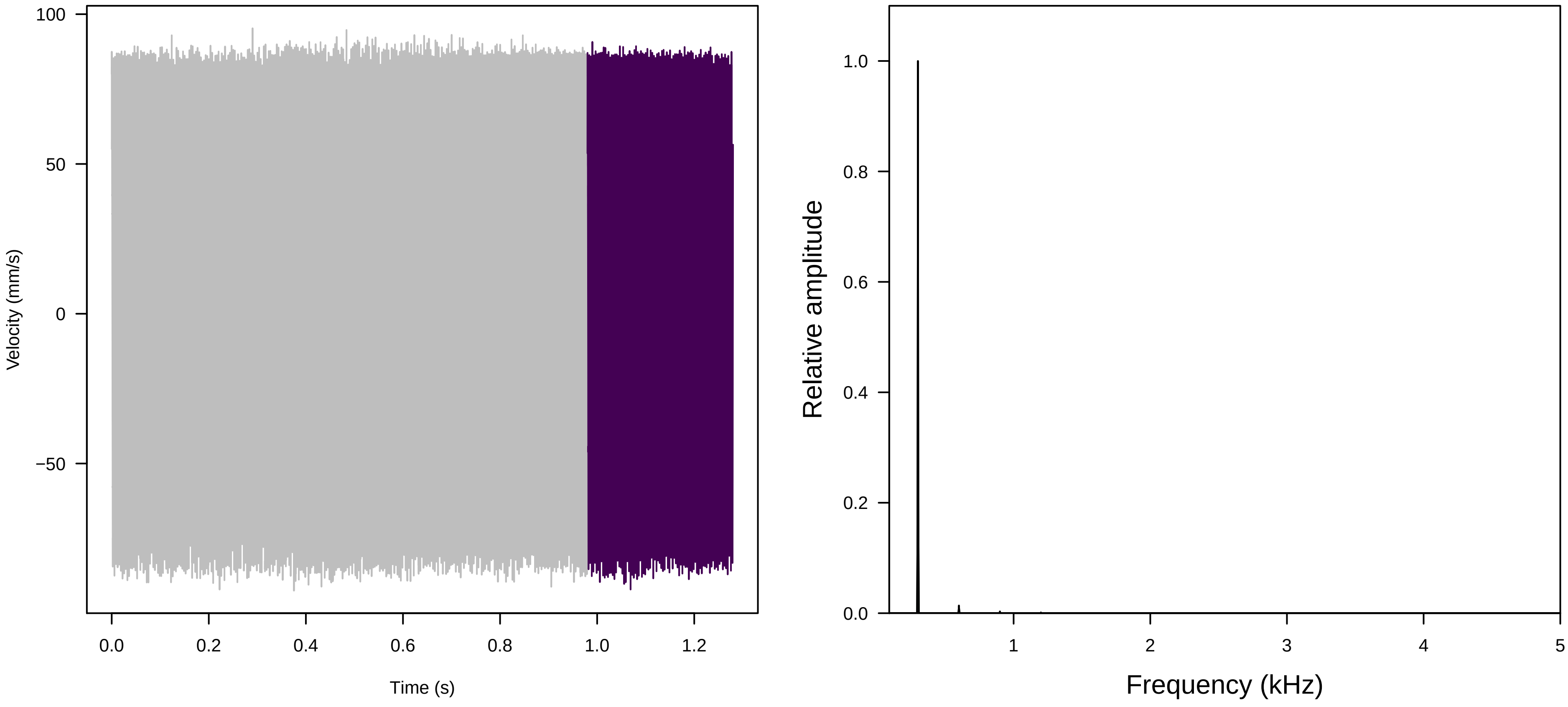

Vel. = 0.057 ; Str. = Receptacle ; Axis = y ; Fl. accession = 10−s−77−12

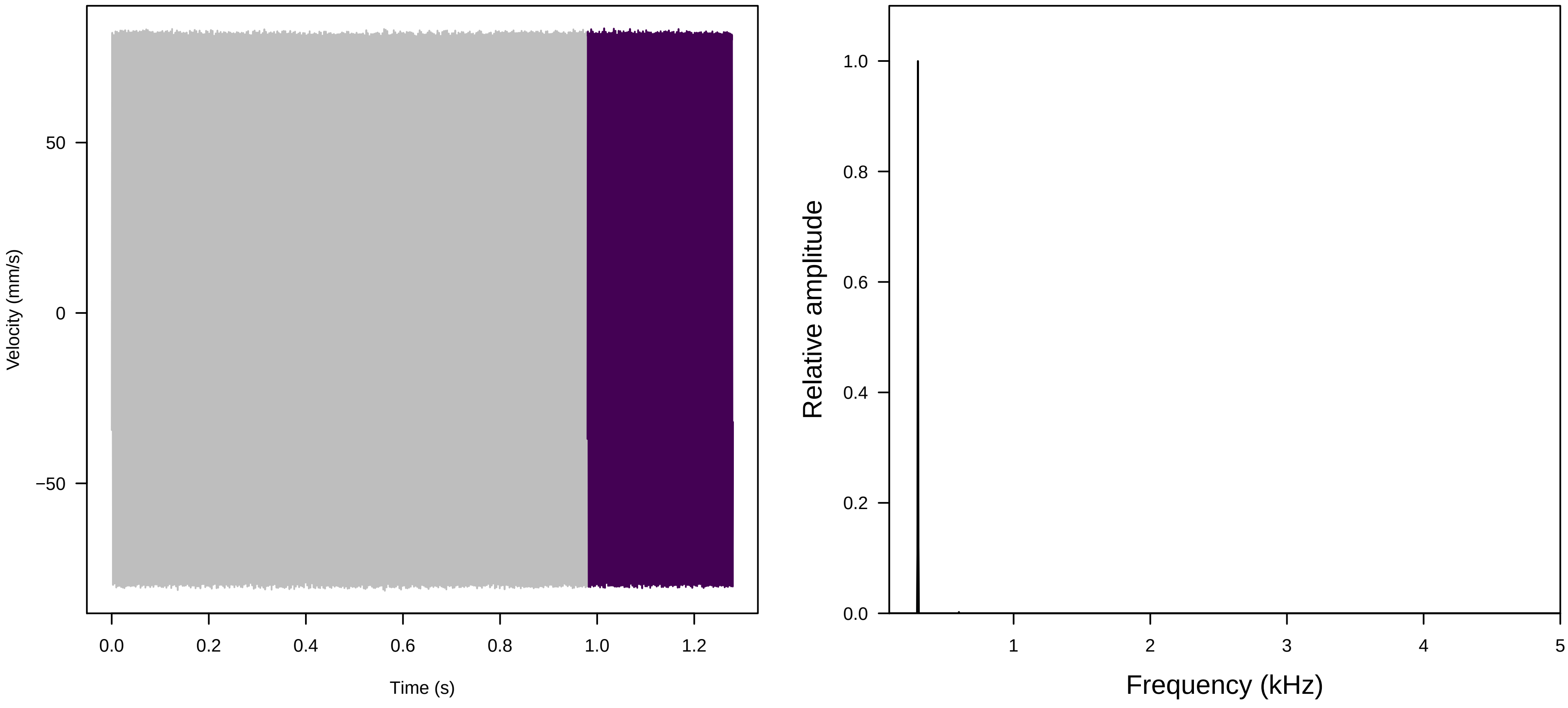

Vel. = 0.057 ; Str. = FA ; Axis = y ; Fl. accession = 10−s−77−12

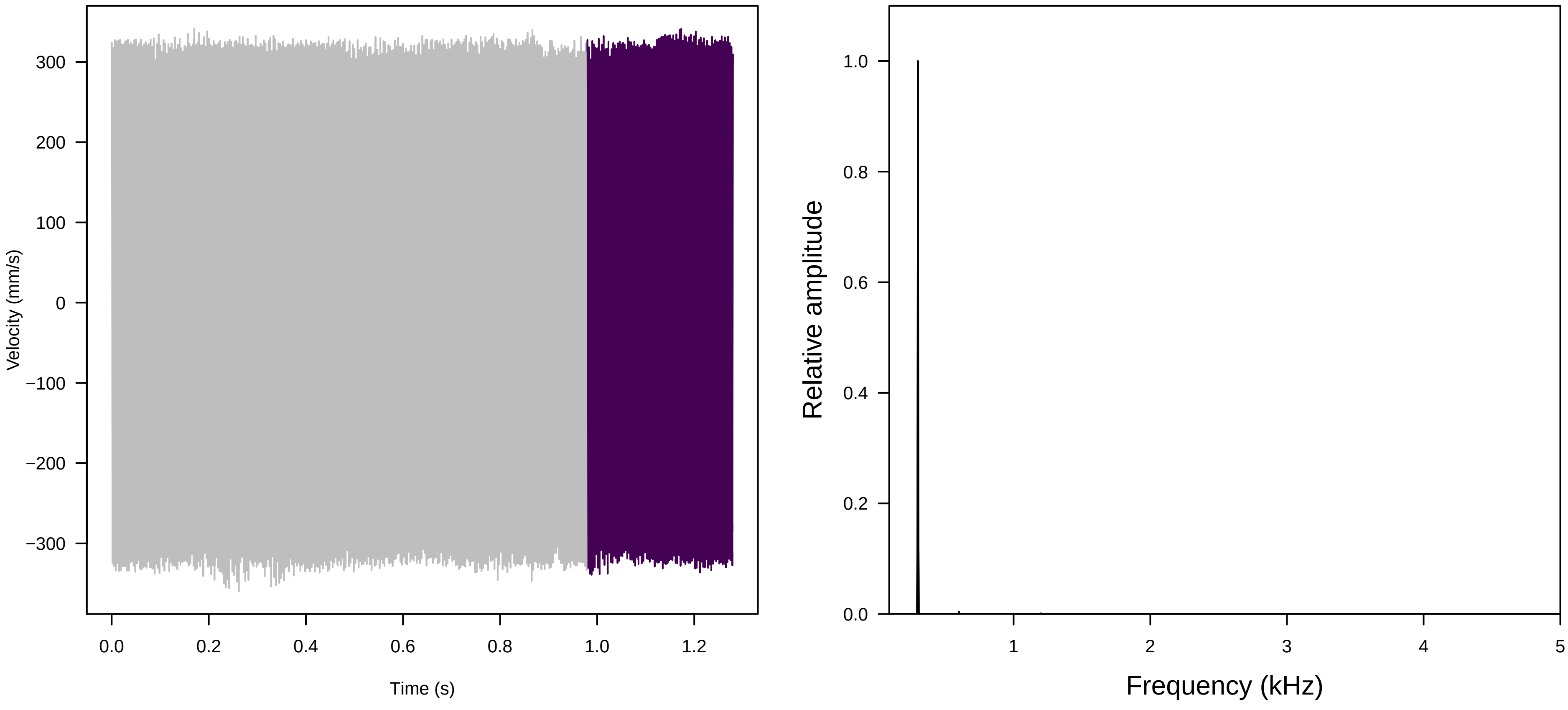

Vel. = 0.057 ; Str. = Receptacle ; Axis = y ; Fl. accession = 10−s−77−12

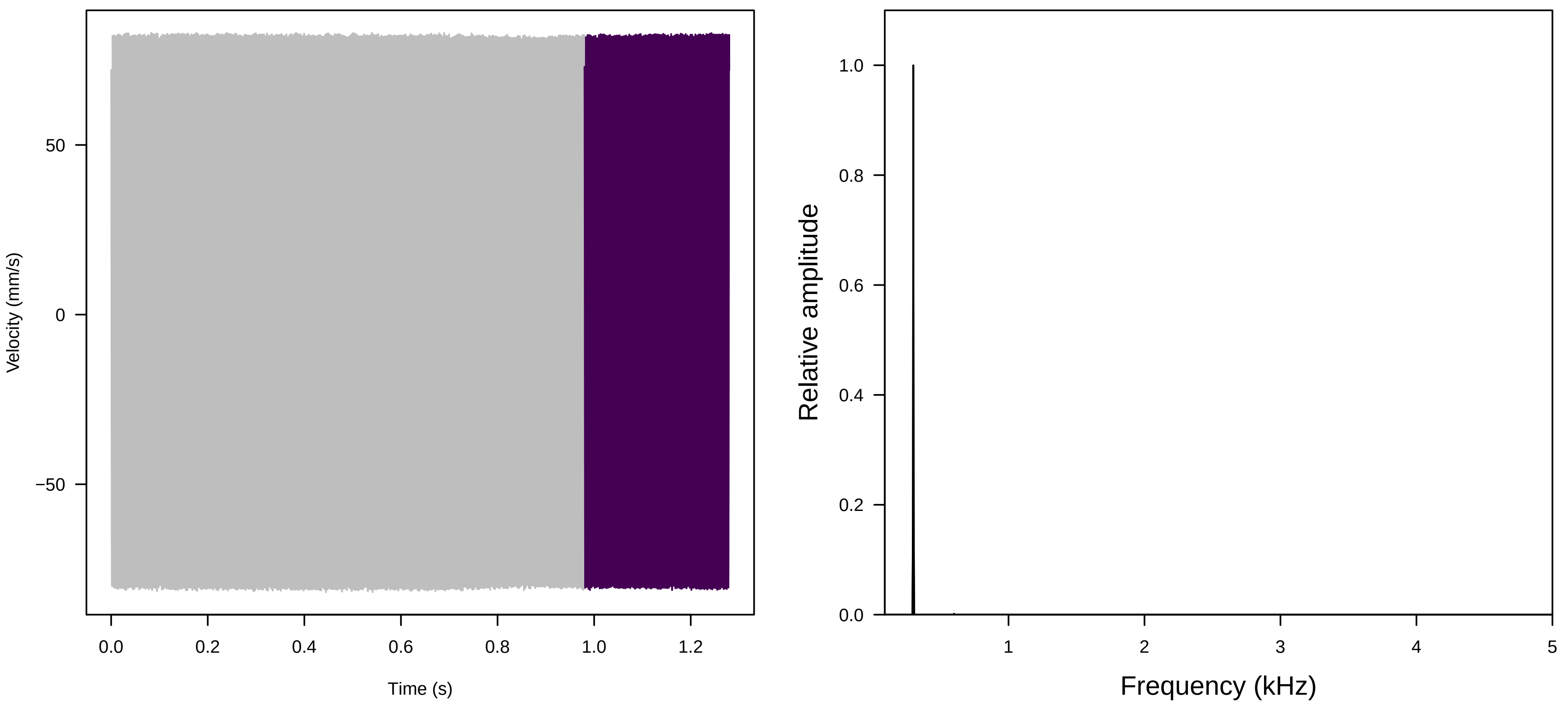

Vel. = 0.057 ; Str. = PA ; Axis = y ; Fl. accession = 10−s−77−12

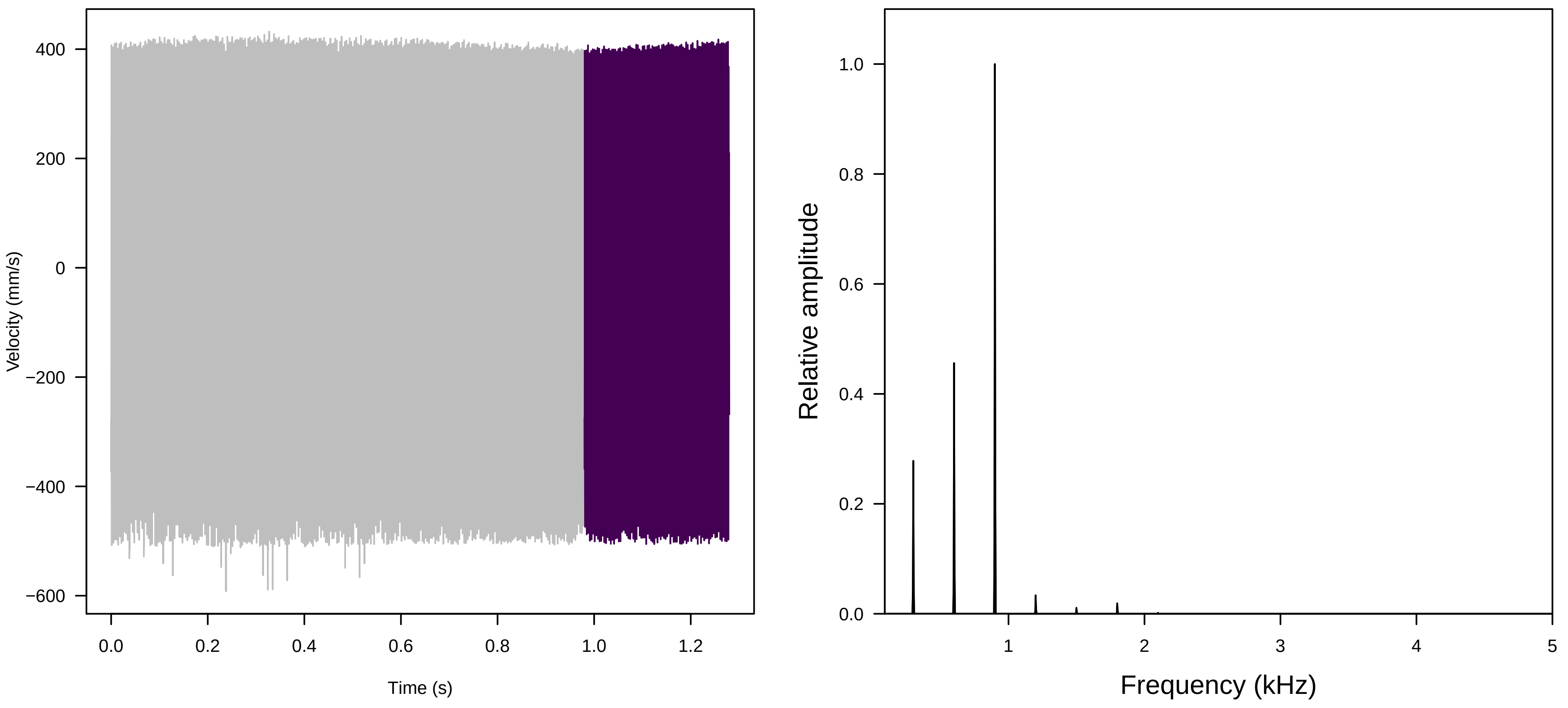

Vel. = 0.057 ; Str. = Receptacle ; Axis = y ; Fl. accession = 10−s−77−12

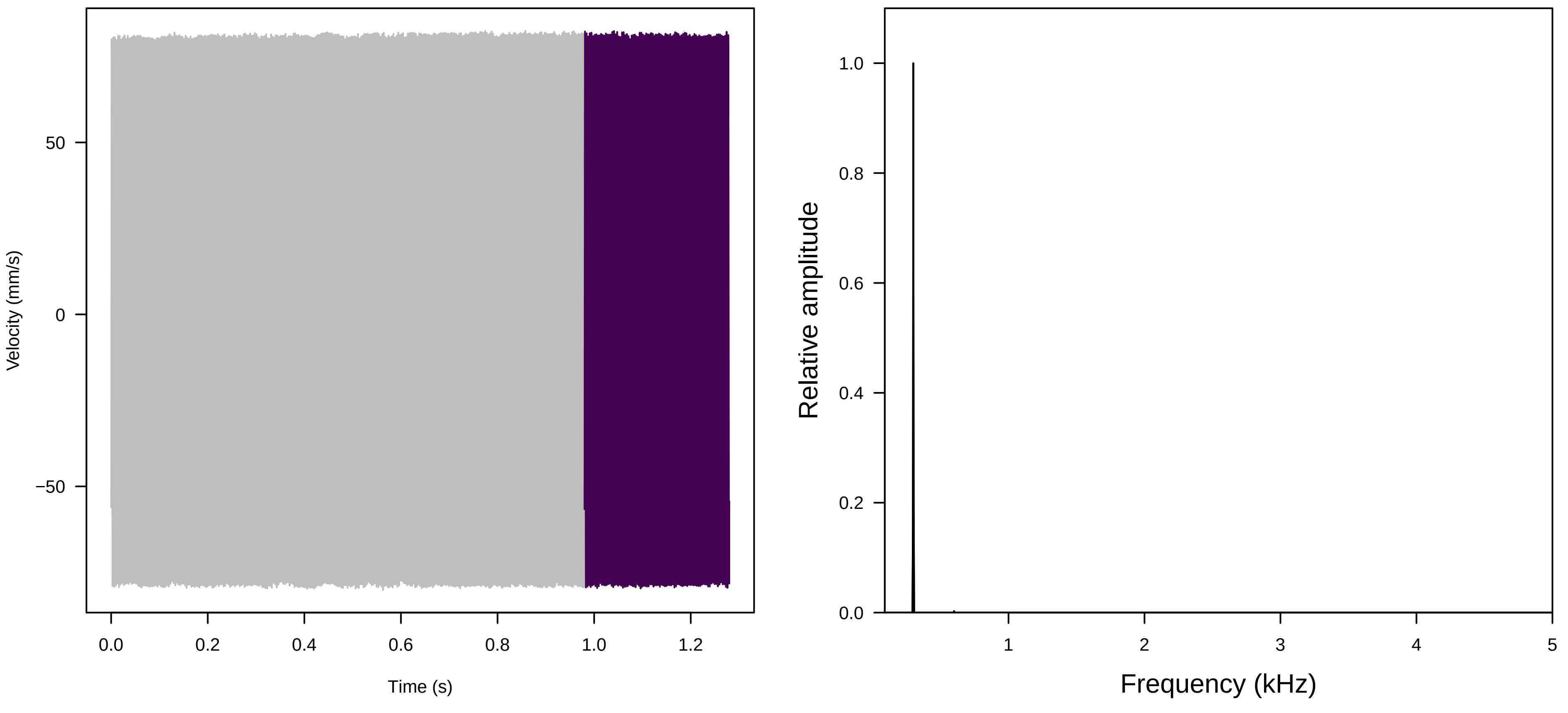

Vel. = 0.014 ; Str. = Corolla ; Axis = y ; Fl. accession = 10−s−86

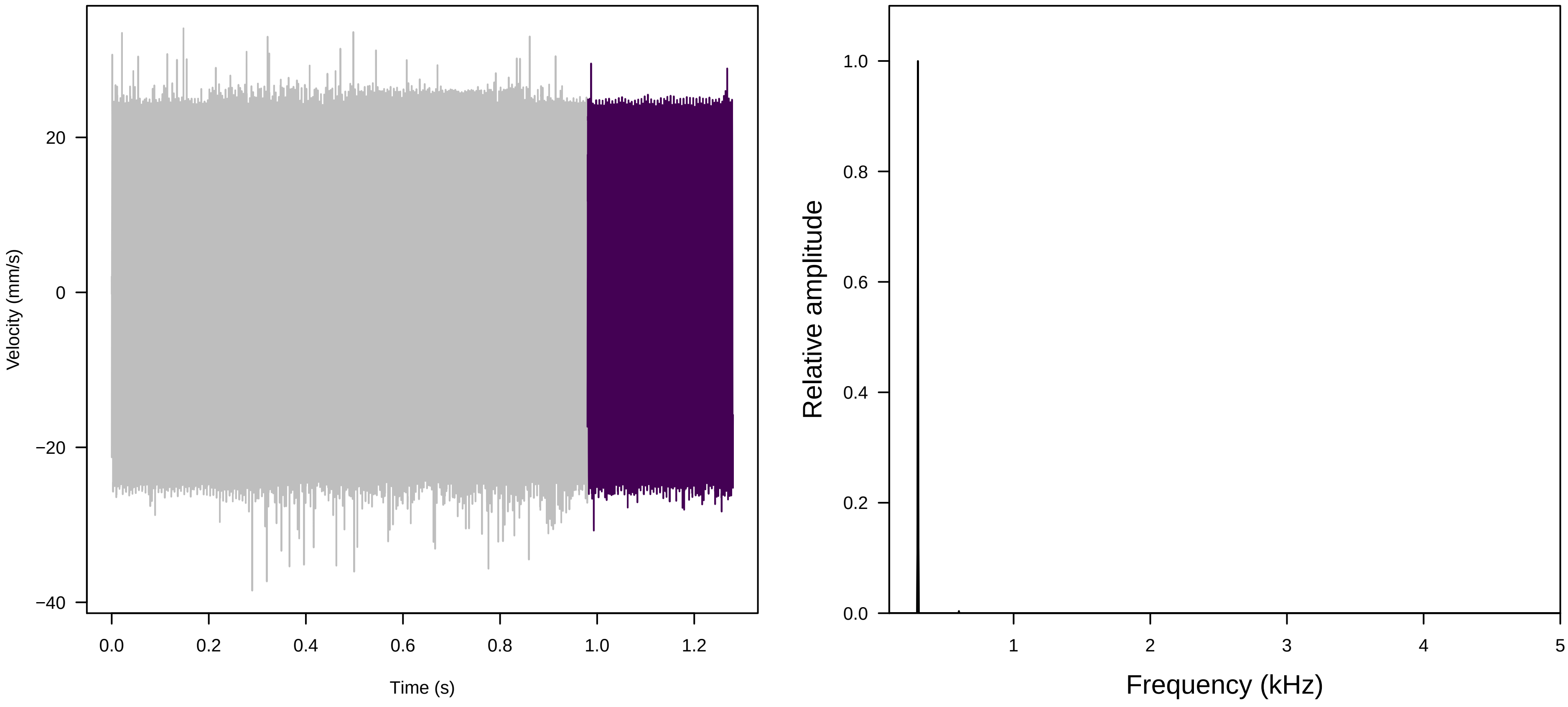

Vel. = 0.014 ; Str. = Receptacle ; Axis = y ; Fl. accession = 10−s−86

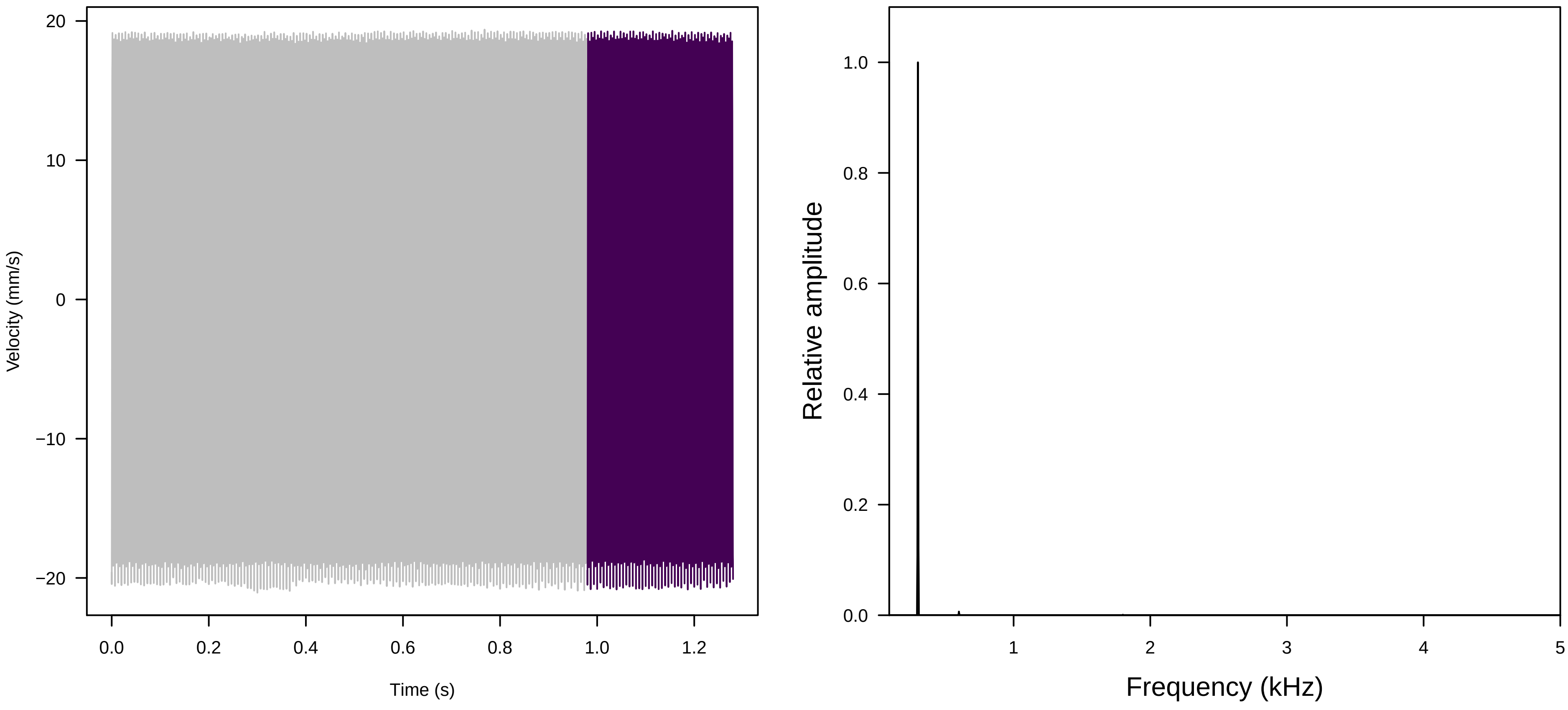

Vel. = 0.014 ; Str. = FA ; Axis = y ; Fl. accession = 10−s−86

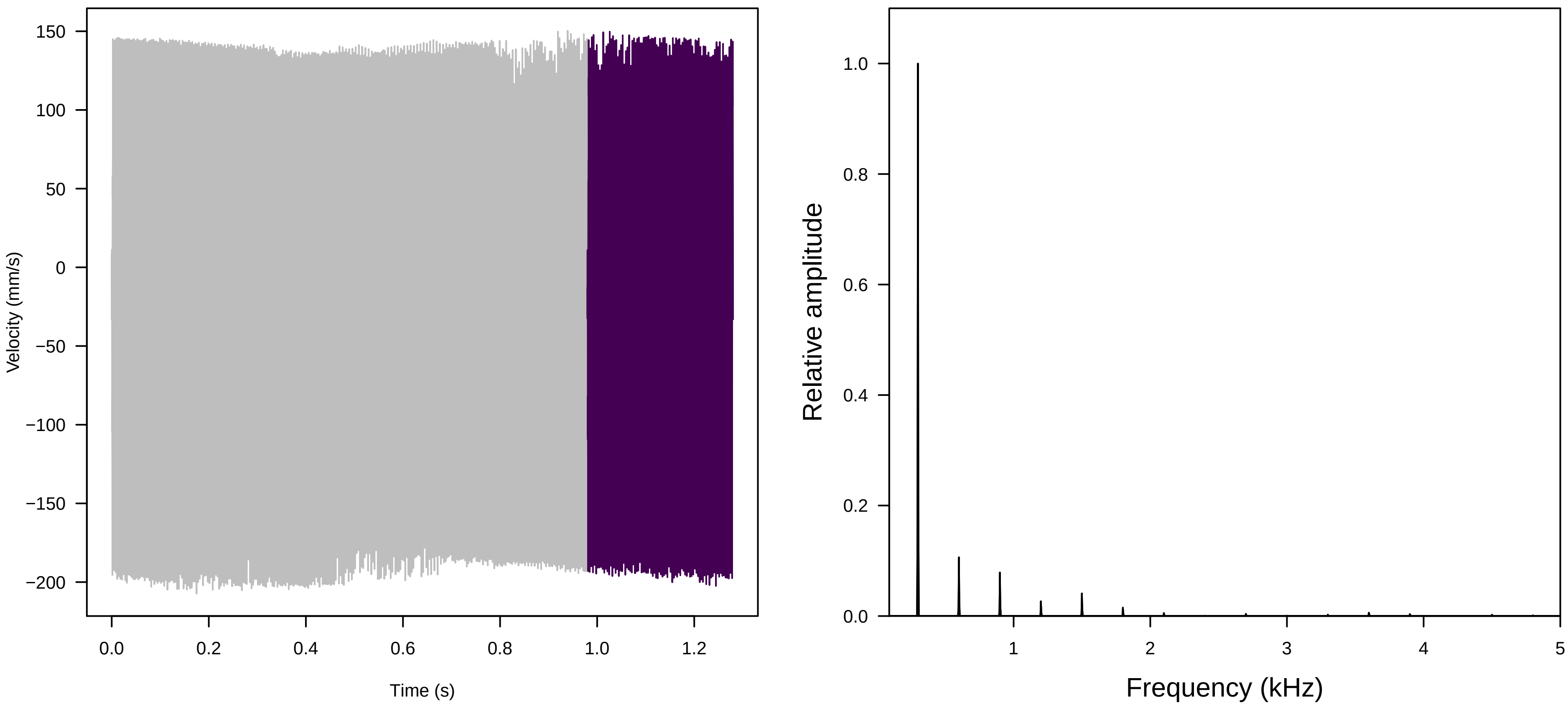

Vel. = 0.014 ; Str. = Receptacle ; Axis = y ; Fl. accession = 10−s−86

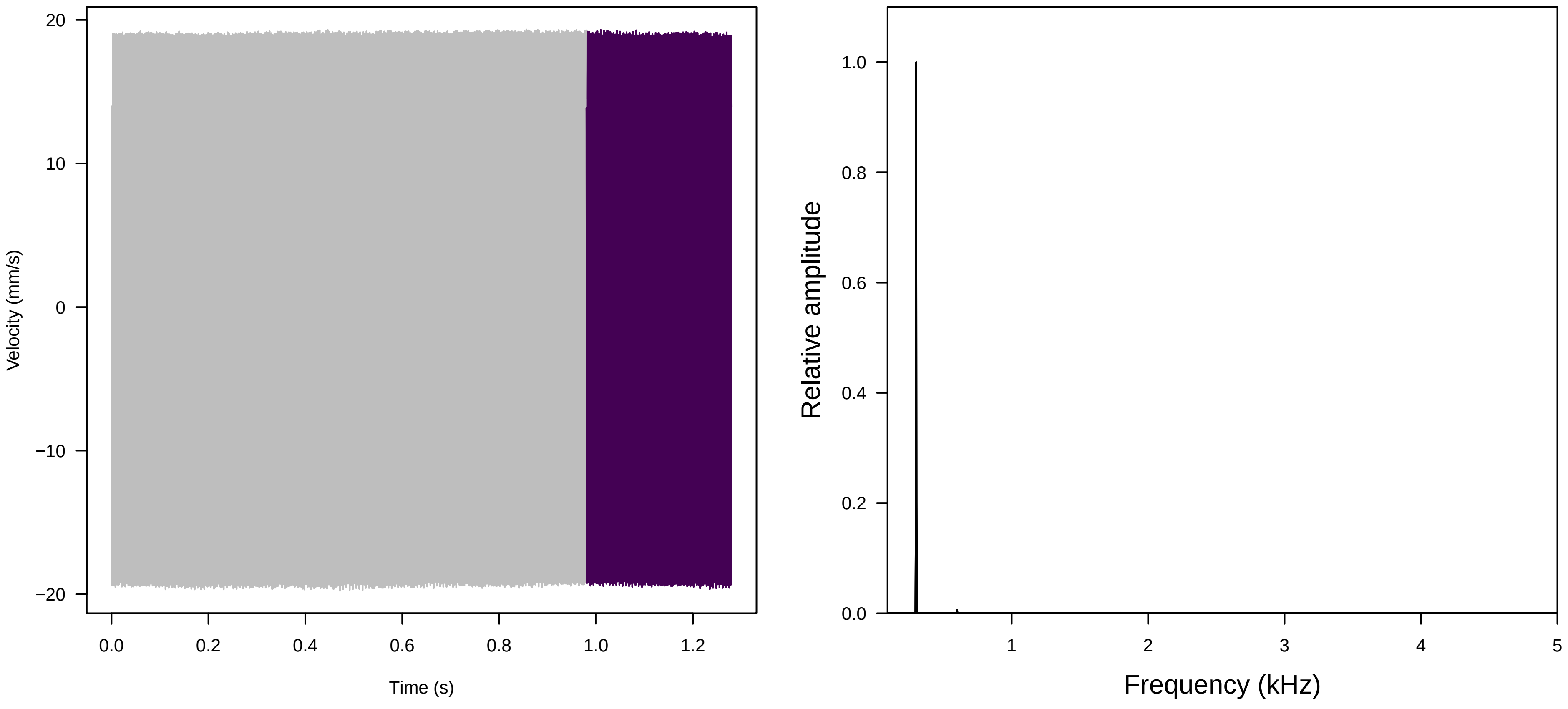

Vel. = 0.014 ; Str. = PA ; Axis = y ; Fl. accession = 10−s−86

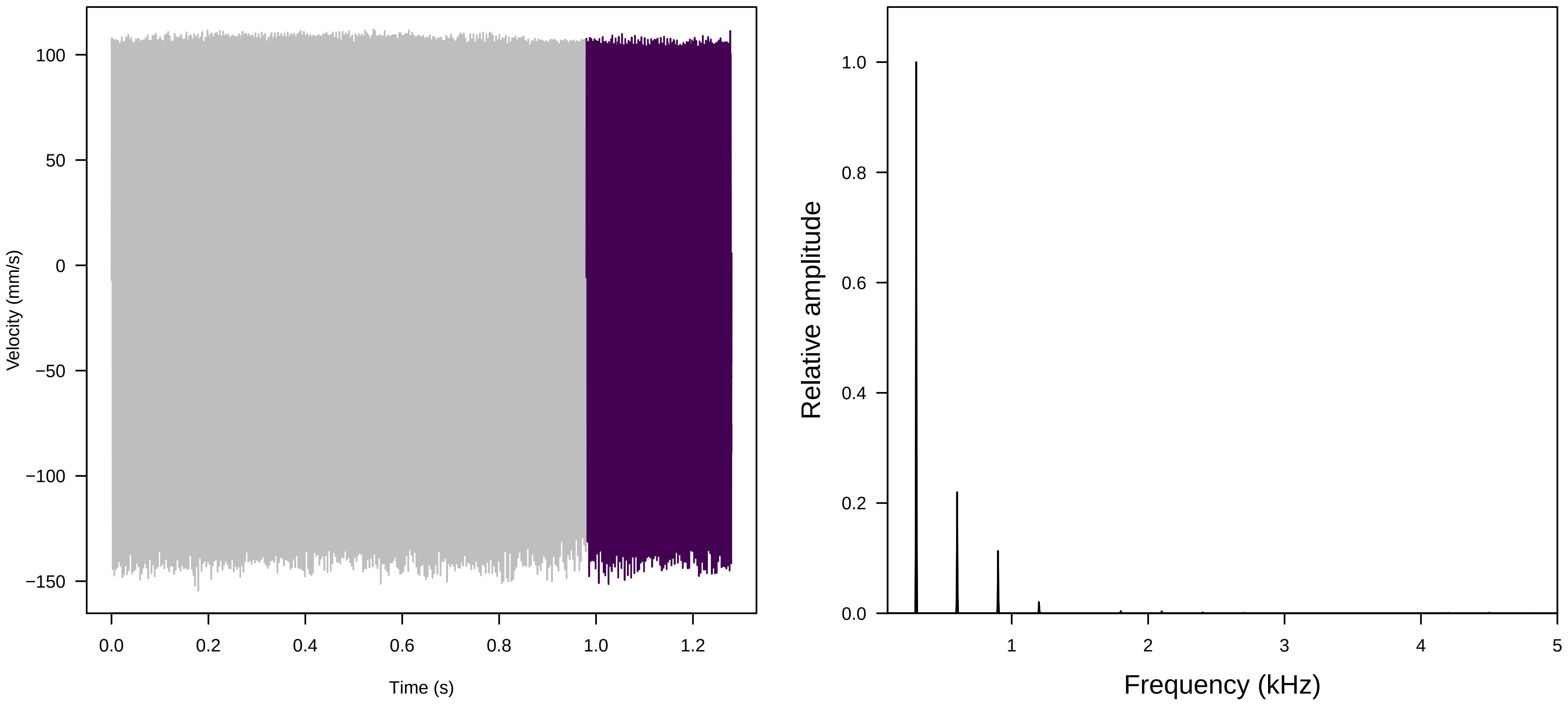

Vel. = 0.014 ; Str. = Receptacle ; Axis = y ; Fl. accession = 10−s−86

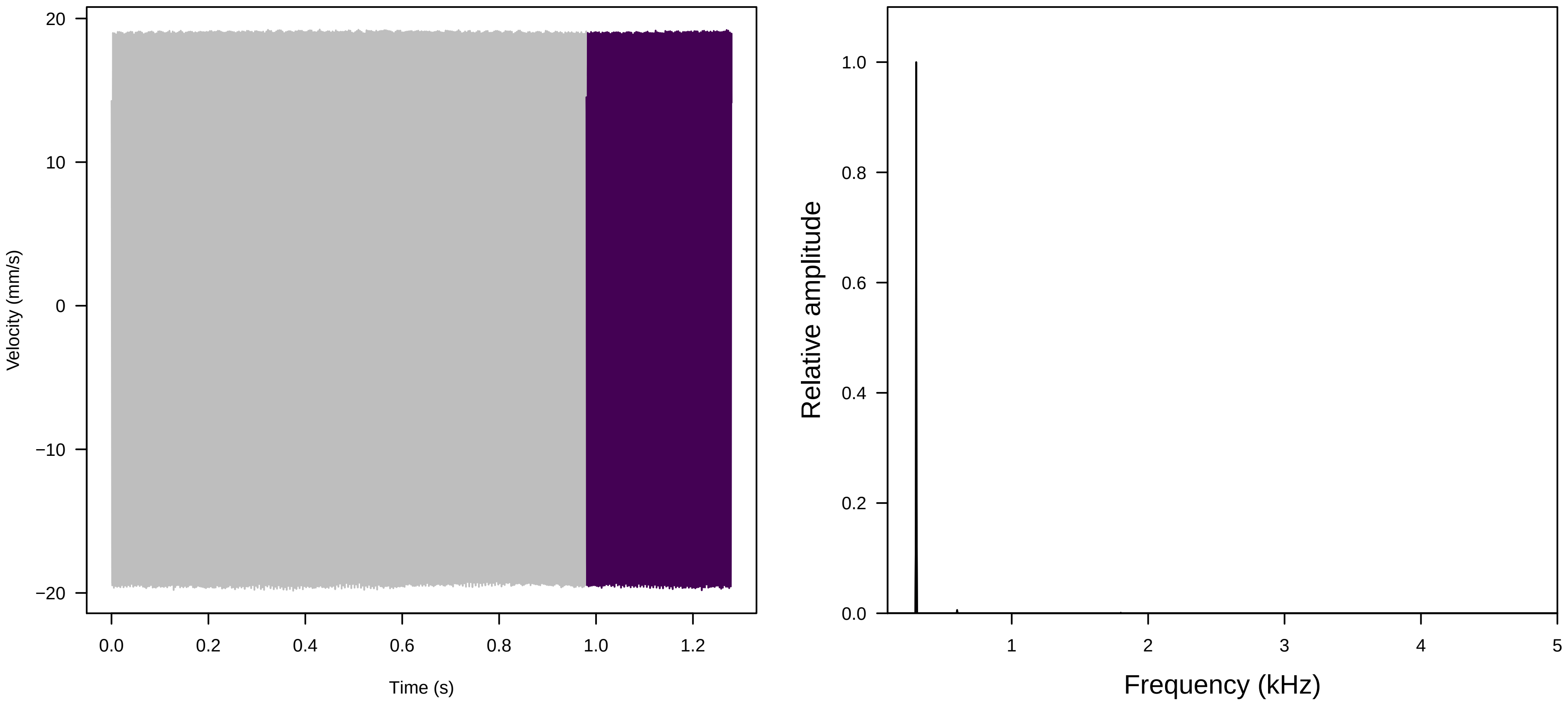

Vel. = 0.028 ; Str. = PA ; Axis = y ; Fl. accession = 10−s−86

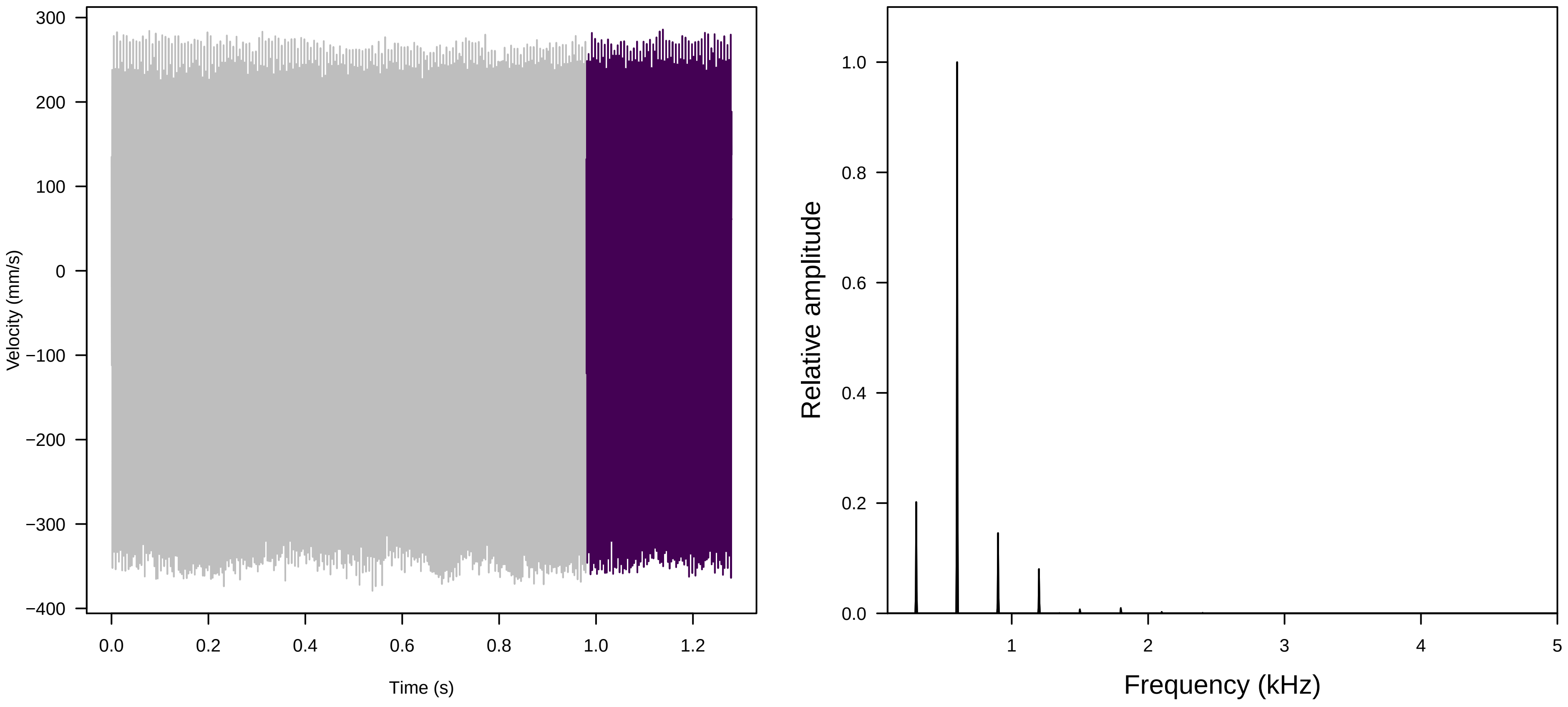

Vel. = 0.028 ; Str. = Receptacle ; Axis = y ; Fl. accession = 10−s−86

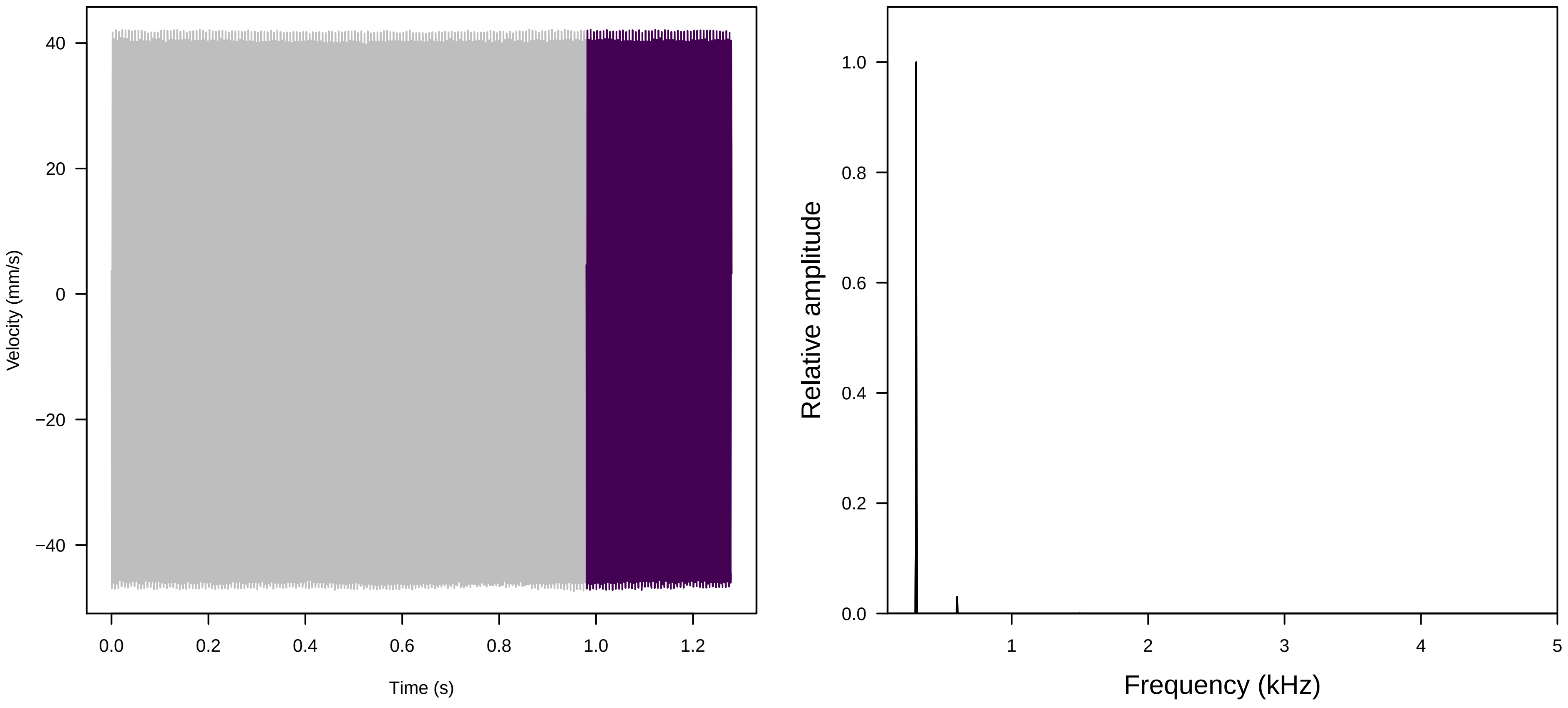

Vel. = 0.028 ; Str. = FA ; Axis = y ; Fl. accession = 10−s−86

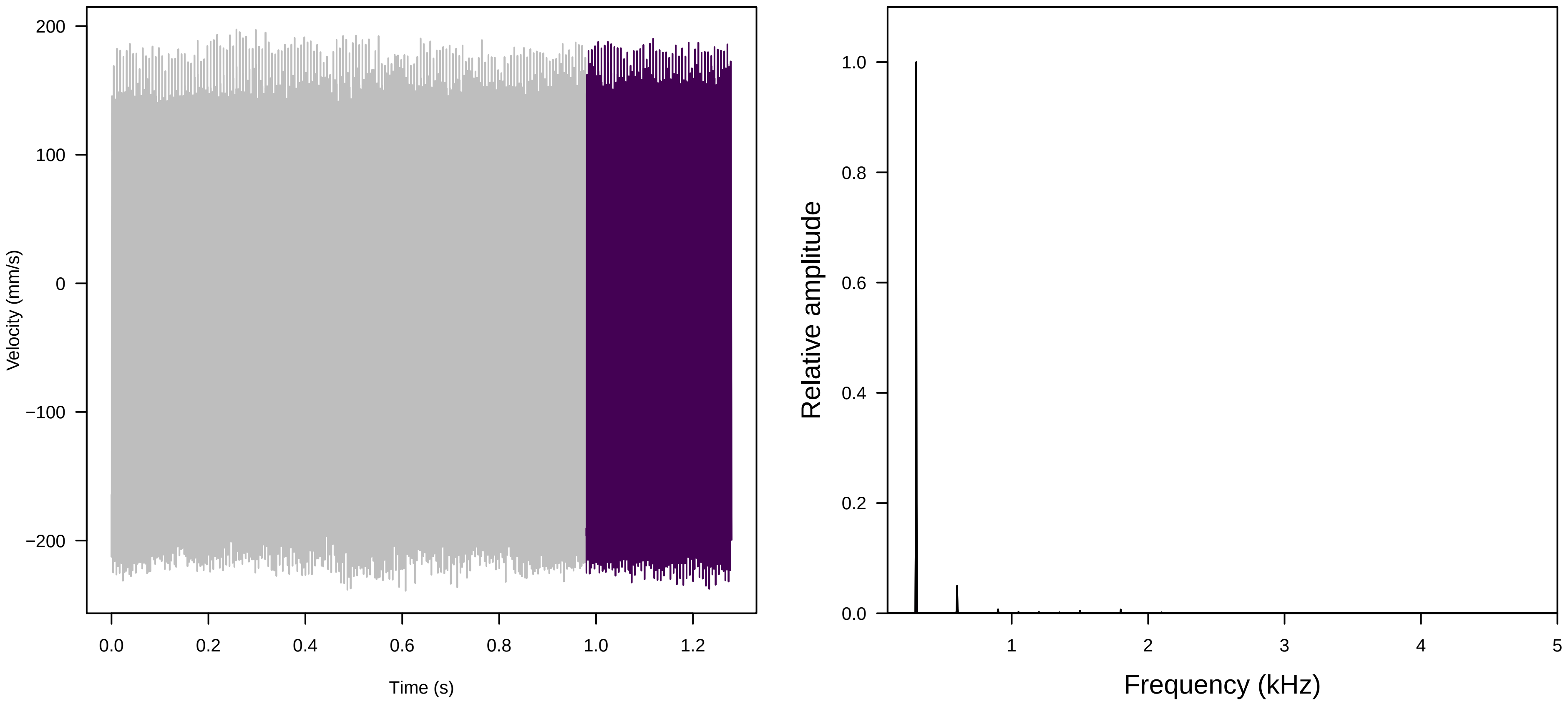

Vel. = 0.028 ; Str. = Receptacle ; Axis = y ; Fl. accession = 10−s−86

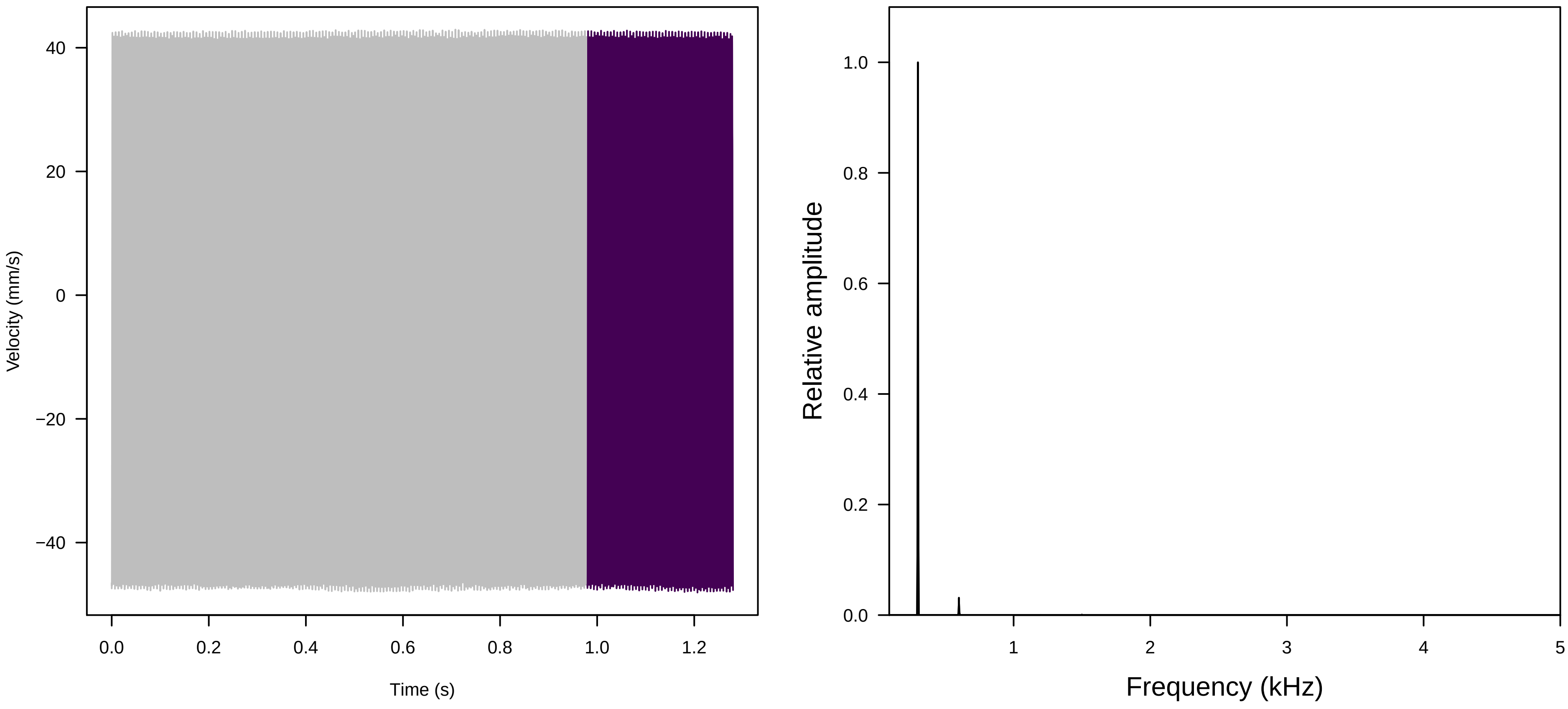

Vel. = 0.028 ; Str. = Corolla ; Axis = y ; Fl. accession = 10−s−86

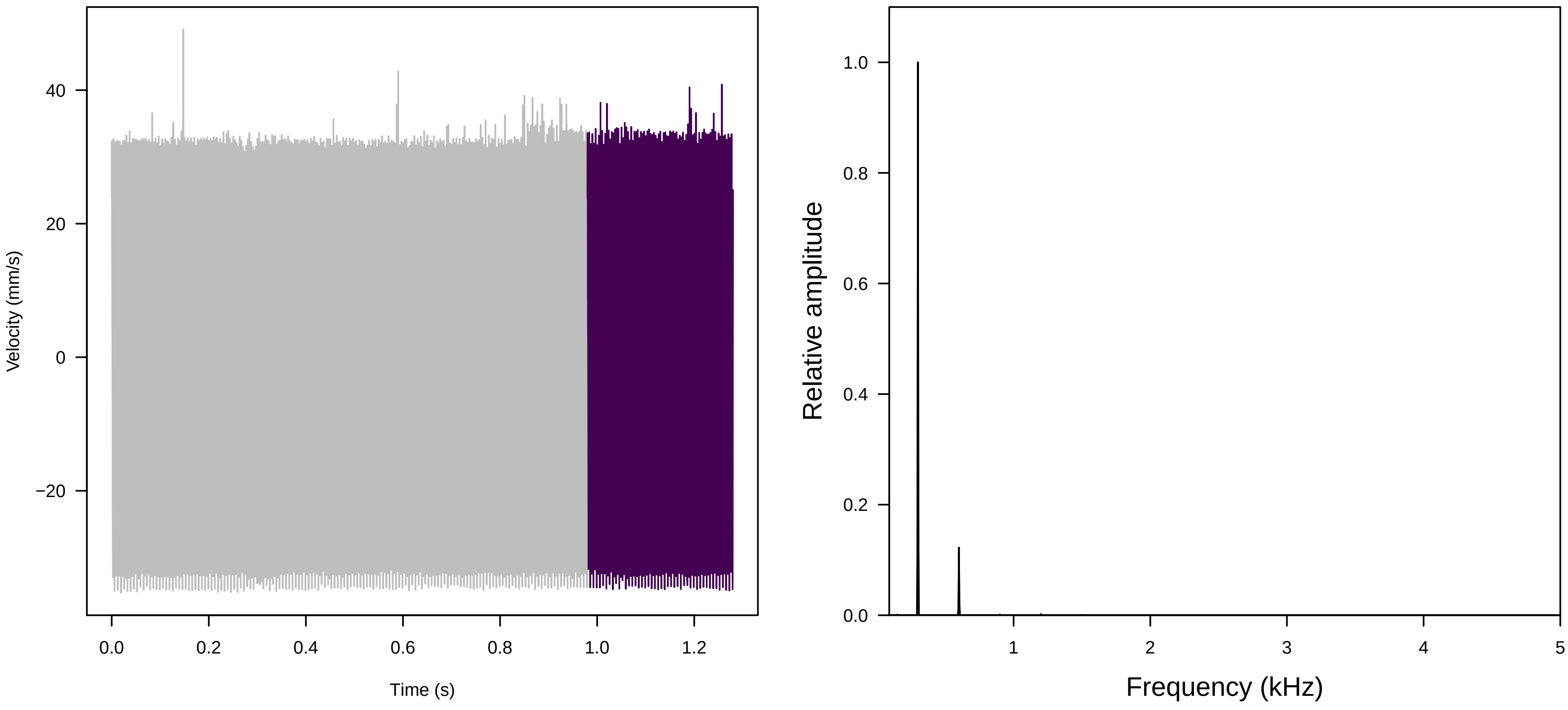

Vel. = 0.028 ; Str. = Receptacle ; Axis = y ; Fl. accession = 10−s−86

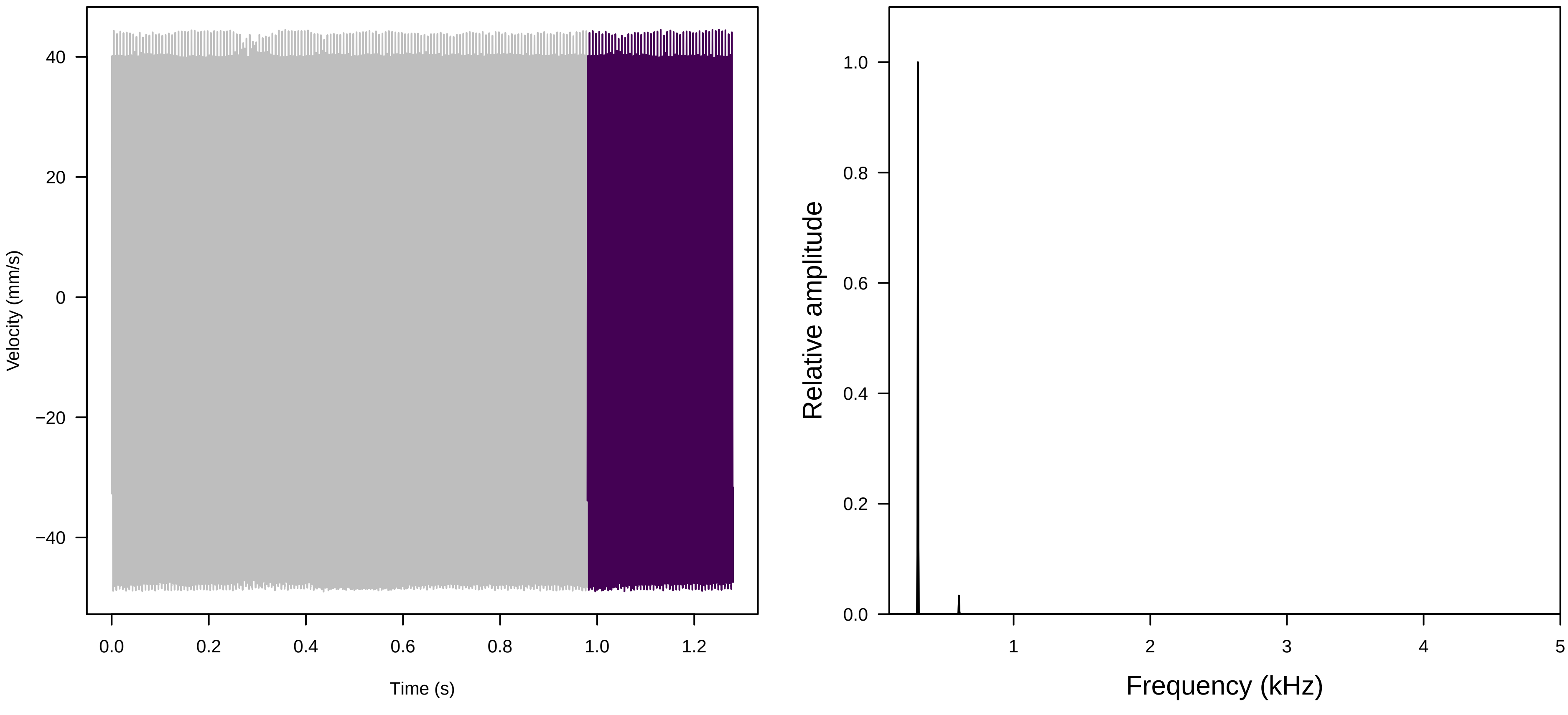

Vel. = 0.057 ; Str. = Corolla ; Axis = y ; Fl. accession = 10−s−86

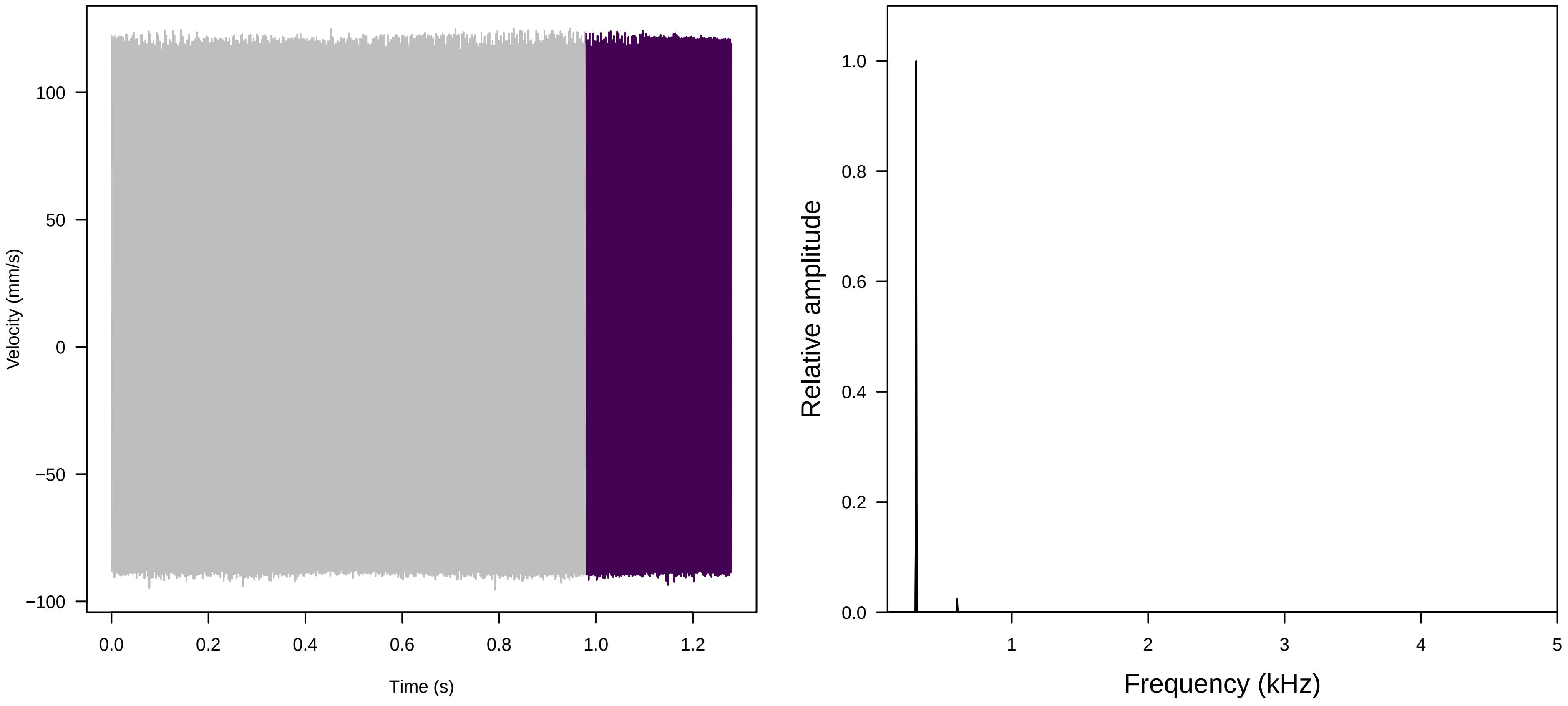

Vel. = 0.057 ; Str. = Receptacle ; Axis = y ; Fl. accession = 10−s−86

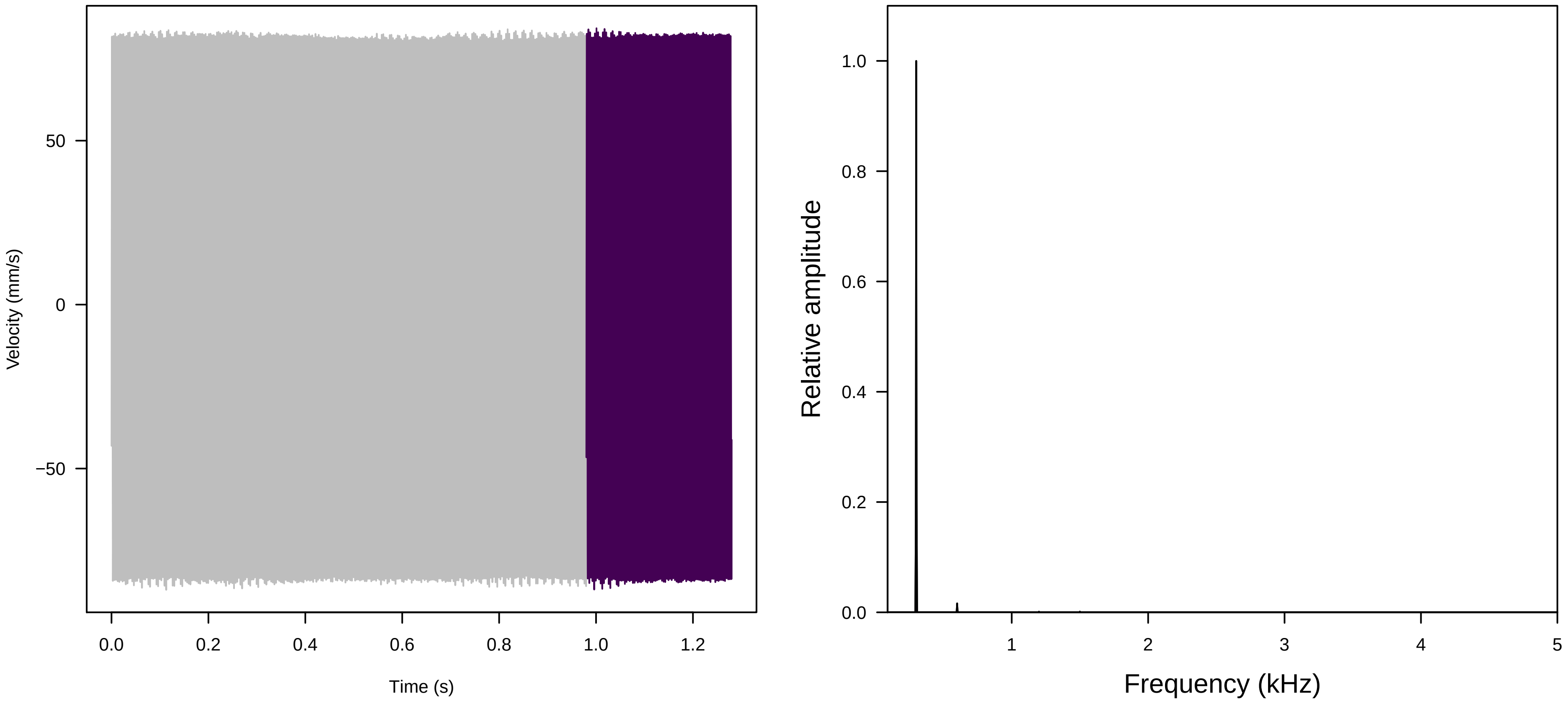

Vel. = 0.057 ; Str. = FA ; Axis = y ; Fl. accession = 10−s−86

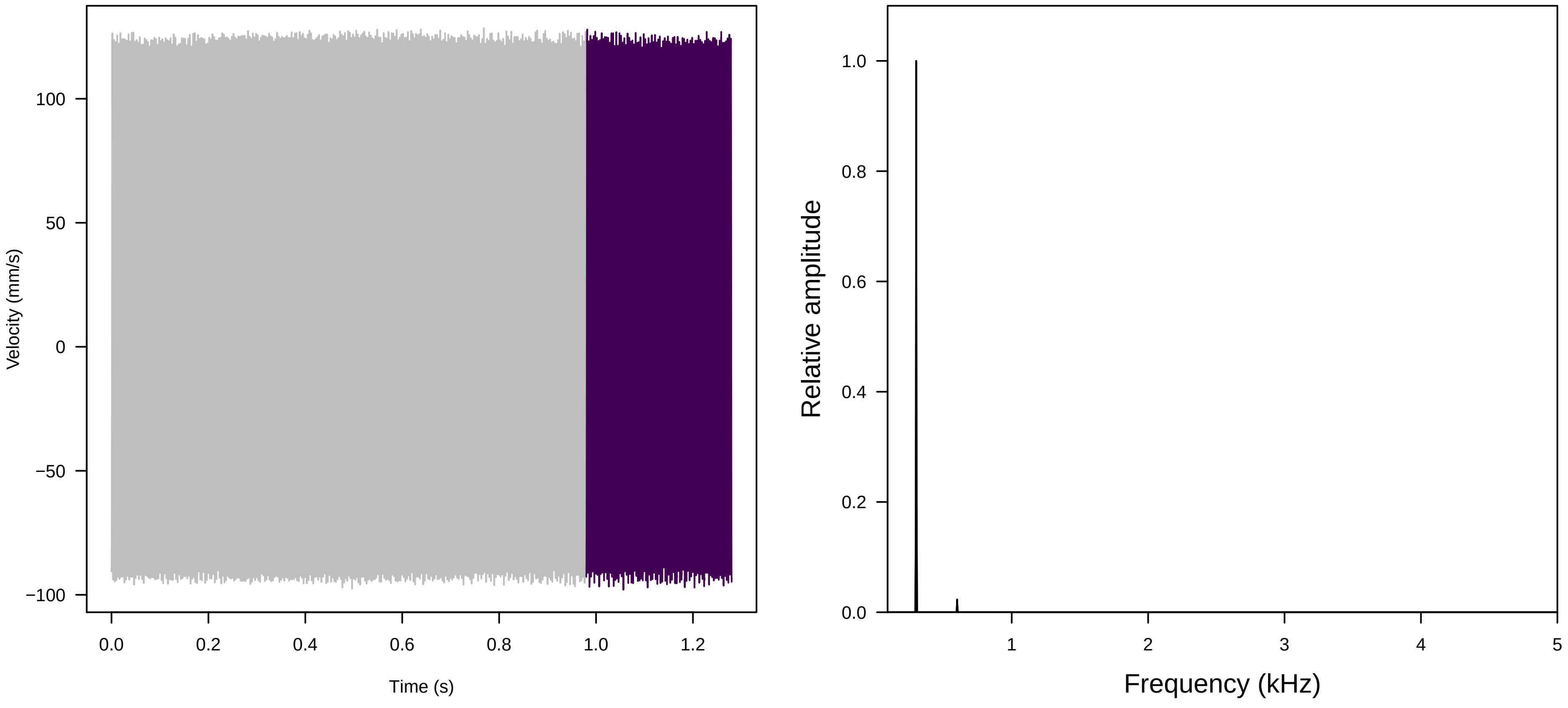

Vel. = 0.057 ; Str. = Receptacle ; Axis = y ; Fl. accession = 10−s−86

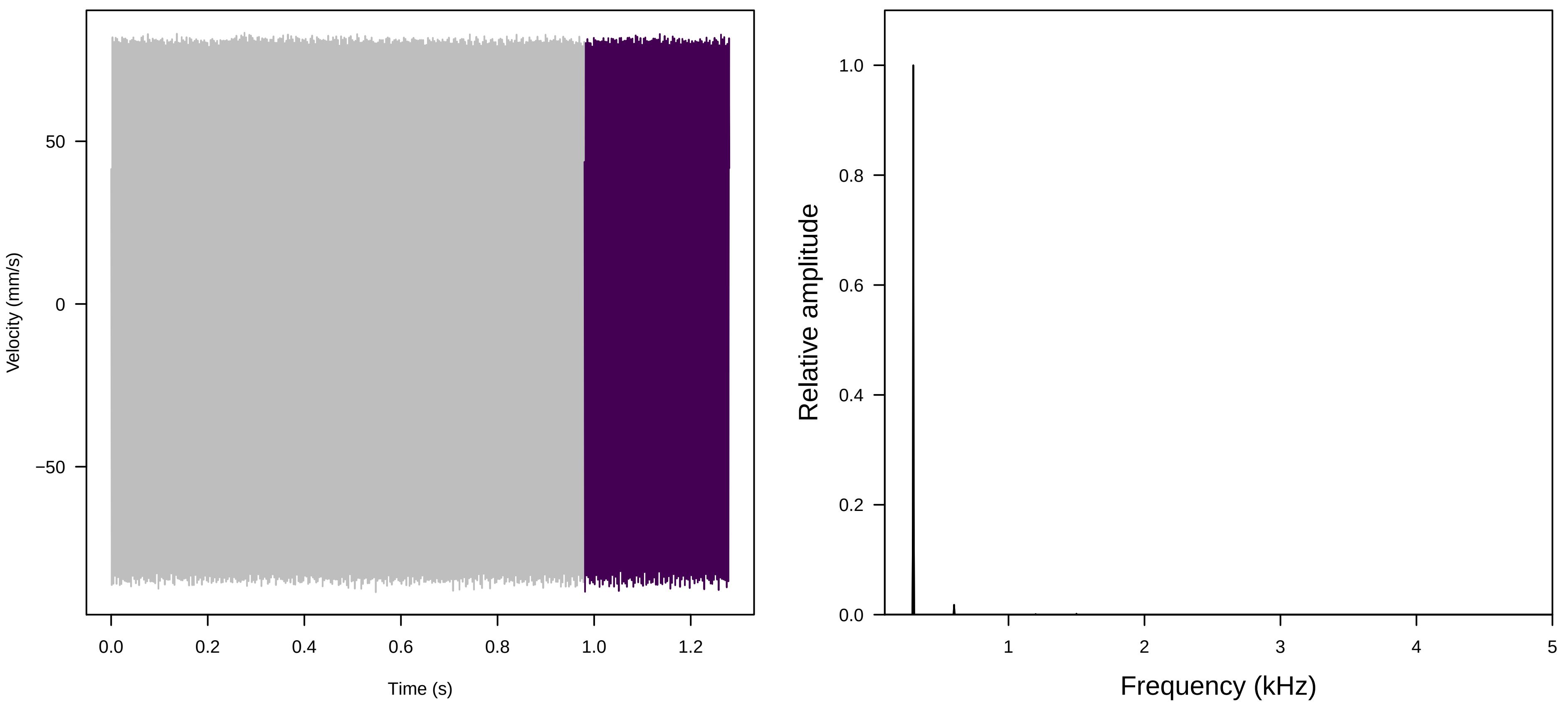

Vel. = 0.057 ; Str. = PA ; Axis = y ; Fl. accession = 10−s−86

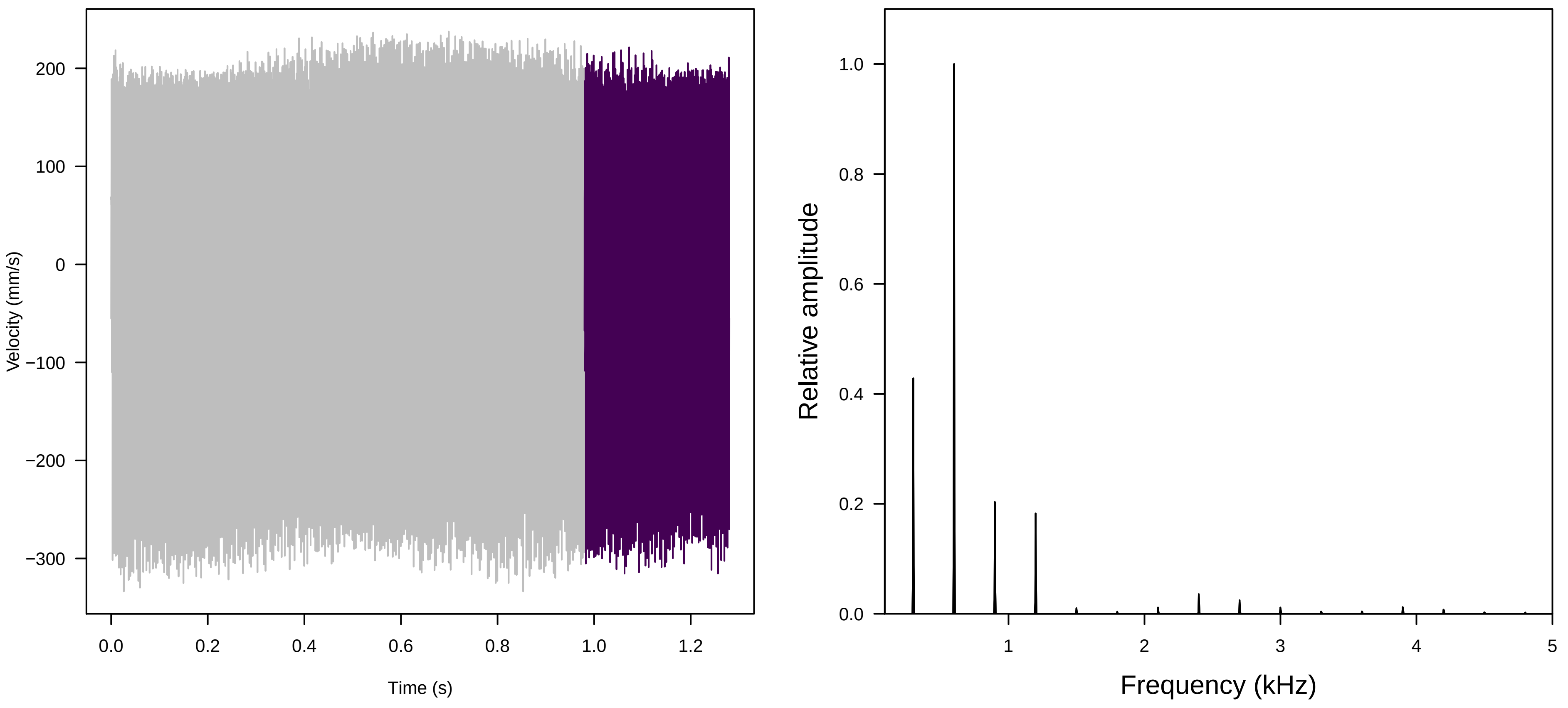

Vel. = 0.057 ; Str. = Receptacle ; Axis = y ; Fl. accession = 10−s−86

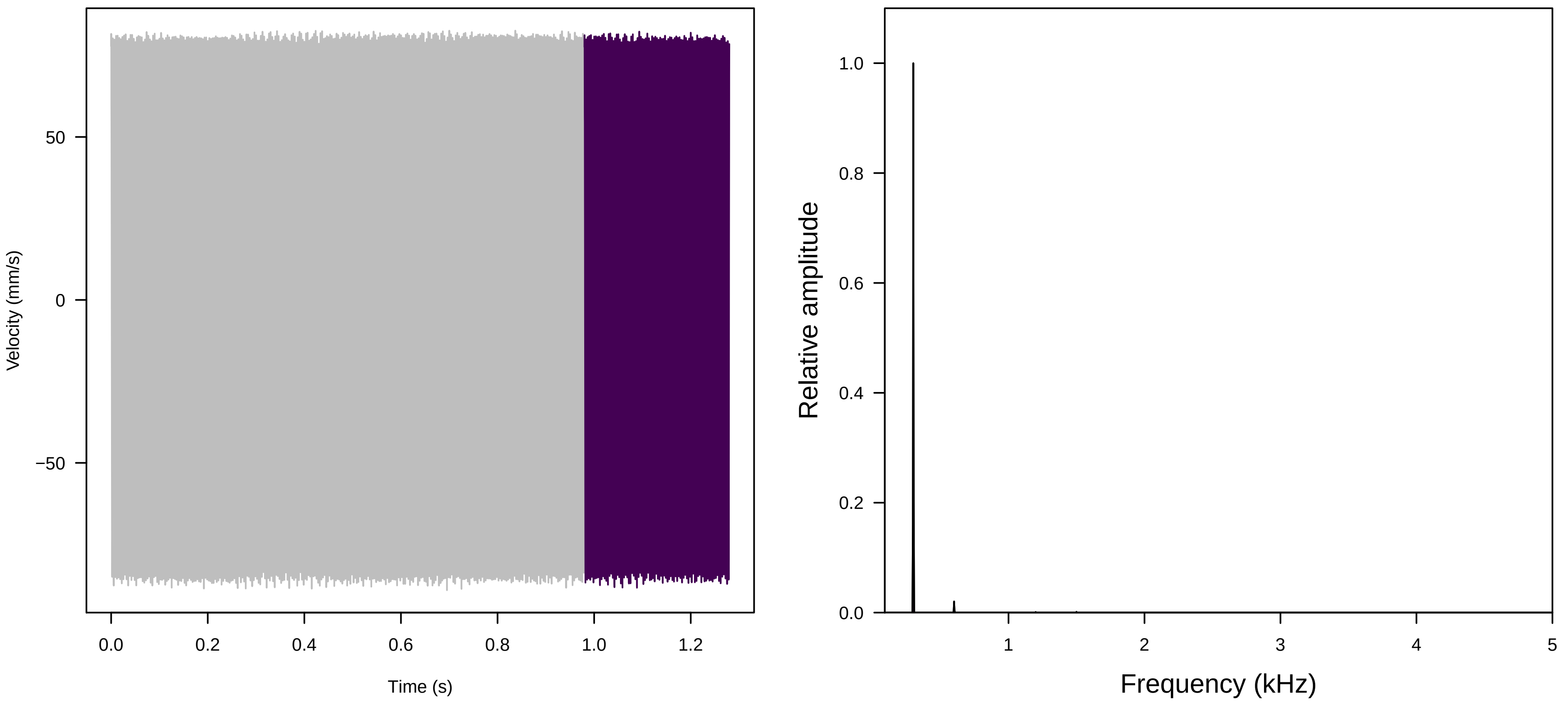

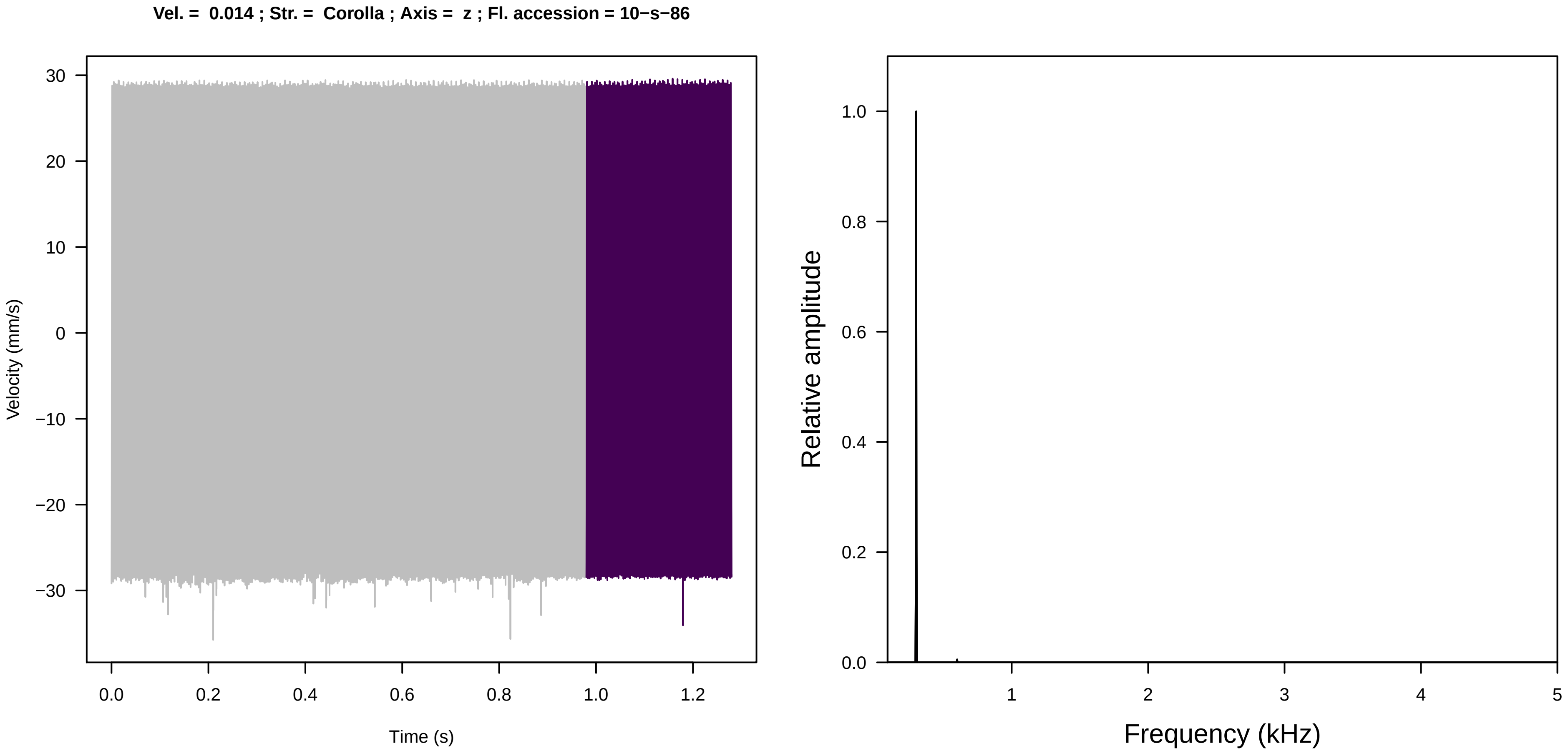
Vel. = 0.014 ; Str. = Corolla ; Axis = z ; Fl. accession = 10−s−86
Velocity (mm/s)
30
20
10
0
−10
−20
−30
Time (s)
0.0
0.2
0.4
0.6
0.8
1.0
1.2
Relative amplitude
1.0
0.8
0.6
0.4
0.2
0.0
Frequency (kHz)
1
2
3
4
5

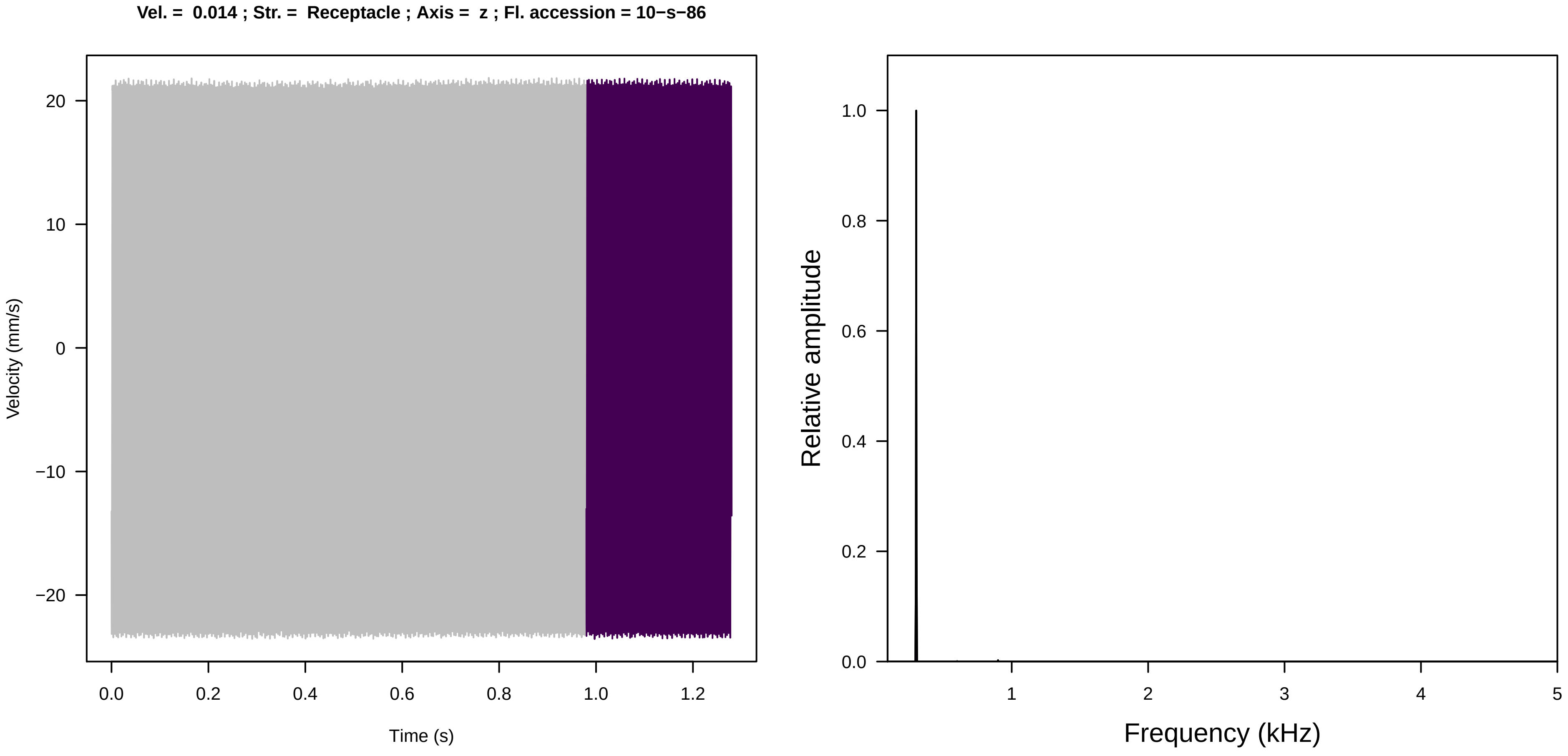
Vel. = 0.014 ; Str. = Receptacle ; Axis = z ; Fl. accession = 10−s−86
Velocity (mm/s)
20
10
0
−10
−20
Time (s)
0.0
0.2
0.4
0.6
0.8
1.0
1.2
Relative amplitude
1.0
0.8
0.6
0.4
0.2
0.0
Frequency (kHz)
1
2
3
4
5

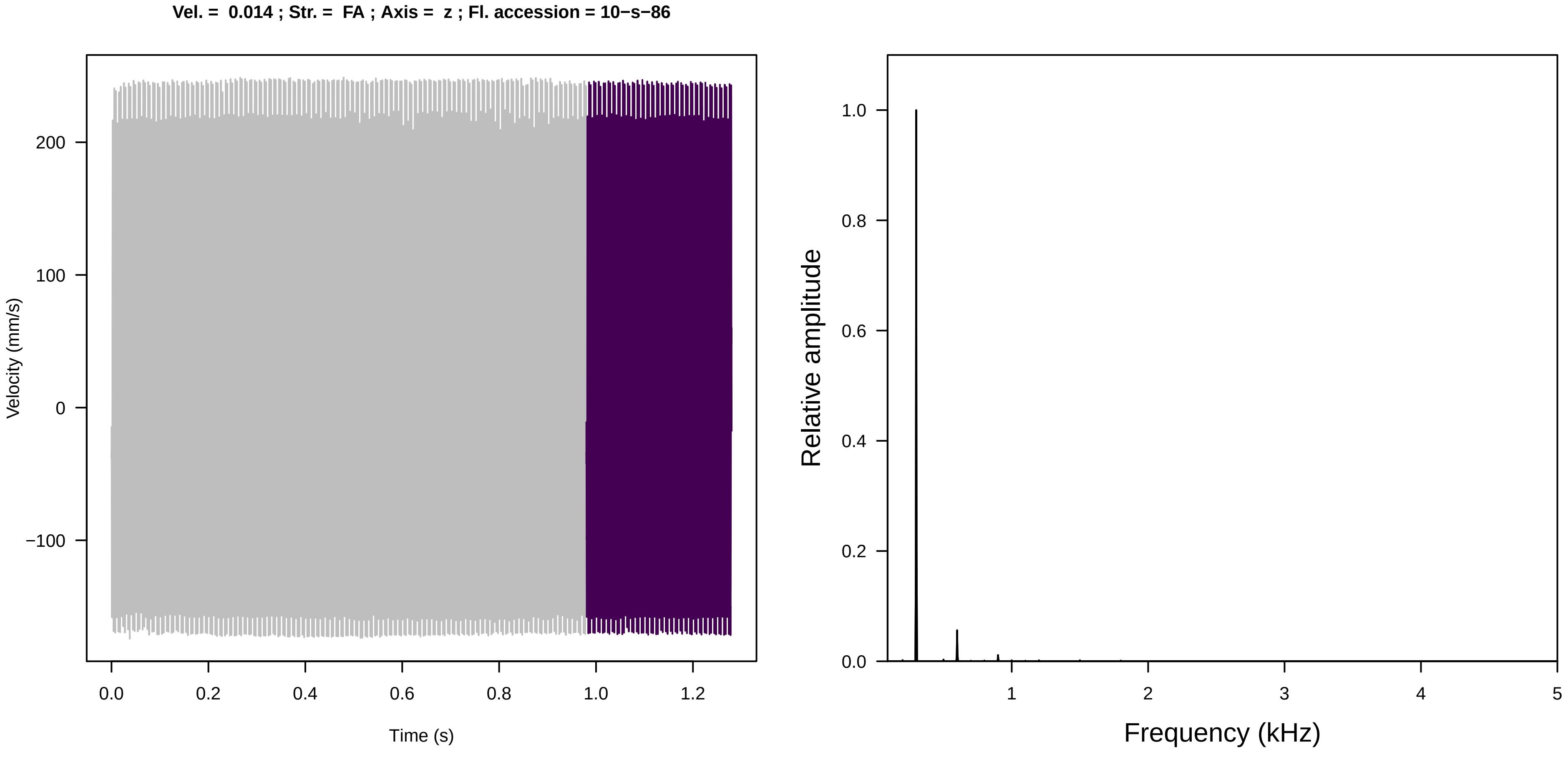
Vel. = 0.014 ; Str. = FA ; Axis = z ; Fl. accession = 10−s−86
Velocity (mm/s)
200
100
0
−100
Time (s)
0.0
0.2
0.4
0.6
0.8
1.0
1.2
Relative amplitude
1.0
0.8
0.6
0.4
0.2
0.0
Frequency (kHz)
1
2
3
4
5

Vel. = 0.014 ; Str. = Receptacle ; Axis = z ; Fl. accession = 10−s−86

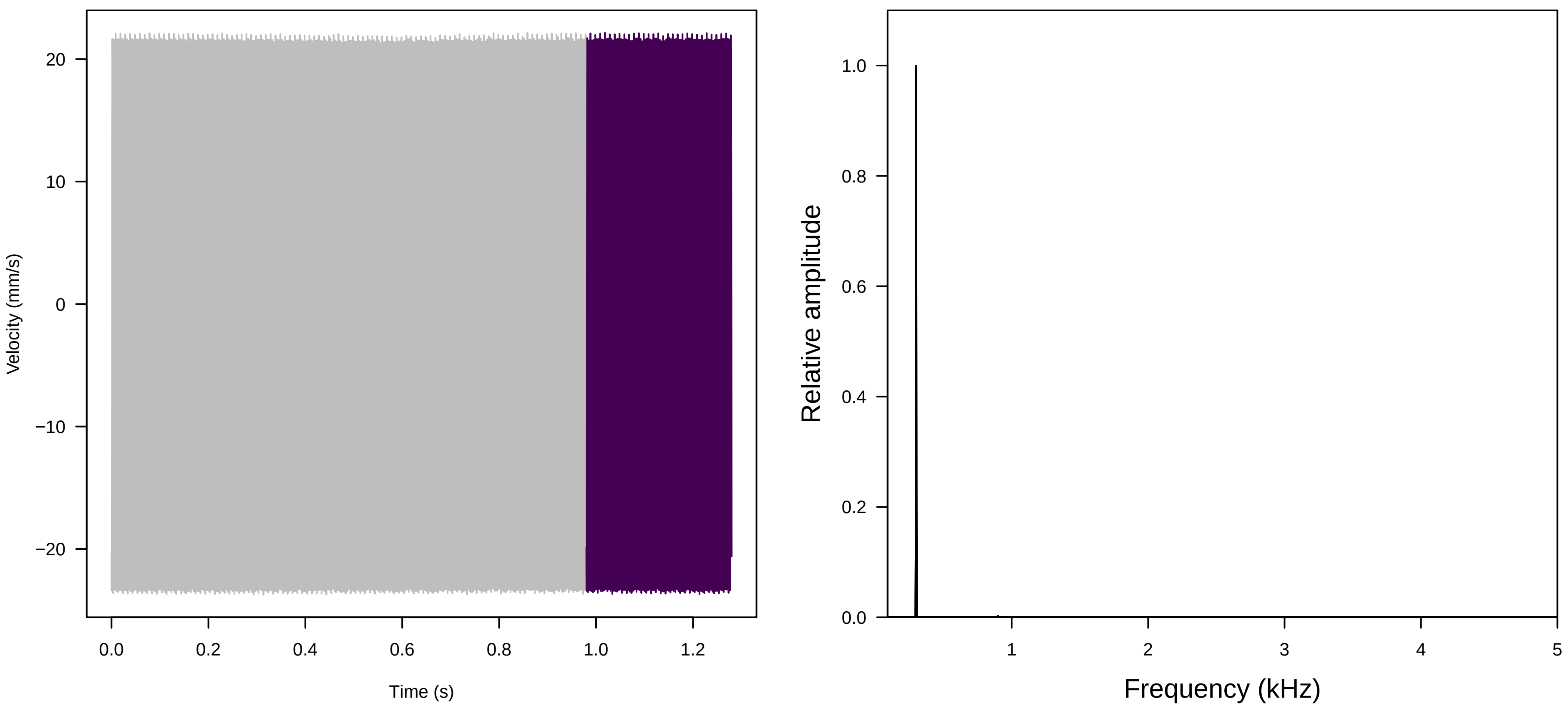

Vel. = 0.014 ; Str. = PA ; Axis = z ; Fl. accession = 10−s−86

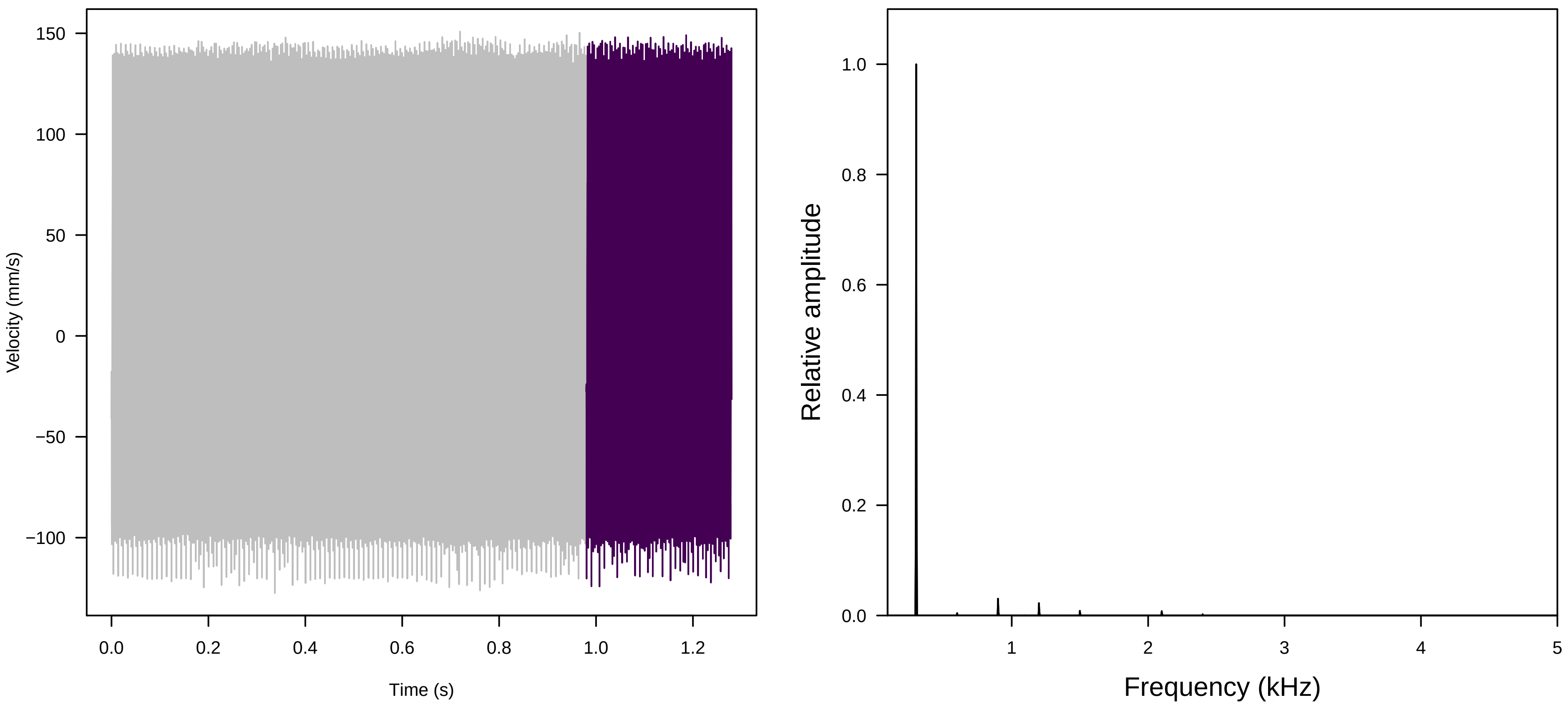

Vel. = 0.014 ; Str. = Receptacle ; Axis = z ; Fl. accession = 10−s−86

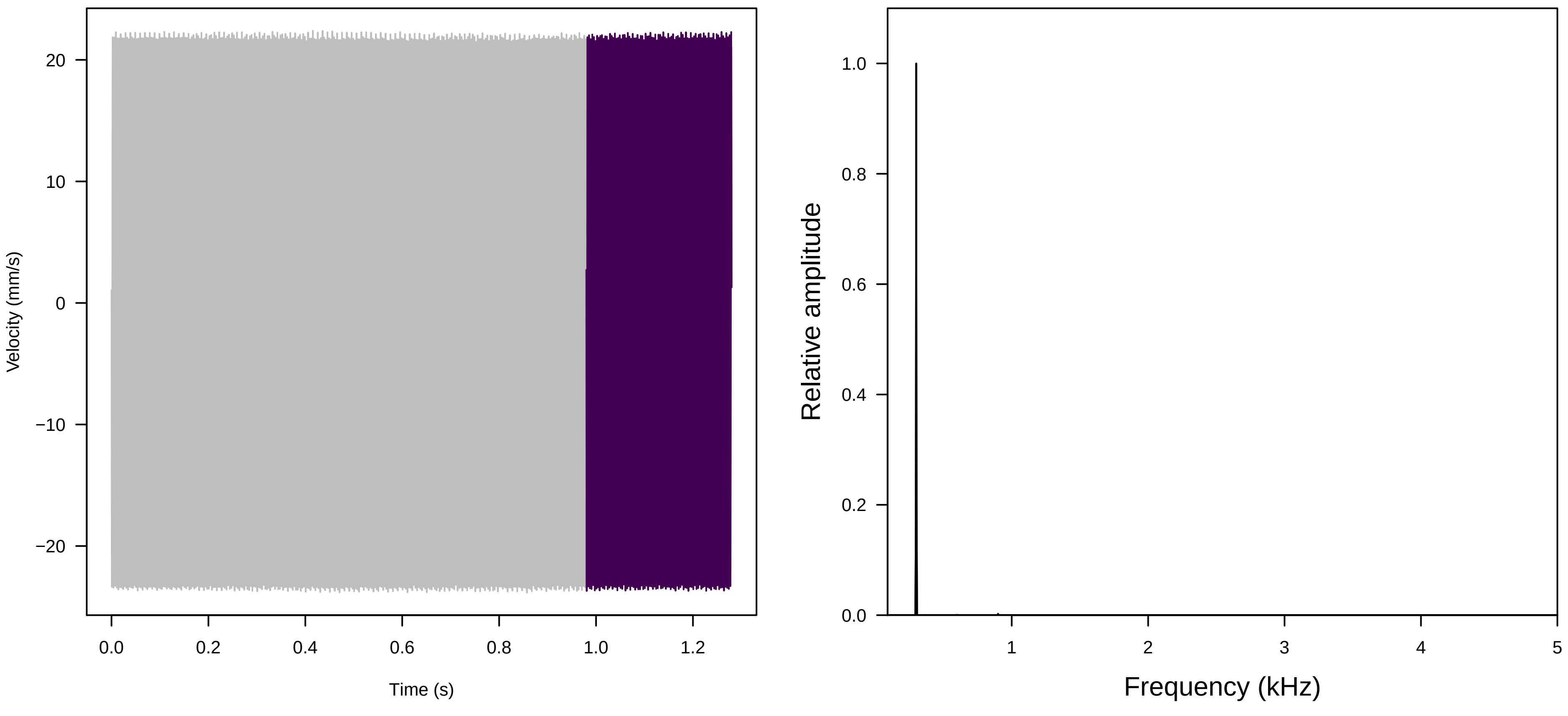

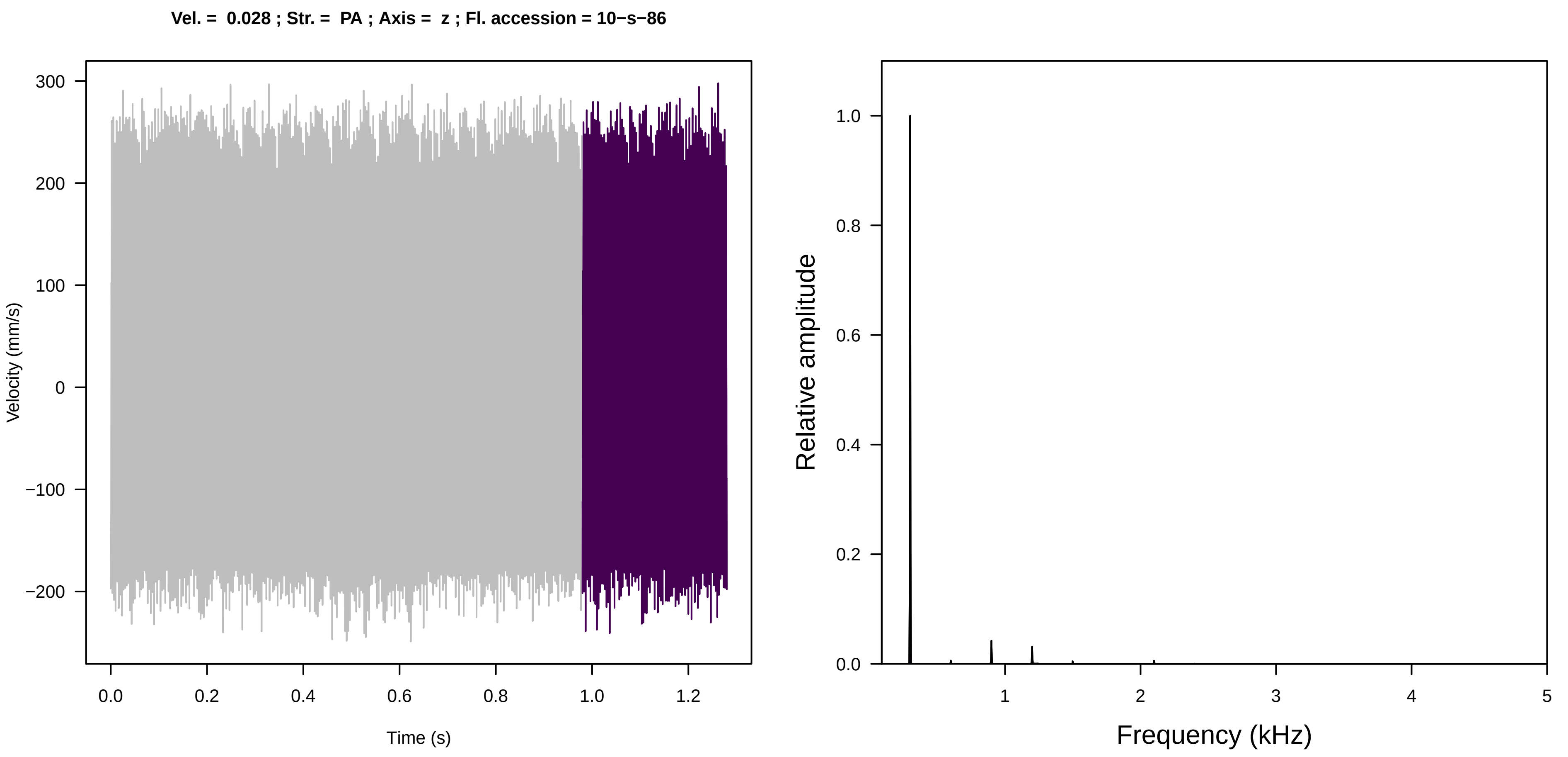
Vel. = 0.028 ; Str. = PA ; Axis = z ; Fl. accession = 10−s−86
Velocity (mm/s)
Time (s)
Relative amplitude
Frequency (kHz)

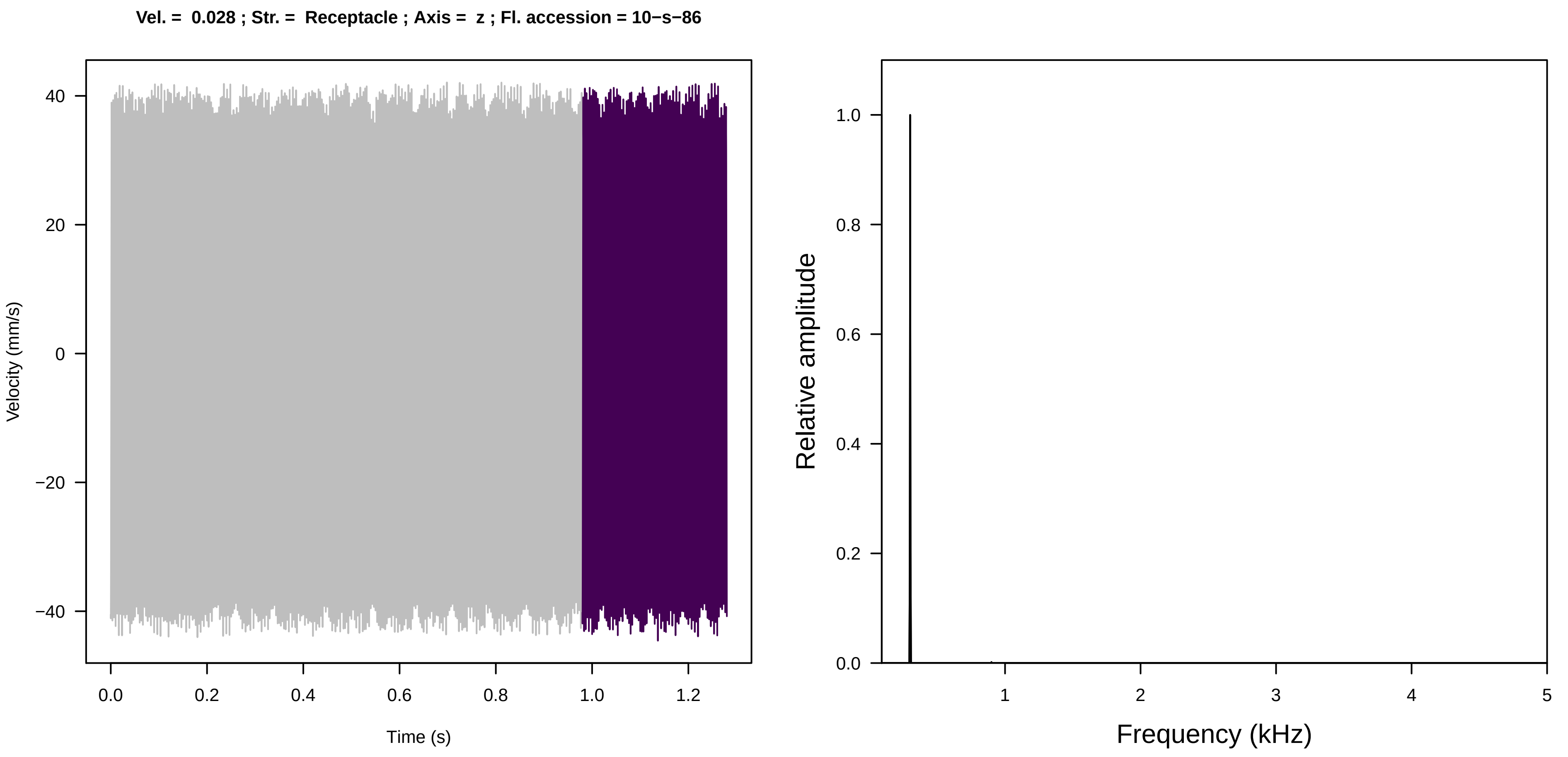
Vel. = 0.028 ; Str. = Receptacle ; Axis = z ; Fl. accession = 10−s−86
Velocity (mm/s)
Time (s)
Relative amplitude
Frequency (kHz)

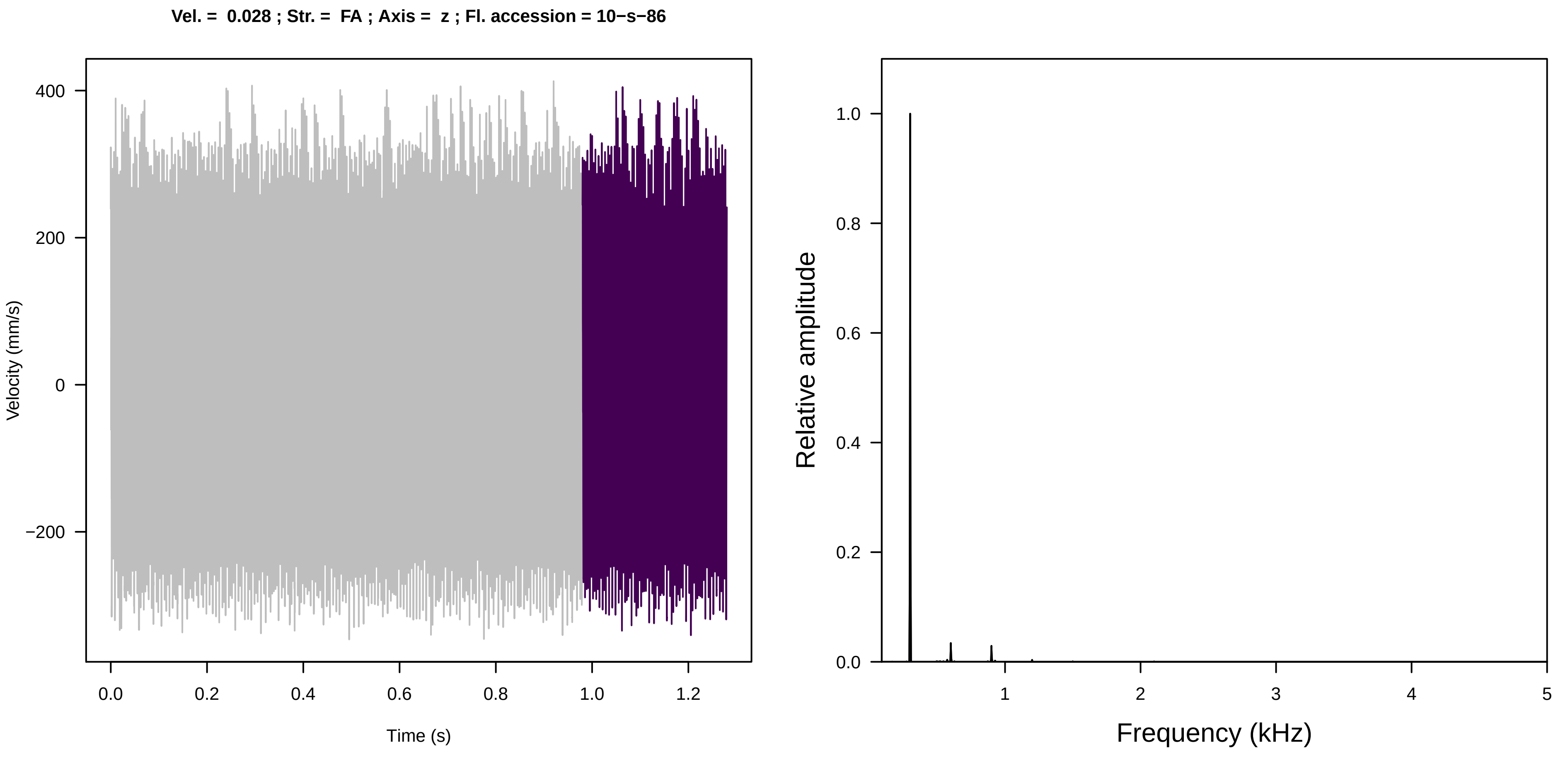
Vel. = 0.028 ; Str. = FA ; Axis = z ; Fl. accession = 10−s−86
Velocity (mm/s)
Time (s)
Relative amplitude
Frequency (kHz)

Vel. = 0.028 ; Str. = Receptacle ; Axis = z ; Fl. accession = 10−s−86

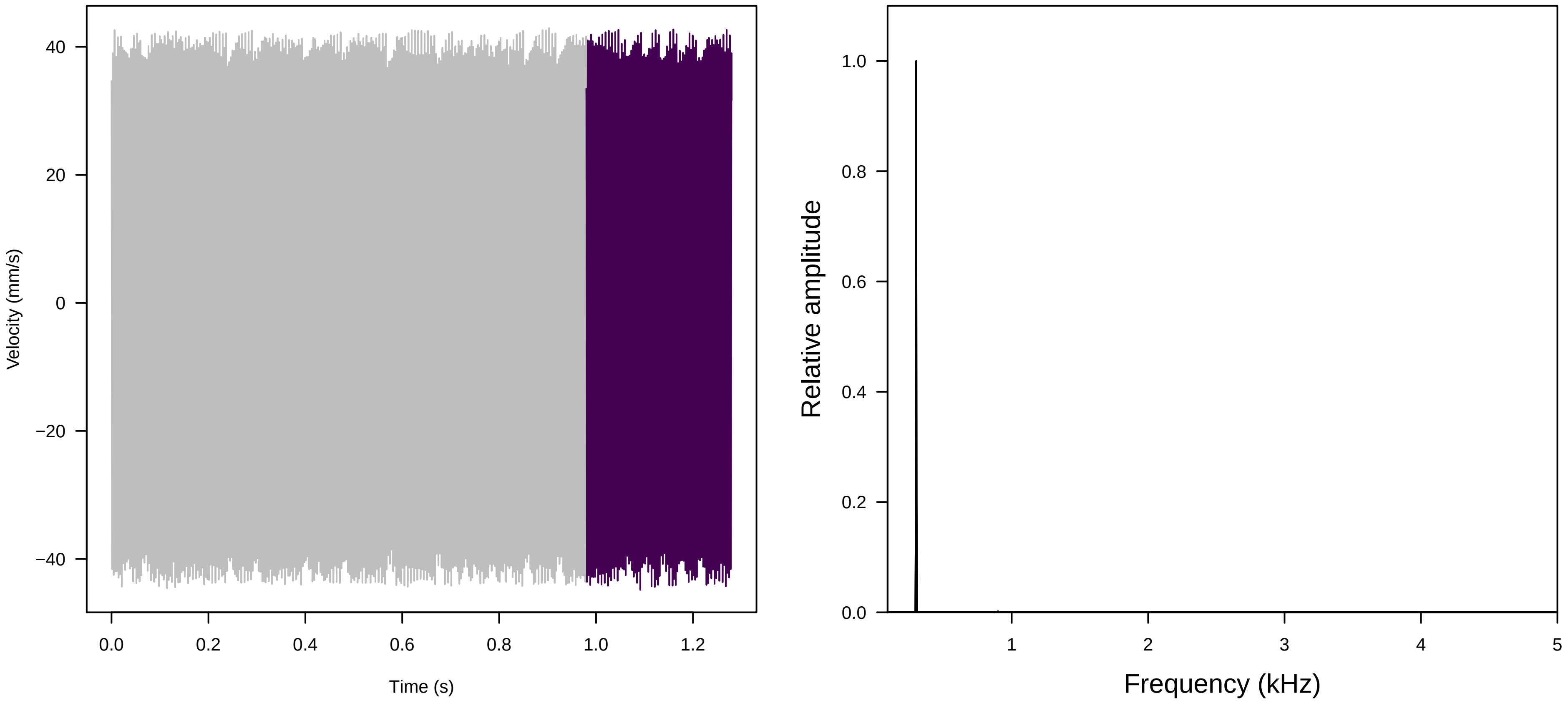

Vel. = 0.028 ; Str. = Corolla ; Axis = z ; Fl. accession = 10−s−86

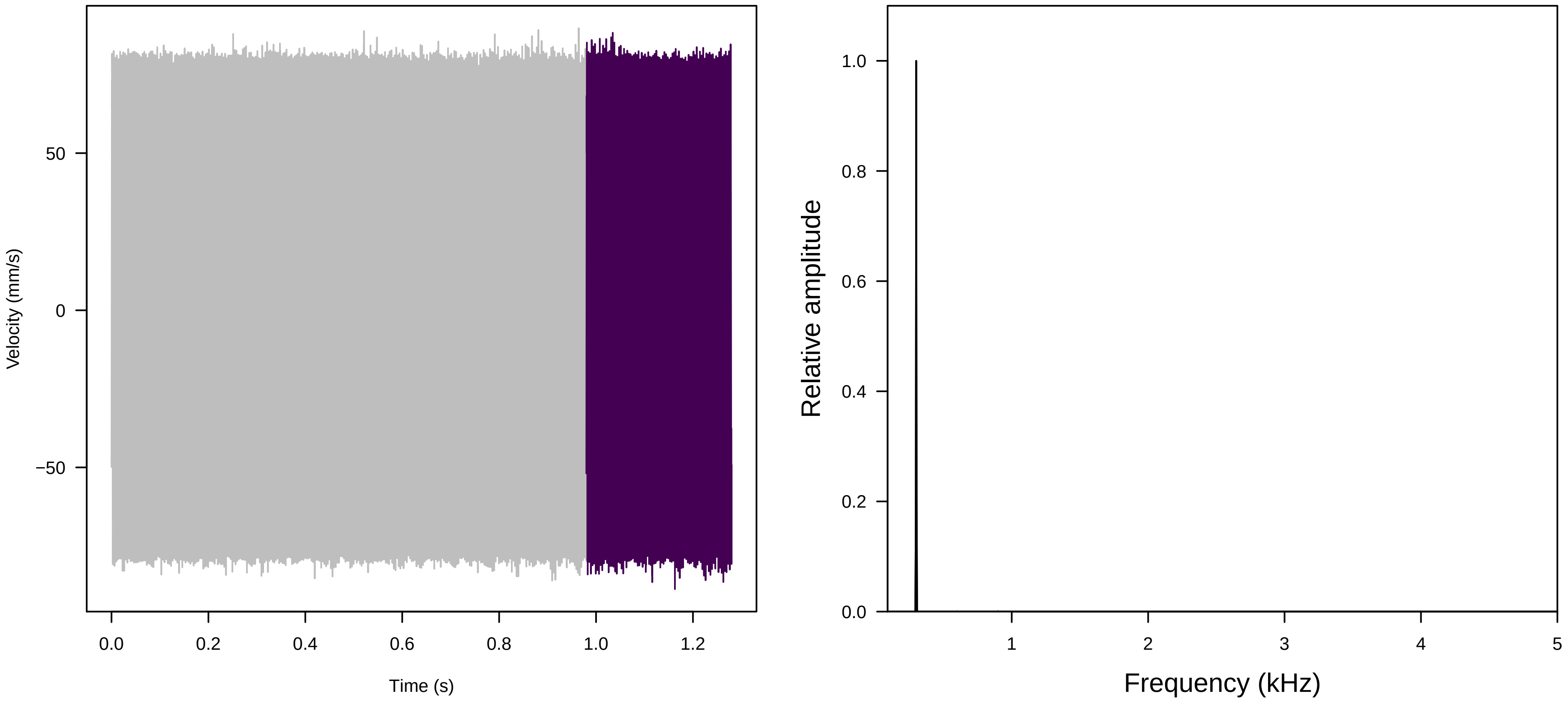

Vel. = 0.028 ; Str. = Receptacle ; Axis = z ; Fl. accession = 10−s−86

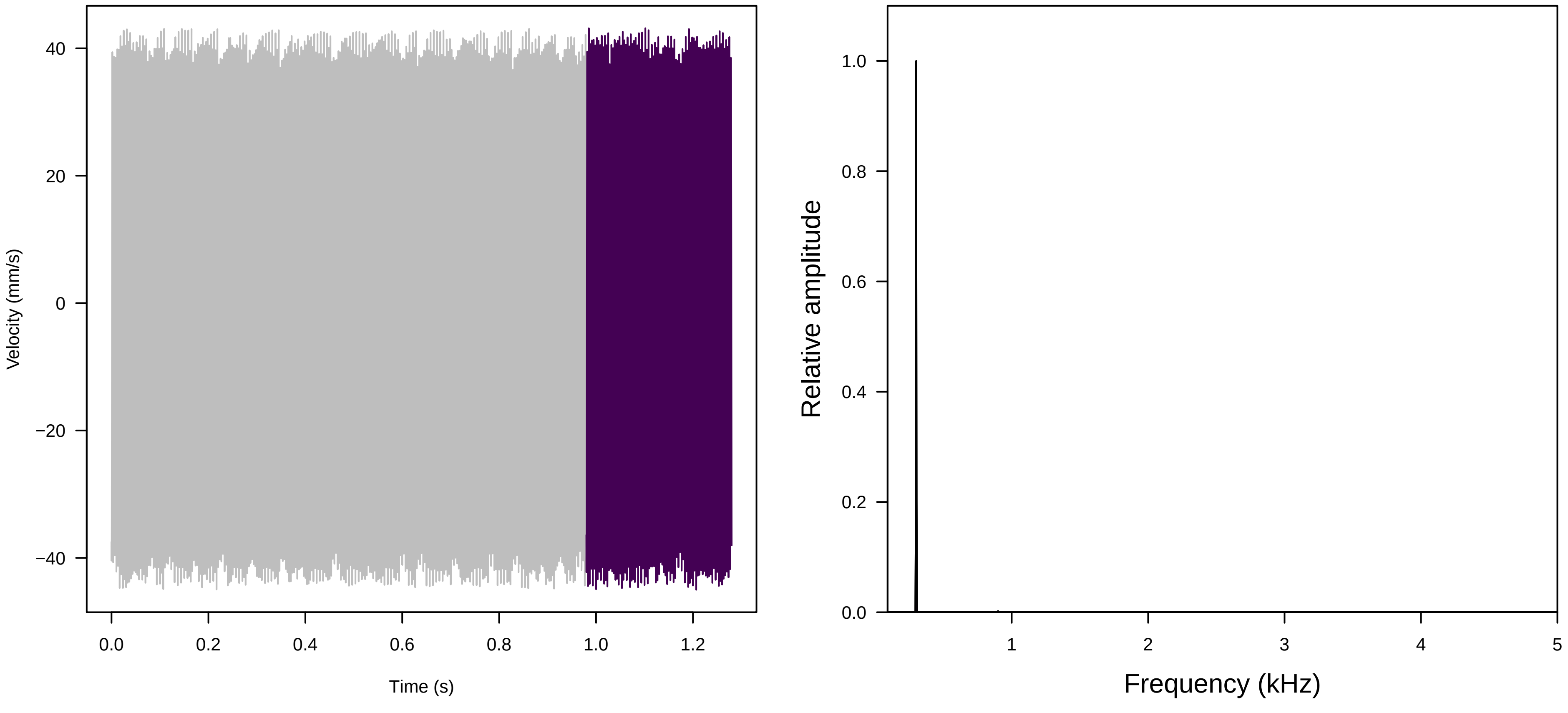

Vel. = 0.057 ; Str. = Corolla ; Axis = z ; Fl. accession = 10−s−86

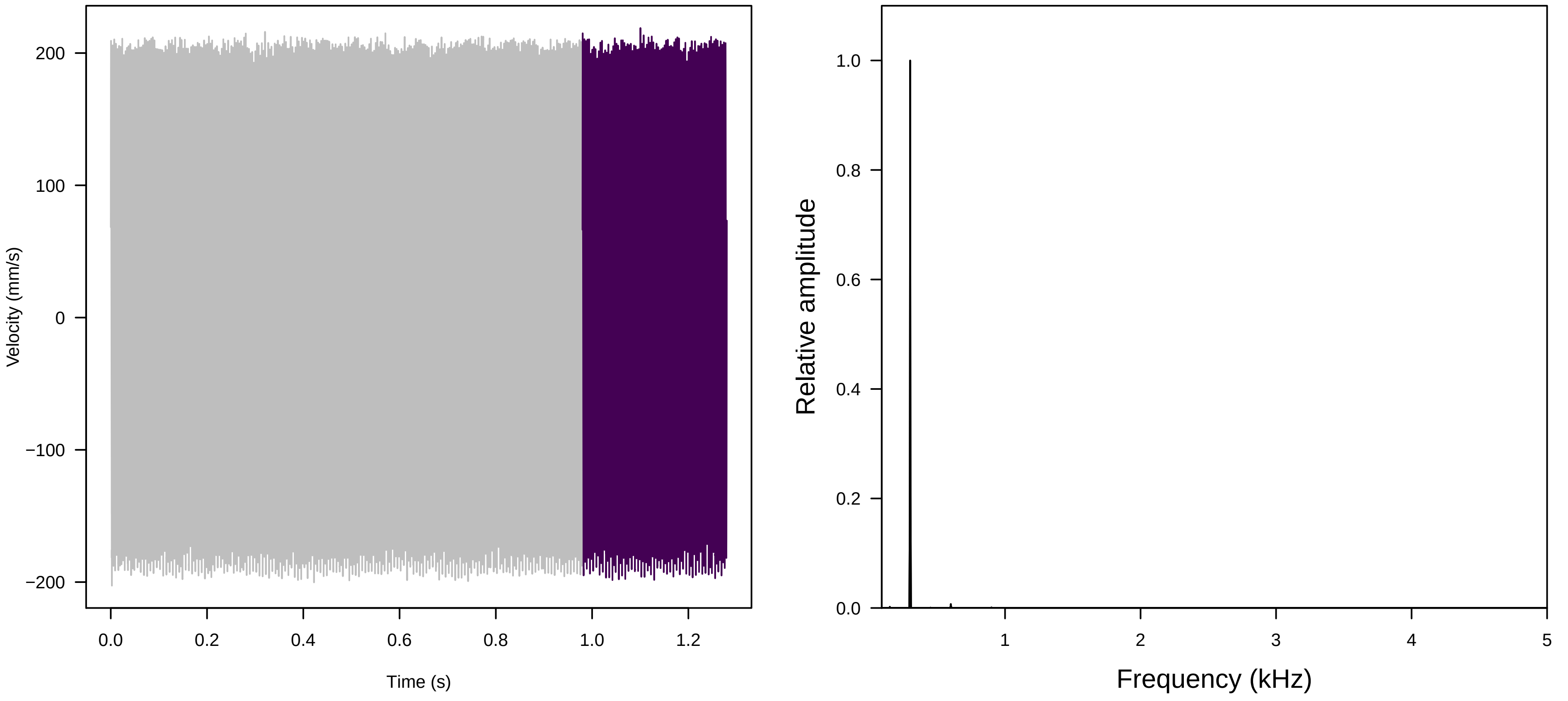

Vel. = 0.057 ; Str. = Receptacle ; Axis = z ; Fl. accession = 10−s−86

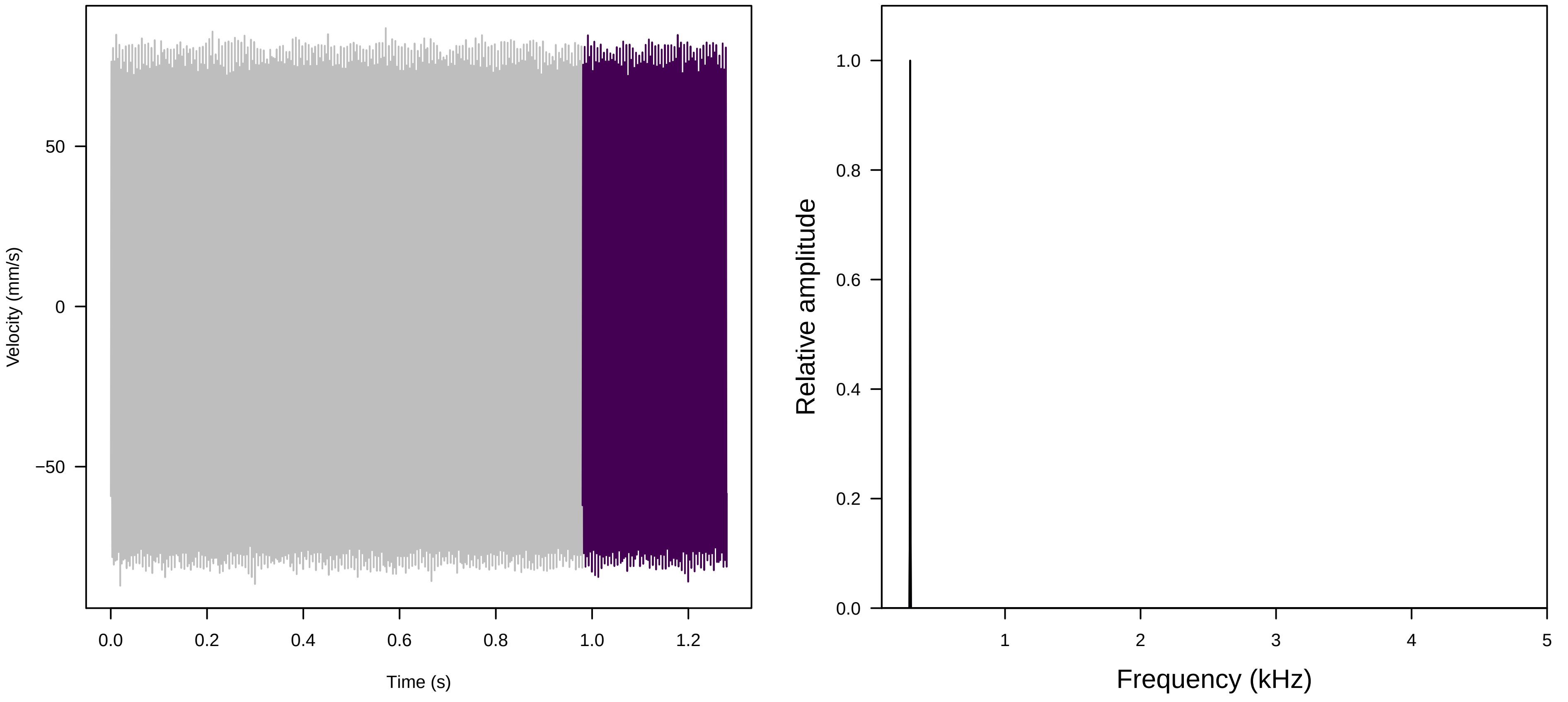

Vel. = 0.057 ; Str. = FA ; Axis = z ; Fl. accession = 10−s−86

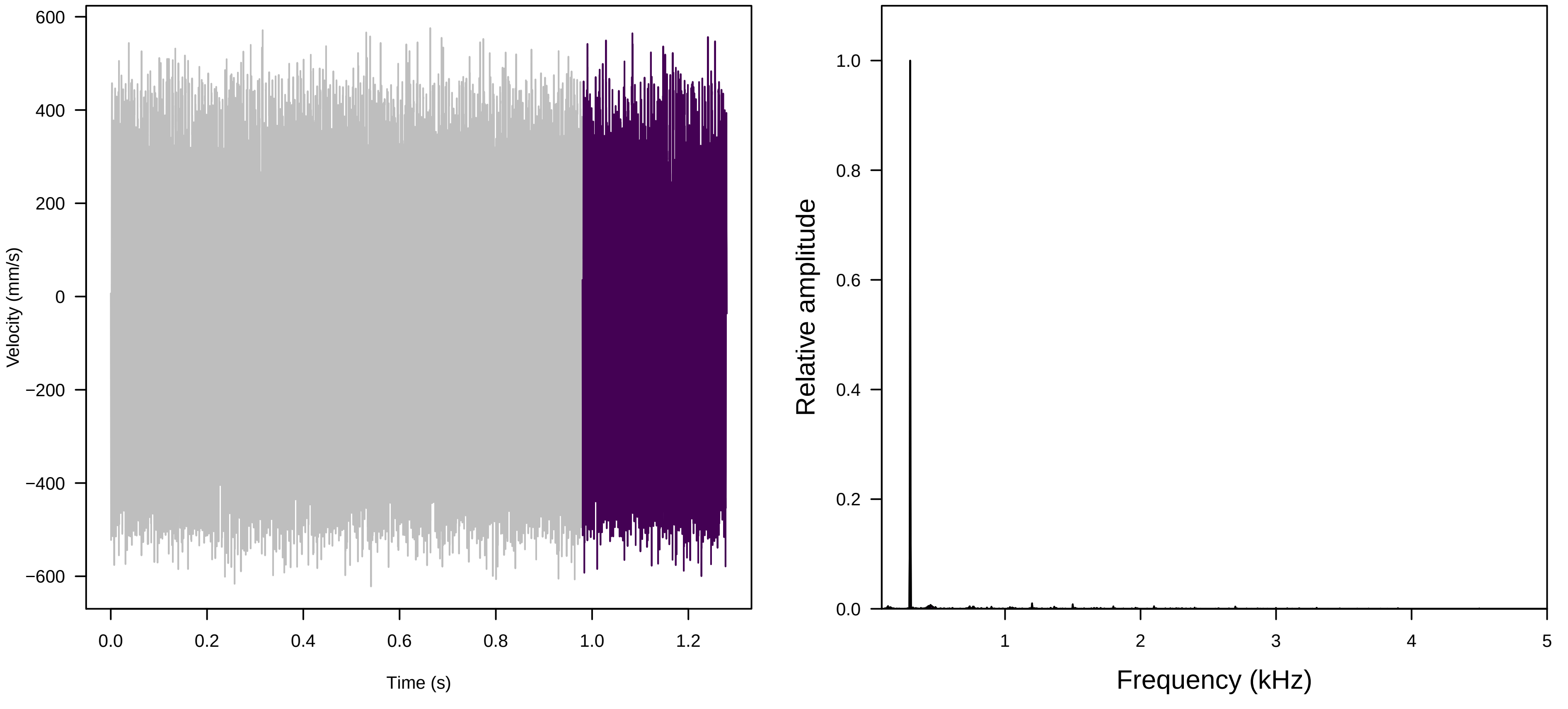

Vel. = 0.057 ; Str. = Receptacle ; Axis = z ; Fl. accession = 10−s−86

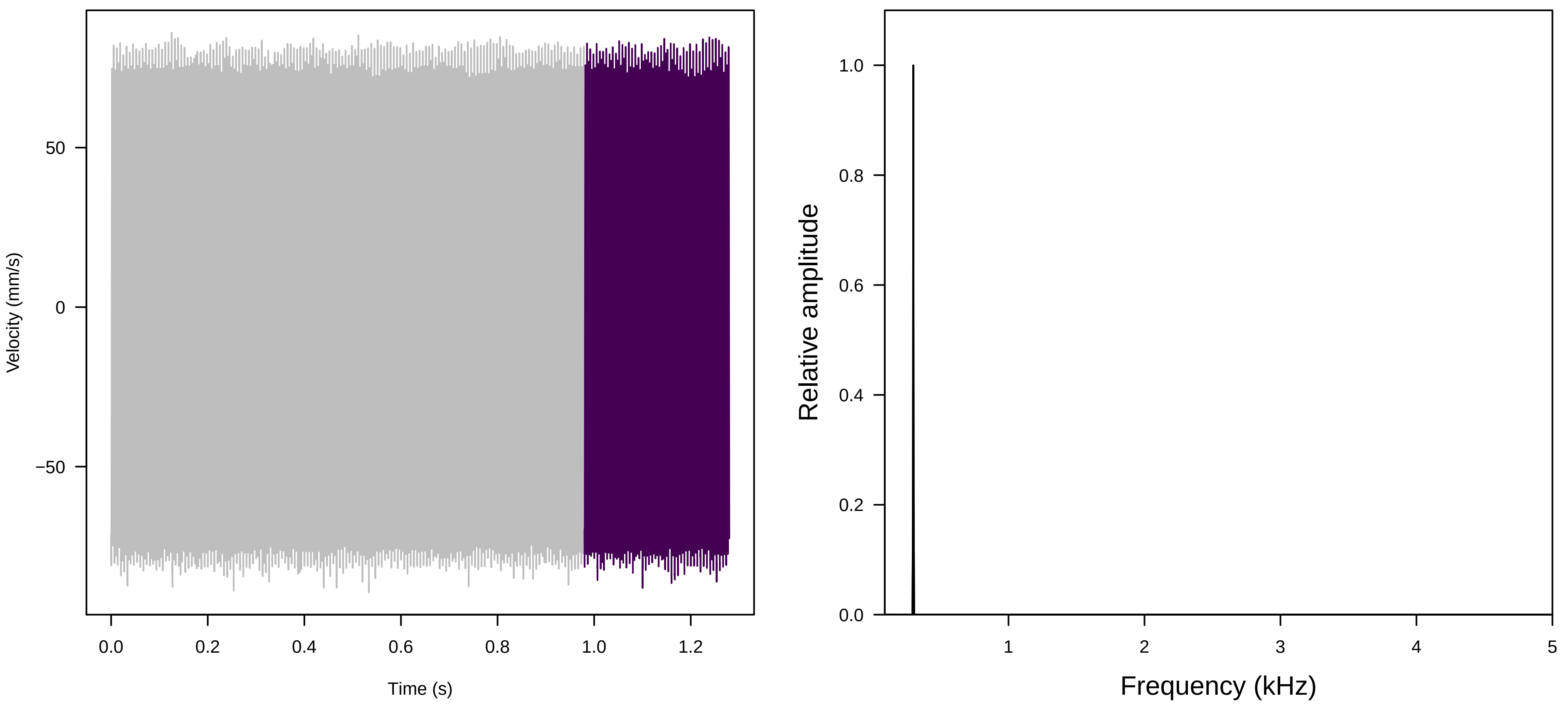

Vel. = 0.057 ; Str. = PA ; Axis = z ; Fl. accession = 10−s−86

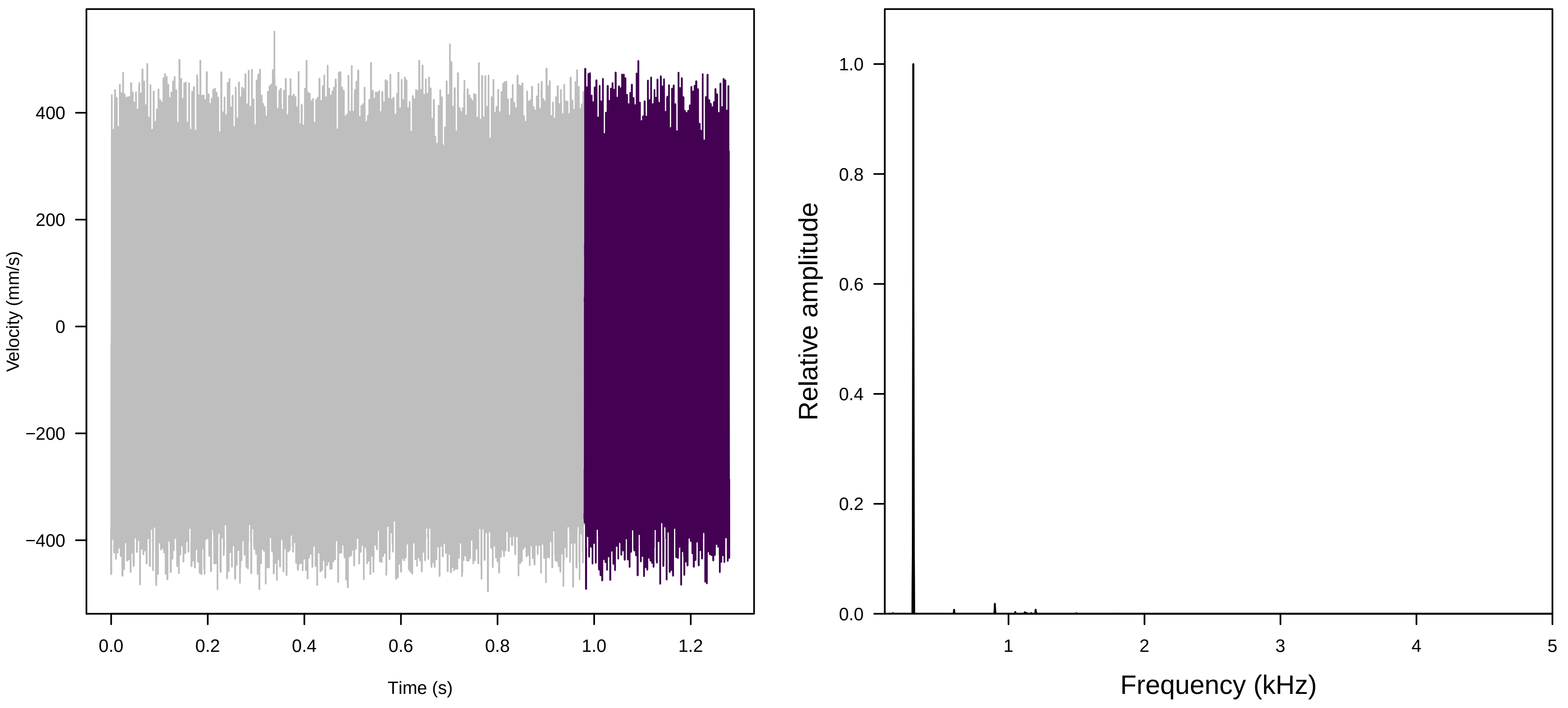

Vel. = 0.057 ; Str. = Receptacle ; Axis = z ; Fl. accession = 10−s−86

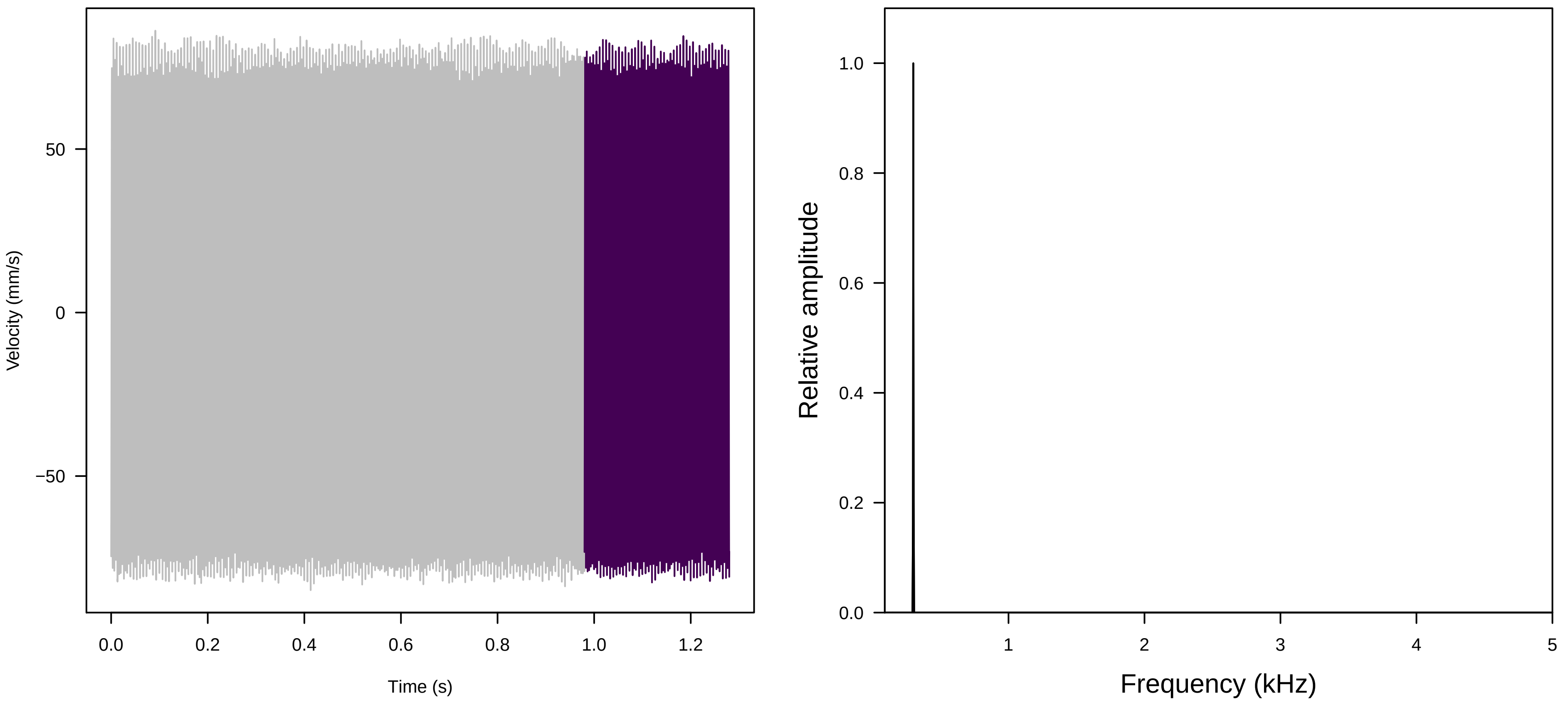

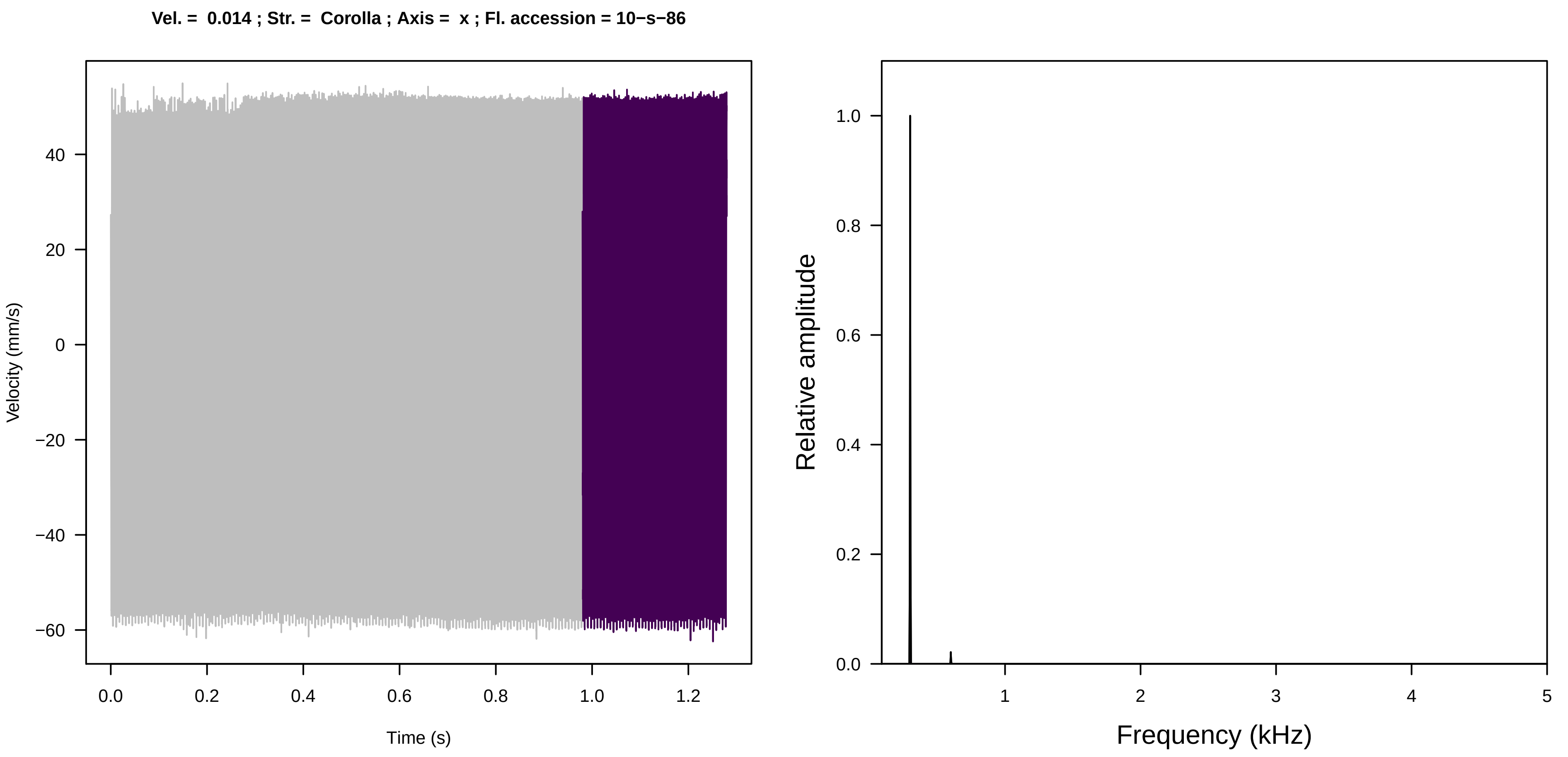
Vel. = 0.014 ; Str. = Corolla ; Axis = x ; Fl. accession = 10−s−86
Velocity (mm/s)
Time (s)
Relative amplitude
Frequency (kHz)

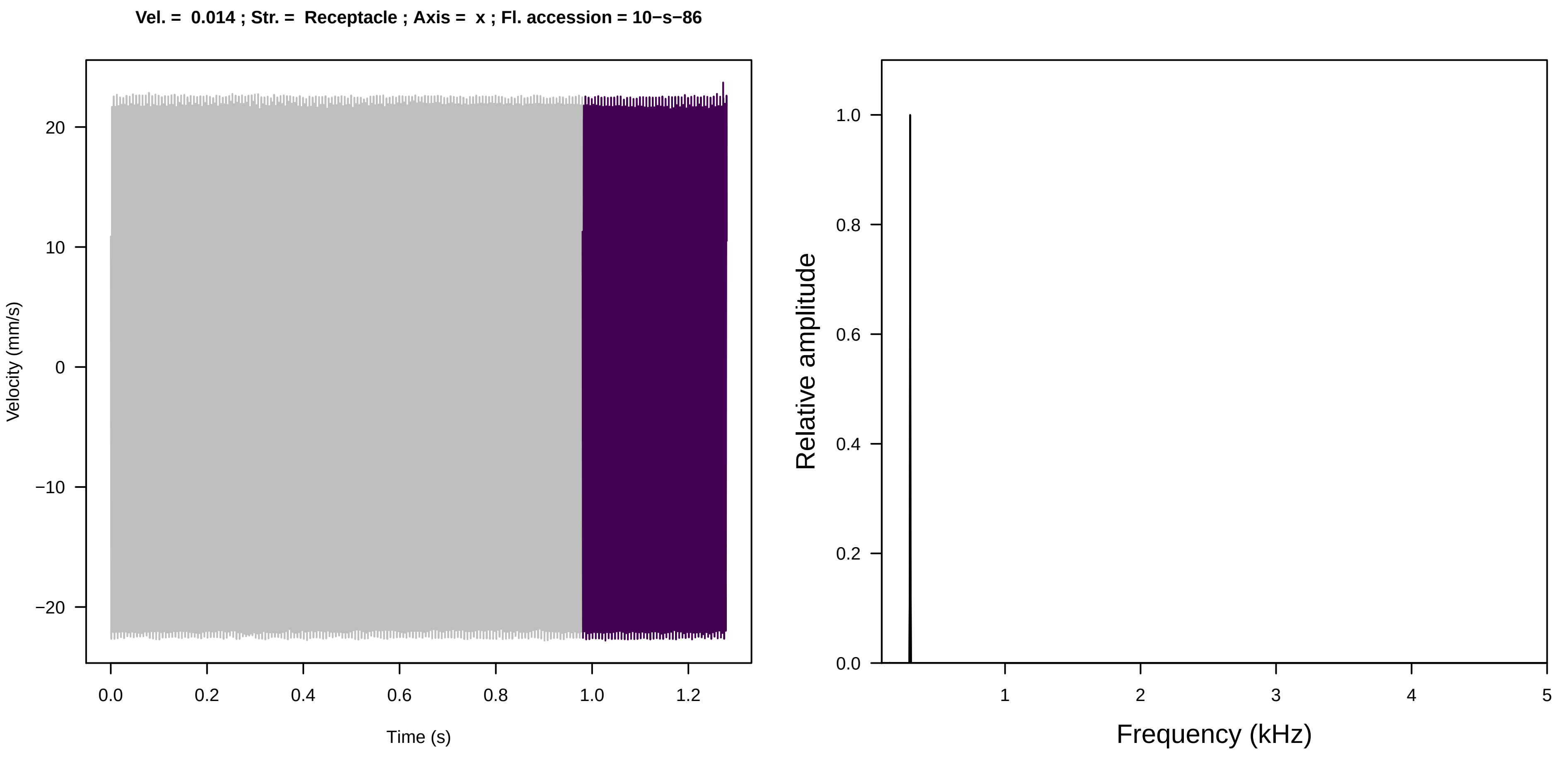
Vel. = 0.014 ; Str. = Receptacle ; Axis = x ; Fl. accession = 10−s−86
Velocity (mm/s)
Time (s)
Relative amplitude
Frequency (kHz)

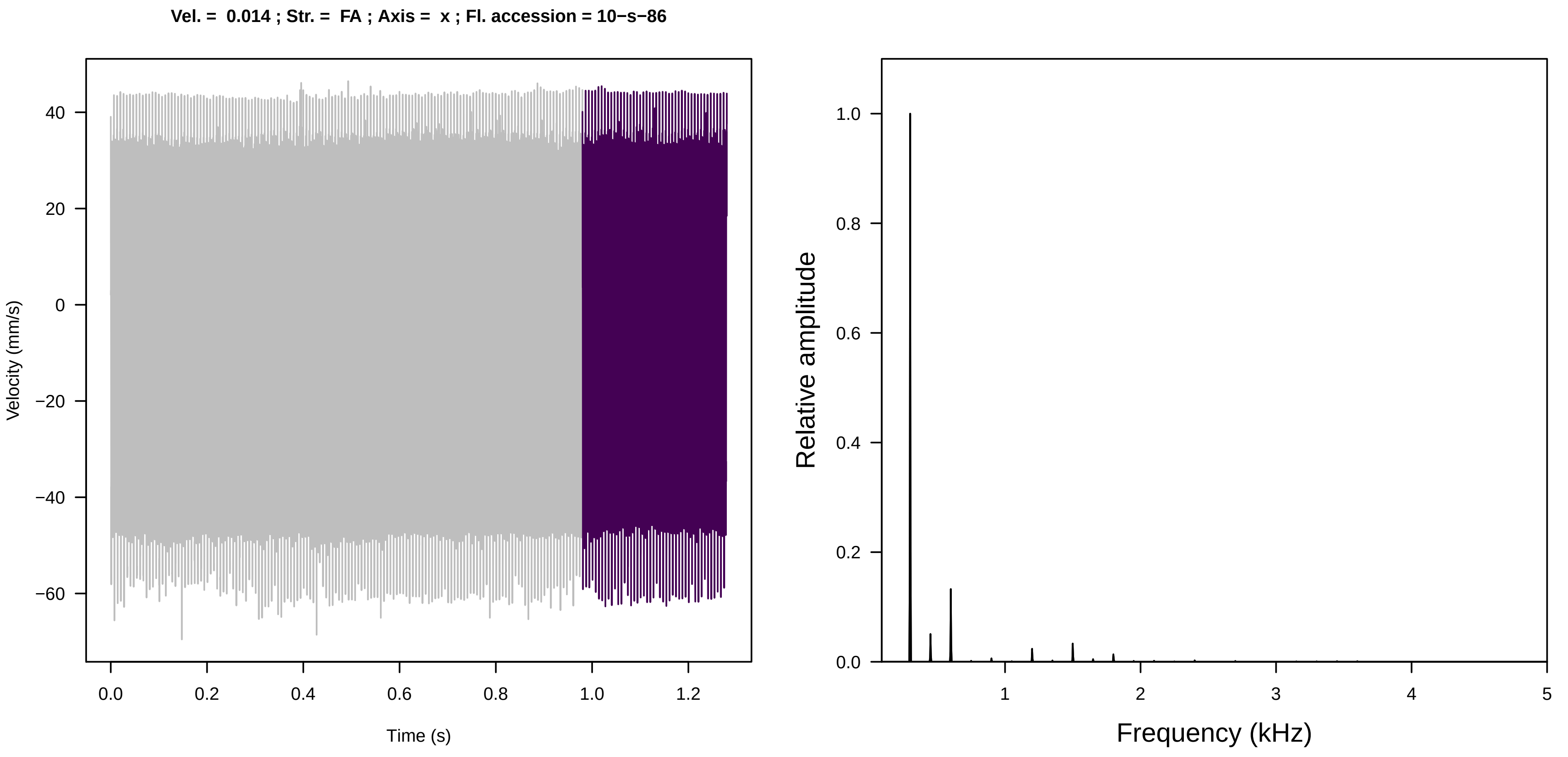
Vel. = 0.014 ; Str. = FA ; Axis = x ; Fl. accession = 10−s−86
Velocity (mm/s)
Time (s)
Relative amplitude
Frequency (kHz)

Vel. = 0.014 ; Str. = Receptacle ; Axis = x ; Fl. accession = 10−s−86

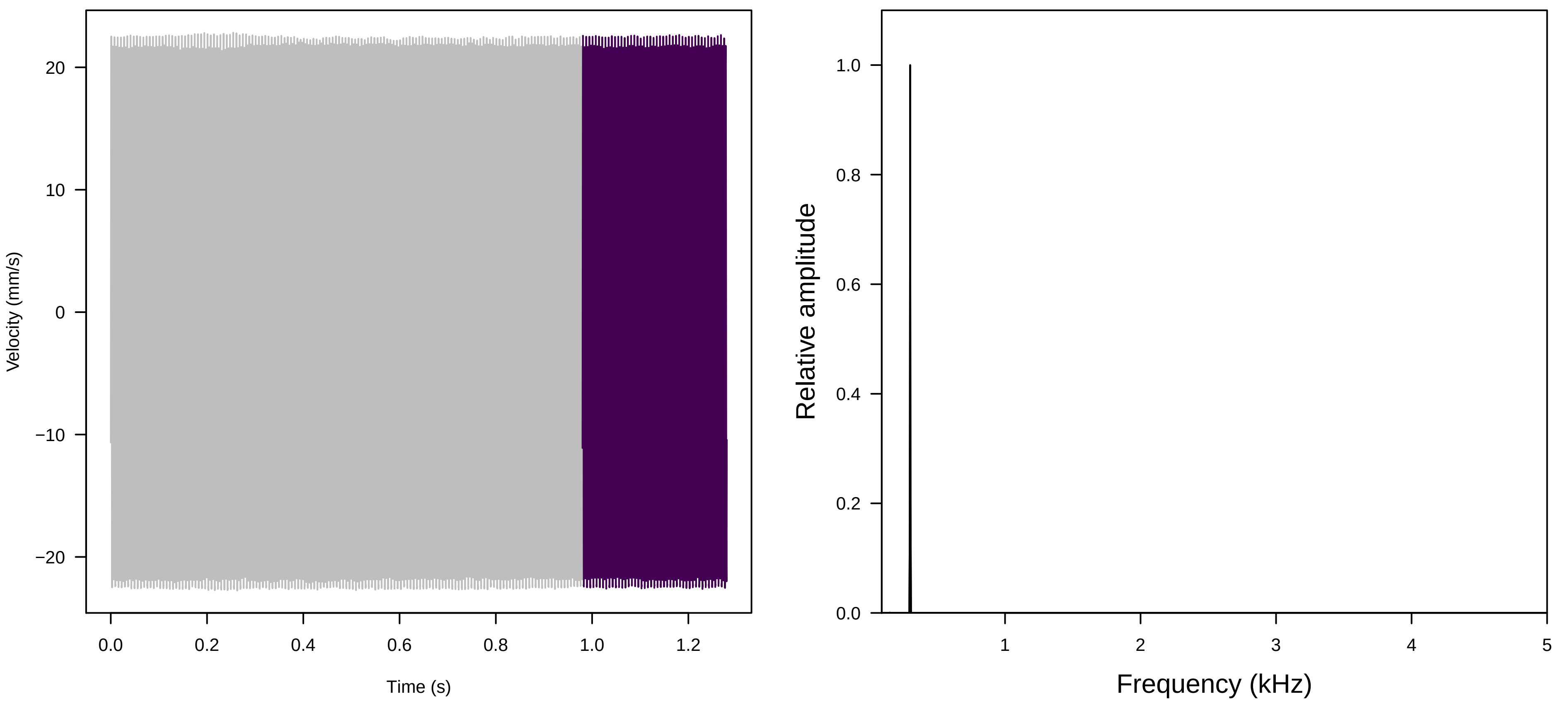

Vel. = 0.014 ; Str. = PA ; Axis = x ; Fl. accession = 10−s−86

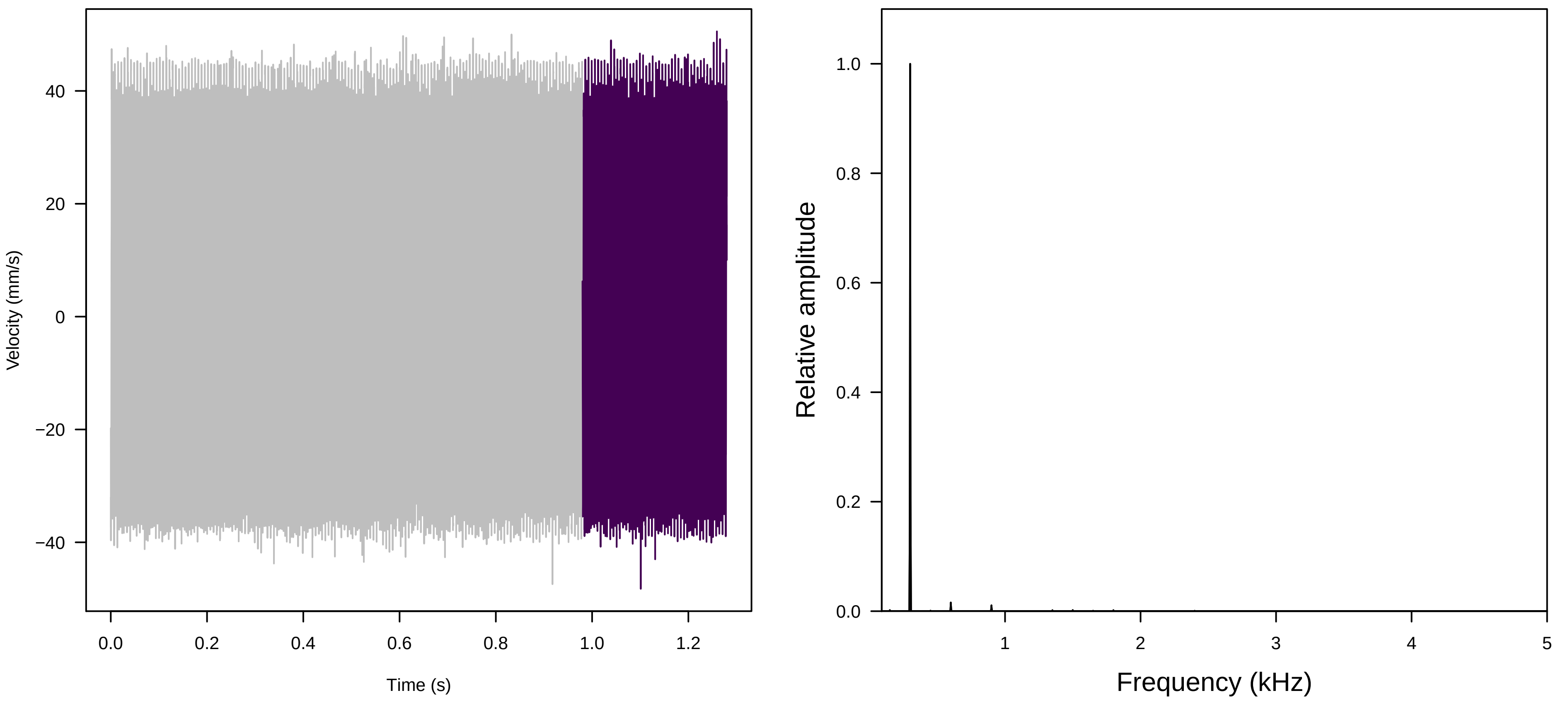

Vel. = 0.014 ; Str. = Receptacle ; Axis = x ; Fl. accession = 10−s−86

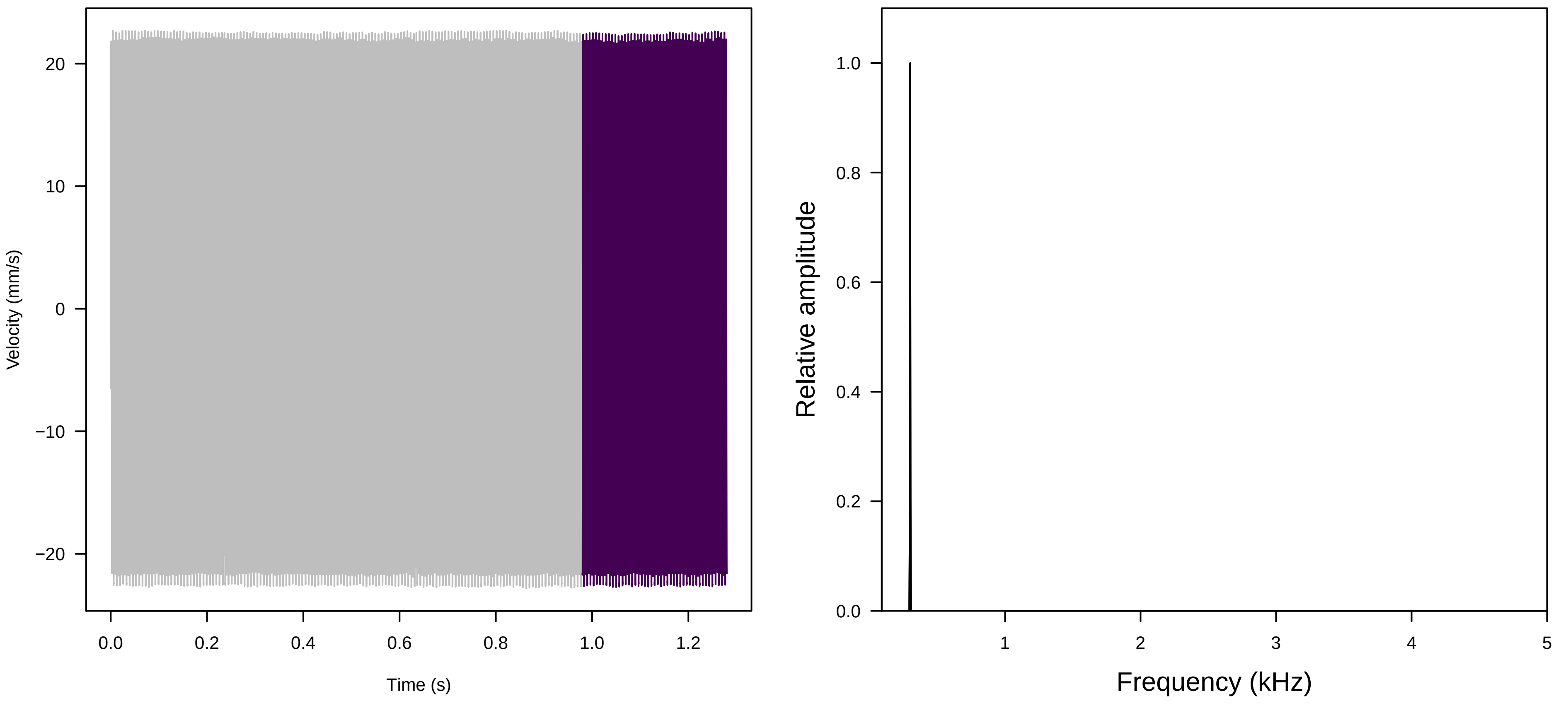

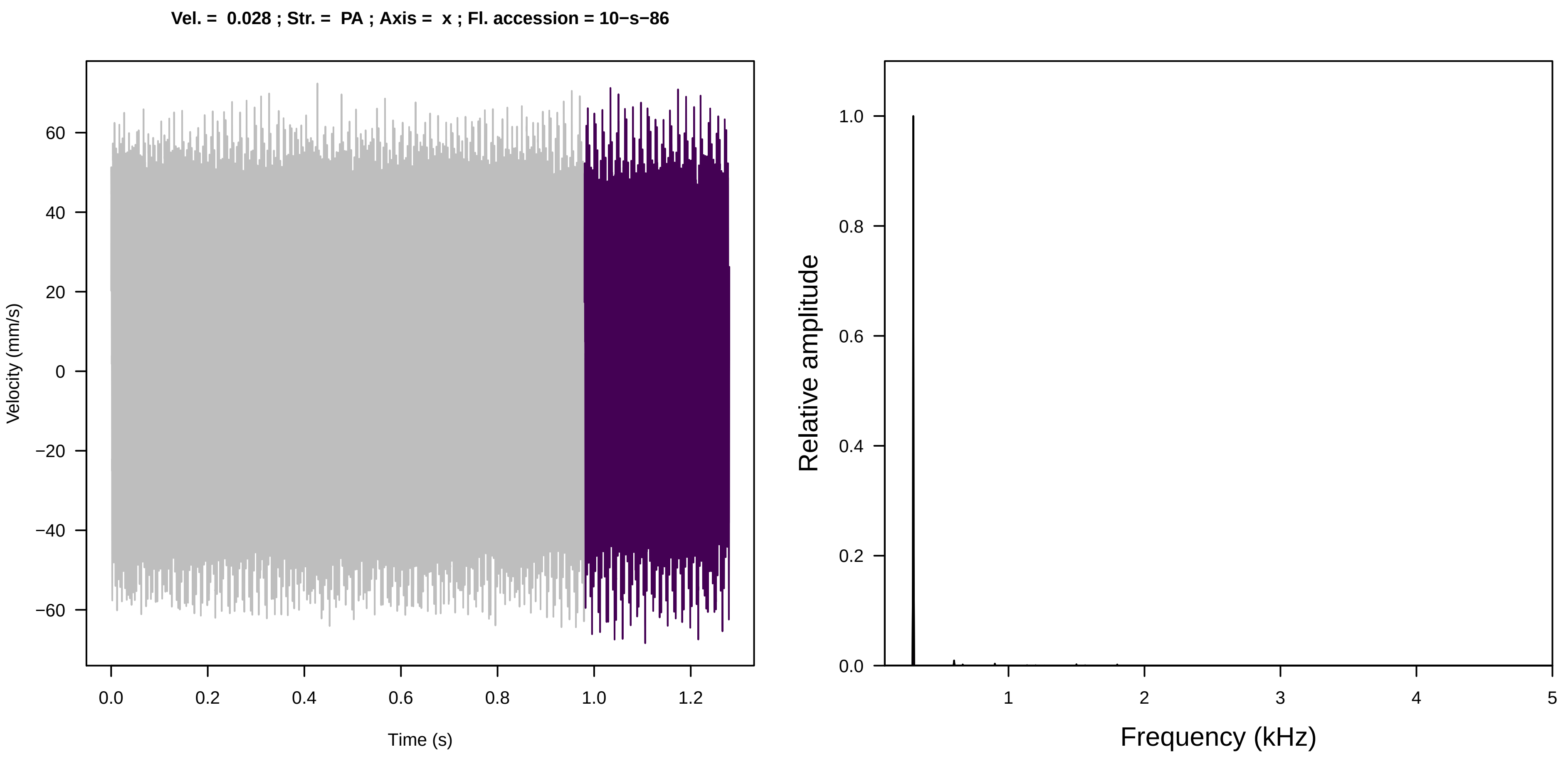
Vel. = 0.028 ; Str. = PA ; Axis = x ; Fl. accession = 10−s−86
Velocity (mm/s)
Time (s)
Relative amplitude
Frequency (kHz)

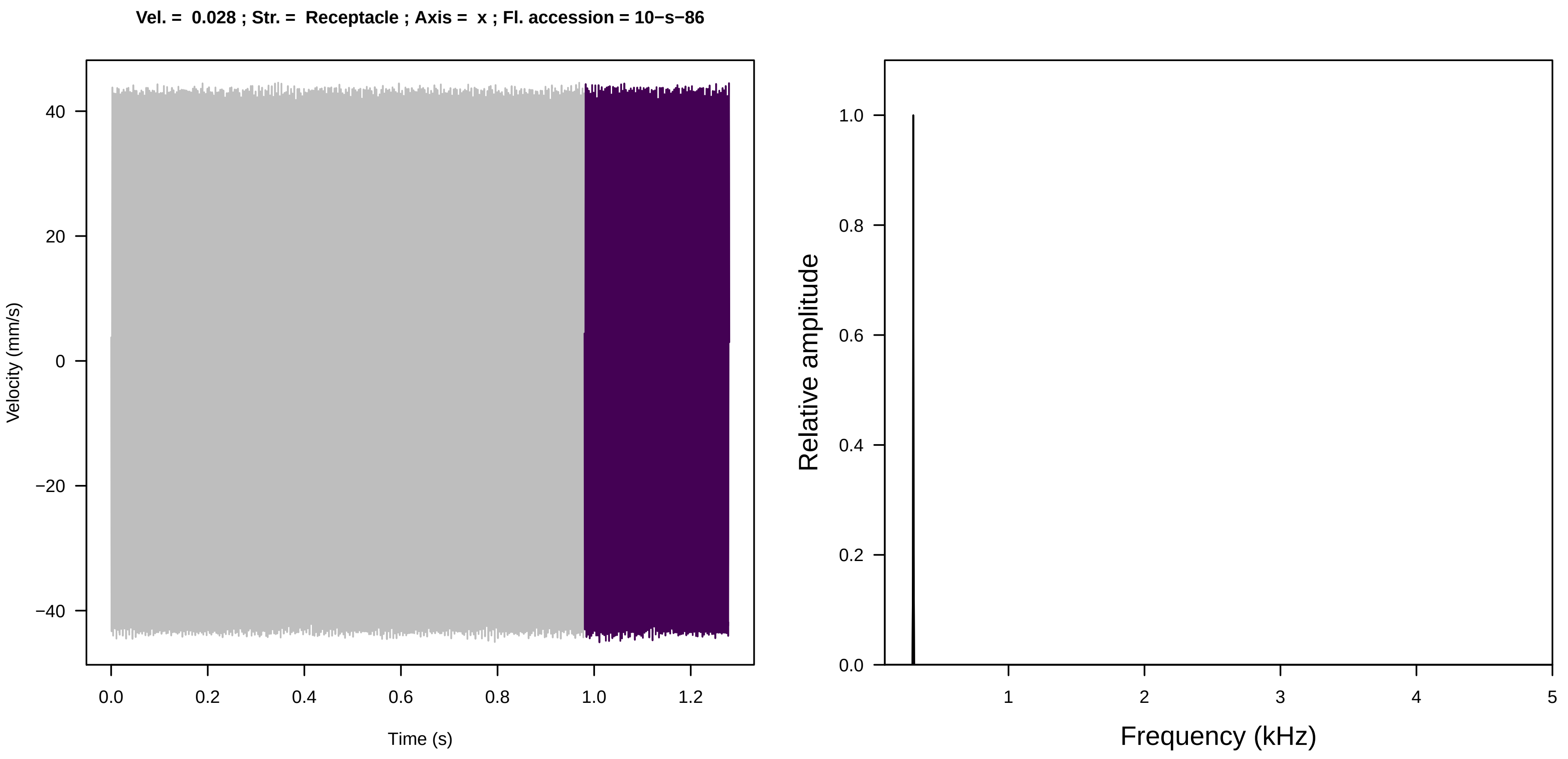
Vel. = 0.028 ; Str. = Receptacle ; Axis = x ; Fl. accession = 10−s−86
Velocity (mm/s)
Time (s)
Relative amplitude
Frequency (kHz)

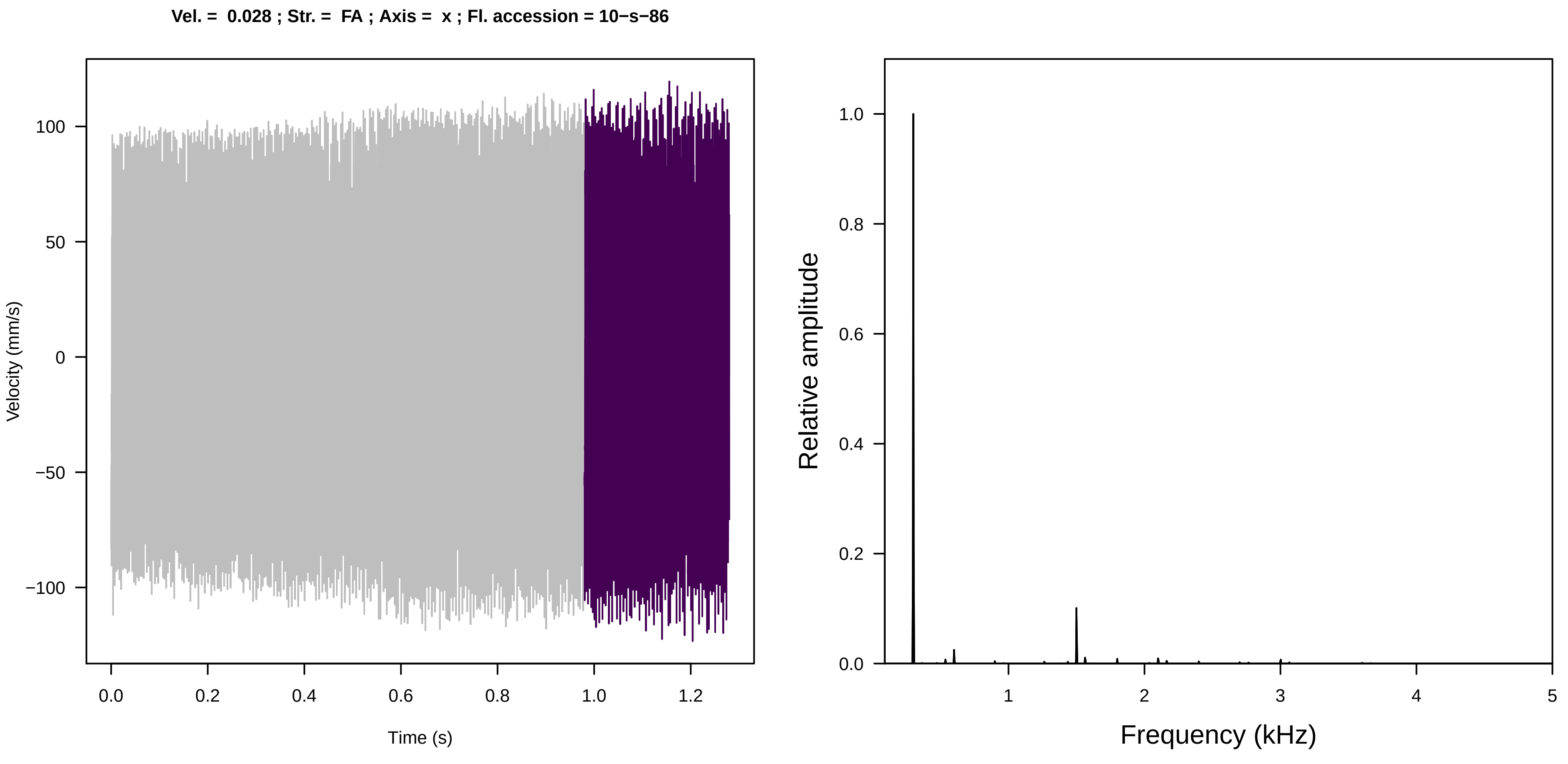
Vel. = 0.028 ; Str. = FA ; Axis = x ; Fl. accession = 10−s−86
Velocity (mm/s)
Time (s)
Relative amplitude
Frequency (kHz)

Vel. = 0.028 ; Str. = Receptacle ; Axis = x ; Fl. accession = 10−s−86

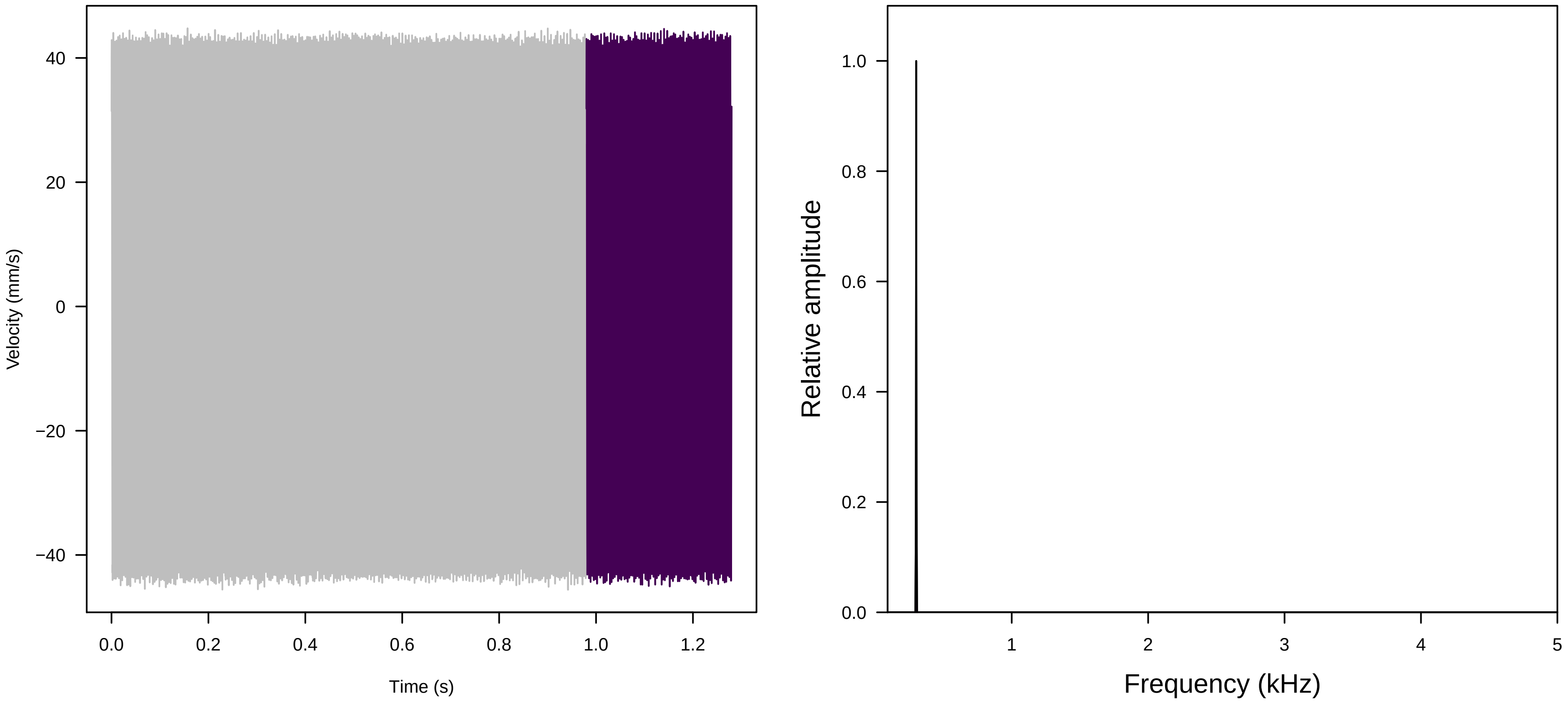

Vel. = 0.028 ; Str. = Corolla ; Axis = x ; Fl. accession = 10−s−86

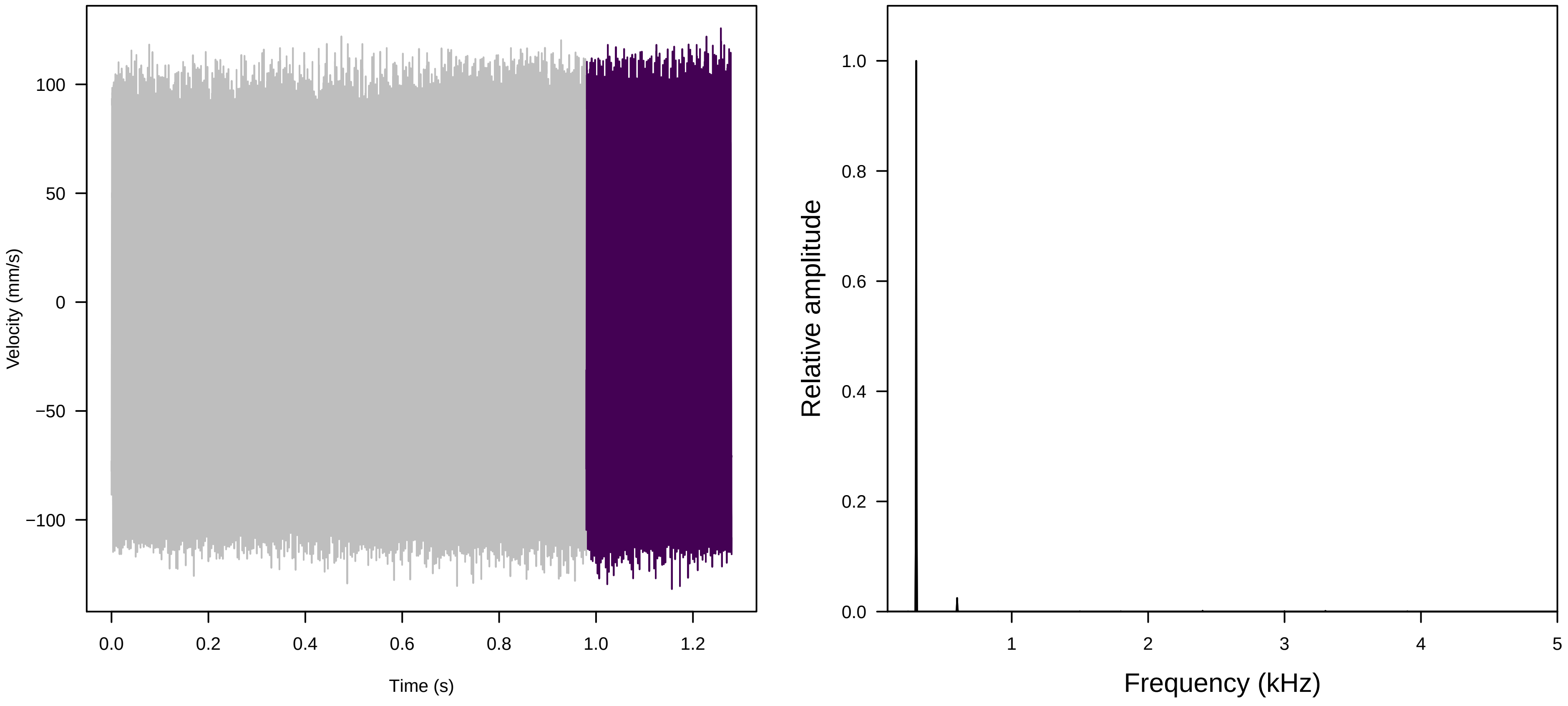

Vel. = 0.028 ; Str. = Receptacle ; Axis = x ; Fl. accession = 10−s−86

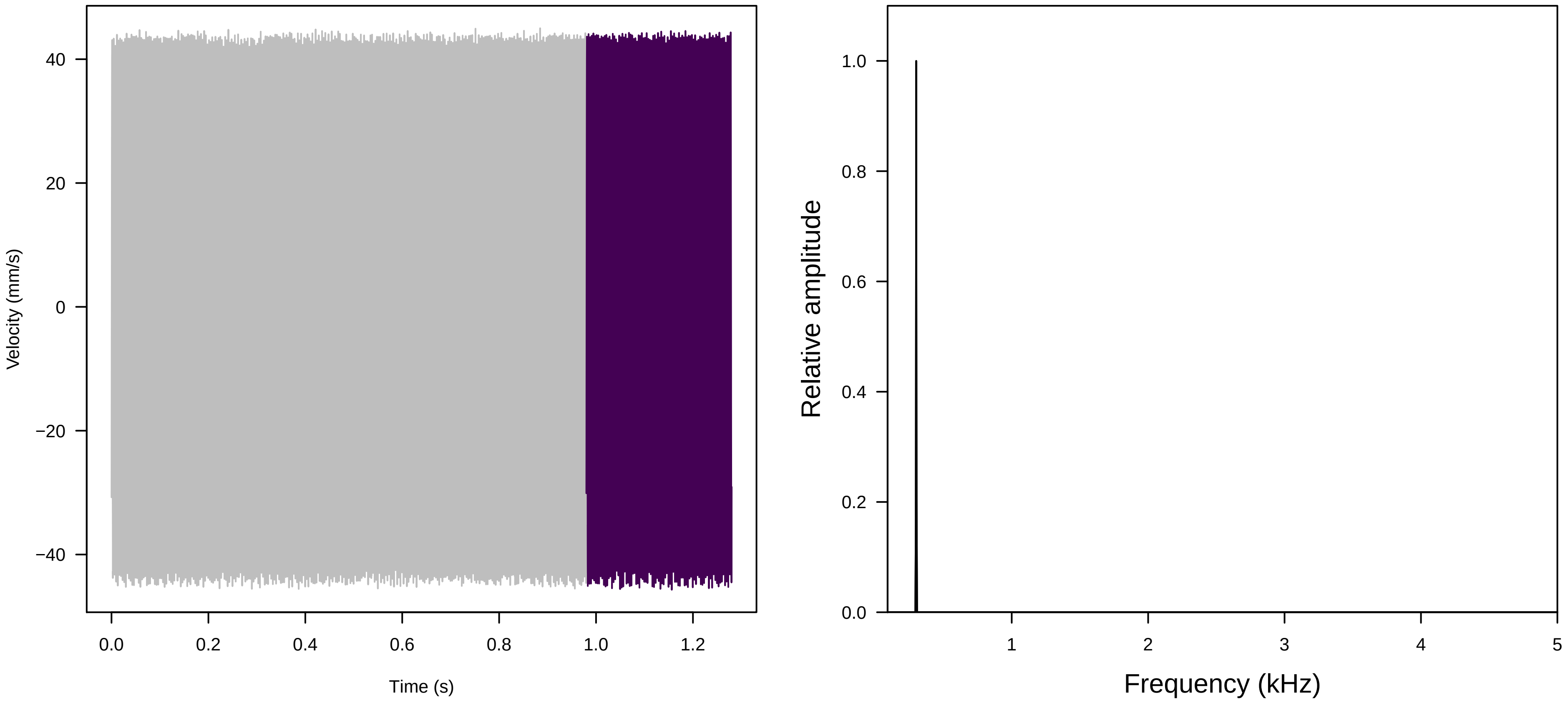

Vel. = 0.057 ; Str. = Corolla ; Axis = x ; Fl. accession = 10−s−86

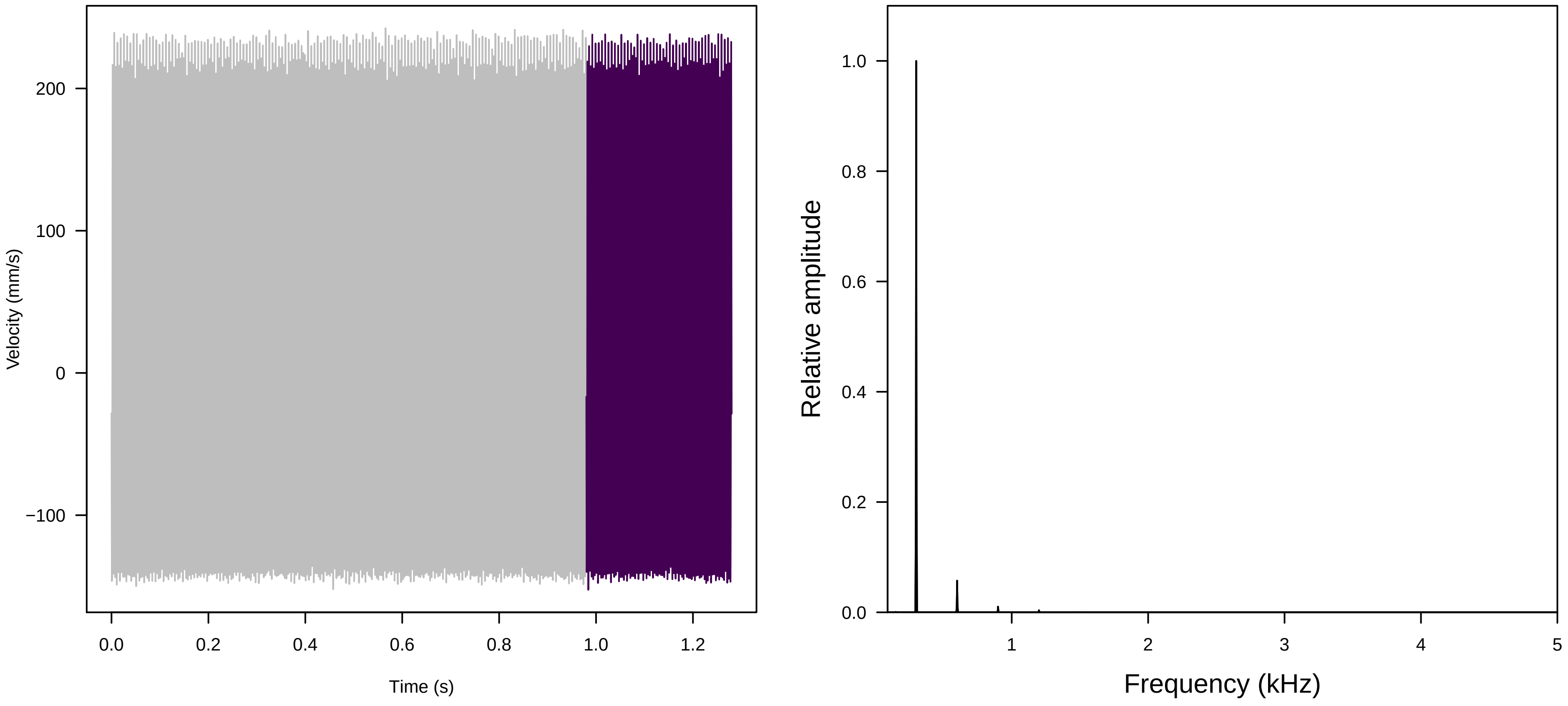

Vel. = 0.057 ; Str. = Receptacle ; Axis = x ; Fl. accession = 10−s−86

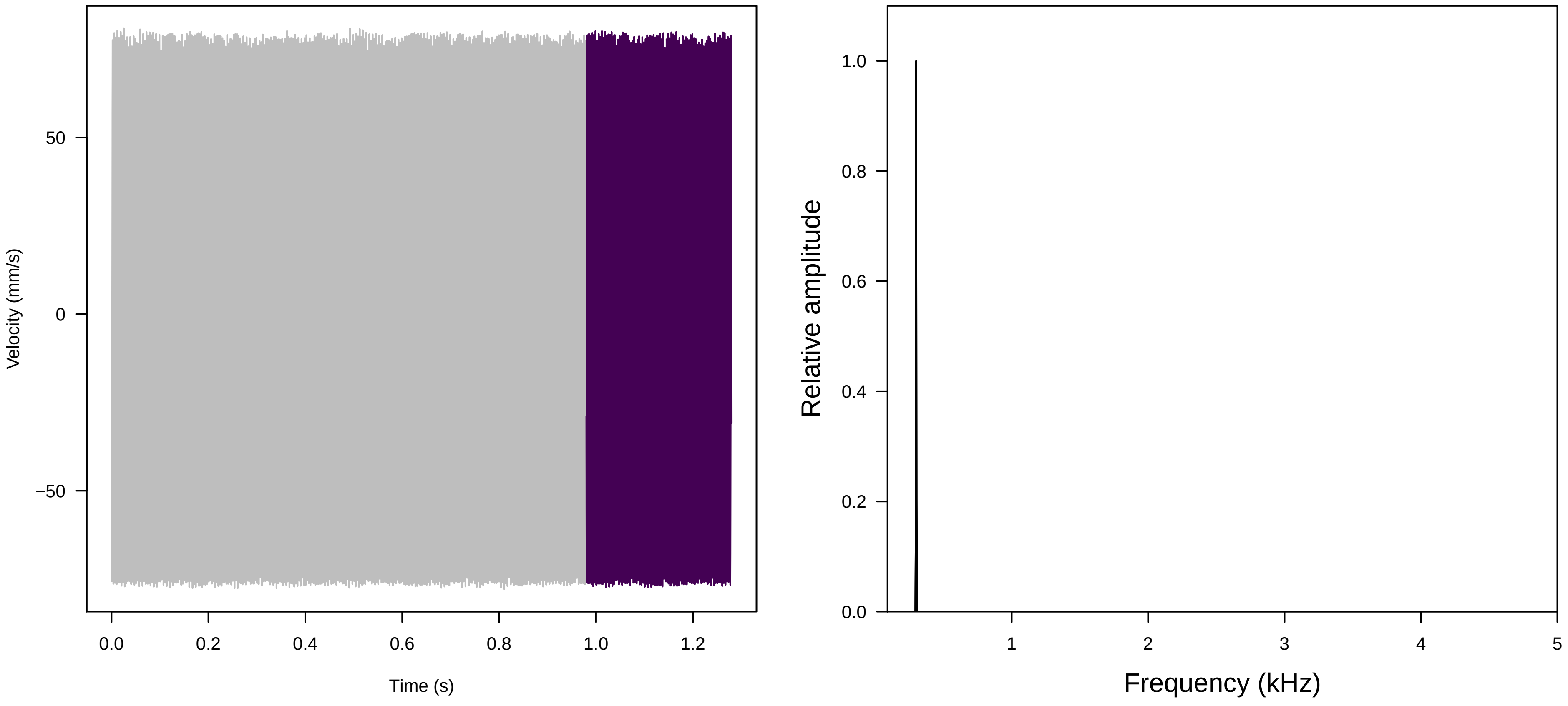

Vel. = 0.057 ; Str. = FA ; Axis = x ; Fl. accession = 10−s−86

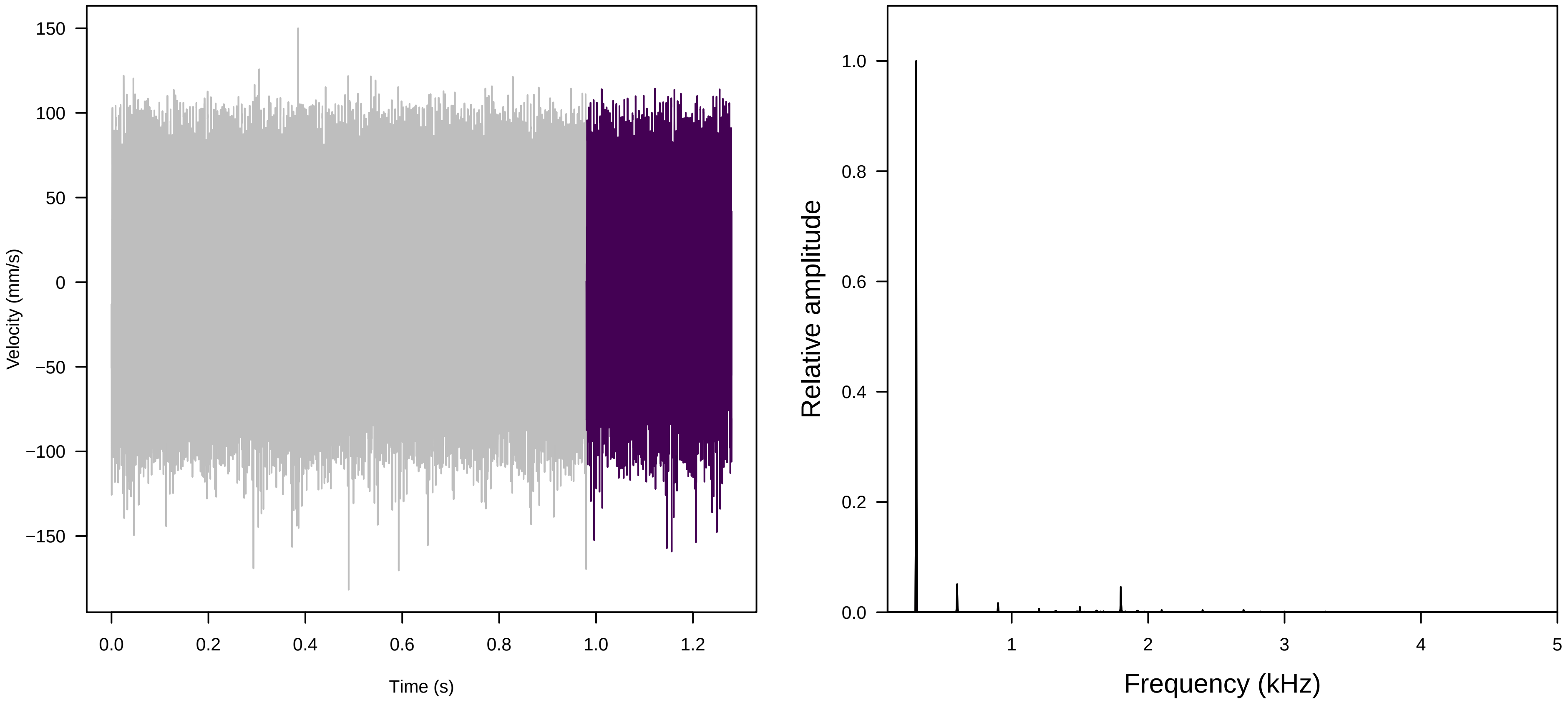

Vel. = 0.057 ; Str. = Receptacle ; Axis = x ; Fl. accession = 10−s−86

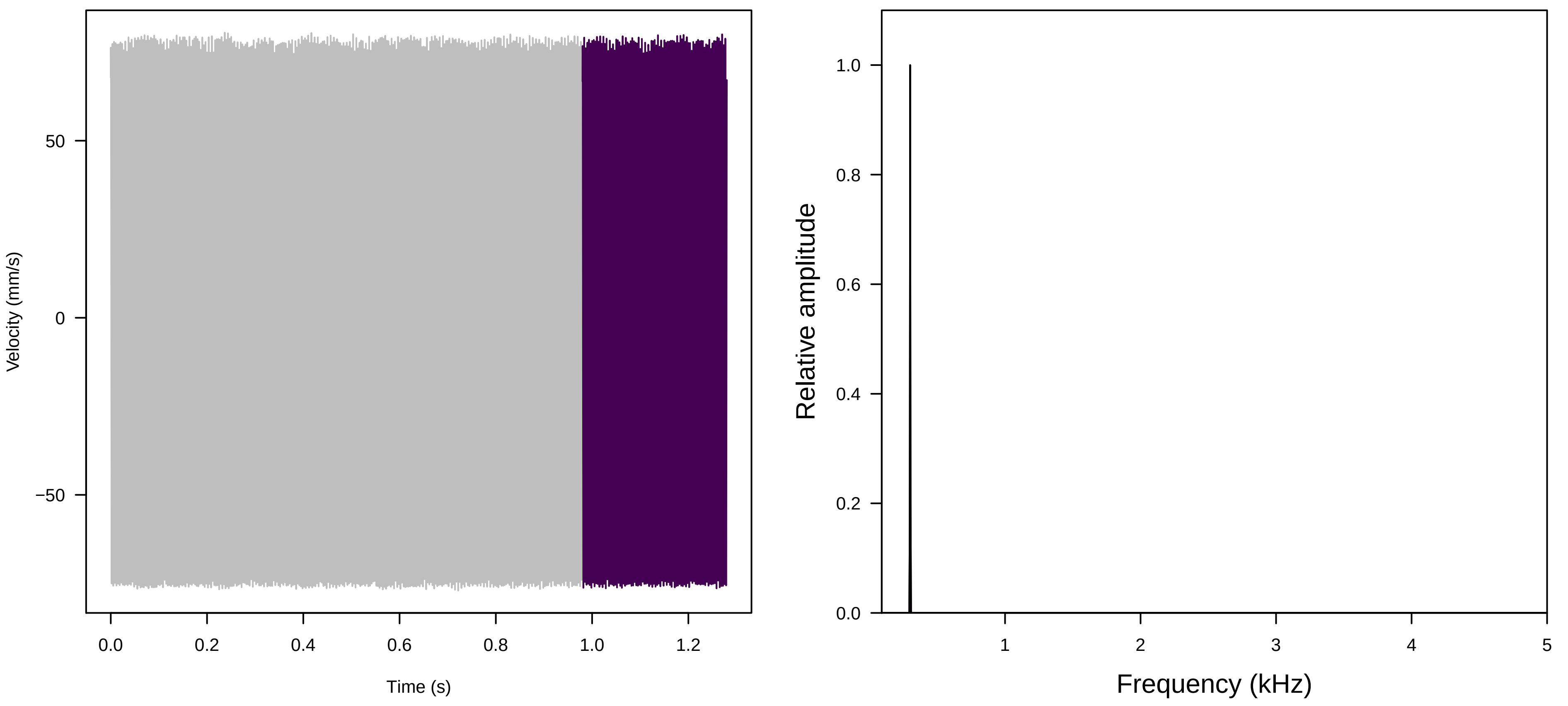

Vel. = 0.057 ; Str. = PA ; Axis = x ; Fl. accession = 10−s−86

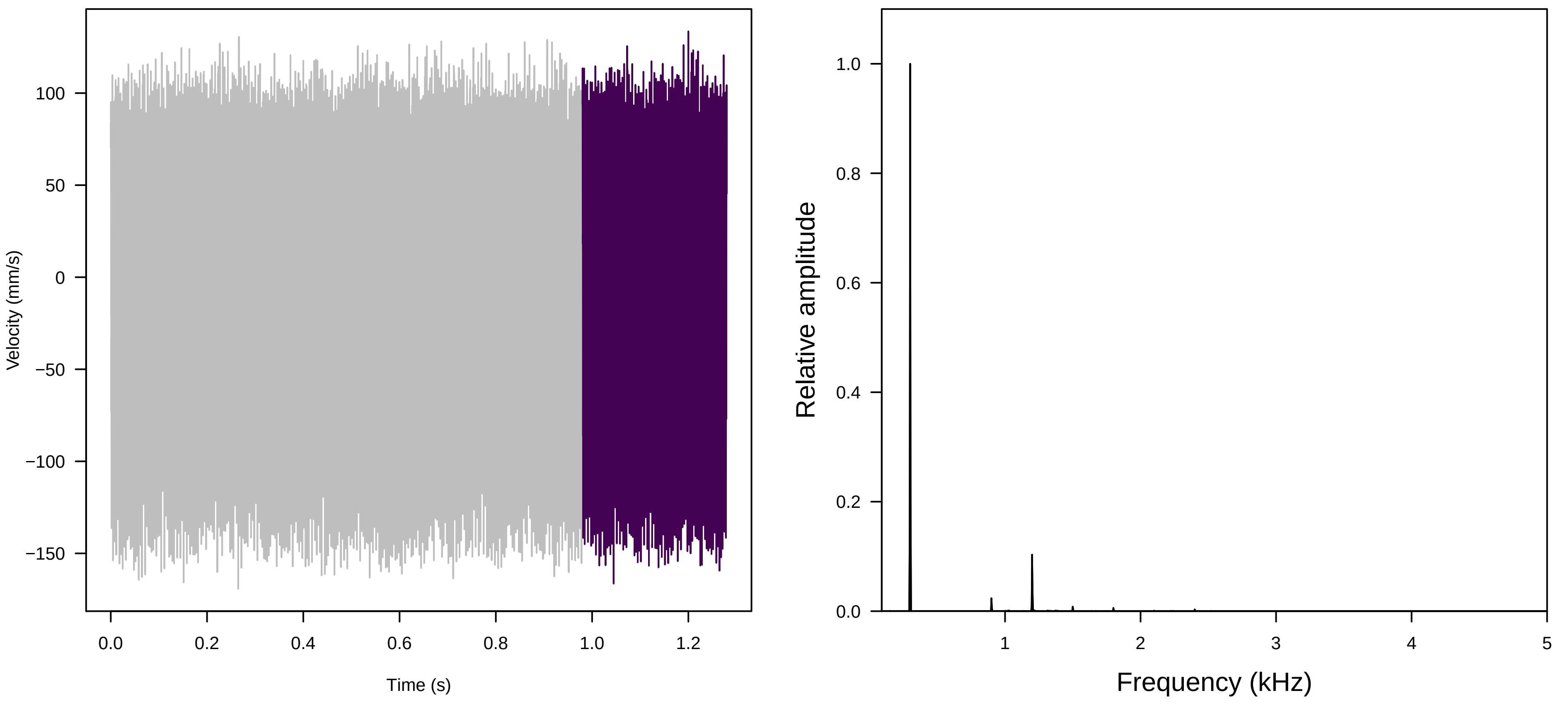

Vel. = 0.057 ; Str. = Receptacle ; Axis = x ; Fl. accession = 10−s−86

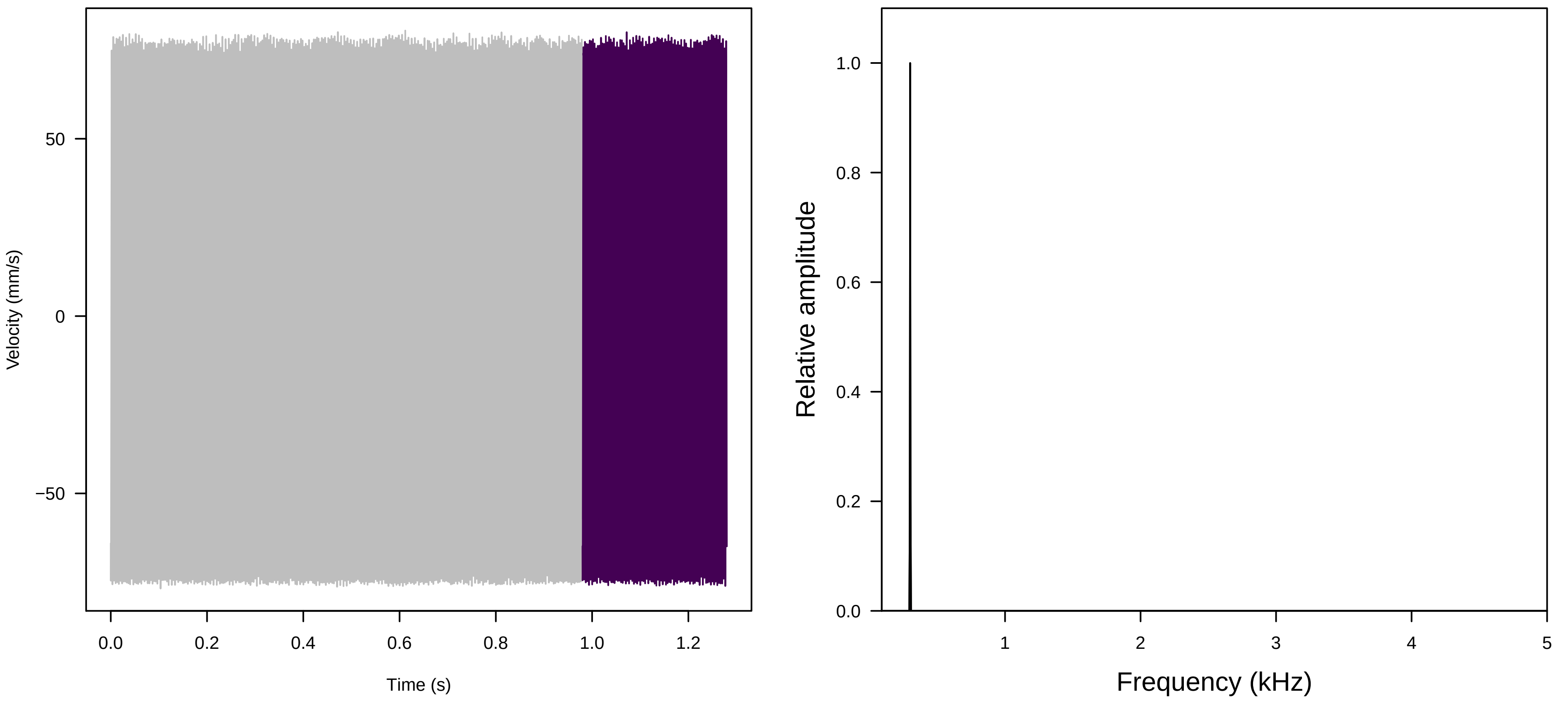

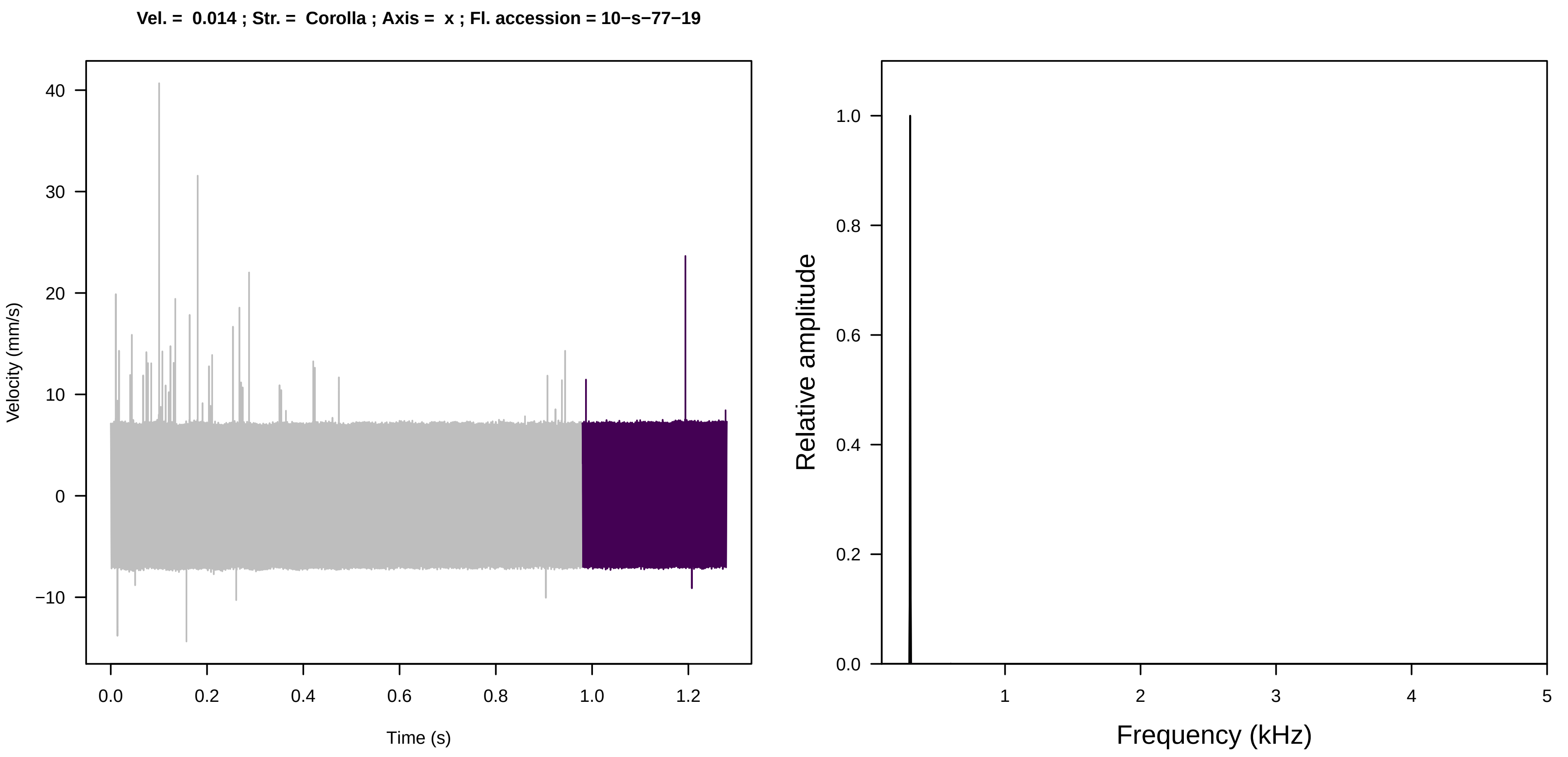
Vel. = 0.014 ; Str. = Corolla ; Axis = x ; Fl. accession = 10−s−77−19
Velocity (mm/s)
Time (s)
Relative amplitude
Frequency (kHz)

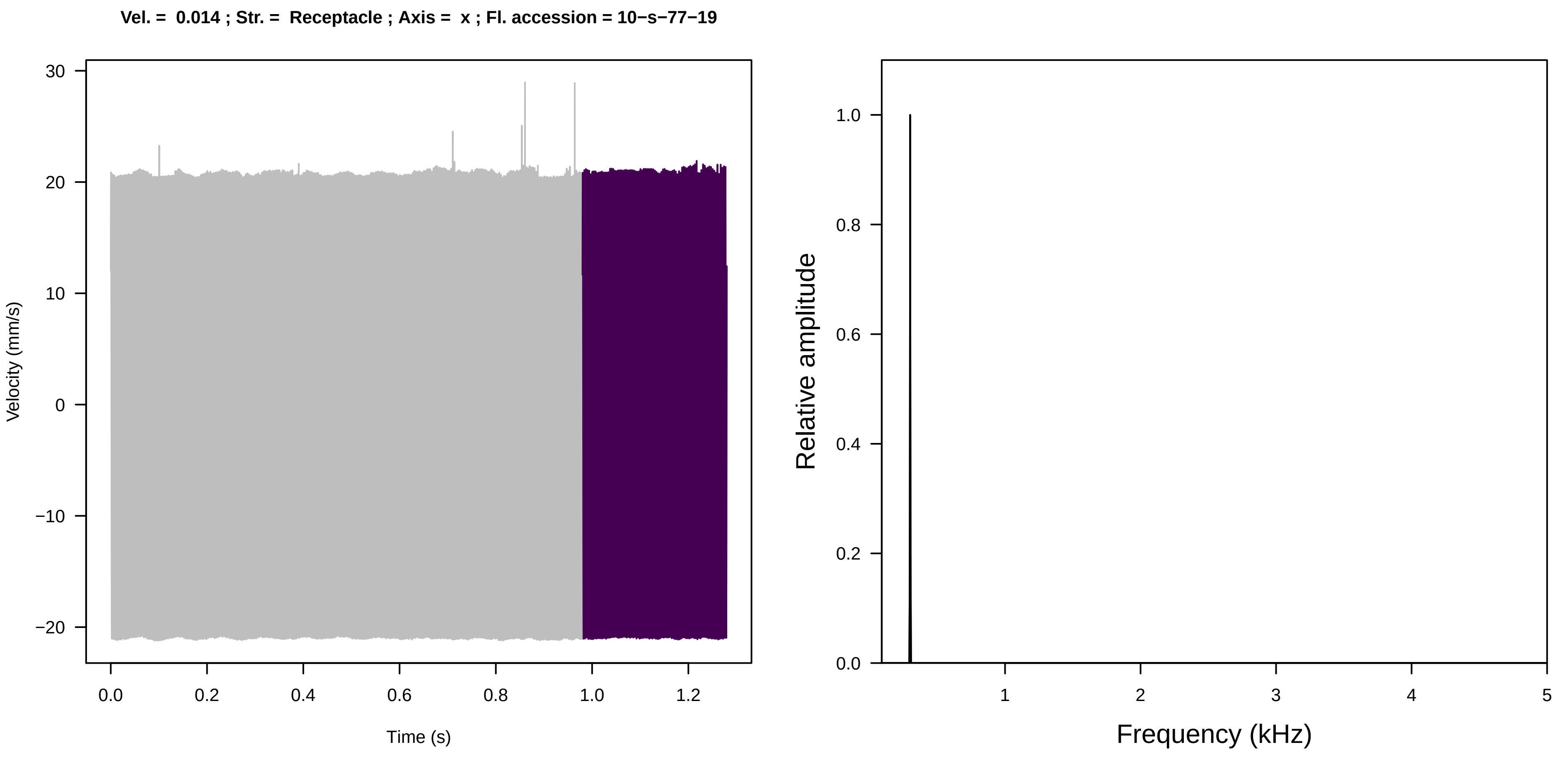
Vel. = 0.014 ; Str. = Receptacle ; Axis = x ; Fl. accession = 10−s−77−19
Velocity (mm/s)
Time (s)
Relative amplitude
Frequency (kHz)

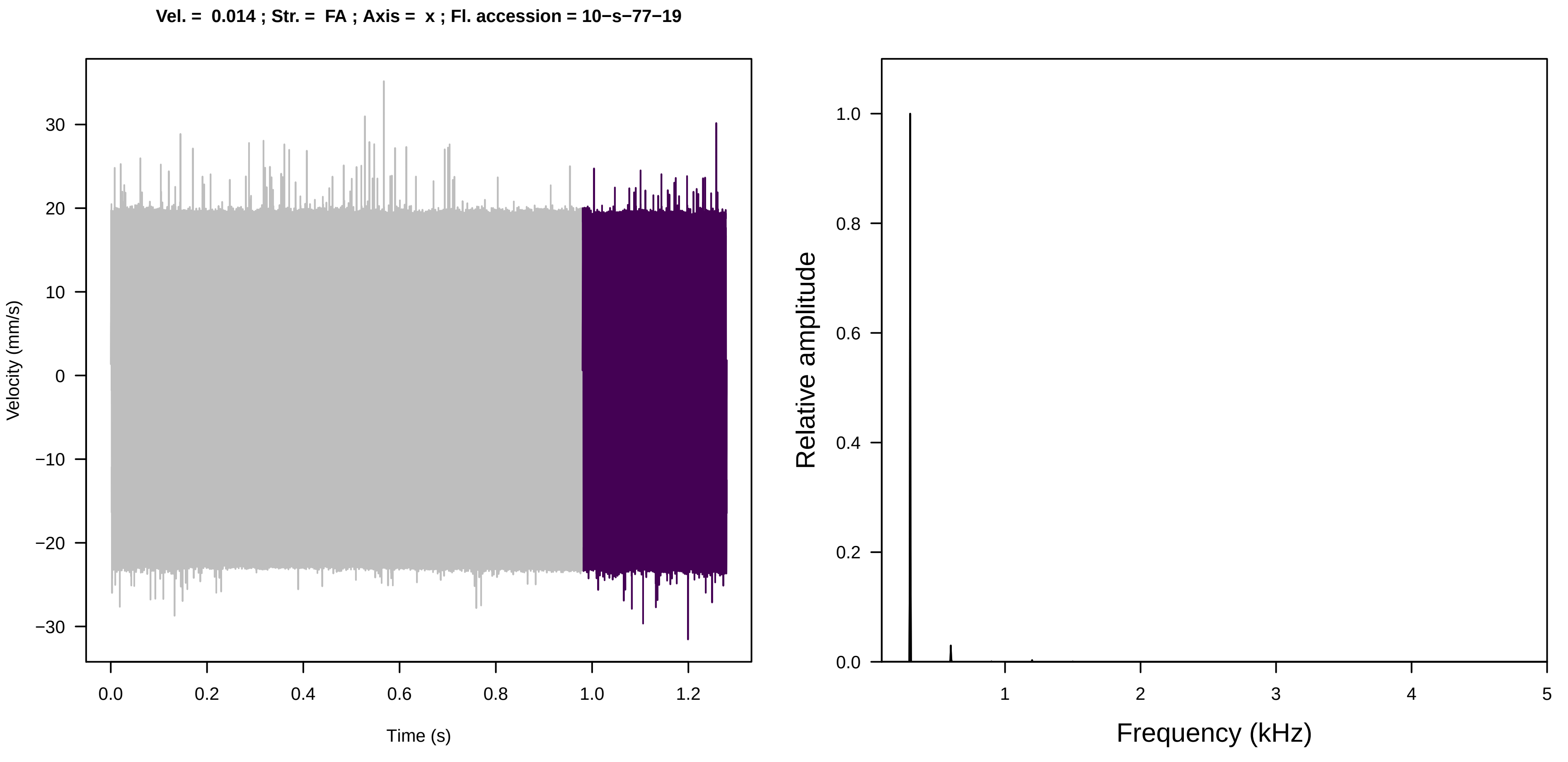
Vel. = 0.014 ; Str. = FA ; Axis = x ; Fl. accession = 10−s−77−19
Velocity (mm/s)
Time (s)
Relative amplitude
Frequency (kHz)

Vel. = 0.014 ; Str. = Receptacle ; Axis = x ; Fl. accession = 10−s−77−19

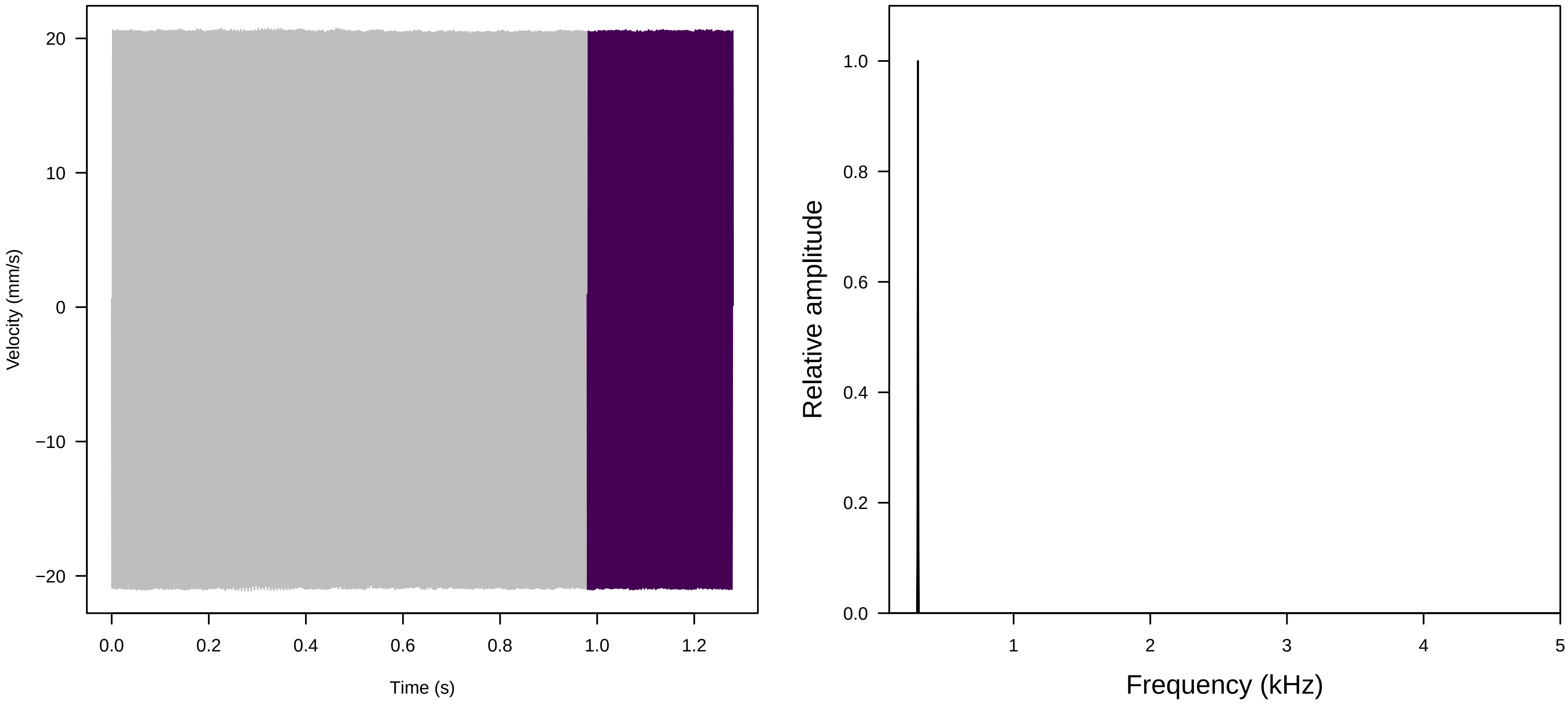

Vel. = 0.014 ; Str. = PA ; Axis = x ; Fl. accession = 10−s−77−19

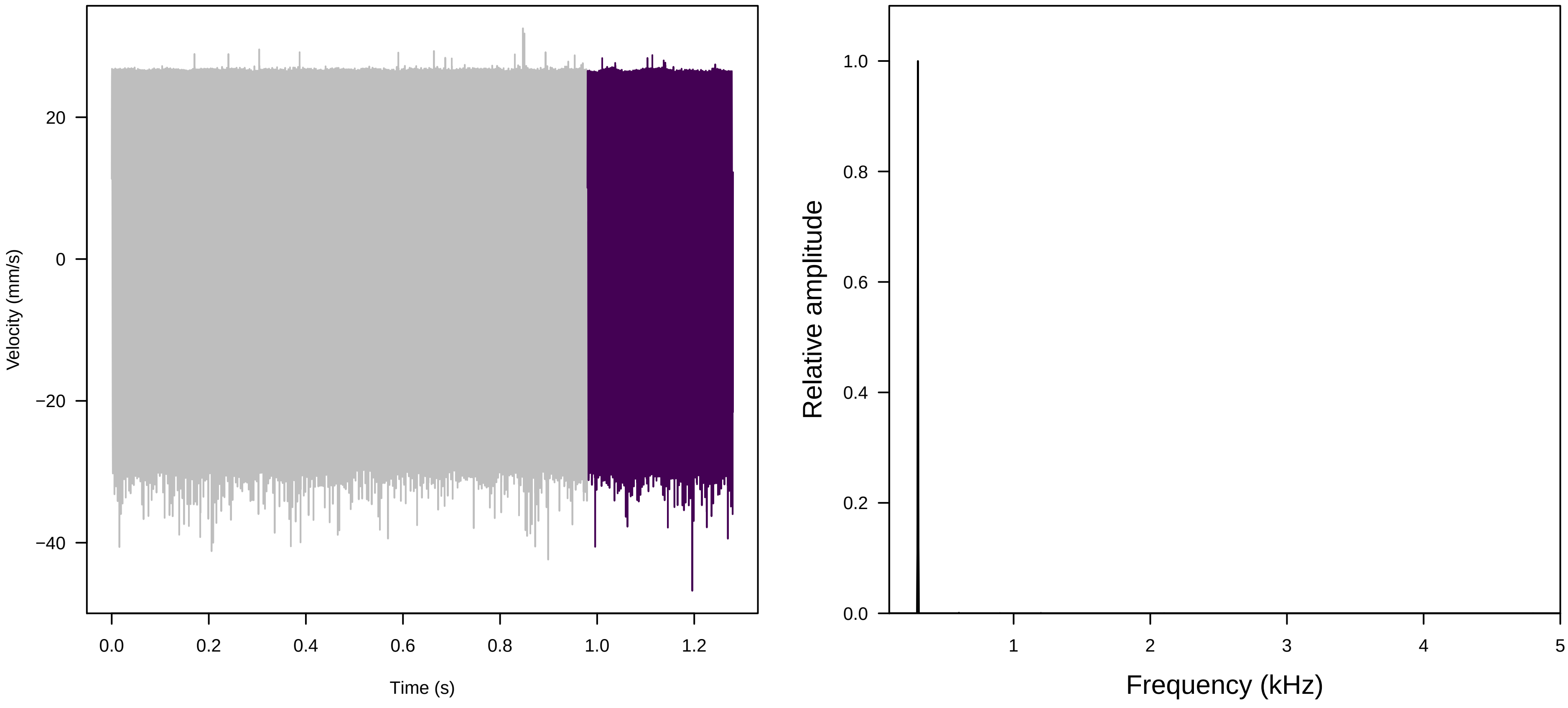

Vel. = 0.014 ; Str. = Receptacle ; Axis = x ; Fl. accession = 10−s−77−19

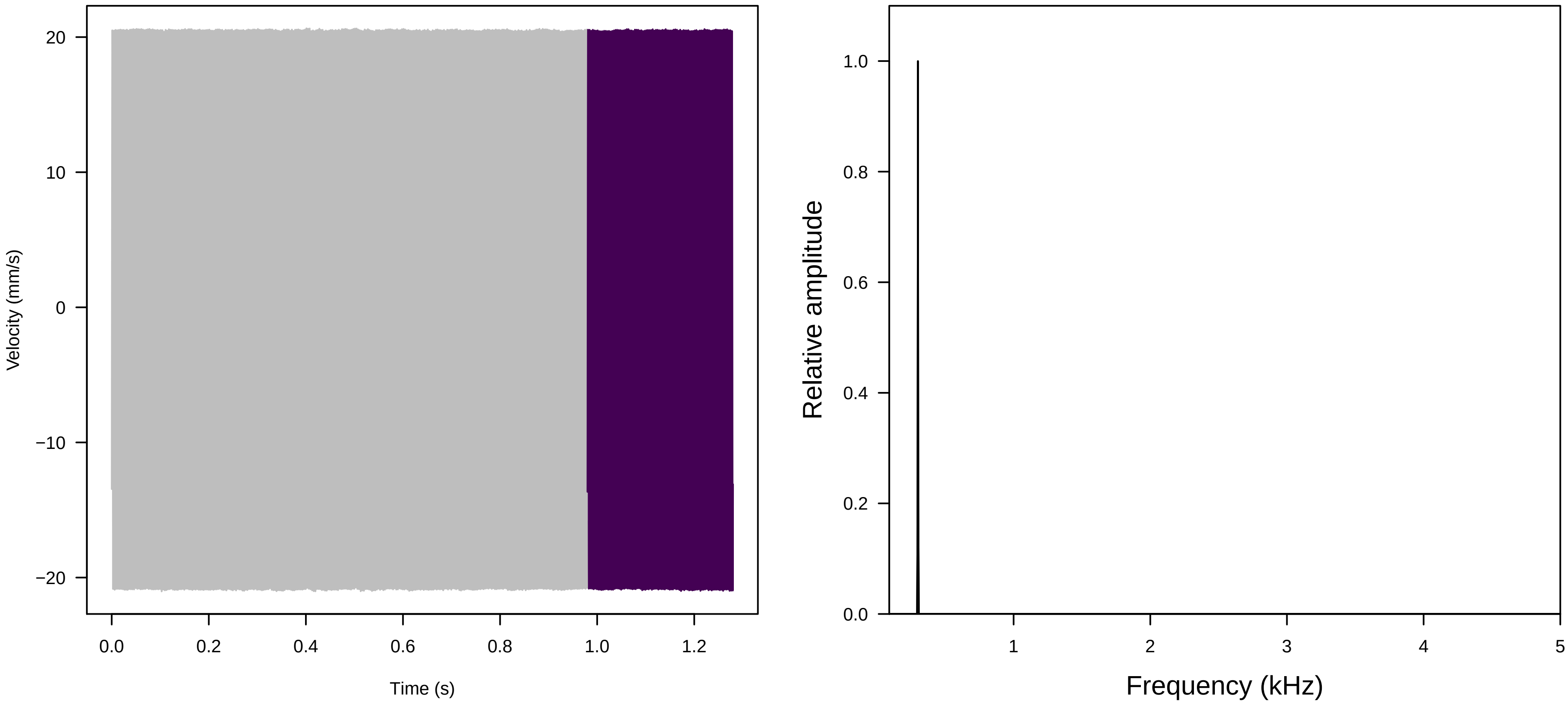

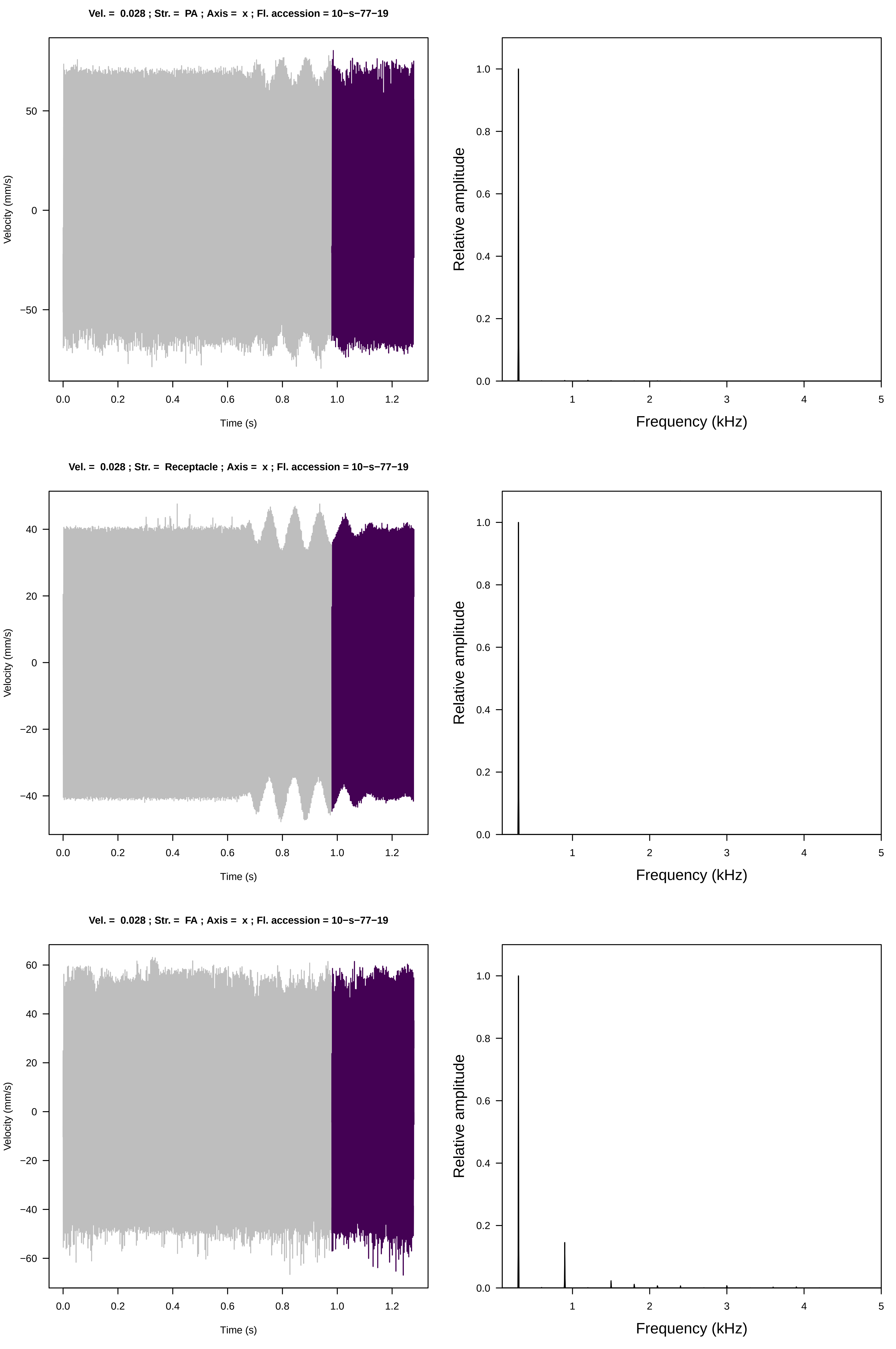
Vel. = 0.028 ; Str. = PA ; Axis = x ; Fl. accession = 10−s−77−19
Velocity (mm/s)
Time (s)
Relative amplitude
Frequency (kHz)
Vel. = 0.028 ; Str. = Receptacle ; Axis = x ; Fl. accession = 10−s−77−19
Velocity (mm/s)
Time (s)
Relative amplitude
Frequency (kHz)
Vel. = 0.028 ; Str. = FA ; Axis = x ; Fl. accession = 10−s−77−19
Velocity (mm/s)
Time (s)
Relative amplitude
Frequency (kHz)

Vel. = 0.028 ; Str. = Receptacle ; Axis = x ; Fl. accession = 10−s−77−19

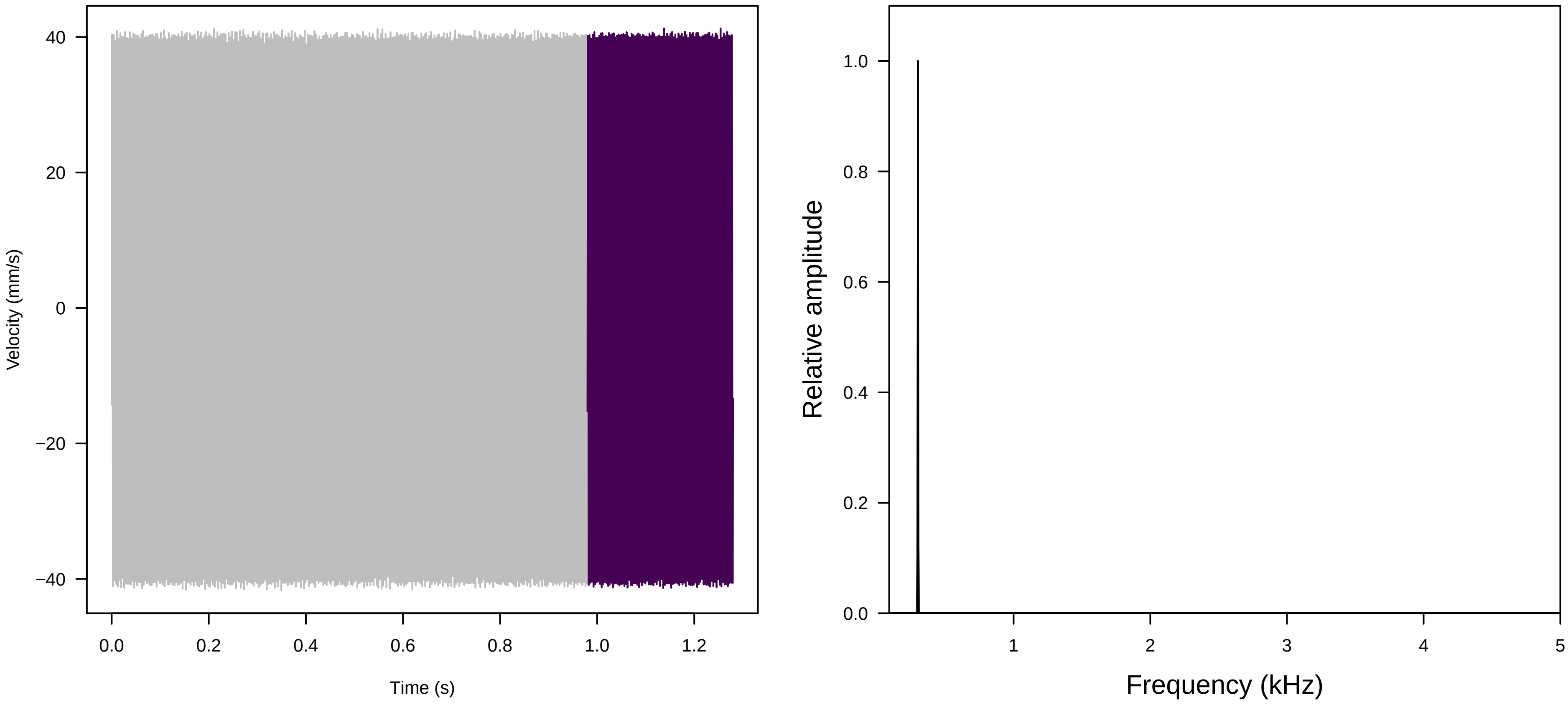

Vel. = 0.028 ; Str. = Corolla ; Axis = x ; Fl. accession = 10−s−77−19

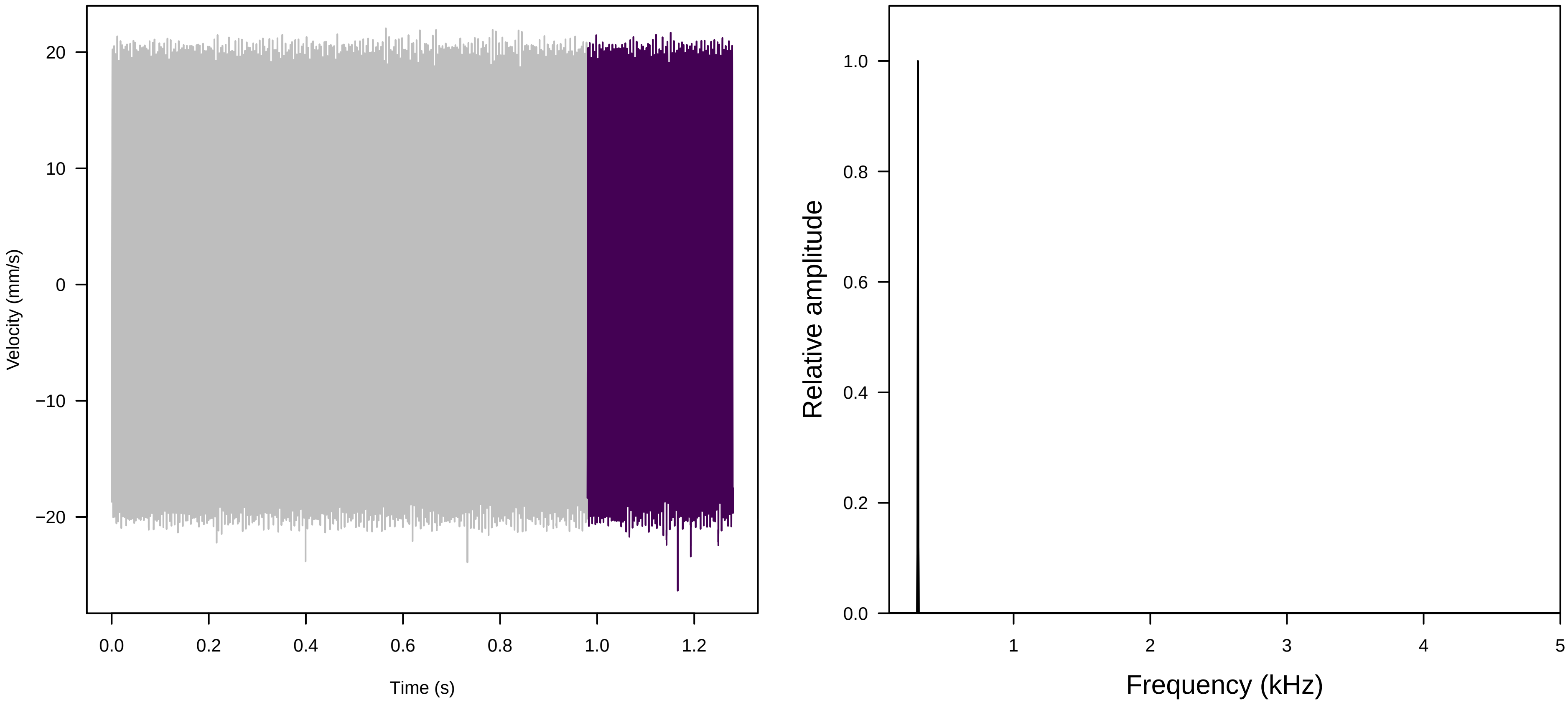

Vel. = 0.028 ; Str. = Receptacle ; Axis = x ; Fl. accession = 10−s−77−19

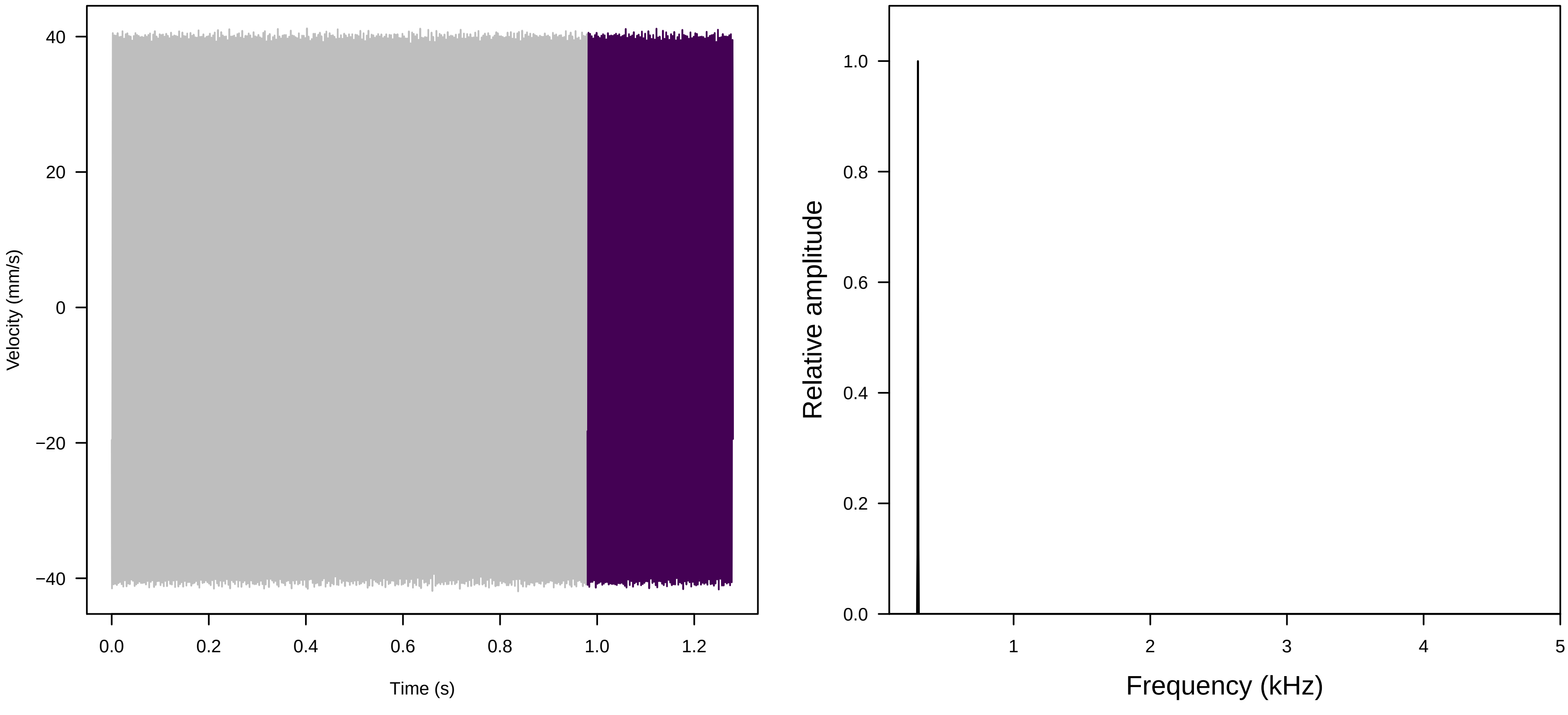

Vel. = 0.057 ; Str. = Corolla ; Axis = x ; Fl. accession = 10−s−77−19

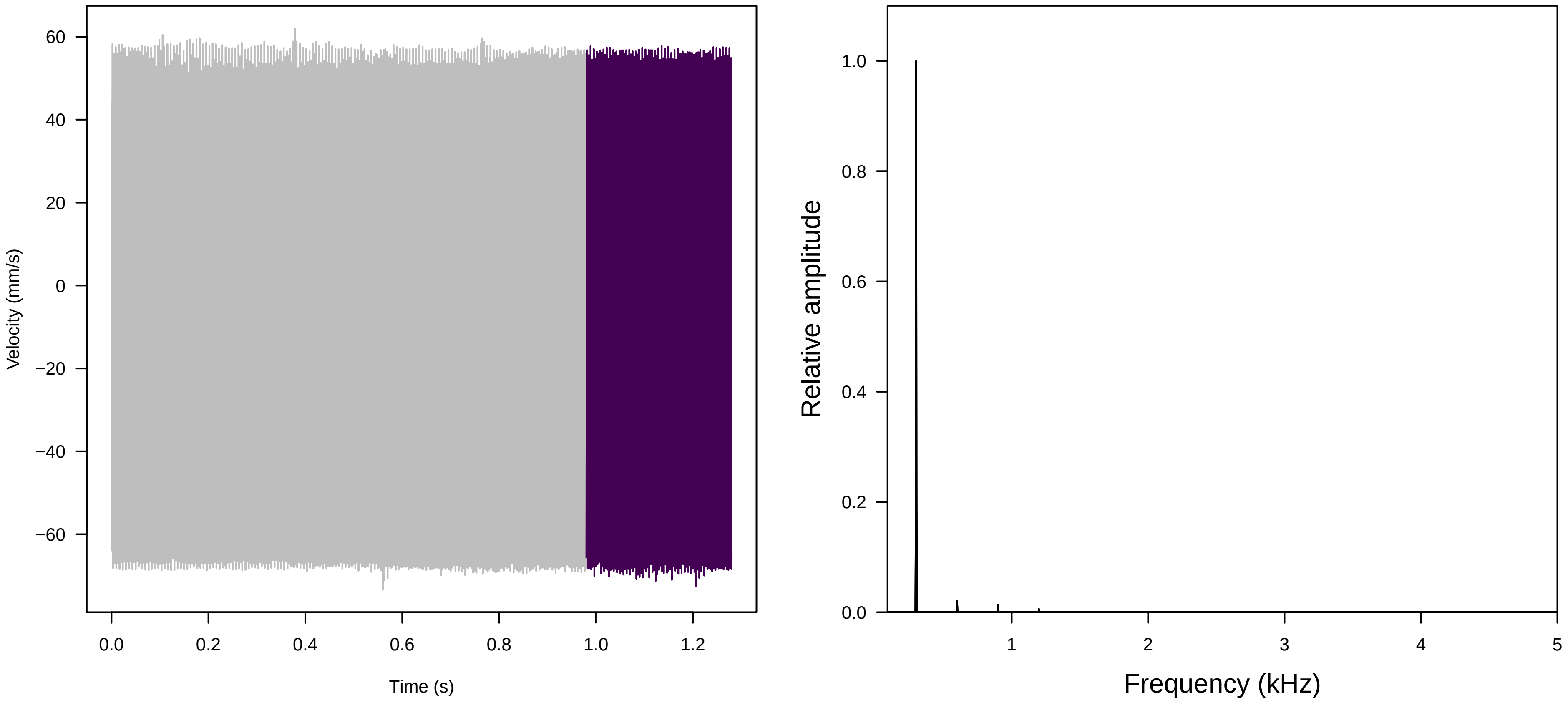

Vel. = 0.057 ; Str. = Receptacle ; Axis = x ; Fl. accession = 10−s−77−19

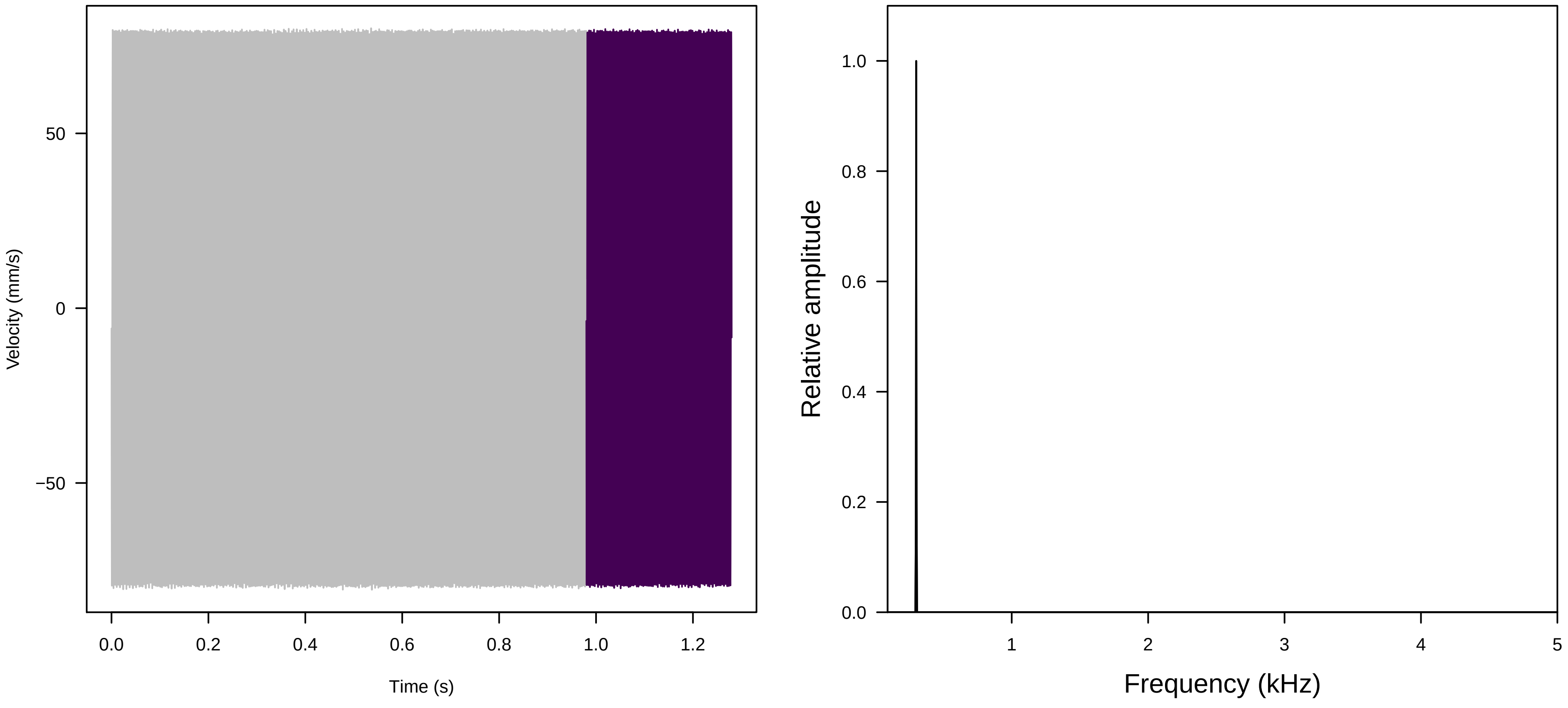

Vel. = 0.057 ; Str. = FA ; Axis = x ; Fl. accession = 10−s−77−19

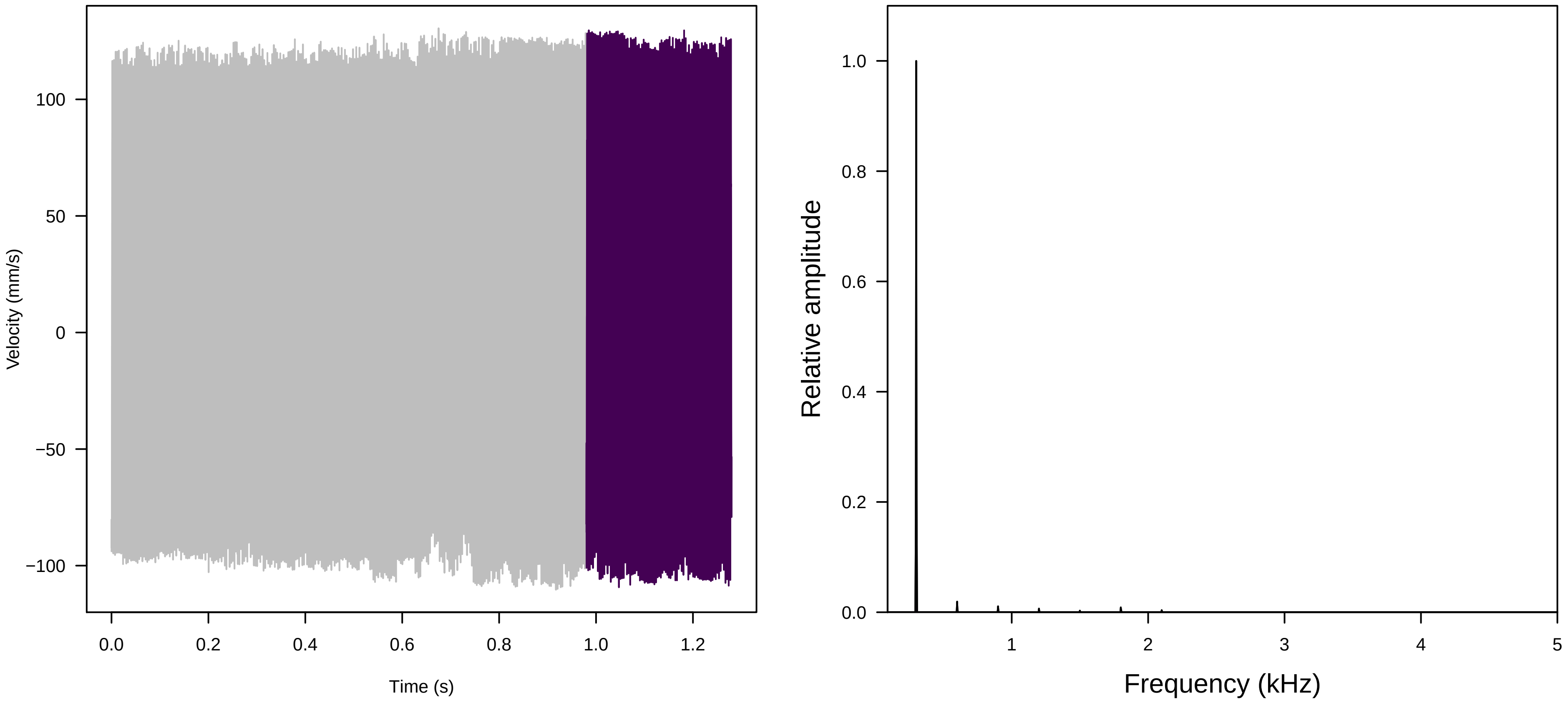

Vel. = 0.057 ; Str. = Receptacle ; Axis = x ; Fl. accession = 10−s−77−19

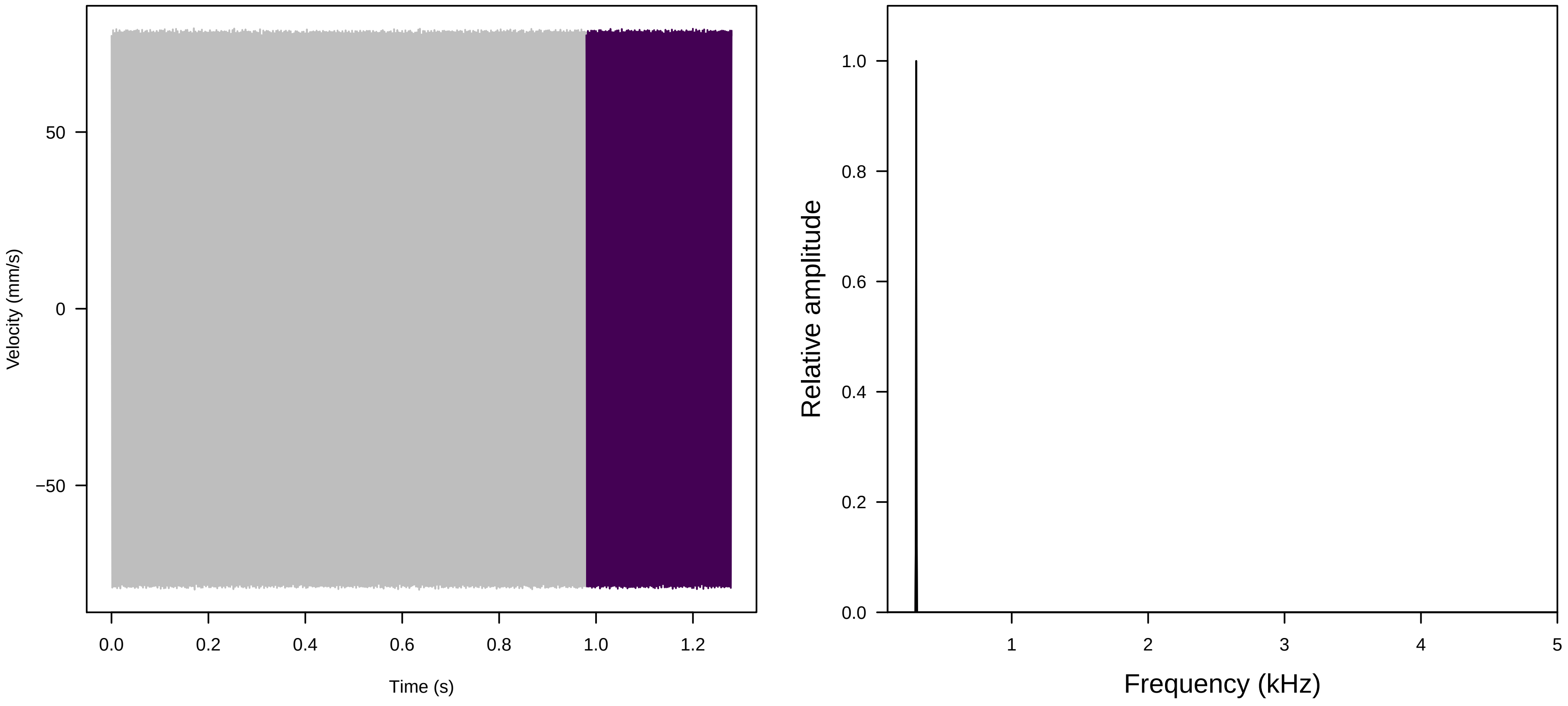

Vel. = 0.057 ; Str. = PA ; Axis = x ; Fl. accession = 10−s−77−19

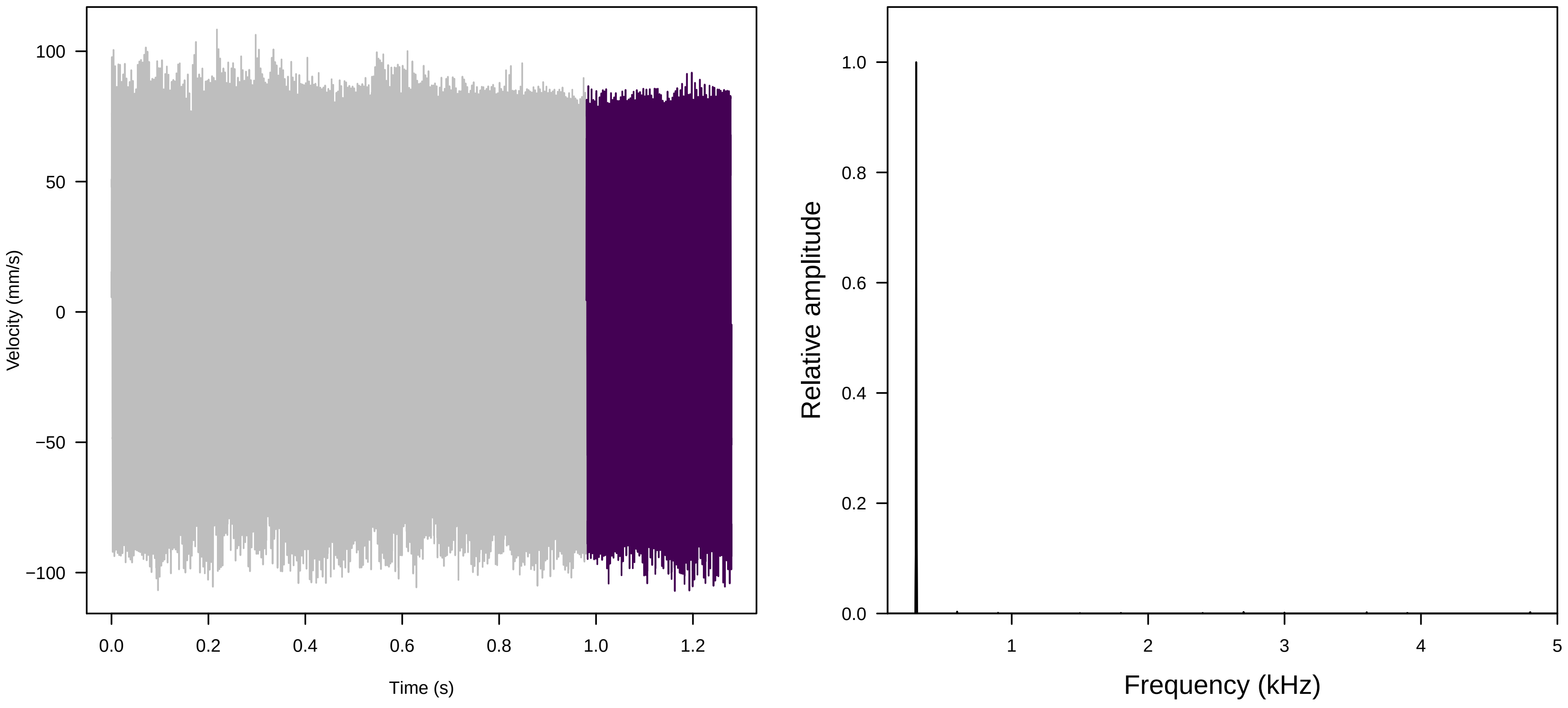

Vel. = 0.057 ; Str. = Receptacle ; Axis = x ; Fl. accession = 10−s−77−19

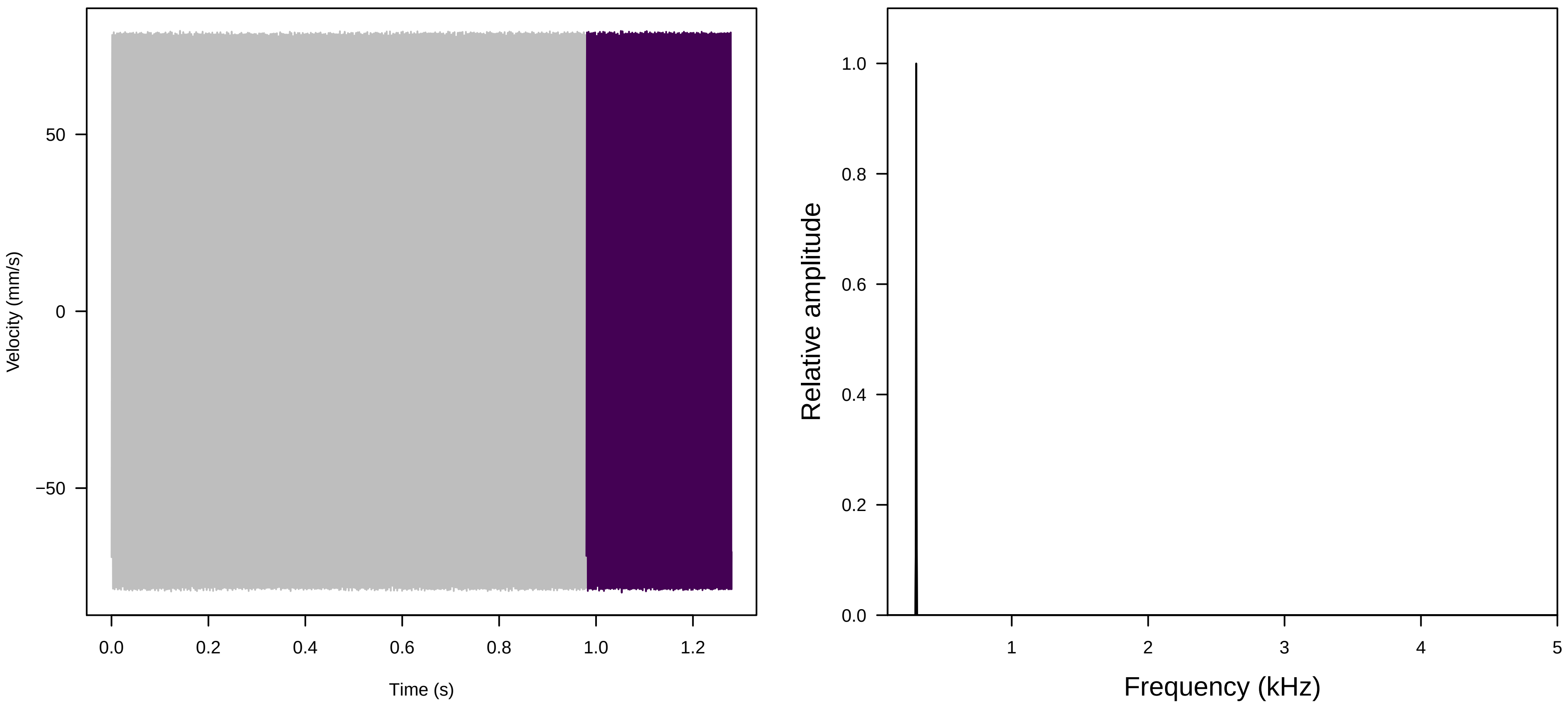

Vel. = 0.057 ; Str. = Corolla ; Axis = z ; Fl. accession = 10−s−77−19

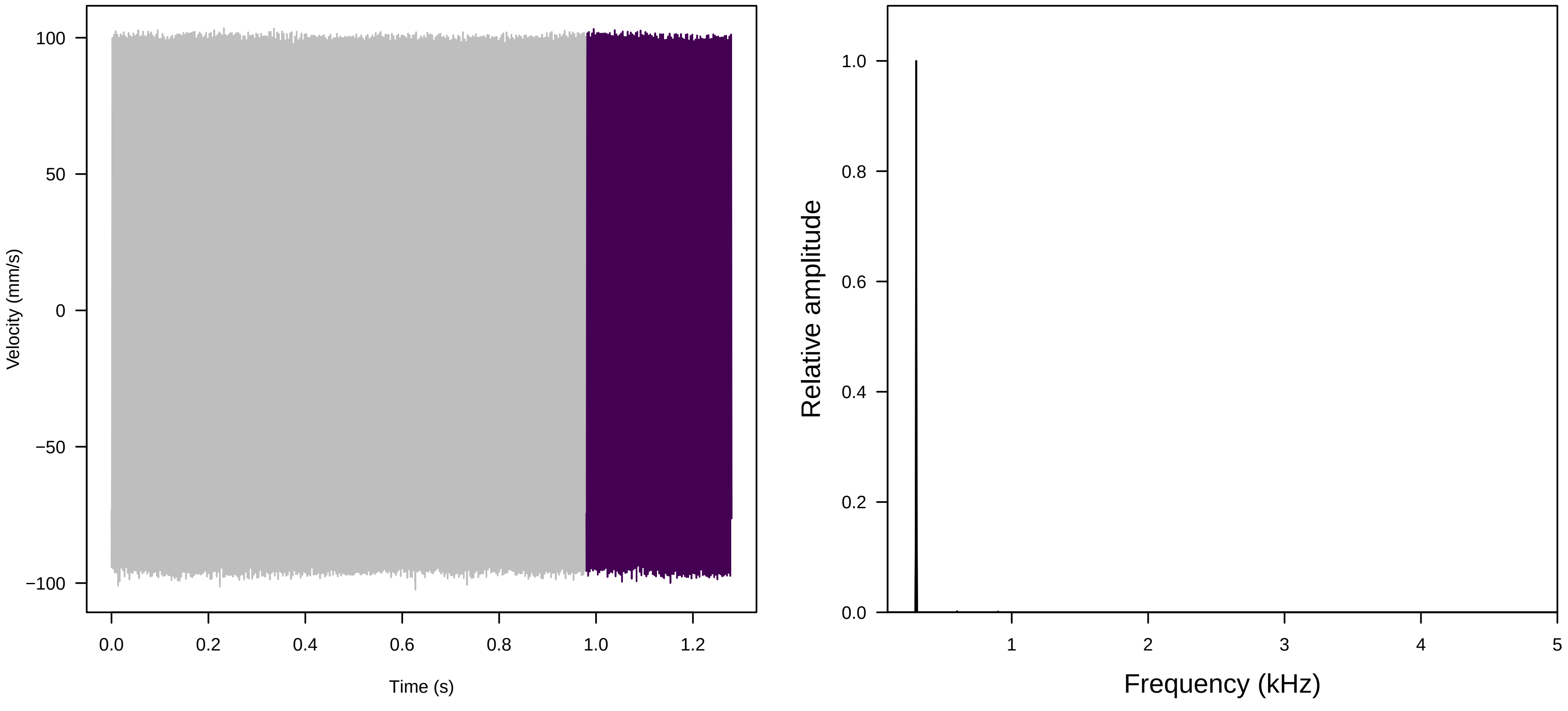

Vel. = 0.057 ; Str. = Receptacle ; Axis = z ; Fl. accession = 10−s−77−19

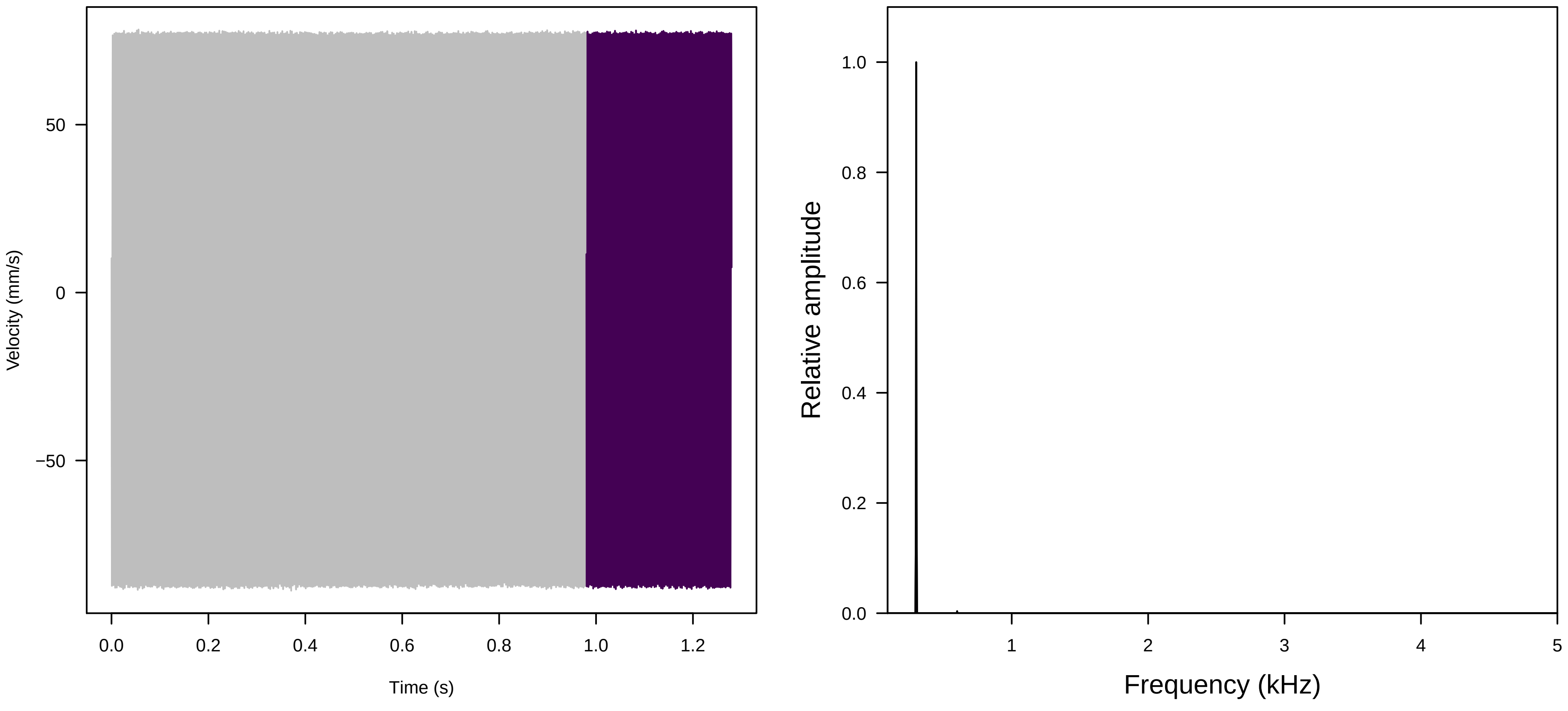

Vel. = 0.057 ; Str. = FA ; Axis = z ; Fl. accession = 10−s−77−19

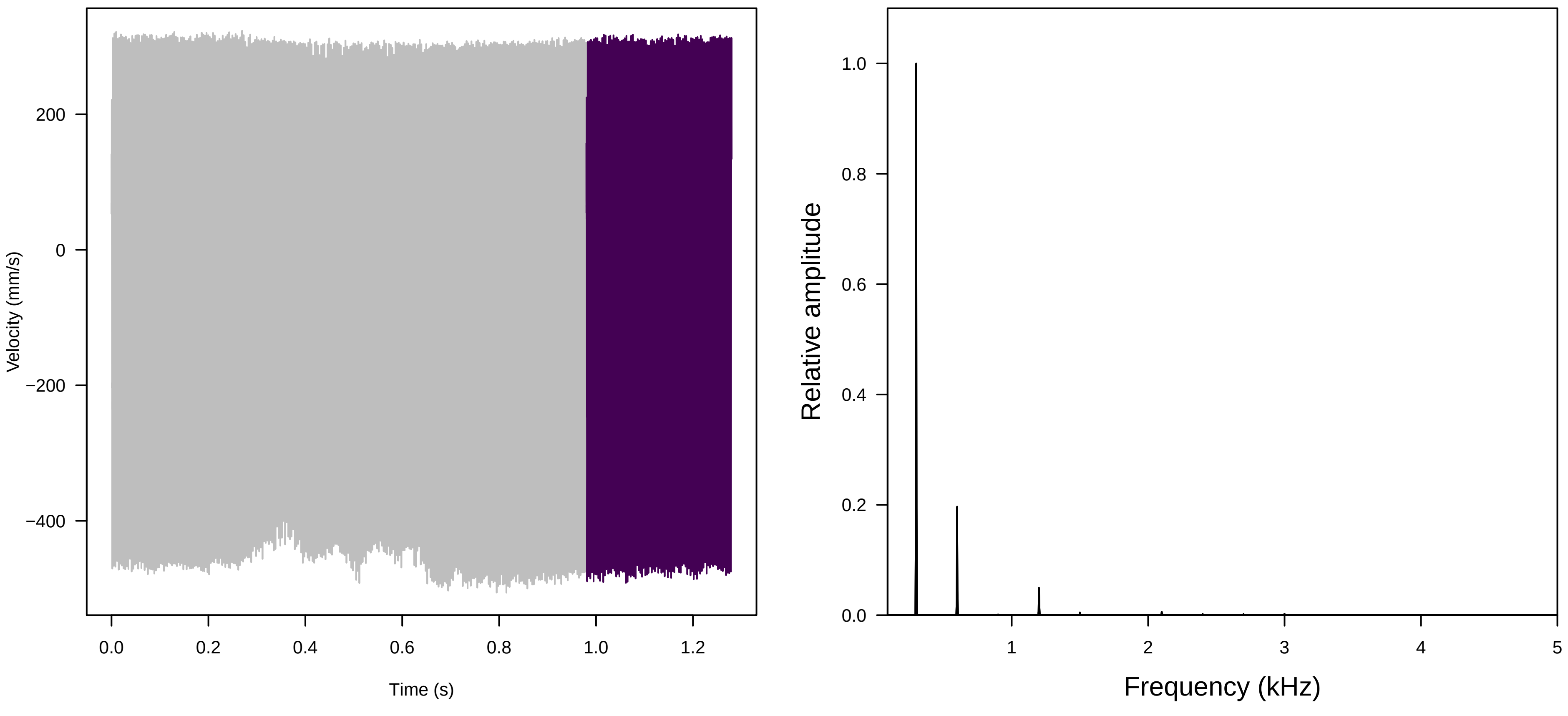

Vel. = 0.057 ; Str. = Receptacle ; Axis = z ; Fl. accession = 10−s−77−19

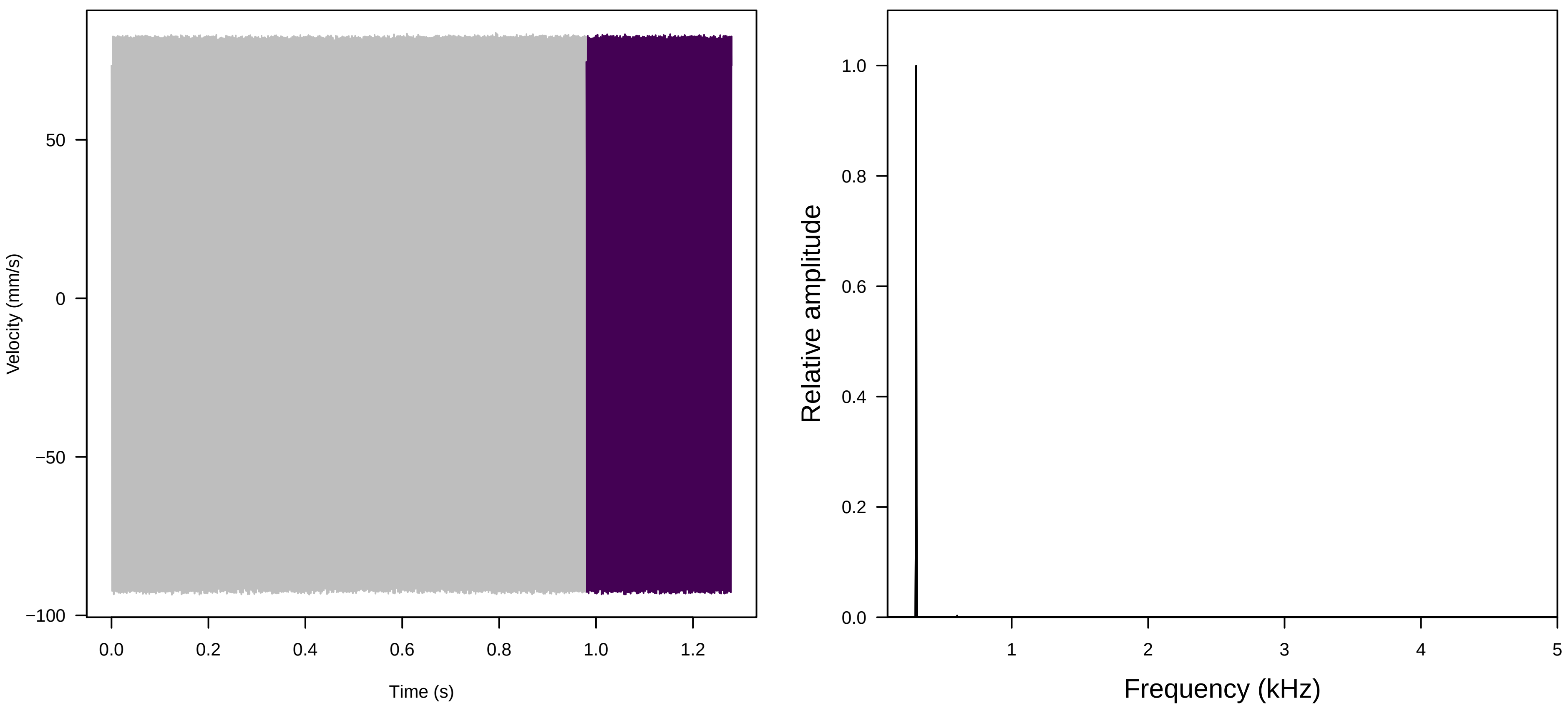

Vel. = 0.057 ; Str. = PA ; Axis = z ; Fl. accession = 10−s−77−19

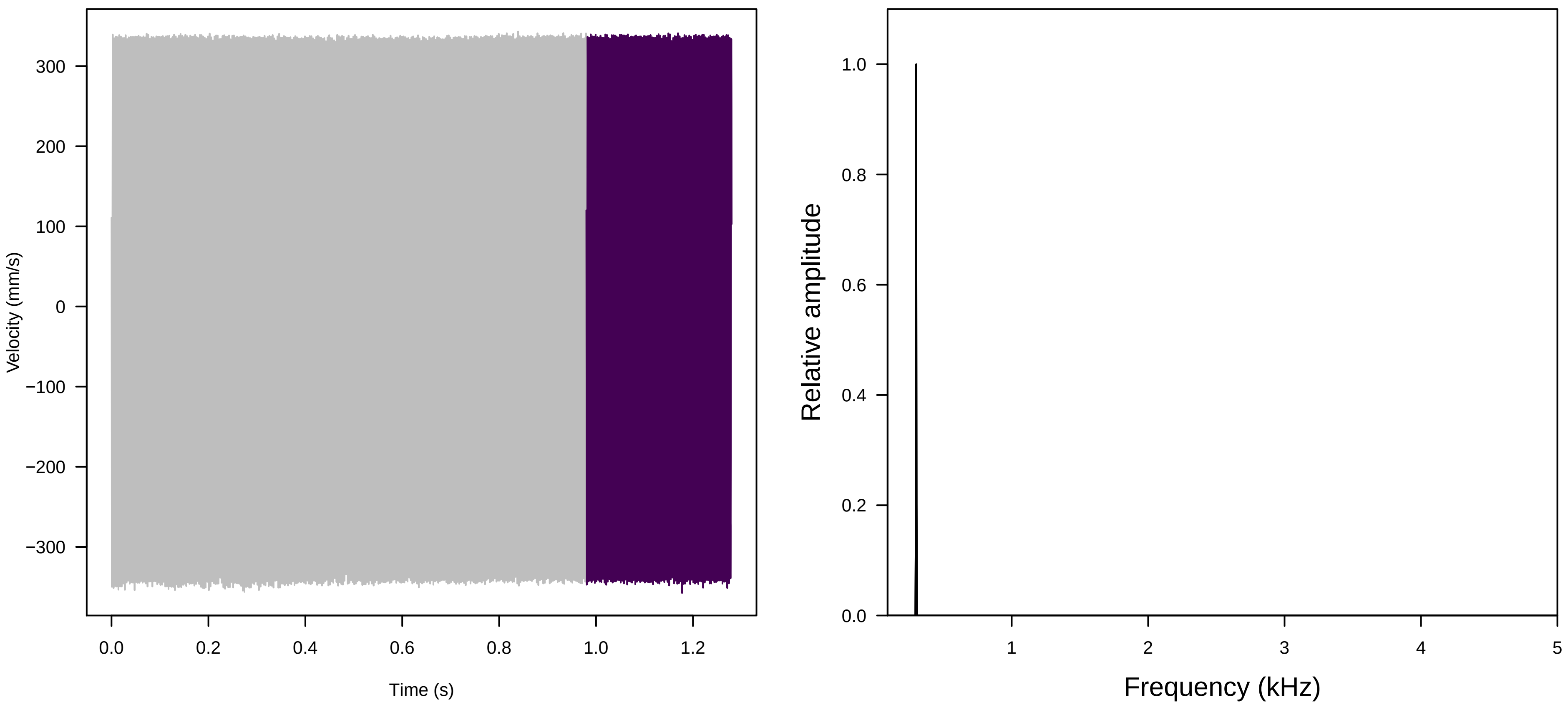

Vel. = 0.057 ; Str. = Receptacle ; Axis = z ; Fl. accession = 10−s−77−19

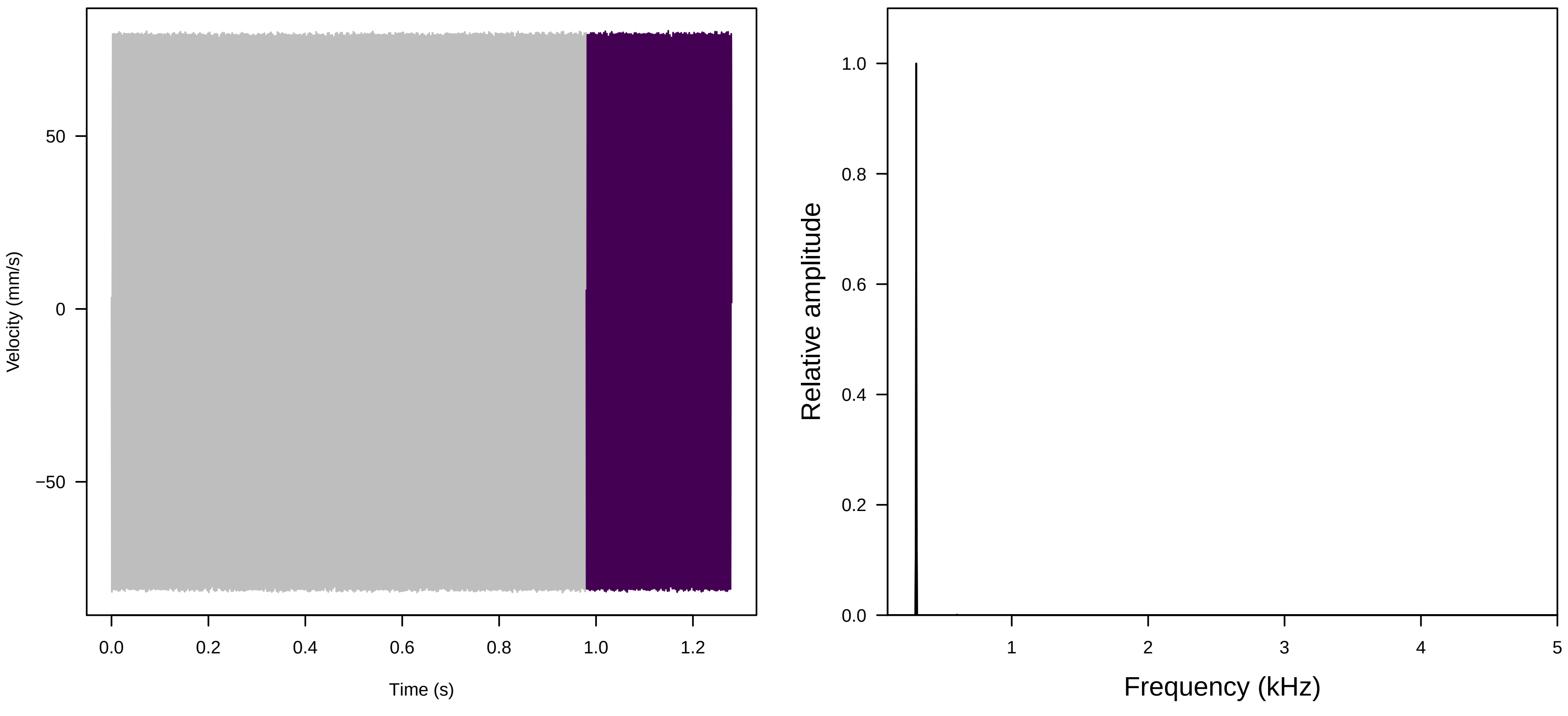

Vel. = 0.028 ; Str. = PA ; Axis = z ; Fl. accession = 10−s−77−19

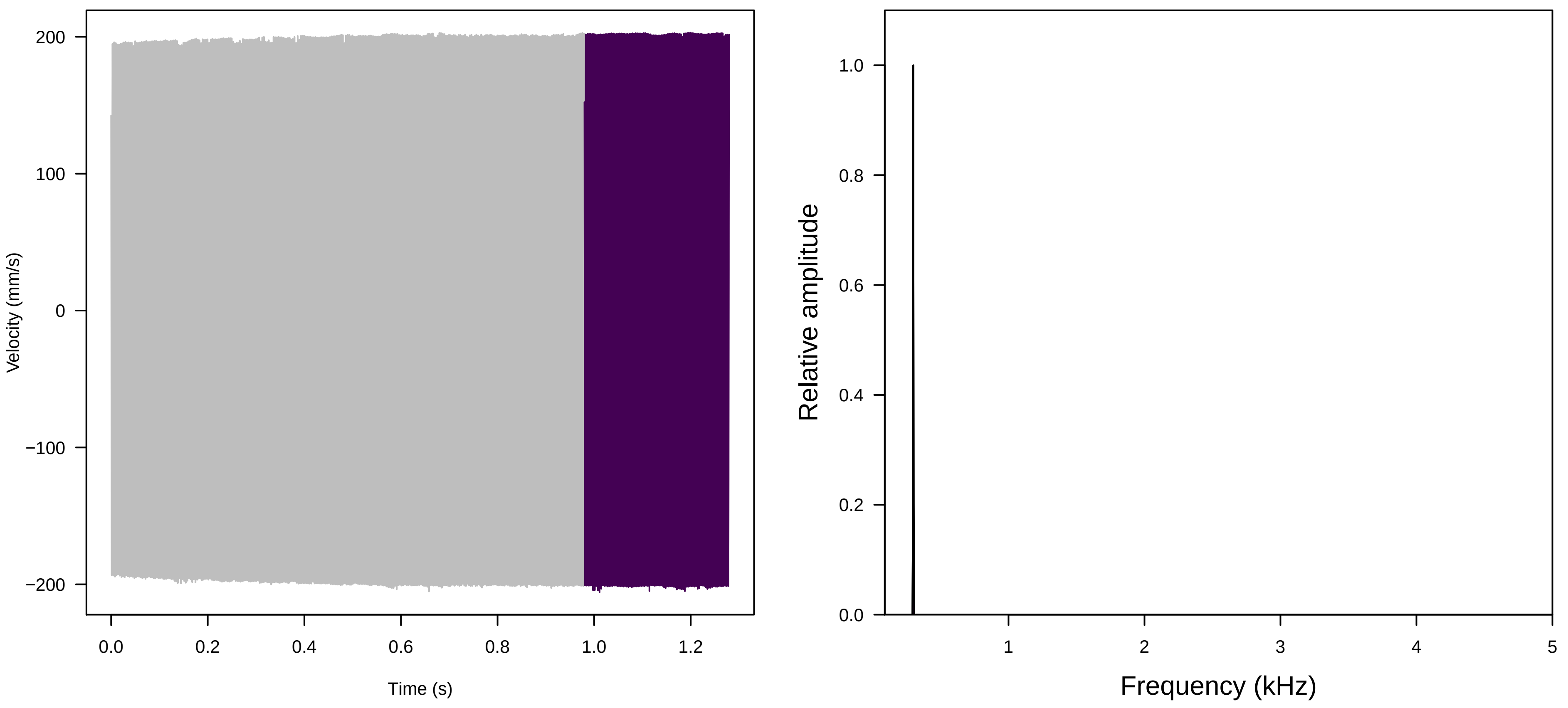

Vel. = 0.028 ; Str. = Receptacle ; Axis = z ; Fl. accession = 10−s−77−19

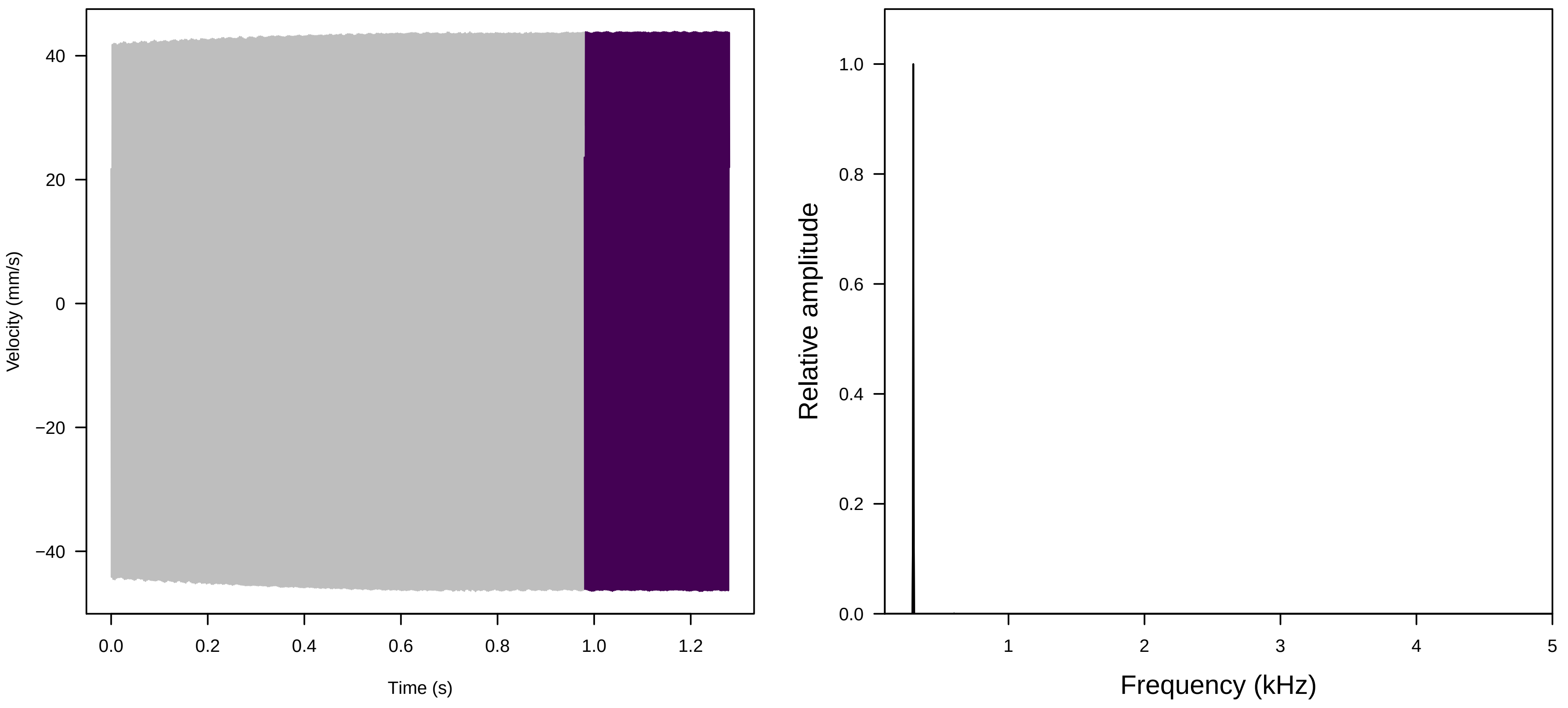

Vel. = 0.028 ; Str. = FA ; Axis = z ; Fl. accession = 10−s−77−19

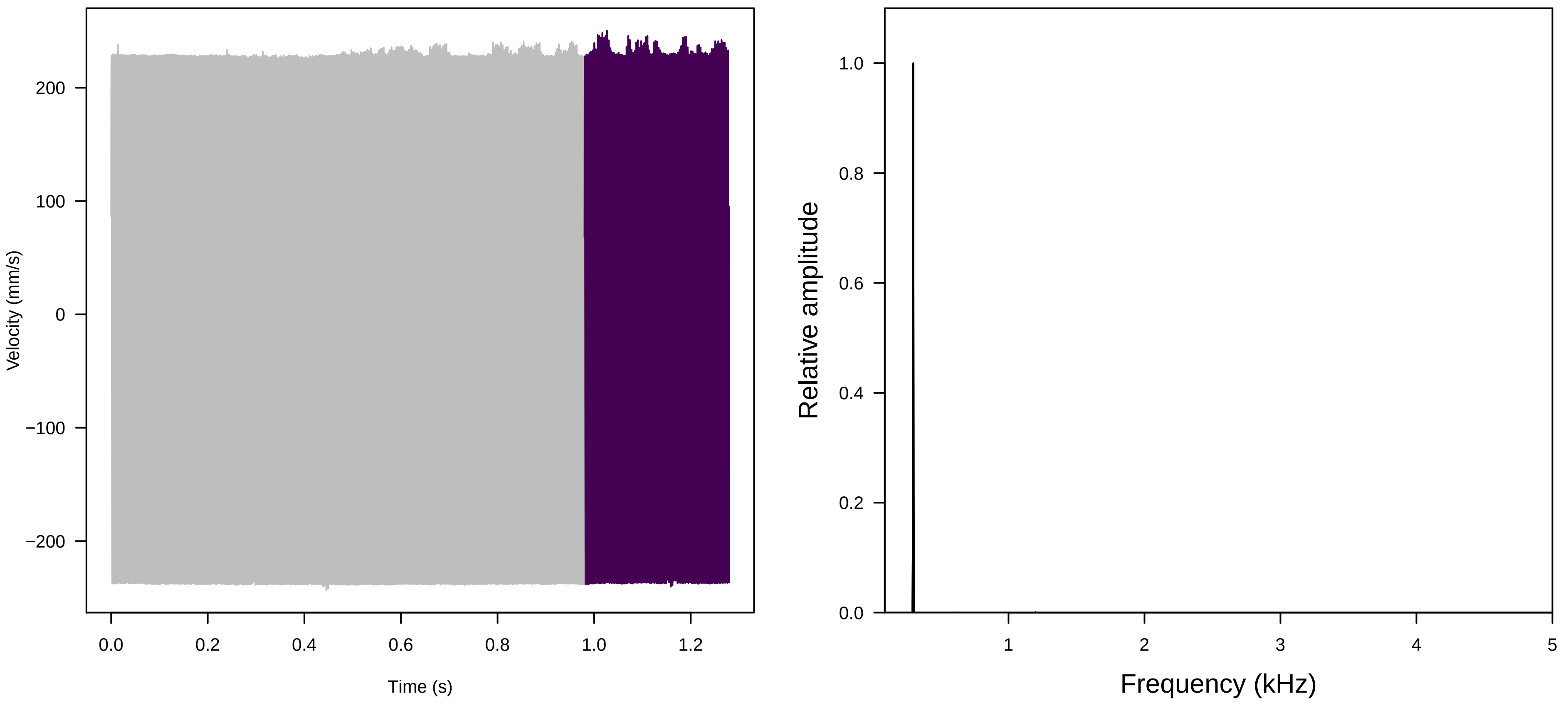

Vel. = 0.028 ; Str. = Receptacle ; Axis = z ; Fl. accession = 10−s−77−19

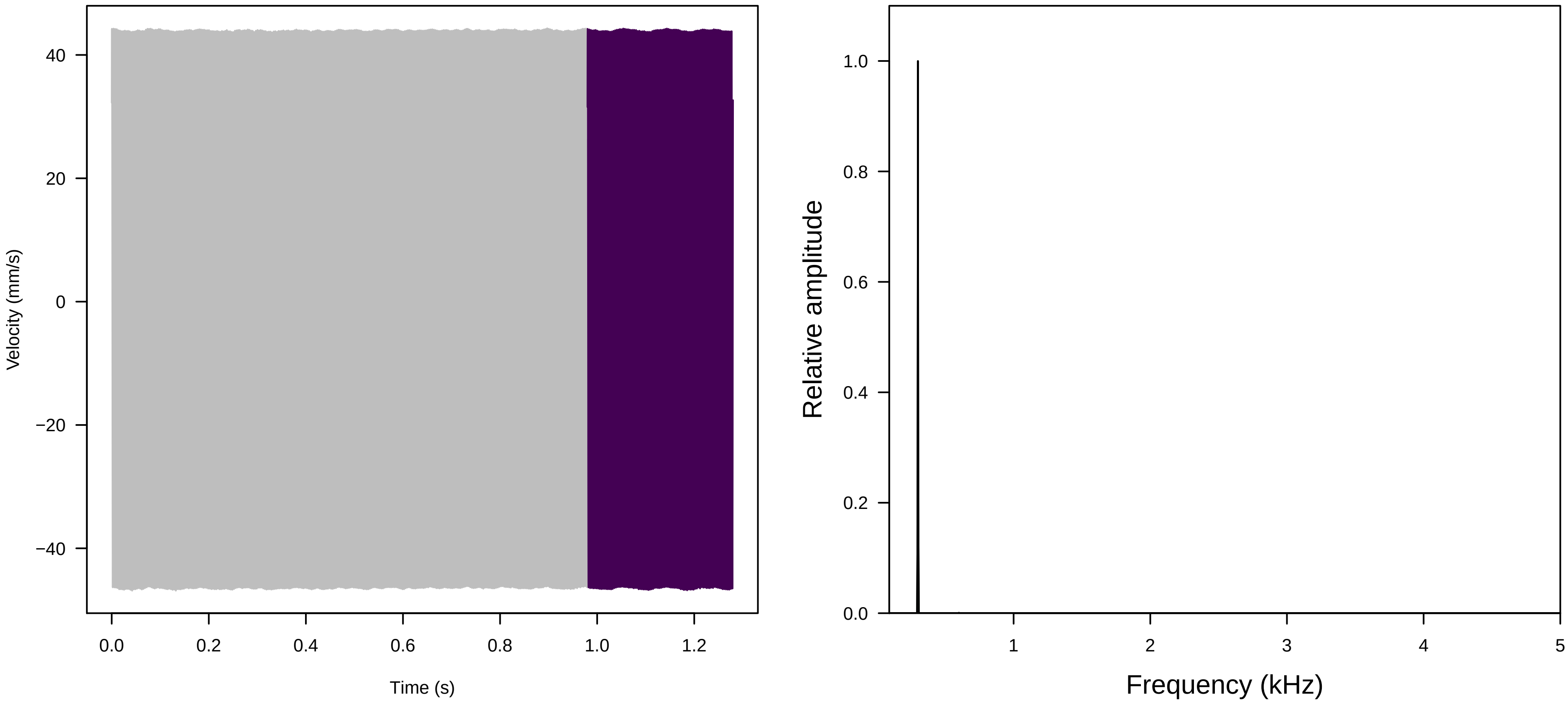

Vel. = 0.028 ; Str. = Corolla ; Axis = z ; Fl. accession = 10−s−77−19

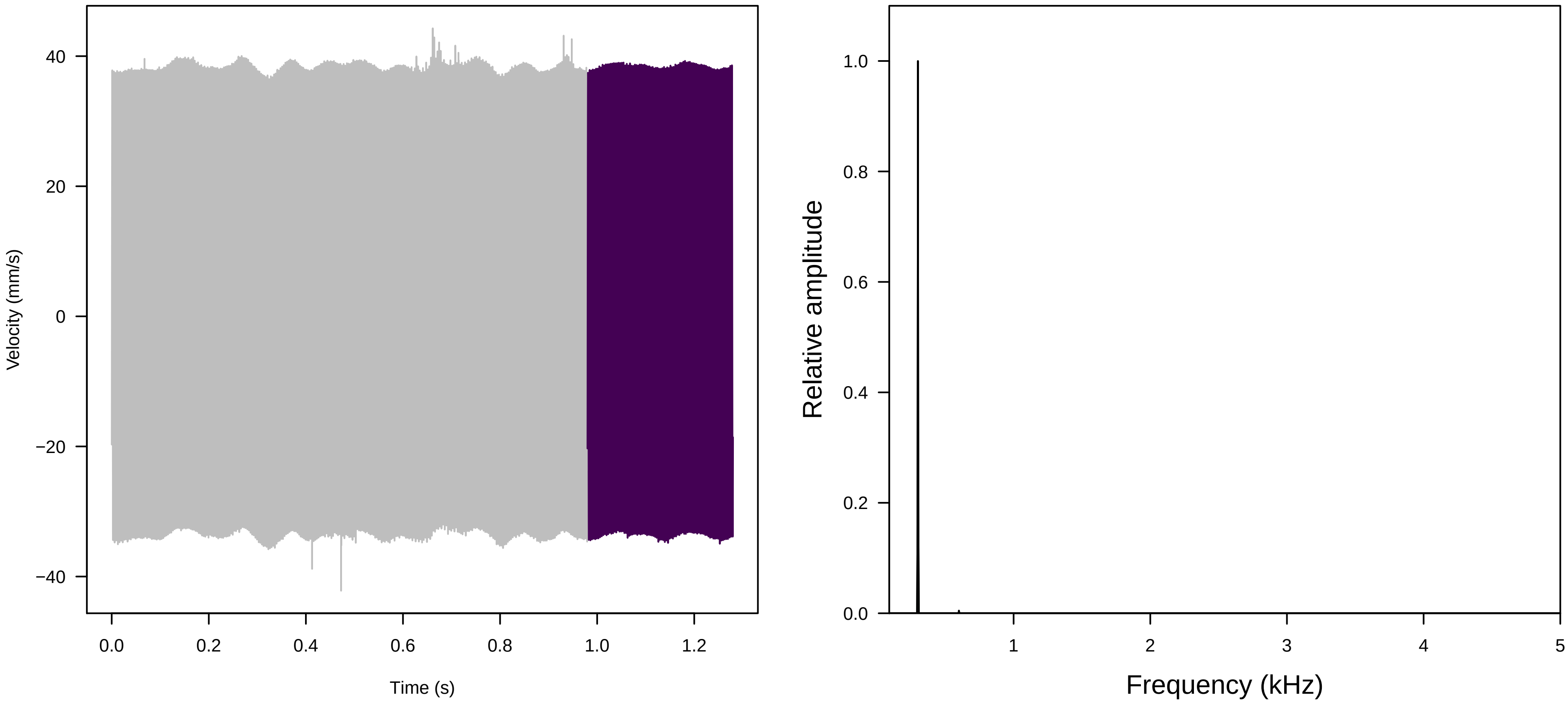

Vel. = 0.028 ; Str. = Receptacle ; Axis = z ; Fl. accession = 10−s−77−19

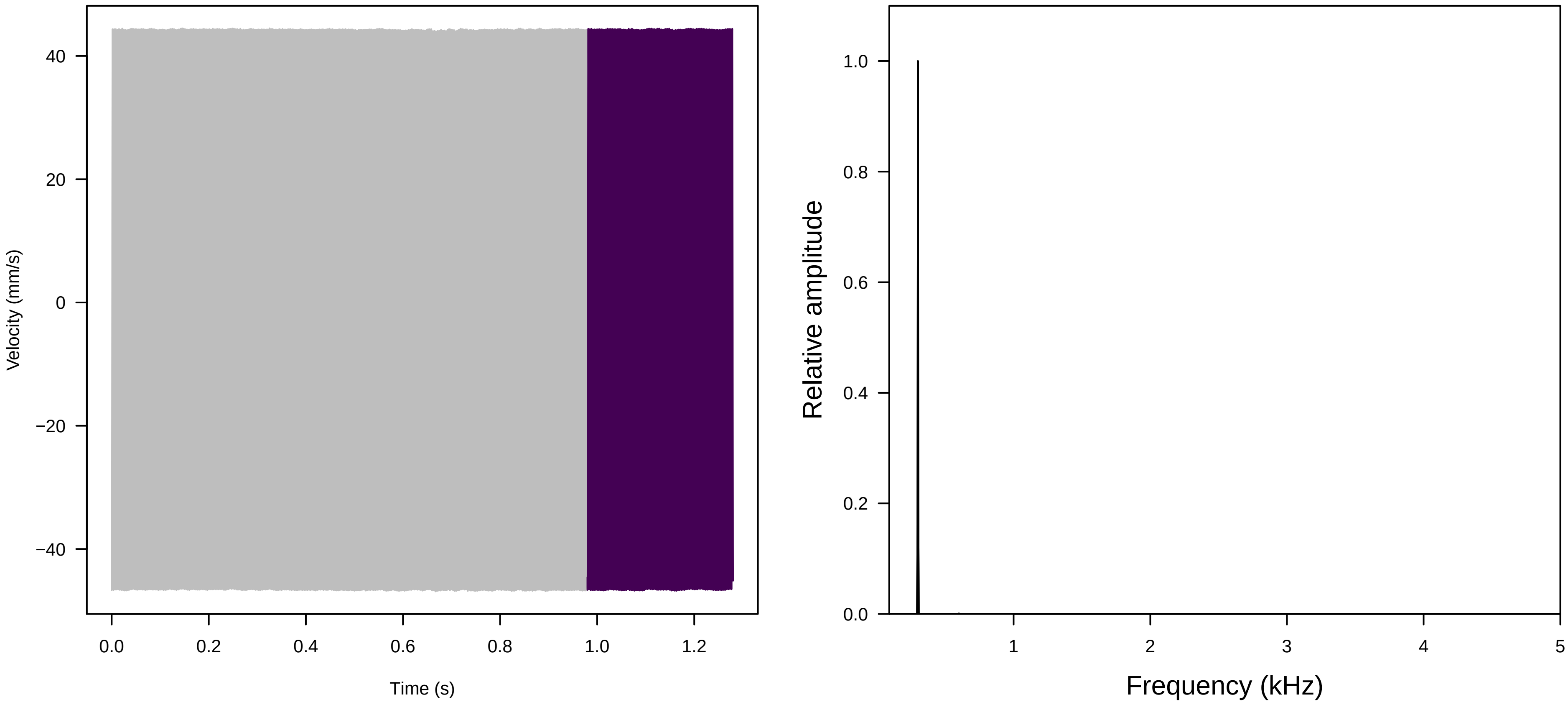

Vel. = 0.014 ; Str. = Corolla ; Axis = z ; Fl. accession = 10−s−77−19

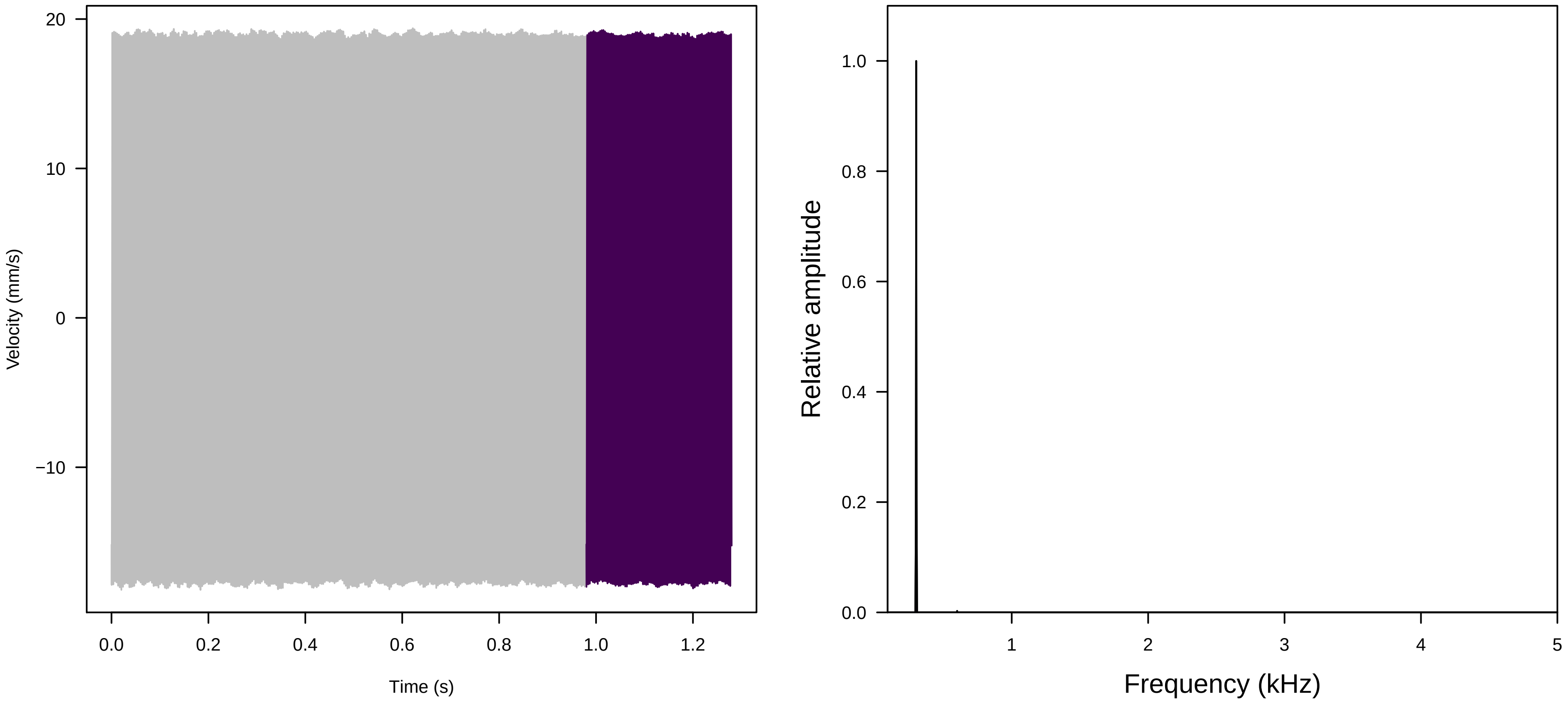

Vel. = 0.014 ; Str. = Receptacle ; Axis = z ; Fl. accession = 10−s−77−19

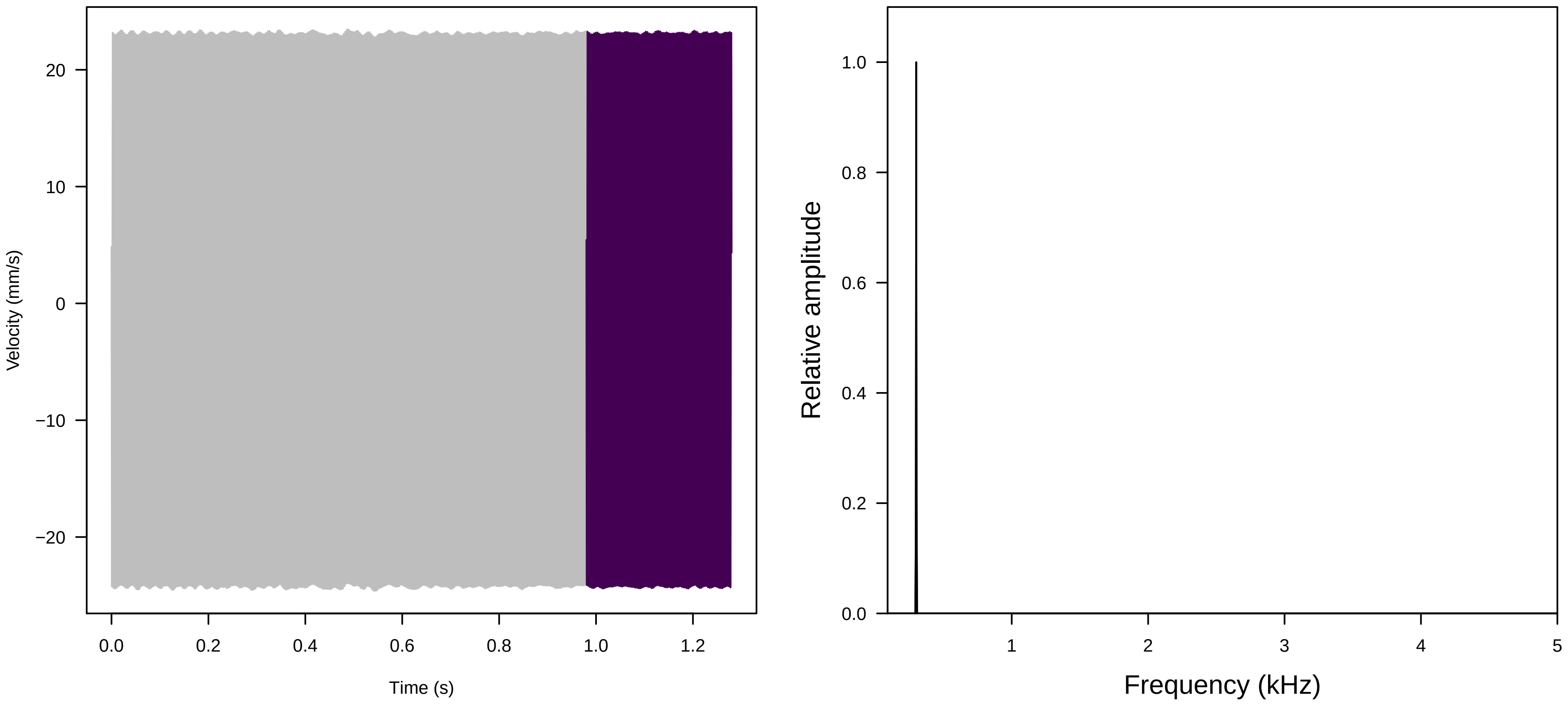

Vel. = 0.014 ; Str. = FA ; Axis = z ; Fl. accession = 10−s−77−19

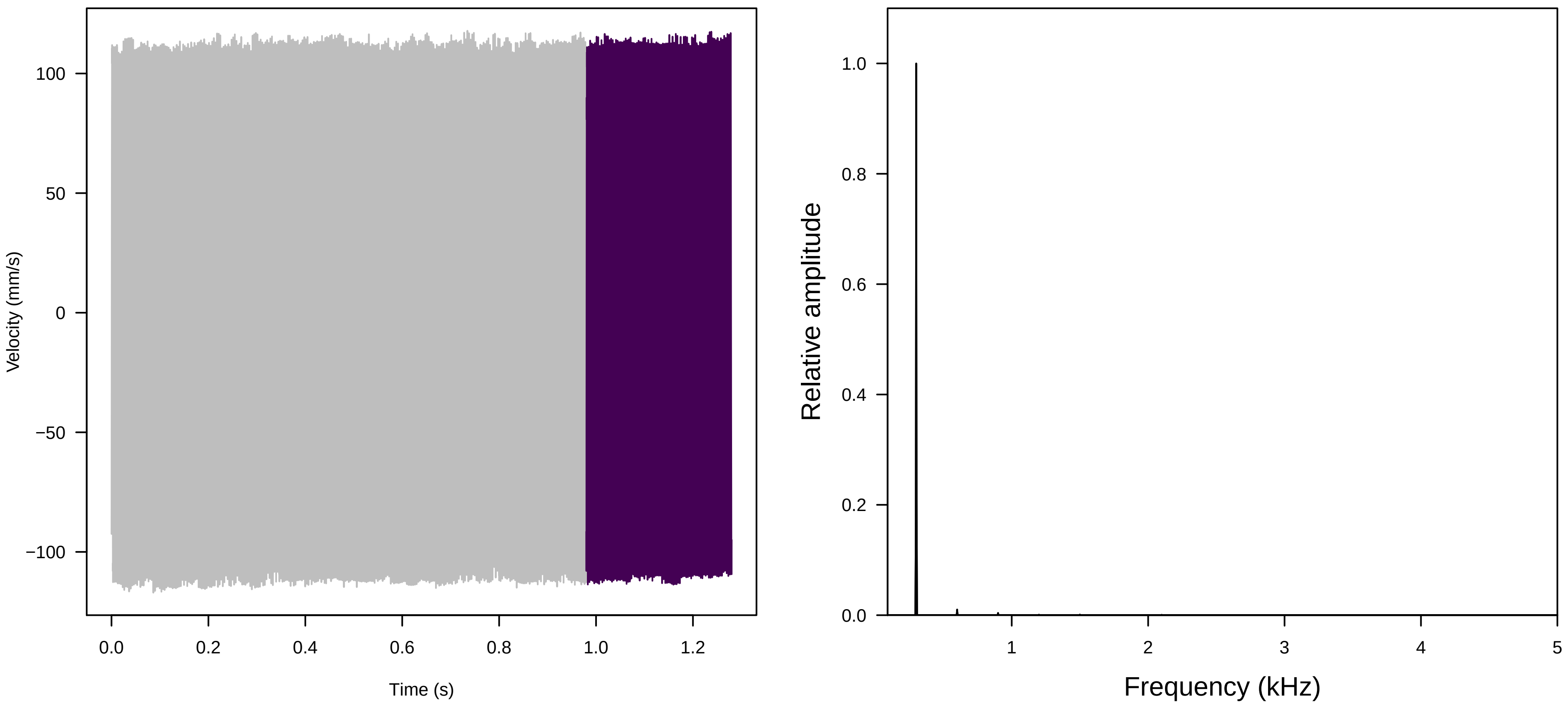

Vel. = 0.014 ; Str. = Receptacle ; Axis = z ; Fl. accession = 10−s−77−19

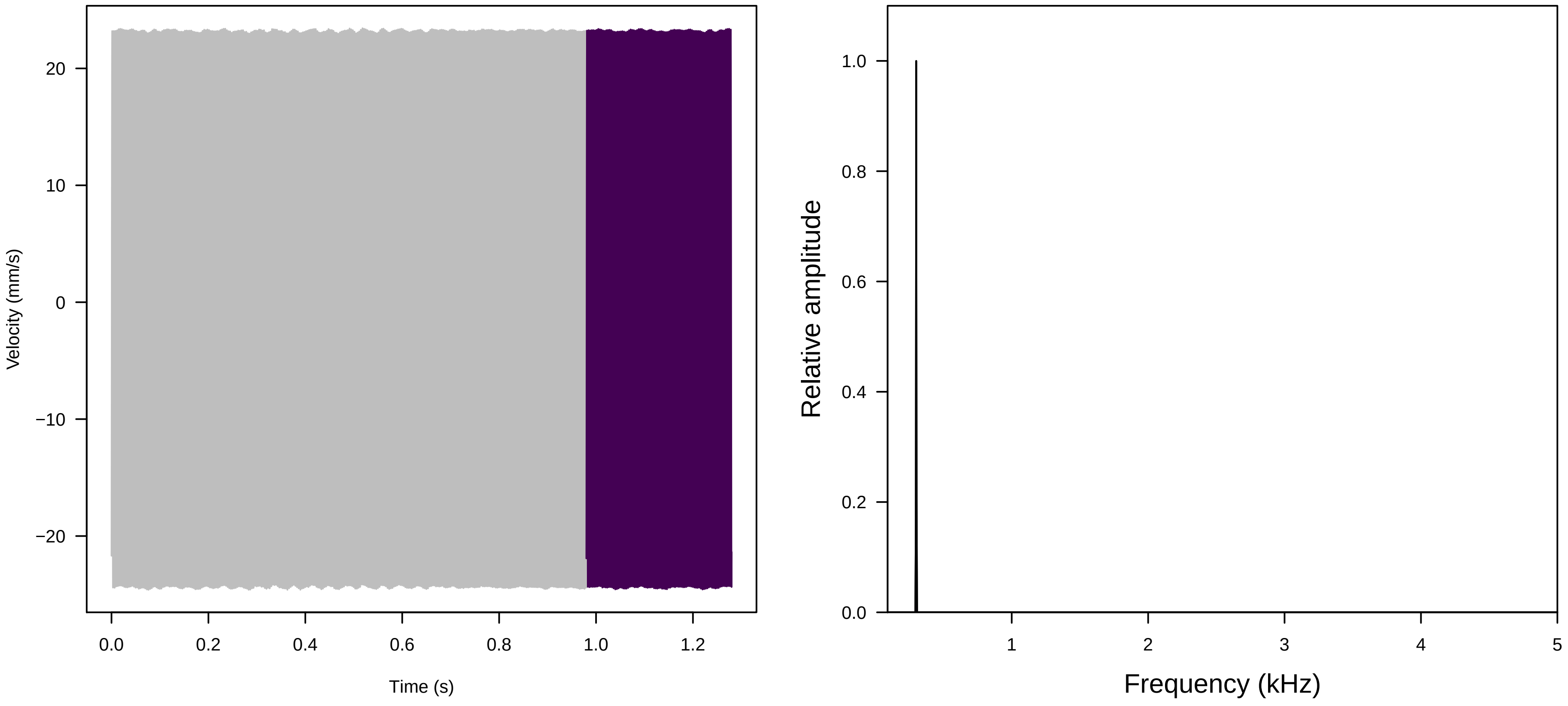

Vel. = 0.014 ; Str. = PA ; Axis = z ; Fl. accession = 10−s−77−19

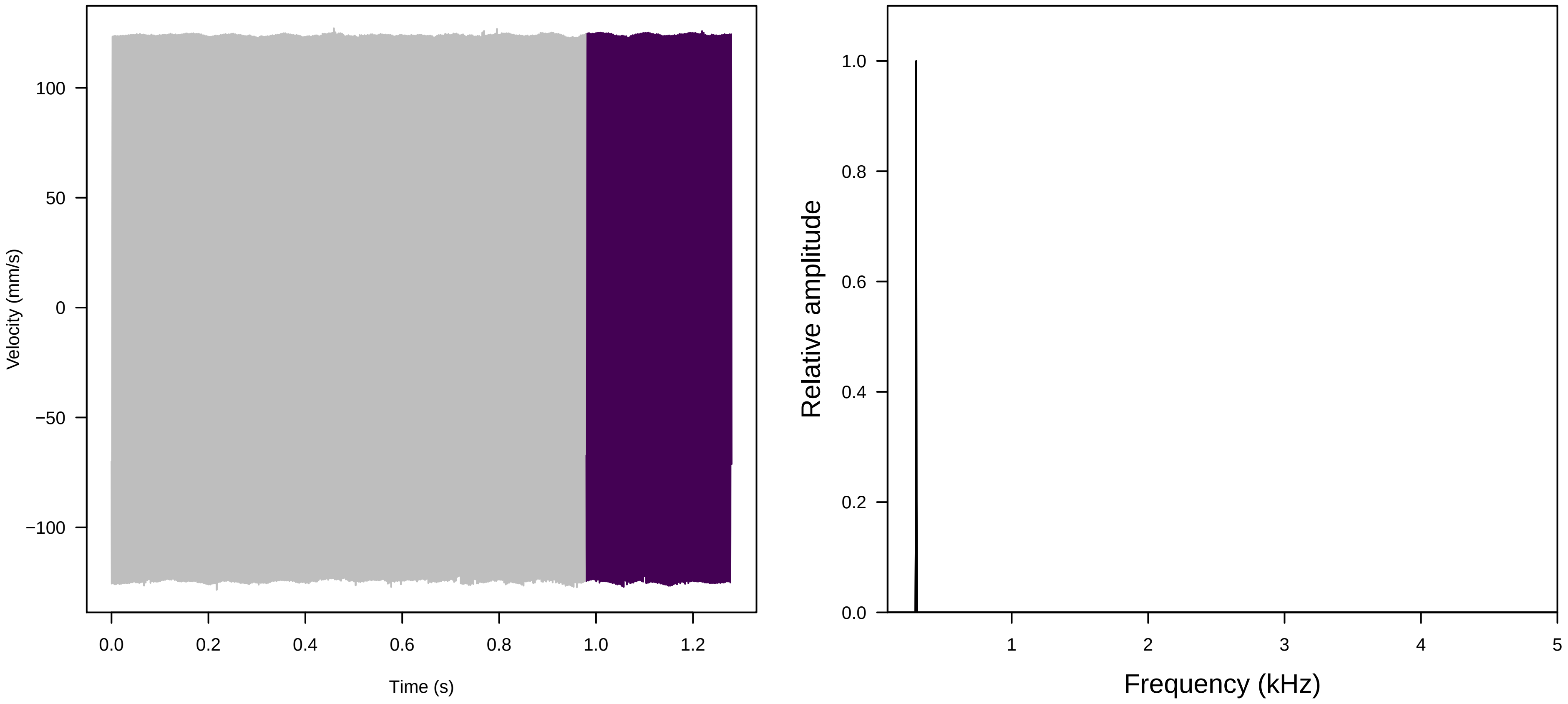

Vel. = 0.014 ; Str. = Receptacle ; Axis = z ; Fl. accession = 10−s−77−19

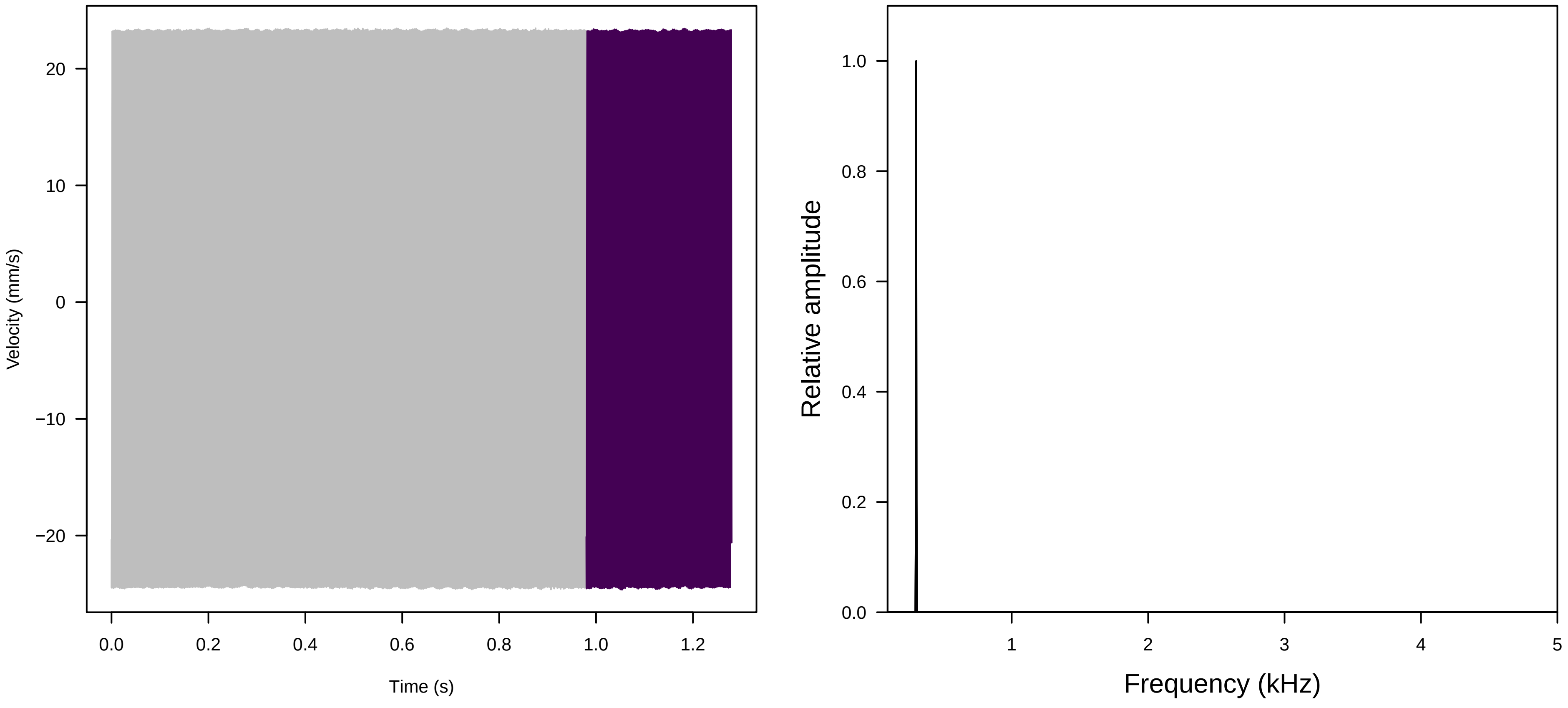

Vel. = 0.014 ; Str. = Corolla ; Axis = y ; Fl. accession = 10−s−77−19

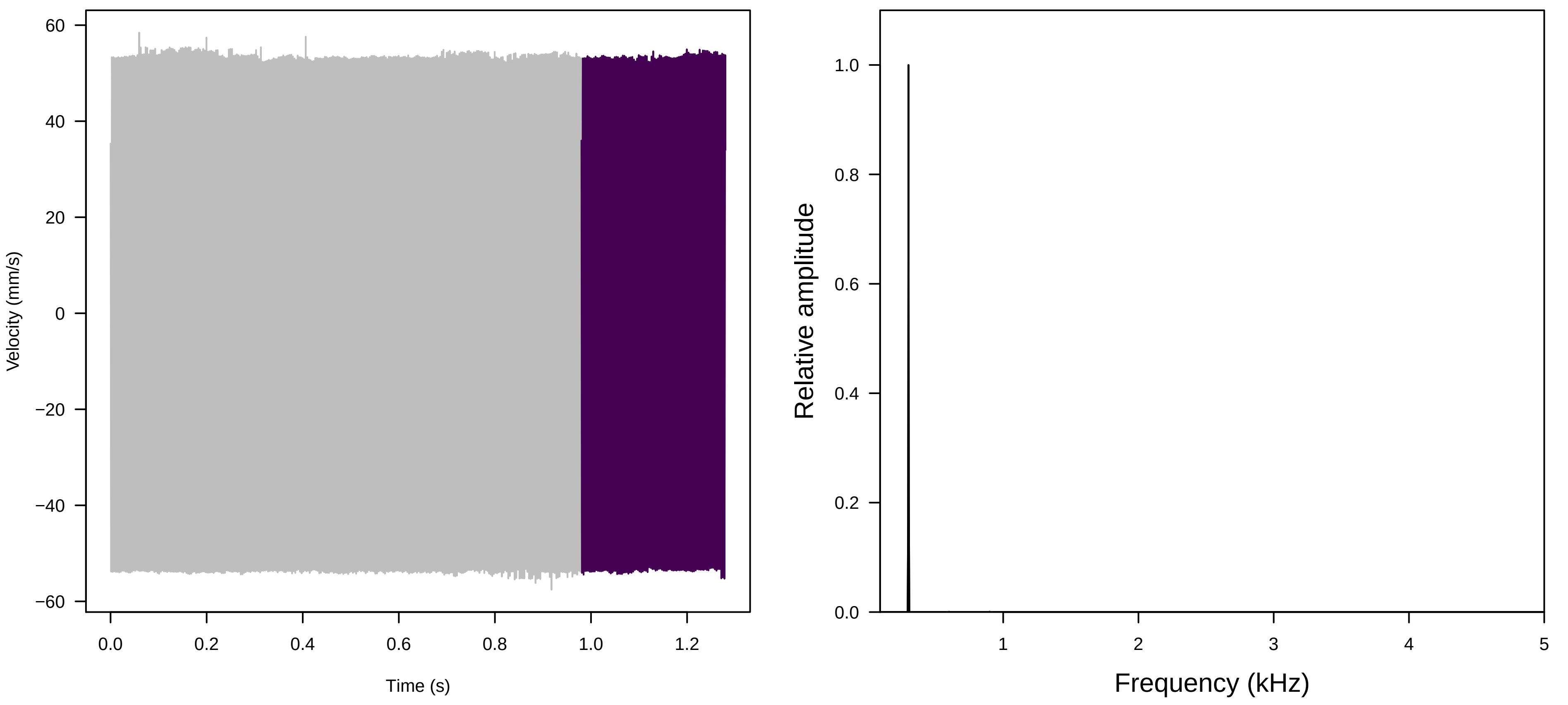

Vel. = 0.014 ; Str. = Receptacle ; Axis = y ; Fl. accession = 10−s−77−19

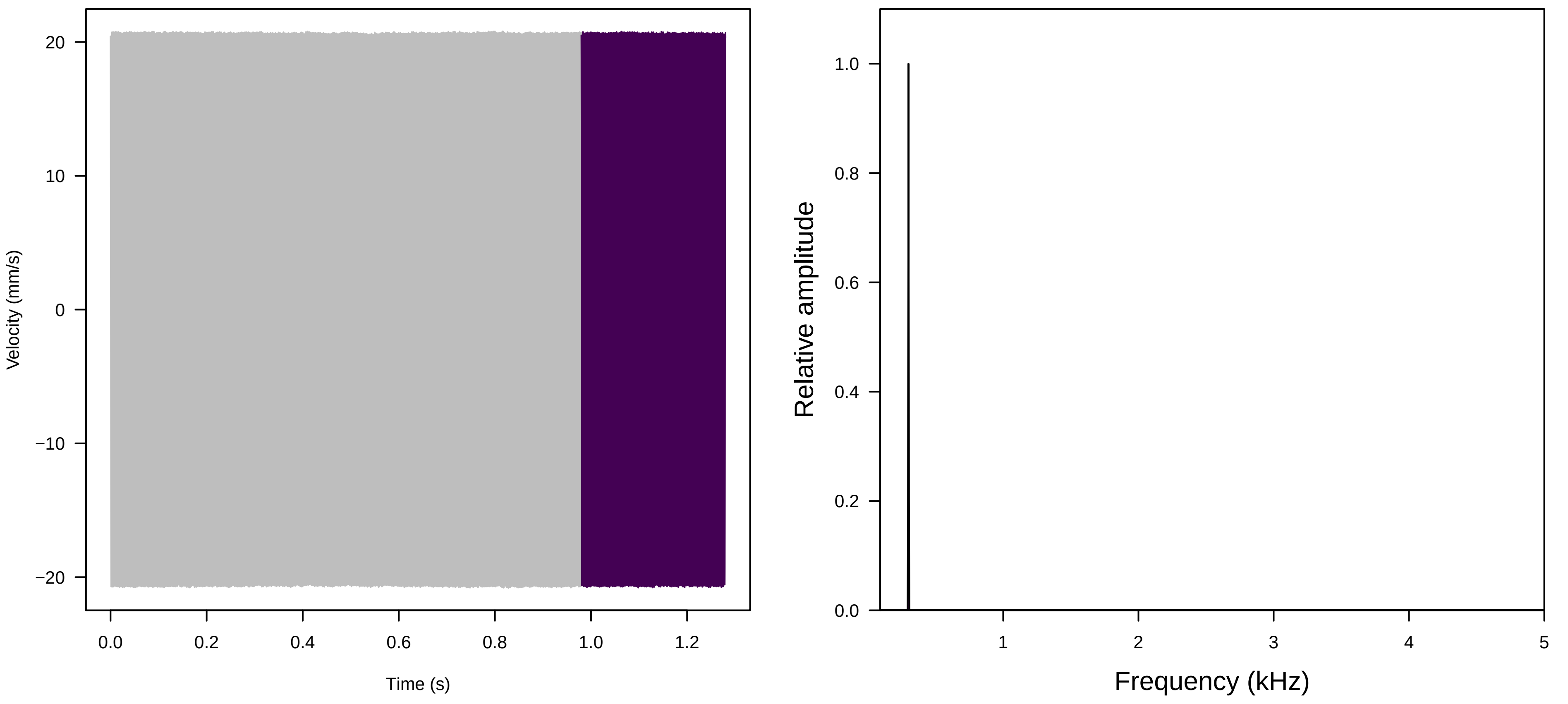

Vel. = 0.014 ; Str. = FA ; Axis = y ; Fl. accession = 10−s−77−19

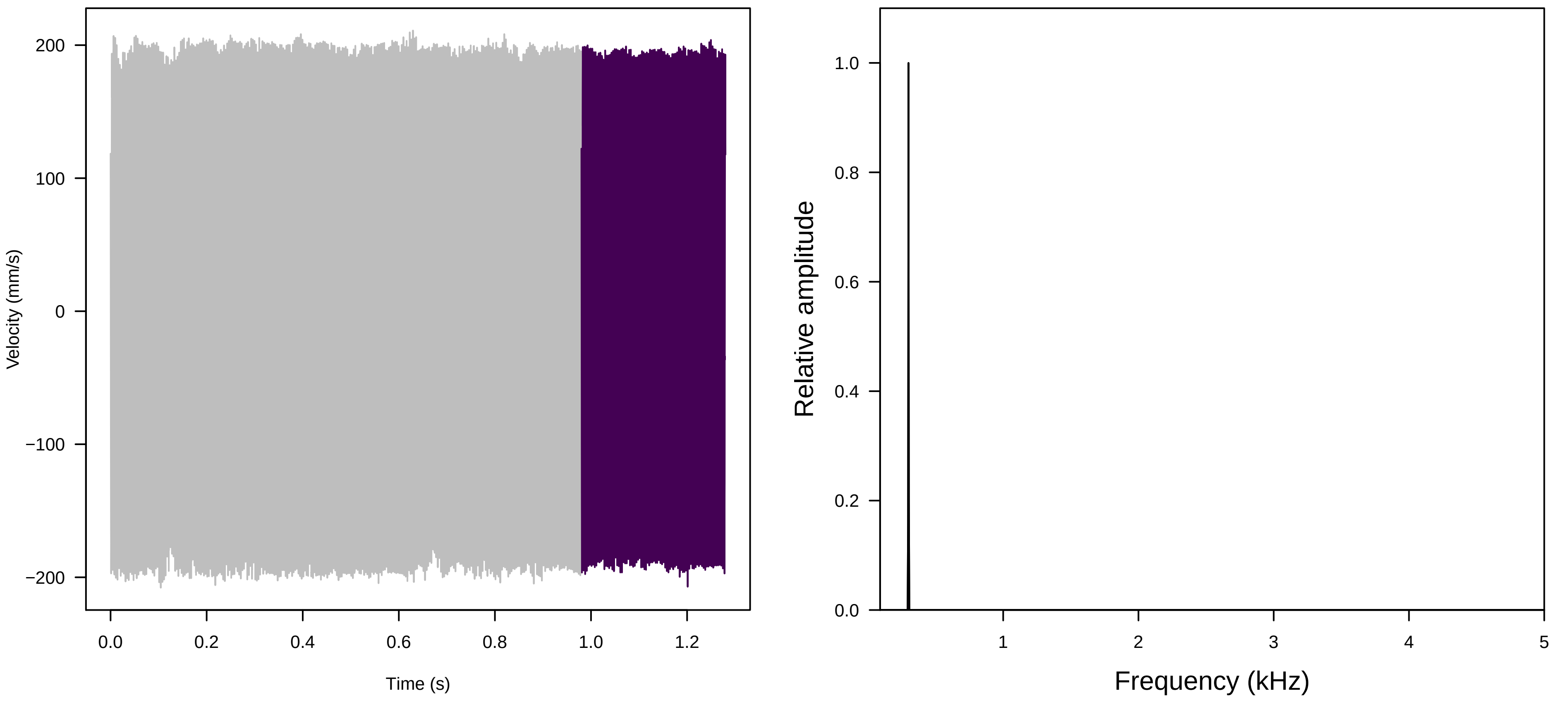

Vel. = 0.014 ; Str. = Receptacle ; Axis = y ; Fl. accession = 10−s−77−19

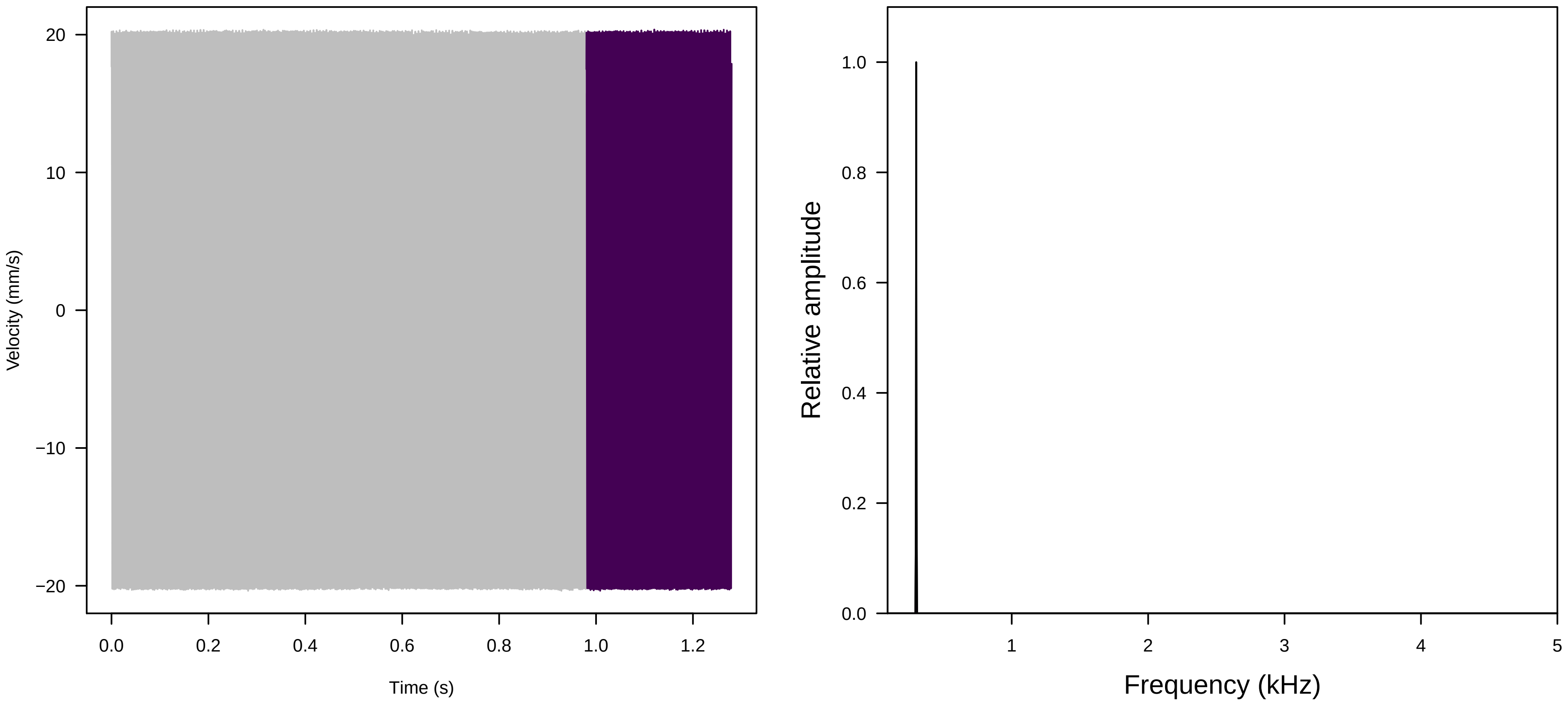

Vel. = 0.014 ; Str. = PA ; Axis = y ; Fl. accession = 10−s−77−19

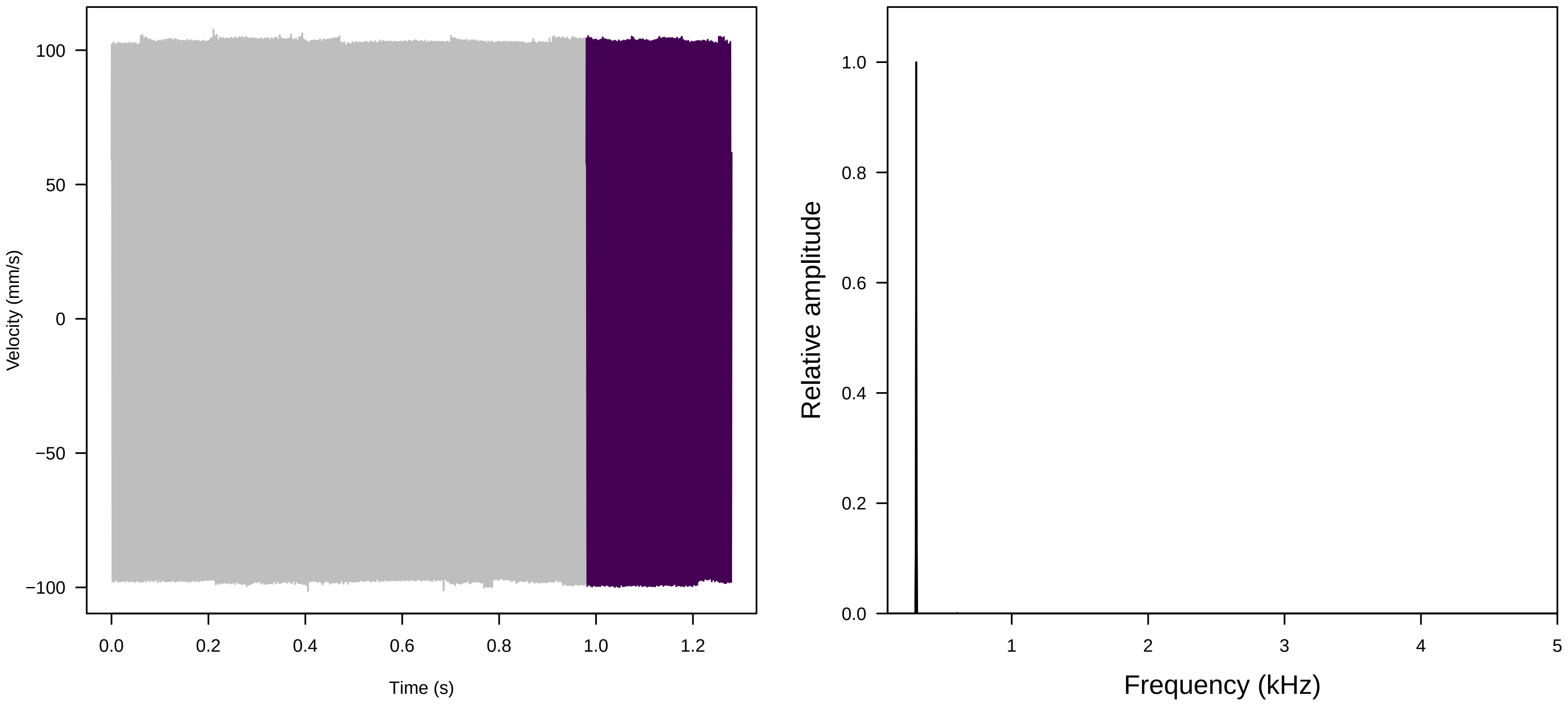

Vel. = 0.014 ; Str. = Receptacle ; Axis = y ; Fl. accession = 10−s−77−19

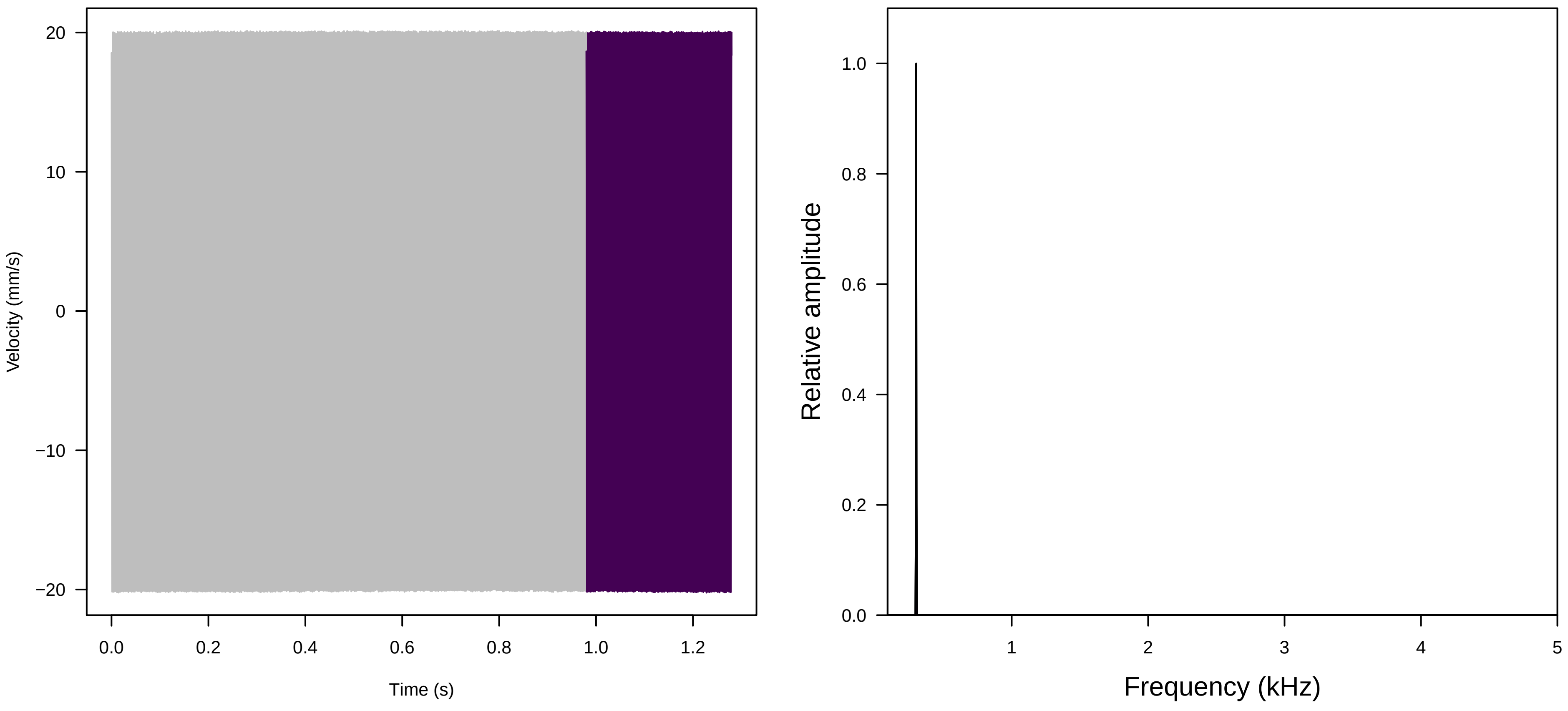

Vel. = 0.028 ; Str. = PA ; Axis = y ; Fl. accession = 10−s−77−19

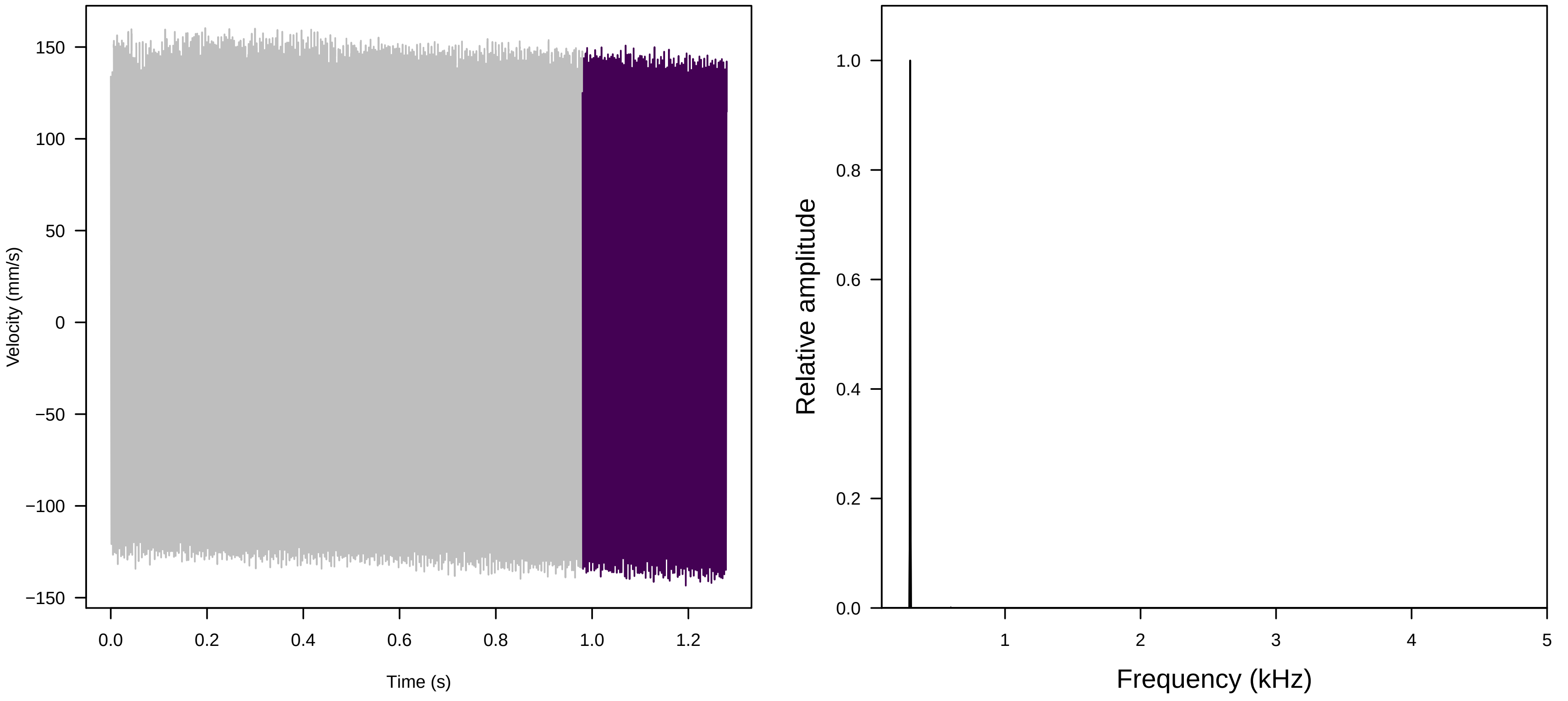

Vel. = 0.028 ; Str. = Receptacle ; Axis = y ; Fl. accession = 10−s−77−19

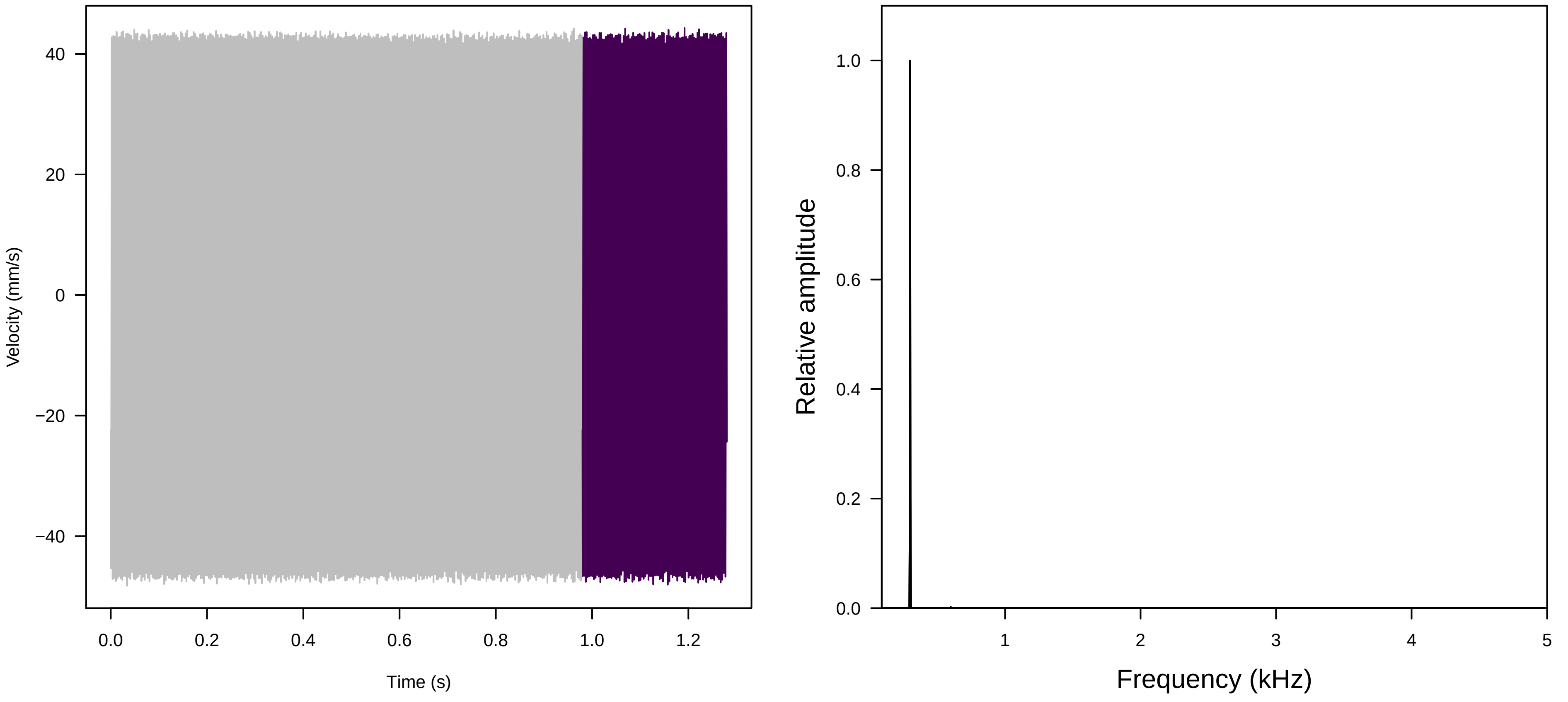

Vel. = 0.028 ; Str. = FA ; Axis = y ; Fl. accession = 10−s−77−19

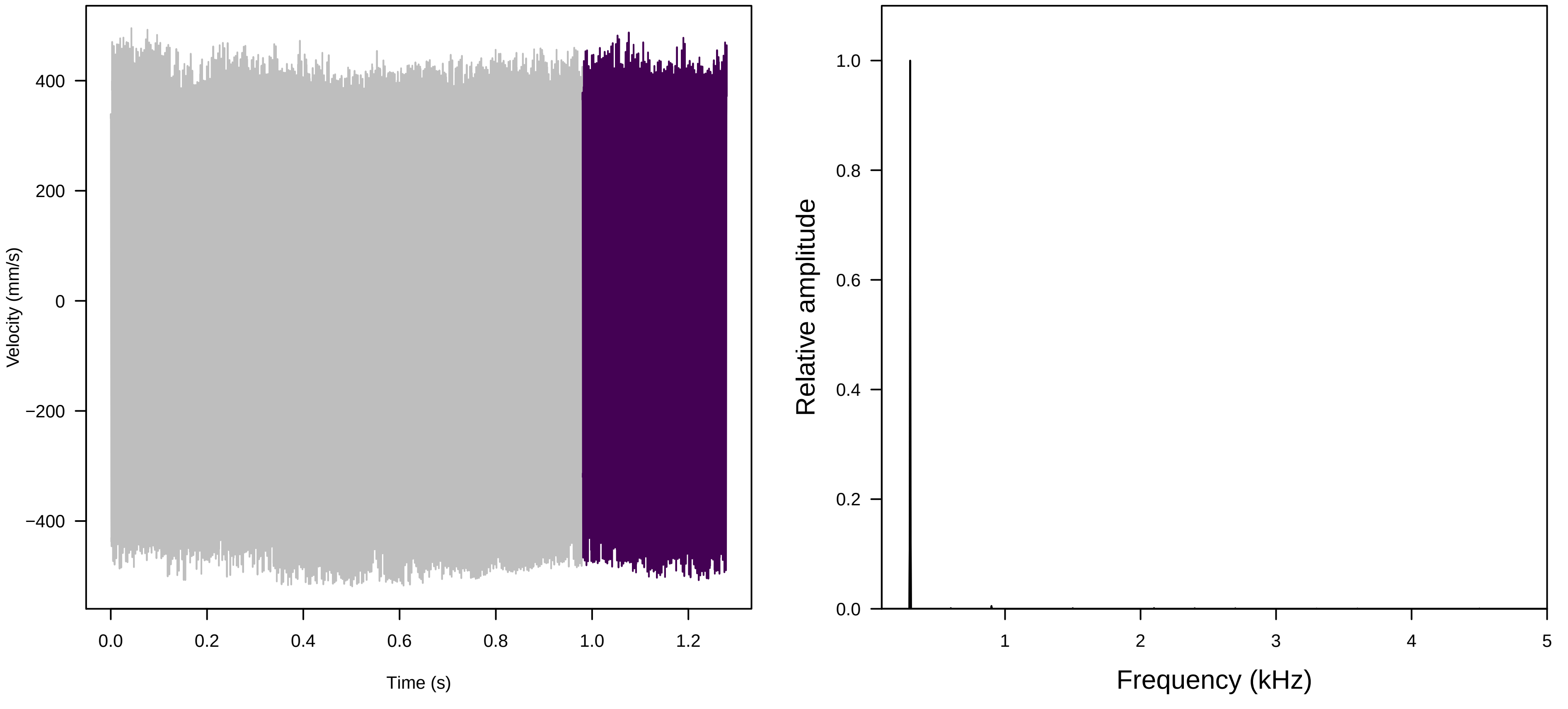

Vel. = 0.028 ; Str. = Receptacle ; Axis = y ; Fl. accession = 10−s−77−19

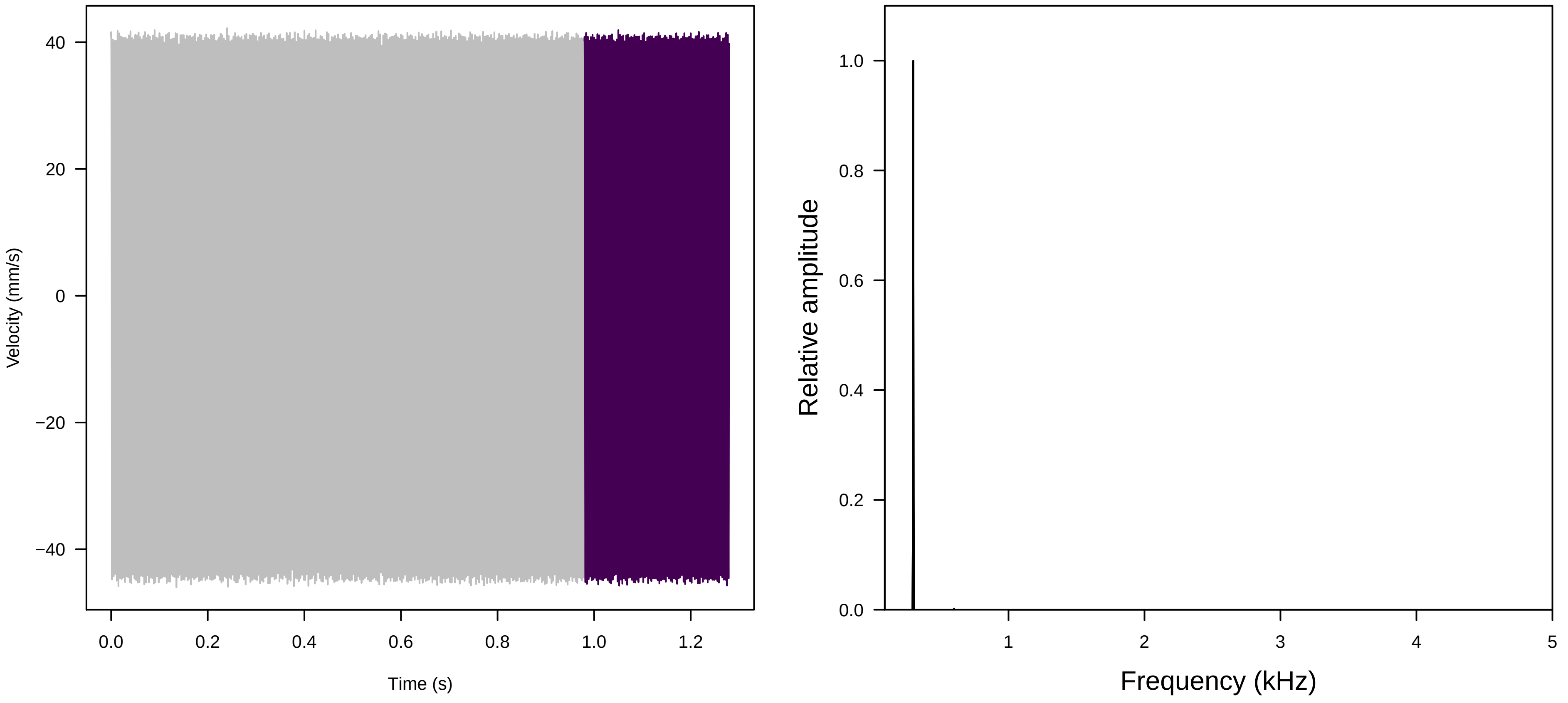

Vel. = 0.028 ; Str. = Corolla ; Axis = y ; Fl. accession = 10−s−77−19

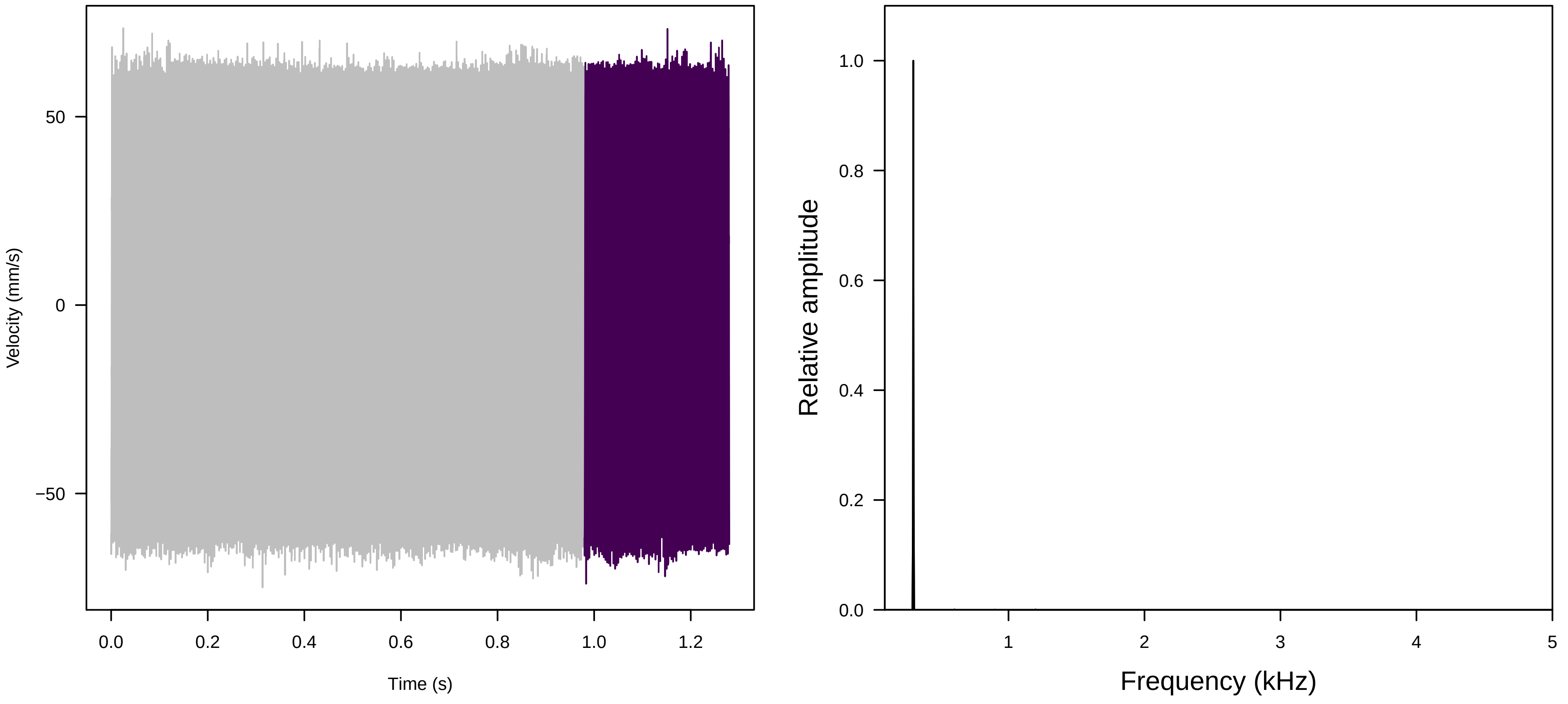

Vel. = 0.028 ; Str. = Receptacle ; Axis = y ; Fl. accession = 10−s−77−19

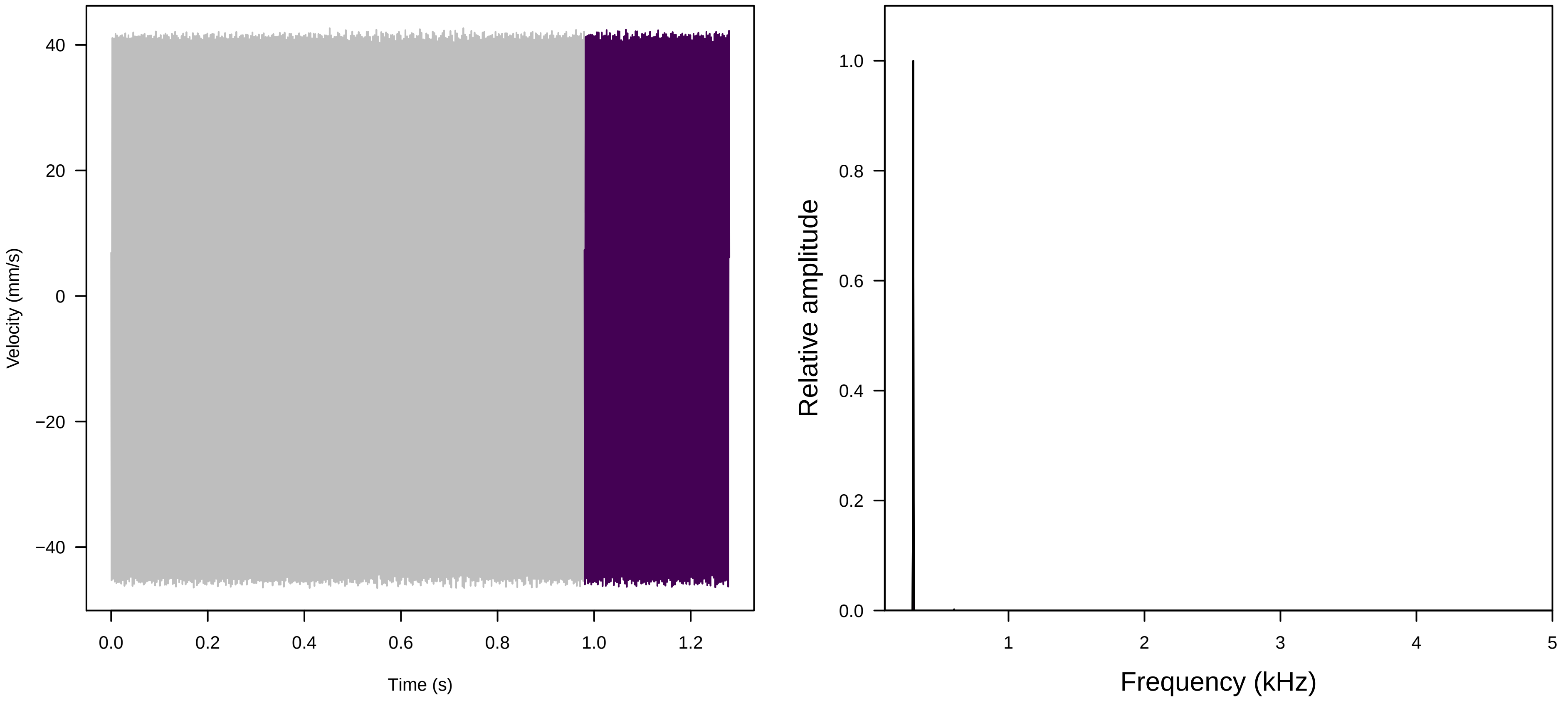

Vel. = 0.057 ; Str. = Corolla ; Axis = y ; Fl. accession = 10−s−77−19

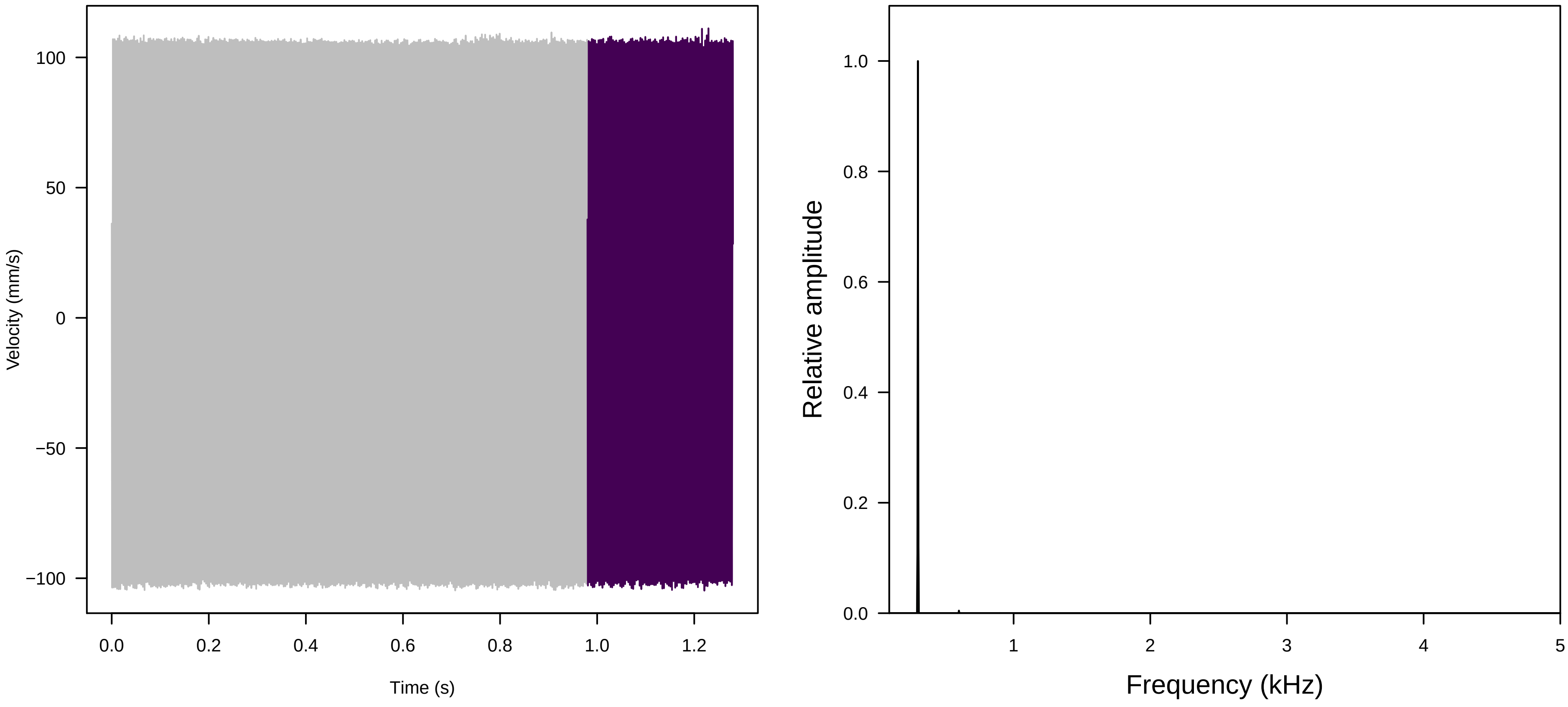

Vel. = 0.057 ; Str. = Receptacle ; Axis = y ; Fl. accession = 10−s−77−19

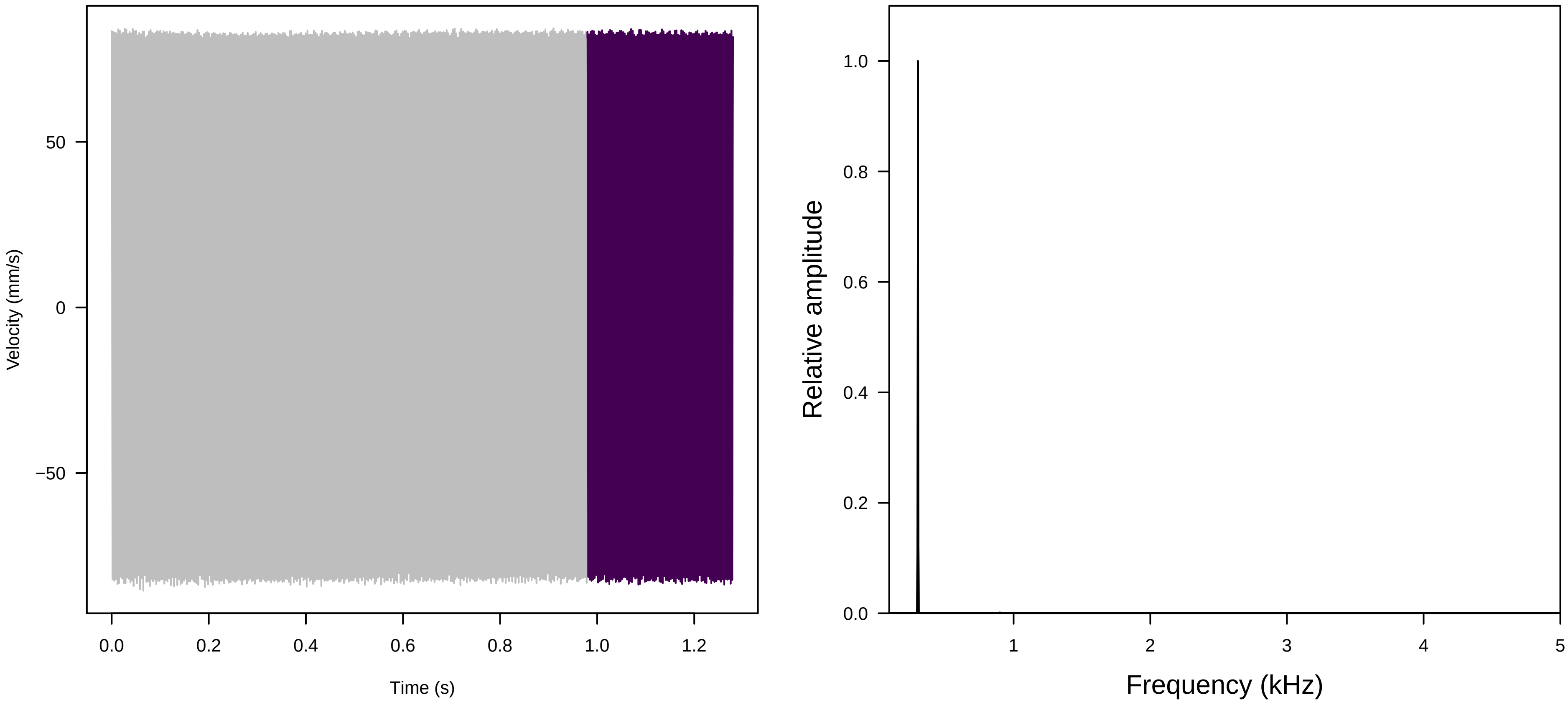

Vel. = 0.057 ; Str. = Receptacle ; Axis = y ; Fl. accession = 10−s−77−19

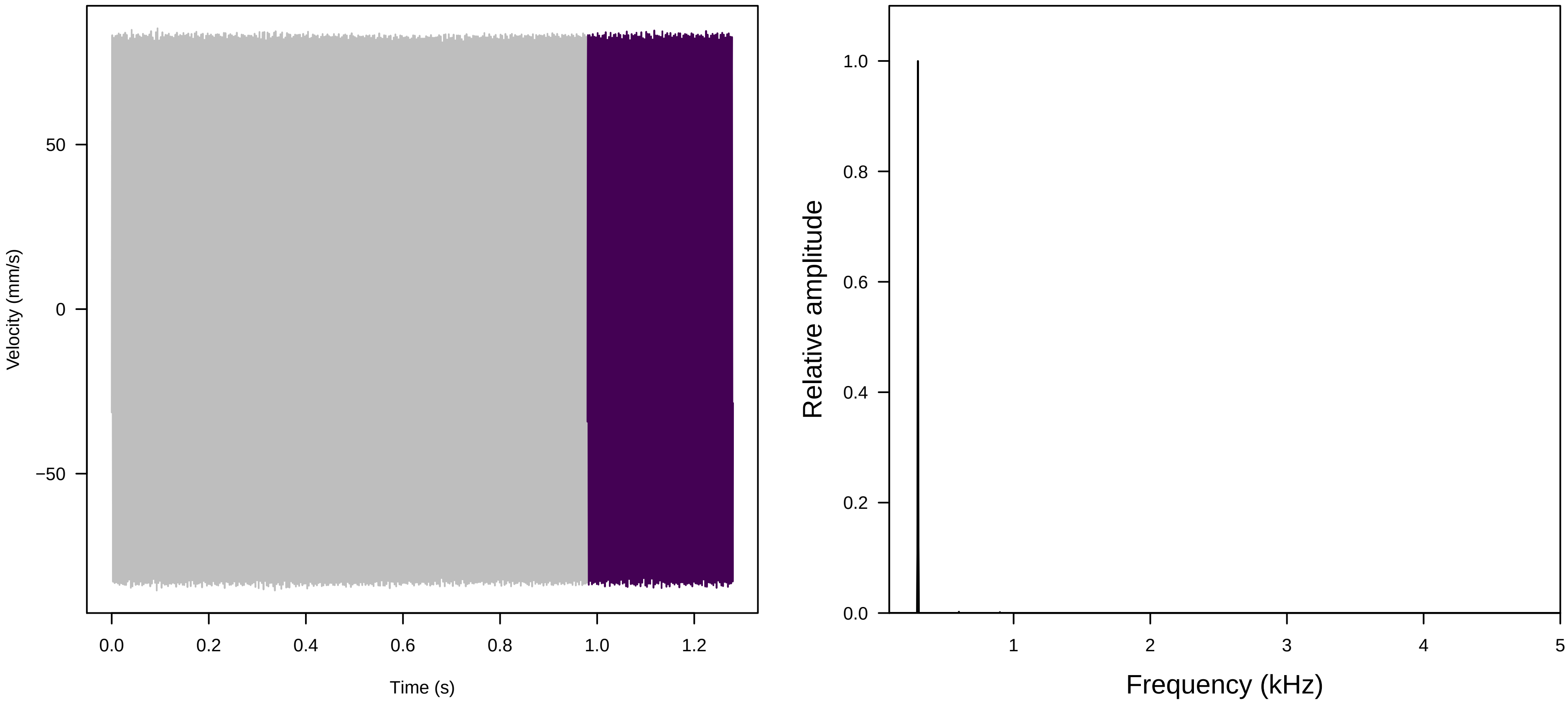

Vel. = 0.057 ; Str. = PA ; Axis = y ; Fl. accession = 10−s−77−19

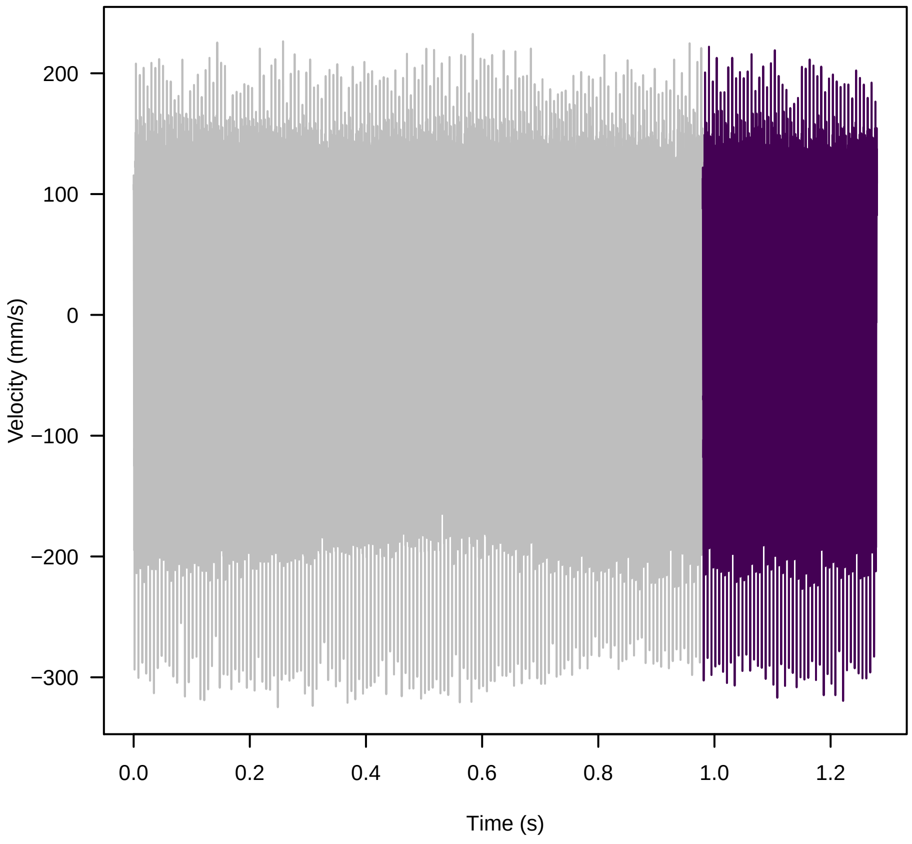

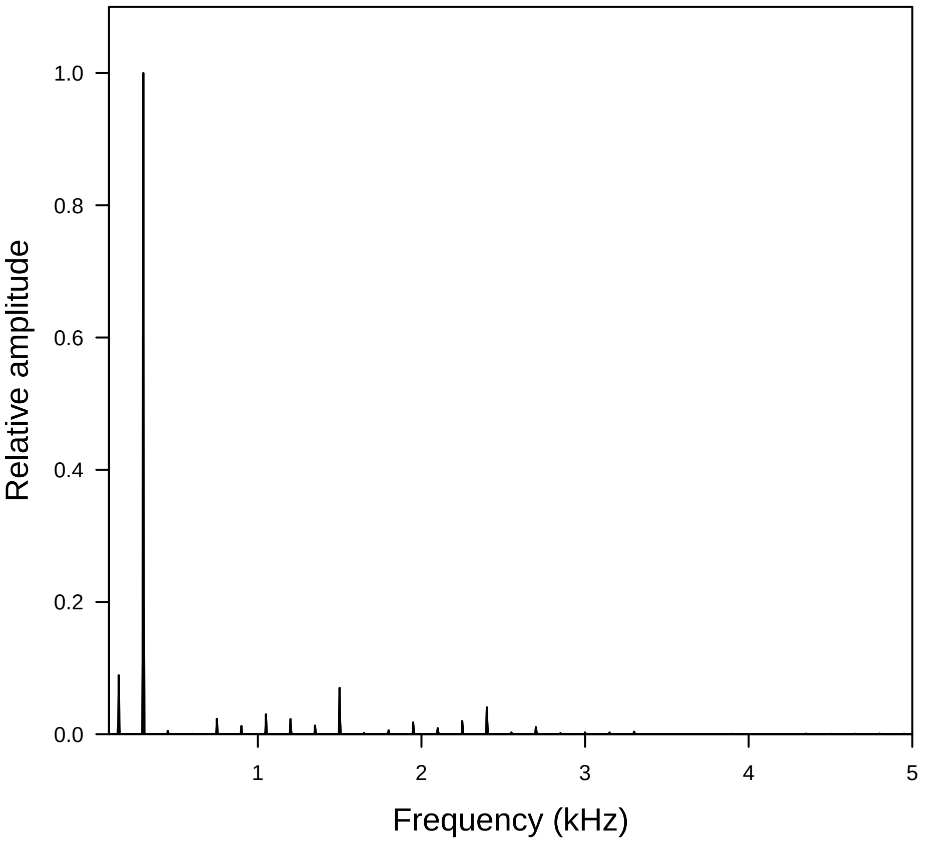

Vel. = 0.057 ; Str. = Receptacle ; Axis = y ; Fl. accession = 10−s−77−19

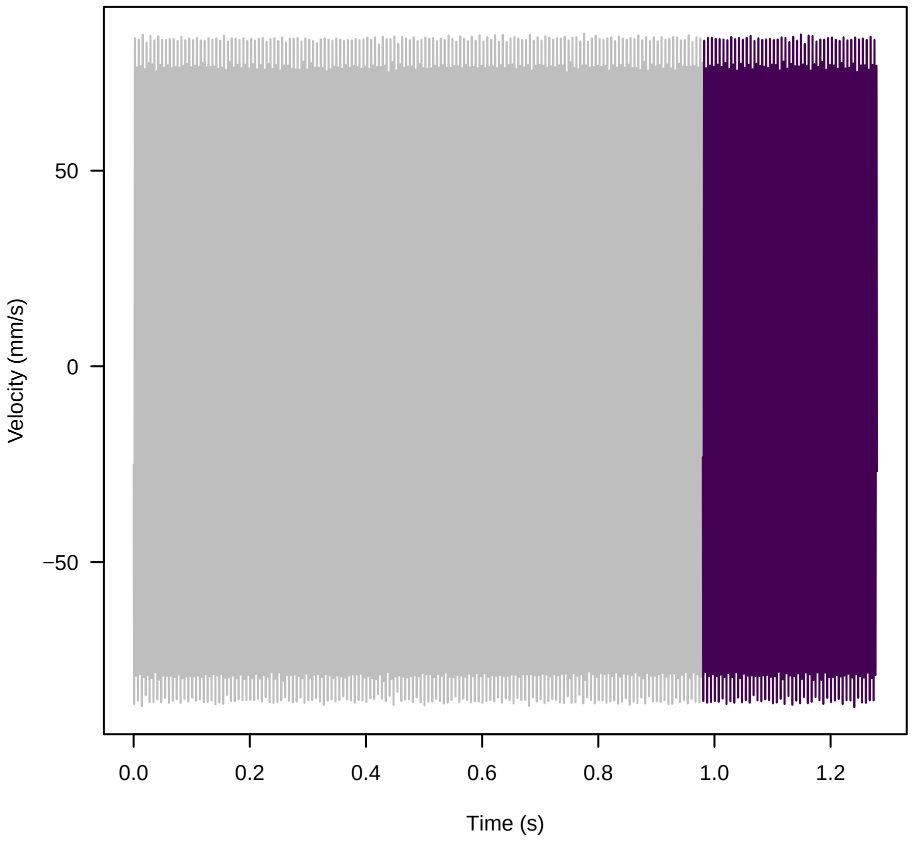

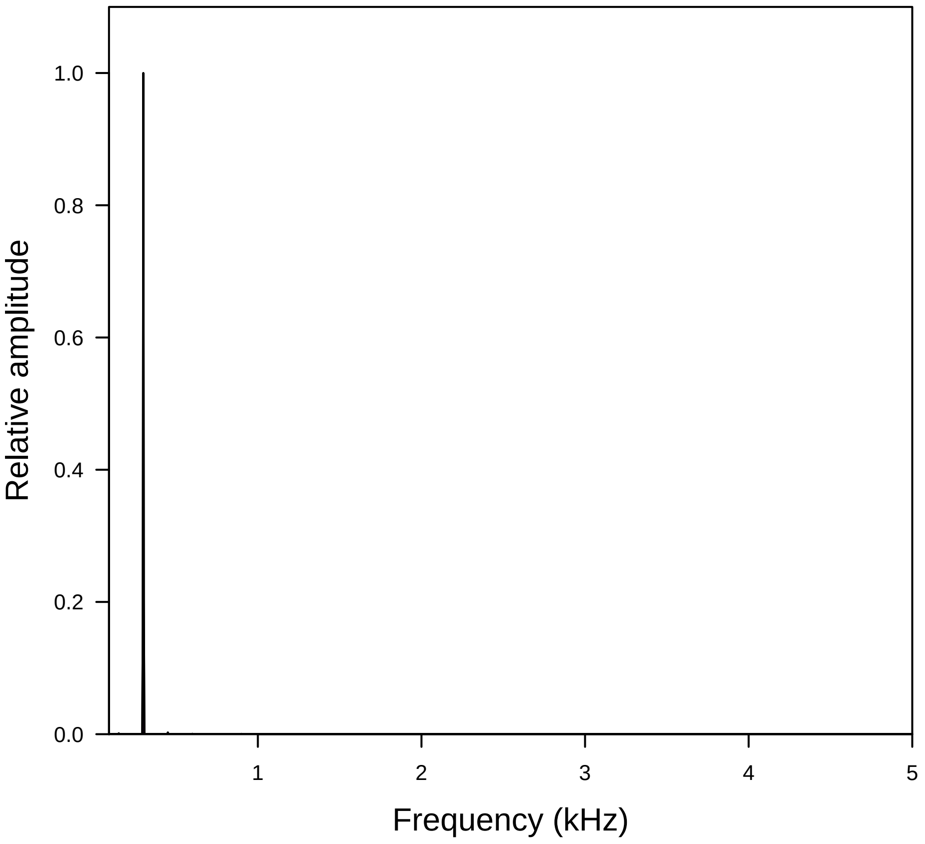

Vel. = 0.014 ; Str. = Corolla ; Axis = y ; Fl. accession = 10−s−77−3

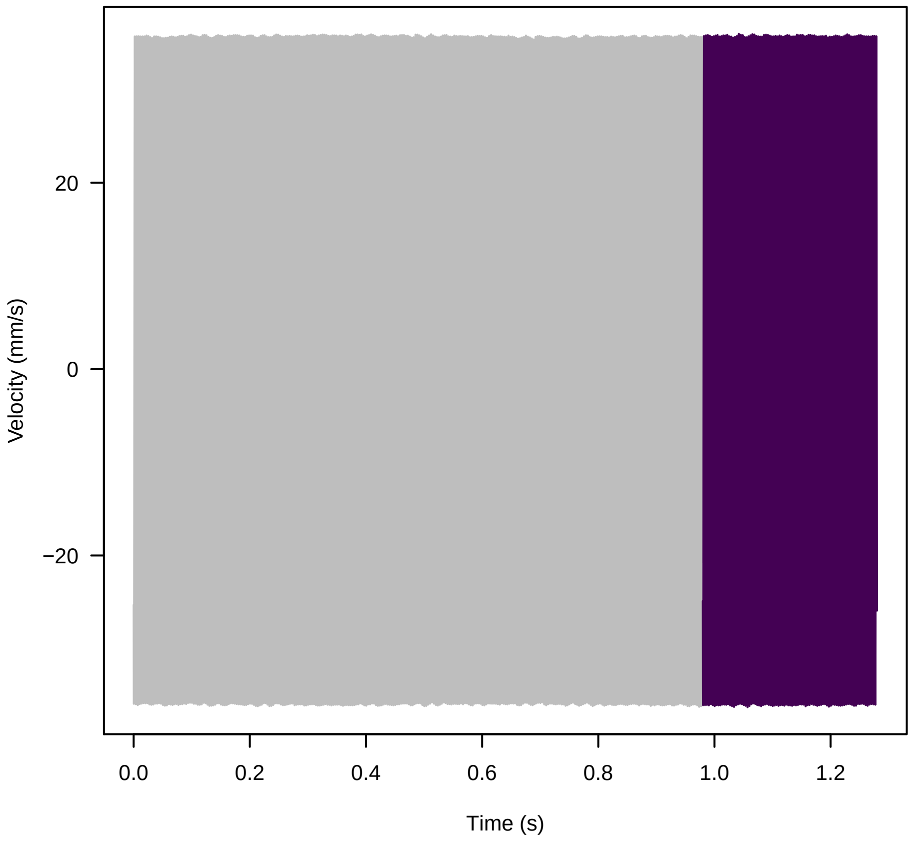

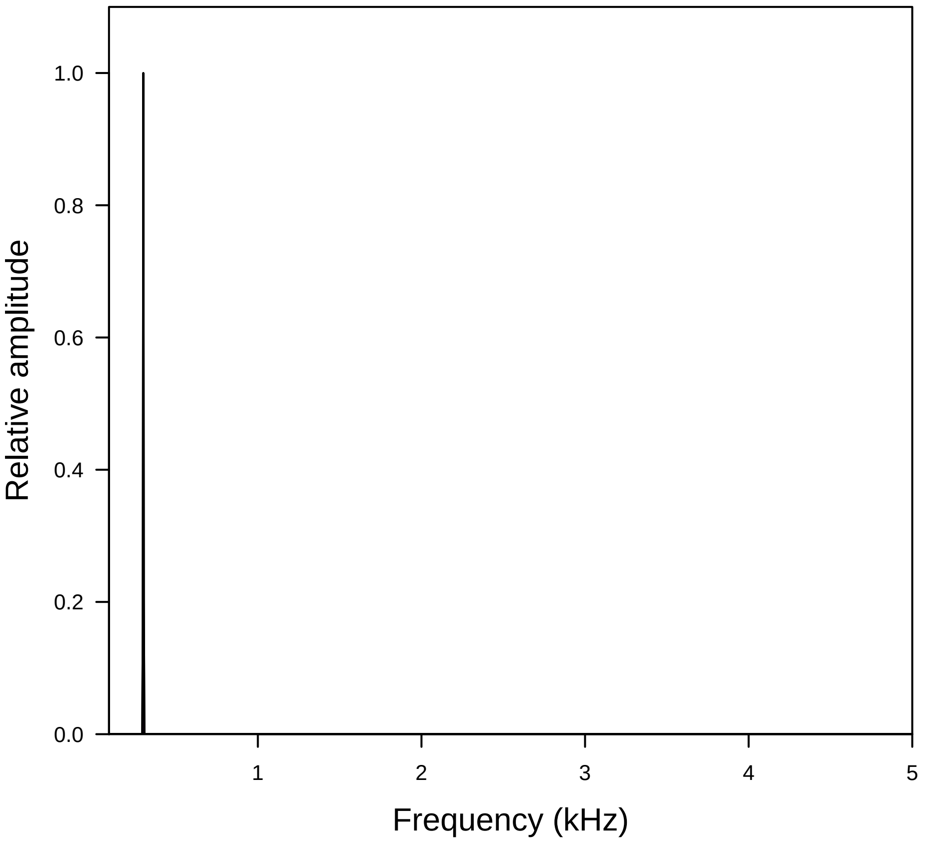

Vel. = 0.014 ; Str. = Receptacle ; Axis = y ; Fl. accession = 10−s−77−3

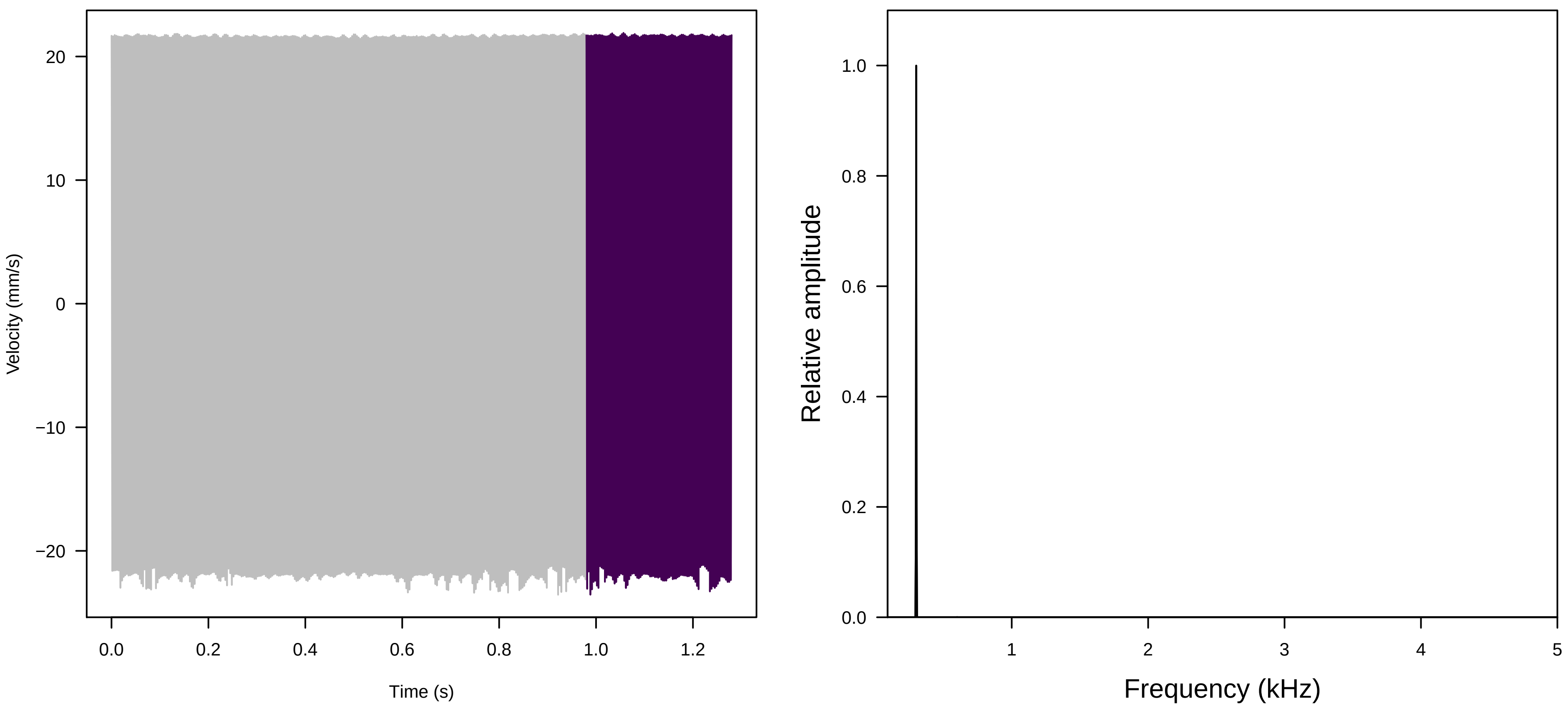

Vel. = 0.014 ; Str. = FA ; Axis = y ; Fl. accession = 10−s−77−3

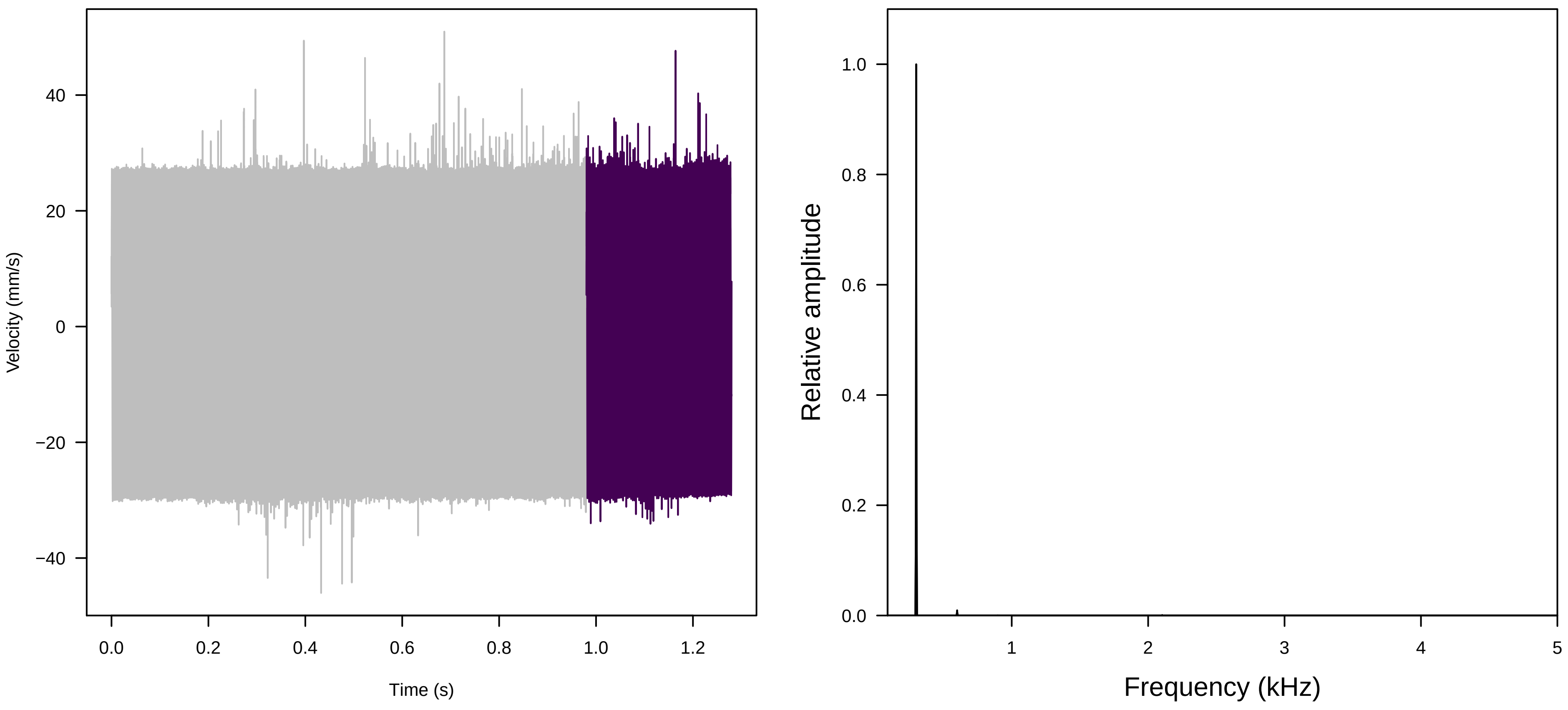

Vel. = 0.014 ; Str. = Receptacle ; Axis = y ; Fl. accession = 10−s−77−3

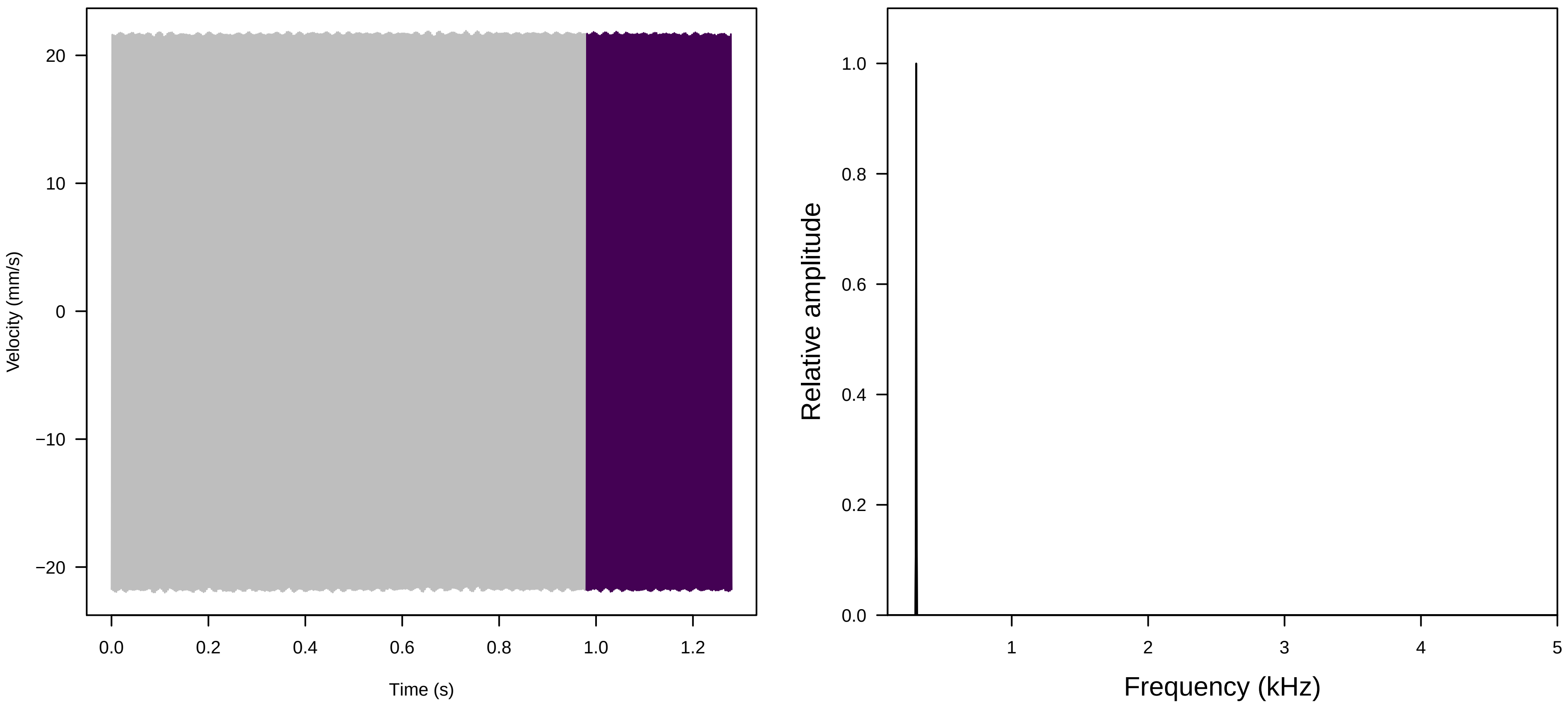

Vel. = 0.014 ; Str. = PA ; Axis = y ; Fl. accession = 10−s−77−3

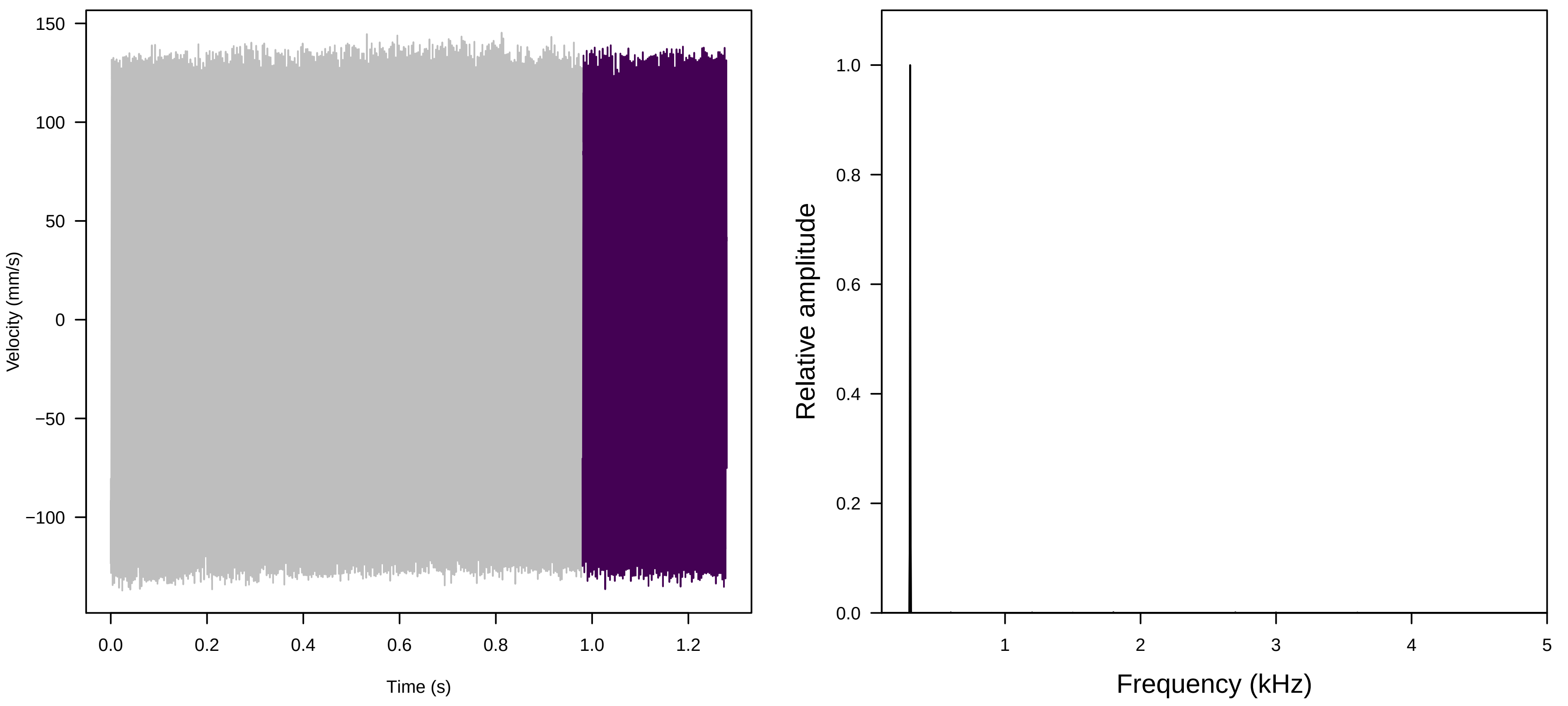

Vel. = 0.014 ; Str. = Receptacle ; Axis = y ; Fl. accession = 10−s−77−3

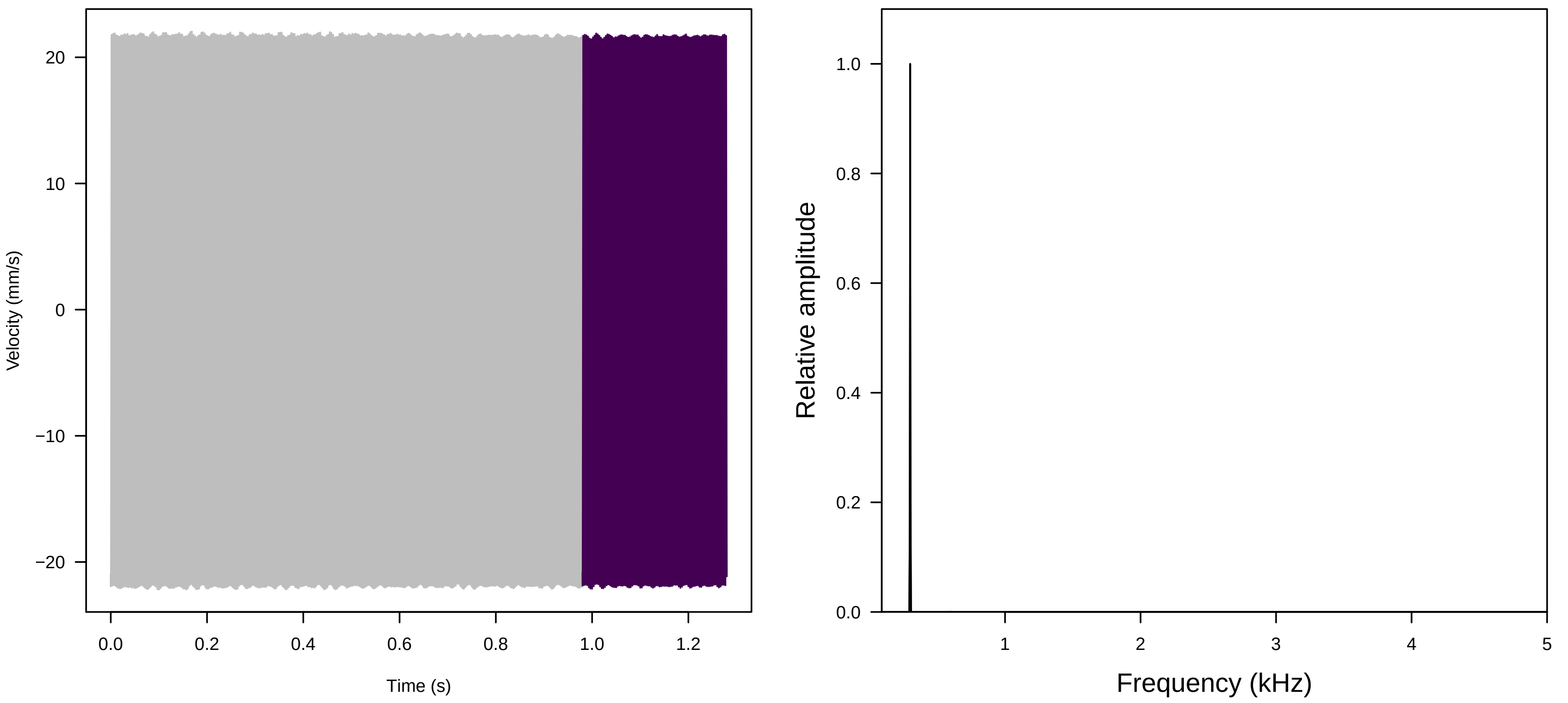

Vel. = 0.028 ; Str. = PA ; Axis = y ; Fl. accession = 10−s−77−3

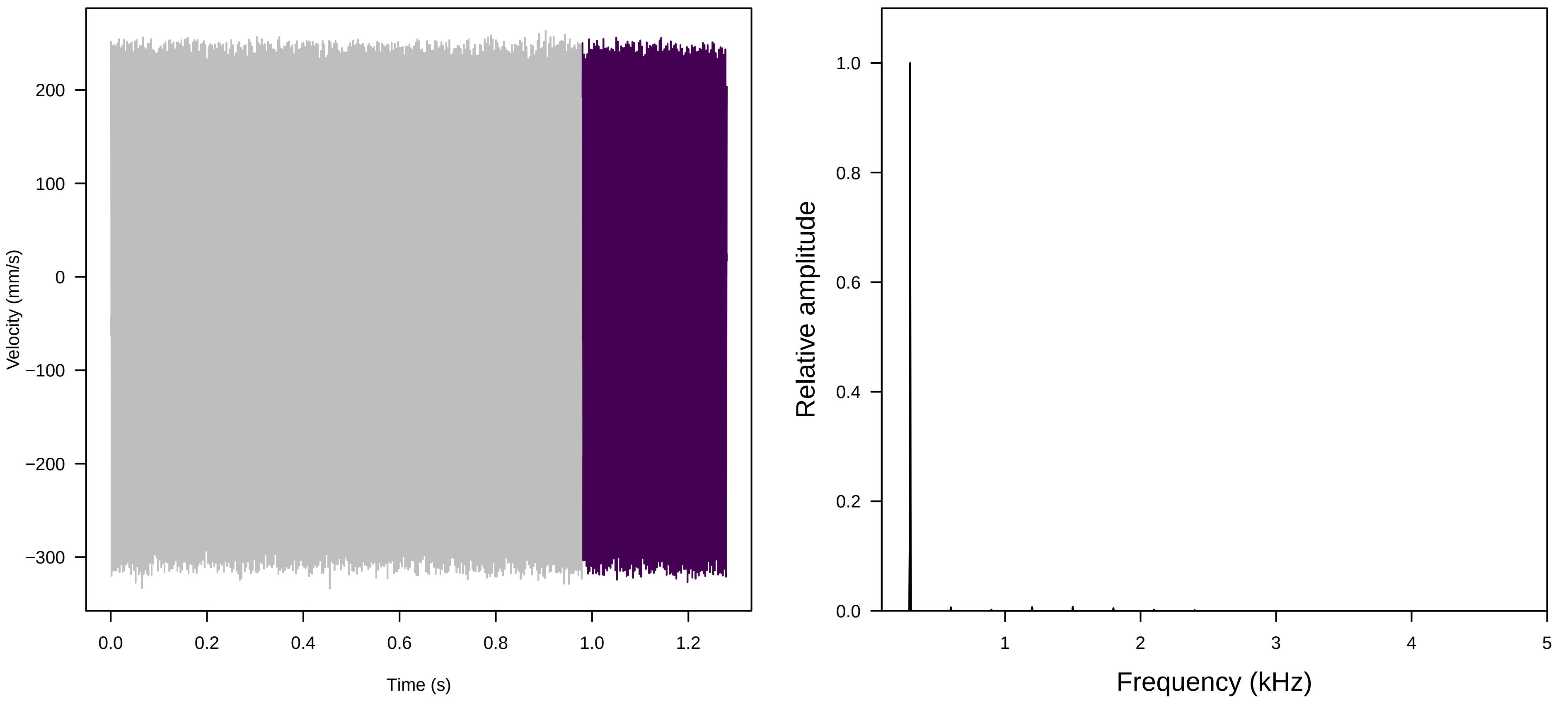

Vel. = 0.028 ; Str. = Receptacle ; Axis = y ; Fl. accession = 10−s−77−3

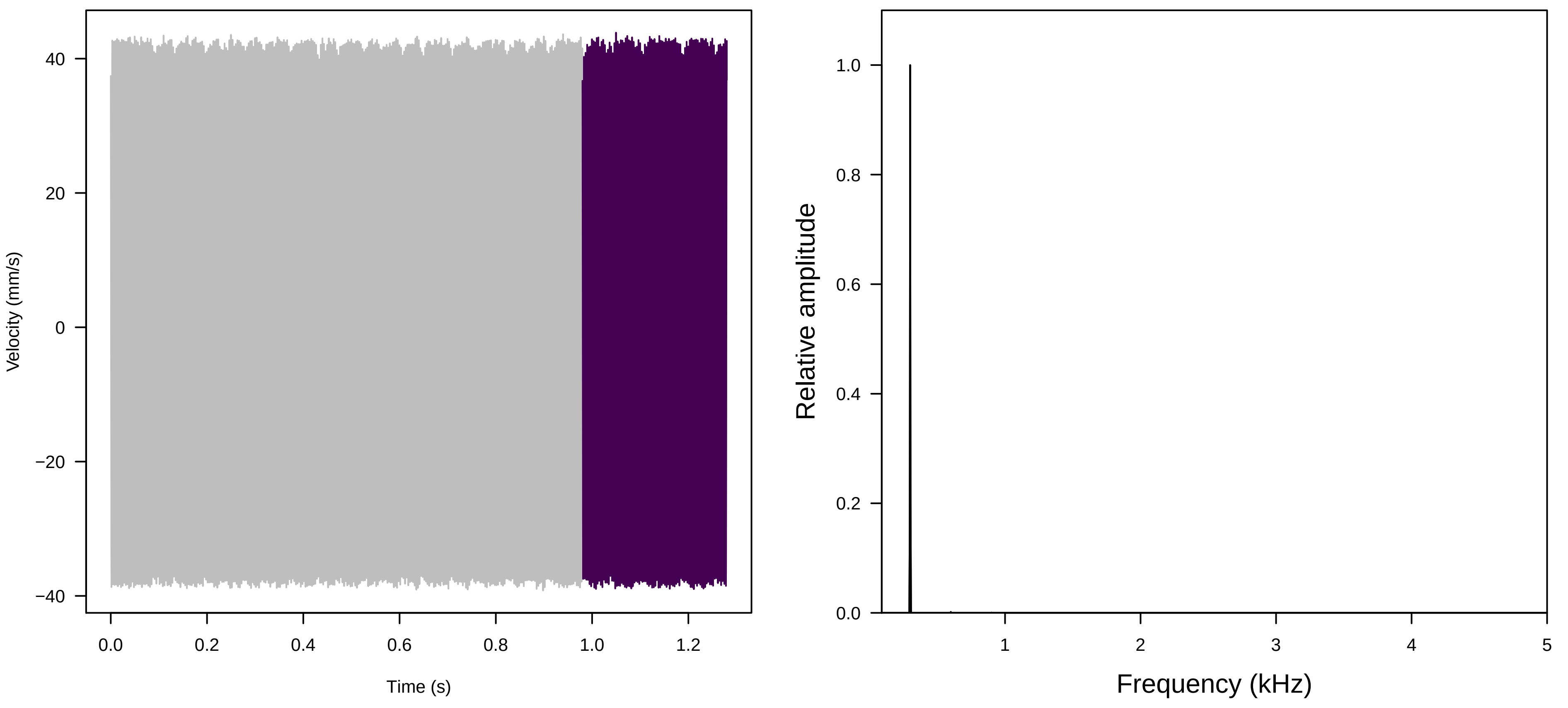

Vel. = 0.028 ; Str. = FA ; Axis = y ; Fl. accession = 10−s−77−3

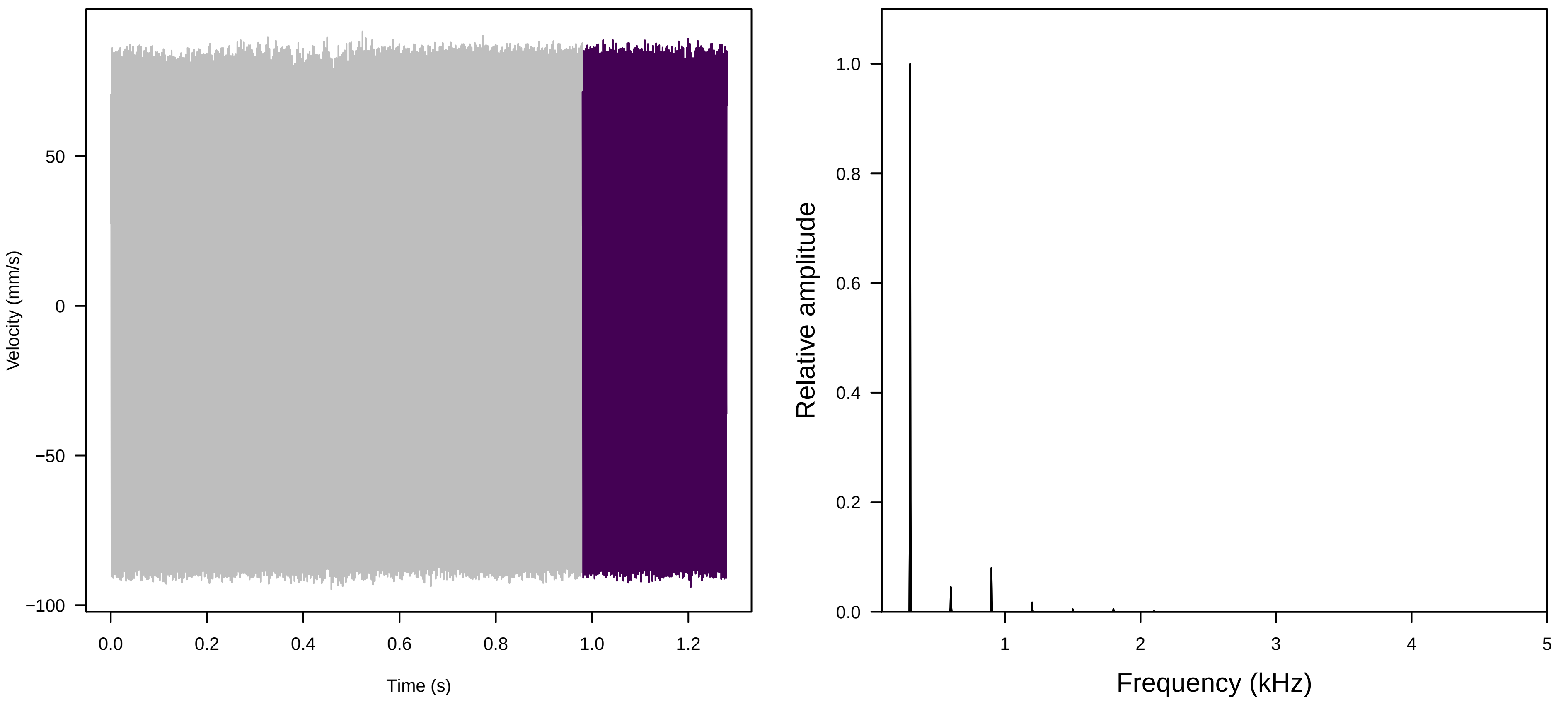

Vel. = 0.028 ; Str. = Receptacle ; Axis = y ; Fl. accession = 10−s−77−3

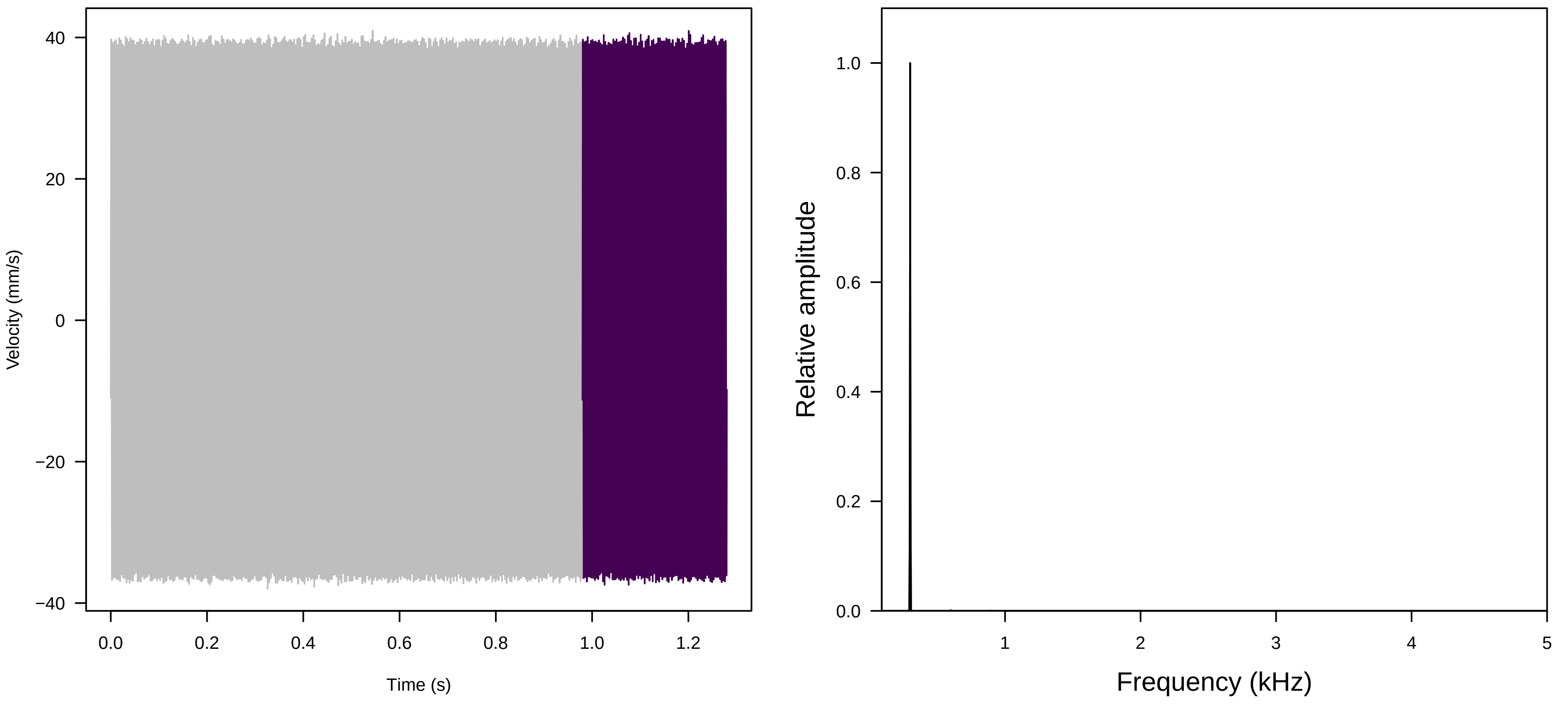

Vel. = 0.028 ; Str. = Corolla ; Axis = y ; Fl. accession = 10−s−77−3

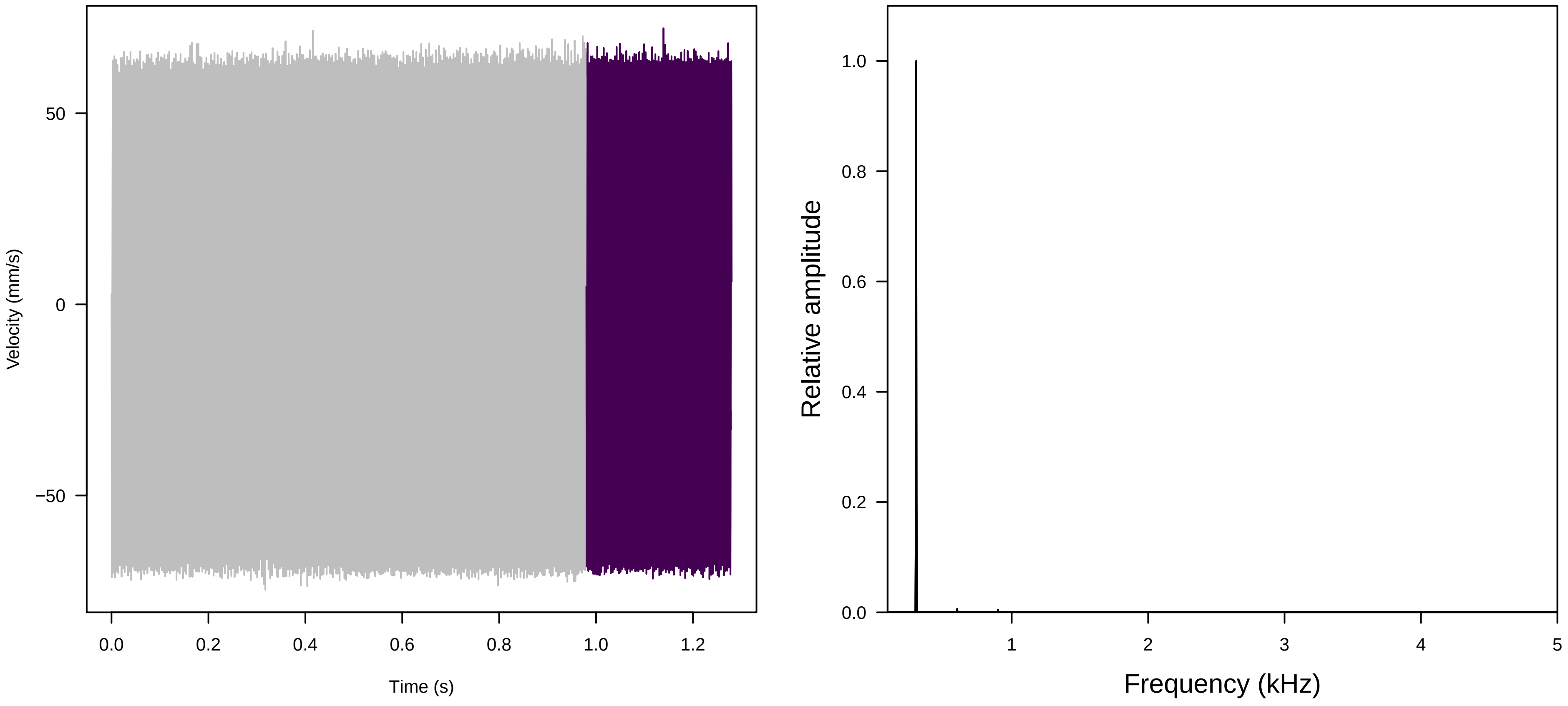

Vel. = 0.028 ; Str. = Receptacle ; Axis = y ; Fl. accession = 10−s−77−3

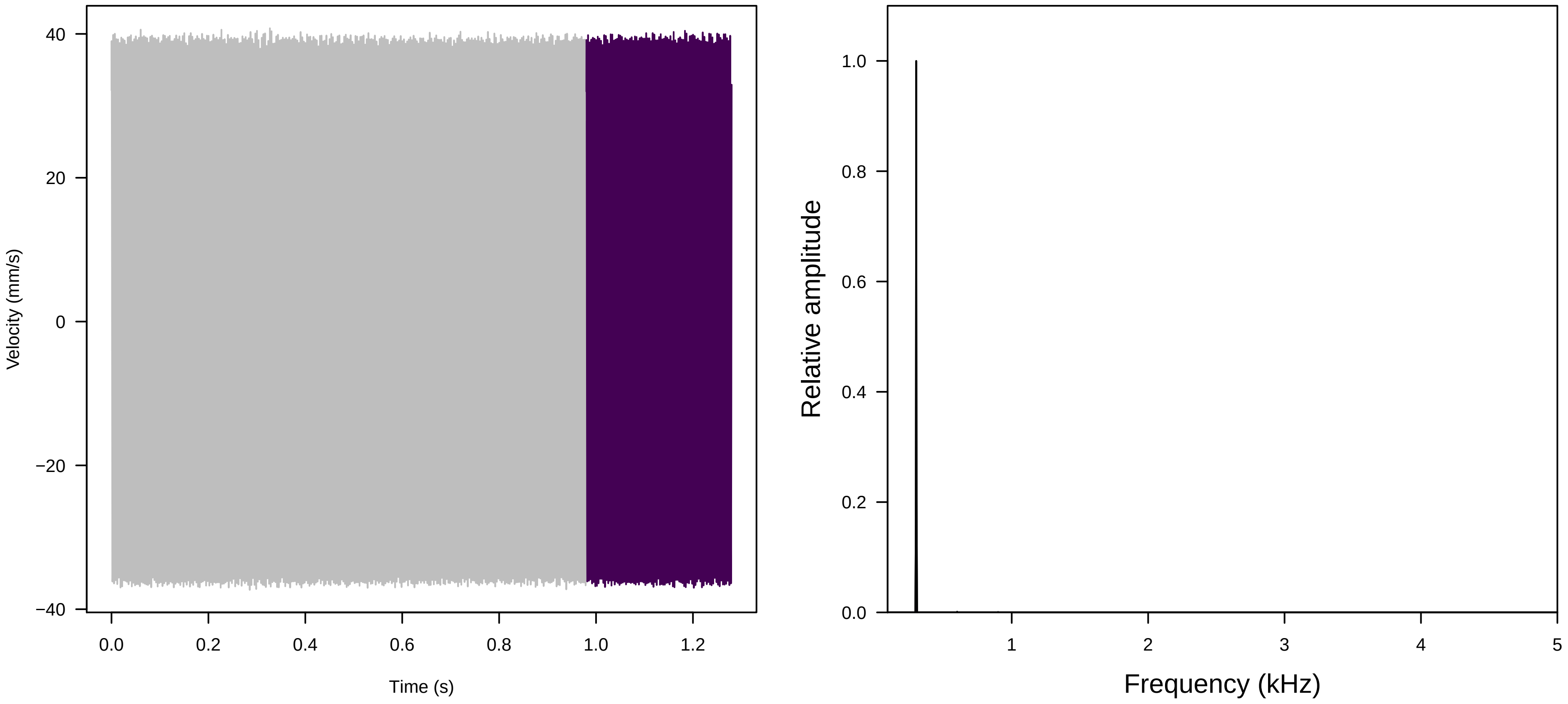

Vel. = 0.057 ; Str. = Corolla ; Axis = y ; Fl. accession = 10−s−77−3

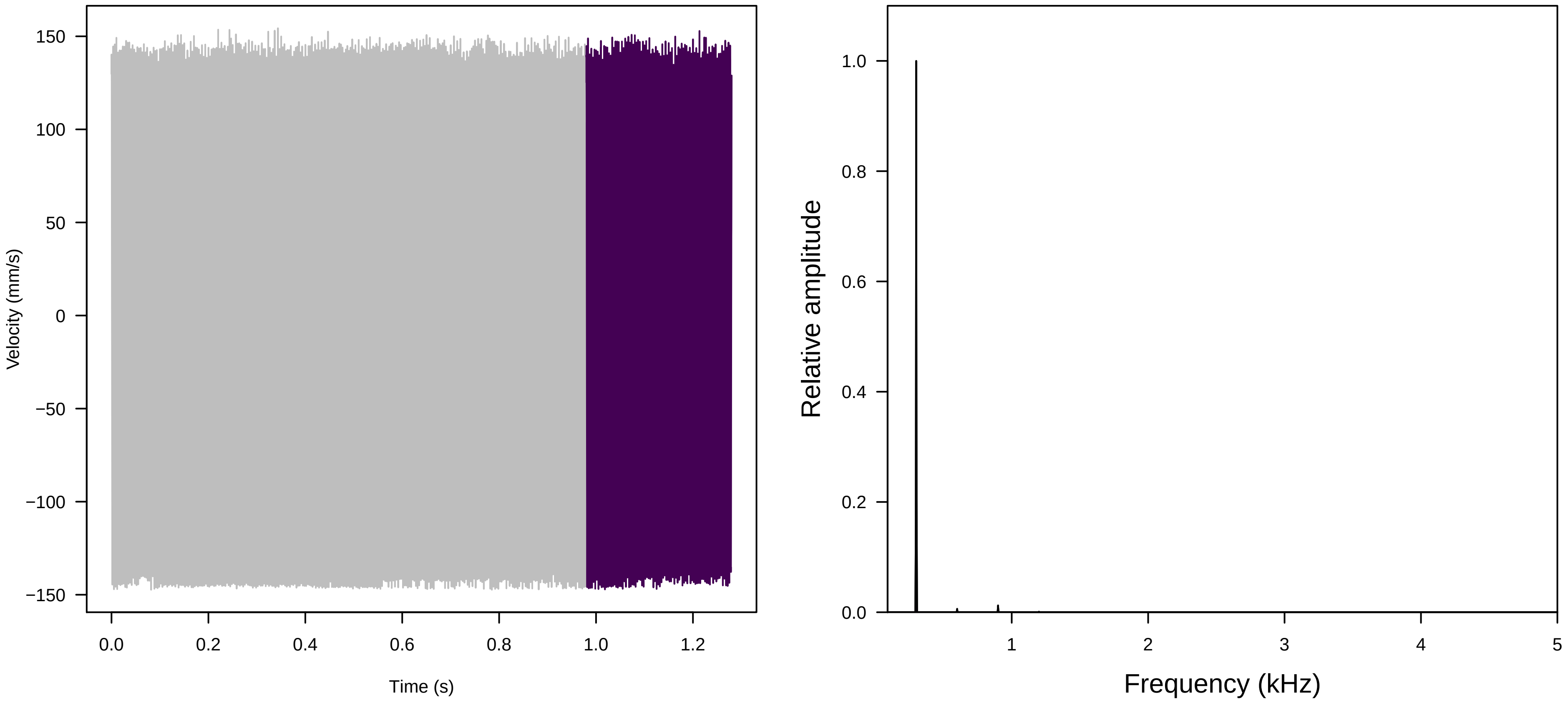

Vel. = 0.057 ; Str. = Receptacle ; Axis = y ; Fl. accession = 10−s−77−3

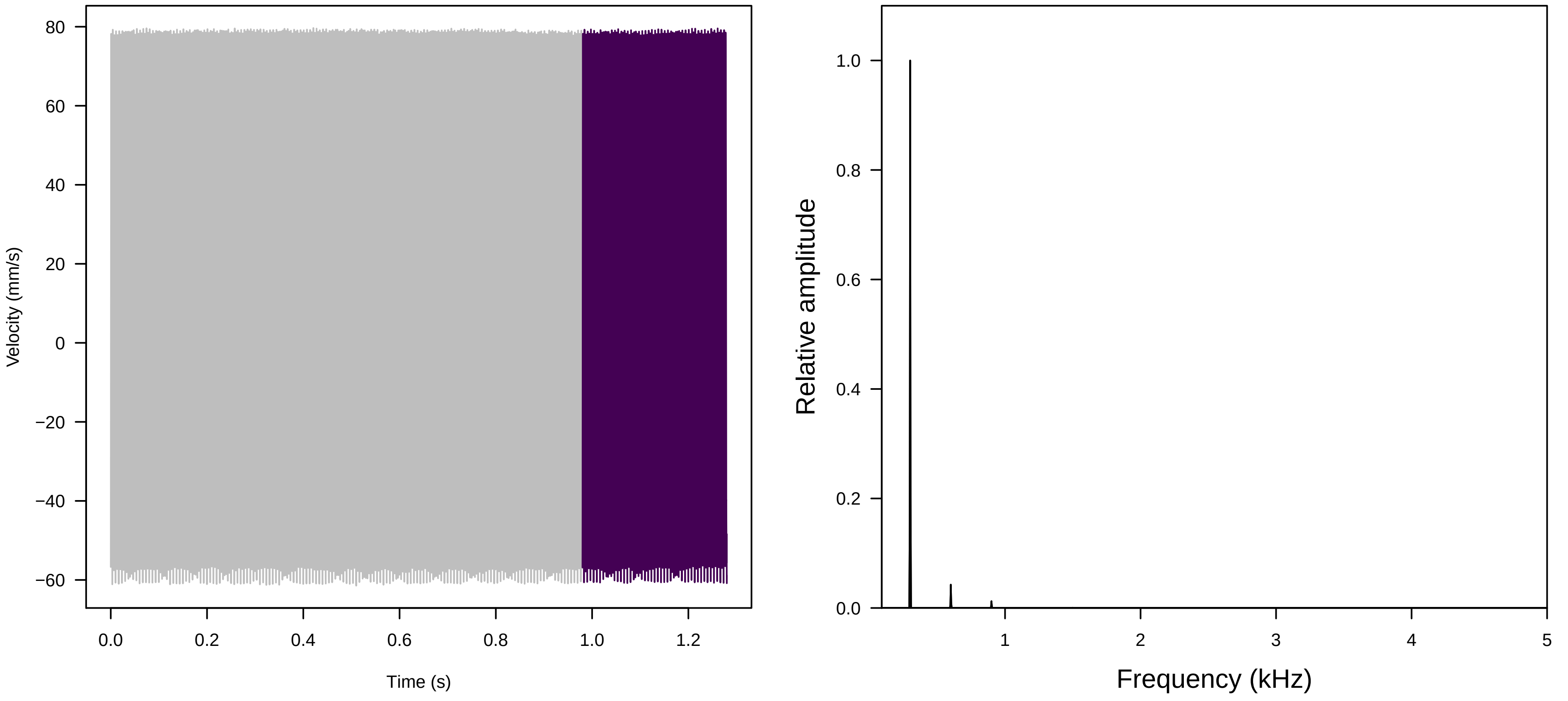

Vel. = 0.057 ; Str. = FA ; Axis = y ; Fl. accession = 10−s−77−3

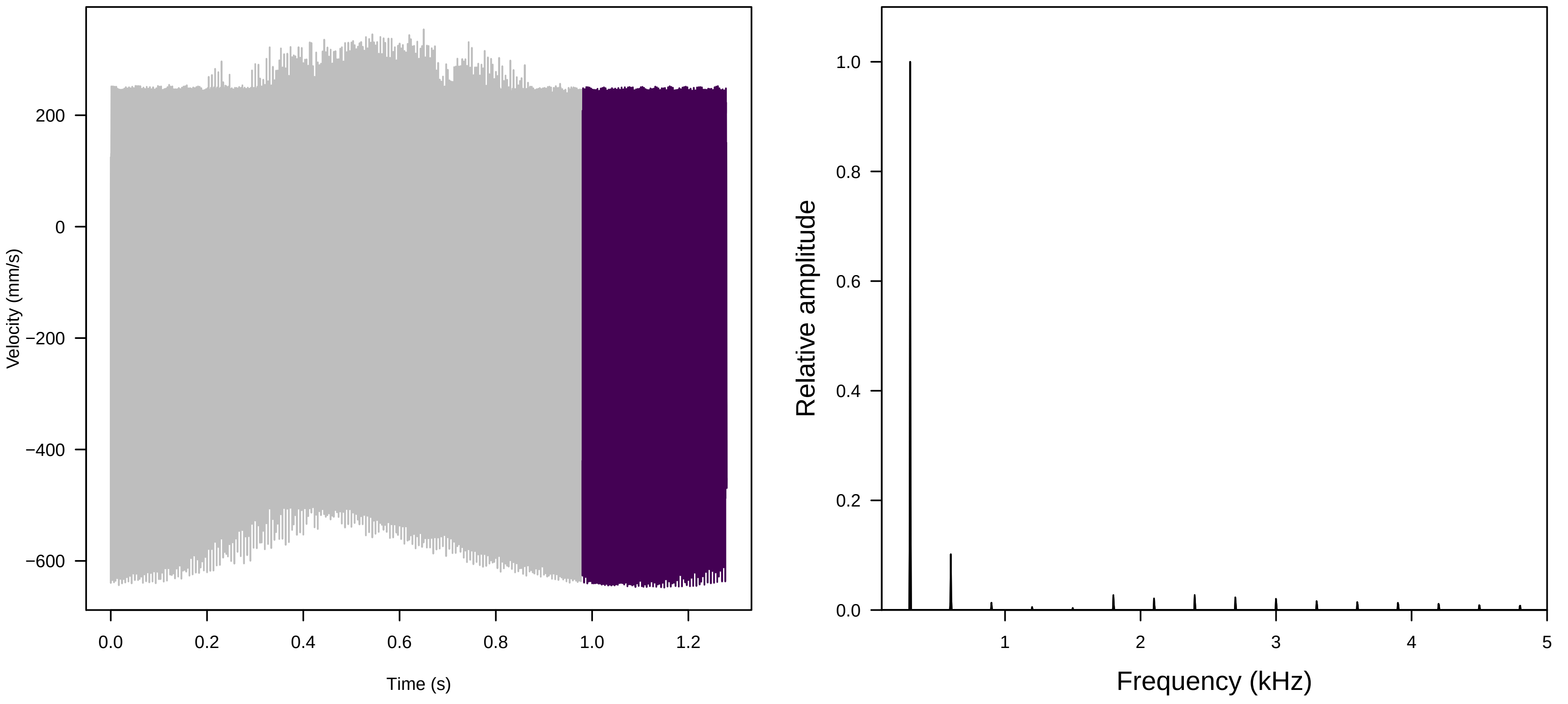

Vel. = 0.057 ; Str. = Receptacle ; Axis = y ; Fl. accession = 10−s−77−3

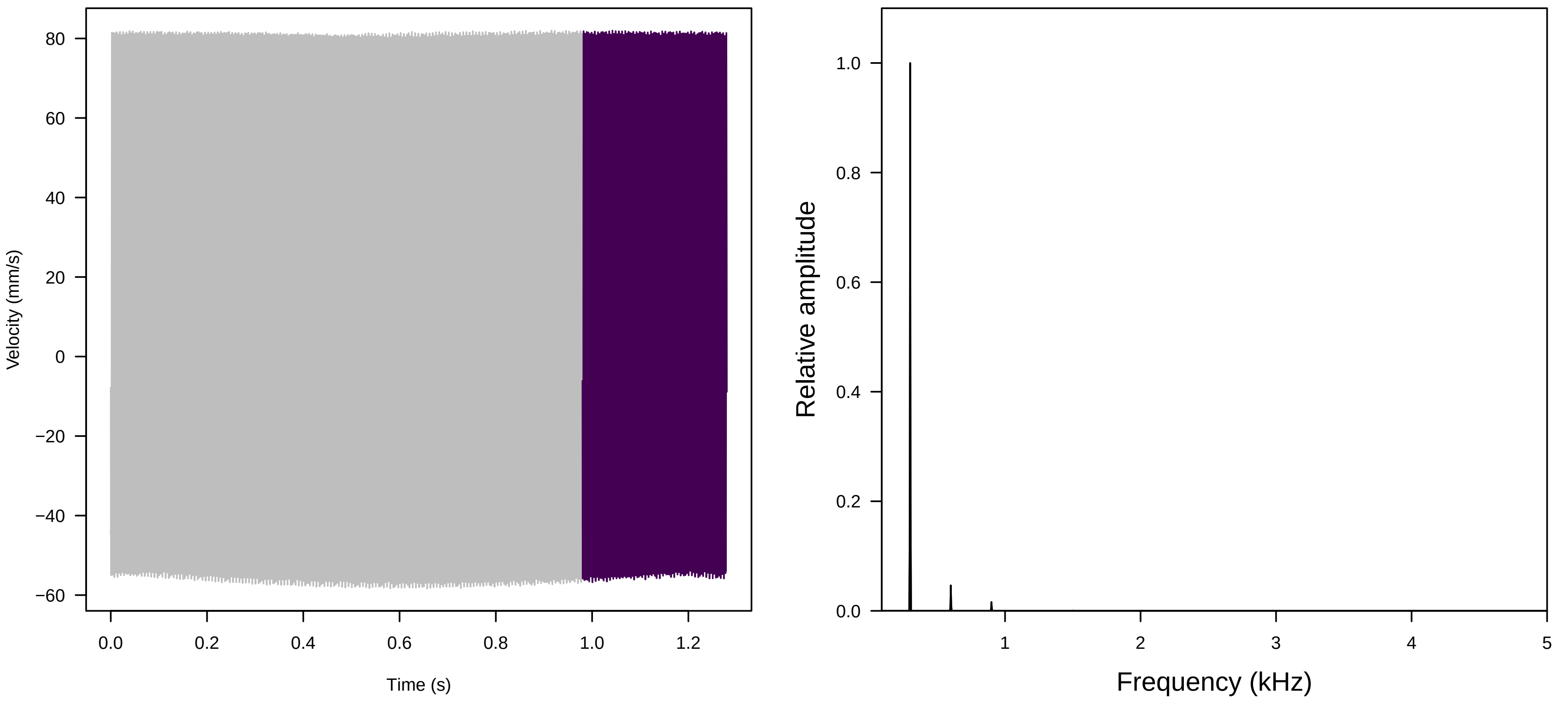

Vel. = 0.057 ; Str. = PA ; Axis = y ; Fl. accession = 10−s−77−3

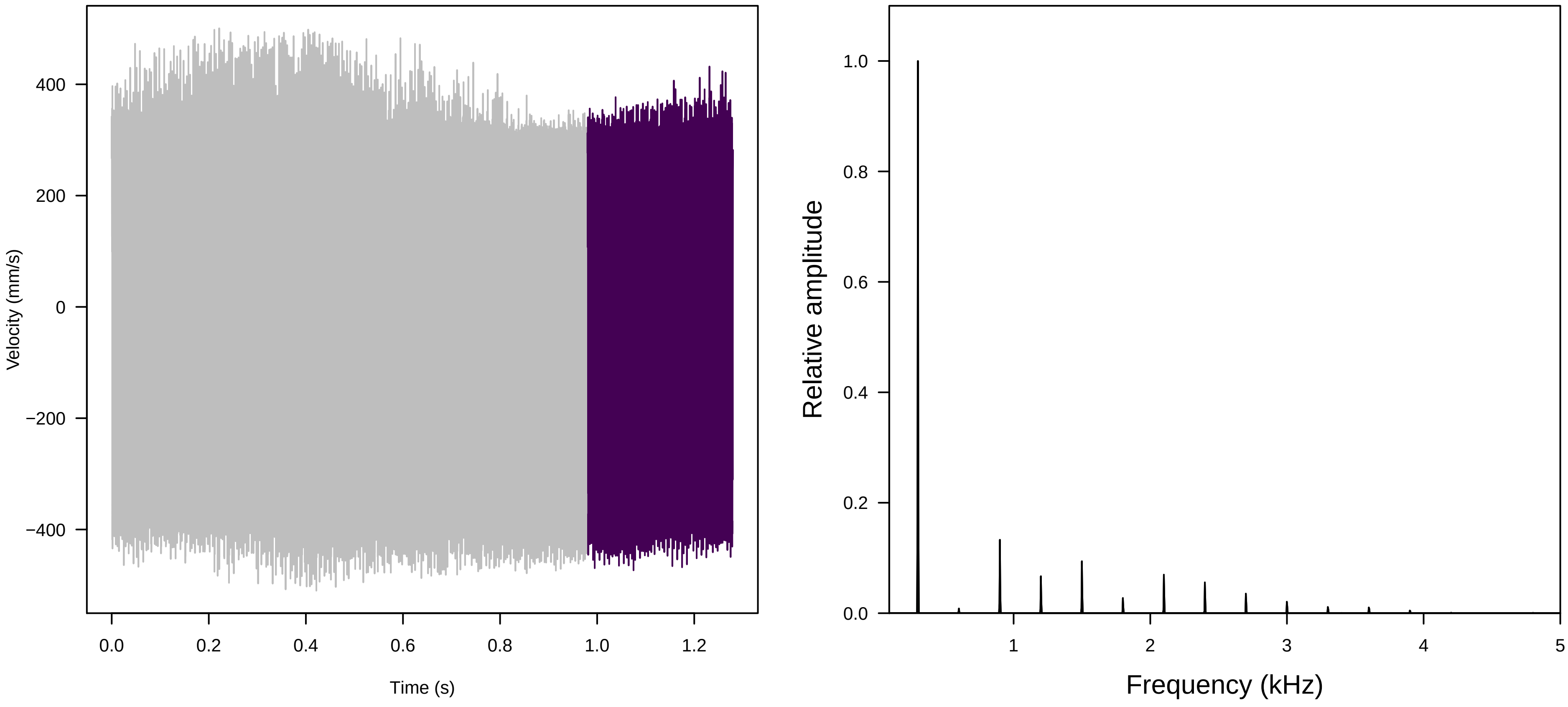

Vel. = 0.057 ; Str. = Receptacle ; Axis = y ; Fl. accession = 10−s−77−3

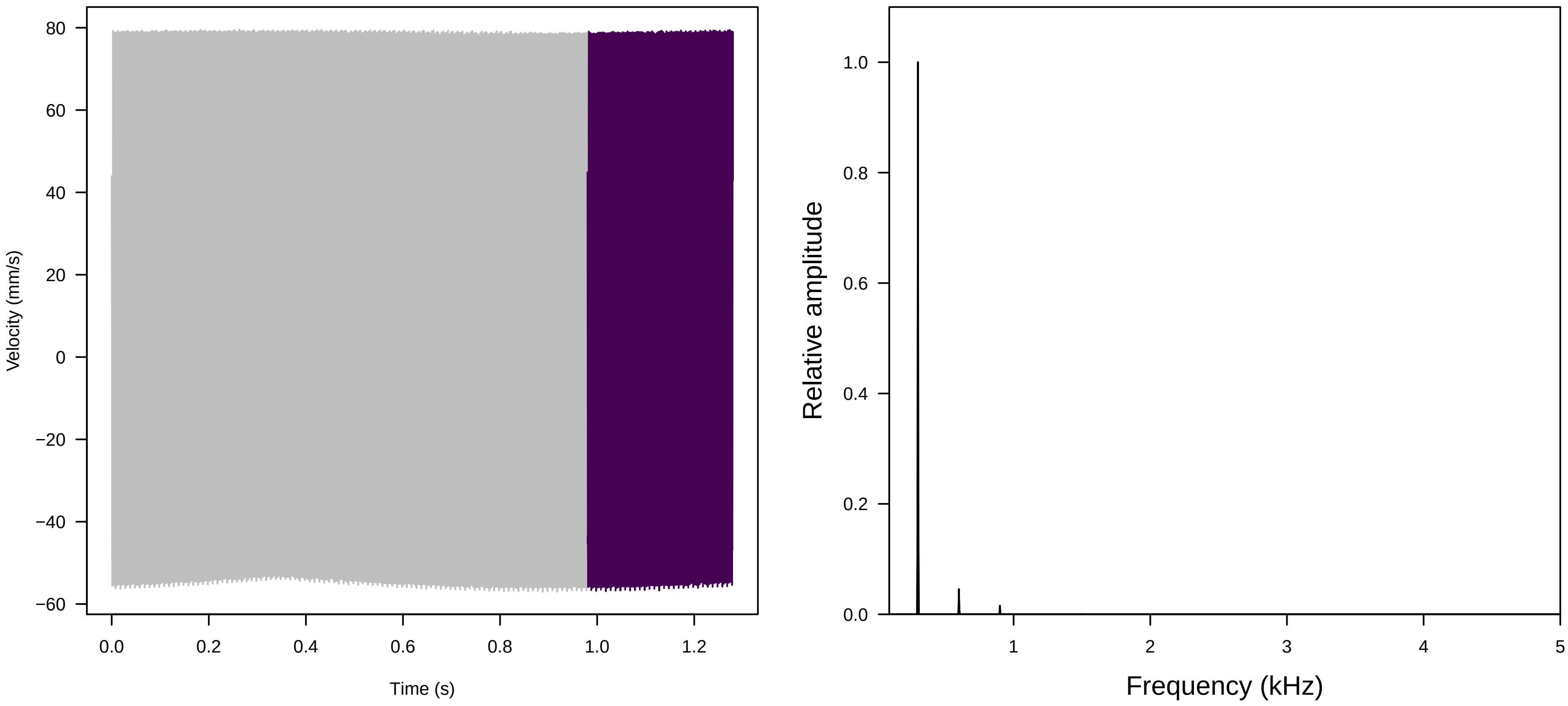

Vel. = 0.057 ; Str. = Corolla ; Axis = z ; Fl. accession = 10−s−77−3

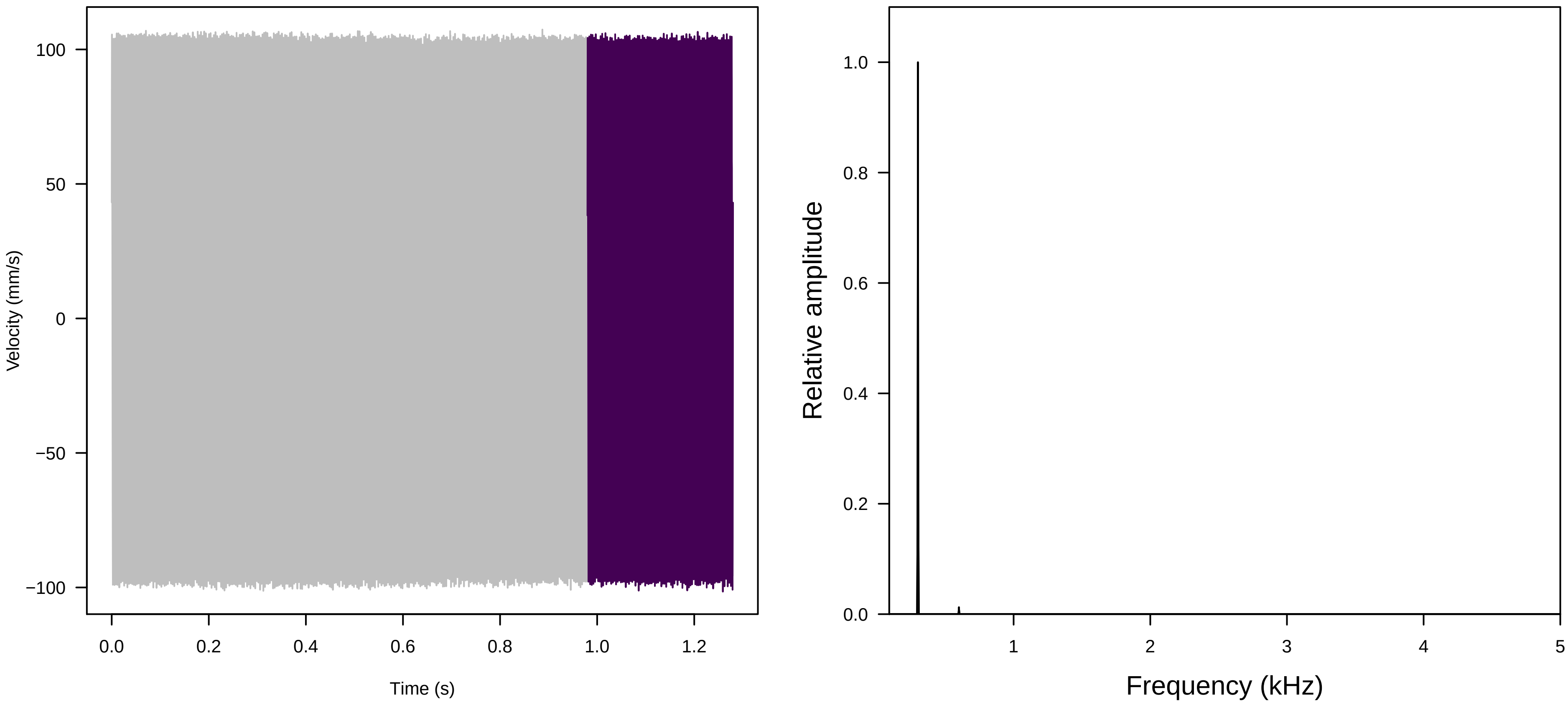

Vel. = 0.057 ; Str. = Receptacle ; Axis = z ; Fl. accession = 10−s−77−3

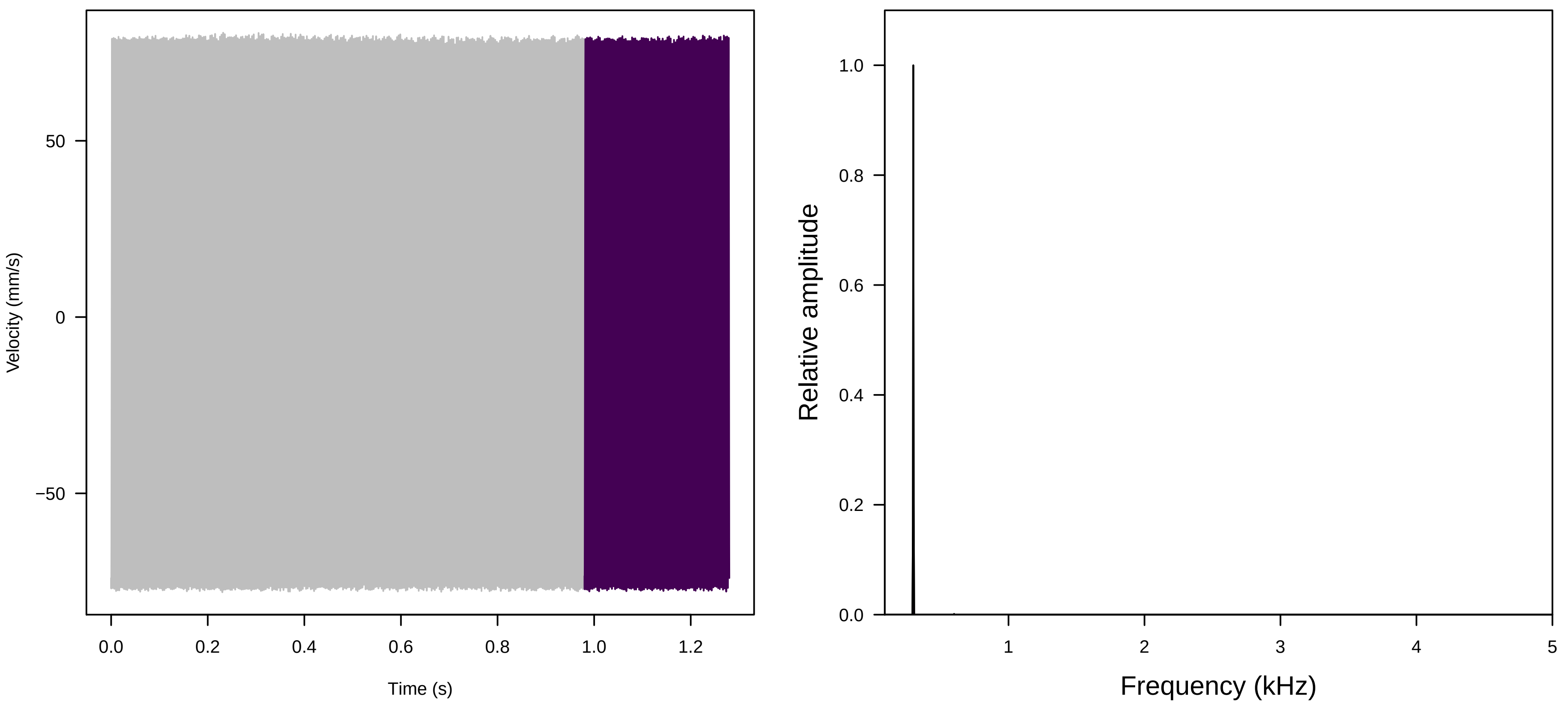

Vel. = 0.057 ; Str. = FA ; Axis = z ; Fl. accession = 10−s−77−3

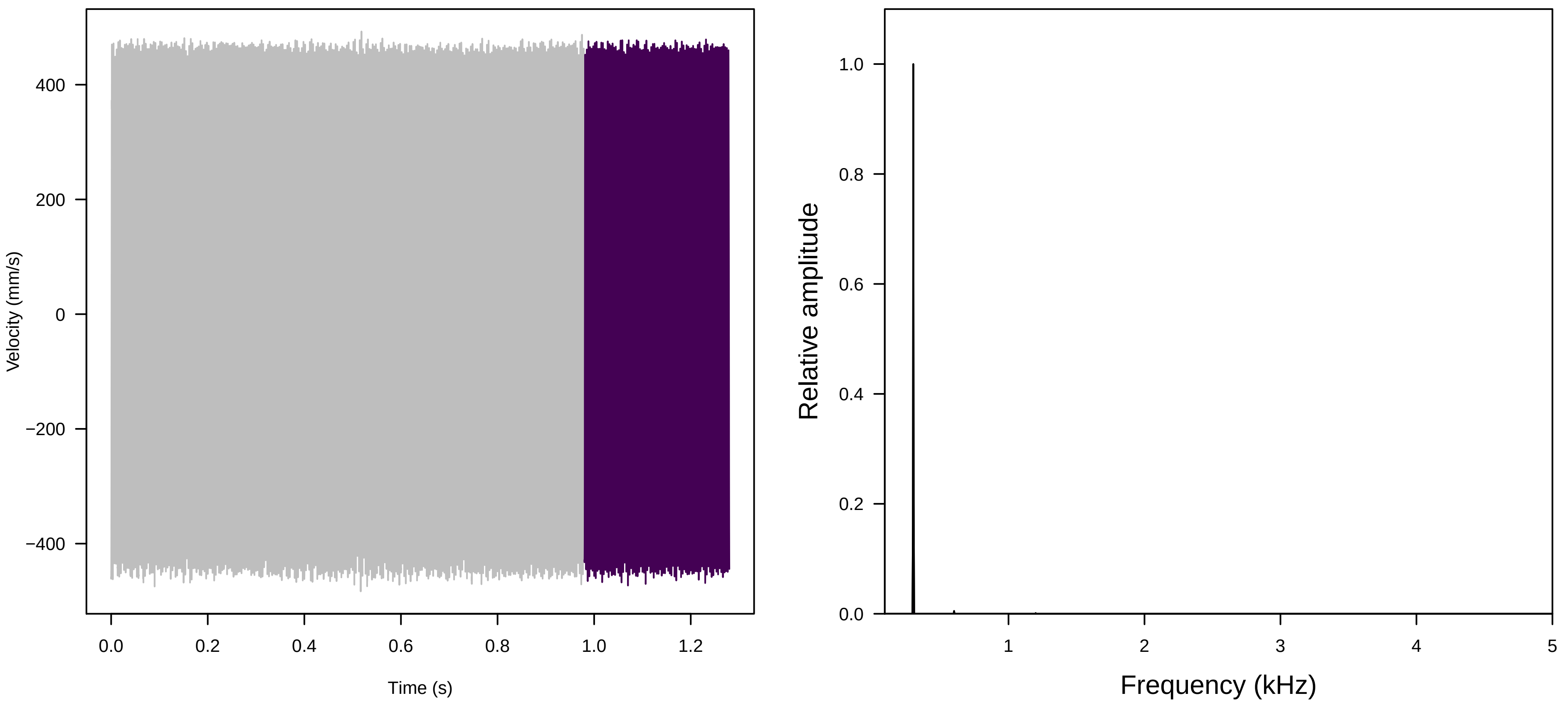

Vel. = 0.057 ; Str. = Receptacle ; Axis = z ; Fl. accession = 10−s−77−3

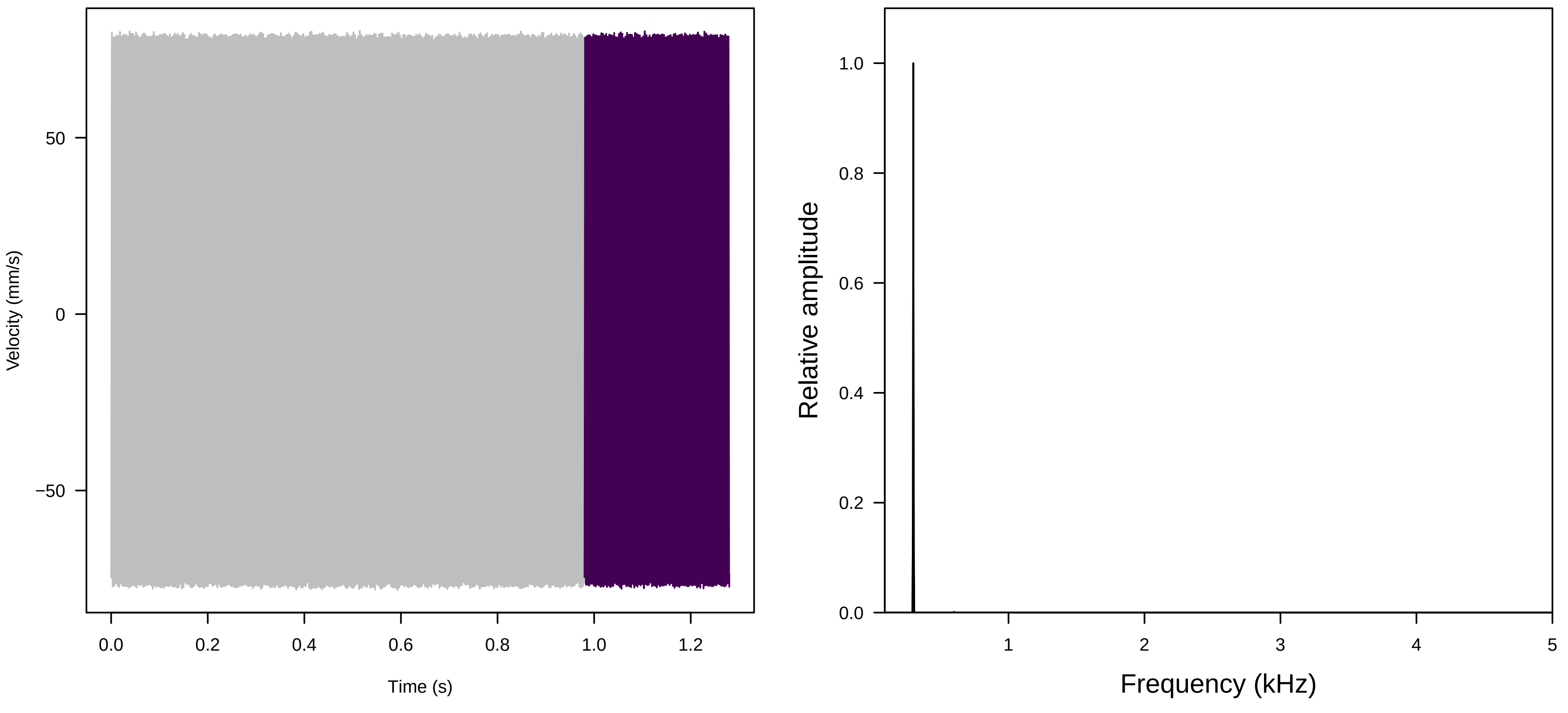

Vel. = 0.057 ; Str. = PA ; Axis = z ; Fl. accession = 10−s−77−3

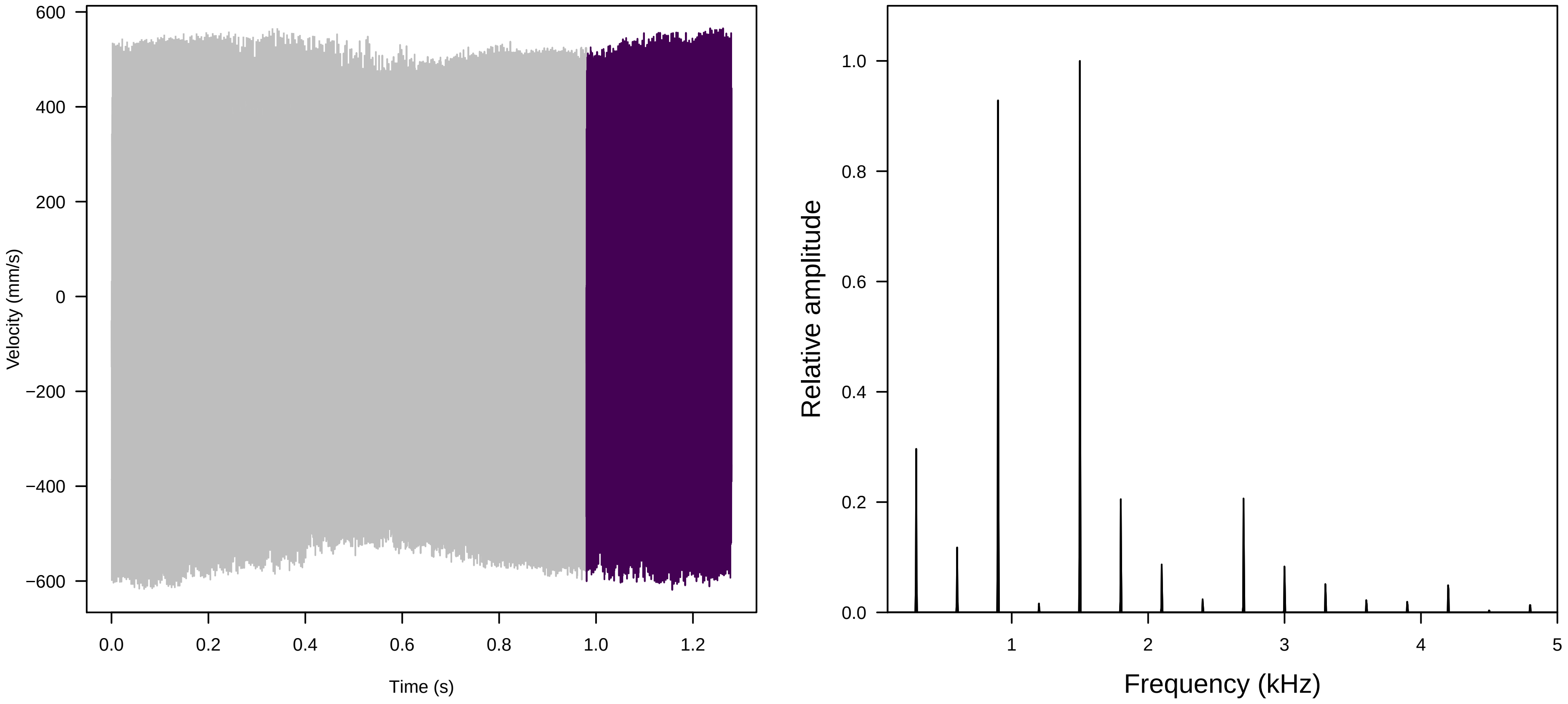

Vel. = 0.057 ; Str. = Receptacle ; Axis = z ; Fl. accession = 10−s−77−3

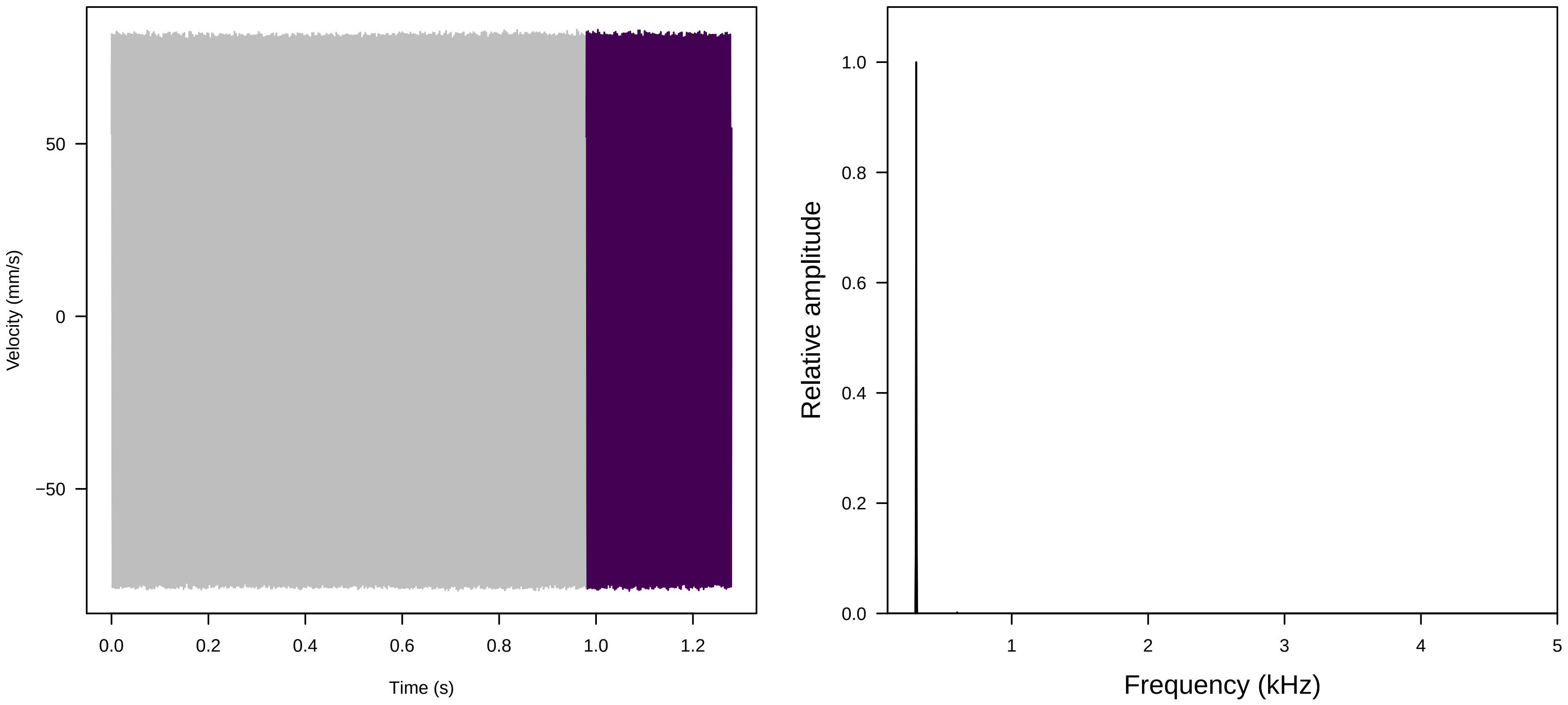

Vel. = 0.028 ; Str. = PA ; Axis = z ; Fl. accession = 10−s−77−3

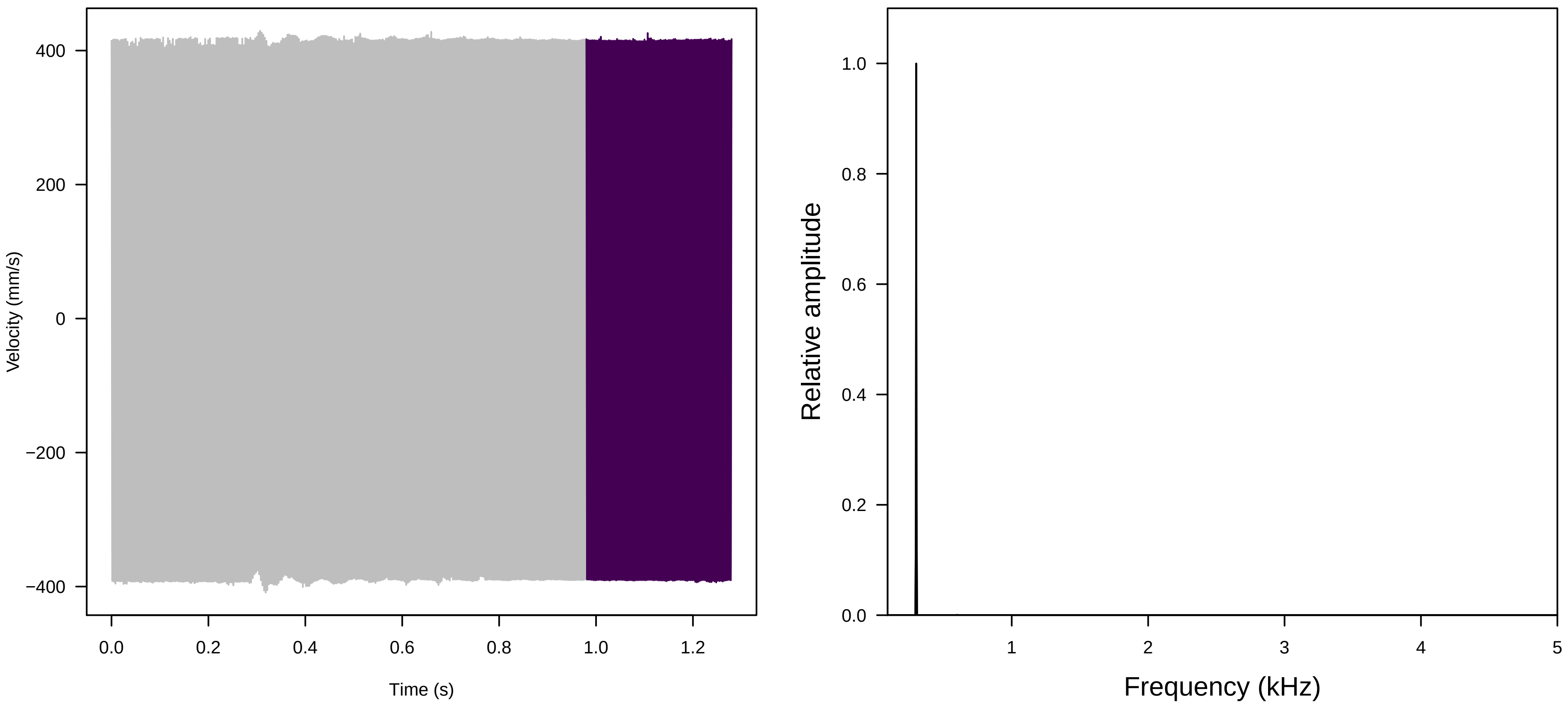

Vel. = 0.028 ; Str. = Receptacle ; Axis = z ; Fl. accession = 10−s−77−3

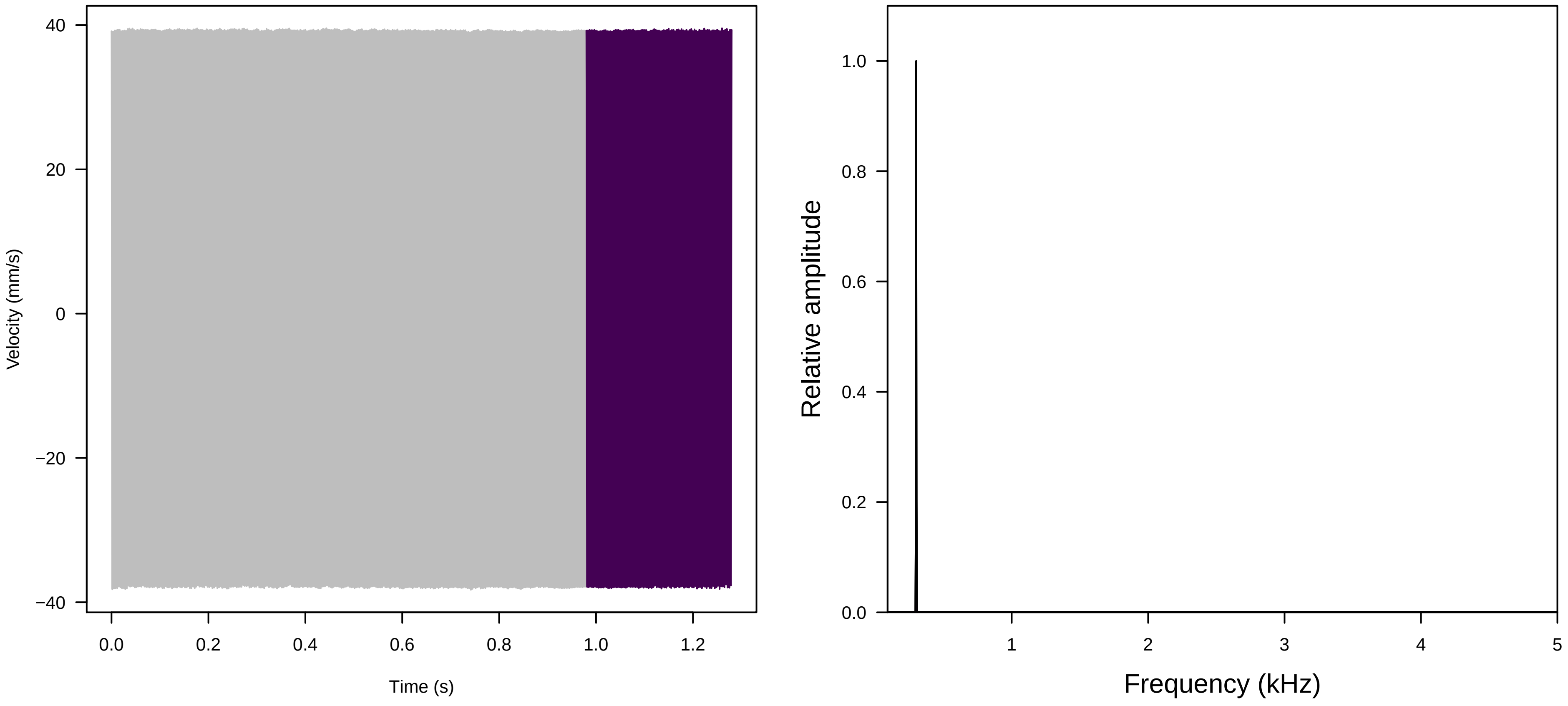

Vel. = 0.028 ; Str. = FA ; Axis = z ; Fl. accession = 10−s−77−3

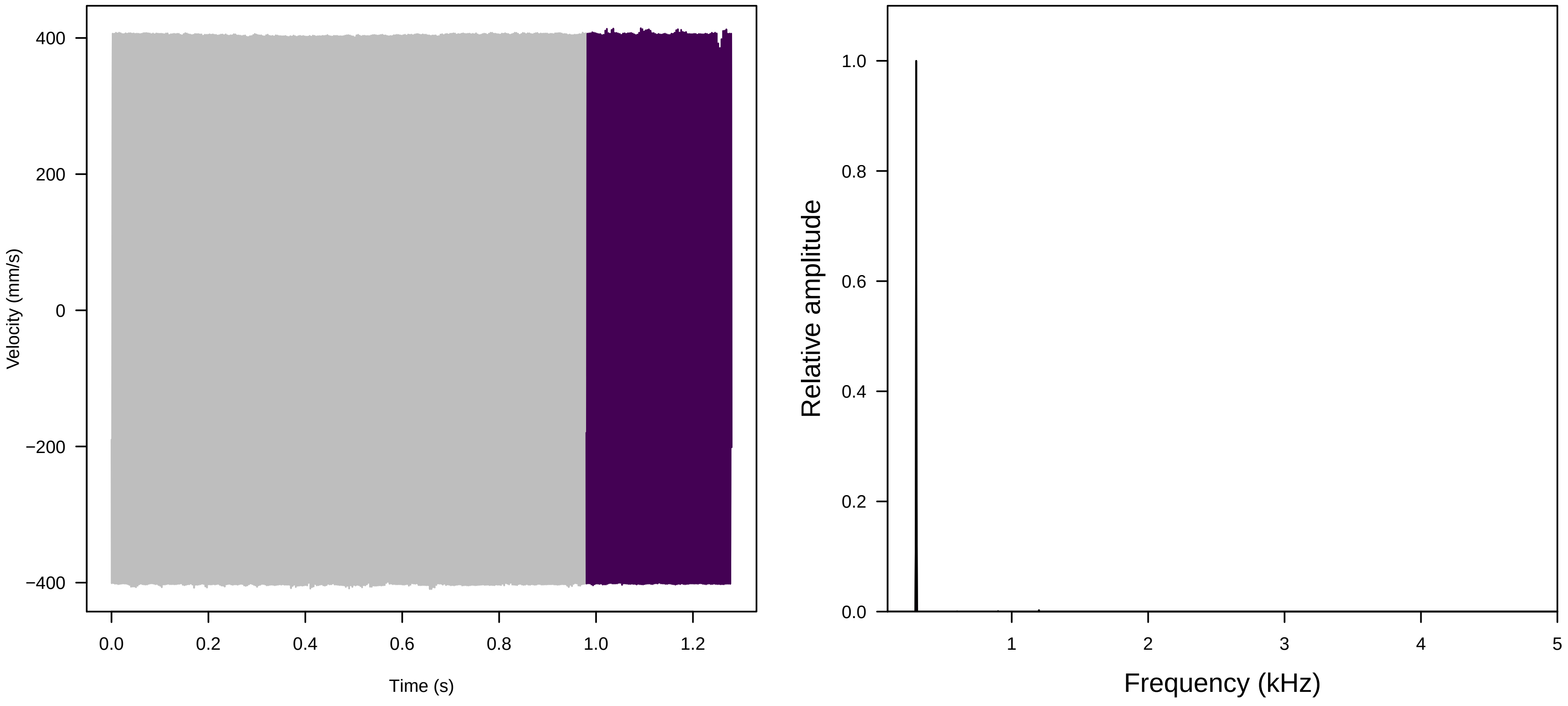

Vel. = 0.028 ; Str. = Receptacle ; Axis = z ; Fl. accession = 10−s−77−3

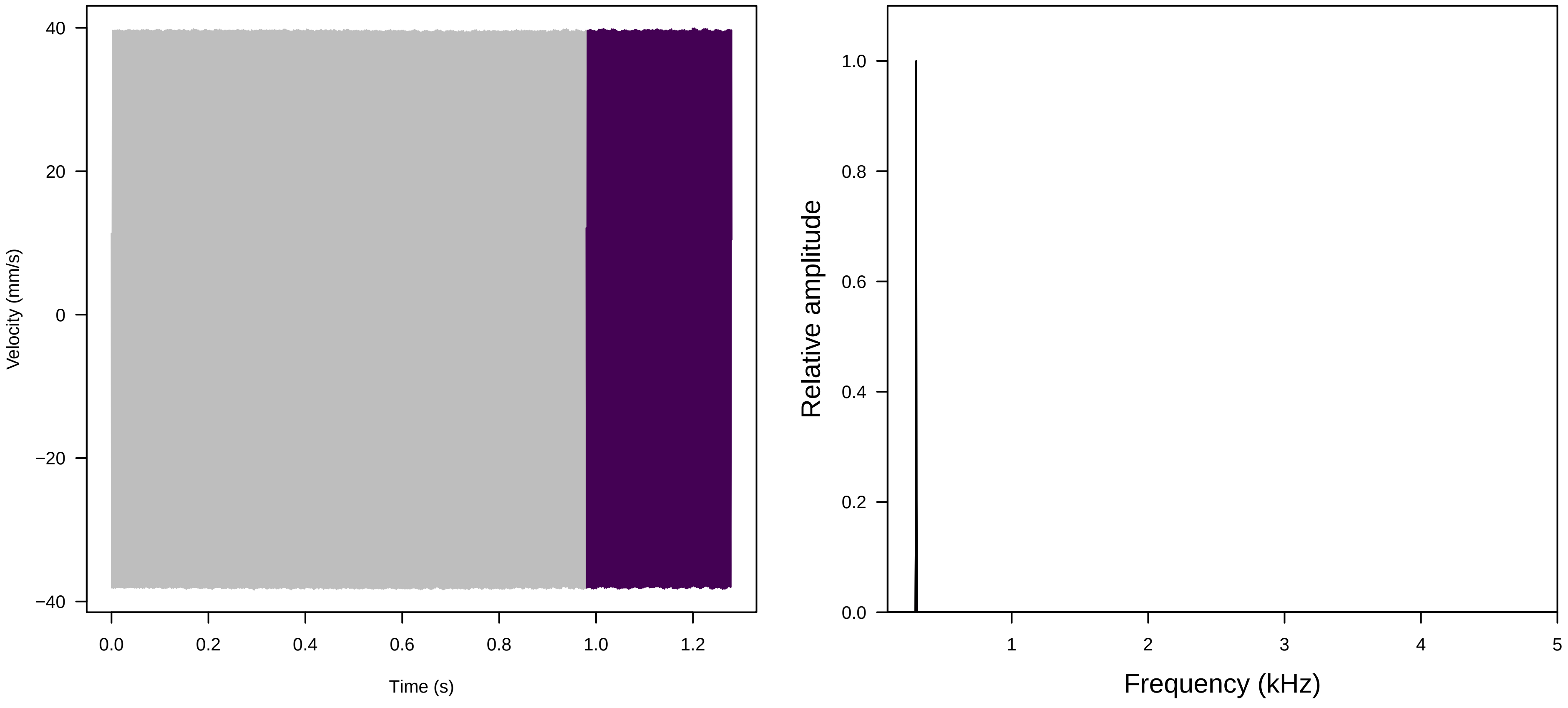

Vel. = 0.028 ; Str. = Corolla ; Axis = z ; Fl. accession = 10−s−77−3

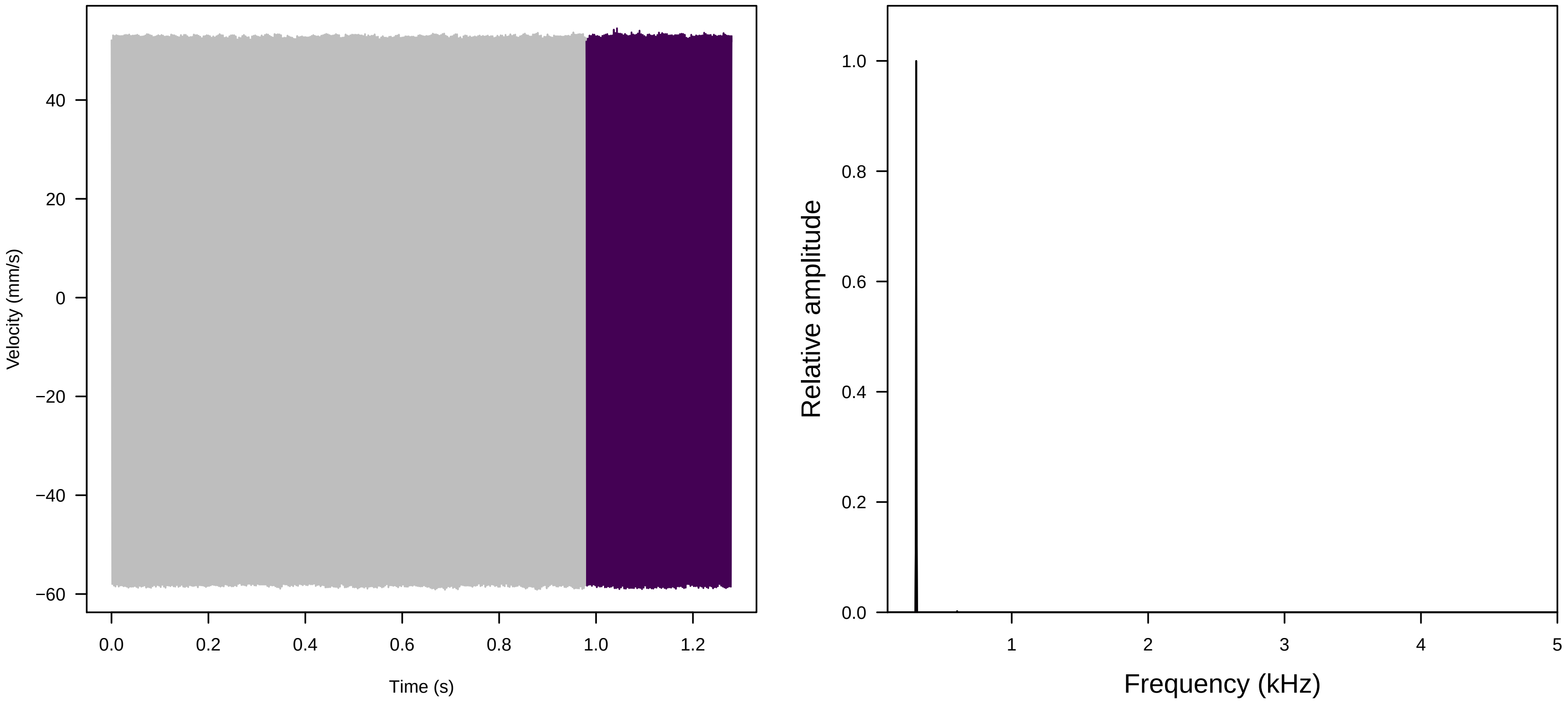

Vel. = 0.028 ; Str. = Receptacle ; Axis = z ; Fl. accession = 10−s−77−3

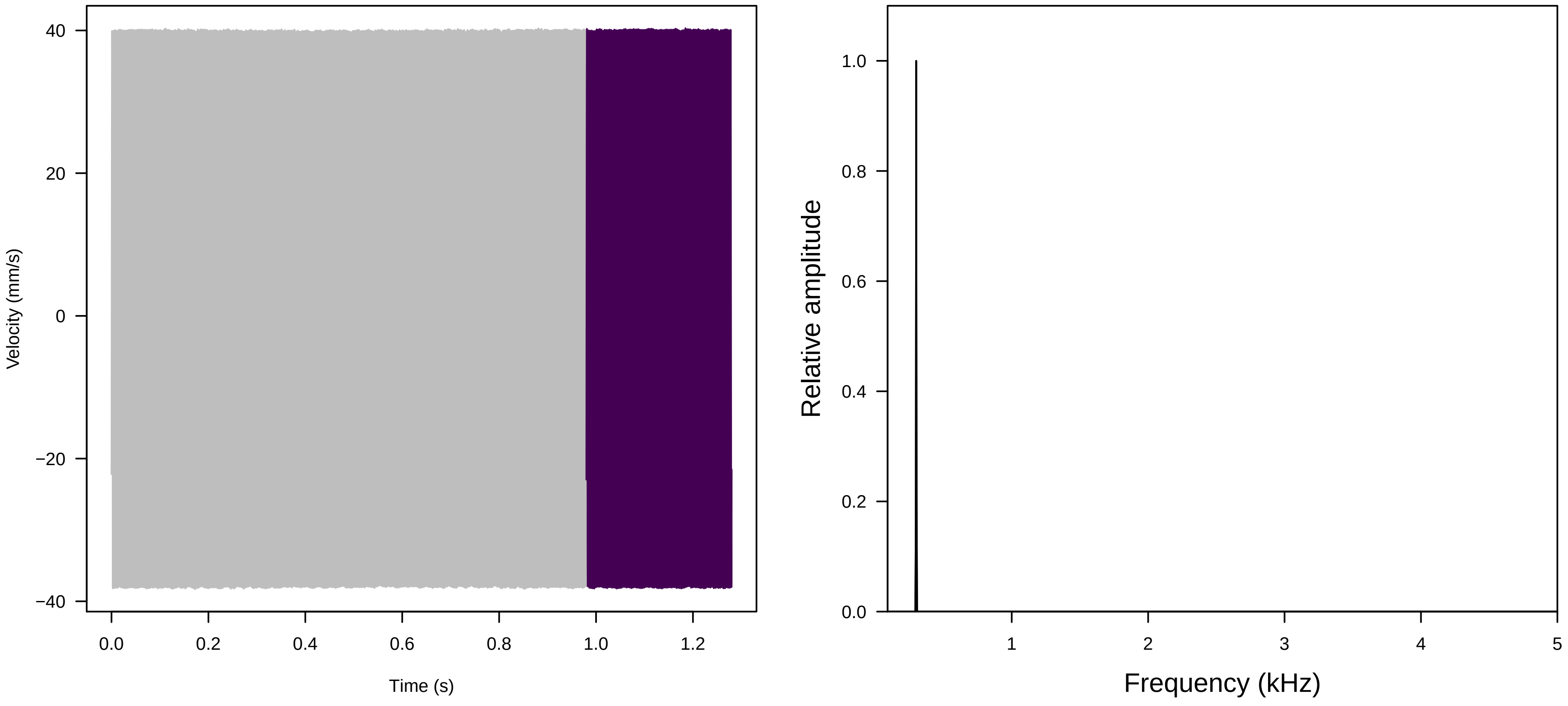

Vel. = 0.014 ; Str. = Corolla ; Axis = z ; Fl. accession = 10−s−77−3

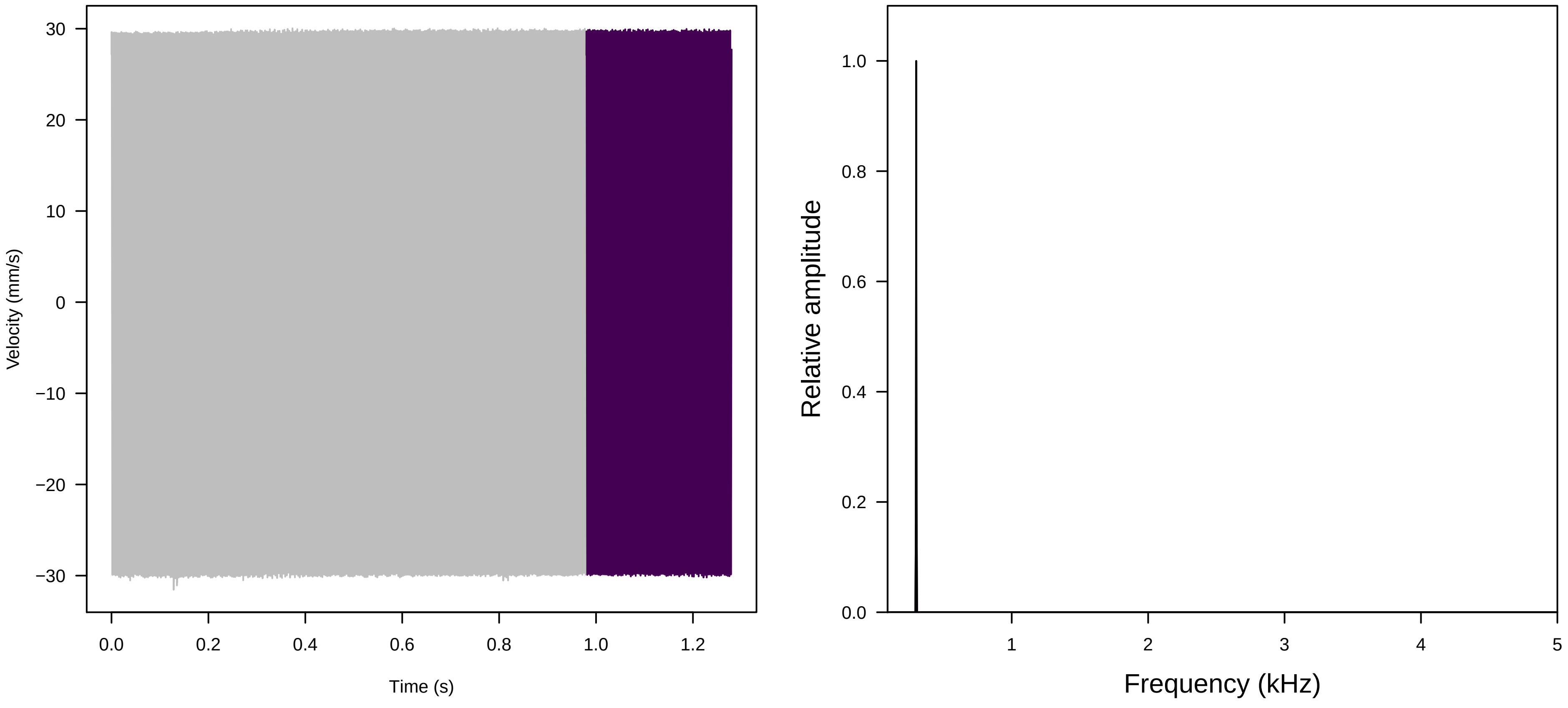

Vel. = 0.014 ; Str. = Receptacle ; Axis = z ; Fl. accession = 10−s−77−3

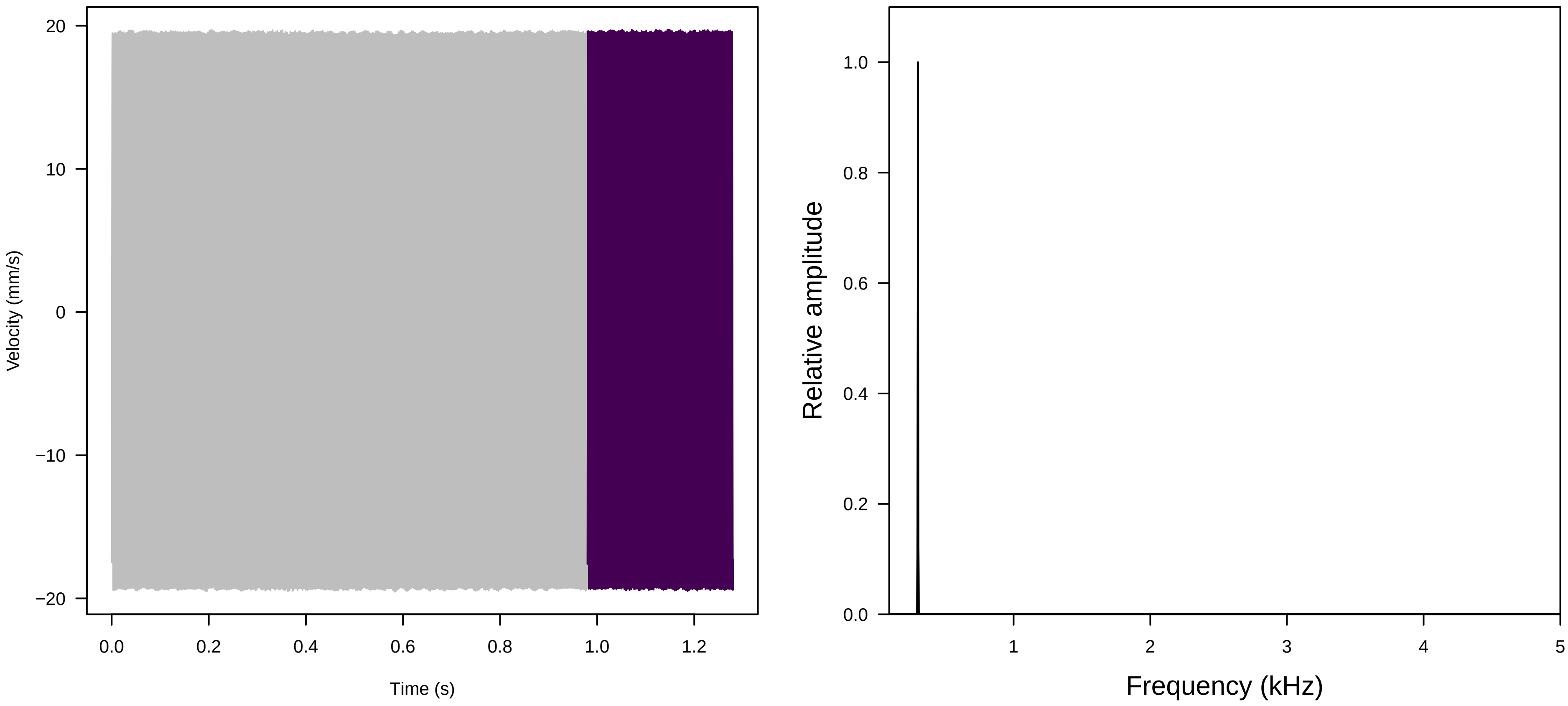

Vel. = 0.014 ; Str. = FA ; Axis = z ; Fl. accession = 10−s−77−3

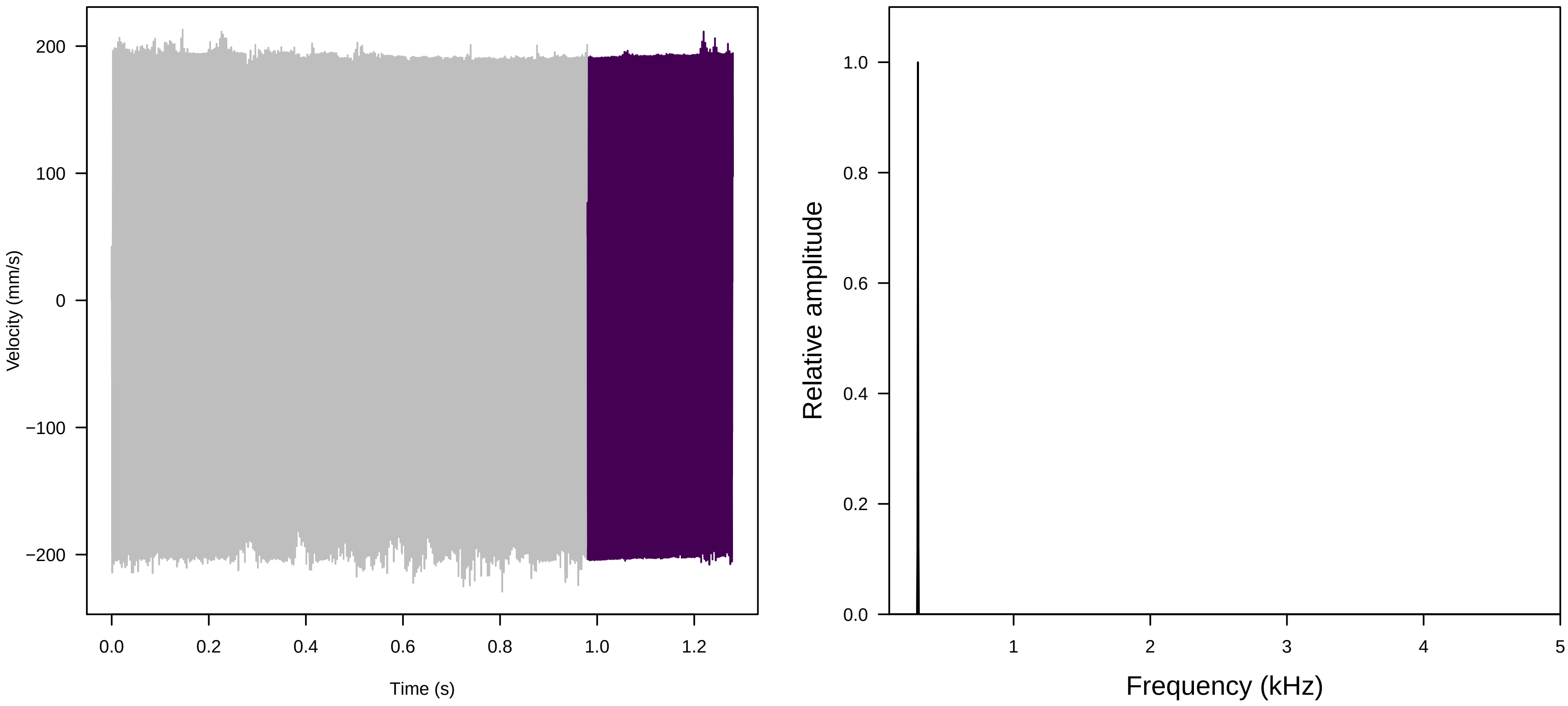

Vel. = 0.014 ; Str. = Receptacle ; Axis = z ; Fl. accession = 10−s−77−3

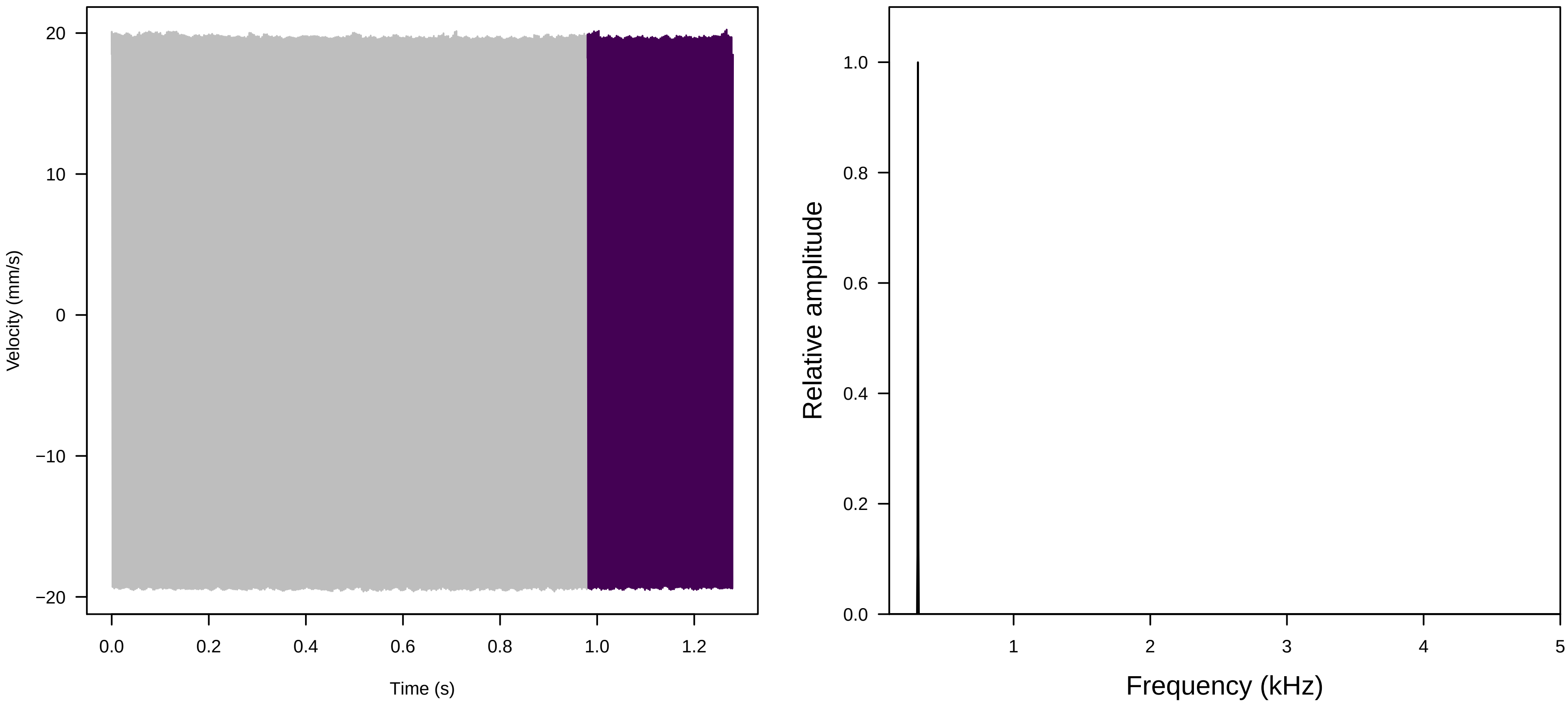

Vel. = 0.014 ; Str. = PA ; Axis = z ; Fl. accession = 10−s−77−3

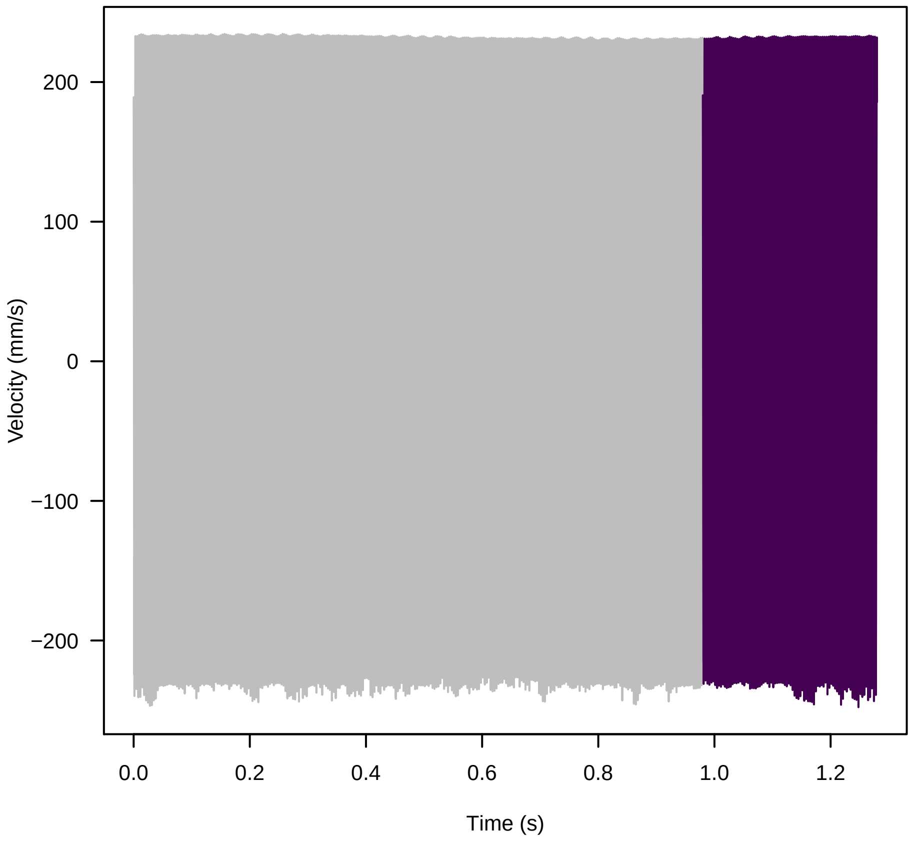

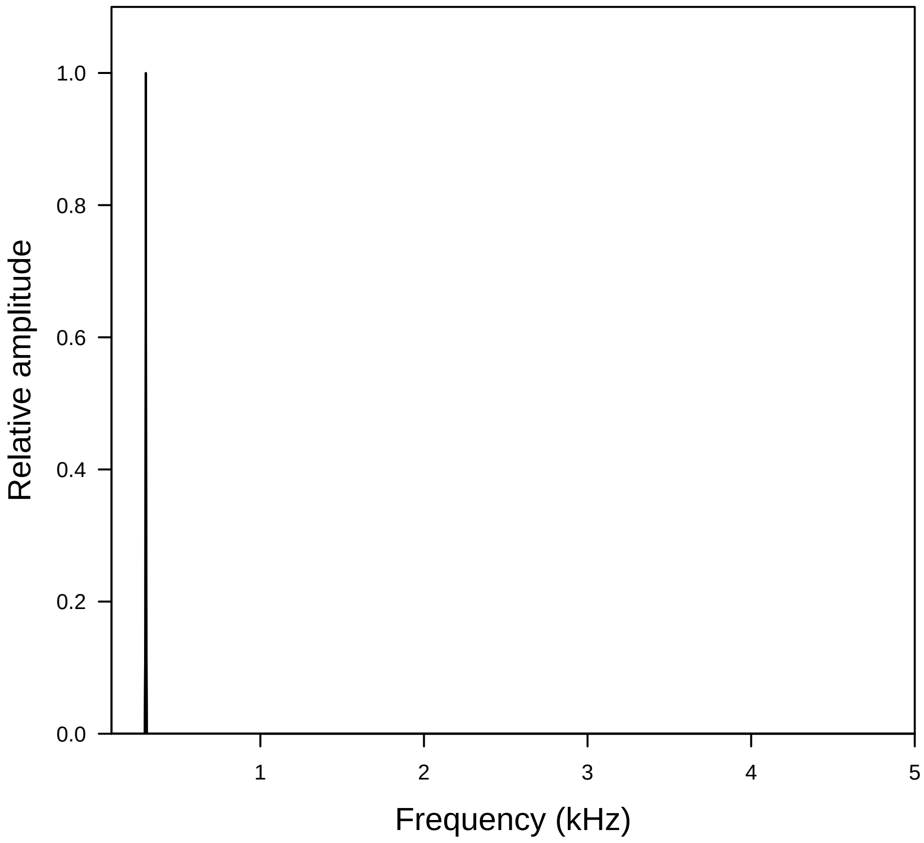

Vel. = 0.014 ; Str. = Receptacle ; Axis = z ; Fl. accession = 10−s−77−3

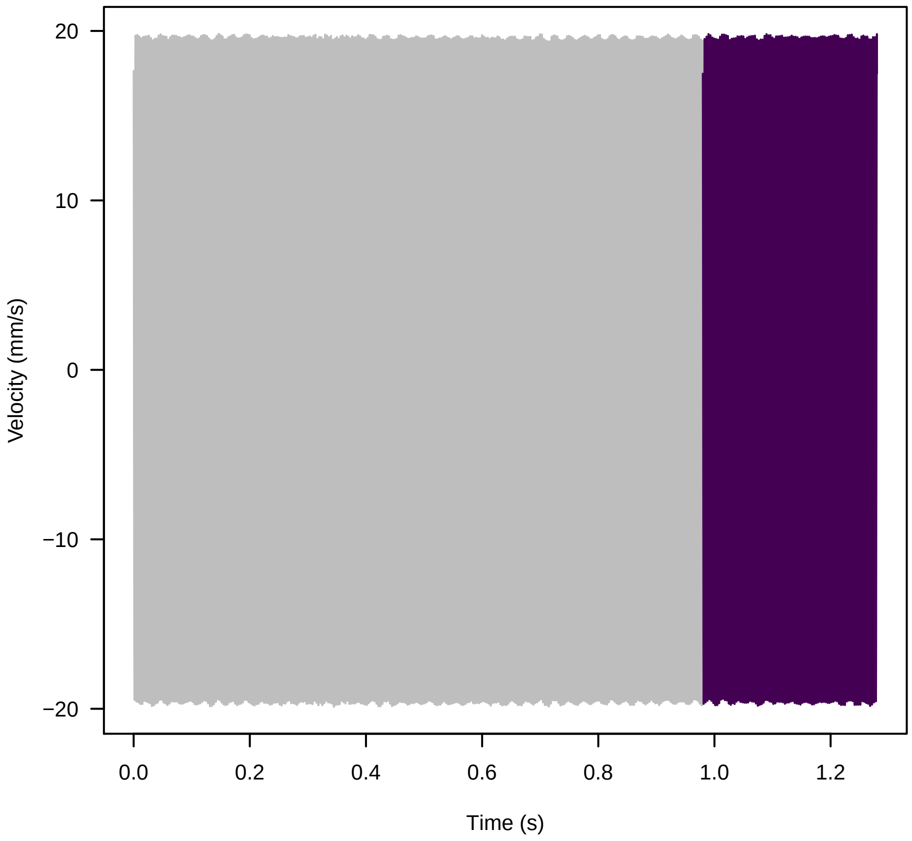

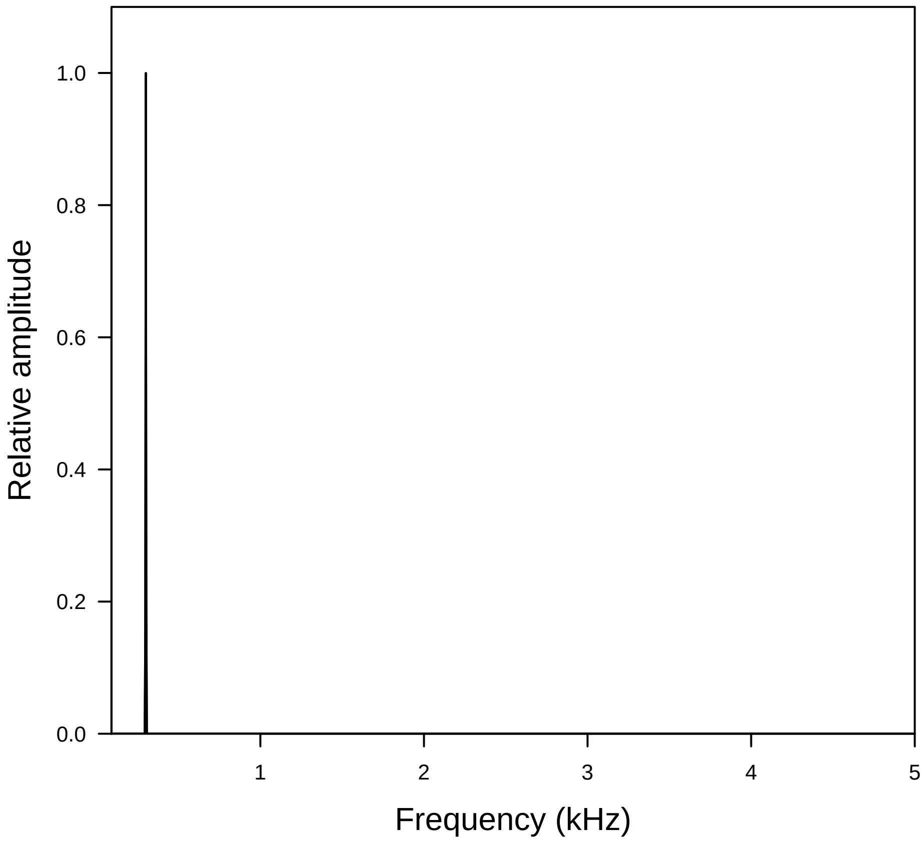

Vel. = 0.014 ; Str. = Corolla ; Axis = x ; Fl. accession = 10−s−77−3

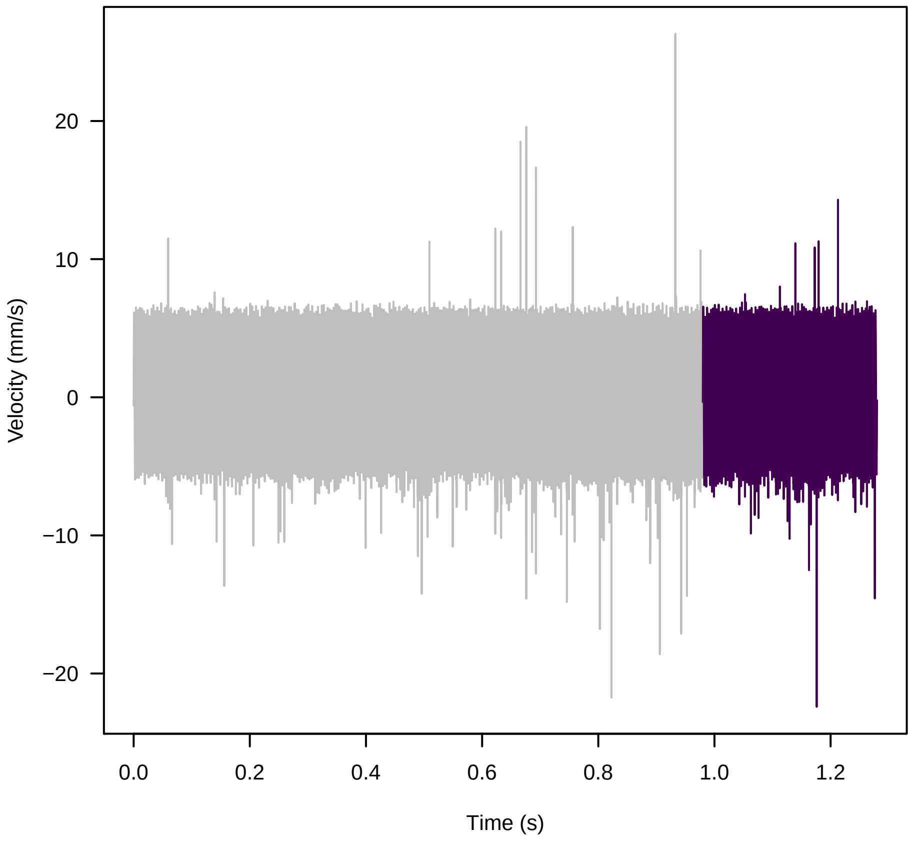

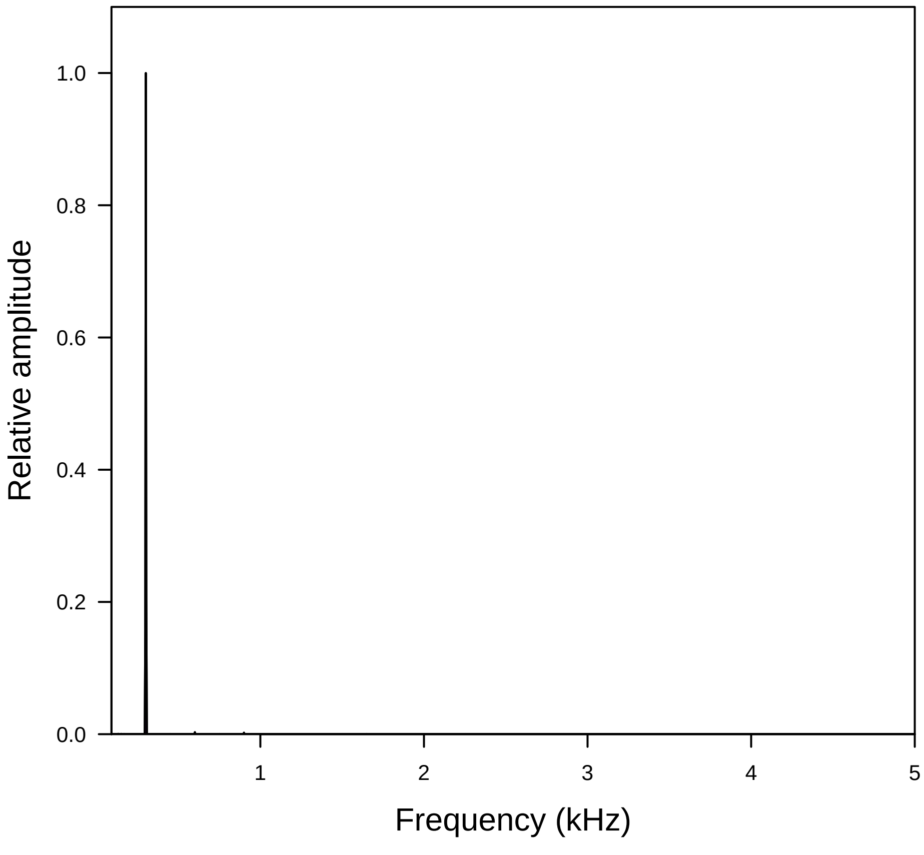

Vel. = 0.014 ; Str. = Receptacle ; Axis = x ; Fl. accession = 10−s−77−3

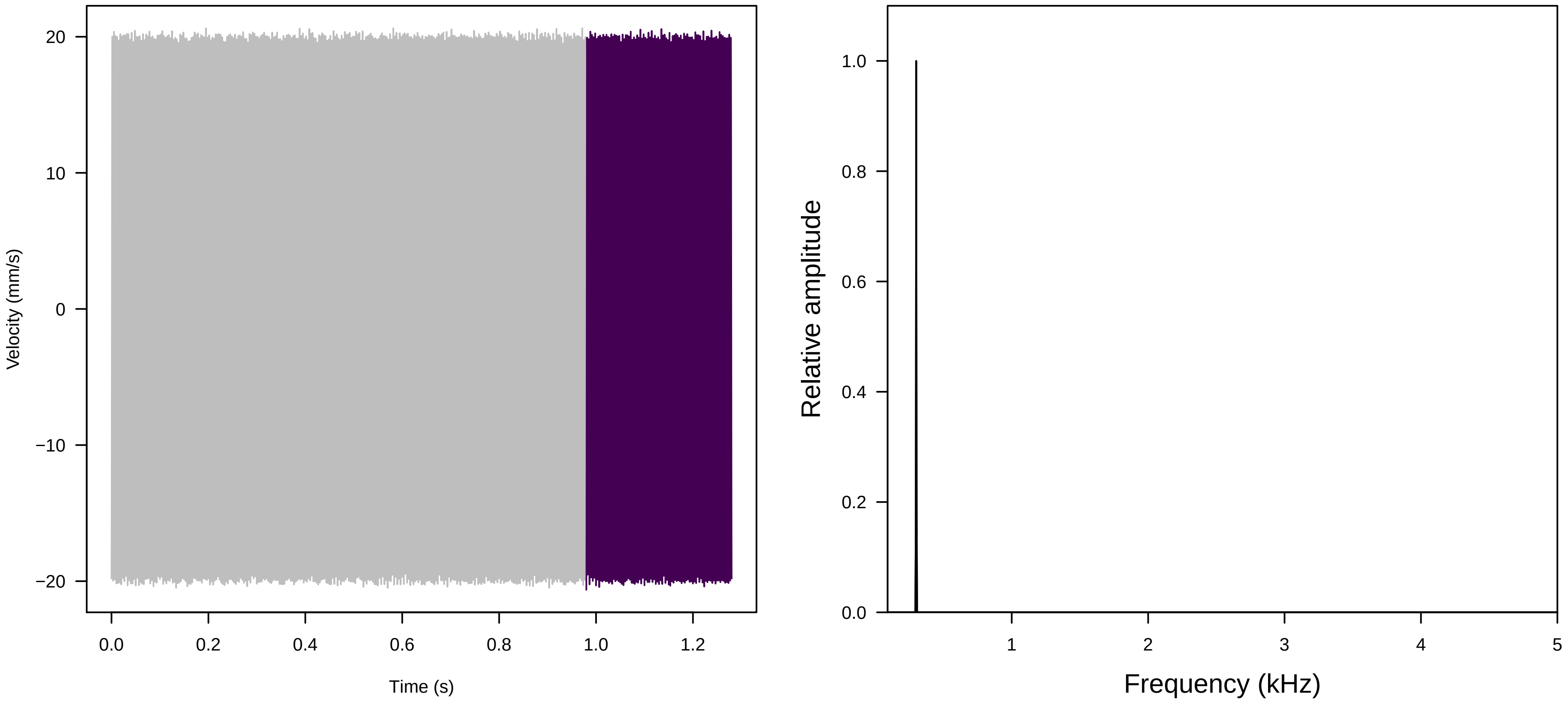

Vel. = 0.014 ; Str. = FA ; Axis = x ; Fl. accession = 10−s−77−3

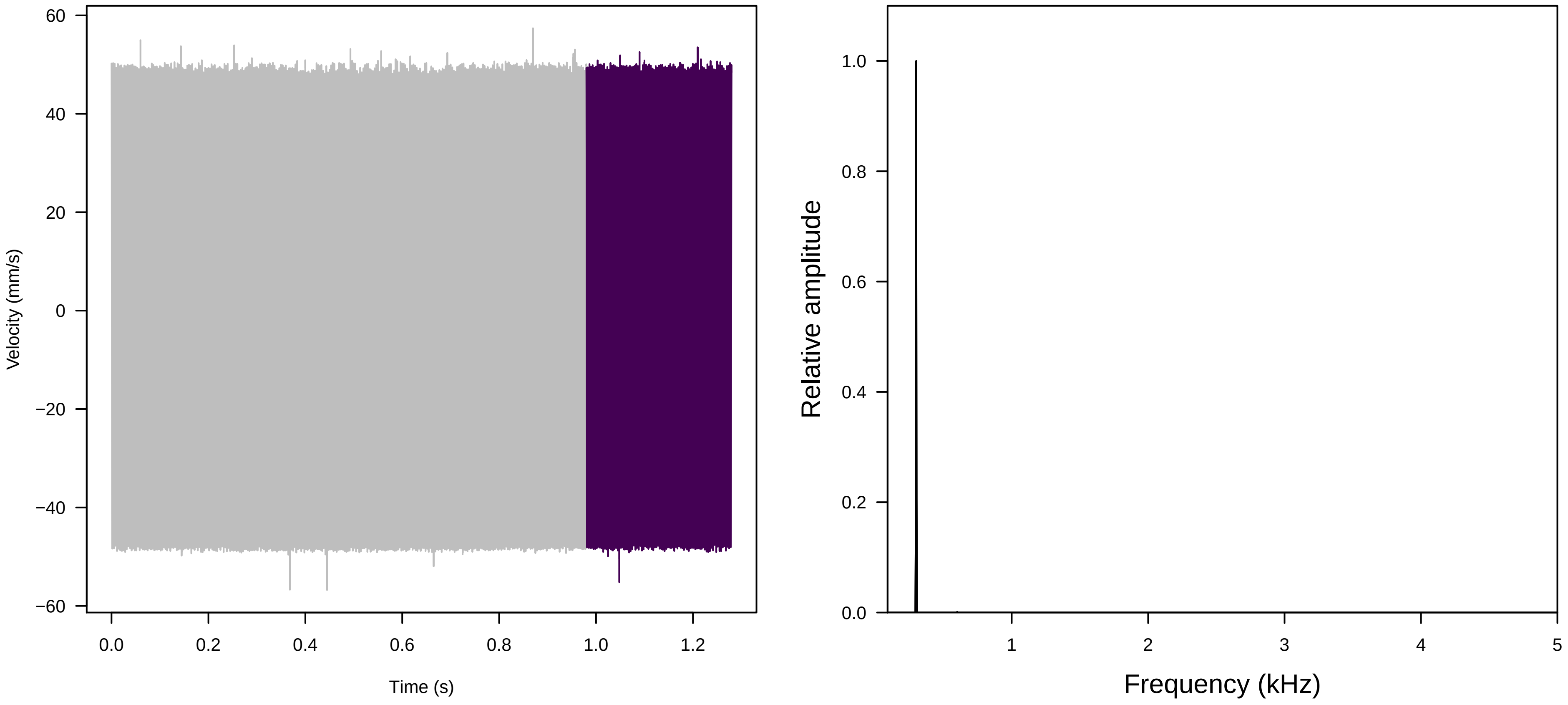

Vel. = 0.014 ; Str. = Receptacle ; Axis = x ; Fl. accession = 10−s−77−3

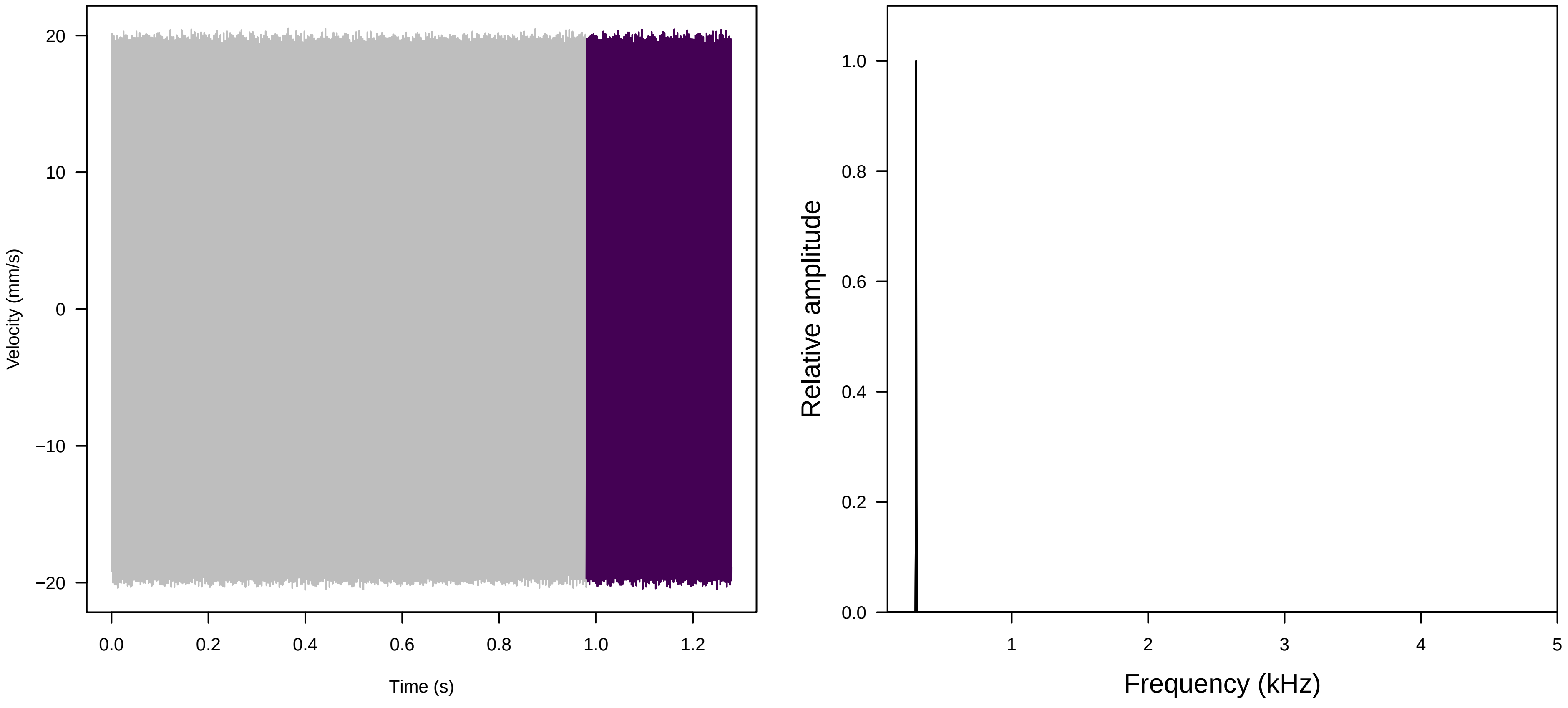

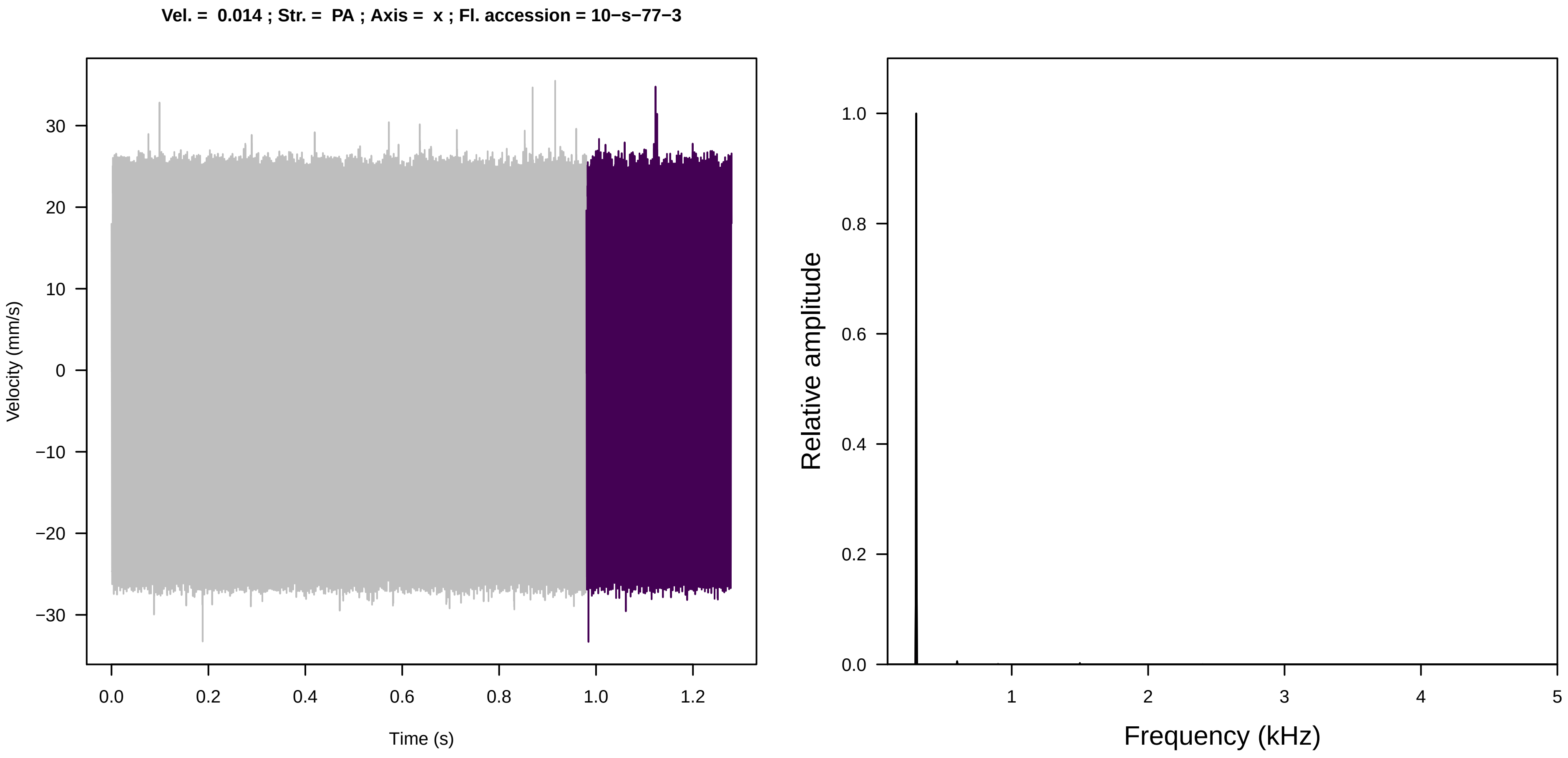
Vel. = 0.014 ; Str. = PA ; Axis = x ; Fl. accession = 10−s−77−3
Velocity (mm/s)
Time (s)
Relative amplitude
Frequency (kHz)

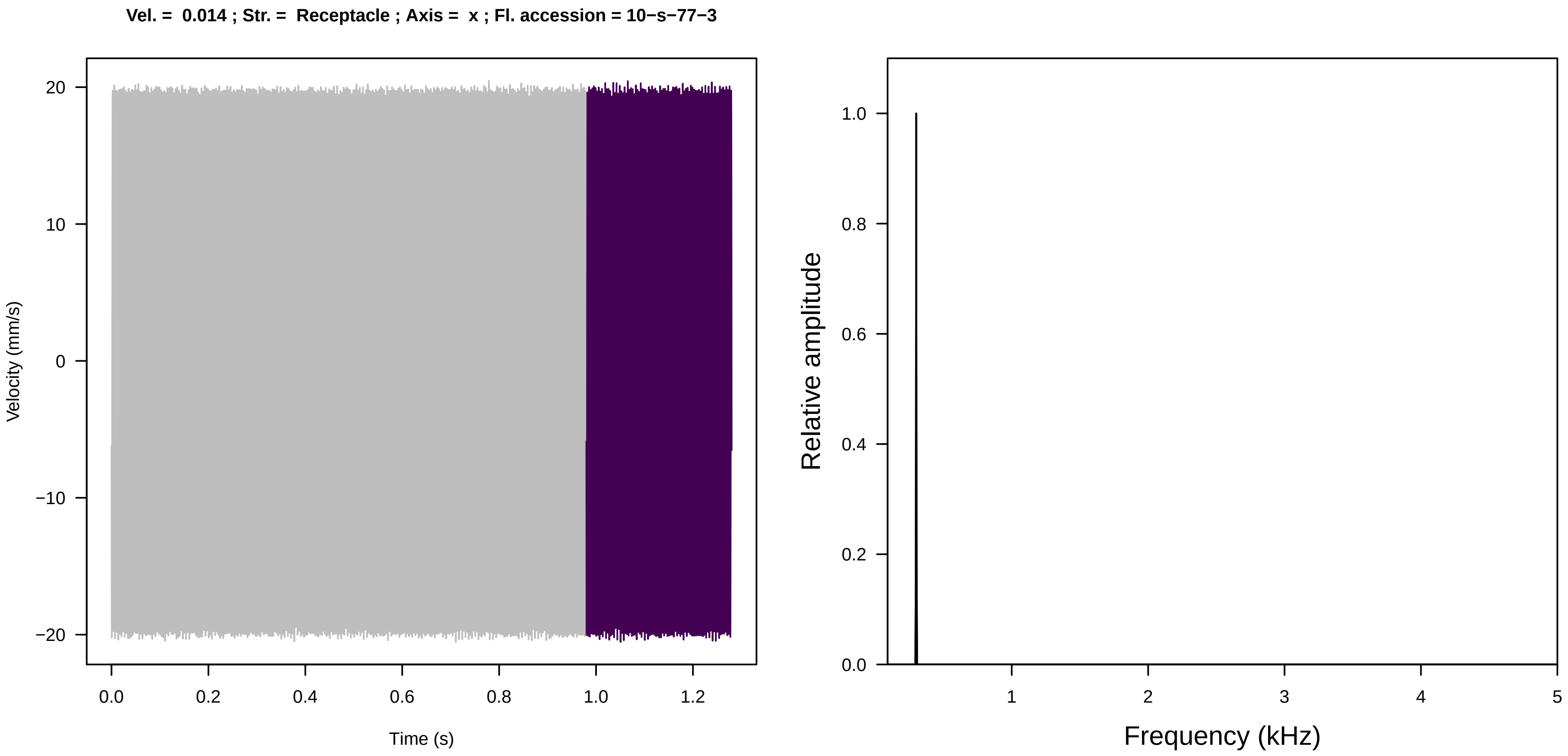
Vel. = 0.014 ; Str. = Receptacle ; Axis = x ; Fl. accession = 10−s−77−3
Velocity (mm/s)
Time (s)
Relative amplitude
Frequency (kHz)

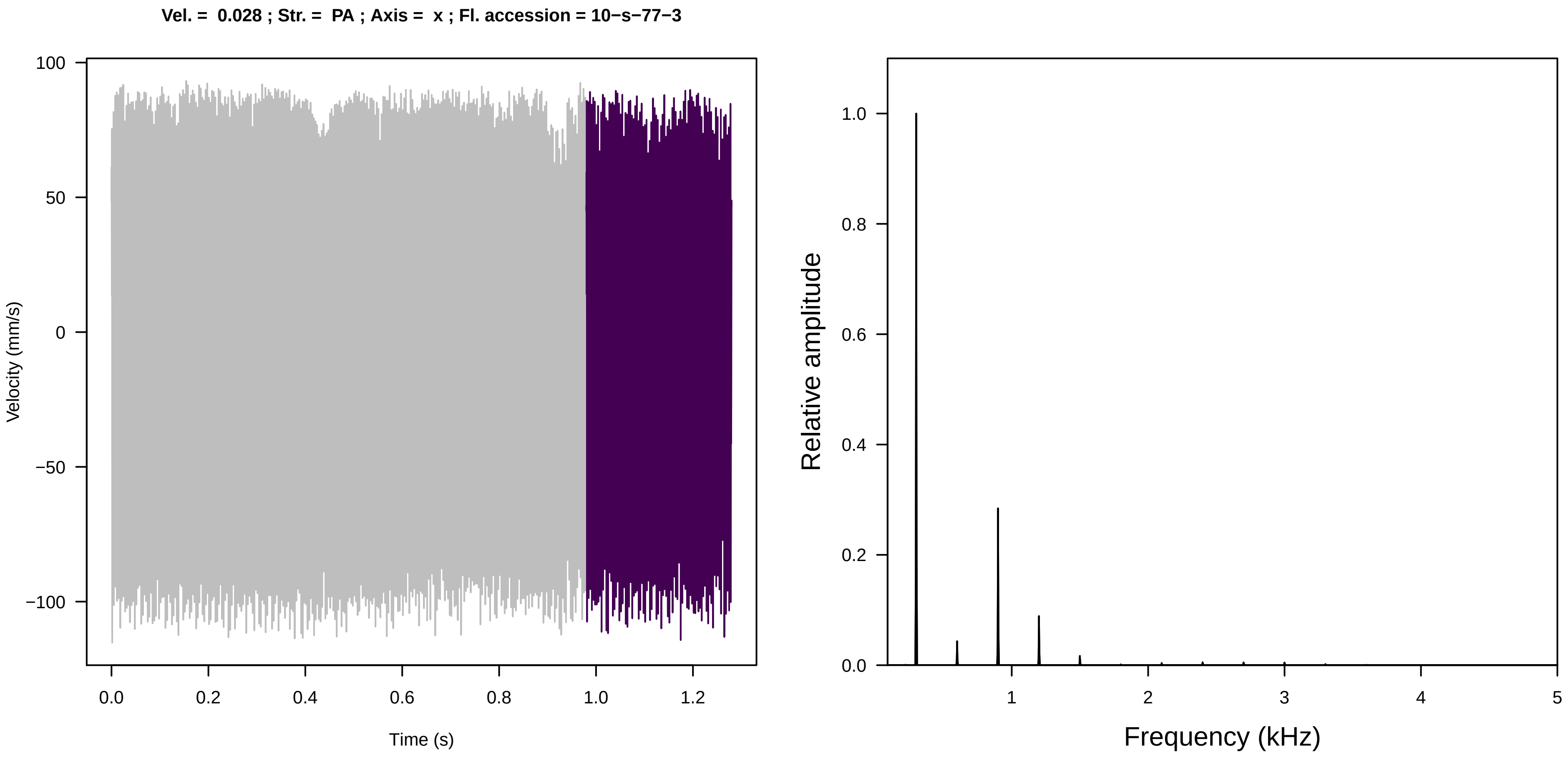
Vel. = 0.028 ; Str. = PA ; Axis = x ; Fl. accession = 10−s−77−3
Velocity (mm/s)
Time (s)
Relative amplitude
Frequency (kHz)

Vel. = 0.028 ; Str. = Receptacle ; Axis = x ; Fl. accession = 10−s−77−3

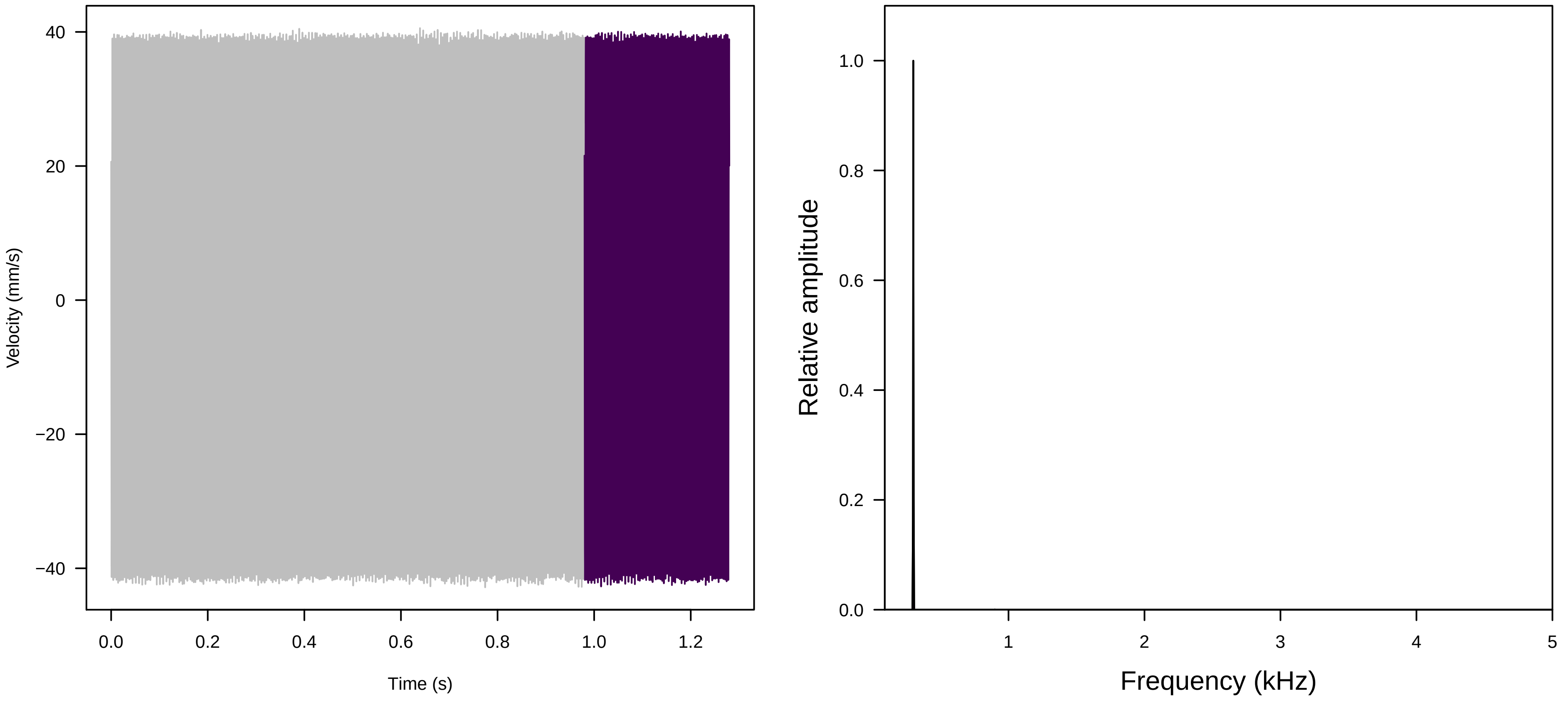

Vel. = 0.028 ; Str. = FA ; Axis = x ; Fl. accession = 10−s−77−3

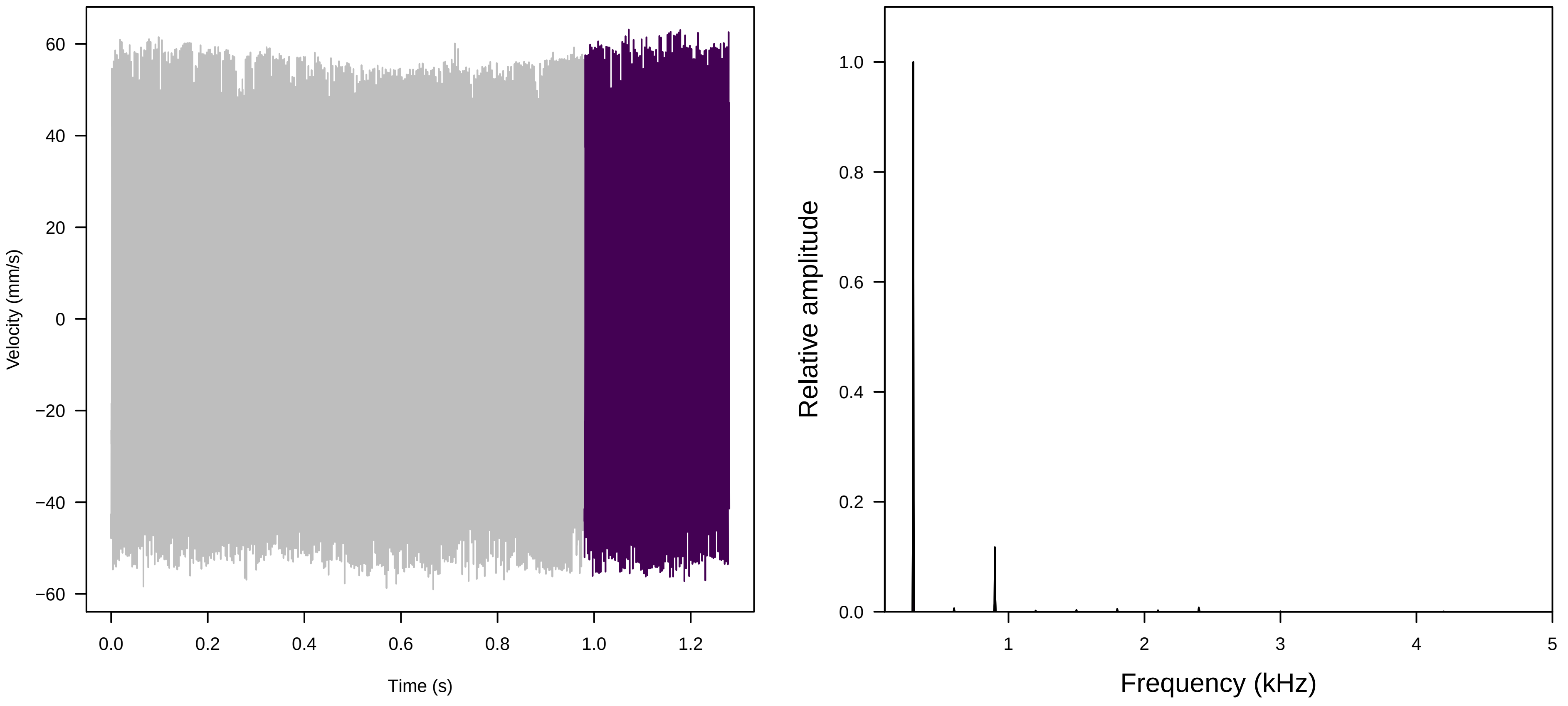

Vel. = 0.028 ; Str. = Receptacle ; Axis = x ; Fl. accession = 10−s−77−3

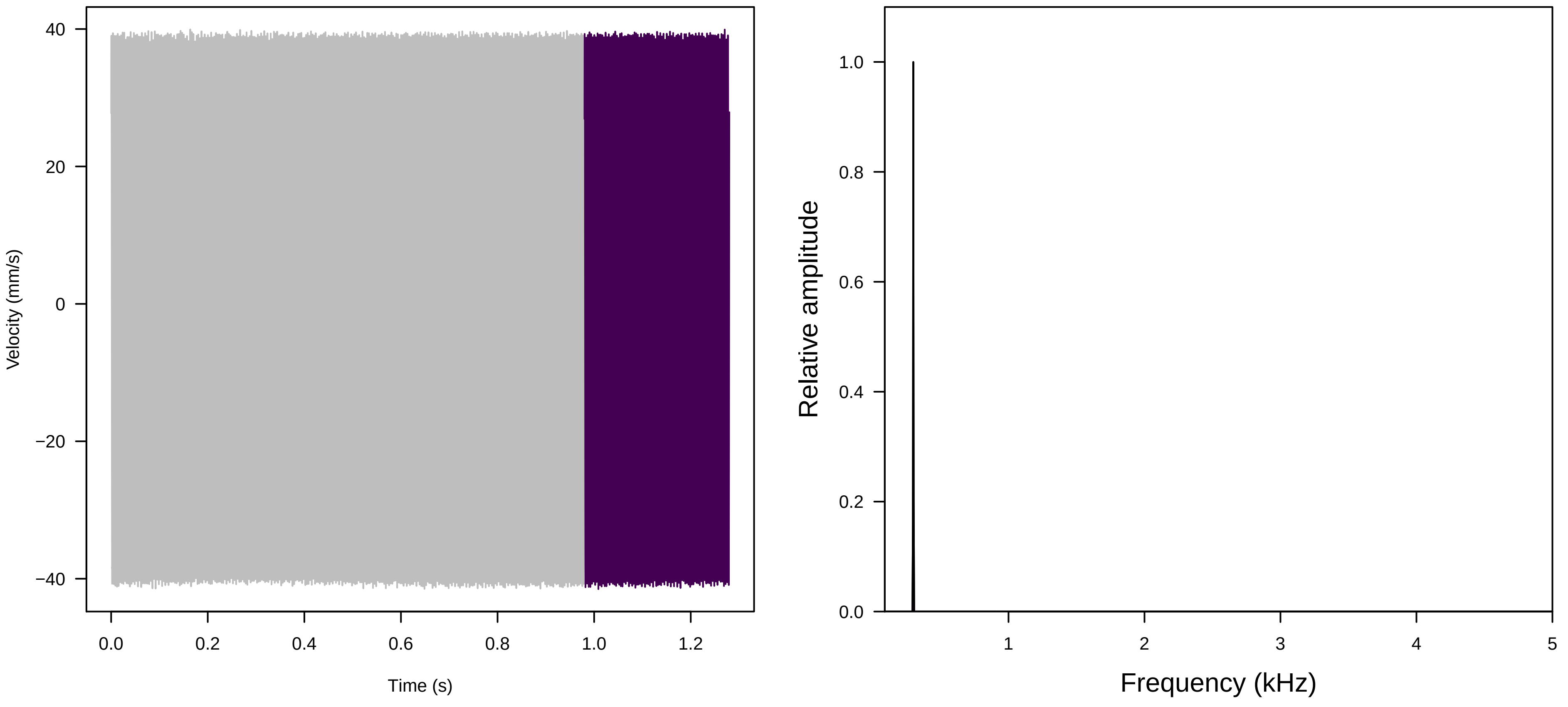

Vel. = 0.028 ; Str. = Corolla ; Axis = x ; Fl. accession = 10−s−77−3

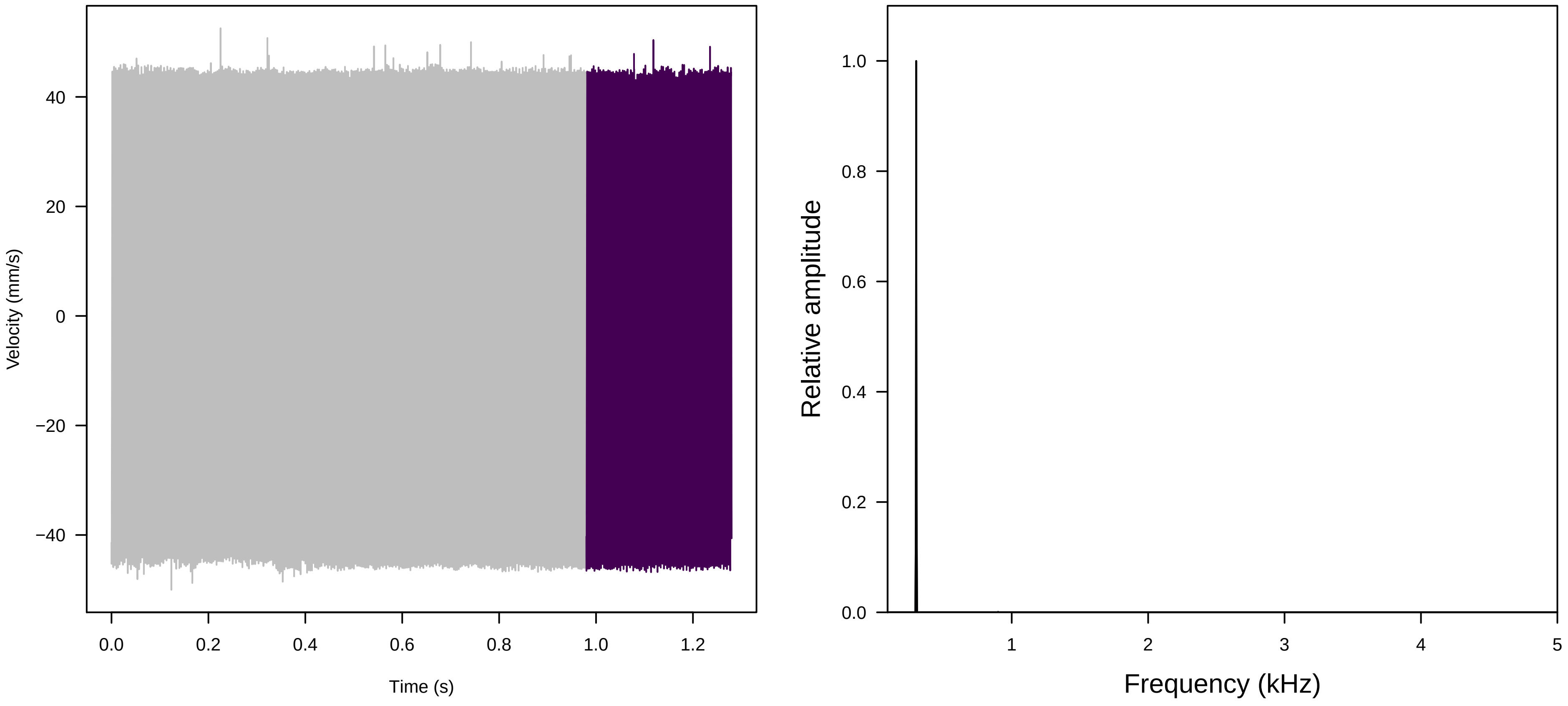

Vel. = 0.028 ; Str. = Receptacle ; Axis = x ; Fl. accession = 10−s−77−3

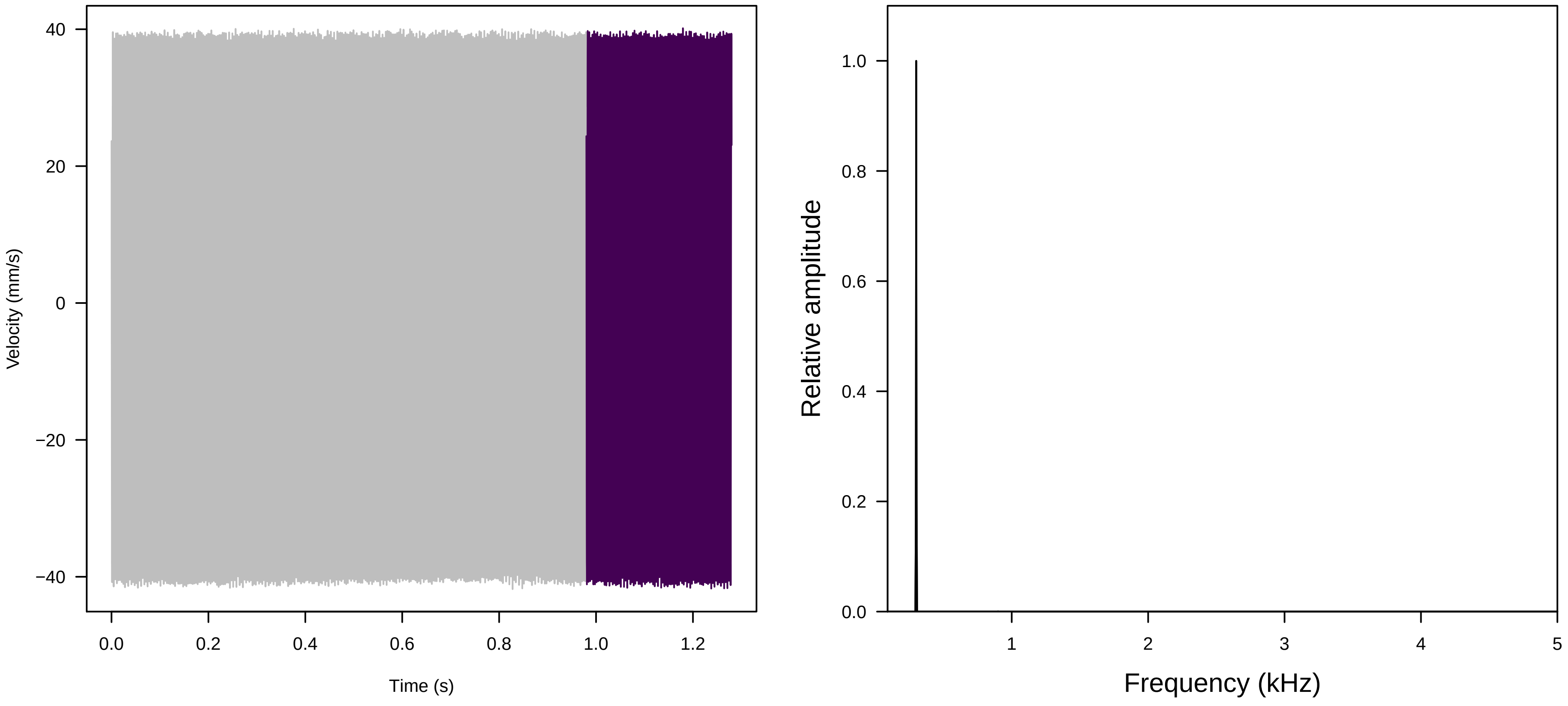

Vel. = 0.057 ; Str. = Corolla ; Axis = x ; Fl. accession = 10−s−77−3

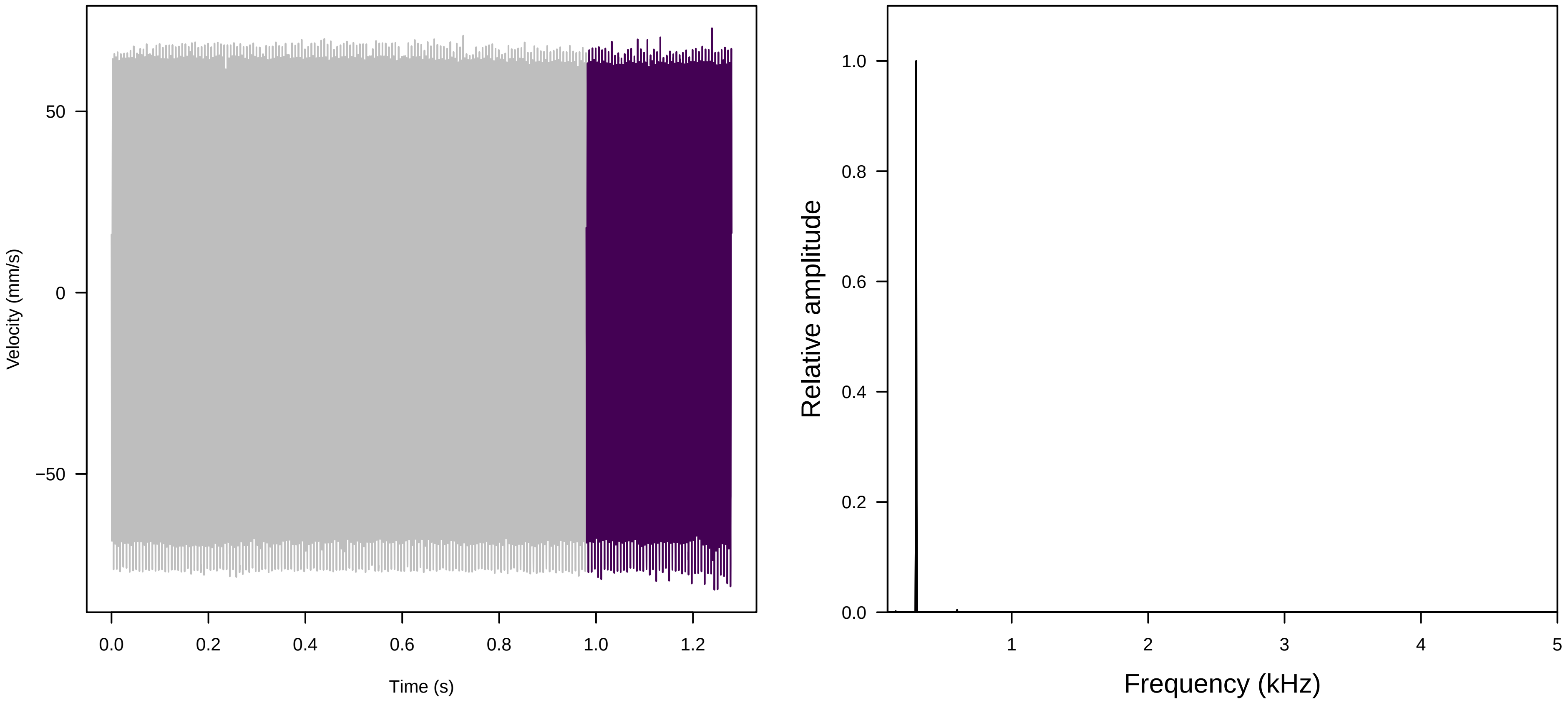

Vel. = 0.057 ; Str. = Receptacle ; Axis = x ; Fl. accession = 10−s−77−3

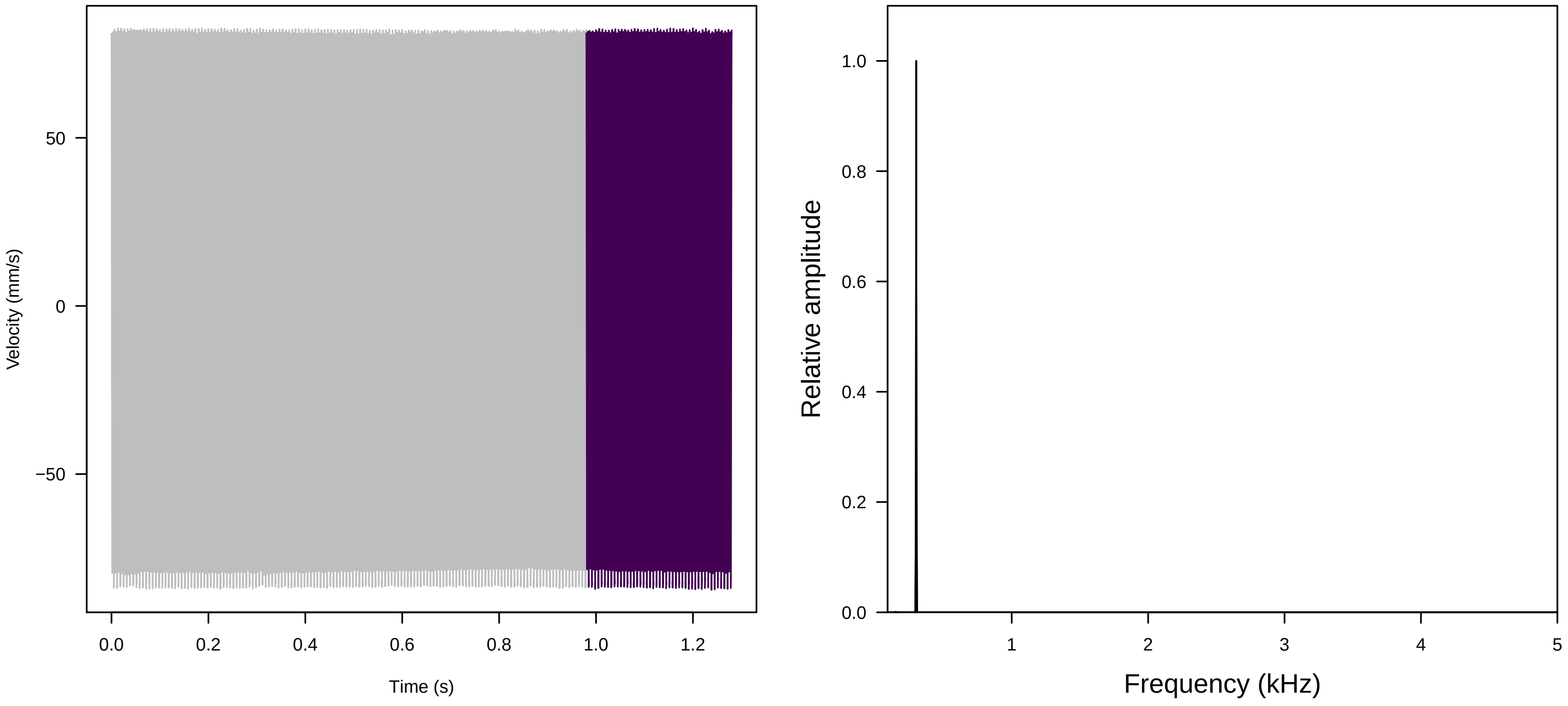

Vel. = 0.057 ; Str. = FA ; Axis = x ; Fl. accession = 10−s−77−3

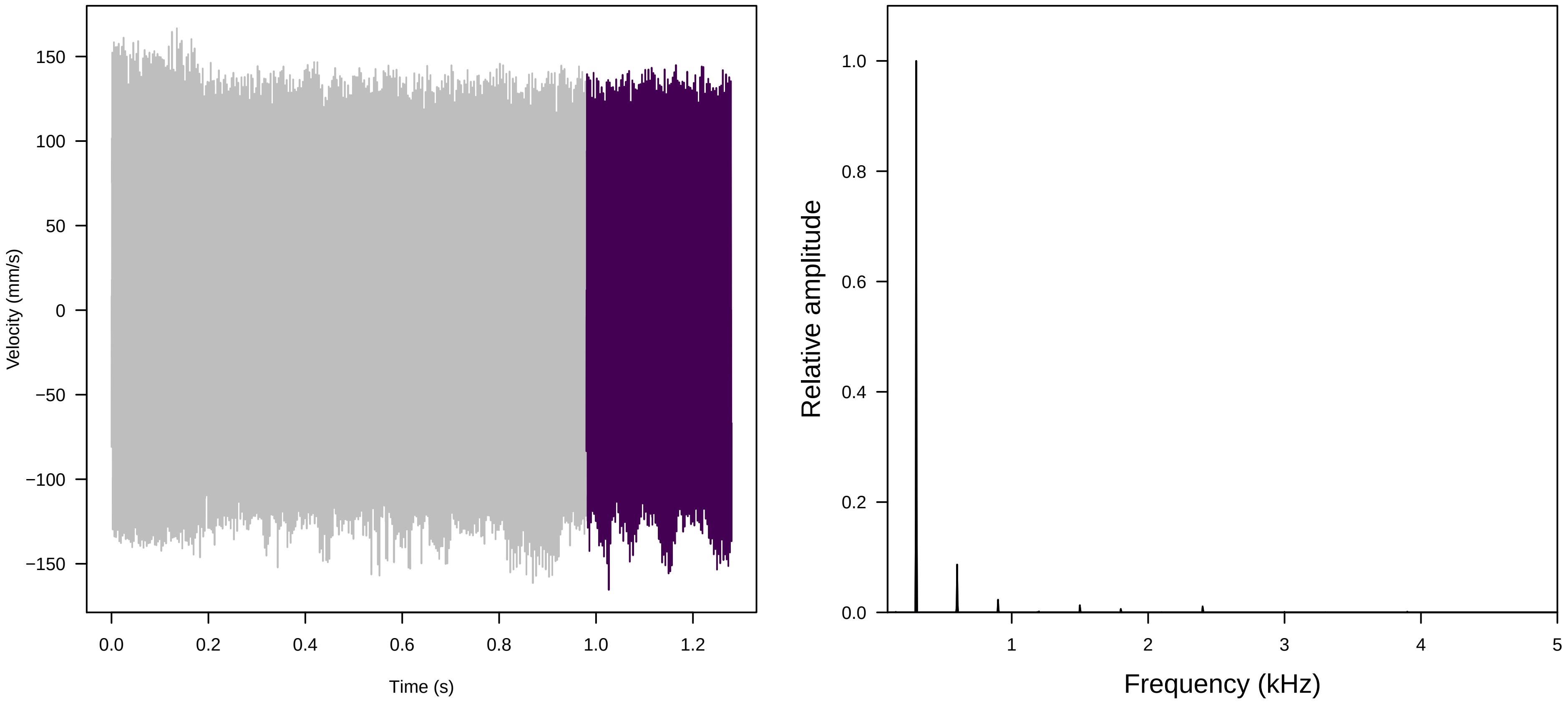

Vel. = 0.057 ; Str. = Receptacle ; Axis = x ; Fl. accession = 10−s−77−3

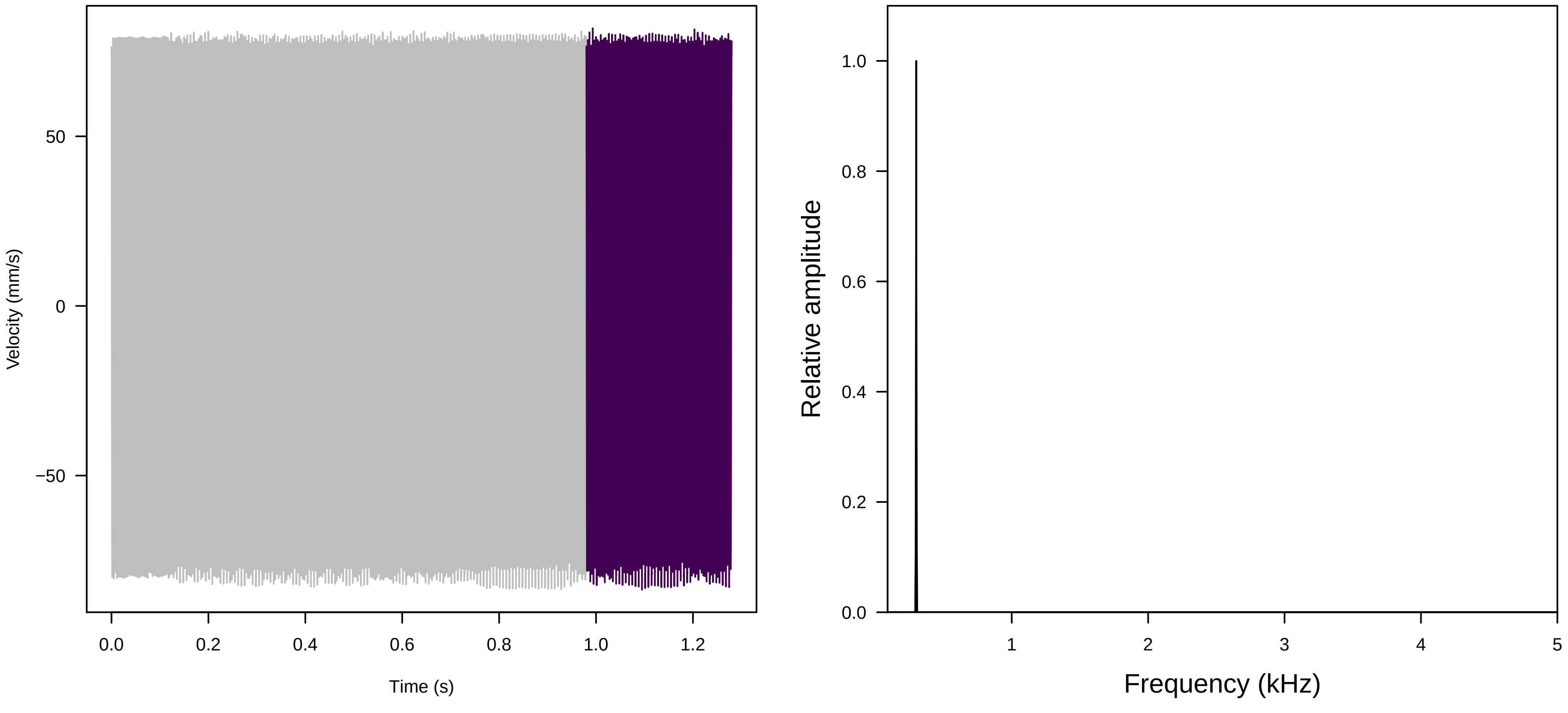

Vel. = 0.057 ; Str. = PA ; Axis = x ; Fl. accession = 10−s−77−3

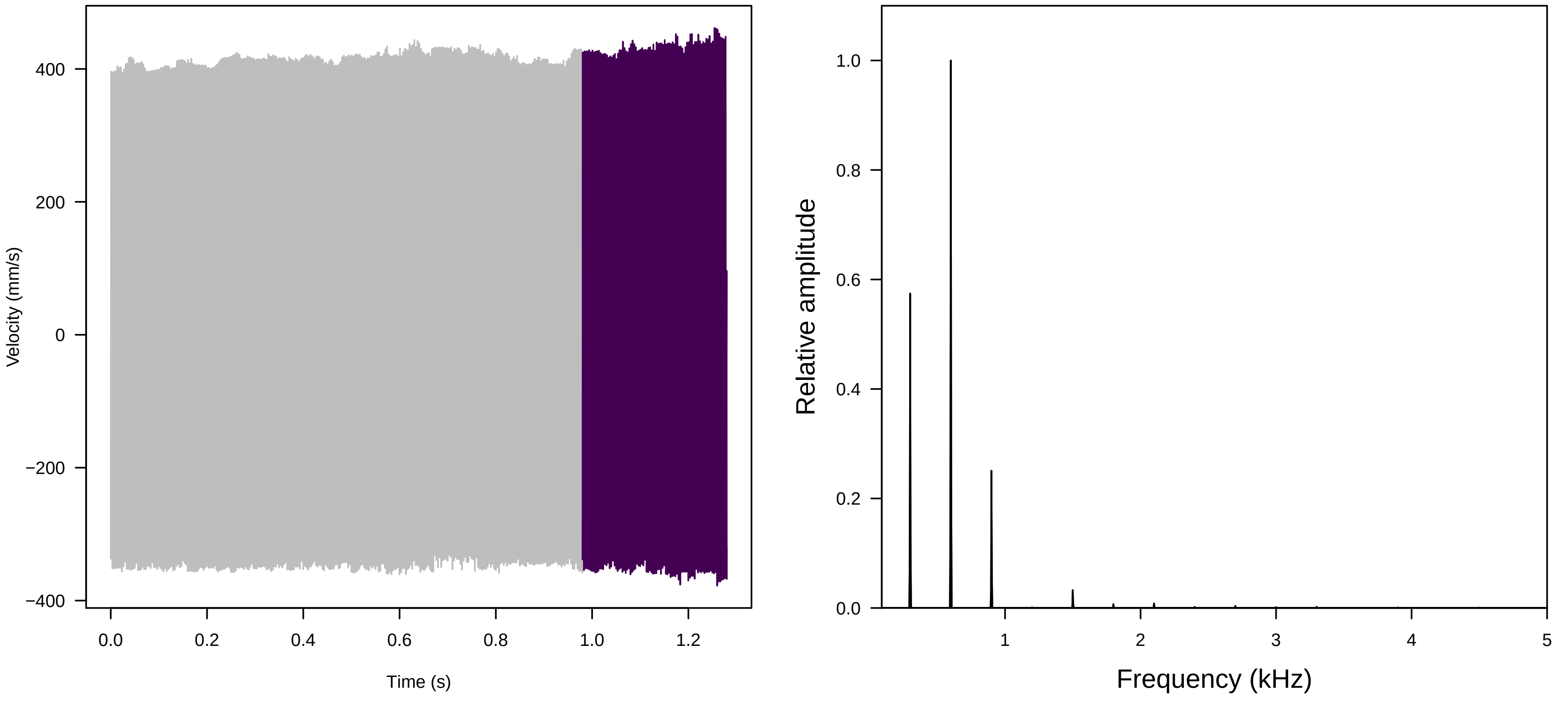

Vel. = 0.057 ; Str. = Receptacle ; Axis = x ; Fl. accession = 10−s−77−3

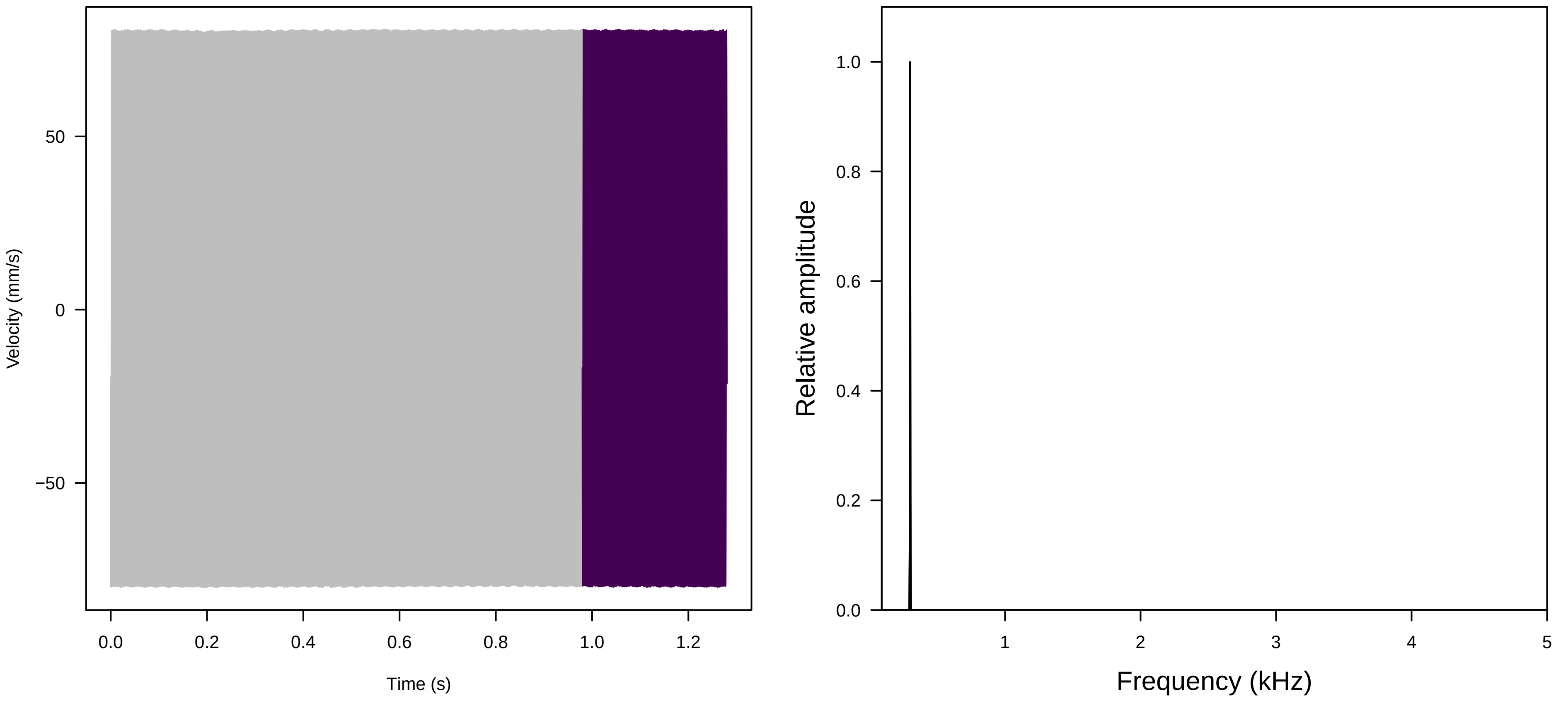

Vel. = 0.014 ; Str. = Corolla ; Axis = x ; Fl. accession = 10−s−79−27

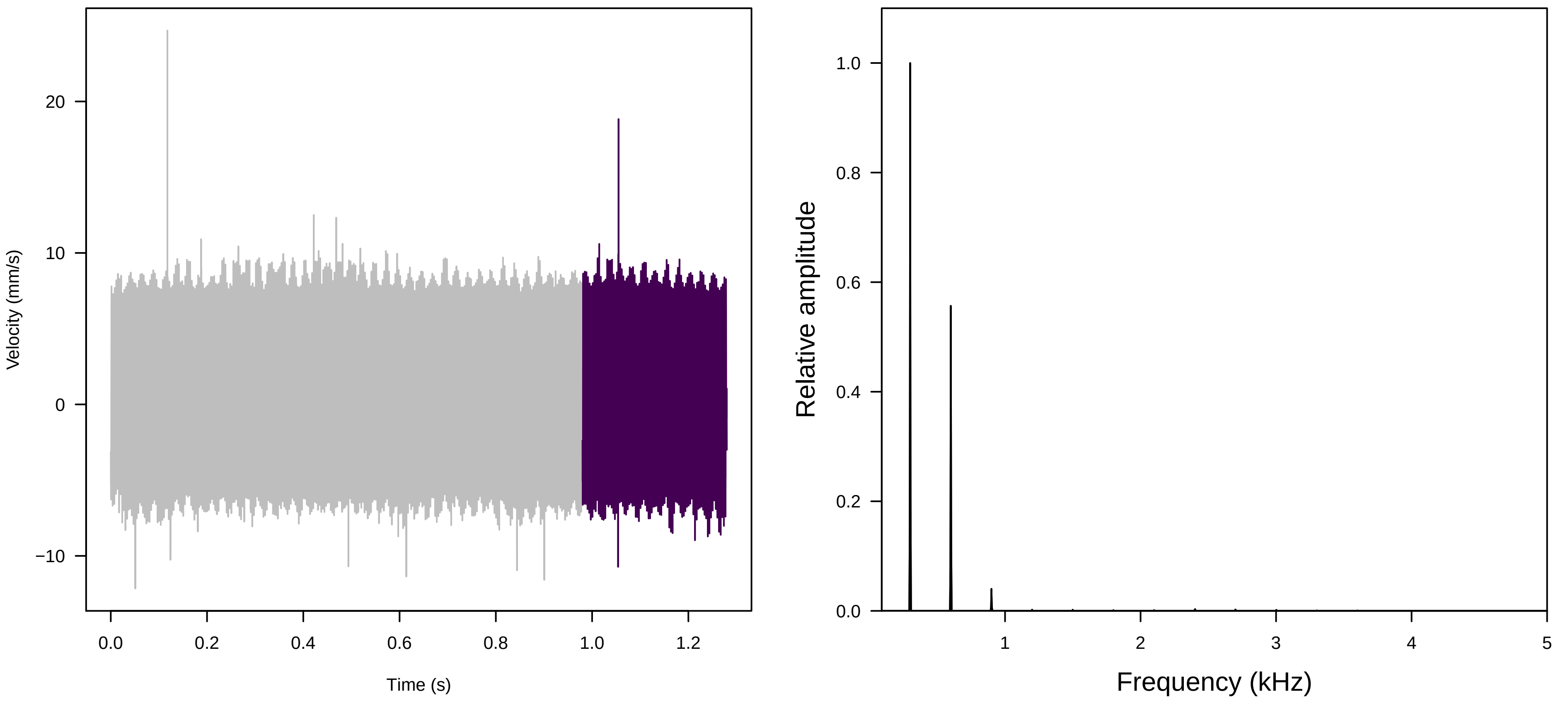

Vel. = 0.014 ; Str. = Receptacle ; Axis = x ; Fl. accession = 10−s−79−27

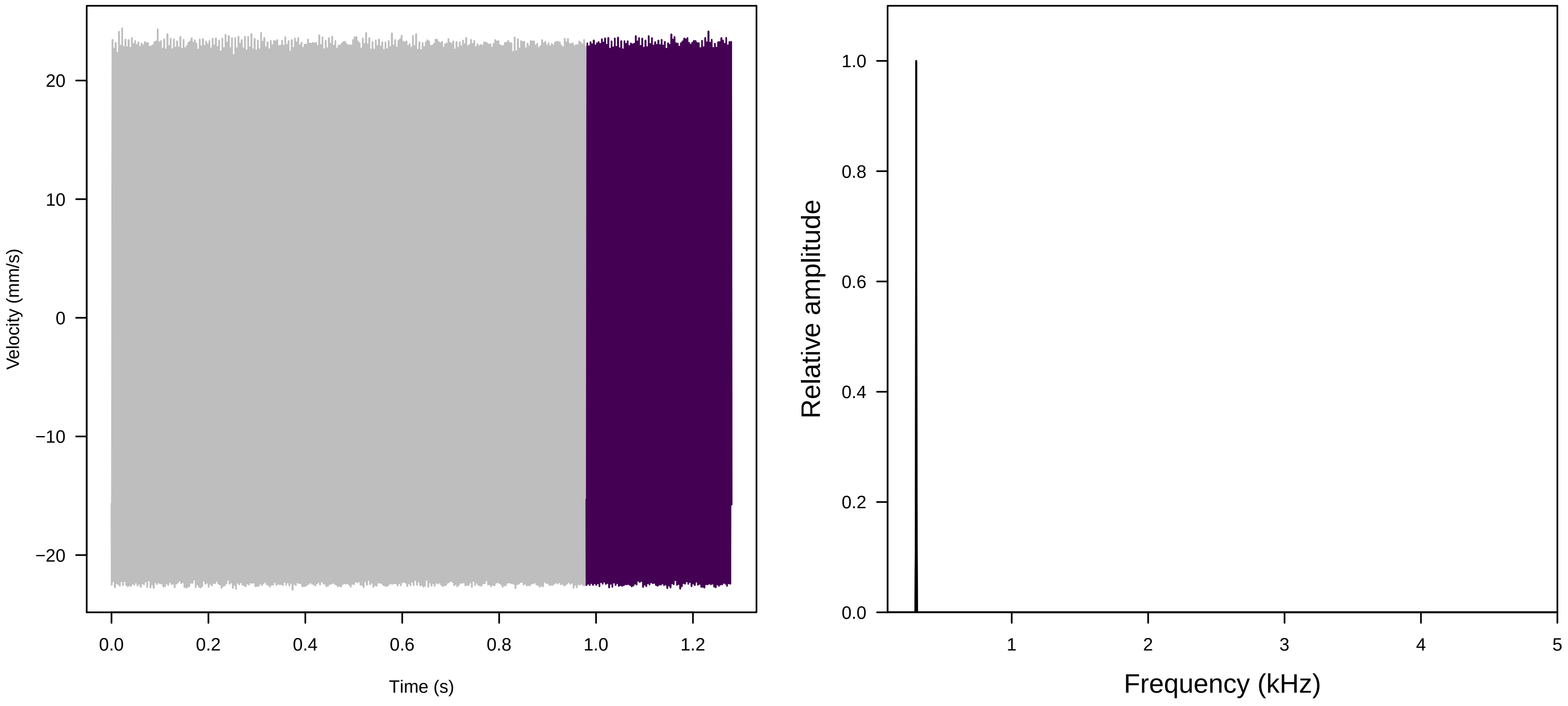

Vel. = 0.014 ; Str. = FA ; Axis = x ; Fl. accession = 10−s−79−27

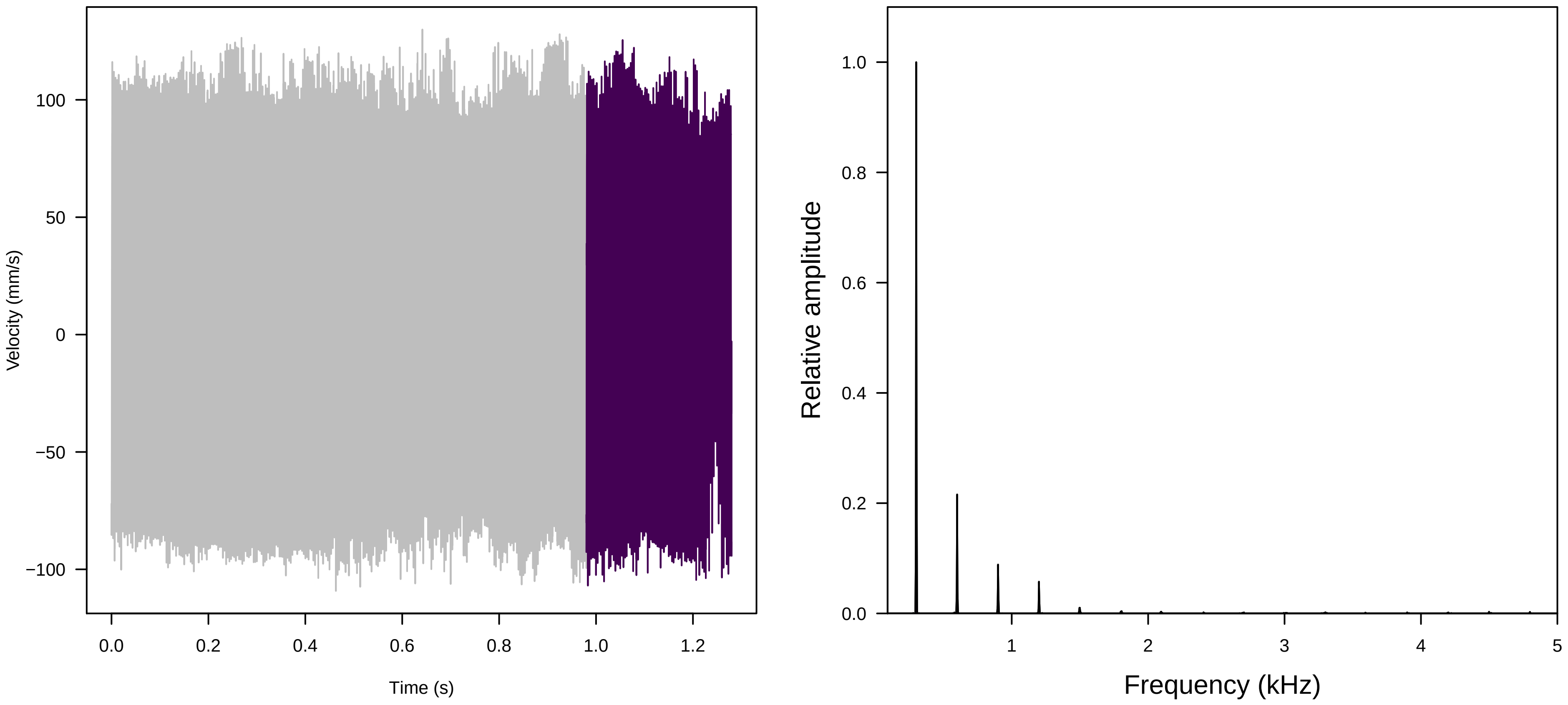

Vel. = 0.014 ; Str. = Receptacle ; Axis = x ; Fl. accession = 10−s−79−27

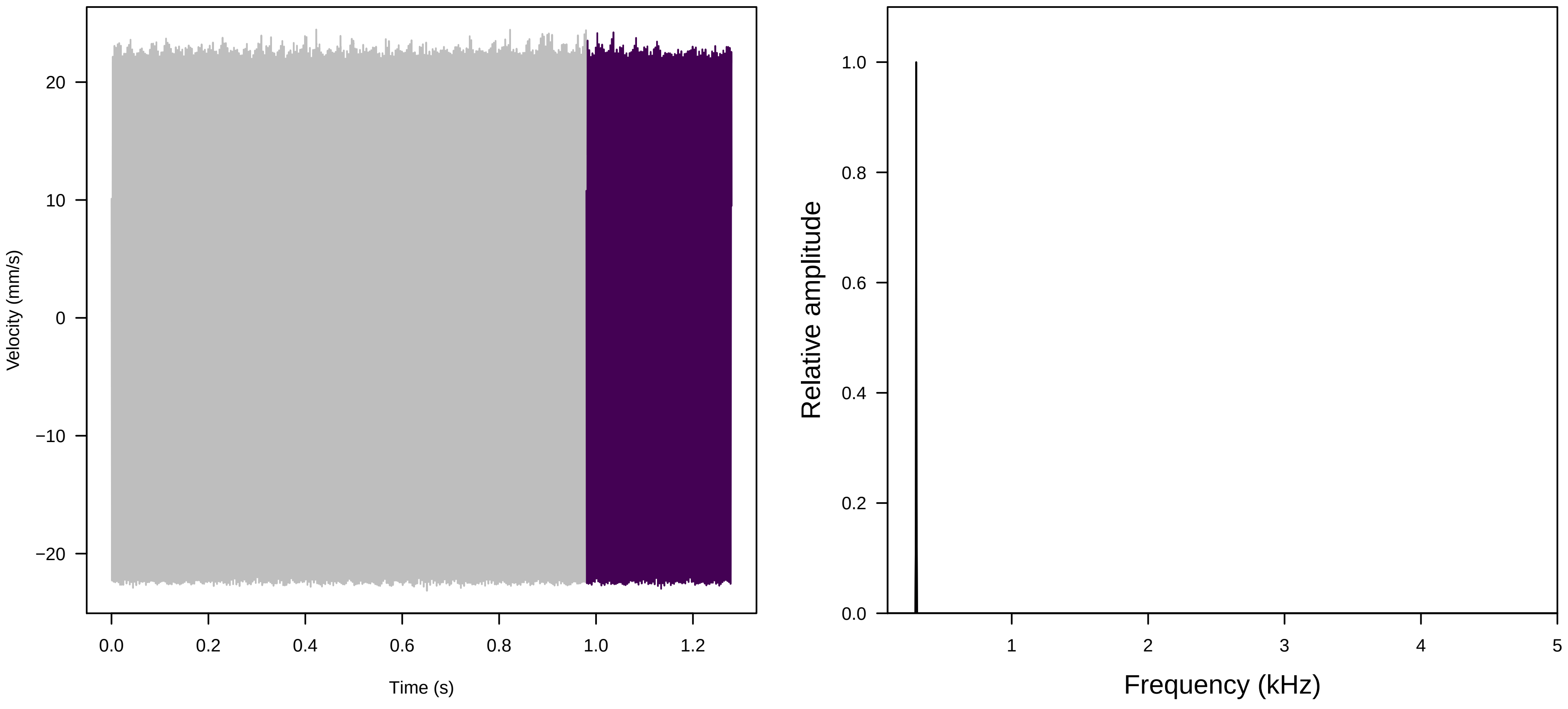

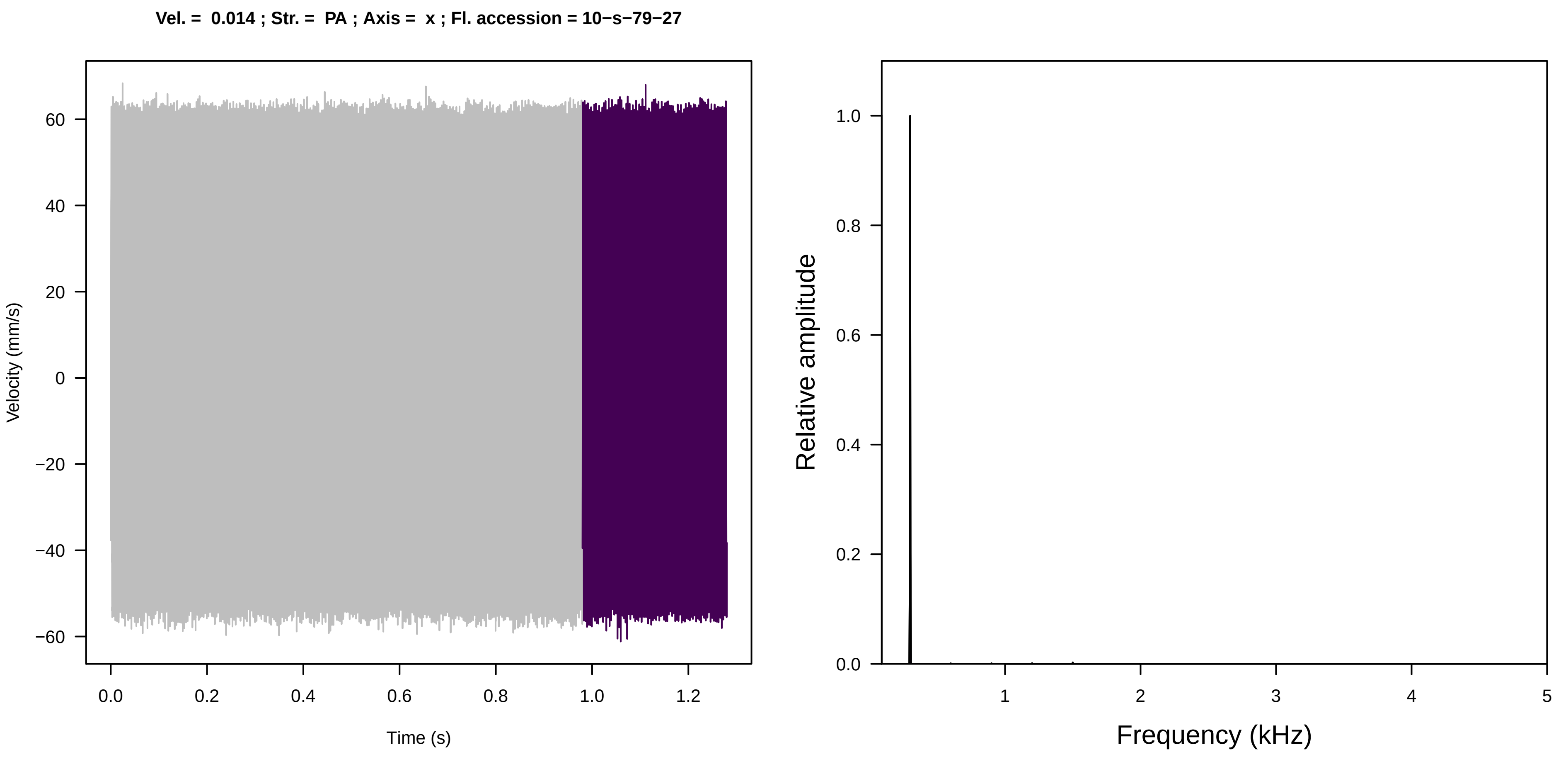
Vel. = 0.014 ; Str. = PA ; Axis = x ; Fl. accession = 10−s−79−27
Velocity (mm/s)
Time (s)
Relative amplitude
Frequency (kHz)

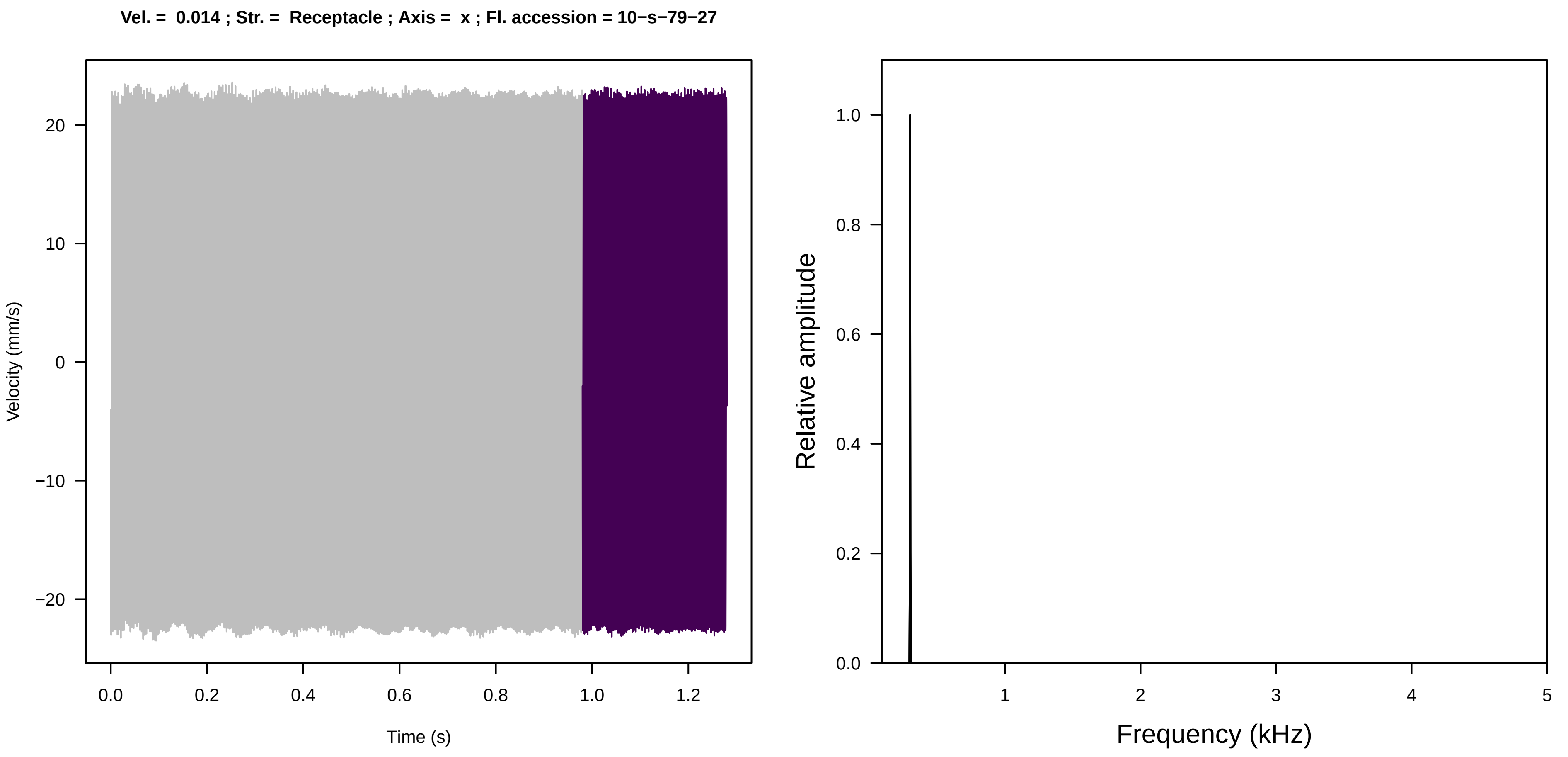
Vel. = 0.014 ; Str. = Receptacle ; Axis = x ; Fl. accession = 10−s−79−27
Velocity (mm/s)
Time (s)
Relative amplitude
Frequency (kHz)

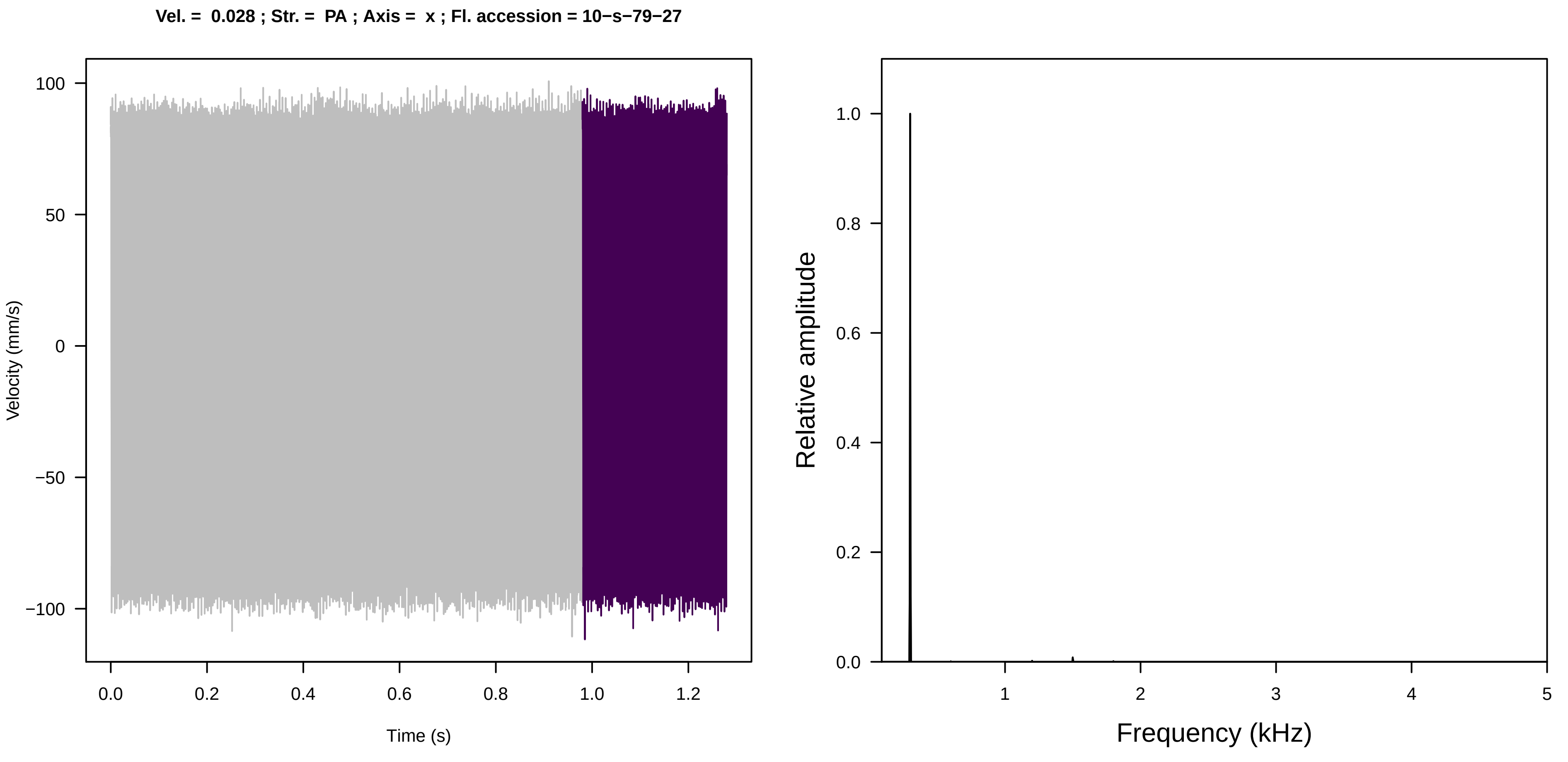
Vel. = 0.028 ; Str. = PA ; Axis = x ; Fl. accession = 10−s−79−27
Velocity (mm/s)
Time (s)
Relative amplitude
Frequency (kHz)

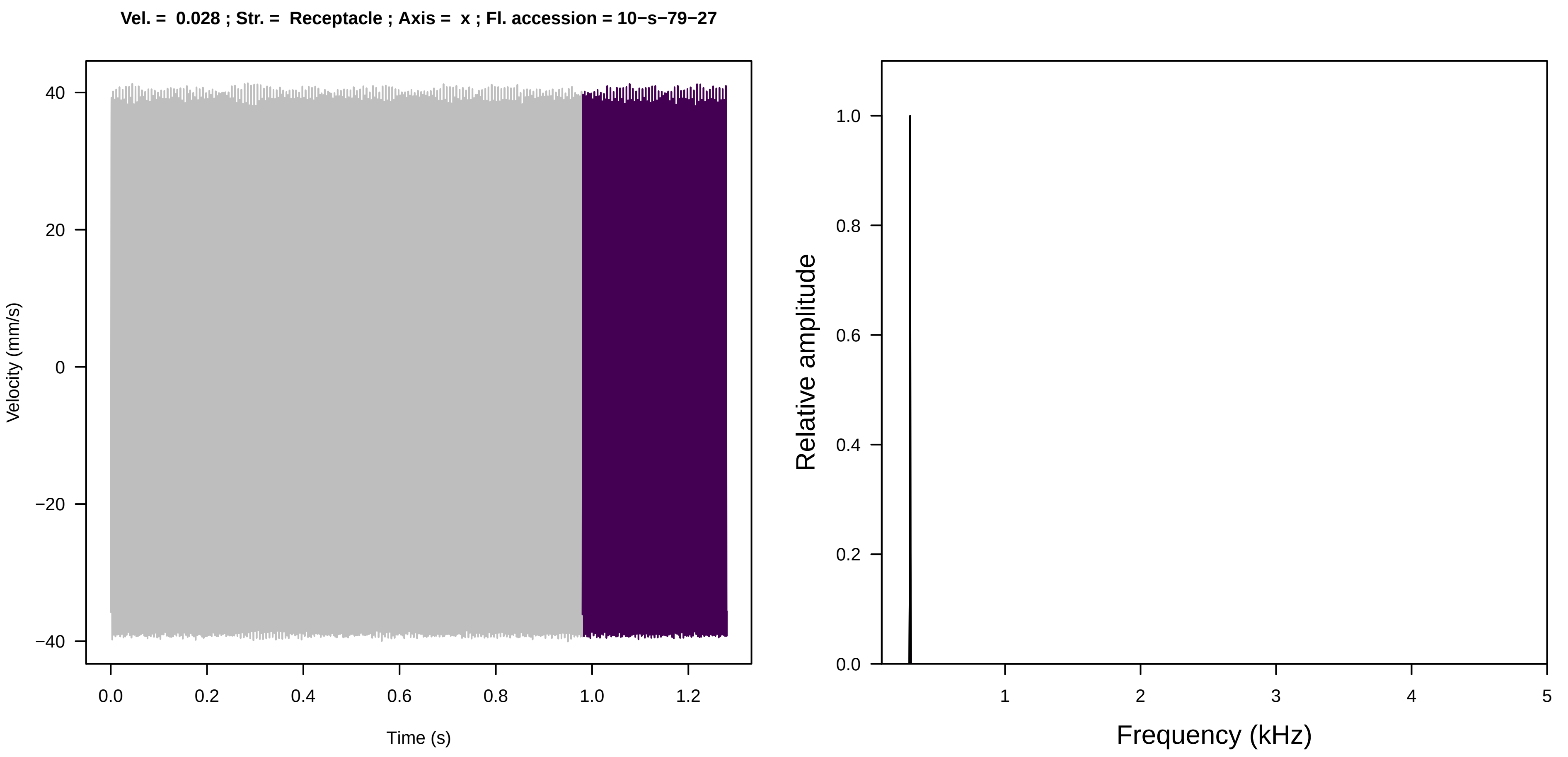
Vel. = 0.028 ; Str. = Receptacle ; Axis = x ; Fl. accession = 10−s−79−27
Velocity (mm/s)
Time (s)
Relative amplitude
Frequency (kHz)

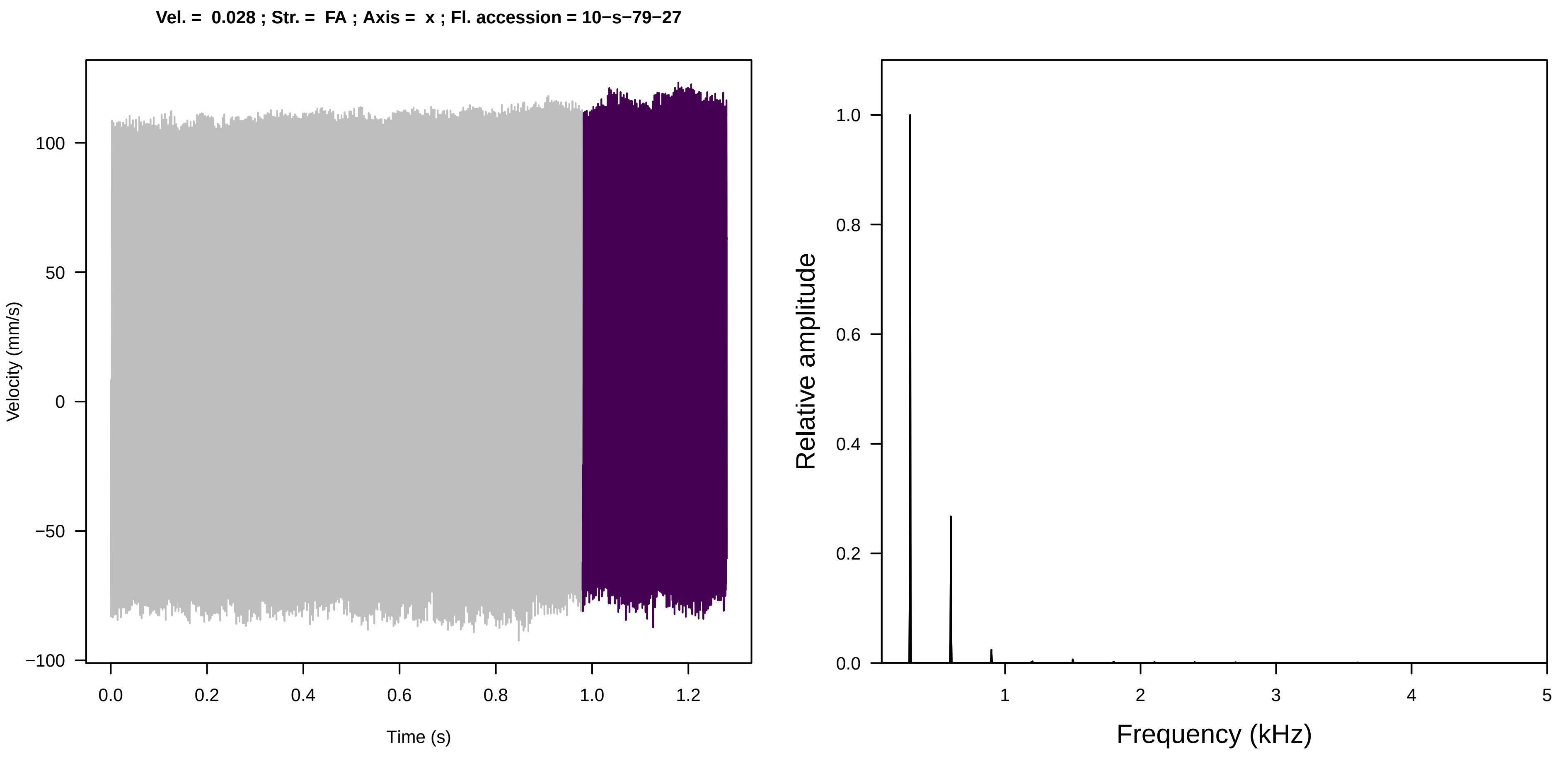
Vel. = 0.028 ; Str. = FA ; Axis = x ; Fl. accession = 10−s−79−27
Velocity (mm/s)
Time (s)
Relative amplitude
Frequency (kHz)

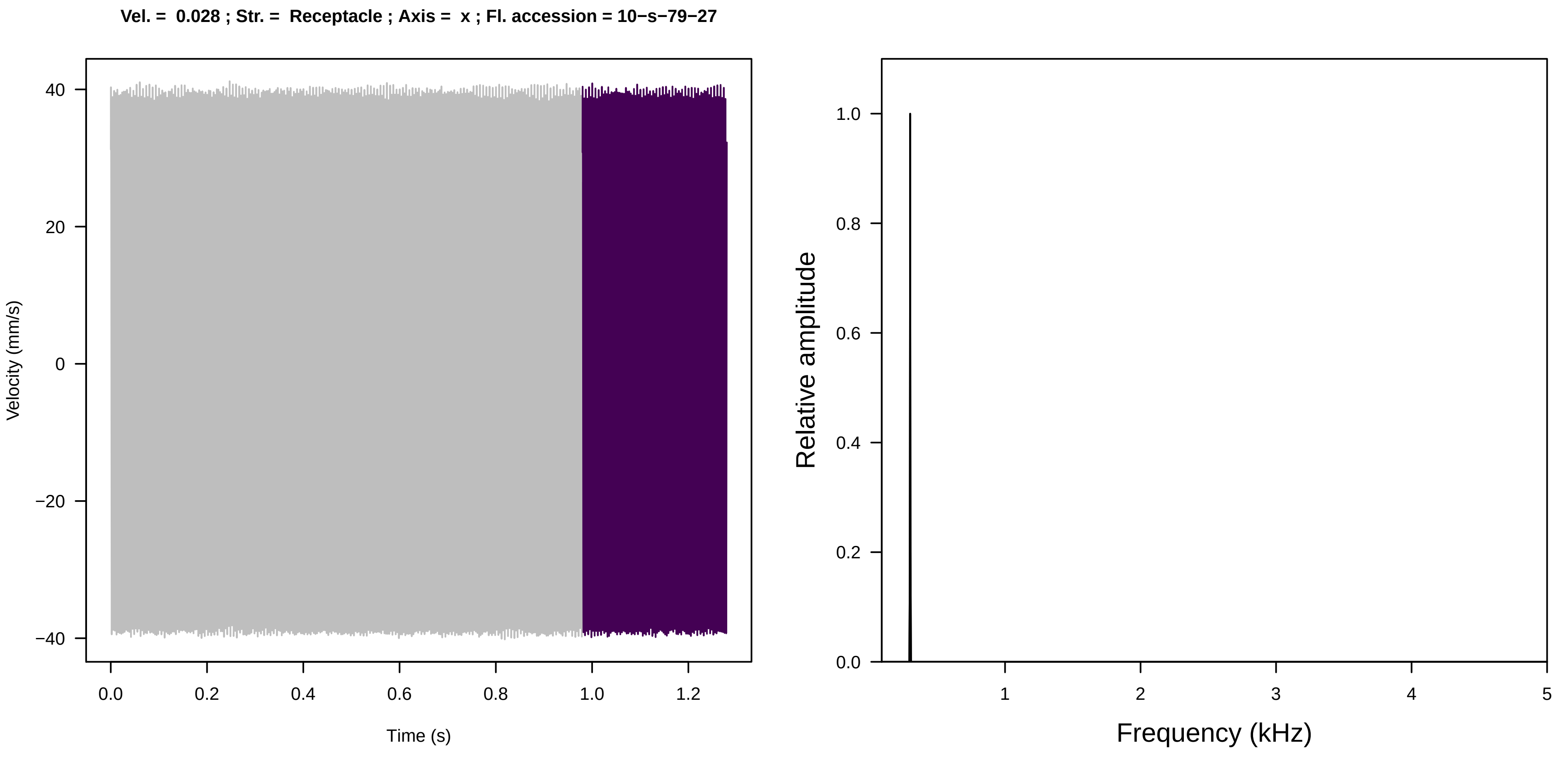
Vel. = 0.028 ; Str. = Receptacle ; Axis = x ; Fl. accession = 10−s−79−27
Velocity (mm/s)
Time (s)
Relative amplitude
Frequency (kHz)

Vel. = 0.028 ; Str. = Corolla ; Axis = x ; Fl. accession = 10−s−79−27

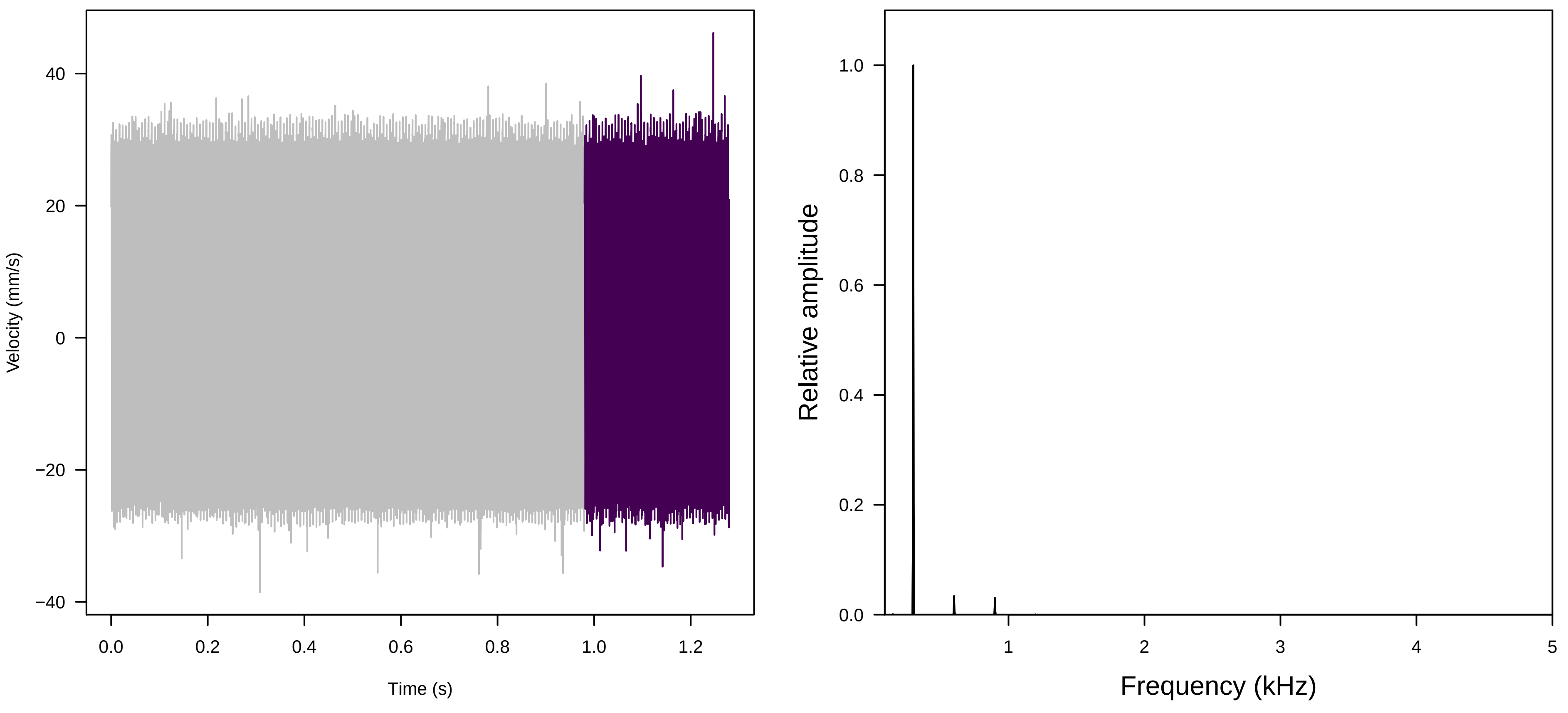

Vel. = 0.028 ; Str. = Receptacle ; Axis = x ; Fl. accession = 10−s−79−27

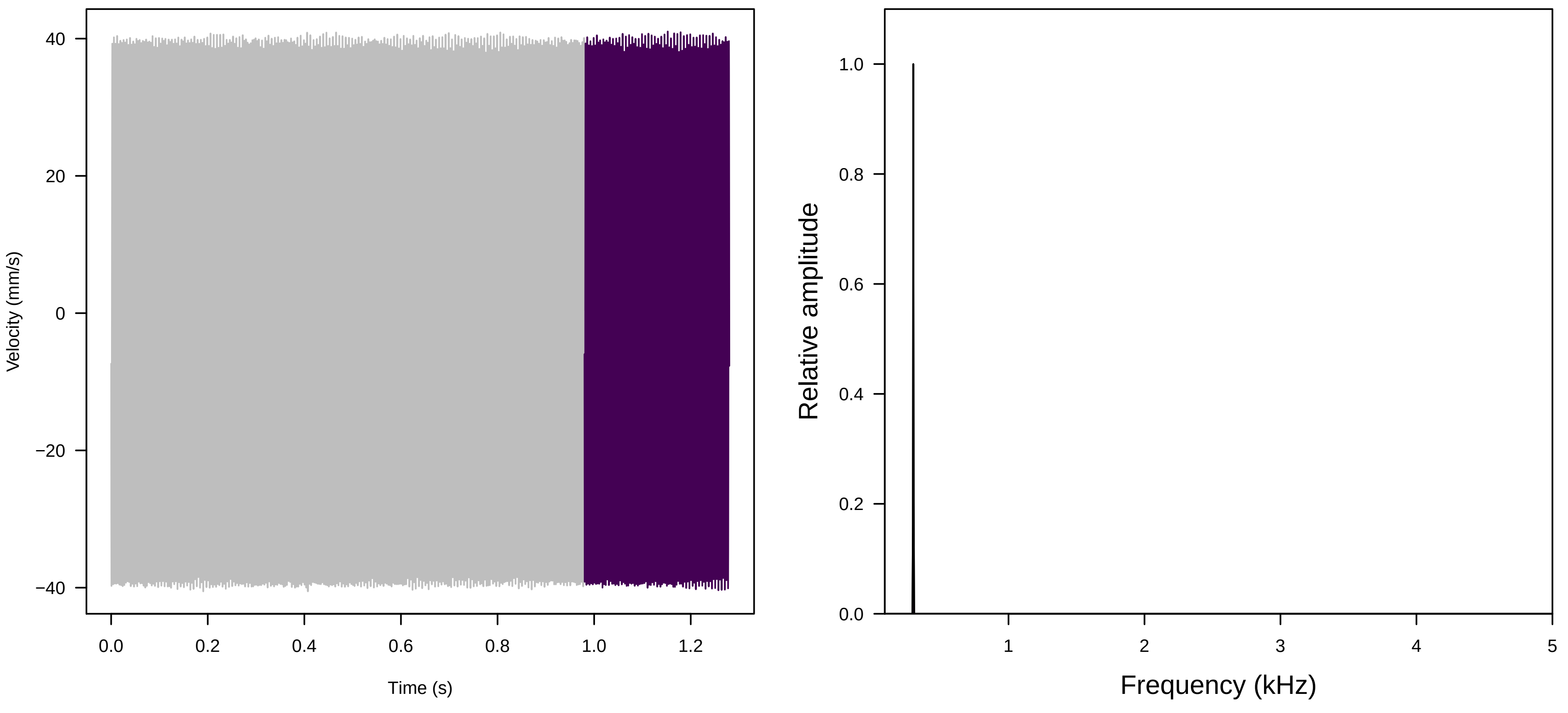

Vel. = 0.057 ; Str. = Corolla ; Axis = x ; Fl. accession = 10−s−79−27

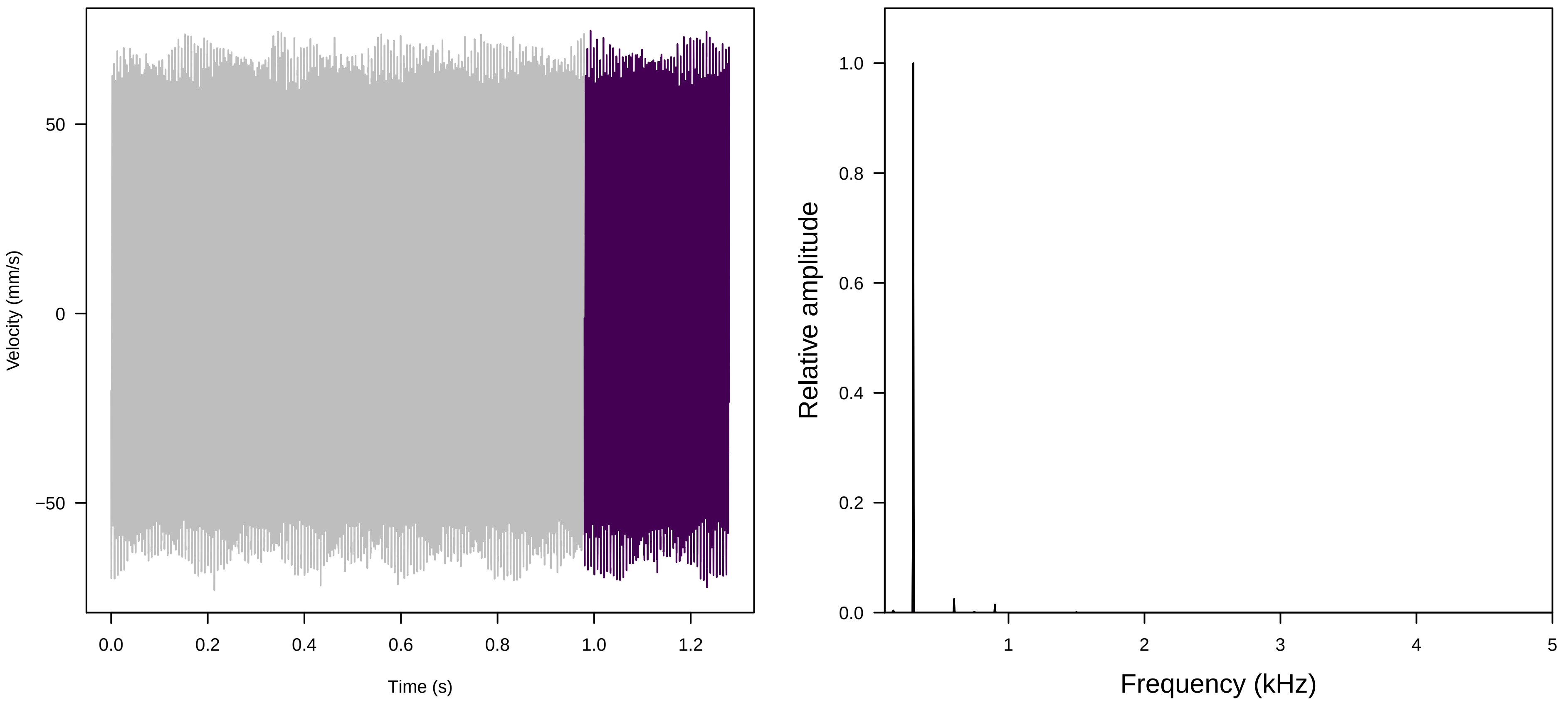

Vel. = 0.057 ; Str. = Receptacle ; Axis = x ; Fl. accession = 10−s−79−27

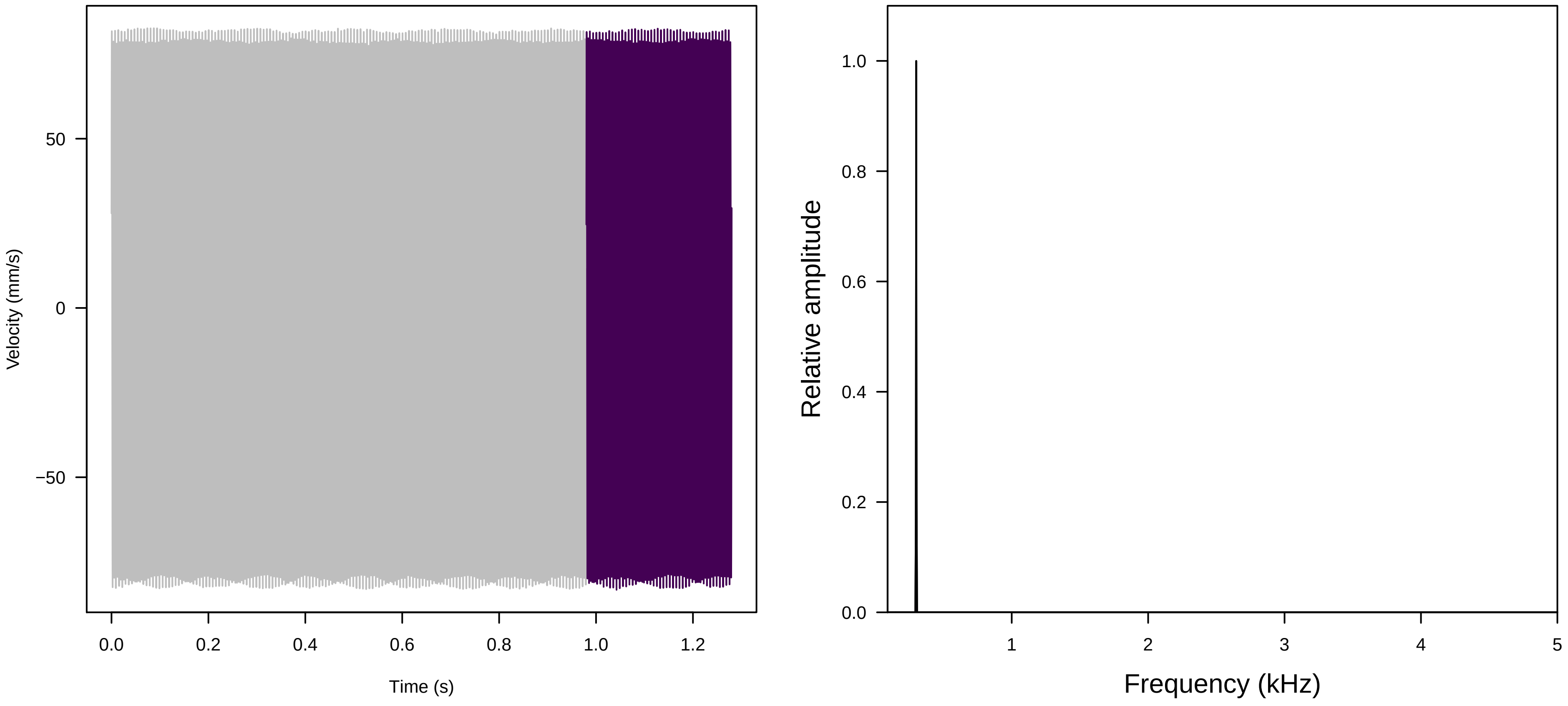

Vel. = 0.057 ; Str. = FA ; Axis = x ; Fl. accession = 10−s−79−27

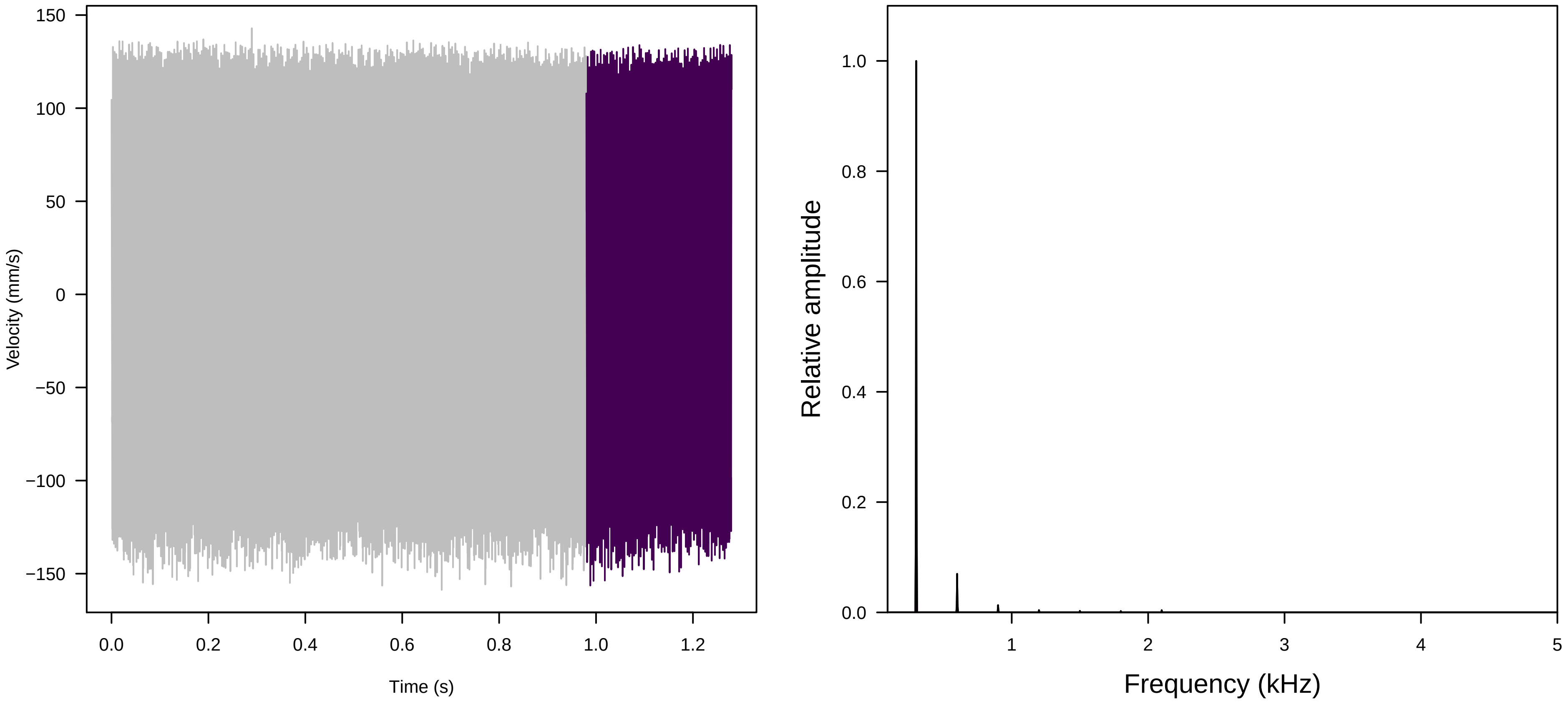

Vel. = 0.057 ; Str. = Receptacle ; Axis = x ; Fl. accession = 10−s−79−27

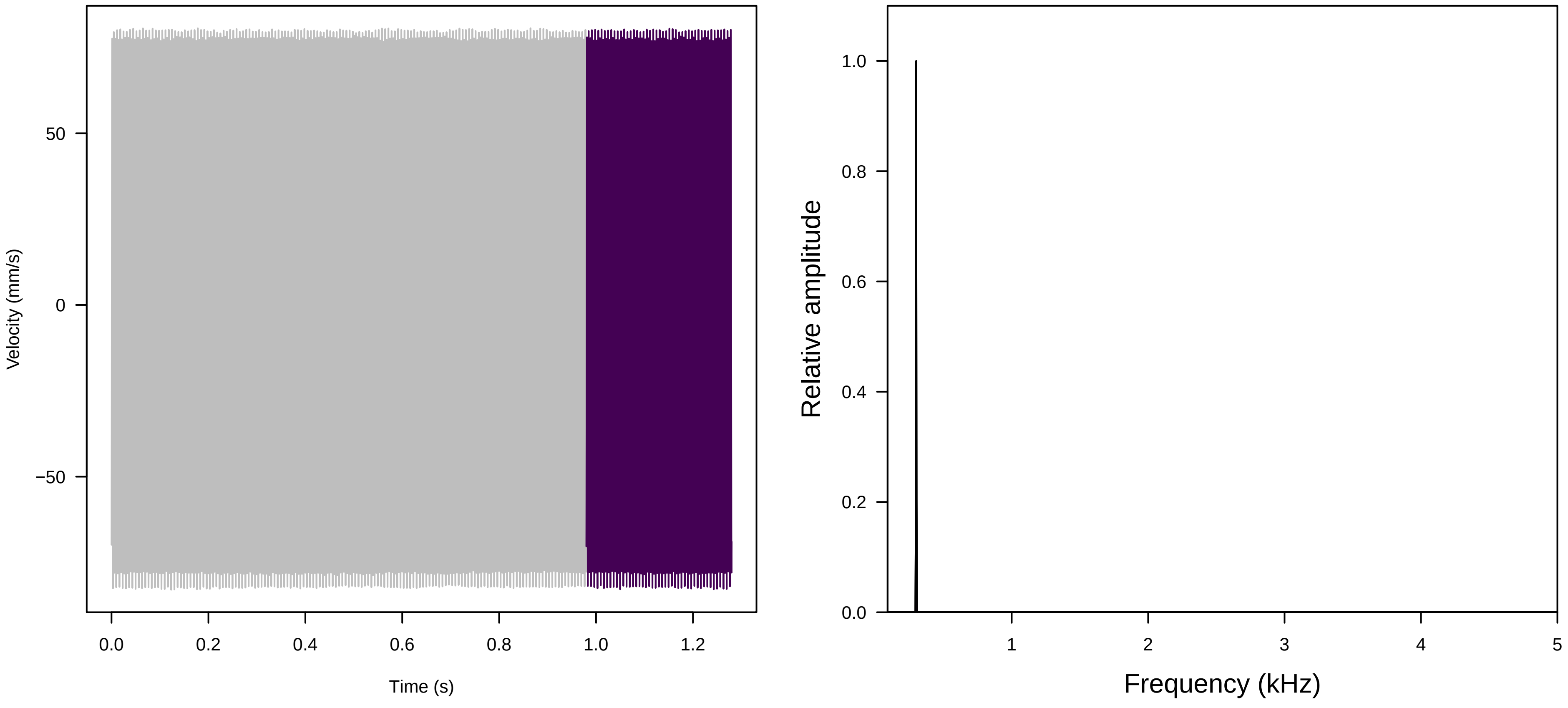

Vel. = 0.057 ; Str. = PA ; Axis = x ; Fl. accession = 10−s−79−27

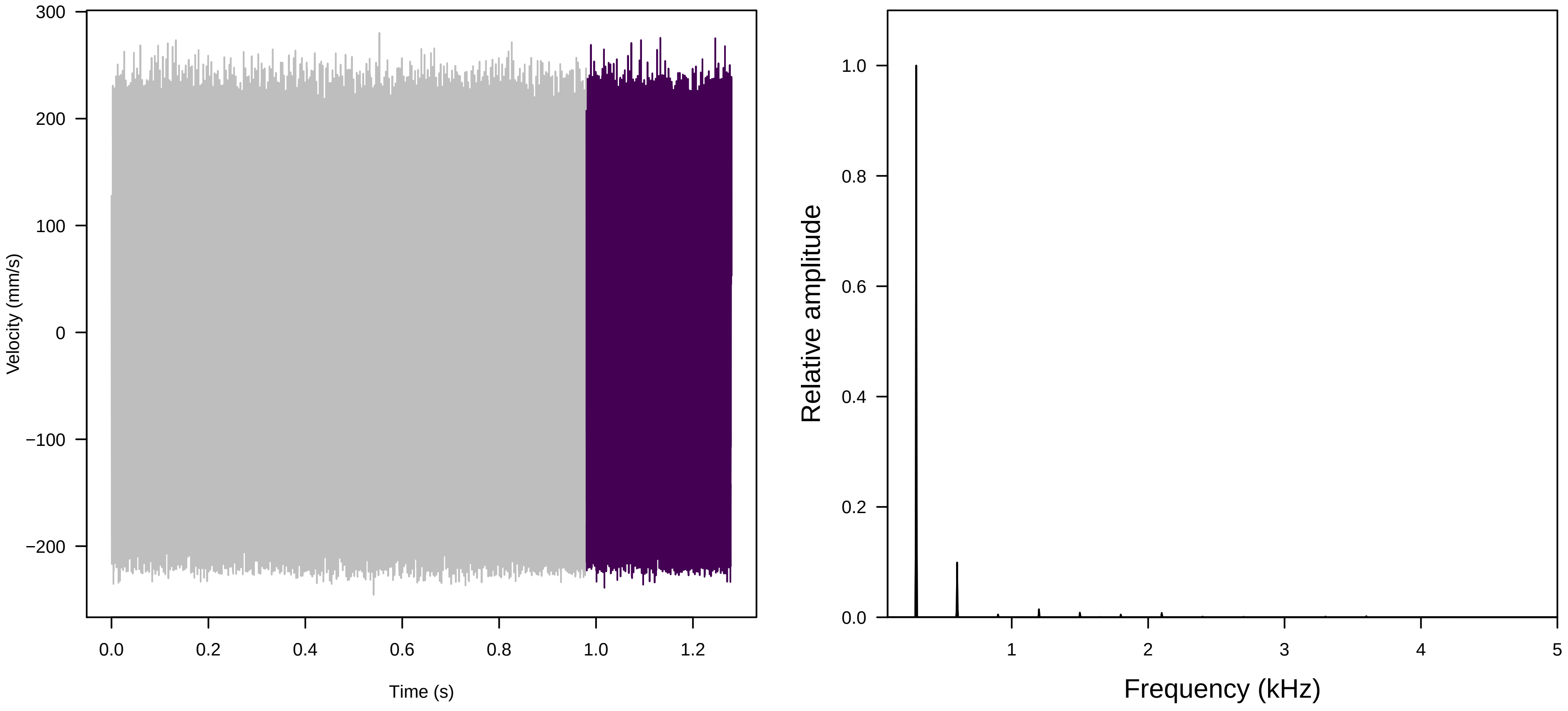

Vel. = 0.057 ; Str. = Receptacle ; Axis = x ; Fl. accession = 10−s−79−27

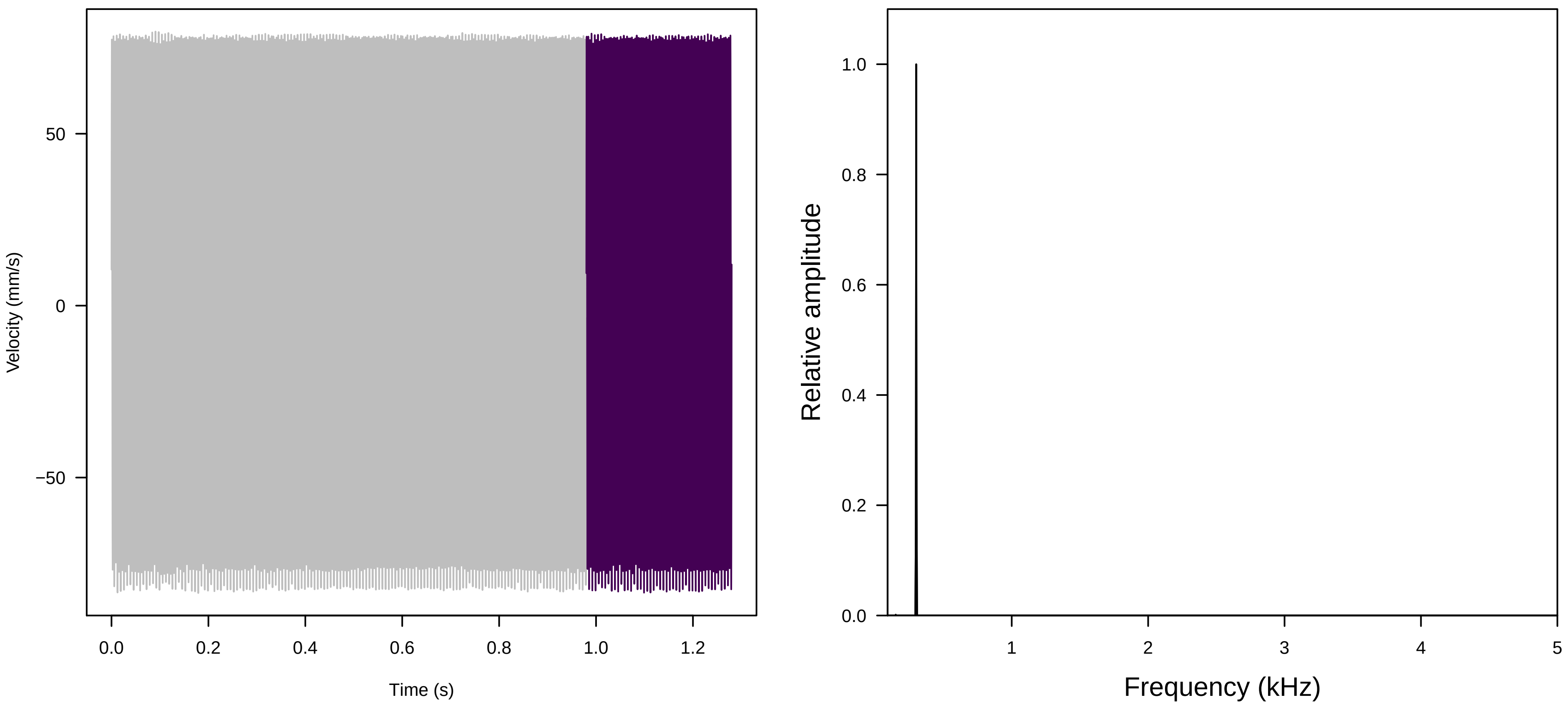

Vel. = 0.057 ; Str. = Corolla ; Axis = z ; Fl. accession = 10−s−79−27

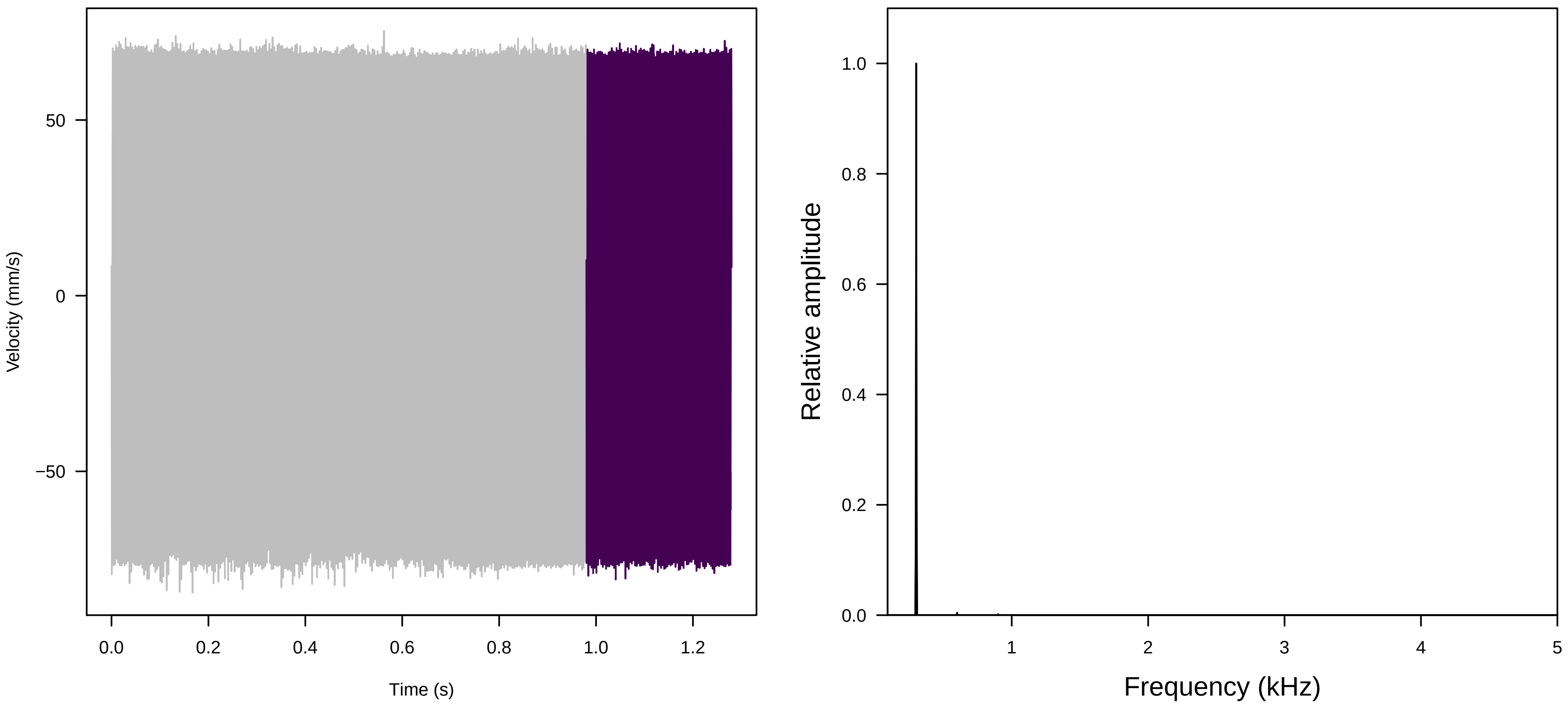

Vel. = 0.057 ; Str. = Receptacle ; Axis = z ; Fl. accession = 10−s−79−27

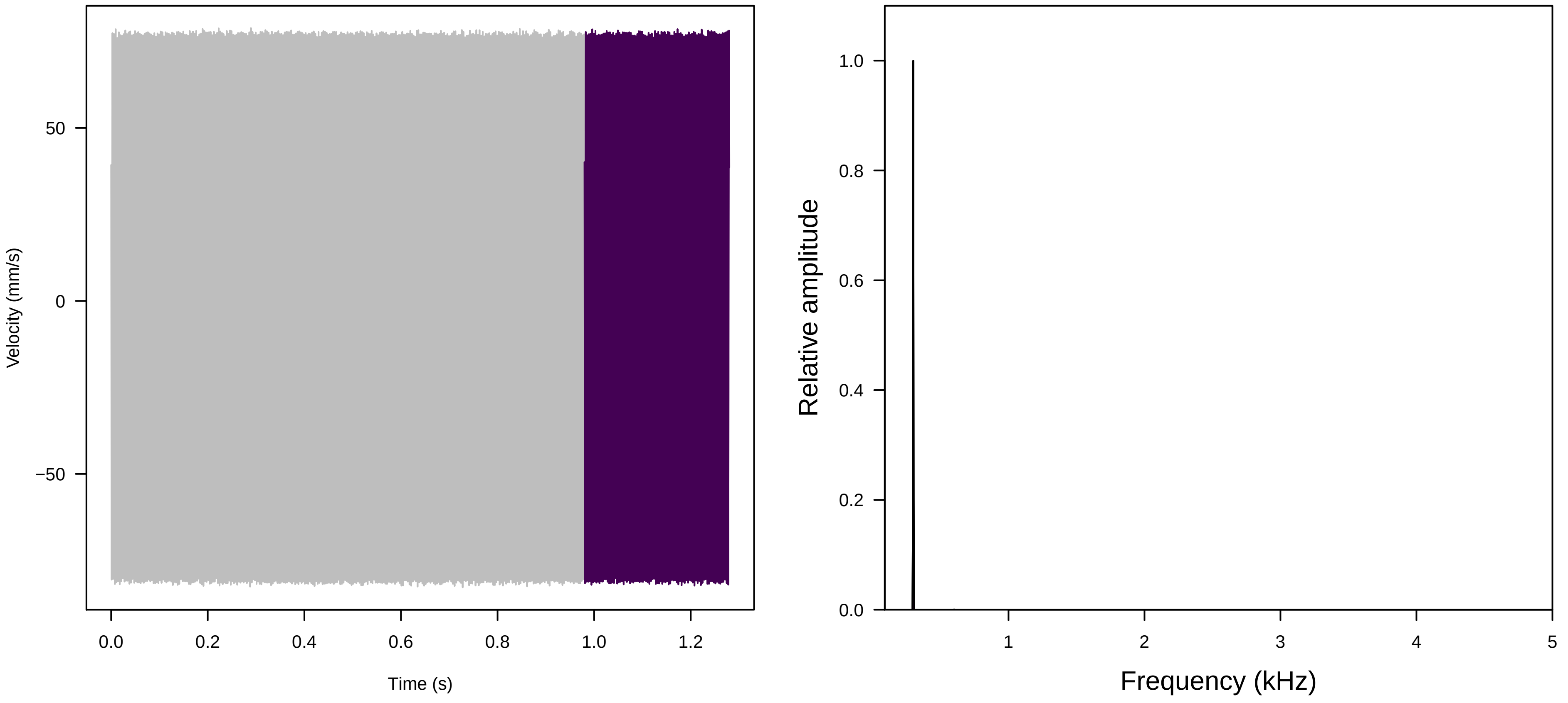

Vel. = 0.057 ; Str. = FA ; Axis = z ; Fl. accession = 10−s−79−27

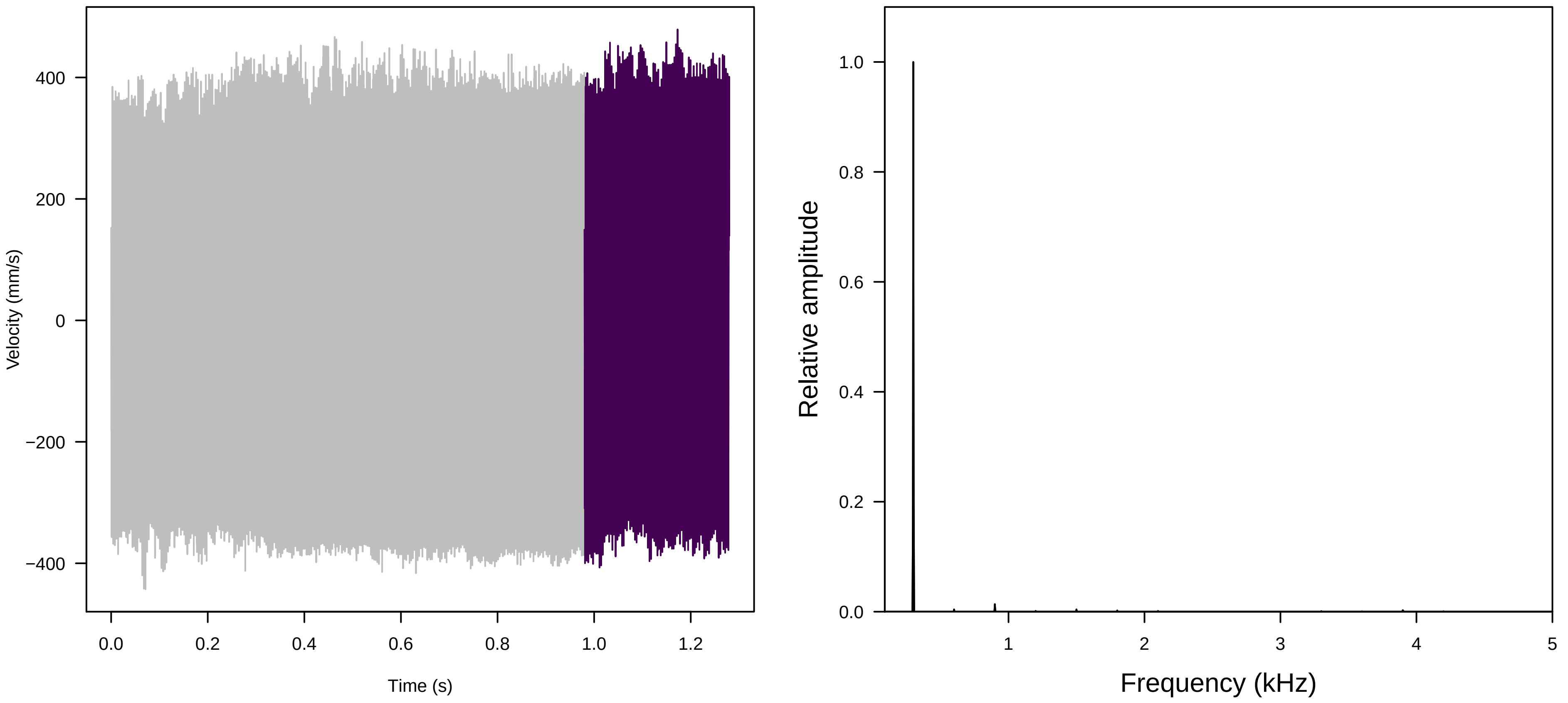

Vel. = 0.057 ; Str. = Receptacle ; Axis = z ; Fl. accession = 10−s−79−27

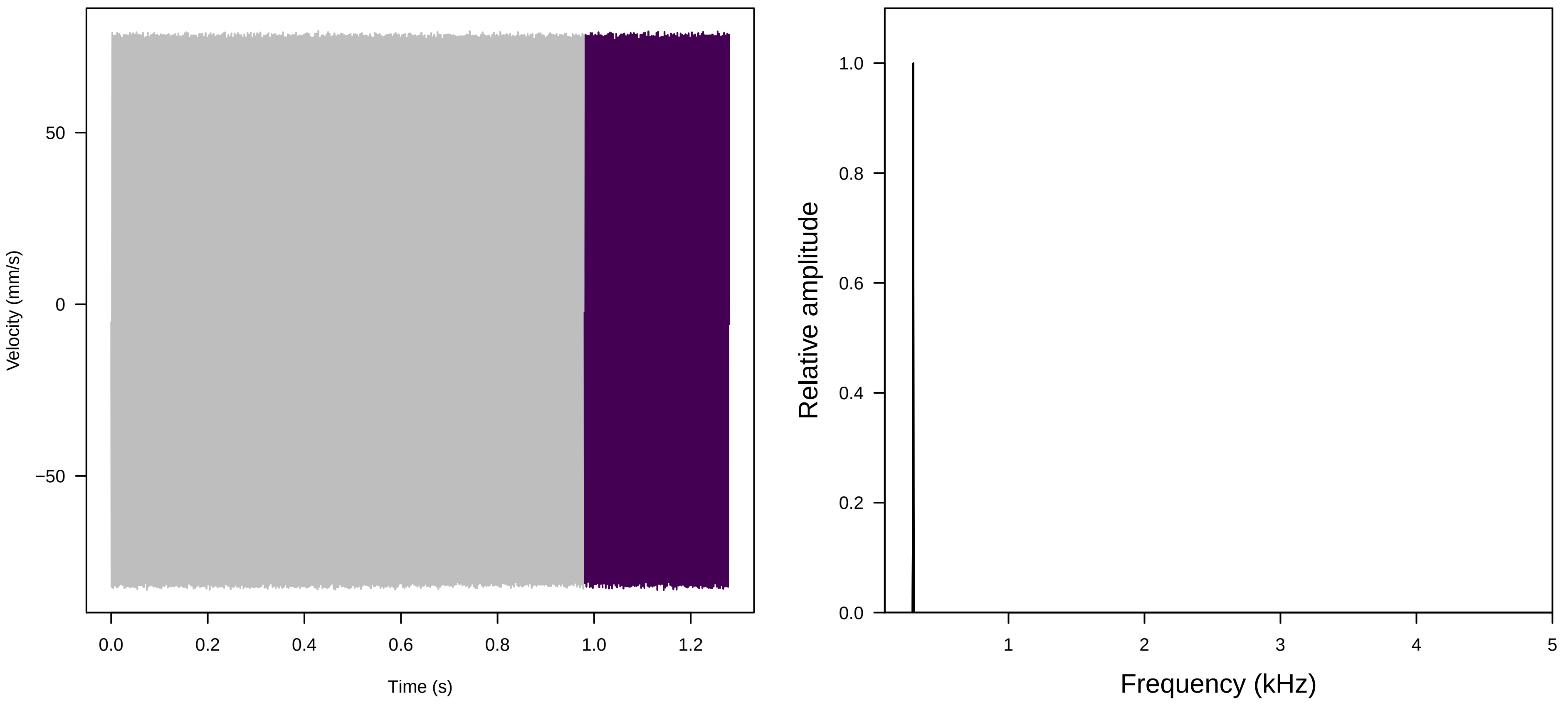

Vel. = 0.057 ; Str. = PA ; Axis = z ; Fl. accession = 10−s−79−27

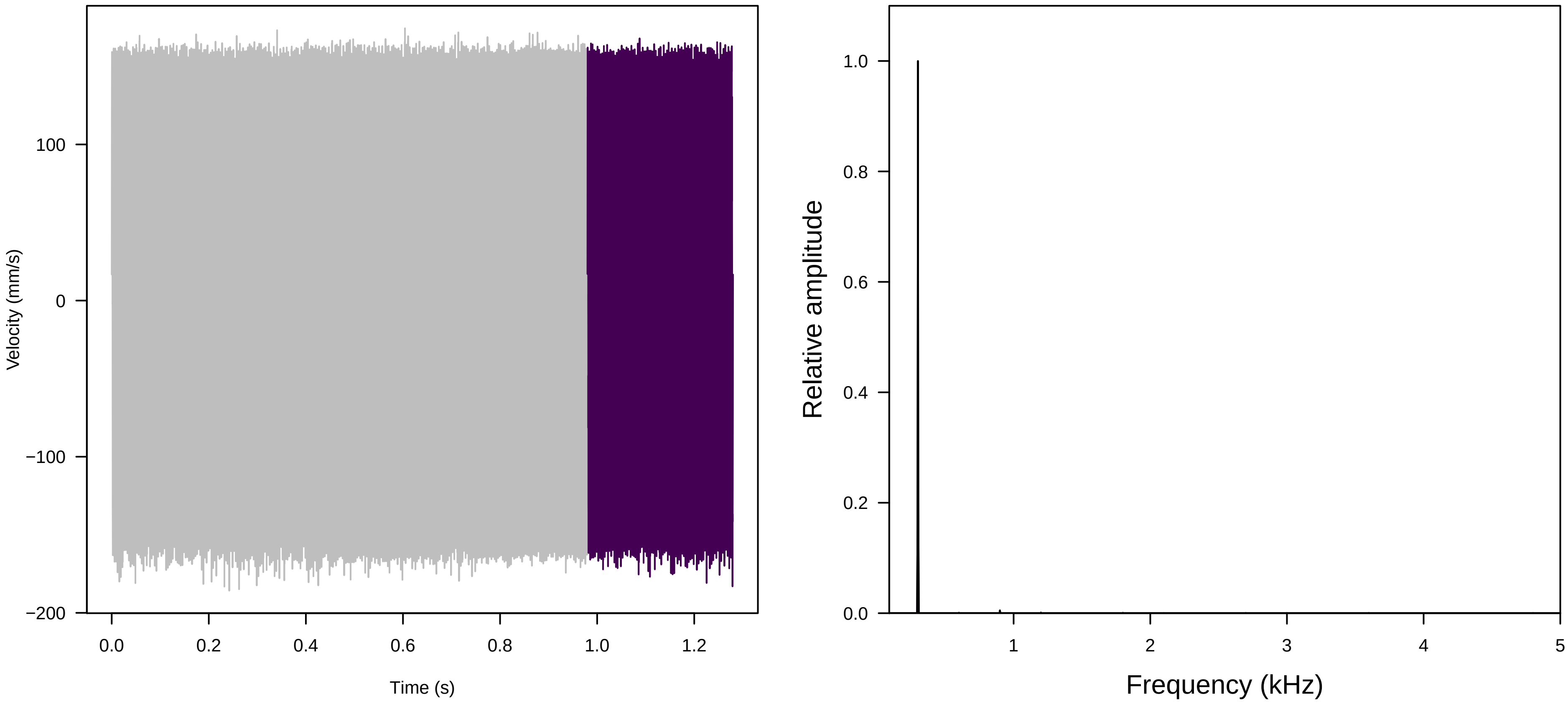

Vel. = 0.057 ; Str. = Receptacle ; Axis = z ; Fl. accession = 10−s−79−27

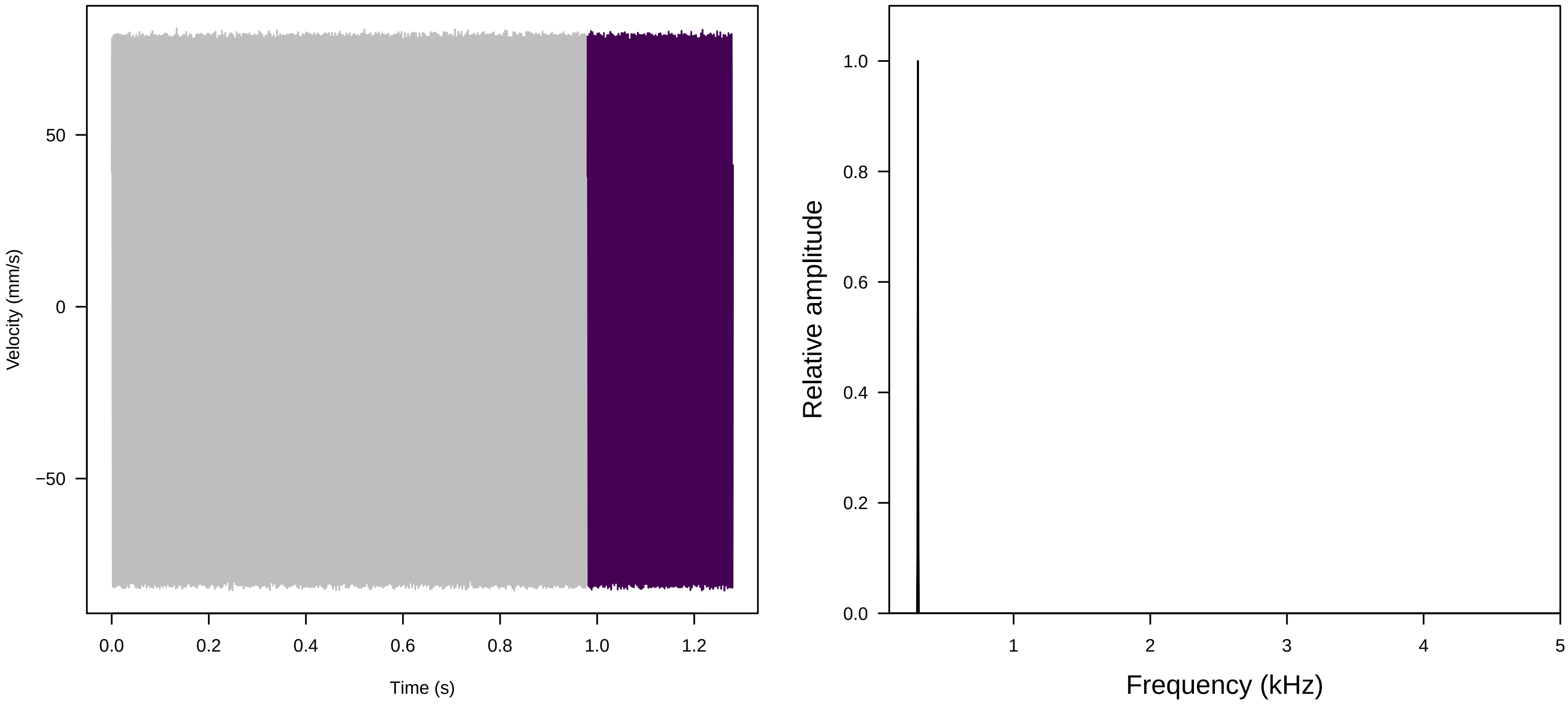

Vel. = 0.028 ; Str. = PA ; Axis = z ; Fl. accession = 10−s−79−27

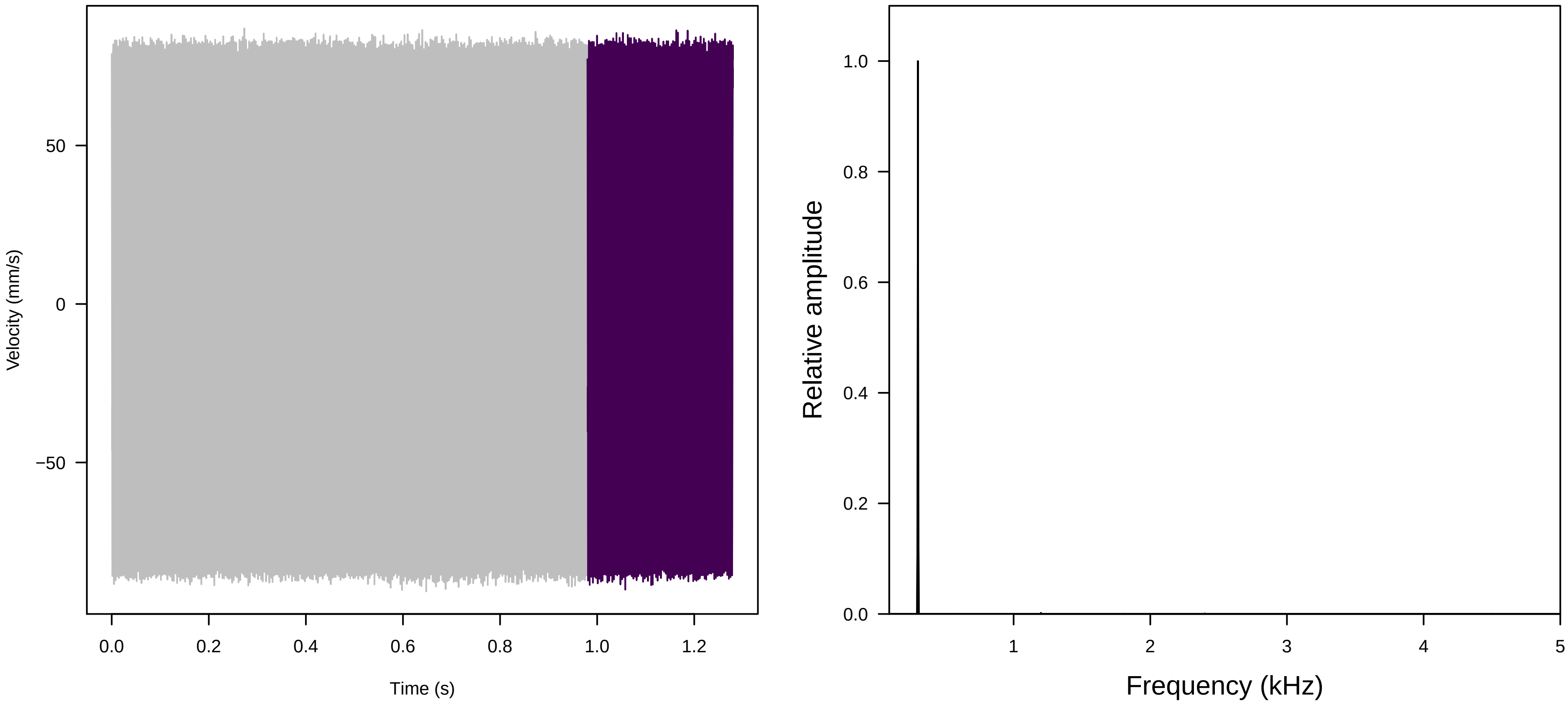

Vel. = 0.028 ; Str. = Receptacle ; Axis = z ; Fl. accession = 10−s−79−27

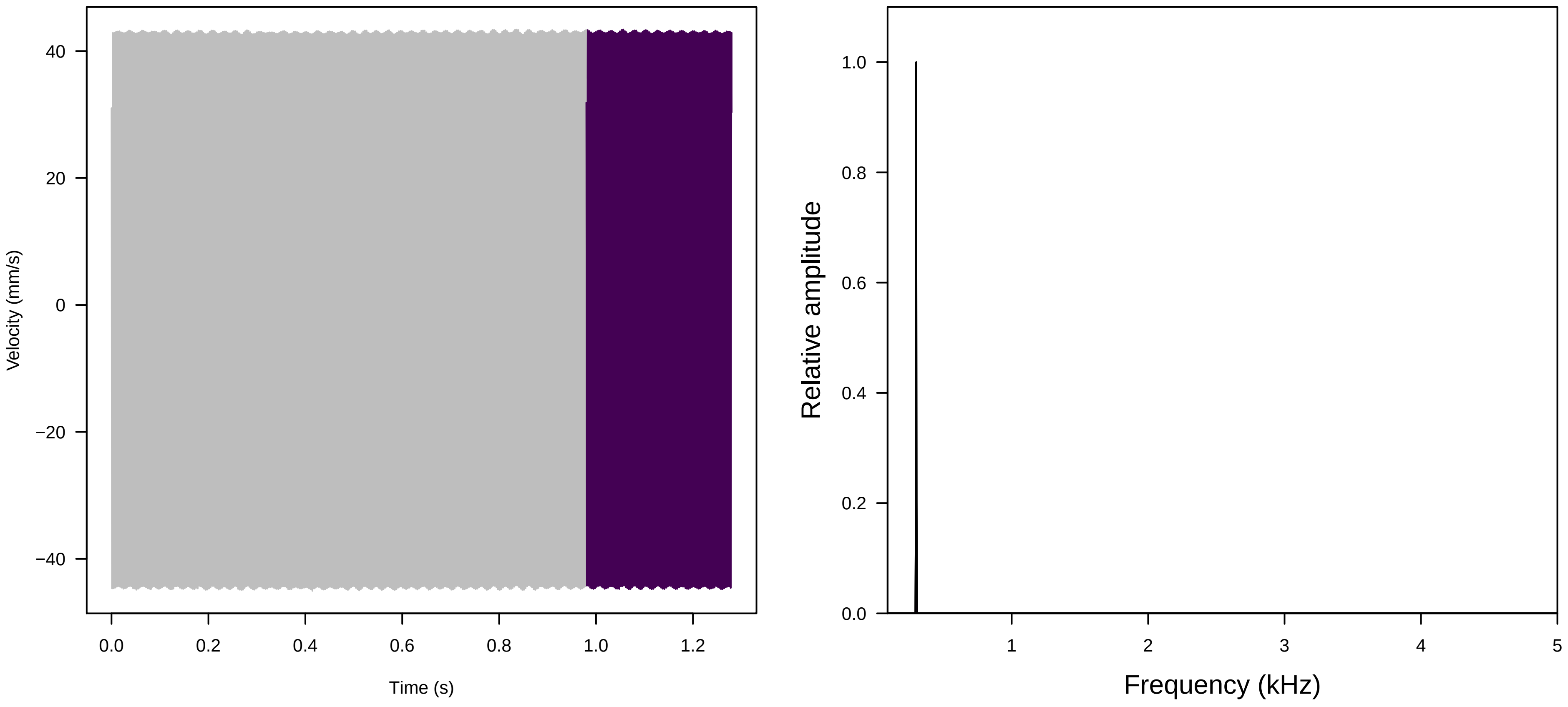

Vel. = 0.028 ; Str. = FA ; Axis = z ; Fl. accession = 10−s−79−27

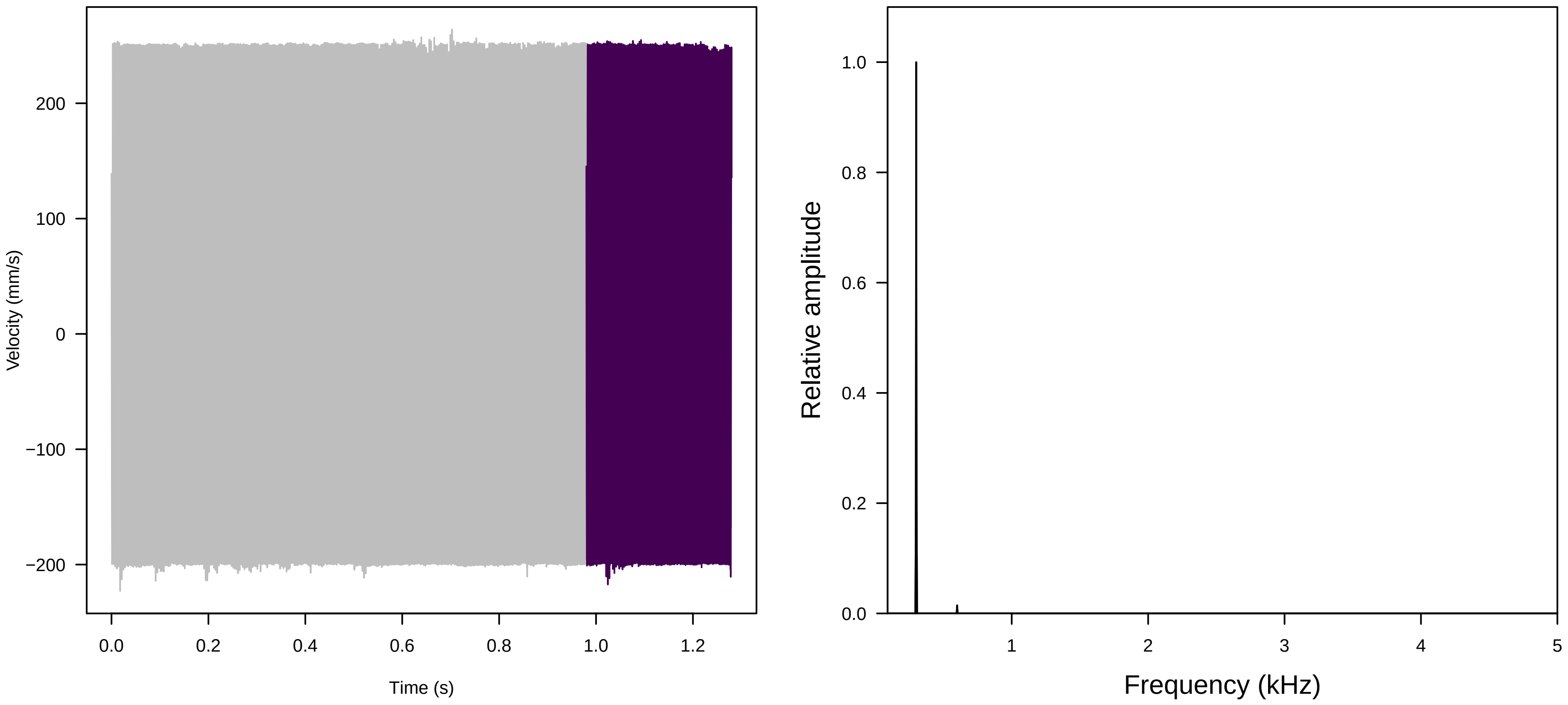

Vel. = 0.028 ; Str. = Receptacle ; Axis = z ; Fl. accession = 10−s−79−27

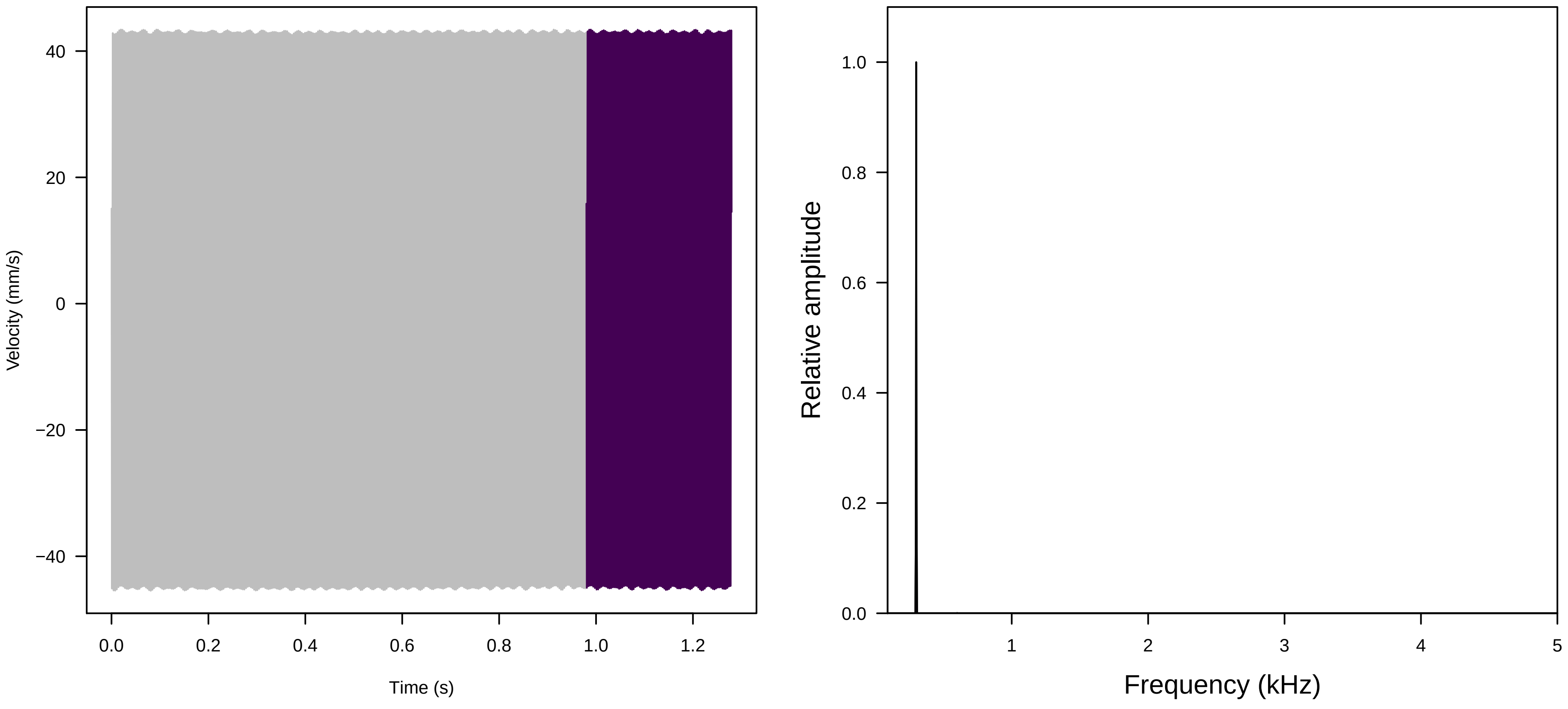

Vel. = 0.028 ; Str. = Corolla ; Axis = z ; Fl. accession = 10−s−79−27

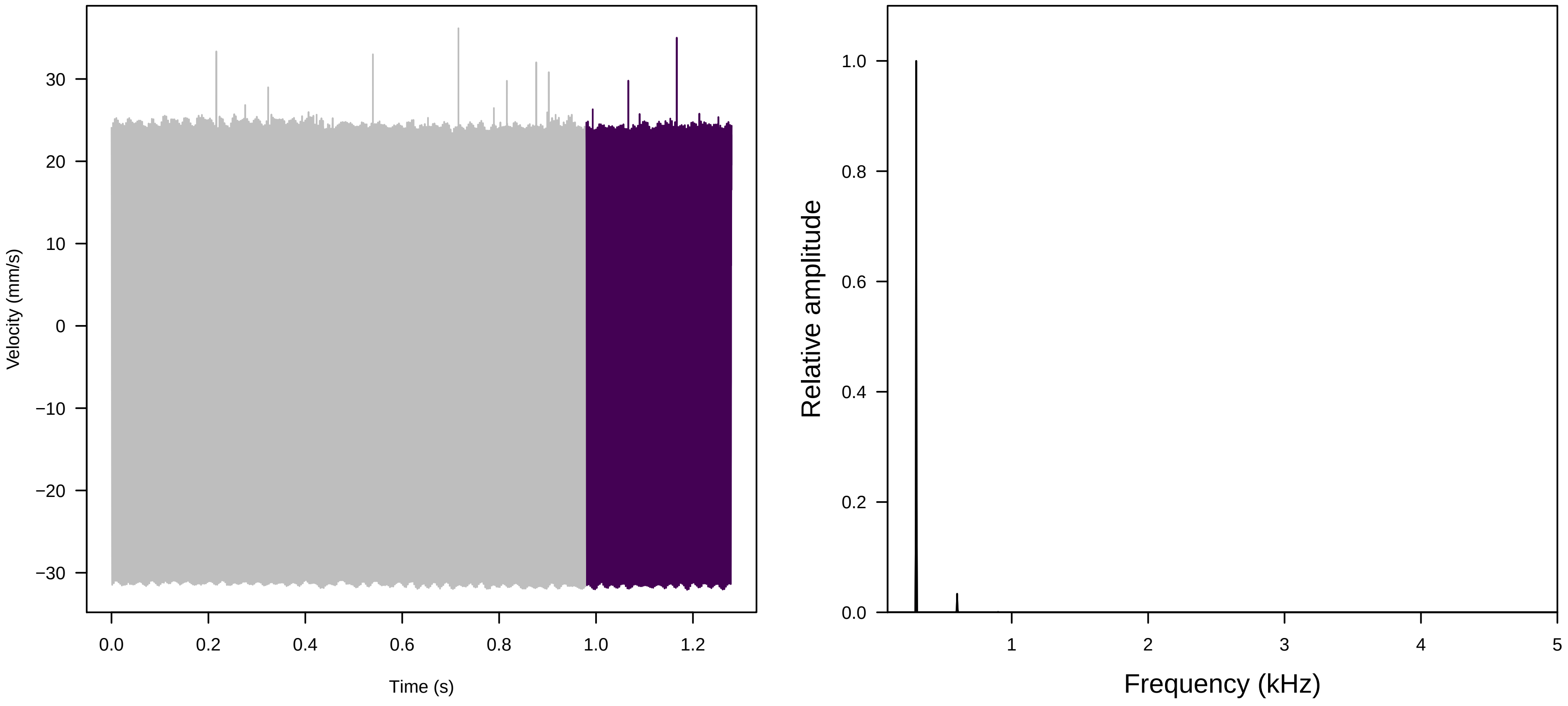

Vel. = 0.028 ; Str. = Receptacle ; Axis = z ; Fl. accession = 10−s−79−27

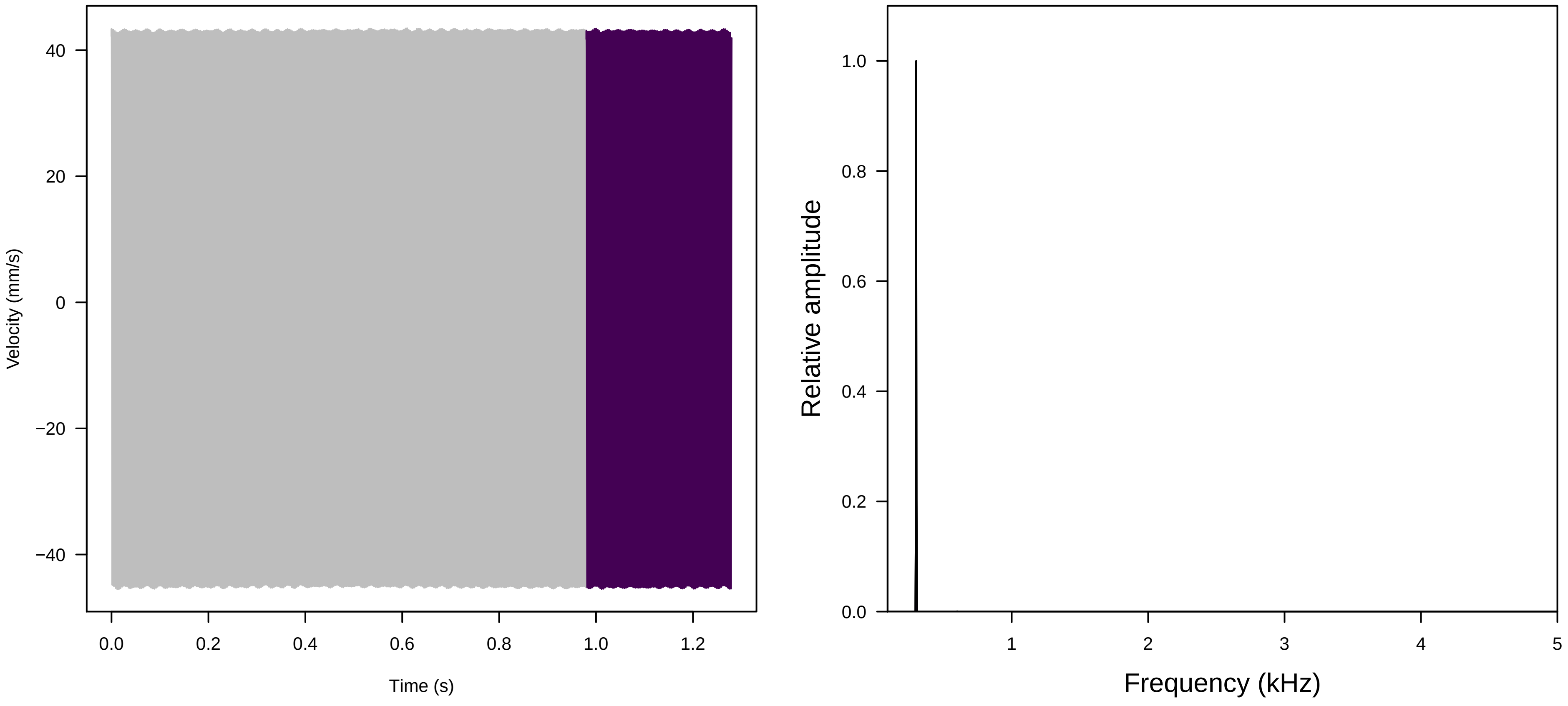

Vel. = 0.014 ; Str. = Corolla ; Axis = z ; Fl. accession = 10−s−79−27

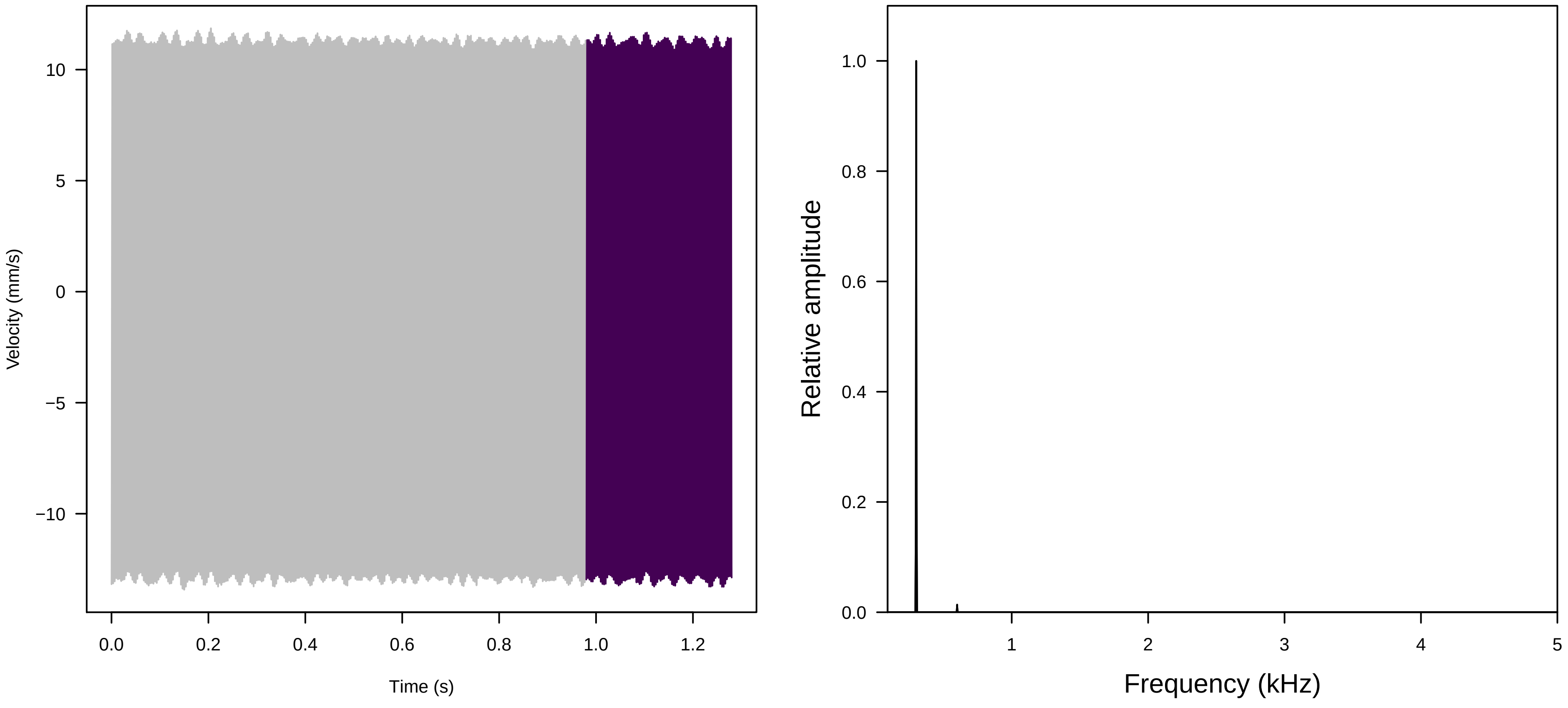

Vel. = 0.014 ; Str. = Receptacle ; Axis = z ; Fl. accession = 10−s−79−27

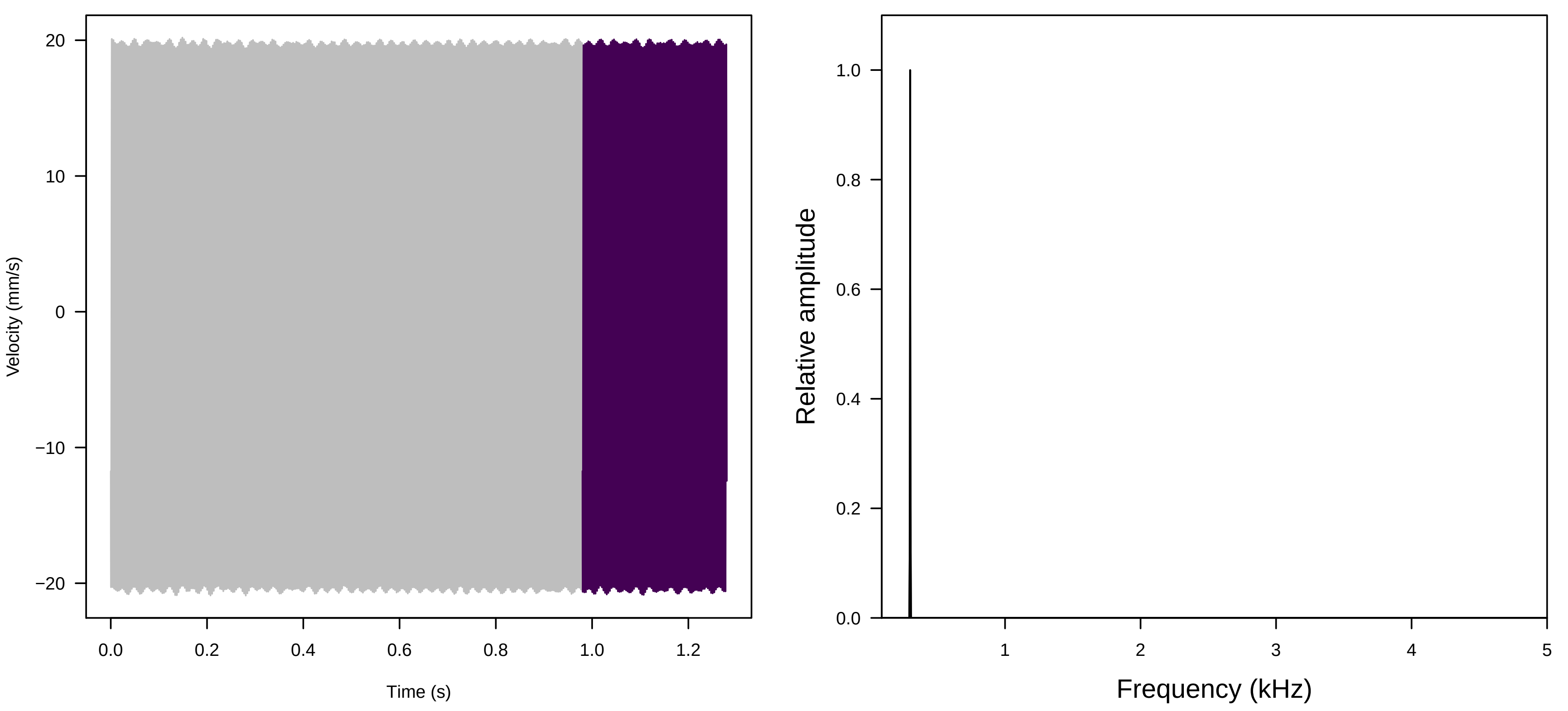

Vel. = 0.014 ; Str. = FA ; Axis = z ; Fl. accession = 10−s−79−27

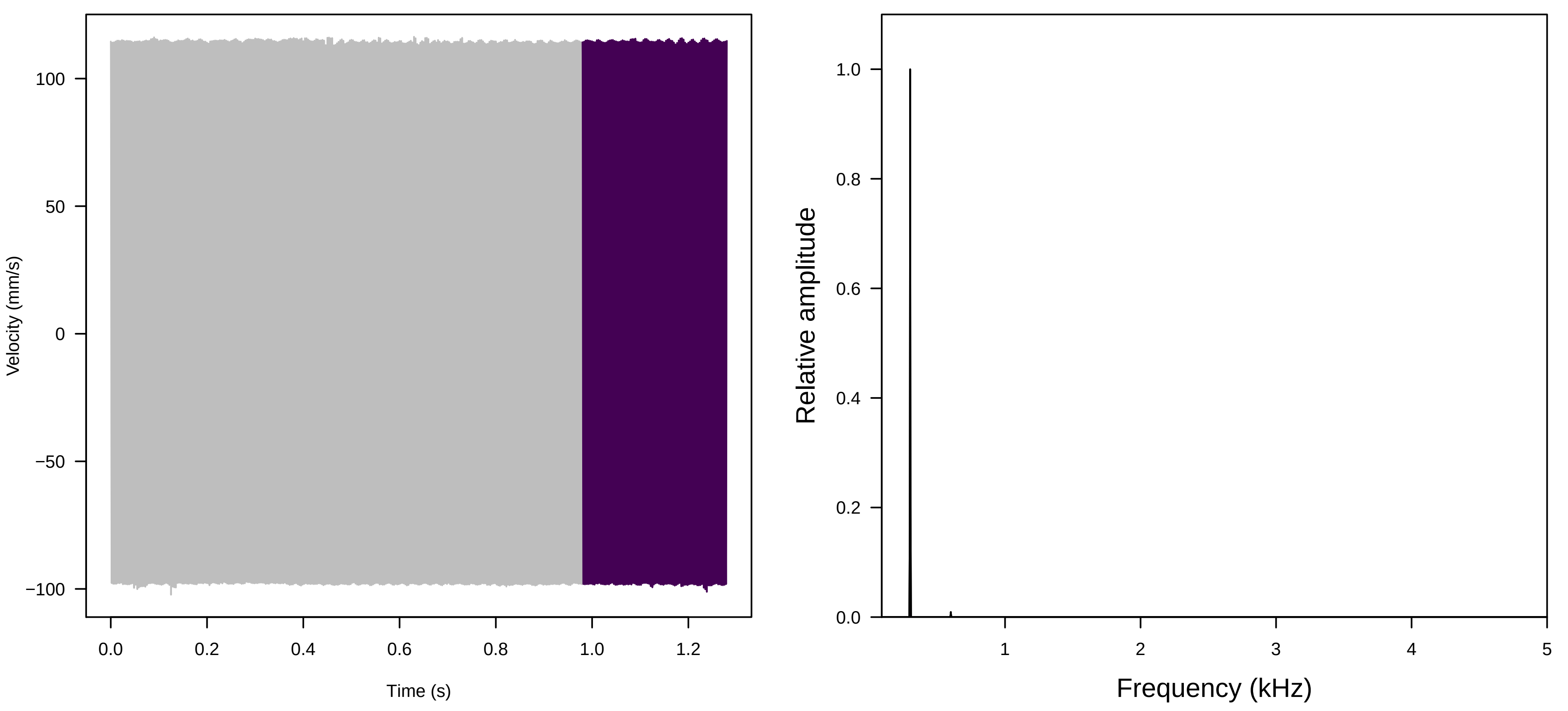

Vel. = 0.014 ; Str. = Receptacle ; Axis = z ; Fl. accession = 10−s−79−27

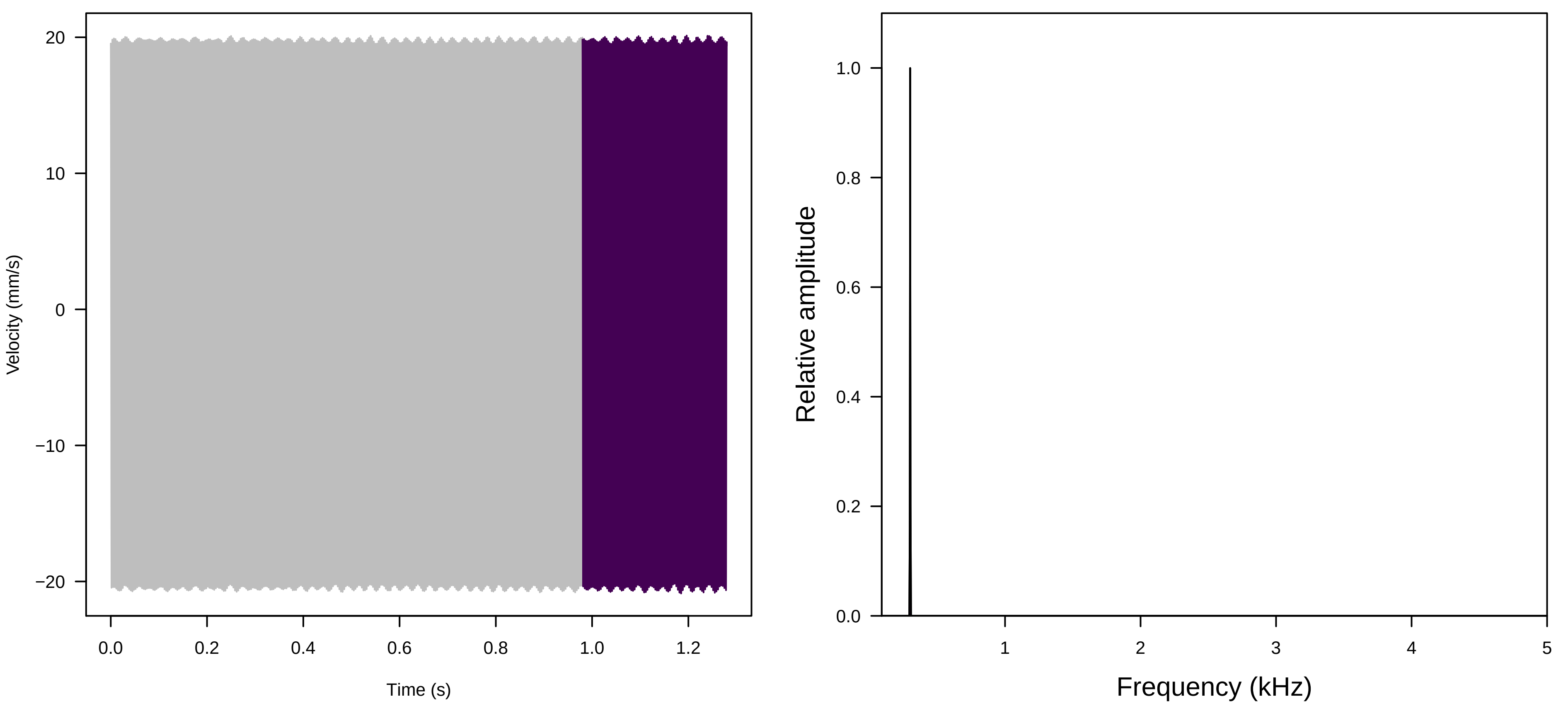

Vel. = 0.014 ; Str. = PA ; Axis = z ; Fl. accession = 10−s−79−27

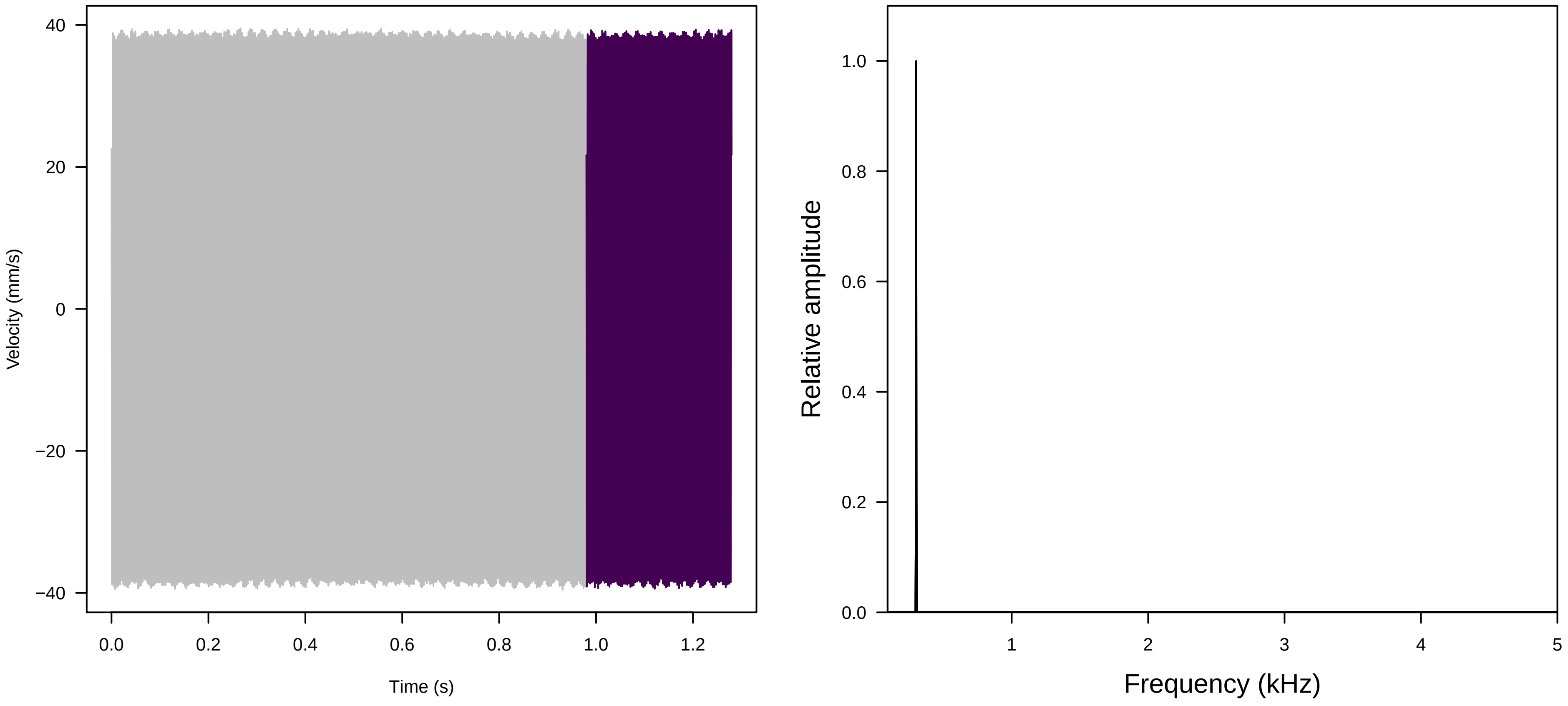

Vel. = 0.014 ; Str. = Receptacle ; Axis = z ; Fl. accession = 10−s−79−27

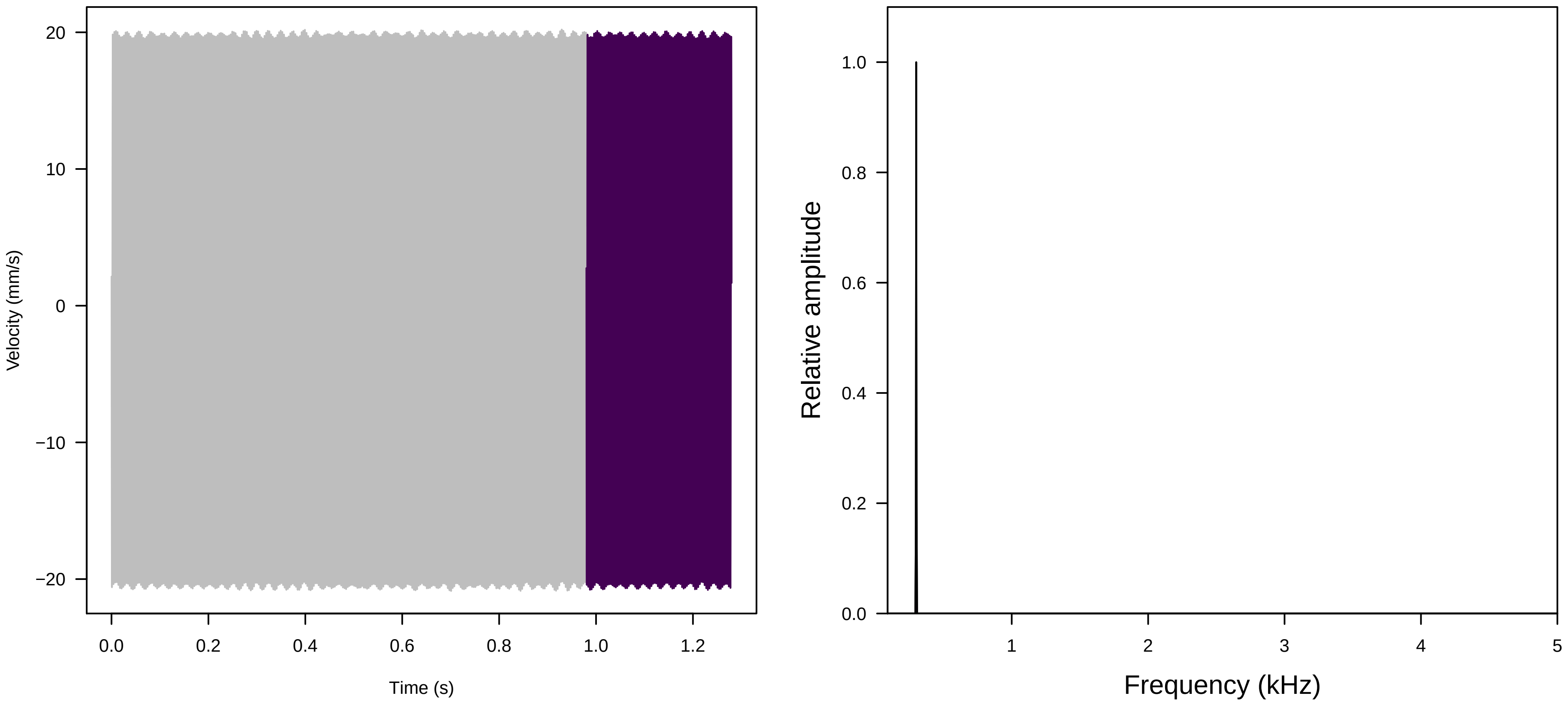

Vel. = 0.014 ; Str. = Corolla ; Axis = y ; Fl. accession = 10−s−79−27

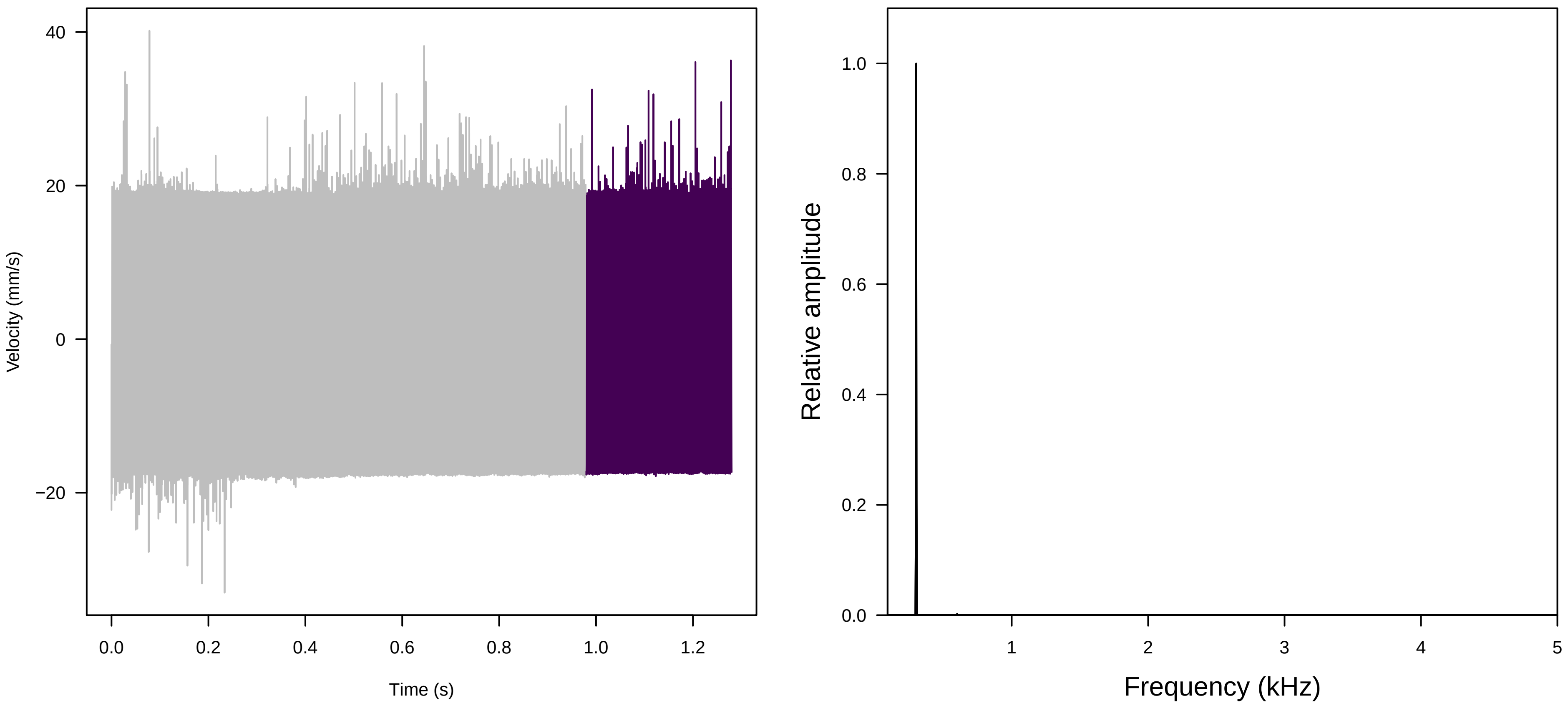

Vel. = 0.014 ; Str. = Receptacle ; Axis = y ; Fl. accession = 10−s−79−27

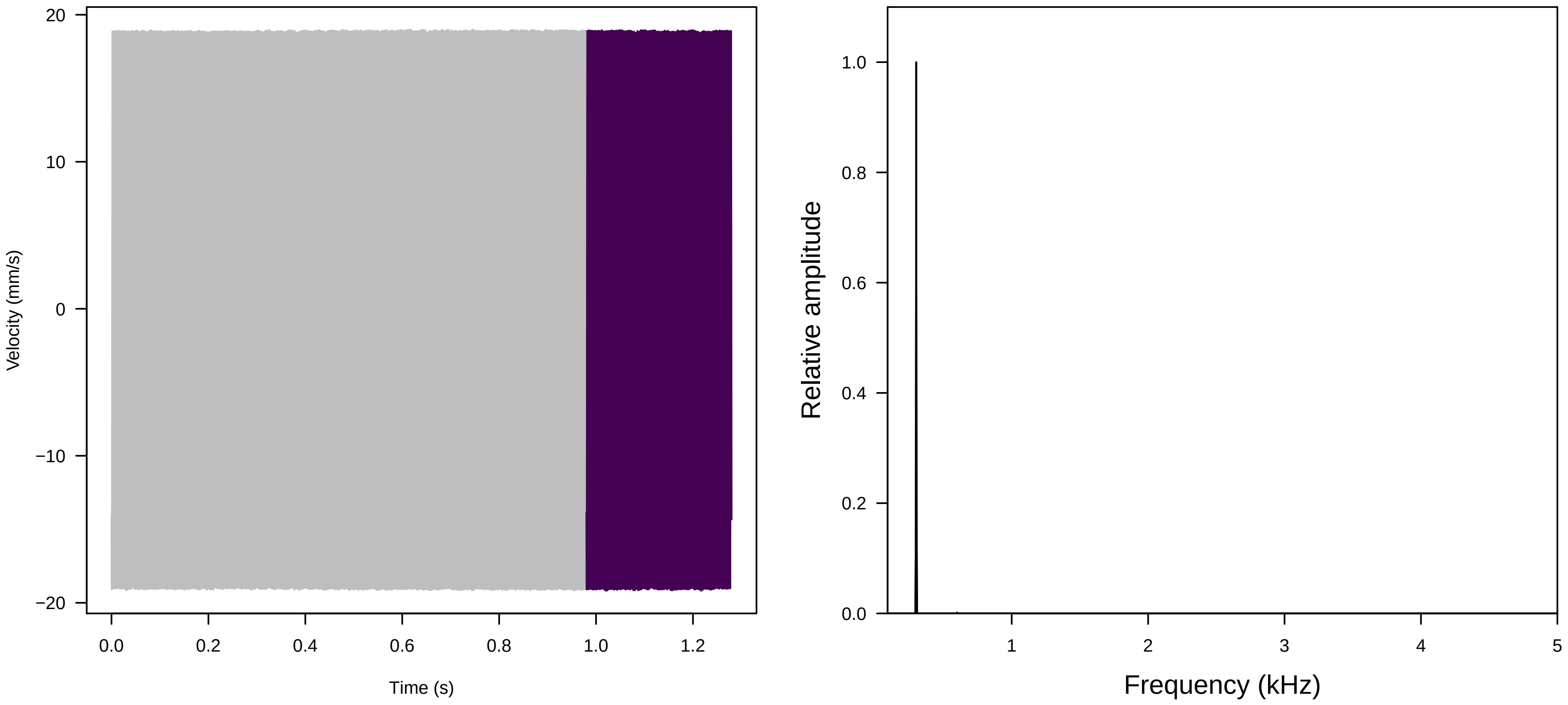

Vel. = 0.014 ; Str. = FA ; Axis = y ; Fl. accession = 10−s−79−27

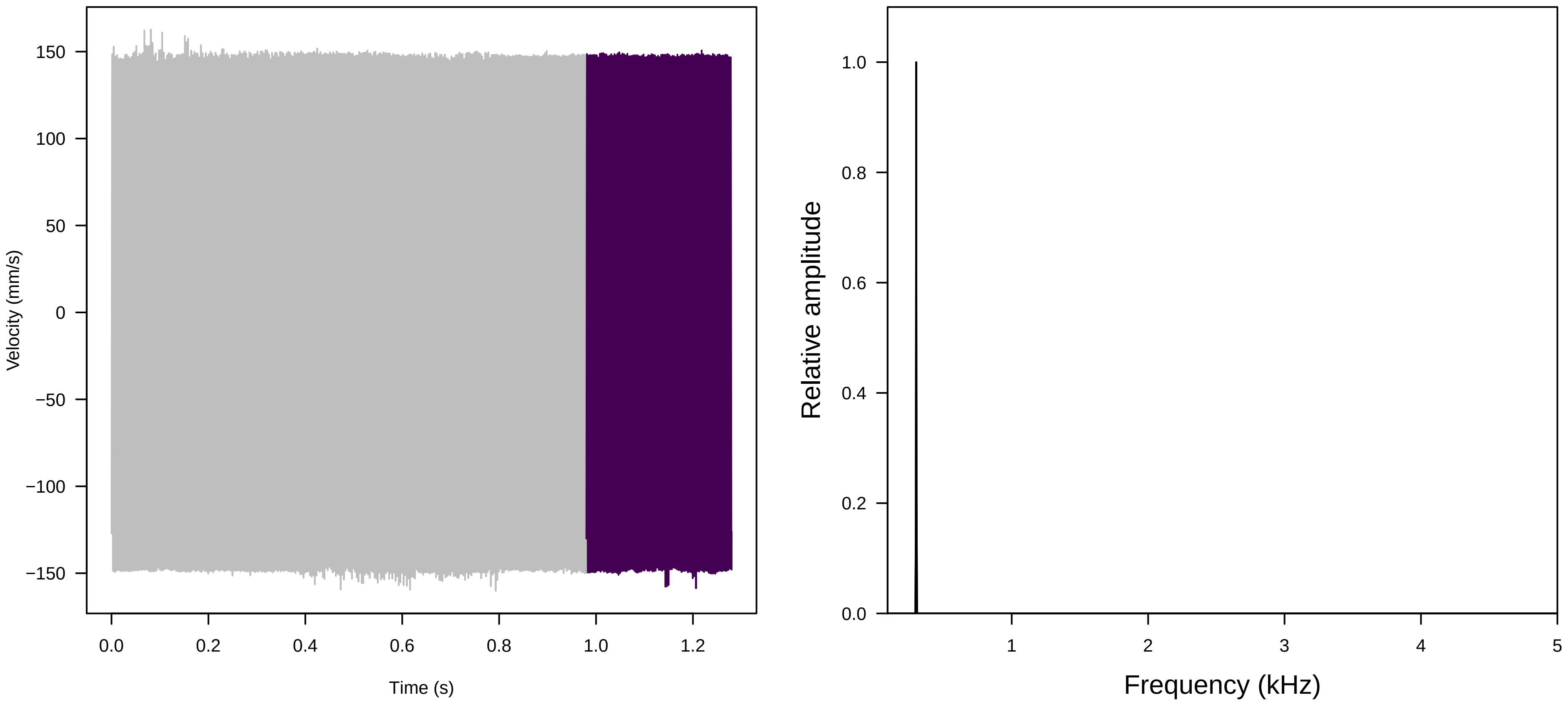

Vel. = 0.014 ; Str. = Receptacle ; Axis = y ; Fl. accession = 10−s−79−27

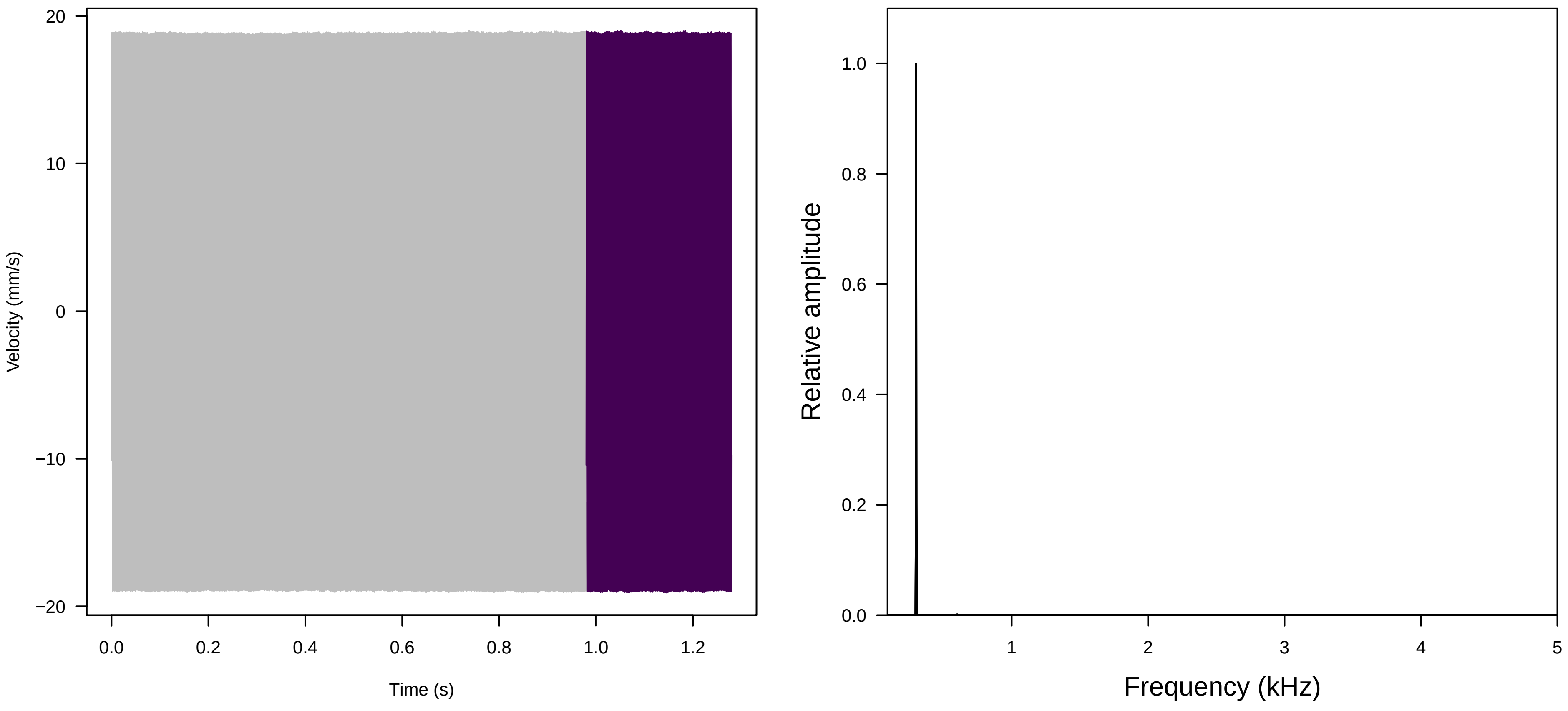

Vel. = 0.014 ; Str. = PA ; Axis = y ; Fl. accession = 10−s−79−27

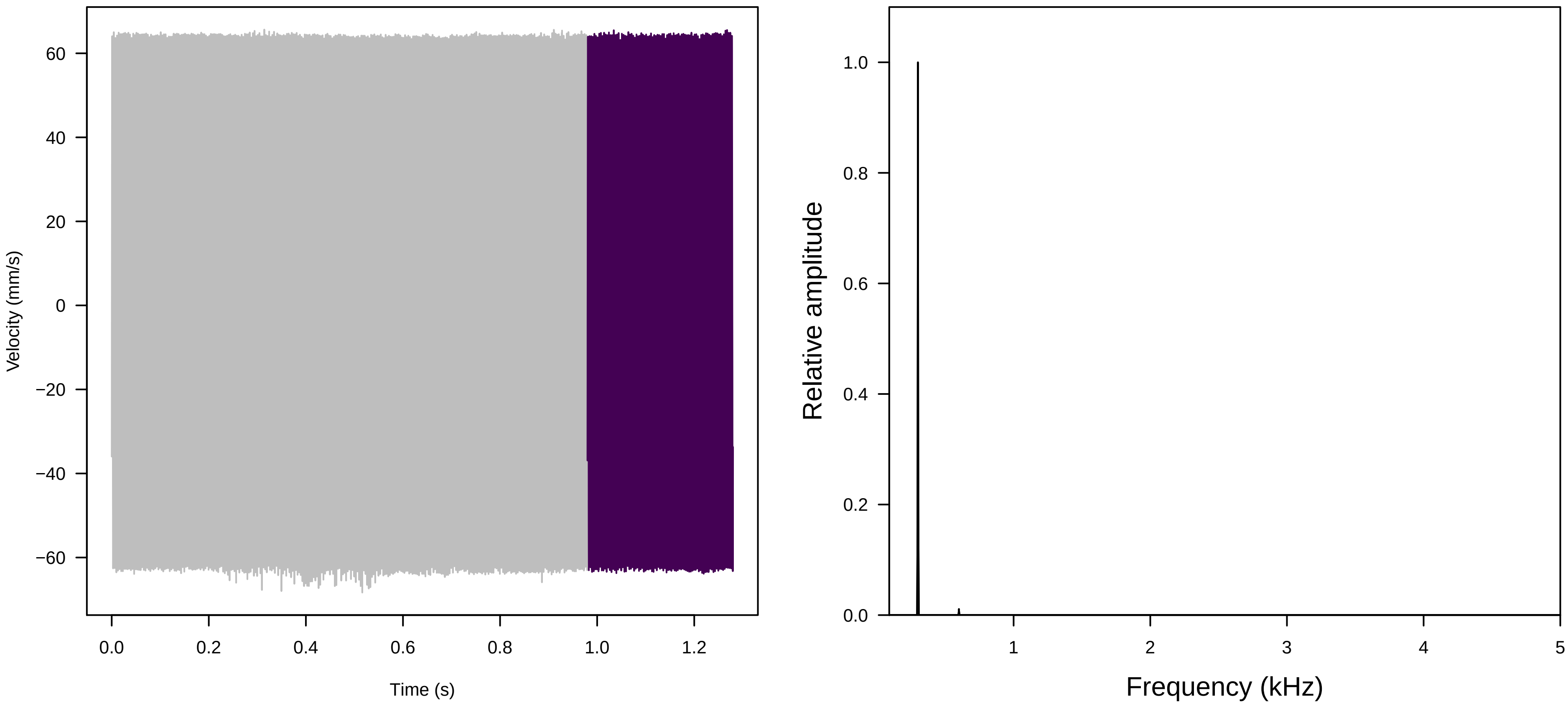

Vel. = 0.014 ; Str. = Receptacle ; Axis = y ; Fl. accession = 10−s−79−27

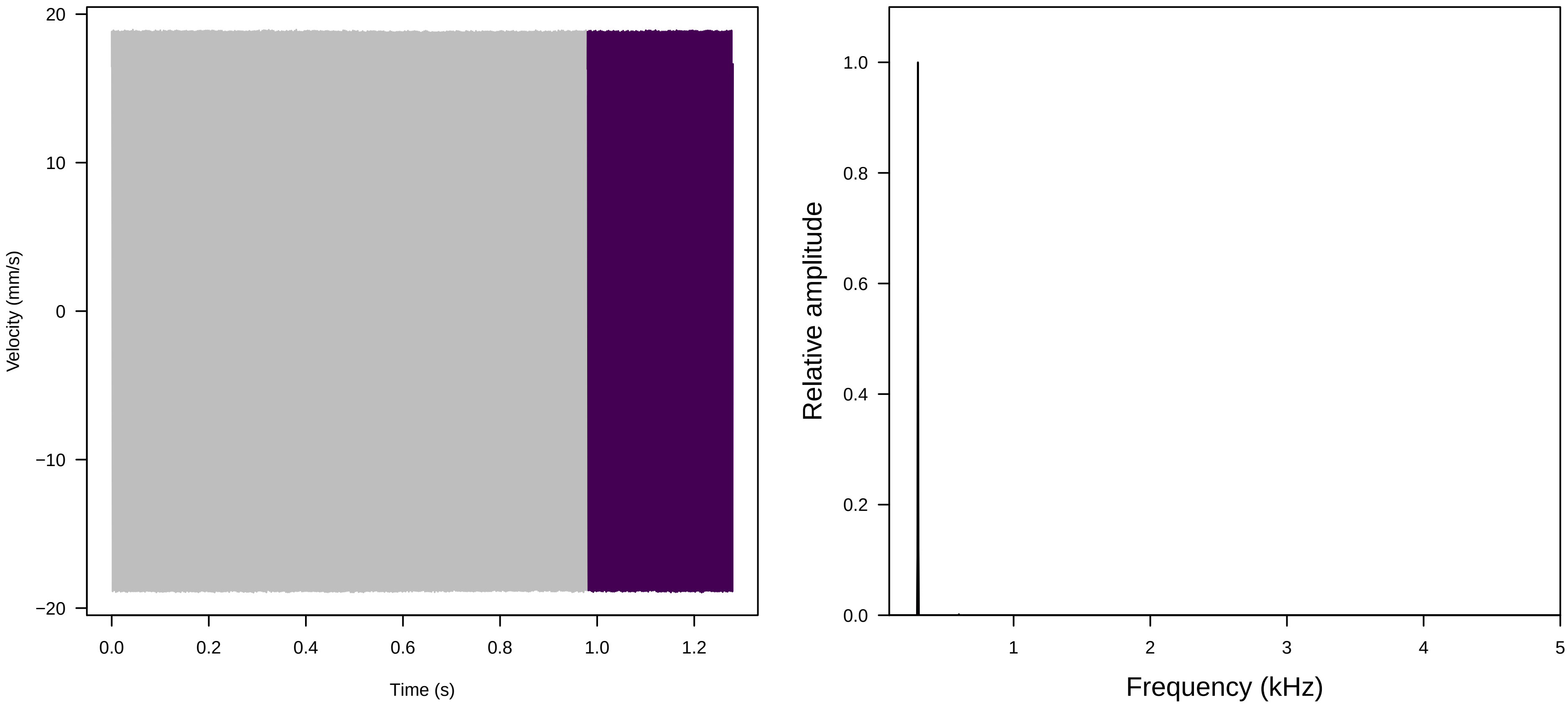

Vel. = 0.028 ; Str. = PA ; Axis = y ; Fl. accession = 10−s−79−27

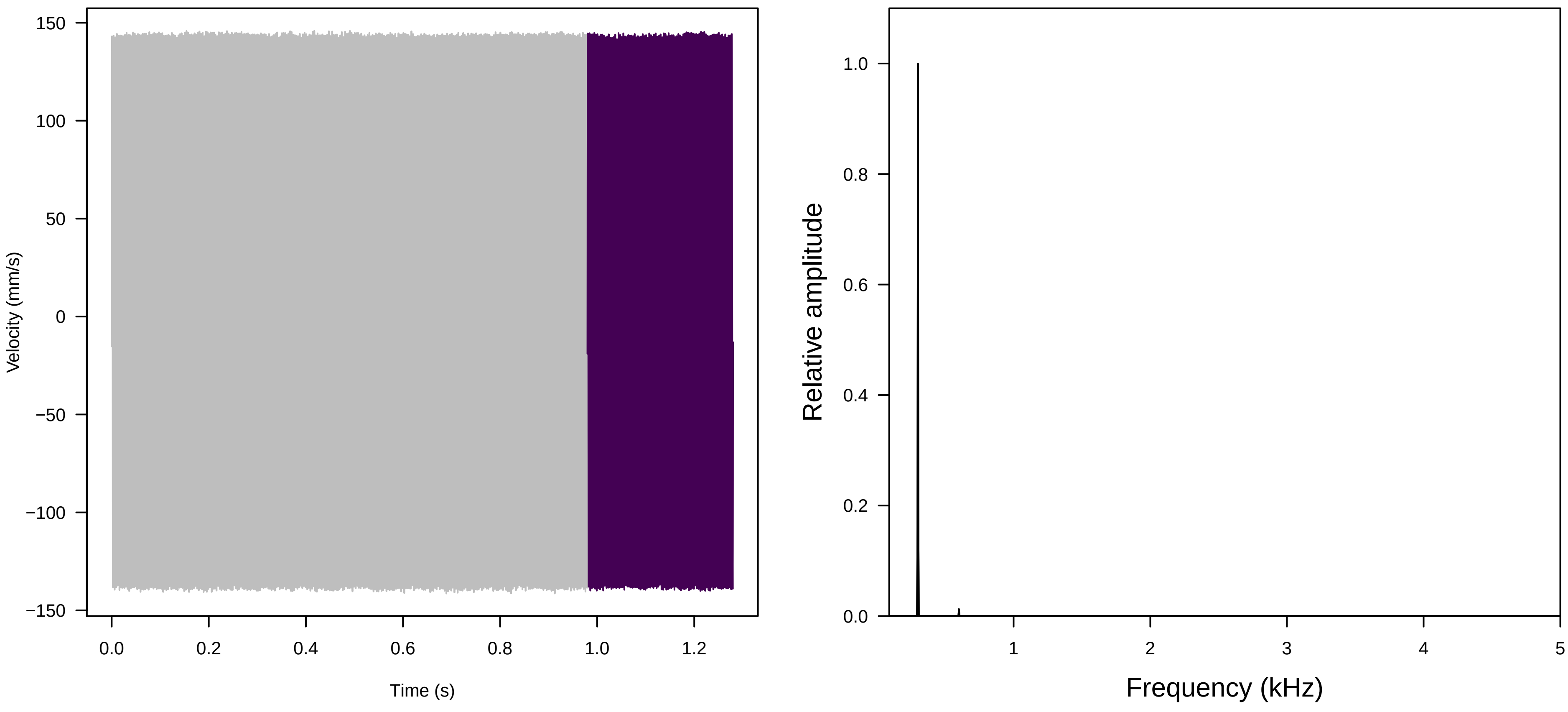

Vel. = 0.028 ; Str. = Receptacle ; Axis = y ; Fl. accession = 10−s−79−27

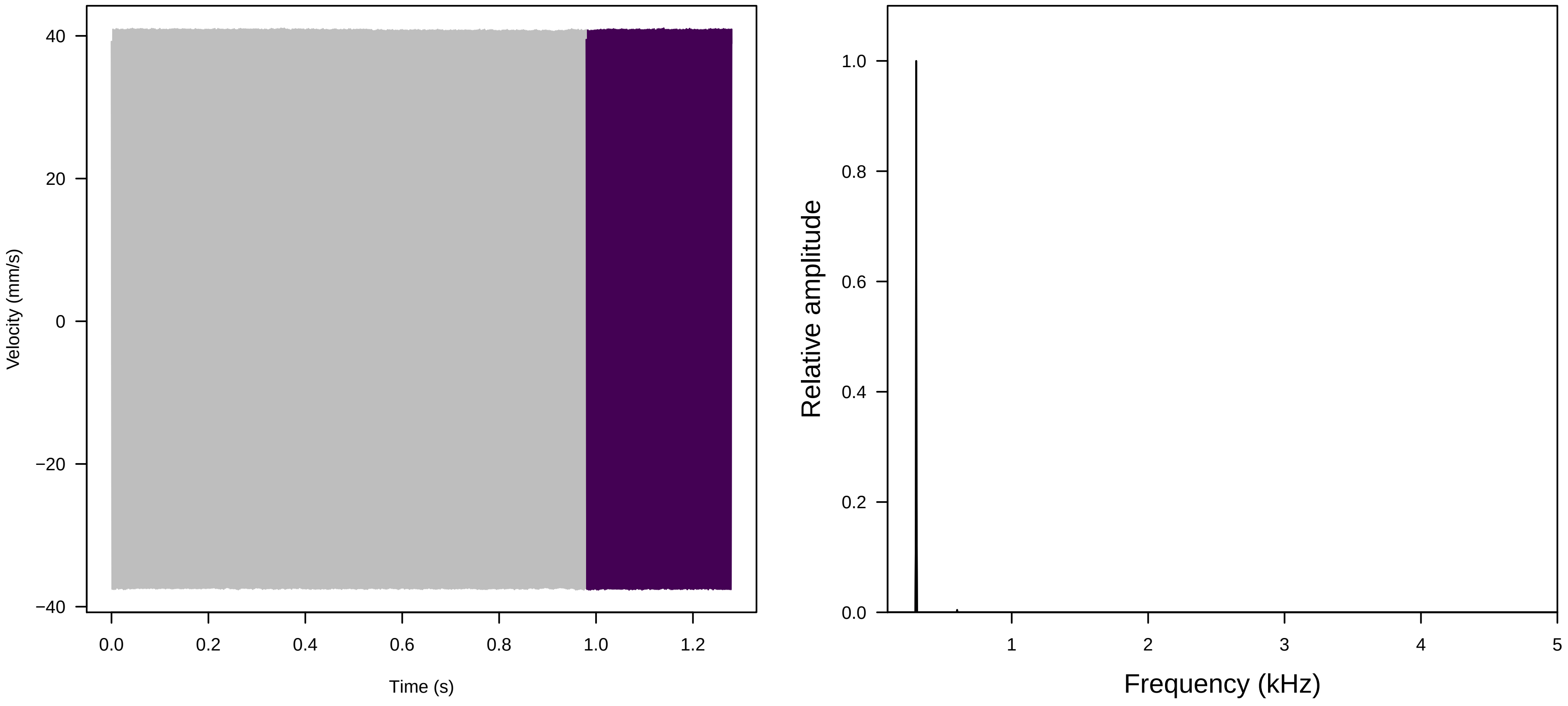

Vel. = 0.028 ; Str. = FA ; Axis = y ; Fl. accession = 10−s−79−27

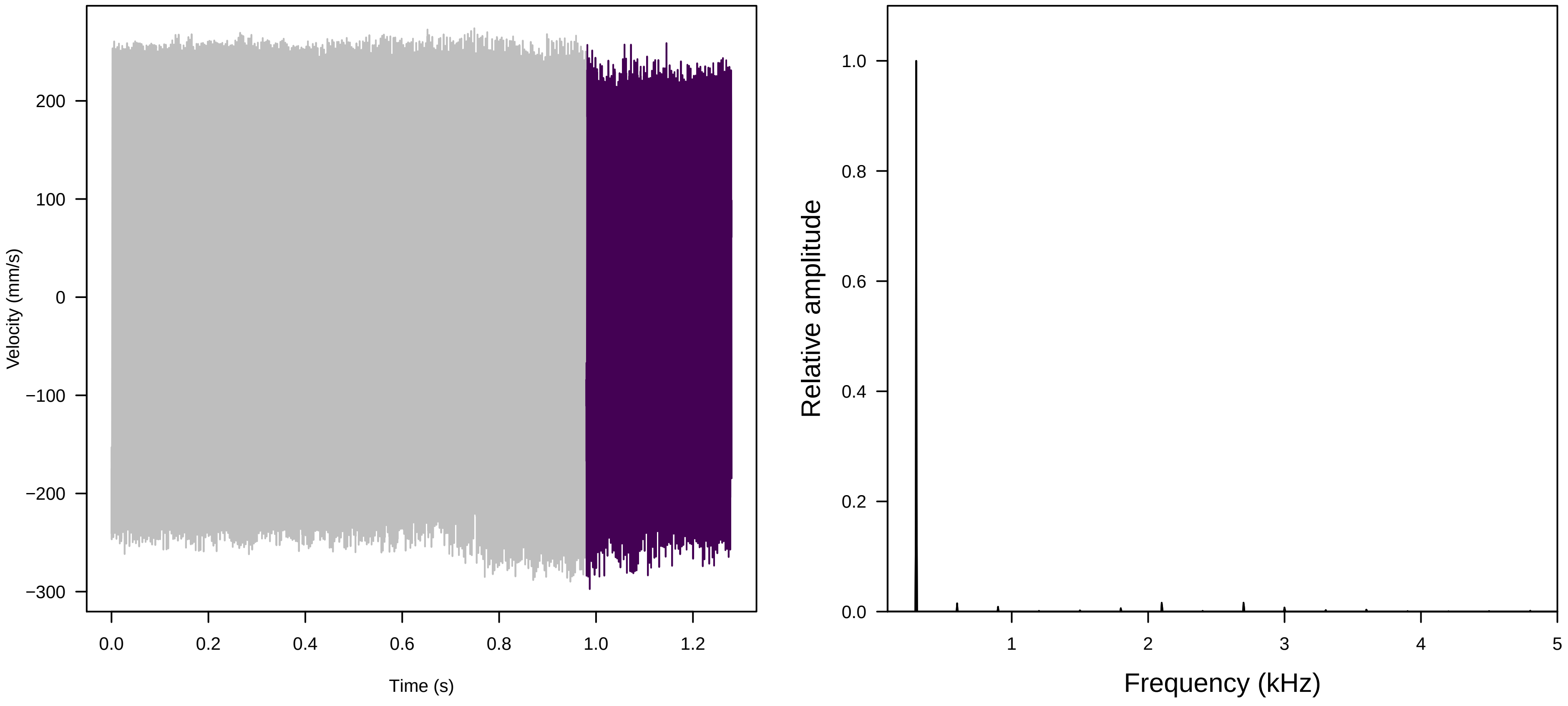

Vel. = 0.028 ; Str. = Receptacle ; Axis = y ; Fl. accession = 10−s−79−27

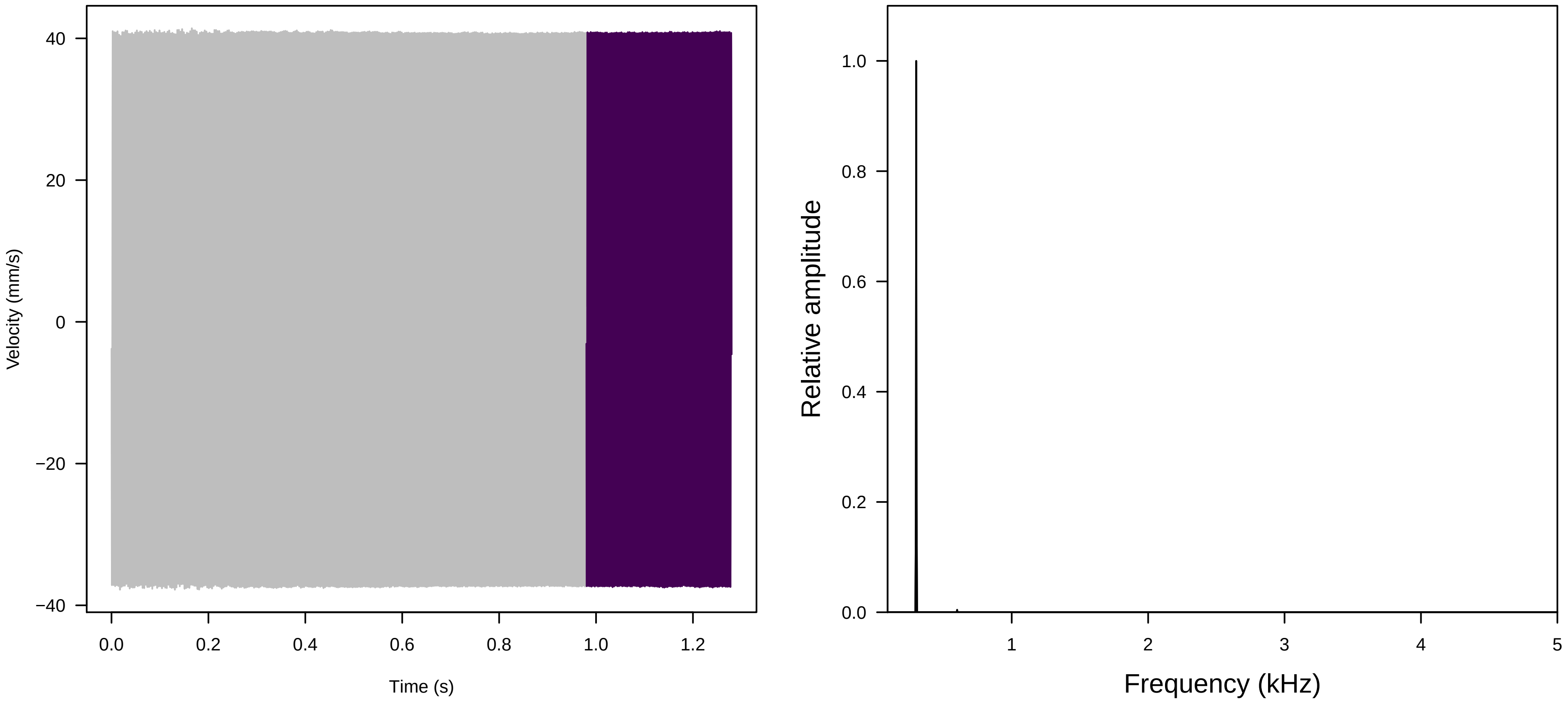

Vel. = 0.028 ; Str. = Corolla ; Axis = y ; Fl. accession = 10−s−79−27

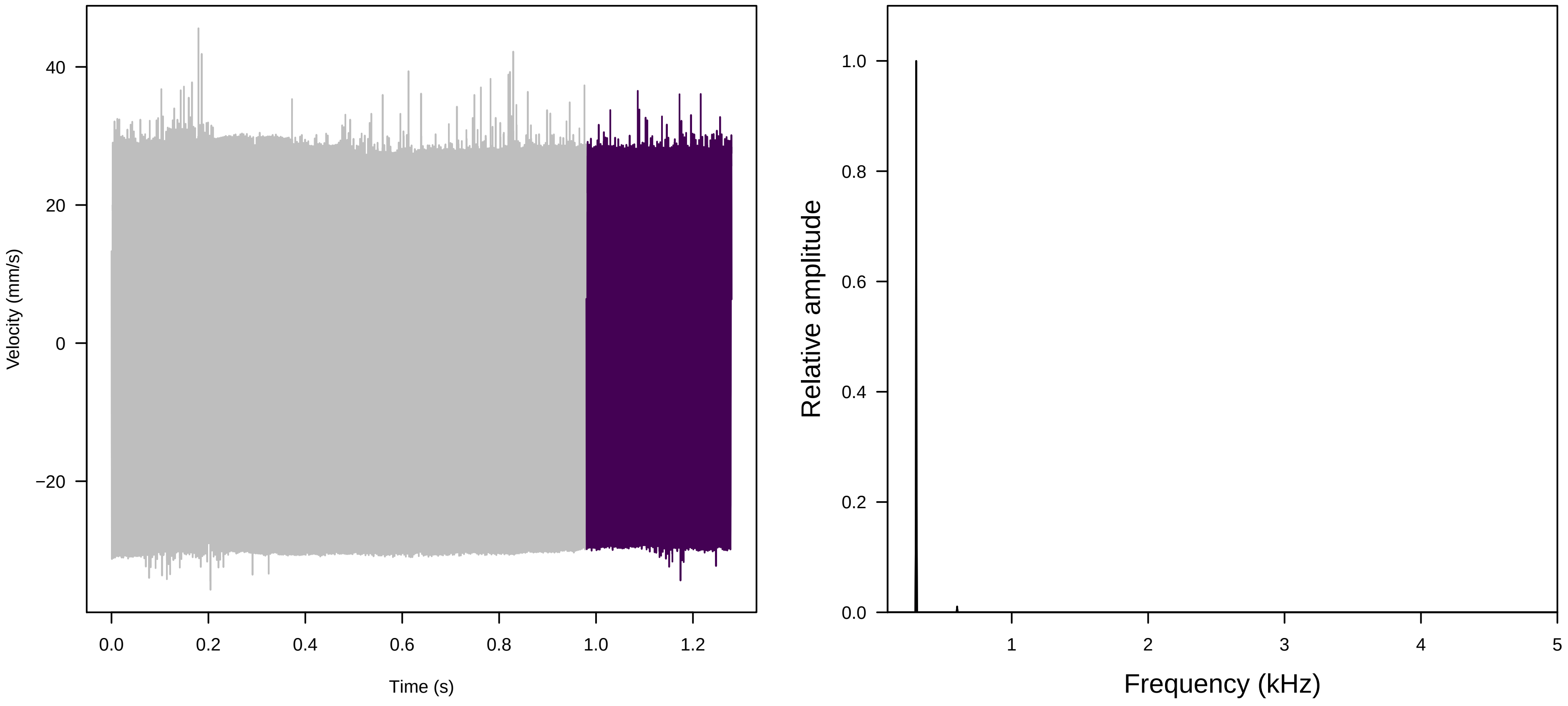

Vel. = 0.028 ; Str. = Receptacle ; Axis = y ; Fl. accession = 10−s−79−27

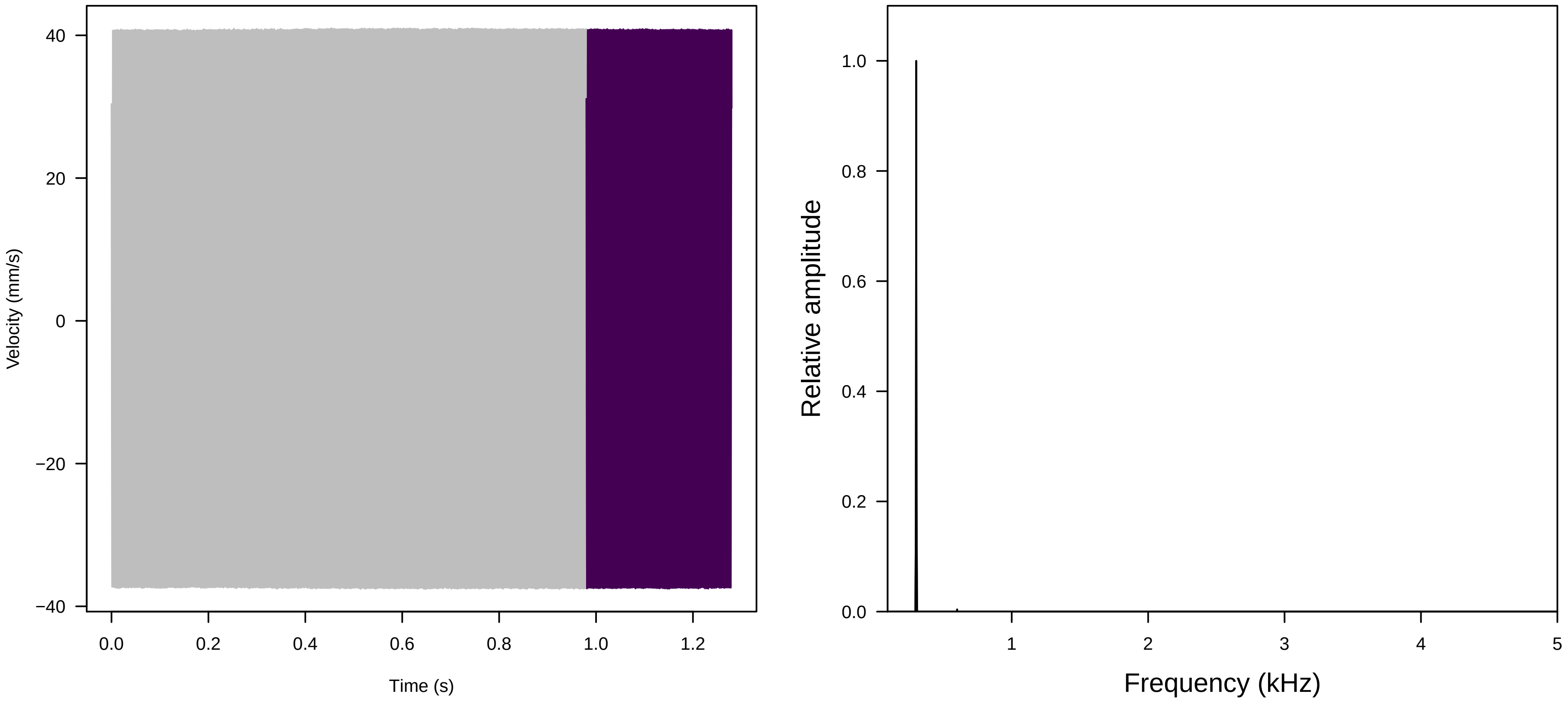

Vel. = 0.057 ; Str. = Corolla ; Axis = y ; Fl. accession = 10−s−79−27

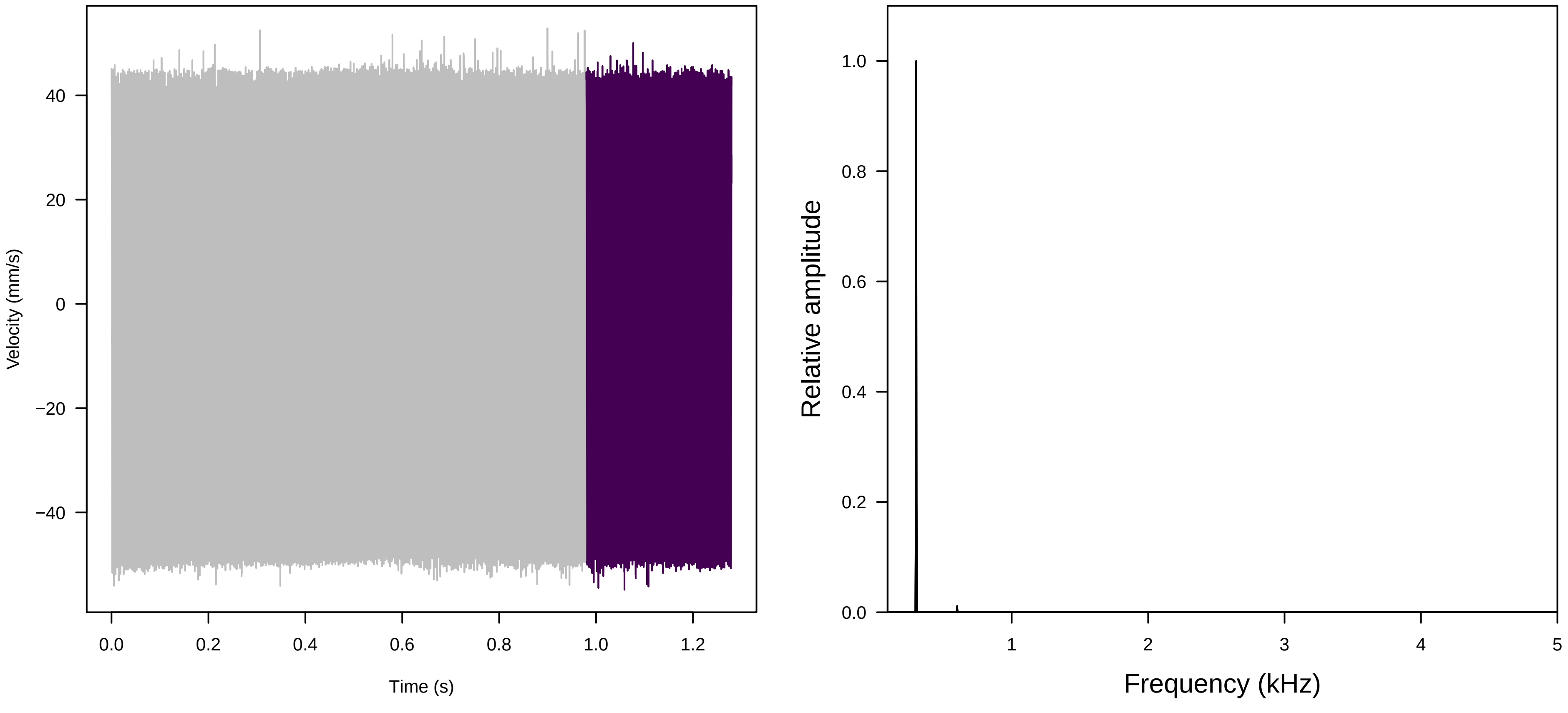

Vel. = 0.057 ; Str. = Receptacle ; Axis = y ; Fl. accession = 10−s−79−27

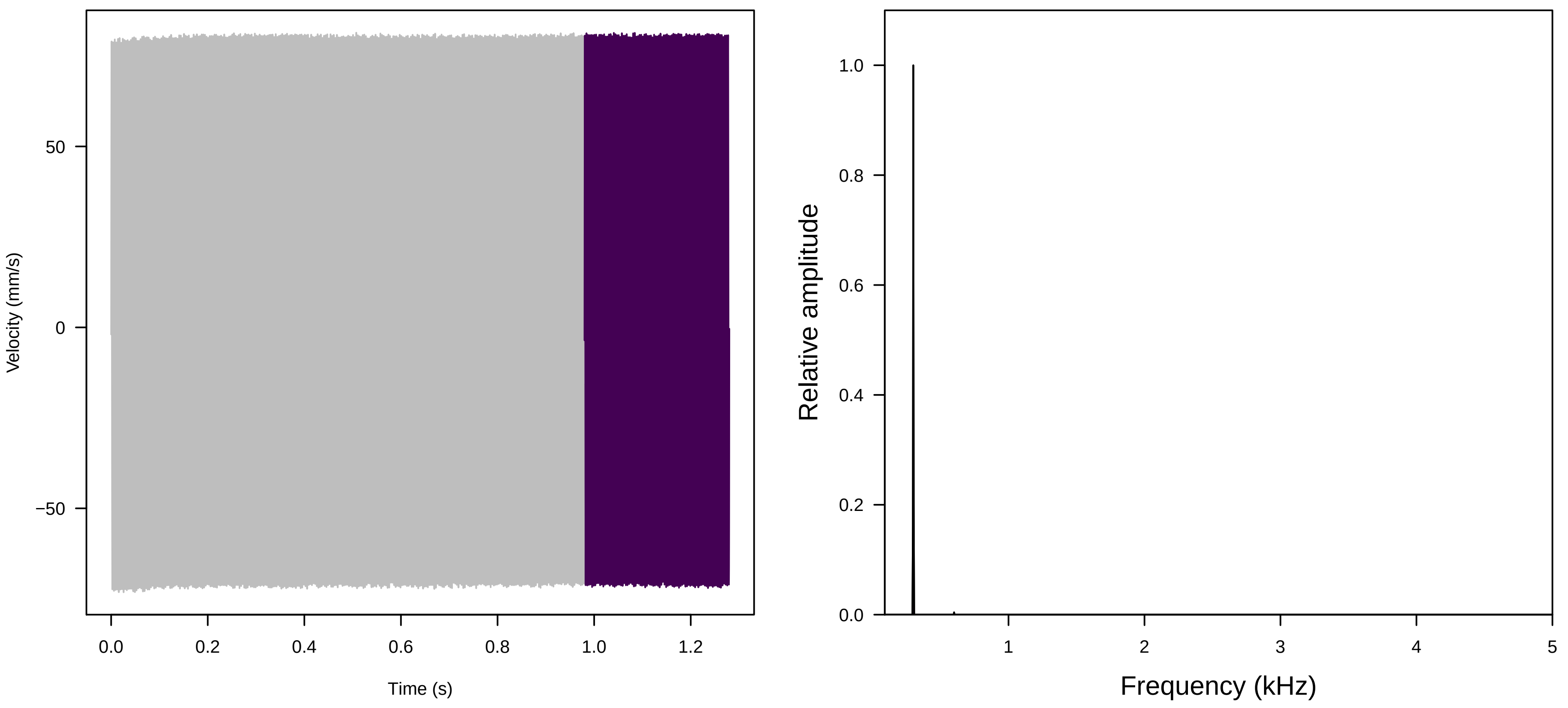

Vel. = 0.057 ; Str. = FA ; Axis = y ; Fl. accession = 10−s−79−27

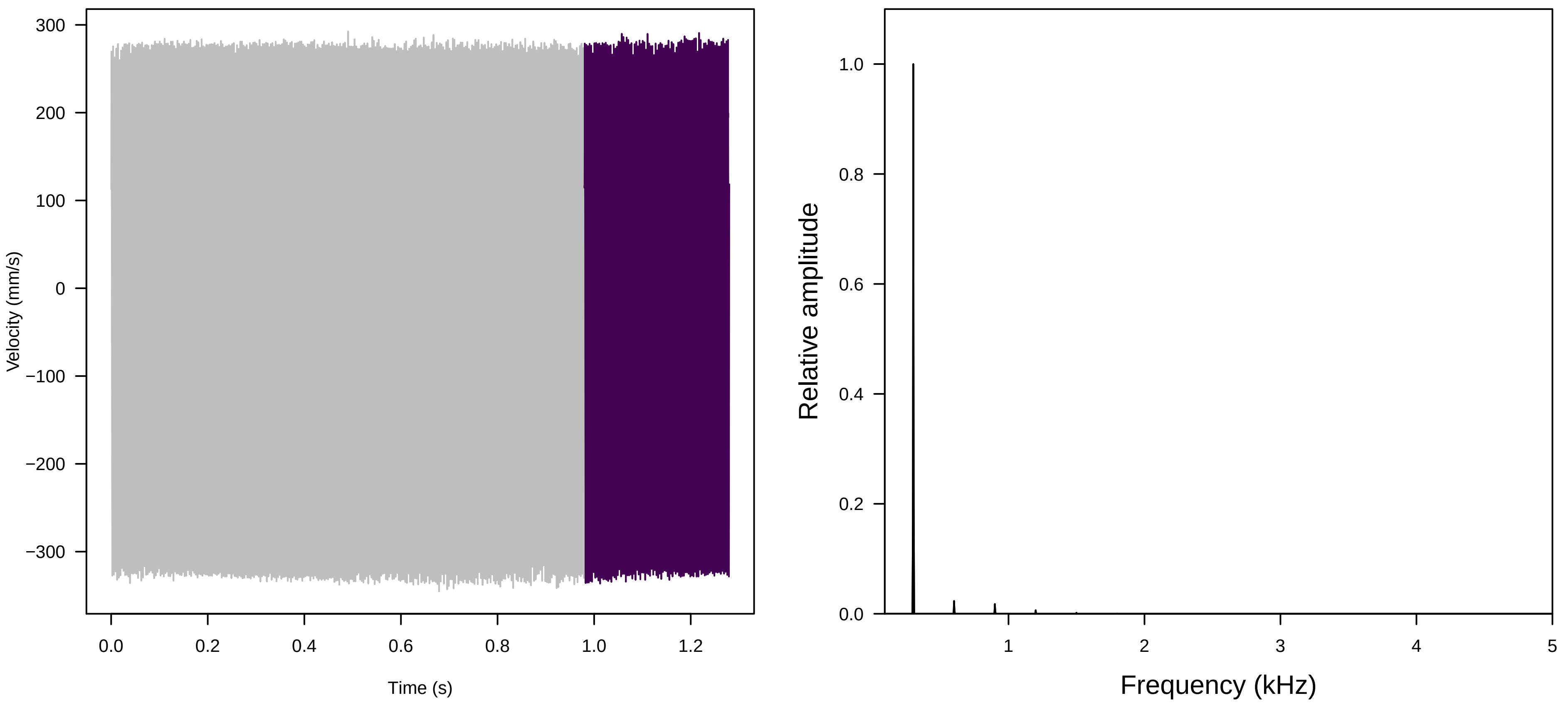

Vel. = 0.057 ; Str. = Receptacle ; Axis = y ; Fl. accession = 10−s−79−27

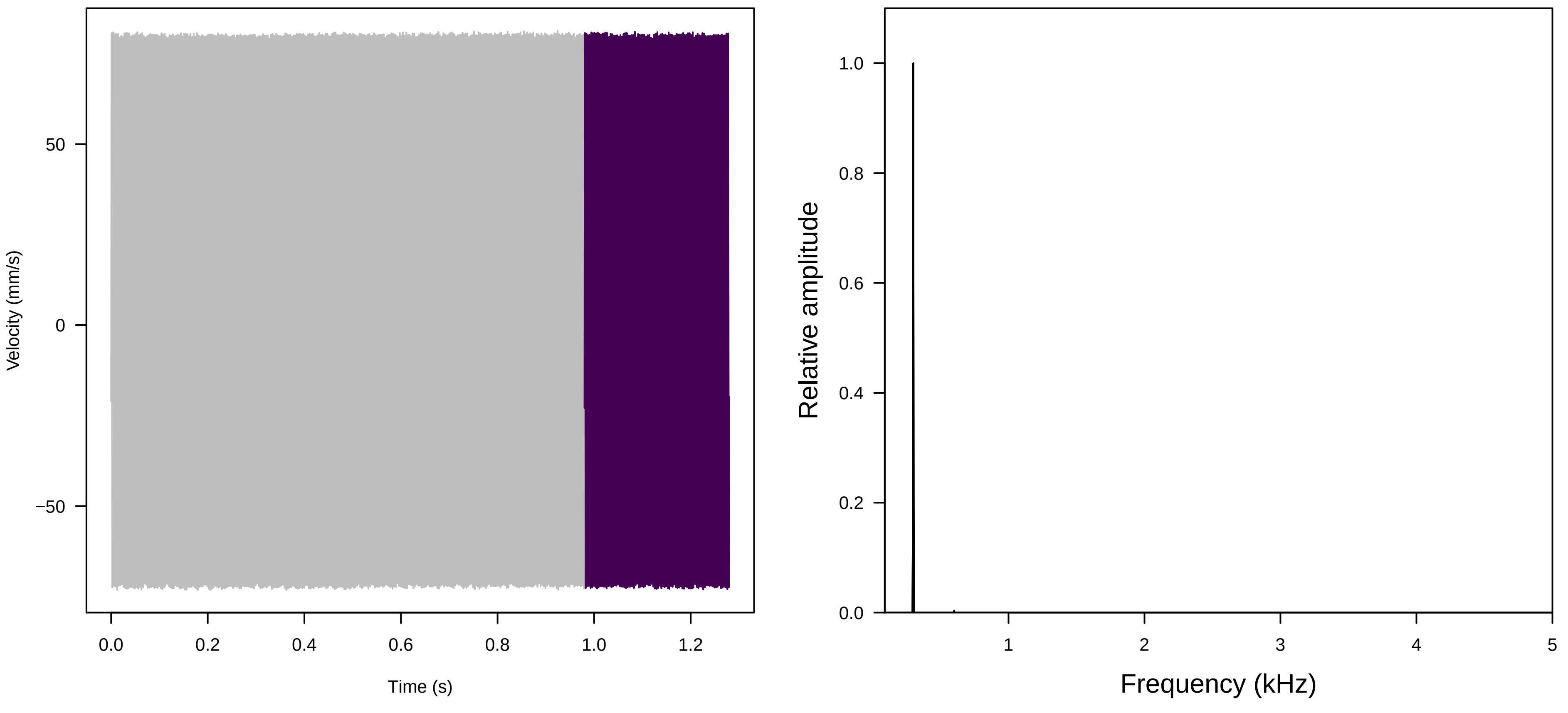

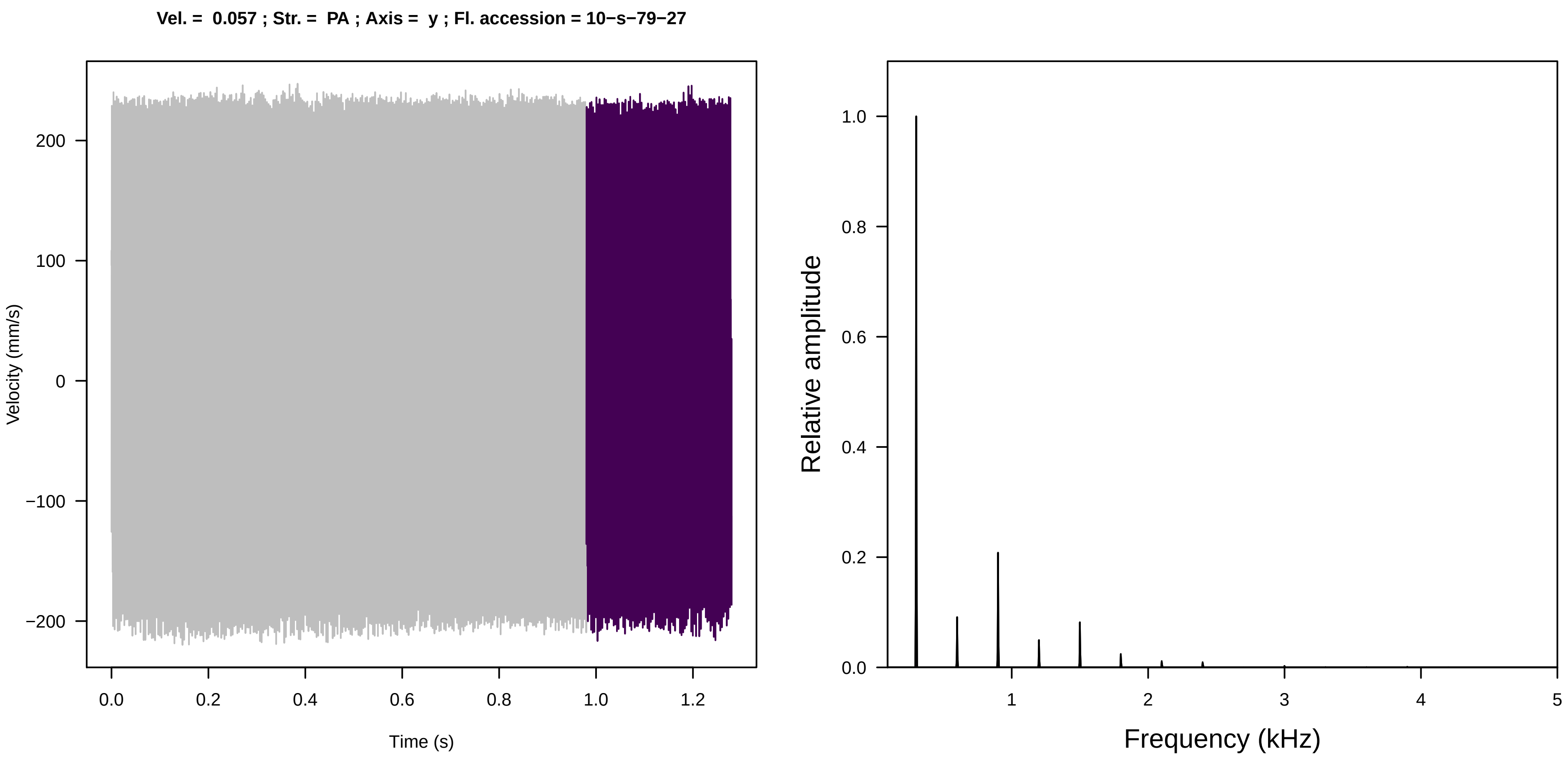
Vel. = 0.057 ; Str. = PA ; Axis = y ; Fl. accession = 10−s−79−27
Velocity (mm/s)
Time (s)
Relative amplitude
Frequency (kHz)

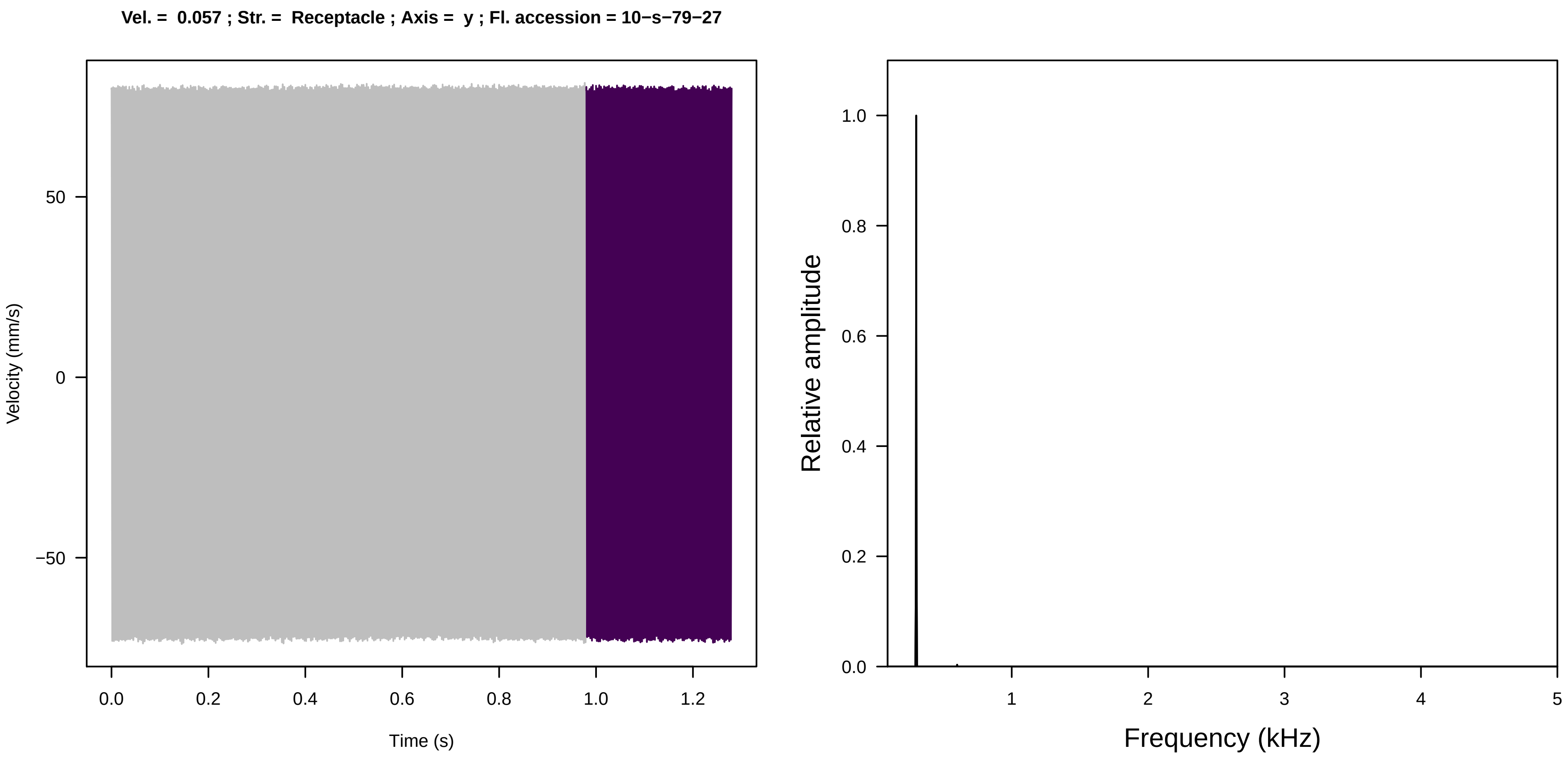
Vel. = 0.057 ; Str. = Receptacle ; Axis = y ; Fl. accession = 10−s−79−27
Velocity (mm/s)
Time (s)
Relative amplitude
Frequency (kHz)

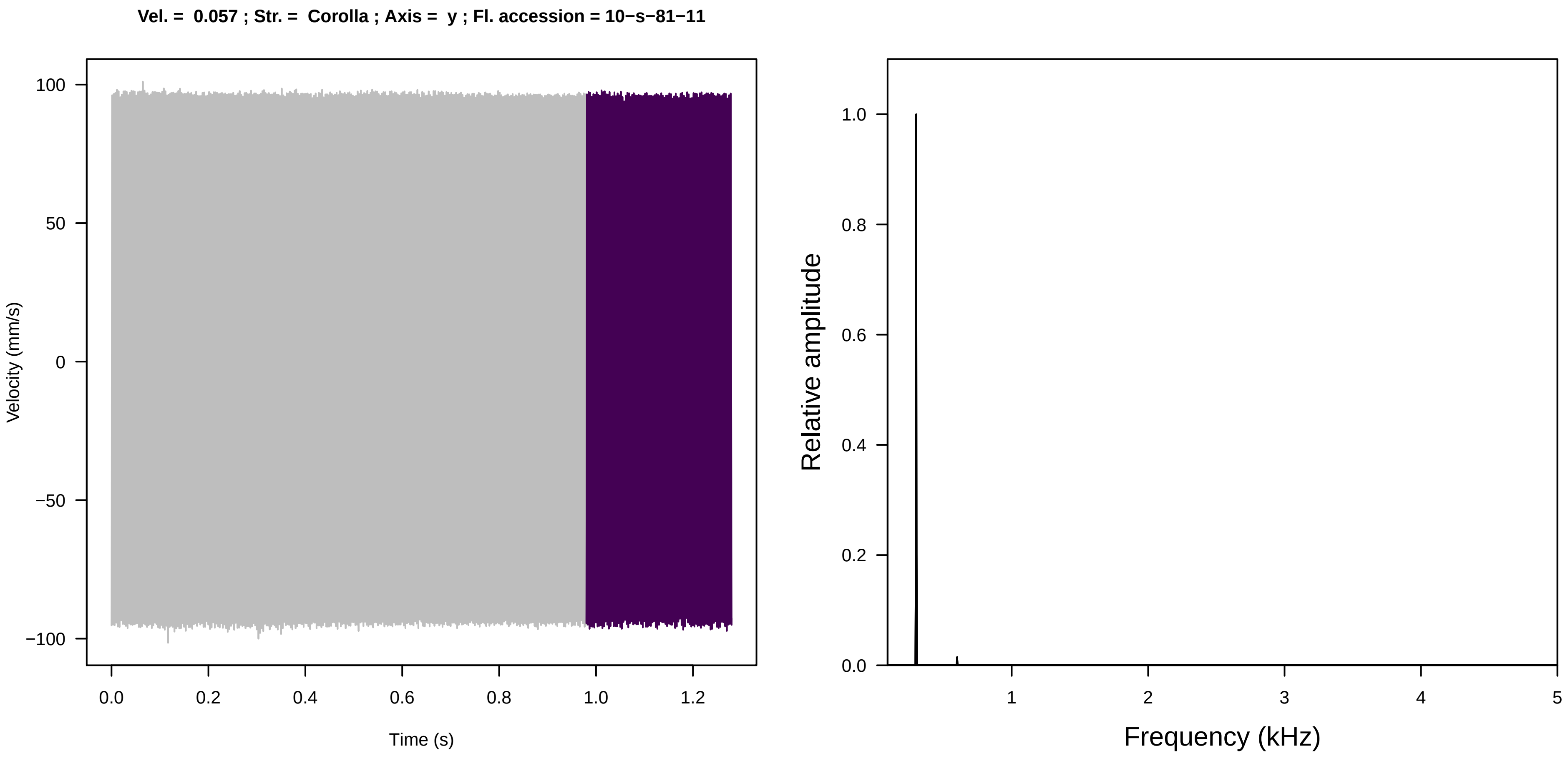
Vel. = 0.057 ; Str. = Corolla ; Axis = y ; Fl. accession = 10−s−81−11
Velocity (mm/s)
Time (s)
Relative amplitude
Frequency (kHz)

Vel. = 0.057 ; Str. = Receptacle ; Axis = y ; Fl. accession = 10−s−81−11

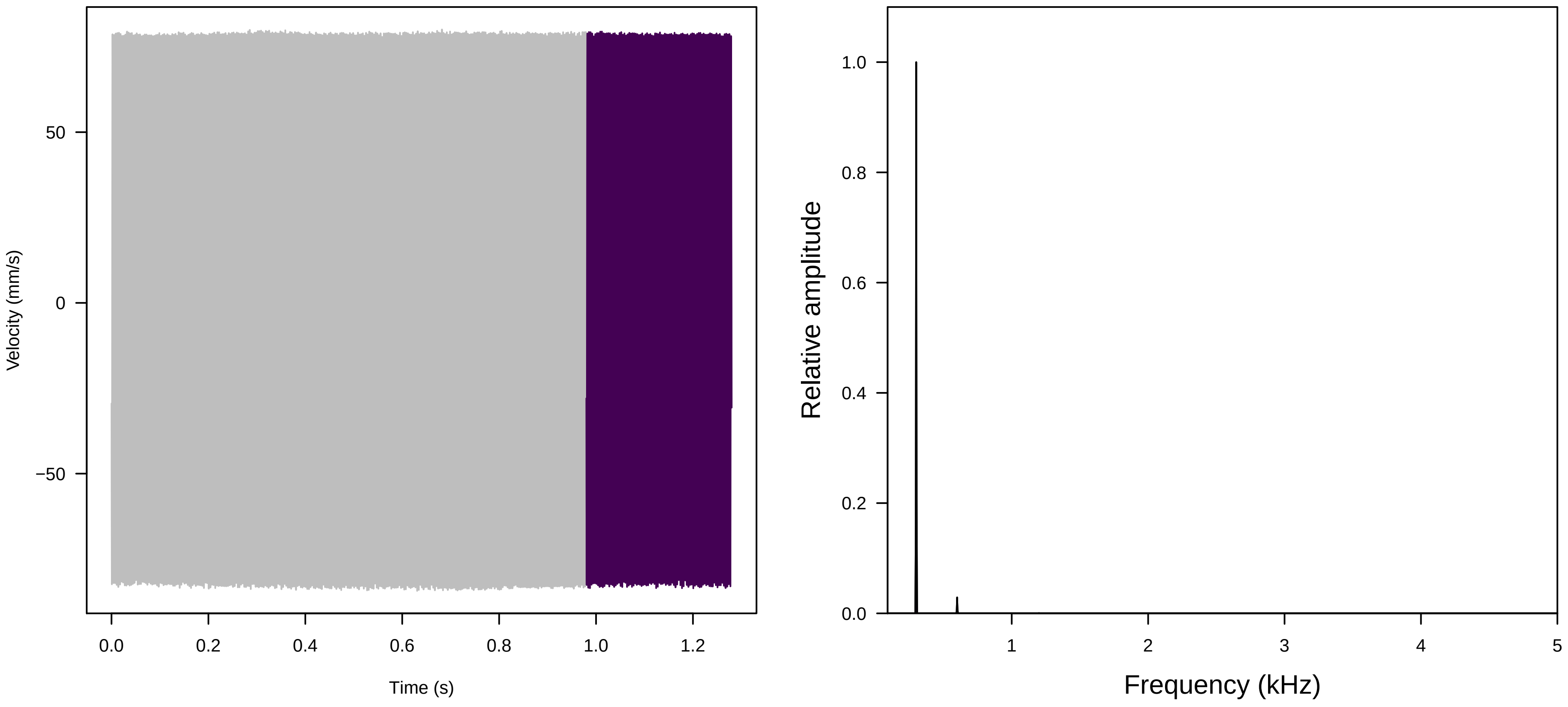

Vel. = 0.057 ; Str. = FA ; Axis = y ; Fl. accession = 10−s−81−11

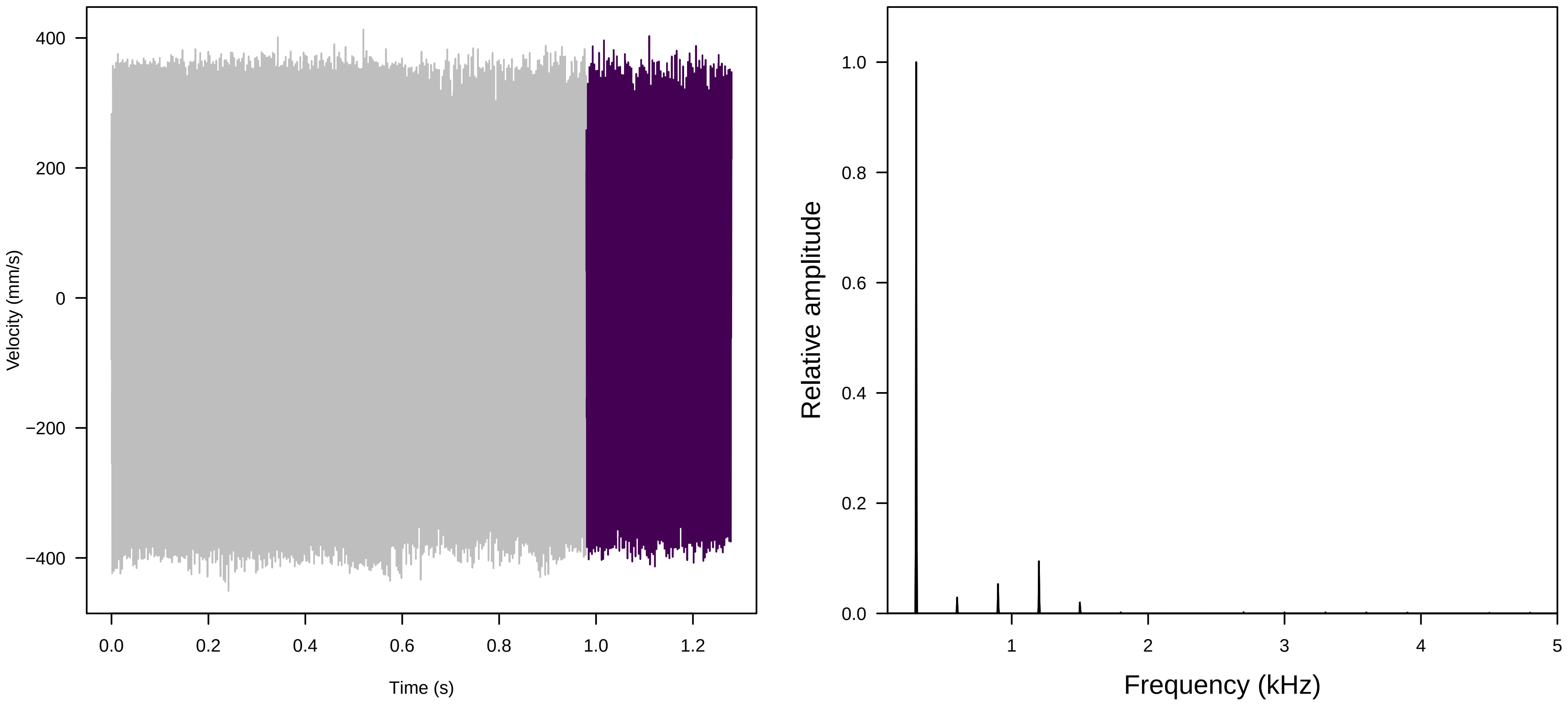

Vel. = 0.057 ; Str. = Receptacle ; Axis = y ; Fl. accession = 10−s−81−11

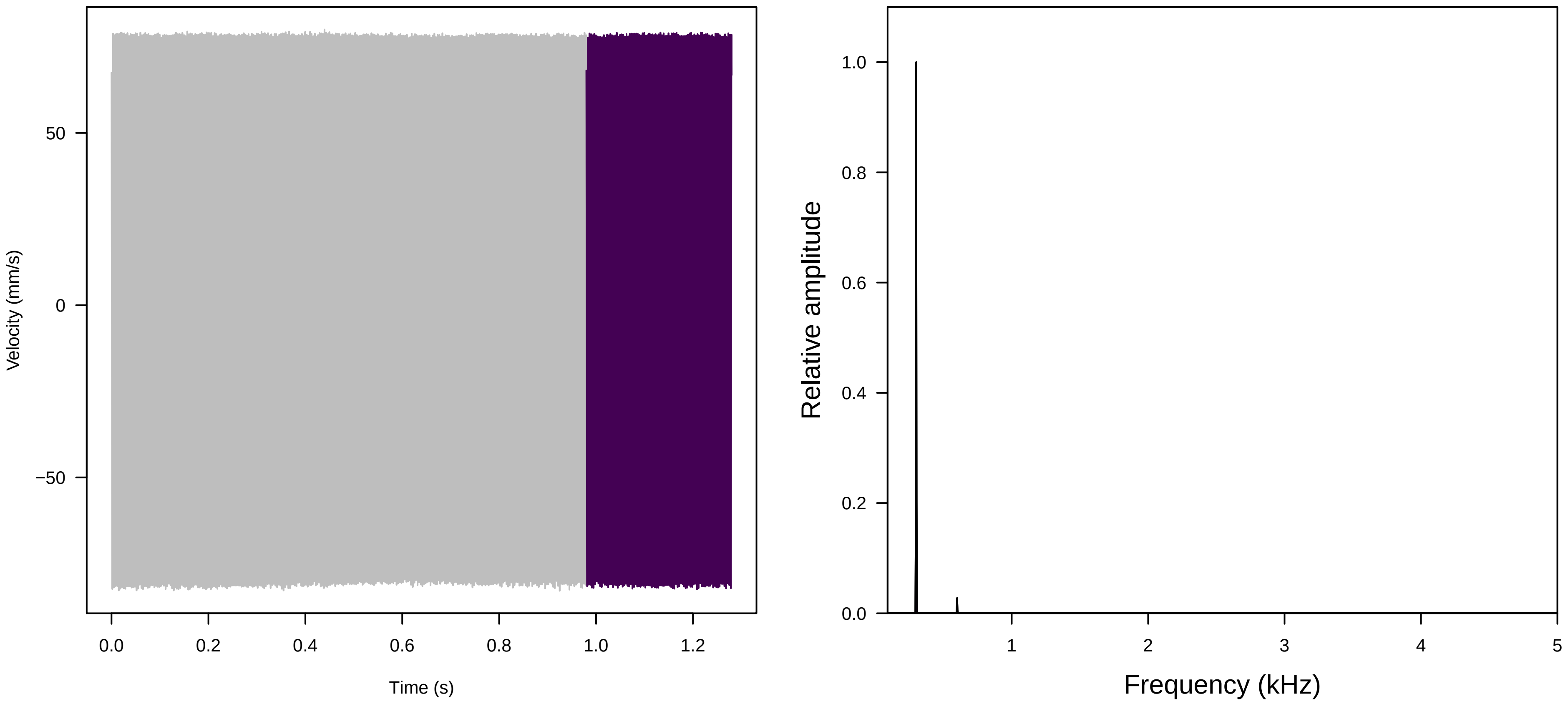

Vel. = 0.057 ; Str. = PA ; Axis = y ; Fl. accession = 10−s−81−11

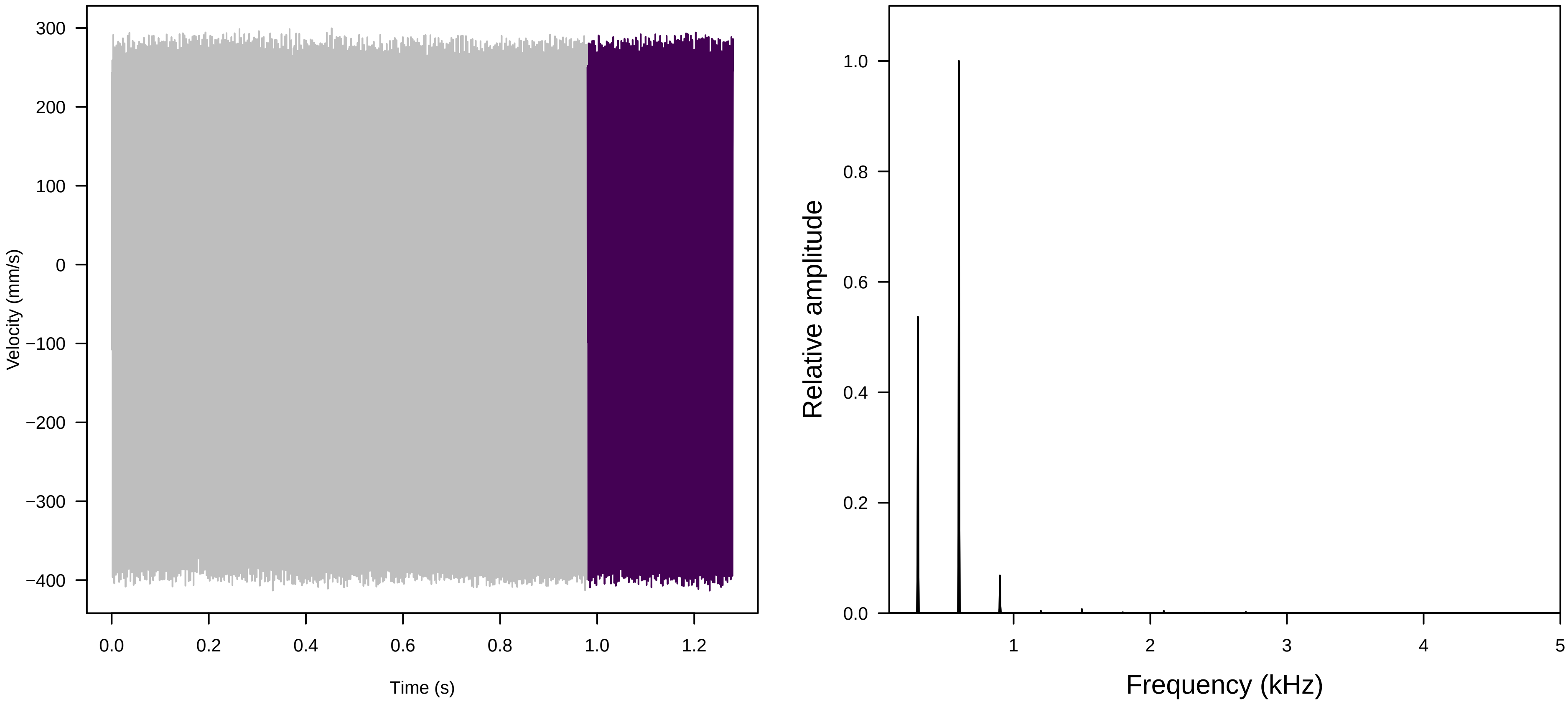

Vel. = 0.057 ; Str. = Receptacle ; Axis = y ; Fl. accession = 10−s−81−11

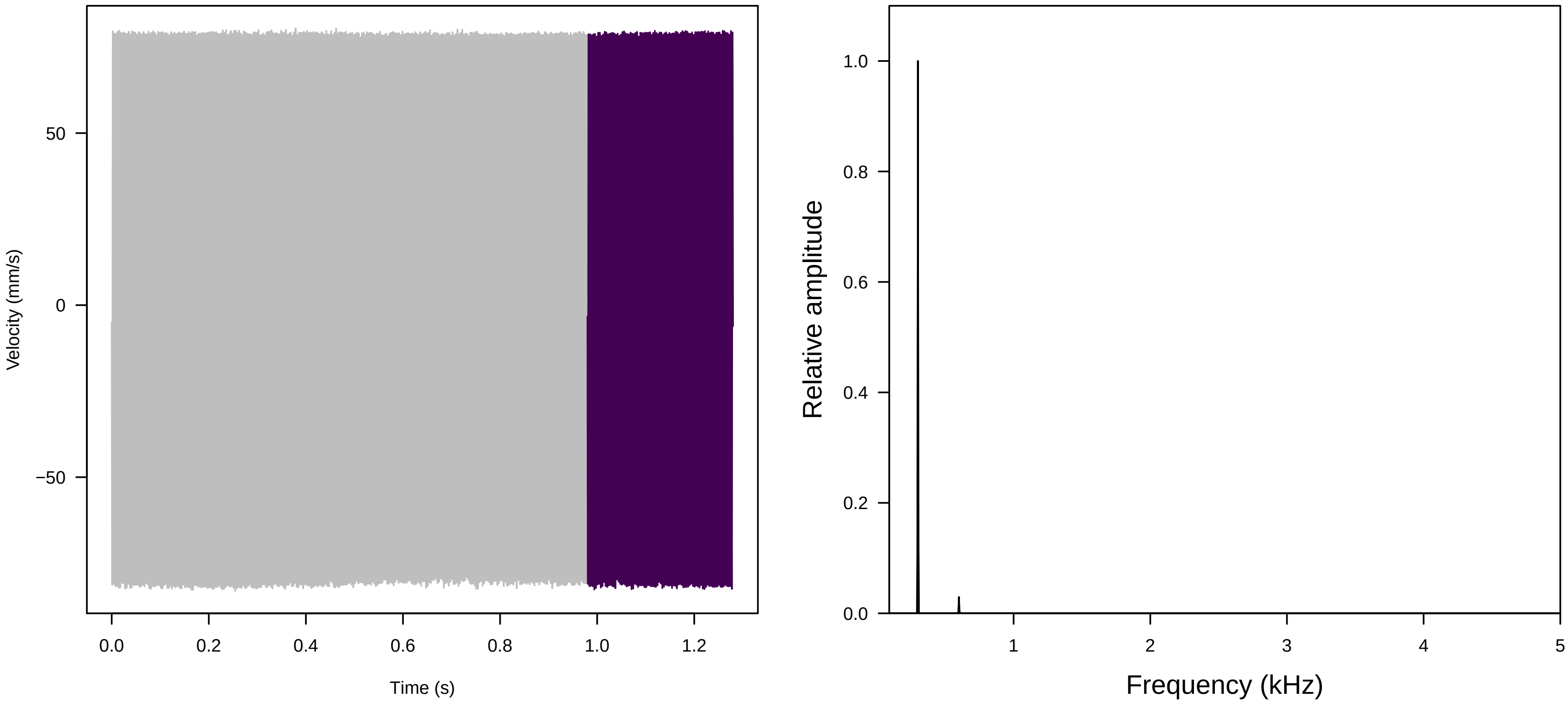

Vel. = 0.028 ; Str. = PA ; Axis = y ; Fl. accession = 10−s−81−11

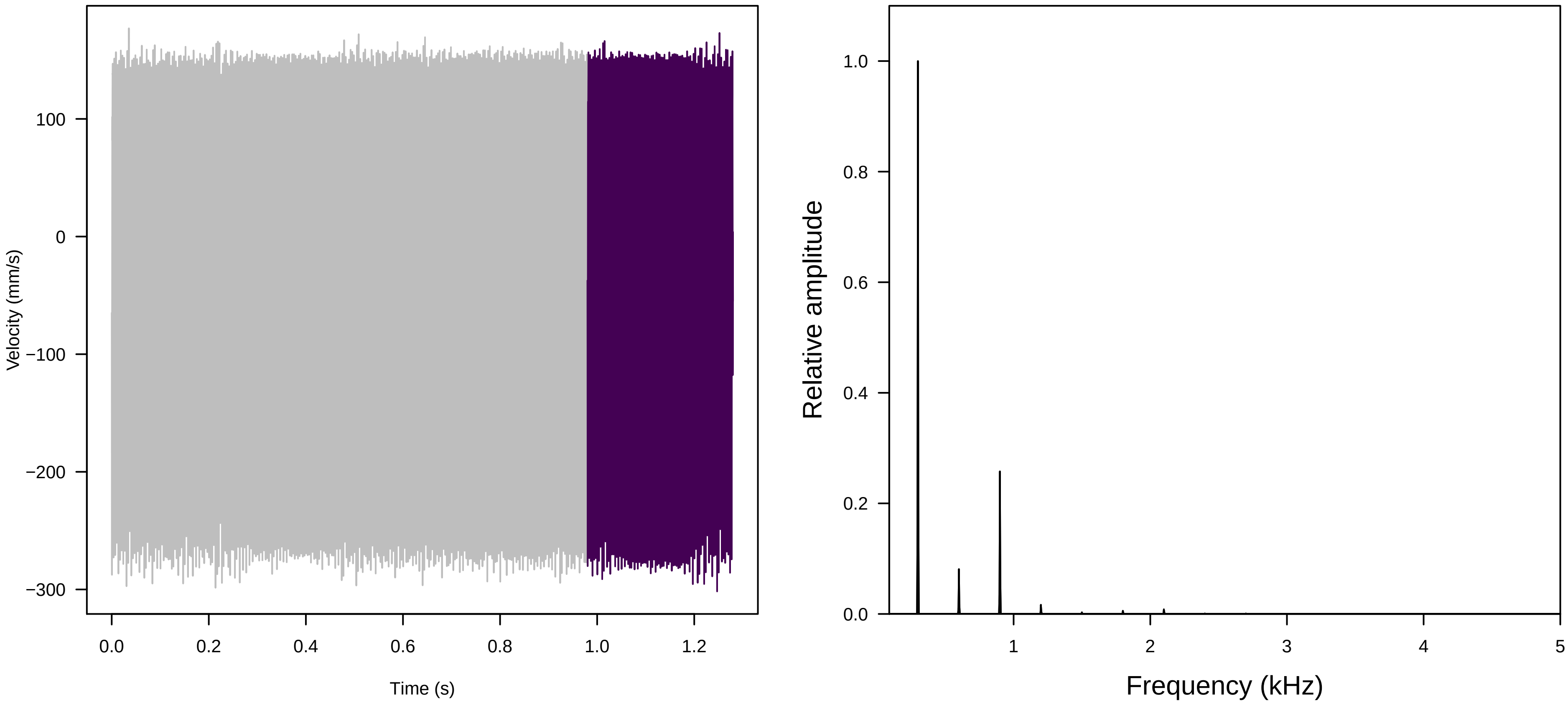

Vel. = 0.028 ; Str. = Receptacle ; Axis = y ; Fl. accession = 10−s−81−11

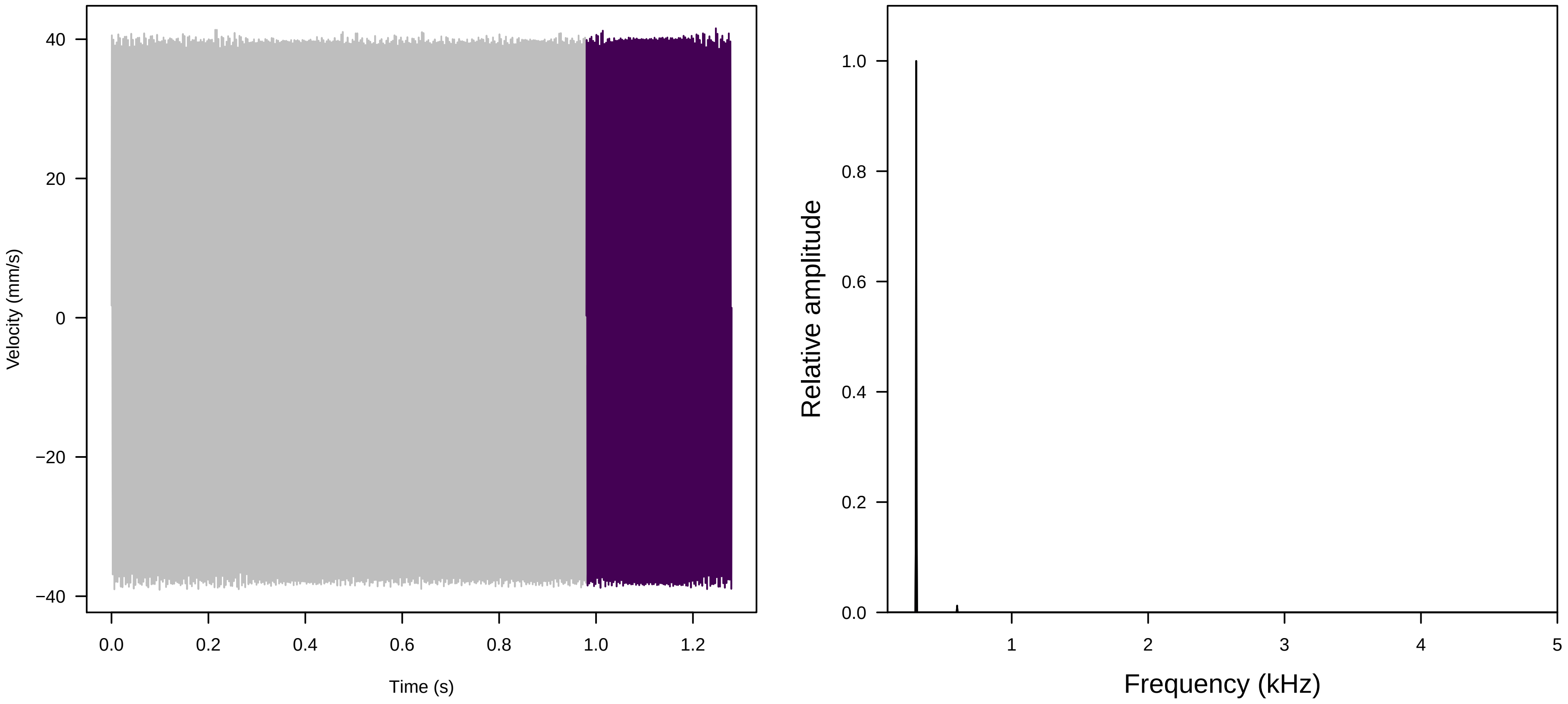

Vel. = 0.028 ; Str. = FA ; Axis = y ; Fl. accession = 10−s−81−11

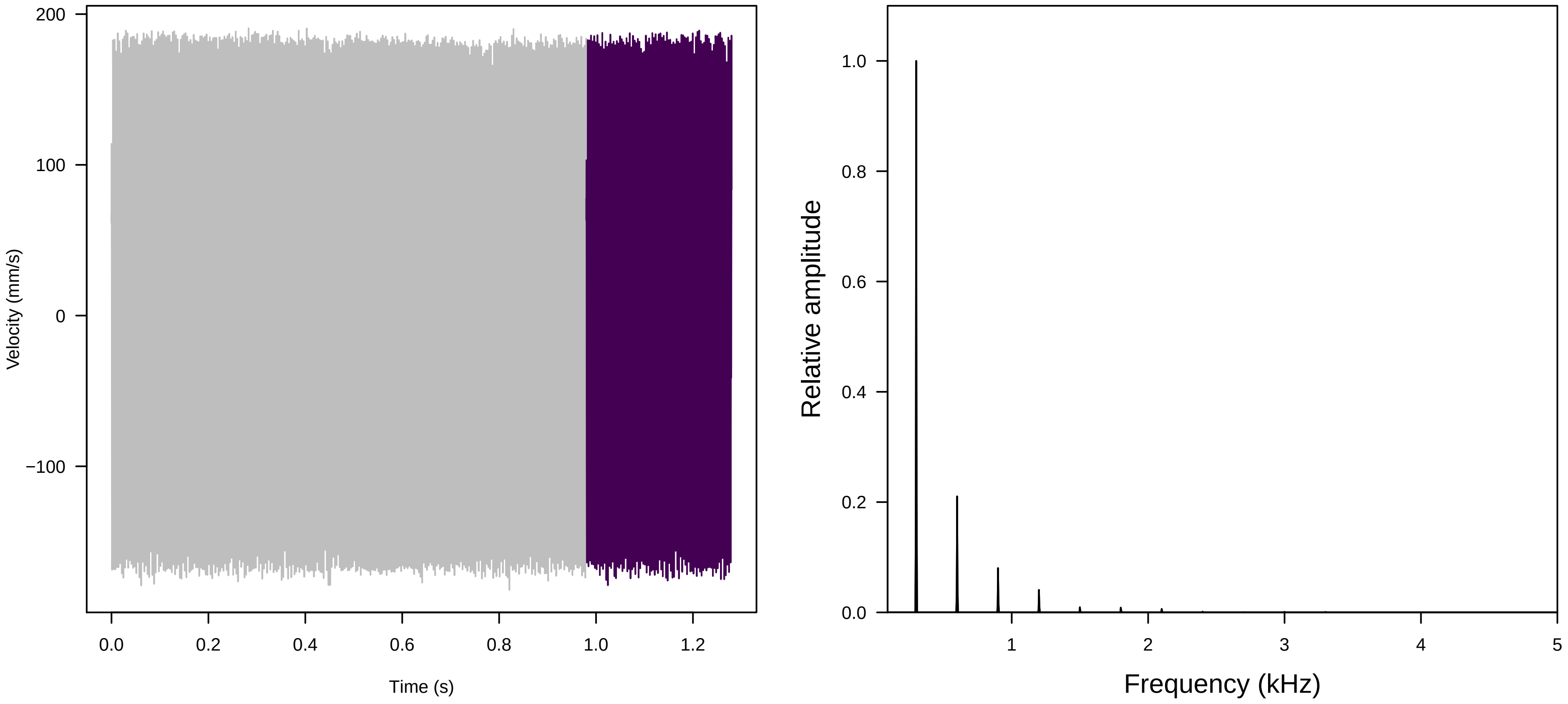

Vel. = 0.028 ; Str. = Receptacle ; Axis = y ; Fl. accession = 10−s−81−11

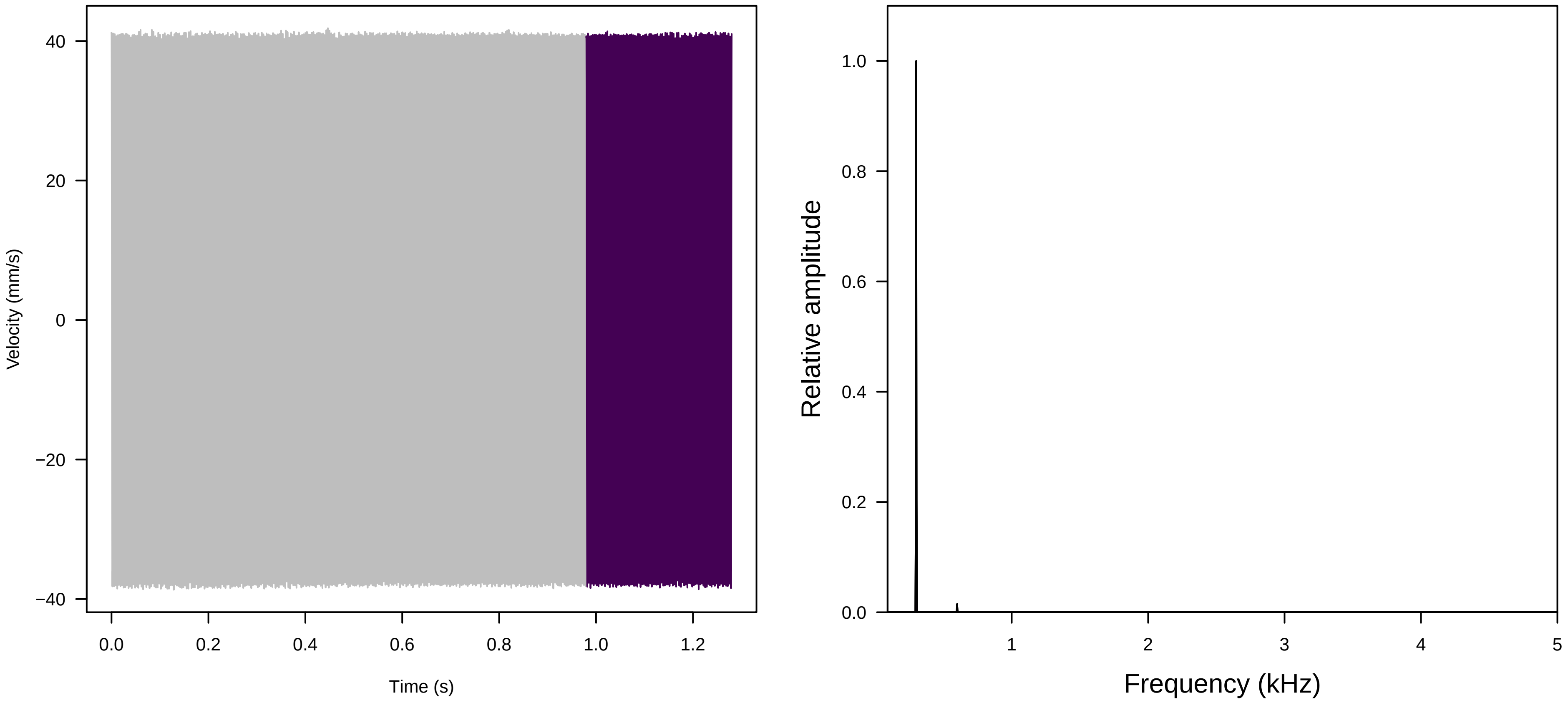

Vel. = 0.028 ; Str. = Corolla ; Axis = y ; Fl. accession = 10−s−81−11

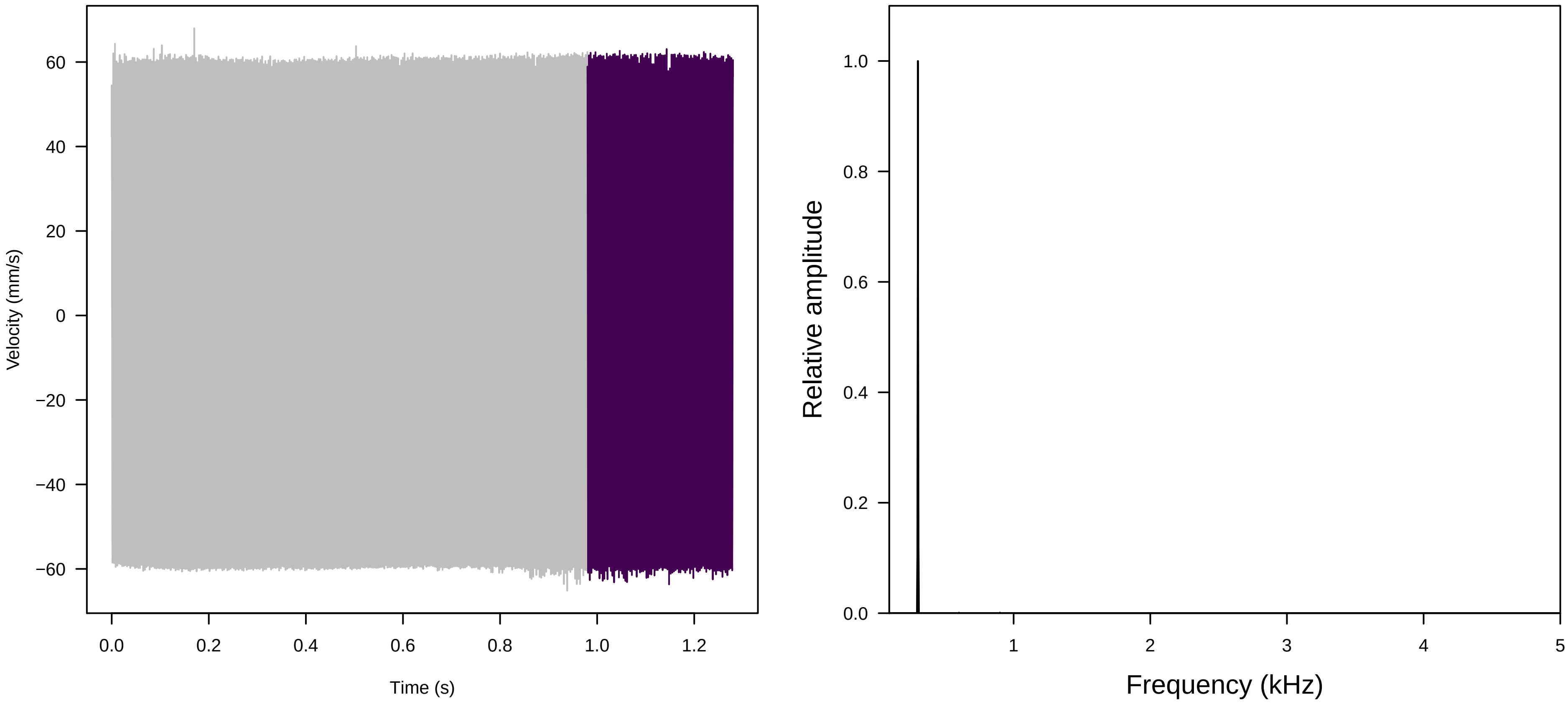

Vel. = 0.028 ; Str. = Receptacle ; Axis = y ; Fl. accession = 10−s−81−11

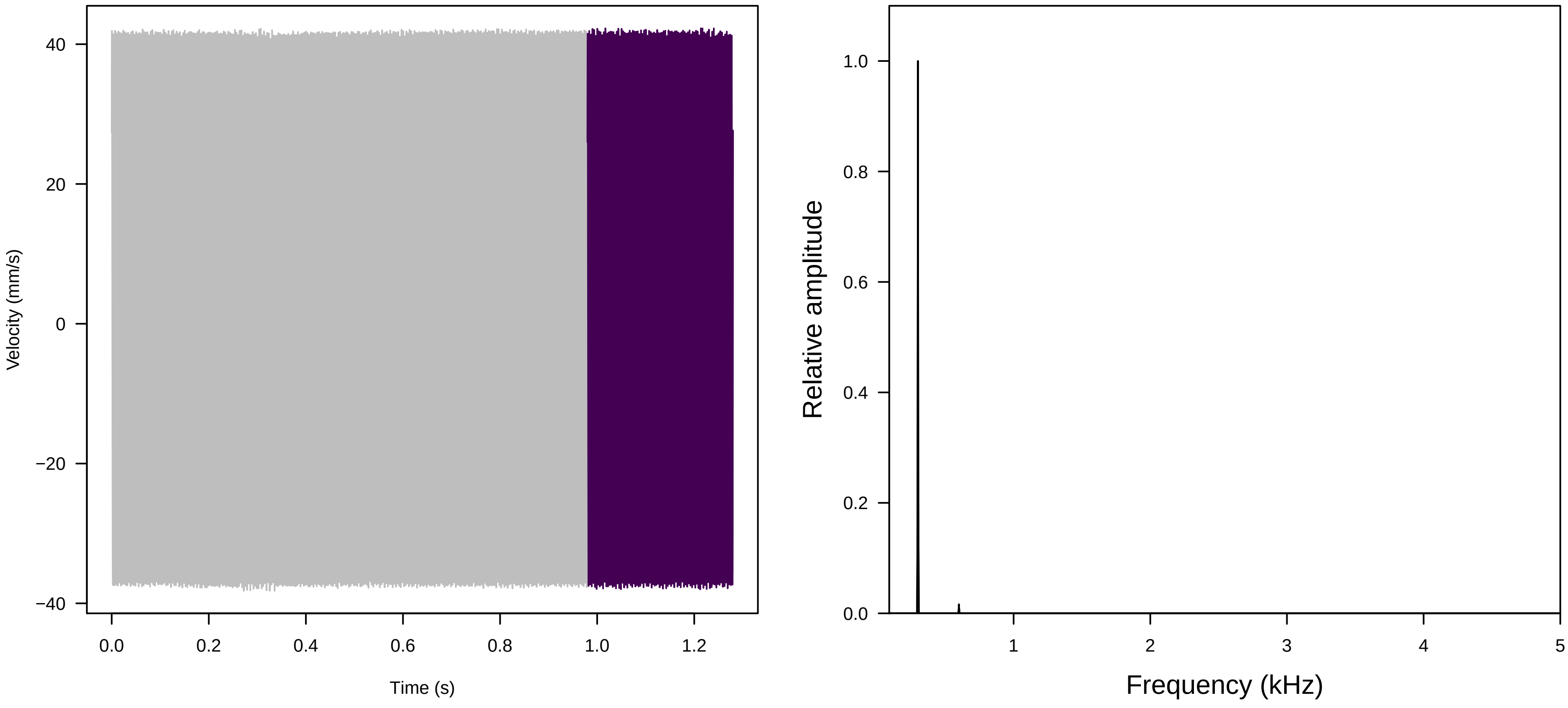

Vel. = 0.014 ; Str. = Corolla ; Axis = y ; Fl. accession = 10−s−81−11

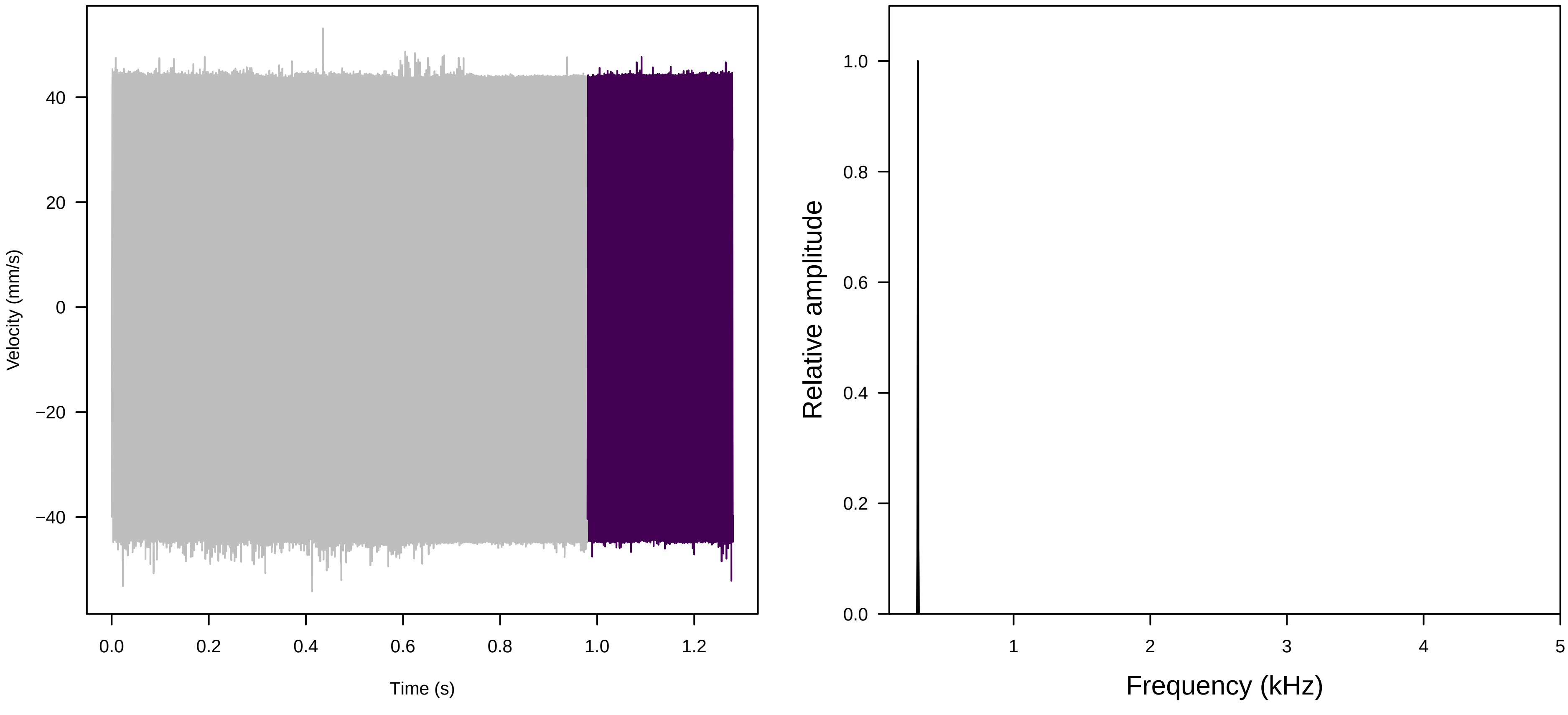

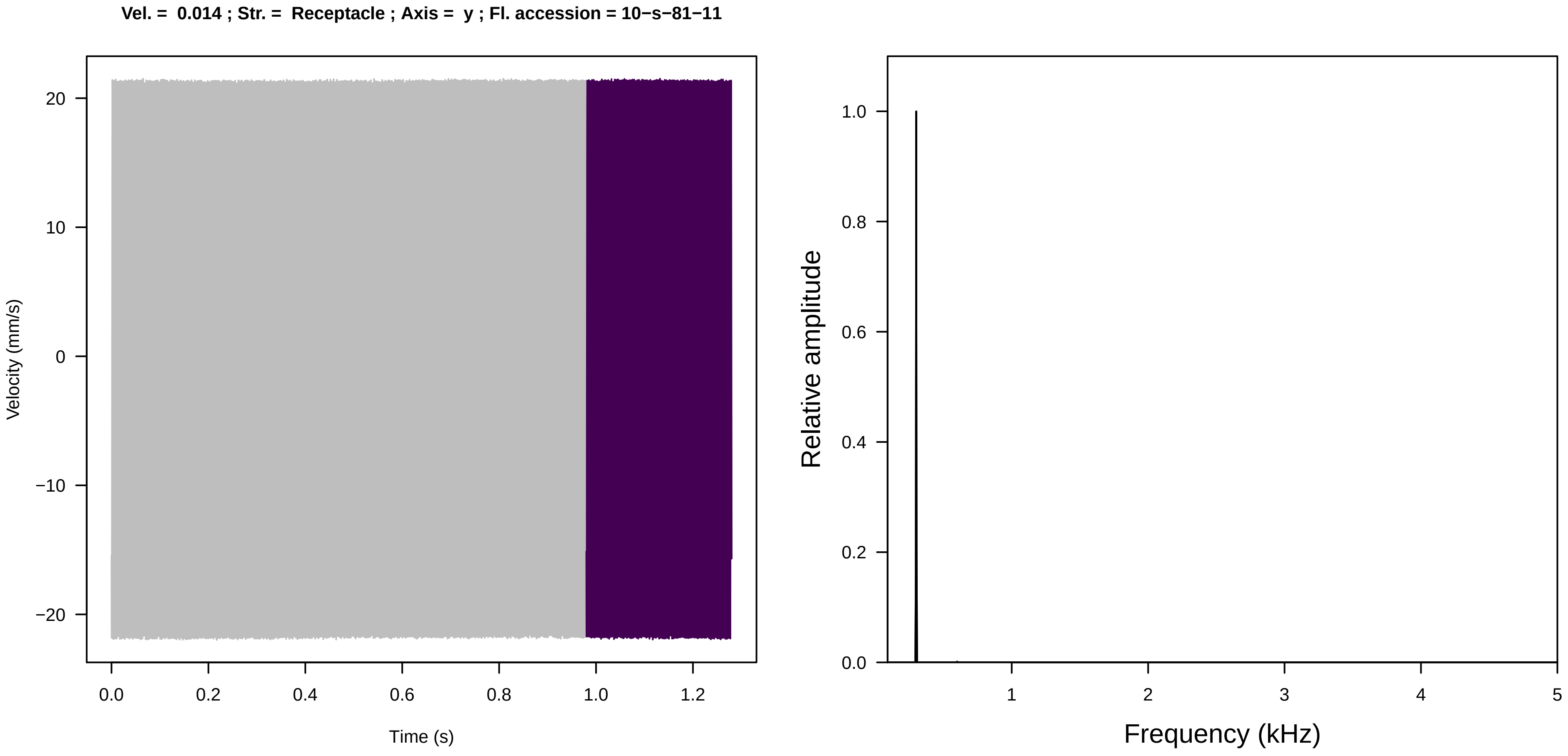
Vel. = 0.014 ; Str. = Receptacle ; Axis = y ; Fl. accession = 10−s−81−11
Velocity (mm/s)
Time (s)
Relative amplitude
Frequency (kHz)

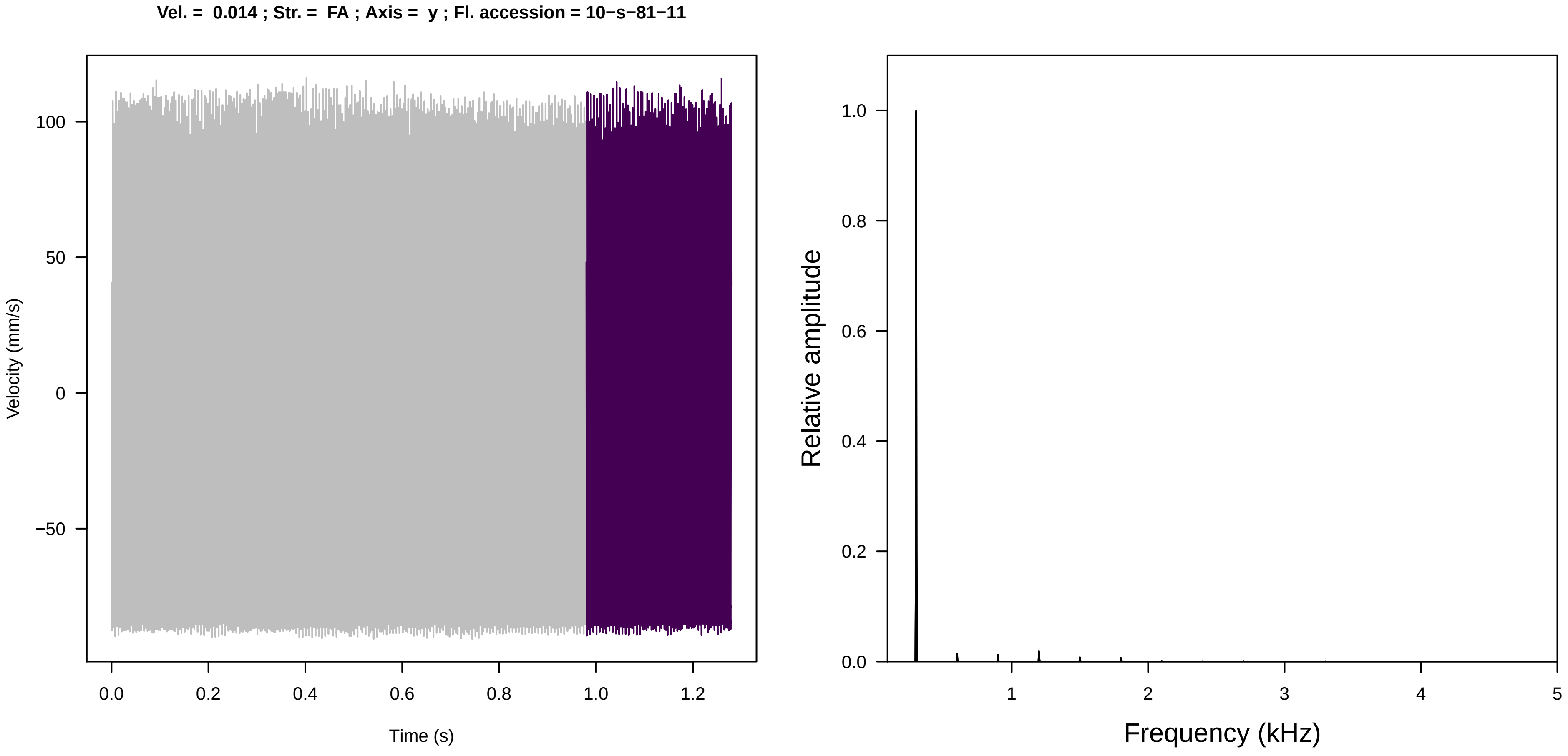
Vel. = 0.014 ; Str. = FA ; Axis = y ; Fl. accession = 10−s−81−11
Velocity (mm/s)
Time (s)
Relative amplitude
Frequency (kHz)

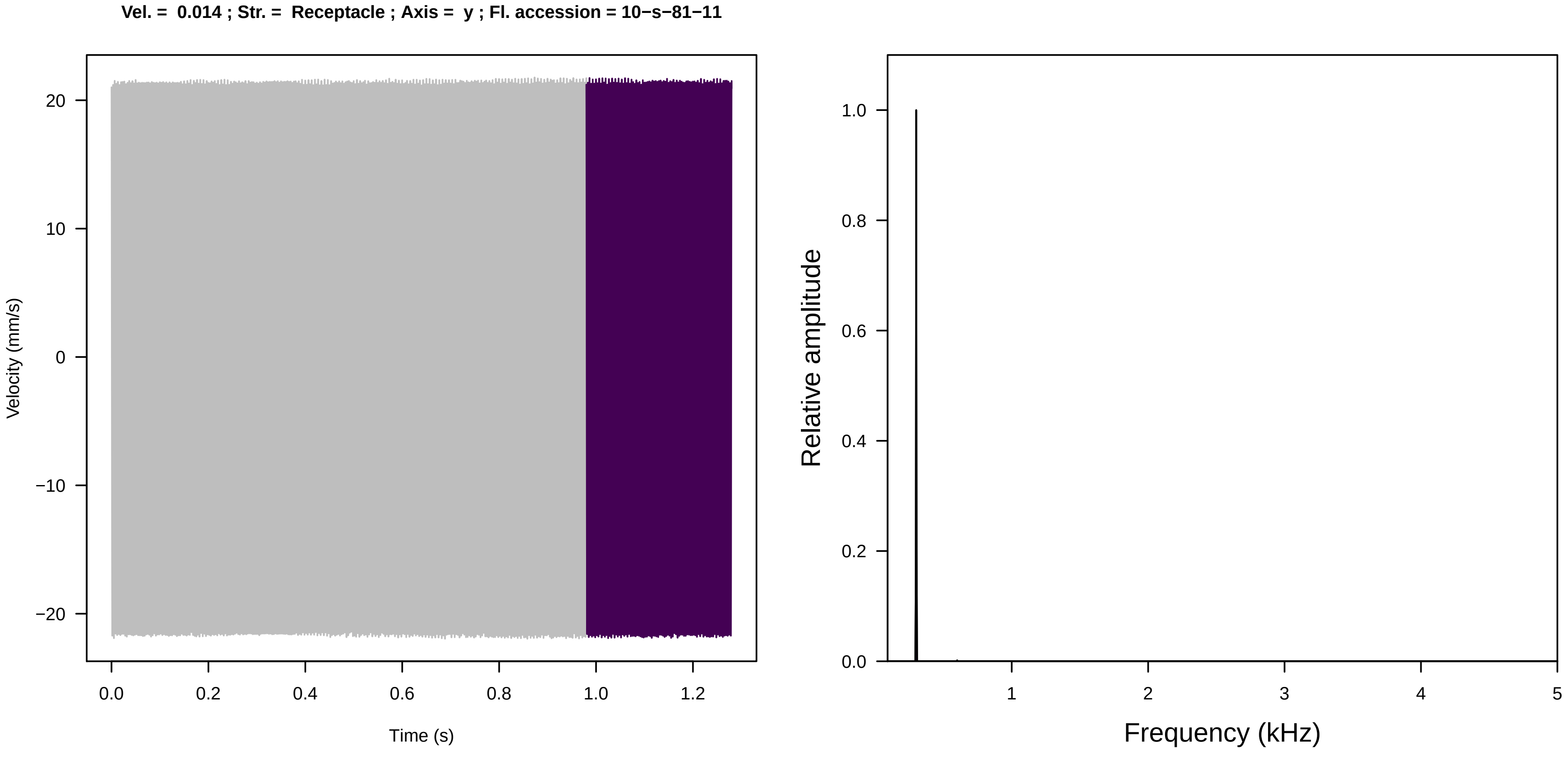
Vel. = 0.014 ; Str. = Receptacle ; Axis = y ; Fl. accession = 10−s−81−11
Velocity (mm/s)
Time (s)
Relative amplitude
Frequency (kHz)

Vel. = 0.014 ; Str. = PA ; Axis = y ; Fl. accession = 10−s−81−11

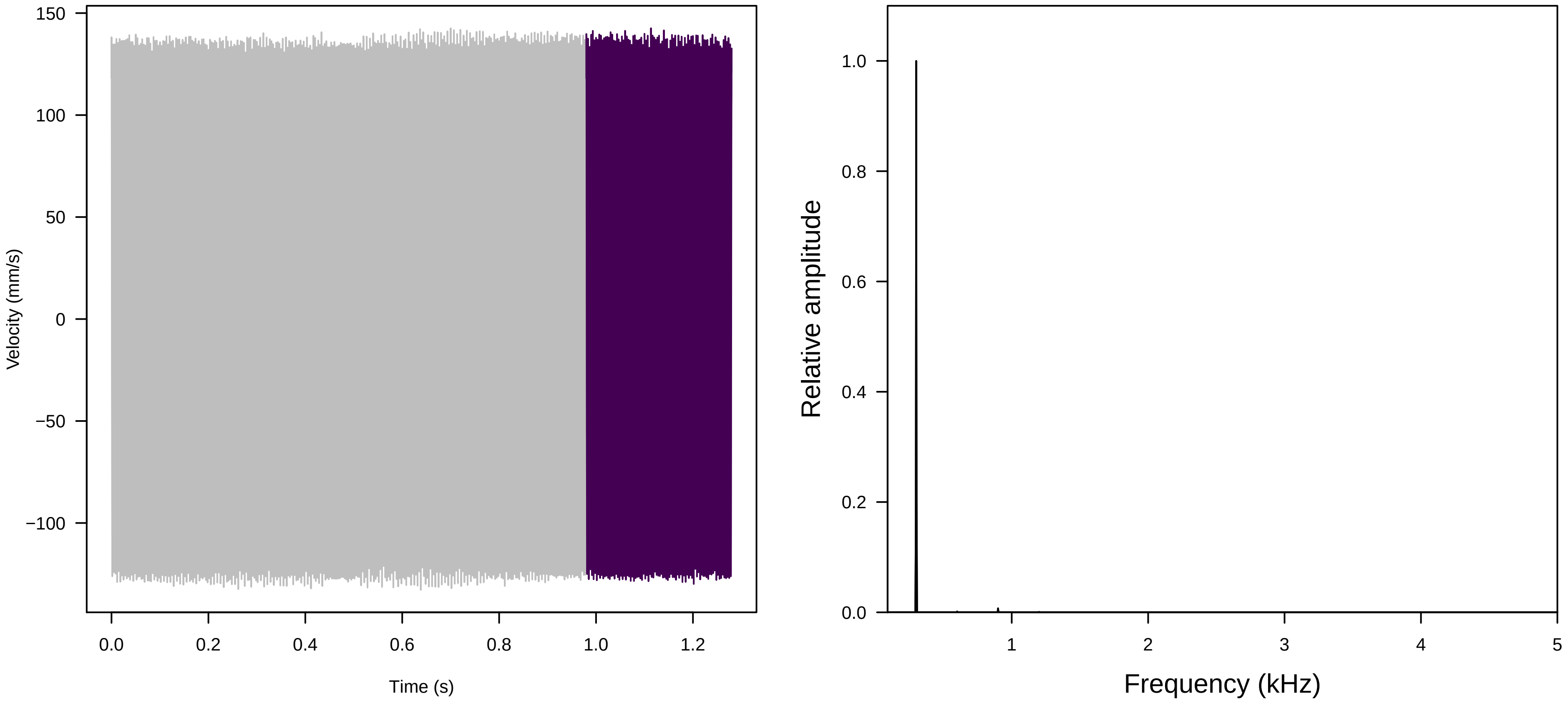

Vel. = 0.014 ; Str. = Receptacle ; Axis = y ; Fl. accession = 10−s−81−11

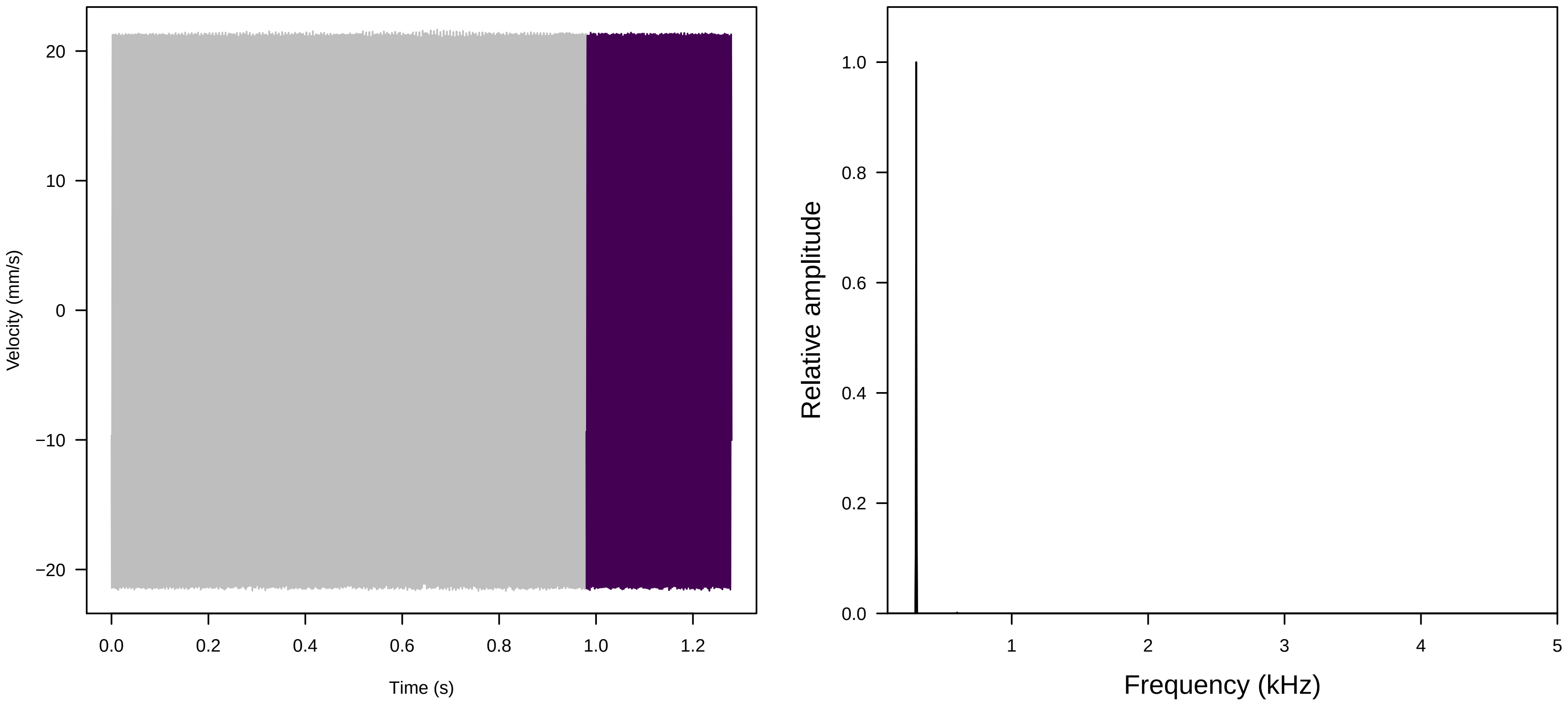

Vel. = 0.014 ; Str. = Corolla ; Axis = z ; Fl. accession = 10−s−81−11

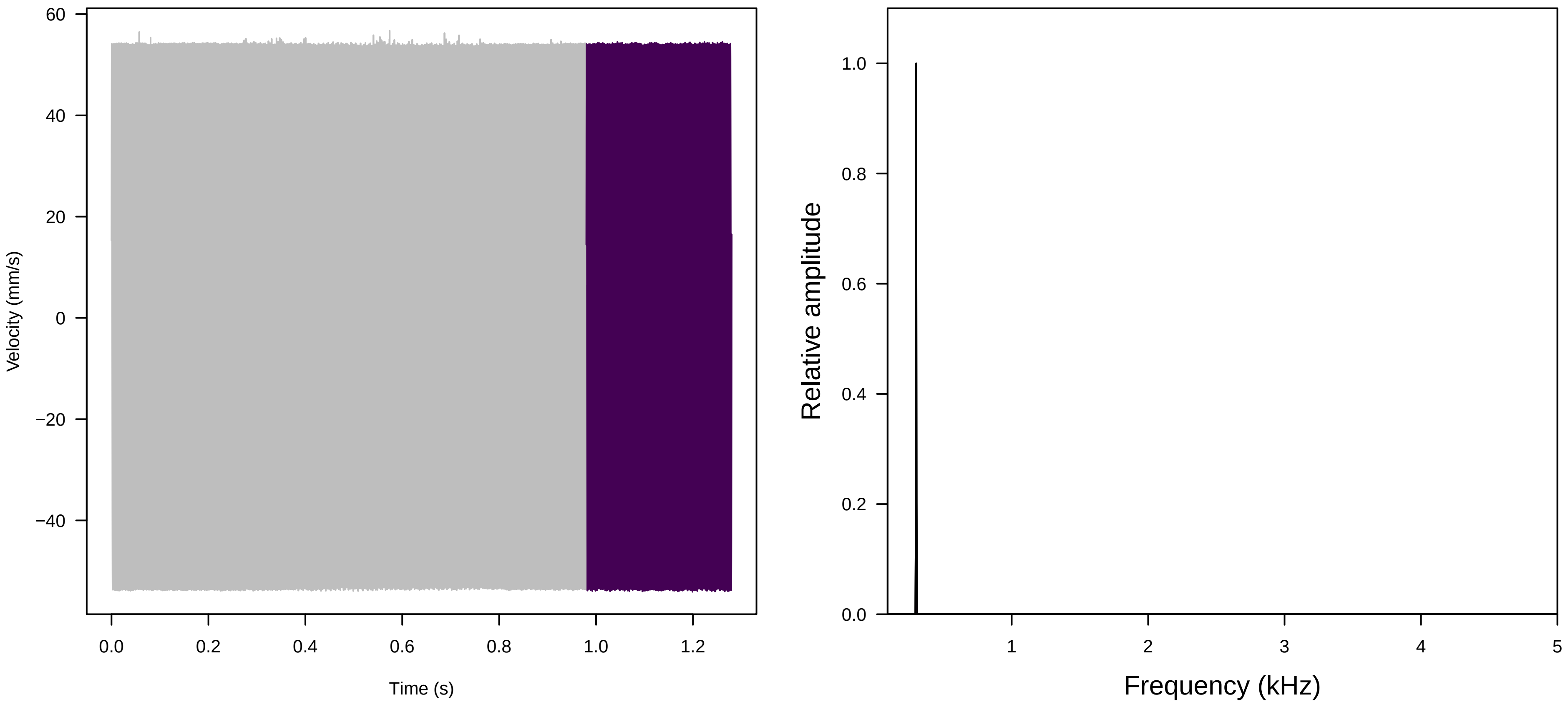

Vel. = 0.014 ; Str. = Receptacle ; Axis = z ; Fl. accession = 10−s−81−11

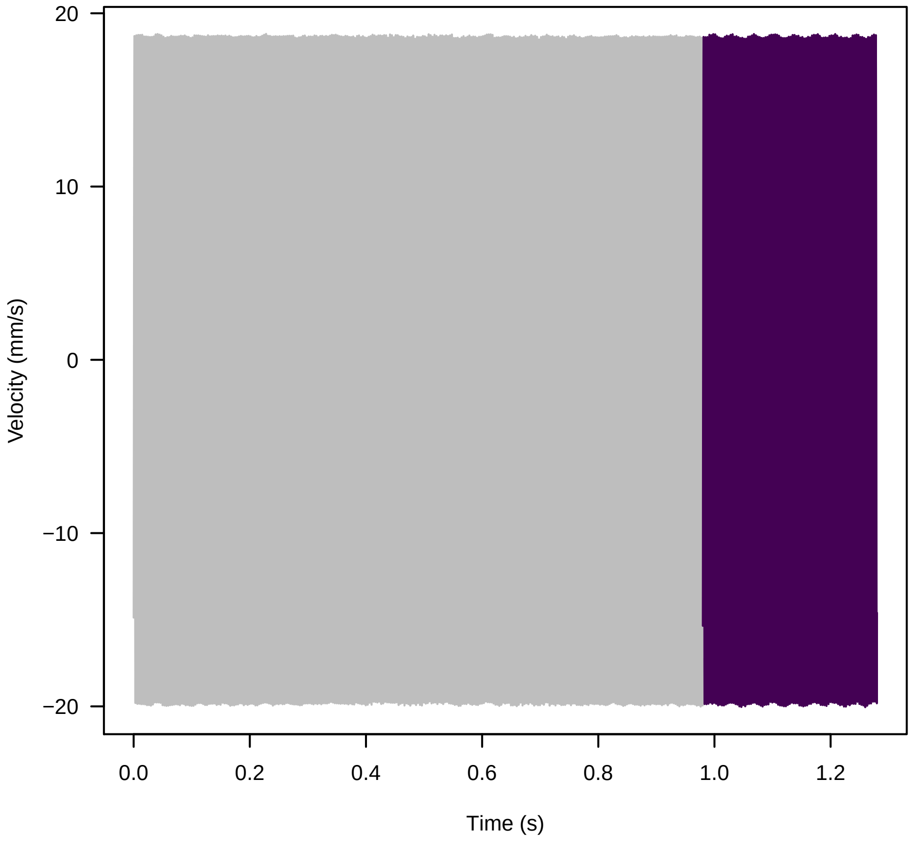

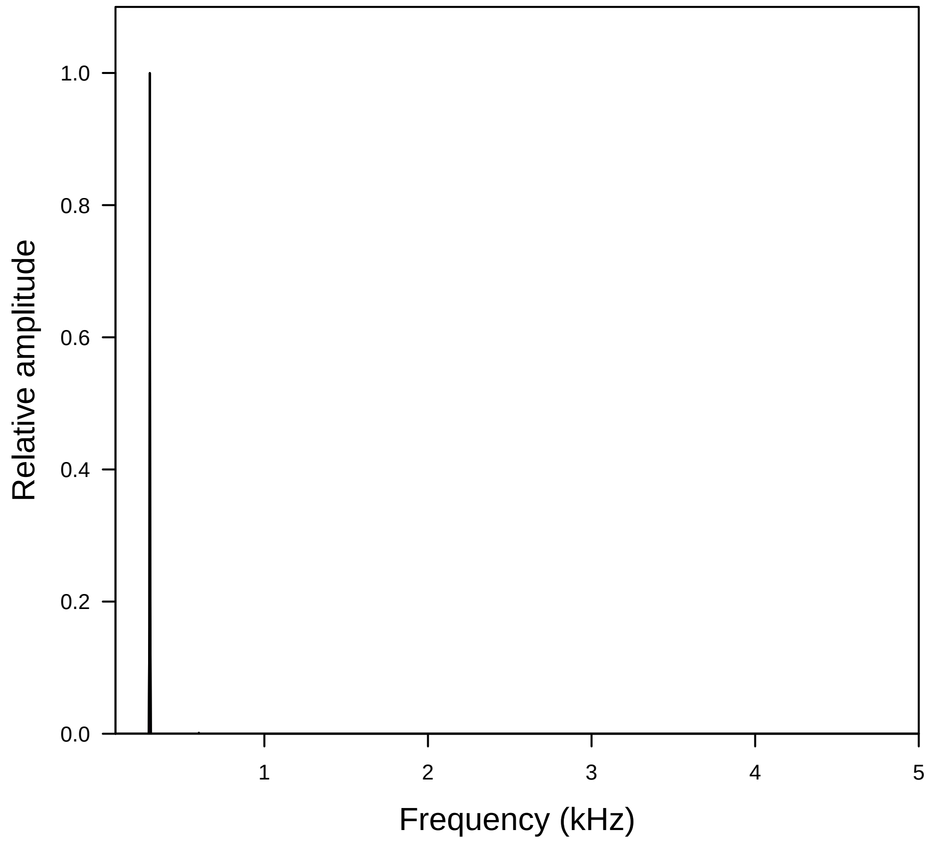

Vel. = 0.014 ; Str. = FA ; Axis = z ; Fl. accession = 10−s−81−11

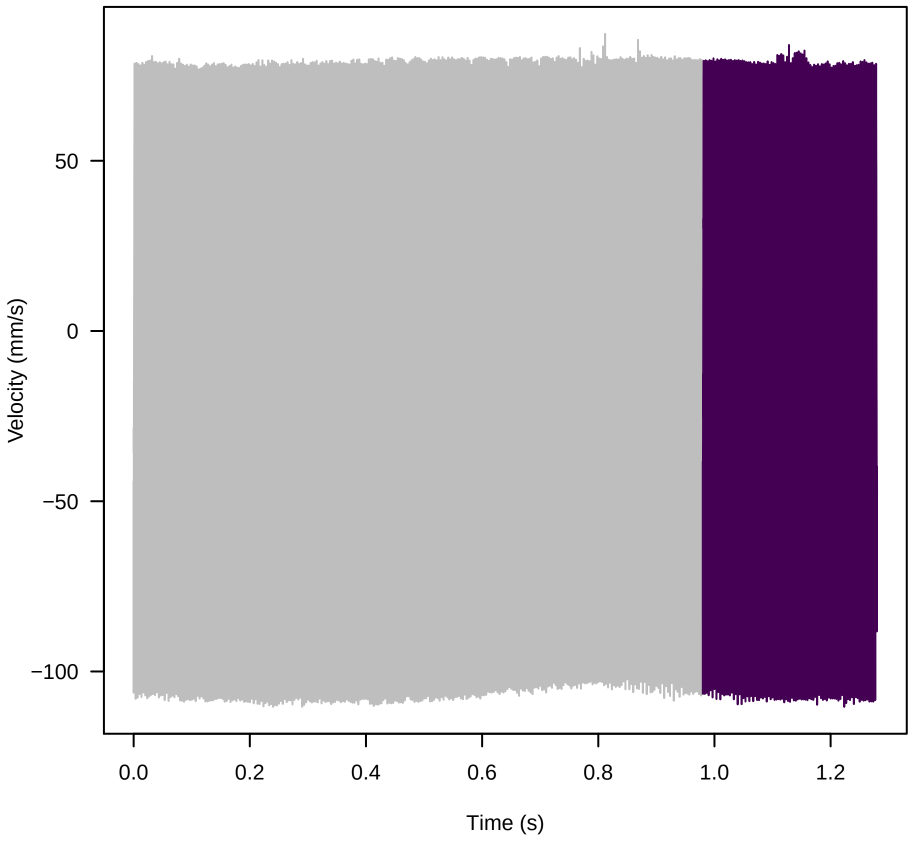

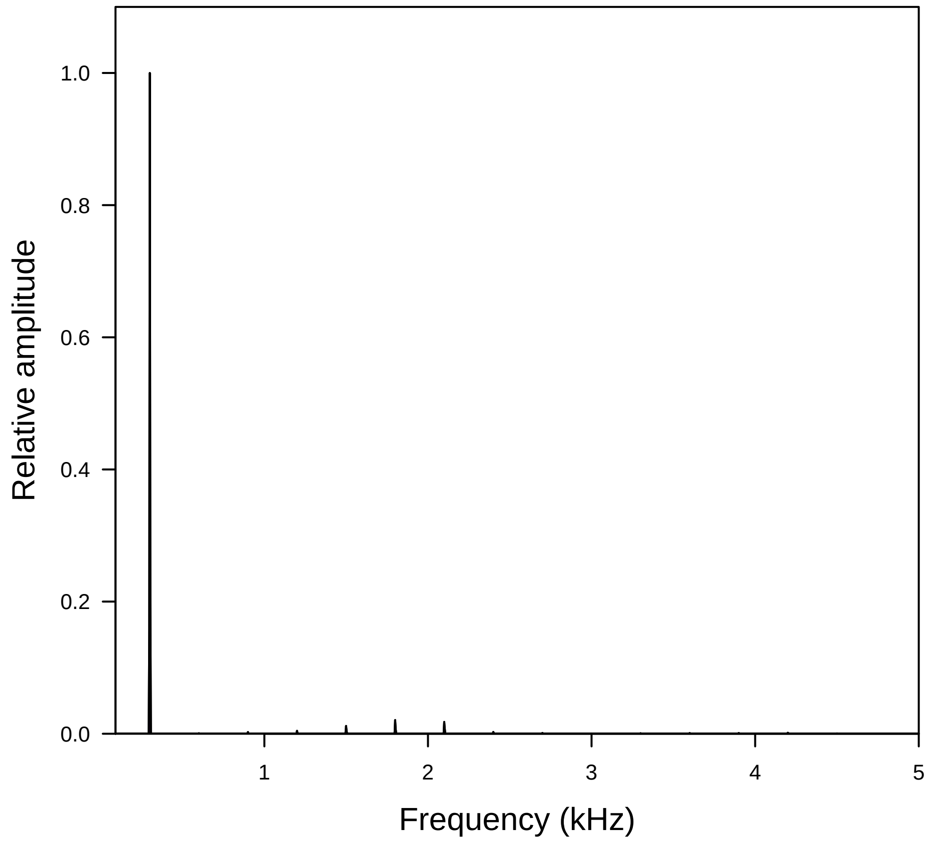

Vel. = 0.014 ; Str. = Receptacle ; Axis = z ; Fl. accession = 10−s−81−11

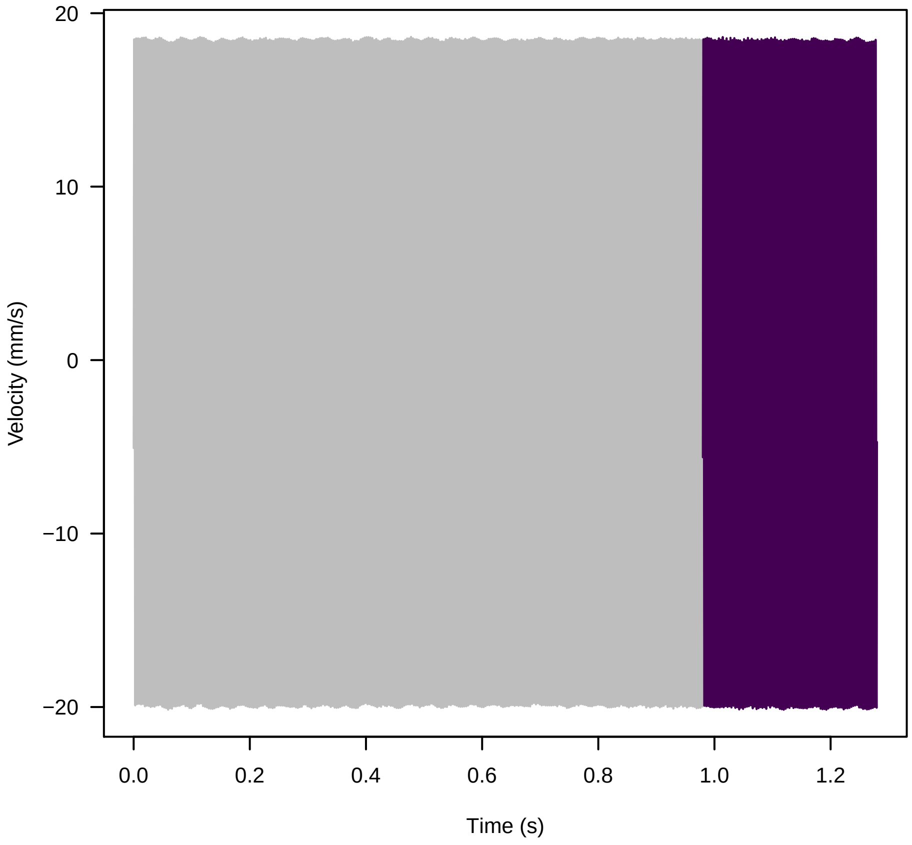

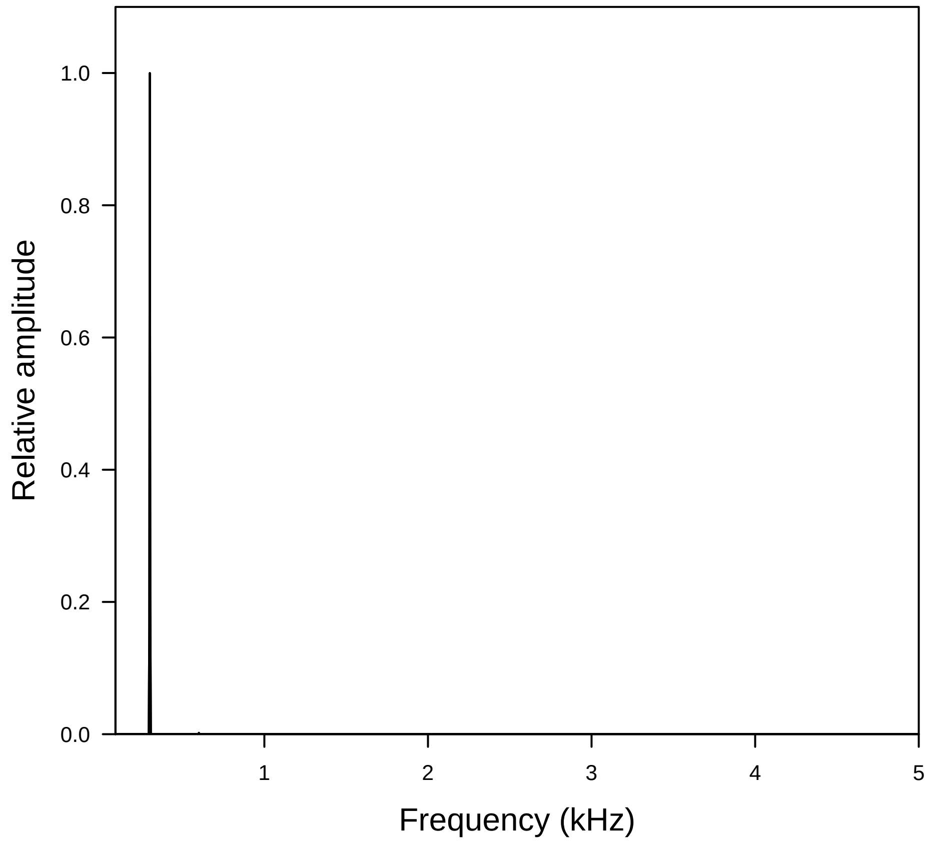

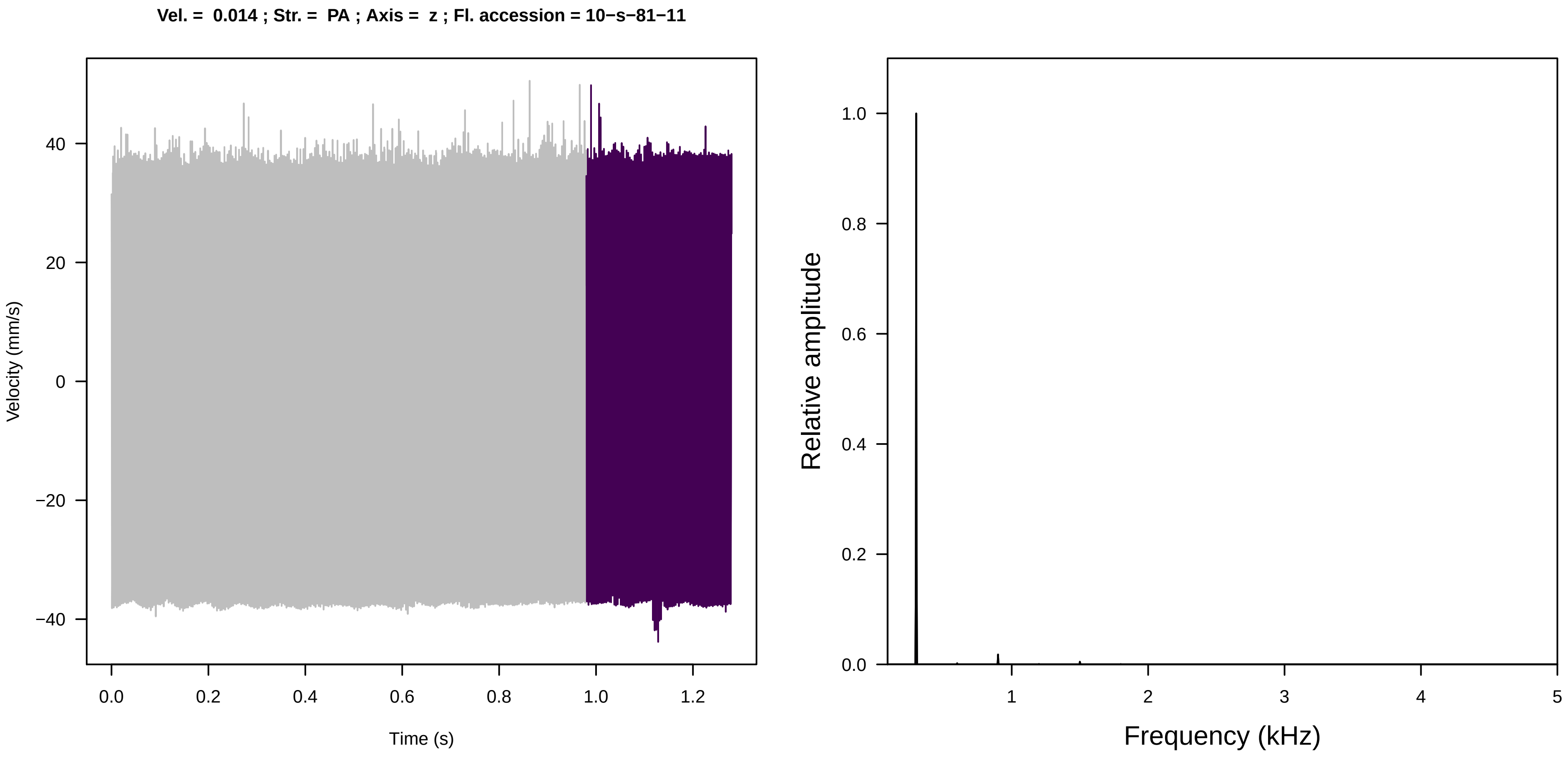
Vel. = 0.014 ; Str. = PA ; Axis = z ; Fl. accession = 10−s−81−11
Velocity (mm/s)
Time (s)
Relative amplitude
Frequency (kHz)

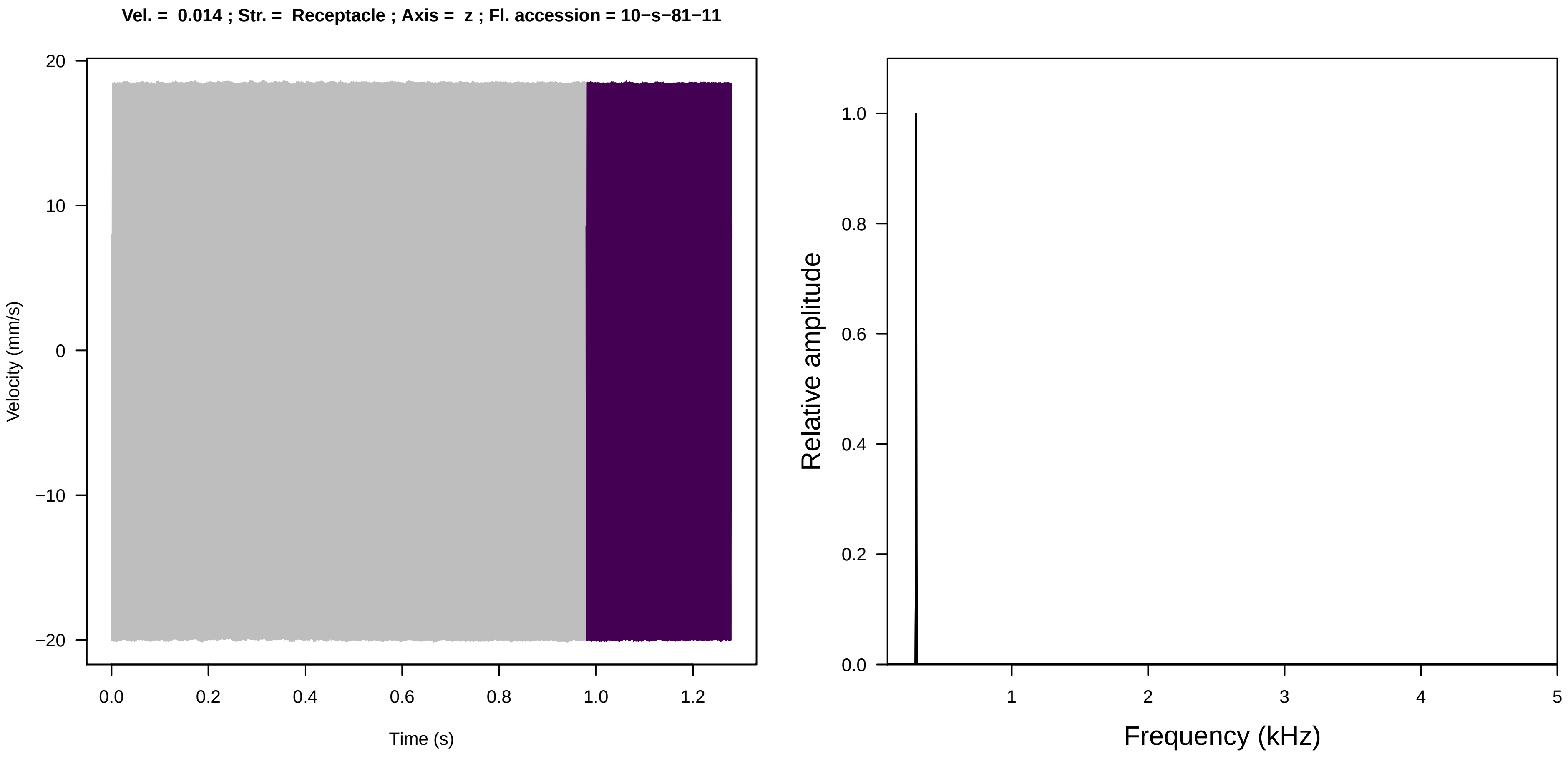
Vel. = 0.014 ; Str. = Receptacle ; Axis = z ; Fl. accession = 10−s−81−11
Velocity (mm/s)
Time (s)
Relative amplitude
Frequency (kHz)

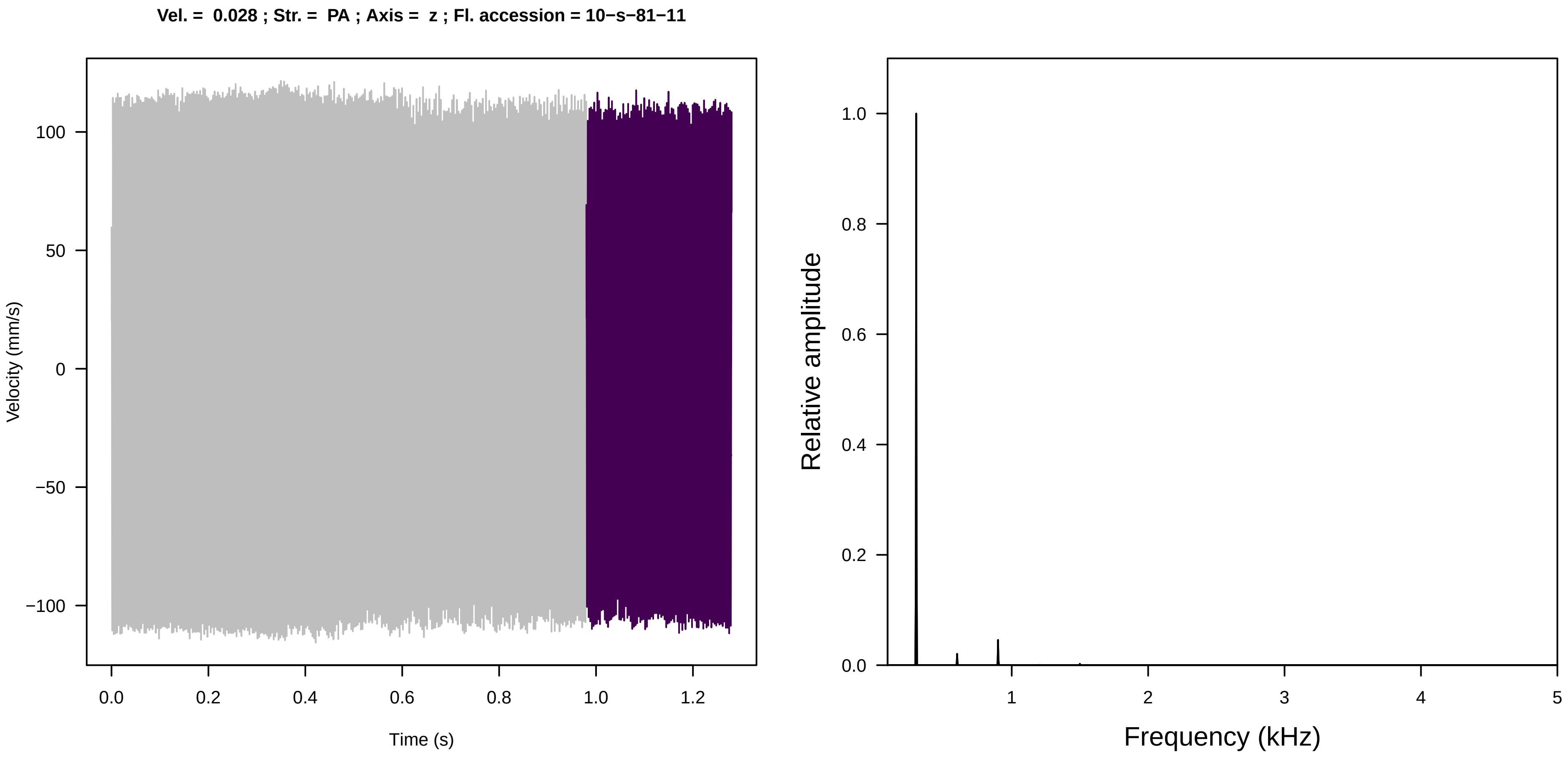
Vel. = 0.028 ; Str. = PA ; Axis = z ; Fl. accession = 10−s−81−11
Velocity (mm/s)
Time (s)
Relative amplitude
Frequency (kHz)

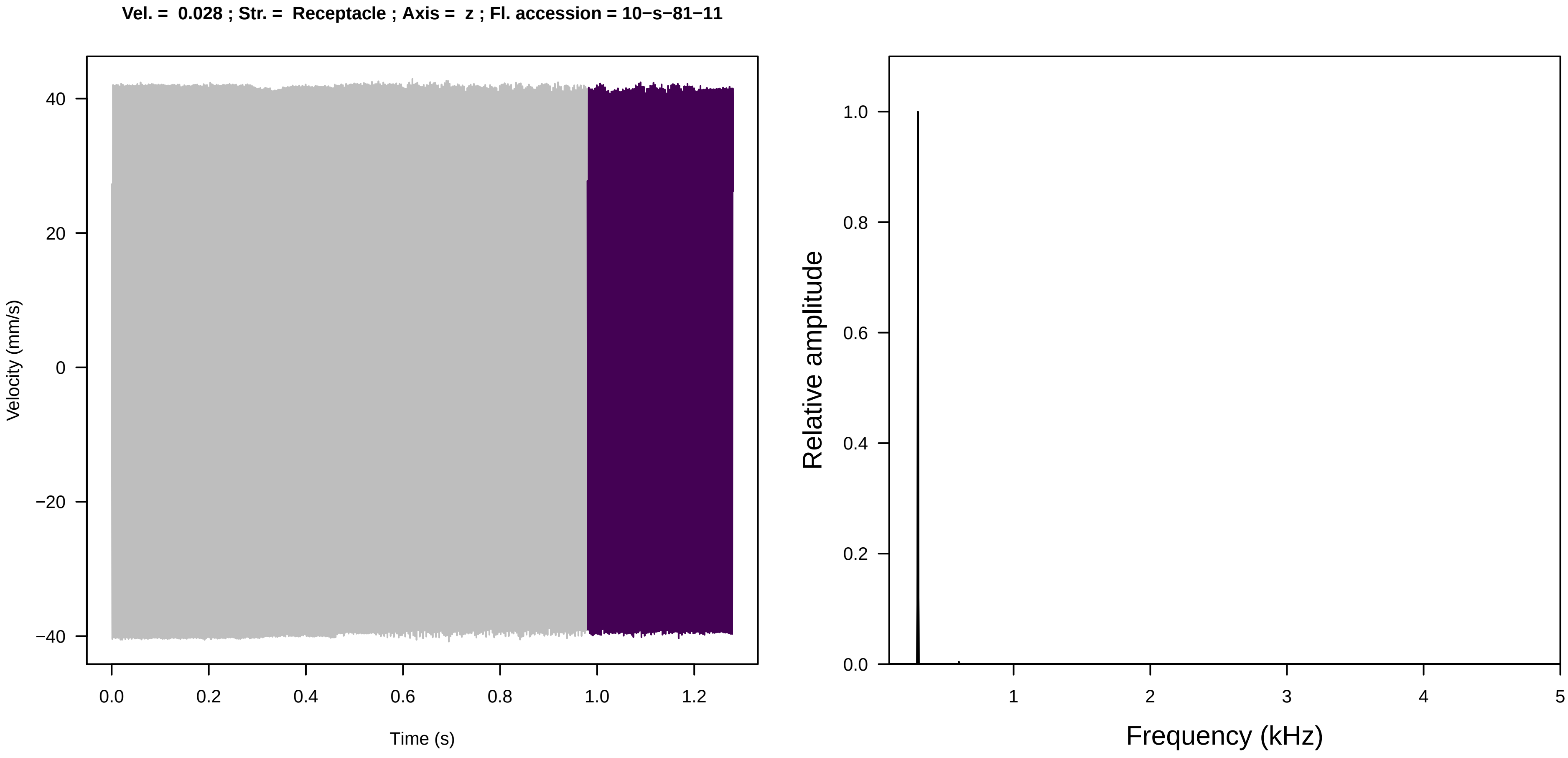
Vel. = 0.028 ; Str. = Receptacle ; Axis = z ; Fl. accession = 10−s−81−11
Velocity (mm/s)
Time (s)
Relative amplitude
Frequency (kHz)

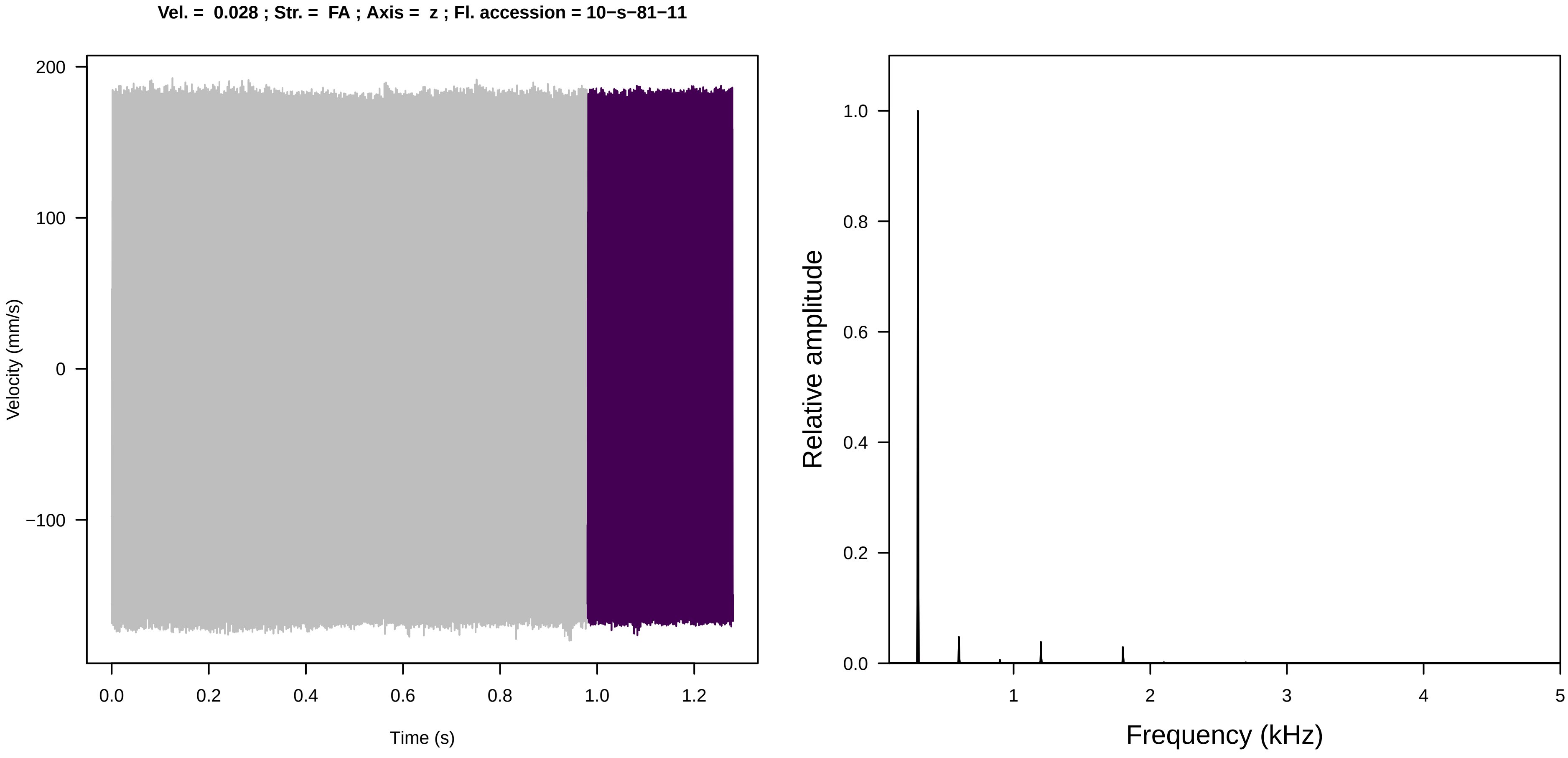
Vel. = 0.028 ; Str. = FA ; Axis = z ; Fl. accession = 10−s−81−11
Velocity (mm/s)
Time (s)
Relative amplitude
Frequency (kHz)

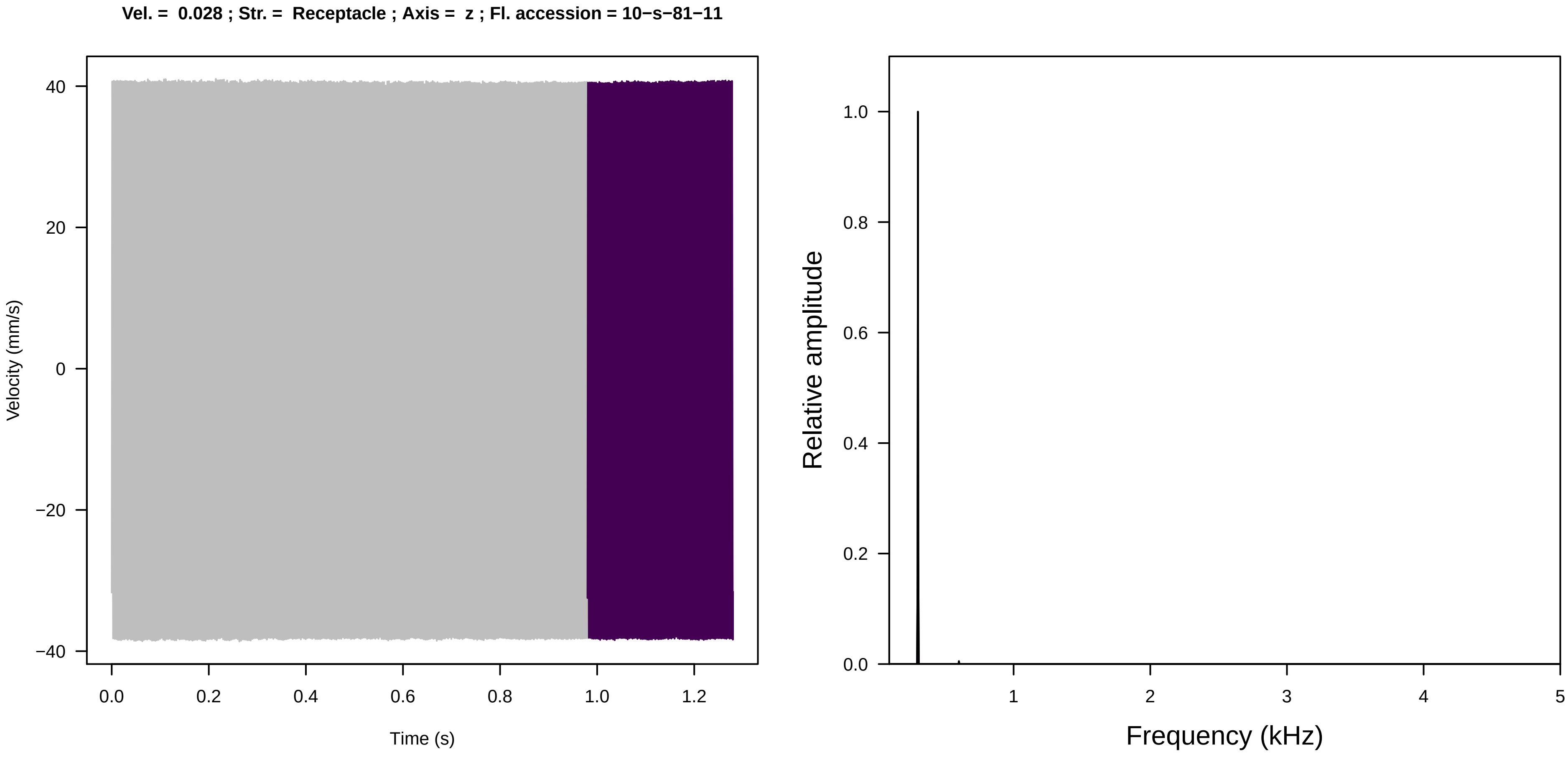
Vel. = 0.028 ; Str. = Receptacle ; Axis = z ; Fl. accession = 10−s−81−11
Velocity (mm/s)
Time (s)
Relative amplitude
Frequency (kHz)

Vel. = 0.028 ; Str. = Corolla ; Axis = z ; Fl. accession = 10−s−81−11

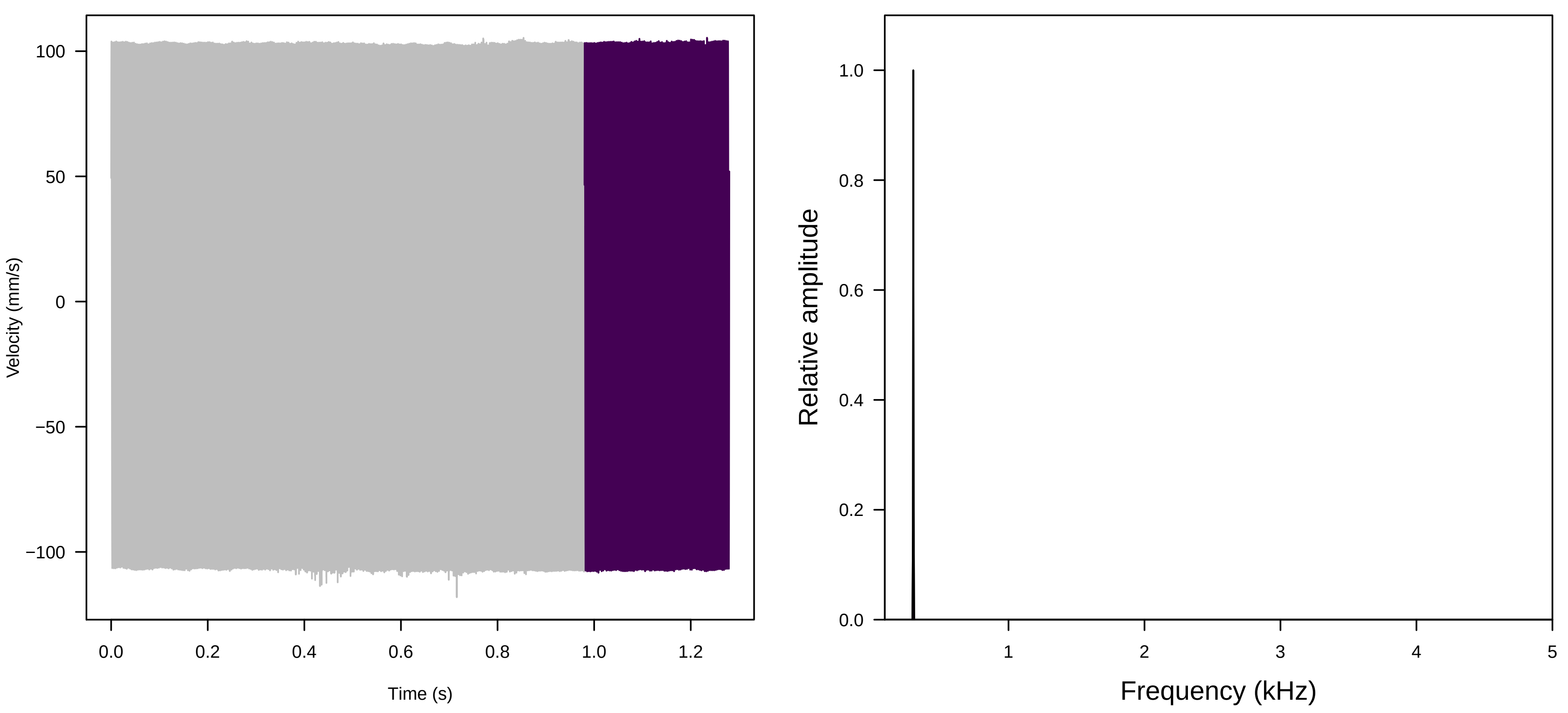

Vel. = 0.028 ; Str. = Receptacle ; Axis = z ; Fl. accession = 10−s−81−11

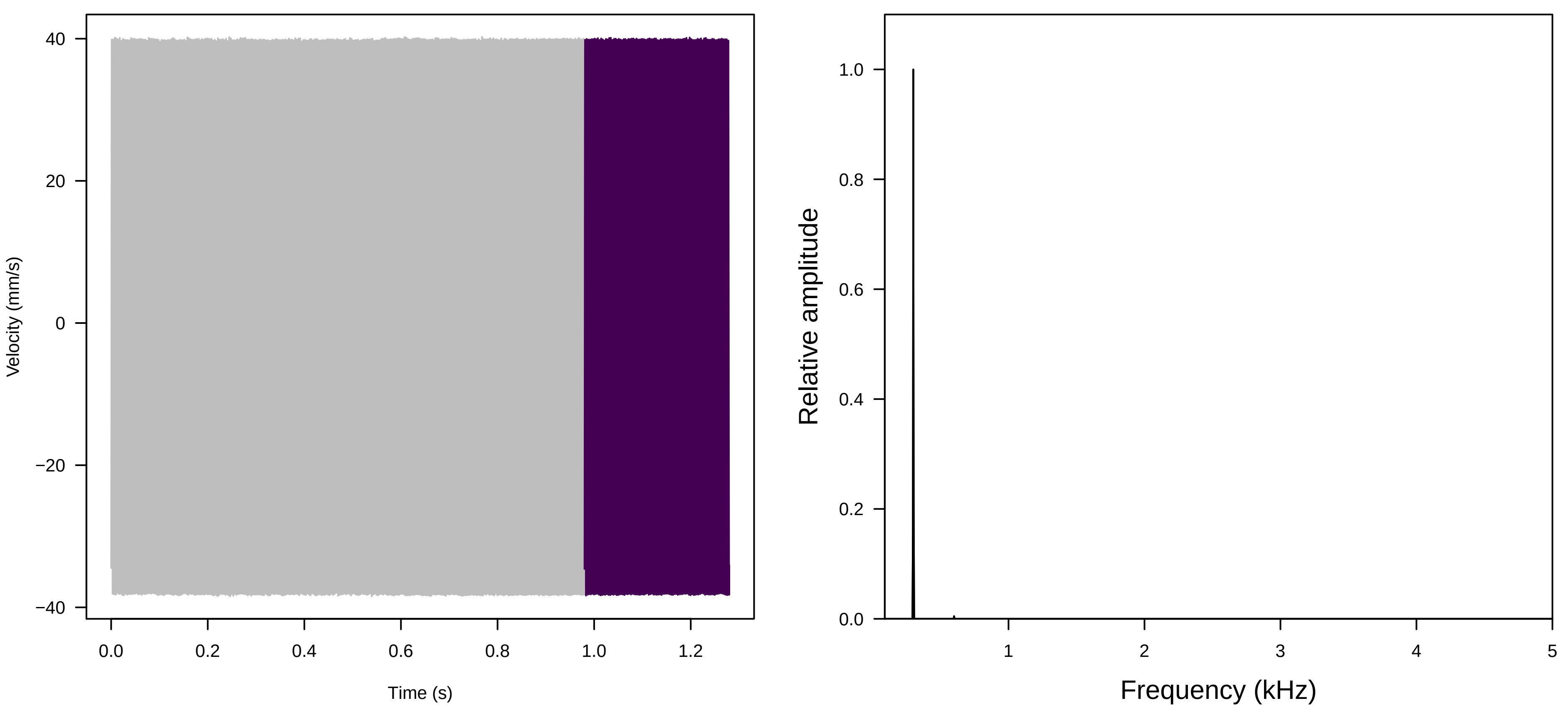

Vel. = 0.057 ; Str. = Corolla ; Axis = z ; Fl. accession = 10−s−81−11

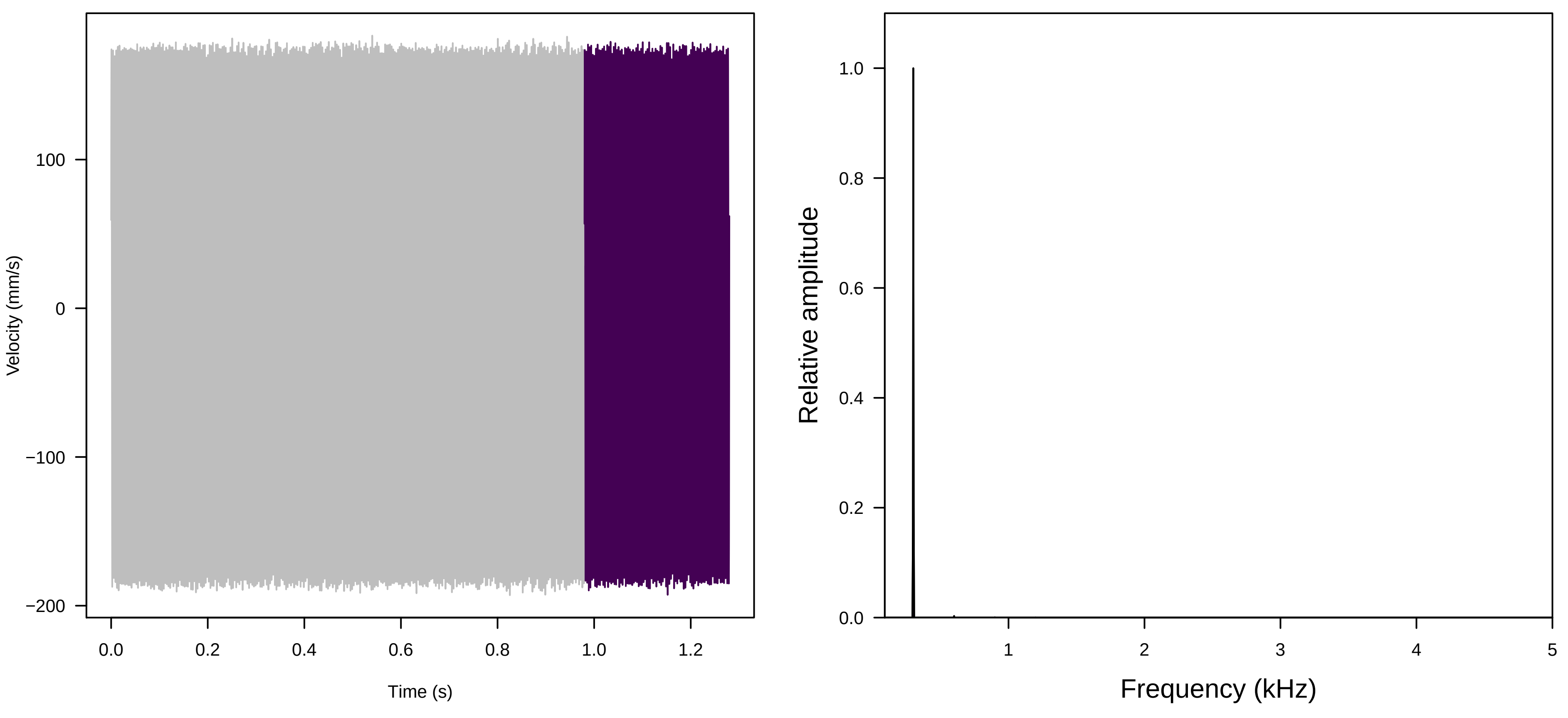

Vel. = 0.057 ; Str. = Receptacle ; Axis = z ; Fl. accession = 10−s−81−11

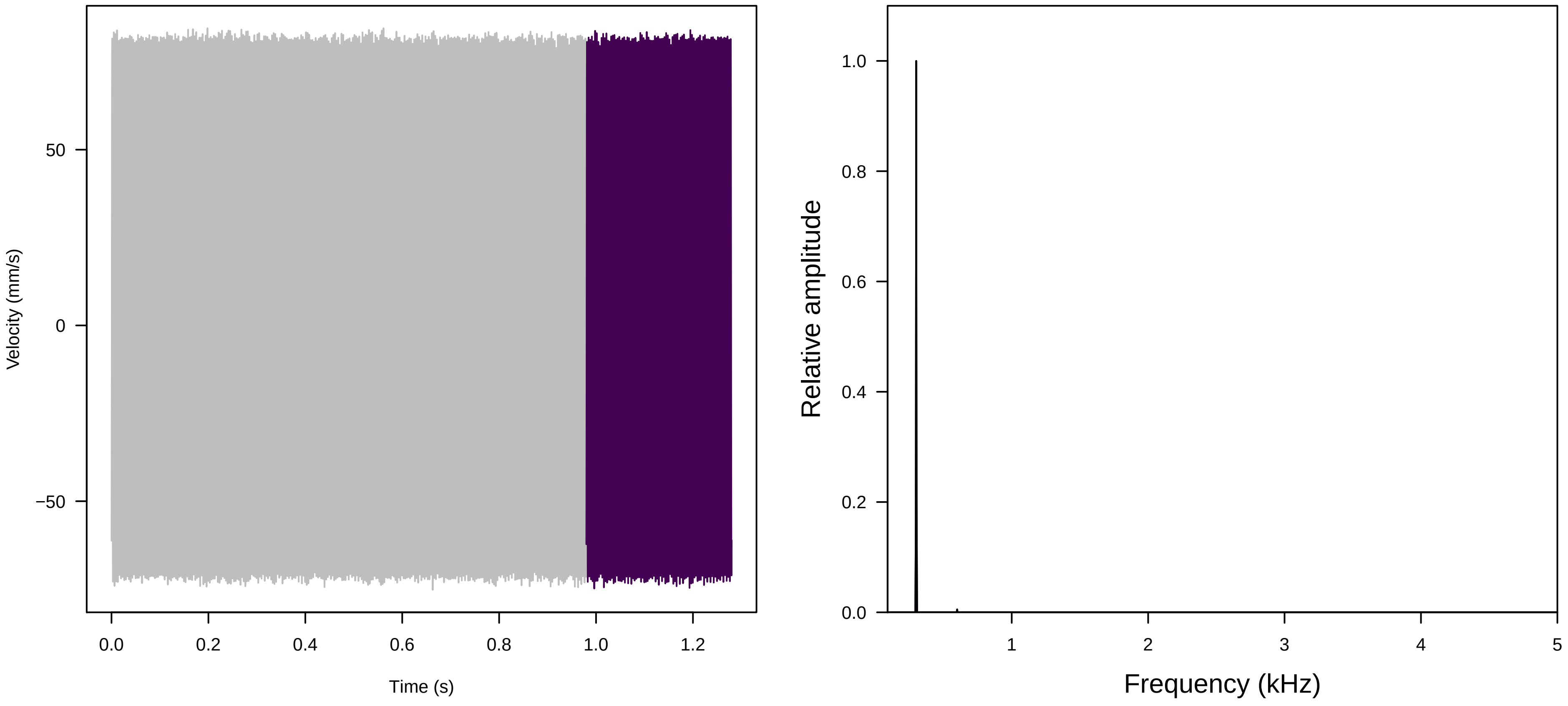

Vel. = 0.057 ; Str. = FA ; Axis = z ; Fl. accession = 10−s−81−11

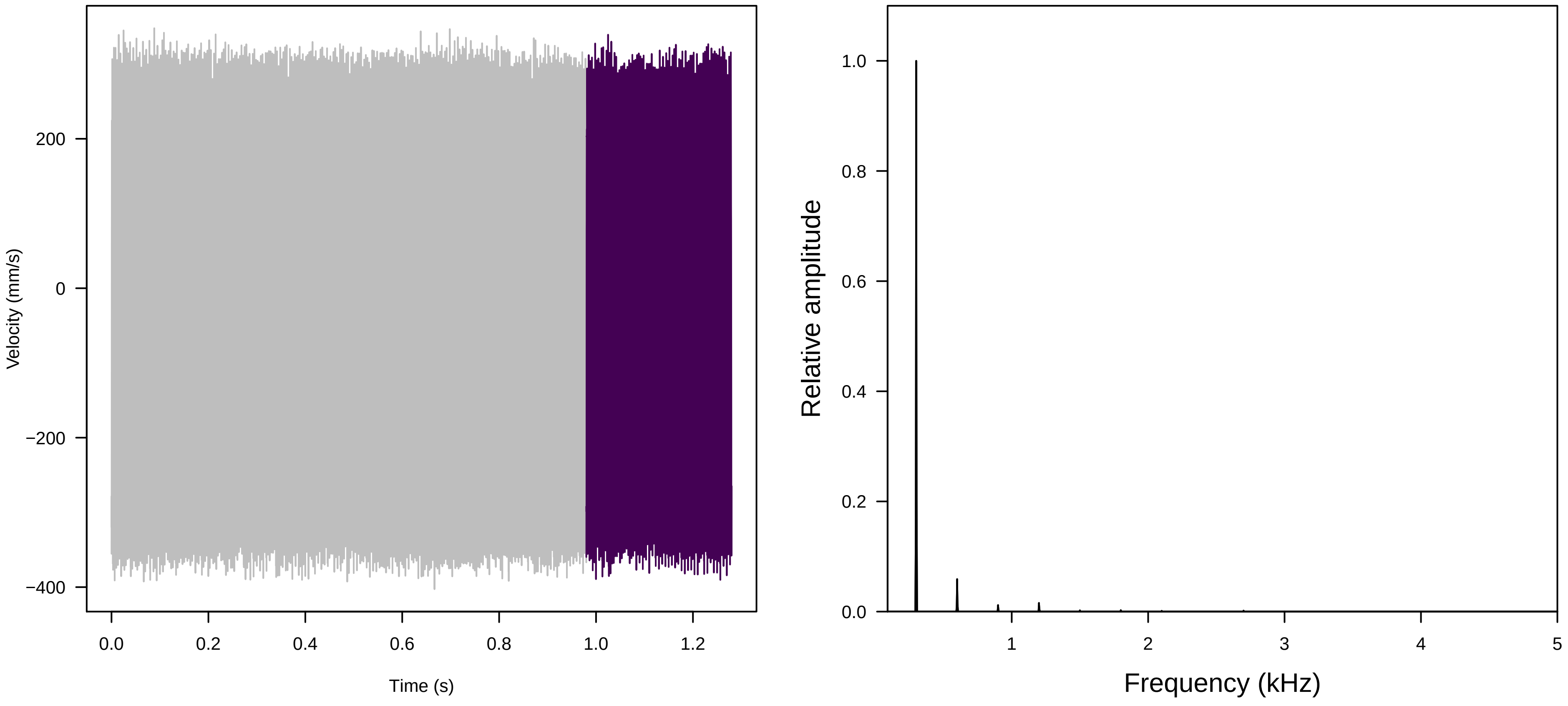

Vel. = 0.057 ; Str. = Receptacle ; Axis = z ; Fl. accession = 10−s−81−11

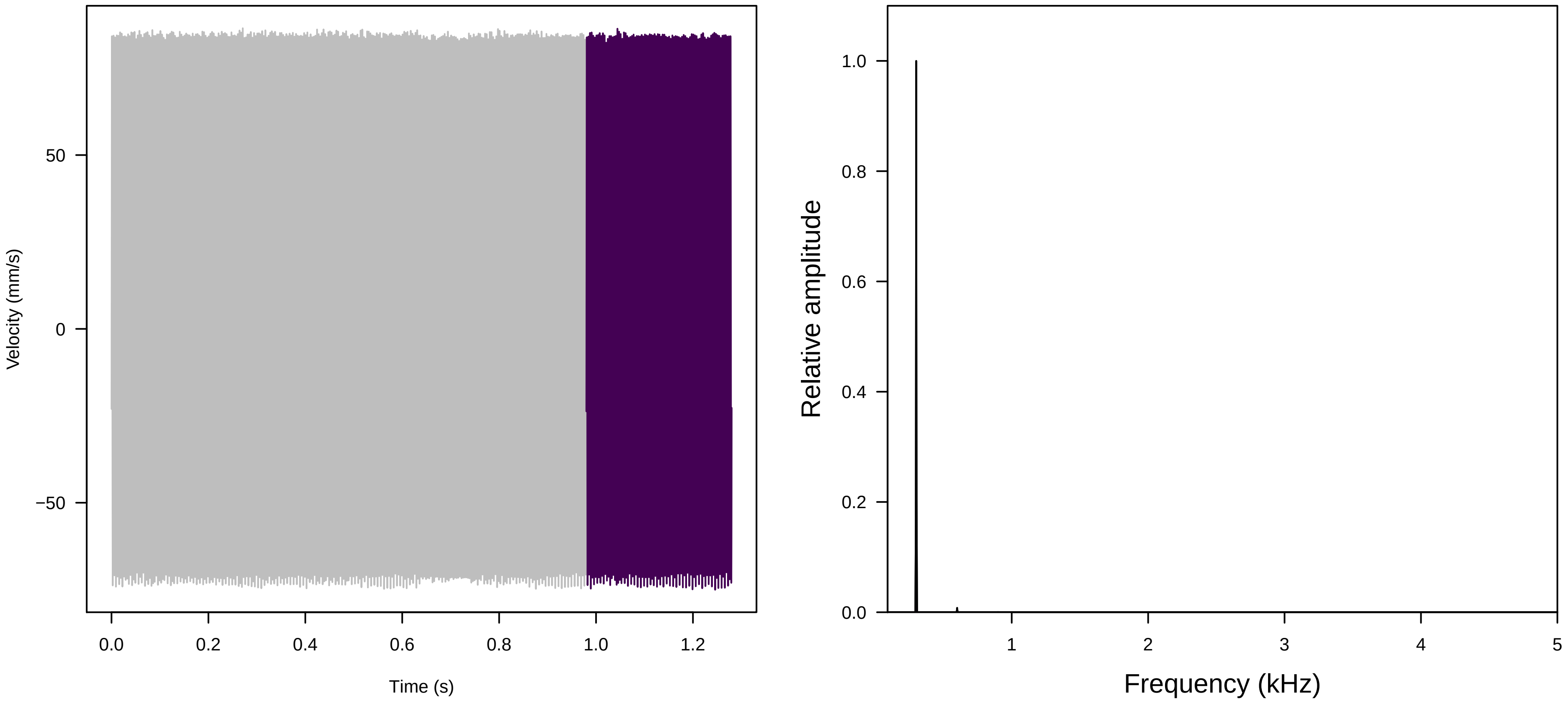

Vel. = 0.057 ; Str. = PA ; Axis = z ; Fl. accession = 10−s−81−11

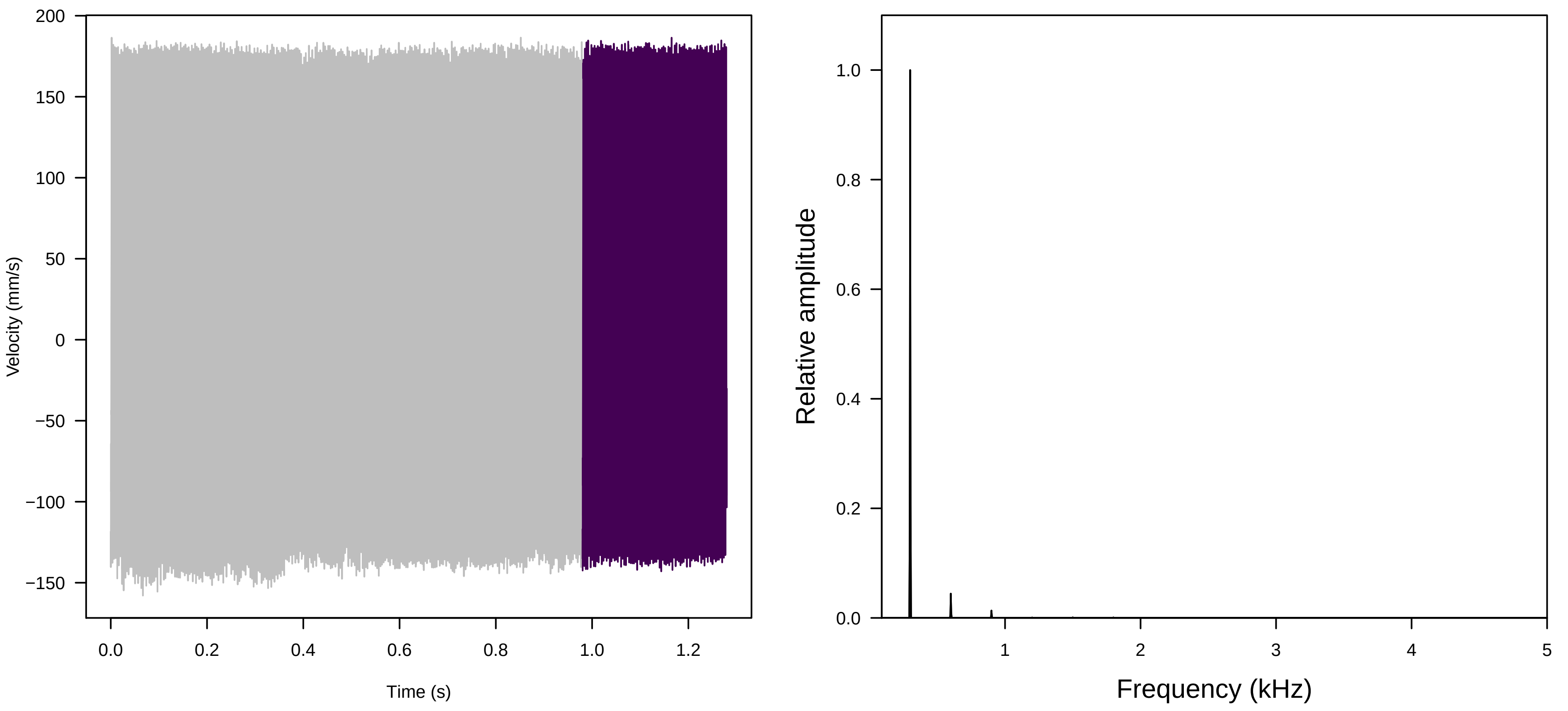

Vel. = 0.057 ; Str. = Receptacle ; Axis = z ; Fl. accession = 10−s−81−11

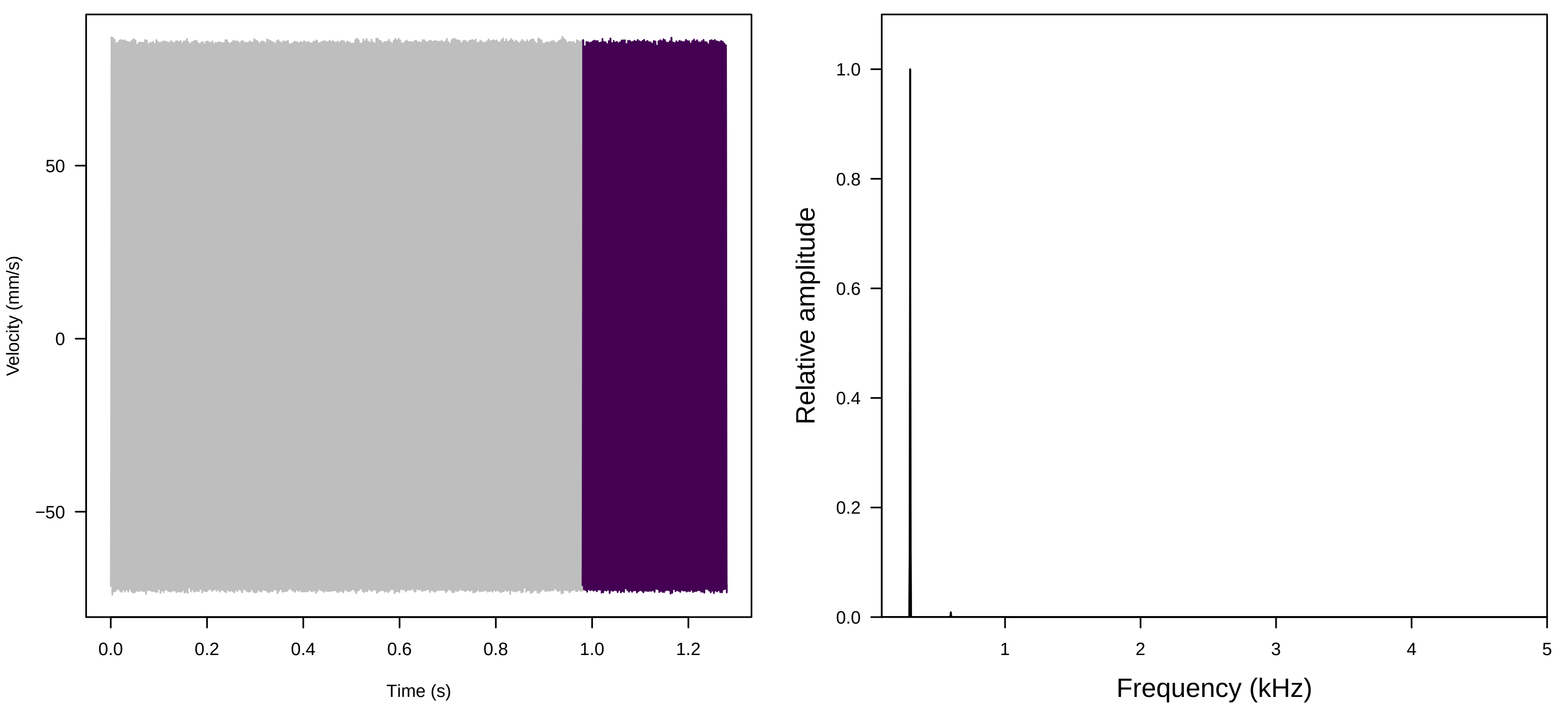

Vel. = 0.014 ; Str. = Corolla ; Axis = x ; Fl. accession = 10−s−81−11

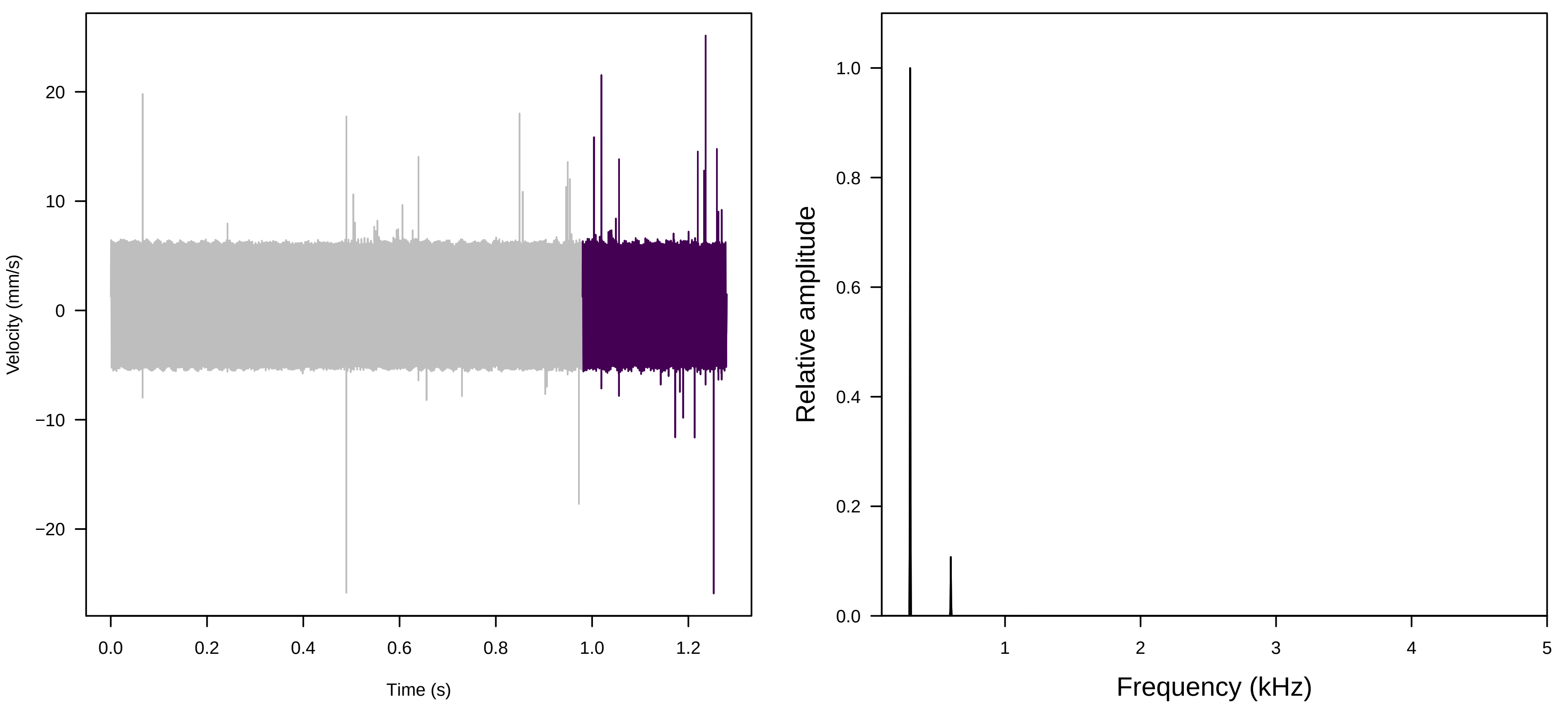

Vel. = 0.014 ; Str. = Receptacle ; Axis = x ; Fl. accession = 10−s−81−11

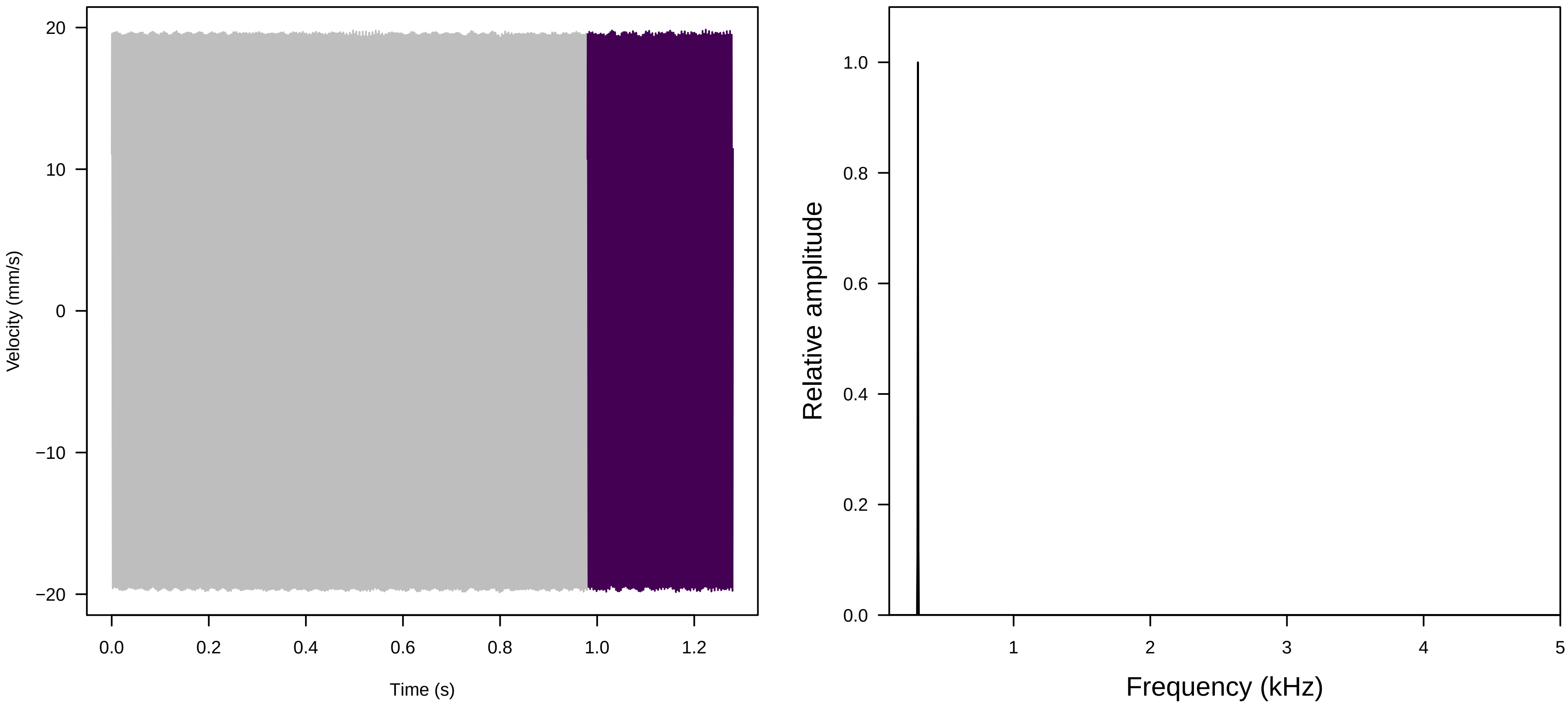

Vel. = 0.014 ; Str. = FA ; Axis = x ; Fl. accession = 10−s−81−11

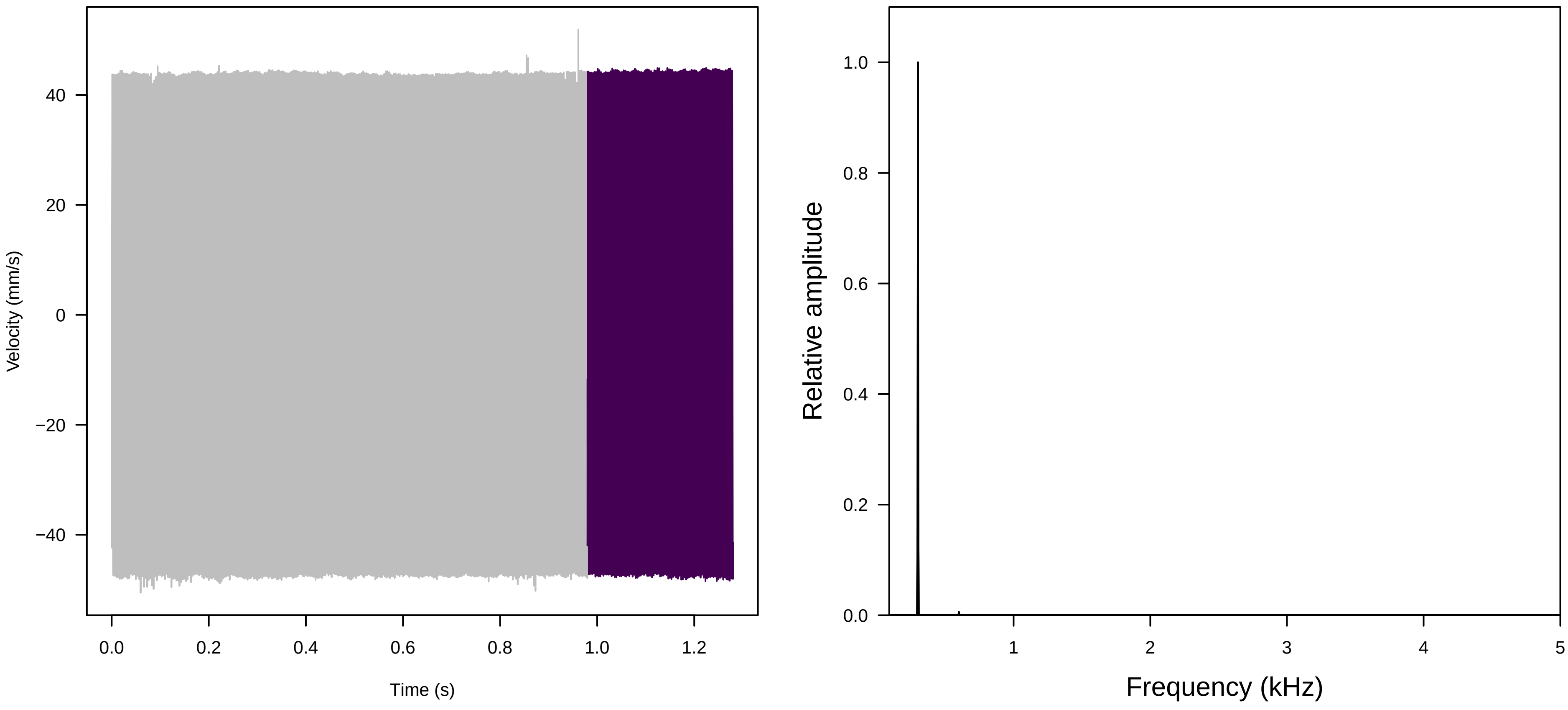

Vel. = 0.014 ; Str. = Receptacle ; Axis = x ; Fl. accession = 10−s−81−11

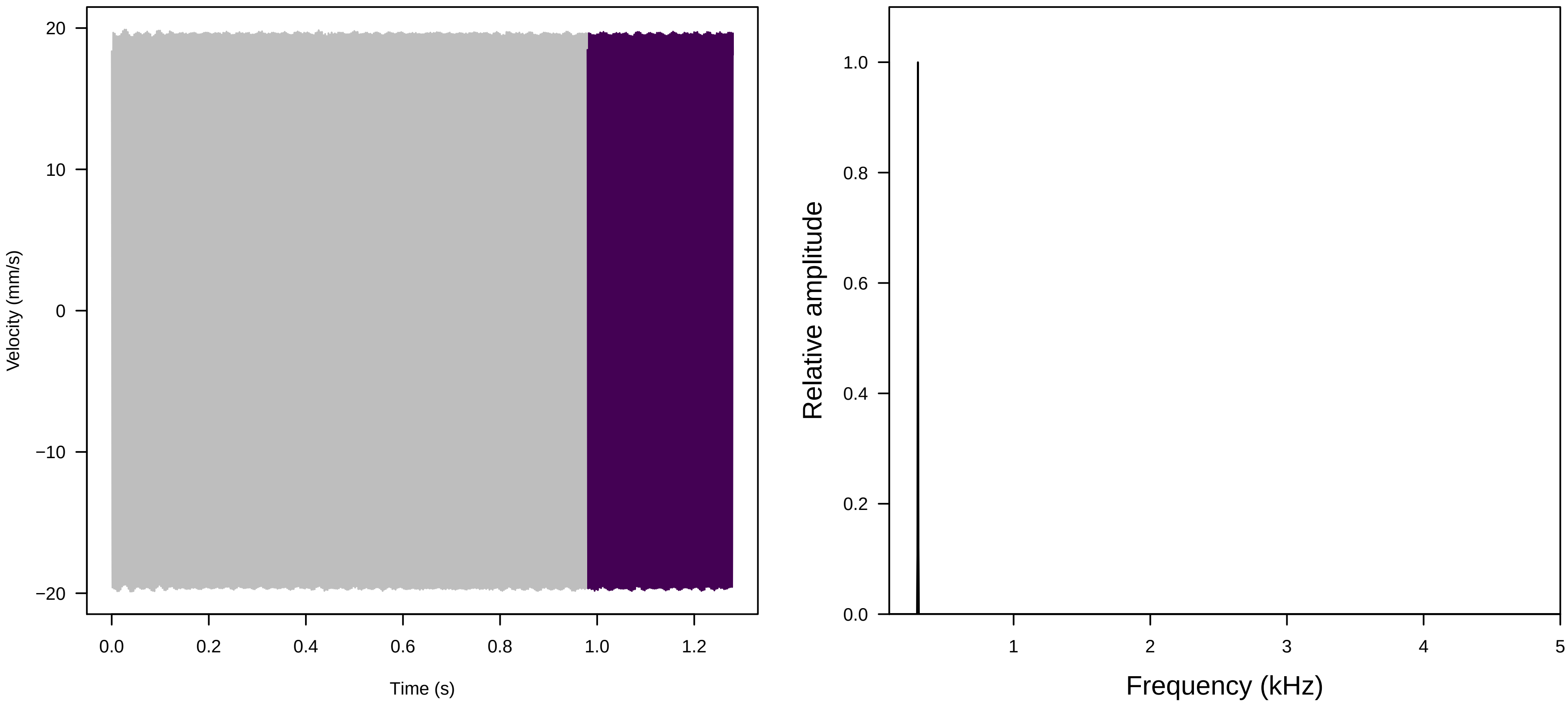

Vel. = 0.014 ; Str. = PA ; Axis = x ; Fl. accession = 10−s−81−11

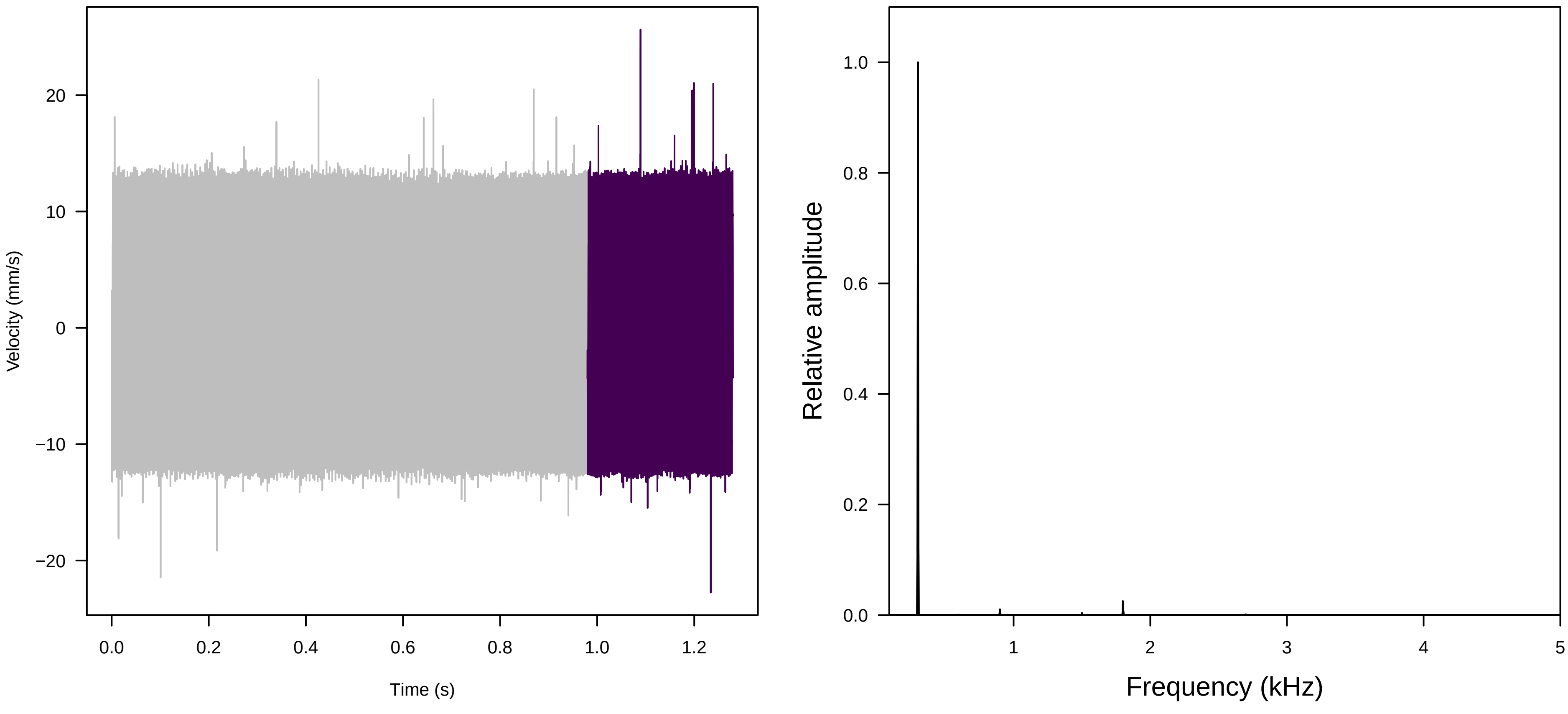

Vel. = 0.014 ; Str. = Receptacle ; Axis = x ; Fl. accession = 10−s−81−11

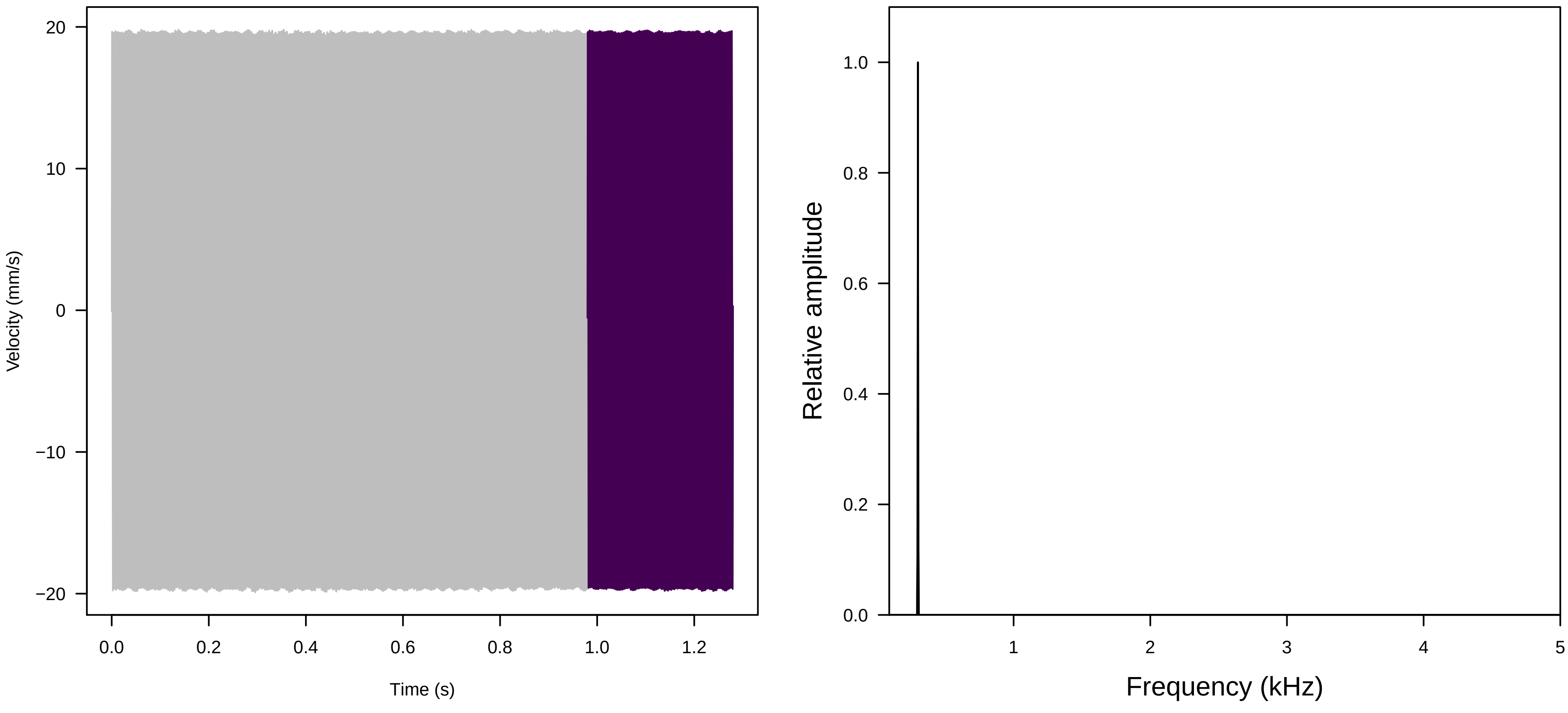

Vel. = 0.028 ; Str. = PA ; Axis = x ; Fl. accession = 10−s−81−11

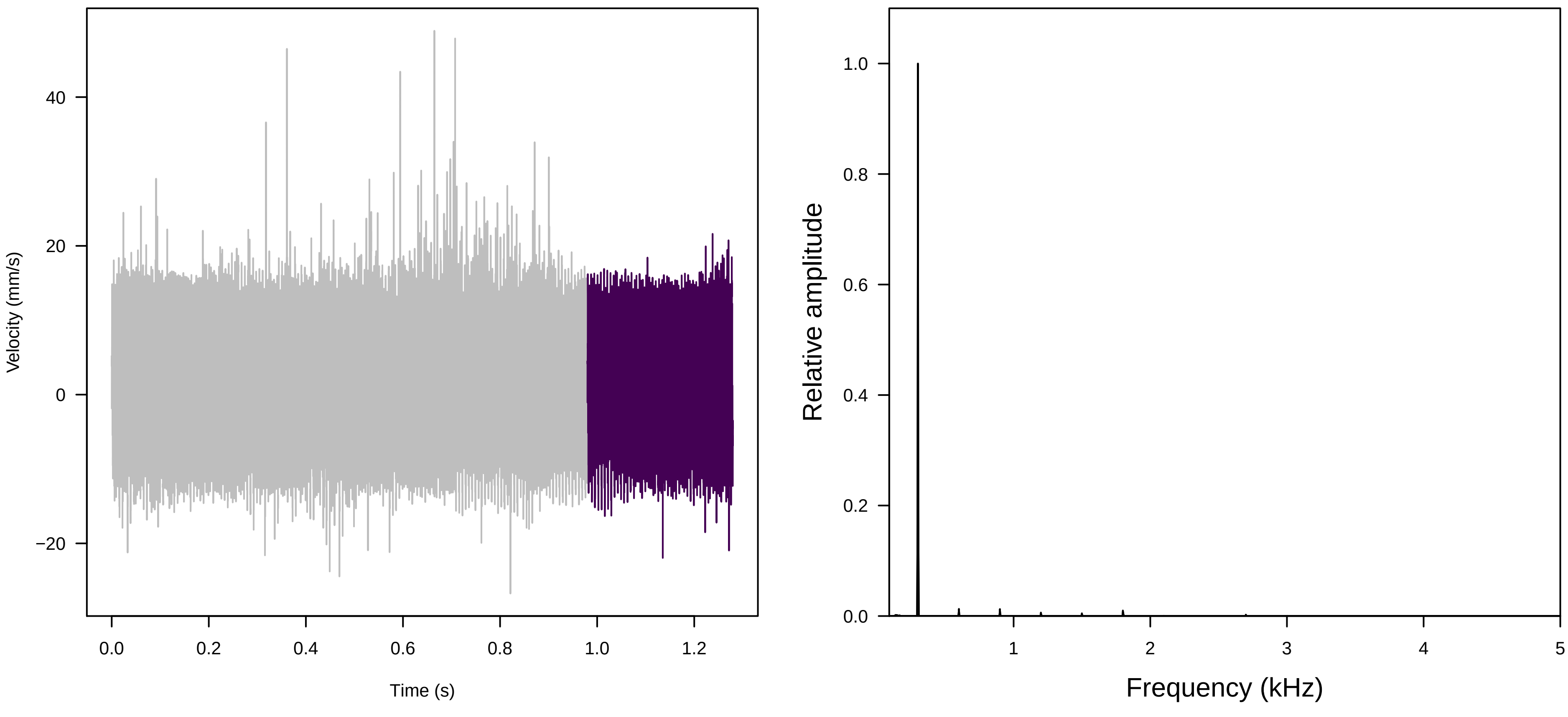

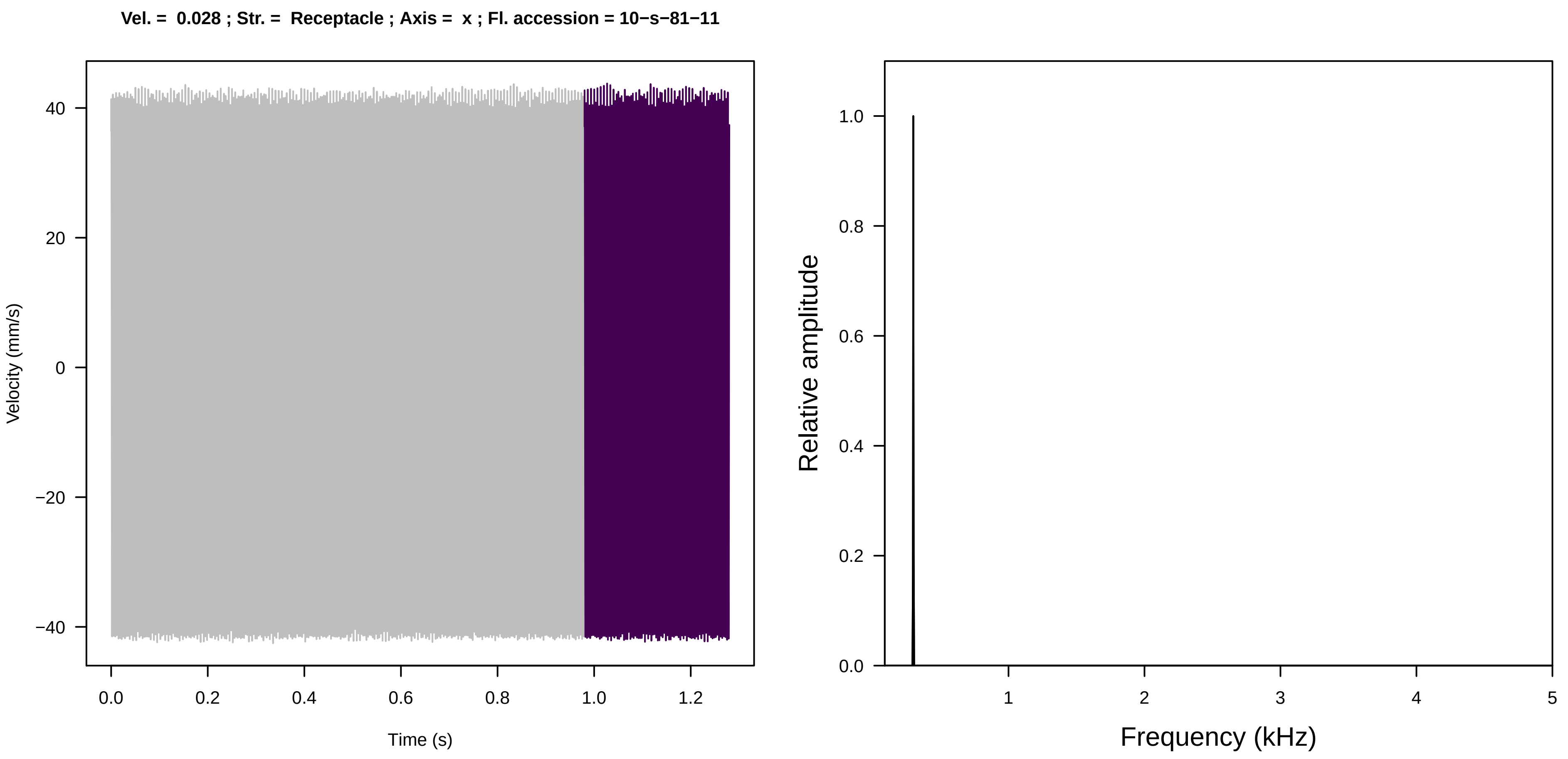
Vel. = 0.028 ; Str. = Receptacle ; Axis = x ; Fl. accession = 10−s−81−11
Velocity (mm/s)
Time (s)
Relative amplitude
Frequency (kHz)

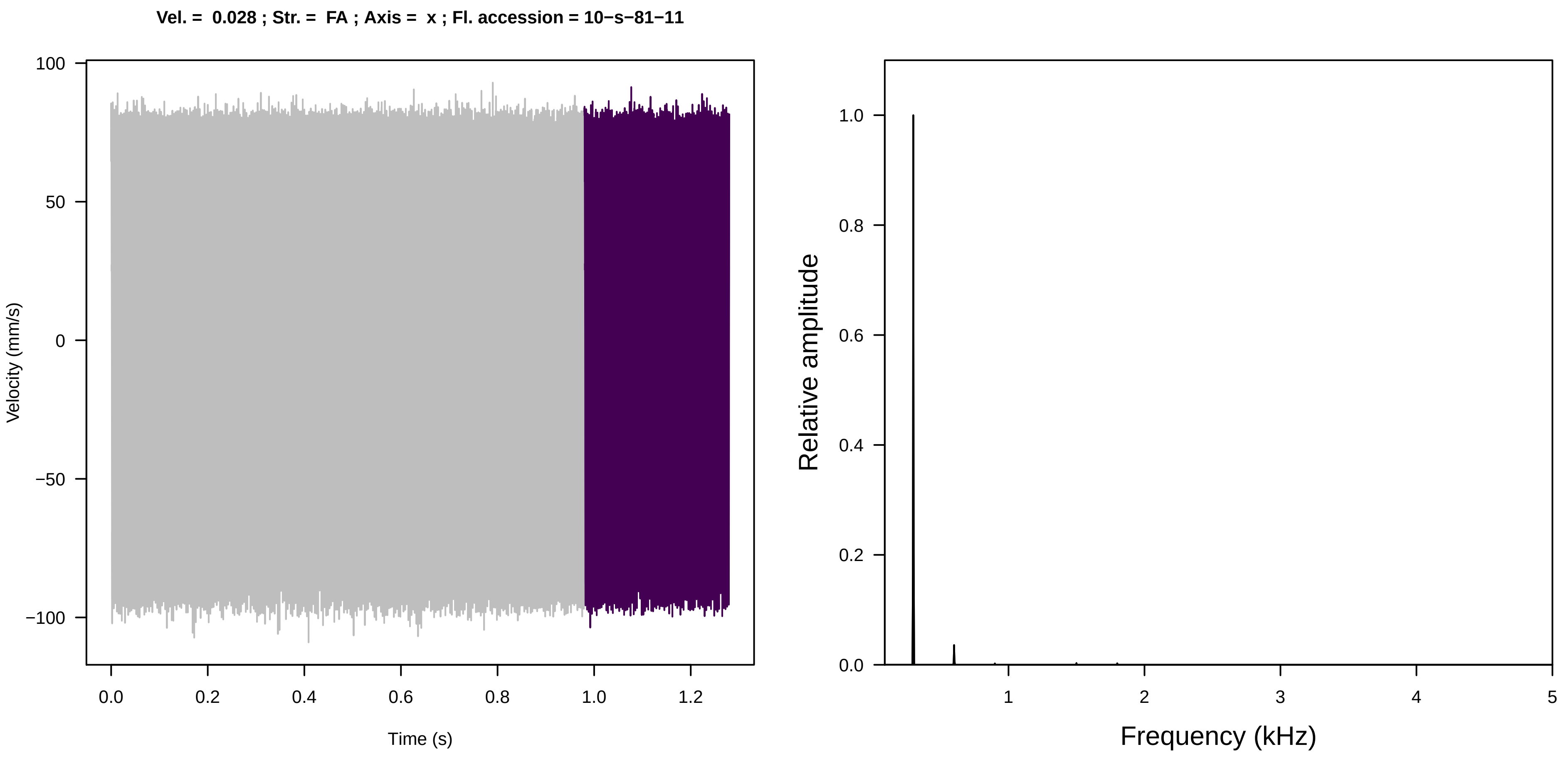
Vel. = 0.028 ; Str. = FA ; Axis = x ; Fl. accession = 10−s−81−11
Velocity (mm/s)
Time (s)
Relative amplitude
Frequency (kHz)

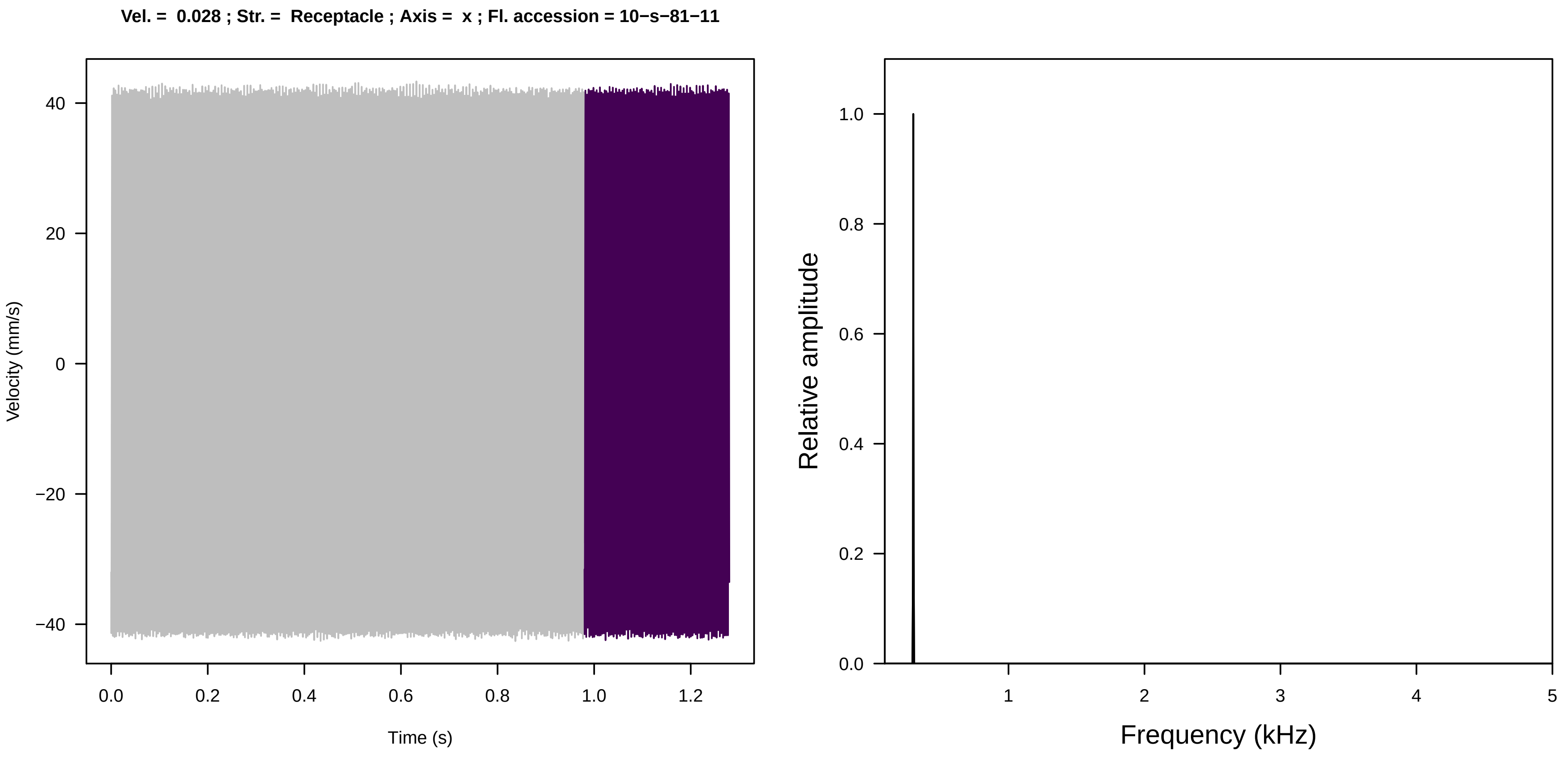
Vel. = 0.028 ; Str. = Receptacle ; Axis = x ; Fl. accession = 10−s−81−11
Velocity (mm/s)
Time (s)
Relative amplitude
Frequency (kHz)

Vel. = 0.028 ; Str. = Corolla ; Axis = x ; Fl. accession = 10−s−81−11

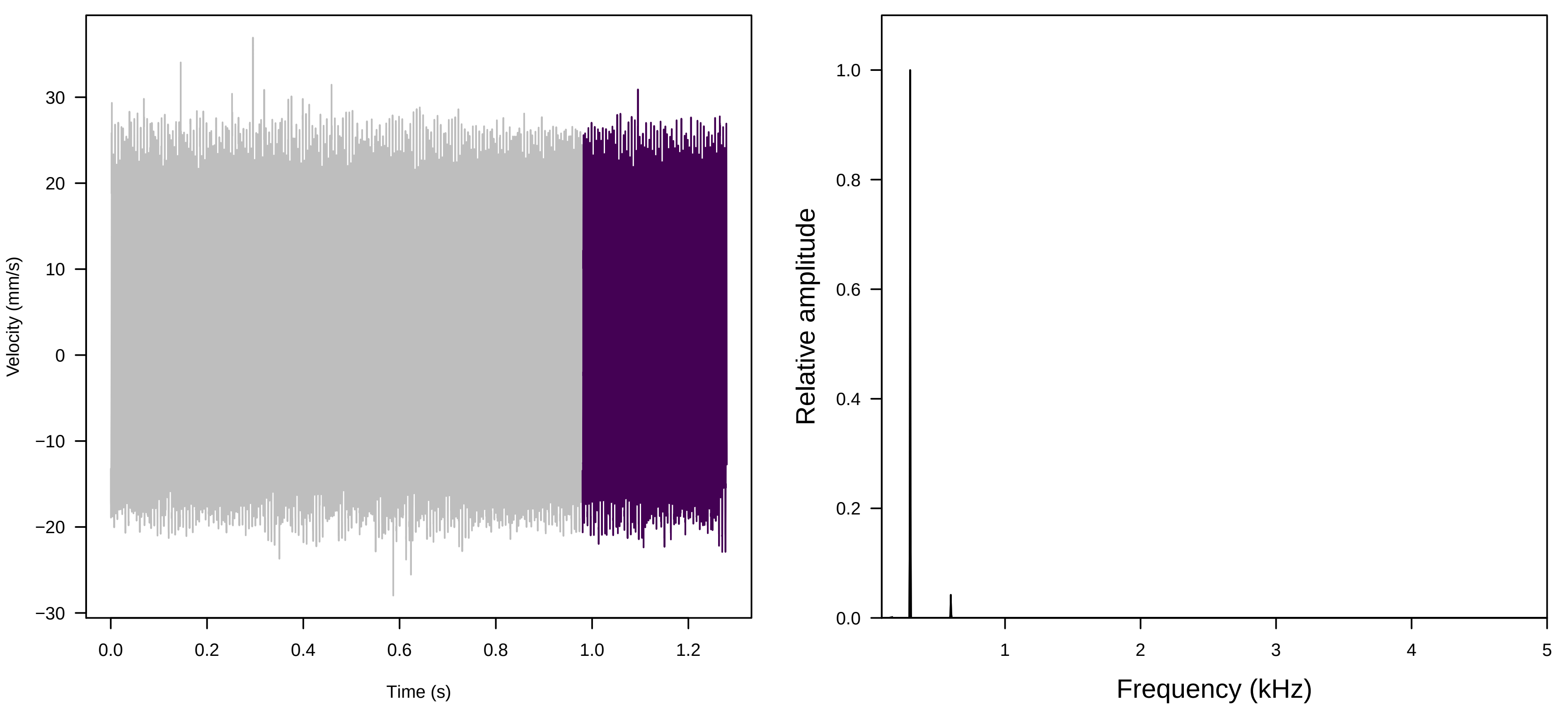

Vel. = 0.028 ; Str. = Receptacle ; Axis = x ; Fl. accession = 10−s−81−11

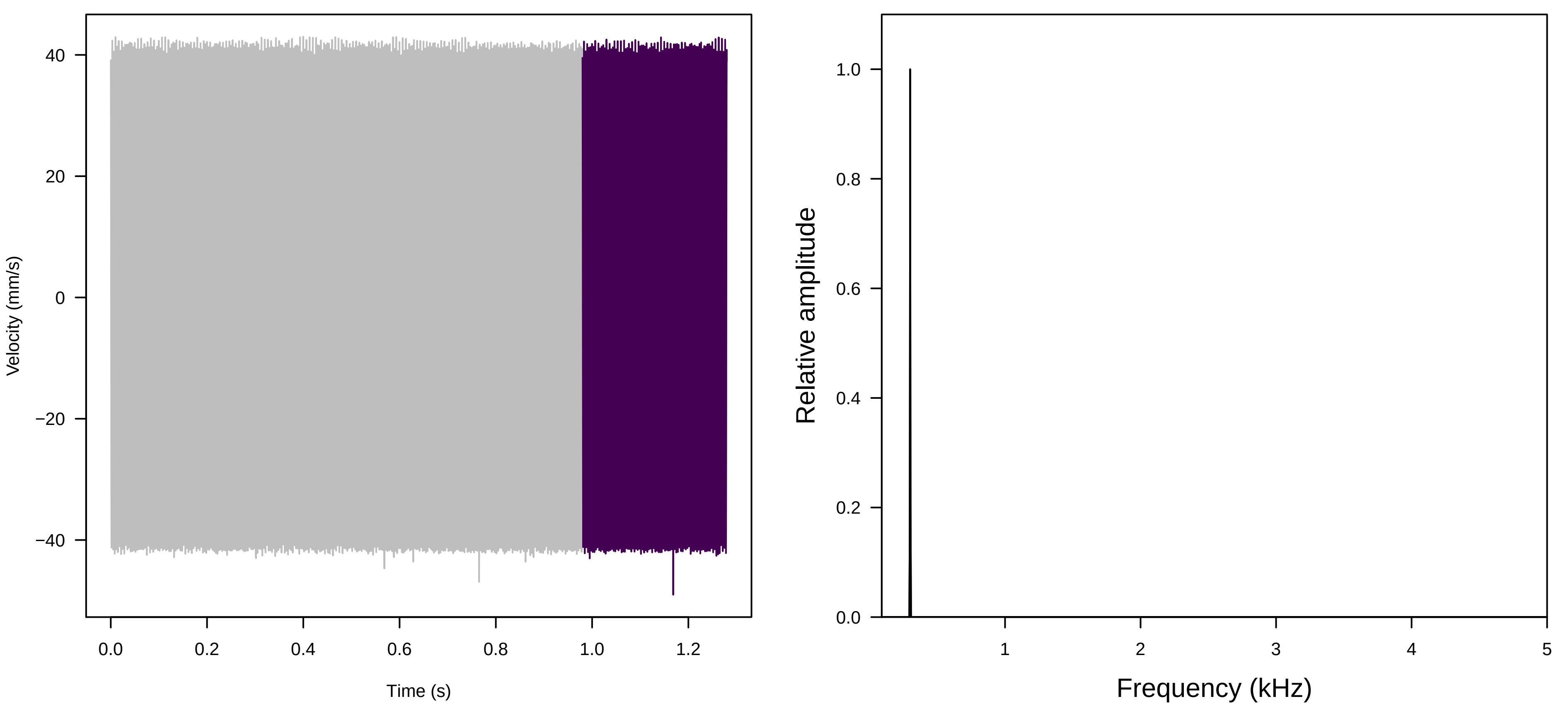

Vel. = 0.057 ; Str. = Corolla ; Axis = x ; Fl. accession = 10−s−81−11

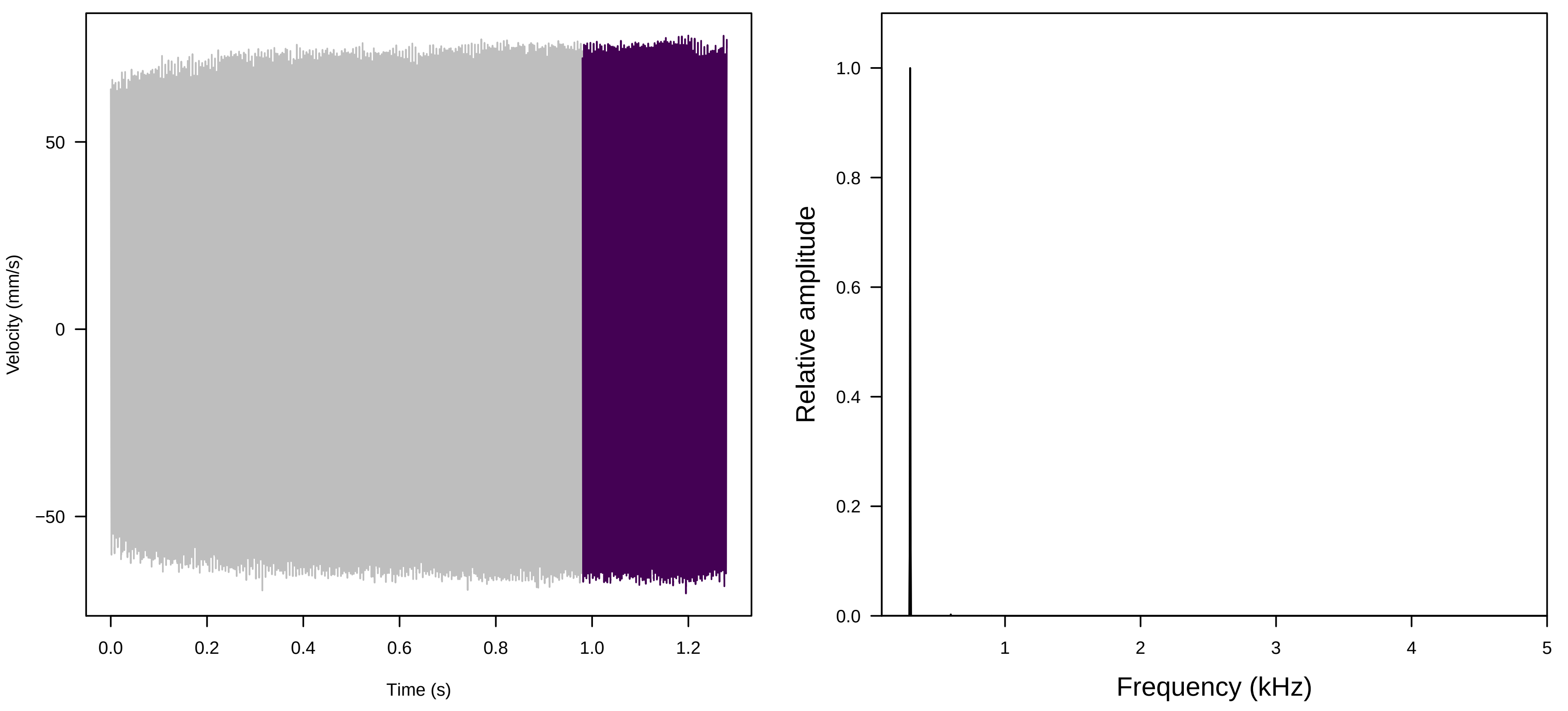

Vel. = 0.057 ; Str. = Receptacle ; Axis = x ; Fl. accession = 10−s−81−11

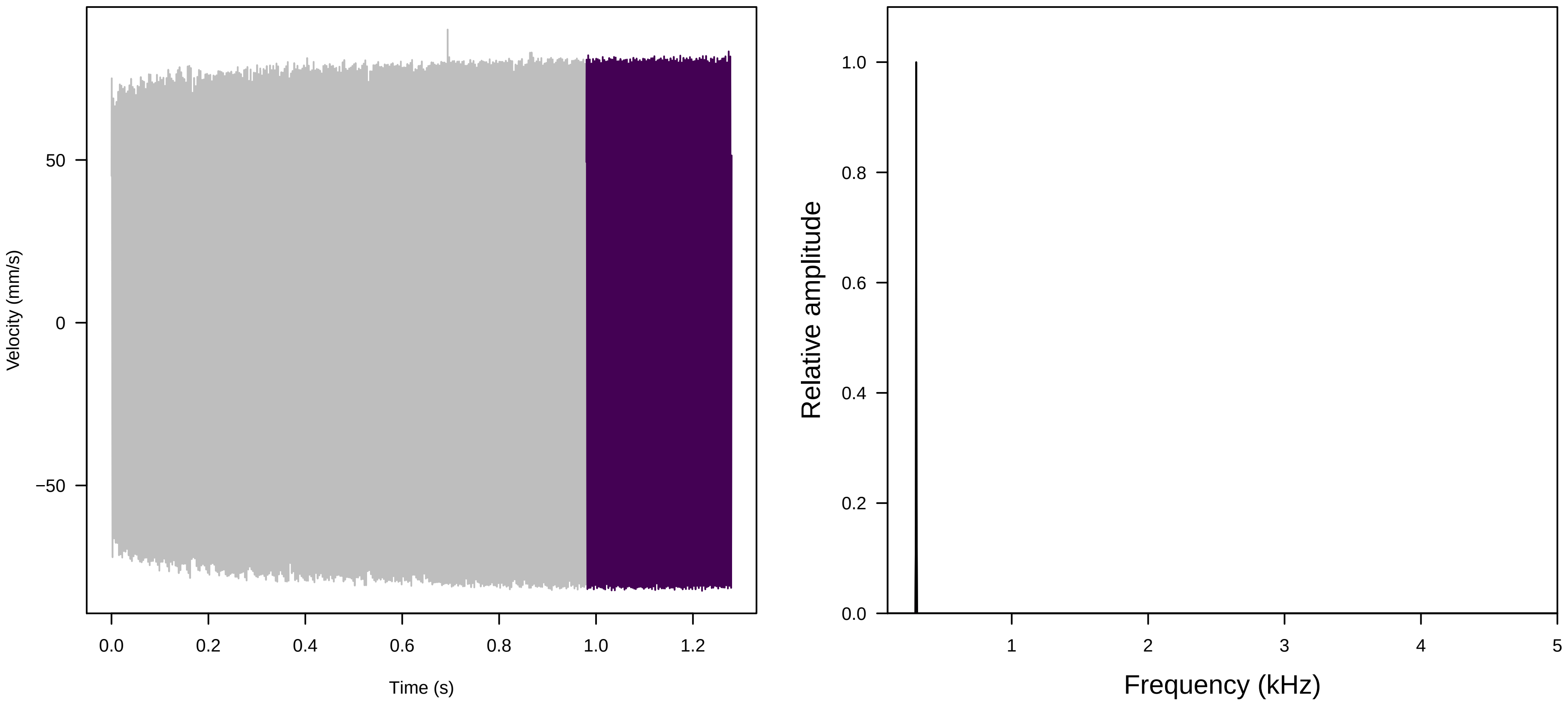

Vel. = 0.057 ; Str. = FA ; Axis = x ; Fl. accession = 10−s−81−11

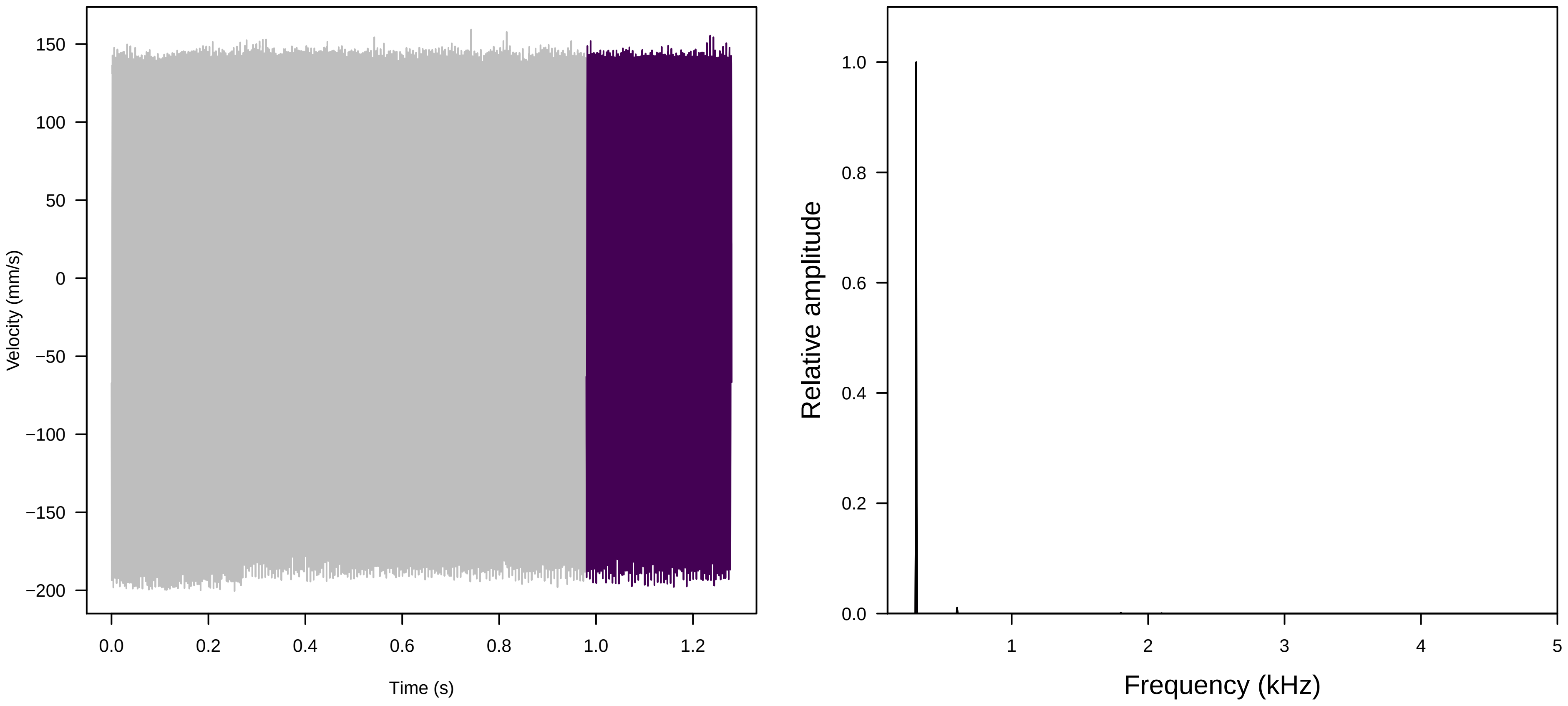

Vel. = 0.057 ; Str. = Receptacle ; Axis = x ; Fl. accession = 10−s−81−11

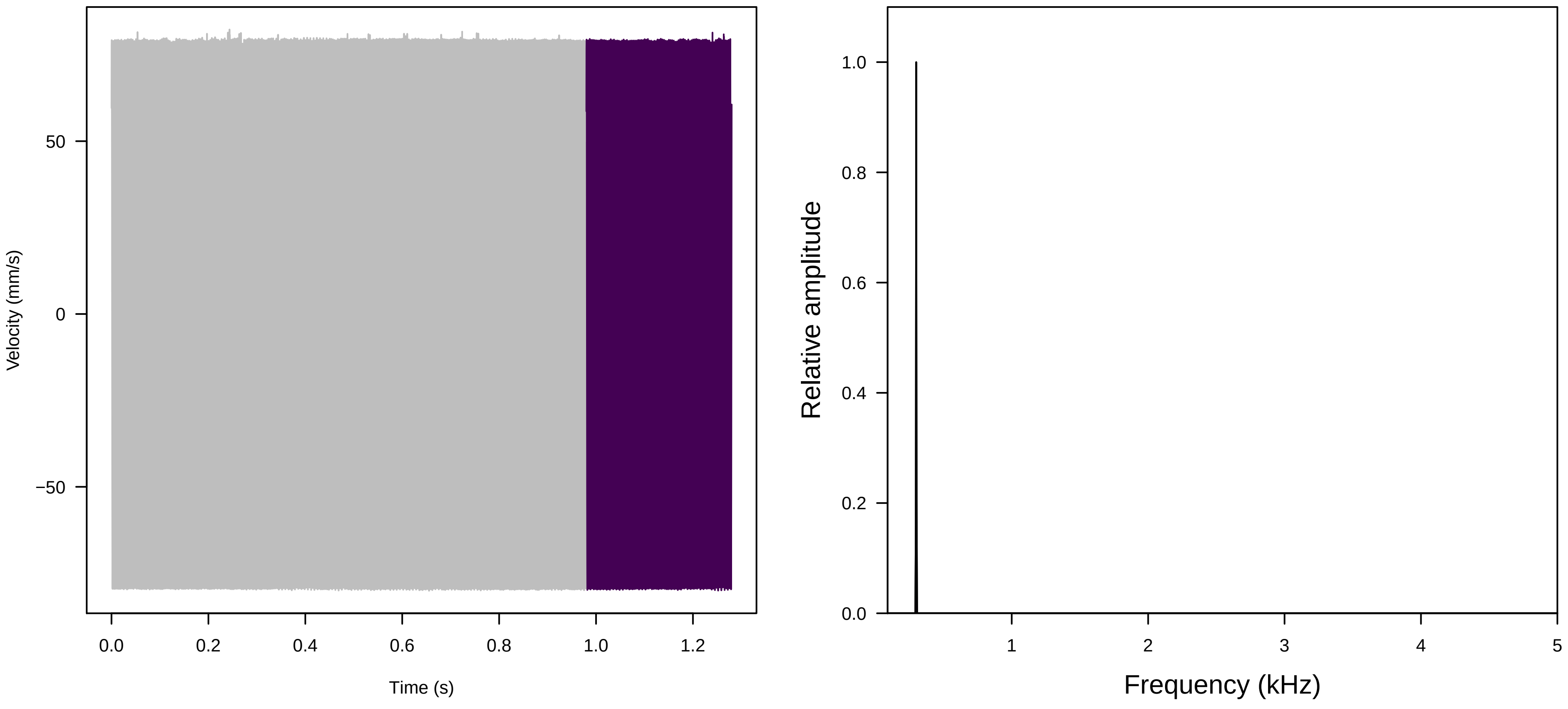

Vel. = 0.057 ; Str. = PA ; Axis = x ; Fl. accession = 10−s−81−11

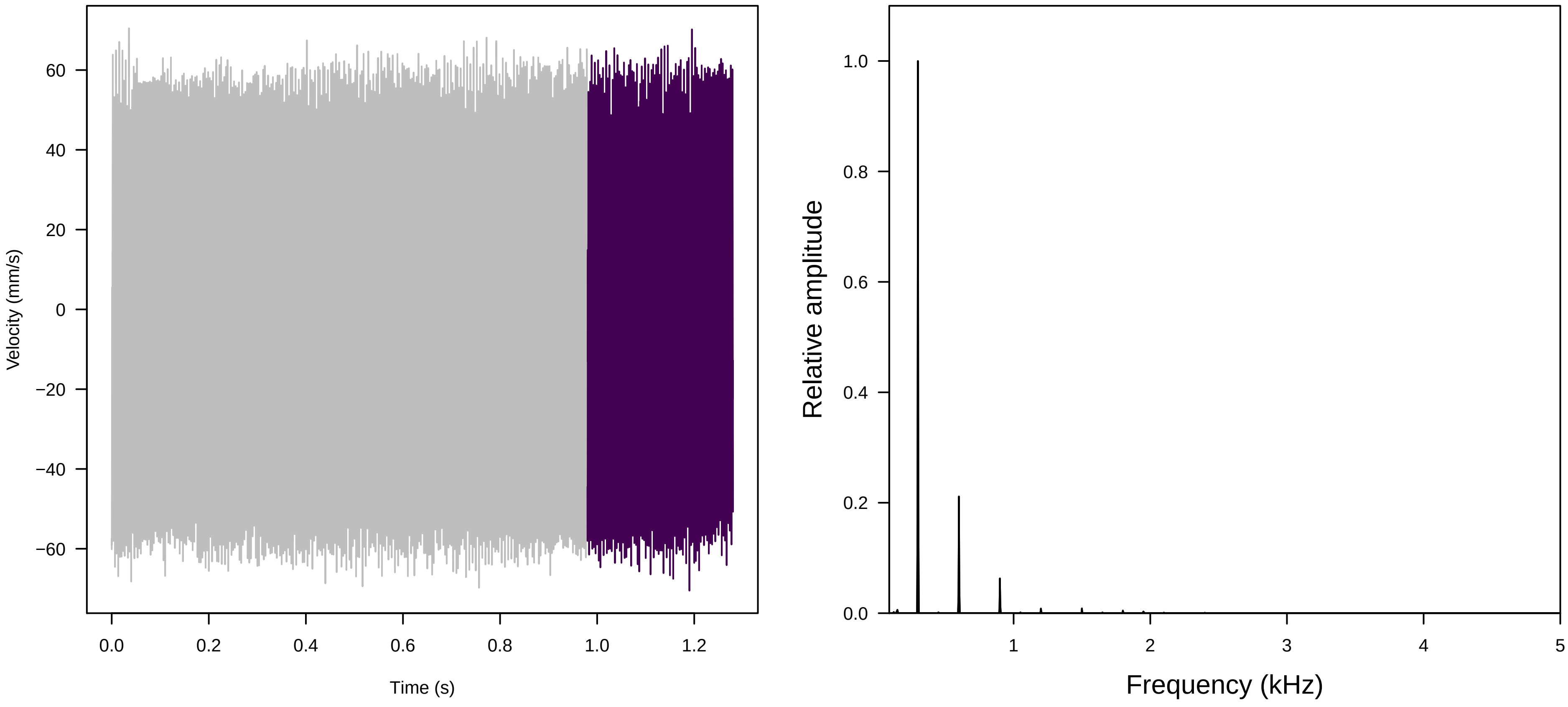

Vel. = 0.057 ; Str. = Receptacle ; Axis = x ; Fl. accession = 10−s−81−11

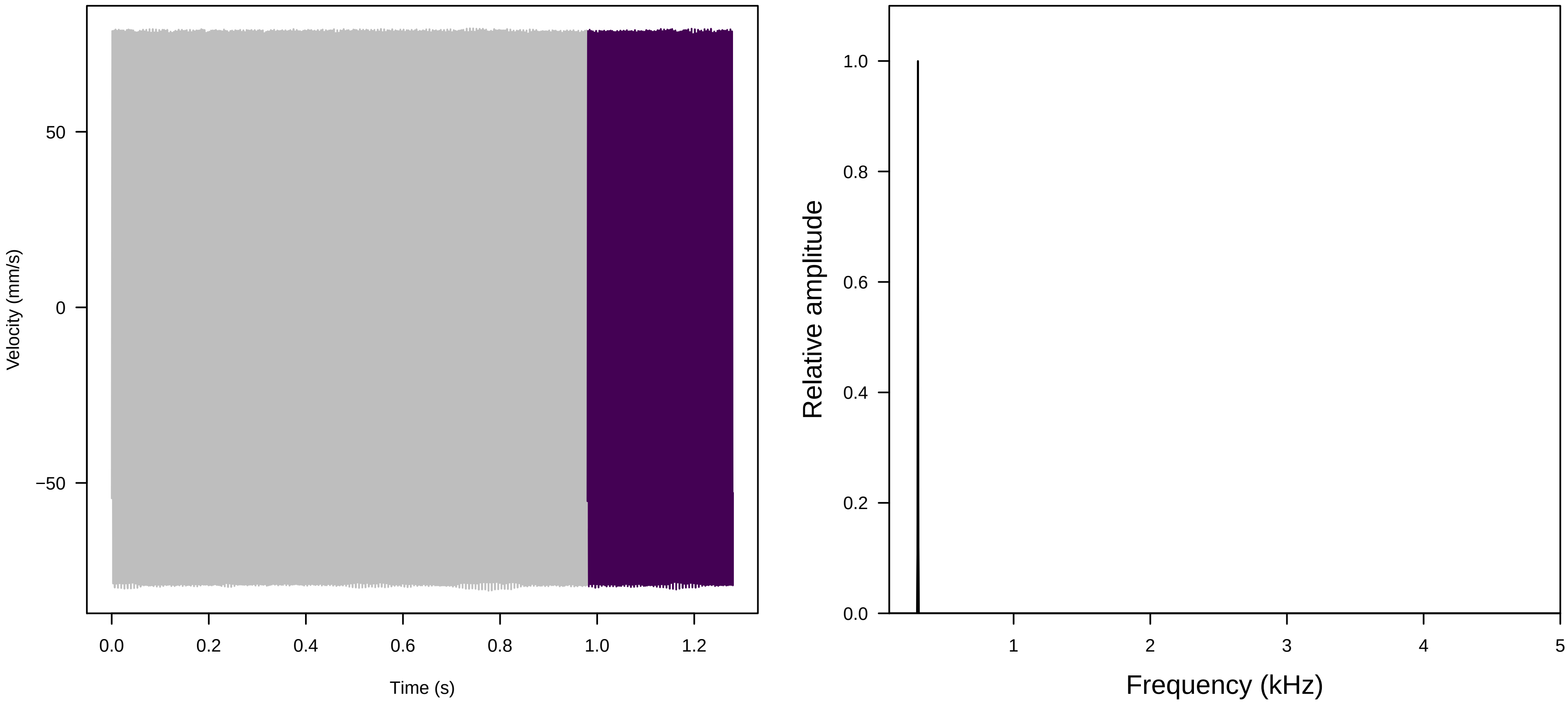

Supplement: Supplementary Figure S2 [file rsos201010supp2.pdf]
