# Supplementary material for: Biomechanical properties of a buzz-pollinated flower
Source: R Soc Open Sci. 2020 Sep 16;7(9):201010. doi: 10.1098/rsos.201010 (PMC7540744; doi:10.1098/rsos.201010)
Supplement: Supplementary Table S1 [file rsos201010supp3.docx]

**Supplementary Table S1.** Transmission of vibrations in buzz-pollinated flowers of *Solanum rostratum* (Solanaceae). Input vibrations were applied with a mechanical shaker at the base of the flower (receptacle), and measured at one of three floral structures: petals, feeding anthers or pollinating anthers. The vibrations were applied and measured along the same axis (x, y or z; see Figure 1). Input vibrations had a frequency of 300 Hz, and a RMS amplitude velocity (V_RMS_) of either 14, 18 or 57 mm s^-1^. The table shows the mean V_RMS_ and the 95% confidence intervals (CI; in parenthesis) of measured floral vibrations in the flower’s receptacle. Negative or positive values indicate damping or amplifying effects during vibration transmission, respectively. Sample size: 540 vibration measurements from 10 flowers.

| **Spatial axis** | **Target input V_RMS_ (mm s-1)** | **Observed V_RMS_ (mm s^-1^) at flower’s receptacle** |
| --- | --- | --- |
| **x** | **14** | 15.2 (14.8 – 15.5)  **8%** |
|  | **28** | 29 (28.5 – 29.4)  **4%** |
|  | **57** | 56.8 (56.0 – 57.5)  **-0.4%** |
| **y** | **14** | 14.2 (13.8 14.6)  **1%** |
|  | **28** | 29.2 (28.5 – 29.8)  **4%** |
|  | **57** | 53.4 (53.7 – 56.5)  **-4%** |
| **z** | **14** | 14.9 (14.4 – 15.4)  **6%** |
|  | **28** | 29 (28.4 – 29.6)  **4%** |
|  | **57** | 57.1 (56.4 – 57.8)  **0.3%** |
