# Supplementary material for: Biomechanical properties of a buzz-pollinated flower
Source: R Soc Open Sci. 2020 Sep 16;7(9):201010. doi: 10.1098/rsos.201010 (PMC7540744; doi:10.1098/rsos.201010)
Supplement: Supplementary Table S2 [file rsos201010supp4.docx]

| **model** | **K** | **AIC** | **ΔAIC** | **Wt** | **LL** |
| --- | --- | --- | --- | --- | --- |
| ***str* + *axes* + *rec* + *str***axes* + *str***rec* + *str***axes*** | 16 | 2797.62 | 0.00 | 0.70 | 0.70 |
| ***str* + *axes* + *rec* + *str***axes* + *str***rec*** | 14 | 2800.11 | 2.49 | 0.20 | 0.91 |
| ***str***axes***rec*** | 20 | 2801.67 | 4.05 | 0.09 | 1.00 |
| ***str* + *axes* + *rec* + *str***axes* + *rec***axes*** | 14 | 2810.18 | 12.57 | 0.00 | 1.00 |
| ***str* + *axes* + *rec* + *str***axes*** | 12 | 2812.21 | 14.59 | 0.00 | 1.00 |
| ***str* + *axes* + *rec* + *str***rec* + *rec***axes*** | 12 | 2824.71 | 27.09 | 0.00 | 1.00 |
| ***str* + *axes* + *rec* + *str***rec*** | 10 | 2826.34 | 28.72 | 0.00 | 1.00 |
| ***str* + *axes* + *rec* + *rec***axes*** | 10 | 2834.66 | 37.04 | 0.00 | 1.00 |
| ***str* + *axes* + *rec*** | 8 | 2835.96 | 38.34 | 0.00 | 1.00 |
| **null** | 3 | 3051.70 | 254.09 | 0.00 | 1.00 |

**Supplementary Table S2.** Comparison of mixed-effects models explaining the vibration amplitude recorded in floral structures of *Solanum rostratum*. In all models, V_RMS_ measured in floral structures was considered the response variable and floral structure (*str* = corolla, feeding or pollinating anther), axis of measurement (*axes* = x, y or z) and/or recorded V_RMS_ in the receptacle (*rec*) were considered fixed effects. Plant accession was considered a random effect. Models were built in a decreasing order of complexity from a full model including interactions. * = interaction; K = number of parameters; AIC = Akaike information criteria; ΔAIC = difference between the AIC for the considered model and the minimum AIC among all the models; Wt = model probabilities; LL = Log Likelihood. Sample size: 540 vibration measurements from 10 flowers.
