# Supplementary material for: Biomechanical properties of a buzz-pollinated flower
Source: R Soc Open Sci. 2020 Sep 16;7(9):201010. doi: 10.1098/rsos.201010 (PMC7540744; doi:10.1098/rsos.201010)
Supplement: Supplementary Table S3 [file rsos201010supp5.docx]

**Supplementary Table S3.** Statistical analysis of the effect of floral structure (corolla, feeding or pollinating anther), axis of measurement (x, y or z) and amplitude of input vibrations on the Root Mean Squared amplitude velocity (V_RMS_; mm s^-1^) of transmitted vibrations measured in different parts of a buzz-pollinated flower of *Solanum rostratum*. Floral vibrations were applied at the base of the flower using a mechanical shaker attached to the flower’s receptacle and measured either at the anther tips of feeding and pollinating anthers or at the distal end (1/4) of the upper petal. The model shown here was selected among competing linear mixed-effects models using AIC. The model includes all second-order interactions among input amplitude velocity (V_RMS_ measured in the receptacle), floral structure and axis of measurement. In this model, input amplitude velocity, axis of measurement, and floral structure measured were considered as fixed effects and plant accession as a random effect. The reference values for the model (intercept) are petal and z-axis. Only fixed-effects are shown in the table. Sample size: 540 vibration measurements from 10 flowers.

|  | **Estimate ± SD** | ***F*** | ***P*-value** |
| --- | --- | --- | --- |
| **Intercept** (Corolla, x-axis) | 11.6 ± 13.4 |  |  |
| **Floral structure** |  | 2.3 | 0.104 |
| Feeding anther | -24.6 ± 15.7 |  |  |
| Pollinating anther | -15.7 ± 15.6 |  |  |
| **Spatial axis** |  | 2.6 | < 0.076 |
| y | -13.5 ± 15.6 |  |  |
| z | -20.5 ± 15.6 |  |  |
| **Input amplitude velocity (mm s^-1^)** | 0.8 ± 0.3 | 206.3 | < 0.001 |
| **Floral structure** × **Spatial axis** |  | 9.7 | < 0.001 |
| Feeding anther × y | 76.8 ± 14.8 |  |  |
| Pollinating anther × y | 46.7 ± 14.7 |  |  |
| Feeding anther × z | 79.4 ± 14.7 |  |  |
| Pollinating anther × z | 52.6 ± 14.7 |  |  |
| **Floral structure** × **Input velocity** |  | 8.8 | < 0.001 |
| Feeding anther × Input velocity | 1.3 ± 0.3 |  |  |
| Pollinating anther × Input velocity | 1.2 ± 0.3 |  |  |
| **Spatial axis** × **Input velocity** |  | 3.5 | 0.030 |
| y × Input velocity | 0.4 ± 0.4 |  |  |
| z × Input velocity | 0.9 ± 0.3 |  |  |
